# Singularity and Commonality in Response to SARS-CoV-2 in Lung and Colon Cell Models

**DOI:** 10.3390/ijms231810451

**Published:** 2022-09-09

**Authors:** Anastasia Meshcheryakova, Philip Zimmermann, Martina Salzmann, Peter Pietschmann, Diana Mechtcheriakova

**Affiliations:** 1 Department of Pathophysiology and Allergy Research, Center of Pathophysiology, Infectiology and Immunology, Medical University of Vienna, 1090 Vienna, Austria; 2 Nebion AG, 8048 Zurich, Switzerland

**Keywords:** SARS-CoV-2, SARS-CoV, Calu-3, Caco-2, lung, colon, systems biology, APOBECs, APOBEC3G

## Abstract

The systemic nature of COVID-19 with multiple extrapulmonary manifestations of disease, largely due to the wide tissue expression of SARS-CoV-2 major entry factors, as well as the patient-specific features of COVID-19 pathobiology, determine important directions for basic and translational research. In the current study, we addressed the questions of singularities and commonalities in cellular responses to SARS-CoV-2 and related SARS-CoV on the basis of compendium-wide analysis of publicly available transcriptomic datasets as part of the herein implemented multi-modular UNCOVIDING approach. We focused on cellular models attributed to the epithelial cells of the respiratory system, the Calu-3 cell line, and epithelial cells of the gastrointestinal tract, the Caco-2 cell line, infected with either SARS-CoV-2 or SARS-CoV. Here, we report the outcome of a comparative analysis based on differentially expressed genes in terms of perturbations and diseases, Canonical pathways, and Upstream Regulators. We furthermore performed compendium-wide analysis across more than 19,000 mRNASeq datasets and dissected the condition-specific gene signatures. Information was gained with respect to common and unique cellular responses and molecular events. We identified that in cell lines of colon or lung origin, both viruses show similarities in cellular responses; by contrast, there are cell type-specific regulators that differed for Calu-3 and Caco-2 cells. Among the major findings is the impact of the interferon system for lung Calu-3 cells and novel links to the liver- and lipid-metabolism-associated responses for colon Caco-2 cells as part of the extrapulmonary pathomechanisms in the course of COVID-19. Among differently expressed genes, we specifically dissected the expression pattern of the APOBEC family members and propose APOBEC3G as a promising intrinsic antiviral factor of the host response to SARS-CoV-2. Overall, our study provides gene expression level evidence for the cellular responses attributed to pulmonary and gastrointestinal manifestations of COVID-19.

## 1. Introduction

*Coronaviridae* are RNA viruses that cause respiratory diseases with mild to severe symptoms. In the human population, six coronaviruses were known until 2019. Four of them are associated with the common cold and cause mild symptoms, whereas the severe acute respiratory syndrome coronavirus (SARS-CoV) and the Middle East respiratory syndrome coronavirus (MERS-CoV), which emerged in the years 2002 and 2012, respectively, caused severe, life-threatening diseases [1,2]. In December 2019, a new coronavirus was identified and named SARS-CoV-2, given its close relationship to SARS-CoV. The severe disease induced by this virus was named as Coronavirus disease 19 (COVID-19) [3,4]. The SARS-CoV-2 virus is a single-stranded positive-sense RNA virus that mediates entry to the host cells through interaction between the viral spike (S) glycoprotein and the cell surface receptor on the target cell, followed by membrane fusion. Similarly to SARS-CoV, the cellular receptor is the metallopeptidase known as angiotensin-converting enzyme 2 (ACE2) [5,6]. Additionally, entry into the host cell depends on the action of proteases that fulfill the function of activators of the fusion potential of the viral S protein. The most critical player is thereby the transmembrane serine protease 2, TMPRSS2, used for priming [6,7]. Meta analyses of gene expression of the major virus entry factors—ACE2 and TMPRSS2—across various organs, tissues, and cell types indicated already at the beginning of the pandemic the systemic nature of COVID-19, going beyond the targeting and damaging of the upper airways and the lung by the virus [8,9,10]. Indeed, the compendium of aggregated clinical data reported multiple extrapulmonary manifestations of disease, including cardiovascular, hematological, renal, neurological, dermatological, and gastrointestinal manifestations [11,12]. In the course of COVID-19, gastrointestinal symptoms have been reported in up to 60% of patients and may be associated with prolonged disease duration and/or poorer disease course [13,14]. Understanding of organ- and tissue-specific molecular programs triggered by SARS-CoV-2 will undoubtedly contribute to the identification of novel biomarkers and treatment strategies.

The activation-induced cytidine deaminase (AID)/Apolipoprotein B mRNA editing enzyme catalytic subunit (APOBEC) family of cytosine-to-uracil (C-to-U) deaminases is an integral part of the host defense system, involved in immune and non-immune processes [15,16,17]. APOBECs thereby represent an important class of intrinsic antiviral factors, which restrict viral infection by a multifaceted mode of action. This includes (i) the introduction of lethal mutations into the viral genome by a process known as hypermutation, (ii) blocking the viral nucleic acid synthesis, (iii) the package of APOBECs into budding virions and inhibition of infection spreading into viral naïve cells, and (iv) the enchantment of the adaptive immune responses, performed by cytotoxic T lymphocytes and B cells, and the antiviral action of natural killer cells [18,19,20]. The APOBEC family comprises ten members (APOBEC1, APOBEC2, APOBEC3A, APOBEC3B, APOBEC3C, APOBEC3D, APOBEC3F, APOBEC3G, APOBEC3H, and APOBEC4) and act against multiple DNA-type and RNA-type viruses [18,20]. Each cell type exhibits its own repertoire of APOBECs; thereby, the cell type-, tissue type-, and organ-attributed APOBEC-driven antiviral defense capability is defined. The molecular mechanisms by which APOBECs may contribute to host defense against SARS-CoV-2 in various targeted organs have not yet been elucidated. On the basis of comprehensive data analysis and data mining, Meshcheryakova et al. [21] addressed key aspects interrelating the AID/APOBECs and SARS-CoV-2 in light of the patient-specific course of COVID-19.

Understanding of the organ-specific manifestations of COVID-19 is of particular importance for dissecting the patient-specific nature of disease pathobiology. The accumulated pool of transcriptomic datasets as well as the availability of powerful tools for compendium-wide analysis enables efficient extraction of novel information and knowledge gain. As an analytical solution, we developed and implemented a sophisticated analysis strategy, named by us as UNCOVIDING: Understanding COVID-19 by Integrative Data Mining. We focused on the comparison of datasets derived from transcriptomic analysis of the cellular responses triggered by SARS-CoV-2 or SARS-CoV on the basis of cellular models. This includes Calu-3 cells, representing the epithelial cells attributed to the respiratory system, and Caco-2 cells, representing epithelial cells of the gastrointestinal tract. Comparative analyses were performed on the level of differentially expressed genes, the attributed Canonical Pathways, and Upstream Regulators. Furthermore, condition-specific gene signatures were dissected. We thereby juxtaposed both the two cell lines under investigation as well as the two viruses under investigation. Overall, the study identified the singularities and commonalities of the cellular responses. Our analysis gives novel additive information and knowledge to those recently published by Wyler E et al. [22]. Additionally, a special focus of the presented study was given to the expression and regulation pattern of APOBECs upon infection with SARS-CoV-2 or SARS-CoV. As a major outcome of this part of the analysis, we propose APOBEC3G among the relevant factors of the host antiviral response. 

## 2. Results

### 2.1. Expression of ACE2 and TMPRSS2 in Lung and Colon Cell Lines

**Figure 1:**
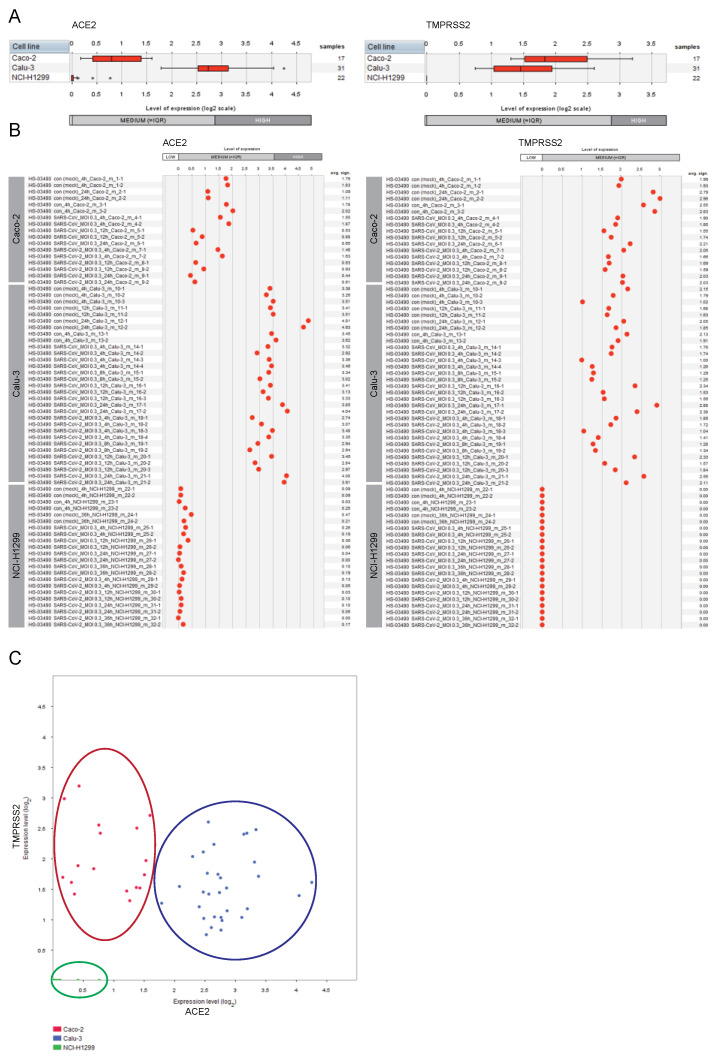
Expression patterns of *ACE2* and *TMPRSS2* across lung and colon cell lines. GENEVESTIGATOR-based analysis was performed to extract the expression levels of the genes ACE2 and TMPRSS2 from mRNA sequencing (mRNASeq) data (GSE148729) across Caco-2, Calu-3, and NCI-H1299 cells. Mock-infected cells and those infected with SARS-CoV or SARS-CoV-2 viruses are included (various time points upon infection). The levels of expression are given as log2 transformed values sub-divided into low, medium, and high expression according to GENEVESTIGATOR. (**A**) Box-plots representing the expression levels of *ACE2* and *TMPRSS2* across all conditions attributed to a given cell type. Outliers are represented as stars. (**B**) The expression patterns of *ACE2* and *TMPRSS2* are shown for each individual sample separately. (**C**) 2-Gene plot shows the expression of *ACE2* and *TMPRSS2* simultaneously; color code: *red*, Caco-2; *blue*, Calu-3, *green*, NCI-H1299. The data were assessed and extracted from GENEVESTIGATOR on 9 January 2021, 5 May 2021, and 28 August 2022.

We first assessed and compared the expression levels of the genes encoding the *ACE2* receptor and the *TMPRSS2* protease in Caco-2 cells, Calu-3 cells, and NCI-H1299 cells (Figure 1). 

The Calu-3 cells were characterized by moderate/high expression level of *ACE2* and moderate expression level of *TMPRSS2*, whereas the second lung-derived cell line NCI-H1299 showed low/undetectable expression of both genes. For the colon-derived Caco-2 cells we observed moderate expression of both *ACE2* and *TMPRSS2*. Given the observed no/low expression of the entry receptor and the critical protease in NCI-H1299 cells, complemented by described low susceptibility of NCI-H1299 to the infection by the SARS-CoV and SARS-CoV-2 viruses (reported by Wyler E et al. [22]), we excluded this cell line from our follow-up analyses. 

### 2.2. Expression of ACE2 and TMPRSS2 in Cell Types of the Respiratory System and the Gastrointestinal Tract

**Figure 2:**
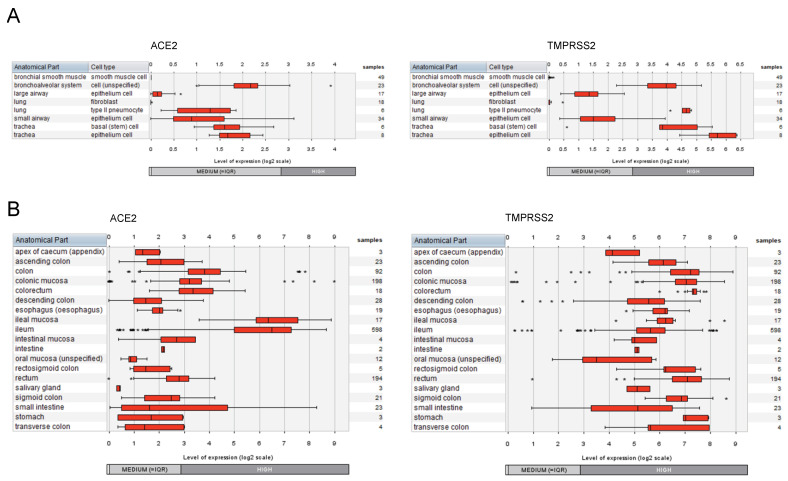
Expression patterns of *ACE2* and *TMPRSS2* across cell types and tissues of the respiratory and the gastrointestinal systems. The expression levels of *ACE2* and *TMPRSS2* were assessed by GENEVESTIGATOR-based compendium-wide analysis. (**A**) To extract the gene expression pattern, we applied the following filter on mRNASeq datasets: Anatomy > Cell Type > Respiratory System Cell (n = 167 datasets). The box plots represent the expression signature across various cell types from different anatomical parts of the respiratory system. Outliers are represented as stars. (**B**) To extract the gene expression pattern, we applied the following filter on mRNASeq datasets: Anatomy > Tissue_Alimentary System > Gastrointestinal Tract (n = 1309 datasets). The box plots represent the expression signature across various tissue types of the gastrointestinal system. Outliers are represented as stars. The data were assessed and extracted from GENEVESTIGATOR on 13 and 15 October 2021 and 28 and 29 August 2022.

Complementary to the analysis of the cell line models, we assessed the expression pattern of *ACE2* and *TMPRSS2* in the multitude of primary cell types of the respiratory system as well as of the gastrointestinal tract. For this, the corresponding datasets from the compendium of mRNASeq datasets covering various cell types and tissues (n = 167 datasets attributed to the respiratory system; n = 1309 datasets attributed to the gastrointestinal tract) were investigated using the GENEVESTIGATOR platform. The integrative analysis revealed moderate to high expression of both the receptor and the protease in various cell types and tissues of the respiratory and the gastrointestinal systems (Figure 2). For the respiratory tract, this includes, besides others, the tracheal, small and large airway epithelial cells, and cells of the bronchoalveolar system (Figure 2A). Pulmonary lung fibroblasts and bronchial smooth muscle cells showed no/low expression of both genes. Expression mapping of *ACE2* and *TMPRSS2* across the gastrointestinal tract showed moderate to high expression for all anatomical parts, including oral mucosa, esophagus, stomach, small intestine, and colon (Figure 2B).

### 2.3. Understanding the Pathobiology of COVID-19 by Applying a Multi-Modular Integrative Approach

**Figure 3:**
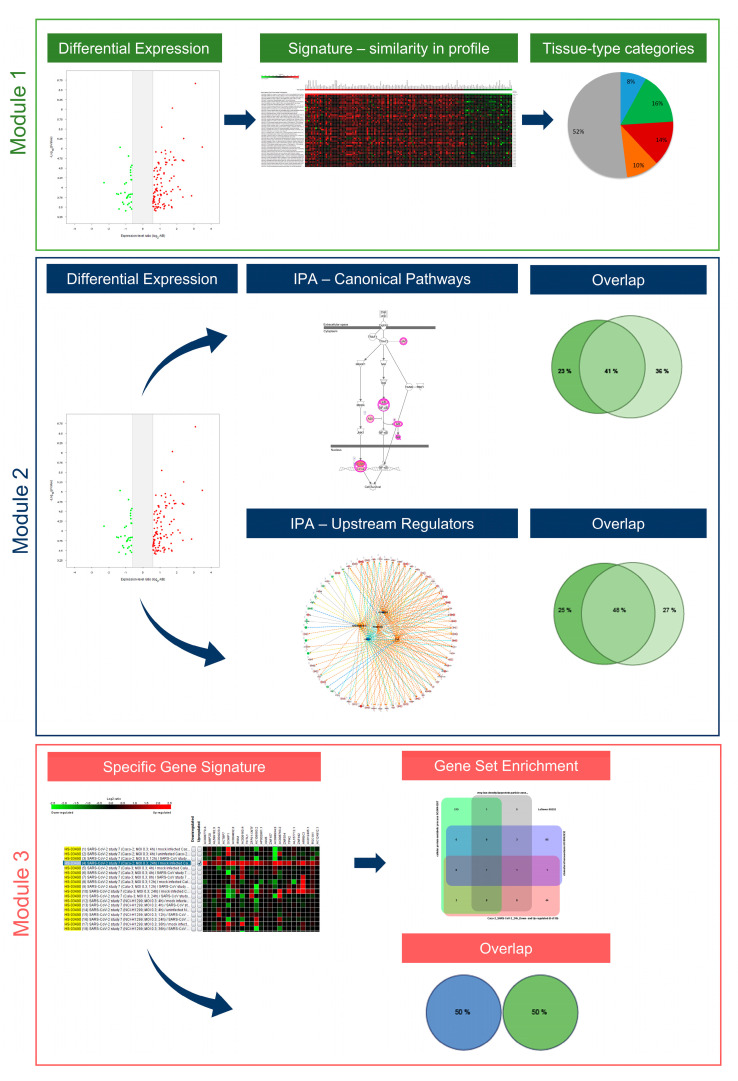
The UNCOVIDING approach and the individual analytical modules. The figure illustrates the 3-modular algorithm on the basis of the compendium-wide analysis of transcriptomic datasets.

Multi-modular analysis algorithms are the cornerstone for the understanding of the pathobiology of complex multifactorial diseases. Thus far, two integrative strategies—the MuSiCO and the DIICO algorithms—have been developed and successfully applied by us in the field of oncology and onco-immunology [23,24,25,26,27]. We herein present our newly developed analysis algorithm, named by us as UNCOVIDING: Understanding COVID-19 by Integrative Data Mining, which consolidates multiple analytical modules for integrative compendium-wide analysis of transcriptomics datasets followed by the dissection of disease-relevant Canonical Pathways and Upstream Regulators as well as the nomination of disease-specific gene signatures. Figure 3 gives an overview of the individual modules of the UNCOVIDING strategy applied in the current study. *Module 1* takes as the basis differentially expressed genes, which are then combined into a signature for a compendium-wide analysis against all datasets available within GENEVESTIGATOR to identify conditions with the strongest similarity. The outcomes are classified into categories, with the main focus given to respiratory system- and the gastrointestinal system-associated studies. The central part of *Module 2* is the Ingenuity Pathway Analysis (IPA)-based analysis that dissects Canonical Pathways and Upstream Regulators on the basis of differentially expressed genes, here defined via GENEVESTIGATOR; as outcomes, the results are compared to elucidate singularities or commonalities among Canonical Pathways and Upstream Regulators on the basis of Venn diagrams. Within *Module 3*, we dissect the condition-attributed specific gene signatures and extract additional information on the genes that are part of those signatures using the *Gene Set Enrichment* Tool from GENEVESTIGATOR. 

UNCOVIDING represents an analytical workflow for better understanding COVID-19-related processes. It consists of data scouting, curation, single-study analysis, compendium-wide analyses across a multitude of studies, and interpretation against other transcriptomic datasets and against literature-based knowledge, using GENEVESTIGATOR and IPA, respectively. It is universal in the sense that it can be applied for addressing research questions in any COVID-19-related study and is therefore of interest for the community. Furthermore, the analytical strategy can be used to dissect the pathomechanisms of other complex multifactorial diseases.

### 2.4. Tissue-Specific Response to SARS-CoV and SARS-CoV-2

The multi-modular integrative analysis was applied to address the question of the tissue type specificity of cellular responses triggered by SARS-CoV or SARS-CoV-2. Within *Module 1* (Figure 3) we first applied the *Differential Expression Tool* from GENEVESTIGATOR to identify genes showing significant upregulation or downregulation in SARS-CoV or SARS-CoV-2 infected cells (24 h time point) in comparison to mock-infected control cells. The analysis was performed for Calu-3 and Caco-2 cells separately. Next, the list of differentially expressed genes was taken as a signature for analysis using the *Signature Tool*. The alignment was performed across the whole compendium of the mRNASeq datasets (n = 19,230) available within GENEVESTIGATOR at the time of analysis. We utilized the power of this tool to extract experimental or disease conditions showing similarity to transcriptional patterns triggered by SARS-CoV or SARS-CoV-2. The maximal number of genes that can be subjected to this type of analysis is 400. Thus, the filters for the extraction of differentially expressed genes were set accordingly to gain as outcome ≤ 400 genes (Supplementary Tables S1–S4). Analysis on the basis of the *Signature Tool* revealed the top 50 conditions that showed the highest similarity with the virus-attributed gene expression pattern (Supplementary Figure S1). Next, we sub-classified the top 50 outcomes into the following categories: (i) SARS-CoV- and SARS-CoV-2-related studies, studies attributed (ii) to the respiratory tract, (iii) to the gastrointestinal tract, (iv) to the liver as well as (v) all other studies that were not part of the above listed categories. In addition to our main focus on the respiratory and the gastrointestinal systems, we included liver as a separate category given the multitude of liver-attributed conditions identified among the top 50 outcomes (Supplementary Figure S1). 

**Figure 4:**
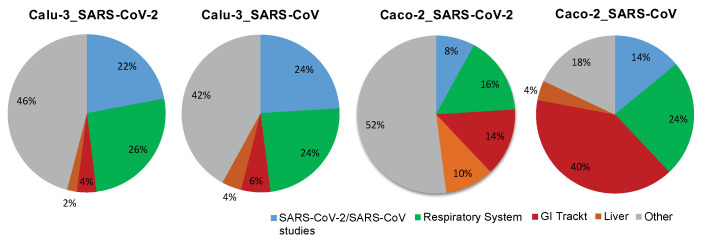
The tissue type-specific nature of cellular responses to infection with SARS-CoV-2 or SARS-CoV. The pie chart illustrates the outcome of a comprehensive analysis, including (i) the extraction of differentially expressed genes, (ii) the assessment of conditions with similarity to the differentially expressed genes-based signature, and (iii) the alignment with the listed categories. The final outcome is shown in the form of pie charts. Color code: *blue*, SARS-CoV-2 and SARS-CoV-attributed studies; *green*, studies attributed to the respiratory system; *red*, studies attributed to the gastrointestinal (GI) tract; *orange*, studies attributed to the liver; *grey*, all other studies that are not part of the above-described categories. Included into the analysis are Calu-3 cells, of lung origin, and Caco-2 cells, of colonic origin, infected with SARS-CoV-2 or SARS-CoV (time point: 24 h post infection). The underlying GENEVESTIGATOR-based analyses were performed, and the corresponding data were extracted in September 2020.

The overall results of the analysis performed within *Module 1* are illustrated by pie charts (Figure 4). The data demonstrate that for both SARS-CoV- and SARS-CoV-2-infected cells, in the case of Calu-3 the preponderance is given to conditions attributed to the respiratory system and in the case of Caco-2 cells to those of the gastrointestinal tract and the liver (Figure 4). Thus, the response to SARS-CoV or SARS-CoV-2 observed in the cell-based model using Calu-3 cells showed similarity to studies with other viruses, such as the influenza virus, parainfluenza virus, or respiratory syncytial virus (all applied on cells of the respiratory system), as well as to cells attributed to disease conditions of the respiratory system such as pulmonary tuberculosis and asthma [28,29,30]. Differential to this was the outcome for Caco-2 infected with SARS-CoV or SARS-CoV-2. Here we observed similarities to disease conditions attributed to the gastrointestinal tract and the liver, including Crohn’s disease, ulcerative colitis, and nonalcoholic steatohepatitis [31,32,33]. 

Taken together, the results indicate that the cellular response to the virus on the gene expression level is tissue type-specific, in comparison with conditions attributed to the respiratory and the gastrointestinal system. Of note, among the differentially expressed, strongly up-regulated genes for the condition Calu-3_SARS-CoV-2 and Calu-3_SARS-CoV, we find genes associated with the cellular interferon system; this is not the case for the conditions attributed to Caco-2_SARS-CoV-2 and Caco-2_SARS-CoV (Supplementary Figure S1). 

### 2.5. Singularity and Commonality in Canonical Pathways and Upstream Regulators Activated in Response to SARS-CoV-2 and SARS-CoV in Lung and Colon Cell Models

**Figure 5:**
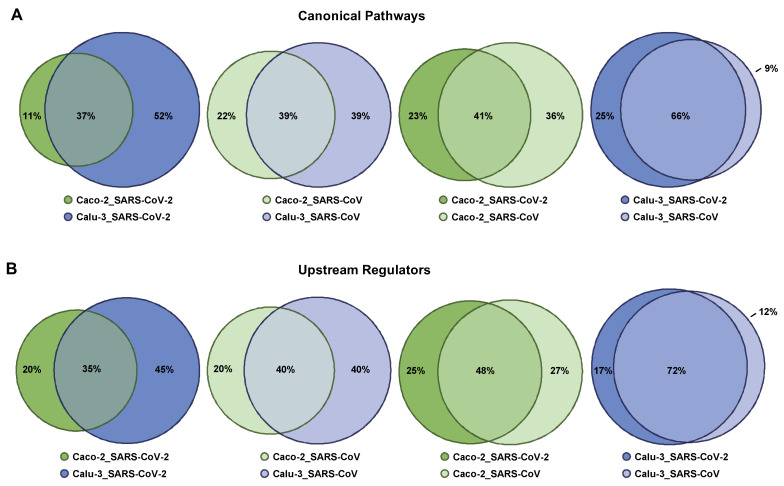
Overlap in Canonical Pathways and Upstream Regulators attributed to Calu-3 cells and Caco-2 cells infected with SARS-CoV-2 and SARS-CoV. R-based comparisons are visualized by scaled Venn diagrams, where circles are sized proportionally. The color code indicates the condition—the cell type (Caco-2 or Calu-3) and the virus type (infection with SARS-CoV-2 or SARS-CoV). Differentially expressed genes for each condition, obtained via GENEVESTIGATOR-based analysis, were taken as the basis for IPA-based Core Analysis to dissect the corresponding Canonical Pathways and Upstream Regulators. Comparison of (**A**) Canonical Pathways and (**B**) Upstream Regulators is shown. The rounding up or rounding off of the values was performed by the R program automatically, according to standard mathematical rules; therefore, the total sum of the three values in a Venn diagram might not be equal to 100.

In *Module 2* (Figure 3), we used the lists with the differently expressed genes attributed to Calu-3 and Caco-2 infected with SARS-CoV-2 or SARS-CoV to elucidate the singularity and/or commonality in response to SARS-CoV and SARS-CoV-2 in lung and colon cell models. We subjected these gene lists to the IPA tool and ran the Core Analysis (gene lists are given in Supplementary Tables S5–S8). As an outcome, we obtained the Canonical Pathways and Upstream Regulators significantly associated with the differentially expressed genes for a given condition (Supplementary Tables S9–S16). To analyze and interpret the results and to find the singularity and/or commonality, we made use of VENNY-based comparisons and R-based visualization on the basis of Venn Diagrams; the unique and overlapping Canonical Pathways and Upstream Regulators are summarized in Supplementary Tables S17–S24. Visualization of the overlaps for Canonical Pathways and Upstream Regulators is given in Figure 5A,B. The primary focus was the comparison between the lung-derived Calu-3 cells and colon-derived Caco-2 cells, infected with a given virus type. The second question was the alignment of outcomes attributed to SARS-CoV-2 and SARS-CoV in a given cell line model. 

In respect of Canonical Pathways and Upstream Regulators, on the basis of the entire IPA-based outcomes, we found overlaps classified by us as moderate (30% to 50%) when comparing the conditions (i) Caco-2_SARS-CoV-2 and Calu-3_SARS-CoV-2, (ii) Caco-2_SARS-CoV and Calu-3_SARS-CoV, and (iii) Caco-2_SARS-CoV-2 and Caco-2_SARS-CoV. Comparison of the conditions (iv) Calu-3_SARS-CoV-2 and Calu-3_SARS-CoV was characterized by a considerable degree of overlap (>50%) (Figure 5A,B). 

Next, we focused on the top five IPA-based outcomes. Among the top five Canonical Pathways attributed to (i) Caco-2_SARS-CoV-2, we found TNFR2 Signaling, MSP-RON Signaling In Cancer Cells Pathway, Aryl Hydrocarbon Receptor Signaling, Acute Phase Response Signaling, and Hepatic Fibrosis Signaling Pathway; in (ii) Calu-3_SARS-CoV-2 we found Superpathway of Cholesterol Biosynthesis, Dendritic Cell Maturation, Neuroinflammation Signaling Pathway, Antigen Presentation Pathway, and Role of Pattern Recognition Receptors in Recognition of Bacteria and Viruses; in (iii) Caco-2_SARS-CoV we found Molecular Mechanisms of Cancer, Tec Kinase Signaling, ERK/MAPK Signaling, Tumor Microenvironment Pathway, and Aryl Hydrocarbon Receptor Signaling; and in (iv) Calu-3_SARS-CoV we found the Neuroinflammation Signaling Pathway, TNFR2 Signaling, Death Receptor Signaling, Activation of IRF by Cytosolic Pattern Recognition Receptors, and Dendritic Cell Maturation (Supplementary Tables S9–S12). The top five Canonical Pathways attributed to Caco-2_SARS-CoV-2 showed overlap with Canonical Pathways attributed to three other conditions: Calu-3_SARS-CoV-2, Caco-2_SARS-CoV, and Calu-3_SARS-CoV (Table 1). The same applies for Caco-2_SARS-CoV (Table 1). In contrast, for the condition Calu-3_SARS-CoV-2, we found a strong overlap of the top five Canonical Pathways with those attributed to Calu-3_SARS-CoV and only minor overlap for the remaining two conditions (Table 1). For the condition Calu-3_SARS-CoV, we observed a strong overlap of the top five Canonical Pathways with those attributed to Calu-3_SARS-CoV-2 and a partial overlap for the conditions Caco-2_SARS-CoV-2 and Caco-2_SARS-CoV (Table 1). Taken together, this analysis revealed that the top Canonical Pathways triggered in Caco-2 cells in response to both viruses, SARS-CoV-2 and SARS-CoV, show commonality. Furthermore, those Canonical Pathways were found to be linked to the infection of Calu-3 cells with SARS-CoV-2 and SARS-CoV. By contrast, the top Canonical Pathways associated with the infection of Calu-3 cells with SARS-CoV-2 (and to a minor extent with SARS-CoV) are diverse from those triggered in Caco-2 cells upon infection with both viruses. 

Among the top five Upstream Regulators attributed to (i) Caco-2_SARS-CoV-2 we found PDGF BB, TNF, PD98059 (MEK1/2 inhibitor), U0126 (MEK1/2 inhibitor), and beta-estradiol; in (ii) Calu-3_SARS-CoV-2 we found lipopolysaccharide, IFNG, poly rI:rC-RNA, Interferon alpha, and TNF; in (iii) Caco-2_SARS-CoV we found PDGF BB, U0126, PD98059, beta-estradiol, and TP63; and in (iv) Calu-3_SARS-CoV we found Interferon alpha, lipopolysaccharide, poly rI:rC-RNA, TNF, and IFNG (Supplementary Tables S13–S16). The top five Upstream Regulators attributed to Caco-2_SARS-CoV-2 overlapped with the corresponding Upstream Regulators attributed to Calu-3_SARS-CoV-2, Caco-2_SARS-CoV, and Calu-3_SARS-CoV, although they were at varying positions within the corresponding lists (Table 2). Of note, the high similarity of Caco-2_SARS-CoV-2 and Caco-2_SARS-CoV was revealed given that the top five Upstream Regulators of the first are within the top 10 positions of the second. A similar outcome was found for the condition Calu-3_SARS-CoV-2—overlaps with corresponding Upstream Regulators attributed to Caco-2_SARS-CoV-2, Caco-2_SARS-CoV, and Calu-3_SARS-CoV (Table 2), and they showed very close similarity to Calu-3_SARS-CoV. One thing to highlight is the Upstream Regulator Interferon alpha, which was found at position 4 for Calu-3_SARS-CoV-2 and position 1 for Calu-3_SARS-CoV, while it was identified only at positions 465 and 644 for Caco-2_SARS-CoV-2 and Caco-2_SARS-CoV, respectively (Table 2). This is in line with our previous finding (discussed in the sub-chapter *Tissue-specific response to SARS-CoV and SARS-CoV-2*) that the virus-triggered response in Calu-3 but not in Caco-2 is strongly linked to the interferon system. When analyzing the top five Upstream Regulators attributed to Caco-2_SARS-CoV, we observed a strong overlap with the outcomes for Caco-2_SARS-CoV-2. Furthermore, dissecting the top five Upstream Regulators attributed to Calu-3_SARS-CoV revealed a complete overlap, with the top five Upstream Regulators attributed to Calu-3_SARS-CoV-2 only with the individual Upstream Regulators to be ranked in a different order among the top five outcomes (Table 2). Overall, these data suggest that in a particular cell line—of colon or lung origins—both viruses show similarity in the cellular response in respect of Upstream Regulators; on the contrary, there are cell type-specific regulators that differed for Caco-2 and Calu-3.

### 2.6. The Cell Type-Specific Gene Signatures

**Figure 6:**
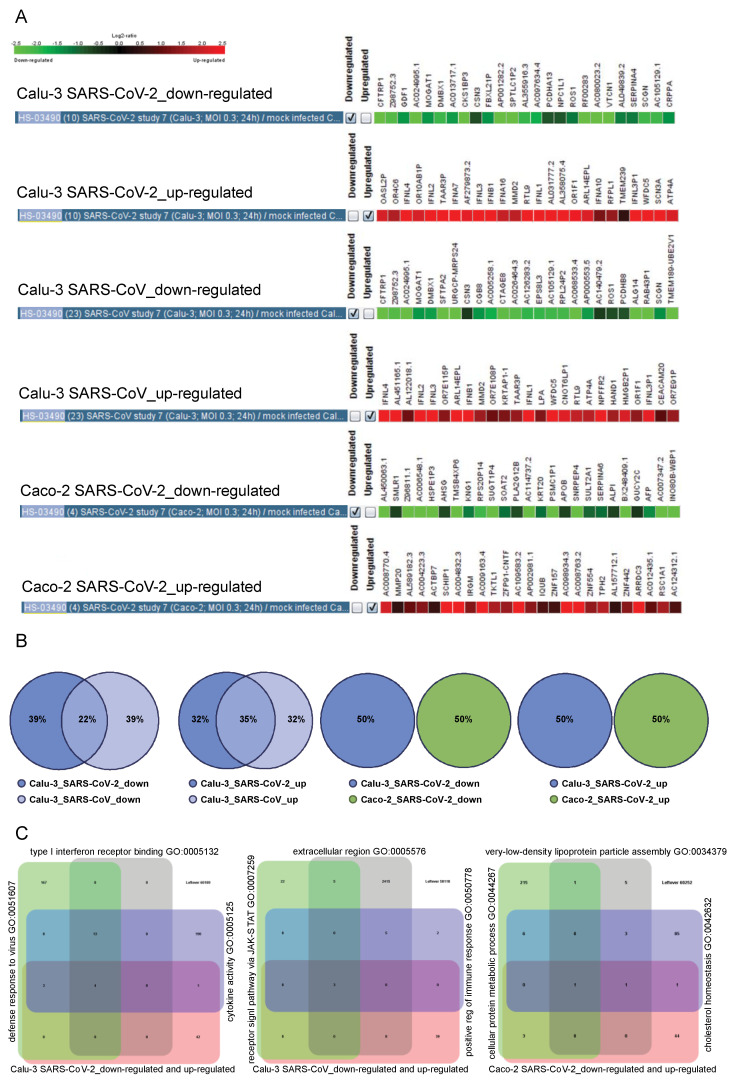
The 25-gene specific signatures for Calu-3 treated with SARS-CoV-2 or SARS-CoV and Caco-3 treated with SARS-CoV-2. (**A**) GENEVESTIGATOR-based analysis identified the 25-gene specific signatures of down-regulated and up-regulated genes for the indicated conditions. (**B**) R-based comparisons are visualized by scaled Venn diagrams. The color code indicates the condition—the cell type (Caco-2 or Calu-3) and the virus type (infection with SARS-CoV-2 or SARS-CoV). The rounding up or rounding off of the values was performed by the R program automatically, according to standard mathematical rules; therefore the total sum of the three values in a Venn diagram might not be equal to 100. (**C**) The GENEVESTIGATOR-based analysis aligned the genes that are part of the signatures shown in (**A**) to the biological process using the *Gene Set Enrichment* Tool. Thereby, the input gene set (the corresponding specific 50-gene signature covering the down-regulated and up-regulated genes for a given condition) was compared to the background collection of gene sets on the basis of the Gene Ontology categories. The outcome was visualized by Venn diagrams consisting of the input gene set, shown in *rose*, and the top three outcomes with the highest similarity, shown in different colors. The numbers in the grid indicate either the number of genes unique in a particular gene set or the number of overlapping genes. Around the Venn diagram (left, up, right), the names of the top three outcomes are given. The underlying GENEVESTIGATOR-based analyses were performed, and the corresponding data were extracted in April 2021.

Complementary knowledge was intended to be gained by us within *Module 3* of the UNCOVIDING approach (Figure 3). We utilized the power of GENEVESTIGATOR by a compendium-wide analysis to extract those genes representing the cell type-specific gene signatures characteristic for Calu-3 cells infected with SARS-CoV-2 (or SARS-CoV) and for Caco-2 cells infected with SARS-CoV-2. The underlying algorithm from the *Gene Search Tool* within the GENEVESTIGATOR platform is used to identify genes specifically expressed in a pre-defined biological context. The condition of interest (such as Calu-3 cells infected with SARS-CoV-2) was compared against a wide variety of other conditions, named as base (all mRNASeq samples available in GENEVESTIGATOR, n = 4084 perturbations, at the date of analysis). As outcome, genes showing the most specific expression in the condition of interest were identified, with no/low expression in the base conditions. The exported specific signatures were composed of 25 up-regulated genes and 25 down-regulated genes (named as the 50-gene signature) (Figure 6A and Supplementary Figure S2). Comparative analysis revealed an overlap of 9 (22%) down-regulated and 13 (35.1%) up-regulated genes between the Calu-3_SARS-CoV-2 and the Calu-3-SARS-CoV conditions (Figure 6B and Supplementary Tables S25 and S26). When aligning the data for Calu-3_SARS-CoV-2 and Caco-2_SARS-CoV-2, we identified no overlap, neither in down-regulated nor in up-regulated genes (Figure 6B and Supplementary Tables S27 and S28). This finding further supports the specificity of the dissected gene signatures. 

To gain insight into the potential biological function of genes composing the specific signatures (genes are described in detail in Supplementary Tables S29–S34), we next performed *Gene Set Enrichment* analysis within the GENEVESTIGATOR platform (Figure 6C). The top three conditions identified when analyzing the 50-gene Calu-3_SARS-CoV-2-attributed specific signature are “Cytokine Activity” (GO:0005125), “Defense Response to Virus” (GO:0051607), and “Type I Interferon Receptor Binding” (GO:0005132); for the 50-gene Calu-3_SARS-CoV-attributed specific signature, this includes “Positive Regulation of Immune Response” (GO:0050778), “Receptor Signaling Pathway, vis. JAK-STAT” (GO:0007259), and “Extracellular Region” (GO:0005576). Of note, for these two conditions, the outcome is dominated by biological functions attributed to the cellular immune response (Supplementary Tables S35 and S36). Different from those are the conditions identified when analyzing the 50-gene Caco-2_SARS-CoV-2-attributed specific signature. Here, biological functions associated with lipid metabolism are dominating (Supplementary Table S37). This includes among the top three conditions such categories as “Very-low-density Lipoprotein Particle Assembly” (GO:0034379), “Cellular Protein Metabolic Process” (GO:0044267), and “Cholesterol Homeostasis” (GO:0042632) (Figure 6C). To summarize, the findings clearly demonstrated that, for cellular models of lung and colon origins infected with SARS-CoV-2, the specific gene signatures are composed of distinct sets of genes, which are in turn associated with distinct cellular events and responses. 

### 2.7. The Road Map of APOBECs in Caco-2 and Calu-3 Models and the Upregulation of APOBEC3G

**Figure 7:**
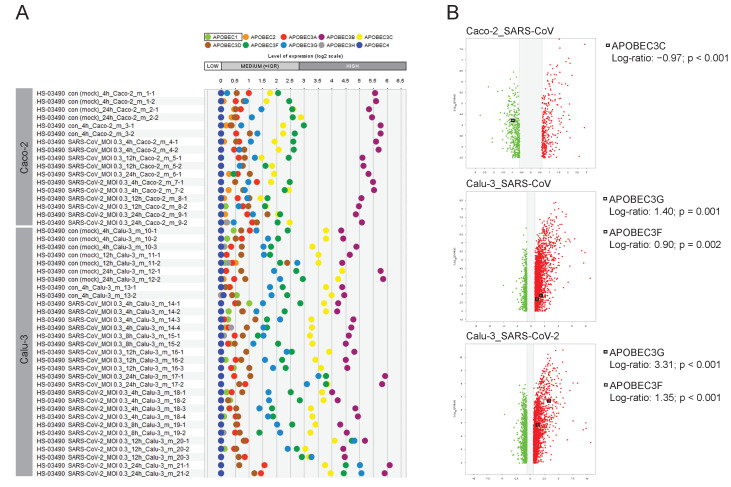
Expression map of APOBECs in Caco-2 cells and Calu-3 cells and their regulation on the transcriptional level upon infection with SARS-CoV-2 or SARS-CoV. Here the GENEVESTIGATOR-based analysis strategy is applied with the focus given to a particular gene set, the APOBECs. (**A**) The expression pattern of 10 *APOBECs* across the individual conditions is shown by scatterplots on the basis of GENEVESTIGATOR analysis of the mRNAseq dataset GSE148729 across Caco-2 and Calu-3 cells. The levels of expression of mock-infected cells and those infected with SARS-CoV or SARS-CoV-2 viruses are shown (various time points upon infection are given as log2 transformed values, sub-divided into low, medium, and high expression according to GENEVESTIGATOR). (**B**) Volcano plots illustrate the differentially expressed genes for a given condition; only conditions where APOBEC(s) were found among the differentially expressed genes are indicated. Squares indicate the position of the corresponding APOBEC; the Log-ratios and *p*-values are given. GENEVESTIGATOR-based analyses were performed, and the corresponding data were extracted in September 2020 and March 2022.

Of particular interest for us was the continuation of our research work linking the APOBECs and SARS-CoV-2 [21]. To do so we dissected the expression pattern of all 10 family members of the APOBEC family in Caco-2 and Calu-3 cells upon infection with SARS-CoV-2 or SARS-CoV. The outcome illustrating the expression profiles is given in Figure 7A. Thereby, we found differential expression patterns of the individual APOBECs. For Caco-2 we observed no expression of *APOBEC4*. Among the genes that show low/moderate expression, we found *APOBEC1*, *APOBEC2*, *APOBEC3A*, *APOBEC3D*, *APOBEC3H*, and *APOBEC3G*. The genes *APOBEC3C* and *APOBEC3F* were characterized by moderate mRNA expression levels. Especially important to highlight is the *APOBEC3B* family member that showed the highest expression levels in Caco-2 cells. The APOBEC’s road map in Calu-3 was found to be distinct. Thus, *APOBEC4* and additionally *APOBEC2* were not detected in Calu-3. As for Caco-2, *APOBEC1*, *APOBEC3A*, *APOBEC3D*, and *APOBEC3H* were characterized by a low/moderate expression. In contrast to Caco-2, *APOBEC3G* showed moderate expression in Calu-3. *APOBEC3F* was found to be expressed at the moderate/high level and *APOBEC3C* showed high expression in Calu-3 cells; both genes were characterized by moderate mRNA levels in Caco-2 cells. Interestingly, *APOBEC3B* in Calu-3 cells also showed the highest expression level among APOBEC family members. We furthermore addressed the question of whether the regulation of the expression levels of the APOBECs take place upon infection (Figure 7B). We found *APOBEC3C* among the differentially expressed genes when comparing Caco-2 cells infected with SARS-CoV to the mock-infected control. Thereby, *APOBEC3C* (Log-ratio: −0.97; *p* < 0.001) was downregulated. For the condition where Caco-2 cells were infected with the SARS-CoV-2 virus, APOBECs were not among the differentially expressed genes. For Calu-3 cells, for both viruses, we found upregulation of *APOBEC3G* (Calu-3_SARS-CoV: Log-ratio: 1.40; *p* = 0.001 and Calu-3_SARS-CoV-2: Log-ratio: 3.31; *p* < 0.001) and *APOBEC3F* (Calu-3_SARS-CoV: Log-ratio: 0.90; *p* = 0.002 and Calu-3_SARS-CoV-2: Log-ratio: 1.35; *p* < 0.001). These data revealed strong upregulation of *APOBEC3G*, encoding the host anti-virus restriction factor upon infection of Calu-3 cells with SARS-CoV-2. 

## 3. Discussion

The COVID-19 pandemic, being a serious life-threatening challenge, at the same time led to intensive research and accumulation of multifaceted data and information, which upon interpretation and judgment by scientists and clinical specialists formed the essential basis for anti-COVID developments and clinical recommendations. The herein presented and implemented UNCOVIDING approach for comprehensive analysis of large transcriptomic datasets enables extraction of novel information on the complex interplay of SARS-CoV-2 and host that might be translated into applied knowledge. A current limitation of the UNCOVIDING workflow is that its transcriptomic analysis can be applied only one platform at a time, such as microarray, mRNASeq, or singe-cell mRNASeq. The reason for this is because individual measurements are not easily comparable across platforms due to technological differences. Although in the current study we focused on extrapulmonary manifestations in the gastrointestinal tract, the UNCOVIDING approach can be further applied to dissect the SARS-CoV-2-driven pathomechanisms in other virus-infected and -affected cell types, tissues, and organs. This is furthermore of importance in the light of newly appearing variants of SARS-CoV-2, which might trigger other symptoms by shifting the viral preference to other ACE2/TMPRSS2-positive tissues.

The herein compendium-wide analysis of the expression patterns of major entry factors, ACE2 and TMPRSS2, revealed strong expression in the multitude of anatomical parts attributed to the entire gastrointestinal tract, from the upper to the lower parts. This implies a potential additional virus entry site—the mouth mucosa. Involvement of the gastrointestinal system is among the major extrapulmonary manifestations of disease, and this was already documented early in the pandemic by the determination of SARS-CoV-2 viral RNA in fecal samples from patients with COVID-19 [34,35]. In respect of the intestinal tract, an active replication of the SARS-CoV-2 was further demonstrated [36]. 

Among the major findings of this study is the uncovering of liver- and lipid metabolism-associated responses preferentially linked to the infection of the gastrointestinal system. Thus, within the first Module of the UNCOVIDING approach we identified multiple liver-associated conditions that showed similarity to molecular events attributed to Caco-2 cells infected with SARS-CoV-2. This is in line with the COVID-19-associated clinical manifestations, which were shown to include lowered levels of low-density lipoproteins (LDL), high-density lipoproteins (HDL), and blood cholesterol in patients with COVID-19 [37]. Besides this, the entry of SARS-CoV-2 into the host cell was found to be linked to cholesterol-enriched lipid rafts [37]. Furthermore, based on the accumulated evidence, the patient’s lipid profile was nominated as a marker of disease severity. More concrete, high triglycerides and low HDL levels can be used as predictive markers associated with a severe course of COVID-19 [38]. Our discovery is furthermore supported by the findings obtained within the third module of the systems biology-based integrative analysis. There, a compendium-wide alignment that was performed across a multitude of mRNASeq datasets (>19,000) revealed the 50-gene Caco-2_SARS-CoV-2-attributed specific signature. The biological functions and cellular events that are interrelated with this specific signature are strongly associated with lipid metabolism, including the processes linked to cholesterol homeostasis. Taken into account that extrapulmonary viral infections, including ones in the gut, as well as the virus-induced consequences of those are more difficult to diagnose and track in human patients, the monitoring of blood cholesterol levels and/or the above listed parameters from lipid profile might represent an important direction in healthcare during the course of COVID-19 and, furthermore, as part of the clinical investigations performed in patients with Post COVID-19 Syndrome. 

Valuable insights into singularity and commonality were gained by detailed analyses of IPA-based outcomes. Here, our study identified that in Caco-2 cellular models the Upstream Regulators are strongly focused on MAPK/MEK/ERK signaling cascades. The effect is Caco-2-specific thereby reflecting the potential abnormalities taking place in the gut. Among the cellular responses linked to MEK/ERK are those triggered by growth factors (including PDGF BB, position 1, Table 2), typically driving migration, proliferation, and survival of cells, and by pro-inflammatory mediators (including TNF, position 2, Table 2) as a complementary arm to the NFkappaB signaling [39,40,41]. A further link to MAPK/MEK/ERK signaling is the finding of MEK1/2 modulators among the top Upstream Regulators for Caco-2 (in contrast to Calu-3) for both viruses. Taking into account the multitude of transcription factors downstream of MEK/EKR, in particular the pluripotent transcription factors of the EGR family, this suggests a virus-mediated modulation of EGR-driven transcriptional programs 24 h post SARS-CoV-2 infection. In line with this, EGR1 is among the top differentially expressed genes. It is interesting to note that EGR1 was recently identified by a genome-wide clustered, regularly interspaced short palindromic repeats CRISPR-associated protein 9, CRISPR-Cas9, knockout screen as a critical factor for SARS-CoV-2 infection of cells with high viral load [42]. On the contrary, the virus-triggered response in Calu-3 is strongly linked to the interferon system. This illustrates that the well-known “classical” virus-attributed molecular events were found to be characteristic for cells of lung origin. 

The study additionally attracts attention to the APOBEC family and their intrinsic antiviral potential. We were among the first who showed tissue- and cell type-specific gene expression signatures of the individual APOBECs and emphasized the necessity to dissect the patient-specific antiviral cell state attributed to the APOBECs as a clinically relevant scenario for SARS-CoV-2 infection [21]. In continuation, we herein have brought attention to APOBEC3G as an antiviral factor being upregulated as part of the host defense machinery. In this respect, it is important to note that APOBEC3G was previously found to target replication of other types of viruses, including mumps, measles, and respiratory syncytial viruses [43,44]. Moreover, APOBEC3G is one of the critical antiviral factors restricting HIV-1 infection [45]. The molecular mechanisms by which APOBECs may contribute to host defense in SARS-CoV-2-targeted organs such as lung versus colon have not yet been elucidated. Novel patient stratification strategies based on the expression patterns of APOBECs might be identified in follow-up studies. 

The novelty and originality of the study is given by the implementation of a multi-modular comprehensive analysis strategy that enabled the dissection of cell type-specific disease-associated molecular events. The obtained findings are complementary to the information gained by Wyler et al. [22] and open new perspectives for the uncovering of prognostic markers linked to extrapulmonary manifestations and disease severity and potential supportive treatment options. 

## 4. Materials and Methods

### 4.1. Comprehensive Analysis of Transcriptomic Data

Compendium-wide analyses were performed using the GENEVESTIGATOR platform (https://genevestigator.com/, accessed on 28 August 2022). GENEVESTIGATOR is a database and analysis platform for manually curated and publicly available transcriptomic datasets, including microarrays and mRNASeq datasets. The cornerstone of this study is the analysis of the GSE148729 dataset. Detailed experimental setup is described in [22]. In short, the in vitro cell-based study includes the analysis of three epithelial cell lines assessed via expression profiling by high throughput sequencing: Calu-3 cells, a lung adenocarcinoma cell line, NCI-H1299 cells, a non-small cell lung carcinoma cell line, and Caco-2 cells, a colorectal adenocarcinoma cell line. The three cell lines were infected with SARS-CoV or SARS-CoV-2 viruses and analyzed at different time points upon infection. This dataset was curated and integrated to GENEVESTIGATOR. Comprehensive analysis was carried out, not only for this particular transcriptomic dataset; it was also complemented by compendium-wide investigations by alignment with a great variety of datasets attributed to cell-based models, perturbations, and treatment conditions, as well as to a wide set of diseases. The individual analytical steps, applied tools, and the accession dates are described in detail in the corresponding sub-chapters of the Results and the corresponding Figure Legends. 

### 4.2. Core Analysis for Canonical Pathways and Upstream Regulators

We made use of the IPA tool (https://digitalinsights.qiagen.com/products-overview/discovery-insights-portfolio/analysis-and-visualization/qiagen-ipa/, accessed on 28 August 2022) and ran the Core Analysis on the basis of GENEVESTIGATOR-derived outcomes covering the differentially expressed genes for a given condition. As an outcome, we obtained Canonical Pathways and Upstream Regulators. The ranking was based on the *p*-values. Only statistically significant outcomes were taken for follow-up analyses. 

### 4.3. Comparative Analysis and Data Visualization

The comparison of various lists of genes, Canonical Pathways, and Upstream Regulators was performed using VENNY 2.1 [46]. The Venn diagrams were created using the RStudio and the eulerr package. Venn diagrams illustrate the degree of overlap; with gene sets of this size, we considered the overlap as low (<30%), moderate (30–50%), or strong (>50%).

## Figures and Tables

**Table 1:** The top five Canonical Pathways and the overlap among the conditions. The top five Canonical Pathways for the conditions “Caco-2_SARS-CoV-2”, “Calu-3_SARS-CoV-2”, “Caco-2_SARS-CoV”, and “Calu-3_SARS-CoV-2” are given. For each condition, the top five Canonical Pathways are compared with those in the remaining three conditions; the position in the corresponding list for the indicated condition is given. Only statistically significant Canonical Pathways are included in the analyses.

Top Five Canonical Pathways for Caco-2_SARS-CoV-2				
Canonical Pathway	Caco-2_SARS-CoV-2	Calu-3_SARS-CoV-2	Caco-2_SARS-CoV	Calu-3_SARS-CoV
TNFR2 Signaling	1	36	37	2
MSP-RON Signaling In Cancer Cells Pathway	2	111	16	127
Aryl Hydrocarbon Receptor Signaling	3	29	5	98
Acute Phase Response Signaling	4	18	101	38
Hepatic Fibrosis Signaling Pathway	5	24	6	16
**Top Five Canonical Pathways for Calu-3_SARS-CoV-2**				
**Canonical Pathway**	**Caco-2_SARS-CoV-2**	**Calu-3_SARS-CoV-2**	**Caco-2_SARS-CoV**	**Calu-3_SARS-CoV**
Superpathway of Cholesterol Biosynthesis	-	1	173	193
Dendritic Cell Maturation	-	2	-	5
Neuroinflammation Signaling Pathway	107	3	94	1
Antigen Presentation Pathway	-	4	-	25
Role of PRRs in Recognition of Bacteria and Viruses	-	5	148	10
**Top Five Canonical Pathways for Caco-2_SARS-CoV**				
**Canonical Pathway**	**Caco-2_SARS-CoV-2**	**Calu-3_SARS-CoV-2**	**Caco-2_SARS-CoV**	**Calu-3_SARS-CoV**
Molecular Mechanisms of Cancer	13	53	1	96
Tec Kinase Signaling	40	61	2	90
ERK/MAPK Signaling	8	161	3	128
Tumor Microenvironment Pathway	18	15	4	11
Aryl Hydrocarbon Receptor Signaling	3	29	5	98
**Top Five Canonical Pathways for Calu-3_SARS-CoV**				
**Canonical Pathway**	**Caco-2_SARS-CoV-2**	**Calu-3_SARS-CoV-2**	**Caco-2_SARS-CoV**	**Calu-3_SARS-CoV**
Neuroinflammation Signaling Pathway	107	3	94	1
TNFR2 Signaling	1	36	37	2
Death Receptor Signaling	34	13	20	3
Activation of IRF by Cytosolic PRRs	144	9	-	4
Dendritic Cell Maturation	-	2	-	5

**Table 2:** The top five Upstream Regulators and the overlap among the conditions. The top five Upstream Regulators for the conditions “Caco-2_SARS-CoV-2”, “Calu-3_SARS-CoV-2”, “Caco-2_SARS-CoV”, and “Calu-3_SARS-CoV-2” are given. For each condition, the top five Upstream Regulators are compared with those in the remaining three conditions; the position in the corresponding list for the indicated condition is given. Only statistically significant Upstream Regulators are included into the analyses.

Top Five Upstream Regulators for Caco-2_SARS-CoV-2				
Upstream Regulator	Caco-2_SARS-CoV-2	Calu-3_SARS-CoV-2	Caco-2_SARS-CoV	Calu-3_SARS-CoV
PDGF BB	1	85	1	56
TNF	2	5	9	4
PD98059	3	87	3	115
U0126	4	55	2	62
beta-estradiol	5	10	4	26
**Top Five Upstream Regulators for Calu-3_SARS-CoV-2**				
**Upstream Regulator**	**Caco-2_SARS-CoV-2**	**Calu-3_SARS-CoV-2**	**Caco-2_SARS-CoV**	**Calu-3_SARS-CoV**
lipopolysaccharide	43	1	60	2
IFNG	55	2	38	5
poly rI:rC-RNA	46	3	54	3
Interferon alpha	465	4	644	1
TNF	2	5	9	4
**Top Five Upstream Regulators for Caco-2_SARS-CoV**				
**Upstream Regulator**	**Caco-2_SARS-CoV-2**	**Calu-3_SARS-CoV-2**	**Caco-2_SARS-CoV**	**Calu-3_SARS-CoV**
PDGF BB	1	85	1	56
U0126	4	55	2	62
PD98059	3	87	3	115
beta-estradiol	5	10	4	26
TP63	41	298	5	182
**Top Five Upstream Regulators for Calu-3_SARS-CoV**				
**Upstream Regulator**	**Caco-2_SARS-CoV-2**	**Calu-3_SARS-CoV-2**	**Caco-2_SARS-CoV**	**Calu-3_SARS-CoV**
Interferon alpha	465	4	644	1
lipopolysaccharide	43	1	60	2
poly rI:rC-RNA	46	3	54	3
TNF	2	5	9	4
IFNG	55	2	38	5

## Data Availability

The original contributions presented in the study are included in the article/Supplementary Material; further inquiries can be directed to the corresponding authors.

## Supplementary Materials

The supporting information can be downloaded at: https://www.mdpi.com/article/10.3390/ijms231810451/s1.

## Abbreviations

ACE2	angiotensin-converting enzyme 2
AID	activation-induced cytidine deaminase
APOBEC	apolipoprotein B mRNA editing enzyme catalytic subunit
C-to-U	cytosine-to-uracil
COVID-19	Coronavirus disease 19
CRISPR-Cas9	clustered regularly interspaced short palindromic repeats-CRISPR-associated protein 9
EGR	early growth response
ERK	extracellular signal-regulated kinase
GI	gastrointestinal
HDL	high-density lipoproteins
IFNG	interferon gamma
IPA	Ingenuity Pathway Analysis
JAK-STAT	janus kinases-signal transducer and activator of transcription proteins
LDL	low-density lipoproteins
MAPK	mitogen-activated protein kinase
MEK	also known as MAP2K, mitogen-activated protein kinase kinase
MERS-CoV	Middle East respiratory syndrome coronavirus
mRNASeq	mRNA sequencing
MSP	macrophage-stimulating protein
NFkappaB	nuclear factor kappa-light-chain-enhancer of activated B cells
PDGF	platelet derived growth factor
RON	recepteur d’origine nantais
SARS-CoV	severe acute respiratory syndrome coronavirus
S	spike
TMPRSS2	transmembrane serine protease 2
TNF	tumor necrosis factor
TNFR2	tumor necrosis factor receptor 2
TP63	tumor protein p63
UNCOVIDING	Understanding COVID-19 by Integrative Data Mining
