# Supplementary material for: Singularity and Commonality in Response to SARS-CoV-2 in Lung and Colon Cell Models
Source: Int J Mol Sci. 2022 Sep 9;23(18):10451. doi: 10.3390/ijms231810451 (PMC9499647; doi:10.3390/ijms231810451)
Supplement: Supplementary file 1 [file ijms-23-10451-s001.zip › Supplement_Figures_Tables captions.pdf]

International Journal of *Molecular Sciences*

Supplementary Figure S1. The top 50 conditions with similarity identified by GENEVESTIGATOR on the basis of differentially expressed genes. As input, the signatures of differentially expressed genes for a given condition (Supplementary Tables S1 – S4) were used. The Signature Tool was applied to dissect the conditions, which show similarity to the signature. The conditions include "Calu-3_SARS-CoV-2", "Calu-3_SARS-CoV", "Caco-2_SARS-CoV-2", and "Caco-2_SARS-CoV".

Supplementary Figure S2. The 25-gene specific signatures for Calu-3 treated with SARS-CoV-2 or SARS-CoV and Caco-3 treated with SARS-CoV-2. The indicated condition of interest was aligned against all mRNASeq data sets available in GENEVESTIGATOR (n = 4084 perturbations). The outcomes of the analyses are shown. The GENEVESTIGATOR-based analyses were performed and the corresponding data was extracted on April 14, 2021.

Supplementary Table S1. List of differentially expressed genes for the condition "Calu-3_SARS-CoV-2 versus Calu-3_Mock". The filters for the extraction were defined as Log-ratio ≥ 2 and FDR ≤ 0.05. The number of differentially expressed genes is 385.

Supplementary Table S2. List of differentially expressed genes for the condition "Calu-3_SARS-CoV versus Calu-3_Mock". The filters for the extraction were defined as Log-ratio ≥ 2 and FDR ≤ 0.05. The number of differentially expressed genes is 315.

Supplementary Table S3. List of differentially expressed genes for the condition "Caco-2_SARS-CoV-2 versus Caco-2_Mock". The filters for the extraction were defined as Log-ratio ≥ 0.6 and FDR ≤ 0.05. The number of differentially expressed genes is 141.

Supplementary Table S4. List of differentially expressed genes for the condition "Caco-2_SARS-CoV versus Caco-2_Mock". The filters for the extraction were defined as Log-ratio ≥ 1.0 and FDR ≤ 0.05. The number of differentially expressed genes is 187.

Supplementary Table S5. List of differentially expressed genes for the condition "Calu-3_SARS-CoV-2 versus Calu-3_Mock" subjected to IPA Core Analysis. The number of differentially expressed genes is 2890.

Supplementary Table S6. List of differentially expressed genes for the condition "Calu-3_SARS-CoV versus Calu-3_Mock" subjected to IPA Core Analysis. The number of differentially expressed genes is 2343.

Supplementary Table S7. List of differentially expressed genes for the condition "Caco-2_SARS-CoV-2 versus Caco-2_Mock" subjected to IPA Core Analysis. The number of differentially expressed genes is 128.

Supplementary Table S8. List of differentially expressed genes for the condition "Caco-2_SARS-CoV versus Caco-2_Mock" subjected to IPA Core Analysis. The number of differentially expressed genes is 656.

Supplementary Table S9. List of Canonical Pathways for the condition "Calu-3_SARS-CoV-2 ". The IPA-based Core Analysis was performed on the basis of differentially expressed genes dissected for a given condition by GENEVESTIGATOR-based analysis. Only statistically significant Canonical Pathways are included (n = 275). The data are taken as the basis for R-based visualization by Venn diagrams shown in Figure 5.

Supplementary Table S10. List of Canonical Pathways for the condition "Calu-3_SARS-CoV". The IPA-based Core Analysis was performed on the basis of differentially expressed genes dissected for a given condition by GENEVESTIGATOR-based analysis. Only statistically significant Canonical Pathways are included (n = 227). The data are taken as the basis for R-based visualization by Venn diagrams shown in Figure 5.

Supplementary Table S11. List of Canonical Pathways for the condition "Caco-2_SARS-CoV-2 ". The IPA-based Core Analysis was performed on the basis of differentially expressed genes dissected for a given condition by GENEVESTIGATOR-based analysis. Only statistically significant Canonical Pathways are included (n = 147). The data are taken as the basis for R-based visualization by Venn diagrams shown in Figure 5.

Supplementary Table S12. List of Canonical Pathways for the condition "Caco-2_SARS-CoV". The IPA-based Core Analysis was performed on the basis of differentially expressed genes dissected for a given condition by GENEVESTIGATOR-based analysis. Only statistically significant Canonical Pathways are included (n = 177). The data are taken as the basis for R-based visualization by Venn diagrams shown in Figure 5.

Supplementary Table S13. List of Upstream Regulators for the condition "Calu-3_SARS-CoV-2 ". The IPA-based Core Analysis was performed on the basis of differentially expressed genes dissected for a given condition by GENEVESTIGATOR-based analysis. Only statistically significant Upstream Regulators are included (n = 5284). The data are taken as the basis for R-based visualization by Venn diagrams shown in Figure 5.

Supplementary Table S14. List of Upstream Regulators for the condition "Calu-3_SARS-CoV". The IPA-based Core Analysis was performed on the basis of differentially expressed genes dissected for a given condition by GENEVESTIGATOR-based analysis. Only statistically significant Upstream Regulators are included (n = 4984). The data are taken as the basis for R-based visualization by Venn diagrams shown in Figure 5.

Supplementary Table S15. List of Upstream Regulators for the condition "Caco-2_SARS-CoV-2 ". The IPA-based Core Analysis was performed on the basis of differentially expressed genes dissected for a given condition by GENEVESTIGATOR-based analysis. Only statistically significant Upstream Regulators are included (n = 3639). The data are taken as the basis for R-based visualization by Venn diagrams shown in Figure 5.

Supplementary Table S16. List of Upstream Regulators for the condition "Caco-2_SARS-CoV". The IPA-based Core Analysis was performed on the basis of differentially expressed genes dissected for a given condition by GENEVESTIGATOR-based analysis. Only statistically significant Upstream Regulators are included (n = 3736). The data are taken as the basis for R-based visualization by Venn diagrams shown in Figure 5.

Supplementary Table S17. Unique and overlapping Canonical Pathways for the compassion of the conditions "Caco-2_SARS-CoV-2" and "Calu-3_SARS-CoV-2". Comparative analysis was done using VENNY 2.1. Data visualization was done by R-based Venn diagrams shown in Figure 5.

Supplementary Table S18. Unique and overlapping Canonical Pathways for the compassion of the conditions "Caco-2_SARS-CoV" and "Calu-3_SARS-CoV". Comparative analysis was done using VENNY 2.1. Data visualization was done by R-based Venn diagrams shown in Figure 5.

Supplementary Table S19. Unique and overlapping Canonical Pathways for the compassion of the conditions "Caco-2_SARS-CoV-2" and "Caco-2_SARS-CoV". Comparative analysis was done using VENNY 2.1. Data visualization was done by R-based Venn diagrams shown in Figure 5.

Supplementary Table S20. Unique and overlapping Canonical Pathways for the compassion of the conditions "Calu-3_SARS-CoV-2" and "Calu-3_SARS-CoV". Comparative analysis was done using VENNY 2.1. Data visualization was done by R-based Venn diagrams shown in Figure 5.

Supplementary Table S21. Unique and overlapping Upstream Regulators for the compassion of the conditions "Caco-2_SARS-CoV-2" and "Calu-3_SARS-CoV-2". Comparative analysis was done using VENNY 2.1. Data visualization was done by R-based Venn diagrams shown in Figure 5.

Supplementary Table S22. Unique and overlapping Upstream Regulators for the compassion of the conditions "Caco-2_SARS-CoV" and "Calu-3_SARS-CoV". Comparative analysis was done using VENNY 2.1. Data visualization was done by R-based Venn diagrams shown in Figure 5.

Supplementary Table S23. Unique and overlapping Upstream Regulators for the compassion of the conditions "Caco-2_SARS-CoV-2" and "Caco-2_SARS-CoV". Comparative analysis was done using VENNY 2.1. Data visualization was done by R-based Venn diagrams shown in Figure 5.

Supplementary Table S24. Unique and overlapping Upstream Regulators for the compassion of the conditions "Calu-3_SARS-CoV-2" and "Calu-3_SARS-CoV". Comparative analysis was done using VENNY 2.1. Data visualization was done by R-based Venn diagrams shown in Figure 5.

Supplementary Table S25. Unique and overlapping genes composing the specific 25-gene signatures for the compassion of the conditions "Calu-3_SARS-CoV-2_down-regulated" and "Calu-3_SARS-CoV_down-regulated". Comparative analysis was done using VENNY 2.1. Data visualization was done by R-based Venn diagrams shown in Figure 6B.

Supplementary Table S26. Unique and overlapping genes composing the specific 25-gene signatures for the compassion of the conditions "Calu-3_SARS-CoV-2_up-regulated" and "Calu-3_SARS-CoV_up-regulated". Comparative analysis was done using VENNY 2.1. Data visualization was done by R-based Venn diagrams shown in Figure 6B.

Supplementary Table S27. Unique and overlapping genes composing the specific 25-gene signatures for the compassion of the conditions "Calu-3_SARS-CoV-2_down-regulated" and "Caco-2_SARS-CoV-2_down-regulated". Comparative analysis was done using VENNY 2.1. Data visualization was done by R-based Venn diagrams shown in Figure 6B.

Supplementary Table S28. Unique and overlapping genes composing the specific 25-gene signatures for the compassion of the conditions "Calu-3_SARS-CoV-2_up-regulated" and "Caco-2_SARS-CoV-2_up-regulated". Comparative analysis was done using VENNY 2.1. Data visualization was done by R-based Venn diagrams shown in Figure 6B.

Supplementary Table S29. Genes composing the 25-gene signature specific for the condition “Calu-3_SARS-CoV-2_down-regulated”. Gene symbol, gene ID, synonyms, full name(s), information on the gene type and a short description from NCBI are provided.

Supplementary Table S30. Genes composing the 25-gene signature specific for the condition “Calu-3_SARS-CoV-2_up-regulated”. Gene symbol, gene ID, synonyms, full name(s), information on the gene type and a short description from NCBI are provided.

Supplementary Table S31. Genes composing the 25-gene signature specific for the condition “Calu-3_SARS-CoV_down-regulated”. Gene symbol, gene ID, synonyms, full name(s), information on the gene type and a short description from NCBI are provided.

Supplementary Table S32. Genes composing the 25-gene signature specific for the condition “Calu-3_SARS-CoV_up-regulated”. Gene symbol, gene ID, synonyms, full name(s), information on the gene type and a short description from NCBI are provided.

Supplementary Table S33. Genes composing the 25-gene signature specific for the condition “Caco-2_SARS-CoV-2_down-regulated”. Gene symbol, gene ID, synonyms, full name(s), information on the gene type and a short description from NCBI are provided.

Supplementary Table S34. Genes composing the 25-gene signature specific for the condition “Caco-2_SARS-CoV-2_up-regulated”. Gene symbol, gene ID, synonyms, full name(s), information on the gene type and a short description from NCBI are provided.

Supplementary Table S35. Gene Set Table for the 50-gene signature specific for the condition “Calu-3_SARS-CoV-2_down-regulated_and_up-regulated”. The genes of the input list representing the 50-gene signature was aligned to known biological processes by the use of the *Gene Set Enrichment* Tool in GENEVESTIGATOR. For this type of analysis the 25-gene signature covering the down-regulated genes for a given condition was combined with the 25-gene signatures covering the up-regulated genes for the same condition. The p-values and the false discovery rates (FDR) are indicated. Only significant outcomes ($p < 0.05$) are shown.

Supplementary Table S36. Gene Set Table for the 50-gene signature specific for the condition "Calu-3_SARS-CoV_down-regulated_and_up-regulated". The genes of the input list representing the 50-gene signature was aligned to known biological processes by the use of the *Gene Set Enrichment* Tool in GENEVESTIGATOR. For this type of analysis the 25-gene signature covering the down-regulated genes for a given condition was combined with the 25-gene signatures covering the up-regulated genes for the same condition. The p-values and the false discovery rates (FDR) are indicated. Only significant outcomes ($p < 0.05$) are shown.

Supplementary Table S37. Gene Set Table for the 50-gene signature specific for the condition "Caco-2_SARS-CoV-2_down-regulated_and_up-regulated". The genes of the input list representing the 50-gene signature was aligned to known biological processes by the use of the *Gene Set Enrichment* Tool in GENEVESTIGATOR. For this type of analysis the 25-gene signature covering the down-regulated genes for a given condition was combined with the 25-gene signatures covering the up-regulated genes for the same condition. The p-values and the false discovery rates (FDR) are indicated. Only significant outcomes ($p < 0.05$) are shown.
