# Supplementary material for: Singularity and Commonality in Response to SARS-CoV-2 in Lung and Colon Cell Models
Source: Int J Mol Sci. 2022 Sep 9;23(18):10451. doi: 10.3390/ijms231810451 (PMC9499647; doi:10.3390/ijms231810451)
Supplement: Supplementary file 1 [file ijms-23-10451-s001.zip › Supplement_Figures_Tables_combined_p285.pdf]

Calu-3_SARS-CoV-2

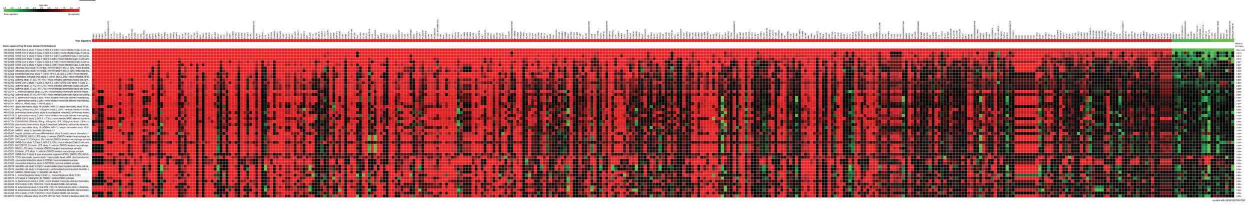

Calu-3_SARS-CoV

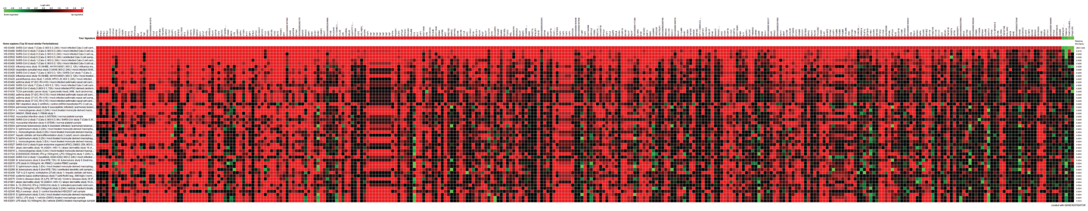

Caco-2_SARS-CoV-2

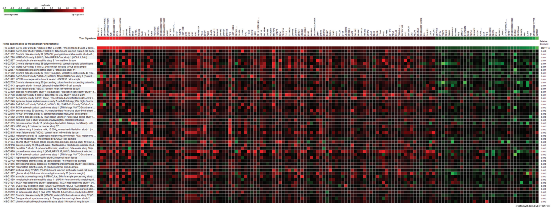

Caco-2_SARS-CoV

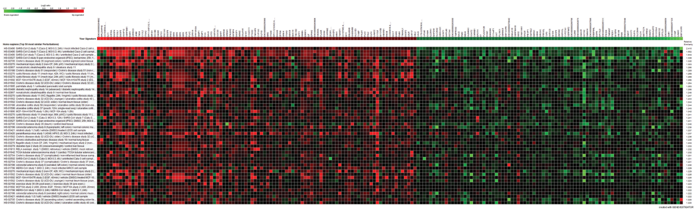

Figure S1

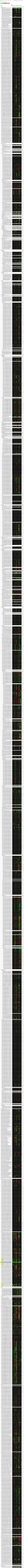

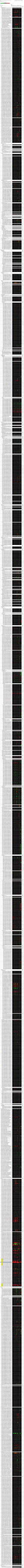

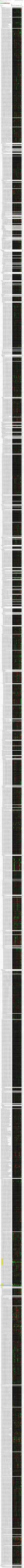

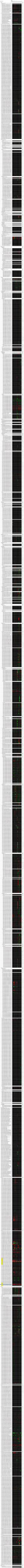

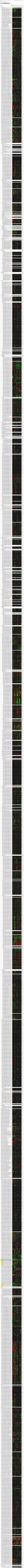

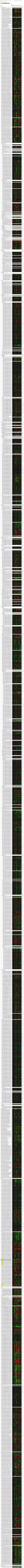

**Supplementary Table S1. List of differentially expressed genes for the condition "Calu-3_SARS-CoV-2 versus Calu-3_Mock".** The filters for the extraction were defined as Log-ratio ≥ 2 and FDR ≤ 0.05. The number of differentially expressed genes is 385.

| Gene Symbol | Gene | Log-ratio | p-value | minFDR |
|---|---|---|---|---|
| IFNB1 | ENSG00000171855 | 10.68533857 | 0.00033178 | 0.003145759 |
| IFNL1 | ENSG00000182393 | 9.853500038 | 2.68855E-05 | 0.000544627 |
| IFNL2 | ENSG00000183709 | 9.825969696 | 1.05161E-05 | 0.000297032 |
| IFNL3 | ENSG00000197110 | 9.369905472 | 3.45795E-05 | 0.000651474 |
| CXCL10 | ENSG00000169245 | 8.372399092 | 9.65434E-09 | 3.59331E-06 |
| | ENSG00000161570 | 7.588433385 | 7.40347E-06 | 0.000236878 |
| CCL5 | ENSG00000271503 | 7.588433385 | 7.40347E-06 | 0.000236878 |
| CXCL11 | ENSG00000169248 | 7.554400921 | 9.30436E-09 | 3.59331E-06 |
| IFIT2 | ENSG00000119922 | 6.995261192 | 3.48184E-08 | 8.16571E-06 |
| CH25H | ENSG00000138135 | 6.57968387 | 0.000202101 | 0.002200131 |
| IDO1 | ENSG00000131203 | 6.352767467 | 1.29973E-09 | 1.32252E-06 |
| IL6 | ENSG00000136244 | 6.102925301 | 3.21052E-07 | 3.47767E-05 |
| ATF3 | ENSG00000162772 | 5.808253765 | 6.53105E-07 | 5.25147E-05 |
| PPP1R15A | ENSG00000087074 | 5.790798187 | 1.27314E-07 | 2.04211E-05 |
| RSAD2 | ENSG00000134321 | 5.769803286 | 2.86237E-11 | 4.97444E-07 |
| IFIT3 | ENSG00000119917 | 5.727488995 | 2.41532E-10 | 1.01993E-06 |
| OASL | ENSG00000135114 | 5.510419846 | 5.42505E-09 | 2.79905E-06 |
| SLC15A3 | ENSG00000110446 | 5.435538292 | 2.44546E-08 | 6.30868E-06 |
| BST2 | ENSG00000130303 | 5.330863953 | 4.54738E-08 | 1.02728E-05 |
| ZBP1 | ENSG00000124256 | 5.3059057 | 3.87088E-07 | 3.85512E-05 |
| MT2A | ENSG00000125148 | 5.268557787 | 1.07685E-08 | 3.75711E-06 |
| GBP4 | ENSG00000162654 | 5.114412785 | 1.82952E-09 | 1.53072E-06 |
| SCN3A | ENSG00000153253 | 5.105288163 | 2.84559E-05 | 0.000565557 |
| KLF4 | ENSG00000136826 | 5.054516792 | 1.85614E-06 | 9.80554E-05 |
| TRIM22 | ENSG00000132274 | 4.971087933 | 4.256E-09 | 2.53371E-06 |
| ETV7 | ENSG00000010030 | 4.902749658 | 3.26317E-07 | 3.50019E-05 |
| ISG20 | ENSG00000172183 | 4.779579163 | 1.65327E-08 | 4.92118E-06 |
| CMPK2 | ENSG00000134326 | 4.760830164 | 2.71155E-09 | 1.86263E-06 |
| LAMP3 | ENSG00000078081 | 4.753958702 | 6.48784E-10 | 1.24669E-06 |
| FAP | ENSG00000078098 | 4.699071974 | 7.86588E-07 | 5.8142E-05 |
| HLA-F | ENSG00000229698 | 4.692797422 | 1.91032E-06 | 0.000100064 |
| IFITM2 | ENSG00000185201 | 4.689437389 | 3.30782E-06 | 0.000140082 |
| CD274 | ENSG00000120217 | 4.653267741 | 2.44388E-07 | 2.95528E-05 |
| IFI27 | ENSG00000165949 | 4.652518272 | 1.5294E-08 | 4.64173E-06 |
| IFITM1 | ENSG00000185885 | 4.622877121 | 1.1425E-07 | 1.91381E-05 |
| IFNL3P1 | ENSG00000268510 | 4.599015713 | 8.81167E-05 | 0.001227093 |
| MX2 | ENSG00000183486 | 4.587594032 | 1.16531E-09 | 1.32252E-06 |
| IFNL4 | ENSG00000272395 | 4.55917263 | 1.13446E-05 | 0.000309423 |
| DHX58 | ENSG00000108771 | 4.555215836 | 3.2463E-07 | 3.50019E-05 |
| HERC5 | ENSG00000138646 | 4.548906803 | 1.02139E-08 | 3.65979E-06 |
| IFIT1 | ENSG00000185745 | 4.498027325 | 3.21378E-11 | 4.97444E-07 |
| NFKBIA | ENSG00000100906 | 4.497161865 | 5.66882E-07 | 4.80319E-05 |

| | | | | |
|---|---|---|---|---|
| BBC3 | ENSG00000105327 | 4.491630793 | 2.82929E-07 | 3.19658E-05 |
| IGF2 | ENSG00000167244 | 4.434125364 | 2.17386E-05 | 0.000473376 |
| IL1A | ENSG00000115008 | 4.403689384 | 2.76301E-07 | 3.16795E-05 |
| CX3CL1 | ENSG00000006210 | 4.395323753 | 7.93192E-10 | 1.24669E-06 |
| GBP5 | ENSG00000154451 | 4.36655724 | 3.07386E-06 | 0.000134784 |
| SAMD9L | ENSG00000177409 | 4.255639553 | 5.08757E-09 | 2.66942E-06 |
| APOL3 | ENSG00000128284 | 4.23366785 | 1.6678E-07 | 2.36836E-05 |
| WFDC5 | ENSG00000175121 | 4.21932888 | 2.13932E-06 | 0.00010699 |
| USP18 | ENSG00000184979 | 4.188988209 | 6.77008E-10 | 1.24669E-06 |
| BATF2 | ENSG00000168062 | 4.177585125 | 1.48354E-08 | 4.59259E-06 |
| OTUD1 | ENSG00000165312 | 4.175064564 | 2.24839E-07 | 2.81475E-05 |
| RTP4 | ENSG00000136514 | 4.160068512 | 1.81023E-07 | 2.47961E-05 |
| | ENSG00000260178 | 4.111994028 | 4.24342E-06 | 0.000165237 |
| HLA-K | ENSG00000232708 | 4.083731115 | 1.61006E-06 | 9.02943E-05 |
| IFIH1 | ENSG00000115267 | 4.078417778 | 7.32552E-10 | 1.24669E-06 |
| RGAG1,RTL9 | ENSG00000243978 | 4.052238711 | 1.75466E-05 | 0.000411507 |
| LTB | ENSG00000227507 | 4.052009881 | 1.63541E-06 | 9.12204E-05 |
| HLA-F | ENSG00000137403 | 4.044341326 | 0.000231222 | 0.002413335 |
| EPSTI1 | ENSG00000133106 | 4.033581972 | 1.42833E-08 | 4.46633E-06 |
| FAM65B,RIPOR2 | ENSG00000111913 | 3.997346103 | 4.28793E-05 | 0.0007508 |
| PMAIP1 | ENSG00000141682 | 3.963897228 | 4.46687E-07 | 4.16509E-05 |
| TNFAIP3 | ENSG00000118503 | 3.952318192 | 7.43202E-07 | 5.59672E-05 |
| OAS2 | ENSG00000111335 | 3.950830936 | 1.05985E-09 | 1.32252E-06 |
| HLA-F | ENSG00000206509 | 3.947155952 | 9.16349E-07 | 6.40348E-05 |
| HLA-F | ENSG00000237508 | 3.947155952 | 9.16349E-07 | 6.40348E-05 |
| GBP1 | ENSG00000117228 | 3.924322605 | 3.29702E-10 | 1.01993E-06 |
| SLC1A3 | ENSG00000079215 | 3.90671885 | 3.87818E-06 | 0.000155127 |
| ZC3HAV1 | ENSG00000105939 | 3.86101675 | 5.08236E-09 | 2.66942E-06 |
| HLA-F | ENSG00000235220 | 3.845899105 | 8.5728E-06 | 0.000259001 |
| ARRDC3 | ENSG00000113369 | 3.837264061 | 1.89654E-07 | 2.53065E-05 |
| IRF1 | ENSG00000125347 | 3.822230339 | 1.23764E-08 | 4.03303E-06 |
| PLAUR | ENSG00000011422 | 3.769240379 | 6.44692E-09 | 3.16117E-06 |
| PTX3 | ENSG00000163661 | 3.766088963 | 2.7109E-06 | 0.000123233 |
| ACTN2 | ENSG00000077522 | 3.764203072 | 4.31598E-06 | 0.000167012 |
| RAET1L | ENSG00000155918 | 3.748281658 | 5.71759E-06 | 0.000201365 |
| FAM46A,TENT5A | ENSG00000112773 | 3.726854563 | 3.34935E-08 | 7.97584E-06 |
| CYP1A1 | ENSG00000140465 | 3.713707685 | 4.19535E-06 | 0.000164583 |
| CALM2P2 | ENSG00000229097 | 3.702192485 | 0.00266233 | 0.015262544 |
| NLRC5 | ENSG00000140853 | 3.694151163 | 1.09478E-07 | 1.85198E-05 |
| CREB5 | ENSG00000146592 | 3.690357208 | 6.94574E-07 | 5.37548E-05 |
| GBP1P1 | ENSG00000225492 | 3.677712262 | 4.46002E-06 | 0.000170666 |
| SECTM1 | ENSG00000141574 | 3.669166327 | 1.20497E-07 | 1.97311E-05 |
| TAP1 | ENSG00000168394 | 3.619572163 | 3.53634E-09 | 2.28072E-06 |
| DND1 | ENSG00000256453 | 3.614782333 | 0.006532364 | 0.031101567 |
| NT5C3A | ENSG00000122643 | 3.578699589 | 1.18228E-10 | 7.65774E-07 |
| TNFSF13B | ENSG00000102524 | 3.563028812 | 8.52084E-06 | 0.000258354 |
| APOL6 | ENSG00000221963 | 3.551050186 | 4.06273E-10 | 1.04808E-06 |

| | | | | |
|---|---|---|---|---|
| TNF | ENSG00000228978 | 3.537700981 | 0.000604152 | 0.004864173 |
| ACKR4 | ENSG00000129048 | 3.53308773 | 6.89996E-07 | 5.36852E-05 |
| CCL20 | ENSG00000115009 | 3.501783371 | 1.50439E-07 | 2.24226E-05 |
| ISG15 | ENSG00000187608 | 3.500461578 | 1.28612E-09 | 1.32252E-06 |
| HSD17B14 | ENSG00000087076 | 3.499348998 | 1.44481E-05 | 0.000362269 |
| CSF3 | ENSG00000108342 | 3.452747971 | 3.12323E-06 | 0.000135712 |
| RASGRP3 | ENSG00000152689 | 3.446791887 | 7.21234E-08 | 1.35153E-05 |
| IRF7 | ENSG00000185507 | 3.44092989 | 1.02853E-08 | 3.65979E-06 |
| GMPR | ENSG00000137198 | 3.418964148 | 2.21783E-05 | 0.000478783 |
| HLA-B | ENSG00000223532 | 3.418859482 | 1.55088E-08 | 4.66122E-06 |
| IFNA7 | ENSG00000214042 | 3.413051605 | 5.84518E-06 | 0.000205158 |
| XAF1 | ENSG00000132530 | 3.404103279 | 1.36992E-09 | 1.32527E-06 |
| IFI35 | ENSG00000068079 | 3.399718761 | 1.89005E-10 | 9.75174E-07 |
| MXD1 | ENSG00000059728 | 3.367039442 | 2.28324E-07 | 2.81475E-05 |
| OR52K3P | ENSG00000225101 | 3.357060298 | 0.000125286 | 0.00157032 |
| SOCS1 | ENSG00000185338 | 3.349912882 | 8.77143E-07 | 6.19948E-05 |
| TAP1 | ENSG00000206297 | 3.348761082 | 2.53252E-09 | 1.79874E-06 |
| TAP1 | ENSG00000226173 | 3.348761082 | 2.53252E-09 | 1.79874E-06 |
| SERPINB2 | ENSG00000197632 | 3.348421574 | 3.38424E-08 | 7.9974E-06 |
| ZEB2 | ENSG00000169554 | 3.342930861 | 7.12508E-06 | 0.000230964 |
| TRANK1 | ENSG00000168016 | 3.33041954 | 1.80471E-08 | 5.2706E-06 |
| CASP1 | ENSG00000137752 | 3.328335166 | 8.42107E-07 | 6.03452E-05 |
| PLA2G4C | ENSG00000105499 | 3.318217278 | 3.75201E-06 | 0.000152629 |
| MAP3K8 | ENSG00000107968 | 3.315573692 | 5.25399E-08 | 1.09897E-05 |
| TAP1 | ENSG00000227816 | 3.311964989 | 2.3971E-08 | 6.28873E-06 |
| TAP1 | ENSG00000230705 | 3.311964989 | 2.3971E-08 | 6.28873E-06 |
| APOBEC3G | ENSG00000239713 | 3.310438871 | 2.29884E-07 | 2.81475E-05 |
| XXbac-BPG246D15.9,AL669918.1 | ENSG00000250264 | 3.302415252 | 4.52485E-05 | 0.000779931 |
| TAP1 | ENSG00000232367 | 3.298174381 | 9.70739E-09 | 3.59331E-06 |
| MX1 | ENSG00000157601 | 3.295430183 | 2.0182E-09 | 1.64414E-06 |
| TRAF1 | ENSG00000056558 | 3.282377183 | 2.16353E-07 | 2.7791E-05 |
| IFIT5 | ENSG00000152778 | 3.270525455 | 1.53029E-09 | 1.43556E-06 |
| OAS1,AC004551.1 | ENSG00000089127 | 3.269338608 | 1.61742E-09 | 1.47266E-06 |
| PPM1K | ENSG00000163644 | 3.263245583 | 4.19794E-07 | 4.03589E-05 |
| CITED2 | ENSG00000164442 | 3.2622509 | 6.3181E-07 | 5.14709E-05 |
| IFI16 | ENSG00000163565 | 3.256719589 | 7.21448E-09 | 3.19055E-06 |
| PSMB9 | ENSG00000242711 | 3.254411697 | 2.12406E-09 | 1.67989E-06 |
| PSMB9 | ENSG00000240508 | 3.251120567 | 2.22488E-09 | 1.67989E-06 |
| PSMB9 | ENSG00000243958 | 3.251120567 | 2.22488E-09 | 1.67989E-06 |
| HELZ2 | ENSG00000130589 | 3.245970249 | 3.13684E-10 | 1.01993E-06 |
| NOCT | ENSG00000151014 | 3.236107349 | 1.35562E-07 | 2.1195E-05 |
| TNFSF10 | ENSG00000121858 | 3.185429573 | 2.86055E-10 | 1.01993E-06 |
| IL4I1 | ENSG00000104951 | 3.154675722 | 4.05104E-06 | 0.000160574 |
| HRASLS2,PLAAT2 | ENSG00000133328 | 3.136966705 | 3.12509E-05 | 0.000608831 |
| IFITM3 | ENSG00000142089 | 3.129700184 | 1.83693E-08 | 5.31065E-06 |
| WARS | ENSG00000140105 | 3.12092495 | 6.22393E-11 | 6.42248E-07 |
| UBA7 | ENSG00000182179 | 3.116344929 | 2.1913E-07 | 2.79433E-05 |

| | | | | |
|---|---|---|---|---|
| DDX58 | ENSG00000107201 | 3.104232788 | 1.23684E-10 | 7.65774E-07 |
| HLA-B | ENSG00000232126 | 3.101418495 | 1.0934E-08 | 3.76092E-06 |
| DUSP1 | ENSG00000120129 | 3.098156929 | 2.26243E-07 | 2.81475E-05 |
| CFB | ENSG00000241253 | 3.097182512 | 8.15801E-07 | 5.94229E-05 |
| LGALS9 | ENSG00000168961 | 3.075212955 | 4.74561E-10 | 1.10255E-06 |
| | ENSG00000187990 | 3.066690922 | 9.373E-05 | 0.001284544 |
| PTGER4 | ENSG00000171522 | 3.065007925 | 2.76608E-08 | 6.85036E-06 |
| SAMD9 | ENSG00000205413 | 3.048261642 | 4.94299E-08 | 1.04808E-05 |
| UBE2L6 | ENSG00000156587 | 3.047037125 | 5.64343E-09 | 2.864E-06 |
| TAAR3,TAAR3P | ENSG00000179073 | 3.045184165 | 1.31305E-05 | 0.000339017 |
| HLA-B | ENSG00000206450 | 3.038875103 | 3.62413E-10 | 1.01993E-06 |
| CXCL2 | ENSG00000081041 | 3.009992123 | 1.11902E-06 | 7.15733E-05 |
| HLA-H | ENSG00000206341 | 3.009297848 | 7.20462E-06 | 0.00023234 |
| IL12A | ENSG00000168811 | 3.003383517 | 6.09539E-06 | 0.000209728 |
| HSPB8 | ENSG00000152137 | 2.985280156 | 7.77182E-07 | 5.77549E-05 |
| ZFP36 | ENSG00000128016 | 2.975497246 | 6.25488E-08 | 1.24837E-05 |
| ZNFX1 | ENSG00000124201 | 2.968083858 | 9.13829E-10 | 1.32252E-06 |
| JUN | ENSG00000177606 | 2.950293541 | 5.1315E-07 | 4.52581E-05 |
| RND1 | ENSG00000172602 | 2.947559237 | 1.10648E-05 | 0.000304852 |
| C4A | ENSG00000244731 | 2.941365242 | 0.001978966 | 0.012069662 |
| CEACAM1 | ENSG00000079385 | 2.937015533 | 3.79566E-09 | 2.35005E-06 |
| TIPARP | ENSG00000163659 | 2.932439804 | 2.00986E-07 | 2.64762E-05 |
| DNAJB4 | ENSG00000162616 | 2.93095088 | 1.82943E-06 | 9.7525E-05 |
| GSDMB | ENSG00000073605 | 2.917886734 | 3.27582E-08 | 7.8612E-06 |
| LMO2 | ENSG00000135363 | 2.914696932 | 2.10954E-05 | 0.000465468 |
| DDX60L | ENSG00000181381 | 2.904570103 | 2.49466E-08 | 6.3301E-06 |
| CPXM1 | ENSG00000088882 | 2.884370863 | 3.31555E-06 | 0.000140218 |
| C6orf58 | ENSG00000184530 | 2.877307057 | 1.64343E-05 | 0.000394313 |
| TYMP | ENSG00000025708 | 2.875247478 | 1.14953E-08 | 3.86803E-06 |
| FST | ENSG00000134363 | 2.868207932 | 4.59921E-08 | 1.02728E-05 |
| HLA-B | ENSG00000234745 | 2.849991798 | 5.13044E-08 | 1.08043E-05 |
| | ENSG00000261660 | 2.84534359 | 8.05434E-10 | 1.24669E-06 |
| CD68 | ENSG00000129226 | 2.841193676 | 1.23709E-08 | 4.03303E-06 |
| CD38 | ENSG00000004468 | 2.832970858 | 2.43701E-06 | 0.000115355 |
| C1orf168,FYB2 | ENSG00000187889 | 2.827637475 | 2.26715E-05 | 0.000485704 |
| TLDC2 | ENSG00000101342 | 2.814217567 | 5.93797E-06 | 0.00020724 |
| PARP10 | ENSG00000178685 | 2.808107376 | 1.19546E-09 | 1.32252E-06 |
| | ENSG00000266796 | 2.805694282 | 1.7693E-05 | 0.000412751 |
| PARP14 | ENSG00000173193 | 2.798897743 | 4.97043E-09 | 2.66942E-06 |
| THEMIS2 | ENSG00000130775 | 2.79654181 | 1.44722E-07 | 2.20697E-05 |
| IL7R | ENSG00000168685 | 2.786833927 | 4.96622E-05 | 0.000831922 |
| CSRNP1 | ENSG00000144655 | 2.773226023 | 1.32985E-06 | 8.02499E-05 |
| INHBA | ENSG00000122641 | 2.765369415 | 7.59036E-07 | 5.67572E-05 |
| CSF1 | ENSG00000184371 | 2.762135983 | 1.65605E-07 | 2.3625E-05 |
| PLEKHA4 | ENSG00000105559 | 2.7516222 | 4.8678E-08 | 1.03926E-05 |
| HLA-B | ENSG00000224608 | 2.750433922 | 3.9474E-08 | 9.05183E-06 |
| CARD16 | ENSG00000204397 | 2.74534893 | 5.81767E-05 | 0.000913738 |

| | | | | |
|---|---|---|---|---|
| SAMHD1 | ENSG00000101347 | 2.743910789 | 4.98617E-10 | 1.10255E-06 |
| CSAG3 | ENSG00000268916 | 2.743319273 | 3.2171E-05 | 0.000621671 |
| NAV2 | ENSG00000166833 | 2.74130106 | 1.26703E-06 | 7.76705E-05 |
| NT5C3AP1 | ENSG00000213492 | 2.727416158 | 2.33778E-05 | 0.000493662 |
| C4B | ENSG00000228267 | 2.720826864 | 1.2748E-05 | 0.00033219 |
| EPHA4 | ENSG00000116106 | 2.712821126 | 5.57401E-07 | 4.75393E-05 |
| HLA-F | ENSG00000204642 | 2.71192503 | 0.001845541 | 0.011394575 |
| HLA-C | ENSG00000206435 | 2.711062908 | 0.000300299 | 0.002910568 |
| NUAK2 | ENSG00000163545 | 2.701996803 | 1.30523E-06 | 7.92275E-05 |
| TLR3 | ENSG00000164342 | 2.680511475 | 1.21101E-07 | 1.97311E-05 |
| HCG4B | ENSG00000231941 | 2.674328089 | 1.36452E-05 | 0.000348144 |
| PLAC8 | ENSG00000145287 | 2.672288656 | 0.000806341 | 0.006020722 |
| PPP1R18 | ENSG00000236428 | 2.672189236 | 6.4404E-06 | 0.000216914 |
| JAK2 | ENSG00000096968 | 2.668018341 | 1.01589E-07 | 1.74717E-05 |
| KCNN1 | ENSG00000105642 | 2.655708037 | 3.43056E-06 | 0.000142742 |
| OR10AB1P | ENSG00000176716 | 2.633870929 | 4.40672E-05 | 0.000765967 |
| NCOA7 | ENSG00000111912 | 2.630745411 | 1.6887E-09 | 1.49363E-06 |
| GIMAP2 | ENSG00000106560 | 2.618235707 | 3.80115E-06 | 0.000153747 |
| CPEB3 | ENSG00000107864 | 2.616337538 | 5.37608E-06 | 0.000191691 |
| FBXO6 | ENSG00000116663 | 2.587986231 | 0.000522773 | 0.004368652 |
| IL15RA | ENSG00000134470 | 2.586229324 | 1.16691E-08 | 3.8843E-06 |
| IL10RA | ENSG00000110324 | 2.579790037 | 8.25596E-06 | 0.000253049 |
| HSH2D | ENSG00000196684 | 2.577697039 | 5.96769E-08 | 1.19962E-05 |
| C5orf56 | ENSG00000197536 | 2.573081732 | 2.53745E-06 | 0.000118479 |
| NEURL3 | ENSG00000163121 | 2.567317963 | 1.23682E-07 | 1.99819E-05 |
| IFI44 | ENSG00000137965 | 2.556812763 | 1.17517E-09 | 1.32252E-06 |
| G0S2 | ENSG00000123689 | 2.548935413 | 5.39149E-05 | 0.000874881 |
| STAT2 | ENSG00000170581 | 2.546236515 | 1.85273E-08 | 5.31065E-06 |
| CHAC1 | ENSG00000128965 | 2.538776159 | 4.61243E-06 | 0.000174556 |
| FOS | ENSG00000170345 | 2.537586689 | 1.91172E-05 | 0.000434061 |
| APOL4 | ENSG00000100336 | 2.527667284 | 2.53187E-05 | 0.000521832 |
| MB21D2 | ENSG00000180611 | 2.526196003 | 8.74283E-07 | 6.1934E-05 |
| ATP4A | ENSG00000105675 | 2.521784306 | 2.89416E-06 | 0.000129285 |
| | ENSG00000006075 | 2.520884514 | 1.51275E-05 | 0.000374058 |
| SNPH | ENSG00000101298 | 2.506401181 | 4.20573E-06 | 0.000164583 |
| NFKBIZ | ENSG00000144802 | 2.502514839 | 7.24729E-08 | 1.35153E-05 |
| DDX60 | ENSG00000137628 | 2.501703262 | 6.74633E-09 | 3.16117E-06 |
| GLCCI1 | ENSG00000106415 | 2.500458241 | 5.7891E-06 | 0.00020342 |
| RNF213,AC124319.1 | ENSG00000173821 | 2.478383064 | 7.22518E-10 | 1.24669E-06 |
| NKX3-1 | ENSG00000167034 | 2.478213787 | 0.000155771 | 0.001832846 |
| KCNV1 | ENSG00000164794 | 2.476224422 | 1.79519E-05 | 0.000416588 |
| MICB | ENSG00000231372 | 2.473977208 | 5.6367E-05 | 0.000900389 |
| TAPBPL | ENSG00000139192 | 2.470232964 | 1.13861E-06 | 7.2676E-05 |
| PLCG2 | ENSG00000197943 | 2.470173955 | 2.70777E-06 | 0.000123233 |
| HSD17B7P2 | ENSG00000099251 | 2.468547821 | 2.30544E-05 | 0.000489168 |
| RARRES3,PLAAT4 | ENSG00000133321 | 2.459376812 | 3.01584E-08 | 7.40962E-06 |
| BCL2L14 | ENSG00000121380 | 2.455078959 | 8.4369E-06 | 0.000256311 |

| | | | | |
|---|---|---|---|---|
| TTC9B | ENSG00000174521 | 2.450932354 | 0.000265768 | 0.002674976 |
| TNFRSF9 | ENSG00000049249 | 2.44412072 | 5.089E-06 | 0.000184906 |
| TRIM5 | ENSG00000132256 | 2.442986488 | 6.58865E-09 | 3.16117E-06 |
| IFI6 | ENSG00000126709 | 2.436706543 | 5.71401E-07 | 4.80319E-05 |
| SEC16B | ENSG00000120341 | 2.435987115 | 1.58971E-06 | 8.9315E-05 |
| CYLD | ENSG00000083799 | 2.433567047 | 1.02404E-06 | 6.83214E-05 |
| OR1F1 | ENSG00000168124 | 2.432582587 | 7.3316E-05 | 0.001079754 |
| TXNIP | ENSG00000265972 | 2.42911911 | 3.10653E-09 | 2.04614E-06 |
| ARL14EPL | ENSG00000268223 | 2.427643061 | 0.00033446 | 0.003158592 |
| C19orf66 | ENSG00000130813 | 2.420735836 | 2.60676E-08 | 6.56077E-06 |
| SLFN5 | ENSG00000166750 | 2.416670799 | 1.69322E-07 | 2.3826E-05 |
| NMI | ENSG00000123609 | 2.410617828 | 2.1359E-08 | 5.95383E-06 |
| IFI44L | ENSG00000137959 | 2.406975746 | 3.19664E-07 | 3.47767E-05 |
| PML | ENSG00000140464 | 2.405802727 | 6.77596E-09 | 3.16117E-06 |
| ANGPT2 | ENSG00000091879 | 2.398910105 | 9.47952E-06 | 0.000277633 |
| SP110 | ENSG00000135899 | 2.395410538 | 1.52167E-08 | 4.64173E-06 |
| FZD4 | ENSG00000174804 | 2.389435291 | 2.22495E-07 | 2.81134E-05 |
| BACH2 | ENSG00000112182 | 2.388124108 | 7.28766E-06 | 0.000234272 |
| OAS3 | ENSG00000111331 | 2.380886078 | 1.32436E-09 | 1.32252E-06 |
| FLT3LG | ENSG00000090554 | 2.377220154 | 1.74902E-05 | 0.000410581 |
| PINLYP | ENSG00000234465 | 2.364657104 | 0.000305911 | 0.002956634 |
| CXCL8 | ENSG00000169429 | 2.355402946 | 3.81048E-07 | 3.84238E-05 |
| HERC6 | ENSG00000138642 | 2.346012115 | 3.73249E-08 | 8.62288E-06 |
| HAS2 | ENSG00000170961 | 2.344397187 | 1.50921E-06 | 8.68E-05 |
| HLA-J | ENSG00000243336 | 2.343358576 | 0.000194723 | 0.002149508 |
| LST1 | ENSG00000234514 | 2.338863147 | 6.18905E-05 | 0.000960372 |
| NPIPA5 | ENSG00000183793 | 2.33178997 | 0.001242596 | 0.00836604 |
| PARP12 | ENSG00000059378 | 2.32708931 | 3.64591E-09 | 2.3034E-06 |
| PDGFRL | ENSG00000104213 | 2.317726612 | 2.04339E-05 | 0.000454433 |
| SEMA3A | ENSG00000075213 | 2.314947844 | 2.28018E-08 | 6.13806E-06 |
| ACHE | ENSG00000087085 | 2.31242013 | 1.38867E-05 | 0.000351217 |
| CFB | ENSG00000243649 | 2.309847832 | 3.36718E-05 | 0.000640674 |
| LAP3 | ENSG00000002549 | 2.308947563 | 1.1739E-09 | 1.32252E-06 |
| OPTN | ENSG00000123240 | 2.307404995 | 2.76775E-09 | 1.86263E-06 |
| TRIM21 | ENSG00000132109 | 2.302456856 | 8.86443E-09 | 3.47362E-06 |
| RNASEK-C17orf49 | ENSG00000161939 | 2.298914611 | 0.009173047 | 0.041131232 |
| TAP2 | ENSG00000223481 | 2.287401199 | 8.2465E-09 | 3.44982E-06 |
| TAP2 | ENSG00000225967 | 2.287401199 | 8.2465E-09 | 3.44982E-06 |
| PPP4R4 | ENSG00000119698 | 2.28598991 | 3.28157E-05 | 0.000629025 |
| TAP2 | ENSG00000204267 | 2.281030416 | 4.17975E-05 | 0.000739386 |
| TAP2 | ENSG00000206235 | 2.281030416 | 4.17975E-05 | 0.000739386 |
| TAP2 | ENSG00000206299 | 2.281030416 | 4.17975E-05 | 0.000739386 |
| TAP2 | ENSG00000228582 | 2.281030416 | 4.17975E-05 | 0.000739386 |
| | ENSG00000117289 | 2.280943632 | 8.46026E-07 | 6.0486E-05 |
| MMD2 | ENSG00000136297 | 2.28020215 | 7.52721E-06 | 0.000238505 |
| BIRC3 | ENSG00000023445 | 2.274824142 | 1.20315E-09 | 1.32252E-06 |
| TAP2 | ENSG00000232326 | 2.268094063 | 7.12589E-08 | 1.35153E-05 |

| | | | | |
|---|---|---|---|---|
| NPFFR2 | ENSG00000056291 | 2.267457604 | 6.86064E-05 | 0.001028498 |
| | ENSG00000269882 | 2.263358474 | 1.86907E-06 | 9.84786E-05 |
| ZNF442 | ENSG00000198342 | 2.258573294 | 0.00082696 | 0.006134725 |
| HCG27 | ENSG00000206344 | 2.247710824 | 0.002198854 | 0.013133306 |
| NCALD | ENSG00000104490 | 2.242054582 | 5.88007E-06 | 0.000205916 |
| IL32 | ENSG00000008517 | 2.239489555 | 5.98627E-07 | 4.955E-05 |
| CFAP57 | ENSG00000243710 | 2.236504972 | 1.1834E-06 | 7.47642E-05 |
| HIVEP2 | ENSG00000010818 | 2.235404015 | 2.9578E-07 | 3.32962E-05 |
| C1S | ENSG00000182326 | 2.223181725 | 5.51447E-06 | 0.000195546 |
| GTF2H4 | ENSG00000234370 | 2.221033812 | 1.98869E-06 | 0.00010233 |
| HLA-C | ENSG00000225691 | 2.217868805 | 7.83472E-09 | 3.3686E-06 |
| ZBTB20 | ENSG00000181722 | 2.217278004 | 8.40127E-05 | 0.001187231 |
| ANKRD33B | ENSG00000164236 | 2.215722084 | 2.39118E-05 | 0.000502197 |
| HLA-DOB | ENSG00000239457 | 2.199644614 | 3.18578E-05 | 0.000617547 |
| HLA-DOB | ENSG00000243612 | 2.199644614 | 3.18578E-05 | 0.000617547 |
| CNOT6LP1 | ENSG00000230183 | 2.195508987 | 8.06553E-05 | 0.001157018 |
| HLA-C | ENSG00000228299 | 2.195345879 | 1.05453E-09 | 1.32252E-06 |
| WIF1 | ENSG00000156076 | 2.19330883 | 0.000136789 | 0.001671758 |
| NUB1 | ENSG00000013374 | 2.190966606 | 4.39693E-09 | 2.56822E-06 |
| XRN1 | ENSG00000114127 | 2.190029621 | 9.52621E-08 | 1.67559E-05 |
| | ENSG00000265434 | 2.188321412 | 7.40959E-06 | 0.000236878 |
| HIST1H4H | ENSG00000158406 | 2.181166887 | 6.49759E-05 | 0.000990379 |
| GDF15 | ENSG00000130513 | 2.178212166 | 6.70659E-08 | 1.30379E-05 |
| GBP3 | ENSG00000117226 | 2.177538872 | 3.71181E-08 | 8.62288E-06 |
| KDM6A | ENSG00000147050 | 2.162010193 | 8.85295E-07 | 6.22865E-05 |
| SAA1 | ENSG00000173432 | 2.157037735 | 0.000107685 | 0.001415542 |
| AC015688.3,AC015688.4 | ENSG00000266728 | 2.149764746 | 0.002282176 | 0.0135214 |
| DTX3L | ENSG00000163840 | 2.142318726 | 1.8004E-09 | 1.53072E-06 |
| RP11-61N20.3,AC135977.1 | ENSG00000226268 | 2.138516665 | 0.000309664 | 0.002985444 |
| BTN3A3 | ENSG00000111801 | 2.137117863 | 3.42972E-07 | 3.56288E-05 |
| TRPC4 | ENSG00000133107 | 2.133843422 | 1.79328E-05 | 0.000416588 |
| SPRY2 | ENSG00000136158 | 2.132361412 | 1.50658E-07 | 2.24226E-05 |
| | ENSG00000168274 | 2.12571454 | 0.001713064 | 0.010748142 |
| COL16A1 | ENSG00000084636 | 2.125272512 | 1.03797E-05 | 0.000294252 |
| TNF | ENSG00000204490 | 2.124778487 | 6.78482E-06 | 0.000222733 |
| TNF | ENSG00000206439 | 2.124778487 | 6.78482E-06 | 0.000222733 |
| TNF | ENSG00000223952 | 2.124778487 | 6.78482E-06 | 0.000222733 |
| TNF | ENSG00000228321 | 2.124778487 | 6.78482E-06 | 0.000222733 |
| TNF | ENSG00000228849 | 2.124778487 | 6.78482E-06 | 0.000222733 |
| TNF | ENSG00000230108 | 2.124778487 | 6.78482E-06 | 0.000222733 |
| TNF | ENSG00000232810 | 2.124778487 | 6.78482E-06 | 0.000222733 |
| PTGS2 | ENSG00000073756 | 2.123975277 | 6.78073E-08 | 1.30379E-05 |
| EGR1 | ENSG00000120738 | 2.123416901 | 1.84694E-06 | 9.79483E-05 |
| CFH | ENSG00000000971 | 2.118200302 | 1.20883E-06 | 7.57524E-05 |
| PDGFA | ENSG00000197461 | 2.117143869 | 1.81933E-05 | 0.000419679 |
| ETS2 | ENSG00000157557 | 2.116069794 | 5.03789E-09 | 2.66942E-06 |
| C1R | ENSG00000159403 | 2.109933853 | 3.40899E-05 | 0.000645852 |

| | | | | |
|---|---|---|---|---|
| C4B | ENSG00000224389 | 2.108299971 | 0.00354854 | 0.019084805 |
| PA2G4P4 | ENSG00000230457 | 2.107978463 | 9.43367E-05 | 0.001291061 |
| TNFSF15 | ENSG00000181634 | 2.105346918 | 9.62333E-07 | 6.5331E-05 |
| TREX1 | ENSG00000213689 | 2.103308201 | 2.38038E-06 | 0.000114247 |
| CD74 | ENSG00000019582 | 2.101313591 | 1.9674E-08 | 5.58759E-06 |
| NEU4 | ENSG00000204099 | 2.098898858 | 7.37406E-05 | 0.00108446 |
| HLA-A | ENSG00000206505 | 2.098513126 | 6.05817E-07 | 4.97687E-05 |
| STX19 | ENSG00000178750 | 2.095807314 | 4.03021E-05 | 0.000720758 |
| PTPRZ1 | ENSG00000106278 | 2.090885818 | 8.88475E-06 | 0.000265744 |
| VAMP5 | ENSG00000168899 | 2.084590435 | 6.42293E-05 | 0.0009819 |
| PRKD2 | ENSG00000105287 | 2.084326267 | 6.47545E-09 | 3.16117E-06 |
| HSPA6 | ENSG00000173110 | 2.079734564 | 7.6504E-06 | 0.000240226 |
| AC129492.1,PER1 | ENSG00000179094 | 2.078415871 | 1.84182E-05 | 0.00042329 |
| HLA-DOB | ENSG00000241106 | 2.077943638 | 8.1774E-05 | 0.001168152 |
| HLA-DRA | ENSG00000206308 | 2.075824261 | 2.3295E-05 | 0.000492374 |
| HLA-DRA | ENSG00000230726 | 2.075824261 | 2.3295E-05 | 0.000492374 |
| HLA-DRA | ENSG00000234794 | 2.075824261 | 2.3295E-05 | 0.000492374 |
| RANBP3L | ENSG00000164188 | 2.071104778 | 5.07959E-05 | 0.00084497 |
| NR4A3 | ENSG00000119508 | 2.070281819 | 2.23233E-06 | 0.000109693 |
| NT5DC4 | ENSG00000144130 | 2.069213867 | 0.001358665 | 0.008971882 |
| HCG4B | ENSG00000220391 | 2.06427522 | 1.57739E-05 | 0.000384497 |
| TEP1 | ENSG00000129566 | 2.05975008 | 5.41921E-08 | 1.11842E-05 |
| CIITA | ENSG00000179583 | 2.056010842 | 6.64394E-07 | 5.29715E-05 |
| MSANTD3-TMEFF1 | ENSG00000251349 | 2.053621054 | 6.31427E-06 | 0.000214453 |
| DUSP15 | ENSG00000149599 | 2.052247256 | 0.000375895 | 0.003436965 |
| TAP2 | ENSG00000237599 | 2.051775455 | 6.84169E-09 | 3.16117E-06 |
| DUSP10 | ENSG00000143507 | 2.041623592 | 7.75376E-06 | 0.000241725 |
| LRRN3 | ENSG00000173114 | 2.034935474 | 4.94617E-06 | 0.000182106 |
| CTSS | ENSG00000163131 | 2.026389122 | 1.08015E-08 | 3.75711E-06 |
| SPTA1 | ENSG00000163554 | 2.025212049 | 1.76685E-05 | 0.000412492 |
| LSMEM1 | ENSG00000181016 | 2.022124648 | 0.000107057 | 0.00141149 |
| PIK3R3 | ENSG00000117461 | 2.019496202 | 1.41352E-06 | 8.25628E-05 |
| IKZF3 | ENSG00000161405 | 2.017696381 | 7.91433E-07 | 5.83343E-05 |
| RELB | ENSG00000104856 | 2.006031036 | 3.57062E-07 | 3.6588E-05 |
| EGR4 | ENSG00000135625 | 2.003061473 | 7.73946E-05 | 0.001122204 |
| LRP2 | ENSG00000081479 | 2.002017335 | 1.72124E-05 | 0.000406132 |
| HLA-E | ENSG00000206493 | 2.001878262 | 8.74118E-09 | 3.46924E-06 |
| HLA-E | ENSG00000229252 | 2.001878262 | 8.74118E-09 | 3.46924E-06 |
| HLA-E | ENSG00000230254 | 2.001878262 | 8.74118E-09 | 3.46924E-06 |
| HLA-E | ENSG00000233904 | 2.001878262 | 8.74118E-09 | 3.46924E-06 |
| DMBX1 | ENSG00000197587 | -2.052419066 | 0.000754539 | 0.00570787 |
| RDH5 | ENSG00000135437 | -2.062438086 | 0.000679166 | 0.005294623 |
| UCP2 | ENSG00000175567 | -2.101583958 | 0.00015854 | 0.001854148 |
| RP11-293I14.2,AC073612.1 | ENSG00000258064 | -2.107569247 | 0.003202509 | 0.017618638 |
| | ENSG00000263203 | -2.114664912 | 0.00605667 | 0.029310043 |
| CYS1 | ENSG00000205795 | -2.133723378 | 0.00016872 | 0.001940214 |
| CLDN10 | ENSG00000134873 | -2.133868217 | 6.56957E-06 | 0.000219864 |

| | | | | |
|---|---|---|---|---|
| MSH5 | ENSG00000230961 | -2.165792882 | 0.000214431 | 0.002293757 |
| RP11-719K4.3,AC136443.1 | ENSG00000254609 | -2.177305222 | 0.000815862 | 0.006073335 |
| LINC00998,SMIM30 | ENSG00000214194 | -2.183322787 | 0.005466151 | 0.02700537 |
| RP11-347C12.3,AC106782.1 | ENSG00000258130 | -2.201026887 | 0.002167698 | 0.012970461 |
| TUBA1A | ENSG00000167552 | -2.262236118 | 5.7312E-05 | 0.000907523 |
| METTL7A | ENSG00000185432 | -2.285845757 | 8.15032E-05 | 0.001164864 |
| VTCN1 | ENSG00000134258 | -2.41821754 | 1.11376E-05 | 0.000305932 |
| FKBPL | ENSG00000223666 | -2.764073133 | 0.000216906 | 0.002309064 |
| | ENSG00000214696 | -2.865833044 | 2.368E-05 | 0.000498004 |
| RING1 | ENSG00000206287 | -2.986469269 | 0.002699778 | 0.015428655 |
| RING1 | ENSG00000235107 | -2.986469269 | 0.002699778 | 0.015428655 |
| PSMC1P5 | ENSG00000250273 | -3.034074068 | 0.002977541 | 0.016677354 |
| | ENSG00000256178 | -3.287495136 | 0.001453681 | 0.009462071 |
| DNAJC25-GNG10 | ENSG00000244115 | -4.550075263 | 2.31559E-06 | 0.000111831 |

**Supplementary Table S2. List of differentially expressed genes for the condition "Calu-3_SARS-CoV versus Calu-3_Mock".** The filters for the extraction were defined as Log-ratio ≥ 2 and FDR ≤ 0.05. The number of differentially expressed genes is 315.

| **Gene Symbol** | **Gene** | **Log-ratio** | **p-value** | **minFDR** |
|---|---|---|---|---|
| IFNB1 | ENSG00000171855 | 8.645527437 | 0.00012353 | 0.002557948 |
| IFNL2 | ENSG00000183709 | 7.891514778 | 3.83322E-06 | 0.000452369 |
| IFNL1 | ENSG00000182393 | 7.721117646 | 1.5933E-05 | 0.000835216 |
| IFNL3 | ENSG00000197110 | 7.430757046 | 2.92655E-06 | 0.000394722 |
| CXCL10 | ENSG00000169245 | 6.366409063 | 5.9515E-07 | 0.000188011 |
| CYP1A1 | ENSG00000140465 | 5.713742971 | 8.98039E-07 | 0.000226021 |
| HSPA6 | ENSG00000173110 | 5.689805269 | 2.37893E-05 | 0.001034332 |
| CXCL11 | ENSG00000169248 | 5.631275654 | 7.12669E-07 | 0.000206414 |
| CH25H | ENSG00000138135 | 5.443694681 | 0.000206455 | 0.003436021 |
| ATF3 | ENSG00000162772 | 5.419966221 | 6.08214E-07 | 0.000189409 |
| IFIT2 | ENSG00000119922 | 5.138520241 | 2.15327E-08 | 8.80842E-05 |
| PTX3 | ENSG00000163661 | 5.124304295 | 8.75635E-07 | 0.000225892 |
| PPP1R15A | ENSG00000087074 | 5.108056068 | 2.98194E-08 | 8.80842E-05 |
| IL6 | ENSG00000136244 | 4.985556602 | 2.4614E-07 | 0.000129188 |
| IL1A | ENSG00000115008 | 4.857690811 | 1.54428E-06 | 0.000287989 |
| TNF | ENSG00000228978 | 4.759708256 | 0.000438942 | 0.005451717 |
| RSAD2 | ENSG00000134321 | 4.604132891 | 1.55188E-08 | 8.80842E-05 |
|  | ENSG00000161570 | 4.56435883 | 6.54297E-06 | 0.000559533 |
| CCL5 | ENSG00000271503 | 4.56435883 | 6.54297E-06 | 0.000559533 |
| IFIT3 | ENSG00000119917 | 4.523255825 | 8.43711E-08 | 9.7969E-05 |
| NFKBIA | ENSG00000100906 | 4.323368073 | 5.54335E-07 | 0.000188011 |
| TNFAIP3 | ENSG00000118503 | 4.306954861 | 1.0918E-06 | 0.000242465 |
| CMPK2 | ENSG00000134326 | 4.266804934 | 8.39466E-08 | 9.7969E-05 |
| CXCL2 | ENSG00000081041 | 4.228332996 | 1.92062E-06 | 0.000314585 |
| DUSP1 | ENSG00000120129 | 4.209573746 | 7.75088E-07 | 0.00021309 |
| KLF4 | ENSG00000136826 | 4.161236763 | 6.56966E-07 | 0.000199389 |
| WFDC5 | ENSG00000175121 | 4.1466465 | 2.19827E-05 | 0.000988094 |
| SLC15A3 | ENSG00000110446 | 4.13424778 | 4.41976E-06 | 0.000488651 |
| MX2 | ENSG00000183486 | 4.126367569 | 1.42503E-07 | 0.000114788 |
| TRAF1 | ENSG00000056558 | 4.084455431 | 1.45903E-05 | 0.000815295 |
| CD274 | ENSG00000120217 | 4.07962358 | 8.58145E-06 | 0.000620542 |
| TRIM22 | ENSG00000132274 | 4.040036678 | 4.15447E-07 | 0.000162797 |
| HERC5 | ENSG00000138646 | 4.036915302 | 1.02662E-08 | 8.80842E-05 |
| IDO1 | ENSG00000131203 | 3.996575832 | 4.63374E-06 | 0.000499217 |
| ETV7 | ENSG00000010030 | 3.964395165 | 2.11542E-05 | 0.000968013 |
| OASL | ENSG00000135114 | 3.960576057 | 1.00415E-07 | 0.000100276 |
| ARRDC3 | ENSG00000113369 | 3.91421032 | 6.17963E-07 | 0.000189409 |
| HLA-K | ENSG00000232708 | 3.893367231 | 0.00012448 | 0.002563673 |
| USP18 | ENSG00000184979 | 3.862231731 | 4.90176E-07 | 0.000182824 |
| CSF3 | ENSG00000108342 | 3.788201004 | 2.98574E-05 | 0.001165568 |
| BATF2 | ENSG00000168062 | 3.770451546 | 6.96579E-07 | 0.000206414 |
| FAM46A,TENT5A | ENSG00000112773 | 3.76995635 | 1.954E-07 | 0.000126021 |
| CREB5 | ENSG00000146592 | 3.761869431 | 4.71004E-07 | 0.000180011 |
| BBC3 | ENSG00000105327 | 3.753449678 | 8.44645E-07 | 0.000223484 |
| LTB | ENSG00000227507 | 3.752802193 | 0.000139217 | 0.002705429 |

| | | | | |
|---|---|---|---|---|
| LAMP3 | ENSG00000078081 | 3.750243187 | 3.05182E-07 | 0.000136921 |
| GBP4 | ENSG00000162654 | 3.727169514 | 3.56918E-08 | 8.80842E-05 |
| OTUD1 | ENSG00000165312 | 3.703802586 | 2.3984E-06 | 0.000353559 |
| IFIT1 | ENSG00000185745 | 3.692374706 | 2.96128E-08 | 8.80842E-05 |
| ZBP1 | ENSG00000124256 | 3.675982833 | 2.9174E-05 | 0.001155477 |
| IFNL4 | ENSG00000272395 | 3.658265829 | 5.787E-05 | 0.001637553 |
| MT2A | ENSG00000125148 | 3.630337477 | 1.51334E-06 | 0.000283931 |
| ZC3HAV1 | ENSG00000105939 | 3.611983776 | 4.44319E-09 | 7.48089E-05 |
| EPSTI1 | ENSG00000133106 | 3.601566553 | 1.10424E-06 | 0.000242465 |
| CCL2 | ENSG00000108691 | 3.54889822 | 8.49402E-06 | 0.000620542 |
| GBP5 | ENSG00000154451 | 3.538462043 | 0.000599515 | 0.006611754 |
| DHX58 | ENSG00000108771 | 3.522194862 | 3.83242E-06 | 0.000452369 |
| OAS2 | ENSG00000111335 | 3.517900944 | 5.95183E-07 | 0.000188011 |
| BST2 | ENSG00000130303 | 3.516386986 | 1.04642E-05 | 0.000689608 |
| TRANK1 | ENSG00000168016 | 3.500861168 | 1.04472E-06 | 0.000242465 |
| IRF1 | ENSG00000125347 | 3.474999428 | 1.1877E-08 | 8.80842E-05 |
| | ENSG00000187990 | 3.462145805 | 0.000119759 | 0.002522028 |
| ISG20 | ENSG00000172183 | 3.445830345 | 5.99705E-08 | 9.28254E-05 |
| MAP3K8 | ENSG00000107968 | 3.441730499 | 1.4942E-06 | 0.000283931 |
| IFITM1 | ENSG00000185885 | 3.42606926 | 3.19065E-05 | 0.0011958 |
| | ENSG00000260178 | 3.415021658 | 0.000187674 | 0.003270637 |
| NLRC5 | ENSG00000140853 | 3.405679464 | 7.4752E-06 | 0.00058066 |
| NUAK2 | ENSG00000163545 | 3.390328407 | 1.65644E-06 | 0.000295913 |
| GBP1 | ENSG00000117228 | 3.365758419 | 4.65806E-08 | 8.80842E-05 |
| IFIH1 | ENSG00000115267 | 3.364337921 | 4.83309E-09 | 7.48089E-05 |
| MXD1 | ENSG00000059728 | 3.354865313 | 8.1176E-06 | 0.000609943 |
| EGR2 | ENSG00000122877 | 3.342299938 | 1.15893E-05 | 0.000723327 |
| CSRNP1 | ENSG00000144655 | 3.325723886 | 9.811E-06 | 0.000674931 |
| NOCT | ENSG00000151014 | 3.324230671 | 1.16453E-06 | 0.000248624 |
| | ENSG00000264889 | 3.318348885 | 1.87367E-05 | 0.000905564 |
| FOSB | ENSG00000125740 | 3.316786051 | 1.04798E-05 | 0.000689608 |
| PLA2G4C | ENSG00000105499 | 3.271391869 | 3.07149E-05 | 0.001182369 |
| AC129492.1,PER1 | ENSG00000179094 | 3.267480373 | 9.71036E-07 | 0.000236696 |
| CCL20 | ENSG00000115009 | 3.266313553 | 7.75759E-06 | 0.000594161 |
| JUN | ENSG00000177606 | 3.238517761 | 3.59717E-07 | 0.000150483 |
| SAMD9L | ENSG00000177409 | 3.228846073 | 1.67998E-07 | 0.00012032 |
| HELZ2 | ENSG00000130589 | 3.224295139 | 8.91565E-08 | 9.7969E-05 |
| AL031777.1,RP1-34B20.4 | ENSG00000217275 | 3.223608837 | 0.001656377 | 0.012848022 |
| PPP4R4 | ENSG00000119698 | 3.22319141 | 3.81611E-05 | 0.001298189 |
| CSF2 | ENSG00000164400 | 3.214499995 | 3.1128E-05 | 0.001182369 |
| ARL14EPL | ENSG00000268223 | 3.164648533 | 0.00015728 | 0.002942621 |
| DUSP8 | ENSG00000184545 | 3.159594536 | 1.00696E-06 | 0.000239789 |
| TAP1 | ENSG00000168394 | 3.146584988 | 1.50742E-06 | 0.000283931 |
| TNF | ENSG00000204490 | 3.145395018 | 1.05617E-05 | 0.000689608 |
| TNF | ENSG00000206439 | 3.145395018 | 1.05617E-05 | 0.000689608 |
| TNF | ENSG00000223952 | 3.145395018 | 1.05617E-05 | 0.000689608 |
| TNF | ENSG00000228321 | 3.145395018 | 1.05617E-05 | 0.000689608 |
| TNF | ENSG00000228849 | 3.145395018 | 1.05617E-05 | 0.000689608 |
| TNF | ENSG00000230108 | 3.145395018 | 1.05617E-05 | 0.000689608 |
| TNF | ENSG00000232810 | 3.145395018 | 1.05617E-05 | 0.000689608 |

| | | | | |
|---|---|---|---|---|
| RTP4 | ENSG00000136514 | 3.132684708 | 3.67832E-05 | 0.001272905 |
| PMAIP1 | ENSG00000141682 | 3.13042593 | 2.31889E-07 | 0.000129188 |
| IKZF3 | ENSG00000161405 | 3.128968239 | 2.04416E-06 | 0.000322862 |
| APOL6 | ENSG00000221963 | 3.125056267 | 4.83713E-08 | 8.80842E-05 |
| PPM1K | ENSG00000163644 | 3.119034767 | 9.36086E-06 | 0.000648916 |
| DNAJB4 | ENSG00000162616 | 3.107779264 | 1.86211E-06 | 0.000311596 |
| C6orf58 | ENSG00000184530 | 3.105012536 | 4.80923E-05 | 0.001461165 |
| FOS | ENSG00000170345 | 3.090422153 | 4.21211E-05 | 0.001374018 |
| MX1 | ENSG00000157601 | 3.084752083 | 4.75746E-08 | 8.80842E-05 |
| XAF1 | ENSG00000132530 | 3.068871498 | 8.30794E-08 | 9.7969E-05 |
| HIST1H3D | ENSG00000197409 | 3.064462185 | 1.06429E-05 | 0.000690044 |
| CFB | ENSG00000241253 | 3.04198432 | 6.27421E-06 | 0.000553722 |
| ZC3H12A | ENSG00000163874 | 3.034065247 | 1.10247E-07 | 0.000104556 |
| HCG4B | ENSG00000231941 | 3.024304867 | 0.000131339 | 0.002624957 |
| HIST1H4H | ENSG00000158406 | 3.007132769 | 0.000295226 | 0.004315204 |
| INHBA | ENSG00000122641 | 2.979223251 | 5.14624E-06 | 0.000516378 |
| HSPB8 | ENSG00000152137 | 2.969990849 | 2.83032E-05 | 0.001133139 |
| IRF7 | ENSG00000185507 | 2.960988522 | 5.09682E-07 | 0.000183684 |
| MB21D2 | ENSG00000180611 | 2.960822582 | 1.33302E-06 | 0.000269714 |
| DHRS2 | ENSG00000100867 | 2.948078871 | 5.24923E-05 | 0.001540669 |
| NPFFR2 | ENSG00000056291 | 2.939808488 | 3.06226E-05 | 0.001182369 |
| ACHE | ENSG00000087085 | 2.931080341 | 4.91757E-05 | 0.001479428 |
| DDX58 | ENSG00000107201 | 2.928350449 | 2.01631E-08 | 8.80842E-05 |
| CYLD | ENSG00000083799 | 2.920273781 | 8.55587E-07 | 0.000224461 |
| IGF2 | ENSG00000167244 | 2.916782081 | 0.000160394 | 0.002980381 |
| CX3CL1 | ENSG00000006210 | 2.90555954 | 4.77583E-07 | 0.000180299 |
| IFI16 | ENSG00000163565 | 2.894404888 | 9.97126E-07 | 0.000239287 |
| NKX3-1 | ENSG00000167034 | 2.887364149 | 0.000909349 | 0.008630716 |
| HLA-H | ENSG00000206341 | 2.882062435 | 6.59349E-06 | 0.000560755 |
| IFI27 | ENSG00000165949 | 2.880156517 | 1.80523E-05 | 0.000889115 |
| HLA-F | ENSG00000229698 | 2.87268281 | 9.8092E-05 | 0.002239406 |
| COL7A1 | ENSG00000114270 | 2.87083149 | 5.57323E-06 | 0.000535809 |
| IFIT5 | ENSG00000152778 | 2.867919445 | 4.7721E-08 | 8.80842E-05 |
| RASGRP3 | ENSG00000152689 | 2.863472223 | 1.84737E-06 | 0.000311596 |
| TIPARP | ENSG00000163659 | 2.862968445 | 7.18135E-08 | 9.66579E-05 |
| RAB30 | ENSG00000137502 | 2.856419325 | 1.68118E-05 | 0.00085535 |
| DDX60L | ENSG00000181381 | 2.855362415 | 1.50577E-06 | 0.000283931 |
| TLDC2 | ENSG00000101342 | 2.8330338 | 0.000265302 | 0.004025962 |
| REL | ENSG00000162924 | 2.819662094 | 4.50219E-07 | 0.000174218 |
| HMGB1P3 | ENSG00000250011 | 2.819127157 | 0.002753548 | 0.018498608 |
| NEURL3 | ENSG00000163121 | 2.810498714 | 3.60973E-06 | 0.000445205 |
| HLA-F | ENSG00000137403 | 2.807108164 | 0.004164133 | 0.024961619 |
| BMP2 | ENSG00000125845 | 2.801396608 | 2.03086E-05 | 0.000945485 |
| FAM65B,RIPOR2 | ENSG00000111913 | 2.788742483 | 0.00083358 | 0.008171356 |
| ZEB2 | ENSG00000169554 | 2.785652228 | 9.5089E-05 | 0.002201434 |
| RAET1L | ENSG00000155918 | 2.77593106 | 0.000290723 | 0.004282971 |
| IFNL3P1 | ENSG00000268510 | 2.77382946 | 0.000704785 | 0.007311669 |
| ISG15 | ENSG00000187608 | 2.768993378 | 9.8519E-07 | 0.00023827 |
| | ENSG00000262933 | 2.767766297 | 0.000160964 | 0.002986359 |
| ZFP36 | ENSG00000128016 | 2.765545845 | 2.50008E-07 | 0.000129188 |

| | | | | |
|---|---|---|---|---|
| KMO | ENSG00000117009 | 2.751908123 | 0.001832009 | 0.013815614 |
| DUSP15 | ENSG00000149599 | 2.751177758 | 0.000121924 | 0.002549132 |
| OAS1,AC004551.1 | ENSG00000089127 | 2.741504669 | 2.95206E-07 | 0.000136495 |
| TNFRSF9 | ENSG00000049249 | 2.737144068 | 2.06726E-05 | 0.000955165 |
| TAP1 | ENSG00000227816 | 2.723752022 | 1.22447E-06 | 0.000257864 |
| TAP1 | ENSG00000230705 | 2.723752022 | 1.22447E-06 | 0.000257864 |
| TAP1 | ENSG00000206297 | 2.708345413 | 2.26781E-06 | 0.00034239 |
| TAP1 | ENSG00000226173 | 2.708345413 | 2.26781E-06 | 0.00034239 |
| NT5C3A | ENSG00000122643 | 2.708090305 | 3.38226E-08 | 8.80842E-05 |
| | ENSG00000184348 | 2.707293957 | 0.003503408 | 0.021985609 |
| | ENSG00000197846 | 2.692678928 | 2.85986E-05 | 0.001140885 |
| TXNIP | ENSG00000265972 | 2.688961029 | 1.37534E-07 | 0.000114788 |
| TAP1 | ENSG00000232367 | 2.681231976 | 8.30865E-07 | 0.000221734 |
| HIST3H2BB | ENSG00000196890 | 2.675552964 | 0.00711716 | 0.036979846 |
| SIPA1L2 | ENSG00000116991 | 2.667143047 | 3.64878E-06 | 0.000445437 |
| CPEB2 | ENSG00000137449 | 2.666138411 | 1.13697E-06 | 0.000245398 |
| RNF213,AC124319.1 | ENSG00000173821 | 2.659548759 | 9.47534E-08 | 9.7969E-05 |
| RASGEF1B | ENSG00000138670 | 2.648212433 | 4.88721E-06 | 0.000507201 |
| APOL3 | ENSG00000128284 | 2.645727634 | 8.28469E-05 | 0.002013103 |
| CSF1 | ENSG00000184371 | 2.643229961 | 4.06196E-05 | 0.00134776 |
| BCL3 | ENSG00000069399 | 2.637298584 | 1.06666E-05 | 0.000690044 |
| GMPR | ENSG00000137198 | 2.618448973 | 4.13665E-05 | 0.001358747 |
| RELB | ENSG00000104856 | 2.617918015 | 2.89744E-06 | 0.000393404 |
| EGR4 | ENSG00000135625 | 2.614015996 | 0.000290776 | 0.004282971 |
| PARP14 | ENSG00000173193 | 2.596164703 | 7.13451E-07 | 0.000206414 |
| DDX60 | ENSG00000137628 | 2.590519428 | 3.55605E-07 | 0.000150483 |
| HLA-F | ENSG00000206509 | 2.587547302 | 0.000583788 | 0.006490222 |
| HLA-F | ENSG00000237508 | 2.587547302 | 0.000583788 | 0.006490222 |
| | ENSG00000117289 | 2.576401472 | 1.49719E-05 | 0.000817311 |
| IL10RA | ENSG00000110324 | 2.569458883 | 4.33325E-05 | 0.001398793 |
| IFI35 | ENSG00000068079 | 2.566558361 | 5.14002E-07 | 0.000183684 |
| TNFAIP2 | ENSG00000185215 | 2.566084862 | 1.45562E-06 | 0.000283931 |
| ZNFX1 | ENSG00000124201 | 2.562953472 | 3.94323E-08 | 8.80842E-05 |
| RND1 | ENSG00000172602 | 2.559008479 | 0.000265266 | 0.004025962 |
| SAMHD1 | ENSG00000101347 | 2.55395174 | 1.80651E-07 | 0.00012032 |
| OR52K3P | ENSG00000225101 | 2.553416595 | 0.002445116 | 0.016918518 |
| CNOT6LP1 | ENSG00000230183 | 2.540828496 | 0.000554743 | 0.006258445 |
| | ENSG00000266796 | 2.53205055 | 2.33764E-05 | 0.001025019 |
| UBE2L6 | ENSG00000156587 | 2.501804352 | 2.86231E-06 | 0.000392073 |
| SLC1A3 | ENSG00000079215 | 2.494547963 | 0.000168866 | 0.0030841 |
| DUOX1 | ENSG00000137857 | 2.494302511 | 1.05369E-05 | 0.000689608 |
| | ENSG00000198518 | 2.48996377 | 7.8346E-05 | 0.001962263 |
| HLA-F | ENSG00000235220 | 2.486290455 | 0.002343993 | 0.016445034 |
| C11orf96 | ENSG00000187479 | 2.47970587 | 0.00065584 | 0.006988931 |
| EGR1 | ENSG00000120738 | 2.469434738 | 3.88446E-06 | 0.000454595 |
| SCN3A | ENSG00000153253 | 2.467128649 | 4.66142E-05 | 0.001443023 |
| TNFSF9 | ENSG00000125657 | 2.460440278 | 0.000862927 | 0.008350154 |
| HSH2D | ENSG00000196684 | 2.448411226 | 8.02234E-06 | 0.000605726 |
| ZNF442 | ENSG00000198342 | 2.445371151 | 0.000194225 | 0.003319899 |
| RP11-1280N14.3,AC145138.1 | ENSG00000251634 | 2.435585737 | 0.001277433 | 0.010822518 |

| | | | | |
|---|---|---|---|---|
| GBP1P1 | ENSG00000225492 | 2.416740239 | 0.000103486 | 0.00230974 |
| COL16A1 | ENSG00000084636 | 2.413737535 | 1.63059E-05 | 0.000846072 |
| PINLYP | ENSG00000234465 | 2.413685501 | 0.000489712 | 0.005829411 |
| IFI44 | ENSG00000137965 | 2.409427166 | 7.08489E-08 | 9.66579E-05 |
| DNAH17 | ENSG00000187775 | 2.404188871 | 8.92529E-06 | 0.000633716 |
| RGAG1,RTL9 | ENSG00000243978 | 2.400226363 | 9.44457E-05 | 0.002197477 |
| JAK2 | ENSG00000096968 | 2.397353649 | 1.6634E-06 | 0.000295913 |
| OAS3 | ENSG00000111331 | 2.394680977 | 5.51143E-08 | 8.97987E-05 |
| PARP10 | ENSG00000178685 | 2.394044876 | 2.44196E-07 | 0.000129188 |
| CXCL3 | ENSG00000163734 | 2.384198189 | 1.40748E-06 | 0.000279304 |
| PARP12 | ENSG00000059378 | 2.374707699 | 2.37327E-07 | 0.000129188 |
| HIST1H2BC | ENSG00000180596 | 2.373980522 | 0.000176942 | 0.003152705 |
| PIM3 | ENSG00000198355 | 2.370022774 | 1.44612E-07 | 0.000114788 |
| IL11 | ENSG00000095752 | 2.36909169 | 5.64802E-05 | 0.001618303 |
| DAPP1 | ENSG00000070190 | 2.360961914 | 1.467E-05 | 0.000815351 |
| NCOA7 | ENSG00000111912 | 2.35841465 | 2.50389E-07 | 0.000129188 |
| THEMIS2 | ENSG00000130775 | 2.351515412 | 9.90982E-06 | 0.000675723 |
| LRRN3 | ENSG00000173114 | 2.350860834 | 3.10398E-05 | 0.001182369 |
| AHRR | ENSG00000063438 | 2.347205281 | 0.000361809 | 0.004861462 |
| GEM | ENSG00000164949 | 2.339888096 | 6.93221E-05 | 0.001831062 |
| HLA-J | ENSG00000243336 | 2.338973582 | 0.002393892 | 0.016682383 |
| | ENSG00000182217 | 2.337333679 | 5.37868E-06 | 0.000526924 |
| KLF6 | ENSG00000067082 | 2.333143234 | 4.0592E-07 | 0.000161104 |
| IRAK2 | ENSG00000134070 | 2.329335213 | 2.85753E-07 | 0.000136495 |
| | ENSG00000261660 | 2.327109337 | 1.5331E-07 | 0.000117235 |
| HLA-C | ENSG00000206435 | 2.316351891 | 0.007325081 | 0.037810725 |
| UBA7 | ENSG00000182179 | 2.308647156 | 6.10218E-05 | 0.001685148 |
| SLFN5 | ENSG00000166750 | 2.308335304 | 7.24039E-06 | 0.000575549 |
| LGALS9 | ENSG00000168961 | 2.306843281 | 2.02384E-07 | 0.000127391 |
| PTGER4 | ENSG00000171522 | 2.305876493 | 1.12122E-07 | 0.000104556 |
| STAT2 | ENSG00000170581 | 2.297055721 | 1.05133E-06 | 0.000242465 |
| SAMD9 | ENSG00000205413 | 2.294365883 | 1.75246E-05 | 0.000876427 |
| SOCS1 | ENSG00000185338 | 2.294297457 | 0.000114915 | 0.002470439 |
| ZBTB20 | ENSG00000181722 | 2.292137146 | 5.1396E-06 | 0.000516378 |
| RIPK2 | ENSG00000104312 | 2.290173054 | 1.8248E-07 | 0.00012032 |
| HLA-V | ENSG00000206444 | 2.289546818 | 8.89613E-05 | 0.00211073 |
| HLA-B | ENSG00000223532 | 2.279584885 | 1.86188E-05 | 0.000903418 |
| TSC22D2 | ENSG00000196428 | 2.274520874 | 2.24751E-07 | 0.000129188 |
| ICAM1 | ENSG00000090339 | 2.274106026 | 1.81804E-05 | 0.000889115 |
| IL4I1 | ENSG00000104951 | 2.271526098 | 0.000196161 | 0.003342073 |
| HIVEP2 | ENSG00000010818 | 2.26820612 | 5.48929E-06 | 0.000531038 |
| IFITM2 | ENSG00000185201 | 2.264898777 | 0.000262954 | 0.004004074 |
| CASP1 | ENSG00000137752 | 2.263835311 | 0.000119222 | 0.002517568 |
| PSMB9 | ENSG00000240508 | 2.246178627 | 5.90423E-07 | 0.000188011 |
| PSMB9 | ENSG00000242711 | 2.246178627 | 5.90423E-07 | 0.000188011 |
| PSMB9 | ENSG00000243958 | 2.246178627 | 5.90423E-07 | 0.000188011 |
| C19orf66 | ENSG00000130813 | 2.243360996 | 1.29156E-06 | 0.000268341 |
| XRN1 | ENSG00000114127 | 2.242507458 | 1.51064E-06 | 0.000283931 |
| IFITM3 | ENSG00000142089 | 2.228624821 | 1.56773E-05 | 0.000828195 |
| TYMP | ENSG00000025708 | 2.223926067 | 4.70093E-05 | 0.001445591 |

| | | | | |
|---|---|---|---|---|
| CSNK2B,DADB-127H9.10 | ENSG00000258589 | 2.21693182 | 0.008135114 | 0.040969371 |
| ARRDC4 | ENSG00000140450 | 2.203893661 | 5.68922E-07 | 0.000188011 |
| FILIP1L | ENSG00000168386 | 2.190280914 | 4.15215E-05 | 0.001360192 |
| ZBTB10 | ENSG00000205189 | 2.188535929 | 1.79058E-05 | 0.000888315 |
| SYNE1 | ENSG00000131018 | 2.18548584 | 3.43075E-07 | 0.000149586 |
| ACKR4 | ENSG00000129048 | 2.185175419 | 0.000304123 | 0.00439735 |
| ZNF433 | ENSG00000197647 | 2.182504058 | 2.16777E-05 | 0.000978246 |
| PTGS2 | ENSG00000073756 | 2.177765369 | 2.39224E-06 | 0.000353559 |
| CXCL8 | ENSG00000169429 | 2.174015999 | 1.49917E-05 | 0.000817311 |
| ANKRD33B | ENSG00000164236 | 2.171113014 | 3.6801E-05 | 0.001272905 |
| IFI44L | ENSG00000137959 | 2.170928001 | 3.25726E-05 | 0.001203289 |
| APOL4 | ENSG00000100336 | 2.161408663 | 0.00126888 | 0.010770692 |
| IL32 | ENSG00000008517 | 2.158648491 | 6.08317E-06 | 0.000553722 |
| DUSP10 | ENSG00000143507 | 2.158569813 | 3.60212E-06 | 0.000445205 |
| TTC9B | ENSG00000174521 | 2.158174843 | 0.000731576 | 0.007486739 |
| GPCPD1 | ENSG00000125772 | 2.151395559 | 2.06899E-06 | 0.000323882 |
| | ENSG00000183941 | 2.150698662 | 2.48393E-05 | 0.001063555 |
| CFB | ENSG00000243649 | 2.143897057 | 0.000861462 | 0.008341656 |
| PELI1 | ENSG00000197329 | 2.141742706 | 2.02514E-06 | 0.000322862 |
| EFNA1 | ENSG00000169242 | 2.137276173 | 1.45537E-05 | 0.000814717 |
| SNPH | ENSG00000101298 | 2.135411859 | 4.03217E-05 | 0.001343283 |
| PLAUR | ENSG00000011422 | 2.131796837 | 2.99824E-07 | 0.000136495 |
| NFKBIZ | ENSG00000144802 | 2.131692886 | 1.09216E-06 | 0.000242465 |
| SP110 | ENSG00000135899 | 2.130768776 | 5.37521E-07 | 0.000188011 |
| FAM214A | ENSG00000047346 | 2.123968601 | 1.2282E-05 | 0.000745519 |
| STX19 | ENSG00000178750 | 2.122432947 | 0.00032503 | 0.004571543 |
| STX11 | ENSG00000135604 | 2.1204229 | 0.001435163 | 0.011603121 |
| FST | ENSG00000134363 | 2.116599083 | 1.05265E-05 | 0.000689608 |
| NR1D1 | ENSG00000126368 | 2.111507416 | 1.8024E-06 | 0.000311596 |
| TNFSF10 | ENSG00000121858 | 2.109423637 | 3.72823E-07 | 0.000153887 |
| THSD7A | ENSG00000005108 | 2.106934071 | 2.04053E-06 | 0.000322862 |
| OPTN | ENSG00000123240 | 2.102813721 | 1.14834E-07 | 0.000104556 |
| HMGB2P1 | ENSG00000267736 | 2.10140419 | 0.001671207 | 0.012920971 |
| | ENSG00000237683 | 2.099866867 | 0.008775779 | 0.043356495 |
| NAV2 | ENSG00000166833 | 2.097501278 | 2.64228E-06 | 0.000367515 |
| ACTN2 | ENSG00000077522 | 2.097239971 | 0.000389707 | 0.005071102 |
| LMO2 | ENSG00000135363 | 2.09670949 | 0.003248478 | 0.020824835 |
| | ENSG00000198339 | 2.094578862 | 0.000229475 | 0.00365022 |
| CPEB3 | ENSG00000107864 | 2.091117144 | 7.07311E-06 | 0.000571703 |
| CTAGE4 | ENSG00000225932 | 2.089536428 | 0.001144217 | 0.010060078 |
| EPHA4 | ENSG00000116106 | 2.086579204 | 6.26214E-06 | 0.000553722 |
| C5orf56 | ENSG00000197536 | 2.080994368 | 6.38424E-05 | 0.001728263 |
| SH3TC1 | ENSG00000125089 | 2.076715708 | 5.25654E-06 | 0.000521648 |
| ATP4A | ENSG00000105675 | 2.072595119 | 1.69034E-05 | 0.000856429 |
| CTC-487M23.8 | ENSG00000272869 | 2.072095156 | 0.010219198 | 0.048752615 |
| RXRB | ENSG00000235712 | 2.0720855 | 4.48351E-05 | 0.001419184 |
| IL15RA | ENSG00000134470 | 2.071744442 | 7.3546E-07 | 0.000208877 |
| TNFSF13B | ENSG00000102524 | 2.063539505 | 0.000754949 | 0.007638921 |
| PGLYRP2 | ENSG00000161031 | 2.0594147 | 0.002508845 | 0.01725535 |
| MICB | ENSG00000204516 | 2.053634405 | 0.000141045 | 0.002728396 |

| | | | | |
|---|---|---|---|---|
| IGHE | ENSG00000211891 | 2.047626972 | 0.004606121 | 0.026855232 |
| | ENSG00000270340 | 2.047626972 | 0.004606121 | 0.026855232 |
| ITGAM | ENSG00000169896 | 2.044259071 | 0.000134014 | 0.002650917 |
| ZC3H12C | ENSG00000149289 | 2.031696081 | 2.07154E-06 | 0.000323882 |
| FLRT3 | ENSG00000125848 | 2.030714273 | 6.31062E-06 | 0.000553722 |
| SYNPO | ENSG00000171992 | 2.030555725 | 2.72955E-05 | 0.001107453 |
| BACH2 | ENSG00000112182 | 2.023777127 | 1.22704E-05 | 0.000745519 |
| BRSK2 | ENSG00000174672 | 2.021775246 | 0.000302927 | 0.004390803 |
| ADORA2A | ENSG00000128271 | 2.021773141 | 0.009996783 | 0.047950172 |
| SOD2 | ENSG00000112096 | 2.018375397 | 8.57088E-08 | 9.7969E-05 |
| HERC6 | ENSG00000138642 | 2.015695095 | 2.52299E-06 | 0.000364648 |
| STYK1 | ENSG00000060140 | 2.013912201 | 5.17095E-06 | 0.000516378 |
| | ENSG00000196966 | 2.009919882 | 0.009348483 | 0.045582137 |
| FSTL3 | ENSG00000070404 | 2.007456303 | 1.36499E-05 | 0.000787322 |
| SERPINA10 | ENSG00000140093 | 2.004845906 | 0.000447676 | 0.005528 |
| KDM6B | ENSG00000132510 | 2.004181862 | 2.10018E-07 | 0.000127481 |
| ZNF321P,ZNF816-ZNF321P | ENSG00000221874 | -2.100255489 | 0.000179403 | 0.003183407 |
| TUBA1A | ENSG00000167552 | -2.141874313 | 0.000472059 | 0.005695063 |
| TGIF2-C20orf24,TGIF2-RAB5IF | ENSG00000259399 | -2.630449705 | 0.000816617 | 0.008052032 |
| PSMC1P5 | ENSG00000250273 | -4.013153702 | 0.000326972 | 0.004586078 |

**Supplementary Table S3. List of differentially expressed genes for the condition "Caco-2_SARS-CoV-2 versus Caco-2_Mock".** The filters for the extraction were defined as Log-ratio ≥ 0.6 and FDR ≤ 0.05. The number of differentially expressed genes is 141.

| Gene Symbol | Gene | Log-ratio | p-value | minFDR |
|---|---|---|---|---|
| POLR2J2,RP11-577H5.5,AC105052.3 | ENSG00000267645 | 3.504205227 | 9.19121E-06 | 0.02812339 |
| ARRDC3 | ENSG00000113369 | 3.100578308 | 2.19811E-07 | 0.006804678 |
|  | ENSG00000264082 | 2.875876665 | 0.000162102 | 0.041478386 |
|  | ENSG00000182217 | 2.414260507 | 2.0898E-05 | 0.02812339 |
| JUN | ENSG00000177606 | 2.402955055 | 5.65754E-06 | 0.02812339 |
|  | ENSG00000183941 | 2.361856461 | 0.000175066 | 0.041478386 |
| ATF3 | ENSG00000162772 | 2.334241867 | 1.98216E-05 | 0.02812339 |
|  | ENSG00000255798 | 2.195330143 | 0.000113263 | 0.041478386 |
| CXCL2 | ENSG00000081041 | 2.081483364 | 9.67496E-05 | 0.041478386 |
| ZNF844 | ENSG00000223547 | 1.938457966 | 1.98227E-05 | 0.02812339 |
| SERPINE1 | ENSG00000106366 | 1.860815048 | 2.00756E-05 | 0.02812339 |
| TNS4 | ENSG00000131746 | 1.831270099 | 5.10586E-05 | 0.033090547 |
| KRT6A | ENSG00000205420 | 1.828607202 | 1.68505E-05 | 0.02812339 |
| ZNF554 | ENSG00000172006 | 1.756035924 | 0.000113669 | 0.041478386 |
| EGR1 | ENSG00000120738 | 1.752364159 | 9.34461E-07 | 0.014464057 |
| VGF | ENSG00000128564 | 1.72901845 | 2.63455E-05 | 0.02812339 |
| NR1D1 | ENSG00000126368 | 1.717155933 | 0.000332171 | 0.045270347 |
| JUNB | ENSG00000171223 | 1.695165634 | 0.000311757 | 0.045270347 |
| HIST2H3C | ENSG00000203811 | 1.692119479 | 5.7855E-05 | 0.033090547 |
| HIST2H3A | ENSG00000203852 | 1.692119479 | 5.7855E-05 | 0.033090547 |
|  | ENSG00000270263 | 1.692119479 | 5.7855E-05 | 0.033090547 |
|  | ENSG00000270827 | 1.692119479 | 5.7855E-05 | 0.033090547 |
| MDM2 | ENSG00000135679 | 1.676239252 | 0.000126445 | 0.041478386 |
| ZBTB20 | ENSG00000181722 | 1.666024148 | 0.0002102 | 0.041478386 |
| AC129492.1,PER1 | ENSG00000179094 | 1.646231174 | 1.22138E-05 | 0.02812339 |
| MXD1 | ENSG00000059728 | 1.581274986 | 6.85094E-05 | 0.035347406 |
| HIVEP2 | ENSG00000010818 | 1.574655056 | 1.99758E-05 | 0.02812339 |
| HIST1H3D | ENSG00000197409 | 1.543391943 | 0.000285501 | 0.044637583 |
| IER3 | ENSG00000227231 | 1.438555956 | 0.000149708 | 0.041478386 |
| IER3 | ENSG00000230128 | 1.438555956 | 0.000149708 | 0.041478386 |
| IER3 | ENSG00000235030 | 1.438555956 | 0.000149708 | 0.041478386 |
| IER3 | ENSG00000237155 | 1.438555956 | 0.000149708 | 0.041478386 |
| PPP1R15A | ENSG00000087074 | 1.42048645 | 2.30932E-05 | 0.02812339 |
| PLAU | ENSG00000122861 | 1.375488758 | 0.00012705 | 0.041478386 |
| DUSP1 | ENSG00000120129 | 1.360269547 | 2.60447E-05 | 0.02812339 |
| PLK2 | ENSG00000145632 | 1.356685162 | 2.28328E-05 | 0.02812339 |
| HDAC9 | ENSG00000048052 | 1.353957176 | 1.38785E-05 | 0.02812339 |
| AKAP12 | ENSG00000131016 | 1.32122016 | 1.1155E-05 | 0.02812339 |
| TIPARP | ENSG00000163659 | 1.317221642 | 1.81869E-05 | 0.02812339 |
| ZNF442 | ENSG00000198342 | 1.3171767 | 0.000293774 | 0.045021563 |
| ZNF627 | ENSG00000198551 | 1.270971537 | 0.000138326 | 0.041478386 |
| ZNF555 | ENSG00000186300 | 1.261097193 | 0.000312654 | 0.045270347 |
| FOS | ENSG00000170345 | 1.259091616 | 0.000350847 | 0.045913355 |

| | | | | |
|---|---|---|---|---|
| HIST1H2BD | ENSG00000158373 | 1.25831461 | 0.00013851 | 0.041478386 |
| SEPW1,SELENOW | ENSG00000178980 | 1.25448513 | 0.000263979 | 0.043915682 |
| DUSP6 | ENSG00000139318 | 1.252747536 | 4.45434E-05 | 0.032068133 |
| CYP1A1 | ENSG00000140465 | 1.24665451 | 4.23562E-05 | 0.03150835 |
| IER2 | ENSG00000160888 | 1.176316261 | 0.000276677 | 0.043915682 |
| ITGA2 | ENSG00000164171 | 1.129992485 | 9.49863E-05 | 0.041415361 |
| NUAK2 | ENSG00000163545 | 1.118688583 | 2.86461E-06 | 0.02812339 |
| KLF6 | ENSG00000067082 | 1.105168819 | 1.27293E-05 | 0.02812339 |
| RP11-1280N14.3,AC145138.1 | ENSG00000251634 | 1.093912721 | 7.66697E-05 | 0.037085385 |
| KLF10 | ENSG00000155090 | 1.092367649 | 0.000154461 | 0.041478386 |
| BHLHE40 | ENSG00000134107 | 1.074317217 | 0.000160868 | 0.041478386 |
| ZNF441 | ENSG00000197044 | 1.067896366 | 5.9324E-05 | 0.033090547 |
| PARD6B | ENSG00000124171 | 1.064840317 | 3.16921E-05 | 0.029730093 |
| TNFAIP3 | ENSG00000118503 | 1.048531532 | 0.000329824 | 0.045270347 |
| IGFBP4 | ENSG00000141753 | 1.044826031 | 0.000148315 | 0.041478386 |
| GEM | ENSG00000164949 | 1.038532734 | 0.000119615 | 0.041478386 |
| FBXW10 | ENSG00000171931 | 1.01608026 | 3.57397E-05 | 0.030795168 |
| CYLD | ENSG00000083799 | 1.001839638 | 0.000193377 | 0.041478386 |
| ZC3H12A | ENSG00000163874 | 0.976932049 | 0.000237628 | 0.043915682 |
| ZNF608 | ENSG00000168916 | 0.967037916 | 0.000209245 | 0.041478386 |
| CSRNP1 | ENSG00000144655 | 0.956901073 | 2.48766E-05 | 0.02812339 |
| SPRY4 | ENSG00000187678 | 0.95191431 | 4.64809E-05 | 0.032626663 |
| NFKBIA | ENSG00000100906 | 0.938340187 | 2.29855E-05 | 0.02812339 |
| TSC22D2 | ENSG00000196428 | 0.930682182 | 0.000315574 | 0.045270347 |
| ASB2 | ENSG00000100628 | 0.909536362 | 0.000265314 | 0.043915682 |
| ANKRD1 | ENSG00000148677 | 0.90144968 | 2.29426E-05 | 0.02812339 |
| | ENSG00000262138 | 0.890758485 | 0.000316259 | 0.045270347 |
| EMP1 | ENSG00000134531 | 0.88564682 | 0.000156928 | 0.041478386 |
| ZFP36 | ENSG00000128016 | 0.87397337 | 4.74271E-05 | 0.032626663 |
| ERRFI1 | ENSG00000116285 | 0.859928131 | 3.80678E-05 | 0.031012256 |
| TXNIP | ENSG00000265972 | 0.856842995 | 0.000122585 | 0.041478386 |
| BIRC3 | ENSG00000023445 | 0.856700897 | 5.62446E-05 | 0.033090547 |
| CCND1 | ENSG00000110092 | 0.855659485 | 8.40694E-05 | 0.03863993 |
| YOD1 | ENSG00000180667 | 0.854465008 | 0.000334323 | 0.045270347 |
| PALD1 | ENSG00000107719 | 0.830342531 | 0.000105265 | 0.041478386 |
| ETS1 | ENSG00000134954 | 0.804966927 | 6.23216E-05 | 0.033759972 |
| UBASH3B | ENSG00000154127 | 0.799459934 | 3.07639E-05 | 0.029730093 |
| SPRED1 | ENSG00000166068 | 0.795418739 | 0.00011558 | 0.041478386 |
| MAFF | ENSG00000185022 | 0.794477701 | 0.000309681 | 0.045270347 |
| PHLDA2 | ENSG00000181649 | 0.785306931 | 0.0001187 | 0.041478386 |
| TRRAP | ENSG00000196367 | 0.768744469 | 1.22199E-05 | 0.02812339 |
| DYRK1A | ENSG00000157540 | 0.766916275 | 0.000397629 | 0.048846888 |
| NFKBIZ | ENSG00000144802 | 0.75342679 | 0.000273466 | 0.043915682 |
| EPHA2 | ENSG00000142627 | 0.750493526 | 0.000110011 | 0.041478386 |
| LCA5 | ENSG00000135338 | 0.728319168 | 0.000346757 | 0.045678955 |
| TBX3 | ENSG00000135111 | 0.718566656 | 0.000131309 | 0.041478386 |
| RHOB | ENSG00000143878 | 0.708977699 | 0.000128925 | 0.041478386 |
| ITGA6 | ENSG00000091409 | 0.706949711 | 3.27273E-05 | 0.029798249 |

| | | | | |
|---|---|---|---|---|
| RGS2 | ENSG00000116741 | 0.706371784 | 0.000171598 | 0.041478386 |
| ICAM1 | ENSG00000090339 | 0.69771862 | 0.000236876 | 0.043915682 |
| ZBTB43 | ENSG00000169155 | 0.697408676 | 0.000376409 | 0.04736789 |
| ABL2 | ENSG00000143322 | 0.686911583 | 8.62981E-05 | 0.038717833 |
| BCL10 | ENSG00000142867 | 0.684662819 | 5.26994E-05 | 0.033090547 |
| FAM46A,TENT5A | ENSG00000112773 | 0.67272377 | 0.000180168 | 0.041478386 |
| TNFRSF10B | ENSG00000120889 | 0.666843414 | 3.73178E-05 | 0.031012256 |
| MYC | ENSG00000136997 | 0.662443161 | 0.000207381 | 0.041478386 |
| SLC25A25 | ENSG00000148339 | 0.65127039 | 0.000166326 | 0.041478386 |
| VIM | ENSG00000026025 | 0.650017262 | 0.000312239 | 0.045270347 |
| AFAP1 | ENSG00000196526 | 0.643449306 | 0.000141436 | 0.041478386 |
| SPATA2 | ENSG00000158480 | 0.635526657 | 0.000194594 | 0.041478386 |
| | ENSG00000182319 | 0.631081104 | 0.00021036 | 0.041478386 |
| AXL | ENSG00000167601 | 0.629801273 | 0.000222075 | 0.042967436 |
| DCBLD2 | ENSG00000057019 | 0.611116886 | 0.000305125 | 0.045270347 |
| LDLR | ENSG00000130164 | 0.60457325 | 0.000235087 | 0.043915682 |
| PHLDA1 | ENSG00000139289 | 0.600854874 | 0.000250697 | 0.043915682 |
| SEPHS2 | ENSG00000179918 | -0.618843079 | 0.000147545 | 0.041478386 |
| CTSV | ENSG00000136943 | -0.633986473 | 2.74511E-05 | 0.028326815 |
| FABP1 | ENSG00000163586 | -0.645821095 | 6.43421E-05 | 0.033759972 |
| EPHX2 | ENSG00000120915 | -0.656505108 | 0.000357077 | 0.046058428 |
| PMM1 | ENSG00000100417 | -0.672981739 | 0.000239266 | 0.043915682 |
| ALDH1A1 | ENSG00000165092 | -0.686564445 | 6.34517E-05 | 0.033759972 |
| TSPAN8 | ENSG00000127324 | -0.697278023 | 3.01126E-05 | 0.029730093 |
| AGT | ENSG00000135744 | -0.703473568 | 4.92603E-05 | 0.033090547 |
| SMIM14 | ENSG00000163683 | -0.714872837 | 3.58118E-05 | 0.030795168 |
| B4GALNT2 | ENSG00000167080 | -0.717692375 | 0.000148461 | 0.041478386 |
| AHSG | ENSG00000145192 | -0.718997955 | 4.10888E-05 | 0.03150835 |
| PDZK1 | ENSG00000174827 | -0.73416996 | 0.000322782 | 0.045270347 |
| SERINC5 | ENSG00000164300 | -0.748405933 | 0.00017636 | 0.041478386 |
| MXD4 | ENSG00000123933 | -0.799379826 | 0.000256992 | 0.043915682 |
| HZGJ,RP11-474G23.1,AC017083.3 | ENSG00000273398 | -0.809195518 | 0.000150601 | 0.041478386 |
| OAT | ENSG00000065154 | -0.83012104 | 1.58607E-05 | 0.02812339 |
| FGG | ENSG00000171557 | -0.867315769 | 0.00016373 | 0.041478386 |
| SLC39A5 | ENSG00000139540 | -0.872833729 | 0.000277456 | 0.043915682 |
| REEP6 | ENSG00000115255 | -0.892915726 | 0.000175311 | 0.041478386 |
| TTR | ENSG00000118271 | -0.92826128 | 0.00017869 | 0.041478386 |
| AKR1C3 | ENSG00000196139 | -0.939577579 | 0.000185813 | 0.041478386 |
| LAMA1 | ENSG00000101680 | -0.97177887 | 7.52822E-05 | 0.037085385 |
| CTD-2278I10.6,AC010463.1 | ENSG00000269307 | -1.002472281 | 0.000396387 | 0.048846888 |
| NDRG1 | ENSG00000104419 | -1.044463158 | 0.000133881 | 0.041478386 |
| | ENSG00000261023 | -1.059534073 | 8.23057E-05 | 0.038605113 |
| GSTA2 | ENSG00000244067 | -1.118533611 | 0.000268291 | 0.043915682 |
| SERPINA6 | ENSG00000170099 | -1.179878712 | 0.000240423 | 0.043915682 |
| PRR15L | ENSG00000167183 | -1.283981323 | 0.000140213 | 0.041478386 |
| AFP | ENSG00000081051 | -1.326880932 | 9.32744E-06 | 0.02812339 |
| GJB1 | ENSG00000169562 | -1.391951561 | 0.000354469 | 0.045913355 |
| PI3 | ENSG00000124102 | -1.439826965 | 0.000149853 | 0.041478386 |

| | | | | |
|---|---|---|---|---|
| | ENSG00000197775 | -1.525665522 | 0.000144937 | 0.041478386 |
| INAFM1 | ENSG00000257704 | -2.267014265 | 7.56323E-05 | 0.037085385 |

**Supplementary Table S4. List of differentially expressed genes for the condition "Caco-2_SARS-CoV versus Caco-2_Mock".** The filters for the extraction were defined as Log-ratio ≥ 1.0 and FDR ≤ 0.05. The number of differentially expressed genes is 187.

| **Gene Symbol** | **Gene** | **Log-ratio** | **p-value** | **minFDR** |
|---|---|---|---|---|
| | ENSG00000264082 | 2.913762331 | 1.9432E-08 | 0.000297252 |
| RPL23AP42 | ENSG00000234851 | 2.892400026 | 0.000133103 | 0.010279325 |
| AC104534.3,AC008982.1 | ENSG00000268083 | 2.609372377 | 0.002847 | 0.046337842 |
| IL11 | ENSG00000095752 | 2.218338013 | 5.19207E-07 | 0.000765385 |
| KRT6A | ENSG00000205420 | 2.118908525 | 1.43861E-07 | 0.000678141 |
| DUSP4 | ENSG00000120875 | 2.115226746 | 0.000396497 | 0.016977258 |
| FOSL1 | ENSG00000175592 | 2.088470459 | 5.74049E-05 | 0.006908422 |
| DUSP5 | ENSG00000138166 | 2.082179546 | 1.0777E-05 | 0.003096294 |
| AKAP12 | ENSG00000131016 | 2.044735193 | 3.57063E-05 | 0.005238678 |
| SERPINE1 | ENSG00000106366 | 2.044528484 | 8.40442E-08 | 0.000650439 |
| EGR1 | ENSG00000120738 | 1.994570255 | 1.52809E-06 | 0.001244867 |
| ARRDC3 | ENSG00000113369 | 1.942744732 | 0.000198099 | 0.012289703 |
| JUNB | ENSG00000171223 | 1.893191814 | 1.22493E-07 | 0.000678141 |
| TNS4 | ENSG00000131746 | 1.867113233 | 1.26793E-06 | 0.001159846 |
| CXCL2 | ENSG00000081041 | 1.796375275 | 2.48431E-05 | 0.004601301 |
| ITGA2 | ENSG00000164171 | 1.740913868 | 3.69452E-05 | 0.00535128 |
| RP11-347C12.1,NPIPB13 | ENSG00000198064 | 1.738726139 | 0.000128032 | 0.010059585 |
| NR1D1 | ENSG00000126368 | 1.679545879 | 1.31995E-06 | 0.001159846 |
| HOXB8 | ENSG00000120068 | 1.65054512 | 4.96001E-05 | 0.00639779 |
| SH2D5 | ENSG00000189410 | 1.636523962 | 7.75702E-05 | 0.007873245 |
| ETS1 | ENSG00000134954 | 1.635138512 | 1.49739E-05 | 0.003511723 |
| RSC1A1 | ENSG00000215695 | 1.608579874 | 0.000223275 | 0.013095038 |
| DUSP6 | ENSG00000139318 | 1.605638504 | 5.91887E-06 | 0.002406185 |
| RGS2 | ENSG00000116741 | 1.575938702 | 9.3279E-05 | 0.008653657 |
| IER3 | ENSG00000227231 | 1.567859888 | 3.77932E-07 | 0.000765385 |
| IER3 | ENSG00000230128 | 1.567859888 | 3.77932E-07 | 0.000765385 |
| IER3 | ENSG00000235030 | 1.567859888 | 3.77932E-07 | 0.000765385 |
| IER3 | ENSG00000237155 | 1.567859888 | 3.77932E-07 | 0.000765385 |
| KLF6 | ENSG00000067082 | 1.566819191 | 2.88063E-08 | 0.000297252 |
| PLK2 | ENSG00000145632 | 1.559546471 | 3.63636E-06 | 0.001974563 |
| SPRY4 | ENSG00000187678 | 1.531535625 | 6.20914E-06 | 0.002406185 |
| PPP1R15A | ENSG00000087074 | 1.530666351 | 1.109E-08 | 0.000297252 |
| MMP1 | ENSG00000196611 | 1.530371666 | 0.001376585 | 0.031262106 |
| EMP1 | ENSG00000134531 | 1.528762341 | 6.75115E-05 | 0.007411181 |
| FOS | ENSG00000170345 | 1.518527746 | 1.16884E-06 | 0.001159846 |
| NAV3 | ENSG00000067798 | 1.512347937 | 5.03346E-05 | 0.006441695 |
| ATF3 | ENSG00000162772 | 1.491110802 | 0.00018922 | 0.012152859 |
| AGPAT9,GPAT3 | ENSG00000138678 | 1.463978291 | 0.000253978 | 0.013891142 |
| SOCS3 | ENSG00000184557 | 1.453872442 | 1.21703E-05 | 0.003143347 |
| MAFF | ENSG00000185022 | 1.435178995 | 1.75247E-07 | 0.000678141 |
| ZNF844 | ENSG00000223547 | 1.432496786 | 0.000711549 | 0.022387328 |
| GREM1 | ENSG00000166923 | 1.429099083 | 0.000375822 | 0.016556145 |

| | | | | |
|---|---|---|---|---|
| PHLDA2 | ENSG00000181649 | 1.421082973 | 1.17213E-05 | 0.003143347 |
| IGFBP4 | ENSG00000141753 | 1.393435001 | 2.0787E-07 | 0.000715003 |
| DUSP1 | ENSG00000120129 | 1.382104397 | 0.003146604 | 0.048718859 |
| MDM2 | ENSG00000135679 | 1.371710539 | 4.97246E-07 | 0.000765385 |
| C6orf141 | ENSG00000197261 | 1.357028723 | 0.001090098 | 0.027736599 |
| MXD1 | ENSG00000059728 | 1.334882498 | 0.000296815 | 0.01472515 |
| COLEC11 | ENSG00000118004 | 1.33024504 | 0.000647875 | 0.021178749 |
| PTGS2 | ENSG00000073756 | 1.307275534 | 6.64378E-06 | 0.002533586 |
| TNFAIP3 | ENSG00000118503 | 1.305113316 | 7.55453E-07 | 0.000989953 |
| CYP1A1 | ENSG00000140465 | 1.298588753 | 7.83553E-06 | 0.002758996 |
| KLF10 | ENSG00000155090 | 1.297531128 | 4.14197E-06 | 0.002089151 |
| | ENSG00000256390 | 1.28362456 | 0.001549136 | 0.033303202 |
| IER2 | ENSG00000160888 | 1.264362812 | 4.57513E-07 | 0.000765385 |
| ITGA6 | ENSG00000091409 | 1.257591724 | 0.000295693 | 0.01472515 |
| RP11-1212A22.4,NPIPA9 | ENSG00000233024 | 1.231235981 | 0.002329182 | 0.041415563 |
| RASA3 | ENSG00000185989 | 1.188993752 | 0.00026435 | 0.014195812 |
| ZFP36 | ENSG00000128016 | 1.187001228 | 8.3145E-07 | 0.001029568 |
| WNK4 | ENSG00000126562 | 1.185547948 | 0.000142632 | 0.010663601 |
| ITGA3 | ENSG00000005884 | 1.183428288 | 7.90418E-06 | 0.002758996 |
| HDAC9 | ENSG00000048052 | 1.180295467 | 0.00075999 | 0.02297559 |
| MUC13 | ENSG00000173702 | 1.179741859 | 2.52973E-07 | 0.000765385 |
| TBC1D2 | ENSG00000095383 | 1.175106525 | 0.001347759 | 0.031021436 |
| MTF1 | ENSG00000188786 | 1.17098093 | 0.000111502 | 0.009278913 |
| INHBA | ENSG00000122641 | 1.170943648 | 1.96298E-05 | 0.004162182 |
| ERRFI1 | ENSG00000116285 | 1.167915821 | 1.08696E-06 | 0.001121638 |
| LIF | ENSG00000128342 | 1.155982018 | 9.7233E-07 | 0.001075015 |
| EPHA2 | ENSG00000142627 | 1.152861595 | 3.92592E-06 | 0.002025579 |
| | ENSG00000263008 | 1.147356749 | 0.000610239 | 0.020785182 |
| SH2D3A | ENSG00000125731 | 1.141928673 | 0.000641282 | 0.021111919 |
| PLAUR | ENSG00000011422 | 1.13404274 | 3.99836E-05 | 0.005600774 |
| ESAM | ENSG00000149564 | 1.133100033 | 2.29185E-05 | 0.004419956 |
| CAPRIN2 | ENSG00000110888 | 1.130262613 | 6.21187E-05 | 0.00714873 |
| ZRSR1,ZRSR2P1 | ENSG00000212643 | 1.117001176 | 0.000362623 | 0.016411864 |
| HIVEP2 | ENSG00000010818 | 1.107463598 | 0.001692539 | 0.035047452 |
| HRH1 | ENSG00000196639 | 1.104166389 | 3.21004E-05 | 0.005011071 |
| TM4SF1 | ENSG00000169908 | 1.098994255 | 7.32471E-05 | 0.007686473 |
| AC107982.4,AC107982.1 | ENSG00000250111 | 1.095039368 | 0.002745619 | 0.045258178 |
| MYC | ENSG00000136997 | 1.081147194 | 0.000114845 | 0.009326656 |
| BHLHE40 | ENSG00000134107 | 1.081020355 | 2.47602E-06 | 0.001630857 |
| GADD45B | ENSG00000099860 | 1.079658985 | 7.73415E-06 | 0.002758996 |
| ALOXE3 | ENSG00000179148 | 1.076103687 | 0.000963699 | 0.026145387 |
| TXNIP | ENSG00000265972 | 1.075985432 | 4.93635E-07 | 0.000765385 |
| PALD1 | ENSG00000107719 | 1.074549437 | 2.59333E-05 | 0.004601301 |
| PLK3 | ENSG00000173846 | 1.07282877 | 2.68246E-05 | 0.004601301 |
| FHL2 | ENSG00000115641 | 1.068516254 | 2.1894E-06 | 0.001506159 |
| KLF4 | ENSG00000136826 | 1.065795422 | 7.05518E-06 | 0.002631412 |
| LAMC2 | ENSG00000058085 | 1.061277866 | 0.001508598 | 0.032842605 |

| | | | | |
|---|---|---|---|---|
| CXCL8 | ENSG00000169429 | 1.059239626 | 0.000637033 | 0.021111919 |
| MYO15B | ENSG00000266714 | 1.047090292 | 0.000773799 | 0.023099794 |
| ANKRD1 | ENSG00000148677 | 1.04338789 | 1.67927E-07 | 0.000678141 |
| RHOB | ENSG00000143878 | 1.036942959 | 2.95373E-05 | 0.004787357 |
| AC093616.4,AC093616.1 | ENSG00000234231 | 1.031179667 | 0.002266641 | 0.040724552 |
| NOCT | ENSG00000151014 | 1.030690193 | 8.66212E-05 | 0.008443539 |
| EGR2 | ENSG00000122877 | 1.028788567 | 6.03321E-05 | 0.007022514 |
| FMN1 | ENSG00000248905 | 1.027231932 | 1.62812E-06 | 0.001260046 |
| TINAGL1 | ENSG00000142910 | 1.01947546 | 0.000667192 | 0.021582308 |
| DUSP2 | ENSG00000158050 | 1.01544714 | 0.00160204 | 0.033824257 |
| LDLR | ENSG00000130164 | 1.014693737 | 4.77109E-05 | 0.006232011 |
| KRT17 | ENSG00000128422 | 1.010590553 | 0.003066873 | 0.048169052 |
| ABL2 | ENSG00000143322 | 1.002349854 | 1.34879E-06 | 0.001159846 |
| VAV3 | ENSG00000134215 | -1.002313256 | 0.000189886 | 0.012170425 |
| CTD-2278I10.6,AC010463.1 | ENSG00000269307 | -1.002472281 | 3.49187E-05 | 0.005238678 |
| VPS45 | ENSG00000136631 | -1.014633417 | 2.4008E-06 | 0.001615689 |
| ICAM4 | ENSG00000105371 | -1.016668797 | 0.001411699 | 0.031714061 |
| IL22RA1 | ENSG00000142677 | -1.017124772 | 0.001807892 | 0.036107695 |
| MAP2K6 | ENSG00000108984 | -1.021133423 | 0.000363725 | 0.016437707 |
| SLC26A2 | ENSG00000155850 | -1.024189472 | 1.14801E-05 | 0.003143347 |
| ADD3-AS1 | ENSG00000203876 | -1.025950238 | 0.001764887 | 0.035686217 |
| GCNT1 | ENSG00000187210 | -1.031867266 | 9.36452E-05 | 0.008653657 |
| HAPLN3 | ENSG00000140511 | -1.034358025 | 0.000169338 | 0.011597767 |
| GSTA4 | ENSG00000170899 | -1.039404631 | 0.00025905 | 0.014042403 |
| | ENSG00000263330 | -1.040331841 | 3.43026E-05 | 0.005205423 |
| TMPRSS2 | ENSG00000184012 | -1.042368054 | 1.15747E-05 | 0.003143347 |
| NAV2 | ENSG00000166833 | -1.046043396 | 0.00020153 | 0.012452593 |
| RMI2 | ENSG00000175643 | -1.047966719 | 9.11789E-05 | 0.008553406 |
| NEU4 | ENSG00000204099 | -1.052319288 | 0.000617871 | 0.020858718 |
| ALDH6A1 | ENSG00000119711 | -1.058932066 | 8.44709E-06 | 0.002796863 |
| SOAT2 | ENSG00000167780 | -1.05986917 | 0.00035497 | 0.016279736 |
| CHRNB1 | ENSG00000170175 | -1.06359452 | 0.000693753 | 0.022209429 |
| EPSTI1 | ENSG00000133106 | -1.068480492 | 2.16824E-05 | 0.004343695 |
| F7 | ENSG00000057593 | -1.076420426 | 0.00019986 | 0.012374146 |
| PLA2G12B | ENSG00000138308 | -1.078021288 | 0.00023266 | 0.01331321 |
| ROM1 | ENSG00000149489 | -1.083981484 | 0.000451248 | 0.018189178 |
| EHHADH | ENSG00000113790 | -1.085677862 | 7.14338E-05 | 0.007599235 |
| C11orf71 | ENSG00000180425 | -1.091627121 | 0.00128565 | 0.030289081 |
| OAT | ENSG00000065154 | -1.096996784 | 7.6748E-07 | 0.000989953 |
| PLXNC1 | ENSG00000136040 | -1.105926394 | 5.30451E-05 | 0.006648246 |
| SERINC5 | ENSG00000164300 | -1.118800163 | 6.07642E-06 | 0.002406185 |
| EEF1A1P6 | ENSG00000233476 | -1.118864655 | 0.000911454 | 0.025399266 |
| | ENSG00000270942 | -1.119078517 | 0.001079061 | 0.027652712 |
| DNAJC15 | ENSG00000120675 | -1.119117498 | 0.000106934 | 0.009176887 |
| SERPINA10 | ENSG00000140093 | -1.120224833 | 9.30557E-05 | 0.008653657 |
| G0S2 | ENSG00000123689 | -1.121457577 | 3.28698E-05 | 0.005037373 |
| FAM114A1 | ENSG00000197712 | -1.121939659 | 0.000234212 | 0.013352688 |

| | | | | |
|---|---|---|---|---|
| GALM | ENSG00000143891 | -1.132113695 | 2.29871E-05 | 0.004419956 |
| PBXIP1 | ENSG00000163346 | -1.134217262 | 7.62971E-05 | 0.007820962 |
| SMTNL2 | ENSG00000188176 | -1.135017395 | 2.57198E-05 | 0.004601301 |
| NDRG1 | ENSG00000104419 | -1.137112856 | 2.03639E-06 | 0.001466056 |
| ADH4 | ENSG00000198099 | -1.143721372 | 0.000223742 | 0.013095038 |
| DRICH1 | ENSG00000189269 | -1.144576073 | 0.000159324 | 0.011241338 |
| PRR15L | ENSG00000167183 | -1.146744728 | 5.51736E-05 | 0.006777817 |
| TJP3 | ENSG00000105289 | -1.14744091 | 0.000205464 | 0.012595134 |
| AKR1C3 | ENSG00000196139 | -1.149261236 | 8.32888E-06 | 0.002796863 |
| MXD4 | ENSG00000123933 | -1.159184694 | 4.90799E-06 | 0.002170524 |
| DUSP9 | ENSG00000130829 | -1.160633326 | 7.71131E-06 | 0.002758996 |
| ZNF362 | ENSG00000160094 | -1.17718184 | 0.000114962 | 0.009326656 |
| HSPA1L | ENSG00000226704 | -1.178649783 | 6.17816E-05 | 0.00713647 |
| CYP27A1 | ENSG00000135929 | -1.183240414 | 3.89348E-05 | 0.005503671 |
| SERPINF2 | ENSG00000167711 | -1.185405731 | 4.62859E-06 | 0.002107165 |
| | ENSG00000269798 | -1.194560528 | 7.65115E-06 | 0.002758996 |
| CLDN3 | ENSG00000165215 | -1.210625768 | 0.000229736 | 0.013243848 |
| | ENSG00000262073 | -1.210625768 | 0.000229736 | 0.013243848 |
| AMACR | ENSG00000242110 | -1.211127281 | 1.27134E-05 | 0.003199743 |
| GSTA2 | ENSG00000244067 | -1.215881348 | 1.70748E-05 | 0.003777876 |
| SMIM14 | ENSG00000163683 | -1.217028141 | 1.10916E-05 | 0.003125565 |
| | ENSG00000267596 | -1.222908735 | 0.00040186 | 0.017127333 |
| RBBP8NL | ENSG00000130701 | -1.227137089 | 0.000188837 | 0.012152859 |
| ESPN | ENSG00000187017 | -1.232202888 | 0.001289326 | 0.030329539 |
| DPYSL3 | ENSG00000113657 | -1.236472845 | 0.001228246 | 0.029489552 |
| | ENSG00000265111 | -1.236798763 | 1.40625E-05 | 0.003371672 |
| PDCD4 | ENSG00000150593 | -1.245380878 | 0.000259465 | 0.014042403 |
| TMEM37 | ENSG00000171227 | -1.263756275 | 7.14037E-05 | 0.007599235 |
| PDZK1 | ENSG00000174827 | -1.2672472 | 3.03797E-05 | 0.004898255 |
| MYBPH | ENSG00000133055 | -1.278821111 | 0.001592379 | 0.033787038 |
| ANXA9 | ENSG00000143412 | -1.291270018 | 3.65927E-05 | 0.005343398 |
| RGN | ENSG00000130988 | -1.301668167 | 0.000635628 | 0.021111919 |
| | ENSG00000270349 | -1.301668167 | 0.000635628 | 0.021111919 |
| AMOT | ENSG00000126016 | -1.303818941 | 0.000143371 | 0.010669064 |
| ACOT1 | ENSG00000184227 | -1.311069489 | 0.000687127 | 0.0220429 |
| ALG1L | ENSG00000189366 | -1.314981699 | 0.000329968 | 0.015550778 |
| SLC29A4 | ENSG00000164638 | -1.320050478 | 9.56495E-05 | 0.008677762 |
| KLC4 | ENSG00000137171 | -1.399628639 | 8.96356E-05 | 0.008467741 |
| METTL7A | ENSG00000185432 | -1.417626381 | 2.87439E-05 | 0.004733109 |
| SORBS2 | ENSG00000154556 | -1.425181031 | 2.93015E-05 | 0.004780139 |
| EHMT2 | ENSG00000224143 | -1.499618411 | 0.001221416 | 0.029471063 |
| B4GALNT2 | ENSG00000167080 | -1.505003214 | 0.000478634 | 0.018521322 |
| EXOC3L4 | ENSG00000205436 | -1.508199096 | 0.000193794 | 0.012242365 |
| HMGCS2 | ENSG00000134240 | -1.539391994 | 2.58612E-06 | 0.001633849 |
| SPTLC3 | ENSG00000172296 | -1.628162622 | 2.71793E-05 | 0.004601301 |
| | ENSG00000268059 | -1.66115427 | 5.80105E-05 | 0.006910025 |
| GJB1 | ENSG00000169562 | -1.782355309 | 2.84417E-06 | 0.00172641 |

| | | | | |
|---|---|---|---|---|
| | ENSG00000257123 | -2.073791265 | 0.000618733 | 0.020865036 |
| CYMP | ENSG00000240194 | -2.08025527 | 0.000410845 | 0.017375038 |
| LAMA1 | ENSG00000101680 | -2.27575326 | 1.75534E-06 | 0.001325369 |
| EEF1A1P5 | ENSG00000196205 | -2.724304199 | 3.5643E-05 | 0.005238678 |

**Supplementary Table S5. List of differentially expressed genes for the condition "Calu-3_SARS-CoV-2 versus Calu-3_Mock" subjected to IPA Core Analysis.**
The number of differentially expressed genes is 2890.

| Symbol | ID | Entrez Gene Name | Expr Log Ratio | Location | Type(s) |
|---|---|---|---|---|---|
| A4GALT | ENSG00000128274 | alpha 1,4-galactosyltransferase (P blood group) | 0,812 | Cytoplasm | enzyme |
| AADAT | ENSG00000109576 | aminoadipate aminotransferase | -0,892 | Cytoplasm | enzyme |
| AASS | ENSG00000008311 | aminoadipate-semialdehyde synthase | 0,802 | Cytoplasm | enzyme |
| ABAT | ENSG00000183044 | 4-aminobutyrate aminotransferase | -0,773 | Cytoplasm | enzyme |
| ABCA2 | ENSG00000107331 | ATP binding cassette subfamily A member 2 | -0,687 | Plasma Membrane | transporter |
| ABCA3 | ENSG00000167972 | ATP binding cassette subfamily A member 3 | -1,299 | Plasma Membrane | transporter |
| ABCA5 | ENSG00000154265 | ATP binding cassette subfamily A member 5 | 0,779 | Plasma Membrane | transporter |
| ABCC4 | ENSG00000125257 | ATP binding cassette subfamily C member 4 | -0,976 | Plasma Membrane | transporter |
| ABCC9 | ENSG00000069431 | ATP binding cassette subfamily C member 9 | 0,639 | Plasma Membrane | ion channel |
| ABCD1 | ENSG00000101986 | ATP binding cassette subfamily D member 1 | 0,769 | Plasma Membrane | transporter |
| ABCD3 | ENSG00000117528 | ATP binding cassette subfamily D member 3 | -0,625 | Cytoplasm | transporter |
| ABCF1 | ENSG00000204574 | ATP binding cassette subfamily F member 1 | 0,617 | Cytoplasm | transporter |
| ABCG1 | ENSG00000160179 | ATP binding cassette subfamily G member 1 | 1,231 | Plasma Membrane | transporter |
| ABHD14A | ENSG00000248487 | abhydrolase domain containing 14A | -0,625 | Cytoplasm | enzyme |
| ABHD14B | ENSG00000114779 | abhydrolase domain containing 14B | -0,665 | Cytoplasm | enzyme |
| ABHD15 | ENSG00000168792 | abhydrolase domain containing 15 | -0,792 | Extracellular Space | other |
| ABHD16A | ENSG00000204427 | abhydrolase domain containing 16A, phospholipase | 0,895 | Other | other |
| ABHD2 | ENSG00000140526 | abhydrolase domain containing 2, acylglycerol lipase | -1,015 | Cytoplasm | enzyme |
| ABI3BP | ENSG00000154175 | ABI family member 3 binding protein | -0,829 | Extracellular Space | other |
| ABL2 | ENSG00000143322 | ABL proto-oncogene 2, non-receptor tyrosine kinase | 1,183 | Cytoplasm | kinase |
| ABRAXAS2 | ENSG00000165660 | abraxas 2, BRISC complex subunit | 0,619 | Nucleus | other |
| ABTB1 | ENSG00000114626 | ankyrin repeat and BTB domain containing 1 | -0,703 | Cytoplasm | translation regulator |
| ABTB2 | ENSG00000166016 | ankyrin repeat and BTB domain containing 2 | 0,903 | Nucleus | other |
| AC0156883 | ENSG00000266728 | | 2,150 | Other | other |
| AC0937242 | ENSG00000213222 | | 0,959 | Other | other |
| ACAA2 | ENSG00000167315 | acetyl-CoA acyltransferase 2 | -0,769 | Cytoplasm | enzyme |
| ACADSB | ENSG00000196177 | acyl-CoA dehydrogenase short/branched chain | -0,715 | Cytoplasm | enzyme |
| ACAT2 | ENSG00000120437 | acetyl-CoA acetyltransferase 2 | -1,352 | Cytoplasm | enzyme |
| ACE2 | ENSG00000130234 | angiotensin I converting enzyme 2 | -0,765 | Plasma Membrane | peptidase |
| ACHE | ENSG00000087085 | acetylcholinesterase (Cartwright blood group) | 2,312 | Plasma Membrane | enzyme |
| ACKR4 | ENSG00000129048 | atypical chemokine receptor 4 | 3,533 | Plasma Membrane | G-protein coupled receptor |
| ACO1 | ENSG00000122729 | aconitase 1 | -1,312 | Cytoplasm | enzyme |
| ACP2 | ENSG00000134575 | acid phosphatase 2, lysosomal | 0,799 | Cytoplasm | phosphatase |
| ACP7 | ENSG00000183760 | acid phosphatase 7, tartrate resistant (putative) | 0,876 | Other | enzyme |
| ACSF2 | ENSG00000167107 | acyl-CoA synthetase family member 2 | -0,809 | Cytoplasm | enzyme |
| ACSM3 | ENSG00000005187 | acyl-CoA synthetase medium chain family member 3 | -1,271 | Cytoplasm | enzyme |
| ACSS2 | ENSG00000131069 | acyl-CoA synthetase short chain family member 2 | -1,066 | Cytoplasm | enzyme |
| ACTA2 | ENSG00000107796 | actin alpha 2, smooth muscle | 0,885 | Cytoplasm | other |
| ACTL6A | ENSG00000136518 | actin like 6A | -0,612 | Nucleus | other |
| ACTN2 | ENSG00000077522 | actinin alpha 2 | 3,764 | Nucleus | transcription regulator |
| ACTR1B | ENSG00000115073 | actin related protein 1B | -0,627 | Cytoplasm | other |
| ACTR2 | ENSG00000138071 | actin related protein 2 | -0,793 | Plasma Membrane | other |
| ACVR1C | ENSG00000123612 | activin A receptor type 1C | 0,897 | Plasma Membrane | kinase |
| ACY3 | ENSG00000132744 | aminoacylase 3 | 1,060 | Cytoplasm | enzyme |
| ACYP1 | ENSG00000119640 | acylphosphatase 1 | -0,680 | Cytoplasm | enzyme |
| ADAM22 | ENSG00000008277 | ADAM metallopeptidase domain 22 | -0,860 | Plasma Membrane | peptidase |
| ADAM28 | ENSG00000042980 | ADAM metallopeptidase domain 28 | 0,803 | Plasma Membrane | peptidase |
| ADAM32 | ENSG00000197140 | ADAM metallopeptidase domain 32 | 0,782 | Other | peptidase |
| ADAM8 | ENSG00000151651 | ADAM metallopeptidase domain 8 | 1,587 | Plasma Membrane | peptidase |
| ADAMTS6 | ENSG00000049192 | ADAM metallopeptidase with thrombospondin type 1 motif 6 | 0,712 | Extracellular Space | peptidase |
| ADAMTSL3 | ENSG00000156218 | ADAMTS like 3 | 0,786 | Other | other |
| ADAR | ENSG00000160710 | adenosine deaminase RNA specific | 1,291 | Nucleus | enzyme |
| ADAT3 | ENSG00000213638 | adenosine deaminase tRNA specific 3 | 0,832 | Nucleus | enzyme |
| ADGRF4 | ENSG00000153294 | adhesion G protein-coupled receptor F4 | 0,946 | Plasma Membrane | G-protein coupled receptor |
| ADGRG1 | ENSG00000205336 | adhesion G protein-coupled receptor G1 | -0,610 | Plasma Membrane | G-protein coupled receptor |
| ADH5 | ENSG00000197894 | alcohol dehydrogenase 5 (class III), chi polypeptide | -0,657 | Cytoplasm | enzyme |
| ADH6 | ENSG00000172955 | alcohol dehydrogenase 6 (class V) | -0,756 | Cytoplasm | enzyme |
| ADI1 | ENSG00000182551 | acireductone dioxygenase 1 | -0,778 | Nucleus | enzyme |
| ADK | ENSG00000156110 | adenosine kinase | -0,632 | Nucleus | kinase |
| ADM | ENSG00000148926 | adrenomedullin | 1,755 | Extracellular Space | other |
| ADM2 | ENSG00000128165 | adrenomedullin 2 | 1,068 | Extracellular Space | other |
| ADORA1 | ENSG00000163485 | adenosine A1 receptor | -1,127 | Plasma Membrane | G-protein coupled receptor |
| ADPRS | ENSG00000116863 | ADP-ribosylserine hydrolase | 1,017 | Cytoplasm | enzyme |
| ADRA2A | ENSG00000150594 | adrenoceptor alpha 2A | -1,168 | Plasma Membrane | G-protein coupled receptor |
| ADRB2 | ENSG00000169252 | adrenoceptor beta 2 | 1,873 | Plasma Membrane | G-protein coupled receptor |
| ADSS1 | ENSG00000185100 | adenylosuccinate synthase 1 | -1,237 | Cytoplasm | enzyme |
| AEN | ENSG00000181026 | apoptosis enhancing nuclease | 0,972 | Nucleus | enzyme |
| AFAP1 | ENSG00000196526 | actin filament associated protein 1 | 1,805 | Cytoplasm | other |
| AFF4 | ENSG00000072364 | AF4/FMR2 family member 4 | 0,877 | Nucleus | transcription regulator |
| AGAP6 (includes others) | ENSG00000172650 | ArfGAP with GTPase domain, ankyrin repeat and PH domain 4 | 0,974 | Other | other |
| AGBL5 | ENSG00000084693 | ATP/GTP binding protein like 5 | -0,817 | Cytoplasm | peptidase |
| AGER | ENSG00000231268 | advanced glycosylation end-product specific receptor | 1,628 | Plasma Membrane | transmembrane receptor |
| AGFG2 | ENSG00000106351 | ArfGAP with FG repeats 2 | -0,633 | Other | other |
| AGO4 | ENSG00000134698 | argonaute RISC component 4 | -0,743 | Cytoplasm | translation regulator |
| AGT | ENSG00000135744 | angiotensinogen | -0,981 | Extracellular Space | growth factor |
| AGTPBP1 | ENSG00000135049 | ATP/GTP binding protein 1 | -0,935 | Nucleus | peptidase |
| AHCY | ENSG00000101444 | adenosylhomocysteinase | -0,690 | Cytoplasm | enzyme |
| AHCYL2 | ENSG00000158467 | adenosylhomocysteinase like 2 | 0,956 | Other | enzyme |
| AHNAK | ENSG00000124942 | AHNAK nucleoprotein | -1,619 | Nucleus | other |
| AHNAK2 | ENSG00000185567 | AHNAK nucleoprotein 2 | -1,494 | Cytoplasm | other |
| AHR | ENSG00000106546 | aryl hydrocarbon receptor | 1,003 | Nucleus | ligand-dependent nuclear receptor |
| AHRR | ENSG00000063438 | aryl-hydrocarbon receptor repressor | 1,622 | Nucleus | transcription regulator |
| AIF1L | ENSG00000126878 | allograft inflammatory factor 1 like | -1,150 | Plasma Membrane | other |
| AIFM2 | ENSG00000042286 | apoptosis inducing factor mitochondria associated 2 | 0,796 | Cytoplasm | enzyme |
| AK4 | ENSG00000162433 | adenylate kinase 4 | -0,633 | Cytoplasm | kinase |
| AKAP7 | ENSG00000118507 | A-kinase anchoring protein 7 | 1,108 | Plasma Membrane | other |
| AKAP8L | ENSG00000011243 | A-kinase anchoring protein 8 like | 0,946 | Nucleus | other |
| AKR1B10 | ENSG00000198074 | aldo-keto reductase family 1 member B10 | -1,479 | Cytoplasm | enzyme |
| AKR1C1/AKR1C2 | ENSG00000187134 | aldo-keto reductase family 1 member C2 | -0,890 | Cytoplasm | enzyme |
| AKR1C3 | ENSG00000196139 | aldo-keto reductase family 1 member C3 | -0,973 | Cytoplasm | enzyme |
| AKR7A2 | ENSG00000053371 | aldo-keto reductase family 7 member A2 | -0,704 | Cytoplasm | enzyme |
| ALCAM | ENSG00000170017 | activated leukocyte cell adhesion molecule | -1,203 | Plasma Membrane | other |
| ALDH16A1 | ENSG00000161618 | aldehyde dehydrogenase 16 family member A1 | -0,683 | Cytoplasm | enzyme |
| ALDH1A2 | ENSG00000128918 | aldehyde dehydrogenase 1 family member A2 | -0,675 | Cytoplasm | enzyme |
| ALDH3B2 | ENSG00000132746 | aldehyde dehydrogenase 3 family member B2 | -1,152 | Cytoplasm | enzyme |
| ALDH4A1 | ENSG00000159423 | aldehyde dehydrogenase 4 family member A1 | -0,685 | Cytoplasm | enzyme |

| | | | | | |
|---|---|---|---|---|---|
| ALDH6A1 | ENSG00000119711 | aldehyde dehydrogenase 6 family member A1 | -0,677 | Cytoplasm | enzyme |
| ALDH7A1 | ENSG00000164904 | aldehyde dehydrogenase 7 family member A1 | -0,947 | Cytoplasm | enzyme |
| ALG14 | ENSG00000172339 | ALG14 UDP-N-acetylglucosaminyltransferase subunit | -0,944 | Cytoplasm | enzyme |
| ALKBH7 | ENSG00000125652 | alkB homolog 7 | -0,625 | Cytoplasm | other |
| ALX1 | ENSG00000180318 | ALX homeobox 1 | 0,679 | Nucleus | transcription regulator |
| AMBRA1 | ENSG00000110497 | autophagy and beclin 1 regulator 1 | 1,579 | Cytoplasm | other |
| AMER1 | ENSG00000184675 | APC membrane recruitment protein 1 | 0,741 | Plasma Membrane | other |
| AMOTL2 | ENSG00000114019 | angiomotin like 2 | 0,982 | Plasma Membrane | other |
| AMZ2 | ENSG00000196704 | archaelysin family metallopeptidase 2 | -0,665 | Other | other |
| ANAPC10 | ENSG00000164162 | anaphase promoting complex subunit 10 | 0,774 | Nucleus | other |
| ANAPC11 | ENSG00000141552 | anaphase promoting complex subunit 11 | -0,693 | Cytoplasm | enzyme |
| ANAPC15 | ENSG00000110200 | anaphase promoting complex subunit 15 | -1,113 | Nucleus | other |
| ANAPC16 | ENSG00000166295 | anaphase promoting complex subunit 16 | -0,700 | Nucleus | other |
| ANG | ENSG00000214274 | angiogenin | -0,849 | Extracellular Space | enzyme |
| ANGPT2 | ENSG00000091879 | angiopoietin 2 | 2,399 | Extracellular Space | growth factor |
| ANGPTL4 | ENSG00000167772 | angiopoietin like 4 | 1,616 | Extracellular Space | other |
| ANKFY1 | ENSG00000185722 | ankyrin repeat and FYVE domain containing 1 | 0,673 | Cytoplasm | transcription regulator |
| ANKH | ENSG00000154122 | ANKH inorganic pyrophosphate transport regulator | -0,878 | Plasma Membrane | transporter |
| ANKIB1 | ENSG00000001629 | ankyrin repeat and IBR domain containing 1 | 0,877 | Nucleus | transcription regulator |
| ANKMY1 | ENSG00000144504 | ankyrin repeat and MYND domain containing 1 | -0,745 | Other | other |
| ANKRD1 | ENSG00000148677 | ankyrin repeat domain 1 | 1,280 | Cytoplasm | transcription regulator |
| ANKRD10 | ENSG00000088448 | ankyrin repeat domain 10 | 0,632 | Nucleus | transcription regulator |
| ANKRD12 | ENSG00000101745 | ankyrin repeat domain 12 | 0,871 | Nucleus | other |
| ANKRD18B | ENSG00000230453 | ankyrin repeat domain 18B | 1,002 | Other | other |
| ANKRD33B | ENSG00000164236 | ankyrin repeat domain 33B | 2,216 | Other | other |
| ANKS4B | ENSG00000175311 | ankyrin repeat and sterile alpha motif domain containing 4B | -0,689 | Nucleus | transcription regulator |
| ANO3 | ENSG00000134343 | anoctamin 3 | 0,631 | Plasma Membrane | transporter |
| ANO9 | ENSG00000185101 | anoctamin 9 | 0,671 | Plasma Membrane | ion channel |
| ANP32E | ENSG00000143401 | acidic nuclear phosphoprotein 32 family member E | -0,693 | Nucleus | other |
| ANTXR2 | ENSG00000163297 | ANTXR cell adhesion molecule 2 | 0,687 | Plasma Membrane | transmembrane receptor |
| ANXA10 | ENSG00000109511 | annexin A10 | -0,989 | Cytoplasm | other |
| ANXA13 | ENSG00000104537 | annexin A13 | -1,763 | Plasma Membrane | other |
| ANXA2 | ENSG00000182718 | annexin A2 | -0,841 | Plasma Membrane | other |
| ANXA2R | ENSG00000177721 | annexin A2 receptor | -0,717 | Plasma Membrane | other |
| ANXA4 | ENSG00000196975 | annexin A4 | -0,951 | Plasma Membrane | other |
| ANXA9 | ENSG00000143412 | annexin A9 | -1,435 | Plasma Membrane | transmembrane receptor |
| AP000350.10 | ENSG00000251357 | | -1,891 | Other | other |
| AP1S2 | ENSG00000182287 | adaptor related protein complex 1 subunit sigma 2 | -0,626 | Cytoplasm | transporter |
| AP2M1 | ENSG00000161203 | adaptor related protein complex 2 subunit mu 1 | -0,808 | Cytoplasm | other |
| AP3B2 | ENSG00000103723 | adaptor related protein complex 3 subunit beta 2 | 0,924 | Cytoplasm | other |
| AP5Z1 | ENSG00000242802 | adaptor related protein complex 5 subunit zeta 1 | 1,004 | Nucleus | other |
| APBA3 | ENSG00000011132 | amyloid beta precursor protein binding family A member 3 | 0,771 | Cytoplasm | transporter |
| APOBEC3F | ENSG00000128394 | apolipoprotein B mRNA editing enzyme catalytic subunit 3F | 1,353 | Cytoplasm | enzyme |
| APOBEC3G | ENSG00000239713 | apolipoprotein B mRNA editing enzyme catalytic subunit 3G | 3,310 | Nucleus | enzyme |
| APOE | ENSG00000130203 | apolipoprotein E | -0,710 | Extracellular Space | transporter |
| APOL1 | ENSG00000100342 | apolipoprotein L1 | 1,612 | Extracellular Space | transporter |
| APOL2 | ENSG00000128335 | apolipoprotein L2 | 1,910 | Cytoplasm | other |
| APOL3 | ENSG00000128284 | apolipoprotein L3 | 4,234 | Cytoplasm | transporter |
| APOL4 | ENSG00000100336 | apolipoprotein L4 | 2,528 | Extracellular Space | other |
| APOL6 | ENSG00000221963 | apolipoprotein L6 | 3,551 | Extracellular Space | transporter |
| AQP4 | ENSG00000171885 | aquaporin 4 | -1,226 | Plasma Membrane | transporter |
| ARAP2 | ENSG00000047365 | ArfGAP with RhoGAP domain, ankyrin repeat and PH domain 2 | 1,380 | Cytoplasm | other |
| ARC | ENSG00000198576 | activity regulated cytoskeleton associated protein | 1,432 | Cytoplasm | other |
| AREG | ENSG00000109321 | amphiregulin | 1,371 | Extracellular Space | growth factor |
| ARHGAP11A | ENSG00000198826 | Rho GTPase activating protein 11A | -0,691 | Cytoplasm | other |
| ARHGAP22 | ENSG00000128805 | Rho GTPase activating protein 22 | 1,347 | Cytoplasm | other |
| ARHGAP24 | ENSG00000138639 | Rho GTPase activating protein 24 | 1,409 | Cytoplasm | other |
| ARHGAP26 | ENSG00000145819 | Rho GTPase activating protein 26 | 1,616 | Cytoplasm | other |
| ARHGAP33 | ENSG00000004777 | Rho GTPase activating protein 33 | -0,818 | Plasma Membrane | transporter |
| ARHGAP42 | ENSG00000165895 | Rho GTPase activating protein 42 | 0,840 | Other | other |
| ARHGAP44 | ENSG00000006740 | Rho GTPase activating protein 44 | 0,981 | Cytoplasm | other |
| ARHGDIB | ENSG00000111348 | Rho GDP dissociation inhibitor beta | -1,569 | Cytoplasm | enzyme |
| ARHGEF11 | ENSG00000132694 | Rho guanine nucleotide exchange factor 11 | 0,608 | Cytoplasm | other |
| ARHGEF17 | ENSG00000110237 | Rho guanine nucleotide exchange factor 17 | -0,603 | Cytoplasm | other |
| ARHGEF2 | ENSG00000116584 | Rho/Rac guanine nucleotide exchange factor 2 | 0,871 | Cytoplasm | other |
| ARHGEF28 | ENSG00000214944 | Rho guanine nucleotide exchange factor 28 | 1,228 | Cytoplasm | other |
| ARHGEF38 | ENSG00000236699 | Rho guanine nucleotide exchange factor 38 | 0,729 | Other | other |
| ARHGEF9 | ENSG00000131089 | Cdc42 guanine nucleotide exchange factor 9 | -0,620 | Cytoplasm | other |
| ARID3A | ENSG00000116017 | AT-rich interaction domain 3A | 0,913 | Nucleus | transcription regulator |
| ARID3B | ENSG00000179361 | AT-rich interaction domain 3B | 1,153 | Nucleus | transcription regulator |
| ARID4B | ENSG00000054267 | AT-rich interaction domain 4B | 1,333 | Nucleus | transcription regulator |
| ARID5A | ENSG00000196843 | AT-rich interaction domain 5A | 1,006 | Nucleus | transcription regulator |
| ARID5B | ENSG00000150347 | AT-rich interaction domain 5B | 1,753 | Nucleus | transcription regulator |
| ARIH1 | ENSG00000166233 | ariadne RBR E3 ubiquitin protein ligase 1 | 0,868 | Cytoplasm | enzyme |
| ARL14 | ENSG00000179674 | ADP ribosylation factor like GTPase 14 | 1,486 | Other | other |
| ARL14EPL | ENSG00000268223 | ADP ribosylation factor like GTPase 14 effector protein like | 2,428 | Other | other |
| ARL3 | ENSG00000138175 | ADP ribosylation factor like GTPase 3 | -1,115 | Cytoplasm | enzyme |
| ARL4A | ENSG00000122644 | ADP ribosylation factor like GTPase 4A | 0,801 | Nucleus | enzyme |
| ARL4C | ENSG00000188042 | ADP ribosylation factor like GTPase 4C | -0,732 | Nucleus | enzyme |
| ARL4D | ENSG00000175906 | ADP ribosylation factor like GTPase 4D | 0,938 | Nucleus | enzyme |
| ARL5B | ENSG00000165997 | ADP ribosylation factor like GTPase 5B | 1,686 | Other | enzyme |
| ARL6IP5 | ENSG00000144746 | ADP ribosylation factor like GTPase 6 interacting protein 5 | -1,004 | Cytoplasm | other |
| ARMC10 | ENSG00000170632 | armadillo repeat containing 10 | -0,673 | Cytoplasm | other |
| ARMC3 | ENSG00000165309 | armadillo repeat containing 3 | -0,859 | Cytoplasm | other |
| ARMC5 | ENSG00000140691 | armadillo repeat containing 5 | 0,734 | Cytoplasm | other |
| ARMC7 | ENSG00000125449 | armadillo repeat containing 7 | -0,605 | Cytoplasm | other |
| ARMC9 | ENSG00000135931 | armadillo repeat containing 9 | -0,834 | Cytoplasm | other |
| ARMCX1 | ENSG00000126947 | armadillo repeat containing X-linked 1 | 0,647 | Other | other |
| ARNTL2 | ENSG00000029153 | aryl hydrocarbon receptor nuclear translocator like 2 | 0,698 | Nucleus | transcription regulator |
| ARPC5 | ENSG00000162704 | actin related protein 2/3 complex subunit 5 | -0,606 | Cytoplasm | other |
| ARPIN/ARPIN-AP3S2 | ENSG00000242498 | ARPIN-AP3S2 readthrough | -0,625 | Plasma Membrane | other |
| ARRB2 | ENSG00000141480 | arrestin beta 2 | -0,640 | Cytoplasm | other |
| ARRDC3 | ENSG00000113369 | arrestin domain containing 3 | 3,837 | Plasma Membrane | other |
| ARRDC4 | ENSG00000140450 | arrestin domain containing 4 | 1,516 | Plasma Membrane | other |
| ARSD | ENSG00000006756 | arylsulfatase D | -1,013 | Cytoplasm | enzyme |
| ARSL | ENSG00000157399 | arylsulfatase L | -1,207 | Cytoplasm | enzyme |
| ART3 | ENSG00000156219 | ADP-ribosyltransferase 3 | 1,180 | Plasma Membrane | enzyme |
| ASAH1 | ENSG00000104763 | N-acylsphingosine amidohydrolase 1 | -0,987 | Cytoplasm | enzyme |
| ASAP1 | ENSG00000153317 | ArfGAP with SH3 domain, ankyrin repeat and PH domain 1 | 1,122 | Plasma Membrane | other |

| | | | | | |
|---|---|---|---|---|---|
| ASAP2 | ENSG00000151693 | ArfGAP with SH3 domain, ankyrin repeat and PH domain 2 | 0,962 | Nucleus | other |
| ASNS | ENSG00000070669 | asparagine synthetase (glutamine-hydrolyzing) | 1,377 | Cytoplasm | enzyme |
| ASPHD2 | ENSG00000128203 | aspartate beta-hydroxylase domain containing 2 | 1,206 | Other | enzyme |
| ASPM | ENSG00000066279 | assembly factor for spindle microtubules | -0,785 | Nucleus | other |
| ATAD2B | ENSG00000119778 | ATPase family AAA domain containing 2B | 0,742 | Nucleus | other |
| ATF3 | ENSG00000162772 | activating transcription factor 3 | 5,808 | Nucleus | transcription regulator |
| ATF4 | ENSG00000128272 | activating transcription factor 4 | 1,101 | Nucleus | transcription regulator |
| ATP13A4 | ENSG00000127249 | ATPase 13A4 | 0,855 | Cytoplasm | transporter |
| ATP1A1 | ENSG00000163399 | ATPase Na+/K+ transporting subunit alpha 1 | -0,761 | Plasma Membrane | transporter |
| ATP23 | ENSG00000166896 | ATP23 metallopeptidase and ATP synthase assembly factor homolog | -1,134 | Plasma Membrane | kinase |
| ATP4A | ENSG00000105675 | ATPase H+/K+ transporting subunit alpha | 2,522 | Plasma Membrane | transporter |
| ATP5IF1 | ENSG00000130770 | ATP synthase inhibitory factor subunit 1 | -0,782 | Cytoplasm | other |
| ATP5MC2 | ENSG00000135390 | ATP synthase membrane subunit c locus 2 | -0,613 | Cytoplasm | transporter |
| ATP5ME | ENSG00000169020 | ATP synthase membrane subunit e | -0,614 | Cytoplasm | transporter |
| ATP5MF | ENSG00000241468 | ATP synthase membrane subunit f | -0,619 | Cytoplasm | transporter |
| ATP5MG | ENSG00000167283 | ATP synthase membrane subunit g | -0,682 | Cytoplasm | enzyme |
| ATP5MPL | ENSG00000156411 | ATP synthase membrane subunit 6.8PL | -0,809 | Cytoplasm | other |
| ATP5PB | ENSG00000116459 | ATP synthase peripheral stalk-membrane subunit b | -0,631 | Cytoplasm | transporter |
| ATP5PD | ENSG00000167863 | ATP synthase peripheral stalk subunit d | -0,674 | Cytoplasm | enzyme |
| ATP5PF | ENSG00000154723 | ATP synthase peripheral stalk subunit F6 | -0,684 | Cytoplasm | transporter |
| ATP6V0E2 | ENSG00000171130 | ATPase H+ transporting V0 subunit e2 | -0,713 | Cytoplasm | enzyme |
| ATP9A | ENSG00000054793 | ATPase phospholipid transporting 9A (putative) | -1,265 | Cytoplasm | transporter |
| ATP9B | ENSG00000166377 | ATPase phospholipid transporting 9B (putative) | -0,919 | Cytoplasm | transporter |
| ATPSCKMT | ENSG00000150756 | ATP synthase c subunit lysine N-methyltransferase | -1,387 | Cytoplasm | enzyme |
| ATRAID | ENSG00000138085 | all-trans retinoic acid induced differentiation factor | -0,796 | Nucleus | other |
| ATRIP | ENSG00000164053 | ATR interacting protein | 0,646 | Nucleus | kinase |
| ATRN | ENSG00000088812 | attractin | -0,686 | Extracellular Space | other |
| ATXN2L | ENSG00000168488 | ataxin 2 like | 0,630 | Nucleus | other |
| ATXN7 | ENSG00000163635 | ataxin 7 | 0,798 | Nucleus | other |
| ATXN7L1 | ENSG00000146776 | ataxin 7 like 1 | 0,614 | Other | other |
| ATXN7L2 | ENSG00000162650 | ataxin 7 like 2 | 1,032 | Other | other |
| AURKA | ENSG00000087586 | aurora kinase A | -0,790 | Nucleus | kinase |
| AVPI1 | ENSG00000119986 | arginine vasopressin induced 1 | 1,265 | Other | other |
| AZI2 | ENSG00000163512 | 5-azacytidine induced 2 | 0,861 | Cytoplasm | other |
| B2M | ENSG00000166710 | beta-2-microglobulin | 1,489 | Plasma Membrane | transmembrane receptor |
| B3GALNT1 | ENSG00000169255 | beta-1,3-N-acetylgalactosaminyltransferase 1 (globoside blood group) | 0,708 | Cytoplasm | enzyme |
| B3GNT2 | ENSG00000170340 | UDP-GlcNAc:betaGal beta-1,3-N-acetylglucosaminyltransferase 2 | 0,926 | Cytoplasm | enzyme |
| B3GNT5 | ENSG00000176597 | UDP-GlcNAc:betaGal beta-1,3-N-acetylglucosaminyltransferase 5 | 1,177 | Cytoplasm | enzyme |
| B3GNT7 | ENSG00000156966 | UDP-GlcNAc:betaGal beta-1,3-N-acetylglucosaminyltransferase 7 | 1,517 | Cytoplasm | enzyme |
| B4GALNT2 | ENSG00000167080 | beta-1,4-N-acetyl-galactosaminyltransferase 2 | -0,820 | Cytoplasm | enzyme |
| B4GALT5 | ENSG00000158470 | beta-1,4-galactosyltransferase 5 | 1,218 | Cytoplasm | enzyme |
| BAAT | ENSG00000136881 | bile acid-CoA:amino acid N-acyltransferase | 0,746 | Cytoplasm | enzyme |
| BABAM2 | ENSG00000158019 | BRISC and BRCA1 A complex member 2 | -0,915 | Cytoplasm | other |
| BACH1 | ENSG00000156273 | BTB domain and CNC homolog 1 | 1,182 | Nucleus | transcription regulator |
| BACH2 | ENSG00000112182 | BTB domain and CNC homolog 2 | 2,388 | Nucleus | transcription regulator |
| BAD | ENSG00000002330 | BCL2 associated agonist of cell death | -0,602 | Cytoplasm | other |
| BAG1 | ENSG00000107262 | BAG cochaperone 1 | 0,968 | Cytoplasm | other |
| BAG3 | ENSG00000151929 | BAG cochaperone 3 | 0,690 | Cytoplasm | other |
| BAG6 | ENSG00000233348 | BAG cochaperone 6 | -0,833 | Nucleus | enzyme |
| BAK1 | ENSG00000030110 | BCL2 antagonist/killer 1 | 0,619 | Cytoplasm | other |
| BATF2 | ENSG00000168062 | basic leucine zipper ATF-like transcription factor 2 | 4,178 | Other | other |
| BAZ1A | ENSG00000198604 | bromodomain adjacent to zinc finger domain 1A | 1,069 | Nucleus | other |
| BAZ2A | ENSG00000076108 | bromodomain adjacent to zinc finger domain 2A | 0,652 | Nucleus | transcription regulator |
| BAZ2B | ENSG00000123636 | bromodomain adjacent to zinc finger domain 2B | 1,142 | Extracellular Space | other |
| BBC3 | ENSG00000105327 | BCL2 binding component 3 | 4,492 | Cytoplasm | other |
| BCAR3 | ENSG00000137936 | BCAR3 adaptor protein, NSP family member | 0,700 | Cytoplasm | other |
| BCAS1 | ENSG00000064787 | brain enriched myelin associated protein 1 | -1,524 | Plasma Membrane | other |
| BCAS4 | ENSG00000124243 | breast carcinoma amplified sequence 4 | -0,633 | Cytoplasm | other |
| BCL10 | ENSG00000142867 | BCL10 immune signaling adaptor | 1,070 | Cytoplasm | transcription regulator |
| BCL2L11 | ENSG00000153094 | BCL2 like 11 | 1,419 | Cytoplasm | other |
| BCL2L13 | ENSG00000099968 | BCL2 like 13 | 0,777 | Cytoplasm | other |
| BCL2L14 | ENSG00000121380 | BCL2 like 14 | 2,455 | Cytoplasm | other |
| BCL3 | ENSG00000069399 | BCL3 transcription coactivator | 1,749 | Nucleus | transcription regulator |
| BCL6 | ENSG00000113916 | BCL6 transcription repressor | 0,691 | Nucleus | transcription regulator |
| BCL7C | ENSG00000099385 | BAF chromatin remodeling complex subunit BCL7C | -0,672 | Nucleus | other |
| BCO1 | ENSG00000135697 | beta-carotene oxygenase 1 | 1,653 | Cytoplasm | enzyme |
| BDH2 | ENSG00000164039 | 3-hydroxybutyrate dehydrogenase 2 | -0,947 | Cytoplasm | enzyme |
| BDNF | ENSG00000176697 | brain derived neurotrophic factor | 1,520 | Extracellular Space | growth factor |
| BDP1 | ENSG00000145734 | B double prime 1, subunit of RNA polymerase III transcription initiation factor IIIB | 0,807 | Nucleus | transcription regulator |
| BEST3 | ENSG00000127325 | bestrophin 3 | 0,806 | Nucleus | ion channel |
| BICC1 | ENSG00000122870 | BicC family RNA binding protein 1 | -1,196 | Other | other |
| BIK | ENSG00000100290 | BCL2 interacting killer | 0,791 | Cytoplasm | other |
| BIRC2 | ENSG00000110330 | baculoviral IAP repeat containing 2 | 0,646 | Cytoplasm | enzyme |
| BIRC3 | ENSG00000023445 | baculoviral IAP repeat containing 3 | 2,275 | Cytoplasm | enzyme |
| BIRC5 | ENSG00000089685 | baculoviral IAP repeat containing 5 | -0,842 | Cytoplasm | other |
| BLOC1S1 | ENSG00000135441 | biogenesis of lysosomal organelles complex 1 subunit 1 | -0,788 | Cytoplasm | other |
| BLVRB | ENSG00000090013 | biliverdin reductase B | -0,672 | Cytoplasm | enzyme |
| BLZF1 | ENSG00000117475 | basic leucine zipper nuclear factor 1 | 1,420 | Cytoplasm | transcription regulator |
| BMERB1 | ENSG00000166780 | bMERB domain containing 1 | -0,670 | Other | other |
| BMP2 | ENSG00000125845 | bone morphogenetic protein 2 | 1,721 | Extracellular Space | growth factor |
| BMP8B | ENSG00000116985 | bone morphogenetic protein 8b | -0,806 | Extracellular Space | growth factor |
| BNIP3L | ENSG00000104765 | BCL2 interacting protein 3 like | -1,067 | Cytoplasm | other |
| BNIP5 | ENSG00000189325 | BCL2 interacting protein 5 | 1,031 | Other | other |
| BOC | ENSG00000144857 | BOC cell adhesion associated, oncogene regulated | 1,870 | Plasma Membrane | other |
| BOP1 | ENSG00000261236 | BOP1 ribosomal biogenesis factor | -0,710 | Nucleus | other |
| BPHL | ENSG00000137274 | biphenyl hydrolase like | -0,819 | Cytoplasm | enzyme |
| BPIFB1 | ENSG00000125999 | BPI fold containing family B member 1 | -0,811 | Extracellular Space | other |
| BRD2 | ENSG00000236227 | bromodomain containing 2 | 1,709 | Nucleus | kinase |
| BRF2 | ENSG00000104221 | BRF2 RNA polymerase III transcription initiation factor subunit | 1,006 | Nucleus | transcription regulator |
| BRI3BP | ENSG00000184992 | BRI3 binding protein | -1,125 | Extracellular Space | other |
| BRIP1 | ENSG00000136492 | BRCA1 interacting protein C-terminal helicase 1 | 0,605 | Nucleus | enzyme |
| BRPF1 | ENSG00000156983 | bromodomain and PHD finger containing 1 | 0,669 | Nucleus | transporter |
| BRSK1 | ENSG00000160469 | BR serine/threonine kinase 1 | 1,060 | Cytoplasm | kinase |
| BSDC1 | ENSG00000160058 | BSD domain containing 1 | 0,618 | Other | other |
| BST2 | ENSG00000130303 | bone marrow stromal cell antigen 2 | 5,331 | Plasma Membrane | other |
| BTC | ENSG00000174808 | betacellulin | 1,712 | Extracellular Space | growth factor |
| BTD | ENSG00000169814 | biotinidase | -0,965 | Extracellular Space | enzyme |
| BTG2 | ENSG00000159388 | BTG anti-proliferation factor 2 | 0,887 | Nucleus | transcription regulator |
| BTN2A1 | ENSG00000112763 | butyrophilin subfamily 2 member A1 | 0,784 | Plasma Membrane | other |

| | | | | | |
|---|---|---|---|---|---|
| BTN2A2 | ENSG00000124508 | butyrophilin subfamily 2 member A2 | 1,654 | Cytoplasm | other |
| BTN3A1 | ENSG00000026950 | butyrophilin subfamily 3 member A1 | 1,908 | Extracellular Space | other |
| BTN3A2 | ENSG00000186470 | butyrophilin subfamily 3 member A2 | 1,243 | Plasma Membrane | other |
| BTN3A3 | ENSG00000111801 | butyrophilin subfamily 3 member A3 | 2,137 | Plasma Membrane | other |
| BUB1B | ENSG00000156970 | BUB1 mitotic checkpoint serine/threonine kinase B | -0,841 | Nucleus | kinase |
| BUD31 | ENSG00000106245 | BUD31 homolog | 0,614 | Nucleus | transcription regulator |
| BX255923.2 | ENSG00000204790 | | -0,912 | Other | other |
| C11orf24 | ENSG00000171067 | chromosome 11 open reading frame 24 | 0,693 | Extracellular Space | other |
| C11orf45 | ENSG00000174370 | chromosome 11 open reading frame 45 | -0,763 | Extracellular Space | other |
| C11orf49 | ENSG00000149179 | chromosome 11 open reading frame 49 | -0,692 | Nucleus | other |
| C11orf54 | ENSG00000182919 | chromosome 11 open reading frame 54 | -0,807 | Nucleus | enzyme |
| C11orf80 | ENSG00000173715 | chromosome 11 open reading frame 80 | -1,000 | Other | other |
| C11orf96 | ENSG00000187479 | chromosome 11 open reading frame 96 | 1,389 | Other | other |
| C15orf39 | ENSG00000167173 | chromosome 15 open reading frame 39 | 0,823 | Cytoplasm | other |
| C15orf48 | ENSG00000166920 | chromosome 15 open reading frame 48 | 1,924 | Nucleus | other |
| C16orf70 | ENSG00000125149 | chromosome 16 open reading frame 70 | 0,673 | Cytoplasm | other |
| C16orf87 | ENSG00000155330 | chromosome 16 open reading frame 87 | 0,869 | Other | other |
| C17orf67 | ENSG00000214226 | chromosome 17 open reading frame 67 | 0,711 | Other | other |
| C17orf97 | ENSG00000187624 | chromosome 17 open reading frame 97 | -0,714 | Other | other |
| C18orf25 | ENSG00000152242 | chromosome 18 open reading frame 25 | 1,072 | Other | other |
| C1orf52 | ENSG00000162642 | chromosome 1 open reading frame 52 | 1,161 | Nucleus | other |
| C1QTNF1 | ENSG00000173918 | C1q and TNF related 1 | 1,545 | Extracellular Space | other |
| C1R | ENSG00000159403 | complement C1r | 2,110 | Extracellular Space | peptidase |
| C1S | ENSG00000182326 | complement C1s | 2,223 | Extracellular Space | peptidase |
| C2 | ENSG00000166278 | complement C2 | 0,901 | Extracellular Space | peptidase |
| C21orf58 | ENSG00000160298 | chromosome 21 open reading frame 58 | -0,627 | Other | other |
| C21orf91 | ENSG00000154642 | chromosome 21 open reading frame 91 | 1,150 | Other | other |
| C2CD4A | ENSG00000198535 | C2 calcium dependent domain containing 4A | 0,764 | Nucleus | other |
| C2orf15 | ENSG00000273045 | chromosome 2 open reading frame 15 | -0,829 | Other | other |
| C2orf81 | ENSG00000159239 | chromosome 2 open reading frame 81 | -1,206 | Other | other |
| C3 | ENSG00000125730 | complement C3 | 1,540 | Extracellular Space | peptidase |
| C3orf33 | ENSG00000174928 | chromosome 3 open reading frame 33 | 0,806 | Extracellular Space | other |
| C3orf38 | ENSG00000179021 | chromosome 3 open reading frame 38 | 0,765 | Nucleus | peptidase |
| C3orf52 | ENSG00000114529 | chromosome 3 open reading frame 52 | 0,899 | Other | other |
| C3orf80 | ENSG00000180044 | chromosome 3 open reading frame 80 | -0,917 | Other | other |
| C4A/C4B | ENSG00000244731 | complement C4B (Chido blood group) | 2,941 | Extracellular Space | other |
| C4BPB | ENSG00000123843 | complement component 4 binding protein beta | 1,227 | Extracellular Space | other |
| C4orf33 | ENSG00000151470 | chromosome 4 open reading frame 33 | 0,703 | Other | other |
| C5 | ENSG00000106804 | complement C5 | -1,281 | Extracellular Space | cytokine |
| C5orf63 | ENSG00000164241 | chromosome 5 open reading frame 63 | -0,912 | Cytoplasm | other |
| C6orf58 | ENSG00000184530 | chromosome 6 open reading frame 58 | 2,877 | Cytoplasm | other |
| C6orf62 | ENSG00000112308 | chromosome 6 open reading frame 62 | 0,829 | Other | other |
| C7orf31 | ENSG00000153790 | chromosome 7 open reading frame 31 | 0,712 | Cytoplasm | transporter |
| CA12 | ENSG00000074410 | carbonic anhydrase 12 | -1,422 | Plasma Membrane | enzyme |
| CA8 | ENSG00000178538 | carbonic anhydrase 8 | 0,935 | Cytoplasm | enzyme |
| CA9 | ENSG00000107159 | carbonic anhydrase 9 | -1,840 | Nucleus | enzyme |
| CACNA1D | ENSG00000157388 | calcium voltage-gated channel subunit alpha1 D | -1,161 | Plasma Membrane | ion channel |
| CACNA1I | ENSG00000100346 | calcium voltage-gated channel subunit alpha1 I | 0,818 | Plasma Membrane | ion channel |
| CACNB2 | ENSG00000165995 | calcium voltage-gated channel auxiliary subunit beta 2 | 0,755 | Plasma Membrane | ion channel |
| CACNB3 | ENSG00000167535 | calcium voltage-gated channel auxiliary subunit beta 3 | -0,670 | Plasma Membrane | ion channel |
| CACNG4 | ENSG00000075461 | calcium voltage-gated channel auxiliary subunit gamma 4 | -1,673 | Plasma Membrane | ion channel |
| CACTIN | ENSG00000105298 | cactin, spliceosome C complex subunit | 0,728 | Nucleus | other |
| CALCRL | ENSG00000064989 | calcitonin receptor like receptor | 0,751 | Plasma Membrane | G-protein coupled receptor |
| CALM2P2 | ENSG00000229097 | calmodulin 2 pseudogene 2 | 3,702 | Other | other |
| CAMK2N1 | ENSG00000162545 | calcium/calmodulin dependent protein kinase II inhibitor 1 | -0,691 | Plasma Membrane | kinase |
| CAPN13 | ENSG00000162949 | calpain 13 | -1,124 | Other | other |
| CAPN2 | ENSG00000162909 | calpain 2 | -0,607 | Cytoplasm | peptidase |
| CAPN5 | ENSG00000149260 | calpain 5 | -1,201 | Cytoplasm | peptidase |
| CAPN8 | ENSG00000203697 | calpain 8 | -0,705 | Cytoplasm | peptidase |
| CAPS | ENSG00000105519 | calcyphosine | -1,451 | Cytoplasm | other |
| CARD16 | ENSG00000204397 | caspase recruitment domain family member 16 | 2,745 | Other | other |
| CARD6 | ENSG00000132357 | caspase recruitment domain family member 6 | 0,745 | Cytoplasm | other |
| CARHSP1 | ENSG00000153048 | calcium regulated heat stable protein 1 | -0,807 | Cytoplasm | transcription regulator |
| CARS1 | ENSG00000110619 | cysteinyl-tRNA synthetase 1 | 0,753 | Cytoplasm | enzyme |
| CASP1 | ENSG00000137752 | caspase 1 | 3,328 | Cytoplasm | peptidase |
| CASP10 | ENSG00000003400 | caspase 10 | 0,857 | Cytoplasm | peptidase |
| CASP4 | ENSG00000196954 | caspase 4 | 0,866 | Cytoplasm | peptidase |
| CASP7 | ENSG00000165806 | caspase 7 | 1,067 | Cytoplasm | peptidase |
| CASP8 | ENSG00000064012 | caspase 8 | 0,744 | Nucleus | peptidase |
| CASR | ENSG00000036828 | calcium sensing receptor | -0,640 | Plasma Membrane | G-protein coupled receptor |
| CASTOR1 | ENSG00000239282 | cytosolic arginine sensor for mTORC1 subunit 1 | -1,072 | Cytoplasm | other |
| CAT | ENSG00000121691 | catalase | -1,360 | Cytoplasm | enzyme |
| CAV1 | ENSG00000105974 | caveolin 1 | -0,738 | Plasma Membrane | transmembrane receptor |
| CAV2 | ENSG00000105971 | caveolin 2 | -0,701 | Plasma Membrane | other |
| CAVIN1 | ENSG00000177469 | caveolae associated protein 1 | -0,676 | Nucleus | transcription regulator |
| CAVIN4 | ENSG00000170681 | caveolae associated protein 4 | 0,629 | Cytoplasm | other |
| CBR3 | ENSG00000159231 | carbonyl reductase 3 | 0,891 | Cytoplasm | enzyme |
| CBWD3/CBWD6 | ENSG00000215126 | COBW domain containing 3 | 1,197 | Other | other |
| CBX4 | ENSG00000141582 | chromobox 4 | 0,976 | Nucleus | transcription regulator |
| CBX6 | ENSG00000183741 | chromobox 6 | -0,806 | Nucleus | other |
| CBX7 | ENSG00000100307 | chromobox 7 | -1,197 | Nucleus | other |
| CC2D1B | ENSG00000154222 | coiled-coil and C2 domain containing 1B | 0,713 | Nucleus | transcription regulator |
| CCDC115 | ENSG00000136710 | coiled-coil domain containing 115 | -1,125 | Cytoplasm | other |
| CCDC117 | ENSG00000159873 | coiled-coil domain containing 117 | 0,859 | Other | other |
| CCDC134 | ENSG00000100147 | coiled-coil domain containing 134 | 0,857 | Other | other |
| CCDC142 | ENSG00000135637 | coiled-coil domain containing 142 | 0,881 | Other | other |
| CCDC157 | ENSG00000187860 | coiled-coil domain containing 157 | 0,606 | Other | other |
| CCDC167 | ENSG00000198937 | coiled-coil domain containing 167 | -1,150 | Other | other |
| CCDC17 | ENSG00000159588 | coiled-coil domain containing 17 | 0,884 | Other | other |
| CCDC174 | ENSG00000154781 | coiled-coil domain containing 174 | 1,076 | Nucleus | other |
| CCDC181 | ENSG00000117477 | coiled-coil domain containing 181 | 0,870 | Nucleus | other |
| CCDC183 | ENSG00000213213 | coiled-coil domain containing 183 | -0,694 | Other | other |
| CCDC191 | ENSG00000163617 | coiled-coil domain containing 191 | -0,760 | Other | other |
| CCDC34 | ENSG00000109881 | coiled-coil domain containing 34 | -0,961 | Other | other |
| CCDC68 | ENSG00000166510 | coiled-coil domain containing 68 | 0,734 | Cytoplasm | other |
| CCDC71L | ENSG00000253276 | coiled-coil domain containing 71 like | 1,078 | Other | other |
| CCDC74B | ENSG00000152076 | coiled-coil domain containing 74B | -1,286 | Other | other |
| CCDC85C | ENSG00000205476 | coiled-coil domain containing 85C | -0,742 | Plasma Membrane | other |
| CCDC9 | ENSG00000105321 | coiled-coil domain containing 9 | 0,756 | Extracellular Space | other |

| | | | | | |
|---|---|---|---|---|---|
| CCHCR1 | ENSG00000206355 | coiled-coil alpha-helical rod protein 1 | 1,409 | Cytoplasm | other |
| CCL2 | ENSG00000108691 | C-C motif chemokine ligand 2 | 0,986 | Extracellular Space | cytokine |
| CCL20 | ENSG00000115009 | C-C motif chemokine ligand 20 | 3,502 | Extracellular Space | cytokine |
| CCL22 | ENSG00000102962 | C-C motif chemokine ligand 22 | 1,510 | Extracellular Space | cytokine |
| CCL28 | ENSG00000151882 | C-C motif chemokine ligand 28 | 1,094 | Extracellular Space | cytokine |
| CCL5 | ENSG00000271503 | C-C motif chemokine ligand 5 | 7,588 | Extracellular Space | cytokine |
| CCNB1 | ENSG00000134057 | cyclin B1 | -0,839 | Cytoplasm | kinase |
| CCNB1IP1 | ENSG00000100814 | cyclin B1 interacting protein 1 | 0,632 | Nucleus | enzyme |
| CCNB2 | ENSG00000157456 | cyclin B2 | -1,142 | Cytoplasm | other |
| CCND3 | ENSG00000112576 | cyclin D3 | 0,787 | Nucleus | kinase |
| CCNDBP1 | ENSG00000166946 | cyclin D1 binding protein 1 | -0,780 | Nucleus | other |
| CCNL1 | ENSG00000163660 | cyclin L1 | 1,362 | Nucleus | other |
| CCR1 | ENSG00000163823 | C-C motif chemokine receptor 1 | 1,831 | Plasma Membrane | G-protein coupled receptor |
| CCR4 | ENSG00000183813 | C-C motif chemokine receptor 4 | 0,614 | Plasma Membrane | G-protein coupled receptor |
| CCT6P3 | ENSG00000234585 | chaperonin containing TCP1 subunit 6 pseudogene 3 | 0,759 | Other | other |
| CD109 | ENSG00000156535 | CD109 molecule | -0,961 | Plasma Membrane | other |
| CD24 | ENSG00000272398 | CD24 molecule | -0,808 | Plasma Membrane | other |
| CD274 | ENSG00000120217 | CD274 molecule | 4,653 | Plasma Membrane | enzyme |
| CD2AP | ENSG00000198087 | CD2 associated protein | 0,922 | Cytoplasm | other |
| CD38 | ENSG00000004468 | CD38 molecule | 2,833 | Plasma Membrane | enzyme |
| CD40 | ENSG00000101017 | CD40 molecule | 1,560 | Plasma Membrane | transmembrane receptor |
| CD46 | ENSG00000117335 | CD46 molecule | -0,800 | Plasma Membrane | transmembrane receptor |
| CD47 | ENSG00000196776 | CD47 molecule | 1,342 | Plasma Membrane | transmembrane receptor |
| CD55 | ENSG00000196352 | CD55 molecule (Cromer blood group) | 1,326 | Plasma Membrane | other |
| CD63 | ENSG00000135404 | CD63 molecule | -0,655 | Plasma Membrane | other |
| CD68 | ENSG00000129226 | CD68 molecule | 2,841 | Plasma Membrane | other |
| CD69 | ENSG00000110848 | CD69 molecule | 1,886 | Plasma Membrane | transmembrane receptor |
| CD74 | ENSG00000019582 | CD74 molecule | 2,101 | Plasma Membrane | transmembrane receptor |
| CD83 | ENSG00000112149 | CD83 molecule | 1,166 | Plasma Membrane | transmembrane receptor |
| CD99L2 | ENSG00000102181 | CD99 molecule like 2 | -0,750 | Plasma Membrane | other |
| CDA | ENSG00000158825 | cytidine deaminase | -0,995 | Nucleus | enzyme |
| CDC14A | ENSG00000079335 | cell division cycle 14A | 1,317 | Nucleus | phosphatase |
| CDC14B | ENSG00000081377 | cell division cycle 14B | -0,783 | Nucleus | phosphatase |
| CDC20 | ENSG00000117399 | cell division cycle 20 | -0,828 | Nucleus | other |
| CDC25C | ENSG00000158402 | cell division cycle 25C | -0,621 | Nucleus | phosphatase |
| CDC42EP3 | ENSG00000163171 | CDC42 effector protein 3 | 0,931 | Cytoplasm | other |
| CDCA3 | ENSG00000111665 | cell division cycle associated 3 | -1,414 | Cytoplasm | other |
| CDCA7 | ENSG00000144354 | cell division cycle associated 7 | -0,788 | Nucleus | other |
| CDCP1 | ENSG00000163814 | CUB domain containing protein 1 | 0,692 | Plasma Membrane | other |
| CDH2 | ENSG00000170558 | cadherin 2 | -0,984 | Plasma Membrane | other |
| CDH24 | ENSG00000139880 | cadherin 24 | -0,896 | Plasma Membrane | other |
| CDK11A | ENSG00000008128 | cyclin dependent kinase 11A | 0,714 | Nucleus | kinase |
| CDK12 | ENSG00000167258 | cyclin dependent kinase 12 | 0,701 | Nucleus | kinase |
| CDK17 | ENSG00000059758 | cyclin dependent kinase 17 | 1,239 | Cytoplasm | kinase |
| CDK18 | ENSG00000117266 | cyclin dependent kinase 18 | 1,035 | Cytoplasm | kinase |
| CDK19 | ENSG00000155111 | cyclin dependent kinase 19 | -0,923 | Nucleus | kinase |
| CDKAL1 | ENSG00000145996 | CDK5 regulatory subunit associated protein 1 like 1 | 0,614 | Cytoplasm | enzyme |
| CDKN1A | ENSG00000124762 | cyclin dependent kinase inhibitor 1A | 1,479 | Nucleus | kinase |
| CDKN2AIP | ENSG00000168564 | CDKN2A interacting protein | 0,823 | Nucleus | transcription regulator |
| CDKN2B | ENSG00000147883 | cyclin dependent kinase inhibitor 2B | 1,197 | Nucleus | transcription regulator |
| CDKN3 | ENSG00000100526 | cyclin dependent kinase inhibitor 3 | -1,238 | Cytoplasm | phosphatase |
| CDPF1 | ENSG00000205643 | cysteine rich DPF motif domain containing 1 | -0,807 | Extracellular Space | other |
| CDRT1 | ENSG00000241322 | CMT1A duplicated region transcript 1 | 0,907 | Other | other |
| CEACAM1 | ENSG00000079385 | CEA cell adhesion molecule 1 | 2,937 | Plasma Membrane | transporter |
| CEBPB | ENSG00000172216 | CCAAT enhancer binding protein beta | 1,415 | Nucleus | transcription regulator |
| CEBPD | ENSG00000221869 | CCAAT enhancer binding protein delta | 1,080 | Nucleus | transcription regulator |
| CEBPZ | ENSG00000115816 | CCAAT enhancer binding protein zeta | 0,734 | Nucleus | transcription regulator |
| CEBPZOS | ENSG00000218739 | CEBPZ opposite strand | -0,696 | Other | other |
| CELSR1 | ENSG00000075275 | cadherin EGF LAG seven-pass G-type receptor 1 | -0,850 | Plasma Membrane | G-protein coupled receptor |
| CELSR3 | ENSG00000008300 | cadherin EGF LAG seven-pass G-type receptor 3 | 0,739 | Plasma Membrane | G-protein coupled receptor |
| CENPA | ENSG00000115163 | centromere protein A | -0,779 | Nucleus | other |
| CENPE | ENSG00000138778 | centromere protein E | -0,632 | Nucleus | other |
| CENPF | ENSG00000117724 | centromere protein F | -1,294 | Nucleus | other |
| CENPI | ENSG00000102384 | centromere protein I | -0,900 | Nucleus | other |
| CENPM | ENSG00000100162 | centromere protein M | -0,606 | Cytoplasm | other |
| CENPX | ENSG00000169689 | centromere protein X | -0,629 | Nucleus | other |
| CEP164 | ENSG00000110274 | centrosomal protein 164 | 0,887 | Cytoplasm | other |
| CEP20 | ENSG00000133393 | centrosomal protein 20 | -0,631 | Cytoplasm | other |
| CEP350 | ENSG00000135837 | centrosomal protein 350 | 1,193 | Cytoplasm | other |
| CEP55 | ENSG00000138180 | centrosomal protein 55 | -0,717 | Cytoplasm | other |
| CEP70 | ENSG00000114107 | centrosomal protein 70 | -0,654 | Cytoplasm | other |
| CERCAM | ENSG00000167123 | cerebral endothelial cell adhesion molecule | -0,857 | Other | other |
| CERK | ENSG00000100422 | ceramide kinase | -0,979 | Plasma Membrane | kinase |
| CETN2 | ENSG00000147400 | centrin 2 | -0,954 | Nucleus | enzyme |
| CFAP126 | ENSG00000188931 | cilia and flagella associated protein 126 | -1,605 | Other | other |
| CFAP36 | ENSG00000163001 | cilia and flagella associated protein 36 | -0,777 | Extracellular Space | other |
| CFAP54 | ENSG00000188596 | cilia and flagella associated protein 54 | 1,438 | Cytoplasm | other |
| CFAP57 | ENSG00000243710 | cilia and flagella associated protein 57 | 2,237 | Extracellular Space | other |
| CFB | ENSG00000241253 | complement factor B | 3,097 | Extracellular Space | peptidase |
| CFH | ENSG00000000971 | complement factor H | 2,118 | Extracellular Space | other |
| CFI | ENSG00000205403 | complement factor I | -1,319 | Extracellular Space | peptidase |
| CFLAR | ENSG00000003402 | CASP8 and FADD like apoptosis regulator | 1,185 | Cytoplasm | other |
| CFTR | ENSG00000001626 | CF transmembrane conductance regulator | -1,374 | Plasma Membrane | ion channel |
| CGAS | ENSG00000164430 | cyclic GMP-AMP synthase | 0,956 | Cytoplasm | enzyme |
| CH25H | ENSG00000138135 | cholesterol 25-hydroxylase | 6,580 | Cytoplasm | enzyme |
| CHAC1 | ENSG00000128965 | ChaC glutathione specific gamma-glutamylcyclotransferase 1 | 2,539 | Cytoplasm | enzyme |
| CHD1 | ENSG00000153922 | chromodomain helicase DNA binding protein 1 | 0,880 | Nucleus | enzyme |
| CHD2 | ENSG00000173575 | chromodomain helicase DNA binding protein 2 | 1,516 | Nucleus | enzyme |
| CHD7 | ENSG00000171316 | chromodomain helicase DNA binding protein 7 | 0,825 | Nucleus | enzyme |
| CHD8 | ENSG00000100888 | chromodomain helicase DNA binding protein 8 | 0,957 | Nucleus | enzyme |
| CHD9 | ENSG00000177200 | chromodomain helicase DNA binding protein 9 | 0,835 | Cytoplasm | other |
| CHEK2 | ENSG00000183765 | checkpoint kinase 2 | 1,050 | Nucleus | kinase |
| CHKA | ENSG00000110721 | choline kinase alpha | -0,650 | Cytoplasm | kinase |
| CHKB | ENSG00000100288 | choline kinase beta | 0,806 | Cytoplasm | kinase |
| CHN2 | ENSG00000106069 | chimerin 2 | 0,630 | Cytoplasm | other |
| CHP1 | ENSG00000187446 | calcineurin like EF-hand protein 1 | -0,653 | Cytoplasm | other |
| CHRDL1 | ENSG00000101938 | chordin like 1 | 0,967 | Extracellular Space | other |
| CHRNA7 | ENSG00000175344 | cholinergic receptor nicotinic alpha 7 subunit | 1,237 | Plasma Membrane | transmembrane receptor |
| CHST10 | ENSG00000115526 | carbohydrate sulfotransferase 10 | 0,616 | Cytoplasm | enzyme |

| | | | | | |
|---|---|---|---|---|---|
| CHST4 | ENSG00000140835 | carbohydrate sulfotransferase 4 | 0,602 | Cytoplasm | enzyme |
| CHST9 | ENSG00000154080 | carbohydrate sulfotransferase 9 | -0,954 | Cytoplasm | enzyme |
| CHSY1 | ENSG00000131873 | chondroitin sulfate synthase 1 | 0,813 | Cytoplasm | enzyme |
| CHSY3 | ENSG00000198108 | chondroitin sulfate synthase 3 | 1,584 | Cytoplasm | enzyme |
| CIB1 | ENSG00000185043 | calcium and integrin binding 1 | -0,673 | Nucleus | other |
| CIITA | ENSG00000179583 | class II major histocompatibility complex transactivator | 2,056 | Nucleus | transcription regulator |
| CITED2 | ENSG00000164442 | Cbp/p300 interacting transactivator with Glu/Asp rich carboxy-terminal domain 2 | 3,262 | Nucleus | transcription regulator |
| CKAP4 | ENSG00000136026 | cytoskeleton associated protein 4 | 0,625 | Cytoplasm | other |
| CKLF | ENSG00000217555 | chemokine like factor | -0,748 | Extracellular Space | cytokine |
| CLCN2 | ENSG00000114859 | chloride voltage-gated channel 2 | 0,795 | Plasma Membrane | ion channel |
| CLDN10 | ENSG00000134873 | claudin 10 | -2,134 | Plasma Membrane | other |
| CLDN16 | ENSG00000113946 | claudin 16 | -0,623 | Plasma Membrane | transporter |
| CLDN23 | ENSG00000253958 | claudin 23 | 0,611 | Plasma Membrane | other |
| CLDND1 | ENSG00000080822 | claudin domain containing 1 | 0,709 | Plasma Membrane | other |
| CLEC18A/CLEC18C | ENSG00000157322 | C-type lectin domain family 18 member A | 0,642 | Other | other |
| CLEC2D | ENSG00000069493 | C-type lectin domain family 2 member D | 0,622 | Plasma Membrane | transmembrane receptor |
| CLEC7A | ENSG00000172243 | C-type lectin domain containing 7A | 1,575 | Plasma Membrane | transmembrane receptor |
| CLGN | ENSG00000153132 | calmegin | -0,890 | Cytoplasm | peptidase |
| CLK1 | ENSG00000013441 | CDC like kinase 1 | 1,162 | Nucleus | kinase |
| CLK4 | ENSG00000113240 | CDC like kinase 4 | 1,054 | Nucleus | kinase |
| CLTRN | ENSG00000147003 | collectrin, amino acid transport regulator | 1,991 | Plasma Membrane | other |
| CMPK2 | ENSG00000134326 | cytidine/uridine monophosphate kinase 2 | 4,761 | Cytoplasm | kinase |
| CMTM4 | ENSG00000183723 | CKLF like MARVEL transmembrane domain containing 4 | -1,215 | Extracellular Space | cytokine |
| CMTM6 | ENSG00000091317 | CKLF like MARVEL transmembrane domain containing 6 | -0,679 | Extracellular Space | cytokine |
| CMTR1 | ENSG00000137200 | cap methyltransferase 1 | 1,244 | Nucleus | enzyme |
| CNKSR3 | ENSG00000153721 | CNKSR family member 3 | 0,896 | Plasma Membrane | kinase |
| CNOT4 | ENSG00000080802 | CCR4-NOT transcription complex subunit 4 | 0,883 | Cytoplasm | enzyme |
| CNOT6LP1 | ENSG00000230183 | CNOT6L pseudogene 1 | 2,196 | Other | other |
| CNP | ENSG00000173786 | 2',3'-cyclic nucleotide 3' phosphodiesterase | 1,453 | Cytoplasm | enzyme |
| CNPY4 | ENSG00000166997 | canopy FGF signaling regulator 4 | -0,697 | Other | other |
| CNTNAP3 | ENSG00000106714 | contactin associated protein family member 3 | -0,934 | Extracellular Space | other |
| CNTNAP3B | ENSG00000154529 | contactin associated protein family member 3B | -1,029 | Other | other |
| COA3 | ENSG00000183978 | cytochrome c oxidase assembly factor 3 | -0,992 | Cytoplasm | other |
| COL11A2 | ENSG00000230930 | collagen type XI alpha 2 chain | 1,558 | Extracellular Space | other |
| COL16A1 | ENSG00000084636 | collagen type XVI alpha 1 chain | 2,125 | Extracellular Space | other |
| COL22A1 | ENSG00000169436 | collagen type XXII alpha 1 chain | 0,996 | Extracellular Space | other |
| COL4A1 | ENSG00000187498 | collagen type IV alpha 1 chain | 1,079 | Extracellular Space | other |
| COL7A1 | ENSG00000114270 | collagen type VII alpha 1 chain | 0,806 | Extracellular Space | other |
| COL8A1 | ENSG00000144810 | collagen type VIII alpha 1 chain | 0,805 | Extracellular Space | other |
| COLCA2 | ENSG00000214290 | colorectal cancer associated 2 | -1,316 | Cytoplasm | other |
| COMMD3-BMI1 | ENSG00000269897 | COMMD3-BMI1 readthrough | -1,139 | Nucleus | transcription regulator |
| COMMD7 | ENSG00000149600 | COMM domain containing 7 | -0,774 | Cytoplasm | other |
| COMMD9 | ENSG00000110442 | COMM domain containing 9 | -0,716 | Cytoplasm | other |
| COPA | ENSG00000122218 | COPI coat complex subunit alpha | -0,664 | Cytoplasm | transporter |
| COPS6 | ENSG00000168090 | COP9 signalosome subunit 6 | -0,699 | Nucleus | other |
| COQ10B | ENSG00000115520 | coenzyme Q10B | 0,738 | Cytoplasm | other |
| COX11 | ENSG00000166260 | cytochrome c oxidase copper chaperone COX11 | -0,935 | Cytoplasm | enzyme |
| COX19 | ENSG00000240230 | cytochrome c oxidase assembly factor COX19 | 0,613 | Cytoplasm | other |
| COX20 | ENSG00000203667 | cytochrome c oxidase assembly factor COX20 | -0,927 | Cytoplasm | other |
| CPEB2 | ENSG00000137449 | cytoplasmic polyadenylation element binding protein 2 | 1,667 | Cytoplasm | translation regulator |
| CPEB3 | ENSG00000107864 | cytoplasmic polyadenylation element binding protein 3 | 2,616 | Cytoplasm | translation regulator |
| CPEB4 | ENSG00000113742 | cytoplasmic polyadenylation element binding protein 4 | 1,359 | Plasma Membrane | other |
| CPNE2 | ENSG00000140848 | copine 2 | 1,166 | Cytoplasm | other |
| CPSF1P1 | ENSG00000214076 | cleavage and polyadenylation specific factor 1 pseudogene 1 | 1,665 | Other | other |
| CPXM1 | ENSG00000088882 | carboxypeptidase X, M14 family member 1 | 2,884 | Extracellular Space | peptidase |
| CRAMP1 | ENSG00000007545 | cramped chromatin regulator homolog 1 | 0,615 | Other | other |
| CRAT | ENSG00000095321 | carnitine O-acetyltransferase | -0,726 | Cytoplasm | enzyme |
| CREB1 | ENSG00000118260 | cAMP responsive element binding protein 1 | 0,700 | Nucleus | transcription regulator |
| CREB5 | ENSG00000146592 | cAMP responsive element binding protein 5 | 3,690 | Nucleus | transcription regulator |
| CREBBP | ENSG00000005339 | CREB binding protein | 0,661 | Nucleus | transcription regulator |
| CREBL2 | ENSG00000111269 | cAMP responsive element binding protein like 2 | -0,691 | Nucleus | transcription regulator |
| CREBRF | ENSG00000164463 | CREB3 regulatory factor | 0,947 | Nucleus | transcription regulator |
| CRIP2 | ENSG00000182809 | cysteine rich protein 2 | -1,404 | Nucleus | other |
| CRP | ENSG00000132693 | C-reactive protein | -0,802 | Extracellular Space | other |
| CRPPA | ENSG00000214960 | CDP-L-ribitol pyrophosphorylase A | -1,412 | Cytoplasm | enzyme |
| CRYL1 | ENSG00000165475 | crystallin lambda 1 | -1,037 | Cytoplasm | enzyme |
| CRYM | ENSG00000103316 | crystallin mu | -0,943 | Cytoplasm | enzyme |
| CRYZ | ENSG00000116791 | crystallin zeta | -1,295 | Cytoplasm | enzyme |
| CSAG2/CSAG3 | ENSG00000268916 | CSAG family member 3 | 2,743 | Other | other |
| CSF1 | ENSG00000184371 | colony stimulating factor 1 | 2,762 | Extracellular Space | cytokine |
| CSF1R | ENSG00000182578 | colony stimulating factor 1 receptor | 0,849 | Plasma Membrane | kinase |
| CSF2RA | ENSG00000198223 | colony stimulating factor 2 receptor subunit alpha | 0,981 | Plasma Membrane | transmembrane receptor |
| CSF3 | ENSG00000108342 | colony stimulating factor 3 | 3,453 | Extracellular Space | cytokine |
| CSN3 | ENSG00000171209 | casein kappa | -0,826 | Extracellular Space | other |
| CSPG5 | ENSG00000114646 | chondroitin sulfate proteoglycan 5 | -0,736 | Extracellular Space | growth factor |
| CSRNP1 | ENSG00000144655 | cysteine and serine rich nuclear protein 1 | 2,773 | Nucleus | transcription regulator |
| CSRNP2 | ENSG00000110925 | cysteine and serine rich nuclear protein 2 | 0,726 | Nucleus | transcription regulator |
| CTAGE9 (includes others) | ENSG00000225932 | CTAGE family member 9 | 1,512 | Other | other |
| CTC_479C512 | ENSG00000261884 | | 1,592 | Other | other |
| CTD-3088G3.8 | ENSG00000188897 | | 1,180 | Other | other |
| CTDSPL | ENSG00000144677 | CTD small phosphatase like | -1,094 | Nucleus | other |
| CTH | ENSG00000116761 | cystathionine gamma-lyase | 1,678 | Cytoplasm | enzyme |
| CTNND2 | ENSG00000169862 | catenin delta 2 | -1,158 | Plasma Membrane | other |
| CTSD | ENSG00000117984 | cathepsin D | -0,829 | Cytoplasm | peptidase |
| CTSH | ENSG00000103811 | cathepsin H | -1,412 | Cytoplasm | peptidase |
| CTSS | ENSG00000163131 | cathepsin S | 2,026 | Cytoplasm | peptidase |
| CTSV | ENSG00000136943 | cathepsin V | -0,899 | Cytoplasm | peptidase |
| CTXN1 | ENSG00000178531 | cortexin 1 | -0,949 | Other | other |
| CUEDC2 | ENSG00000107874 | CUE domain containing 2 | -0,686 | Extracellular Space | other |
| CUL7 | ENSG00000044090 | cullin 7 | -0,755 | Cytoplasm | enzyme |
| CUTA | ENSG00000112514 | cutA divalent cation tolerance homolog | -0,727 | Cytoplasm | other |
| CUTC | ENSG00000119929 | cutC copper transporter | -0,971 | Cytoplasm | other |
| CUX1 | ENSG00000257923 | cut like homeobox 1 | 0,680 | Nucleus | transcription regulator |
| CX3CL1 | ENSG00000006210 | C-X3-C motif chemokine ligand 1 | 4,395 | Extracellular Space | cytokine |
| CXCL1 | ENSG00000163739 | C-X-C motif chemokine ligand 1 | 1,177 | Extracellular Space | cytokine |
| CXCL10 | ENSG00000169245 | C-X-C motif chemokine ligand 10 | 8,372 | Extracellular Space | cytokine |
| CXCL11 | ENSG00000169248 | C-X-C motif chemokine ligand 11 | 7,554 | Extracellular Space | cytokine |
| CXCL16 | ENSG00000161921 | C-X-C motif chemokine ligand 16 | 0,728 | Extracellular Space | cytokine |
| CXCL2 | ENSG00000081041 | C-X-C motif chemokine ligand 2 | 3,010 | Extracellular Space | cytokine |

| | | | | | |
|---|---|---|---|---|---|
| CXCL3 | ENSG00000163734 | C-X-C motif chemokine ligand 3 | 1,744 | Extracellular Space | cytokine |
| CXCL8 | ENSG00000169429 | C-X-C motif chemokine ligand 8 | 2,355 | Extracellular Space | cytokine |
| CXCL9 | ENSG00000138755 | C-X-C motif chemokine ligand 9 | 1,994 | Extracellular Space | cytokine |
| CXorf38 | ENSG00000185753 | chromosome X open reading frame 38 | 0,856 | Other | other |
| CXXC4 | ENSG00000168772 | CXXC finger protein 4 | 1,511 | Cytoplasm | other |
| CXXC5 | ENSG00000171604 | CXXC finger protein 5 | -0,668 | Nucleus | other |
| CYBRD1 | ENSG00000071967 | cytochrome b reductase 1 | -0,864 | Cytoplasm | enzyme |
| CYFIP2 | ENSG00000055163 | cytoplasmic FMR1 interacting protein 2 | -0,780 | Cytoplasm | other |
| CYLD | ENSG00000083799 | CYLD lysine 63 deubiquitinase | 2,434 | Nucleus | transcription regulator |
| CYP1A1 | ENSG00000140465 | cytochrome P450 family 1 subfamily A member 1 | 3,714 | Cytoplasm | enzyme |
| CYP1B1 | ENSG00000138061 | cytochrome P450 family 1 subfamily B member 1 | 1,020 | Cytoplasm | enzyme |
| CYP21A1P | ENSG00000239882 | cytochrome P450 family 21 subfamily A member 1, pseudogene | 0,975 | Other | other |
| CYP24A1 | ENSG00000019186 | cytochrome P450 family 24 subfamily A member 1 | -1,276 | Cytoplasm | enzyme |
| CYP27A1 | ENSG00000135929 | cytochrome P450 family 27 subfamily A member 1 | -1,645 | Cytoplasm | enzyme |
| CYP27B1 | ENSG00000111012 | cytochrome P450 family 27 subfamily B member 1 | 0,688 | Cytoplasm | enzyme |
| CYP2B6 | ENSG00000197408 | cytochrome P450 family 2 subfamily B member 6 | 1,399 | Cytoplasm | enzyme |
| CYP2B7P | ENSG00000256612 | cytochrome P450 family 2 subfamily B member 7, pseudogene | 0,940 | Other | other |
| CYP2J2 | ENSG00000134716 | cytochrome P450 family 2 subfamily J member 2 | 1,896 | Cytoplasm | enzyme |
| CYP2U1 | ENSG00000155016 | cytochrome P450 family 2 subfamily U member 1 | -0,696 | Cytoplasm | enzyme |
| CYP2W1 | ENSG00000073067 | cytochrome P450 family 2 subfamily W member 1 | 0,862 | Cytoplasm | enzyme |
| CYP39A1 | ENSG00000146233 | cytochrome P450 family 39 subfamily A member 1 | -0,665 | Cytoplasm | enzyme |
| CYP51A1 | ENSG00000001630 | cytochrome P450 family 51 subfamily A member 1 | -1,104 | Cytoplasm | enzyme |
| CYS1 | ENSG00000205795 | cystin 1 | -2,134 | Cytoplasm | transcription regulator |
| CYSLTR1 | ENSG00000173198 | cysteinyl leukotriene receptor 1 | 0,635 | Plasma Membrane | G-protein coupled receptor |
| CYTH1 | ENSG00000108669 | cytohesin 1 | 0,754 | Cytoplasm | other |
| CYTH4 | ENSG00000100055 | cytohesin 4 | 0,776 | Cytoplasm | other |
| D2HGDH | ENSG00000180902 | D-2-hydroxyglutarate dehydrogenase | -0,717 | Cytoplasm | enzyme |
| DAB2IP | ENSG00000136848 | DAB2 interacting protein | 1,140 | Plasma Membrane | other |
| DAG1 | ENSG00000173402 | dystroglycan 1 | -0,649 | Plasma Membrane | transmembrane receptor |
| DAPP1 | ENSG00000070190 | dual adaptor of phosphotyrosine and 3-phosphoinositides 1 | 1,736 | Cytoplasm | other |
| DARS2 | ENSG00000117593 | aspartyl-tRNA synthetase 2, mitochondrial | -0,962 | Cytoplasm | enzyme |
| DAXX | ENSG00000204209 | death domain associated protein | 1,202 | Nucleus | transcription regulator |
| DAZAP2 | ENSG00000183283 | DAZ associated protein 2 | -0,760 | Nucleus | other |
| DBI | ENSG00000155368 | diazepam binding inhibitor, acyl-CoA binding protein | -0,866 | Cytoplasm | other |
| DCAF6 | ENSG00000143164 | DDB1 and CUL4 associated factor 6 | -0,760 | Nucleus | transcription regulator |
| DCAKD | ENSG00000172992 | dephospho-CoA kinase domain containing | -0,666 | Cytoplasm | other |
| DCLRE1C | ENSG00000152457 | DNA cross-link repair 1C | 1,237 | Nucleus | enzyme |
| DCP1A | ENSG00000272886 | decapping mRNA 1A | 1,292 | Nucleus | other |
| DCP1B | ENSG00000151065 | decapping mRNA 1B | 0,642 | Cytoplasm | other |
| DCTN3 | ENSG00000137100 | dynactin subunit 3 | -0,645 | Nucleus | other |
| DCUN1D3 | ENSG00000188215 | defective in cullin neddylation 1 domain containing 3 | 0,850 | Cytoplasm | other |
| DDB1 | ENSG00000167986 | damage specific DNA binding protein 1 | -0,632 | Nucleus | other |
| DDC | ENSG00000132437 | dopa decarboxylase | -1,044 | Cytoplasm | enzyme |
| DDIT3 | ENSG00000175197 | DNA damage inducible transcript 3 | 1,647 | Nucleus | transcription regulator |
| DDO | ENSG00000203797 | D-aspartate oxidase | 1,564 | Cytoplasm | enzyme |
| DDX55 | ENSG00000111364 | DEAD-box helicase 55 | 0,635 | Nucleus | enzyme |
| DDX58 | ENSG00000107201 | DExD/H-box helicase 58 | 3,104 | Cytoplasm | enzyme |
| DDX60 | ENSG00000137628 | DExD/H-box helicase 60 | 2,502 | Cytoplasm | enzyme |
| DDX60L | ENSG00000181381 | DExD/H-box 60 like | 2,905 | Other | other |
| DENND10 | ENSG00000119979 | DENN domain containing 10 | -0,611 | Other | other |
| DENND4B | ENSG00000198837 | DENN domain containing 4B | 0,702 | Extracellular Space | other |
| DENND5A | ENSG00000184014 | DENN domain containing 5A | 0,651 | Cytoplasm | other |
| DENND6B | ENSG00000205593 | DENN domain containing 6B | -0,763 | Cytoplasm | other |
| DEPDC1 | ENSG00000024526 | DEP domain containing 1 | -0,924 | Nucleus | transcription regulator |
| DEPDC1B | ENSG00000035499 | DEP domain containing 1B | -0,710 | Cytoplasm | other |
| DEPDC7 | ENSG00000121690 | DEP domain containing 7 | 1,043 | Cytoplasm | other |
| DEPP1 | ENSG00000165507 | DEPP1 autophagy regulator | 1,144 | Cytoplasm | other |
| DERA | ENSG00000023697 | deoxyribose-phosphate aldolase | -0,646 | Cytoplasm | enzyme |
| DESI1 | ENSG00000100418 | desumoylating isopeptidase 1 | 0,604 | Cytoplasm | other |
| DHCR24 | ENSG00000116133 | 24-dehydrocholesterol reductase | -1,138 | Cytoplasm | enzyme |
| DHCR7 | ENSG00000172893 | 7-dehydrocholesterol reductase | -1,489 | Cytoplasm | enzyme |
| DHFR | ENSG00000228716 | dihydrofolate reductase | -1,181 | Nucleus | enzyme |
| DHRS2 | ENSG00000100867 | dehydrogenase/reductase 2 | 1,732 | Nucleus | enzyme |
| DHRS4L2 | ENSG00000187630 | dehydrogenase/reductase 4 like 2 | -0,807 | Other | other |
| DHRS7B | ENSG00000109016 | dehydrogenase/reductase 7B | 0,603 | Other | other |
| DHX36 | ENSG00000174953 | DEAH-box helicase 36 | 0,742 | Cytoplasm | enzyme |
| DHX58 | ENSG00000108771 | DExH-box helicase 58 | 4,555 | Cytoplasm | enzyme |
| DICER1 | ENSG00000100697 | dicer 1, ribonuclease III | 1,417 | Cytoplasm | enzyme |
| DIO2 | ENSG00000211448 | iodothyronine deiodinase 2 | 1,318 | Cytoplasm | enzyme |
| DIP2A | ENSG00000160305 | disco interacting protein 2 homolog A | -0,665 | Nucleus | transcription regulator |
| DIP2B | ENSG00000066084 | disco interacting protein 2 homolog B | 0,900 | Cytoplasm | other |
| DIRAS2 | ENSG00000165023 | DIRAS family GTPase 2 | -0,744 | Plasma Membrane | enzyme |
| DIRAS3 | ENSG00000162595 | DIRAS family GTPase 3 | 1,935 | Plasma Membrane | enzyme |
| DLC1 | ENSG00000164741 | DLC1 Rho GTPase activating protein | 0,647 | Cytoplasm | other |
| DLEU1 | ENSG00000176124 | deleted in lymphocytic leukemia 1 | -1,092 | Other | other |
| DLGAP5 | ENSG00000126787 | DLG associated protein 5 | -0,973 | Nucleus | other |
| DLX2 | ENSG00000115844 | distal-less homeobox 2 | 1,010 | Nucleus | transcription regulator |
| DMBX1 | ENSG00000197587 | diencephalon/mesencephalon homeobox 1 | -2,052 | Nucleus | transcription regulator |
| DNAAF3 | ENSG00000167646 | dynein axonemal assembly factor 3 | -1,257 | Other | other |
| DNAH17 | ENSG00000187775 | dynein axonemal heavy chain 17 | 1,179 | Cytoplasm | other |
| DNAH5 | ENSG00000039139 | dynein axonemal heavy chain 5 | -0,724 | Cytoplasm | enzyme |
| DNAJA4 | ENSG00000140403 | DnaJ heat shock protein family (Hsp40) member A4 | 0,811 | Nucleus | other |
| DNAJB4 | ENSG00000162616 | DnaJ heat shock protein family (Hsp40) member B4 | 2,931 | Nucleus | other |
| DNAJB5 | ENSG00000137094 | DnaJ heat shock protein family (Hsp40) member B5 | 1,077 | Cytoplasm | other |
| DNAJB9 | ENSG00000128590 | DnaJ heat shock protein family (Hsp40) member B9 | 0,883 | Nucleus | other |
| DNAJC22 | ENSG00000178401 | DnaJ heat shock protein family (Hsp40) member C22 | -1,566 | Other | other |
| DNAJC25 | ENSG00000059769 | DnaJ heat shock protein family (Hsp40) member C25 | 1,618 | Other | other |
| DNAJC25-GNG10 | ENSG00000244115 | DNAJC25-GNG10 readthrough | -4,550 | Other | other |
| DNAJC4 | ENSG00000110011 | DnaJ heat shock protein family (Hsp40) member C4 | -0,936 | Cytoplasm | other |
| DNAL4 | ENSG00000100246 | dynein axonemal light chain 4 | -0,830 | Cytoplasm | enzyme |
| DND1 | ENSG00000256453 | DND microRNA-mediated repression inhibitor 1 | 3,615 | Cytoplasm | other |
| DNM1 | ENSG00000106976 | dynamin 1 | -1,170 | Cytoplasm | enzyme |
| DNMBP | ENSG00000107554 | dynamin binding protein | 0,721 | Cytoplasm | other |
| DNPEP | ENSG00000123992 | aspartyl aminopeptidase | 0,944 | Cytoplasm | peptidase |
| DNPH1 | ENSG00000112667 | 2'-deoxynucleoside 5'-phosphate N-hydrolase 1 | -0,724 | Nucleus | enzyme |
| DNTTIP2 | ENSG00000067334 | deoxynucleotidyltransferase terminal interacting protein 2 | 0,658 | Nucleus | other |
| DOHH | ENSG00000129932 | deoxyhypusine hydroxylase | 0,694 | Cytoplasm | enzyme |
| DPCD | ENSG00000166171 | deleted in primary ciliary dyskinesia homolog (mouse) | -0,794 | Nucleus | other |
| DPH6 | ENSG00000134146 | diphthamine biosynthesis 6 | -1,028 | Cytoplasm | enzyme |

| | | | | | |
|---|---|---|---|---|---|
| DPM3 | ENSG00000179085 | dolichyl-phosphate mannosyltransferase subunit 3, regulatory | -1,023 | Cytoplasm | enzyme |
| DPY19L4 | ENSG00000156162 | dpy-19 like 4 | -0,708 | Other | other |
| DPYSL2 | ENSG00000092964 | dihydropyrimidinase like 2 | -1,346 | Cytoplasm | enzyme |
| DPYSL3 | ENSG00000113657 | dihydropyrimidinase like 3 | -0,661 | Cytoplasm | enzyme |
| DRAM1 | ENSG00000136048 | DNA damage regulated autophagy modulator 1 | 0,969 | Cytoplasm | other |
| DRAM2 | ENSG00000156171 | DNA damage regulated autophagy modulator 2 | -0,610 | Cytoplasm | other |
| DSC3 | ENSG00000134762 | desmocollin 3 | -0,627 | Plasma Membrane | other |
| DSEL | ENSG00000262102 | dermatan sulfate epimerase like | -1,043 | Extracellular Space | enzyme |
| DSG2 | ENSG00000046604 | desmoglein 2 | -0,668 | Plasma Membrane | other |
| DSTN | ENSG00000125868 | destrin, actin depolymerizing factor | -0,752 | Cytoplasm | other |
| DTD2 | ENSG00000129480 | D-aminoacyl-tRNA deacylase 2 | -0,883 | Other | other |
| DTNB | ENSG00000138101 | dystrobrevin beta | -0,908 | Plasma Membrane | other |
| DTWD1 | ENSG00000104047 | DTW domain containing 1 | -0,633 | Nucleus | enzyme |
| DTX3L | ENSG00000163840 | deltex E3 ubiquitin ligase 3L | 2,142 | Cytoplasm | enzyme |
| DTX4 | ENSG00000110042 | deltex E3 ubiquitin ligase 4 | -0,655 | Cytoplasm | enzyme |
| DTYMK | ENSG00000168393 | deoxythymidylate kinase | -0,830 | Cytoplasm | kinase |
| DUOX1 | ENSG00000137857 | dual oxidase 1 | 1,894 | Plasma Membrane | enzyme |
| DUS3L | ENSG00000141994 | dihydrouridine synthase 3 like | 0,749 | Other | other |
| DUSP1 | ENSG00000120129 | dual specificity phosphatase 1 | 3,098 | Nucleus | phosphatase |
| DUSP10 | ENSG00000143507 | dual specificity phosphatase 10 | 2,042 | Nucleus | phosphatase |
| DUSP15 | ENSG00000149599 | dual specificity phosphatase 15 | 2,052 | Cytoplasm | phosphatase |
| DUSP16 | ENSG00000111266 | dual specificity phosphatase 16 | 1,271 | Nucleus | phosphatase |
| DUSP2 | ENSG00000158050 | dual specificity phosphatase 2 | 0,959 | Nucleus | phosphatase |
| DUSP4 | ENSG00000120875 | dual specificity phosphatase 4 | 0,878 | Nucleus | phosphatase |
| DUSP5 | ENSG00000138166 | dual specificity phosphatase 5 | 1,662 | Nucleus | phosphatase |
| DUSP8 | ENSG00000184545 | dual specificity phosphatase 8 | 1,673 | Nucleus | phosphatase |
| DYNC2H1 | ENSG00000187240 | dynein cytoplasmic 2 heavy chain 1 | -0,940 | Cytoplasm | other |
| DYNC2LI1 | ENSG00000138036 | dynein cytoplasmic 2 light intermediate chain 1 | -0,636 | Cytoplasm | other |
| DYRK1A | ENSG00000157540 | dual specificity tyrosine phosphorylation regulated kinase 1A | 1,104 | Nucleus | kinase |
| E2F6 | ENSG00000169016 | E2F transcription factor 6 | 0,608 | Nucleus | transcription regulator |
| E2F7 | ENSG00000165891 | E2F transcription factor 7 | 1,119 | Nucleus | transcription regulator |
| EBF4 | ENSG00000088881 | EBF family member 4 | 1,618 | Nucleus | transcription regulator |
| EBP | ENSG00000147155 | EBP cholestenol delta-isomerase | -1,034 | Cytoplasm | enzyme |
| ECH1 | ENSG00000104823 | enoyl-CoA hydratase 1 | -1,087 | Cytoplasm | enzyme |
| ECHDC1 | ENSG00000093144 | ethylmalonyl-CoA decarboxylase 1 | -0,671 | Cytoplasm | enzyme |
| ECI2 | ENSG00000198721 | enoyl-CoA delta isomerase 2 | -1,056 | Cytoplasm | enzyme |
| EDN1 | ENSG00000078401 | endothelin 1 | 0,817 | Extracellular Space | cytokine |
| EDNRA | ENSG00000151617 | endothelin receptor type A | 0,668 | Plasma Membrane | transmembrane receptor |
| EEF1A1 | ENSG00000156508 | eukaryotic translation elongation factor 1 alpha 1 | -0,874 | Cytoplasm | translation regulator |
| EEF1A1P24 | ENSG00000223668 | eukaryotic translation elongation factor 1 alpha 1 pseudogene 24 | -0,924 | Other | other |
| EEF1A2 | ENSG00000101210 | eukaryotic translation elongation factor 1 alpha 2 | -0,757 | Cytoplasm | translation regulator |
| EEF1AKMT1 | ENSG00000150456 | EEF1A lysine methyltransferase 1 | -0,880 | Cytoplasm | enzyme |
| EEF1G | ENSG00000254772 | eukaryotic translation elongation factor 1 gamma | -0,682 | Cytoplasm | translation regulator |
| EFCAB11 | ENSG00000140025 | EF-hand calcium binding domain 11 | -0,879 | Other | other |
| EFHC2 | ENSG00000183690 | EF-hand domain containing 2 | -0,818 | Other | other |
| EFNA1 | ENSG00000169242 | ephrin A1 | 0,734 | Plasma Membrane | other |
| EGLN3 | ENSG00000129521 | egl-9 family hypoxia inducible factor 3 | 0,820 | Cytoplasm | enzyme |
| EGR1 | ENSG00000120738 | early growth response 1 | 2,123 | Nucleus | transcription regulator |
| EGR2 | ENSG00000122877 | early growth response 2 | 1,794 | Nucleus | transcription regulator |
| EGR3 | ENSG00000179388 | early growth response 3 | 1,115 | Nucleus | transcription regulator |
| EGR4 | ENSG00000135625 | early growth response 4 | 2,003 | Nucleus | transcription regulator |
| EHD4 | ENSG00000103966 | EH domain containing 4 | 1,268 | Plasma Membrane | enzyme |
| EHMT2 | ENSG00000206376 | euchromatic histone lysine methyltransferase 2 | -0,794 | Nucleus | transcription regulator |
| EIF2AK2 | ENSG00000055332 | eukaryotic translation initiation factor 2 alpha kinase 2 | 1,689 | Cytoplasm | kinase |
| EIF4E3 | ENSG00000163412 | eukaryotic translation initiation factor 4E family member 3 | -1,095 | Cytoplasm | translation regulator |
| EIF4EBP3 | ENSG00000243056 | eukaryotic translation initiation factor 4E binding protein 3 | 0,966 | Cytoplasm | other |
| EIF5A2 | ENSG00000163577 | eukaryotic translation initiation factor 5A2 | 0,635 | Cytoplasm | translation regulator |
| ELF1 | ENSG00000120690 | E74 like ETS transcription factor 1 | 0,601 | Nucleus | transcription regulator |
| ELL | ENSG00000105656 | elongation factor for RNA polymerase II | 0,717 | Nucleus | transcription regulator |
| ELL2 | ENSG00000118985 | elongation factor for RNA polymerase II 2 | 0,816 | Nucleus | transcription regulator |
| ELOVL3 | ENSG00000119915 | ELOVL fatty acid elongase 3 | 1,779 | Cytoplasm | enzyme |
| EMP2 | ENSG00000213853 | epithelial membrane protein 2 | -0,873 | Plasma Membrane | other |
| ENDOD1 | ENSG00000149218 | endonuclease domain containing 1 | 1,080 | Extracellular Space | enzyme |
| ENDOG | ENSG00000167136 | endonuclease G | -0,618 | Cytoplasm | enzyme |
| ENO1 | ENSG00000074800 | enolase 1 | -0,616 | Cytoplasm | enzyme |
| ENPP1 | ENSG00000197594 | ectonucleotide pyrophosphatase/phosphodiesterase 1 | 0,683 | Plasma Membrane | enzyme |
| ENTPD2 | ENSG00000054179 | ectonucleoside triphosphate diphosphohydrolase 2 | -0,852 | Cytoplasm | enzyme |
| ENTPD3 | ENSG00000168032 | ectonucleoside triphosphate diphosphohydrolase 3 | -1,050 | Plasma Membrane | enzyme |
| EPB41L1 | ENSG00000088367 | erythrocyte membrane protein band 4.1 like 1 | -0,773 | Plasma Membrane | other |
| EPB41L4A | ENSG00000129595 | erythrocyte membrane protein band 4.1 like 4A | -0,748 | Extracellular Space | other |
| EPC1 | ENSG00000120616 | enhancer of polycomb homolog 1 | 1,386 | Nucleus | transcription regulator |
| EPC2 | ENSG00000135999 | enhancer of polycomb homolog 2 | 0,836 | Other | other |
| EPHA4 | ENSG00000116106 | EPH receptor A4 | 2,713 | Plasma Membrane | kinase |
| EPHA7 | ENSG00000135333 | EPH receptor A7 | 0,609 | Plasma Membrane | kinase |
| EPHX1 | ENSG00000143819 | epoxide hydrolase 1 | -1,105 | Cytoplasm | peptidase |
| EPN3 | ENSG00000049283 | epsin 3 | -0,627 | Cytoplasm | other |
| EPS8 | ENSG00000151491 | epidermal growth factor receptor pathway substrate 8 | 0,789 | Plasma Membrane | peptidase |
| EPSTI1 | ENSG00000133106 | epithelial stromal interaction 1 | 4,034 | Other | other |
| ERAP1 | ENSG00000164307 | endoplasmic reticulum aminopeptidase 1 | 1,397 | Extracellular Space | peptidase |
| ERAP2 | ENSG00000164308 | endoplasmic reticulum aminopeptidase 2 | 1,372 | Cytoplasm | peptidase |
| ERCC2 | ENSG00000104884 | ERCC excision repair 2, TFIIH core complex helicase subunit | -0,856 | Nucleus | enzyme |
| ERCC4 | ENSG00000175595 | ERCC excision repair 4, endonuclease catalytic subunit | 0,645 | Nucleus | enzyme |
| ERCC6L2 | ENSG00000182150 | ERCC excision repair 6 like 2 | 0,935 | Other | enzyme |
| EREG | ENSG00000124882 | epiregulin | 1,148 | Extracellular Space | growth factor |
| ERG28 | ENSG00000133935 | ergosterol biosynthesis 28 homolog | -0,663 | Cytoplasm | other |
| ERGIC1 | ENSG00000113719 | endoplasmic reticulum-golgi intermediate compartment 1 | -0,688 | Cytoplasm | other |
| ERLIN2 | ENSG00000147475 | ER lipid raft associated 2 | -0,732 | Cytoplasm | other |
| ERMARD | ENSG00000130023 | ER membrane associated RNA degradation | -0,732 | Other | other |
| ERMP1 | ENSG00000099219 | endoplasmic reticulum metallopeptidase 1 | -0,875 | Cytoplasm | other |
| ERN1 | ENSG00000178607 | endoplasmic reticulum to nucleus signaling 1 | 0,729 | Cytoplasm | kinase |
| ERO1B | ENSG00000086619 | endoplasmic reticulum oxidoreductase 1 beta | 1,112 | Cytoplasm | enzyme |
| ERRFI1 | ENSG00000116285 | ERBB receptor feedback inhibitor 1 | 0,981 | Cytoplasm | other |
| ESF1 | ENSG00000089048 | ESF1 nucleolar pre-rRNA processing protein homolog | 0,718 | Nucleus | other |
| ETFB | ENSG00000105379 | electron transfer flavoprotein subunit beta | -1,037 | Cytoplasm | transporter |
| ETS2 | ENSG00000157557 | ETS proto-oncogene 2, transcription factor | 2,116 | Nucleus | transcription regulator |
| ETV3 | ENSG00000117036 | ETS variant transcription factor 3 | 1,680 | Nucleus | transcription regulator |
| ETV7 | ENSG00000010030 | ETS variant transcription factor 7 | 4,903 | Nucleus | transcription regulator |
| EVL | ENSG00000196405 | Enah/Vasp-like | -0,691 | Plasma Membrane | other |
| EXOC3L1 | ENSG00000179044 | exocyst complex component 3 like 1 | 1,506 | Cytoplasm | other |

| | | | | | |
|---|---|---|---|---|---|
| EXOC3L4 | ENSG00000205436 | exocyst complex component 3 like 4 | 1,521 | Other | other |
| EXT1 | ENSG00000182197 | exostosin glycosyltransferase 1 | 1,196 | Cytoplasm | enzyme |
| EYA1 | ENSG00000104313 | EYA transcriptional coactivator and phosphatase 1 | 1,475 | Nucleus | phosphatase |
| F12 | ENSG00000131187 | coagulation factor XII | -0,753 | Extracellular Space | peptidase |
| F2RL1 | ENSG00000164251 | F2R like trypsin receptor 1 | 0,905 | Plasma Membrane | G-protein coupled receptor |
| F3 | ENSG00000117525 | coagulation factor III, tissue factor | 0,755 | Plasma Membrane | transmembrane receptor |
| FA2H | ENSG00000103089 | fatty acid 2-hydroxylase | 1,298 | Cytoplasm | enzyme |
| FAAP20 | ENSG00000162585 | FA core complex associated protein 20 | -0,666 | Nucleus | other |
| FABP6 | ENSG00000170231 | fatty acid binding protein 6 | -1,448 | Cytoplasm | transporter |
| FADS1 | ENSG00000149485 | fatty acid desaturase 1 | -0,826 | Plasma Membrane | enzyme |
| FADS2 | ENSG00000134824 | fatty acid desaturase 2 | -1,084 | Plasma Membrane | enzyme |
| FADS3 | ENSG00000221968 | fatty acid desaturase 3 | -0,744 | Plasma Membrane | enzyme |
| FAHD1 | ENSG00000180185 | fumarylacetoacetate hydrolase domain containing 1 | -0,627 | Cytoplasm | enzyme |
| FAM102B | ENSG00000162636 | family with sequence similarity 102 member B | -0,714 | Other | other |
| FAM104B | ENSG00000182518 | family with sequence similarity 104 member B | -1,147 | Other | other |
| FAM110B | ENSG00000169122 | family with sequence similarity 110 member B | 0,883 | Cytoplasm | other |
| FAM122C | ENSG00000156500 | family with sequence similarity 122C | 0,710 | Other | other |
| FAM126B | ENSG00000155744 | family with sequence similarity 126 member B | 0,634 | Other | other |
| FAM149A | ENSG00000109794 | family with sequence similarity 149 member A | -0,940 | Other | other |
| FAM153A/FAM153B | ENSG00000182230 | family with sequence similarity 153 member B | 0,703 | Other | other |
| FAM155B | ENSG00000130054 | family with sequence similarity 155 member B | 0,631 | Plasma Membrane | transmembrane receptor |
| FAM160B1 | ENSG00000151553 | family with sequence similarity 160 member B1 | 0,785 | Other | other |
| FAM161A | ENSG00000170264 | FAM161 centrosomal protein A | 0,836 | Cytoplasm | other |
| FAM162A | ENSG00000114023 | family with sequence similarity 162 member A | -0,988 | Cytoplasm | other |
| FAM166C | ENSG00000173557 | family with sequence similarity 166 member C | 0,697 | Nucleus | other |
| FAM168A | ENSG00000054965 | family with sequence similarity 168 member A | -0,892 | Other | other |
| FAM168B | ENSG00000152102 | family with sequence similarity 168 member B | -0,707 | Cytoplasm | other |
| FAM169A | ENSG00000198780 | family with sequence similarity 169 member A | 0,714 | Other | other |
| FAM185A | ENSG00000222011 | family with sequence similarity 185 member A | -0,677 | Cytoplasm | other |
| FAM193A | ENSG00000125386 | family with sequence similarity 193 member A | 0,745 | Other | other |
| FAM20B | ENSG00000116199 | FAM20B glycosaminoglycan xylosylkinase | -0,635 | Extracellular Space | enzyme |
| FAM210B | ENSG00000124098 | family with sequence similarity 210 member B | -0,743 | Extracellular Space | other |
| FAM214A | ENSG00000047346 | family with sequence similarity 214 member A | 0,990 | Other | other |
| FAM219A | ENSG00000164970 | family with sequence similarity 219 member A | 1,106 | Other | other |
| FAM221A | ENSG00000188732 | family with sequence similarity 221 member A | 0,634 | Other | other |
| FAM43A | ENSG00000185112 | family with sequence similarity 43 member A | 1,642 | Other | other |
| FAM47E-STBD1 | ENSG00000272414 | FAM47E-STBD1 readthrough | 0,746 | Other | other |
| FAM53C | ENSG00000120709 | family with sequence similarity 53 member C | 0,793 | Other | other |
| FAM83B | ENSG00000168143 | family with sequence similarity 83 member B | 0,697 | Cytoplasm | other |
| FAM89B | ENSG00000176973 | family with sequence similarity 89 member B | -0,635 | Cytoplasm | other |
| FANCM | ENSG00000187790 | FA complementation group M | 0,794 | Nucleus | enzyme |
| FAP | ENSG00000078098 | fibroblast activation protein alpha | 4,699 | Cytoplasm | peptidase |
| FARP1 | ENSG00000152767 | FERM, ARH/RhoGEF and pleckstrin domain protein 1 | -0,942 | Plasma Membrane | other |
| FARSB | ENSG00000116120 | phenylalanyl-tRNA synthetase subunit beta | -0,996 | Cytoplasm | enzyme |
| FAS | ENSG00000026103 | Fas cell surface death receptor | 1,893 | Plasma Membrane | transmembrane receptor |
| FASN | ENSG00000169710 | fatty acid synthase | -0,796 | Cytoplasm | enzyme |
| FAT2 | ENSG00000086570 | FAT atypical cadherin 2 | 0,740 | Plasma Membrane | other |
| FBN2 | ENSG00000138829 | fibrillin 2 | 0,615 | Extracellular Space | other |
| FBP1 | ENSG00000165140 | fructose-bisphosphatase 1 | -1,485 | Cytoplasm | phosphatase |
| FBXL12 | ENSG00000127452 | F-box and leucine rich repeat protein 12 | 0,750 | Cytoplasm | other |
| FBXL21P | ENSG00000164616 | F-box and leucine rich repeat protein 21, pseudogene | -1,091 | Cytoplasm | enzyme |
| FBXL22 | ENSG00000197361 | F-box and leucine rich repeat protein 22 | -0,712 | Other | enzyme |
| FBXO15 | ENSG00000141665 | F-box protein 15 | -0,786 | Cytoplasm | transcription regulator |
| FBXO17 | ENSG00000269190 | F-box protein 17 | -0,870 | Cytoplasm | enzyme |
| FBXO2 | ENSG00000116661 | F-box protein 2 | -0,638 | Cytoplasm | enzyme |
| FBXO38 | ENSG00000145868 | F-box protein 38 | 0,697 | Other | other |
| FBXO42 | ENSG00000037637 | F-box protein 42 | 0,677 | Other | other |
| FBXO6 | ENSG00000116663 | F-box protein 6 | 2,588 | Cytoplasm | other |
| FBXO9 | ENSG00000112146 | F-box protein 9 | -0,829 | Cytoplasm | enzyme |
| FBXW10 | ENSG00000171931 | F-box and WD repeat domain containing 10 | 1,802 | Cytoplasm | other |
| FBXW7 | ENSG00000109670 | F-box and WD repeat domain containing 7 | 0,891 | Nucleus | enzyme |
| FCGRT | ENSG00000104870 | Fc fragment of IgG receptor and transporter | -0,725 | Plasma Membrane | transmembrane receptor |
| FCRL5 | ENSG00000143297 | Fc receptor like 5 | 0,614 | Plasma Membrane | other |
| FDFT1 | ENSG00000079459 | farnesyl-diphosphate farnesyltransferase 1 | -1,230 | Cytoplasm | enzyme |
| FDPS | ENSG00000160752 | farnesyl diphosphate synthase | -1,212 | Cytoplasm | enzyme |
| FEM1A | ENSG00000141965 | fem-1 homolog A | 0,781 | Nucleus | transcription regulator |
| FEZ1 | ENSG00000149557 | fasciculation and elongation protein zeta 1 | 0,920 | Cytoplasm | other |
| FFAR4 | ENSG00000186188 | free fatty acid receptor 4 | -0,689 | Plasma Membrane | G-protein coupled receptor |
| FGD6 | ENSG00000180263 | FYVE, RhoGEF and PH domain containing 6 | 0,847 | Cytoplasm | other |
| FGF2 | ENSG00000138685 | fibroblast growth factor 2 | 0,625 | Extracellular Space | growth factor |
| FGFR1OP2 | ENSG00000111790 | FGFR1 oncogene partner 2 | 0,717 | Cytoplasm | other |
| FGFR3 | ENSG00000068078 | fibroblast growth factor receptor 3 | -1,716 | Plasma Membrane | kinase |
| FGFR4 | ENSG00000160867 | fibroblast growth factor receptor 4 | -0,735 | Plasma Membrane | kinase |
| FH | ENSG00000091483 | fumarate hydratase | -0,635 | Cytoplasm | enzyme |
| FIG4 | ENSG00000112367 | FIG4 phosphoinositide 5-phosphatase | -0,607 | Cytoplasm | enzyme |
| FILIP1L | ENSG00000168386 | filamin A interacting protein 1 like | 1,460 | Nucleus | other |
| FITM2 | ENSG00000197296 | fat storage inducing transmembrane protein 2 | -0,870 | Cytoplasm | other |
| FIZ1 | ENSG00000179943 | FLT3 interacting zinc finger 1 | 0,761 | Nucleus | transcription regulator |
| FKBPL | ENSG00000223666 | FKBP prolyl isomerase like | -2,764 | Nucleus | other |
| FKRP | ENSG00000181027 | fukutin related protein | 0,937 | Cytoplasm | other |
| FLCN | ENSG00000154803 | folliculin | 0,837 | Cytoplasm | other |
| FLRT3 | ENSG00000125848 | fibronectin leucine rich transmembrane protein 3 | 1,535 | Plasma Membrane | other |
| FLT3LG | ENSG00000090554 | fms related receptor tyrosine kinase 3 ligand | 2,377 | Extracellular Space | cytokine |
| FLVCR1 | ENSG00000162769 | FLVCR heme transporter 1 | -0,713 | Plasma Membrane | transporter |
| FLYWCH2 | ENSG00000162076 | FLYWCH family member 2 | -0,924 | Other | other |
| FMR1 | ENSG00000102081 | FMRP translational regulator 1 | 0,984 | Cytoplasm | translation regulator |
| FN1 | ENSG00000115414 | fibronectin 1 | 0,829 | Extracellular Space | enzyme |
| FNBP4 | ENSG00000109920 | formin binding protein 4 | 0,674 | Nucleus | other |
| FNDC3A | ENSG00000102531 | fibronectin type III domain containing 3A | 1,982 | Cytoplasm | other |
| FNIP1 | ENSG00000217128 | folliculin interacting protein 1 | 0,773 | Cytoplasm | other |
| FOCAD | ENSG00000188352 | focadhesin | -0,940 | Plasma Membrane | other |
| FOLR1 | ENSG00000110195 | folate receptor alpha | -1,232 | Plasma Membrane | transporter |
| FOS | ENSG00000170345 | Fos proto-oncogene, AP-1 transcription factor subunit | 2,538 | Nucleus | transcription regulator |
| FOSB | ENSG00000125740 | FosB proto-oncogene, AP-1 transcription factor subunit | 1,971 | Nucleus | transcription regulator |
| FOSL1 | ENSG00000175592 | FOS like 1, AP-1 transcription factor subunit | 1,272 | Nucleus | transcription regulator |
| FOSL2 | ENSG00000075426 | FOS like 2, AP-1 transcription factor subunit | 1,157 | Nucleus | transcription regulator |
| FOXN2 | ENSG00000170802 | forkhead box N2 | 0,770 | Nucleus | transcription regulator |
| FOXN3 | ENSG00000053254 | forkhead box N3 | -1,032 | Nucleus | transcription regulator |
| FOXO1 | ENSG00000150907 | forkhead box O1 | 1,452 | Nucleus | transcription regulator |

| | | | | | |
|---|---|---|---|---|---|
| FOXRED2 | ENSG00000100350 | FAD dependent oxidoreductase domain containing 2 | -0,609 | Cytoplasm | other |
| FPGT | ENSG00000254685 | fucose-1-phosphate guanylyltransferase | -0,764 | Cytoplasm | enzyme |
| FRAS1 | ENSG00000138759 | Fraser extracellular matrix complex subunit 1 | -1,093 | Extracellular Space | other |
| FRMD3 | ENSG00000172159 | FERM domain containing 3 | 0,897 | Other | other |
| FRMD4B | ENSG00000114541 | FERM domain containing 4B | 0,612 | Cytoplasm | other |
| FRS2 | ENSG00000166225 | fibroblast growth factor receptor substrate 2 | 1,569 | Plasma Membrane | other |
| FRYL | ENSG00000075539 | FRY like transcription coactivator | 1,008 | Other | other |
| FSCN1 | ENSG00000075618 | fascin actin-bundling protein 1 | 1,449 | Cytoplasm | other |
| FSD1L | ENSG00000106701 | fibronectin type III and SPRY domain containing 1 like | 0,992 | Other | other |
| FST | ENSG00000134363 | follistatin | 2,868 | Extracellular Space | other |
| FSTL3 | ENSG00000070404 | follistatin like 3 | 0,819 | Extracellular Space | other |
| FUT4 | ENSG00000196371 | fucosyltransferase 4 | 1,025 | Cytoplasm | enzyme |
| FXYD2 | ENSG00000137731 | FXYD domain containing ion transport regulator 2 | -1,360 | Plasma Membrane | ion channel |
| FXYD6 | ENSG00000137726 | FXYD domain containing ion transport regulator 6 | 0,968 | Plasma Membrane | ion channel |
| FYB1 | ENSG00000082074 | FYN binding protein 1 | 1,018 | Nucleus | other |
| FYB2 | ENSG00000187889 | FYN binding protein 2 | 2,828 | Plasma Membrane | other |
| FZD4 | ENSG00000174804 | frizzled class receptor 4 | 2,389 | Plasma Membrane | G-protein coupled receptor |
| FZD5 | ENSG00000163251 | frizzled class receptor 5 | 0,748 | Plasma Membrane | G-protein coupled receptor |
| G0S2 | ENSG00000123689 | G0/G1 switch 2 | 2,549 | Cytoplasm | other |
| G6PD | ENSG00000160211 | glucose-6-phosphate dehydrogenase | -0,799 | Cytoplasm | enzyme |
| GAA | ENSG00000171298 | glucosidase alpha, acid | -0,626 | Cytoplasm | enzyme |
| GABARAP | ENSG00000170296 | GABA type A receptor-associated protein | -0,697 | Cytoplasm | transporter |
| GABRE | ENSG00000102287 | gamma-aminobutyric acid type A receptor subunit epsilon | 0,637 | Plasma Membrane | ion channel |
| GABRP | ENSG00000094755 | gamma-aminobutyric acid type A receptor subunit pi | -0,630 | Plasma Membrane | ion channel |
| GADD45A | ENSG00000116717 | growth arrest and DNA damage inducible alpha | 1,417 | Nucleus | other |
| GADD45B | ENSG00000099860 | growth arrest and DNA damage inducible beta | 1,699 | Cytoplasm | other |
| GADD45G | ENSG00000130222 | growth arrest and DNA damage inducible gamma | 1,123 | Nucleus | other |
| GALM | ENSG00000143891 | galactose mutarotase | -1,005 | Cytoplasm | enzyme |
| GALNT1 | ENSG00000141429 | polypeptide N-acetylgalactosaminyltransferase 1 | -0,830 | Cytoplasm | enzyme |
| GALNT10 | ENSG00000164574 | polypeptide N-acetylgalactosaminyltransferase 10 | -1,009 | Cytoplasm | enzyme |
| GALNT12 | ENSG00000119514 | polypeptide N-acetylgalactosaminyltransferase 12 | -1,033 | Cytoplasm | enzyme |
| GALNT5 | ENSG00000136542 | polypeptide N-acetylgalactosaminyltransferase 5 | 0,612 | Cytoplasm | enzyme |
| GAMT | ENSG00000130005 | guanidinoacetate N-methyltransferase | -1,000 | Cytoplasm | enzyme |
| GANC | ENSG00000214013 | glucosidase alpha, neutral C | 0,679 | Extracellular Space | enzyme |
| GAREM1 | ENSG00000141441 | GRB2 associated regulator of MAPK1 subtype 1 | 0,699 | Nucleus | other |
| GARS1 | ENSG00000106105 | glycyl-tRNA synthetase 1 | 0,601 | Cytoplasm | enzyme |
| GAS2 | ENSG00000148935 | growth arrest specific 2 | -0,688 | Cytoplasm | other |
| GAS7 | ENSG00000007237 | growth arrest specific 7 | 1,413 | Cytoplasm | transcription regulator |
| GATA3 | ENSG00000107485 | GATA binding protein 3 | 1,725 | Nucleus | transcription regulator |
| GATA4 | ENSG00000136574 | GATA binding protein 4 | 1,957 | Nucleus | transcription regulator |
| GATA6 | ENSG00000141448 | GATA binding protein 6 | 0,761 | Nucleus | transcription regulator |
| GATD1 | ENSG00000177225 | glutamine amidotransferase like class 1 domain containing 1 | -0,763 | Cytoplasm | other |
| GBP1 | ENSG00000117228 | guanylate binding protein 1 | 3,924 | Cytoplasm | enzyme |
| GBP1P1 | ENSG00000225492 | guanylate binding protein 1 pseudogene 1 | 3,678 | Other | other |
| GBP2 | ENSG00000162645 | guanylate binding protein 2 | 0,830 | Cytoplasm | enzyme |
| GBP3 | ENSG00000117226 | guanylate binding protein 3 | 2,178 | Cytoplasm | enzyme |
| GBP4 | ENSG00000162654 | guanylate binding protein 4 | 5,114 | Cytoplasm | enzyme |
| GBP5 | ENSG00000154451 | guanylate binding protein 5 | 4,367 | Plasma Membrane | enzyme |
| GBP6 | ENSG00000183347 | guanylate binding protein family member 6 | 0,835 | Cytoplasm | enzyme |
| GBP7 | ENSG00000213512 | guanylate binding protein 7 | 1,631 | Cytoplasm | enzyme |
| GCA | ENSG00000115271 | grancalcin | 1,437 | Cytoplasm | other |
| GCAT | ENSG00000100116 | glycine C-acetyltransferase | -0,790 | Cytoplasm | enzyme |
| GCC1 | ENSG00000179562 | GRIP and coiled-coil domain containing 1 | 0,732 | Cytoplasm | other |
| GCH1 | ENSG00000131979 | GTP cyclohydrolase 1 | 1,556 | Cytoplasm | enzyme |
| GCHFR | ENSG00000137880 | GTP cyclohydrolase I feedback regulator | -0,833 | Cytoplasm | other |
| GCNA | ENSG00000147174 | germ cell nuclear acidic peptidase | 0,965 | Nucleus | other |
| GCNT2 | ENSG00000111846 | glucosaminyl (N-acetyl) transferase 2 (I blood group) | 0,606 | Cytoplasm | enzyme |
| GCNT4 | ENSG00000176928 | glucosaminyl (N-acetyl) transferase 4 | 1,028 | Cytoplasm | enzyme |
| GCSH | ENSG00000140905 | glycine cleavage system protein H | -0,780 | Cytoplasm | enzyme |
| GDF15 | ENSG00000130513 | growth differentiation factor 15 | 2,178 | Extracellular Space | growth factor |
| GEM | ENSG00000164949 | GTP binding protein overexpressed in skeletal muscle | 1,384 | Plasma Membrane | enzyme |
| GEMIN8 | ENSG00000046647 | gem nuclear organelle associated protein 8 | -0,836 | Cytoplasm | other |
| GET1 | ENSG00000182093 | guided entry of tail-anchored proteins factor 1 | -1,424 | Extracellular Space | other |
| GGA3 | ENSG00000125447 | golgi associated, gamma adaptin ear containing, ARF binding protein 3 | 0,671 | Cytoplasm | transporter |
| GGH | ENSG00000137563 | gamma-glutamyl hydrolase | -0,650 | Cytoplasm | peptidase |
| GGT1 | ENSG00000100031 | gamma-glutamyltransferase 1 | -1,307 | Plasma Membrane | enzyme |
| GIMAP2 | ENSG00000106560 | GTPase, IMAP family member 2 | 2,618 | Cytoplasm | other |
| GJB1 | ENSG00000169562 | gap junction protein beta 1 | -1,558 | Plasma Membrane | transporter |
| GLCCI1 | ENSG00000106415 | glucocorticoid induced 1 | 2,500 | Cytoplasm | other |
| GLIS2 | ENSG00000126603 | GLIS family zinc finger 2 | -0,730 | Nucleus | transcription regulator |
| GLO1 | ENSG00000124767 | glyoxalase I | -0,831 | Cytoplasm | enzyme |
| GLTP | ENSG00000139433 | glycolipid transfer protein | -0,690 | Cytoplasm | transporter |
| GLTPD2 | ENSG00000182327 | glycolipid transfer protein domain containing 2 | -1,020 | Other | other |
| GMPR | ENSG00000137198 | guanosine monophosphate reductase | 3,419 | Cytoplasm | enzyme |
| GNA13 | ENSG00000120063 | G protein subunit alpha 13 | 0,688 | Plasma Membrane | enzyme |
| GNB1L | ENSG00000185838 | G protein subunit beta 1 like | -0,608 | Cytoplasm | other |
| GNB4 | ENSG00000114450 | G protein subunit beta 4 | 1,413 | Plasma Membrane | enzyme |
| GNG10 | ENSG00000242616 | G protein subunit gamma 10 | -1,001 | Plasma Membrane | other |
| GNG12 | ENSG00000172380 | G protein subunit gamma 12 | -0,992 | Plasma Membrane | enzyme |
| GNL1 | ENSG00000226882 | G protein nucleolar 1 (putative) | -0,839 | Nucleus | other |
| GNPAT | ENSG00000116906 | glyceronephosphate O-acyltransferase | -0,677 | Cytoplasm | enzyme |
| GNS | ENSG00000135677 | glucosamine (N-acetyl)-6-sulfatase | -0,692 | Cytoplasm | enzyme |
| GOLIM4 | ENSG00000173905 | golgi integral membrane protein 4 | -0,908 | Cytoplasm | other |
| GOLM2 | ENSG00000166734 | golgi membrane protein 2 | -0,677 | Cytoplasm | other |
| GORAB | ENSG00000120370 | golgin, RAB6 interacting | 0,864 | Cytoplasm | other |
| GOSR1 | ENSG00000108587 | golgi SNAP receptor complex member 1 | 0,618 | Cytoplasm | transporter |
| GPATCH2 | ENSG00000092978 | G-patch domain containing 2 | 0,870 | Nucleus | other |
| GPBP1 | ENSG00000062194 | GC-rich promoter binding protein 1 | 1,259 | Nucleus | transcription regulator |
| GPC3 | ENSG00000147257 | glypican 3 | -1,240 | Plasma Membrane | other |
| GPC4 | ENSG00000076716 | glypican 4 | -0,875 | Plasma Membrane | transmembrane receptor |
| GPCPD1 | ENSG00000125772 | glycerophosphocholine phosphodiesterase 1 | 1,486 | Cytoplasm | enzyme |
| GPNMB | ENSG00000136235 | glycoprotein nmb | -0,798 | Plasma Membrane | enzyme |
| GPR107 | ENSG00000148358 | G protein-coupled receptor 107 | -0,871 | Plasma Membrane | G-protein coupled receptor |
| GPR132 | ENSG00000183484 | G protein-coupled receptor 132 | -0,719 | Plasma Membrane | G-protein coupled receptor |
| GPR160 | ENSG00000173890 | G protein-coupled receptor 160 | -0,845 | Plasma Membrane | G-protein coupled receptor |
| GPR37 | ENSG00000170775 | G protein-coupled receptor 37 | 0,776 | Plasma Membrane | G-protein coupled receptor |
| GPR87 | ENSG00000138271 | G protein-coupled receptor 87 | 0,810 | Plasma Membrane | G-protein coupled receptor |
| GPSM2 | ENSG00000121957 | G protein signaling modulator 2 | -1,251 | Nucleus | other |
| GPT2 | ENSG00000166123 | glutamic--pyruvic transaminase 2 | 1,355 | Cytoplasm | enzyme |

| | | | | | |
|---|---|---|---|---|---|
| GPX3 | ENSG00000211445 | glutathione peroxidase 3 | 0,785 | Extracellular Space | enzyme |
| GRAMD1B | ENSG00000023171 | GRAM domain containing 1B | 0,677 | Other | other |
| GRB10 | ENSG00000106070 | growth factor receptor bound protein 10 | 1,399 | Cytoplasm | other |
| GRB14 | ENSG00000115290 | growth factor receptor bound protein 14 | -0,710 | Plasma Membrane | other |
| GREB1L | ENSG00000141449 | GREB1 like retinoic acid receptor coactivator | 0,790 | Extracellular Space | other |
| GRIN3A | ENSG00000198785 | glutamate ionotropic receptor NMDA type subunit 3A | 0,783 | Plasma Membrane | ion channel |
| GRINA | ENSG00000178719 | glutamate ionotropic receptor NMDA type subunit associated protein 1 | 0,923 | Other | ion channel |
| GRPR | ENSG00000126010 | gastrin releasing peptide receptor | 0,709 | Plasma Membrane | G-protein coupled receptor |
| GRWD1 | ENSG00000105447 | glutamate rich WD repeat containing 1 | 0,672 | Nucleus | other |
| GSDMB | ENSG00000073605 | gasdermin B | 2,918 | Cytoplasm | other |
| GSDMD | ENSG00000104518 | gasdermin D | 0,723 | Extracellular Space | other |
| GSN | ENSG00000148180 | gelsolin | -0,825 | Extracellular Space | other |
| GSR | ENSG00000104687 | glutathione-disulfide reductase | -0,693 | Cytoplasm | enzyme |
| GSTM3 | ENSG00000134202 | glutathione S-transferase mu 3 | -0,803 | Cytoplasm | enzyme |
| GSTO2 | ENSG00000065621 | glutathione S-transferase omega 2 | -0,630 | Cytoplasm | enzyme |
| GSTZ1 | ENSG00000100577 | glutathione S-transferase zeta 1 | -0,675 | Cytoplasm | enzyme |
| GTF2B | ENSG00000137947 | general transcription factor IIB | 0,952 | Nucleus | transcription regulator |
| GTF2H4 | ENSG00000234370 | general transcription factor IIH subunit 4 | 2,221 | Nucleus | transcription regulator |
| GTF2IP4 | ENSG00000233369 | general transcription factor IIi pseudogene 4 | -0,738 | Other | other |
| GTPBP1 | ENSG00000100226 | GTP binding protein 1 | 1,532 | Cytoplasm | enzyme |
| GTPBP10 | ENSG00000105793 | GTP binding protein 10 | 0,647 | Cytoplasm | other |
| GTPBP2 | ENSG00000172432 | GTP binding protein 2 | 0,901 | Extracellular Space | enzyme |
| GTSE1 | ENSG00000075218 | G2 and S-phase expressed 1 | -0,854 | Cytoplasm | other |
| GUCA1B | ENSG00000112599 | guanylate cyclase activator 1B | 0,910 | Cytoplasm | other |
| GUCY1B1 | ENSG00000061918 | guanylate cyclase 1 soluble subunit beta 1 | -0,604 | Cytoplasm | enzyme |
| GYG2 | ENSG00000056998 | glycogenin 2 | -0,966 | Cytoplasm | enzyme |
| H1-0 | ENSG00000189060 | H1.0 linker histone | 1,935 | Nucleus | other |
| H1-10 | ENSG00000184897 | H1.10 linker histone | 0,710 | Nucleus | other |
| H1-2 | ENSG00000187837 | H1.2 linker histone, cluster member | 0,656 | Nucleus | other |
| H2AC18/H2AC19 | ENSG00000272196 | H2A clustered histone 18 | 1,767 | Nucleus | other |
| H2AC6 | ENSG00000180573 | H2A clustered histone 6 | 0,854 | Nucleus | other |
| H2AW | ENSG00000181218 | H2A.W histone | 1,879 | Nucleus | other |
| H2AZ1 | ENSG00000164032 | H2A.Z variant histone 1 | -0,624 | Nucleus | other |
| H2AZ2 | ENSG00000105968 | H2A.Z variant histone 2 | -0,702 | Nucleus | other |
| H2BC11 | ENSG00000124635 | H2B clustered histone 11 | 0,986 | Nucleus | other |
| H2BC21 | ENSG00000184678 | H2B clustered histone 21 | 0,720 | Nucleus | other |
| H2BC5 | ENSG00000158373 | H2B clustered histone 5 | 0,747 | Nucleus | other |
| H3C14 | ENSG00000203811 | H3 clustered histone 14 | 1,264 | Nucleus | other |
| H3C15 | ENSG00000203852 | H3 clustered histone 15 | 1,264 | Nucleus | other |
| H3C4 | ENSG00000197409 | H3 clustered histone 4 | 1,794 | Nucleus | other |
| H3P6 | ENSG00000235655 | H3 histone pseudogene 6 | -0,673 | Other | other |
| H4C8 | ENSG00000158406 | H4 clustered histone 8 | 2,181 | Nucleus | other |
| H6PD | ENSG00000049239 | hexose-6-phosphate dehydrogenase/glucose 1-dehydrogenase | -0,733 | Cytoplasm | enzyme |
| HABP4 | ENSG00000130956 | hyaluronan binding protein 4 | -0,604 | Cytoplasm | other |
| HACD2 | ENSG00000206527 | 3-hydroxyacyl-CoA dehydratase 2 | -0,935 | Cytoplasm | phosphatase |
| HACD3 | ENSG00000074696 | 3-hydroxyacyl-CoA dehydratase 3 | -0,799 | Cytoplasm | enzyme |
| HADH | ENSG00000138796 | hydroxyacyl-CoA dehydrogenase | -1,227 | Cytoplasm | enzyme |
| HADHB | ENSG00000138029 | hydroxyacyl-CoA dehydrogenase trifunctional multienzyme complex subunit beta | -0,800 | Cytoplasm | enzyme |
| HAGHL | ENSG00000103253 | hydroxyacylglutathione hydrolase like | -0,625 | Other | enzyme |
| HAP1 | ENSG00000173805 | huntingtin associated protein 1 | 0,783 | Cytoplasm | other |
| HAPLN3 | ENSG00000140511 | hyaluronan and proteoglycan link protein 3 | 1,505 | Extracellular Space | other |
| HAS2 | ENSG00000170961 | hyaluronan synthase 2 | 2,344 | Plasma Membrane | enzyme |
| HAVCR1 | ENSG00000113249 | hepatitis A virus cellular receptor 1 | -1,502 | Plasma Membrane | other |
| HBEGF | ENSG00000113070 | heparin binding EGF like growth factor | 1,813 | Extracellular Space | growth factor |
| HCFC1R1 | ENSG00000103145 | host cell factor C1 regulator 1 | -0,954 | Nucleus | other |
| HCG27 | ENSG00000206344 | HLA complex group 27 | 2,248 | Other | other |
| HCG4B | ENSG00000231941 | HLA complex group 4B | 2,674 | Other | other |
| HDAC9 | ENSG00000048052 | histone deacetylase 9 | 1,280 | Nucleus | transcription regulator |
| HDX | ENSG00000165259 | highly divergent homeobox | 1,840 | Other | other |
| HEATR5A | ENSG00000129493 | HEAT repeat containing 5A | 0,633 | Other | other |
| HEBP1 | ENSG00000013583 | heme binding protein 1 | -0,831 | Cytoplasm | other |
| HEBP2 | ENSG00000051620 | heme binding protein 2 | -0,840 | Cytoplasm | other |
| HELB | ENSG00000127311 | DNA helicase B | 1,114 | Nucleus | enzyme |
| HELQ | ENSG00000163312 | helicase, POLQ like | 0,909 | Nucleus | enzyme |
| HELZ2 | ENSG00000130589 | helicase with zinc finger 2 | 3,246 | Nucleus | transcription regulator |
| HEPH | ENSG00000089472 | hephaestin | 0,960 | Plasma Membrane | transporter |
| HERC5 | ENSG00000138646 | HECT and RLD domain containing E3 ubiquitin protein ligase 5 | 4,549 | Cytoplasm | enzyme |
| HERC6 | ENSG00000138642 | HECT and RLD domain containing E3 ubiquitin protein ligase family member 6 | 2,346 | Cytoplasm | enzyme |
| HERPUD1 | ENSG00000051108 | homocysteine inducible ER protein with ubiquitin like domain 1 | 1,317 | Cytoplasm | other |
| HES4 | ENSG00000188290 | hes family bHLH transcription factor 4 | 1,610 | Other | other |
| HEXD | ENSG00000169660 | hexosaminidase D | 1,195 | Extracellular Space | enzyme |
| HFE | ENSG00000010704 | homeostatic iron regulator | -0,660 | Plasma Membrane | transmembrane receptor |
| HGD | ENSG00000113924 | homogentisate 1,2-dioxygenase | -1,393 | Cytoplasm | enzyme |
| HGSNAT | ENSG00000165102 | heparan-alpha-glucosaminide N-acetyltransferase | -0,854 | Cytoplasm | enzyme |
| HID1 | ENSG00000167861 | HID1 domain containing | -0,732 | Plasma Membrane | other |
| HIP1R | ENSG00000130787 | huntingtin interacting protein 1 related | 1,163 | Cytoplasm | other |
| HIPK3 | ENSG00000110422 | homeodomain interacting protein kinase 3 | 1,080 | Nucleus | kinase |
| HIRA | ENSG00000100084 | histone cell cycle regulator | 0,921 | Nucleus | transcription regulator |
| HIVEP1 | ENSG00000095951 | HIVEP zinc finger 1 | 0,662 | Nucleus | transcription regulator |
| HIVEP2 | ENSG00000010818 | HIVEP zinc finger 2 | 2,235 | Nucleus | transcription regulator |
| HK2 | ENSG00000159399 | hexokinase 2 | 0,836 | Cytoplasm | kinase |
| HLA-A | ENSG00000206505 | major histocompatibility complex, class I, A | 2,099 | Plasma Membrane | other |
| HLA-B | ENSG00000223532 | major histocompatibility complex, class I, B | 3,419 | Plasma Membrane | transmembrane receptor |
| HLA-C | ENSG00000206435 | major histocompatibility complex, class I, C | 2,711 | Plasma Membrane | other |
| HLA-DMA | ENSG00000243719 | major histocompatibility complex, class II, DM alpha | 0,743 | Plasma Membrane | transmembrane receptor |
| HLA-DMB | ENSG00000239329 | major histocompatibility complex, class II, DM beta | 1,575 | Plasma Membrane | transmembrane receptor |
| HLA-DOB | ENSG00000243612 | major histocompatibility complex, class II, DO beta | 2,200 | Plasma Membrane | transmembrane receptor |
| HLA-DPA1 | ENSG00000229685 | major histocompatibility complex, class II, DP alpha 1 | 1,346 | Plasma Membrane | transmembrane receptor |
| HLA-DPB1 | ENSG00000215048 | major histocompatibility complex, class II, DP beta 1 | 1,849 | Plasma Membrane | transmembrane receptor |
| HLA-DQA1 | ENSG00000228284 | major histocompatibility complex, class II, DQ alpha 1 | 1,497 | Plasma Membrane | transmembrane receptor |
| HLA-DQB1 | ENSG00000231286 | major histocompatibility complex, class II, DQ beta 1 | 1,617 | Plasma Membrane | other |
| HLA-DRA | ENSG00000234794 | major histocompatibility complex, class II, DR alpha | 2,076 | Plasma Membrane | transmembrane receptor |
| HLA-DRB1 | ENSG00000206306 | major histocompatibility complex, class II, DR beta 1 | 1,355 | Plasma Membrane | transmembrane receptor |
| HLA-DRB3 | ENSG00000196101 | major histocompatibility complex, class II, DR beta 3 | 1,588 | Cytoplasm | other |
| HLA-DRB4 | ENSG00000231021 | major histocompatibility complex, class II, DR beta 4 | 1,506 | Plasma Membrane | transmembrane receptor |
| HLA-E | ENSG00000233904 | major histocompatibility complex, class I, E | 2,002 | Plasma Membrane | transmembrane receptor |
| HLA-F | ENSG00000229698 | major histocompatibility complex, class I, F | 4,693 | Plasma Membrane | transmembrane receptor |
| HLA-H | ENSG00000206341 | major histocompatibility complex, class I, H (pseudogene) | 3,009 | Plasma Membrane | other |
| HLA-J | ENSG00000243336 | major histocompatibility complex, class I, J (pseudogene) | 2,343 | Other | other |

| | | | | | |
|---|---|---|---|---|---|
| HLA-K | ENSG00000232708 | major histocompatibility complex, class I, K (pseudogene) | 4,084 | Plasma Membrane | other |
| HLA-L | ENSG00000243492 | major histocompatibility complex, class I, L (pseudogene) | 0,882 | Other | other |
| HLCS | ENSG00000159267 | holocarboxylase synthetase | -0,789 | Cytoplasm | enzyme |
| HLTF | ENSG00000071794 | helicase like transcription factor | -0,741 | Nucleus | transcription regulator |
| HMCES | ENSG00000183624 | 5-hydroxymethylcytosine binding, ES cell specific | -0,618 | Nucleus | other |
| HMGB1 | ENSG00000189403 | high mobility group box 1 | -0,741 | Nucleus | transcription regulator |
| HMGB3 | ENSG00000029993 | high mobility group box 3 | -0,939 | Nucleus | other |
| HMGCR | ENSG00000113161 | 3-hydroxy-3-methylglutaryl-CoA reductase | -0,967 | Cytoplasm | enzyme |
| HMGCS1 | ENSG00000112972 | 3-hydroxy-3-methylglutaryl-CoA synthase 1 | -0,663 | Cytoplasm | enzyme |
| HMGN5 | ENSG00000198157 | high mobility group nucleosome binding domain 5 | -0,666 | Nucleus | transcription regulator |
| HMMR | ENSG00000072571 | hyaluronan mediated motility receptor | -1,263 | Plasma Membrane | transmembrane receptor |
| HMSD | ENSG00000221887 | histocompatibility minor serpin domain containing | -0,859 | Other | other |
| HNF4A | ENSG00000101076 | hepatocyte nuclear factor 4 alpha | 0,877 | Nucleus | transcription regulator |
| HNMT | ENSG00000150540 | histamine N-methyltransferase | -0,812 | Cytoplasm | enzyme |
| HNRNPA1P48 | ENSG00000224578 | heterogeneous nuclear ribonucleoprotein A1 pseudogene 48 | -0,606 | Other | other |
| HNRNPUL2 | ENSG00000214753 | heterogeneous nuclear ribonucleoprotein U like 2 | -0,677 | Nucleus | other |
| HOGA1 | ENSG00000241935 | 4-hydroxy-2-oxoglutarate aldolase 1 | -1,068 | Cytoplasm | enzyme |
| HOMER2 | ENSG00000103942 | homer scaffold protein 2 | -0,905 | Plasma Membrane | other |
| HOXB7 | ENSG00000260027 | homeobox B7 | -0,670 | Nucleus | transcription regulator |
| HOXB8 | ENSG00000120068 | homeobox B8 | -0,623 | Nucleus | transcription regulator |
| HPF1 | ENSG00000056050 | histone PARylation factor 1 | -0,668 | Nucleus | other |
| HPSE | ENSG00000173083 | heparanase | 0,644 | Plasma Membrane | enzyme |
| HRH1 | ENSG00000196639 | histamine receptor H1 | 1,128 | Plasma Membrane | G-protein coupled receptor |
| HROB | ENSG00000125319 | homologous recombination factor with OB-fold | 0,871 | Other | other |
| HS2ST1 | ENSG00000153936 | heparan sulfate 2-O-sulfotransferase 1 | -0,651 | Cytoplasm | enzyme |
| HS3ST1 | ENSG00000002587 | heparan sulfate-glucosamine 3-sulfotransferase 1 | 0,738 | Cytoplasm | enzyme |
| HSBP1L1 | ENSG00000226742 | heat shock factor binding protein 1 like 1 | -0,899 | Other | other |
| HSCB | ENSG00000100209 | HscB mitochondrial iron-sulfur cluster cochaperone | 1,531 | Cytoplasm | other |
| HSD11B1L | ENSG00000167733 | hydroxysteroid 11-beta dehydrogenase 1 like | -1,590 | Nucleus | other |
| HSD17B14 | ENSG00000087076 | hydroxysteroid 17-beta dehydrogenase 14 | 3,499 | Cytoplasm | enzyme |
| HSD17B2 | ENSG00000086696 | hydroxysteroid 17-beta dehydrogenase 2 | -1,171 | Cytoplasm | enzyme |
| HSD17B4 | ENSG00000133835 | hydroxysteroid 17-beta dehydrogenase 4 | -0,836 | Cytoplasm | enzyme |
| HSD17B7 | ENSG00000132196 | hydroxysteroid 17-beta dehydrogenase 7 | -0,730 | Cytoplasm | enzyme |
| HSD17B7P2 | ENSG00000099251 | hydroxysteroid 17-beta dehydrogenase 7 pseudogene 2 | 2,469 | Other | other |
| HSD17B8 | ENSG00000232357 | hydroxysteroid 17-beta dehydrogenase 8 | -1,099 | Cytoplasm | enzyme |
| HSDL2 | ENSG00000119471 | hydroxysteroid dehydrogenase like 2 | -0,643 | Cytoplasm | transporter |
| HSFX1/HSFX2 | ENSG00000268738 | heat shock transcription factor family, X-linked 1 | -0,734 | Nucleus | transcription regulator |
| HSH2D | ENSG00000196684 | hematopoietic SH2 domain containing | 2,578 | Cytoplasm | other |
| HSPA1A/HSPA1B | ENSG00000224501 | heat shock protein family A (Hsp70) member 1A | 1,146 | Cytoplasm | enzyme |
| HSPA5 | ENSG00000044574 | heat shock protein family A (Hsp70) member 5 | 0,910 | Cytoplasm | enzyme |
| HSPA6 | ENSG00000173110 | heat shock protein family A (Hsp70) member 6 | 2,080 | Nucleus | enzyme |
| HSPB8 | ENSG00000152137 | heat shock protein family B (small) member 8 | 2,985 | Cytoplasm | kinase |
| HTATSF1 | ENSG00000102241 | HIV-1 Tat specific factor 1 | -0,659 | Nucleus | transcription regulator |
| HYAL3 | ENSG00000186792 | hyaluronidase 3 | -0,925 | Cytoplasm | enzyme |
| HYAL4 | ENSG00000106302 | hyaluronidase 4 | 1,257 | Plasma Membrane | enzyme |
| IARS2 | ENSG00000067704 | isoleucyl-tRNA synthetase 2, mitochondrial | -0,889 | Cytoplasm | enzyme |
| ICA1L | ENSG00000163596 | islet cell autoantigen 1 like | 0,844 | Cytoplasm | other |
| ICAM1 | ENSG00000090339 | intercellular adhesion molecule 1 | 1,880 | Plasma Membrane | transmembrane receptor |
| ICAM5 | ENSG00000105376 | intercellular adhesion molecule 5 | 0,691 | Plasma Membrane | other |
| ICOSLG/LOC102723996 | ENSG00000160223 | inducible T cell costimulator ligand | 0,689 | Plasma Membrane | other |
| ID3 | ENSG00000117318 | inhibitor of DNA binding 3, HLH protein | 1,959 | Nucleus | transcription regulator |
| ID4 | ENSG00000172201 | inhibitor of DNA binding 4, HLH protein | 1,101 | Nucleus | transcription regulator |
| IDH1 | ENSG00000138413 | isocitrate dehydrogenase (NADP(+)) 1 | -1,002 | Cytoplasm | enzyme |
| IDH2 | ENSG00000182054 | isocitrate dehydrogenase (NADP(+)) 2 | -0,895 | Cytoplasm | enzyme |
| IDI1 | ENSG00000067064 | isopentenyl-diphosphate delta isomerase 1 | -0,927 | Cytoplasm | enzyme |
| IDO1 | ENSG00000131203 | indoleamine 2,3-dioxygenase 1 | 6,353 | Cytoplasm | enzyme |
| IER2 | ENSG00000160888 | immediate early response 2 | 0,936 | Cytoplasm | transcription regulator |
| IER3 | ENSG00000237155 | immediate early response 3 | 0,895 | Cytoplasm | other |
| IER5 | ENSG00000162783 | immediate early response 5 | 1,325 | Nucleus | other |
| IFI16 | ENSG00000163565 | interferon gamma inducible protein 16 | 3,257 | Nucleus | transcription regulator |
| IFI27 | ENSG00000165949 | interferon alpha inducible protein 27 | 4,653 | Cytoplasm | other |
| IFI30 | ENSG00000216490 | IFI30 lysosomal thiol reductase | 1,390 | Cytoplasm | enzyme |
| IFI35 | ENSG00000068079 | interferon induced protein 35 | 3,400 | Nucleus | other |
| IFI44 | ENSG00000137965 | interferon induced protein 44 | 2,557 | Cytoplasm | other |
| IFI44L | ENSG00000137959 | interferon induced protein 44 like | 2,407 | Nucleus | other |
| IFI6 | ENSG00000126709 | interferon alpha inducible protein 6 | 2,437 | Cytoplasm | other |
| IFIH1 | ENSG00000115267 | interferon induced with helicase C domain 1 | 4,078 | Nucleus | enzyme |
| IFIT1 | ENSG00000185745 | interferon induced protein with tetratricopeptide repeats 1 | 4,498 | Cytoplasm | other |
| IFIT2 | ENSG00000119922 | interferon induced protein with tetratricopeptide repeats 2 | 6,995 | Cytoplasm | other |
| IFIT3 | ENSG00000119917 | interferon induced protein with tetratricopeptide repeats 3 | 5,727 | Cytoplasm | other |
| IFIT5 | ENSG00000152778 | interferon induced protein with tetratricopeptide repeats 5 | 3,271 | Plasma Membrane | other |
| IFITM1 | ENSG00000185885 | interferon induced transmembrane protein 1 | 4,623 | Plasma Membrane | transmembrane receptor |
| IFITM10 | ENSG00000244242 | interferon induced transmembrane protein 10 | -1,008 | Other | other |
| IFITM2 | ENSG00000185201 | interferon induced transmembrane protein 2 | 4,689 | Cytoplasm | other |
| IFITM3 | ENSG00000142089 | interferon induced transmembrane protein 3 | 3,130 | Plasma Membrane | other |
| IFNA1/IFNA13 | ENSG00000197919 | interferon alpha 1 | 0,983 | Extracellular Space | cytokine |
| IFNA7 | ENSG00000214042 | interferon alpha 7 | 3,413 | Extracellular Space | cytokine |
| IFNB1 | ENSG00000171855 | interferon beta 1 | 10,685 | Extracellular Space | cytokine |
| IFNL1 | ENSG00000182393 | interferon lambda 1 | 9,854 | Extracellular Space | cytokine |
| IFNL2 | ENSG00000183709 | interferon lambda 2 | 9,826 | Extracellular Space | other |
| IFNL3 | ENSG00000197110 | interferon lambda 3 | 9,370 | Extracellular Space | cytokine |
| IFNL3P1 | ENSG00000268510 | interferon lambda 3 pseudogene 1 | 4,599 | Other | other |
| IFNL4 | ENSG00000272395 | interferon lambda 4 (gene/pseudogene) | 4,559 | Other | cytokine |
| IFNLR1 | ENSG00000185436 | interferon lambda receptor 1 | 0,735 | Plasma Membrane | transmembrane receptor |
| IFRD1 | ENSG00000006652 | interferon related developmental regulator 1 | 0,753 | Nucleus | other |
| IFT122 | ENSG00000163913 | intraflagellar transport 122 | -0,979 | Nucleus | other |
| IFT140 | ENSG00000187535 | intraflagellar transport 140 | -0,664 | Extracellular Space | other |
| IFT27 | ENSG00000100360 | intraflagellar transport 27 | -0,705 | Extracellular Space | enzyme |
| IFT80 | ENSG00000068885 | intraflagellar transport 80 | -0,957 | Extracellular Space | other |
| IGF2 | ENSG00000167244 | insulin like growth factor 2 | 4,434 | Extracellular Space | growth factor |
| IGFBP4 | ENSG00000141753 | insulin like growth factor binding protein 4 | 1,820 | Extracellular Space | other |
| IGHMBP2 | ENSG00000132740 | immunoglobulin mu DNA binding protein 2 | 0,784 | Nucleus | enzyme |
| IGIP | ENSG00000182700 | IgA inducing protein | -0,788 | Other | other |
| IGSF9 | ENSG00000085552 | immunoglobulin superfamily member 9 | -0,940 | Plasma Membrane | other |
| IKBKE | ENSG00000263528 | inhibitor of nuclear factor kappa B kinase subunit epsilon | 1,196 | Cytoplasm | kinase |
| IKZF2 | ENSG00000030419 | IKAROS family zinc finger 2 | 0,957 | Nucleus | transcription regulator |
| IKZF3 | ENSG00000161405 | IKAROS family zinc finger 3 | 2,018 | Nucleus | transcription regulator |
| IKZF5 | ENSG00000095574 | IKAROS family zinc finger 5 | 0,622 | Nucleus | transcription regulator |
| IL10RA | ENSG00000110324 | interleukin 10 receptor subunit alpha | 2,580 | Plasma Membrane | transmembrane receptor |

| | | | | | |
|---|---|---|---|---|---|
| IL11 | ENSG00000095752 | interleukin 11 | 1,679 | Extracellular Space | cytokine |
| IL12A | ENSG00000168811 | interleukin 12A | 3,003 | Extracellular Space | cytokine |
| IL15 | ENSG00000164136 | interleukin 15 | 1,416 | Extracellular Space | cytokine |
| IL15RA | ENSG00000134470 | interleukin 15 receptor subunit alpha | 2,586 | Plasma Membrane | transmembrane receptor |
| IL17RD | ENSG00000144730 | interleukin 17 receptor D | -0,802 | Cytoplasm | other |
| IL18R1 | ENSG00000115604 | interleukin 18 receptor 1 | 1,497 | Plasma Membrane | transmembrane receptor |
| IL1A | ENSG00000115008 | interleukin 1 alpha | 4,404 | Extracellular Space | cytokine |
| IL1B | ENSG00000125538 | interleukin 1 beta | 1,834 | Extracellular Space | cytokine |
| IL1RN | ENSG00000136689 | interleukin 1 receptor antagonist | 1,111 | Extracellular Space | cytokine |
| IL22RA1 | ENSG00000142677 | interleukin 22 receptor subunit alpha 1 | 1,978 | Plasma Membrane | transmembrane receptor |
| IL23A | ENSG00000110944 | interleukin 23 subunit alpha | 1,474 | Extracellular Space | cytokine |
| IL2RG | ENSG00000147168 | interleukin 2 receptor subunit gamma | 1,979 | Plasma Membrane | transmembrane receptor |
| IL32 | ENSG00000008517 | interleukin 32 | 2,239 | Extracellular Space | cytokine |
| IL3RA | ENSG00000185291 | interleukin 3 receptor subunit alpha | 1,114 | Plasma Membrane | transmembrane receptor |
| IL4I1 | ENSG00000104951 | interleukin 4 induced 1 | 3,155 | Cytoplasm | enzyme |
| IL6 | ENSG00000136244 | interleukin 6 | 6,103 | Extracellular Space | cytokine |
| IL7 | ENSG00000104432 | interleukin 7 | 1,817 | Extracellular Space | cytokine |
| IL7R | ENSG00000168685 | interleukin 7 receptor | 2,787 | Plasma Membrane | transmembrane receptor |
| ILVBL | ENSG00000105135 | ilvB acetolactate synthase like | -0,628 | Cytoplasm | enzyme |
| IMPA1 | ENSG00000133731 | inositol monophosphatase 1 | 1,112 | Cytoplasm | phosphatase |
| IMPA2 | ENSG00000141401 | inositol monophosphatase 2 | -0,761 | Cytoplasm | phosphatase |
| INA | ENSG00000148798 | internexin neuronal intermediate filament protein alpha | 0,715 | Cytoplasm | other |
| ING3 | ENSG00000071243 | inhibitor of growth family member 3 | 0,865 | Nucleus | other |
| INHBA | ENSG00000122641 | inhibin subunit beta A | 2,765 | Extracellular Space | growth factor |
| INHBE | ENSG00000139269 | inhibin subunit beta E | 0,843 | Extracellular Space | growth factor |
| INO80 | ENSG00000128908 | INO80 complex ATPase subunit | 0,687 | Nucleus | enzyme |
| INO80D | ENSG00000114933 | INO80 complex subunit D | 1,247 | Nucleus | other |
| INSIG1 | ENSG00000186480 | insulin induced gene 1 | -0,867 | Cytoplasm | other |
| INTS6 | ENSG00000102786 | integrator complex subunit 6 | 1,148 | Nucleus | enzyme |
| IPPK | ENSG00000127080 | inositol-pentakisphosphate 2-kinase | 0,835 | Cytoplasm | kinase |
| IQCG | ENSG00000114473 | IQ motif containing G | -0,611 | Cytoplasm | other |
| IQCK | ENSG00000174628 | IQ motif containing K | -1,148 | Other | other |
| IQGAP3 | ENSG00000183856 | IQ motif containing GTPase activating protein 3 | -1,078 | Plasma Membrane | other |
| IRAK1 | ENSG00000184216 | interleukin 1 receptor associated kinase 1 | -0,601 | Plasma Membrane | kinase |
| IRAK2 | ENSG00000134070 | interleukin 1 receptor associated kinase 2 | 1,573 | Plasma Membrane | kinase |
| IRF1 | ENSG00000125347 | interferon regulatory factor 1 | 3,822 | Nucleus | transcription regulator |
| IRF1-AS1 | ENSG00000197536 | IRF1 antisense RNA 1 | 2,573 | Other | other |
| IRF2 | ENSG00000168310 | interferon regulatory factor 2 | 1,273 | Nucleus | transcription regulator |
| IRF6 | ENSG00000117595 | interferon regulatory factor 6 | 0,784 | Nucleus | transcription regulator |
| IRF7 | ENSG00000185507 | interferon regulatory factor 7 | 3,441 | Nucleus | transcription regulator |
| IRF8 | ENSG00000140968 | interferon regulatory factor 8 | 1,355 | Nucleus | transcription regulator |
| IRF9 | ENSG00000213928 | interferon regulatory factor 9 | 1,363 | Nucleus | transcription regulator |
| IRGQ | ENSG00000167378 | immunity related GTPase Q | 0,936 | Other | other |
| IRS2 | ENSG00000185950 | insulin receptor substrate 2 | 0,883 | Cytoplasm | enzyme |
| IRX3 | ENSG00000177508 | iroquois homeobox 3 | 1,020 | Nucleus | transcription regulator |
| IRX5 | ENSG00000176842 | iroquois homeobox 5 | 1,202 | Nucleus | transcription regulator |
| ISG15 | ENSG00000187608 | ISG15 ubiquitin like modifier | 3,500 | Extracellular Space | other |
| ISG20 | ENSG00000172183 | interferon stimulated exonuclease gene 20 | 4,780 | Nucleus | enzyme |
| ITGA5 | ENSG00000161638 | integrin subunit alpha 5 | 0,803 | Plasma Membrane | transmembrane receptor |
| ITGAE | ENSG00000083457 | integrin subunit alpha E | -1,368 | Plasma Membrane | other |
| ITGAM | ENSG00000169896 | integrin subunit alpha M | 1,493 | Plasma Membrane | transmembrane receptor |
| ITGAV | ENSG00000138448 | integrin subunit alpha V | -0,899 | Plasma Membrane | transmembrane receptor |
| ITGB1 | ENSG00000150093 | integrin subunit beta 1 | -0,650 | Plasma Membrane | transmembrane receptor |
| ITGB4 | ENSG00000132470 | integrin subunit beta 4 | -0,678 | Plasma Membrane | transmembrane receptor |
| ITGB5 | ENSG00000082781 | integrin subunit beta 5 | -0,833 | Plasma Membrane | other |
| ITGB6 | ENSG00000115221 | integrin subunit beta 6 | -1,063 | Plasma Membrane | other |
| ITIH2 | ENSG00000151655 | inter-alpha-trypsin inhibitor heavy chain 2 | -0,882 | Extracellular Space | other |
| ITIH5 | ENSG00000123243 | inter-alpha-trypsin inhibitor heavy chain 5 | -0,685 | Plasma Membrane | other |
| ITM2A | ENSG00000078596 | integral membrane protein 2A | -1,472 | Plasma Membrane | other |
| ITPKC | ENSG00000086544 | inositol-trisphosphate 3-kinase C | 1,121 | Cytoplasm | kinase |
| ITPRIPL2 | ENSG00000205730 | ITPRIP like 2 | 0,683 | Other | other |
| JADE2 | ENSG00000043143 | jade family PHD finger 2 | 0,786 | Nucleus | enzyme |
| JAK2 | ENSG00000096968 | Janus kinase 2 | 2,668 | Cytoplasm | kinase |
| JAKMIP3 | ENSG00000188385 | Janus kinase and microtubule interacting protein 3 | 0,870 | Other | other |
| JAZF1 | ENSG00000153814 | JAZF zinc finger 1 | 1,740 | Nucleus | transcription regulator |
| JDP2 | ENSG00000140044 | Jun dimerization protein 2 | 0,975 | Nucleus | transcription regulator |
| JMJD1C | ENSG00000171988 | jumonji domain containing 1C | 0,808 | Nucleus | enzyme |
| JUN | ENSG00000177606 | Jun proto-oncogene, AP-1 transcription factor subunit | 2,950 | Nucleus | transcription regulator |
| JUNB | ENSG00000171223 | JunB proto-oncogene, AP-1 transcription factor subunit | 1,231 | Nucleus | transcription regulator |
| KAAG1 | ENSG00000146049 | kidney associated antigen 1 | 0,853 | Extracellular Space | other |
| KALRN | ENSG00000160145 | kalirin RhoGEF kinase | 1,015 | Cytoplasm | kinase |
| KANSL1 | ENSG00000120071 | KAT8 regulatory NSL complex subunit 1 | 0,834 | Nucleus | other |
| KAT6A | ENSG00000083168 | lysine acetyltransferase 6A | 0,632 | Nucleus | enzyme |
| KBTBD3 | ENSG00000182359 | kelch repeat and BTB domain containing 3 | -0,771 | Extracellular Space | other |
| KBTBD8 | ENSG00000163376 | kelch repeat and BTB domain containing 8 | 1,075 | Cytoplasm | other |
| KCNC4 | ENSG00000116396 | potassium voltage-gated channel subfamily C member 4 | -0,927 | Plasma Membrane | ion channel |
| KCND1 | ENSG00000102057 | potassium voltage-gated channel subfamily D member 1 | -0,892 | Plasma Membrane | ion channel |
| KCNE3 | ENSG00000175538 | potassium voltage-gated channel subfamily E regulatory subunit 3 | -1,332 | Plasma Membrane | ion channel |
| KCNH7 | ENSG00000184611 | potassium voltage-gated channel subfamily H member 7 | 0,612 | Plasma Membrane | ion channel |
| KCNH8 | ENSG00000183960 | potassium voltage-gated channel subfamily H member 8 | 0,637 | Plasma Membrane | ion channel |
| KCNJ15 | ENSG00000157551 | potassium inwardly rectifying channel subfamily J member 15 | 1,068 | Plasma Membrane | ion channel |
| KCNMB4 | ENSG00000135643 | potassium calcium-activated channel subfamily M regulatory beta subunit 4 | -0,829 | Plasma Membrane | ion channel |
| KCNN1 | ENSG00000105642 | potassium calcium-activated channel subfamily N member 1 | 2,656 | Plasma Membrane | ion channel |
| KCNQ1 | ENSG00000053918 | potassium voltage-gated channel subfamily Q member 1 | -0,840 | Plasma Membrane | ion channel |
| KCNQ3 | ENSG00000184156 | potassium voltage-gated channel subfamily Q member 3 | -1,062 | Plasma Membrane | ion channel |
| KCNT2 | ENSG00000162687 | potassium sodium-activated channel subfamily T member 2 | 1,498 | Plasma Membrane | ion channel |
| KCNV1 | ENSG00000164794 | potassium voltage-gated channel modifier subfamily V member 1 | 2,476 | Plasma Membrane | ion channel |
| KCTD12 | ENSG00000178695 | potassium channel tetramerization domain containing 12 | -0,867 | Plasma Membrane | ion channel |
| KDM2A | ENSG00000173120 | lysine demethylase 2A | 0,915 | Nucleus | enzyme |
| KDM5B | ENSG00000117139 | lysine demethylase 5B | 0,640 | Nucleus | transcription regulator |
| KDM6A | ENSG00000147050 | lysine demethylase 6A | 2,162 | Nucleus | enzyme |
| KDM6B | ENSG00000132510 | lysine demethylase 6B | 0,735 | Extracellular Space | enzyme |
| KDM7A | ENSG00000006459 | lysine demethylase 7A | 0,862 | Nucleus | enzyme |
| KEAP1 | ENSG00000079999 | kelch like ECH associated protein 1 | 0,680 | Cytoplasm | transcription regulator |
| KHK | ENSG00000138030 | ketohexokinase | -0,843 | Cytoplasm | kinase |
| KIAA0040 | ENSG00000235750 | KIAA0040 | 1,268 | Other | other |
| KIAA0100 | ENSG00000007202 | KIAA0100 | -0,728 | Extracellular Space | other |
| KIAA0930 | ENSG00000100364 | KIAA0930 | -0,753 | Other | other |
| KIAA1217 | ENSG00000120549 | KIAA1217 | 1,000 | Cytoplasm | other |

| | | | | | |
|---|---|---|---|---|---|
| KIAA1549L | ENSG00000110427 | KIAA1549 like | -0,644 | Cytoplasm | other |
| KIAA1841 | ENSG00000162929 | KIAA1841 | -0,828 | Other | other |
| KIF12 | ENSG00000136883 | kinesin family member 12 | -1,281 | Cytoplasm | other |
| KIF14 | ENSG00000118193 | kinesin family member 14 | -0,726 | Cytoplasm | enzyme |
| KIF1C | ENSG00000129250 | kinesin family member 1C | -1,002 | Cytoplasm | other |
| KIF20A | ENSG00000112984 | kinesin family member 20A | -1,730 | Cytoplasm | other |
| KIF23 | ENSG00000137807 | kinesin family member 23 | -0,671 | Cytoplasm | other |
| KIF3C | ENSG00000084731 | kinesin family member 3C | -0,775 | Cytoplasm | other |
| KIF4A | ENSG00000090889 | kinesin family member 4A | -1,158 | Nucleus | other |
| KIFC2 | ENSG00000167702 | kinesin family member C2 | -0,629 | Cytoplasm | other |
| KIN | ENSG00000151657 | Kin17 DNA and RNA binding protein | 0,616 | Nucleus | other |
| KITLG | ENSG00000049130 | KIT ligand | -0,632 | Extracellular Space | growth factor |
| KLC4 | ENSG00000137171 | kinesin light chain 4 | -0,989 | Other | other |
| KLF10 | ENSG00000155090 | Kruppel like factor 10 | 0,679 | Nucleus | transcription regulator |
| KLF2 | ENSG00000127528 | Kruppel like factor 2 | 1,621 | Nucleus | transcription regulator |
| KLF4 | ENSG00000136826 | Kruppel like factor 4 | 5,055 | Nucleus | transcription regulator |
| KLF6 | ENSG00000067082 | Kruppel like factor 6 | 1,986 | Nucleus | transcription regulator |
| KLHDC2 | ENSG00000165516 | kelch domain containing 2 | -0,751 | Cytoplasm | other |
| KLHDC7A | ENSG00000179023 | kelch domain containing 7A | -0,823 | Other | other |
| KLHDC7B | ENSG00000130487 | kelch domain containing 7B | 1,486 | Other | other |
| KLHDC8B | ENSG00000185909 | kelch domain containing 8B | -0,728 | Cytoplasm | other |
| KLHL15 | ENSG00000174010 | kelch like family member 15 | 0,652 | Nucleus | other |
| KLHL20 | ENSG00000076321 | kelch like family member 20 | 0,869 | Plasma Membrane | enzyme |
| KLHL26 | ENSG00000167487 | kelch like family member 26 | 0,883 | Other | other |
| KLHL29 | ENSG00000119771 | kelch like family member 29 | 0,987 | Other | other |
| KLHL3 | ENSG00000146021 | kelch like family member 3 | 0,946 | Cytoplasm | other |
| KLK6 | ENSG00000167755 | kallikrein related peptidase 6 | -0,849 | Extracellular Space | peptidase |
| KLK7 | ENSG00000169035 | kallikrein related peptidase 7 | -1,420 | Extracellular Space | peptidase |
| KLRG2 | ENSG00000188883 | killer cell lectin like receptor G2 | 0,718 | Other | other |
| KMO | ENSG00000117009 | kynurenine 3-monooxygenase | 1,935 | Cytoplasm | enzyme |
| KMT2C | ENSG00000055609 | lysine methyltransferase 2C | 1,045 | Nucleus | transcription regulator |
| KMT2E | ENSG00000005483 | lysine methyltransferase 2E (inactive) | 1,065 | Nucleus | enzyme |
| KPNA2 | ENSG00000182481 | karyopherin subunit alpha 2 | -0,643 | Nucleus | transporter |
| KPNA5 | ENSG00000196911 | karyopherin subunit alpha 5 | 0,867 | Cytoplasm | other |
| KRT10 | ENSG00000186395 | keratin 10 | -0,710 | Cytoplasm | other |
| KRT17 | ENSG00000128422 | keratin 17 | 1,267 | Cytoplasm | other |
| KRT17P2 | ENSG00000186831 | keratin 17 pseudogene 2 | 1,873 | Other | other |
| KRT6B | ENSG00000185479 | keratin 6B | 1,086 | Cytoplasm | other |
| KSR1 | ENSG00000141068 | kinase suppressor of ras 1 | 1,597 | Cytoplasm | kinase |
| KTI12 | ENSG00000198841 | KTI12 chromatin associated homolog | 0,675 | Other | other |
| KYNU | ENSG00000115919 | kynureninase | 0,743 | Cytoplasm | enzyme |
| LA16C_431H66 | ENSG00000261732 | | 0,677 | Other | other |
| LACTB2 | ENSG00000147592 | lactamase beta 2 | -0,702 | Cytoplasm | enzyme |
| LAD1 | ENSG00000159166 | ladinin 1 | -0,656 | Extracellular Space | other |
| LAGE3 | ENSG00000196976 | L antigen family member 3 | -0,995 | Nucleus | other |
| LAMA2 | ENSG00000196569 | laminin subunit alpha 2 | 1,130 | Extracellular Space | other |
| LAMP3 | ENSG00000078081 | lysosomal associated membrane protein 3 | 4,754 | Plasma Membrane | other |
| LAMTOR2 | ENSG00000116586 | late endosomal/lysosomal adaptor, MAPK and MTOR activator 2 | -0,612 | Cytoplasm | other |
| LAMTOR4 | ENSG00000188186 | late endosomal/lysosomal adaptor, MAPK and MTOR activator 4 | -0,898 | Cytoplasm | other |
| LAMTOR5 | ENSG00000134248 | late endosomal/lysosomal adaptor, MAPK and MTOR activator 5 | -0,660 | Cytoplasm | other |
| LANCL1 | ENSG00000115365 | LanC like 1 | -0,653 | Plasma Membrane | enzyme |
| LAP3 | ENSG00000002549 | leucine aminopeptidase 3 | 2,309 | Cytoplasm | peptidase |
| LAPTM4B | ENSG00000104341 | lysosomal protein transmembrane 4 beta | -0,711 | Cytoplasm | other |
| LASP1 | ENSG00000002834 | LIM and SH3 protein 1 | -0,845 | Cytoplasm | transporter |
| LAT2 | ENSG00000086730 | linker for activation of T cells family member 2 | 0,842 | Plasma Membrane | other |
| LATS1 | ENSG00000131023 | large tumor suppressor kinase 1 | 0,608 | Nucleus | kinase |
| LATS2 | ENSG00000150457 | large tumor suppressor kinase 2 | 0,937 | Nucleus | kinase |
| LBR | ENSG00000143815 | lamin B receptor | -0,653 | Nucleus | enzyme |
| LCORL | ENSG00000178177 | ligand dependent nuclear receptor corepressor like | 0,727 | Nucleus | transcription regulator |
| LDC1P | ENSG00000260386 | leucine decarboxylase 1, pseudogene | 1,819 | Other | other |
| LDHA | ENSG00000134333 | lactate dehydrogenase A | -0,798 | Cytoplasm | enzyme |
| LDHAL6B | ENSG00000171989 | lactate dehydrogenase A like 6B | 1,663 | Cytoplasm | enzyme |
| LEAP2 | ENSG00000164406 | liver enriched antimicrobial peptide 2 | 0,780 | Extracellular Space | other |
| LEKR1 | ENSG00000197980 | leucine, glutamate and lysine rich 1 | 1,126 | Other | other |
| LEMD1 | ENSG00000186007 | LEM domain containing 1 | -1,405 | Other | other |
| LEPR | ENSG00000116678 | leptin receptor | -0,986 | Plasma Membrane | transmembrane receptor |
| LEPROT | ENSG00000213625 | leptin receptor overlapping transcript | -0,782 | Plasma Membrane | other |
| LGALS3BP | ENSG00000108679 | galectin 3 binding protein | 1,148 | Plasma Membrane | transmembrane receptor |
| LGALS4 | ENSG00000171747 | galectin 4 | -1,286 | Extracellular Space | other |
| LGALS8 | ENSG00000116977 | galectin 8 | 0,635 | Extracellular Space | other |
| LGALS9 | ENSG00000168961 | galectin 9 | 3,075 | Plasma Membrane | other |
| LGMN | ENSG00000100600 | legumain | 1,509 | Cytoplasm | peptidase |
| LHPP | ENSG00000107902 | phospholysine phosphohistidine inorganic pyrophosphate phosphatase | -1,058 | Cytoplasm | phosphatase |
| LHX9 | ENSG00000143355 | LIM homeobox 9 | 0,929 | Nucleus | transcription regulator |
| LIF | ENSG00000128342 | LIF interleukin 6 family cytokine | 1,108 | Extracellular Space | cytokine |
| LIFR | ENSG00000113594 | LIF receptor subunit alpha | 1,446 | Plasma Membrane | transmembrane receptor |
| LIG1 | ENSG00000105486 | DNA ligase 1 | -0,624 | Nucleus | enzyme |
| LIG4 | ENSG00000174405 | DNA ligase 4 | 0,744 | Nucleus | enzyme |
| LIMCH1 | ENSG00000064042 | LIM and calponin homology domains 1 | 0,670 | Cytoplasm | other |
| LIMD2 | ENSG00000136490 | LIM domain containing 2 | 0,777 | Other | other |
| LIN37 | ENSG00000267796 | lin-37 DREAM MuvB core complex component | 0,667 | Nucleus | other |
| LIN54 | ENSG00000189308 | lin-54 DREAM MuvB core complex component | 0,854 | Nucleus | other |
| LINC00923 | ENSG00000251209 | long intergenic non-protein coding RNA 923 | 0,675 | Other | other |
| LINC01145 | ENSG00000272419 | long intergenic non-protein coding RNA 1145 | 0,699 | Other | other |
| LINC01949 | ENSG00000233828 | long intergenic non-protein coding RNA 1949 | 0,666 | Other | other |
| LINC02656 | ENSG00000212743 | long intergenic non-protein coding RNA 2656 | -0,620 | Other | other |
| LIPA | ENSG00000107798 | lipase A, lysosomal acid type | 0,905 | Cytoplasm | enzyme |
| LIPH | ENSG00000163898 | lipase H | 0,631 | Extracellular Space | enzyme |
| LMBRD2 | ENSG00000164187 | LMBR1 domain containing 2 | -0,758 | Other | other |
| LMCD1 | ENSG00000071282 | LIM and cysteine rich domains 1 | -0,978 | Cytoplasm | transcription regulator |
| LMNB1 | ENSG00000113368 | lamin B1 | -0,708 | Nucleus | other |
| LMO2 | ENSG00000135363 | LIM domain only 2 | 2,915 | Nucleus | transcription regulator |
| LMO4 | ENSG00000143013 | LIM domain only 4 | -0,998 | Nucleus | transcription regulator |
| LNP1 | ENSG00000206535 | leukemia NUP98 fusion partner 1 | -0,989 | Other | other |
| LNPEP | ENSG00000113441 | leucyl and cystinyl aminopeptidase | 0,850 | Cytoplasm | peptidase |
| LNPK | ENSG00000144320 | lunapark, ER junction formation factor | -0,763 | Cytoplasm | other |
| LNX1 | ENSG00000072201 | ligand of numb-protein X 1 | 0,792 | Cytoplasm | enzyme |
| LOC100419170 | ENSG00000250771 | toll like receptor 2 pseudogene | 0,775 | Other | other |
| LOC105375724 | ENSG00000225885 | uncharacterized LOC105375724 | 1,276 | Other | other |

| | | | | | |
|---|---|---|---|---|---|
| LOC107987457 | ENSG00000236428 | | 2,672 | Other | other |
| LONRF3 | ENSG00000175556 | LON peptidase N-terminal domain and ring finger 3 | 0,697 | Other | other |
| LOXL2 | ENSG00000134013 | lysyl oxidase like 2 | -0,831 | Extracellular Space | enzyme |
| LOXL3 | ENSG00000115318 | lysyl oxidase like 3 | 0,964 | Extracellular Space | enzyme |
| LPAR1 | ENSG00000198121 | lysophosphatidic acid receptor 1 | 1,304 | Plasma Membrane | G-protein coupled receptor |
| LRAT | ENSG00000121207 | lecithin retinol acyltransferase | 0,678 | Cytoplasm | enzyme |
| LRCH3 | ENSG00000186001 | leucine rich repeats and calponin homology domain containing 3 | 0,713 | Cytoplasm | other |
| LRFN1 | ENSG00000128011 | leucine rich repeat and fibronectin type III domain containing 1 | 0,695 | Nucleus | other |
| LRIF1 | ENSG00000121931 | ligand dependent nuclear receptor interacting factor 1 | 0,640 | Nucleus | other |
| LRP1 | ENSG00000123384 | LDL receptor related protein 1 | -1,138 | Plasma Membrane | transmembrane receptor |
| LRP2 | ENSG00000081479 | LDL receptor related protein 2 | 2,002 | Plasma Membrane | transporter |
| LRRC20 | ENSG00000172731 | leucine rich repeat containing 20 | -1,162 | Other | other |
| LRRC23 | ENSG00000010626 | leucine rich repeat containing 23 | -0,832 | Plasma Membrane | other |
| LRRC75B | ENSG00000178026 | leucine rich repeat containing 75B | -0,984 | Other | other |
| LRRN3 | ENSG00000173114 | leucine rich repeat neuronal 3 | 2,035 | Extracellular Space | other |
| LSM12P1 | ENSG00000232024 | LSM12 pseudogene 1 | 0,608 | Other | other |
| LSM4 | ENSG00000130520 | LSM4 homolog, U6 small nuclear RNA and mRNA degradation associated | -0,668 | Nucleus | other |
| LSM7 | ENSG00000130332 | LSM7 homolog, U6 small nuclear RNA and mRNA degradation associated | -0,949 | Nucleus | other |
| LSMEM1 | ENSG00000181016 | leucine rich single-pass membrane protein 1 | 2,022 | Cytoplasm | other |
| LSS | ENSG00000160285 | lanosterol synthase | -1,075 | Cytoplasm | enzyme |
| LST1 | ENSG00000234514 | leukocyte specific transcript 1 | 2,339 | Plasma Membrane | other |
| LTB | ENSG00000227507 | lymphotoxin beta | 4,052 | Extracellular Space | cytokine |
| LTBP2 | ENSG00000119681 | latent transforming growth factor beta binding protein 2 | -0,692 | Extracellular Space | other |
| LTBP3 | ENSG00000168056 | latent transforming growth factor beta binding protein 3 | -0,687 | Extracellular Space | other |
| LTBP4 | ENSG00000090006 | latent transforming growth factor beta binding protein 4 | -0,753 | Extracellular Space | growth factor |
| LUC7L | ENSG00000007392 | LUC7 like | 0,689 | Nucleus | other |
| LXN | ENSG00000079257 | latexin | -0,612 | Cytoplasm | other |
| LY6E | ENSG00000160932 | lymphocyte antigen 6 family member E | 1,652 | Plasma Membrane | other |
| LYG1 | ENSG00000144214 | lysozyme g1 | 0,679 | Extracellular Space | other |
| LYN | ENSG00000254087 | LYN proto-oncogene, Src family tyrosine kinase | 1,004 | Cytoplasm | kinase |
| LYPD3 | ENSG00000124466 | LY6/PLAUR domain containing 3 | 0,819 | Plasma Membrane | other |
| LYPD5 | ENSG00000159871 | LY6/PLAUR domain containing 5 | 0,774 | Other | other |
| LYPD6B | ENSG00000150556 | LY6/PLAUR domain containing 6B | -1,087 | Other | other |
| LYPLAL1 | ENSG00000143353 | lysophospholipase like 1 | -0,725 | Cytoplasm | enzyme |
| LYSMD2 | ENSG00000140280 | LysM domain containing 2 | 1,007 | Other | other |
| LYST | ENSG00000143669 | lysosomal trafficking regulator | 0,607 | Cytoplasm | transporter |
| LYZ | ENSG00000090382 | lysozyme | -0,843 | Extracellular Space | enzyme |
| LZTS3 | ENSG00000088899 | leucine zipper tumor suppressor family member 3 | -0,912 | Cytoplasm | other |
| MACROD1 | ENSG00000133315 | mono-ADP ribosylhydrolase 1 | -0,703 | Cytoplasm | enzyme |
| MAD2L1BP | ENSG00000124688 | MAD2L1 binding protein | 0,852 | Nucleus | other |
| MAFA | ENSG00000182759 | MAF bZIP transcription factor A | 1,066 | Nucleus | transcription regulator |
| MAFF | ENSG00000185022 | MAF bZIP transcription factor F | 1,594 | Nucleus | transcription regulator |
| MAFG | ENSG00000197063 | MAF bZIP transcription factor G | 0,895 | Nucleus | transcription regulator |
| MAGED1 | ENSG00000179222 | MAGE family member D1 | -0,968 | Plasma Membrane | transcription regulator |
| MAGIX | ENSG00000269313 | MAGI family member, X-linked | -1,029 | Other | other |
| MAGT1 | ENSG00000102158 | magnesium transporter 1 | -0,625 | Plasma Membrane | enzyme |
| MALL | ENSG00000144063 | mal, T cell differentiation protein like | -0,736 | Plasma Membrane | other |
| MAN1A2 | ENSG00000198162 | mannosidase alpha class 1A member 2 | -0,943 | Cytoplasm | enzyme |
| MAN2A1 | ENSG00000112893 | mannosidase alpha class 2A member 1 | -0,705 | Cytoplasm | enzyme |
| MAOA | ENSG00000189221 | monoamine oxidase A | -0,972 | Cytoplasm | enzyme |
| MAOB | ENSG00000069535 | monoamine oxidase B | -1,524 | Cytoplasm | enzyme |
| MAP11 | ENSG00000146826 | microtubule associated protein 11 | 0,864 | Cytoplasm | other |
| MAP1LC3B | ENSG00000140941 | microtubule associated protein 1 light chain 3 beta | 0,628 | Cytoplasm | other |
| MAP1S | ENSG00000130479 | microtubule associated protein 1S | 0,653 | Cytoplasm | enzyme |
| MAP2 | ENSG00000078018 | microtubule associated protein 2 | 1,239 | Plasma Membrane | other |
| MAP2K6 | ENSG00000108984 | mitogen-activated protein kinase kinase 6 | 0,914 | Cytoplasm | kinase |
| MAP3K10 | ENSG00000130758 | mitogen-activated protein kinase kinase kinase 10 | 0,731 | Cytoplasm | kinase |
| MAP3K20 | ENSG00000091436 | mitogen-activated protein kinase kinase kinase 20 | -0,609 | Cytoplasm | kinase |
| MAP3K8 | ENSG00000107968 | mitogen-activated protein kinase kinase kinase 8 | 3,316 | Cytoplasm | kinase |
| MAP4 | ENSG00000047849 | microtubule associated protein 4 | -0,710 | Cytoplasm | other |
| MAP4K4 | ENSG00000071054 | mitogen-activated protein kinase kinase kinase kinase 4 | 0,961 | Cytoplasm | kinase |
| MAPK3 | ENSG00000102882 | mitogen-activated protein kinase 3 | -0,961 | Cytoplasm | kinase |
| MAPK8IP2 | ENSG00000008735 | mitogen-activated protein kinase 8 interacting protein 2 | 1,180 | Cytoplasm | other |
| MAPKAP1 | ENSG00000119487 | MAPK associated protein 1 | -0,653 | Cytoplasm | other |
| MARCHF5 | ENSG00000198060 | membrane associated ring-CH-type finger 5 | 1,080 | Cytoplasm | enzyme |
| MARF1 | ENSG00000166783 | meiosis regulator and mRNA stability factor 1 | -0,673 | Cytoplasm | other |
| MAST4 | ENSG00000069020 | microtubule associated serine/threonine kinase family member 4 | 0,894 | Other | kinase |
| MASTL | ENSG00000120539 | microtubule associated serine/threonine kinase like | 1,312 | Cytoplasm | kinase |
| MATN2 | ENSG00000132561 | matrilin 2 | -0,838 | Extracellular Space | other |
| MAU2 | ENSG00000129933 | MAU2 sister chromatid cohesion factor | 0,672 | Nucleus | other |
| MAX | ENSG00000125952 | MYC associated factor X | 0,639 | Nucleus | transcription regulator |
| MAZ | ENSG00000103495 | MYC associated zinc finger protein | -0,704 | Nucleus | transcription regulator |
| MB21D2 | ENSG00000180611 | Mab-21 domain containing 2 | 2,526 | Other | other |
| MBD1 | ENSG00000141644 | methyl-CpG binding domain protein 1 | 0,932 | Nucleus | transcription regulator |
| MBNL3 | ENSG00000076770 | muscleblind like splicing regulator 3 | -0,913 | Nucleus | other |
| MBOAT2 | ENSG00000143797 | membrane bound O-acyltransferase domain containing 2 | -1,057 | Cytoplasm | enzyme |
| MCF2L2 | ENSG00000053524 | MCF.2 cell line derived transforming sequence-like 2 | 0,654 | Other | other |
| MCL1 | ENSG00000143384 | MCL1 apoptosis regulator, BCL2 family member | 1,152 | Cytoplasm | transporter |
| MCM6 | ENSG00000076003 | minichromosome maintenance complex component 6 | -0,882 | Nucleus | enzyme |
| MCPH1 | ENSG00000147316 | microcephalin 1 | 0,673 | Nucleus | other |
| MCTP1 | ENSG00000175471 | multiple C2 and transmembrane domain containing 1 | 0,854 | Cytoplasm | other |
| MDFI | ENSG00000112559 | MyoD family inhibitor | 0,665 | Cytoplasm | other |
| MDFIC | ENSG00000135272 | MyoD family inhibitor domain containing | -0,679 | Nucleus | other |
| MDGA1 | ENSG00000112139 | MAM domain containing glycosylphosphatidylinositol anchor 1 | 0,997 | Plasma Membrane | other |
| MDH1 | ENSG00000014641 | malate dehydrogenase 1 | -0,853 | Cytoplasm | enzyme |
| MDK | ENSG00000110492 | midkine | 1,354 | Extracellular Space | growth factor |
| MED1 | ENSG00000125686 | mediator complex subunit 1 | 0,667 | Nucleus | transcription regulator |
| MED12L | ENSG00000144893 | mediator complex subunit 12L | 1,781 | Extracellular Space | other |
| MED13 | ENSG00000108510 | mediator complex subunit 13 | 0,653 | Nucleus | transcription regulator |
| MED22 | ENSG00000148297 | mediator complex subunit 22 | -0,614 | Cytoplasm | other |
| MED26 | ENSG00000105085 | mediator complex subunit 26 | 1,008 | Nucleus | transcription regulator |
| MEF2C | ENSG00000081189 | myocyte enhancer factor 2C | 0,997 | Nucleus | transcription regulator |
| MEF2D | ENSG00000116604 | myocyte enhancer factor 2D | 0,642 | Nucleus | transcription regulator |
| MEFV | ENSG00000103313 | MEFV innate immuity regulator, pyrin | 0,784 | Nucleus | other |
| MEGF6 | ENSG00000162591 | multiple EGF like domains 6 | -0,751 | Cytoplasm | other |
| MEGF8 | ENSG00000105429 | multiple EGF like domains 8 | -0,902 | Extracellular Space | other |
| MEIS1 | ENSG00000143995 | Meis homeobox 1 | 0,905 | Nucleus | transcription regulator |
| MEMO1 | ENSG00000162959 | mediator of cell motility 1 | -0,656 | Cytoplasm | other |
| METRN | ENSG00000103260 | meteorin, glial cell differentiation regulator | -0,690 | Extracellular Space | other |

| | | | | | |
|---|---|---|---|---|---|
| METRNL | ENSG00000176845 | meteorin like, glial cell differentiation regulator | 1,173 | Cytoplasm | other |
| METTL7A | ENSG00000185432 | methyltransferase like 7A | -2,286 | Cytoplasm | other |
| METTL7B | ENSG00000170439 | methyltransferase like 7B | -1,267 | Other | enzyme |
| MFSD12 | ENSG00000161091 | major facilitator superfamily domain containing 12 | 1,297 | Cytoplasm | other |
| MFSD14A | ENSG00000156875 | major facilitator superfamily domain containing 14A | 1,663 | Other | transporter |
| MFSD2A | ENSG00000168389 | major facilitator superfamily domain containing 2A | 1,432 | Plasma Membrane | transporter |
| MGAM2 | ENSG00000257743 | maltase-glucoamylase 2 (putative) | -1,043 | Other | other |
| MGAT3 | ENSG00000128268 | beta-1,4-mannosyl-glycoprotein 4-beta-N-acetylglucosaminyltransferase | -0,627 | Cytoplasm | enzyme |
| MGLL | ENSG00000074416 | monoglyceride lipase | -0,891 | Plasma Membrane | enzyme |
| MGST2 | ENSG00000085871 | microsomal glutathione S-transferase 2 | -0,891 | Cytoplasm | enzyme |
| MGST3 | ENSG00000143198 | microsomal glutathione S-transferase 3 | -0,629 | Cytoplasm | enzyme |
| MIB1 | ENSG00000101752 | mindbomb E3 ubiquitin protein ligase 1 | -0,686 | Cytoplasm | enzyme |
| MICB | ENSG00000231372 | MHC class I polypeptide-related sequence B | 2,474 | Plasma Membrane | transmembrane receptor |
| MID2 | ENSG00000080561 | midline 2 | 0,605 | Cytoplasm | other |
| MIER1 | ENSG00000198160 | MIER1 transcriptional regulator | 0,780 | Nucleus | transcription regulator |
| MIER3 | ENSG00000155545 | MIER family member 3 | 0,778 | Nucleus | transcription regulator |
| MINDY4 | ENSG00000106125 | MINDY lysine 48 deubiquitinase 4 | -0,672 | Other | other |
| MIPEP | ENSG00000027001 | mitochondrial intermediate peptidase | -0,973 | Cytoplasm | peptidase |
| MIS12 | ENSG00000167842 | MIS12 kinetochore complex component | 0,638 | Nucleus | other |
| MKI67 | ENSG00000148773 | marker of proliferation Ki-67 | -0,705 | Nucleus | other |
| MKKS | ENSG00000125863 | McKusick-Kaufman syndrome | -0,675 | Cytoplasm | other |
| MLF1 | ENSG00000178053 | myeloid leukemia factor 1 | -0,783 | Nucleus | other |
| MLKL | ENSG00000168404 | mixed lineage kinase domain like pseudokinase | 1,483 | Cytoplasm | kinase |
| MLPH | ENSG00000115648 | melanophilin | -1,060 | Cytoplasm | other |
| MMAB | ENSG00000139428 | metabolism of cobalamin associated B | -1,265 | Cytoplasm | enzyme |
| MMD2 | ENSG00000136297 | monocyte to macrophage differentiation associated 2 | 2,280 | Other | kinase |
| MME | ENSG00000196549 | membrane metalloendopeptidase | -0,708 | Plasma Membrane | peptidase |
| MMP1 | ENSG00000196611 | matrix metallopeptidase 1 | 1,062 | Extracellular Space | peptidase |
| MMP25 | ENSG00000008516 | matrix metallopeptidase 25 | 0,797 | Plasma Membrane | peptidase |
| MMP7 | ENSG00000137673 | matrix metallopeptidase 7 | -0,927 | Extracellular Space | peptidase |
| MMUT | ENSG00000146085 | methylmalonyl-CoA mutase | -0,884 | Cytoplasm | enzyme |
| MNS1 | ENSG00000138587 | meiosis specific nuclear structural 1 | -1,081 | Cytoplasm | other |
| MOB3B | ENSG00000120162 | MOB kinase activator 3B | -0,739 | Other | other |
| MOB3C | ENSG00000142961 | MOB kinase activator 3C | 1,223 | Other | other |
| MOBP | ENSG00000168314 | myelin associated oligodendrocyte basic protein | 1,380 | Cytoplasm | other |
| MOCOS | ENSG00000075643 | molybdenum cofactor sulfurase | 0,770 | Cytoplasm | enzyme |
| MOGAT1 | ENSG00000124003 | monoacylglycerol O-acyltransferase 1 | -1,585 | Cytoplasm | enzyme |
| MORC2 | ENSG00000133422 | MORC family CW-type zinc finger 2 | 0,658 | Nucleus | enzyme |
| MORC3 | ENSG00000159256 | MORC family CW-type zinc finger 3 | 0,686 | Nucleus | other |
| MORN2 | ENSG00000188010 | MORN repeat containing 2 | -1,231 | Other | other |
| MOSPD3 | ENSG00000106330 | motile sperm domain containing 3 | -1,152 | Other | other |
| MOV10 | ENSG00000155363 | Mov10 RISC complex RNA helicase | 1,639 | Nucleus | enzyme |
| MOXD1 | ENSG00000079931 | monooxygenase DBH like 1 | -0,877 | Cytoplasm | enzyme |
| MPC2 | ENSG00000143158 | mitochondrial pyruvate carrier 2 | -0,916 | Plasma Membrane | other |
| MPI | ENSG00000178802 | mannose phosphate isomerase | -0,691 | Cytoplasm | enzyme |
| MPND | ENSG00000008382 | MPN domain containing | -1,019 | Other | other |
| MPP7 | ENSG00000150054 | membrane palmitoylated protein 7 | -1,032 | Plasma Membrane | other |
| MPV17 | ENSG00000115204 | mitochondrial inner membrane protein MPV17 | -0,820 | Cytoplasm | ion channel |
| MPV17L | ENSG00000156968 | MPV17 mitochondrial inner membrane protein like | -0,631 | Cytoplasm | other |
| MRC2 | ENSG00000011028 | mannose receptor C type 2 | -0,696 | Plasma Membrane | transmembrane receptor |
| MRI1 | ENSG00000037757 | methylthioribose-1-phosphate isomerase 1 | -0,640 | Cytoplasm | translation regulator |
| MRNIP | ENSG00000161010 | MRN complex interacting protein | 0,677 | Nucleus | other |
| MRPL24 | ENSG00000143314 | mitochondrial ribosomal protein L24 | -0,704 | Cytoplasm | other |
| MRPL33 | ENSG00000243147 | mitochondrial ribosomal protein L33 | -0,641 | Cytoplasm | other |
| MRPL43 | ENSG00000055950 | mitochondrial ribosomal protein L43 | -0,619 | Cytoplasm | translation regulator |
| MRPL44 | ENSG00000135900 | mitochondrial ribosomal protein L44 | 0,604 | Cytoplasm | enzyme |
| MSANTD3-TMEFF1 | ENSG00000251349 | MSANTD3-TMEFF1 readthrough | 2,054 | Other | other |
| MSH5 | ENSG00000230961 | mutS homolog 5 | -2,166 | Nucleus | enzyme |
| MSH5-SAPCD1 | ENSG00000254405 | MSH5-SAPCD1 readthrough (NMD candidate) | -0,834 | Other | other |
| MSH6 | ENSG00000116062 | mutS homolog 6 | -0,777 | Nucleus | enzyme |
| MSMO1 | ENSG00000052802 | methylsterol monooxygenase 1 | -1,123 | Cytoplasm | enzyme |
| MSTO2P | ENSG00000203761 | misato family member 2, pseudogene | 0,699 | Other | other |
| MT-ND1 | ENSG00000198888 | NADH dehydrogenase, subunit 1 (complex I) | 0,684 | Cytoplasm | enzyme |
| MT1F | ENSG00000198417 | metallothionein 1F | 1,064 | Other | other |
| MT2A | ENSG00000125148 | metallothionein 2A | 5,269 | Cytoplasm | other |
| MTF1 | ENSG00000188786 | metal regulatory transcription factor 1 | 0,648 | Nucleus | transcription regulator |
| MTF2 | ENSG00000143033 | metal response element binding transcription factor 2 | 0,837 | Nucleus | transcription regulator |
| MTFR1L | ENSG00000117640 | mitochondrial fission regulator 1 like | -0,702 | Other | other |
| MTHFD2 | ENSG00000065911 | methylenetetrahydrofolate dehydrogenase (NADP+ dependent) 2, methenyltetrahydrofolate cyclohydrolase | 0,801 | Cytoplasm | enzyme |
| MTIF3 | ENSG00000122033 | mitochondrial translational initiation factor 3 | -0,639 | Cytoplasm | translation regulator |
| MTMR3 | ENSG00000100330 | myotubularin related protein 3 | 0,633 | Cytoplasm | phosphatase |
| MTRNR2L3 | ENSG00000256222 | MT-RNR2 like 3 | 0,628 | Other | other |
| MUC13 | ENSG00000173702 | mucin 13, cell surface associated | 1,933 | Extracellular Space | other |
| MUC20 | ENSG00000176945 | mucin 20, cell surface associated | -0,861 | Plasma Membrane | other |
| MUC20P1 | ENSG00000224769 | mucin 20, cell surface associated pseudogene 1 | -1,074 | Other | other |
| MUC5B | ENSG00000117983 | mucin 5B, oligomeric mucus/gel-forming | -0,714 | Extracellular Space | peptidase |
| MUC6 | ENSG00000184956 | mucin 6, oligomeric mucus/gel-forming | -1,024 | Extracellular Space | other |
| MVD | ENSG00000167508 | mevalonate diphosphate decarboxylase | -1,071 | Cytoplasm | enzyme |
| MVK | ENSG00000110921 | mevalonate kinase | -0,818 | Cytoplasm | kinase |
| MX1 | ENSG00000157601 | MX dynamin like GTPase 1 | 3,295 | Cytoplasm | enzyme |
| MX2 | ENSG00000183486 | MX dynamin like GTPase 2 | 4,588 | Nucleus | enzyme |
| MXD1 | ENSG00000059728 | MAX dimerization protein 1 | 3,367 | Nucleus | transcription regulator |
| MXD3 | ENSG00000213347 | MAX dimerization protein 3 | -1,366 | Nucleus | transcription regulator |
| MXD4 | ENSG00000123933 | MAX dimerization protein 4 | -1,388 | Nucleus | transcription regulator |
| MXI1 | ENSG00000119950 | MAX interactor 1, dimerization protein | -0,955 | Nucleus | transcription regulator |
| MXRA5 | ENSG00000101825 | matrix remodeling associated 5 | 0,696 | Extracellular Space | other |
| MXRA7 | ENSG00000182534 | matrix remodeling associated 7 | -1,028 | Extracellular Space | other |
| MXRA8 | ENSG00000162576 | matrix remodeling associated 8 | -0,667 | Cytoplasm | other |
| MYC | ENSG00000136997 | MYC proto-oncogene, bHLH transcription factor | 0,778 | Nucleus | transcription regulator |
| MYCBP2 | ENSG00000005810 | MYC binding protein 2 | 1,298 | Nucleus | enzyme |
| MYCL | ENSG00000116990 | MYCL proto-oncogene, bHLH transcription factor | 0,841 | Nucleus | transcription regulator |
| MYD88 | ENSG00000172936 | MYD88 innate immune signal transduction adaptor | 1,868 | Plasma Membrane | other |
| MYH10 | ENSG00000133026 | myosin heavy chain 10 | -0,944 | Cytoplasm | enzyme |
| MYH9 | ENSG00000100345 | myosin heavy chain 9 | -0,660 | Cytoplasm | enzyme |
| MYL9 | ENSG00000101335 | myosin light chain 9 | -1,081 | Cytoplasm | other |
| MYLIP | ENSG00000007944 | myosin regulatory light chain interacting protein | 0,876 | Cytoplasm | enzyme |
| MYLK | ENSG00000065534 | myosin light chain kinase | -1,117 | Cytoplasm | kinase |
| MYO1C | ENSG00000197879 | myosin IC | -0,770 | Cytoplasm | enzyme |
| MYOM1 | ENSG00000101605 | myomesin 1 | -0,633 | Cytoplasm | other |

| | | | | | |
|---|---|---|---|---|---|
| MYORG | ENSG00000164976 | myogenesis regulating glycosidase (putative) | -1,007 | Nucleus | other |
| MYRFL | ENSG00000166268 | myelin regulatory factor like | 0,975 | Other | other |
| MYSM1 | ENSG00000162601 | Myb like, SWIRM and MPN domains 1 | 0,797 | Nucleus | enzyme |
| N4BP1 | ENSG00000102921 | NEDD4 binding protein 1 | 1,534 | Cytoplasm | other |
| N4BP3 | ENSG00000145911 | NEDD4 binding protein 3 | 0,718 | Cytoplasm | other |
| NAA25 | ENSG00000111300 | N-alpha-acetyltransferase 25, NatB auxiliary subunit | 0,749 | Cytoplasm | other |
| NAALADL1 | ENSG00000168060 | N-acetylated alpha-linked acidic dipeptidase like 1 | 1,905 | Plasma Membrane | peptidase |
| NAALADL2 | ENSG00000177694 | N-acetylated alpha-linked acidic dipeptidase like 2 | -0,913 | Nucleus | other |
| NADSYN1 | ENSG00000172890 | NAD synthetase 1 | -0,669 | Cytoplasm | enzyme |
| NAGA | ENSG00000198951 | alpha-N-acetylgalactosaminidase | -0,651 | Cytoplasm | enzyme |
| NAMPT | ENSG00000105835 | nicotinamide phosphoribosyltransferase | 1,096 | Extracellular Space | cytokine |
| NAMPTP1 | ENSG00000229644 | nicotinamide phosphoribosyltransferase pseudogene 1 | 1,094 | Other | other |
| NAPA | ENSG00000105402 | NSF attachment protein alpha | 1,099 | Cytoplasm | transporter |
| NARF | ENSG00000141562 | nuclear prelamin A recognition factor | -0,679 | Nucleus | enzyme |
| NAT1 | ENSG00000171428 | N-acetyltransferase 1 | 0,648 | Cytoplasm | enzyme |
| NAV2 | ENSG00000166833 | neuron navigator 2 | 2,741 | Nucleus | other |
| NAV3 | ENSG00000067798 | neuron navigator 3 | 1,051 | Nucleus | other |
| NBEA | ENSG00000172915 | neurobeachin | -0,920 | Cytoplasm | other |
| NBPF10 (includes others) | ENSG00000271383 | NBPF member 19 | 0,748 | Other | other |
| NCALD | ENSG00000104490 | neurocalcin delta | 2,242 | Cytoplasm | other |
| NCAPD2 | ENSG00000010292 | non-SMC condensin I complex subunit D2 | -1,154 | Nucleus | other |
| NCAPG | ENSG00000109805 | non-SMC condensin I complex subunit G | -0,808 | Nucleus | other |
| NCCRP1 | ENSG00000188505 | NCCRP1, F-box associated domain containing | 0,890 | Cytoplasm | other |
| NCEH1 | ENSG00000144959 | neutral cholesterol ester hydrolase 1 | 0,709 | Plasma Membrane | enzyme |
| NCF2 | ENSG00000116701 | neutrophil cytosolic factor 2 | 0,749 | Cytoplasm | enzyme |
| NCOA3 | ENSG00000124151 | nuclear receptor coactivator 3 | 0,719 | Nucleus | transcription regulator |
| NCOA7 | ENSG00000111912 | nuclear receptor coactivator 7 | 2,631 | Nucleus | transcription regulator |
| NCOR2 | ENSG00000196498 | nuclear receptor corepressor 2 | -0,629 | Nucleus | transcription regulator |
| NDC1 | ENSG00000058804 | NDC1 transmembrane nucleoporin | -0,648 | Nucleus | other |
| NDRG1 | ENSG00000104419 | N-myc downstream regulated 1 | -1,539 | Nucleus | kinase |
| NDRG2 | ENSG00000165795 | NDRG family member 2 | -0,641 | Cytoplasm | other |
| NDRG3 | ENSG00000101079 | NDRG family member 3 | -0,748 | Cytoplasm | other |
| NDUFA1 | ENSG00000125356 | NADH:ubiquinone oxidoreductase subunit A1 | -0,631 | Cytoplasm | enzyme |
| NDUFA2 | ENSG00000131495 | NADH:ubiquinone oxidoreductase subunit A2 | -0,870 | Cytoplasm | enzyme |
| NDUFAF8 | ENSG00000224877 | NADH:ubiquinone oxidoreductase complex assembly factor 8 | -0,833 | Cytoplasm | other |
| NDUFB10 | ENSG00000140990 | NADH:ubiquinone oxidoreductase subunit B10 | -0,680 | Cytoplasm | enzyme |
| NDUFB2 | ENSG00000090266 | NADH:ubiquinone oxidoreductase subunit B2 | -0,639 | Cytoplasm | enzyme |
| NDUFB6 | ENSG00000165264 | NADH:ubiquinone oxidoreductase subunit B6 | -0,779 | Cytoplasm | enzyme |
| NEB | ENSG00000183091 | nebulin | -1,450 | Cytoplasm | other |
| NECAP1 | ENSG00000089818 | NECAP endocytosis associated 1 | 0,815 | Plasma Membrane | other |
| NECTIN2 | ENSG00000130202 | nectin cell adhesion molecule 2 | 0,969 | Plasma Membrane | transmembrane receptor |
| NEDD1 | ENSG00000139350 | NEDD1 gamma-tubulin ring complex targeting factor | 1,218 | Cytoplasm | other |
| NEDD4 | ENSG00000069869 | NEDD4 E3 ubiquitin protein ligase | 0,785 | Cytoplasm | enzyme |
| NEDD9 | ENSG00000111859 | neural precursor cell expressed, developmentally down-regulated 9 | 1,475 | Nucleus | other |
| NEK2 | ENSG00000117650 | NIMA related kinase 2 | -1,006 | Cytoplasm | kinase |
| NEK6 | ENSG00000119408 | NIMA related kinase 6 | -0,769 | Nucleus | kinase |
| NEMP2 | ENSG00000189362 | nuclear envelope integral membrane protein 2 | -0,668 | Other | other |
| NEU4 | ENSG00000204099 | neuraminidase 4 | 2,099 | Cytoplasm | enzyme |
| NEURL3 | ENSG00000163121 | neuralized E3 ubiquitin protein ligase 3 | 2,567 | Other | enzyme |
| NFAT5 | ENSG00000102908 | nuclear factor of activated T cells 5 | 1,302 | Nucleus | transcription regulator |
| NFE2L2 | ENSG00000116044 | nuclear factor, erythroid 2 like 2 | 0,620 | Nucleus | transcription regulator |
| NFE2L3 | ENSG00000050344 | nuclear factor, erythroid 2 like 3 | 1,382 | Nucleus | transcription regulator |
| NFIL3 | ENSG00000165030 | nuclear factor, interleukin 3 regulated | 0,973 | Nucleus | transcription regulator |
| NFIX | ENSG00000008441 | nuclear factor I X | 0,913 | Nucleus | transcription regulator |
| NFKB1 | ENSG00000109320 | nuclear factor kappa B subunit 1 | 1,123 | Nucleus | transcription regulator |
| NFKB2 | ENSG00000077150 | nuclear factor kappa B subunit 2 | 1,897 | Nucleus | transcription regulator |
| NFKBIA | ENSG00000100906 | NFKB inhibitor alpha | 4,497 | Cytoplasm | transcription regulator |
| NFKBIB | ENSG00000104825 | NFKB inhibitor beta | 0,737 | Nucleus | transcription regulator |
| NFKBID | ENSG00000167604 | NFKB inhibitor delta | 0,969 | Nucleus | transcription regulator |
| NFKBIE | ENSG00000146232 | NFKB inhibitor epsilon | 1,691 | Nucleus | transcription regulator |
| NFKBIZ | ENSG00000144802 | NFKB inhibitor zeta | 2,503 | Nucleus | transcription regulator |
| NFS1 | ENSG00000244005 | NFS1 cysteine desulfurase | -0,855 | Cytoplasm | enzyme |
| NGFR | ENSG00000064300 | nerve growth factor receptor | 1,112 | Plasma Membrane | transmembrane receptor |
| NIBAN1 | ENSG00000135842 | niban apoptosis regulator 1 | 1,156 | Cytoplasm | other |
| NIBAN2 | ENSG00000136830 | niban apoptosis regulator 2 | -0,630 | Cytoplasm | transcription regulator |
| NIPAL2 | ENSG00000104361 | NIPA like domain containing 2 | -0,802 | Other | other |
| NIPSNAP1 | ENSG00000184117 | nipsnap homolog 1 | -0,686 | Cytoplasm | enzyme |
| NIPSNAP2 | ENSG00000146729 | nipsnap homolog 2 | -0,770 | Plasma Membrane | other |
| NKAIN1 | ENSG00000084628 | sodium/potassium transporting ATPase interacting 1 | 1,570 | Plasma Membrane | other |
| NKIRAS1 | ENSG00000197885 | NFKB inhibitor interacting Ras like 1 | 0,784 | Other | enzyme |
| NKX3-1 | ENSG00000167034 | NK3 homeobox 1 | 2,478 | Nucleus | transcription regulator |
| NLK | ENSG00000087095 | nemo like kinase | 0,649 | Nucleus | kinase |
| NLRC5 | ENSG00000140853 | NLR family CARD domain containing 5 | 3,694 | Cytoplasm | transcription regulator |
| NLRP3 | ENSG00000162711 | NLR family pyrin domain containing 3 | 1,062 | Cytoplasm | other |
| NMB | ENSG00000197696 | neuromedin B | -0,736 | Extracellular Space | other |
| NME1-NME2 | ENSG00000011052 | NME1-NME2 readthrough | -0,673 | Cytoplasm | other |
| NME3 | ENSG00000103024 | NME/NM23 nucleoside diphosphate kinase 3 | -0,777 | Cytoplasm | kinase |
| NMI | ENSG00000123609 | N-myc and STAT interactor | 2,411 | Cytoplasm | transcription regulator |
| NMU | ENSG00000109255 | neuromedin U | -1,177 | Extracellular Space | other |
| NNT | ENSG00000112992 | nicotinamide nucleotide transhydrogenase | -1,078 | Cytoplasm | enzyme |
| NOCT | ENSG00000151014 | nocturnin | 3,236 | Nucleus | transcription regulator |
| NOD1 | ENSG00000106100 | nucleotide binding oligomerization domain containing 1 | 1,213 | Cytoplasm | other |
| NOD2 | ENSG00000167207 | nucleotide binding oligomerization domain containing 2 | 0,761 | Cytoplasm | other |
| NOL6 | ENSG00000165271 | nucleolar protein 6 | 0,788 | Nucleus | other |
| NONOP2 | ENSG00000237522 | non-POU domain containing, octamer-binding pseudogene 2 | 0,834 | Other | other |
| NOS3 | ENSG00000164867 | nitric oxide synthase 3 | 1,383 | Cytoplasm | enzyme |
| NOTCH3 | ENSG00000074181 | notch receptor 3 | -1,130 | Plasma Membrane | transcription regulator |
| NPC1L1 | ENSG00000015520 | NPC1 like intracellular cholesterol transporter 1 | -0,927 | Cytoplasm | transporter |
| NPC2 | ENSG00000119655 | NPC intracellular cholesterol transporter 2 | -0,656 | Extracellular Space | transporter |
| NPFFR2 | ENSG00000056291 | neuropeptide FF receptor 2 | 2,267 | Plasma Membrane | G-protein coupled receptor |
| NPHP1 | ENSG00000144061 | nephrocystin 1 | -1,000 | Nucleus | other |
| NPIPA5 (includes others) | ENSG00000183793 | nuclear pore complex interacting protein family member A5 | 2,332 | Nucleus | other |
| NPNT | ENSG00000168743 | nephronectin | -0,748 | Plasma Membrane | other |
| NPTX1 | ENSG00000171246 | neuronal pentraxin 1 | 1,417 | Extracellular Space | other |
| NR1D1 | ENSG00000126368 | nuclear receptor subfamily 1 group D member 1 | 1,192 | Nucleus | ligand-dependent nuclear receptor |
| NR1D2 | ENSG00000174738 | nuclear receptor subfamily 1 group D member 2 | 0,810 | Nucleus | ligand-dependent nuclear receptor |
| NR1I2 | ENSG00000144852 | nuclear receptor subfamily 1 group I member 2 | 1,032 | Nucleus | ligand-dependent nuclear receptor |
| NR2F1 | ENSG00000175745 | nuclear receptor subfamily 2 group F member 1 | 1,160 | Nucleus | ligand-dependent nuclear receptor |
| NR3C1 | ENSG00000113580 | nuclear receptor subfamily 3 group C member 1 | 1,475 | Nucleus | ligand-dependent nuclear receptor |

| | | | | | |
|---|---|---|---|---|---|
| NR4A1 | ENSG00000123358 | nuclear receptor subfamily 4 group A member 1 | 1,021 | Nucleus | ligand-dependent nuclear receptor |
| NR4A2 | ENSG00000153234 | nuclear receptor subfamily 4 group A member 2 | 1,792 | Nucleus | ligand-dependent nuclear receptor |
| NR4A3 | ENSG00000119508 | nuclear receptor subfamily 4 group A member 3 | 2,070 | Nucleus | ligand-dependent nuclear receptor |
| NRBF2 | ENSG00000148572 | nuclear receptor binding factor 2 | 0,666 | Nucleus | transcription regulator |
| NRG3 | ENSG00000185737 | neuregulin 3 | -0,648 | Extracellular Space | growth factor |
| NRM | ENSG00000137404 | nurim | -1,742 | Nucleus | other |
| NRP1 | ENSG00000099250 | neuropilin 1 | 0,671 | Plasma Membrane | transmembrane receptor |
| NSDHL | ENSG00000147383 | NAD(P) dependent steroid dehydrogenase-like | -0,728 | Cytoplasm | enzyme |
| NSMCE1 | ENSG00000169189 | NSE1 homolog, SMC5-SMC6 complex component | -0,831 | Nucleus | transporter |
| NSRP1 | ENSG00000126653 | nuclear speckle splicing regulatory protein 1 | 0,739 | Nucleus | other |
| NSUN5 | ENSG00000130305 | NOP2/Sun RNA methyltransferase 5 | 0,790 | Nucleus | enzyme |
| NSUN6 | ENSG00000241058 | NOP2/Sun RNA methyltransferase 6 | 0,818 | Cytoplasm | enzyme |
| NSUN7 | ENSG00000179299 | NOP2/Sun RNA methyltransferase family member 7 | -0,936 | Other | other |
| NT5C3A | ENSG00000122643 | 5'-nucleotidase, cytosolic IIIA | 3,579 | Cytoplasm | phosphatase |
| NT5C3AP1 | ENSG00000213492 | NT5C3A pseudogene 1 | 2,727 | Other | other |
| NT5DC2 | ENSG00000168268 | 5'-nucleotidase domain containing 2 | -1,300 | Cytoplasm | other |
| NT5DC4 | ENSG00000144130 | 5'-nucleotidase domain containing 4 | 2,069 | Other | other |
| NT5E | ENSG00000135318 | 5'-nucleotidase ecto | 1,097 | Plasma Membrane | phosphatase |
| NTMT1 | ENSG00000148335 | N-terminal Xaa-Pro-Lys N-methyltransferase 1 | 0,655 | Nucleus | enzyme |
| NTRK2 | ENSG00000148053 | neurotrophic receptor tyrosine kinase 2 | -0,629 | Plasma Membrane | kinase |
| NUAK2 | ENSG00000163545 | NUAK family kinase 2 | 2,702 | Nucleus | kinase |
| NUB1 | ENSG00000013374 | negative regulator of ubiquitin like proteins 1 | 2,191 | Nucleus | other |
| NUDCD1 | ENSG00000120526 | NudC domain containing 1 | 0,759 | Nucleus | other |
| NUDT12 | ENSG00000112874 | nudix hydrolase 12 | -0,665 | Cytoplasm | phosphatase |
| NUDT2 | ENSG00000164978 | nudix hydrolase 2 | -0,746 | Plasma Membrane | phosphatase |
| NUDT3 | ENSG00000272325 | nudix hydrolase 3 | -0,873 | Cytoplasm | phosphatase |
| NUDT8 | ENSG00000167799 | nudix hydrolase 8 | -1,225 | Cytoplasm | other |
| NUF2 | ENSG00000143228 | NUF2 component of NDC80 kinetochore complex | -0,798 | Nucleus | other |
| NUP37 | ENSG00000075188 | nucleoporin 37 | -0,740 | Nucleus | transporter |
| NUSAP1 | ENSG00000137804 | nucleolar and spindle associated protein 1 | -0,818 | Nucleus | other |
| NUTF2 | ENSG00000102898 | nuclear transport factor 2 | -0,612 | Nucleus | transporter |
| NXF1 | ENSG00000162231 | nuclear RNA export factor 1 | 0,736 | Nucleus | other |
| NYNRIN | ENSG00000205978 | NYN domain and retroviral integrase containing | -0,908 | Other | other |
| OAF | ENSG00000184232 | out at first homolog | 1,410 | Cytoplasm | other |
| OAS1 | ENSG00000089127 | 2'-5'-oligoadenylate synthetase 1 | 3,269 | Cytoplasm | enzyme |
| OAS2 | ENSG00000111335 | 2'-5'-oligoadenylate synthetase 2 | 3,951 | Cytoplasm | enzyme |
| OAS3 | ENSG00000111331 | 2'-5'-oligoadenylate synthetase 3 | 2,381 | Cytoplasm | enzyme |
| OASL | ENSG00000135114 | 2'-5'-oligoadenylate synthetase like | 5,510 | Cytoplasm | enzyme |
| OAT | ENSG00000065154 | ornithine aminotransferase | -0,908 | Cytoplasm | enzyme |
| ODAM | ENSG00000109205 | odontogenic, ameloblast associated | -1,051 | Extracellular Space | other |
| ODF2 | ENSG00000136811 | outer dense fiber of sperm tails 2 | -0,769 | Cytoplasm | other |
| ODF3B | ENSG00000177989 | outer dense fiber of sperm tails 3B | 1,773 | Other | enzyme |
| OFD1 | ENSG00000046651 | OFD1 centriole and centriolar satellite protein | 0,682 | Plasma Membrane | other |
| OGFR | ENSG00000060491 | opioid growth factor receptor | 1,929 | Plasma Membrane | other |
| OIP5 | ENSG00000104147 | Opa interacting protein 5 | -0,641 | Nucleus | other |
| OLFM2 | ENSG00000105088 | olfactomedin 2 | -0,645 | Cytoplasm | other |
| OLFM4 | ENSG00000102837 | olfactomedin 4 | -1,608 | Extracellular Space | other |
| OPHN1 | ENSG00000079482 | oligophrenin 1 | 0,796 | Cytoplasm | other |
| OPRK1 | ENSG00000082556 | opioid receptor kappa 1 | 0,813 | Plasma Membrane | G-protein coupled receptor |
| OPTN | ENSG00000123240 | optineurin | 2,307 | Cytoplasm | other |
| OR10AB1P | ENSG00000176716 | olfactory receptor family 10 subfamily AB member 1 pseudogene | 2,634 | Other | other |
| OR1F1 | ENSG00000168124 | olfactory receptor family 1 subfamily F member 1 (gene/pseudogene) | 2,433 | Plasma Membrane | other |
| OR2I1P | ENSG00000232049 | olfactory receptor family 2 subfamily I member 1 pseudogene | 1,177 | Plasma Membrane | other |
| OR4C6 | ENSG00000181903 | olfactory receptor family 4 subfamily C member 6 | 1,750 | Plasma Membrane | G-protein coupled receptor |
| OR52K3P | ENSG00000225101 | olfactory receptor family 52 subfamily K member 3 pseudogene | 3,357 | Other | other |
| ORC6 | ENSG00000091651 | origin recognition complex subunit 6 | 0,666 | Nucleus | other |
| OSER1 | ENSG00000132823 | oxidative stress responsive serine rich 1 | 0,622 | Other | enzyme |
| OSGIN1 | ENSG00000140961 | oxidative stress induced growth inhibitor 1 | 0,847 | Other | growth factor |
| OSR2 | ENSG00000164920 | odd-skipped related transciption factor 2 | 0,965 | Nucleus | transcription regulator |
| OSTM1 | ENSG00000081087 | osteoclastogenesis associated transmembrane protein 1 | 0,786 | Cytoplasm | other |
| OTUD1 | ENSG00000165312 | OTU deubiquitinase 1 | 4,175 | Other | peptidase |
| OTUD4 | ENSG00000164164 | OTU deubiquitinase 4 | 1,033 | Cytoplasm | peptidase |
| OVOL1 | ENSG00000172818 | ovo like transcriptional repressor 1 | 1,312 | Nucleus | transcription regulator |
| OVOL2 | ENSG00000125850 | ovo like zinc finger 2 | -1,022 | Nucleus | transcription regulator |
| OXCT1 | ENSG00000083720 | 3-oxoacid CoA-transferase 1 | -0,840 | Cytoplasm | enzyme |
| P3H2 | ENSG00000090530 | prolyl 3-hydroxylase 2 | 1,311 | Cytoplasm | enzyme |
| PA2G4P4 | ENSG00000230457 | proliferation-associated 2G4 pseudogene 4 | 2,108 | Other | other |
| PABPC1P4 | ENSG00000255642 | poly(A) binding protein cytoplasmic 1 pseudogene 4 | 0,793 | Other | other |
| PADI2 | ENSG00000117115 | peptidyl arginine deiminase 2 | -0,930 | Cytoplasm | enzyme |
| PAFAH1B3 | ENSG00000079462 | platelet activating factor acetylhydrolase 1b catalytic subunit 3 | -0,601 | Cytoplasm | enzyme |
| PAFAH2 | ENSG00000158006 | platelet activating factor acetylhydrolase 2 | -0,703 | Cytoplasm | enzyme |
| PAH | ENSG00000171759 | phenylalanine hydroxylase | -0,881 | Cytoplasm | enzyme |
| PAIP2B | ENSG00000124374 | poly(A) binding protein interacting protein 2B | -1,137 | Other | translation regulator |
| PAK3 | ENSG00000077264 | p21 (RAC1) activated kinase 3 | 0,807 | Cytoplasm | kinase |
| PALM3 | ENSG00000187867 | paralemmin 3 | -1,107 | Other | other |
| PALMD | ENSG00000099260 | palmdelphin | -1,673 | Cytoplasm | other |
| PAM | ENSG00000145730 | peptidylglycine alpha-amidating monooxygenase | -1,000 | Plasma Membrane | enzyme |
| PANX1 | ENSG00000110218 | pannexin 1 | 0,613 | Plasma Membrane | transporter |
| PAPOLG | ENSG00000115421 | poly(A) polymerase gamma | 0,793 | Nucleus | enzyme |
| PAPSS1 | ENSG00000138801 | 3'-phosphoadenosine 5'-phosphosulfate synthase 1 | -0,838 | Cytoplasm | enzyme |
| PAPSS2 | ENSG00000198682 | 3'-phosphoadenosine 5'-phosphosulfate synthase 2 | -1,346 | Cytoplasm | enzyme |
| PARD6B | ENSG00000124171 | par-6 family cell polarity regulator beta | 0,910 | Plasma Membrane | other |
| PARP10 | ENSG00000178685 | poly(ADP-ribose) polymerase family member 10 | 2,808 | Cytoplasm | enzyme |
| PARP12 | ENSG00000059378 | poly(ADP-ribose) polymerase family member 12 | 2,327 | Nucleus | enzyme |
| PARP14 | ENSG00000173193 | poly(ADP-ribose) polymerase family member 14 | 2,799 | Cytoplasm | enzyme |
| PARP3 | ENSG00000041880 | poly(ADP-ribose) polymerase family member 3 | 1,623 | Nucleus | enzyme |
| PARP8 | ENSG00000151883 | poly(ADP-ribose) polymerase family member 8 | 1,181 | Other | other |
| PARP9 | ENSG00000138496 | poly(ADP-ribose) polymerase family member 9 | 1,815 | Nucleus | enzyme |
| PARPBP | ENSG00000185480 | PARP1 binding protein | -0,812 | Nucleus | other |
| PARVB | ENSG00000188677 | parvin beta | -0,637 | Cytoplasm | other |
| PATJ | ENSG00000132849 | PATJ crumbs cell polarity complex component | -0,606 | Plasma Membrane | other |
| PATL1 | ENSG00000166889 | PAT1 homolog 1, processing body mRNA decay factor | 1,062 | Cytoplasm | translation regulator |
| PATL2 | ENSG00000229474 | PAT1 homolog 2 | 1,877 | Cytoplasm | translation regulator |
| PAXIP1-AS2 | ENSG00000214106 | PAXIP1 antisense RNA 2 | 1,045 | Other | other |
| PBK | ENSG00000168078 | PDZ binding kinase | -0,735 | Cytoplasm | kinase |
| PBX1 | ENSG00000185630 | PBX homeobox 1 | -1,185 | Nucleus | transcription regulator |
| PBX3 | ENSG00000167081 | PBX homeobox 3 | -0,804 | Nucleus | transcription regulator |
| PBXIP1 | ENSG00000163346 | PBX homeobox interacting protein 1 | -1,082 | Nucleus | transcription regulator |
| PC | ENSG00000173599 | pyruvate carboxylase | -0,636 | Cytoplasm | enzyme |

| | | | | | |
|---|---|---|---|---|---|
| PCDH9 | ENSG00000184226 | protocadherin 9 | 0,817 | Plasma Membrane | other |
| PCDHA13 | ENSG00000239389 | protocadherin alpha 13 | -0,628 | Plasma Membrane | other |
| PCDHGA9 | ENSG00000261934 | protocadherin gamma subfamily A, 9 | -0,708 | Other | other |
| PCGF5 | ENSG00000180628 | polycomb group ring finger 5 | 1,747 | Cytoplasm | other |
| PCMTD2 | ENSG00000203880 | protein-L-isoaspartate (D-aspartate) O-methyltransferase domain containing 2 | -0,640 | Cytoplasm | enzyme |
| PCOLCE2 | ENSG00000163710 | procollagen C-endopeptidase enhancer 2 | -0,739 | Extracellular Space | other |
| PCSK9 | ENSG00000169174 | proprotein convertase subtilisin/kexin type 9 | -1,266 | Extracellular Space | peptidase |
| PCYOX1 | ENSG00000116005 | prenylcysteine oxidase 1 | -0,647 | Cytoplasm | enzyme |
| PCYOX1L | ENSG00000145882 | prenylcysteine oxidase 1 like | -0,920 | Other | other |
| PCYT2 | ENSG00000185813 | phosphate cytidylyltransferase 2, ethanolamine | -0,796 | Cytoplasm | enzyme |
| PDCD1LG2 | ENSG00000197646 | programmed cell death 1 ligand 2 | 1,513 | Plasma Membrane | enzyme |
| PDCD4 | ENSG00000150593 | programmed cell death 4 | -1,405 | Nucleus | other |
| PDCL3 | ENSG00000115539 | phosducin like 3 | 1,044 | Cytoplasm | other |
| PDE4B | ENSG00000184588 | phosphodiesterase 4B | 1,046 | Cytoplasm | enzyme |
| PDE4D | ENSG00000113448 | phosphodiesterase 4D | 0,678 | Cytoplasm | enzyme |
| PDE5A | ENSG00000138735 | phosphodiesterase 5A | -0,683 | Cytoplasm | enzyme |
| PDGFA | ENSG00000197461 | platelet derived growth factor subunit A | 2,117 | Extracellular Space | growth factor |
| PDGFD | ENSG00000170962 | platelet derived growth factor D | -1,408 | Extracellular Space | growth factor |
| PDGFRL | ENSG00000104213 | platelet derived growth factor receptor like | 2,318 | Plasma Membrane | kinase |
| PDK1 | ENSG00000152256 | pyruvate dehydrogenase kinase 1 | -1,041 | Cytoplasm | kinase |
| PDK2 | ENSG00000005882 | pyruvate dehydrogenase kinase 2 | -0,806 | Cytoplasm | kinase |
| PDZD2 | ENSG00000133401 | PDZ domain containing 2 | 1,574 | Plasma Membrane | other |
| PDZK1IP1 | ENSG00000162366 | PDZK1 interacting protein 1 | -0,737 | Extracellular Space | other |
| PELI1 | ENSG00000197329 | pellino E3 ubiquitin protein ligase 1 | 1,860 | Cytoplasm | enzyme |
| PEMT | ENSG00000133027 | phosphatidylethanolamine N-methyltransferase | -0,643 | Cytoplasm | enzyme |
| PER1 | ENSG00000179094 | period circadian regulator 1 | 2,078 | Nucleus | transcription regulator |
| PER2 | ENSG00000132326 | period circadian regulator 2 | 1,066 | Nucleus | transcription regulator |
| PERP | ENSG00000112378 | p53 apoptosis effector related to PMP22 | -1,049 | Plasma Membrane | other |
| PET100 | ENSG00000229833 | PET100 cytochrome c oxidase chaperone | -0,674 | Other | other |
| PEX12 | ENSG00000108733 | peroxisomal biogenesis factor 12 | 0,646 | Cytoplasm | other |
| PFKFB3 | ENSG00000170525 | 6-phosphofructo-2-kinase/fructose-2,6-biphosphatase 3 | -0,866 | Cytoplasm | kinase |
| PGAP1 | ENSG00000197121 | post-GPI attachment to proteins inositol deacylase 1 | 0,807 | Cytoplasm | enzyme |
| PGAP3 | ENSG00000161395 | post-GPI attachment to proteins phospholipase 3 | -1,101 | Cytoplasm | enzyme |
| PGGHG | ENSG00000142102 | protein-glucosylgalactosylhydroxylysine glucosidase | 0,661 | Cytoplasm | enzyme |
| PGK1 | ENSG00000102144 | phosphoglycerate kinase 1 | -0,921 | Cytoplasm | kinase |
| PGLYRP2 | ENSG00000161031 | peptidoglycan recognition protein 2 | 1,182 | Plasma Membrane | transmembrane receptor |
| PGRMC1 | ENSG00000101856 | progesterone receptor membrane component 1 | -0,789 | Plasma Membrane | transmembrane receptor |
| PGRMC2 | ENSG00000164040 | progesterone receptor membrane component 2 | -0,654 | Nucleus | other |
| PHACTR4 | ENSG00000204138 | phosphatase and actin regulator 4 | 1,159 | Plasma Membrane | other |
| PHC2 | ENSG00000134686 | polyhomeotic homolog 2 | 0,794 | Nucleus | other |
| PHF11 | ENSG00000136147 | PHD finger protein 11 | 1,823 | Nucleus | other |
| PHF7 | ENSG00000010318 | PHD finger protein 7 | -0,792 | Nucleus | other |
| PHKB | ENSG00000102893 | phosphorylase kinase regulatory subunit beta | -1,072 | Cytoplasm | kinase |
| PHLDB2 | ENSG00000144824 | pleckstrin homology like domain family B member 2 | 0,642 | Cytoplasm | other |
| PHLDB3 | ENSG00000176531 | pleckstrin homology like domain family B member 3 | 0,839 | Other | other |
| PHPT1 | ENSG00000054148 | phosphohistidine phosphatase 1 | -0,657 | Cytoplasm | phosphatase |
| PHYH | ENSG00000107537 | phytanoyl-CoA 2-hydroxylase | -0,713 | Cytoplasm | enzyme |
| PHYHIP | ENSG00000168490 | phytanoyl-CoA 2-hydroxylase interacting protein | 0,897 | Cytoplasm | other |
| PI4K2B | ENSG00000038210 | phosphatidylinositol 4-kinase type 2 beta | 0,949 | Cytoplasm | kinase |
| PIEZO1 | ENSG00000103335 | piezo type mechanosensitive ion channel component 1 | 0,600 | Cytoplasm | ion channel |
| PIF1 | ENSG00000140451 | PIF1 5'-to-3' DNA helicase | -1,313 | Nucleus | enzyme |
| PIGA | ENSG00000165195 | phosphatidylinositol glycan anchor biosynthesis class A | 0,655 | Cytoplasm | enzyme |
| PIGF | ENSG00000151665 | phosphatidylinositol glycan anchor biosynthesis class F | -0,779 | Cytoplasm | enzyme |
| PIGS | ENSG00000087111 | phosphatidylinositol glycan anchor biosynthesis class S | -0,731 | Cytoplasm | enzyme |
| PIGZ | ENSG00000119227 | phosphatidylinositol glycan anchor biosynthesis class Z | -0,768 | Cytoplasm | enzyme |
| PIK3AP1 | ENSG00000155629 | phosphoinositide-3-kinase adaptor protein 1 | 1,187 | Cytoplasm | other |
| PIK3C2B | ENSG00000133056 | phosphatidylinositol-4-phosphate 3-kinase catalytic subunit type 2 beta | -0,727 | Cytoplasm | kinase |
| PIK3IP1 | ENSG00000100100 | phosphoinositide-3-kinase interacting protein 1 | -1,581 | Cytoplasm | other |
| PIK3R1 | ENSG00000145675 | phosphoinositide-3-kinase regulatory subunit 1 | 1,274 | Cytoplasm | kinase |
| PIK3R2 | ENSG00000105647 | phosphoinositide-3-kinase regulatory subunit 2 | -0,959 | Cytoplasm | kinase |
| PIK3R3 | ENSG00000117461 | phosphoinositide-3-kinase regulatory subunit 3 | 2,019 | Cytoplasm | kinase |
| PIM3 | ENSG00000198355 | Pim-3 proto-oncogene, serine/threonine kinase | 1,054 | Cytoplasm | kinase |
| PIMREG | ENSG00000129195 | PICALM interacting mitotic regulator | -1,178 | Nucleus | other |
| PINLYP | ENSG00000234465 | phospholipase A2 inhibitor and LY6/PLAUR domain containing | 2,365 | Other | other |
| PIP5K1A | ENSG00000143398 | phosphatidylinositol-4-phosphate 5-kinase type 1 alpha | 0,962 | Cytoplasm | kinase |
| PISD | ENSG00000241878 | phosphatidylserine decarboxylase | 0,746 | Cytoplasm | enzyme |
| PITPNC1 | ENSG00000154217 | phosphatidylinositol transfer protein cytoplasmic 1 | -1,020 | Cytoplasm | transporter |
| PITPNM3 | ENSG00000091622 | PITPNM family member 3 | -0,821 | Cytoplasm | enzyme |
| PITX1 | ENSG00000069011 | paired like homeodomain 1 | 0,714 | Nucleus | transcription regulator |
| PIWIL4 | ENSG00000134627 | piwi like RNA-mediated gene silencing 4 | 0,776 | Cytoplasm | other |
| PKDCC | ENSG00000162878 | protein kinase domain containing, cytoplasmic | -1,128 | Cytoplasm | kinase |
| PKIA | ENSG00000171033 | cAMP-dependent protein kinase inhibitor alpha | -1,177 | Cytoplasm | other |
| PKIB | ENSG00000135549 | cAMP-dependent protein kinase inhibitor beta | 0,880 | Other | other |
| PLA2G4C | ENSG00000105499 | phospholipase A2 group IVC | 3,318 | Plasma Membrane | enzyme |
| PLA2G6 | ENSG00000184381 | phospholipase A2 group VI | 0,628 | Cytoplasm | enzyme |
| PLA2R1 | ENSG00000153246 | phospholipase A2 receptor 1 | -0,852 | Plasma Membrane | transmembrane receptor |
| PLAAT2 | ENSG00000133328 | phospholipase A and acyltransferase 2 | 3,137 | Cytoplasm | enzyme |
| PLAAT4 | ENSG00000133321 | phospholipase A and acyltransferase 4 | 2,459 | Cytoplasm | enzyme |
| PLAAT5 | ENSG00000168004 | phospholipase A and acyltransferase 5 | -0,768 | Other | enzyme |
| PLAC8 | ENSG00000145287 | placenta associated 8 | 2,672 | Nucleus | other |
| PLAC9 | ENSG00000189129 | placenta associated 9 | -0,910 | Other | other |
| PLAT | ENSG00000104368 | plasminogen activator, tissue type | 1,193 | Extracellular Space | peptidase |
| PLAUR | ENSG00000011422 | plasminogen activator, urokinase receptor | 3,769 | Plasma Membrane | transmembrane receptor |
| PLB1 | ENSG00000163803 | phospholipase B1 | 1,453 | Cytoplasm | enzyme |
| PLCE1 | ENSG00000138193 | phospholipase C epsilon 1 | -0,833 | Cytoplasm | enzyme |
| PLCG2 | ENSG00000197943 | phospholipase C gamma 2 | 2,470 | Cytoplasm | enzyme |
| PLCH1 | ENSG00000114805 | phospholipase C eta 1 | 0,878 | Cytoplasm | enzyme |
| PLD1 | ENSG00000075651 | phospholipase D1 | -0,858 | Cytoplasm | enzyme |
| PLD6 | ENSG00000179598 | phospholipase D family member 6 | 0,720 | Cytoplasm | enzyme |
| PLEKHA4 | ENSG00000105559 | pleckstrin homology domain containing A4 | 2,752 | Cytoplasm | other |
| PLEKHA7 | ENSG00000166689 | pleckstrin homology domain containing A7 | 0,784 | Cytoplasm | other |
| PLEKHB1 | ENSG00000021300 | pleckstrin homology domain containing B1 | -1,139 | Cytoplasm | other |
| PLEKHB2 | ENSG00000115762 | pleckstrin homology domain containing B2 | -0,619 | Other | other |
| PLEKHG5 | ENSG00000171680 | pleckstrin homology and RhoGEF domain containing G5 | 1,186 | Cytoplasm | other |
| PLEKHG6 | ENSG00000008323 | pleckstrin homology and RhoGEF domain containing G6 | 0,691 | Cytoplasm | other |
| PLEKHG7 | ENSG00000187510 | pleckstrin homology and RhoGEF domain containing G7 | 1,528 | Other | other |
| PLEKHN1 | ENSG00000187583 | pleckstrin homology domain containing N1 | 0,831 | Cytoplasm | other |
| PLIN3 | ENSG00000105355 | perilipin 3 | -0,694 | Cytoplasm | other |
| PLK1 | ENSG00000166851 | polo like kinase 1 | -0,952 | Nucleus | kinase |

| | | | | | |
|---|---|---|---|---|---|
| PLK3 | ENSG00000173846 | polo like kinase 3 | 1,049 | Nucleus | kinase |
| PLLP | ENSG00000102934 | plasmolipin | -0,875 | Plasma Membrane | transporter |
| PLOD3 | ENSG00000106397 | procollagen-lysine,2-oxoglutarate 5-dioxygenase 3 | 0,611 | Cytoplasm | enzyme |
| PLPP1 | ENSG00000067113 | phospholipid phosphatase 1 | -0,678 | Plasma Membrane | phosphatase |
| PLS1 | ENSG00000120756 | plastin 1 | -0,903 | Plasma Membrane | other |
| PLS3 | ENSG00000102024 | plastin 3 | -1,062 | Cytoplasm | other |
| PLSCR1 | ENSG00000188313 | phospholipid scramblase 1 | 1,053 | Plasma Membrane | enzyme |
| PLXNA2 | ENSG00000076356 | plexin A2 | 1,859 | Plasma Membrane | transmembrane receptor |
| PMAIP1 | ENSG00000141682 | phorbol-12-myristate-13-acetate-induced protein 1 | 3,964 | Cytoplasm | other |
| PMEPA1 | ENSG00000124225 | prostate transmembrane protein, androgen induced 1 | -0,976 | Plasma Membrane | other |
| PML | ENSG00000140464 | promyelocytic leukemia | 2,406 | Nucleus | transcription regulator |
| PMM1 | ENSG00000100417 | phosphomannomutase 1 | -0,745 | Cytoplasm | enzyme |
| PMVK | ENSG00000163344 | phosphomevalonate kinase | -0,908 | Cytoplasm | kinase |
| PNKD | ENSG00000127838 | PNKD metallo-beta-lactamase domain containing | -0,687 | Nucleus | other |
| PNMT | ENSG00000141744 | phenylethanolamine N-methyltransferase | -1,479 | Cytoplasm | enzyme |
| PNP | ENSG00000198805 | purine nucleoside phosphorylase | 0,613 | Nucleus | enzyme |
| PNPLA3 | ENSG00000100344 | patatin like phospholipase domain containing 3 | -1,235 | Cytoplasm | enzyme |
| PNPLA4 | ENSG00000006757 | patatin like phospholipase domain containing 4 | -0,935 | Cytoplasm | enzyme |
| PNPT1 | ENSG00000138035 | polyribonucleotide nucleotidyltransferase 1 | 1,814 | Cytoplasm | enzyme |
| PNRC1 | ENSG00000146278 | proline rich nuclear receptor coactivator 1 | 1,535 | Nucleus | other |
| PNRC2 | ENSG00000189266 | proline rich nuclear receptor coactivator 2 | 1,996 | Nucleus | other |
| POC1B-GALNT4 | ENSG00000259075 | POC1B-GALNT4 readthrough | -1,429 | Other | other |
| POC5 | ENSG00000152359 | POC5 centriolar protein | 0,718 | Cytoplasm | other |
| PODXL | ENSG00000128567 | podocalyxin like | 1,494 | Plasma Membrane | kinase |
| POLD2 | ENSG00000106628 | DNA polymerase delta 2, accessory subunit | -0,841 | Nucleus | enzyme |
| POLD4 | ENSG00000175482 | DNA polymerase delta 4, accessory subunit | -0,968 | Nucleus | enzyme |
| POLR2J2/POLR2J3 | ENSG00000267645 | RNA polymerase II subunit J2 | -0,646 | Other | transcription regulator |
| POLR3D | ENSG00000168495 | RNA polymerase III subunit D | 0,858 | Nucleus | transcription regulator |
| POU2F2 | ENSG00000028277 | POU class 2 homeobox 2 | 1,360 | Nucleus | transcription regulator |
| POU2F3 | ENSG00000137709 | POU class 2 homeobox 3 | 0,669 | Nucleus | transcription regulator |
| PPA2 | ENSG00000138777 | inorganic pyrophosphatase 2 | -0,744 | Cytoplasm | enzyme |
| PPAN | ENSG00000130810 | peter pan homolog | 0,674 | Nucleus | other |
| PPARGC1A | ENSG00000109819 | PPARG coactivator 1 alpha | 0,734 | Nucleus | transcription regulator |
| PPIL4 | ENSG00000131013 | peptidylprolyl isomerase like 4 | 0,751 | Nucleus | enzyme |
| PPM1D | ENSG00000170836 | protein phosphatase, Mg2+/Mn2+ dependent 1D | 0,709 | Cytoplasm | phosphatase |
| PPM1K | ENSG00000163644 | protein phosphatase, Mg2+/Mn2+ dependent 1K | 3,263 | Cytoplasm | phosphatase |
| PPOX | ENSG00000143224 | protoporphyrinogen oxidase | -0,864 | Cytoplasm | enzyme |
| PPP1R10 | ENSG00000204569 | protein phosphatase 1 regulatory subunit 10 | 0,941 | Nucleus | other |
| PPP1R15A | ENSG00000087074 | protein phosphatase 1 regulatory subunit 15A | 5,791 | Cytoplasm | other |
| PPP1R15B | ENSG00000158615 | protein phosphatase 1 regulatory subunit 15B | 1,026 | Cytoplasm | phosphatase |
| PPP1R18 | ENSG00000146112 | protein phosphatase 1 regulatory subunit 18 | 0,630 | Other | other |
| PPP1R7 | ENSG00000115685 | protein phosphatase 1 regulatory subunit 7 | -0,692 | Nucleus | phosphatase |
| PPP2R2A | ENSG00000221914 | protein phosphatase 2 regulatory subunit Balpha | 0,887 | Cytoplasm | phosphatase |
| PPP2R3A | ENSG00000073711 | protein phosphatase 2 regulatory subunit B''alpha | -0,761 | Nucleus | phosphatase |
| PPP3CA | ENSG00000138814 | protein phosphatase 3 catalytic subunit alpha | -0,803 | Cytoplasm | phosphatase |
| PPP4R1L | ENSG00000124224 | protein phosphatase 4 regulatory subunit 1 like (pseudogene) | 0,797 | Other | other |
| PPP4R4 | ENSG00000119698 | protein phosphatase 4 regulatory subunit 4 | 2,286 | Cytoplasm | other |
| PPT1 | ENSG00000131238 | palmitoyl-protein thioesterase 1 | -0,610 | Cytoplasm | enzyme |
| PPTC7 | ENSG00000196850 | protein phosphatase targeting COQ7 | 0,860 | Cytoplasm | phosphatase |
| PRADC1 | ENSG00000135617 | protease associated domain containing 1 | -1,169 | Extracellular Space | other |
| PRAF2 | ENSG00000243279 | PRA1 domain family member 2 | -0,728 | Other | other |
| PRDM1 | ENSG00000057657 | PR/SET domain 1 | 1,800 | Nucleus | transcription regulator |
| PRDM11 | ENSG00000019485 | PR/SET domain 11 | 0,673 | Other | other |
| PRDM6 | ENSG00000061455 | PR/SET domain 6 | 0,917 | Nucleus | transcription regulator |
| PRDM8 | ENSG00000152784 | PR/SET domain 8 | 0,875 | Nucleus | transcription regulator |
| PRDX2 | ENSG00000167815 | peroxiredoxin 2 | -0,772 | Cytoplasm | enzyme |
| PRDX3 | ENSG00000165672 | peroxiredoxin 3 | -0,667 | Cytoplasm | enzyme |
| PRDX5 | ENSG00000126432 | peroxiredoxin 5 | -0,614 | Cytoplasm | enzyme |
| PRICKLE3 | ENSG00000012211 | prickle planar cell polarity protein 3 | 1,782 | Other | other |
| PRKACA | ENSG00000072062 | protein kinase cAMP-activated catalytic subunit alpha | -0,935 | Cytoplasm | kinase |
| PRKACB | ENSG00000142875 | protein kinase cAMP-activated catalytic subunit beta | -0,930 | Cytoplasm | kinase |
| PRKAR2A | ENSG00000114302 | protein kinase cAMP-dependent type II regulatory subunit alpha | -0,775 | Cytoplasm | kinase |
| PRKCE | ENSG00000171132 | protein kinase C epsilon | 0,641 | Cytoplasm | kinase |
| PRKCQ | ENSG00000065675 | protein kinase C theta | 0,647 | Cytoplasm | kinase |
| PRKD2 | ENSG00000105287 | protein kinase D2 | 2,084 | Cytoplasm | kinase |
| PRKDC | ENSG00000253729 | protein kinase, DNA-activated, catalytic subunit | -0,957 | Nucleus | kinase |
| PROCR | ENSG00000101000 | protein C receptor | 0,711 | Plasma Membrane | other |
| PROM2 | ENSG00000155066 | prominin 2 | -1,125 | Plasma Membrane | transmembrane receptor |
| PRORSD1P | ENSG00000162997 | prolyl-tRNA synthetase associated domain containing 1, pseudogene | 0,996 | Other | other |
| PROS1 | ENSG00000184500 | protein S | -0,988 | Extracellular Space | other |
| PRPS1P2 | ENSG00000232630 | phosphoribosyl pyrophosphate synthetase 1 pseudogene 2 | 1,088 | Other | other |
| PRR11 | ENSG00000068489 | proline rich 11 | -1,283 | Other | other |
| PRR15 | ENSG00000176532 | proline rich 15 | 1,377 | Other | other |
| PRR3 | ENSG00000229202 | proline rich 3 | 0,794 | Other | other |
| PRRC2A | ENSG00000226618 | proline rich coiled-coil 2A | 0,806 | Cytoplasm | other |
| PRSS16 | ENSG00000112812 | serine protease 16 | -0,737 | Extracellular Space | peptidase |
| PRSS3 | ENSG00000010438 | serine protease 3 | -0,763 | Extracellular Space | peptidase |
| PRSS33 | ENSG00000103355 | serine protease 33 | -0,742 | Extracellular Space | peptidase |
| PRX | ENSG00000105227 | periaxin | -0,757 | Nucleus | other |
| PRXL2A | ENSG00000122378 | peroxiredoxin like 2A | -0,729 | Extracellular Space | other |
| PSAP | ENSG00000197746 | prosaposin | -0,707 | Extracellular Space | enzyme |
| PSAT1 | ENSG00000135069 | phosphoserine aminotransferase 1 | 0,728 | Cytoplasm | enzyme |
| PSMA6 | ENSG00000100902 | proteasome 20S subunit alpha 6 | 0,825 | Cytoplasm | peptidase |
| PSMB10 | ENSG00000205220 | proteasome 20S subunit beta 10 | 1,539 | Cytoplasm | peptidase |
| PSMB8 | ENSG00000230034 | proteasome 20S subunit beta 8 | 1,754 | Cytoplasm | peptidase |
| PSMB9 | ENSG00000242711 | proteasome 20S subunit beta 9 | 3,254 | Cytoplasm | peptidase |
| PSMC1P5 | ENSG00000250273 | | -3,034 | Other | other |
| PSMC3IP | ENSG00000131470 | PSMC3 interacting protein | 0,655 | Nucleus | transcription regulator |
| PSMD10 | ENSG00000101843 | proteasome 26S subunit, non-ATPase 10 | -0,783 | Cytoplasm | transcription regulator |
| PSME1 | ENSG00000092010 | proteasome activator subunit 1 | 1,104 | Cytoplasm | other |
| PSME2 | ENSG00000100911 | proteasome activator subunit 2 | 1,235 | Cytoplasm | peptidase |
| PSORS1C1 | ENSG00000206458 | psoriasis susceptibility 1 candidate 1 | 1,071 | Other | other |
| PSPH | ENSG00000146733 | phosphoserine phosphatase | 0,757 | Cytoplasm | phosphatase |
| PSRC1 | ENSG00000134222 | proline and serine rich coiled-coil 1 | -1,058 | Cytoplasm | other |
| PTGER2 | ENSG00000125384 | prostaglandin E receptor 2 | 1,845 | Plasma Membrane | G-protein coupled receptor |
| PTGER4 | ENSG00000171522 | prostaglandin E receptor 4 | 3,065 | Plasma Membrane | G-protein coupled receptor |
| PTGR1 | ENSG00000106853 | prostaglandin reductase 1 | -0,674 | Cytoplasm | enzyme |
| PTGR2 | ENSG00000140043 | prostaglandin reductase 2 | -0,769 | Cytoplasm | enzyme |
| PTGS1 | ENSG00000095303 | prostaglandin-endoperoxide synthase 1 | 0,818 | Cytoplasm | enzyme |

| | | | | | |
|---|---|---|---|---|---|
| PTGS2 | ENSG00000073756 | prostaglandin-endoperoxide synthase 2 | 2,124 | Cytoplasm | enzyme |
| PTMS | ENSG00000159335 | parathymosin | -0,745 | Nucleus | other |
| PTN | ENSG00000105894 | pleiotrophin | 1,385 | Extracellular Space | growth factor |
| PTP4A1 | ENSG00000112245 | protein tyrosine phosphatase 4A1 | 0,614 | Cytoplasm | phosphatase |
| PTPN18 | ENSG00000072135 | protein tyrosine phosphatase non-receptor type 18 | -0,857 | Nucleus | phosphatase |
| PTPN20 | ENSG00000204179 | protein tyrosine phosphatase non-receptor type 20 | 1,238 | Extracellular Space | phosphatase |
| PTPRB | ENSG00000127329 | protein tyrosine phosphatase receptor type B | -0,802 | Plasma Membrane | phosphatase |
| PTPRF | ENSG00000142949 | protein tyrosine phosphatase receptor type F | -0,962 | Plasma Membrane | phosphatase |
| PTPRS | ENSG00000105426 | protein tyrosine phosphatase receptor type S | -0,809 | Plasma Membrane | phosphatase |
| PTPRU | ENSG00000060656 | protein tyrosine phosphatase receptor type U | -1,089 | Plasma Membrane | phosphatase |
| PTPRZ1 | ENSG00000106278 | protein tyrosine phosphatase receptor type Z1 | 2,091 | Plasma Membrane | phosphatase |
| PTTG1 | ENSG00000164611 | PTTG1 regulator of sister chromatid separation, securin | -0,928 | Nucleus | transcription regulator |
| PTTG1IP | ENSG00000183255 | PTTG1 interacting protein | -0,754 | Nucleus | other |
| PTX3 | ENSG00000163661 | pentraxin 3 | 3,766 | Extracellular Space | other |
| PUS7 | ENSG00000091127 | pseudouridine synthase 7 | 0,716 | Nucleus | enzyme |
| PXK | ENSG00000168297 | PX domain containing serine/threonine kinase like | 1,105 | Cytoplasm | kinase |
| PXMP4 | ENSG00000101417 | peroxisomal membrane protein 4 | -1,167 | Cytoplasm | other |
| PYGL | ENSG00000100504 | glycogen phosphorylase L | -0,877 | Cytoplasm | enzyme |
| PYHIN1 | ENSG00000163564 | pyrin and HIN domain family member 1 | 1,401 | Nucleus | other |
| QARS1 | ENSG00000172053 | glutaminyl-tRNA synthetase 1 | -0,633 | Cytoplasm | enzyme |
| QKI | ENSG00000112531 | QKI, KH domain containing RNA binding | 0,764 | Nucleus | other |
| QPCT | ENSG00000115828 | glutaminyl-peptide cyclotransferase | -0,780 | Cytoplasm | enzyme |
| QPCTL | ENSG00000011478 | glutaminyl-peptide cyclotransferase like | -0,647 | Cytoplasm | enzyme |
| QRFPR | ENSG00000186867 | pyroglutamylated RFamide peptide receptor | -0,955 | Plasma Membrane | G-protein coupled receptor |
| RAB19 | ENSG00000146955 | RAB19, member RAS oncogene family | 1,344 | Cytoplasm | enzyme |
| RAB24 | ENSG00000169228 | RAB24, member RAS oncogene family | 1,394 | Cytoplasm | other |
| RAB26 | ENSG00000167964 | RAB26, member RAS oncogene family | -0,979 | Plasma Membrane | enzyme |
| RAB30 | ENSG00000137502 | RAB30, member RAS oncogene family | 1,501 | Cytoplasm | enzyme |
| RAB40B | ENSG00000141542 | RAB40B, member RAS oncogene family | -1,261 | Plasma Membrane | enzyme |
| RAB4A | ENSG00000168118 | RAB4A, member RAS oncogene family | -0,943 | Cytoplasm | enzyme |
| RAB6B | ENSG00000154917 | RAB6B, member RAS oncogene family | -1,054 | Cytoplasm | enzyme |
| RAB8B | ENSG00000166128 | RAB8B, member RAS oncogene family | 1,422 | Cytoplasm | enzyme |
| RABGAP1L | ENSG00000152061 | RAB GTPase activating protein 1 like | 1,095 | Cytoplasm | other |
| RAD52 | ENSG00000002016 | RAD52 homolog, DNA repair protein | 0,738 | Nucleus | other |
| RAET1L | ENSG00000155918 | retinoic acid early transcript 1L | 3,748 | Plasma Membrane | other |
| RALBP1 | ENSG00000017797 | ralA binding protein 1 | -0,636 | Cytoplasm | enzyme |
| RALGAPA1 | ENSG00000174373 | Ral GTPase activating protein catalytic subunit alpha 1 | 1,096 | Cytoplasm | other |
| RALGAPA2 | ENSG00000188559 | Ral GTPase activating protein catalytic subunit alpha 2 | -0,630 | Cytoplasm | other |
| RALGPS1 | ENSG00000136828 | Ral GEF with PH domain and SH3 binding motif 1 | -0,844 | Cytoplasm | other |
| RAMP1 | ENSG00000132329 | receptor activity modifying protein 1 | -1,572 | Plasma Membrane | G-protein coupled receptor |
| RANBP17 | ENSG00000204764 | RAN binding protein 17 | -1,037 | Nucleus | transporter |
| RANBP3L | ENSG00000164188 | RAN binding protein 3 like | 2,071 | Other | other |
| RAPGEF2 | ENSG00000109756 | Rap guanine nucleotide exchange factor 2 | 0,754 | Cytoplasm | other |
| RARRES1 | ENSG00000118849 | retinoic acid receptor responder 1 | -0,779 | Plasma Membrane | other |
| RASAL1 | ENSG00000111344 | RAS protein activator like 1 | -0,915 | Cytoplasm | other |
| RASD1 | ENSG00000108551 | ras related dexamethasone induced 1 | 1,081 | Cytoplasm | enzyme |
| RASGEF1B | ENSG00000138670 | RasGEF domain family member 1B | 1,848 | Other | other |
| RASGRF2 | ENSG00000113319 | Ras protein specific guanine nucleotide releasing factor 2 | 0,709 | Cytoplasm | other |
| RASGRP1 | ENSG00000172575 | RAS guanyl releasing protein 1 | 0,981 | Cytoplasm | other |
| RASGRP3 | ENSG00000152689 | RAS guanyl releasing protein 3 | 3,447 | Cytoplasm | other |
| RASSF10 | ENSG00000189431 | Ras association domain family member 10 | 0,676 | Other | other |
| RBAK | ENSG00000146587 | RB associated KRAB zinc finger | 0,608 | Nucleus | transcription regulator |
| RBBP6 | ENSG00000122257 | RB binding protein 6, ubiquitin ligase | 0,840 | Nucleus | enzyme |
| RBBP8NL | ENSG00000130701 | RBBP8 N-terminal like | -0,910 | Extracellular Space | other |
| RBCK1 | ENSG00000125826 | RANBP2-type and C3HC4-type zinc finger containing 1 | 1,152 | Cytoplasm | transcription regulator |
| RBL2 | ENSG00000103479 | RB transcriptional corepressor like 2 | -0,829 | Nucleus | other |
| RBM11 | ENSG00000185272 | RNA binding motif protein 11 | 1,460 | Nucleus | other |
| RBM25 | ENSG00000119707 | RNA binding motif protein 25 | 0,680 | Nucleus | other |
| RBM33 | ENSG00000184863 | RNA binding motif protein 33 | 0,717 | Other | other |
| RBM39 | ENSG00000131051 | RNA binding motif protein 39 | 0,794 | Nucleus | transcription regulator |
| RBM43 | ENSG00000184898 | RNA binding motif protein 43 | 1,082 | Other | other |
| RBM48 | ENSG00000127993 | RNA binding motif protein 48 | 1,056 | Nucleus | other |
| RBM5 | ENSG00000003756 | RNA binding motif protein 5 | 1,024 | Nucleus | other |
| RBM7 | ENSG00000076053 | RNA binding motif protein 7 | 0,768 | Nucleus | other |
| RBMS2 | ENSG00000076067 | RNA binding motif single stranded interacting protein 2 | 1,276 | Nucleus | other |
| RBMS3 | ENSG00000144642 | RNA binding motif single stranded interacting protein 3 | 1,383 | Cytoplasm | other |
| RC3H1 | ENSG00000135870 | ring finger and CCCH-type domains 1 | 0,985 | Cytoplasm | enzyme |
| RCAN1 | ENSG00000159200 | regulator of calcineurin 1 | 1,921 | Nucleus | transcription regulator |
| RCN2 | ENSG00000117906 | reticulocalbin 2 | -0,668 | Cytoplasm | other |
| RDH10 | ENSG00000121039 | retinol dehydrogenase 10 | -0,743 | Nucleus | enzyme |
| RDH5 | ENSG00000135437 | retinol dehydrogenase 5 | -2,062 | Cytoplasm | enzyme |
| REC8 | ENSG00000100918 | REC8 meiotic recombination protein | 0,780 | Nucleus | other |
| REEP1 | ENSG00000068615 | receptor accessory protein 1 | -0,728 | Cytoplasm | other |
| REEP6 | ENSG00000115255 | receptor accessory protein 6 | -1,262 | Plasma Membrane | other |
| REL | ENSG00000162924 | REL proto-oncogene, NF-kB subunit | 1,789 | Nucleus | transcription regulator |
| RELB | ENSG00000104856 | RELB proto-oncogene, NF-kB subunit | 2,006 | Nucleus | transcription regulator |
| RELT | ENSG00000054967 | RELT TNF receptor | 0,626 | Plasma Membrane | transmembrane receptor |
| REPS2 | ENSG00000169891 | RALBP1 associated Eps domain containing 2 | -1,337 | Cytoplasm | other |
| RERG | ENSG00000134533 | RAS like estrogen regulated growth inhibitor | -1,686 | Nucleus | enzyme |
| RERGL | ENSG00000111404 | RERG like | -1,161 | Other | other |
| RET | ENSG00000165731 | ret proto-oncogene | 1,103 | Plasma Membrane | kinase |
| REV3L | ENSG00000009413 | REV3 like, DNA directed polymerase zeta catalytic subunit | 1,180 | Nucleus | enzyme |
| REXO1 | ENSG00000079313 | RNA exonuclease 1 homolog | 0,952 | Nucleus | transcription regulator |
| RFPL2 | ENSG00000128253 | ret finger protein like 2 | 1,352 | Other | other |
| RFX5 | ENSG00000143390 | regulatory factor X5 | 0,859 | Nucleus | transcription regulator |
| RGMB | ENSG00000174136 | repulsive guidance molecule BMP co-receptor b | 1,192 | Plasma Membrane | other |
| RGPD3 (includes others) | ENSG00000169629 | RANBP2 like and GRIP domain containing 3 | 1,242 | Nucleus | enzyme |
| RGS16 | ENSG00000143333 | regulator of G protein signaling 16 | 0,947 | Cytoplasm | enzyme |
| RGS20 | ENSG00000147509 | regulator of G protein signaling 20 | 1,282 | Cytoplasm | enzyme |
| RHBDF2 | ENSG00000129667 | rhomboid 5 homolog 2 | 0,853 | Cytoplasm | other |
| RHEBL1 | ENSG00000167550 | RHEB like 1 | 1,719 | Cytoplasm | enzyme |
| RHNO1 | ENSG00000171792 | RAD9-HUS1-RAD1 interacting nuclear orphan 1 | -0,635 | Nucleus | other |
| RIC1 | ENSG00000107036 | RIC1 homolog, RAB6A GEF complex partner 1 | 0,675 | Cytoplasm | other |
| RICTOR | ENSG00000164327 | RPTOR independent companion of MTOR complex 2 | 1,259 | Cytoplasm | other |
| RILP | ENSG00000167705 | Rab interacting lysosomal protein | 1,048 | Cytoplasm | other |
| RIMKLB | ENSG00000166532 | ribosomal modification protein rimK like family member B | 0,808 | Cytoplasm | enzyme |
| RING1 | ENSG00000235107 | ring finger protein 1 | -2,986 | Nucleus | transcription regulator |
| RINT1 | ENSG00000135249 | RAD50 interactor 1 | 0,714 | Nucleus | other |
| RIOK1 | ENSG00000124784 | RIO kinase 1 | 0,600 | Cytoplasm | kinase |

| | | | | | |
|---|---|---|---|---|---|
| RIOK2 | ENSG00000058729 | RIO kinase 2 | 0,625 | Cytoplasm | kinase |
| RIOK3 | ENSG00000101782 | RIO kinase 3 | 0,724 | Cytoplasm | kinase |
| RIPK1 | ENSG00000137275 | receptor interacting serine/threonine kinase 1 | 1,060 | Plasma Membrane | kinase |
| RIPK2 | ENSG00000104312 | receptor interacting serine/threonine kinase 2 | 1,586 | Plasma Membrane | kinase |
| RIPOR2 | ENSG00000111913 | RHO family interacting cell polarization regulator 2 | 3,997 | Cytoplasm | other |
| RLF | ENSG00000117000 | RLF zinc finger | 1,007 | Nucleus | transcription regulator |
| RLIM | ENSG00000131263 | ring finger protein, LIM domain interacting | 1,120 | Nucleus | enzyme |
| RNASE10 | ENSG00000182545 | ribonuclease A family member 10 (inactive) | 1,401 | Extracellular Space | other |
| RNASE4 | ENSG00000258818 | ribonuclease A family member 4 | -1,381 | Extracellular Space | enzyme |
| RNASEH2B | ENSG00000136104 | ribonuclease H2 subunit B | -0,770 | Nucleus | enzyme |
| RNASEK | ENSG00000219200 | ribonuclease K | -0,613 | Other | peptidase |
| RNASEK-C17orf49 | ENSG00000161939 | RNASEK-C17orf49 readthrough | 2,299 | Other | other |
| RNASEL | ENSG00000135828 | ribonuclease L | 0,950 | Cytoplasm | enzyme |
| RND1 | ENSG00000172602 | Rho family GTPase 1 | 2,948 | Cytoplasm | enzyme |
| RND3 | ENSG00000115963 | Rho family GTPase 3 | 1,415 | Cytoplasm | enzyme |
| RNF111 | ENSG00000157450 | ring finger protein 111 | 0,786 | Nucleus | enzyme |
| RNF122 | ENSG00000133874 | ring finger protein 122 | 0,836 | Cytoplasm | enzyme |
| RNF128 | ENSG00000133135 | ring finger protein 128 | -0,825 | Cytoplasm | enzyme |
| RNF130 | ENSG00000113269 | ring finger protein 130 | -0,925 | Cytoplasm | peptidase |
| RNF144A | ENSG00000151692 | ring finger protein 144A | 0,903 | Nucleus | enzyme |
| RNF149 | ENSG00000163162 | ring finger protein 149 | 1,154 | Cytoplasm | enzyme |
| RNF187 | ENSG00000168159 | ring finger protein 187 | -0,853 | Nucleus | enzyme |
| RNF19B | ENSG00000116514 | ring finger protein 19B | 1,856 | Cytoplasm | enzyme |
| RNF2 | ENSG00000121481 | ring finger protein 2 | 0,678 | Nucleus | transcription regulator |
| RNF213 | ENSG00000173821 | ring finger protein 213 | 2,478 | Cytoplasm | enzyme |
| RNF223 | ENSG00000237330 | ring finger protein 223 | 0,950 | Other | other |
| RNF25 | ENSG00000163481 | ring finger protein 25 | 0,644 | Nucleus | transcription regulator |
| RNF31 | ENSG00000092098 | ring finger protein 31 | 0,631 | Cytoplasm | enzyme |
| RNLS | ENSG00000184719 | renalase, FAD dependent amine oxidase | -0,643 | Extracellular Space | enzyme |
| RNPEP | ENSG00000176393 | arginyl aminopeptidase | -0,707 | Cytoplasm | peptidase |
| RNPEPL1 | ENSG00000142327 | arginyl aminopeptidase like 1 | -0,641 | Other | peptidase |
| ROGDI | ENSG00000067836 | rogdi atypical leucine zipper | -0,824 | Nucleus | other |
| ROR1 | ENSG00000185483 | receptor tyrosine kinase like orphan receptor 1 | -0,841 | Plasma Membrane | kinase |
| ROS1 | ENSG00000047936 | ROS proto-oncogene 1, receptor tyrosine kinase | -1,499 | Plasma Membrane | kinase |
| RP11-347C12.3 | ENSG00000258130 | | -2,201 | Other | other |
| RP11-468E2.1 | ENSG00000254692 | | -1,008 | Other | other |
| RP11-561B11.2 | ENSG00000258790 | | -1,608 | Other | other |
| RP11-73M18.2 | ENSG00000256500 | | -0,794 | Other | other |
| RP11-986E7.7 | ENSG00000273259 | | -0,942 | Other | other |
| RP11_1035H133 | ENSG00000260342 | | -1,200 | Other | other |
| RP11_293I142 | ENSG00000258064 | | -2,108 | Other | other |
| RP11_295K31 | ENSG00000250644 | | -1,427 | Other | other |
| RP11_438J11 | ENSG00000272410 | | 1,262 | Other | other |
| RP11_463D192 | ENSG00000258677 | | -1,150 | Other | other |
| RP11_514P86 | ENSG00000205236 | | -0,643 | Other | other |
| RP11_61N203 | ENSG00000226268 | | 2,139 | Other | other |
| RP11_719K43 | ENSG00000254609 | | -2,177 | Other | other |
| RP11_736I102 | ENSG00000255331 | | 0,868 | Other | other |
| RP11_977G1910 | ENSG00000144785 | | -1,192 | Other | other |
| RP13-582O9.6 | ENSG00000264668 | | 0,697 | Other | other |
| RP5_1021I204 | ENSG00000258653 | | 0,955 | Other | other |
| RPL10P13 | ENSG00000258245 | ribosomal protein L10 pseudogene 13 | 1,139 | Other | other |
| RPL3P2 | ENSG00000227656 | ribosomal protein L3 pseudogene 2 | -0,880 | Other | other |
| RPLP0 | ENSG00000089157 | ribosomal protein lateral stalk subunit P0 | -0,754 | Cytoplasm | other |
| RPP38 | ENSG00000152464 | ribonuclease P/MRP subunit p38 | 0,707 | Nucleus | enzyme |
| RPS18P9 | ENSG00000220848 | ribosomal protein S18 pseudogene 9 | 0,823 | Other | other |
| RPS27L | ENSG00000185088 | ribosomal protein S27 like | -0,669 | Cytoplasm | translation regulator |
| RPS3 | ENSG00000149273 | ribosomal protein S3 | -0,622 | Cytoplasm | enzyme |
| RPS3A | ENSG00000145425 | ribosomal protein S3A | -0,604 | Nucleus | other |
| RPS4X | ENSG00000198034 | ribosomal protein S4 X-linked | -0,600 | Cytoplasm | other |
| RPS6KC1 | ENSG00000136643 | ribosomal protein S6 kinase C1 | 1,620 | Cytoplasm | kinase |
| RPTOR | ENSG00000141564 | regulatory associated protein of MTOR complex 1 | 0,602 | Cytoplasm | other |
| RRAD | ENSG00000166592 | RRAD, Ras related glycolysis inhibitor and calcium channel regulator | 1,592 | Cytoplasm | enzyme |
| RRM1 | ENSG00000167325 | ribonucleotide reductase catalytic subunit M1 | -0,647 | Nucleus | enzyme |
| RSAD1 | ENSG00000136444 | radical S-adenosyl methionine domain containing 1 | -0,849 | Cytoplasm | other |
| RSAD2 | ENSG00000134321 | radical S-adenosyl methionine domain containing 2 | 5,770 | Cytoplasm | enzyme |
| RSC1A1 | ENSG00000215695 | regulator of solute carriers 1 | 0,766 | Nucleus | other |
| RSF1 | ENSG00000048649 | remodeling and spacing factor 1 | 0,974 | Nucleus | transcription regulator |
| RSPH1 | ENSG00000160188 | radial spoke head component 1 | -1,185 | Nucleus | other |
| RSPRY1 | ENSG00000159579 | ring finger and SPRY domain containing 1 | 0,812 | Other | other |
| RSRC2 | ENSG00000111011 | arginine and serine rich coiled-coil 2 | 1,351 | Other | other |
| RTKN2 | ENSG00000182010 | rhotekin 2 | 1,068 | Plasma Membrane | other |
| RTL9 | ENSG00000243978 | retrotransposon Gag like 9 | 4,052 | Other | other |
| RTN3 | ENSG00000133318 | reticulon 3 | -0,792 | Cytoplasm | other |
| RTP4 | ENSG00000136514 | receptor transporter protein 4 | 4,160 | Plasma Membrane | other |
| RUBCN | ENSG00000145016 | rubicon autophagy regulator | 0,888 | Cytoplasm | other |
| RUFY4 | ENSG00000188282 | RUN and FYVE domain containing 4 | 1,469 | Other | other |
| RUNX2 | ENSG00000124813 | RUNX family transcription factor 2 | 0,878 | Nucleus | transcription regulator |
| RYR2 | ENSG00000198626 | ryanodine receptor 2 | 0,877 | Plasma Membrane | ion channel |
| S100A1 | ENSG00000160678 | S100 calcium binding protein A1 | 0,657 | Cytoplasm | other |
| S100P | ENSG00000163993 | S100 calcium binding protein P | 1,429 | Cytoplasm | other |
| S1PR3 | ENSG00000213694 | sphingosine-1-phosphate receptor 3 | 0,858 | Plasma Membrane | G-protein coupled receptor |
| SAA1 | ENSG00000173432 | serum amyloid A1 | 2,157 | Extracellular Space | transporter |
| SAA2 | ENSG00000134339 | serum amyloid A2 | 1,880 | Extracellular Space | other |
| SAMD1 | ENSG00000141858 | sterile alpha motif domain containing 1 | -0,660 | Other | other |
| SAMD13 | ENSG00000203943 | sterile alpha motif domain containing 13 | -1,196 | Other | other |
| SAMD4A | ENSG00000020577 | sterile alpha motif domain containing 4A | 1,004 | Cytoplasm | translation regulator |
| SAMD9 | ENSG00000205413 | sterile alpha motif domain containing 9 | 3,048 | Cytoplasm | other |
| SAMD9L | ENSG00000177409 | sterile alpha motif domain containing 9 like | 4,256 | Extracellular Space | other |
| SAMHD1 | ENSG00000101347 | SAM and HD domain containing deoxynucleoside triphosphate triphosphohydrolase 1 | 2,744 | Nucleus | enzyme |
| SAP30 | ENSG00000164105 | Sin3A associated protein 30 | -0,723 | Nucleus | transcription regulator |
| SAPCD2 | ENSG00000186193 | suppressor APC domain containing 2 | -1,024 | Nucleus | other |
| SAR1A | ENSG00000079332 | secretion associated Ras related GTPase 1A | 0,940 | Cytoplasm | enzyme |
| SARAF | ENSG00000133872 | store-operated calcium entry associated regulatory factor | -0,635 | Cytoplasm | other |
| SAT1 | ENSG00000130066 | spermidine/spermine N1-acetyltransferase 1 | 1,689 | Cytoplasm | enzyme |
| SATB2 | ENSG00000119042 | SATB homeobox 2 | 1,195 | Nucleus | transcription regulator |
| SAV1 | ENSG00000151748 | salvador family WW domain containing protein 1 | 0,627 | Cytoplasm | other |
| SBNO2 | ENSG00000064932 | strawberry notch homolog 2 | 0,887 | Other | transcription regulator |
| SC5D | ENSG00000109929 | sterol-C5-desaturase | -1,130 | Cytoplasm | enzyme |

| | | | | | |
|---|---|---|---|---|---|
| SCAF8 | ENSG00000213079 | SR-related CTD associated factor 8 | 0,621 | Nucleus | other |
| SCARB2 | ENSG00000138760 | scavenger receptor class B member 2 | 0,690 | Plasma Membrane | transmembrane receptor |
| SCCPDH | ENSG00000143653 | saccharopine dehydrogenase (putative) | -0,763 | Cytoplasm | other |
| SCD | ENSG00000099194 | stearoyl-CoA desaturase | -1,644 | Cytoplasm | enzyme |
| SCEL | ENSG00000136155 | sciellin | -0,671 | Cytoplasm | other |
| SCGN | ENSG00000079689 | secretagogin, EF-hand calcium binding protein | -1,928 | Cytoplasm | other |
| SCIN | ENSG00000006747 | scinderin | 0,668 | Cytoplasm | other |
| SCN3A | ENSG00000153253 | sodium voltage-gated channel alpha subunit 3 | 5,105 | Plasma Membrane | ion channel |
| SCNN1A | ENSG00000111319 | sodium channel epithelial 1 subunit alpha | -0,746 | Plasma Membrane | ion channel |
| SCP2 | ENSG00000116171 | sterol carrier protein 2 | -0,850 | Cytoplasm | transporter |
| SCRN3 | ENSG00000144306 | secernin 3 | -0,705 | Other | other |
| SDC1 | ENSG00000115884 | syndecan 1 | -1,112 | Plasma Membrane | enzyme |
| SDC4 | ENSG00000124145 | syndecan 4 | 1,372 | Plasma Membrane | other |
| SDCBP2 | ENSG00000125775 | syndecan binding protein 2 | 1,090 | Cytoplasm | other |
| SDE2 | ENSG00000143751 | SDE2 telomere maintenance homolog | 0,781 | Nucleus | other |
| SDK1 | ENSG00000146555 | sidekick cell adhesion molecule 1 | -0,761 | Plasma Membrane | other |
| SDSL | ENSG00000139410 | serine dehydratase like | 0,690 | Cytoplasm | enzyme |
| SEC14L2 | ENSG00000100003 | SEC14 like lipid binding 2 | -0,741 | Cytoplasm | transporter |
| SEC16B | ENSG00000120341 | SEC16 homolog B, endoplasmic reticulum export factor | 2,436 | Nucleus | other |
| SEC24A | ENSG00000113615 | SEC24 homolog A, COPII coat complex component | 0,843 | Cytoplasm | transporter |
| SEC24D | ENSG00000150961 | SEC24 homolog D, COPII coat complex component | 1,042 | Cytoplasm | transporter |
| SEC63 | ENSG00000025796 | SEC63 homolog, protein translocation regulator | -0,710 | Cytoplasm | transporter |
| SECTM1 | ENSG00000141574 | secreted and transmembrane 1 | 3,669 | Extracellular Space | other |
| SELENOK | ENSG00000113811 | selenoprotein K | 0,755 | Cytoplasm | other |
| SELL | ENSG00000188404 | selectin L | 1,194 | Plasma Membrane | transmembrane receptor |
| SEMA3A | ENSG00000075213 | semaphorin 3A | 2,315 | Extracellular Space | other |
| SEMA3D | ENSG00000153993 | semaphorin 3D | 1,849 | Extracellular Space | other |
| SEMA3E | ENSG00000170381 | semaphorin 3E | -1,189 | Extracellular Space | other |
| SEMA4G | ENSG00000095539 | semaphorin 4G | -1,263 | Plasma Membrane | other |
| SEMA7A | ENSG00000138623 | semaphorin 7A (John Milton Hagen blood group) | 1,344 | Plasma Membrane | transmembrane receptor |
| SENP5 | ENSG00000119231 | SUMO specific peptidase 5 | 0,798 | Nucleus | peptidase |
| SEPHS1 | ENSG00000086475 | selenophosphate synthetase 1 | -0,758 | Other | enzyme |
| SEPSECS | ENSG00000109618 | Sep (O-phosphoserine) tRNA:Sec (selenocysteine) tRNA synthase | 0,984 | Cytoplasm | enzyme |
| SEPTIN9 | ENSG00000184640 | septin 9 | -0,865 | Cytoplasm | enzyme |
| SERF1A/SERF1B | ENSG00000172058 | small EDRK-rich factor 1A | 1,480 | Other | other |
| SERINC5 | ENSG00000164300 | serine incorporator 5 | -0,793 | Plasma Membrane | transporter |
| SERPINA1 | ENSG00000197249 | serpin family A member 1 | -0,636 | Extracellular Space | other |
| SERPINA3 | ENSG00000196136 | serpin family A member 3 | -1,104 | Extracellular Space | other |
| SERPINA4 | ENSG00000100665 | serpin family A member 4 | -1,230 | Extracellular Space | other |
| SERPINA5 | ENSG00000188488 | serpin family A member 5 | -0,894 | Extracellular Space | other |
| SERPINB1 | ENSG00000021355 | serpin family B member 1 | 0,683 | Cytoplasm | other |
| SERPINB2 | ENSG00000197632 | serpin family B member 2 | 3,348 | Extracellular Space | other |
| SERPINB8 | ENSG00000166401 | serpin family B member 8 | 0,646 | Cytoplasm | other |
| SERPINB9 | ENSG00000170542 | serpin family B member 9 | 1,458 | Cytoplasm | other |
| SERPINE1 | ENSG00000106366 | serpin family E member 1 | 0,951 | Extracellular Space | other |
| SERPING1 | ENSG00000149131 | serpin family G member 1 | 0,618 | Extracellular Space | other |
| SERTAD1 | ENSG00000197019 | SERTA domain containing 1 | 1,236 | Nucleus | transcription regulator |
| SERTAD2 | ENSG00000179833 | SERTA domain containing 2 | 0,843 | Cytoplasm | transcription regulator |
| SERTAD4 | ENSG00000082497 | SERTA domain containing 4 | -0,866 | Other | other |
| SESN2 | ENSG00000130766 | sestrin 2 | 1,318 | Cytoplasm | enzyme |
| SETD5 | ENSG00000168137 | SET domain containing 5 | 0,771 | Nucleus | enzyme |
| SETD9 | ENSG00000155542 | SET domain containing 9 | -0,965 | Nucleus | enzyme |
| SETDB1 | ENSG00000143379 | SET domain bifurcated histone lysine methyltransferase 1 | 0,654 | Nucleus | enzyme |
| SEZ6L2 | ENSG00000174938 | seizure related 6 homolog like 2 | -0,649 | Cytoplasm | other |
| SF3B5 | ENSG00000169976 | splicing factor 3b subunit 5 | -0,659 | Nucleus | other |
| SFR1 | ENSG00000156384 | SWI5 dependent homologous recombination repair protein 1 | 0,664 | Nucleus | transcription regulator |
| SFSWAP | ENSG00000061936 | splicing factor SWAP | 0,695 | Nucleus | other |
| SFXN3 | ENSG00000107819 | sideroflexin 3 | -0,644 | Cytoplasm | transporter |
| SGCB | ENSG00000163069 | sarcoglycan beta | -0,712 | Plasma Membrane | other |
| SGK1 | ENSG00000118515 | serum/glucocorticoid regulated kinase 1 | 0,758 | Cytoplasm | kinase |
| SGO2 | ENSG00000163535 | shugoshin 2 | -0,732 | Nucleus | other |
| SGPP1 | ENSG00000126821 | sphingosine-1-phosphate phosphatase 1 | -0,839 | Cytoplasm | phosphatase |
| SH2B3 | ENSG00000111252 | SH2B adaptor protein 3 | 0,903 | Plasma Membrane | other |
| SH3BGRL2 | ENSG00000198478 | SH3 domain binding glutamate rich protein like 2 | -0,749 | Nucleus | other |
| SH3GL2 | ENSG00000107295 | SH3 domain containing GRB2 like 2, endophilin A1 | 0,832 | Plasma Membrane | enzyme |
| SH3KBP1 | ENSG00000147010 | SH3 domain containing kinase binding protein 1 | 1,005 | Cytoplasm | other |
| SH3TC1 | ENSG00000125089 | SH3 domain and tetratricopeptide repeats 1 | 1,283 | Extracellular Space | other |
| SH3YL1 | ENSG00000035115 | SH3 and SYLF domain containing 1 | -1,158 | Plasma Membrane | other |
| SHC2 | ENSG00000129946 | SHC adaptor protein 2 | -0,915 | Cytoplasm | other |
| SHFL | ENSG00000130813 | shiftless antiviral inhibitor of ribosomal frameshifting | 2,421 | Other | other |
| SHMT1 | ENSG00000176974 | serine hydroxymethyltransferase 1 | -0,859 | Cytoplasm | enzyme |
| SHOX2 | ENSG00000168779 | short stature homeobox 2 | 0,679 | Nucleus | transcription regulator |
| SHROOM2 | ENSG00000146950 | shroom family member 2 | 1,522 | Plasma Membrane | ion channel |
| SIAE | ENSG00000110013 | sialic acid acetylesterase | -0,974 | Cytoplasm | enzyme |
| SIDT1 | ENSG00000072858 | SID1 transmembrane family member 1 | 1,057 | Other | other |
| SIK1/SIK1B | ENSG00000142178 | salt inducible kinase 1 | 0,825 | Nucleus | kinase |
| SIK3 | ENSG00000160584 | SIK family kinase 3 | 0,821 | Cytoplasm | kinase |
| SIKE1 | ENSG00000052723 | suppressor of IKBKE 1 | 0,620 | Cytoplasm | other |
| SIM2 | ENSG00000159263 | SIM bHLH transcription factor 2 | 0,890 | Nucleus | transcription regulator |
| SIPA1L2 | ENSG00000116991 | signal induced proliferation associated 1 like 2 | 1,707 | Other | other |
| SIRPB2 | ENSG00000196209 | signal regulatory protein beta 2 | 0,970 | Other | other |
| SIRT1 | ENSG00000096717 | sirtuin 1 | 0,765 | Nucleus | transcription regulator |
| SIX1 | ENSG00000126778 | SIX homeobox 1 | 1,243 | Nucleus | transcription regulator |
| SIX4 | ENSG00000100625 | SIX homeobox 4 | 1,273 | Nucleus | transcription regulator |
| SKIV2L | ENSG00000223493 | Ski2 like RNA helicase | 1,005 | Nucleus | enzyme |
| SLC10A7 | ENSG00000120519 | solute carrier family 10 member 7 | 0,620 | Cytoplasm | transporter |
| SLC12A2 | ENSG00000064651 | solute carrier family 12 member 2 | -0,826 | Plasma Membrane | transporter |
| SLC12A7 | ENSG00000113504 | solute carrier family 12 member 7 | 1,222 | Plasma Membrane | transporter |
| SLC15A3 | ENSG00000110446 | solute carrier family 15 member 3 | 5,436 | Cytoplasm | transporter |
| SLC16A3 | ENSG00000141526 | solute carrier family 16 member 3 | 0,809 | Plasma Membrane | transporter |
| SLC16A5 | ENSG00000170190 | solute carrier family 16 member 5 | -0,938 | Plasma Membrane | transporter |
| SLC16A9 | ENSG00000165449 | solute carrier family 16 member 9 | 1,472 | Other | other |
| SLC1A3 | ENSG00000079215 | solute carrier family 1 member 3 | 3,907 | Plasma Membrane | transporter |
| SLC1A4 | ENSG00000115902 | solute carrier family 1 member 4 | 0,644 | Plasma Membrane | transporter |
| SLC22A18 | ENSG00000110628 | solute carrier family 22 member 18 | -0,852 | Plasma Membrane | transporter |
| SLC22A18AS | ENSG00000254827 | solute carrier family 22 member 18 antisense | -1,248 | Other | other |
| SLC22A3 | ENSG00000146477 | solute carrier family 22 member 3 | -0,922 | Plasma Membrane | transporter |
| SLC25A1 | ENSG00000100075 | solute carrier family 25 member 1 | -0,934 | Other | transporter |
| SLC25A10 | ENSG00000183048 | solute carrier family 25 member 10 | -0,603 | Cytoplasm | transporter |

| | | | | | |
|---|---|---|---|---|---|
| SLC25A21 | ENSG00000183032 | solute carrier family 25 member 21 | -1,112 | Cytoplasm | transporter |
| SLC25A22 | ENSG00000177542 | solute carrier family 25 member 22 | 0,785 | Cytoplasm | transporter |
| SLC25A23 | ENSG00000125648 | solute carrier family 25 member 23 | -0,787 | Cytoplasm | transporter |
| SLC25A25 | ENSG00000148339 | solute carrier family 25 member 25 | 1,078 | Cytoplasm | transporter |
| SLC25A28 | ENSG00000155287 | solute carrier family 25 member 28 | 1,978 | Cytoplasm | transporter |
| SLC25A42 | ENSG00000181035 | solute carrier family 25 member 42 | -0,602 | Cytoplasm | transporter |
| SLC25A51 | ENSG00000122696 | solute carrier family 25 member 51 | 0,639 | Cytoplasm | other |
| SLC26A2 | ENSG00000155850 | solute carrier family 26 member 2 | -0,825 | Plasma Membrane | transporter |
| SLC28A3 | ENSG00000197506 | solute carrier family 28 member 3 | 1,323 | Plasma Membrane | transporter |
| SLC29A2 | ENSG00000174669 | solute carrier family 29 member 2 | -0,622 | Plasma Membrane | transporter |
| SLC2A12 | ENSG00000146411 | solute carrier family 2 member 12 | 0,769 | Plasma Membrane | transporter |
| SLC2A13 | ENSG00000151229 | solute carrier family 2 member 13 | 0,648 | Plasma Membrane | transporter |
| SLC2A3 | ENSG00000059804 | solute carrier family 2 member 3 | 1,200 | Plasma Membrane | transporter |
| SLC30A3 | ENSG00000115194 | solute carrier family 30 member 3 | 1,360 | Plasma Membrane | transporter |
| SLC30A4 | ENSG00000104154 | solute carrier family 30 member 4 | 0,717 | Cytoplasm | transporter |
| SLC30A9 | ENSG00000014824 | solute carrier family 30 member 9 | -0,631 | Nucleus | transporter |
| SLC31A2 | ENSG00000136867 | solute carrier family 31 member 2 | 1,698 | Plasma Membrane | transporter |
| SLC35A5 | ENSG00000138459 | solute carrier family 35 member A5 | 0,652 | Other | transporter |
| SLC35E2A | ENSG00000215790 | solute carrier family 35 member E2A | -0,897 | Other | other |
| SLC35G2 | ENSG00000168917 | solute carrier family 35 member G2 | 0,976 | Cytoplasm | other |
| SLC36A4 | ENSG00000180773 | solute carrier family 36 member 4 | -0,631 | Plasma Membrane | transporter |
| SLC39A10 | ENSG00000196950 | solute carrier family 39 member 10 | -0,846 | Extracellular Space | transporter |
| SLC39A8 | ENSG00000138821 | solute carrier family 39 member 8 | -0,866 | Extracellular Space | transporter |
| SLC3A1 | ENSG00000138079 | solute carrier family 3 member 1 | -1,631 | Plasma Membrane | transporter |
| SLC3A2 | ENSG00000168003 | solute carrier family 3 member 2 | 1,025 | Plasma Membrane | transporter |
| SLC40A1 | ENSG00000138449 | solute carrier family 40 member 1 | -0,728 | Plasma Membrane | transporter |
| SLC44A3 | ENSG00000143036 | solute carrier family 44 member 3 | -1,014 | Plasma Membrane | transporter |
| SLC44A4 | ENSG00000231479 | solute carrier family 44 member 4 | -1,425 | Plasma Membrane | transporter |
| SLC49A4 | ENSG00000138463 | solute carrier family 49 member 4 | -0,675 | Other | other |
| SLC4A3 | ENSG00000114923 | solute carrier family 4 member 3 | -0,833 | Plasma Membrane | transporter |
| SLC50A1 | ENSG00000169241 | solute carrier family 50 member 1 | -0,896 | Plasma Membrane | transporter |
| SLC66A3 | ENSG00000162976 | solute carrier family 66 member 3 | -1,122 | Other | other |
| SLC6A13 | ENSG00000010379 | solute carrier family 6 member 13 | 1,427 | Plasma Membrane | transporter |
| SLC6A20 | ENSG00000163817 | solute carrier family 6 member 20 | -0,979 | Plasma Membrane | transporter |
| SLC6A9 | ENSG00000196517 | solute carrier family 6 member 9 | 1,634 | Plasma Membrane | transporter |
| SLC7A5P2 | ENSG00000258186 | solute carrier family 7 member 5 pseudogene 2 | -0,981 | Other | other |
| SLC8A2 | ENSG00000118160 | solute carrier family 8 member A2 | 1,092 | Cytoplasm | transporter |
| SLC9A8 | ENSG00000197818 | solute carrier family 9 member A8 | 0,845 | Cytoplasm | transporter |
| SLCO5A1 | ENSG00000137571 | solute carrier organic anion transporter family member 5A1 | 0,940 | Plasma Membrane | transporter |
| SLFN12L | ENSG00000205045 | schlafen family member 12 like | 0,626 | Other | enzyme |
| SLFN5 | ENSG00000166750 | schlafen family member 5 | 2,417 | Nucleus | enzyme |
| SLX1A/SLX1B | ENSG00000181625 | SLX1 homolog B, structure-specific endonuclease subunit | -0,816 | Nucleus | enzyme |
| SMAD6 | ENSG00000137834 | SMAD family member 6 | -0,730 | Nucleus | transcription regulator |
| SMARCA5 | ENSG00000153147 | SWI/SNF related, matrix associated, actin dependent regulator of chromatin, subfamily a, member 5 | 0,871 | Nucleus | transcription regulator |
| SMARCC1 | ENSG00000173473 | SWI/SNF related, matrix associated, actin dependent regulator of chromatin subfamily c member 1 | -0,680 | Nucleus | transcription regulator |
| SMC2 | ENSG00000136824 | structural maintenance of chromosomes 2 | -0,683 | Nucleus | transporter |
| SMCHD1 | ENSG00000101596 | structural maintenance of chromosomes flexible hinge domain containing 1 | 1,328 | Nucleus | enzyme |
| SMDT1 | ENSG00000272901 | single-pass membrane protein with aspartate rich tail 1 | -0,769 | Extracellular Space | other |
| SMIM10 | ENSG00000184785 | small integral membrane protein 10 | -1,028 | Other | other |
| SMIM11A | ENSG00000205670 | small integral membrane protein 11A | -1,226 | Plasma Membrane | other |
| SMIM14 | ENSG00000163683 | small integral membrane protein 14 | 0,851 | Cytoplasm | other |
| SMIM20 | ENSG00000250317 | small integral membrane protein 20 | -0,683 | Cytoplasm | other |
| SMIM30 | ENSG00000214194 | small integral membrane protein 30 | -2,183 | Other | other |
| SMOC1 | ENSG00000198732 | SPARC related modular calcium binding 1 | 0,781 | Extracellular Space | other |
| SMTNL1 | ENSG00000214872 | smoothelin like 1 | 1,816 | Cytoplasm | other |
| SMURF1 | ENSG00000198742 | SMAD specific E3 ubiquitin protein ligase 1 | 1,059 | Cytoplasm | enzyme |
| SNAI1 | ENSG00000124216 | snail family transcriptional repressor 1 | 1,186 | Nucleus | transcription regulator |
| SNCA | ENSG00000145335 | synuclein alpha | -1,518 | Cytoplasm | enzyme |
| SNIP1 | ENSG00000163877 | Smad nuclear interacting protein 1 | 1,261 | Nucleus | other |
| SNORC | ENSG00000182600 | secondary ossification center associated regulator of chondrocyte maturation | 1,590 | Cytoplasm | other |
| SNPH | ENSG00000101298 | syntaphilin | 2,506 | Plasma Membrane | other |
| SNRK | ENSG00000163788 | SNF related kinase | 0,854 | Cytoplasm | kinase |
| SNTA1 | ENSG00000101400 | syntrophin alpha 1 | -1,077 | Plasma Membrane | other |
| SNX16 | ENSG00000104497 | sorting nexin 16 | 0,933 | Cytoplasm | transporter |
| SNX30 | ENSG00000148158 | sorting nexin family member 30 | -0,645 | Other | other |
| SNX32 | ENSG00000172803 | sorting nexin 32 | 0,617 | Other | other |
| SOCS1 | ENSG00000185338 | suppressor of cytokine signaling 1 | 3,350 | Cytoplasm | other |
| SOCS2 | ENSG00000120833 | suppressor of cytokine signaling 2 | 1,565 | Cytoplasm | other |
| SOCS3 | ENSG00000184557 | suppressor of cytokine signaling 3 | 1,708 | Cytoplasm | phosphatase |
| SOD2 | ENSG00000112096 | superoxide dismutase 2 | 1,384 | Cytoplasm | enzyme |
| SORBS2 | ENSG00000154556 | sorbin and SH3 domain containing 2 | -0,725 | Plasma Membrane | other |
| SORT1 | ENSG00000134243 | sortilin 1 | -0,872 | Plasma Membrane | G-protein coupled receptor |
| SOSTDC1 | ENSG00000171243 | sclerostin domain containing 1 | -1,366 | Extracellular Space | growth factor |
| SOX13 | ENSG00000143842 | SRY-box transcription factor 13 | 0,675 | Nucleus | transcription regulator |
| SOX4 | ENSG00000124766 | SRY-box transcription factor 4 | 0,738 | Nucleus | transcription regulator |
| SOX9 | ENSG00000125398 | SRY-box transcription factor 9 | 0,736 | Nucleus | transcription regulator |
| SP100 | ENSG00000067066 | SP100 nuclear antigen | 1,621 | Nucleus | transcription regulator |
| SP110 | ENSG00000135899 | SP110 nuclear body protein | 2,395 | Nucleus | transcription regulator |
| SP140 | ENSG00000079263 | SP140 nuclear body protein | 1,012 | Nucleus | transcription regulator |
| SP140L | ENSG00000185404 | SP140 nuclear body protein like | 1,542 | Other | other |
| SPA17 | ENSG00000064199 | sperm autoantigenic protein 17 | -0,880 | Plasma Membrane | other |
| SPAG1 | ENSG00000104450 | sperm associated antigen 1 | 0,669 | Cytoplasm | other |
| SPAG16 | ENSG00000144451 | sperm associated antigen 16 | -1,159 | Cytoplasm | other |
| SPAG17 | ENSG00000155761 | sperm associated antigen 17 | 1,820 | Cytoplasm | other |
| SPAG4 | ENSG00000061656 | sperm associated antigen 4 | -1,094 | Cytoplasm | other |
| SPAG5 | ENSG00000076382 | sperm associated antigen 5 | -0,705 | Nucleus | peptidase |
| SPAG9 | ENSG00000008294 | sperm associated antigen 9 | 1,505 | Cytoplasm | other |
| SPATA2 | ENSG00000158480 | spermatogenesis associated 2 | 1,190 | Extracellular Space | other |
| SPATA6 | ENSG00000132122 | spermatogenesis associated 6 | -0,871 | Cytoplasm | other |
| SPDEF | ENSG00000124664 | SAM pointed domain containing ETS transcription factor | -1,109 | Nucleus | transcription regulator |
| SPDYA | ENSG00000163806 | speedy/RINGO cell cycle regulator family member A | 1,289 | Nucleus | other |
| SPDYE12P | ENSG00000184616 | speedy/RINGO cell cycle regulator family member E12, pseudogene | -0,661 | Other | other |
| SPECC1L-ADORA2A | ENSG00000258555 | SPECC1L-ADORA2A readthrough (NMD candidate) | -0,610 | Other | other |
| SPEN | ENSG00000065526 | spen family transcriptional repressor | 0,612 | Nucleus | transcription regulator |
| SPINK1 | ENSG00000164266 | serine peptidase inhibitor Kazal type 1 | -1,239 | Extracellular Space | other |
| SPNS2 | ENSG00000183018 | sphingolipid transporter 2 | -0,870 | Other | transporter |
| SPOCK2 | ENSG00000107742 | SPARC (osteonectin), cwcv and kazal like domains proteoglycan 2 | -0,891 | Extracellular Space | other |
| SPP1 | ENSG00000118785 | secreted phosphoprotein 1 | -0,657 | Extracellular Space | cytokine |
| SPRR3 | ENSG00000163209 | small proline rich protein 3 | -1,151 | Cytoplasm | other |

| | | | | | |
|---|---|---|---|---|---|
| SPRY1 | ENSG00000164056 | sprouty RTK signaling antagonist 1 | 0,700 | Cytoplasm | other |
| SPRY2 | ENSG00000136158 | sprouty RTK signaling antagonist 2 | 2,132 | Plasma Membrane | other |
| SPRY4 | ENSG00000187678 | sprouty RTK signaling antagonist 4 | 0,859 | Plasma Membrane | other |
| SPSB1 | ENSG00000171621 | splA/ryanodine receptor domain and SOCS box containing 1 | 1,148 | Cytoplasm | other |
| SPTA1 | ENSG00000163554 | spectrin alpha, erythrocytic 1 | 2,025 | Cytoplasm | other |
| SQLE | ENSG00000104549 | squalene epoxidase | -1,300 | Cytoplasm | enzyme |
| SRD5A3 | ENSG00000128039 | steroid 5 alpha-reductase 3 | -1,005 | Cytoplasm | enzyme |
| SREBF2 | ENSG00000198911 | sterol regulatory element binding transcription factor 2 | -0,869 | Nucleus | transcription regulator |
| SREK1 | ENSG00000153914 | splicing regulatory glutamic acid and lysine rich protein 1 | 0,664 | Nucleus | other |
| SRFBP1 | ENSG00000151304 | serum response factor binding protein 1 | 1,134 | Nucleus | other |
| SRI | ENSG00000075142 | sorcin | -1,545 | Cytoplasm | transporter |
| SRP9 | ENSG00000143742 | signal recognition particle 9 | -0,609 | Cytoplasm | other |
| SRPK2 | ENSG00000135250 | SRSF protein kinase 2 | 1,078 | Nucleus | kinase |
| SSBP4 | ENSG00000130511 | single stranded DNA binding protein 4 | -0,729 | Nucleus | transcription regulator |
| SSH3 | ENSG00000172830 | slingshot protein phosphatase 3 | -0,623 | Cytoplasm | phosphatase |
| SSPN | ENSG00000123096 | sarcospan | -0,647 | Plasma Membrane | other |
| SSTR2 | ENSG00000180616 | somatostatin receptor 2 | 1,169 | Plasma Membrane | G-protein coupled receptor |
| ST3GAL2 | ENSG00000157350 | ST3 beta-galactoside alpha-2,3-sialyltransferase 2 | 1,566 | Cytoplasm | enzyme |
| ST6GALNAC2 | ENSG00000070731 | ST6 N-acetylgalactosaminide alpha-2,6-sialyltransferase 2 | -1,242 | Cytoplasm | enzyme |
| ST6GALNAC6 | ENSG00000160408 | ST6 N-acetylgalactosaminide alpha-2,6-sialyltransferase 6 | -0,625 | Cytoplasm | enzyme |
| STAP2 | ENSG00000178078 | signal transducing adaptor family member 2 | 0,924 | Cytoplasm | other |
| STARD5 | ENSG00000172345 | StAR related lipid transfer domain containing 5 | 1,539 | Cytoplasm | transporter |
| STARD8 | ENSG00000130052 | StAR related lipid transfer domain containing 8 | 0,684 | Cytoplasm | other |
| STAT1 | ENSG00000115415 | signal transducer and activator of transcription 1 | 1,988 | Nucleus | transcription regulator |
| STAT2 | ENSG00000170581 | signal transducer and activator of transcription 2 | 2,546 | Nucleus | transcription regulator |
| STAT4 | ENSG00000138378 | signal transducer and activator of transcription 4 | 0,780 | Nucleus | transcription regulator |
| STAT5A | ENSG00000126561 | signal transducer and activator of transcription 5A | 1,390 | Nucleus | transcription regulator |
| STC1 | ENSG00000159167 | stanniocalcin 1 | 0,657 | Extracellular Space | kinase |
| STC2 | ENSG00000113739 | stanniocalcin 2 | 1,230 | Extracellular Space | other |
| STEAP4 | ENSG00000127954 | STEAP4 metalloreductase | -1,270 | Plasma Membrane | enzyme |
| STIM2 | ENSG00000109689 | stromal interaction molecule 2 | 0,783 | Plasma Membrane | transporter |
| STING1 | ENSG00000184584 | stimulator of interferon response cGAMP interactor 1 | 1,631 | Cytoplasm | other |
| STK10 | ENSG00000072786 | serine/threonine kinase 10 | 1,318 | Cytoplasm | kinase |
| STK19 | ENSG00000206342 | serine/threonine kinase 19 | 1,044 | Nucleus | kinase |
| STK32A | ENSG00000169302 | serine/threonine kinase 32A | -1,073 | Other | kinase |
| STK38L | ENSG00000211455 | serine/threonine kinase 38 like | 1,193 | Cytoplasm | kinase |
| STKLD1 | ENSG00000198870 | serine/threonine kinase like domain containing 1 | -0,620 | Other | kinase |
| STMN1 | ENSG00000117632 | stathmin 1 | -1,123 | Cytoplasm | other |
| STON2 | ENSG00000140022 | stonin 2 | 1,205 | Cytoplasm | other |
| STX10 | ENSG00000104915 | syntaxin 10 | -0,634 | Cytoplasm | other |
| STX11 | ENSG00000135604 | syntaxin 11 | 1,541 | Plasma Membrane | transporter |
| STX16 | ENSG00000124222 | syntaxin 16 | 0,763 | Cytoplasm | transporter |
| STX17 | ENSG00000136874 | syntaxin 17 | 0,760 | Plasma Membrane | transporter |
| STX19 | ENSG00000178750 | syntaxin 19 | 2,096 | Other | other |
| STYK1 | ENSG00000060140 | serine/threonine/tyrosine kinase 1 | 0,945 | Cytoplasm | kinase |
| SUCLG1 | ENSG00000163541 | succinate-CoA ligase GDP/ADP-forming subunit alpha | -0,651 | Cytoplasm | enzyme |
| SUCLG2 | ENSG00000172340 | succinate-CoA ligase GDP-forming subunit beta | -0,741 | Cytoplasm | enzyme |
| SUCO | ENSG00000094975 | SUN domain containing ossification factor | 0,707 | Other | other |
| SULT1C2 | ENSG00000198203 | sulfotransferase family 1C member 2 | 0,807 | Cytoplasm | enzyme |
| SUMF1 | ENSG00000144455 | sulfatase modifying factor 1 | -1,687 | Cytoplasm | enzyme |
| SUMF2 | ENSG00000129103 | sulfatase modifying factor 2 | -0,670 | Cytoplasm | other |
| SUPT7L | ENSG00000119760 | SPT7 like, STAGA complex subunit gamma | 0,780 | Nucleus | transcription regulator |
| SUSD3 | ENSG00000157303 | sushi domain containing 3 | 1,734 | Plasma Membrane | other |
| SUSD4 | ENSG00000143502 | sushi domain containing 4 | -1,009 | Other | other |
| SVIP | ENSG00000198168 | small VCP interacting protein | -0,856 | Cytoplasm | other |
| SWI5 | ENSG00000175854 | SWI5 homologous recombination repair protein | -0,944 | Nucleus | other |
| SYBU | ENSG00000147642 | syntabulin | -0,654 | Cytoplasm | other |
| SYDE1 | ENSG00000105137 | synapse defective Rho GTPase homolog 1 | 1,756 | Cytoplasm | other |
| SYNE1 | ENSG00000131018 | spectrin repeat containing nuclear envelope protein 1 | 1,447 | Nucleus | other |
| SYNE4 | ENSG00000181392 | spectrin repeat containing nuclear envelope family member 4 | -0,670 | Nucleus | other |
| SYNJ1 | ENSG00000159082 | synaptojanin 1 | 0,909 | Cytoplasm | phosphatase |
| SYNPO | ENSG00000171992 | synaptopodin | 0,966 | Cytoplasm | other |
| SYNPO2 | ENSG00000172403 | synaptopodin 2 | 0,978 | Cytoplasm | other |
| SYS1 | ENSG00000204070 | SYS1 golgi trafficking protein | 0,711 | Cytoplasm | other |
| SYT1 | ENSG00000067715 | synaptotagmin 1 | 1,196 | Cytoplasm | transporter |
| SYT12 | ENSG00000173227 | synaptotagmin 12 | -1,894 | Plasma Membrane | transporter |
| SYT16 | ENSG00000139973 | synaptotagmin 16 | -0,669 | Other | transporter |
| SYT8 | ENSG00000149043 | synaptotagmin 8 | -1,205 | Cytoplasm | transporter |
| SYTL5 | ENSG00000147041 | synaptotagmin like 5 | 0,952 | Other | other |
| TAAR3P | ENSG00000179073 | trace amine associated receptor 3, pseudogene | 3,045 | Plasma Membrane | G-protein coupled receptor |
| TAF8 | ENSG00000137413 | TATA-box binding protein associated factor 8 | 0,783 | Nucleus | transcription regulator |
| TAGLN2 | ENSG00000158710 | transgelin 2 | -0,874 | Cytoplasm | other |
| TAOK3 | ENSG00000135090 | TAO kinase 3 | 0,766 | Cytoplasm | kinase |
| TAP1 | ENSG00000168394 | transporter 1, ATP binding cassette subfamily B member | 3,620 | Cytoplasm | transporter |
| TAP2 | ENSG00000225967 | transporter 2, ATP binding cassette subfamily B member | 2,287 | Cytoplasm | transporter |
| TAPBP | ENSG00000231925 | TAP binding protein | 1,942 | Cytoplasm | transporter |
| TAPBPL | ENSG00000139192 | TAP binding protein like | 2,470 | Cytoplasm | other |
| TAPT1 | ENSG00000169762 | transmembrane anterior posterior transformation 1 | 0,973 | Plasma Membrane | G-protein coupled receptor |
| TAZ | ENSG00000102125 | tafazzin | -0,672 | Nucleus | enzyme |
| TBC1D22B | ENSG00000065491 | TBC1 domain family member 22B | 1,103 | Other | other |
| TBC1D5 | ENSG00000131374 | TBC1 domain family member 5 | -0,866 | Extracellular Space | other |
| TBC1D9 | ENSG00000109436 | TBC1 domain family member 9 | -0,985 | Plasma Membrane | other |
| TBCC | ENSG00000124659 | tubulin folding cofactor C | 0,794 | Cytoplasm | enzyme |
| TC2N | ENSG00000165929 | tandem C2 domains, nuclear | -0,602 | Nucleus | transporter |
| TCEA2 | ENSG00000171703 | transcription elongation factor A2 | -1,096 | Nucleus | transcription regulator |
| TCERG1 | ENSG00000113649 | transcription elongation regulator 1 | 0,602 | Nucleus | transcription regulator |
| TCF12 | ENSG00000140262 | transcription factor 12 | 0,637 | Nucleus | transcription regulator |
| TCF7L2 | ENSG00000148737 | transcription factor 7 like 2 | 0,629 | Nucleus | transcription regulator |
| TCIM | ENSG00000176907 | transcriptional and immune response regulator | 1,920 | Nucleus | other |
| TCN1 | ENSG00000134827 | transcobalamin 1 | -0,879 | Cytoplasm | transporter |
| TCP11L2 | ENSG00000166046 | t-complex 11 like 2 | 0,741 | Other | other |
| TCTEX1D2 | ENSG00000213123 | Tctex1 domain containing 2 | -0,852 | Cytoplasm | other |
| TCTN1 | ENSG00000204852 | tectonic family member 1 | -0,869 | Extracellular Space | other |
| TCTN2 | ENSG00000168778 | tectonic family member 2 | -0,647 | Extracellular Space | other |
| TDRD7 | ENSG00000196116 | tudor domain containing 7 | 1,964 | Cytoplasm | other |
| TDRKH | ENSG00000182134 | tudor and KH domain containing | -0,740 | Cytoplasm | other |
| TEC | ENSG00000135605 | tec protein tyrosine kinase | 0,960 | Cytoplasm | kinase |
| TEN1 | ENSG00000257949 | TEN1 subunit of CST complex | -0,822 | Nucleus | other |
| TENT5A | ENSG00000112773 | terminal nucleotidyltransferase 5A | 3,727 | Other | other |

| | | | | | |
|---|---|---|---|---|---|
| TENT5C | ENSG00000183508 | terminal nucleotidyltransferase 5C | 1,061 | Extracellular Space | enzyme |
| TEP1 | ENSG00000129566 | telomerase associated protein 1 | 2,060 | Nucleus | enzyme |
| TESK2 | ENSG00000070759 | testis associated actin remodelling kinase 2 | 1,299 | Nucleus | kinase |
| TET2 | ENSG00000168769 | tet methylcytosine dioxygenase 2 | 1,140 | Nucleus | enzyme |
| TEX15 | ENSG00000133863 | testis expressed 15, meiosis and synapsis associated | -0,731 | Extracellular Space | other |
| TFRC | ENSG00000072274 | transferrin receptor | -1,235 | Plasma Membrane | transporter |
| TGFB2 | ENSG00000092969 | transforming growth factor beta 2 | -0,618 | Extracellular Space | growth factor |
| TGFBI | ENSG00000120708 | transforming growth factor beta induced | -1,228 | Extracellular Space | other |
| TGFBR1 | ENSG00000106799 | transforming growth factor beta receptor 1 | -1,011 | Plasma Membrane | kinase |
| TGIF2 | ENSG00000118707 | TGFB induced factor homeobox 2 | 0,754 | Nucleus | transcription regulator |
| TGIF2-RAB5IF | ENSG00000259399 | TGIF2-RAB5IF readthrough | 1,937 | Other | other |
| TGM2 | ENSG00000198959 | transglutaminase 2 | 1,049 | Cytoplasm | enzyme |
| TGOLN2 | ENSG00000152291 | trans-golgi network protein 2 | -0,683 | Cytoplasm | other |
| TGS1 | ENSG00000137574 | trimethylguanosine synthase 1 | 1,015 | Nucleus | enzyme |
| THAP1 | ENSG00000131931 | THAP domain containing 1 | 0,686 | Nucleus | transcription regulator |
| THBS3 | ENSG00000169231 | thrombospondin 3 | -0,738 | Extracellular Space | other |
| THEM6 | ENSG00000130193 | thioesterase superfamily member 6 | -0,671 | Other | other |
| THEMIS2 | ENSG00000130775 | thymocyte selection associated family member 2 | 2,797 | Other | other |
| THG1L | ENSG00000113272 | tRNA-histidine guanylyltransferase 1 like | 0,920 | Cytoplasm | enzyme |
| THRA | ENSG00000126351 | thyroid hormone receptor alpha | -0,940 | Nucleus | ligand-dependent nuclear receptor |
| THSD1 | ENSG00000136114 | thrombospondin type 1 domain containing 1 | 1,207 | Extracellular Space | other |
| THSD7A | ENSG00000005108 | thrombospondin type 1 domain containing 7A | 0,947 | Cytoplasm | other |
| TICAM1 | ENSG00000127666 | toll like receptor adaptor molecule 1 | 1,369 | Nucleus | other |
| TIFA | ENSG00000145365 | TRAF interacting protein with forkhead associated domain | 0,841 | Cytoplasm | other |
| TINAG | ENSG00000137251 | tubulointerstitial nephritis antigen | 0,665 | Extracellular Space | peptidase |
| TIPARP | ENSG00000163659 | TCDD inducible poly(ADP-ribose) polymerase | 2,932 | Nucleus | enzyme |
| TJAP1 | ENSG00000137221 | tight junction associated protein 1 | 0,698 | Plasma Membrane | other |
| TKFC | ENSG00000149476 | triokinase and FMN cyclase | -0,695 | Cytoplasm | kinase |
| TKT | ENSG00000163931 | transketolase | -0,613 | Cytoplasm | enzyme |
| TLCD4 | ENSG00000152078 | TLC domain containing 4 | -0,754 | Other | other |
| TLDC2 | ENSG00000101342 | TBC/LysM-associated domain containing 2 | 2,814 | Other | other |
| TLE2 | ENSG00000065717 | TLE family member 2, transcriptional corepressor | -0,834 | Nucleus | transcription regulator |
| TLE4 | ENSG00000106829 | TLE family member 4, transcriptional corepressor | 1,147 | Nucleus | transcription regulator |
| TLK2 | ENSG00000146872 | tousled like kinase 2 | 0,778 | Cytoplasm | kinase |
| TLR1 | ENSG00000174125 | toll like receptor 1 | 1,077 | Plasma Membrane | transmembrane receptor |
| TLR2 | ENSG00000137462 | toll like receptor 2 | 0,872 | Plasma Membrane | transmembrane receptor |
| TLR3 | ENSG00000164342 | toll like receptor 3 | 2,681 | Plasma Membrane | transmembrane receptor |
| TLR5 | ENSG00000187554 | toll like receptor 5 | -0,991 | Plasma Membrane | transmembrane receptor |
| TM4SF4 | ENSG00000169903 | transmembrane 4 L six family member 4 | -1,194 | Plasma Membrane | other |
| TM7SF2 | ENSG00000149809 | transmembrane 7 superfamily member 2 | -1,484 | Cytoplasm | enzyme |
| TMA7 | ENSG00000232112 | translation machinery associated 7 homolog | -0,627 | Other | other |
| TMBIM4 | ENSG00000155957 | transmembrane BAX inhibitor motif containing 4 | -0,810 | Nucleus | other |
| TMBIM6 | ENSG00000139644 | transmembrane BAX inhibitor motif containing 6 | -0,812 | Nucleus | other |
| TMC7 | ENSG00000170537 | transmembrane channel like 7 | 0,751 | Other | other |
| TMCO3 | ENSG00000150403 | transmembrane and coiled-coil domains 3 | -1,049 | Other | transporter |
| TMEFF1 | ENSG00000241697 | transmembrane protein with EGF like and two follistatin like domains 1 | -1,320 | Plasma Membrane | other |
| TMEM106C | ENSG00000134291 | transmembrane protein 106C | -0,985 | Other | other |
| TMEM107 | ENSG00000179029 | transmembrane protein 107 | -1,017 | Extracellular Space | other |
| TMEM120A | ENSG00000189077 | transmembrane protein 120A | -1,176 | Nucleus | other |
| TMEM129 | ENSG00000168936 | transmembrane protein 129, E3 ubiquitin ligase | -0,658 | Cytoplasm | enzyme |
| TMEM132A | ENSG00000006118 | transmembrane protein 132A | 0,650 | Cytoplasm | other |
| TMEM134 | ENSG00000172663 | transmembrane protein 134 | -1,022 | Other | kinase |
| TMEM140 | ENSG00000146859 | transmembrane protein 140 | 1,496 | Other | other |
| TMEM141 | ENSG00000244187 | transmembrane protein 141 | -0,703 | Other | other |
| TMEM144 | ENSG00000164124 | transmembrane protein 144 | -0,755 | Other | other |
| TMEM14A | ENSG00000096092 | transmembrane protein 14A | -0,613 | Cytoplasm | other |
| TMEM156 | ENSG00000121895 | transmembrane protein 156 | 0,731 | Other | other |
| TMEM160 | ENSG00000130748 | transmembrane protein 160 | -0,757 | Cytoplasm | other |
| TMEM168 | ENSG00000146802 | transmembrane protein 168 | -0,801 | Cytoplasm | other |
| TMEM170A | ENSG00000166822 | transmembrane protein 170A | 0,809 | Other | other |
| TMEM171 | ENSG00000157111 | transmembrane protein 171 | 1,567 | Other | other |
| TMEM181 | ENSG00000146433 | transmembrane protein 181 | -0,619 | Other | other |
| TMEM19 | ENSG00000139291 | transmembrane protein 19 | -1,319 | Other | other |
| TMEM198 | ENSG00000188760 | transmembrane protein 198 | 0,725 | Other | other |
| TMEM205 | ENSG00000105518 | transmembrane protein 205 | -0,693 | Cytoplasm | other |
| TMEM213 | ENSG00000214128 | transmembrane protein 213 | -0,752 | Other | other |
| TMEM217 | ENSG00000172738 | transmembrane protein 217 | 0,927 | Nucleus | other |
| TMEM218 | ENSG00000150433 | transmembrane protein 218 | -1,017 | Extracellular Space | other |
| TMEM229B | ENSG00000198133 | transmembrane protein 229B | 1,850 | Other | other |
| TMEM230 | ENSG00000089063 | transmembrane protein 230 | -0,819 | Cytoplasm | other |
| TMEM231 | ENSG00000205084 | transmembrane protein 231 | -0,645 | Extracellular Space | other |
| TMEM237 | ENSG00000155755 | transmembrane protein 237 | -0,747 | Extracellular Space | other |
| TMEM250 | ENSG00000238227 | transmembrane protein 250 | -0,948 | Nucleus | other |
| TMEM251 | ENSG00000153485 | transmembrane protein 251 | -0,673 | Other | other |
| TMEM254 | ENSG00000133678 | transmembrane protein 254 | -1,044 | Other | other |
| TMEM256 | ENSG00000205544 | transmembrane protein 256 | -1,067 | Cytoplasm | other |
| TMEM268 | ENSG00000157693 | transmembrane protein 268 | 0,804 | Cytoplasm | other |
| TMEM38A | ENSG00000072954 | transmembrane protein 38A | -0,812 | Cytoplasm | ion channel |
| TMEM45B | ENSG00000151715 | transmembrane protein 45B | -0,783 | Extracellular Space | other |
| TMEM53 | ENSG00000126106 | transmembrane protein 53 | -0,680 | Nucleus | other |
| TMEM59 | ENSG00000116209 | transmembrane protein 59 | -0,742 | Plasma Membrane | peptidase |
| TMEM62 | ENSG00000137842 | transmembrane protein 62 | 1,451 | Other | enzyme |
| TMEM8B | ENSG00000137103 | transmembrane protein 8B | -1,026 | Plasma Membrane | other |
| TMEM99 | ENSG00000167920 | transmembrane protein 99 (putative) | -0,873 | Other | other |
| TMTC4 | ENSG00000125247 | transmembrane O-mannosyltransferase targeting cadherins 4 | -0,620 | Cytoplasm | enzyme |
| TMX4 | ENSG00000125827 | thioredoxin related transmembrane protein 4 | -0,853 | Cytoplasm | enzyme |
| TNC | ENSG00000041982 | tenascin C | 0,628 | Extracellular Space | other |
| TNF | ENSG00000228978 | tumor necrosis factor | 3,538 | Extracellular Space | cytokine |
| TNFAIP2 | ENSG00000185215 | TNF alpha induced protein 2 | 1,822 | Extracellular Space | other |
| TNFAIP3 | ENSG00000118503 | TNF alpha induced protein 3 | 3,952 | Nucleus | enzyme |
| TNFAIP6 | ENSG00000123610 | TNF alpha induced protein 6 | 0,634 | Extracellular Space | other |
| TNFAIP8L1 | ENSG00000185361 | TNF alpha induced protein 8 like 1 | -0,937 | Cytoplasm | other |
| TNFAIP8L2-SCNM1 | ENSG00000248167 | TNFAIP8L2-SCNM1 readthrough | -1,405 | Other | other |
| TNFRSF10A | ENSG00000104689 | TNF receptor superfamily member 10a | 1,253 | Plasma Membrane | transmembrane receptor |
| TNFRSF10B | ENSG00000120889 | TNF receptor superfamily member 10b | 1,310 | Plasma Membrane | transmembrane receptor |
| TNFRSF10D | ENSG00000173530 | TNF receptor superfamily member 10d | 0,613 | Plasma Membrane | transmembrane receptor |
| TNFRSF11B | ENSG00000164761 | TNF receptor superfamily member 11b | 1,023 | Plasma Membrane | transmembrane receptor |
| TNFRSF14 | ENSG00000157873 | TNF receptor superfamily member 14 | 1,278 | Plasma Membrane | transmembrane receptor |
| TNFRSF1B | ENSG00000028137 | TNF receptor superfamily member 1B | 1,554 | Plasma Membrane | transmembrane receptor |

| | | | | | |
|---|---|---|---|---|---|
| TNFRSF25 | ENSG00000215788 | TNF receptor superfamily member 25 | 1,196 | Plasma Membrane | transmembrane receptor |
| TNFRSF9 | ENSG00000049249 | TNF receptor superfamily member 9 | 2,444 | Plasma Membrane | transmembrane receptor |
| TNFSF10 | ENSG00000121858 | TNF superfamily member 10 | 3,185 | Extracellular Space | cytokine |
| TNFSF12 | ENSG00000239697 | TNF superfamily member 12 | -1,175 | Extracellular Space | cytokine |
| TNFSF13B | ENSG00000102524 | TNF superfamily member 13b | 3,563 | Extracellular Space | cytokine |
| TNFSF15 | ENSG00000181634 | TNF superfamily member 15 | 2,105 | Extracellular Space | cytokine |
| TNFSF9 | ENSG00000125657 | TNF superfamily member 9 | 1,916 | Plasma Membrane | cytokine |
| TNIK | ENSG00000154310 | TRAF2 and NCK interacting kinase | -0,778 | Plasma Membrane | kinase |
| TNIP1 | ENSG00000145901 | TNFAIP3 interacting protein 1 | 0,912 | Nucleus | other |
| TNNC1 | ENSG00000114854 | troponin C1, slow skeletal and cardiac type | -1,706 | Cytoplasm | other |
| TNNI1 | ENSG00000159173 | troponin I1, slow skeletal type | -0,652 | Cytoplasm | other |
| TNNT1 | ENSG00000105048 | troponin T1, slow skeletal type | -0,871 | Cytoplasm | other |
| TNS3 | ENSG00000136205 | tensin 3 | -0,612 | Plasma Membrane | phosphatase |
| TNS4 | ENSG00000131746 | tensin 4 | 1,155 | Cytoplasm | other |
| TOGARAM1 | ENSG00000198718 | TOG array regulator of axonemal microtubules 1 | 0,702 | Extracellular Space | other |
| TOMM40L | ENSG00000158882 | translocase of outer mitochondrial membrane 40 like | 0,702 | Cytoplasm | other |
| TOP1 | ENSG00000198900 | DNA topoisomerase I | 0,703 | Nucleus | enzyme |
| TOP2A | ENSG00000131747 | DNA topoisomerase II alpha | -1,188 | Nucleus | enzyme |
| TOP2B | ENSG00000077097 | DNA topoisomerase II beta | -0,850 | Nucleus | enzyme |
| TOR1B | ENSG00000136816 | torsin family 1 member B | 0,870 | Cytoplasm | enzyme |
| TP53BP2 | ENSG00000143514 | tumor protein p53 binding protein 2 | 0,833 | Nucleus | other |
| TP53I11 | ENSG00000175274 | tumor protein p53 inducible protein 11 | -0,607 | Other | other |
| TPCN1 | ENSG00000186815 | two pore segment channel 1 | -0,693 | Plasma Membrane | ion channel |
| TPD52L1 | ENSG00000111907 | TPD52 like 1 | -0,871 | Cytoplasm | other |
| TPGS2 | ENSG00000134779 | tubulin polyglutamylase complex subunit 2 | -0,674 | Nucleus | other |
| TPH2 | ENSG00000139287 | tryptophan hydroxylase 2 | 0,656 | Plasma Membrane | enzyme |
| TPM1 | ENSG00000140416 | tropomyosin 1 | -1,326 | Cytoplasm | other |
| TPM4 | ENSG00000167460 | tropomyosin 4 | -0,710 | Cytoplasm | other |
| TPPP3 | ENSG00000159713 | tubulin polymerization promoting protein family member 3 | -1,642 | Cytoplasm | other |
| TPX2 | ENSG00000088325 | TPX2 microtubule nucleation factor | -0,763 | Nucleus | other |
| TRA2A | ENSG00000164548 | transformer 2 alpha homolog | 1,136 | Nucleus | other |
| TRAF1 | ENSG00000056558 | TNF receptor associated factor 1 | 3,282 | Cytoplasm | other |
| TRAF3 | ENSG00000131323 | TNF receptor associated factor 3 | 0,666 | Cytoplasm | enzyme |
| TRAF4 | ENSG00000076604 | TNF receptor associated factor 4 | 0,734 | Cytoplasm | other |
| TRAFD1 | ENSG00000135148 | TRAF-type zinc finger domain containing 1 | 1,716 | Other | other |
| TRAM1 | ENSG00000067167 | translocation associated membrane protein 1 | -0,737 | Cytoplasm | other |
| TRANK1 | ENSG00000168016 | tetratricopeptide repeat and ankyrin repeat containing 1 | 3,330 | Nucleus | transcription regulator |
| TRAPPC6A | ENSG00000007255 | trafficking protein particle complex 6A | -1,266 | Cytoplasm | other |
| TRAPPC6B | ENSG00000182400 | trafficking protein particle complex 6B | 0,607 | Cytoplasm | other |
| TRERF1 | ENSG00000124496 | transcriptional regulating factor 1 | 1,358 | Nucleus | transcription regulator |
| TREX1 | ENSG00000213689 | three prime repair exonuclease 1 | 2,103 | Nucleus | enzyme |
| TRHDE | ENSG00000072657 | thyrotropin releasing hormone degrading enzyme | -0,617 | Plasma Membrane | peptidase |
| TRIB1 | ENSG00000173334 | tribbles pseudokinase 1 | 0,983 | Cytoplasm | kinase |
| TRIB2 | ENSG00000071575 | tribbles pseudokinase 2 | 0,667 | Plasma Membrane | kinase |
| TRIB3 | ENSG00000101255 | tribbles pseudokinase 3 | 0,880 | Nucleus | kinase |
| TRIM14 | ENSG00000106785 | tripartite motif containing 14 | 1,504 | Cytoplasm | other |
| TRIM15 | ENSG00000227147 | tripartite motif containing 15 | 0,668 | Other | other |
| TRIM21 | ENSG00000132109 | tripartite motif containing 21 | 2,302 | Nucleus | enzyme |
| TRIM22 | ENSG00000132274 | tripartite motif containing 22 | 4,971 | Cytoplasm | transcription regulator |
| TRIM25 | ENSG00000121060 | tripartite motif containing 25 | 1,231 | Cytoplasm | transcription regulator |
| TRIM26 | ENSG00000137313 | tripartite motif containing 26 | 1,884 | Cytoplasm | other |
| TRIM31 | ENSG00000233573 | tripartite motif containing 31 | 1,536 | Cytoplasm | other |
| TRIM34 | ENSG00000258659 | tripartite motif containing 34 | 0,948 | Cytoplasm | other |
| TRIM36 | ENSG00000152503 | tripartite motif containing 36 | 1,206 | Cytoplasm | enzyme |
| TRIM38 | ENSG00000112343 | tripartite motif containing 38 | 1,837 | Cytoplasm | other |
| TRIM5 | ENSG00000132256 | tripartite motif containing 5 | 2,443 | Cytoplasm | enzyme |
| TRIM56 | ENSG00000169871 | tripartite motif containing 56 | 1,476 | Cytoplasm | enzyme |
| TRIM6-TRIM34 | ENSG00000258588 | TRIM6-TRIM34 readthrough | 0,968 | Cytoplasm | other |
| TRIM69 | ENSG00000185880 | tripartite motif containing 69 | 1,704 | Nucleus | enzyme |
| TRIO | ENSG00000038382 | trio Rho guanine nucleotide exchange factor | 0,679 | Cytoplasm | kinase |
| TRIOBP | ENSG00000100106 | TRIO and F-actin binding protein | -0,667 | Nucleus | other |
| TRMT13 | ENSG00000122435 | tRNA methyltransferase 13 homolog | 0,735 | Other | other |
| TRMT2B | ENSG00000188917 | tRNA methyltransferase 2 homolog B | -0,636 | Cytoplasm | enzyme |
| TRMT44 | ENSG00000155275 | tRNA methyltransferase 44 homolog | 0,903 | Other | other |
| TRPC4 | ENSG00000133107 | transient receptor potential cation channel subfamily C member 4 | 2,134 | Plasma Membrane | ion channel |
| TRPS1 | ENSG00000104447 | transcriptional repressor GATA binding 1 | 1,458 | Nucleus | transcription regulator |
| TRPV1 | ENSG00000196689 | transient receptor potential cation channel subfamily V member 1 | 1,063 | Plasma Membrane | ion channel |
| TRPV4 | ENSG00000111199 | transient receptor potential cation channel subfamily V member 4 | -1,237 | Plasma Membrane | ion channel |
| TSC22D2 | ENSG00000196428 | TSC22 domain family member 2 | 1,307 | Nucleus | transcription regulator |
| TSLP | ENSG00000145777 | thymic stromal lymphopoietin | 1,342 | Extracellular Space | cytokine |
| TSN | ENSG00000211460 | translin | -0,826 | Nucleus | other |
| TSNARE1 | ENSG00000171045 | t-SNARE domain containing 1 | -0,799 | Other | other |
| TSNAXIP1 | ENSG00000102904 | translin associated factor X interacting protein 1 | -0,747 | Cytoplasm | other |
| TSPAN1 | ENSG00000117472 | tetraspanin 1 | -0,811 | Cytoplasm | other |
| TSPAN12 | ENSG00000106025 | tetraspanin 12 | -0,805 | Plasma Membrane | other |
| TSPAN6 | ENSG00000000003 | tetraspanin 6 | -0,724 | Plasma Membrane | other |
| TSPAN8 | ENSG00000127324 | tetraspanin 8 | -1,460 | Plasma Membrane | other |
| TSPYL2 | ENSG00000184205 | TSPY like 2 | 0,868 | Nucleus | other |
| TSTD1 | ENSG00000215845 | thiosulfate sulfurtransferase like domain containing 1 | -1,069 | Cytoplasm | enzyme |
| TTBK2 | ENSG00000128881 | tau tubulin kinase 2 | 0,996 | Cytoplasm | kinase |
| TTC28 | ENSG00000100154 | tetratricopeptide repeat domain 28 | 0,895 | Cytoplasm | other |
| TTC3 | ENSG00000182670 | tetratricopeptide repeat domain 3 | -0,941 | Cytoplasm | enzyme |
| TTC30A | ENSG00000197557 | tetratricopeptide repeat domain 30A | -0,800 | Extracellular Space | other |
| TTC39B | ENSG00000155158 | tetratricopeptide repeat domain 39B | 1,787 | Other | other |
| TTC9B | ENSG00000174521 | tetratricopeptide repeat domain 9B | 2,451 | Other | other |
| TTLL1 | ENSG00000100271 | tubulin tyrosine ligase like 1 | -1,035 | Extracellular Space | enzyme |
| TTLL11 | ENSG00000175764 | tubulin tyrosine ligase like 11 | 0,810 | Cytoplasm | enzyme |
| TTLL4 | ENSG00000135912 | tubulin tyrosine ligase like 4 | 0,656 | Cytoplasm | enzyme |
| TTYH3 | ENSG00000136295 | tweety family member 3 | -0,730 | Plasma Membrane | ion channel |
| TUBA1A | ENSG00000167552 | tubulin alpha 1a | -2,262 | Cytoplasm | other |
| TUBA8 | ENSG00000183785 | tubulin alpha 8 | 1,051 | Cytoplasm | other |
| TUBB2A | ENSG00000137267 | tubulin beta 2A class IIa | 0,607 | Cytoplasm | other |
| TUBB2B | ENSG00000137285 | tubulin beta 2B class IIb | 1,268 | Cytoplasm | other |
| TUBB6 | ENSG00000176014 | tubulin beta 6 class V | -0,686 | Cytoplasm | other |
| TUFT1 | ENSG00000143367 | tuftelin 1 | 0,785 | Cytoplasm | other |
| TWSG1 | ENSG00000128791 | twisted gastrulation BMP signaling modulator 1 | -0,635 | Extracellular Space | other |
| TXK | ENSG00000074966 | TXK tyrosine kinase | 0,906 | Cytoplasm | kinase |
| TXNIP | ENSG00000265972 | thioredoxin interacting protein | 2,429 | Cytoplasm | other |
| TYMP | ENSG00000025708 | thymidine phosphorylase | 2,875 | Extracellular Space | growth factor |

| | | | | | |
|---|---|---|---|---|---|
| TYRO3 | ENSG00000092445 | TYRO3 protein tyrosine kinase | -0,799 | Plasma Membrane | kinase |
| TYW5 | ENSG00000162971 | tRNA-yW synthesizing protein 5 | 0,992 | Cytoplasm | enzyme |
| UBA7 | ENSG00000182179 | ubiquitin like modifier activating enzyme 7 | 3,116 | Cytoplasm | enzyme |
| UBD | ENSG00000226898 | ubiquitin D | 1,804 | Nucleus | other |
| UBE2C | ENSG00000175063 | ubiquitin conjugating enzyme E2 C | -0,733 | Cytoplasm | enzyme |
| UBE2E3 | ENSG00000170035 | ubiquitin conjugating enzyme E2 E3 | -0,895 | Nucleus | enzyme |
| UBE2L6 | ENSG00000156587 | ubiquitin conjugating enzyme E2 L6 | 3,047 | Cytoplasm | enzyme |
| UBE2Z | ENSG00000159202 | ubiquitin conjugating enzyme E2 Z | 0,814 | Nucleus | enzyme |
| UBN2 | ENSG00000157741 | ubinuclein 2 | 1,708 | Nucleus | other |
| UBR2 | ENSG00000024048 | ubiquitin protein ligase E3 component n-recognin 2 | 0,689 | Nucleus | enzyme |
| UCP2 | ENSG00000175567 | uncoupling protein 2 | -2,102 | Cytoplasm | transporter |
| UGT1A10 (includes others) | ENSG00000242366 | UDP glucuronosyltransferase family 1 member A9 | 1,767 | Cytoplasm | enzyme |
| UGT2B7 | ENSG00000171234 | UDP glucuronosyltransferase family 2 member B7 | -1,162 | Cytoplasm | enzyme |
| UGT8 | ENSG00000174607 | UDP glycosyltransferase 8 | -0,623 | Cytoplasm | enzyme |
| UHRF1BP1 | ENSG00000065060 | UHRF1 binding protein 1 | 1,088 | Other | other |
| UNC119B | ENSG00000175970 | unc-119 lipid binding chaperone B | -0,741 | Cytoplasm | other |
| UNC5CL | ENSG00000124602 | unc-5 family C-terminal like | -0,667 | Cytoplasm | peptidase |
| UNC93B1 | ENSG00000110057 | unc-93 homolog B1, TLR signaling regulator | 1,317 | Cytoplasm | other |
| UNC93B7 | ENSG00000251624 | unc-93 homolog B7 (pseudogene) | 1,273 | Other | other |
| UPK1B | ENSG00000114638 | uroplakin 1B | -1,987 | Plasma Membrane | other |
| UPK3B | ENSG00000243566 | uroplakin 3B | -0,700 | Plasma Membrane | other |
| UQCR10 | ENSG00000184076 | ubiquinol-cytochrome c reductase, complex III subunit X | -0,729 | Cytoplasm | enzyme |
| UROD | ENSG00000126088 | uroporphyrinogen decarboxylase | -1,029 | Cytoplasm | enzyme |
| USF1 | ENSG00000158773 | upstream transcription factor 1 | 1,203 | Nucleus | transcription regulator |
| USF3 | ENSG00000176542 | upstream transcription factor family member 3 | 1,137 | Other | other |
| USH1C | ENSG00000006611 | USH1 protein network component harmonin | -1,001 | Plasma Membrane | other |
| USP13 | ENSG00000058056 | ubiquitin specific peptidase 13 | -0,680 | Cytoplasm | peptidase |
| USP15 | ENSG00000135655 | ubiquitin specific peptidase 15 | 1,002 | Cytoplasm | peptidase |
| USP18 | ENSG00000184979 | ubiquitin specific peptidase 18 | 4,189 | Cytoplasm | peptidase |
| USP25 | ENSG00000155313 | ubiquitin specific peptidase 25 | 0,895 | Cytoplasm | peptidase |
| USP42 | ENSG00000106346 | ubiquitin specific peptidase 42 | 0,829 | Nucleus | peptidase |
| USP43 | ENSG00000154914 | ubiquitin specific peptidase 43 | 1,588 | Nucleus | peptidase |
| USP53 | ENSG00000145390 | ubiquitin specific peptidase 53 | 0,649 | Extracellular Space | enzyme |
| VAMP1 | ENSG00000139190 | vesicle associated membrane protein 1 | 1,063 | Cytoplasm | transporter |
| VAMP2 | ENSG00000220205 | vesicle associated membrane protein 2 | 1,081 | Plasma Membrane | other |
| VAMP5 | ENSG00000168899 | vesicle associated membrane protein 5 | 2,085 | Plasma Membrane | transporter |
| VANGL2 | ENSG00000162738 | VANGL planar cell polarity protein 2 | 1,161 | Plasma Membrane | other |
| VARS1 | ENSG00000096171 | valyl-tRNA synthetase 1 | -0,606 | Cytoplasm | enzyme |
| VAT1 | ENSG00000108828 | vesicle amine transport 1 | -1,337 | Plasma Membrane | transporter |
| VAV3 | ENSG00000134215 | vav guanine nucleotide exchange factor 3 | -1,324 | Extracellular Space | cytokine |
| VCL | ENSG00000035403 | vinculin | -0,613 | Plasma Membrane | enzyme |
| VCPKMT | ENSG00000100483 | valosin containing protein lysine methyltransferase | 1,524 | Cytoplasm | enzyme |
| VDR | ENSG00000111424 | vitamin D receptor | 0,735 | Nucleus | transcription regulator |
| VEGFA | ENSG00000112715 | vascular endothelial growth factor A | 0,697 | Extracellular Space | growth factor |
| VEGFC | ENSG00000150630 | vascular endothelial growth factor C | 1,061 | Extracellular Space | growth factor |
| VGLL1 | ENSG00000102243 | vestigial like family member 1 | -0,857 | Nucleus | transcription regulator |
| VILL | ENSG00000136059 | villin like | -0,662 | Cytoplasm | other |
| VIM | ENSG00000026025 | vimentin | -1,891 | Cytoplasm | other |
| VKORC1 | ENSG00000167397 | vitamin K epoxide reductase complex subunit 1 | -1,014 | Cytoplasm | enzyme |
| VNN2 | ENSG00000112303 | vanin 2 | 0,732 | Other | enzyme |
| VOPP1 | ENSG00000154978 | VOPP1 WW domain binding protein | -0,627 | Nucleus | other |
| VPS26B | ENSG00000151502 | VPS26, retromer complex component B | -0,610 | Cytoplasm | transporter |
| VPS28 | ENSG00000160948 | VPS28 subunit of ESCRT-I | -0,660 | Cytoplasm | transporter |
| VSIG10L | ENSG00000186806 | V-set and immunoglobulin domain containing 10 like | 0,871 | Nucleus | other |
| VTCN1 | ENSG00000134258 | V-set domain containing T cell activation inhibitor 1 | -2,418 | Plasma Membrane | other |
| VWA1 | ENSG00000179403 | von Willebrand factor A domain containing 1 | -0,891 | Extracellular Space | other |
| VWA2 | ENSG00000165816 | von Willebrand factor A domain containing 2 | -1,152 | Extracellular Space | other |
| VXN | ENSG00000169085 | vexin | 0,778 | Other | other |
| WARS1 | ENSG00000140105 | tryptophanyl-tRNA synthetase 1 | 3,121 | Cytoplasm | enzyme |
| WASHC4 | ENSG00000136051 | WASH complex subunit 4 | 0,729 | Cytoplasm | other |
| WASHC5 | ENSG00000164961 | WASH complex subunit 5 | -0,897 | Cytoplasm | other |
| WBP1 | ENSG00000239779 | WW domain binding protein 1 | -1,401 | Cytoplasm | other |
| WDFY1 | ENSG00000085449 | WD repeat and FYVE domain containing 1 | 0,768 | Cytoplasm | other |
| WDR24 | ENSG00000127580 | WD repeat domain 24 | 0,949 | Cytoplasm | other |
| WDR25 | ENSG00000176473 | WD repeat domain 25 | 1,113 | Other | other |
| WDR31 | ENSG00000148225 | WD repeat domain 31 | -0,714 | Other | peptidase |
| WDR46 | ENSG00000236222 | WD repeat domain 46 | -1,348 | Nucleus | enzyme |
| WDR54 | ENSG00000005448 | WD repeat domain 54 | -0,846 | Other | other |
| WDR72 | ENSG00000166415 | WD repeat domain 72 | -0,939 | Cytoplasm | other |
| WDR73 | ENSG00000177082 | WD repeat domain 73 | 0,635 | Extracellular Space | other |
| WDR74 | ENSG00000133316 | WD repeat domain 74 | 0,728 | Nucleus | other |
| WDR81 | ENSG00000167716 | WD repeat domain 81 | 0,718 | Plasma Membrane | other |
| WDTC1 | ENSG00000142784 | WD and tetratricopeptide repeats 1 | -0,750 | Nucleus | other |
| WFDC5 | ENSG00000175121 | WAP four-disulfide core domain 5 | 4,219 | Extracellular Space | other |
| WHAMM | ENSG00000156232 | WASP homolog associated with actin, golgi membranes and microtubules | 0,919 | Cytoplasm | other |
| WIF1 | ENSG00000156076 | WNT inhibitory factor 1 | 2,193 | Extracellular Space | other |
| WNK2 | ENSG00000165238 | WNK lysine deficient protein kinase 2 | -1,209 | Cytoplasm | kinase |
| WTAP | ENSG00000146457 | WT1 associated protein | 0,755 | Nucleus | other |
| XAF1 | ENSG00000132530 | XIAP associated factor 1 | 3,404 | Nucleus | other |
| XBP1 | ENSG00000100219 | X-box binding protein 1 | 1,141 | Nucleus | transcription regulator |
| XDH | ENSG00000158125 | xanthine dehydrogenase | 0,718 | Cytoplasm | enzyme |
| XKR9 | ENSG00000221947 | XK related 9 | 1,473 | Plasma Membrane | other |
| XPO6 | ENSG00000169180 | exportin 6 | 0,873 | Cytoplasm | other |
| XRCC5 | ENSG00000079246 | X-ray repair cross complementing 5 | -0,714 | Nucleus | enzyme |
| XRN1 | ENSG00000114127 | 5'-3' exoribonuclease 1 | 2,190 | Cytoplasm | enzyme |
| XXbac-BPG246D15.9 | ENSG00000250264 | | 3,302 | Other | other |
| YBEY | ENSG00000182362 | ybeY metalloendoribonuclease | -0,903 | Cytoplasm | enzyme |
| YBX1 | ENSG00000065978 | Y-box binding protein 1 | -0,616 | Nucleus | transcription regulator |
| YJU2 | ENSG00000105248 | YJU2 splicing factor homolog | 0,833 | Nucleus | other |
| YOD1 | ENSG00000180667 | YOD1 deubiquitinase | 0,836 | Cytoplasm | enzyme |
| YRDC | ENSG00000196449 | yrdC N6-threonylcarbamoyltransferase domain containing | 1,005 | Other | other |
| ZBED5 | ENSG00000236287 | zinc finger BED-type containing 5 | 1,300 | Nucleus | transcription regulator |
| ZBED9 | ENSG00000248496 | zinc finger BED-type containing 9 | 0,648 | Other | other |
| ZBP1 | ENSG00000124256 | Z-DNA binding protein 1 | 5,306 | Cytoplasm | other |
| ZBTB10 | ENSG00000205189 | zinc finger and BTB domain containing 10 | 1,066 | Nucleus | other |
| ZBTB17 | ENSG00000116809 | zinc finger and BTB domain containing 17 | 1,059 | Nucleus | transcription regulator |
| ZBTB20 | ENSG00000181722 | zinc finger and BTB domain containing 20 | 2,217 | Nucleus | transcription regulator |
| ZBTB21 | ENSG00000173276 | zinc finger and BTB domain containing 21 | 1,476 | Nucleus | transcription regulator |
| ZBTB32 | ENSG00000011590 | zinc finger and BTB domain containing 32 | 1,002 | Nucleus | transcription regulator |

| | | | | | |
|---|---|---|---|---|---|
| ZBTB43 | ENSG00000169155 | zinc finger and BTB domain containing 43 | 1,521 | Nucleus | transcription regulator |
| ZBTB44 | ENSG00000196323 | zinc finger and BTB domain containing 44 | -0,776 | Other | other |
| ZBTB48 | ENSG00000204859 | zinc finger and BTB domain containing 48 | 0,956 | Nucleus | transcription regulator |
| ZBTB49 | ENSG00000168826 | zinc finger and BTB domain containing 49 | 0,789 | Nucleus | transcription regulator |
| ZBTB6 | ENSG00000186130 | zinc finger and BTB domain containing 6 | 0,710 | Nucleus | transcription regulator |
| ZC3H12A | ENSG00000163874 | zinc finger CCCH-type containing 12A | 1,428 | Cytoplasm | enzyme |
| ZC3H12C | ENSG00000149289 | zinc finger CCCH-type containing 12C | 1,634 | Other | other |
| ZC3H4 | ENSG00000130749 | zinc finger CCCH-type containing 4 | 0,946 | Nucleus | transcription regulator |
| ZC3HAV1 | ENSG00000105939 | zinc finger CCCH-type containing, antiviral 1 | 3,861 | Plasma Membrane | other |
| ZCCHC2 | ENSG00000141664 | zinc finger CCHC-type containing 2 | 1,319 | Cytoplasm | other |
| ZCCHC8 | ENSG00000033030 | zinc finger CCHC-type containing 8 | 0,632 | Nucleus | other |
| ZCRB1 | ENSG00000139168 | zinc finger CCHC-type and RNA binding motif containing 1 | -0,754 | Nucleus | other |
| ZDHHC1 | ENSG00000159714 | zinc finger DHHC-type containing 1 | -0,651 | Nucleus | enzyme |
| ZDHHC14 | ENSG00000175048 | zinc finger DHHC-type palmitoyltransferase 14 | 0,710 | Cytoplasm | enzyme |
| ZDHHC2 | ENSG00000104219 | zinc finger DHHC-type palmitoyltransferase 2 | -0,806 | Nucleus | enzyme |
| ZEB1 | ENSG00000148516 | zinc finger E-box binding homeobox 1 | 1,547 | Nucleus | transcription regulator |
| ZEB2 | ENSG00000169554 | zinc finger E-box binding homeobox 2 | 3,343 | Nucleus | transcription regulator |
| ZFAND2A | ENSG00000178381 | zinc finger AN1-type containing 2A | 0,629 | Other | other |
| ZFP36 | ENSG00000128016 | ZFP36 ring finger protein | 2,975 | Nucleus | transcription regulator |
| ZFP36L2 | ENSG00000152518 | ZFP36 ring finger protein like 2 | 0,957 | Nucleus | transcription regulator |
| ZFP37 | ENSG00000136866 | ZFP37 zinc finger protein | 0,701 | Nucleus | transcription regulator |
| ZFP69B | ENSG00000187801 | ZFP69 zinc finger protein B | -0,673 | Nucleus | transcription regulator |
| ZFPM2 | ENSG00000169946 | zinc finger protein, FOG family member 2 | 1,254 | Nucleus | transcription regulator |
| ZFX | ENSG00000005889 | zinc finger protein X-linked | 0,873 | Nucleus | transcription regulator |
| ZFYVE1 | ENSG00000165861 | zinc finger FYVE-type containing 1 | 0,736 | Cytoplasm | other |
| ZFYVE26 | ENSG00000072121 | zinc finger FYVE-type containing 26 | 1,015 | Cytoplasm | other |
| ZGPAT | ENSG00000197114 | zinc finger CCCH-type and G-patch domain containing | 0,626 | Nucleus | transcription regulator |
| ZHX2 | ENSG00000178764 | zinc fingers and homeoboxes 2 | 1,039 | Nucleus | transcription regulator |
| ZKSCAN2 | ENSG00000155592 | zinc finger with KRAB and SCAN domains 2 | 0,901 | Nucleus | transcription regulator |
| ZMAT1 | ENSG00000166432 | zinc finger matrin-type 1 | -0,632 | Nucleus | other |
| ZMYND8 | ENSG00000101040 | zinc finger MYND-type containing 8 | 0,700 | Nucleus | transcription regulator |
| ZNF101 | ENSG00000181896 | zinc finger protein 101 | 0,899 | Nucleus | transcription regulator |
| ZNF107 | ENSG00000196247 | zinc finger protein 107 | 1,087 | Nucleus | transcription regulator |
| ZNF114 | ENSG00000178150 | zinc finger protein 114 | 0,669 | Cytoplasm | transcription regulator |
| ZNF14 | ENSG00000105708 | zinc finger protein 14 | 0,930 | Nucleus | transcription regulator |
| ZNF140 | ENSG00000196387 | zinc finger protein 140 | 0,952 | Nucleus | transcription regulator |
| ZNF184 | ENSG00000096654 | zinc finger protein 184 | 0,723 | Nucleus | transcription regulator |
| ZNF185 | ENSG00000147394 | zinc finger protein 185 with LIM domain | -0,640 | Plasma Membrane | other |
| ZNF200 | ENSG00000010539 | zinc finger protein 200 | 1,510 | Nucleus | transcription regulator |
| ZNF213 | ENSG00000085644 | zinc finger protein 213 | 0,661 | Nucleus | transcription regulator |
| ZNF217 | ENSG00000171940 | zinc finger protein 217 | 0,782 | Nucleus | transcription regulator |
| ZNF219 | ENSG00000165804 | zinc finger protein 219 | -0,803 | Nucleus | transcription regulator |
| ZNF222 | ENSG00000159885 | zinc finger protein 222 | 1,406 | Nucleus | transcription regulator |
| ZNF253 | ENSG00000256771 | zinc finger protein 253 | 0,905 | Nucleus | transcription regulator |
| ZNF254 | ENSG00000213096 | zinc finger protein 254 | 0,974 | Nucleus | transcription regulator |
| ZNF263 | ENSG00000006194 | zinc finger protein 263 | 0,694 | Nucleus | transcription regulator |
| ZNF267 | ENSG00000185947 | zinc finger protein 267 | 1,408 | Nucleus | transcription regulator |
| ZNF274 | ENSG00000171606 | zinc finger protein 274 | 1,214 | Nucleus | transcription regulator |
| ZNF280C | ENSG00000056277 | zinc finger protein 280C | 0,699 | Nucleus | transcription regulator |
| ZNF280D | ENSG00000137871 | zinc finger protein 280D | 0,607 | Other | other |
| ZNF292 | ENSG00000188994 | zinc finger protein 292 | 1,173 | Nucleus | transcription regulator |
| ZNF296 | ENSG00000170684 | zinc finger protein 296 | 1,654 | Nucleus | transcription regulator |
| ZNF3 | ENSG00000166526 | zinc finger protein 3 | 1,074 | Nucleus | transcription regulator |
| ZNF300P1 | ENSG00000197083 | zinc finger protein 300 pseudogene 1 | 1,621 | Other | other |
| ZNF317 | ENSG00000130803 | zinc finger protein 317 | 1,234 | Nucleus | transcription regulator |
| ZNF319 | ENSG00000166188 | zinc finger protein 319 | 0,761 | Nucleus | transcription regulator |
| ZNF32 | ENSG00000169740 | zinc finger protein 32 | -0,691 | Nucleus | transcription regulator |
| ZNF324 | ENSG00000083812 | zinc finger protein 324 | 0,665 | Nucleus | transcription regulator |
| ZNF335 | ENSG00000198026 | zinc finger protein 335 | 0,729 | Nucleus | transcription regulator |
| ZNF35 | ENSG00000169981 | zinc finger protein 35 | 0,791 | Nucleus | transcription regulator |
| ZNF385A | ENSG00000161642 | zinc finger protein 385A | 0,838 | Nucleus | other |
| ZNF385C | ENSG00000187595 | zinc finger protein 385C | 1,991 | Other | other |
| ZNF391 | ENSG00000124613 | zinc finger protein 391 | -0,739 | Other | other |
| ZNF394 | ENSG00000160908 | zinc finger protein 394 | 0,662 | Nucleus | transcription regulator |
| ZNF408 | ENSG00000175213 | zinc finger protein 408 | 0,936 | Nucleus | transcription regulator |
| ZNF416 | ENSG00000083817 | zinc finger protein 416 | 0,691 | Nucleus | transcription regulator |
| ZNF426 | ENSG00000130818 | zinc finger protein 426 | 1,215 | Nucleus | transcription regulator |
| ZNF433 | ENSG00000197647 | zinc finger protein 433 | 1,492 | Nucleus | transcription regulator |
| ZNF436 | ENSG00000125945 | zinc finger protein 436 | 0,815 | Nucleus | transcription regulator |
| ZNF44 | ENSG00000197857 | zinc finger protein 44 | 1,047 | Nucleus | transcription regulator |
| ZNF441 | ENSG00000197044 | zinc finger protein 441 | 1,034 | Nucleus | transcription regulator |
| ZNF442 | ENSG00000198342 | zinc finger protein 442 | 2,259 | Nucleus | transcription regulator |
| ZNF460 | ENSG00000197714 | zinc finger protein 460 | 0,644 | Nucleus | transcription regulator |
| ZNF462 | ENSG00000148143 | zinc finger protein 462 | 1,670 | Nucleus | transcription regulator |
| ZNF471 | ENSG00000196263 | zinc finger protein 471 | 1,105 | Nucleus | transcription regulator |
| ZNF484 | ENSG00000127081 | zinc finger protein 484 | 0,710 | Nucleus | transcription regulator |
| ZNF490 | ENSG00000188033 | zinc finger protein 490 | 0,865 | Nucleus | transcription regulator |
| ZNF503 | ENSG00000165655 | zinc finger protein 503 | 1,961 | Nucleus | other |
| ZNF514 | ENSG00000144026 | zinc finger protein 514 | -0,624 | Nucleus | transcription regulator |
| ZNF543 | ENSG00000178229 | zinc finger protein 543 | 1,122 | Other | other |
| ZNF547 | ENSG00000152433 | zinc finger protein 547 | 0,622 | Nucleus | transcription regulator |
| ZNF557 | ENSG00000130544 | zinc finger protein 557 | 0,690 | Nucleus | transcription regulator |
| ZNF562 | ENSG00000171466 | zinc finger protein 562 | 0,973 | Nucleus | transcription regulator |
| ZNF583 | ENSG00000198440 | zinc finger protein 583 | 0,718 | Nucleus | transcription regulator |
| ZNF593 | ENSG00000142684 | zinc finger protein 593 | 0,609 | Nucleus | transcription regulator |
| ZNF619 | ENSG00000177873 | zinc finger protein 619 | 0,961 | Other | other |
| ZNF620 | ENSG00000177842 | zinc finger protein 620 | 1,474 | Nucleus | transcription regulator |
| ZNF625-ZNF20 | ENSG00000213297 | ZNF625-ZNF20 readthrough (NMD candidate) | 0,631 | Other | other |
| ZNF627 | ENSG00000198551 | zinc finger protein 627 | 1,357 | Other | other |
| ZNF654 | ENSG00000175105 | zinc finger protein 654 | 0,610 | Other | other |
| ZNF674 | ENSG00000251192 | zinc finger protein 674 | 0,915 | Other | other |
| ZNF678 | ENSG00000181450 | zinc finger protein 678 | 0,625 | Nucleus | transcription regulator |
| ZNF682 | ENSG00000197124 | zinc finger protein 682 | 0,722 | Nucleus | transcription regulator |
| ZNF689 | ENSG00000156853 | zinc finger protein 689 | 1,151 | Nucleus | transcription regulator |
| ZNF697 | ENSG00000143067 | zinc finger protein 697 | 1,377 | Other | other |
| ZNF7 | ENSG00000147789 | zinc finger protein 7 | 0,619 | Nucleus | transcription regulator |
| ZNF724 | ENSG00000196081 | zinc finger protein 724 | 0,891 | Other | other |
| ZNF746 | ENSG00000181220 | zinc finger protein 746 | 0,934 | Cytoplasm | transcription regulator |
| ZNF75A | ENSG00000162086 | zinc finger protein 75a | 1,033 | Nucleus | transcription regulator |

| | | | | | |
|---|---|---|---|---|---|
| ZNF764 | ENSG00000169951 | zinc finger protein 764 | 0,883 | Other | other |
| ZNF773 | ENSG00000152439 | zinc finger protein 773 | 0,776 | Other | other |
| ZNF787 | ENSG00000142409 | zinc finger protein 787 | 0,998 | Other | other |
| ZNF79 | ENSG00000196152 | zinc finger protein 79 | 0,805 | Nucleus | transcription regulator |
| ZNF791 | ENSG00000173875 | zinc finger protein 791 | 0,728 | Other | other |
| ZNF792 | ENSG00000180884 | zinc finger protein 792 | -0,702 | Other | other |
| ZNF805 | ENSG00000204524 | zinc finger protein 805 | 0,888 | Other | other |
| ZNF830 | ENSG00000198783 | zinc finger protein 830 | 0,698 | Nucleus | other |
| ZNF92 | ENSG00000146757 | zinc finger protein 92 | 1,751 | Nucleus | transcription regulator |
| ZNFX1 | ENSG00000124201 | zinc finger NFX1-type containing 1 | 2,968 | Nucleus | transcription regulator |
| ZNHIT2 | ENSG00000174276 | zinc finger HIT-type containing 2 | 0,608 | Other | other |
| ZRANB3 | ENSG00000121988 | zinc finger RANBP2-type containing 3 | -0,801 | Nucleus | enzyme |
| ZRSR2 | ENSG00000169249 | zinc finger CCCH-type, RNA binding motif and serine/arginine rich 2 | 0,633 | Nucleus | other |
| ZRSR2P1 | ENSG00000212643 | ZRSR2 pseudogene 1 | 1,378 | Nucleus | other |
| ZSCAN12 | ENSG00000158691 | zinc finger and SCAN domain containing 12 | 0,761 | Nucleus | transcription regulator |
| ZSCAN25 | ENSG00000197037 | zinc finger and SCAN domain containing 25 | 0,667 | Other | other |
| ZSCAN29 | ENSG00000140265 | zinc finger and SCAN domain containing 29 | 0,619 | Nucleus | transcription regulator |
| ZSWIM3 | ENSG00000132801 | zinc finger SWIM-type containing 3 | 0,908 | Other | other |
| ZSWIM4 | ENSG00000132003 | zinc finger SWIM-type containing 4 | 0,758 | Other | other |
| ZSWIM6 | ENSG00000130449 | zinc finger SWIM-type containing 6 | 0,638 | Extracellular Space | other |
| ZSWIM7 | ENSG00000214941 | zinc finger SWIM-type containing 7 | -0,676 | Other | other |
| ZUP1 | ENSG00000153975 | zinc finger containing ubiquitin peptidase 1 | 0,729 | Other | other |

**Supplementary Table S6. List of differentially expressed genes for the condition "Calu-3_SARS-CoV versus Calu-3_Mock" subjected to IPA Core Analysis.**
The number of differentially expressed genes is 2343.

| Symbol | ID | Entrez Gene Name | Expr Log Ratio | Location | Type(s) |
|---|---|---|---|---|---|
| AAMDC | ENSG00000087884 | adipogenesis associated Mth938 domain containing | -1,004 | Cytoplasm | other |
| AASS | ENSG00000008311 | aminoadipate-semialdehyde synthase | 1,354 | Cytoplasm | enzyme |
| ABCA1 | ENSG00000165029 | ATP binding cassette subfamily A member 1 | 1,400 | Plasma Membrane | transporter |
| ABCA12 | ENSG00000144452 | ATP binding cassette subfamily A member 12 | 1,387 | Plasma Membrane | transporter |
| ABCA3 | ENSG00000167972 | ATP binding cassette subfamily A member 3 | -0,607 | Plasma Membrane | transporter |
| ABCA5 | ENSG00000154265 | ATP binding cassette subfamily A member 5 | 1,717 | Plasma Membrane | transporter |
| ABCG1 | ENSG00000160179 | ATP binding cassette subfamily G member 1 | 1,116 | Plasma Membrane | transporter |
| ABHD4 | ENSG00000100439 | abhydrolase domain containing 4, N-acyl phospholipase B | 0,687 | Cytoplasm | peptidase |
| ABL2 | ENSG00000143322 | ABL proto-oncogene 2, non-receptor tyrosine kinase | 1,944 | Cytoplasm | kinase |
| ABTB2 | ENSG00000166016 | ankyrin repeat and BTB domain containing 2 | 1,825 | Nucleus | other |
| AC084219.2 | ENSG00000267022 | | 0,640 | Other | other |
| AC0937242 | ENSG00000213222 | | 1,078 | Other | other |
| ACAA2 | ENSG00000167315 | acetyl-CoA acyltransferase 2 | -0,694 | Cytoplasm | enzyme |
| ACAT2 | ENSG00000120437 | acetyl-CoA acetyltransferase 2 | -0,708 | Cytoplasm | enzyme |
| ACE2 | ENSG00000130234 | angiotensin I converting enzyme 2 | -0,775 | Plasma Membrane | peptidase |
| ACHE | ENSG00000087085 | acetylcholinesterase (Cartwright blood group) | 2,931 | Plasma Membrane | enzyme |
| ACKR4 | ENSG00000129048 | atypical chemokine receptor 4 | 2,185 | Plasma Membrane | G-protein coupled receptor |
| ACO1 | ENSG00000122729 | aconitase 1 | -0,969 | Cytoplasm | enzyme |
| ACSM3 | ENSG00000005187 | acyl-CoA synthetase medium chain family member 3 | -0,832 | Cytoplasm | enzyme |
| ACTBP11 | ENSG00000188460 | ACTB pseudogene 11 | 0,966 | Other | other |
| ACTN2 | ENSG00000077522 | actinin alpha 2 | 2,097 | Nucleus | transcription regulator |
| ACVR1 | ENSG00000115170 | activin A receptor type 1 | 0,884 | Plasma Membrane | kinase |
| ACVR2A | ENSG00000121989 | activin A receptor type 2A | 1,017 | Plasma Membrane | kinase |
| ADAM22 | ENSG00000008277 | ADAM metallopeptidase domain 22 | -0,970 | Plasma Membrane | peptidase |
| ADAM8 | ENSG00000151651 | ADAM metallopeptidase domain 8 | 1,372 | Plasma Membrane | peptidase |
| ADAMTS3 | ENSG00000156140 | ADAM metallopeptidase with thrombospondin type 1 motif 3 | 1,097 | Extracellular Space | peptidase |
| ADAMTS9 | ENSG00000163638 | ADAM metallopeptidase with thrombospondin type 1 motif 9 | 0,635 | Extracellular Space | peptidase |
| ADAR | ENSG00000160710 | adenosine deaminase RNA specific | 1,288 | Nucleus | enzyme |
| ADGRD1 | ENSG00000111452 | adhesion G protein-coupled receptor D1 | 0,887 | Plasma Membrane | G-protein coupled receptor |
| ADGRF4 | ENSG00000153294 | adhesion G protein-coupled receptor F4 | 0,760 | Plasma Membrane | G-protein coupled receptor |
| ADH1C | ENSG00000248144 | alcohol dehydrogenase 1C (class I), gamma polypeptide | -0,670 | Cytoplasm | enzyme |
| ADI1 | ENSG00000182551 | acireductone dioxygenase 1 | -0,714 | Nucleus | enzyme |
| ADM | ENSG00000148926 | adrenomedullin | 1,935 | Extracellular Space | other |
| ADM2 | ENSG00000128165 | adrenomedullin 2 | 0,662 | Extracellular Space | other |
| ADNP2 | ENSG00000101544 | ADNP homeobox 2 | 0,828 | Other | other |
| ADORA1 | ENSG00000163485 | adenosine A1 receptor | -0,681 | Plasma Membrane | G-protein coupled receptor |
| ADORA2A | ENSG00000128271 | adenosine A2a receptor | 2,022 | Plasma Membrane | G-protein coupled receptor |
| ADPRS | ENSG00000116863 | ADP-ribosylserine hydrolase | 0,921 | Cytoplasm | enzyme |
| ADRA2A | ENSG00000150594 | adrenoceptor alpha 2A | -0,880 | Plasma Membrane | G-protein coupled receptor |
| ADRB2 | ENSG00000169252 | adrenoceptor beta 2 | 1,547 | Plasma Membrane | G-protein coupled receptor |
| AEN | ENSG00000181026 | apoptosis enhancing nuclease | 0,841 | Nucleus | enzyme |
| AFAP1 | ENSG00000196526 | actin filament associated protein 1 | 0,984 | Cytoplasm | other |
| AFDN | ENSG00000130396 | afadin, adherens junction formation factor | 0,941 | Nucleus | other |
| AFF1 | ENSG00000172493 | AF4/FMR2 family member 1 | 0,693 | Nucleus | transcription regulator |
| AFF4 | ENSG00000072364 | AF4/FMR2 family member 4 | 1,150 | Nucleus | transcription regulator |
| AGAP6 (includes others) | ENSG00000204149 | ArfGAP with GTPase domain, ankyrin repeat and PH domain 4 | 0,967 | Other | other |
| AGBL5 | ENSG00000084693 | ATP/GTP binding protein like 5 | -0,658 | Cytoplasm | peptidase |
| AGO2 | ENSG00000123908 | argonaute RISC catalytic component 2 | 0,999 | Cytoplasm | translation regulator |
| AGO3 | ENSG00000126070 | argonaute RISC catalytic component 3 | 1,114 | Cytoplasm | translation regulator |
| AGR2 | ENSG00000106541 | anterior gradient 2, protein disulphide isomerase family member | -0,793 | Extracellular Space | other |
| AHNAK | ENSG00000124942 | AHNAK nucleoprotein | -0,969 | Nucleus | other |
| AHNAK2 | ENSG00000185567 | AHNAK nucleoprotein 2 | -0,867 | Cytoplasm | other |
| AHR | ENSG00000106546 | aryl hydrocarbon receptor | 1,839 | Nucleus | ligand-dependent nuclear receptor |
| AHRR | ENSG00000063438 | aryl-hydrocarbon receptor repressor | 2,347 | Nucleus | transcription regulator |
| AIF1L | ENSG00000126878 | allograft inflammatory factor 1 like | -0,792 | Plasma Membrane | other |
| AIG1 | ENSG00000146416 | androgen induced 1 | -0,613 | Other | other |
| AKAP10 | ENSG00000108599 | A-kinase anchoring protein 10 | 0,644 | Cytoplasm | other |
| AKAP13 | ENSG00000170776 | A-kinase anchoring protein 13 | 0,820 | Cytoplasm | other |
| AKAP17A | ENSG00000197976 | A-kinase anchoring protein 17A | 0,605 | Nucleus | other |
| AKAP7 | ENSG00000118507 | A-kinase anchoring protein 7 | 0,849 | Plasma Membrane | other |
| AKAP8L | ENSG00000011243 | A-kinase anchoring protein 8 like | 1,116 | Nucleus | other |
| AKIRIN1 | ENSG00000174574 | akirin 1 | 0,938 | Cytoplasm | enzyme |
| AKR1B10 | ENSG00000198074 | aldo-keto reductase family 1 member B10 | -0,653 | Cytoplasm | enzyme |
| AKR1C1/AKR1C2 | ENSG00000151632 | aldo-keto reductase family 1 member C2 | -0,671 | Cytoplasm | enzyme |
| ALDH7A1 | ENSG00000164904 | aldehyde dehydrogenase 7 family member A1 | -0,699 | Cytoplasm | enzyme |
| ALKBH1 | ENSG00000100601 | alkB homolog 1, histone H2A dioxygenase | 0,775 | Cytoplasm | enzyme |
| ALKBH7 | ENSG00000125652 | alkB homolog 7 | -0,776 | Cytoplasm | other |
| ALOXE3 | ENSG00000179148 | arachidonate lipoxygenase 3 | 0,900 | Extracellular Space | enzyme |
| ALS2 | ENSG00000003393 | alsin Rho guanine nucleotide exchange factor ALS2 | 0,689 | Cytoplasm | other |
| ALX1 | ENSG00000180318 | ALX homeobox 1 | 1,031 | Nucleus | transcription regulator |
| AMBRA1 | ENSG00000110497 | autophagy and beclin 1 regulator 1 | 1,630 | Cytoplasm | other |
| AMER1 | ENSG00000184675 | APC membrane recruitment protein 1 | 0,637 | Plasma Membrane | other |
| AMOTL2 | ENSG00000114019 | angiomotin like 2 | 1,552 | Plasma Membrane | other |
| ANAPC16 | ENSG00000166295 | anaphase promoting complex subunit 16 | -0,636 | Nucleus | other |
| ANGEL1 | ENSG00000013523 | angel homolog 1 | 0,613 | Extracellular Space | other |
| ANGPTL4 | ENSG00000167772 | angiopoietin like 4 | 0,917 | Extracellular Space | other |
| ANKFY1 | ENSG00000185722 | ankyrin repeat and FYVE domain containing 1 | 0,763 | Cytoplasm | transcription regulator |
| ANKLE2 | ENSG00000176915 | ankyrin repeat and LEM domain containing 2 | 1,118 | Nucleus | transcription regulator |
| ANKRD1 | ENSG00000148677 | ankyrin repeat domain 1 | 1,265 | Cytoplasm | transcription regulator |
| ANKRD12 | ENSG00000101745 | ankyrin repeat domain 12 | 1,286 | Nucleus | other |
| ANKRD17 | ENSG00000132466 | ankyrin repeat domain 17 | 0,606 | Nucleus | other |
| ANKRD18B | ENSG00000230453 | ankyrin repeat domain 18B | 0,676 | Other | other |
| ANKRD33B | ENSG00000164236 | ankyrin repeat domain 33B | 2,171 | Other | other |
| ANKRD6 | ENSG00000135299 | ankyrin repeat domain 6 | 0,929 | Nucleus | transcription regulator |
| ANKS1A | ENSG00000064999 | ankyrin repeat and sterile alpha motif domain containing 1A | 0,794 | Cytoplasm | other |
| ANO9 | ENSG00000185101 | anoctamin 9 | 1,611 | Plasma Membrane | ion channel |
| ANXA13 | ENSG00000104537 | annexin A13 | -1,077 | Plasma Membrane | other |
| ANXA2 | ENSG00000182718 | annexin A2 | -0,685 | Plasma Membrane | other |
| ANXA2R | ENSG00000177721 | annexin A2 receptor | -0,819 | Plasma Membrane | other |
| ANXA4 | ENSG00000196975 | annexin A4 | -0,730 | Plasma Membrane | other |
| ANXA9 | ENSG00000143412 | annexin A9 | -0,999 | Plasma Membrane | transmembrane receptor |
| AP4E1 | ENSG00000081014 | adaptor related protein complex 4 subunit epsilon 1 | 0,816 | Cytoplasm | other |
| AP5Z1 | ENSG00000242802 | adaptor related protein complex 5 subunit zeta 1 | 1,238 | Nucleus | other |
| APBA3 | ENSG00000011132 | amyloid beta precursor protein binding family A member 3 | 0,757 | Cytoplasm | transporter |
| APLF | ENSG00000169621 | aprataxin and PNKP like factor | 0,728 | Cytoplasm | enzyme |
| APOBEC3F | ENSG00000128394 | apolipoprotein B mRNA editing enzyme catalytic subunit 3F | 0,896 | Cytoplasm | enzyme |

| | | | | | |
|---|---|---|---|---|---|
| APOBEC3G | ENSG00000239713 | apolipoprotein B mRNA editing enzyme catalytic subunit 3G | 1,400 | Nucleus | enzyme |
| APOE | ENSG00000130203 | apolipoprotein E | -1,035 | Extracellular Space | transporter |
| APOL1 | ENSG00000100342 | apolipoprotein L1 | 0,991 | Extracellular Space | transporter |
| APOL2 | ENSG00000128335 | apolipoprotein L2 | 1,256 | Cytoplasm | other |
| APOL3 | ENSG00000128284 | apolipoprotein L3 | 2,646 | Cytoplasm | transporter |
| APOL4 | ENSG00000100336 | apolipoprotein L4 | 2,161 | Extracellular Space | other |
| APOL6 | ENSG00000221963 | apolipoprotein L6 | 3,125 | Extracellular Space | transporter |
| ARAP2 | ENSG00000047365 | ArfGAP with RhoGAP domain, ankyrin repeat and PH domain 2 | 1,448 | Cytoplasm | other |
| ARC | ENSG00000198576 | activity regulated cytoskeleton associated protein | 0,616 | Cytoplasm | other |
| AREG | ENSG00000109321 | amphiregulin | 0,900 | Extracellular Space | growth factor |
| AREL1 | ENSG00000119682 | apoptosis resistant E3 ubiquitin protein ligase 1 | 0,759 | Cytoplasm | enzyme |
| ARG2 | ENSG00000081181 | arginase 2 | 0,830 | Cytoplasm | enzyme |
| ARHGAP17 | ENSG00000140750 | Rho GTPase activating protein 17 | 0,603 | Cytoplasm | other |
| ARHGAP22 | ENSG00000128805 | Rho GTPase activating protein 22 | 1,387 | Cytoplasm | other |
| ARHGAP26 | ENSG00000145819 | Rho GTPase activating protein 26 | 1,336 | Cytoplasm | other |
| ARHGAP32 | ENSG00000134909 | Rho GTPase activating protein 32 | 0,762 | Cytoplasm | other |
| ARHGAP33 | ENSG00000004777 | Rho GTPase activating protein 33 | -0,903 | Plasma Membrane | transporter |
| ARHGAP42 | ENSG00000165895 | Rho GTPase activating protein 42 | 0,703 | Other | other |
| ARHGAP8/PRR5-ARHGAP8 | ENSG00000248405 | Rho GTPase activating protein 8 | 0,899 | Cytoplasm | other |
| ARHGDIB | ENSG00000111348 | Rho GDP dissociation inhibitor beta | -0,803 | Cytoplasm | enzyme |
| ARHGEF28 | ENSG00000214944 | Rho guanine nucleotide exchange factor 28 | 1,150 | Cytoplasm | other |
| ARHGEF38 | ENSG00000236699 | Rho guanine nucleotide exchange factor 38 | 1,141 | Other | other |
| ARHGEF4 | ENSG00000136002 | Rho guanine nucleotide exchange factor 4 | 1,126 | Cytoplasm | other |
| ARID1A | ENSG00000117713 | AT-rich interaction domain 1A | 0,884 | Nucleus | transcription regulator |
| ARID2 | ENSG00000189079 | AT-rich interaction domain 2 | 0,811 | Nucleus | transcription regulator |
| ARID3B | ENSG00000179361 | AT-rich interaction domain 3B | 1,558 | Nucleus | transcription regulator |
| ARID4B | ENSG00000054267 | AT-rich interaction domain 4B | 1,648 | Nucleus | transcription regulator |
| ARID5A | ENSG00000196843 | AT-rich interaction domain 5A | 0,787 | Nucleus | transcription regulator |
| ARID5B | ENSG00000150347 | AT-rich interaction domain 5B | 1,305 | Nucleus | transcription regulator |
| ARIH1 | ENSG00000166233 | ariadne RBR E3 ubiquitin protein ligase 1 | 0,915 | Cytoplasm | enzyme |
| ARL14 | ENSG00000179674 | ADP ribosylation factor like GTPase 14 | 1,404 | Other | other |
| ARL14EPL | ENSG00000268223 | ADP ribosylation factor like GTPase 14 effector protein like | 3,165 | Other | other |
| ARL3 | ENSG00000138175 | ADP ribosylation factor like GTPase 3 | -0,829 | Cytoplasm | enzyme |
| ARL4A | ENSG00000122644 | ADP ribosylation factor like GTPase 4A | 1,390 | Nucleus | enzyme |
| ARL4C | ENSG00000188042 | ADP ribosylation factor like GTPase 4C | -0,687 | Nucleus | enzyme |
| ARL4D | ENSG00000175906 | ADP ribosylation factor like GTPase 4D | 1,228 | Nucleus | enzyme |
| ARL5B | ENSG00000165997 | ADP ribosylation factor like GTPase 5B | 1,384 | Other | enzyme |
| ARL6IP5 | ENSG00000144746 | ADP ribosylation factor like GTPase 6 interacting protein 5 | -0,689 | Cytoplasm | other |
| ARNTL2 | ENSG00000029153 | aryl hydrocarbon receptor nuclear translocator like 2 | 0,984 | Nucleus | transcription regulator |
| ARPIN/ARPIN-AP3S2 | ENSG00000242498 | ARPIN-AP3S2 readthrough | -0,671 | Plasma Membrane | other |
| ARRB1 | ENSG00000137486 | arrestin beta 1 | -0,734 | Cytoplasm | other |
| ARRDC2 | ENSG00000105643 | arrestin domain containing 2 | 1,081 | Other | other |
| ARRDC3 | ENSG00000113369 | arrestin domain containing 3 | 3,914 | Plasma Membrane | other |
| ARRDC4 | ENSG00000140450 | arrestin domain containing 4 | 2,204 | Plasma Membrane | other |
| ARSL | ENSG00000157399 | arylsulfatase L | -0,966 | Cytoplasm | enzyme |
| ARVCF | ENSG00000099889 | ARVCF delta catenin family member | 0,709 | Plasma Membrane | other |
| AS3MT | ENSG00000214435 | arsenite methyltransferase | -1,043 | Cytoplasm | enzyme |
| ASAH1 | ENSG00000104763 | N-acylsphingosine amidohydrolase 1 | -0,797 | Cytoplasm | enzyme |
| ASAP1 | ENSG00000153317 | ArfGAP with SH3 domain, ankyrin repeat and PH domain 1 | 0,982 | Plasma Membrane | other |
| ASAP2 | ENSG00000151693 | ArfGAP with SH3 domain, ankyrin repeat and PH domain 2 | 0,753 | Nucleus | other |
| ASB2 | ENSG00000100628 | ankyrin repeat and SOCS box containing 2 | 1,118 | Nucleus | transcription regulator |
| ASB7 | ENSG00000183475 | ankyrin repeat and SOCS box containing 7 | 0,622 | Cytoplasm | other |
| ASB9 | ENSG00000102048 | ankyrin repeat and SOCS box containing 9 | -0,701 | Nucleus | transcription regulator |
| ASH1L | ENSG00000116539 | ASH1 like histone lysine methyltransferase | 0,614 | Nucleus | transcription regulator |
| ATAD2B | ENSG00000119778 | ATPase family AAA domain containing 2B | 1,398 | Nucleus | other |
| ATF3 | ENSG00000162772 | activating transcription factor 3 | 5,420 | Nucleus | transcription regulator |
| ATF4 | ENSG00000128272 | activating transcription factor 4 | 0,627 | Nucleus | transcription regulator |
| ATF7IP | ENSG00000171681 | activating transcription factor 7 interacting protein | 0,740 | Nucleus | transcription regulator |
| ATG2B | ENSG00000066739 | autophagy related 2B | 1,095 | Nucleus | other |
| ATP10D | ENSG00000145246 | ATPase phospholipid transporting 10D (putative) | 0,711 | Cytoplasm | transporter |
| ATP13A4 | ENSG00000127249 | ATPase 13A4 | 1,281 | Cytoplasm | transporter |
| ATP1A1 | ENSG00000163399 | ATPase Na+/K+ transporting subunit alpha 1 | -0,602 | Plasma Membrane | transporter |
| ATP23 | ENSG00000166896 | ATP23 metallopeptidase and ATP synthase assembly factor homolog | -1,044 | Plasma Membrane | kinase |
| ATP4A | ENSG00000105675 | ATPase H+/K+ transporting subunit alpha | 2,073 | Plasma Membrane | transporter |
| ATP5IF1 | ENSG00000130770 | ATP synthase inhibitory factor subunit 1 | -0,801 | Cytoplasm | other |
| ATP5MPL | ENSG00000156411 | ATP synthase membrane subunit 6.8PL | -0,655 | Cytoplasm | other |
| ATP5PD | ENSG00000167863 | ATP synthase peripheral stalk subunit d | -0,602 | Cytoplasm | enzyme |
| ATP5PF | ENSG00000154723 | ATP synthase peripheral stalk subunit F6 | -0,646 | Cytoplasm | transporter |
| ATP8B2 | ENSG00000143515 | ATPase phospholipid transporting 8B2 | 0,648 | Plasma Membrane | transporter |
| ATRAID | ENSG00000138085 | all-trans retinoic acid induced differentiation factor | -0,657 | Nucleus | other |
| ATXN2 | ENSG00000204842 | ataxin 2 | 0,631 | Nucleus | other |
| ATXN2L | ENSG00000168488 | ataxin 2 like | 0,720 | Nucleus | other |
| ATXN7 | ENSG00000163635 | ataxin 7 | 0,928 | Nucleus | other |
| ATXN7L2 | ENSG00000162650 | ataxin 7 like 2 | 0,782 | Other | other |
| AZI2 | ENSG00000163512 | 5-azacytidine induced 2 | 1,173 | Cytoplasm | other |
| AZIN1 | ENSG00000155096 | antizyme inhibitor 1 | 0,978 | Cytoplasm | enzyme |
| B2M | ENSG00000166710 | beta-2-microglobulin | 0,608 | Plasma Membrane | transmembrane receptor |
| B3GALNT2 | ENSG00000162885 | beta-1,3-N-acetylgalactosaminyltransferase 2 | 0,860 | Cytoplasm | enzyme |
| B3GNT2 | ENSG00000170340 | UDP-GlcNAc:betaGal beta-1,3-N-acetylglucosaminyltransferase 2 | 0,844 | Cytoplasm | enzyme |
| B3GNT5 | ENSG00000176597 | UDP-GlcNAc:betaGal beta-1,3-N-acetylglucosaminyltransferase 5 | 1,465 | Cytoplasm | enzyme |
| B4GALT5 | ENSG00000158470 | beta-1,4-galactosyltransferase 5 | 1,133 | Cytoplasm | enzyme |
| BAAT | ENSG00000136881 | bile acid-CoA:amino acid N-acyltransferase | 1,048 | Cytoplasm | enzyme |
| BACH1 | ENSG00000156273 | BTB domain and CNC homolog 1 | 0,989 | Nucleus | transcription regulator |
| BACH2 | ENSG00000112182 | BTB domain and CNC homolog 2 | 2,024 | Nucleus | transcription regulator |
| BAG1 | ENSG00000107262 | BAG cochaperone 1 | 0,762 | Cytoplasm | other |
| BAG3 | ENSG00000151929 | BAG cochaperone 3 | 1,595 | Cytoplasm | other |
| BAIAP2L2 | ENSG00000128298 | BAR/IMD domain containing adaptor protein 2 like 2 | 1,192 | Cytoplasm | other |
| BANP | ENSG00000172530 | BTG3 associated nuclear protein | 0,724 | Nucleus | other |
| BATF2 | ENSG00000168062 | basic leucine zipper ATF-like transcription factor 2 | 3,770 | Other | other |
| BAZ1A | ENSG00000198604 | bromodomain adjacent to zinc finger domain 1A | 1,033 | Nucleus | other |
| BAZ2A | ENSG00000076108 | bromodomain adjacent to zinc finger domain 2A | 0,791 | Nucleus | transcription regulator |
| BAZ2B | ENSG00000123636 | bromodomain adjacent to zinc finger domain 2B | 1,641 | Extracellular Space | other |
| BBC3 | ENSG00000105327 | BCL2 binding component 3 | 3,753 | Cytoplasm | other |
| BCAR1 | ENSG00000050820 | BCAR1 scaffold protein, Cas family member | 0,646 | Plasma Membrane | enzyme |
| BCAS1 | ENSG00000064787 | brain enriched myelin associated protein 1 | -1,459 | Plasma Membrane | other |
| BCKDHB | ENSG00000083123 | branched chain keto acid dehydrogenase E1 subunit beta | -0,654 | Cytoplasm | enzyme |
| BCL10 | ENSG00000142867 | BCL10 immune signaling adaptor | 1,230 | Cytoplasm | transcription regulator |
| BCL11A | ENSG00000119866 | BAF chromatin remodeling complex subunit BCL11A | -0,656 | Nucleus | transcription regulator |
| BCL2L11 | ENSG00000153094 | BCL2 like 11 | 1,224 | Cytoplasm | other |

| | | | | | |
|---|---|---|---|---|---|
| BCL2L14 | ENSG00000121380 | BCL2 like 14 | 1,044 | Cytoplasm | other |
| BCL3 | ENSG00000069399 | BCL3 transcription coactivator | 2,637 | Nucleus | transcription regulator |
| BCL6 | ENSG00000113916 | BCL6 transcription repressor | 1,074 | Nucleus | transcription regulator |
| BCL9L | ENSG00000186174 | BCL9 like | 0,698 | Cytoplasm | other |
| BDKRB2 | ENSG00000168398 | bradykinin receptor B2 | 0,727 | Plasma Membrane | G-protein coupled receptor |
| BDNF | ENSG00000176697 | brain derived neurotrophic factor | 1,190 | Extracellular Space | growth factor |
| BDP1 | ENSG00000145734 | B double prime 1, subunit of RNA polymerase III transcription initiation factor IIIB | 0,809 | Nucleus | transcription regulator |
| BEST1 | ENSG00000167995 | bestrophin 1 | 0,998 | Plasma Membrane | ion channel |
| BHLHE40 | ENSG00000134107 | basic helix-loop-helix family member e40 | 1,212 | Nucleus | transcription regulator |
| BHLHE41 | ENSG00000123095 | basic helix-loop-helix family member e41 | 0,711 | Nucleus | transcription regulator |
| BIRC2 | ENSG00000110330 | baculoviral IAP repeat containing 2 | 0,650 | Cytoplasm | enzyme |
| BIRC3 | ENSG00000023445 | baculoviral IAP repeat containing 3 | 1,966 | Cytoplasm | enzyme |
| BIRC6 | ENSG00000115760 | baculoviral IAP repeat containing 6 | 0,706 | Cytoplasm | enzyme |
| BLOC1S1 | ENSG00000135441 | biogenesis of lysosomal organelles complex 1 subunit 1 | -0,708 | Cytoplasm | other |
| BLZF1 | ENSG00000117475 | basic leucine zipper nuclear factor 1 | 0,889 | Cytoplasm | transcription regulator |
| BMF | ENSG00000104081 | Bcl2 modifying factor | 1,161 | Cytoplasm | other |
| BMP2 | ENSG00000125845 | bone morphogenetic protein 2 | 2,801 | Extracellular Space | growth factor |
| BMT2 | ENSG00000164603 | base methyltransferase of 25S rRNA 2 homolog | 1,650 | Other | other |
| BNIP3L | ENSG00000104765 | BCL2 interacting protein 3 like | -1,163 | Cytoplasm | other |
| BNIP3P1 | ENSG00000197358 | BCL2 interacting protein 3 pseudogene 1 | -1,017 | Other | other |
| BNIP5 | ENSG00000189325 | BCL2 interacting protein 5 | 1,272 | Other | other |
| BOLA2/BOLA2B | ENSG00000183336 | bolA family member 2 | -0,670 | Cytoplasm | enzyme |
| BORCS8-MEF2B | ENSG00000064489 | BORCS8-MEF2B readthrough | -1,556 | Nucleus | transcription regulator |
| BPIFB1 | ENSG00000125999 | BPI fold containing family B member 1 | -0,701 | Extracellular Space | other |
| BRAF | ENSG00000157764 | B-Raf proto-oncogene, serine/threonine kinase | 1,015 | Cytoplasm | kinase |
| BRD1 | ENSG00000100425 | bromodomain containing 1 | 0,806 | Nucleus | other |
| BRD2 | ENSG00000236227 | bromodomain containing 2 | 1,764 | Nucleus | kinase |
| BRD4 | ENSG00000141867 | bromodomain containing 4 | 0,793 | Nucleus | kinase |
| BRF2 | ENSG00000104221 | BRF2 RNA polymerase III transcription initiation factor subunit | 1,083 | Nucleus | transcription regulator |
| BRIP1 | ENSG00000136492 | BRCA1 interacting protein C-terminal helicase 1 | 0,627 | Nucleus | enzyme |
| BRK1 | ENSG00000254999 | BRICK1 subunit of SCAR/WAVE actin nucleating complex | -0,603 | Cytoplasm | other |
| BRPF1 | ENSG00000156983 | bromodomain and PHD finger containing 1 | 1,043 | Nucleus | transporter |
| BRPF3 | ENSG00000096070 | bromodomain and PHD finger containing 3 | 0,955 | Cytoplasm | other |
| BRSK1 | ENSG00000160469 | BR serine/threonine kinase 1 | 0,810 | Cytoplasm | kinase |
| BRSK2 | ENSG00000174672 | BR serine/threonine kinase 2 | 2,022 | Cytoplasm | kinase |
| BRWD3 | ENSG00000165288 | bromodomain and WD repeat domain containing 3 | 0,998 | Other | other |
| BST2 | ENSG00000130303 | bone marrow stromal cell antigen 2 | 3,516 | Plasma Membrane | other |
| BTBD19 | ENSG00000222009 | BTB domain containing 19 | 1,519 | Other | other |
| BTC | ENSG00000174808 | betacellulin | 0,929 | Extracellular Space | growth factor |
| BTG2 | ENSG00000159388 | BTG anti-proliferation factor 2 | 1,674 | Nucleus | transcription regulator |
| BTN2A1 | ENSG00000112763 | butyrophilin subfamily 2 member A1 | 0,776 | Plasma Membrane | other |
| BTN2A2 | ENSG00000124508 | butyrophilin subfamily 2 member A2 | 1,480 | Cytoplasm | other |
| BTN3A1 | ENSG00000026950 | butyrophilin subfamily 3 member A1 | 1,398 | Extracellular Space | other |
| BTN3A3 | ENSG00000111801 | butyrophilin subfamily 3 member A3 | 1,357 | Plasma Membrane | other |
| C10orf55 | ENSG00000222047 | chromosome 10 open reading frame 55 (putative) | 0,886 | Other | other |
| C11orf24 | ENSG00000171067 | chromosome 11 open reading frame 24 | 0,683 | Extracellular Space | other |
| C11orf45 | ENSG00000174370 | chromosome 11 open reading frame 45 | -0,685 | Extracellular Space | other |
| C11orf96 | ENSG00000187479 | chromosome 11 open reading frame 96 | 2,480 | Other | other |
| C15orf39 | ENSG00000167173 | chromosome 15 open reading frame 39 | 0,741 | Cytoplasm | other |
| C15orf48 | ENSG00000166920 | chromosome 15 open reading frame 48 | 1,388 | Nucleus | other |
| C15orf62 | ENSG00000188277 | chromosome 15 open reading frame 62 | -0,747 | Other | other |
| C16orf46 | ENSG00000166455 | chromosome 16 open reading frame 46 | 0,634 | Nucleus | other |
| C16orf70 | ENSG00000125149 | chromosome 16 open reading frame 70 | 0,800 | Cytoplasm | other |
| C16orf72 | ENSG00000182831 | chromosome 16 open reading frame 72 | 0,679 | Other | other |
| C16orf87 | ENSG00000155330 | chromosome 16 open reading frame 87 | 0,828 | Other | other |
| C17orf67 | ENSG00000214226 | chromosome 17 open reading frame 67 | 0,779 | Other | other |
| C17orf75 | ENSG00000108666 | chromosome 17 open reading frame 75 | -0,657 | Extracellular Space | other |
| C18orf25 | ENSG00000152242 | chromosome 18 open reading frame 25 | 1,141 | Other | other |
| C1orf21 | ENSG00000116667 | chromosome 1 open reading frame 21 | -0,607 | Other | other |
| C1orf52 | ENSG00000162642 | chromosome 1 open reading frame 52 | 1,026 | Nucleus | other |
| C1QL4 | ENSG00000186897 | complement C1q like 4 | -0,722 | Extracellular Space | other |
| C1QTNF1 | ENSG00000173918 | C1q and TNF related 1 | 1,977 | Extracellular Space | other |
| C1S | ENSG00000182326 | complement C1s | 1,585 | Extracellular Space | peptidase |
| C21orf91 | ENSG00000154642 | chromosome 21 open reading frame 91 | 1,337 | Other | other |
| C2CD4A | ENSG00000198535 | C2 calcium dependent domain containing 4A | 0,784 | Nucleus | other |
| C2orf42 | ENSG00000115998 | chromosome 2 open reading frame 42 | 0,642 | Nucleus | other |
| C3 | ENSG00000125730 | complement C3 | 1,528 | Extracellular Space | peptidase |
| C3orf33 | ENSG00000174928 | chromosome 3 open reading frame 33 | 0,606 | Extracellular Space | other |
| C3orf38 | ENSG00000179021 | chromosome 3 open reading frame 38 | 0,983 | Nucleus | peptidase |
| C3orf52 | ENSG00000114529 | chromosome 3 open reading frame 52 | 1,477 | Other | other |
| C4A/C4B | ENSG00000244731 | complement C4B (Chido blood group) | 1,985 | Extracellular Space | other |
| C4orf3 | ENSG00000164096 | chromosome 4 open reading frame 3 | -0,703 | Other | other |
| C5 | ENSG00000106804 | complement C5 | -1,046 | Extracellular Space | cytokine |
| C6orf58 | ENSG00000184530 | chromosome 6 open reading frame 58 | 3,105 | Cytoplasm | other |
| C6orf62 | ENSG00000112308 | chromosome 6 open reading frame 62 | 0,835 | Other | other |
| CA9 | ENSG00000107159 | carbonic anhydrase 9 | -1,534 | Nucleus | enzyme |
| CACNG4 | ENSG00000075461 | calcium voltage-gated channel auxiliary subunit gamma 4 | -0,863 | Plasma Membrane | ion channel |
| CALB1 | ENSG00000104327 | calbindin 1 | -1,187 | Cytoplasm | other |
| CALCA | ENSG00000110680 | calcitonin related polypeptide alpha | 1,533 | Plasma Membrane | other |
| CAPN8 | ENSG00000203697 | calpain 8 | -0,604 | Cytoplasm | peptidase |
| CAPS | ENSG00000105519 | calcyphosine | -0,873 | Cytoplasm | other |
| CARD14 | ENSG00000141527 | caspase recruitment domain family member 14 | 1,214 | Cytoplasm | other |
| CARD16 | ENSG00000204397 | caspase recruitment domain family member 16 | 1,299 | Other | other |
| CARD6 | ENSG00000132357 | caspase recruitment domain family member 6 | 0,721 | Cytoplasm | other |
| CARHSP1 | ENSG00000153048 | calcium regulated heat stable protein 1 | -0,810 | Cytoplasm | transcription regulator |
| CARS1 | ENSG00000110619 | cysteinyl-tRNA synthetase 1 | 0,667 | Cytoplasm | enzyme |
| CASP1 | ENSG00000137752 | caspase 1 | 2,264 | Cytoplasm | peptidase |
| CASP10 | ENSG00000003400 | caspase 10 | 0,903 | Cytoplasm | peptidase |
| CASP7 | ENSG00000165806 | caspase 7 | 1,176 | Cytoplasm | peptidase |
| CASP8 | ENSG00000064012 | caspase 8 | 0,691 | Nucleus | peptidase |
| CASR | ENSG00000036828 | calcium sensing receptor | -0,651 | Plasma Membrane | G-protein coupled receptor |
| CAT | ENSG00000121691 | catalase | -0,967 | Cytoplasm | enzyme |
| CBARP | ENSG00000099625 | CACN subunit beta associated regulatory protein | 0,680 | Other | other |
| CBFA2T2 | ENSG00000078699 | CBFA2/RUNX1 partner transcriptional co-repressor 2 | 0,880 | Nucleus | transcription regulator |
| CBLB | ENSG00000114423 | Cbl proto-oncogene B | 0,741 | Nucleus | enzyme |
| CBR3 | ENSG00000159231 | carbonyl reductase 3 | 1,034 | Cytoplasm | enzyme |
| CBWD3/CBWD6 | ENSG00000215126 | COBW domain containing 3 | 1,160 | Other | other |
| CBX4 | ENSG00000141582 | chromobox 4 | 0,740 | Nucleus | transcription regulator |
| CBX7 | ENSG00000100307 | chromobox 7 | -0,913 | Nucleus | other |

| | | | | | |
|---|---|---|---|---|---|
| CC2D1B | ENSG00000154222 | coiled-coil and C2 domain containing 1B | 0,892 | Nucleus | transcription regulator |
| CCDC115 | ENSG00000136710 | coiled-coil domain containing 115 | -0,675 | Cytoplasm | other |
| CCDC117 | ENSG00000159873 | coiled-coil domain containing 117 | 1,598 | Other | other |
| CCDC130 | ENSG00000104957 | coiled-coil domain containing 130 | 0,969 | Other | other |
| CCDC142 | ENSG00000135637 | coiled-coil domain containing 142 | 0,782 | Other | other |
| CCDC157 | ENSG00000187860 | coiled-coil domain containing 157 | 0,887 | Other | other |
| CCDC167 | ENSG00000198937 | coiled-coil domain containing 167 | -0,829 | Other | other |
| CCDC174 | ENSG00000154781 | coiled-coil domain containing 174 | 1,062 | Nucleus | other |
| CCDC186 | ENSG00000165813 | coiled-coil domain containing 186 | 0,853 | Cytoplasm | other |
| CCDC34 | ENSG00000109881 | coiled-coil domain containing 34 | -0,799 | Other | other |
| CCDC85A | ENSG00000055813 | coiled-coil domain containing 85A | 0,857 | Other | other |
| CCDC9 | ENSG00000105321 | coiled-coil domain containing 9 | 0,701 | Extracellular Space | other |
| CCHCR1 | ENSG00000206355 | coiled-coil alpha-helical rod protein 1 | 0,971 | Cytoplasm | other |
| CCL2 | ENSG00000108691 | C-C motif chemokine ligand 2 | 3,549 | Extracellular Space | cytokine |
| CCL20 | ENSG00000115009 | C-C motif chemokine ligand 20 | 3,266 | Extracellular Space | cytokine |
| CCL22 | ENSG00000102962 | C-C motif chemokine ligand 22 | 0,621 | Extracellular Space | cytokine |
| CCL5 | ENSG00000271503 | C-C motif chemokine ligand 5 | 4,564 | Extracellular Space | cytokine |
| CCN1 | ENSG00000142871 | cellular communication network factor 1 | 0,833 | Extracellular Space | other |
| CCNB1IP1 | ENSG00000100814 | cyclin B1 interacting protein 1 | 0,847 | Nucleus | enzyme |
| CCNB2 | ENSG00000157456 | cyclin B2 | -0,747 | Cytoplasm | other |
| CCNL1 | ENSG00000163660 | cyclin L1 | 1,802 | Nucleus | other |
| CCNT2 | ENSG00000082258 | cyclin T2 | 1,049 | Nucleus | transcription regulator |
| CCR1 | ENSG00000163823 | C-C motif chemokine receptor 1 | 1,300 | Plasma Membrane | G-protein coupled receptor |
| CCR4 | ENSG00000183813 | C-C motif chemokine receptor 4 | 0,661 | Plasma Membrane | G-protein coupled receptor |
| CCT6P3 | ENSG00000234585 | chaperonin containing TCP1 subunit 6 pseudogene 3 | 0,971 | Other | other |
| CD24 | ENSG00000272398 | CD24 molecule | -0,703 | Plasma Membrane | other |
| CD274 | ENSG00000120217 | CD274 molecule | 4,080 | Plasma Membrane | enzyme |
| CD2AP | ENSG00000198087 | CD2 associated protein | 0,783 | Cytoplasm | other |
| CD40 | ENSG00000101017 | CD40 molecule | 0,835 | Plasma Membrane | transmembrane receptor |
| CD63 | ENSG00000135404 | CD63 molecule | -0,614 | Plasma Membrane | other |
| CD68 | ENSG00000129226 | CD68 molecule | 1,461 | Plasma Membrane | other |
| CD69 | ENSG00000110848 | CD69 molecule | 1,949 | Plasma Membrane | transmembrane receptor |
| CD83 | ENSG00000112149 | CD83 molecule | 1,236 | Plasma Membrane | transmembrane receptor |
| CDA | ENSG00000158825 | cytidine deaminase | -0,717 | Nucleus | enzyme |
| CDC14A | ENSG00000079335 | cell division cycle 14A | 1,026 | Nucleus | phosphatase |
| CDC37L1 | ENSG00000106993 | cell division cycle 37 like 1 | 0,705 | Cytoplasm | other |
| CDC42EP2 | ENSG00000149798 | CDC42 effector protein 2 | 0,795 | Plasma Membrane | other |
| CDC42EP3 | ENSG00000163171 | CDC42 effector protein 3 | 0,881 | Cytoplasm | other |
| CDCA3 | ENSG00000111665 | cell division cycle associated 3 | -0,720 | Cytoplasm | other |
| CDK12 | ENSG00000167258 | cyclin dependent kinase 12 | 0,860 | Nucleus | kinase |
| CDK17 | ENSG00000059758 | cyclin dependent kinase 17 | 1,680 | Cytoplasm | kinase |
| CDK18 | ENSG00000117266 | cyclin dependent kinase 18 | 0,829 | Cytoplasm | kinase |
| CDK5 | ENSG00000164885 | cyclin dependent kinase 5 | -0,734 | Nucleus | kinase |
| CDK8 | ENSG00000132964 | cyclin dependent kinase 8 | 0,639 | Nucleus | kinase |
| CDKN1A | ENSG00000124762 | cyclin dependent kinase inhibitor 1A | 1,260 | Nucleus | kinase |
| CDKN1B | ENSG00000111276 | cyclin dependent kinase inhibitor 1B | 0,613 | Nucleus | kinase |
| CDKN2AIP | ENSG00000168564 | CDKN2A interacting protein | 1,232 | Nucleus | transcription regulator |
| CDKN2B | ENSG00000147883 | cyclin dependent kinase inhibitor 2B | 1,601 | Nucleus | transcription regulator |
| CDKN3 | ENSG00000100526 | cyclin dependent kinase inhibitor 3 | -0,617 | Cytoplasm | phosphatase |
| CDR2 | ENSG00000140743 | cerebellar degeneration related protein 2 | 0,709 | Cytoplasm | other |
| CDRT1 | ENSG00000241322 | CMT1A duplicated region transcript 1 | 1,713 | Other | other |
| CEACAM1 | ENSG00000079385 | CEA cell adhesion molecule 1 | 1,557 | Plasma Membrane | transporter |
| CEBPB | ENSG00000172216 | CCAAT enhancer binding protein beta | 1,138 | Nucleus | transcription regulator |
| CEBPD | ENSG00000221869 | CCAAT enhancer binding protein delta | 1,254 | Nucleus | transcription regulator |
| CELSR1 | ENSG00000075275 | cadherin EGF LAG seven-pass G-type receptor 1 | -0,657 | Plasma Membrane | G-protein coupled receptor |
| CELSR3 | ENSG00000008300 | cadherin EGF LAG seven-pass G-type receptor 3 | 0,907 | Plasma Membrane | G-protein coupled receptor |
| CEMIP2 | ENSG00000135048 | cell migration inducing hyaluronidase 2 | 0,604 | Cytoplasm | enzyme |
| CENPA | ENSG00000115163 | centromere protein A | -0,739 | Nucleus | other |
| CENPF | ENSG00000117724 | centromere protein F | -0,734 | Nucleus | other |
| CENPW | ENSG00000203760 | centromere protein W | -0,655 | Nucleus | other |
| CEP135 | ENSG00000174799 | centrosomal protein 135 | 0,650 | Cytoplasm | other |
| CEP164 | ENSG00000110274 | centrosomal protein 164 | 0,697 | Cytoplasm | other |
| CEP350 | ENSG00000135837 | centrosomal protein 350 | 1,290 | Cytoplasm | other |
| CERCAM | ENSG00000167123 | cerebral endothelial cell adhesion molecule | -0,985 | Other | other |
| CETN2 | ENSG00000147400 | centrin 2 | -0,615 | Nucleus | enzyme |
| CFAP410 | ENSG00000160226 | cilia and flagella associated protein 410 | -0,825 | Cytoplasm | other |
| CFAP54 | ENSG00000188596 | cilia and flagella associated protein 54 | 0,992 | Cytoplasm | other |
| CFAP57 | ENSG00000243710 | cilia and flagella associated protein 57 | 0,753 | Extracellular Space | other |
| CFAP91 | ENSG00000183833 | cilia and flagella associated protein 91 | 1,487 | Cytoplasm | other |
| CFB | ENSG00000241253 | complement factor B | 3,042 | Extracellular Space | peptidase |
| CFH | ENSG00000000971 | complement factor H | 1,120 | Extracellular Space | other |
| CFI | ENSG00000205403 | complement factor I | -0,916 | Extracellular Space | peptidase |
| CFLAR | ENSG00000003402 | CASP8 and FADD like apoptosis regulator | 0,986 | Cytoplasm | other |
| CFTR | ENSG00000001626 | CF transmembrane conductance regulator | -0,620 | Plasma Membrane | ion channel |
| CGAS | ENSG00000164430 | cyclic GMP-AMP synthase | 1,271 | Cytoplasm | enzyme |
| CGB3 (includes others) | ENSG00000213030 | chorionic gonadotropin subunit beta 3 | -1,592 | Extracellular Space | other |
| CH25H | ENSG00000138135 | cholesterol 25-hydroxylase | 5,444 | Cytoplasm | enzyme |
| CHAC1 | ENSG00000128965 | ChaC glutathione specific gamma-glutamylcyclotransferase 1 | 1,476 | Cytoplasm | enzyme |
| CHD1 | ENSG00000153922 | chromodomain helicase DNA binding protein 1 | 1,073 | Nucleus | enzyme |
| CHD2 | ENSG00000173575 | chromodomain helicase DNA binding protein 2 | 1,837 | Nucleus | enzyme |
| CHD4 | ENSG00000111642 | chromodomain helicase DNA binding protein 4 | 0,685 | Nucleus | enzyme |
| CHD7 | ENSG00000171316 | chromodomain helicase DNA binding protein 7 | 0,817 | Nucleus | enzyme |
| CHD8 | ENSG00000100888 | chromodomain helicase DNA binding protein 8 | 0,923 | Nucleus | enzyme |
| CHD9 | ENSG00000177200 | chromodomain helicase DNA binding protein 9 | 1,159 | Cytoplasm | other |
| CHIC2 | ENSG00000109220 | cysteine rich hydrophobic domain 2 | 0,873 | Plasma Membrane | other |
| CHKB | ENSG00000100288 | choline kinase beta | 0,995 | Cytoplasm | kinase |
| CHORDC1 | ENSG00000110172 | cysteine and histidine rich domain containing 1 | 0,849 | Other | other |
| CHST2 | ENSG00000175040 | carbohydrate sulfotransferase 2 | 0,868 | Cytoplasm | enzyme |
| CHSY3 | ENSG00000198108 | chondroitin sulfate synthase 3 | 0,910 | Cytoplasm | enzyme |
| CIC | ENSG00000079432 | capicua transcriptional repressor | 0,610 | Nucleus | transcription regulator |
| CIITA | ENSG00000179583 | class II major histocompatibility complex transactivator | 1,699 | Nucleus | transcription regulator |
| CISH | ENSG00000114737 | cytokine inducible SH2 containing protein | 0,708 | Cytoplasm | other |
| CIT | ENSG00000122966 | citron rho-interacting serine/threonine kinase | -0,625 | Cytoplasm | kinase |
| CITED2 | ENSG00000164442 | Cbp/p300 interacting transactivator with Glu/Asp rich carboxy-terminal domain 2 | 1,847 | Nucleus | transcription regulator |
| CLCN2 | ENSG00000114859 | chloride voltage-gated channel 2 | 0,998 | Plasma Membrane | ion channel |
| CLCN4 | ENSG00000073464 | chloride voltage-gated channel 4 | 0,609 | Plasma Membrane | ion channel |
| CLDN10 | ENSG00000134873 | claudin 10 | -1,206 | Plasma Membrane | other |
| CLDN16 | ENSG00000113946 | claudin 16 | -0,711 | Plasma Membrane | transporter |
| CLEC16A | ENSG00000038532 | C-type lectin domain containing 16A | 0,625 | Cytoplasm | other |

| | | | | | |
|---|---|---|---|---|---|
| CLEC2D | ENSG00000069493 | C-type lectin domain family 2 member D | 1,258 | Plasma Membrane | transmembrane receptor |
| CLIP1 | ENSG00000130779 | CAP-Gly domain containing linker protein 1 | 0,649 | Cytoplasm | other |
| CLK1 | ENSG00000013441 | CDC like kinase 1 | 1,784 | Nucleus | kinase |
| CLK2 | ENSG00000261893 | CDC like kinase 2 | 0,705 | Nucleus | kinase |
| CLK4 | ENSG00000113240 | CDC like kinase 4 | 1,587 | Nucleus | kinase |
| CLTRN | ENSG00000147003 | collectrin, amino acid transport regulator | 1,223 | Plasma Membrane | other |
| CMPK2 | ENSG00000134326 | cytidine/uridine monophosphate kinase 2 | 4,267 | Cytoplasm | kinase |
| CMTM4 | ENSG00000183723 | CKLF like MARVEL transmembrane domain containing 4 | -0,619 | Extracellular Space | cytokine |
| CMTR1 | ENSG00000137200 | cap methyltransferase 1 | 1,263 | Nucleus | enzyme |
| CMTR2 | ENSG00000180917 | cap methyltransferase 2 | 0,601 | Other | enzyme |
| CNKSR1 | ENSG00000142675 | connector enhancer of kinase suppressor of Ras 1 | 0,718 | Cytoplasm | other |
| CNKSR3 | ENSG00000153721 | CNKSR family member 3 | 1,902 | Plasma Membrane | kinase |
| CNOT4 | ENSG00000080802 | CCR4-NOT transcription complex subunit 4 | 0,905 | Cytoplasm | enzyme |
| CNOT6LP1 | ENSG00000230183 | CNOT6L pseudogene 1 | 2,541 | Other | other |
| CNST | ENSG00000162852 | consortin, connexin sorting protein | 0,676 | Cytoplasm | other |
| CNTNAP1 | ENSG00000108797 | contactin associated protein 1 | 1,106 | Plasma Membrane | other |
| COA3 | ENSG00000183978 | cytochrome c oxidase assembly factor 3 | -0,756 | Cytoplasm | other |
| COG3 | ENSG00000136152 | component of oligomeric golgi complex 3 | 0,859 | Cytoplasm | other |
| COL11A2 | ENSG00000230930 | collagen type XI alpha 2 chain | 1,997 | Extracellular Space | other |
| COL12A1 | ENSG00000111799 | collagen type XII alpha 1 chain | 0,830 | Extracellular Space | other |
| COL16A1 | ENSG00000084636 | collagen type XVI alpha 1 chain | 2,414 | Extracellular Space | other |
| COL1A1 | ENSG00000108821 | collagen type I alpha 1 chain | 0,953 | Extracellular Space | other |
| COL4A1 | ENSG00000187498 | collagen type IV alpha 1 chain | 0,794 | Extracellular Space | other |
| COL7A1 | ENSG00000114270 | collagen type VII alpha 1 chain | 2,871 | Extracellular Space | other |
| COLCA2 | ENSG00000214290 | colorectal cancer associated 2 | -0,644 | Cytoplasm | other |
| COMMD3-BMI1 | ENSG00000269897 | COMMD3-BMI1 readthrough | -0,859 | Nucleus | transcription regulator |
| COMMD6 | ENSG00000188243 | COMM domain containing 6 | -0,733 | Other | other |
| COMMD9 | ENSG00000110442 | COMM domain containing 9 | -0,654 | Cytoplasm | other |
| COPS9 | ENSG00000172428 | COP9 signalosome subunit 9 | -0,608 | Nucleus | other |
| COQ10B | ENSG00000115520 | coenzyme Q10B | 0,633 | Cytoplasm | other |
| CPAMD8 | ENSG00000160111 | C3 and PZP like alpha-2-macroglobulin domain containing 8 | 1,433 | Extracellular Space | other |
| CPEB2 | ENSG00000137449 | cytoplasmic polyadenylation element binding protein 2 | 2,666 | Cytoplasm | translation regulator |
| CPEB3 | ENSG00000107864 | cytoplasmic polyadenylation element binding protein 3 | 2,091 | Cytoplasm | translation regulator |
| CPEB4 | ENSG00000113742 | cytoplasmic polyadenylation element binding protein 4 | 1,991 | Plasma Membrane | other |
| CPNE8 | ENSG00000139117 | copine 8 | 0,680 | Cytoplasm | other |
| CPSF1P1 | ENSG00000214076 | cleavage and polyadenylation specific factor 1 pseudogene 1 | 0,863 | Other | other |
| CPXM1 | ENSG00000088882 | carboxypeptidase X, M14 family member 1 | 1,723 | Extracellular Space | peptidase |
| CR753842.1 | ENSG00000258589 | | 2,217 | Other | other |
| CRAMP1 | ENSG00000007545 | cramped chromatin regulator homolog 1 | 0,849 | Other | other |
| CRAT | ENSG00000095321 | carnitine O-acetyltransferase | -0,603 | Cytoplasm | enzyme |
| CREB1 | ENSG00000118260 | cAMP responsive element binding protein 1 | 0,787 | Nucleus | transcription regulator |
| CREB5 | ENSG00000146592 | cAMP responsive element binding protein 5 | 3,762 | Nucleus | transcription regulator |
| CREBBP | ENSG00000005339 | CREB binding protein | 1,146 | Nucleus | transcription regulator |
| CREBRF | ENSG00000164463 | CREB3 regulatory factor | 1,419 | Nucleus | transcription regulator |
| CRIM1 | ENSG00000150938 | cysteine rich transmembrane BMP regulator 1 | 0,601 | Extracellular Space | kinase |
| CRIP2 | ENSG00000182809 | cysteine rich protein 2 | -0,904 | Nucleus | other |
| CRKL | ENSG00000099942 | CRK like proto-oncogene, adaptor protein | 0,642 | Cytoplasm | kinase |
| CRPPA | ENSG00000214960 | CDP-L-ribitol pyrophosphorylase A | -0,976 | Cytoplasm | enzyme |
| CRTC2 | ENSG00000160741 | CREB regulated transcription coactivator 2 | 0,611 | Nucleus | other |
| CRY1 | ENSG00000008405 | cryptochrome circadian regulator 1 | 0,708 | Nucleus | enzyme |
| CRY2 | ENSG00000121671 | cryptochrome circadian regulator 2 | 0,621 | Nucleus | enzyme |
| CRYBG2 | ENSG00000176092 | crystallin beta-gamma domain containing 2 | 1,049 | Extracellular Space | other |
| CRYL1 | ENSG00000165475 | crystallin lambda 1 | -0,645 | Cytoplasm | enzyme |
| CRYZ | ENSG00000116791 | crystallin zeta | -0,805 | Cytoplasm | enzyme |
| CSF1 | ENSG00000184371 | colony stimulating factor 1 | 2,643 | Extracellular Space | cytokine |
| CSF1R | ENSG00000182578 | colony stimulating factor 1 receptor | 1,337 | Plasma Membrane | kinase |
| CSF2 | ENSG00000164400 | colony stimulating factor 2 | 3,214 | Extracellular Space | cytokine |
| CSF3 | ENSG00000108342 | colony stimulating factor 3 | 3,788 | Extracellular Space | cytokine |
| CSNK1G1 | ENSG00000169118 | casein kinase 1 gamma 1 | 0,953 | Cytoplasm | kinase |
| CSRNP1 | ENSG00000144655 | cysteine and serine rich nuclear protein 1 | 3,326 | Nucleus | transcription regulator |
| CSRNP2 | ENSG00000110925 | cysteine and serine rich nuclear protein 2 | 0,734 | Nucleus | transcription regulator |
| CTAGE9 (includes others) | ENSG00000225932 | CTAGE family member 9 | 2,090 | Other | other |
| CTDSPL | ENSG00000144677 | CTD small phosphatase like | -0,724 | Nucleus | other |
| CTH | ENSG00000116761 | cystathionine gamma-lyase | 1,557 | Cytoplasm | enzyme |
| CTSH | ENSG00000103811 | cathepsin H | -0,982 | Cytoplasm | peptidase |
| CTSS | ENSG00000163131 | cathepsin S | 0,809 | Cytoplasm | peptidase |
| CTSV | ENSG00000136943 | cathepsin V | -0,824 | Cytoplasm | peptidase |
| CUTA | ENSG00000112514 | cutA divalent cation tolerance homolog | -0,715 | Cytoplasm | other |
| CX3CL1 | ENSG00000006210 | C-X3-C motif chemokine ligand 1 | 2,906 | Extracellular Space | cytokine |
| CXCL1 | ENSG00000163739 | C-X-C motif chemokine ligand 1 | 1,925 | Extracellular Space | cytokine |
| CXCL10 | ENSG00000169245 | C-X-C motif chemokine ligand 10 | 6,366 | Extracellular Space | cytokine |
| CXCL11 | ENSG00000169248 | C-X-C motif chemokine ligand 11 | 5,631 | Extracellular Space | cytokine |
| CXCL2 | ENSG00000081041 | C-X-C motif chemokine ligand 2 | 4,228 | Extracellular Space | cytokine |
| CXCL3 | ENSG00000163734 | C-X-C motif chemokine ligand 3 | 2,384 | Extracellular Space | cytokine |
| CXCL8 | ENSG00000169429 | C-X-C motif chemokine ligand 8 | 2,174 | Extracellular Space | cytokine |
| CXCL9 | ENSG00000138755 | C-X-C motif chemokine ligand 9 | 1,412 | Extracellular Space | cytokine |
| CXorf38 | ENSG00000185753 | chromosome X open reading frame 38 | 0,912 | Other | other |
| CYB5A | ENSG00000166347 | cytochrome b5 type A | -0,612 | Cytoplasm | enzyme |
| CYB5D2 | ENSG00000167740 | cytochrome b5 domain containing 2 | -0,830 | Extracellular Space | other |
| CYBA | ENSG00000051523 | cytochrome b-245 alpha chain | -0,603 | Cytoplasm | enzyme |
| CYBRD1 | ENSG00000071967 | cytochrome b reductase 1 | -0,721 | Cytoplasm | enzyme |
| CYLD | ENSG00000083799 | CYLD lysine 63 deubiquitinase | 2,920 | Nucleus | transcription regulator |
| CYP1A1 | ENSG00000140465 | cytochrome P450 family 1 subfamily A member 1 | 5,714 | Cytoplasm | enzyme |
| CYP1B1 | ENSG00000138061 | cytochrome P450 family 1 subfamily B member 1 | 1,576 | Cytoplasm | enzyme |
| CYP2B6 | ENSG00000197408 | cytochrome P450 family 2 subfamily B member 6 | 1,194 | Cytoplasm | enzyme |
| CYP2J2 | ENSG00000134716 | cytochrome P450 family 2 subfamily J member 2 | 1,416 | Cytoplasm | enzyme |
| CYP3A5 | ENSG00000106258 | cytochrome P450 family 3 subfamily A member 5 | 1,212 | Cytoplasm | enzyme |
| CYP4B1 | ENSG00000142973 | cytochrome P450 family 4 subfamily B member 1 | 0,950 | Cytoplasm | enzyme |
| CYS1 | ENSG00000205795 | cystin 1 | -1,477 | Cytoplasm | transcription regulator |
| CYTH1 | ENSG00000108669 | cytohesin 1 | 0,615 | Cytoplasm | other |
| DAB2IP | ENSG00000136848 | DAB2 interacting protein | 0,995 | Plasma Membrane | other |
| DAD1 | ENSG00000129562 | defender against cell death 1 | -0,683 | Cytoplasm | other |
| DAPP1 | ENSG00000070190 | dual adaptor of phosphotyrosine and 3-phosphoinositides 1 | 2,361 | Cytoplasm | other |
| DAXX | ENSG00000204209 | death domain associated protein | 1,459 | Nucleus | transcription regulator |
| DBI | ENSG00000155368 | diazepam binding inhibitor, acyl-CoA binding protein | -0,730 | Cytoplasm | other |
| DCAF5 | ENSG00000139990 | DDB1 and CUL4 associated factor 5 | 0,631 | Cytoplasm | other |
| DCHS1 | ENSG00000166341 | dachsous cadherin-related 1 | 1,145 | Plasma Membrane | other |
| DCLRE1C | ENSG00000152457 | DNA cross-link repair 1C | 1,523 | Nucleus | enzyme |
| DCP1A | ENSG00000272886 | decapping mRNA 1A | 1,094 | Nucleus | other |

| | | | | | |
|---|---|---|---|---|---|
| DCUN1D3 | ENSG00000188215 | defective in cullin neddylation 1 domain containing 3 | 1,667 | Cytoplasm | other |
| DDAH2 | ENSG00000226634 | dimethylarginine dimethylaminohydrolase 2 | -0,664 | Cytoplasm | enzyme |
| DDC | ENSG00000132437 | dopa decarboxylase | -0,772 | Cytoplasm | enzyme |
| DDIT3 | ENSG00000175197 | DNA damage inducible transcript 3 | 1,618 | Nucleus | transcription regulator |
| DDX3X | ENSG00000215301 | DEAD-box helicase 3 X-linked | 0,631 | Cytoplasm | enzyme |
| DDX47 | ENSG00000213782 | DEAD-box helicase 47 | 0,700 | Nucleus | enzyme |
| DDX58 | ENSG00000107201 | DExD/H-box helicase 58 | 2,928 | Cytoplasm | enzyme |
| DDX60 | ENSG00000137628 | DExD/H-box helicase 60 | 2,591 | Cytoplasm | enzyme |
| DDX60L | ENSG00000181381 | DExD/H-box 60 like | 2,855 | Other | other |
| DEDD2 | ENSG00000160570 | death effector domain containing 2 | 1,140 | Nucleus | other |
| DENND2A | ENSG00000146966 | DENN domain containing 2A | 0,793 | Cytoplasm | other |
| DENND3 | ENSG00000105339 | DENN domain containing 3 | 0,916 | Cytoplasm | other |
| DENND4A | ENSG00000174485 | DENN domain containing 4A | 1,023 | Nucleus | other |
| DENND4B | ENSG00000198837 | DENN domain containing 4B | 0,799 | Extracellular Space | other |
| DENND5A | ENSG00000184014 | DENN domain containing 5A | 0,956 | Cytoplasm | other |
| DEPP1 | ENSG00000165507 | DEPP1 autophagy regulator | 1,312 | Cytoplasm | other |
| DGKD | ENSG00000077044 | diacylglycerol kinase delta | 0,604 | Cytoplasm | kinase |
| DHCR7 | ENSG00000172893 | 7-dehydrocholesterol reductase | -0,643 | Cytoplasm | enzyme |
| DHFR | ENSG00000228716 | dihydrofolate reductase | -0,717 | Nucleus | enzyme |
| DHRS2 | ENSG00000100867 | dehydrogenase/reductase 2 | 2,948 | Nucleus | enzyme |
| DHX34 | ENSG00000134815 | DExH-box helicase 34 | 0,846 | Other | enzyme |
| DHX36 | ENSG00000174953 | DEAH-box helicase 36 | 1,105 | Cytoplasm | enzyme |
| DHX58 | ENSG00000108771 | DExH-box helicase 58 | 3,522 | Cytoplasm | enzyme |
| DICER1 | ENSG00000100697 | dicer 1, ribonuclease III | 1,161 | Cytoplasm | enzyme |
| DIO2 | ENSG00000211448 | iodothyronine deiodinase 2 | 1,020 | Cytoplasm | enzyme |
| DIO3 | ENSG00000197406 | iodothyronine deiodinase 3 | 1,056 | Plasma Membrane | enzyme |
| DIP2B | ENSG00000066084 | disco interacting protein 2 homolog B | 0,920 | Cytoplasm | other |
| DIP2C | ENSG00000151240 | disco interacting protein 2 homolog C | 0,742 | Other | other |
| DIRAS3 | ENSG00000162595 | DIRAS family GTPase 3 | 0,964 | Plasma Membrane | enzyme |
| DIXDC1 | ENSG00000150764 | DIX domain containing 1 | -0,787 | Cytoplasm | other |
| DKK3 | ENSG00000050165 | dickkopf WNT signaling pathway inhibitor 3 | -0,892 | Extracellular Space | cytokine |
| DLC1 | ENSG00000164741 | DLC1 Rho GTPase activating protein | 0,893 | Cytoplasm | other |
| DLEU1 | ENSG00000176124 | deleted in lymphocytic leukemia 1 | -1,057 | Other | other |
| DLGAP5 | ENSG00000126787 | DLG associated protein 5 | -0,749 | Nucleus | other |
| DLX2 | ENSG00000115844 | distal-less homeobox 2 | 0,664 | Nucleus | transcription regulator |
| DMAC1 | ENSG00000137038 | distal membrane arm assembly complex 1 | -0,845 | Cytoplasm | other |
| DMBX1 | ENSG00000197587 | diencephalon/mesencephalon homeobox 1 | -1,690 | Nucleus | transcription regulator |
| DMXL1 | ENSG00000172869 | Dmx like 1 | 0,799 | Extracellular Space | other |
| DNAAF3 | ENSG00000167646 | dynein axonemal assembly factor 3 | -0,602 | Other | other |
| DNAH1 | ENSG00000114841 | dynein axonemal heavy chain 1 | 0,624 | Extracellular Space | other |
| DNAH17 | ENSG00000187775 | dynein axonemal heavy chain 17 | 2,404 | Cytoplasm | other |
| DNAJA4 | ENSG00000140403 | DnaJ heat shock protein family (Hsp40) member A4 | 1,997 | Nucleus | other |
| DNAJB1 | ENSG00000132002 | DnaJ heat shock protein family (Hsp40) member B1 | 0,928 | Nucleus | transcription regulator |
| DNAJB4 | ENSG00000162616 | DnaJ heat shock protein family (Hsp40) member B4 | 3,108 | Nucleus | other |
| DNAJB5 | ENSG00000137094 | DnaJ heat shock protein family (Hsp40) member B5 | 0,989 | Cytoplasm | other |
| DNAJC22 | ENSG00000178401 | DnaJ heat shock protein family (Hsp40) member C22 | -1,273 | Other | other |
| DNAJC25 | ENSG00000059769 | DnaJ heat shock protein family (Hsp40) member C25 | 1,394 | Other | other |
| DNAJC27 | ENSG00000115137 | DnaJ heat shock protein family (Hsp40) member C27 | 0,822 | Cytoplasm | enzyme |
| DNAJC6 | ENSG00000116675 | DnaJ heat shock protein family (Hsp40) member C6 | 0,816 | Cytoplasm | other |
| DNMBP | ENSG00000107554 | dynamin binding protein | 1,100 | Cytoplasm | other |
| DNPEP | ENSG00000123992 | aspartyl aminopeptidase | 0,627 | Cytoplasm | peptidase |
| DOCK10 | ENSG00000135905 | dedicator of cytokinesis 10 | 1,444 | Cytoplasm | other |
| DOCK6 | ENSG00000130158 | dedicator of cytokinesis 6 | 0,737 | Cytoplasm | other |
| DOHH | ENSG00000129932 | deoxyhypusine hydroxylase | 0,605 | Cytoplasm | enzyme |
| DOK4 | ENSG00000125170 | docking protein 4 | -0,744 | Plasma Membrane | other |
| DOP1A | ENSG00000083097 | DOP1 leucine zipper like protein A | 0,721 | Cytoplasm | other |
| DPM3 | ENSG00000179085 | dolichyl-phosphate mannosyltransferase subunit 3, regulatory | -0,899 | Cytoplasm | enzyme |
| DPYSL2 | ENSG00000092964 | dihydropyrimidinase like 2 | -0,806 | Cytoplasm | enzyme |
| DRAM1 | ENSG00000136048 | DNA damage regulated autophagy modulator 1 | 1,107 | Cytoplasm | other |
| DRAM2 | ENSG00000156171 | DNA damage regulated autophagy modulator 2 | -0,623 | Cytoplasm | other |
| DRD1 | ENSG00000184845 | dopamine receptor D1 | 0,982 | Plasma Membrane | G-protein coupled receptor |
| DTD2 | ENSG00000129480 | D-aminoacyl-tRNA deacylase 2 | -0,627 | Other | other |
| DTX3L | ENSG00000163840 | deltex E3 ubiquitin ligase 3L | 1,851 | Cytoplasm | enzyme |
| DUOX1 | ENSG00000137857 | dual oxidase 1 | 2,494 | Plasma Membrane | enzyme |
| DUS3L | ENSG00000141994 | dihydrouridine synthase 3 like | 0,718 | Other | other |
| DUSP1 | ENSG00000120129 | dual specificity phosphatase 1 | 4,210 | Nucleus | phosphatase |
| DUSP10 | ENSG00000143507 | dual specificity phosphatase 10 | 2,159 | Nucleus | phosphatase |
| DUSP15 | ENSG00000149599 | dual specificity phosphatase 15 | 2,751 | Cytoplasm | phosphatase |
| DUSP16 | ENSG00000111266 | dual specificity phosphatase 16 | 1,691 | Nucleus | phosphatase |
| DUSP2 | ENSG00000158050 | dual specificity phosphatase 2 | 0,821 | Nucleus | phosphatase |
| DUSP4 | ENSG00000120875 | dual specificity phosphatase 4 | 0,680 | Nucleus | phosphatase |
| DUSP5 | ENSG00000138166 | dual specificity phosphatase 5 | 1,755 | Nucleus | phosphatase |
| DUSP8 | ENSG00000184545 | dual specificity phosphatase 8 | 3,160 | Nucleus | phosphatase |
| DYRK1A | ENSG00000157540 | dual specificity tyrosine phosphorylation regulated kinase 1A | 1,630 | Nucleus | kinase |
| E2F3 | ENSG00000112242 | E2F transcription factor 3 | 0,700 | Nucleus | transcription regulator |
| E2F6 | ENSG00000169016 | E2F transcription factor 6 | 0,871 | Nucleus | transcription regulator |
| E2F7 | ENSG00000165891 | E2F transcription factor 7 | 1,280 | Nucleus | transcription regulator |
| EAF1 | ENSG00000144597 | ELL associated factor 1 | 0,793 | Nucleus | transcription regulator |
| EBF4 | ENSG00000088881 | EBF family member 4 | 0,738 | Nucleus | transcription regulator |
| ECH1 | ENSG00000104823 | enoyl-CoA hydratase 1 | -0,800 | Cytoplasm | enzyme |
| EDC3 | ENSG00000179151 | enhancer of mRNA decapping 3 | 0,849 | Cytoplasm | enzyme |
| EDN1 | ENSG00000078401 | endothelin 1 | 0,793 | Extracellular Space | cytokine |
| EEF1A1 | ENSG00000156508 | eukaryotic translation elongation factor 1 alpha 1 | -0,677 | Cytoplasm | translation regulator |
| EFNA1 | ENSG00000169242 | ephrin A1 | 2,137 | Plasma Membrane | other |
| EFNB2 | ENSG00000125266 | ephrin B2 | 0,626 | Plasma Membrane | kinase |
| EGFL6 | ENSG00000198759 | EGF like domain multiple 6 | 1,087 | Extracellular Space | other |
| EGFL8 | ENSG00000242038 | EGF like domain multiple 8 | -1,099 | Extracellular Space | other |
| EGFR | ENSG00000146648 | epidermal growth factor receptor | 0,717 | Plasma Membrane | kinase |
| EGLN3 | ENSG00000129521 | egl-9 family hypoxia inducible factor 3 | 1,603 | Cytoplasm | enzyme |
| EGR1 | ENSG00000120738 | early growth response 1 | 2,469 | Nucleus | transcription regulator |
| EGR2 | ENSG00000122877 | early growth response 2 | 3,342 | Nucleus | transcription regulator |
| EGR3 | ENSG00000179388 | early growth response 3 | 1,381 | Nucleus | transcription regulator |
| EGR4 | ENSG00000135625 | early growth response 4 | 2,614 | Nucleus | transcription regulator |
| EHD4 | ENSG00000103966 | EH domain containing 4 | 1,059 | Plasma Membrane | enzyme |
| EIF2AK2 | ENSG00000055332 | eukaryotic translation initiation factor 2 alpha kinase 2 | 1,830 | Cytoplasm | kinase |
| EIF2AK3 | ENSG00000172071 | eukaryotic translation initiation factor 2 alpha kinase 3 | 1,550 | Cytoplasm | kinase |
| EIF4E3 | ENSG00000163412 | eukaryotic translation initiation factor 4E family member 3 | -0,674 | Cytoplasm | translation regulator |
| EIF4EBP3 | ENSG00000243056 | eukaryotic translation initiation factor 4E binding protein 3 | 0,742 | Cytoplasm | other |
| ELF1 | ENSG00000120690 | E74 like ETS transcription factor 1 | 0,781 | Nucleus | transcription regulator |

| | | | | | |
|---|---|---|---|---|---|
| ELF3 | ENSG00000163435 | E74 like ETS transcription factor 3 | 0,930 | Nucleus | transcription regulator |
| ELFN1 | ENSG00000225968 | extracellular leucine rich repeat and fibronectin type III domain containing 1 | 1,113 | Plasma Membrane | other |
| ELL | ENSG00000105656 | elongation factor for RNA polymerase II | 0,727 | Nucleus | transcription regulator |
| ELL2 | ENSG00000118985 | elongation factor for RNA polymerase II 2 | 1,385 | Nucleus | transcription regulator |
| EME2 | ENSG00000197774 | essential meiotic structure-specific endonuclease subunit 2 | 0,868 | Other | other |
| EML1 | ENSG00000066629 | EMAP like 1 | -0,642 | Cytoplasm | other |
| ENTPD2 | ENSG00000054179 | ectonucleoside triphosphate diphosphohydrolase 2 | -0,720 | Cytoplasm | enzyme |
| EOLA1/EOLA2 | ENSG00000197620 | endothelium and lymphocyte associated ASCH domain 1 | -0,959 | Other | other |
| EP300 | ENSG00000100393 | E1A binding protein p300 | 0,730 | Nucleus | transcription regulator |
| EPC1 | ENSG00000120616 | enhancer of polycomb homolog 1 | 1,760 | Nucleus | transcription regulator |
| EPC2 | ENSG00000135999 | enhancer of polycomb homolog 2 | 0,771 | Other | other |
| EPHA4 | ENSG00000116106 | EPH receptor A4 | 2,087 | Plasma Membrane | kinase |
| EPHX1 | ENSG00000143819 | epoxide hydrolase 1 | -0,780 | Cytoplasm | peptidase |
| EPS8 | ENSG00000151491 | epidermal growth factor receptor pathway substrate 8 | 0,761 | Plasma Membrane | peptidase |
| EPS8L3 | ENSG00000198758 | EPS8 like 3 | -1,164 | Extracellular Space | other |
| EPSTI1 | ENSG00000133106 | epithelial stromal interaction 1 | 3,602 | Other | other |
| ERAP1 | ENSG00000164307 | endoplasmic reticulum aminopeptidase 1 | 0,900 | Extracellular Space | peptidase |
| ERAP2 | ENSG00000164308 | endoplasmic reticulum aminopeptidase 2 | 0,741 | Cytoplasm | peptidase |
| ERBIN | ENSG00000112851 | erbb2 interacting protein | 0,616 | Cytoplasm | other |
| ERCC4 | ENSG00000175595 | ERCC excision repair 4, endonuclease catalytic subunit | 1,094 | Nucleus | enzyme |
| ERCC6 | ENSG00000225830 | ERCC excision repair 6, chromatin remodeling factor | 0,811 | Nucleus | transcription regulator |
| ERCC6L2 | ENSG00000182150 | ERCC excision repair 6 like 2 | 0,978 | Other | enzyme |
| EREG | ENSG00000124882 | epiregulin | 1,299 | Extracellular Space | growth factor |
| ERICH5 | ENSG00000177459 | glutamate rich 5 | -0,645 | Other | other |
| ERN1 | ENSG00000178607 | endoplasmic reticulum to nucleus signaling 1 | 1,267 | Cytoplasm | kinase |
| ERO1B | ENSG00000086619 | endoplasmic reticulum oxidoreductase 1 beta | 1,162 | Cytoplasm | enzyme |
| ERRFI1 | ENSG00000116285 | ERBB receptor feedback inhibitor 1 | 1,172 | Cytoplasm | other |
| ESCO1 | ENSG00000141446 | establishment of sister chromatid cohesion N-acetyltransferase 1 | 0,646 | Nucleus | enzyme |
| ETFB | ENSG00000105379 | electron transfer flavoprotein subunit beta | -0,703 | Cytoplasm | transporter |
| ETS2 | ENSG00000157557 | ETS proto-oncogene 2, transcription factor | 1,850 | Nucleus | transcription regulator |
| ETV3 | ENSG00000117036 | ETS variant transcription factor 3 | 1,265 | Nucleus | transcription regulator |
| ETV7 | ENSG00000010030 | ETS variant transcription factor 7 | 3,964 | Nucleus | transcription regulator |
| EXOC3L1 | ENSG00000179044 | exocyst complex component 3 like 1 | 0,691 | Cytoplasm | other |
| EXOC3L4 | ENSG00000205436 | exocyst complex component 3 like 4 | 1,921 | Other | other |
| EXOC8 | ENSG00000116903 | exocyst complex component 8 | 0,883 | Plasma Membrane | other |
| EXT1 | ENSG00000182197 | exostosin glycosyltransferase 1 | 0,990 | Cytoplasm | enzyme |
| EYA1 | ENSG00000104313 | EYA transcriptional coactivator and phosphatase 1 | 1,481 | Nucleus | phosphatase |
| FA2H | ENSG00000103089 | fatty acid 2-hydroxylase | 1,072 | Cytoplasm | enzyme |
| FAAP20 | ENSG00000162585 | FA core complex associated protein 20 | -0,918 | Nucleus | other |
| FADS2 | ENSG00000134824 | fatty acid desaturase 2 | -0,673 | Plasma Membrane | enzyme |
| FADS3 | ENSG00000221968 | fatty acid desaturase 3 | -0,628 | Plasma Membrane | enzyme |
| FAH | ENSG00000103876 | fumarylacetoacetate hydrolase | -0,618 | Cytoplasm | enzyme |
| FAM110B | ENSG00000169122 | family with sequence similarity 110 member B | 1,476 | Cytoplasm | other |
| FAM122C | ENSG00000156500 | family with sequence similarity 122C | 0,709 | Other | other |
| FAM126B | ENSG00000155744 | family with sequence similarity 126 member B | 0,968 | Other | other |
| FAM133B | ENSG00000234545 | family with sequence similarity 133 member B | 0,690 | Other | other |
| FAM13A | ENSG00000138640 | family with sequence similarity 13 member A | 1,168 | Cytoplasm | other |
| FAM151B | ENSG00000152380 | family with sequence similarity 151 member B | 1,804 | Other | other |
| FAM160A1 | ENSG00000164142 | family with sequence similarity 160 member A1 | 0,945 | Other | other |
| FAM160B1 | ENSG00000151553 | family with sequence similarity 160 member B1 | 0,667 | Other | other |
| FAM162A | ENSG00000114023 | family with sequence similarity 162 member A | -0,992 | Cytoplasm | other |
| FAM169A | ENSG00000198780 | family with sequence similarity 169 member A | 1,201 | Other | other |
| FAM193A | ENSG00000125386 | family with sequence similarity 193 member A | 1,290 | Other | other |
| FAM210B | ENSG00000124098 | family with sequence similarity 210 member B | -0,707 | Extracellular Space | other |
| FAM214A | ENSG00000047346 | family with sequence similarity 214 member A | 2,124 | Other | other |
| FAM219A | ENSG00000164970 | family with sequence similarity 219 member A | 0,724 | Other | other |
| FAM222A | ENSG00000139438 | family with sequence similarity 222 member A | 1,354 | Other | other |
| FAM3D | ENSG00000198643 | FAM3 metabolism regulating signaling molecule D | -1,447 | Extracellular Space | cytokine |
| FAM43A | ENSG00000185112 | family with sequence similarity 43 member A | 1,373 | Other | other |
| FAM53C | ENSG00000120709 | family with sequence similarity 53 member C | 1,112 | Other | other |
| FAM72C/FAM72D | ENSG00000215784 | family with sequence similarity 72 member D | -0,845 | Nucleus | other |
| FAM83B | ENSG00000168143 | family with sequence similarity 83 member B | 0,982 | Cytoplasm | other |
| FAM83G | ENSG00000188522 | family with sequence similarity 83 member G | 0,816 | Other | other |
| FAM89B | ENSG00000176973 | family with sequence similarity 89 member B | -0,882 | Cytoplasm | other |
| FAM98B | ENSG00000171262 | family with sequence similarity 98 member B | -0,611 | Nucleus | enzyme |
| FANCM | ENSG00000187790 | FA complementation group M | 0,640 | Nucleus | enzyme |
| FAP | ENSG00000078098 | fibroblast activation protein alpha | 1,726 | Cytoplasm | peptidase |
| FARP1 | ENSG00000152767 | FERM, ARH/RhoGEF and pleckstrin domain protein 1 | -0,608 | Plasma Membrane | other |
| FAS | ENSG00000026103 | Fas cell surface death receptor | 1,886 | Plasma Membrane | transmembrane receptor |
| FAXDC2 | ENSG00000170271 | fatty acid hydroxylase domain containing 2 | 1,044 | Cytoplasm | other |
| FBN2 | ENSG00000138829 | fibrillin 2 | 0,816 | Extracellular Space | other |
| FBP1 | ENSG00000165140 | fructose-bisphosphatase 1 | -1,116 | Cytoplasm | phosphatase |
| FBRS | ENSG00000156860 | fibrosin | 0,644 | Extracellular Space | cytokine |
| FBXL12 | ENSG00000127452 | F-box and leucine rich repeat protein 12 | 0,748 | Cytoplasm | other |
| FBXL21P | ENSG00000164616 | F-box and leucine rich repeat protein 21, pseudogene | -0,727 | Cytoplasm | enzyme |
| FBXO11 | ENSG00000138081 | F-box protein 11 | 0,600 | Cytoplasm | enzyme |
| FBXO15 | ENSG00000141665 | F-box protein 15 | -0,683 | Cytoplasm | transcription regulator |
| FBXO17 | ENSG00000269190 | F-box protein 17 | -0,608 | Cytoplasm | enzyme |
| FBXO38 | ENSG00000145868 | F-box protein 38 | 0,783 | Other | other |
| FBXO6 | ENSG00000116663 | F-box protein 6 | 1,680 | Cytoplasm | other |
| FBXW10 | ENSG00000171931 | F-box and WD repeat domain containing 10 | 1,194 | Cytoplasm | other |
| FBXW7 | ENSG00000109670 | F-box and WD repeat domain containing 7 | 1,215 | Nucleus | enzyme |
| FCGRT | ENSG00000104870 | Fc fragment of IgG receptor and transporter | -0,664 | Plasma Membrane | transmembrane receptor |
| FDFT1 | ENSG00000079459 | farnesyl-diphosphate farnesyltransferase 1 | -0,620 | Cytoplasm | enzyme |
| FDPS | ENSG00000160752 | farnesyl diphosphate synthase | -0,709 | Cytoplasm | enzyme |
| FEM1B | ENSG00000169018 | fem-1 homolog B | 0,659 | Nucleus | transcription regulator |
| FEM1C | ENSG00000145780 | fem-1 homolog C | 1,040 | Nucleus | transcription regulator |
| FERMT2 | ENSG00000073712 | fermitin family member 2 | 0,659 | Cytoplasm | other |
| FERMT3 | ENSG00000149781 | fermitin family member 3 | 0,957 | Cytoplasm | enzyme |
| FEZ1 | ENSG00000149557 | fasciculation and elongation protein zeta 1 | 1,631 | Cytoplasm | other |
| FGD6 | ENSG00000180263 | FYVE, RhoGEF and PH domain containing 6 | 1,347 | Cytoplasm | other |
| FGF18 | ENSG00000156427 | fibroblast growth factor 18 | 1,766 | Extracellular Space | growth factor |
| FGF19 | ENSG00000162344 | fibroblast growth factor 19 | 1,384 | Extracellular Space | growth factor |
| FGF2 | ENSG00000138685 | fibroblast growth factor 2 | 0,710 | Extracellular Space | growth factor |
| FGFBP1 | ENSG00000137440 | fibroblast growth factor binding protein 1 | -0,695 | Extracellular Space | other |
| FGFR1 | ENSG00000077782 | fibroblast growth factor receptor 1 | 0,898 | Plasma Membrane | kinase |
| FGFR1OP2 | ENSG00000111790 | FGFR1 oncogene partner 2 | 0,849 | Cytoplasm | other |
| FGFR3 | ENSG00000068078 | fibroblast growth factor receptor 3 | -0,950 | Plasma Membrane | kinase |
| FILIP1L | ENSG00000168386 | filamin A interacting protein 1 like | 2,190 | Nucleus | other |

| | | | | | |
|---|---|---|---|---|---|
| FKRP | ENSG00000181027 | fukutin related protein | 0,917 | Cytoplasm | other |
| FLCN | ENSG00000154803 | folliculin | 1,866 | Cytoplasm | other |
| FLJ22447 | ENSG00000232774 | uncharacterized LOC400221 | 0,839 | Other | other |
| FLRT3 | ENSG00000125848 | fibronectin leucine rich transmembrane protein 3 | 2,031 | Plasma Membrane | other |
| FLT3LG | ENSG00000090554 | fms related receptor tyrosine kinase 3 ligand | 1,617 | Extracellular Space | cytokine |
| FLYWCH2 | ENSG00000162076 | FLYWCH family member 2 | -0,905 | Other | other |
| FMR1 | ENSG00000102081 | FMRP translational regulator 1 | 1,363 | Cytoplasm | translation regulator |
| FN3K | ENSG00000167363 | fructosamine 3 kinase | -0,620 | Cytoplasm | kinase |
| FNBP4 | ENSG00000109920 | formin binding protein 4 | 0,702 | Nucleus | other |
| FNDC3A | ENSG00000102531 | fibronectin type III domain containing 3A | 1,278 | Cytoplasm | other |
| FNIP1 | ENSG00000217128 | folliculin interacting protein 1 | 1,567 | Cytoplasm | other |
| FNIP2 | ENSG00000052795 | folliculin interacting protein 2 | 0,736 | Cytoplasm | other |
| FOS | ENSG00000170345 | Fos proto-oncogene, AP-1 transcription factor subunit | 3,090 | Nucleus | transcription regulator |
| FOSB | ENSG00000125740 | FosB proto-oncogene, AP-1 transcription factor subunit | 3,317 | Nucleus | transcription regulator |
| FOSL1 | ENSG00000175592 | FOS like 1, AP-1 transcription factor subunit | 1,170 | Nucleus | transcription regulator |
| FOSL2 | ENSG00000075426 | FOS like 2, AP-1 transcription factor subunit | 1,786 | Nucleus | transcription regulator |
| FOXA2 | ENSG00000125798 | forkhead box A2 | 0,765 | Nucleus | transcription regulator |
| FOXA3 | ENSG00000170608 | forkhead box A3 | 0,915 | Nucleus | transcription regulator |
| FOXJ3 | ENSG00000198815 | forkhead box J3 | 0,637 | Nucleus | transcription regulator |
| FOXN2 | ENSG00000170802 | forkhead box N2 | 0,978 | Nucleus | transcription regulator |
| FOXO1 | ENSG00000150907 | forkhead box O1 | 1,619 | Nucleus | transcription regulator |
| FOXO3 | ENSG00000118689 | forkhead box O3 | 1,173 | Nucleus | transcription regulator |
| FOXO4 | ENSG00000184481 | forkhead box O4 | -0,654 | Nucleus | transcription regulator |
| FOXP1 | ENSG00000114861 | forkhead box P1 | 0,888 | Nucleus | transcription regulator |
| FOXP2 | ENSG00000128573 | forkhead box P2 | 0,913 | Nucleus | transcription regulator |
| FRAS1 | ENSG00000138759 | Fraser extracellular matrix complex subunit 1 | -0,655 | Extracellular Space | other |
| FRK | ENSG00000111816 | fyn related Src family tyrosine kinase | 1,113 | Nucleus | kinase |
| FRMD4B | ENSG00000114541 | FERM domain containing 4B | 0,857 | Cytoplasm | other |
| FRMD5 | ENSG00000171877 | FERM domain containing 5 | 0,891 | Plasma Membrane | other |
| FRS2 | ENSG00000166225 | fibroblast growth factor receptor substrate 2 | 1,664 | Plasma Membrane | other |
| FRYL | ENSG00000075539 | FRY like transcription coactivator | 1,229 | Other | other |
| FSCN1 | ENSG00000075618 | fascin actin-bundling protein 1 | 0,919 | Cytoplasm | other |
| FSD1L | ENSG00000106701 | fibronectin type III and SPRY domain containing 1 like | 0,867 | Other | other |
| FST | ENSG00000134363 | follistatin | 2,117 | Extracellular Space | other |
| FSTL3 | ENSG00000070404 | follistatin like 3 | 2,007 | Extracellular Space | other |
| FUT4 | ENSG00000196371 | fucosyltransferase 4 | 0,995 | Cytoplasm | enzyme |
| FUT6 | ENSG00000156413 | fucosyltransferase 6 | 0,776 | Cytoplasm | enzyme |
| FXYD2 | ENSG00000137731 | FXYD domain containing ion transport regulator 2 | -1,402 | Plasma Membrane | ion channel |
| FYB2 | ENSG00000187889 | FYN binding protein 2 | 1,457 | Plasma Membrane | other |
| FZD4 | ENSG00000174804 | frizzled class receptor 4 | 1,815 | Plasma Membrane | G-protein coupled receptor |
| FZD5 | ENSG00000163251 | frizzled class receptor 5 | 0,838 | Plasma Membrane | G-protein coupled receptor |
| G0S2 | ENSG00000123689 | G0/G1 switch 2 | 1,728 | Cytoplasm | other |
| GAB1 | ENSG00000109458 | GRB2 associated binding protein 1 | 0,786 | Cytoplasm | other |
| GABRE | ENSG00000102287 | gamma-aminobutyric acid type A receptor subunit epsilon | 0,679 | Plasma Membrane | ion channel |
| GABRP | ENSG00000094755 | gamma-aminobutyric acid type A receptor subunit pi | -0,737 | Plasma Membrane | ion channel |
| GADD45A | ENSG00000116717 | growth arrest and DNA damage inducible alpha | 1,084 | Nucleus | other |
| GADD45B | ENSG00000099860 | growth arrest and DNA damage inducible beta | 1,443 | Cytoplasm | other |
| GAMT | ENSG00000130005 | guanidinoacetate N-methyltransferase | -0,628 | Cytoplasm | enzyme |
| GAREM1 | ENSG00000141441 | GRB2 associated regulator of MAPK1 subtype 1 | 0,782 | Nucleus | other |
| GARRE1 | ENSG00000166398 | granule associated Rac and RHOG effector 1 | 0,966 | Extracellular Space | other |
| GAS2L3 | ENSG00000139354 | growth arrest specific 2 like 3 | 0,707 | Other | other |
| GAS6 | ENSG00000183087 | growth arrest specific 6 | 1,058 | Extracellular Space | growth factor |
| GATA3 | ENSG00000107485 | GATA binding protein 3 | 1,485 | Nucleus | transcription regulator |
| GATA4 | ENSG00000136574 | GATA binding protein 4 | 0,908 | Nucleus | transcription regulator |
| GATA6 | ENSG00000141448 | GATA binding protein 6 | 1,167 | Nucleus | transcription regulator |
| GATAD2B | ENSG00000261992 | GATA zinc finger domain containing 2B | 0,771 | Nucleus | transcription regulator |
| GATM | ENSG00000171766 | glycine amidinotransferase | -0,698 | Cytoplasm | enzyme |
| GBP1 | ENSG00000117228 | guanylate binding protein 1 | 3,366 | Cytoplasm | enzyme |
| GBP1P1 | ENSG00000225492 | guanylate binding protein 1 pseudogene 1 | 2,417 | Other | other |
| GBP2 | ENSG00000162645 | guanylate binding protein 2 | 1,125 | Cytoplasm | enzyme |
| GBP3 | ENSG00000117226 | guanylate binding protein 3 | 1,557 | Cytoplasm | enzyme |
| GBP4 | ENSG00000162654 | guanylate binding protein 4 | 3,727 | Cytoplasm | enzyme |
| GBP5 | ENSG00000154451 | guanylate binding protein 5 | 3,538 | Plasma Membrane | enzyme |
| GC | ENSG00000145321 | GC vitamin D binding protein | 0,714 | Extracellular Space | transporter |
| GCC1 | ENSG00000179562 | GRIP and coiled-coil domain containing 1 | 0,695 | Cytoplasm | other |
| GCH1 | ENSG00000131979 | GTP cyclohydrolase 1 | 1,224 | Cytoplasm | enzyme |
| GCHFR | ENSG00000137880 | GTP cyclohydrolase I feedback regulator | -0,842 | Cytoplasm | other |
| GCNT3 | ENSG00000140297 | glucosaminyl (N-acetyl) transferase 3, mucin type | 1,891 | Plasma Membrane | enzyme |
| GCNT4 | ENSG00000176928 | glucosaminyl (N-acetyl) transferase 4 | 0,955 | Cytoplasm | enzyme |
| GCSHP5 | ENSG00000224837 | glycine cleavage system protein H pseudogene 5 | -0,681 | Other | other |
| GDF15 | ENSG00000130513 | growth differentiation factor 15 | 1,856 | Extracellular Space | growth factor |
| GEM | ENSG00000164949 | GTP binding protein overexpressed in skeletal muscle | 2,340 | Plasma Membrane | enzyme |
| GET1 | ENSG00000182093 | guided entry of tail-anchored proteins factor 1 | -0,748 | Extracellular Space | other |
| GGA3 | ENSG00000125447 | golgi associated, gamma adaptin ear containing, ARF binding protein 3 | 0,925 | Cytoplasm | transporter |
| GIGYF2 | ENSG00000204120 | GRB10 interacting GYF protein 2 | 0,871 | Cytoplasm | other |
| GIMAP2 | ENSG00000106560 | GTPase, IMAP family member 2 | 1,373 | Cytoplasm | other |
| GJB1 | ENSG00000169562 | gap junction protein beta 1 | -1,147 | Plasma Membrane | transporter |
| GJC1 | ENSG00000182963 | gap junction protein gamma 1 | 0,692 | Plasma Membrane | ion channel |
| GLCCI1 | ENSG00000106415 | glucocorticoid induced 1 | 1,343 | Cytoplasm | other |
| GLOD4 | ENSG00000167699 | glyoxalase domain containing 4 | -0,659 | Cytoplasm | enzyme |
| GLS | ENSG00000115419 | glutaminase | 0,677 | Cytoplasm | enzyme |
| GLT8D1 | ENSG00000016864 | glycosyltransferase 8 domain containing 1 | -0,647 | Cytoplasm | enzyme |
| GM2A | ENSG00000196743 | GM2 ganglioside activator | -0,699 | Cytoplasm | enzyme |
| GMEB2 | ENSG00000101216 | glucocorticoid modulatory element binding protein 2 | 0,953 | Nucleus | transcription regulator |
| GMPR | ENSG00000137198 | guanosine monophosphate reductase | 2,618 | Cytoplasm | enzyme |
| GNA13 | ENSG00000120063 | G protein subunit alpha 13 | 0,885 | Plasma Membrane | enzyme |
| GNB4 | ENSG00000114450 | G protein subunit beta 4 | 1,146 | Plasma Membrane | enzyme |
| GNG12 | ENSG00000172380 | G protein subunit gamma 12 | -0,621 | Plasma Membrane | enzyme |
| GOLGA4 | ENSG00000144674 | golgin A4 | 0,626 | Cytoplasm | other |
| GOLGA6L9 (includes others) | ENSG00000184206 | golgin A6 family like 9 | 1,545 | Other | other |
| GON4L | ENSG00000116580 | gon-4 like | 0,800 | Cytoplasm | transcription regulator |
| GORAB | ENSG00000120370 | golgin, RAB6 interacting | 1,113 | Cytoplasm | other |
| GPAT4 | ENSG00000158669 | glycerol-3-phosphate acyltransferase 4 | 0,648 | Cytoplasm | enzyme |
| GPATCH2 | ENSG00000092978 | G-patch domain containing 2 | 1,044 | Nucleus | other |
| GPATCH2L | ENSG00000089916 | G-patch domain containing 2 like | 1,017 | Other | other |
| GPBP1 | ENSG00000062194 | GC-rich promoter binding protein 1 | 1,316 | Nucleus | transcription regulator |
| GPBP1L1 | ENSG00000159592 | GC-rich promoter binding protein 1 like 1 | 0,736 | Other | other |
| GPC3 | ENSG00000147257 | glypican 3 | -1,113 | Plasma Membrane | other |
| GPCPD1 | ENSG00000125772 | glycerophosphocholine phosphodiesterase 1 | 2,151 | Cytoplasm | enzyme |

| | | | | | |
|---|---|---|---|---|---|
| GPHN | ENSG00000171723 | gephyrin | 0,849 | Plasma Membrane | enzyme |
| GPR176 | ENSG00000166073 | G protein-coupled receptor 176 | 0,790 | Plasma Membrane | G-protein coupled receptor |
| GPR37L1 | ENSG00000170075 | G protein-coupled receptor 37 like 1 | 0,699 | Plasma Membrane | G-protein coupled receptor |
| GPR68 | ENSG00000119714 | G protein-coupled receptor 68 | 0,679 | Plasma Membrane | G-protein coupled receptor |
| GPR87 | ENSG00000138271 | G protein-coupled receptor 87 | 1,161 | Plasma Membrane | G-protein coupled receptor |
| GPSM2 | ENSG00000121957 | G protein signaling modulator 2 | -0,677 | Nucleus | other |
| GPX8 | ENSG00000164294 | glutathione peroxidase 8 (putative) | -0,792 | Cytoplasm | enzyme |
| GRAMD2B | ENSG00000155324 | GRAM domain containing 2B | 0,650 | Extracellular Space | other |
| GREB1L | ENSG00000141449 | GREB1 like retinoic acid receptor coactivator | 0,631 | Extracellular Space | other |
| GRIN2A | ENSG00000183454 | glutamate ionotropic receptor NMDA type subunit 2A | 0,863 | Plasma Membrane | ion channel |
| GRINA | ENSG00000178719 | glutamate ionotropic receptor NMDA type subunit associated protein 1 | 0,712 | Other | ion channel |
| GSDMB | ENSG00000073605 | gasdermin B | 1,145 | Cytoplasm | other |
| GTF2IRD2/GTF2IRD2B | ENSG00000196275 | GTF2I repeat domain containing 2 | 1,084 | Nucleus | other |
| GTF3C3 | ENSG00000119041 | general transcription factor IIIC subunit 3 | 0,652 | Nucleus | transcription regulator |
| GTPBP1 | ENSG00000100226 | GTP binding protein 1 | 1,205 | Cytoplasm | enzyme |
| GTPBP2 | ENSG00000172432 | GTP binding protein 2 | 0,819 | Extracellular Space | enzyme |
| GULP1 | ENSG00000144366 | GULP PTB domain containing engulfment adaptor 1 | 0,650 | Cytoplasm | other |
| GYG2 | ENSG00000056998 | glycogenin 2 | -1,155 | Cytoplasm | enzyme |
| H1-0 | ENSG00000189060 | H1.0 linker histone | 1,038 | Nucleus | other |
| H1-3 | ENSG00000124575 | H1.3 linker histone, cluster member | 1,644 | Nucleus | other |
| H1-4 | ENSG00000168298 | H1.4 linker histone, cluster member | 1,835 | Nucleus | other |
| H2AC11 | ENSG00000196787 | H2A clustered histone 11 | 0,993 | Nucleus | other |
| H2AC6 | ENSG00000180573 | H2A clustered histone 6 | 1,350 | Nucleus | other |
| H2BC12 | ENSG00000197903 | H2B clustered histone 12 | 0,725 | Nucleus | other |
| H2BC15 | ENSG00000233822 | H2B clustered histone 15 | 1,972 | Nucleus | other |
| H2BC18 | ENSG00000203814 | H2B clustered histone 18 | 1,521 | Nucleus | other |
| H2BC21 | ENSG00000184678 | H2B clustered histone 21 | 1,325 | Nucleus | other |
| H2BC4 | ENSG00000180596 | H2B clustered histone 4 | 2,374 | Nucleus | other |
| H2BC5 | ENSG00000158373 | H2B clustered histone 5 | 1,389 | Nucleus | other |
| H2BU1 | ENSG00000196890 | H2B.U histone 1 | 2,676 | Nucleus | other |
| H3-3A/H3-3B | ENSG00000163041 | H3.3 histone A | -0,602 | Nucleus | other |
| H3C14 | ENSG00000203811 | H3 clustered histone 14 | 1,383 | Nucleus | other |
| H3C15 | ENSG00000203852 | H3 clustered histone 15 | 1,383 | Nucleus | other |
| H3C4 | ENSG00000197409 | H3 clustered histone 4 | 3,064 | Nucleus | other |
| H4-16 | ENSG00000197837 | H4 histone 16 | 1,566 | Nucleus | other |
| H4C8 | ENSG00000158406 | H4 clustered histone 8 | 3,007 | Nucleus | other |
| HAND1 | ENSG00000113196 | heart and neural crest derivatives expressed 1 | 1,933 | Nucleus | transcription regulator |
| HAPLN3 | ENSG00000140511 | hyaluronan and proteoglycan link protein 3 | 0,850 | Extracellular Space | other |
| HAS2 | ENSG00000170961 | hyaluronan synthase 2 | 1,156 | Plasma Membrane | enzyme |
| HAVCR1 | ENSG00000113249 | hepatitis A virus cellular receptor 1 | -0,753 | Plasma Membrane | other |
| HBEGF | ENSG00000113070 | heparin binding EGF like growth factor | 1,461 | Extracellular Space | growth factor |
| HCFC1R1 | ENSG00000103145 | host cell factor C1 regulator 1 | -0,644 | Nucleus | other |
| HCFC2 | ENSG00000111727 | host cell factor C2 | 0,758 | Nucleus | transcription regulator |
| HCG27 | ENSG00000206344 | HLA complex group 27 | 1,723 | Other | other |
| HCG4B | ENSG00000231941 | HLA complex group 4B | 3,024 | Other | other |
| HDAC9 | ENSG00000048052 | histone deacetylase 9 | 1,594 | Nucleus | transcription regulator |
| HDX | ENSG00000165259 | highly divergent homeobox | 1,311 | Other | other |
| HELB | ENSG00000127311 | DNA helicase B | 1,513 | Nucleus | enzyme |
| HELQ | ENSG00000163312 | helicase, POLQ like | 0,726 | Nucleus | enzyme |
| HELZ | ENSG00000198265 | helicase with zinc finger | 0,740 | Nucleus | enzyme |
| HELZ2 | ENSG00000130589 | helicase with zinc finger 2 | 3,224 | Nucleus | transcription regulator |
| HERC5 | ENSG00000138646 | HECT and RLD domain containing E3 ubiquitin protein ligase 5 | 4,037 | Cytoplasm | enzyme |
| HERC6 | ENSG00000138642 | HECT and RLD domain containing E3 ubiquitin protein ligase family member 6 | 2,016 | Cytoplasm | enzyme |
| HERPUD1 | ENSG00000051108 | homocysteine inducible ER protein with ubiquitin like domain 1 | 0,785 | Cytoplasm | other |
| HES1 | ENSG00000114315 | hes family bHLH transcription factor 1 | 1,066 | Nucleus | transcription regulator |
| HES4 | ENSG00000188290 | hes family bHLH transcription factor 4 | 1,311 | Other | other |
| HIC2 | ENSG00000169635 | HIC ZBTB transcriptional repressor 2 | 0,619 | Nucleus | transcription regulator |
| HIP1R | ENSG00000130787 | huntingtin interacting protein 1 related | 0,852 | Cytoplasm | other |
| HIPK3 | ENSG00000110422 | homeodomain interacting protein kinase 3 | 0,841 | Nucleus | kinase |
| HIRA | ENSG00000100084 | histone cell cycle regulator | 0,749 | Nucleus | transcription regulator |
| HIVEP1 | ENSG00000095951 | HIVEP zinc finger 1 | 1,429 | Nucleus | transcription regulator |
| HIVEP2 | ENSG00000010818 | HIVEP zinc finger 2 | 2,268 | Nucleus | transcription regulator |
| HKDC1 | ENSG00000156510 | hexokinase domain containing 1 | 0,954 | Cytoplasm | kinase |
| HLA-A | ENSG00000206505 | major histocompatibility complex, class I, A | 1,071 | Plasma Membrane | other |
| HLA-B | ENSG00000223532 | major histocompatibility complex, class I, B | 2,280 | Plasma Membrane | transmembrane receptor |
| HLA-C | ENSG00000206435 | major histocompatibility complex, class I, C | 2,316 | Plasma Membrane | other |
| HLA-DOB | ENSG00000243612 | major histocompatibility complex, class II, DO beta | 1,730 | Plasma Membrane | transmembrane receptor |
| HLA-DPB1 | ENSG00000215048 | major histocompatibility complex, class II, DP beta 1 | 0,895 | Plasma Membrane | transmembrane receptor |
| HLA-DQA1 | ENSG00000196735 | major histocompatibility complex, class II, DQ alpha 1 | 0,971 | Plasma Membrane | transmembrane receptor |
| HLA-DRB4 | ENSG00000231021 | major histocompatibility complex, class II, DR beta 4 | -1,189 | Plasma Membrane | transmembrane receptor |
| HLA-E | ENSG00000233904 | major histocompatibility complex, class I, E | 1,197 | Plasma Membrane | transmembrane receptor |
| HLA-F | ENSG00000229698 | major histocompatibility complex, class I, F | 2,873 | Plasma Membrane | transmembrane receptor |
| HLA-H | ENSG00000206341 | major histocompatibility complex, class I, H (pseudogene) | 2,882 | Plasma Membrane | other |
| HLA-J | ENSG00000243336 | major histocompatibility complex, class I, J (pseudogene) | 2,339 | Other | other |
| HLA-K | ENSG00000232708 | major histocompatibility complex, class I, K (pseudogene) | 3,893 | Plasma Membrane | other |
| HLA-V | ENSG00000206444 | major histocompatibility complex, class I, V (pseudogene) | 2,290 | Other | other |
| HMGB1P3 | ENSG00000250011 | high mobility group box 1 pseudogene 3 | 2,819 | Other | other |
| HMGB2P1 | ENSG00000267736 | high mobility group box 2 pseudogene 1 | 2,101 | Other | other |
| HMGB3 | ENSG00000029993 | high mobility group box 3 | -0,853 | Nucleus | other |
| HMGN3 | ENSG00000118418 | high mobility group nucleosomal binding domain 3 | -0,679 | Nucleus | other |
| HMGXB3 | ENSG00000113716 | HMG-box containing 3 | 0,958 | Nucleus | transcription regulator |
| HMMR | ENSG00000072571 | hyaluronan mediated motility receptor | -0,753 | Plasma Membrane | transmembrane receptor |
| HMOX1 | ENSG00000100292 | heme oxygenase 1 | 1,032 | Cytoplasm | enzyme |
| HNF4A | ENSG00000101076 | hepatocyte nuclear factor 4 alpha | 0,669 | Nucleus | transcription regulator |
| HNMT | ENSG00000150540 | histamine N-methyltransferase | -0,671 | Cytoplasm | enzyme |
| HNRNPA1P7 | ENSG00000215492 | heterogeneous nuclear ribonucleoprotein A1 pseudogene 7 | -0,830 | Other | other |
| HOMER2 | ENSG00000103942 | homer scaffold protein 2 | -0,745 | Plasma Membrane | other |
| HOXB5 | ENSG00000120075 | homeobox B5 | -0,660 | Nucleus | transcription regulator |
| HOXB8 | ENSG00000120068 | homeobox B8 | -0,610 | Nucleus | transcription regulator |
| HPS5 | ENSG00000110756 | HPS5 biogenesis of lysosomal organelles complex 2 subunit 2 | 1,072 | Cytoplasm | other |
| HR | ENSG00000168453 | HR lysine demethylase and nuclear receptor corepressor | -0,637 | Nucleus | transcription regulator |
| HRH1 | ENSG00000196639 | histamine receptor H1 | 1,621 | Plasma Membrane | G-protein coupled receptor |
| HROB | ENSG00000125319 | homologous recombination factor with OB-fold | 0,985 | Other | other |
| HS2ST1 | ENSG00000153936 | heparan sulfate 2-O-sulfotransferase 1 | -0,749 | Cytoplasm | enzyme |
| HSCB | ENSG00000100209 | HscB mitochondrial iron-sulfur cluster cochaperone | 0,725 | Cytoplasm | other |
| HSD17B14 | ENSG00000087076 | hydroxysteroid 17-beta dehydrogenase 14 | 1,551 | Cytoplasm | enzyme |
| HSD17B2 | ENSG00000086696 | hydroxysteroid 17-beta dehydrogenase 2 | -0,902 | Cytoplasm | enzyme |
| HSD17B7P2 | ENSG00000099251 | hydroxysteroid 17-beta dehydrogenase 7 pseudogene 2 | 1,800 | Other | other |
| HSH2D | ENSG00000196684 | hematopoietic SH2 domain containing | 2,448 | Cytoplasm | other |

| | | | | | |
|---|---|---|---|---|---|
| HSPA1A/HSPA1B | ENSG00000224501 | heat shock protein family A (Hsp70) member 1A | 1,954 | Cytoplasm | enzyme |
| HSPA1L | ENSG00000236251 | heat shock protein family A (Hsp70) member 1 like | 0,803 | Cytoplasm | other |
| HSPA4L | ENSG00000164070 | heat shock protein family A (Hsp70) member 4 like | 1,467 | Cytoplasm | other |
| HSPA6 | ENSG00000173110 | heat shock protein family A (Hsp70) member 6 | 5,690 | Nucleus | enzyme |
| HSPA7 | ENSG00000225217 | heat shock protein family A (Hsp70) member 7 (pseudogene) | 1,223 | Cytoplasm | other |
| HSPB8 | ENSG00000152137 | heat shock protein family B (small) member 8 | 2,970 | Cytoplasm | kinase |
| HSPBAP1 | ENSG00000169087 | HSPB1 associated protein 1 | 0,691 | Other | other |
| HSPH1 | ENSG00000120694 | heat shock protein family H (Hsp110) member 1 | 1,079 | Cytoplasm | other |
| HYAL3 | ENSG00000186792 | hyaluronidase 3 | -0,679 | Cytoplasm | enzyme |
| ICAM1 | ENSG00000090339 | intercellular adhesion molecule 1 | 2,274 | Plasma Membrane | transmembrane receptor |
| ICAM5 | ENSG00000105376 | intercellular adhesion molecule 5 | 1,099 | Plasma Membrane | other |
| ICOSLG/LOC102723996 | ENSG00000160223 | inducible T cell costimulator ligand | 1,710 | Plasma Membrane | other |
| ID3 | ENSG00000117318 | inhibitor of DNA binding 3, HLH protein | 0,770 | Nucleus | transcription regulator |
| IDH1 | ENSG00000138413 | isocitrate dehydrogenase (NADP(+)) 1 | -0,699 | Cytoplasm | enzyme |
| IDH2 | ENSG00000182054 | isocitrate dehydrogenase (NADP(+)) 2 | -0,671 | Cytoplasm | enzyme |
| IDO1 | ENSG00000131203 | indoleamine 2,3-dioxygenase 1 | 3,997 | Cytoplasm | enzyme |
| IER2 | ENSG00000160888 | immediate early response 2 | 1,235 | Cytoplasm | transcription regulator |
| IER3 | ENSG00000237155 | immediate early response 3 | 1,342 | Cytoplasm | other |
| IER5 | ENSG00000162783 | immediate early response 5 | 1,669 | Nucleus | other |
| IFI16 | ENSG00000163565 | interferon gamma inducible protein 16 | 2,894 | Nucleus | transcription regulator |
| IFI27 | ENSG00000165949 | interferon alpha inducible protein 27 | 2,880 | Cytoplasm | other |
| IFI35 | ENSG00000068079 | interferon induced protein 35 | 2,567 | Nucleus | other |
| IFI44 | ENSG00000137965 | interferon induced protein 44 | 2,409 | Cytoplasm | other |
| IFI44L | ENSG00000137959 | interferon induced protein 44 like | 2,171 | Nucleus | other |
| IFI6 | ENSG00000126709 | interferon alpha inducible protein 6 | 1,646 | Cytoplasm | other |
| IFIH1 | ENSG00000115267 | interferon induced with helicase C domain 1 | 3,364 | Nucleus | enzyme |
| IFIT1 | ENSG00000185745 | interferon induced protein with tetratricopeptide repeats 1 | 3,692 | Cytoplasm | other |
| IFIT2 | ENSG00000119922 | interferon induced protein with tetratricopeptide repeats 2 | 5,139 | Cytoplasm | other |
| IFIT3 | ENSG00000119917 | interferon induced protein with tetratricopeptide repeats 3 | 4,523 | Cytoplasm | other |
| IFIT5 | ENSG00000152778 | interferon induced protein with tetratricopeptide repeats 5 | 2,868 | Plasma Membrane | other |
| IFITM1 | ENSG00000185885 | interferon induced transmembrane protein 1 | 3,426 | Plasma Membrane | transmembrane receptor |
| IFITM2 | ENSG00000185201 | interferon induced transmembrane protein 2 | 2,265 | Cytoplasm | other |
| IFITM3 | ENSG00000142089 | interferon induced transmembrane protein 3 | 2,229 | Plasma Membrane | other |
| IFNAR2 | ENSG00000159110 | interferon alpha and beta receptor subunit 2 | 0,733 | Plasma Membrane | transmembrane receptor |
| IFNB1 | ENSG00000171855 | interferon beta 1 | 8,646 | Extracellular Space | cytokine |
| IFNGR2 | ENSG00000262795 | interferon gamma receptor 2 | 0,988 | Plasma Membrane | transmembrane receptor |
| IFNL1 | ENSG00000182393 | interferon lambda 1 | 7,721 | Extracellular Space | cytokine |
| IFNL2 | ENSG00000183709 | interferon lambda 2 | 7,892 | Extracellular Space | other |
| IFNL3 | ENSG00000197110 | interferon lambda 3 | 7,431 | Extracellular Space | cytokine |
| IFNL3P1 | ENSG00000268510 | interferon lambda 3 pseudogene 1 | 2,774 | Other | other |
| IFNL4 | ENSG00000272395 | interferon lambda 4 (gene/pseudogene) | 3,658 | Other | cytokine |
| IFNLR1 | ENSG00000185436 | interferon lambda receptor 1 | 0,741 | Plasma Membrane | transmembrane receptor |
| IFRD1 | ENSG00000006652 | interferon related developmental regulator 1 | 1,184 | Nucleus | other |
| IFT57 | ENSG00000114446 | intraflagellar transport 57 | 0,716 | Cytoplasm | other |
| IFTAP | ENSG00000166352 | intraflagellar transport associated protein | -0,703 | Extracellular Space | other |
| IGF2 | ENSG00000167244 | insulin like growth factor 2 | 2,917 | Extracellular Space | growth factor |
| IGFBP2 | ENSG00000115457 | insulin like growth factor binding protein 2 | -0,877 | Extracellular Space | other |
| IGHE | ENSG00000211891 | immunoglobulin heavy constant epsilon | 2,048 | Extracellular Space | other |
| IGHMBP2 | ENSG00000132740 | immunoglobulin mu DNA binding protein 2 | 0,888 | Nucleus | enzyme |
| IKBKE | ENSG00000263528 | inhibitor of nuclear factor kappa B kinase subunit epsilon | 1,386 | Cytoplasm | kinase |
| IKZF2 | ENSG00000030419 | IKAROS family zinc finger 2 | 1,466 | Nucleus | transcription regulator |
| IKZF3 | ENSG00000161405 | IKAROS family zinc finger 3 | 3,129 | Nucleus | transcription regulator |
| IKZF5 | ENSG00000095574 | IKAROS family zinc finger 5 | 0,939 | Nucleus | transcription regulator |
| IL10RA | ENSG00000110324 | interleukin 10 receptor subunit alpha | 2,569 | Plasma Membrane | transmembrane receptor |
| IL11 | ENSG00000095752 | interleukin 11 | 2,369 | Extracellular Space | cytokine |
| IL12A | ENSG00000168811 | interleukin 12A | 1,859 | Extracellular Space | cytokine |
| IL15 | ENSG00000164136 | interleukin 15 | 1,650 | Extracellular Space | cytokine |
| IL15RA | ENSG00000134470 | interleukin 15 receptor subunit alpha | 2,072 | Plasma Membrane | transmembrane receptor |
| IL18R1 | ENSG00000115604 | interleukin 18 receptor 1 | 0,729 | Plasma Membrane | transmembrane receptor |
| IL1A | ENSG00000115008 | interleukin 1 alpha | 4,858 | Extracellular Space | cytokine |
| IL1B | ENSG00000125538 | interleukin 1 beta | 1,382 | Extracellular Space | cytokine |
| IL1RN | ENSG00000136689 | interleukin 1 receptor antagonist | 0,943 | Extracellular Space | cytokine |
| IL22RA1 | ENSG00000142677 | interleukin 22 receptor subunit alpha 1 | 1,319 | Plasma Membrane | transmembrane receptor |
| IL32 | ENSG00000008517 | interleukin 32 | 2,159 | Extracellular Space | cytokine |
| IL4I1 | ENSG00000104951 | interleukin 4 induced 1 | 2,272 | Cytoplasm | enzyme |
| IL6 | ENSG00000136244 | interleukin 6 | 4,986 | Extracellular Space | cytokine |
| IL6R | ENSG00000160712 | interleukin 6 receptor | 0,685 | Plasma Membrane | transmembrane receptor |
| IL6ST | ENSG00000134352 | interleukin 6 signal transducer | 0,656 | Plasma Membrane | transmembrane receptor |
| IL7 | ENSG00000104432 | interleukin 7 | 1,753 | Extracellular Space | cytokine |
| IL7R | ENSG00000168685 | interleukin 7 receptor | 1,053 | Plasma Membrane | transmembrane receptor |
| ILVBL | ENSG00000105135 | ilvB acetolactate synthase like | -0,601 | Cytoplasm | enzyme |
| IMPA1 | ENSG00000133731 | inositol monophosphatase 1 | 0,850 | Cytoplasm | phosphatase |
| ING3 | ENSG00000071243 | inhibitor of growth family member 3 | 1,160 | Nucleus | other |
| ING4 | ENSG00000111653 | inhibitor of growth family member 4 | -0,679 | Nucleus | transcription regulator |
| INHBA | ENSG00000122641 | inhibin subunit beta A | 2,979 | Extracellular Space | growth factor |
| INO80 | ENSG00000128908 | INO80 complex ATPase subunit | 1,142 | Nucleus | enzyme |
| INO80D | ENSG00000114933 | INO80 complex subunit D | 1,446 | Nucleus | other |
| INSIG1 | ENSG00000186480 | insulin induced gene 1 | 0,843 | Cytoplasm | other |
| INTS6 | ENSG00000102786 | integrator complex subunit 6 | 1,464 | Nucleus | enzyme |
| INTU | ENSG00000164066 | inturned planar cell polarity protein | 0,943 | Cytoplasm | other |
| IPPK | ENSG00000127080 | inositol-pentakisphosphate 2-kinase | 1,319 | Cytoplasm | kinase |
| IQCH | ENSG00000103599 | IQ motif containing H | -0,603 | Other | other |
| IQCK | ENSG00000174628 | IQ motif containing K | -0,660 | Other | other |
| IQGAP3 | ENSG00000183856 | IQ motif containing GTPase activating protein 3 | -0,706 | Plasma Membrane | other |
| IRAK2 | ENSG00000134070 | interleukin 1 receptor associated kinase 2 | 2,329 | Plasma Membrane | kinase |
| IREB2 | ENSG00000136381 | iron responsive element binding protein 2 | 0,712 | Cytoplasm | translation regulator |
| IRF1 | ENSG00000125347 | interferon regulatory factor 1 | 3,475 | Nucleus | transcription regulator |
| IRF1-AS1 | ENSG00000197536 | IRF1 antisense RNA 1 | 2,081 | Other | other |
| IRF2 | ENSG00000168310 | interferon regulatory factor 2 | 0,783 | Nucleus | transcription regulator |
| IRF6 | ENSG00000117595 | interferon regulatory factor 6 | 0,879 | Nucleus | transcription regulator |
| IRF7 | ENSG00000185507 | interferon regulatory factor 7 | 2,961 | Nucleus | transcription regulator |
| IRF8 | ENSG00000140968 | interferon regulatory factor 8 | 1,243 | Nucleus | transcription regulator |
| IRF9 | ENSG00000213928 | interferon regulatory factor 9 | 1,549 | Nucleus | transcription regulator |
| IRGQ | ENSG00000167378 | immunity related GTPase Q | 1,488 | Other | other |
| IRS2 | ENSG00000185950 | insulin receptor substrate 2 | 1,259 | Cytoplasm | enzyme |
| IRX5 | ENSG00000176842 | iroquois homeobox 5 | 0,914 | Nucleus | transcription regulator |
| ISG15 | ENSG00000187608 | ISG15 ubiquitin like modifier | 2,769 | Extracellular Space | other |
| ISG20 | ENSG00000172183 | interferon stimulated exonuclease gene 20 | 3,446 | Nucleus | enzyme |
| ISL2 | ENSG00000159556 | ISL LIM homeobox 2 | 0,652 | Nucleus | transcription regulator |

| | | | | | |
|---|---|---|---|---|---|
| ITGA5 | ENSG00000161638 | integrin subunit alpha 5 | 1,276 | Plasma Membrane | transmembrane receptor |
| ITGAE | ENSG00000083457 | integrin subunit alpha E | -1,030 | Plasma Membrane | other |
| ITGAM | ENSG00000169896 | integrin subunit alpha M | 2,044 | Plasma Membrane | transmembrane receptor |
| ITGB8 | ENSG00000105855 | integrin subunit beta 8 | 0,662 | Plasma Membrane | other |
| ITM2A | ENSG00000078596 | integral membrane protein 2A | -1,127 | Plasma Membrane | other |
| ITM2C | ENSG00000135916 | integral membrane protein 2C | -0,672 | Cytoplasm | other |
| ITPKC | ENSG00000086544 | inositol-trisphosphate 3-kinase C | 1,875 | Cytoplasm | kinase |
| ITSN2 | ENSG00000198399 | intersectin 2 | 1,516 | Cytoplasm | other |
| IYD | ENSG00000009765 | iodotyrosine deiodinase | 0,681 | Plasma Membrane | enzyme |
| JAK2 | ENSG00000096968 | Janus kinase 2 | 2,397 | Cytoplasm | kinase |
| JAZF1 | ENSG00000153814 | JAZF zinc finger 1 | 0,655 | Nucleus | transcription regulator |
| JMJD1C | ENSG00000171988 | jumonji domain containing 1C | 1,752 | Nucleus | enzyme |
| JMJD7-PLA2G4B | ENSG00000168970 | JMJD7-PLA2G4B readthrough | 0,768 | Cytoplasm | other |
| JUN | ENSG00000177606 | Jun proto-oncogene, AP-1 transcription factor subunit | 3,239 | Nucleus | transcription regulator |
| JUNB | ENSG00000171223 | JunB proto-oncogene, AP-1 transcription factor subunit | 1,657 | Nucleus | transcription regulator |
| JUND | ENSG00000130522 | JunD proto-oncogene, AP-1 transcription factor subunit | 0,865 | Nucleus | transcription regulator |
| KANSL1 | ENSG00000120071 | KAT8 regulatory NSL complex subunit 1 | 1,074 | Nucleus | other |
| KAT6A | ENSG00000083168 | lysine acetyltransferase 6A | 0,713 | Nucleus | enzyme |
| KBTBD2 | ENSG00000170852 | kelch repeat and BTB domain containing 2 | 0,655 | Other | other |
| KBTBD8 | ENSG00000163376 | kelch repeat and BTB domain containing 8 | 1,392 | Cytoplasm | other |
| KCNE3 | ENSG00000175538 | potassium voltage-gated channel subfamily E regulatory subunit 3 | -0,652 | Plasma Membrane | ion channel |
| KCNH8 | ENSG00000183960 | potassium voltage-gated channel subfamily H member 8 | 0,671 | Plasma Membrane | ion channel |
| KCNJ15 | ENSG00000157551 | potassium inwardly rectifying channel subfamily J member 15 | 0,640 | Plasma Membrane | ion channel |
| KCNMB4 | ENSG00000135643 | potassium calcium-activated channel subfamily M regulatory beta subunit 4 | -0,775 | Plasma Membrane | ion channel |
| KCNN1 | ENSG00000105642 | potassium calcium-activated channel subfamily N member 1 | 1,176 | Plasma Membrane | ion channel |
| KCNT2 | ENSG00000162687 | potassium sodium-activated channel subfamily T member 2 | 1,195 | Plasma Membrane | ion channel |
| KCNV1 | ENSG00000164794 | potassium voltage-gated channel modifier subfamily V member 1 | 1,557 | Plasma Membrane | ion channel |
| KCTD12 | ENSG00000178695 | potassium channel tetramerization domain containing 12 | -0,623 | Plasma Membrane | ion channel |
| KDM2A | ENSG00000173120 | lysine demethylase 2A | 1,138 | Nucleus | enzyme |
| KDM5A | ENSG00000073614 | lysine demethylase 5A | 0,701 | Nucleus | transcription regulator |
| KDM5B | ENSG00000117139 | lysine demethylase 5B | 1,066 | Nucleus | transcription regulator |
| KDM6A | ENSG00000147050 | lysine demethylase 6A | 1,895 | Nucleus | enzyme |
| KDM6B | ENSG00000132510 | lysine demethylase 6B | 2,004 | Extracellular Space | enzyme |
| KDM7A | ENSG00000006459 | lysine demethylase 7A | 1,394 | Nucleus | enzyme |
| KIAA0040 | ENSG00000235750 | KIAA0040 | 0,680 | Other | other |
| KIAA0513 | ENSG00000135709 | KIAA0513 | 0,946 | Other | other |
| KIAA0895 | ENSG00000164542 | KIAA0895 | 1,146 | Other | other |
| KIAA1217 | ENSG00000120549 | KIAA1217 | 0,887 | Cytoplasm | other |
| KIF12 | ENSG00000136883 | kinesin family member 12 | -1,184 | Cytoplasm | other |
| KIF20A | ENSG00000112984 | kinesin family member 20A | -0,821 | Cytoplasm | other |
| KIF21A | ENSG00000139116 | kinesin family member 21A | 0,742 | Cytoplasm | other |
| KIFC1 | ENSG00000237649 | kinesin family member C1 | -0,848 | Nucleus | enzyme |
| KLC4 | ENSG00000137171 | kinesin light chain 4 | -1,069 | Other | other |
| KLF10 | ENSG00000155090 | Kruppel like factor 10 | 1,464 | Nucleus | transcription regulator |
| KLF15 | ENSG00000163884 | Kruppel like factor 15 | 1,265 | Nucleus | transcription regulator |
| KLF2 | ENSG00000127528 | Kruppel like factor 2 | 1,438 | Nucleus | transcription regulator |
| KLF4 | ENSG00000136826 | Kruppel like factor 4 | 4,161 | Nucleus | transcription regulator |
| KLF5 | ENSG00000102554 | Kruppel like factor 5 | 0,756 | Nucleus | transcription regulator |
| KLF6 | ENSG00000067082 | Kruppel like factor 6 | 2,333 | Nucleus | transcription regulator |
| KLHDC10 | ENSG00000128607 | kelch domain containing 10 | 0,896 | Nucleus | other |
| KLHDC2 | ENSG00000165516 | kelch domain containing 2 | -0,679 | Cytoplasm | other |
| KLHL11 | ENSG00000178502 | kelch like family member 11 | 0,627 | Cytoplasm | other |
| KLHL15 | ENSG00000174010 | kelch like family member 15 | 0,872 | Nucleus | other |
| KLHL18 | ENSG00000114648 | kelch like family member 18 | 0,611 | Other | other |
| KLHL20 | ENSG00000076321 | kelch like family member 20 | 0,964 | Plasma Membrane | enzyme |
| KLHL25 | ENSG00000183655 | kelch like family member 25 | 0,912 | Cytoplasm | other |
| KLHL28 | ENSG00000179454 | kelch like family member 28 | 0,759 | Other | other |
| KLHL29 | ENSG00000119771 | kelch like family member 29 | 0,937 | Other | other |
| KLHL5 | ENSG00000109790 | kelch like family member 5 | 0,635 | Extracellular Space | other |
| KLK6 | ENSG00000167755 | kallikrein related peptidase 6 | -0,734 | Extracellular Space | peptidase |
| KLK7 | ENSG00000169035 | kallikrein related peptidase 7 | -0,976 | Extracellular Space | peptidase |
| KMO | ENSG00000117009 | kynurenine 3-monooxygenase | 2,752 | Cytoplasm | enzyme |
| KMT2A | ENSG00000118058 | lysine methyltransferase 2A | 0,805 | Nucleus | transcription regulator |
| KMT2B | ENSG00000272333 | lysine methyltransferase 2B | 0,640 | Nucleus | transcription regulator |
| KMT2C | ENSG00000055609 | lysine methyltransferase 2C | 1,442 | Nucleus | transcription regulator |
| KMT2D | ENSG00000167548 | lysine methyltransferase 2D | 0,702 | Nucleus | transcription regulator |
| KMT2E | ENSG00000005483 | lysine methyltransferase 2E (inactive) | 1,578 | Nucleus | enzyme |
| KPNA4 | ENSG00000186432 | karyopherin subunit alpha 4 | 0,763 | Nucleus | transporter |
| KPNA5 | ENSG00000196911 | karyopherin subunit alpha 5 | 1,108 | Cytoplasm | other |
| KRBA1 | ENSG00000133619 | KRAB-A domain containing 1 | 0,751 | Other | other |
| KRBOX1 | ENSG00000240747 | KRAB box domain containing 1 | -0,768 | Other | other |
| KRT6B | ENSG00000185479 | keratin 6B | 0,719 | Cytoplasm | other |
| KRTAP1-1 | ENSG00000262808 | keratin associated protein 1-1 | 1,103 | Cytoplasm | other |
| KSR1 | ENSG00000141068 | kinase suppressor of ras 1 | 0,916 | Cytoplasm | kinase |
| LAGE3 | ENSG00000196976 | L antigen family member 3 | -0,635 | Nucleus | other |
| LAMB3 | ENSG00000196878 | laminin subunit beta 3 | 0,819 | Extracellular Space | transporter |
| LAMP3 | ENSG00000078081 | lysosomal associated membrane protein 3 | 3,750 | Plasma Membrane | other |
| LAMTOR4 | ENSG00000188186 | late endosomal/lysosomal adaptor, MAPK and MTOR activator 4 | -0,851 | Cytoplasm | other |
| LAMTOR5 | ENSG00000134248 | late endosomal/lysosomal adaptor, MAPK and MTOR activator 5 | -0,761 | Cytoplasm | other |
| LAP3 | ENSG00000002549 | leucine aminopeptidase 3 | 1,749 | Cytoplasm | peptidase |
| LATS1 | ENSG00000131023 | large tumor suppressor kinase 1 | 0,772 | Nucleus | kinase |
| LATS2 | ENSG00000150457 | large tumor suppressor kinase 2 | 1,011 | Nucleus | kinase |
| LCA5 | ENSG00000135338 | lebercilin LCA5 | 0,831 | Extracellular Space | other |
| LCN10 | ENSG00000187922 | lipocalin 10 | 1,193 | Extracellular Space | other |
| LCOR | ENSG00000196233 | ligand dependent nuclear receptor corepressor | 0,880 | Nucleus | transcription regulator |
| LEAP2 | ENSG00000164406 | liver enriched antimicrobial peptide 2 | 0,692 | Extracellular Space | other |
| LEPROT | ENSG00000213625 | leptin receptor overlapping transcript | -0,802 | Plasma Membrane | other |
| LGALS3BP | ENSG00000108679 | galectin 3 binding protein | 0,621 | Plasma Membrane | transmembrane receptor |
| LGALS4 | ENSG00000171747 | galectin 4 | -0,811 | Extracellular Space | other |
| LGALS9 | ENSG00000168961 | galectin 9 | 2,307 | Plasma Membrane | other |
| LIF | ENSG00000128342 | LIF interleukin 6 family cytokine | 1,068 | Extracellular Space | cytokine |
| LIFR | ENSG00000113594 | LIF receptor subunit alpha | 1,352 | Plasma Membrane | transmembrane receptor |
| LIG4 | ENSG00000174405 | DNA ligase 4 | 1,030 | Nucleus | enzyme |
| LIN37 | ENSG00000267796 | lin-37 DREAM MuvB core complex component | 1,099 | Nucleus | other |
| LINC01559 | ENSG00000180861 | long intergenic non-protein coding RNA 1559 | -1,011 | Other | other |
| LINC01949 | ENSG00000233828 | long intergenic non-protein coding RNA 1949 | 0,770 | Other | other |
| LINS1 | ENSG00000140471 | lines homolog 1 | 0,656 | Other | other |
| LIPG | ENSG00000101670 | lipase G, endothelial type | 0,956 | Extracellular Space | enzyme |
| LMO2 | ENSG00000135363 | LIM domain only 2 | 2,097 | Nucleus | transcription regulator |

| | | | | | |
|---|---|---|---|---|---|
| LMO4 | ENSG00000143013 | LIM domain only 4 | -0,723 | Nucleus | transcription regulator |
| LMO7 | ENSG00000136153 | LIM domain 7 | 0,667 | Cytoplasm | enzyme |
| LNPEP | ENSG00000113441 | leucyl and cystinyl aminopeptidase | 0,908 | Cytoplasm | peptidase |
| LNX1 | ENSG00000072201 | ligand of numb-protein X 1 | 1,375 | Cytoplasm | enzyme |
| LNX2 | ENSG00000139517 | ligand of numb-protein X 2 | 0,948 | Other | other |
| LONRF3 | ENSG00000175556 | LON peptidase N-terminal domain and ring finger 3 | 1,257 | Other | other |
| LOX | ENSG00000113083 | lysyl oxidase | -0,676 | Extracellular Space | enzyme |
| LOXL3 | ENSG00000115318 | lysyl oxidase like 3 | 1,020 | Extracellular Space | enzyme |
| LPA | ENSG00000198670 | lipoprotein(a) | 1,381 | Extracellular Space | other |
| LPIN2 | ENSG00000101577 | lipin 2 | 0,943 | Nucleus | phosphatase |
| LRCH1 | ENSG00000136141 | leucine rich repeats and calponin homology domain containing 1 | 0,651 | Cytoplasm | other |
| LRCH3 | ENSG00000186001 | leucine rich repeats and calponin homology domain containing 3 | 0,738 | Cytoplasm | other |
| LRCH4 | ENSG00000077454 | leucine rich repeats and calponin homology domain containing 4 | 0,610 | Cytoplasm | transcription regulator |
| LRIF1 | ENSG00000121931 | ligand dependent nuclear receptor interacting factor 1 | 1,050 | Nucleus | other |
| LRIG2 | ENSG00000198799 | leucine rich repeats and immunoglobulin like domains 2 | 1,114 | Cytoplasm | other |
| LRIG3 | ENSG00000139263 | leucine rich repeats and immunoglobulin like domains 3 | 0,686 | Extracellular Space | other |
| LRP1 | ENSG00000123384 | LDL receptor related protein 1 | -0,681 | Plasma Membrane | transmembrane receptor |
| LRP2 | ENSG00000081479 | LDL receptor related protein 2 | 1,509 | Plasma Membrane | transporter |
| LRRC20 | ENSG00000172731 | leucine rich repeat containing 20 | -0,602 | Other | other |
| LRRC49 | ENSG00000137821 | leucine rich repeat containing 49 | 0,853 | Cytoplasm | other |
| LRRN3 | ENSG00000173114 | leucine rich repeat neuronal 3 | 2,351 | Extracellular Space | other |
| LRSAM1 | ENSG00000148356 | leucine rich repeat and sterile alpha motif containing 1 | 0,688 | Cytoplasm | enzyme |
| LSM7 | ENSG00000130332 | LSM7 homolog, U6 small nuclear RNA and mRNA degradation associated | -0,697 | Nucleus | other |
| LSMEM1 | ENSG00000181016 | leucine rich single-pass membrane protein 1 | 0,940 | Cytoplasm | other |
| LTB | ENSG00000227507 | lymphotoxin beta | 3,753 | Extracellular Space | cytokine |
| LTN1 | ENSG00000198862 | listerin E3 ubiquitin protein ligase 1 | 0,613 | Nucleus | enzyme |
| LXN | ENSG00000079257 | latexin | -0,718 | Cytoplasm | other |
| LY6E | ENSG00000160932 | lymphocyte antigen 6 family member E | 0,790 | Plasma Membrane | other |
| LYN | ENSG00000254087 | LYN proto-oncogene, Src family tyrosine kinase | 1,109 | Cytoplasm | kinase |
| LYRM7 | ENSG00000186687 | LYR motif containing 7 | -0,871 | Cytoplasm | other |
| LYSMD2 | ENSG00000140280 | LysM domain containing 2 | 0,659 | Other | other |
| LYST | ENSG00000143669 | lysosomal trafficking regulator | 1,716 | Cytoplasm | transporter |
| LYZ | ENSG00000090382 | lysozyme | -0,797 | Extracellular Space | enzyme |
| MACC1 | ENSG00000183742 | MET transcriptional regulator MACC1 | 0,883 | Nucleus | transcription regulator |
| MACROD1 | ENSG00000133315 | mono-ADP ribosylhydrolase 1 | -0,692 | Cytoplasm | enzyme |
| MAD2L1BP | ENSG00000124688 | MAD2L1 binding protein | 0,779 | Nucleus | other |
| MAFA | ENSG00000182759 | MAF bZIP transcription factor A | 1,416 | Nucleus | transcription regulator |
| MAFF | ENSG00000185022 | MAF bZIP transcription factor F | 1,489 | Nucleus | transcription regulator |
| MAFG | ENSG00000197063 | MAF bZIP transcription factor G | 1,124 | Nucleus | transcription regulator |
| MAGI3 | ENSG00000081026 | membrane associated guanylate kinase, WW and PDZ domain containing 3 | 0,663 | Cytoplasm | kinase |
| MAML2 | ENSG00000184384 | mastermind like transcriptional coactivator 2 | 0,874 | Nucleus | transcription regulator |
| MAOB | ENSG00000069535 | monoamine oxidase B | -0,669 | Cytoplasm | enzyme |
| MAP11 | ENSG00000146826 | microtubule associated protein 11 | 0,897 | Cytoplasm | other |
| MAP2 | ENSG00000078018 | microtubule associated protein 2 | 1,209 | Plasma Membrane | other |
| MAP2K4 | ENSG00000065559 | mitogen-activated protein kinase kinase 4 | 0,661 | Cytoplasm | kinase |
| MAP3K1 | ENSG00000095015 | mitogen-activated protein kinase kinase kinase 1 | 0,963 | Cytoplasm | kinase |
| MAP3K2 | ENSG00000169967 | mitogen-activated protein kinase kinase kinase 2 | 0,621 | Cytoplasm | kinase |
| MAP3K8 | ENSG00000107968 | mitogen-activated protein kinase kinase kinase 8 | 3,442 | Cytoplasm | kinase |
| MAP3K9 | ENSG00000006432 | mitogen-activated protein kinase kinase kinase 9 | 0,882 | Cytoplasm | kinase |
| MAP4K3 | ENSG00000011566 | mitogen-activated protein kinase kinase kinase kinase 3 | 1,098 | Plasma Membrane | kinase |
| MAP4K4 | ENSG00000071054 | mitogen-activated protein kinase kinase kinase kinase 4 | 0,803 | Cytoplasm | kinase |
| MAP4K5 | ENSG00000012983 | mitogen-activated protein kinase kinase kinase kinase 5 | 0,853 | Cytoplasm | kinase |
| MAPK6 | ENSG00000069956 | mitogen-activated protein kinase 6 | 0,644 | Cytoplasm | kinase |
| MAPK8IP2 | ENSG00000008735 | mitogen-activated protein kinase 8 interacting protein 2 | 1,219 | Cytoplasm | other |
| MAPKBP1 | ENSG00000137802 | mitogen-activated protein kinase binding protein 1 | 0,785 | Cytoplasm | other |
| MARCHF5 | ENSG00000198060 | membrane associated ring-CH-type finger 5 | 0,896 | Cytoplasm | enzyme |
| MARCHF7 | ENSG00000136536 | membrane associated ring-CH-type finger 7 | 0,746 | Extracellular Space | other |
| MARK3 | ENSG00000075413 | microtubule affinity regulating kinase 3 | 0,949 | Cytoplasm | kinase |
| MAST4 | ENSG00000069020 | microtubule associated serine/threonine kinase family member 4 | 1,422 | Other | kinase |
| MASTL | ENSG00000120539 | microtubule associated serine/threonine kinase like | 0,975 | Cytoplasm | kinase |
| MATN4 | ENSG00000124159 | matrilin 4 | 0,628 | Extracellular Space | other |
| MB21D2 | ENSG00000180611 | Mab-21 domain containing 2 | 2,961 | Other | other |
| MBD1 | ENSG00000141644 | methyl-CpG binding domain protein 1 | 1,144 | Nucleus | transcription regulator |
| MBNL3 | ENSG00000076770 | muscleblind like splicing regulator 3 | -0,621 | Nucleus | other |
| MCF2L2 | ENSG00000053524 | MCF.2 cell line derived transforming sequence-like 2 | 0,934 | Other | other |
| MCL1 | ENSG00000143384 | MCL1 apoptosis regulator, BCL2 family member | 0,816 | Cytoplasm | transporter |
| MCM6 | ENSG00000076003 | minichromosome maintenance complex component 6 | -0,644 | Nucleus | enzyme |
| MCPH1 | ENSG00000147316 | microcephalin 1 | 0,658 | Nucleus | other |
| MDH1 | ENSG00000014641 | malate dehydrogenase 1 | -0,601 | Cytoplasm | enzyme |
| MDM4 | ENSG00000198625 | MDM4 regulator of p53 | 0,978 | Nucleus | transcription regulator |
| MED12L | ENSG00000144893 | mediator complex subunit 12L | 1,388 | Extracellular Space | other |
| MED13 | ENSG00000108510 | mediator complex subunit 13 | 1,020 | Nucleus | transcription regulator |
| MED13L | ENSG00000123066 | mediator complex subunit 13L | 1,369 | Nucleus | other |
| MED15 | ENSG00000099917 | mediator complex subunit 15 | 0,762 | Nucleus | transcription regulator |
| MED26 | ENSG00000105085 | mediator complex subunit 26 | 0,938 | Nucleus | transcription regulator |
| MEF2A | ENSG00000068305 | myocyte enhancer factor 2A | 0,687 | Nucleus | transcription regulator |
| MEF2D | ENSG00000116604 | myocyte enhancer factor 2D | 1,002 | Nucleus | transcription regulator |
| MEGF6 | ENSG00000162591 | multiple EGF like domains 6 | -0,768 | Cytoplasm | other |
| MEIS1 | ENSG00000143995 | Meis homeobox 1 | 0,845 | Nucleus | transcription regulator |
| MELTF | ENSG00000163975 | melanotransferrin | -0,915 | Plasma Membrane | other |
| METRN | ENSG00000103260 | meteorin, glial cell differentiation regulator | -0,691 | Extracellular Space | other |
| METTL7A | ENSG00000185432 | methyltransferase like 7A | -1,289 | Cytoplasm | other |
| METTL7B | ENSG00000170439 | methyltransferase like 7B | -0,997 | Other | enzyme |
| MFGE8 | ENSG00000140545 | milk fat globule EGF and factor V/VIII domain containing | -0,664 | Extracellular Space | other |
| MFSD14A | ENSG00000156875 | major facilitator superfamily domain containing 14A | 1,245 | Other | transporter |
| MFSD2A | ENSG00000168389 | major facilitator superfamily domain containing 2A | 1,594 | Plasma Membrane | transporter |
| MGAM2 | ENSG00000257743 | maltase-glucoamylase 2 (putative) | -0,622 | Other | other |
| MGST2 | ENSG00000085871 | microsomal glutathione S-transferase 2 | -0,696 | Cytoplasm | enzyme |
| MICAL3 | ENSG00000243156 | microtubule associated monooxygenase, calponin and LIM domain containing 3 | 1,178 | Cytoplasm | enzyme |
| MICB | ENSG00000204516 | MHC class I polypeptide-related sequence B | 2,054 | Plasma Membrane | transmembrane receptor |
| MICU3 | ENSG00000155970 | mitochondrial calcium uptake family member 3 | 0,888 | Cytoplasm | other |
| MID2 | ENSG00000080561 | midline 2 | 0,892 | Cytoplasm | other |
| MIDEAS | ENSG00000156030 | mitotic deacetylase associated SANT domain protein | 1,046 | Nucleus | transcription regulator |
| MIER1 | ENSG00000198160 | MIER1 transcriptional regulator | 1,053 | Nucleus | transcription regulator |
| MIER2 | ENSG00000105556 | MIER family member 2 | 0,619 | Nucleus | transcription regulator |
| MIER3 | ENSG00000155545 | MIER family member 3 | 0,681 | Nucleus | transcription regulator |
| MKRN2 | ENSG00000075975 | makorin ring finger protein 2 | 0,629 | Other | other |
| MLKL | ENSG00000168404 | mixed lineage kinase domain like pseudokinase | 1,078 | Cytoplasm | kinase |
| MLPH | ENSG00000115648 | melanophilin | -0,683 | Cytoplasm | other |

| | | | | | |
|---|---|---|---|---|---|
| MMAB | ENSG00000139428 | metabolism of cobalamin associated B | -0,777 | Cytoplasm | enzyme |
| MMD | ENSG00000108960 | monocyte to macrophage differentiation associated | 0,642 | Plasma Membrane | kinase |
| MMD2 | ENSG00000136297 | monocyte to macrophage differentiation associated 2 | 1,929 | Other | kinase |
| MME | ENSG00000196549 | membrane metalloendopeptidase | -0,986 | Plasma Membrane | peptidase |
| MMP7 | ENSG00000137673 | matrix metallopeptidase 7 | -0,672 | Extracellular Space | peptidase |
| MNS1 | ENSG00000138587 | meiosis specific nuclear structural 1 | -0,779 | Cytoplasm | other |
| MOB3B | ENSG00000120162 | MOB kinase activator 3B | -0,643 | Other | other |
| MOB3C | ENSG00000142961 | MOB kinase activator 3C | 1,099 | Other | other |
| MOGAT1 | ENSG00000124003 | monoacylglycerol O-acyltransferase 1 | -1,563 | Cytoplasm | enzyme |
| MON2 | ENSG00000061987 | MON2 homolog, regulator of endosome-to-Golgi trafficking | 0,612 | Cytoplasm | other |
| MORC3 | ENSG00000159256 | MORC family CW-type zinc finger 3 | 0,858 | Nucleus | other |
| MORN2 | ENSG00000188010 | MORN repeat containing 2 | -0,832 | Other | other |
| MOSPD3 | ENSG00000106330 | motile sperm domain containing 3 | -0,832 | Other | other |
| MOV10 | ENSG00000155363 | Mov10 RISC complex RNA helicase | 1,003 | Nucleus | enzyme |
| MPC2 | ENSG00000143158 | mitochondrial pyruvate carrier 2 | -0,827 | Plasma Membrane | other |
| MPND | ENSG00000008382 | MPN domain containing | -0,677 | Other | other |
| MPZL3 | ENSG00000160588 | myelin protein zero like 3 | 1,409 | Other | other |
| MRNIP | ENSG00000161010 | MRN complex interacting protein | 0,895 | Nucleus | other |
| MRTFA | ENSG00000196588 | myocardin related transcription factor A | 0,878 | Nucleus | transcription regulator |
| MSANTD3 | ENSG00000066697 | Myb/SANT DNA binding domain containing 3 | 0,616 | Other | other |
| MSH5 | ENSG00000204410 | mutS homolog 5 | 0,637 | Nucleus | enzyme |
| MSL2 | ENSG00000174579 | MSL complex subunit 2 | 0,807 | Nucleus | other |
| MT2A | ENSG00000125148 | metallothionein 2A | 3,630 | Cytoplasm | other |
| MTF1 | ENSG00000188786 | metal regulatory transcription factor 1 | 1,145 | Nucleus | transcription regulator |
| MTF2 | ENSG00000143033 | metal response element binding transcription factor 2 | 0,821 | Nucleus | transcription regulator |
| MTMR3 | ENSG00000100330 | myotubularin related protein 3 | 1,392 | Cytoplasm | phosphatase |
| MTMR9 | ENSG00000104643 | myotubularin related protein 9 | 0,836 | Cytoplasm | phosphatase |
| MTPAP | ENSG00000107951 | mitochondrial poly(A) polymerase | 0,764 | Cytoplasm | enzyme |
| MTRNR2L3 | ENSG00000256222 | MT-RNR2 like 3 | 0,806 | Other | other |
| MTSS2 | ENSG00000132613 | MTSS I-BAR domain containing 2 | -0,813 | Plasma Membrane | other |
| MTURN | ENSG00000180354 | maturin, neural progenitor differentiation regulator homolog | -0,634 | Cytoplasm | other |
| MUC13 | ENSG00000173702 | mucin 13, cell surface associated | 1,695 | Extracellular Space | other |
| MUC20 | ENSG00000176945 | mucin 20, cell surface associated | -0,681 | Plasma Membrane | other |
| MX1 | ENSG00000157601 | MX dynamin like GTPase 1 | 3,085 | Cytoplasm | enzyme |
| MX2 | ENSG00000183486 | MX dynamin like GTPase 2 | 4,126 | Nucleus | enzyme |
| MXD1 | ENSG00000059728 | MAX dimerization protein 1 | 3,355 | Nucleus | transcription regulator |
| MXD3 | ENSG00000213347 | MAX dimerization protein 3 | -0,743 | Nucleus | transcription regulator |
| MXD4 | ENSG00000123933 | MAX dimerization protein 4 | -0,743 | Nucleus | transcription regulator |
| MXRA7 | ENSG00000182534 | matrix remodeling associated 7 | -0,649 | Extracellular Space | other |
| MYCBP2 | ENSG00000005810 | MYC binding protein 2 | 1,374 | Nucleus | enzyme |
| MYCL | ENSG00000116990 | MYCL proto-oncogene, bHLH transcription factor | 0,971 | Nucleus | transcription regulator |
| MYD88 | ENSG00000172936 | MYD88 innate immune signal transduction adaptor | 1,432 | Plasma Membrane | other |
| MYH10 | ENSG00000133026 | myosin heavy chain 10 | -0,649 | Cytoplasm | enzyme |
| MYL6B | ENSG00000196465 | myosin light chain 6B | -0,603 | Cytoplasm | other |
| MYLIP | ENSG00000007944 | myosin regulatory light chain interacting protein | 1,258 | Cytoplasm | enzyme |
| MYO10 | ENSG00000145555 | myosin X | 0,604 | Cytoplasm | enzyme |
| MYRFL | ENSG00000166268 | myelin regulatory factor like | 0,884 | Other | other |
| MYSM1 | ENSG00000162601 | Myb like, SWIRM and MPN domains 1 | 1,020 | Nucleus | enzyme |
| N4BP1 | ENSG00000102921 | NEDD4 binding protein 1 | 1,948 | Cytoplasm | other |
| N4BP3 | ENSG00000145911 | NEDD4 binding protein 3 | 1,236 | Cytoplasm | other |
| NAALADL1 | ENSG00000168060 | N-acetylated alpha-linked acidic dipeptidase like 1 | 1,930 | Plasma Membrane | peptidase |
| NABP1 | ENSG00000173559 | nucleic acid binding protein 1 | 0,756 | Nucleus | other |
| NAIPP4 | ENSG00000251634 | NAIP pseudogene 4 | 2,436 | Other | other |
| NAMPT | ENSG00000105835 | nicotinamide phosphoribosyltransferase | 1,251 | Extracellular Space | cytokine |
| NAMPTP1 | ENSG00000229644 | nicotinamide phosphoribosyltransferase pseudogene 1 | 1,299 | Other | other |
| NAPA | ENSG00000105402 | NSF attachment protein alpha | 1,054 | Cytoplasm | transporter |
| NAV2 | ENSG00000166833 | neuron navigator 2 | 2,098 | Nucleus | other |
| NAV3 | ENSG00000067798 | neuron navigator 3 | 0,841 | Nucleus | other |
| NBPF10 (includes others) | ENSG00000271425 | NBPF member 19 | 1,362 | Other | other |
| NCALD | ENSG00000104490 | neurocalcin delta | 1,462 | Cytoplasm | other |
| NCAPD2 | ENSG00000010292 | non-SMC condensin I complex subunit D2 | -0,659 | Nucleus | other |
| NCAPG | ENSG00000109805 | non-SMC condensin I complex subunit G | -0,602 | Nucleus | other |
| NCOA3 | ENSG00000124151 | nuclear receptor coactivator 3 | 0,866 | Nucleus | transcription regulator |
| NCOA5 | ENSG00000124160 | nuclear receptor coactivator 5 | 0,788 | Nucleus | other |
| NCOA7 | ENSG00000111912 | nuclear receptor coactivator 7 | 2,358 | Nucleus | transcription regulator |
| NDRG1 | ENSG00000104419 | N-myc downstream regulated 1 | -1,985 | Nucleus | kinase |
| NDRG2 | ENSG00000165795 | NDRG family member 2 | -0,956 | Cytoplasm | other |
| NDUFA1 | ENSG00000125356 | NADH:ubiquinone oxidoreductase subunit A1 | -0,666 | Cytoplasm | enzyme |
| NDUFA13 | ENSG00000186010 | NADH:ubiquinone oxidoreductase subunit A13 | -0,627 | Cytoplasm | enzyme |
| NDUFA2 | ENSG00000131495 | NADH:ubiquinone oxidoreductase subunit A2 | -0,709 | Cytoplasm | enzyme |
| NDUFB1 | ENSG00000183648 | NADH:ubiquinone oxidoreductase subunit B1 | -0,693 | Cytoplasm | enzyme |
| NEB | ENSG00000183091 | nebulin | -1,207 | Cytoplasm | other |
| NECAP1 | ENSG00000089818 | NECAP endocytosis associated 1 | 1,157 | Plasma Membrane | other |
| NECTIN4 | ENSG00000143217 | nectin cell adhesion molecule 4 | 0,767 | Plasma Membrane | other |
| NEDD4 | ENSG00000069869 | NEDD4 E3 ubiquitin protein ligase | 1,196 | Cytoplasm | enzyme |
| NEDD4L | ENSG00000049759 | NEDD4 like E3 ubiquitin protein ligase | 0,998 | Cytoplasm | enzyme |
| NEDD9 | ENSG00000111859 | neural precursor cell expressed, developmentally down-regulated 9 | 1,247 | Nucleus | other |
| NEK2 | ENSG00000117650 | NIMA related kinase 2 | -0,734 | Cytoplasm | kinase |
| NEURL3 | ENSG00000163121 | neuralized E3 ubiquitin protein ligase 3 | 2,810 | Other | enzyme |
| NEXN | ENSG00000162614 | nexilin F-actin binding protein | 0,836 | Plasma Membrane | other |
| NFAT5 | ENSG00000102908 | nuclear factor of activated T cells 5 | 1,869 | Nucleus | transcription regulator |
| NFATC2 | ENSG00000101096 | nuclear factor of activated T cells 2 | 1,076 | Nucleus | transcription regulator |
| NFE2L2 | ENSG00000116044 | nuclear factor, erythroid 2 like 2 | 0,764 | Nucleus | transcription regulator |
| NFE2L3 | ENSG00000050344 | nuclear factor, erythroid 2 like 3 | 0,857 | Nucleus | transcription regulator |
| NFIL3 | ENSG00000165030 | nuclear factor, interleukin 3 regulated | 1,177 | Nucleus | transcription regulator |
| NFIX | ENSG00000008441 | nuclear factor I X | 0,674 | Nucleus | transcription regulator |
| NFKB1 | ENSG00000109320 | nuclear factor kappa B subunit 1 | 1,685 | Nucleus | transcription regulator |
| NFKB2 | ENSG00000077150 | nuclear factor kappa B subunit 2 | 1,864 | Nucleus | transcription regulator |
| NFKBIA | ENSG00000100906 | NFKB inhibitor alpha | 4,323 | Cytoplasm | transcription regulator |
| NFKBIB | ENSG00000104825 | NFKB inhibitor beta | 0,802 | Nucleus | transcription regulator |
| NFKBID | ENSG00000167604 | NFKB inhibitor delta | 1,588 | Nucleus | transcription regulator |
| NFKBIE | ENSG00000146232 | NFKB inhibitor epsilon | 1,949 | Nucleus | transcription regulator |
| NFKBIZ | ENSG00000144802 | NFKB inhibitor zeta | 2,132 | Nucleus | transcription regulator |
| NFU1 | ENSG00000169599 | NFU1 iron-sulfur cluster scaffold | -0,607 | Cytoplasm | other |
| NFX1 | ENSG00000086102 | nuclear transcription factor, X-box binding 1 | 0,654 | Nucleus | transcription regulator |
| NGFR | ENSG00000064300 | nerve growth factor receptor | 1,412 | Plasma Membrane | transmembrane receptor |
| NHSL1 | ENSG00000135540 | NHS like 1 | 0,629 | Other | other |
| NHSL2 | ENSG00000204131 | NHS like 2 | 0,966 | Other | other |
| NIBAN1 | ENSG00000135842 | niban apoptosis regulator 1 | 1,021 | Cytoplasm | other |

| | | | | | |
|---|---|---|---|---|---|
| NICN1 | ENSG00000145029 | nicolin 1 | -1,299 | Nucleus | other |
| NID1 | ENSG00000116962 | nidogen 1 | 1,148 | Extracellular Space | other |
| NIPBL | ENSG00000164190 | NIPBL cohesin loading factor | 0,850 | Nucleus | transcription regulator |
| NIPSNAP3A | ENSG00000136783 | nipsnap homolog 3A | -0,603 | Nucleus | other |
| NKD2 | ENSG00000145506 | NKD inhibitor of WNT signaling pathway 2 | 1,816 | Nucleus | other |
| NKIRAS1 | ENSG00000197885 | NFKB inhibitor interacting Ras like 1 | 0,766 | Other | enzyme |
| NKX3-1 | ENSG00000167034 | NK3 homeobox 1 | 2,887 | Nucleus | transcription regulator |
| NLRC5 | ENSG00000140853 | NLR family CARD domain containing 5 | 3,406 | Cytoplasm | transcription regulator |
| NLRP3 | ENSG00000162711 | NLR family pyrin domain containing 3 | 0,607 | Cytoplasm | other |
| NMI | ENSG00000123609 | N-myc and STAT interactor | 1,840 | Cytoplasm | transcription regulator |
| NOCT | ENSG00000151014 | nocturnin | 3,324 | Nucleus | transcription regulator |
| NOD2 | ENSG00000167207 | nucleotide binding oligomerization domain containing 2 | 1,277 | Cytoplasm | other |
| NOL6 | ENSG00000165271 | nucleolar protein 6 | 0,816 | Nucleus | other |
| NONOP2 | ENSG00000237522 | non-POU domain containing, octamer-binding pseudogene 2 | 1,216 | Other | other |
| NOS3 | ENSG00000164867 | nitric oxide synthase 3 | 1,211 | Cytoplasm | enzyme |
| NOTCH1 | ENSG00000148400 | notch receptor 1 | 1,079 | Plasma Membrane | transcription regulator |
| NPC1 | ENSG00000141458 | NPC intracellular cholesterol transporter 1 | 0,719 | Cytoplasm | transporter |
| NPFFR2 | ENSG00000056291 | neuropeptide FF receptor 2 | 2,940 | Plasma Membrane | G-protein coupled receptor |
| NPIPA5 (includes others) | ENSG00000183793 | nuclear pore complex interacting protein family member A5 | 1,049 | Nucleus | other |
| NPIPB5 (includes others) | ENSG00000185864 | nuclear pore complex interacting protein family member B5 | 0,880 | Nucleus | other |
| NPL | ENSG00000135838 | N-acetylneuraminate pyruvate lyase | 0,772 | Cytoplasm | enzyme |
| NPTX1 | ENSG00000171246 | neuronal pentraxin 1 | 1,198 | Extracellular Space | other |
| NR1D1 | ENSG00000126368 | nuclear receptor subfamily 1 group D member 1 | 2,112 | Nucleus | ligand-dependent nuclear receptor |
| NR1D2 | ENSG00000174738 | nuclear receptor subfamily 1 group D member 2 | 1,920 | Nucleus | ligand-dependent nuclear receptor |
| NR2F1 | ENSG00000175745 | nuclear receptor subfamily 2 group F member 1 | 1,096 | Nucleus | ligand-dependent nuclear receptor |
| NR3C1 | ENSG00000113580 | nuclear receptor subfamily 3 group C member 1 | 1,299 | Nucleus | ligand-dependent nuclear receptor |
| NR4A1 | ENSG00000123358 | nuclear receptor subfamily 4 group A member 1 | 0,989 | Nucleus | ligand-dependent nuclear receptor |
| NR4A2 | ENSG00000153234 | nuclear receptor subfamily 4 group A member 2 | 1,047 | Nucleus | ligand-dependent nuclear receptor |
| NR4A3 | ENSG00000119508 | nuclear receptor subfamily 4 group A member 3 | 1,033 | Nucleus | ligand-dependent nuclear receptor |
| NR5A2 | ENSG00000116833 | nuclear receptor subfamily 5 group A member 2 | 1,062 | Nucleus | ligand-dependent nuclear receptor |
| NRBF2 | ENSG00000148572 | nuclear receptor binding factor 2 | 0,721 | Nucleus | transcription regulator |
| NREP | ENSG00000134986 | neuronal regeneration related protein | -0,645 | Cytoplasm | other |
| NRIP1 | ENSG00000180530 | nuclear receptor interacting protein 1 | 0,798 | Nucleus | transcription regulator |
| NRIP3 | ENSG00000175352 | nuclear receptor interacting protein 3 | 1,130 | Other | other |
| NSUN7 | ENSG00000179299 | NOP2/Sun RNA methyltransferase family member 7 | -0,768 | Other | other |
| NT5C3A | ENSG00000122643 | 5'-nucleotidase, cytosolic IIIA | 2,708 | Cytoplasm | phosphatase |
| NT5C3AP1 | ENSG00000213492 | NT5C3A pseudogene 1 | 1,669 | Other | other |
| NT5DC2 | ENSG00000168268 | 5'-nucleotidase domain containing 2 | -0,730 | Cytoplasm | other |
| NT5DC4 | ENSG00000144130 | 5'-nucleotidase domain containing 4 | 0,951 | Other | other |
| NT5E | ENSG00000135318 | 5'-nucleotidase ecto | 0,666 | Plasma Membrane | phosphatase |
| NUAK1 | ENSG00000074590 | NUAK family kinase 1 | 0,762 | Nucleus | kinase |
| NUAK2 | ENSG00000163545 | NUAK family kinase 2 | 3,390 | Nucleus | kinase |
| NUB1 | ENSG00000013374 | negative regulator of ubiquitin like proteins 1 | 1,475 | Nucleus | other |
| NUDCD2 | ENSG00000170584 | NudC domain containing 2 | -0,659 | Cytoplasm | other |
| NUDT1 | ENSG00000106268 | nudix hydrolase 1 | -0,603 | Extracellular Space | phosphatase |
| NUP98 | ENSG00000110713 | nucleoporin 98 and 96 precursor | 0,727 | Nucleus | transporter |
| NXF1 | ENSG00000162231 | nuclear RNA export factor 1 | 0,755 | Nucleus | other |
| NYNRIN | ENSG00000205978 | NYN domain and retroviral integrase containing | -0,821 | Other | other |
| OAS1 | ENSG00000089127 | 2'-5'-oligoadenylate synthetase 1 | 2,742 | Cytoplasm | enzyme |
| OAS2 | ENSG00000111335 | 2'-5'-oligoadenylate synthetase 2 | 3,518 | Cytoplasm | enzyme |
| OAS3 | ENSG00000111331 | 2'-5'-oligoadenylate synthetase 3 | 2,395 | Cytoplasm | enzyme |
| OASL | ENSG00000135114 | 2'-5'-oligoadenylate synthetase like | 3,961 | Cytoplasm | enzyme |
| ODAM | ENSG00000109205 | odontogenic, ameloblast associated | -0,799 | Extracellular Space | other |
| ODC1 | ENSG00000115758 | ornithine decarboxylase 1 | 0,717 | Cytoplasm | enzyme |
| ODF3B | ENSG00000177989 | outer dense fiber of sperm tails 3B | 1,545 | Other | enzyme |
| OFD1 | ENSG00000046651 | OFD1 centriole and centriolar satellite protein | 0,634 | Plasma Membrane | other |
| OGA | ENSG00000198408 | O-GlcNAcase | 1,023 | Cytoplasm | enzyme |
| OGFR | ENSG00000060491 | opioid growth factor receptor | 1,575 | Plasma Membrane | other |
| OGFRL1 | ENSG00000119900 | opioid growth factor receptor like 1 | 0,912 | Other | other |
| OIP5 | ENSG00000104147 | Opa interacting protein 5 | -0,616 | Nucleus | other |
| OLFM4 | ENSG00000102837 | olfactomedin 4 | -1,050 | Extracellular Space | other |
| OPHN1 | ENSG00000079482 | oligophrenin 1 | 0,634 | Cytoplasm | other |
| OPTN | ENSG00000123240 | optineurin | 2,103 | Cytoplasm | other |
| OR4C6 | ENSG00000181903 | olfactory receptor family 4 subfamily C member 6 | 0,755 | Plasma Membrane | G-protein coupled receptor |
| OR52K3P | ENSG00000225101 | olfactory receptor family 52 subfamily K member 3 pseudogene | 2,553 | Other | other |
| OR7E13P | ENSG00000197882 | olfactory receptor family 7 subfamily E member 13 pseudogene | -1,710 | Other | other |
| OR7E91P | ENSG00000205847 | olfactory receptor family 7 subfamily E member 91 pseudogene | 1,298 | Plasma Membrane | other |
| OSBPL11 | ENSG00000144909 | oxysterol binding protein like 11 | 0,623 | Cytoplasm | other |
| OSCP1 | ENSG00000116885 | organic solute carrier partner 1 | 0,711 | Plasma Membrane | transporter |
| OSER1 | ENSG00000132823 | oxidative stress responsive serine rich 1 | 0,792 | Other | enzyme |
| OSGEPL1 | ENSG00000128694 | O-sialoglycoprotein endopeptidase like 1 | -1,298 | Cytoplasm | peptidase |
| OSGIN1 | ENSG00000140961 | oxidative stress induced growth inhibitor 1 | 1,317 | Other | growth factor |
| OSR2 | ENSG00000164920 | odd-skipped related transciption factor 2 | 0,857 | Nucleus | transcription regulator |
| OSTM1 | ENSG00000081087 | osteoclastogenesis associated transmembrane protein 1 | 1,376 | Cytoplasm | other |
| OTUD1 | ENSG00000165312 | OTU deubiquitinase 1 | 3,704 | Other | peptidase |
| OTUD4 | ENSG00000164164 | OTU deubiquitinase 4 | 0,775 | Cytoplasm | peptidase |
| OTUD7B | ENSG00000264522 | OTU deubiquitinase 7B | 1,227 | Cytoplasm | peptidase |
| OTULIN | ENSG00000154124 | OTU deubiquitinase with linear linkage specificity | 1,160 | Cytoplasm | peptidase |
| OVOL1 | ENSG00000172818 | ovo like transcriptional repressor 1 | 1,580 | Nucleus | transcription regulator |
| OXCT1 | ENSG00000083720 | 3-oxoacid CoA-transferase 1 | -0,629 | Cytoplasm | enzyme |
| OXTR | ENSG00000180914 | oxytocin receptor | 1,704 | Plasma Membrane | G-protein coupled receptor |
| P3H2 | ENSG00000090530 | prolyl 3-hydroxylase 2 | 1,969 | Cytoplasm | enzyme |
| PA2G4P4 | ENSG00000230457 | proliferation-associated 2G4 pseudogene 4 | 1,373 | Other | other |
| PAFAH2 | ENSG00000158006 | platelet activating factor acetylhydrolase 2 | -0,727 | Cytoplasm | enzyme |
| PAH | ENSG00000171759 | phenylalanine hydroxylase | -0,711 | Cytoplasm | enzyme |
| PAIP2B | ENSG00000124374 | poly(A) binding protein interacting protein 2B | -0,628 | Other | translation regulator |
| PALMD | ENSG00000099260 | palmdelphin | -1,006 | Cytoplasm | other |
| PANK2 | ENSG00000125779 | pantothenate kinase 2 | 0,713 | Cytoplasm | kinase |
| PAPOLG | ENSG00000115421 | poly(A) polymerase gamma | 0,914 | Nucleus | enzyme |
| PAPSS1 | ENSG00000138801 | 3'-phosphoadenosine 5'-phosphosulfate synthase 1 | -0,641 | Cytoplasm | enzyme |
| PARD3 | ENSG00000148498 | par-3 family cell polarity regulator | 0,624 | Plasma Membrane | other |
| PARD6B | ENSG00000124171 | par-6 family cell polarity regulator beta | 1,477 | Plasma Membrane | other |
| PARP10 | ENSG00000178685 | poly(ADP-ribose) polymerase family member 10 | 2,394 | Cytoplasm | enzyme |
| PARP12 | ENSG00000059378 | poly(ADP-ribose) polymerase family member 12 | 2,375 | Nucleus | enzyme |
| PARP14 | ENSG00000173193 | poly(ADP-ribose) polymerase family member 14 | 2,596 | Cytoplasm | enzyme |
| PARP3 | ENSG00000041880 | poly(ADP-ribose) polymerase family member 3 | 0,780 | Nucleus | enzyme |
| PARP8 | ENSG00000151883 | poly(ADP-ribose) polymerase family member 8 | 1,229 | Other | other |
| PARP9 | ENSG00000138496 | poly(ADP-ribose) polymerase family member 9 | 1,663 | Nucleus | enzyme |
| PATL1 | ENSG00000166889 | PAT1 homolog 1, processing body mRNA decay factor | 0,793 | Cytoplasm | translation regulator |

| | | | | | |
|---|---|---|---|---|---|
| PATL2 | ENSG00000229474 | PAT1 homolog 2 | 1,328 | Cytoplasm | translation regulator |
| PBK | ENSG00000168078 | PDZ binding kinase | -0,695 | Cytoplasm | kinase |
| PBX2 | ENSG00000204304 | PBX homeobox 2 | -1,227 | Nucleus | transcription regulator |
| PBXIP1 | ENSG00000163346 | PBX homeobox interacting protein 1 | -0,955 | Nucleus | transcription regulator |
| PCGF5 | ENSG00000180628 | polycomb group ring finger 5 | 1,071 | Cytoplasm | other |
| PCOLCE2 | ENSG00000163710 | procollagen C-endopeptidase enhancer 2 | -0,696 | Extracellular Space | other |
| PCYOX1 | ENSG00000116005 | prenylcysteine oxidase 1 | -0,736 | Cytoplasm | enzyme |
| PDCD4 | ENSG00000150593 | programmed cell death 4 | -1,288 | Nucleus | other |
| PDE10A | ENSG00000112541 | phosphodiesterase 10A | 0,960 | Cytoplasm | enzyme |
| PDE4B | ENSG00000184588 | phosphodiesterase 4B | 1,181 | Cytoplasm | enzyme |
| PDE4D | ENSG00000113448 | phosphodiesterase 4D | 0,986 | Cytoplasm | enzyme |
| PDGFA | ENSG00000197461 | platelet derived growth factor subunit A | 1,625 | Extracellular Space | growth factor |
| PDGFD | ENSG00000170962 | platelet derived growth factor D | -1,238 | Extracellular Space | growth factor |
| PDZD2 | ENSG00000133401 | PDZ domain containing 2 | 1,421 | Plasma Membrane | other |
| PDZD8 | ENSG00000165650 | PDZ domain containing 8 | 0,627 | Extracellular Space | other |
| PELI1 | ENSG00000197329 | pellino E3 ubiquitin protein ligase 1 | 2,142 | Cytoplasm | enzyme |
| PER1 | ENSG00000179094 | period circadian regulator 1 | 3,267 | Nucleus | transcription regulator |
| PER2 | ENSG00000132326 | period circadian regulator 2 | 1,502 | Nucleus | transcription regulator |
| PET100 | ENSG00000229833 | PET100 cytochrome c oxidase chaperone | -0,919 | Other | other |
| PEX6 | ENSG00000124587 | peroxisomal biogenesis factor 6 | -0,639 | Cytoplasm | enzyme |
| PGAP3 | ENSG00000161395 | post-GPI attachment to proteins phospholipase 3 | -0,610 | Cytoplasm | enzyme |
| PGF | ENSG00000119630 | placental growth factor | 1,331 | Extracellular Space | growth factor |
| PGK1 | ENSG00000102144 | phosphoglycerate kinase 1 | -0,767 | Cytoplasm | kinase |
| PGLYRP2 | ENSG00000161031 | peptidoglycan recognition protein 2 | 2,059 | Plasma Membrane | transmembrane receptor |
| PHACTR2 | ENSG00000112419 | phosphatase and actin regulator 2 | 0,900 | Other | other |
| PHACTR4 | ENSG00000204138 | phosphatase and actin regulator 4 | 1,146 | Plasma Membrane | other |
| PHC2 | ENSG00000134686 | polyhomeotic homolog 2 | 0,734 | Nucleus | other |
| PHC3 | ENSG00000173889 | polyhomeotic homolog 3 | 0,700 | Nucleus | transcription regulator |
| PHF11 | ENSG00000136147 | PHD finger protein 11 | 1,732 | Nucleus | other |
| PHF20L1 | ENSG00000129292 | PHD finger protein 20 like 1 | 0,988 | Other | other |
| PHF21A | ENSG00000135365 | PHD finger protein 21A | 1,359 | Nucleus | other |
| PHF8 | ENSG00000172943 | PHD finger protein 8 | 0,965 | Nucleus | enzyme |
| PHGDH | ENSG00000092621 | phosphoglycerate dehydrogenase | -0,661 | Cytoplasm | enzyme |
| PHLDB2 | ENSG00000144824 | pleckstrin homology like domain family B member 2 | 0,774 | Cytoplasm | other |
| PI4K2B | ENSG00000038210 | phosphatidylinositol 4-kinase type 2 beta | 1,102 | Cytoplasm | kinase |
| PIEZO1 | ENSG00000103335 | piezo type mechanosensitive ion channel component 1 | 0,749 | Cytoplasm | ion channel |
| PIGA | ENSG00000165195 | phosphatidylinositol glycan anchor biosynthesis class A | 1,023 | Cytoplasm | enzyme |
| PIGZ | ENSG00000119227 | phosphatidylinositol glycan anchor biosynthesis class Z | -0,858 | Cytoplasm | enzyme |
| PIK3AP1 | ENSG00000155629 | phosphoinositide-3-kinase adaptor protein 1 | 0,900 | Cytoplasm | other |
| PIK3C2A | ENSG00000011405 | phosphatidylinositol-4-phosphate 3-kinase catalytic subunit type 2 alpha | 0,619 | Cytoplasm | kinase |
| PIK3CA | ENSG00000121879 | phosphatidylinositol-4,5-bisphosphate 3-kinase catalytic subunit alpha | 0,954 | Cytoplasm | kinase |
| PIK3IP1 | ENSG00000100100 | phosphoinositide-3-kinase interacting protein 1 | -0,645 | Cytoplasm | other |
| PIK3R1 | ENSG00000145675 | phosphoinositide-3-kinase regulatory subunit 1 | 0,692 | Cytoplasm | kinase |
| PIK3R3 | ENSG00000117461 | phosphoinositide-3-kinase regulatory subunit 3 | 1,298 | Cytoplasm | kinase |
| PILRB | ENSG00000121716 | paired immunoglobin like type 2 receptor beta | 0,622 | Plasma Membrane | other |
| PIM1 | ENSG00000137193 | Pim-1 proto-oncogene, serine/threonine kinase | 1,742 | Cytoplasm | kinase |
| PIM3 | ENSG00000198355 | Pim-3 proto-oncogene, serine/threonine kinase | 2,370 | Cytoplasm | kinase |
| PINLYP | ENSG00000234465 | phospholipase A2 inhibitor and LY6/PLAUR domain containing | 2,414 | Other | other |
| PIP5K1A | ENSG00000143398 | phosphatidylinositol-4-phosphate 5-kinase type 1 alpha | 1,112 | Cytoplasm | kinase |
| PIPSL | ENSG00000180764 | PIP5K1A and PSMD4 like (pseudogene) | 1,227 | Other | other |
| PITX1 | ENSG00000069011 | paired like homeodomain 1 | 0,906 | Nucleus | transcription regulator |
| PIWIL4 | ENSG00000134627 | piwi like RNA-mediated gene silencing 4 | 0,820 | Cytoplasm | other |
| PKNOX1 | ENSG00000160199 | PBX/knotted 1 homeobox 1 | 0,743 | Nucleus | transcription regulator |
| PLA2G4C | ENSG00000105499 | phospholipase A2 group IVC | 3,271 | Plasma Membrane | enzyme |
| PLA2G4F | ENSG00000168907 | phospholipase A2 group IVF | 0,667 | Cytoplasm | enzyme |
| PLA2G6 | ENSG00000184381 | phospholipase A2 group VI | 1,053 | Cytoplasm | enzyme |
| PLA2R1 | ENSG00000153246 | phospholipase A2 receptor 1 | -0,789 | Plasma Membrane | transmembrane receptor |
| PLAAT4 | ENSG00000133321 | phospholipase A and acyltransferase 4 | 1,369 | Cytoplasm | enzyme |
| PLAG1 | ENSG00000181690 | PLAG1 zinc finger | 0,747 | Nucleus | transcription regulator |
| PLAU | ENSG00000122861 | plasminogen activator, urokinase | 1,024 | Extracellular Space | peptidase |
| PLAUR | ENSG00000011422 | plasminogen activator, urokinase receptor | 2,132 | Plasma Membrane | transmembrane receptor |
| PLCG2 | ENSG00000197943 | phospholipase C gamma 2 | 1,589 | Cytoplasm | enzyme |
| PLCH1 | ENSG00000114805 | phospholipase C eta 1 | 0,623 | Cytoplasm | enzyme |
| PLEKHA4 | ENSG00000105559 | pleckstrin homology domain containing A4 | 1,821 | Cytoplasm | other |
| PLEKHB1 | ENSG00000021300 | pleckstrin homology domain containing B1 | -1,001 | Cytoplasm | other |
| PLEKHF2 | ENSG00000175895 | pleckstrin homology and FYVE domain containing 2 | 0,899 | Cytoplasm | other |
| PLEKHG3 | ENSG00000126822 | pleckstrin homology and RhoGEF domain containing G3 | 1,445 | Other | other |
| PLEKHG5 | ENSG00000171680 | pleckstrin homology and RhoGEF domain containing G5 | 0,871 | Cytoplasm | other |
| PLEKHG7 | ENSG00000187510 | pleckstrin homology and RhoGEF domain containing G7 | 1,347 | Other | other |
| PLEKHH3 | ENSG00000068137 | pleckstrin homology, MyTH4 and FERM domain containing H3 | 0,629 | Extracellular Space | other |
| PLEKHM2 | ENSG00000116786 | pleckstrin homology and RUN domain containing M2 | 0,901 | Extracellular Space | other |
| PLEKHO2 | ENSG00000241839 | pleckstrin homology domain containing O2 | 0,625 | Cytoplasm | other |
| PLK3 | ENSG00000173846 | polo like kinase 3 | 1,598 | Nucleus | kinase |
| PLPP3 | ENSG00000162407 | phospholipid phosphatase 3 | 1,653 | Plasma Membrane | phosphatase |
| PLS1 | ENSG00000120756 | plastin 1 | -0,717 | Plasma Membrane | other |
| PLS3 | ENSG00000102024 | plastin 3 | -1,336 | Cytoplasm | other |
| PLSCR1 | ENSG00000188313 | phospholipid scramblase 1 | 0,872 | Plasma Membrane | enzyme |
| PLXNA2 | ENSG00000076356 | plexin A2 | 0,854 | Plasma Membrane | transmembrane receptor |
| PLXNC1 | ENSG00000136040 | plexin C1 | 0,709 | Plasma Membrane | transmembrane receptor |
| PMAIP1 | ENSG00000141682 | phorbol-12-myristate-13-acetate-induced protein 1 | 3,130 | Cytoplasm | other |
| PML | ENSG00000140464 | promyelocytic leukemia | 1,920 | Nucleus | transcription regulator |
| PMVK | ENSG00000163344 | phosphomevalonate kinase | -0,659 | Cytoplasm | kinase |
| PNPLA3 | ENSG00000100344 | patatin like phospholipase domain containing 3 | -0,673 | Cytoplasm | enzyme |
| PNPT1 | ENSG00000138035 | polyribonucleotide nucleotidyltransferase 1 | 1,897 | Cytoplasm | enzyme |
| PNRC1 | ENSG00000146278 | proline rich nuclear receptor coactivator 1 | 1,810 | Nucleus | other |
| POGZ | ENSG00000143442 | pogo transposable element derived with ZNF domain | 0,863 | Nucleus | enzyme |
| POLD4 | ENSG00000175482 | DNA polymerase delta 4, accessory subunit | -0,659 | Nucleus | enzyme |
| POLR2J | ENSG00000005075 | RNA polymerase II subunit J | -0,750 | Nucleus | enzyme |
| POLR3C | ENSG00000186141 | RNA polymerase III subunit C | 0,633 | Nucleus | enzyme |
| POU2F2 | ENSG00000028277 | POU class 2 homeobox 2 | 1,342 | Nucleus | transcription regulator |
| POU2F3 | ENSG00000137709 | POU class 2 homeobox 3 | 1,393 | Nucleus | transcription regulator |
| PPARGC1A | ENSG00000109819 | PPARG coactivator 1 alpha | 1,827 | Nucleus | transcription regulator |
| PPFIA1 | ENSG00000131626 | PTPRF interacting protein alpha 1 | 0,785 | Plasma Membrane | phosphatase |
| PPIL4 | ENSG00000131013 | peptidylprolyl isomerase like 4 | 0,768 | Nucleus | enzyme |
| PPM1D | ENSG00000170836 | protein phosphatase, Mg2+/Mn2+ dependent 1D | 0,933 | Cytoplasm | phosphatase |
| PPM1K | ENSG00000163644 | protein phosphatase, Mg2+/Mn2+ dependent 1K | 3,119 | Cytoplasm | phosphatase |
| PPOX | ENSG00000143224 | protoporphyrinogen oxidase | -0,676 | Cytoplasm | enzyme |
| PPP1R12A | ENSG00000058272 | protein phosphatase 1 regulatory subunit 12A | 0,804 | Cytoplasm | phosphatase |
| PPP1R15A | ENSG00000087074 | protein phosphatase 1 regulatory subunit 15A | 5,108 | Cytoplasm | other |

| | | | | | |
|---|---|---|---|---|---|
| PPP1R15B | ENSG00000158615 | protein phosphatase 1 regulatory subunit 15B | 1,709 | Cytoplasm | phosphatase |
| PPP1R18 | ENSG00000146112 | protein phosphatase 1 regulatory subunit 18 | 1,004 | Other | other |
| PPP1R7 | ENSG00000115685 | protein phosphatase 1 regulatory subunit 7 | -0,641 | Nucleus | phosphatase |
| PPP2R2A | ENSG00000221914 | protein phosphatase 2 regulatory subunit Balpha | 0,770 | Cytoplasm | phosphatase |
| PPP2R2D | ENSG00000175470 | protein phosphatase 2 regulatory subunit Bdelta | 0,869 | Nucleus | other |
| PPP3CC | ENSG00000120910 | protein phosphatase 3 catalytic subunit gamma | 1,309 | Cytoplasm | phosphatase |
| PPP4R1L | ENSG00000124224 | protein phosphatase 4 regulatory subunit 1 like (pseudogene) | 1,201 | Other | other |
| PPP4R2 | ENSG00000163605 | protein phosphatase 4 regulatory subunit 2 | 0,816 | Cytoplasm | other |
| PPP4R4 | ENSG00000119698 | protein phosphatase 4 regulatory subunit 4 | 3,223 | Cytoplasm | other |
| PPP6C | ENSG00000119414 | protein phosphatase 6 catalytic subunit | 0,656 | Nucleus | phosphatase |
| PPRC1 | ENSG00000148840 | PPARG related coactivator 1 | 0,630 | Nucleus | transcription regulator |
| PPT2 | ENSG00000168452 | palmitoyl-protein thioesterase 2 | 0,879 | Cytoplasm | enzyme |
| PPTC7 | ENSG00000196850 | protein phosphatase targeting COQ7 | 0,884 | Cytoplasm | phosphatase |
| PRADC1 | ENSG00000135617 | protease associated domain containing 1 | -0,997 | Extracellular Space | other |
| PRDM11 | ENSG00000019485 | PR/SET domain 11 | 0,685 | Other | other |
| PRDM4 | ENSG00000110851 | PR/SET domain 4 | 1,009 | Nucleus | transcription regulator |
| PRDX2 | ENSG00000167815 | peroxiredoxin 2 | -0,607 | Cytoplasm | enzyme |
| PREX1 | ENSG00000124126 | phosphatidylinositol-3,4,5-trisphosphate dependent Rac exchange factor 1 | 0,876 | Cytoplasm | other |
| PRKACB | ENSG00000142875 | protein kinase cAMP-activated catalytic subunit beta | -0,794 | Cytoplasm | kinase |
| PRKAG1 | ENSG00000181929 | protein kinase AMP-activated non-catalytic subunit gamma 1 | -0,627 | Nucleus | kinase |
| PRKCE | ENSG00000171132 | protein kinase C epsilon | 0,822 | Cytoplasm | kinase |
| PRKD2 | ENSG00000105287 | protein kinase D2 | 1,927 | Cytoplasm | kinase |
| PROM2 | ENSG00000155066 | prominin 2 | -1,008 | Plasma Membrane | transmembrane receptor |
| PROSER2 | ENSG00000148426 | proline and serine rich 2 | 0,820 | Other | other |
| PROX1 | ENSG00000117707 | prospero homeobox 1 | 1,044 | Nucleus | transcription regulator |
| PRPF39 | ENSG00000185246 | pre-mRNA processing factor 39 | 0,620 | Nucleus | other |
| PRR11 | ENSG00000068489 | proline rich 11 | -0,627 | Other | other |
| PRR15 | ENSG00000176532 | proline rich 15 | 0,934 | Other | other |
| PRR15L | ENSG00000167183 | proline rich 15 like | -0,759 | Other | other |
| PRR3 | ENSG00000233564 | proline rich 3 | 1,009 | Other | other |
| PRRC2C | ENSG00000117523 | proline rich coiled-coil 2C | 0,664 | Cytoplasm | other |
| PRRT3 | ENSG00000163704 | proline rich transmembrane protein 3 | -0,623 | Other | other |
| PRSS16 | ENSG00000112812 | serine protease 16 | -0,614 | Extracellular Space | peptidase |
| PRSS33 | ENSG00000103355 | serine protease 33 | -0,954 | Extracellular Space | peptidase |
| PRTFDC1 | ENSG00000099256 | phosphoribosyl transferase domain containing 1 | -0,601 | Cytoplasm | enzyme |
| PRX | ENSG00000105227 | periaxin | -0,676 | Nucleus | other |
| PSG4 | ENSG00000243137 | pregnancy specific beta-1-glycoprotein 4 | 1,271 | Extracellular Space | other |
| PSMB8 | ENSG00000236443 | proteasome 20S subunit beta 8 | 1,940 | Cytoplasm | peptidase |
| PSMB9 | ENSG00000243958 | proteasome 20S subunit beta 9 | 2,246 | Cytoplasm | peptidase |
| PSMC1P5 | ENSG00000250273 | | -4,013 | Other | other |
| PSME2 | ENSG00000100911 | proteasome activator subunit 2 | 0,729 | Cytoplasm | peptidase |
| PTBP2 | ENSG00000117569 | polypyrimidine tract binding protein 2 | 1,041 | Nucleus | other |
| PTGER2 | ENSG00000125384 | prostaglandin E receptor 2 | 1,710 | Plasma Membrane | G-protein coupled receptor |
| PTGER4 | ENSG00000171522 | prostaglandin E receptor 4 | 2,306 | Plasma Membrane | G-protein coupled receptor |
| PTGS2 | ENSG00000073756 | prostaglandin-endoperoxide synthase 2 | 2,178 | Cytoplasm | enzyme |
| PTMS | ENSG00000159335 | parathymosin | -0,690 | Nucleus | other |
| PTP4A1 | ENSG00000112245 | protein tyrosine phosphatase 4A1 | 1,017 | Cytoplasm | phosphatase |
| PTPN14 | ENSG00000152104 | protein tyrosine phosphatase non-receptor type 14 | 0,826 | Cytoplasm | phosphatase |
| PTPN4 | ENSG00000088179 | protein tyrosine phosphatase non-receptor type 4 | 0,704 | Cytoplasm | phosphatase |
| PTPRZ1 | ENSG00000106278 | protein tyrosine phosphatase receptor type Z1 | 1,580 | Plasma Membrane | phosphatase |
| PTRHD1 | ENSG00000184924 | peptidyl-tRNA hydrolase domain containing 1 | -0,856 | Cytoplasm | other |
| PTTG1 | ENSG00000164611 | PTTG1 regulator of sister chromatid separation, securin | -0,623 | Nucleus | transcription regulator |
| PTX3 | ENSG00000163661 | pentraxin 3 | 5,124 | Extracellular Space | other |
| PVR | ENSG00000073008 | PVR cell adhesion molecule | 0,617 | Plasma Membrane | other |
| PWWP3B | ENSG00000157502 | PWWP domain containing 3B | 0,721 | Cytoplasm | other |
| PXK | ENSG00000168297 | PX domain containing serine/threonine kinase like | 0,693 | Cytoplasm | kinase |
| PXMP4 | ENSG00000101417 | peroxisomal membrane protein 4 | -0,742 | Cytoplasm | other |
| QKI | ENSG00000112531 | QKI, KH domain containing RNA binding | 1,061 | Nucleus | other |
| QPCTL | ENSG00000011478 | glutaminyl-peptide cyclotransferase like | -0,662 | Cytoplasm | enzyme |
| RAB19 | ENSG00000146955 | RAB19, member RAS oncogene family | 1,379 | Cytoplasm | enzyme |
| RAB24 | ENSG00000169228 | RAB24, member RAS oncogene family | 1,493 | Cytoplasm | other |
| RAB26 | ENSG00000167964 | RAB26, member RAS oncogene family | -1,026 | Plasma Membrane | enzyme |
| RAB30 | ENSG00000137502 | RAB30, member RAS oncogene family | 2,856 | Cytoplasm | enzyme |
| RAB3GAP2 | ENSG00000118873 | RAB3 GTPase activating non-catalytic protein subunit 2 | 0,876 | Cytoplasm | enzyme |
| RAB3IP | ENSG00000127328 | RAB3A interacting protein | 0,787 | Cytoplasm | other |
| RAB40B | ENSG00000141542 | RAB40B, member RAS oncogene family | -0,910 | Plasma Membrane | enzyme |
| RAB4A | ENSG00000168118 | RAB4A, member RAS oncogene family | -0,752 | Cytoplasm | enzyme |
| RAB8B | ENSG00000166128 | RAB8B, member RAS oncogene family | 1,086 | Cytoplasm | enzyme |
| RAB9A | ENSG00000123595 | RAB9A, member RAS oncogene family | 0,988 | Cytoplasm | enzyme |
| RABGAP1L | ENSG00000152061 | RAB GTPase activating protein 1 like | 0,816 | Cytoplasm | other |
| RAD52 | ENSG00000002016 | RAD52 homolog, DNA repair protein | 1,195 | Nucleus | other |
| RAD54L2 | ENSG00000164080 | RAD54 like 2 | 0,848 | Nucleus | transcription regulator |
| RAET1L | ENSG00000155918 | retinoic acid early transcript 1L | 2,776 | Plasma Membrane | other |
| RALGAPA1 | ENSG00000174373 | Ral GTPase activating protein catalytic subunit alpha 1 | 1,306 | Cytoplasm | other |
| RALGAPA1P1 | ENSG00000229419 | RALGAPA1 pseudogene 1 | 0,856 | Other | other |
| RANBP10 | ENSG00000141084 | RAN binding protein 10 | 0,667 | Cytoplasm | other |
| RAPGEF2 | ENSG00000109756 | Rap guanine nucleotide exchange factor 2 | 0,728 | Cytoplasm | other |
| RAPGEF6 | ENSG00000158987 | Rap guanine nucleotide exchange factor 6 | 0,848 | Plasma Membrane | other |
| RARA | ENSG00000131759 | retinoic acid receptor alpha | 0,695 | Nucleus | ligand-dependent nuclear receptor |
| RARRES1 | ENSG00000118849 | retinoic acid receptor responder 1 | -0,697 | Plasma Membrane | other |
| RASAL2 | ENSG00000075391 | RAS protein activator like 2 | 0,971 | Extracellular Space | other |
| RASEF | ENSG00000165105 | RAS and EF-hand domain containing | 0,683 | Cytoplasm | other |
| RASGEF1B | ENSG00000138670 | RasGEF domain family member 1B | 2,648 | Other | other |
| RASGRF2 | ENSG00000113319 | Ras protein specific guanine nucleotide releasing factor 2 | 1,316 | Cytoplasm | other |
| RASGRP1 | ENSG00000172575 | RAS guanyl releasing protein 1 | 1,917 | Cytoplasm | other |
| RASGRP3 | ENSG00000152689 | RAS guanyl releasing protein 3 | 2,863 | Cytoplasm | other |
| RASSF10 | ENSG00000189431 | Ras association domain family member 10 | 0,747 | Other | other |
| RASSF6 | ENSG00000169435 | Ras association domain family member 6 | 0,840 | Other | other |
| RASSF9 | ENSG00000198774 | Ras association domain family member 9 | 1,134 | Cytoplasm | transporter |
| RBBP6 | ENSG00000122257 | RB binding protein 6, ubiquitin ligase | 0,868 | Nucleus | enzyme |
| RBBP8 | ENSG00000101773 | RB binding protein 8, endonuclease | 0,622 | Nucleus | enzyme |
| RBCK1 | ENSG00000125826 | RANBP2-type and C3HC4-type zinc finger containing 1 | 0,728 | Cytoplasm | transcription regulator |
| RBL2 | ENSG00000103479 | RB transcriptional corepressor like 2 | -0,649 | Nucleus | other |
| RBM11 | ENSG00000185272 | RNA binding motif protein 11 | 0,825 | Nucleus | other |
| RBM14-RBM4 | ENSG00000248643 | RBM14-RBM4 readthrough | 0,865 | Other | other |
| RBM15 | ENSG00000162775 | RNA binding motif protein 15 | 0,705 | Nucleus | other |
| RBM25 | ENSG00000119707 | RNA binding motif protein 25 | 0,808 | Nucleus | other |
| RBM26 | ENSG00000139746 | RNA binding motif protein 26 | 0,617 | Other | other |
| RBM27 | ENSG00000091009 | RNA binding motif protein 27 | 0,714 | Nucleus | other |

| | | | | | |
|---|---|---|---|---|---|
| RBM33 | ENSG00000184863 | RNA binding motif protein 33 | 0,656 | Other | other |
| RBM39 | ENSG00000131051 | RNA binding motif protein 39 | 0,894 | Nucleus | transcription regulator |
| RBM43 | ENSG00000184898 | RNA binding motif protein 43 | 1,035 | Other | other |
| RBM48 | ENSG00000127993 | RNA binding motif protein 48 | 0,814 | Nucleus | other |
| RBM5 | ENSG00000003756 | RNA binding motif protein 5 | 1,116 | Nucleus | other |
| RBM7 | ENSG00000076053 | RNA binding motif protein 7 | 0,800 | Nucleus | other |
| RBMS2 | ENSG00000076067 | RNA binding motif single stranded interacting protein 2 | 0,825 | Nucleus | other |
| RC3H1 | ENSG00000135870 | ring finger and CCCH-type domains 1 | 1,171 | Cytoplasm | enzyme |
| RC3H2 | ENSG00000056586 | ring finger and CCCH-type domains 2 | 0,662 | Plasma Membrane | enzyme |
| RCAN1 | ENSG00000159200 | regulator of calcineurin 1 | 1,451 | Nucleus | transcription regulator |
| RDH5 | ENSG00000135437 | retinol dehydrogenase 5 | -0,959 | Cytoplasm | enzyme |
| REC8 | ENSG00000100918 | REC8 meiotic recombination protein | 1,326 | Nucleus | other |
| REEP6 | ENSG00000115255 | receptor accessory protein 6 | -0,741 | Plasma Membrane | other |
| REL | ENSG00000162924 | REL proto-oncogene, NF-kB subunit | 2,820 | Nucleus | transcription regulator |
| RELB | ENSG00000104856 | RELB proto-oncogene, NF-kB subunit | 2,618 | Nucleus | transcription regulator |
| RERG | ENSG00000134533 | RAS like estrogen regulated growth inhibitor | -0,916 | Nucleus | enzyme |
| RESF1 | ENSG00000174718 | retroelement silencing factor 1 | 0,947 | Nucleus | other |
| REV3L | ENSG00000009413 | REV3 like, DNA directed polymerase zeta catalytic subunit | 1,247 | Nucleus | enzyme |
| REXO1 | ENSG00000079313 | RNA exonuclease 1 homolog | 1,024 | Nucleus | transcription regulator |
| RFFL | ENSG00000092871 | ring finger and FYVE like domain containing E3 ubiquitin protein ligase | 0,800 | Cytoplasm | enzyme |
| RFTN1 | ENSG00000131378 | raftlin, lipid raft linker 1 | 0,925 | Plasma Membrane | other |
| RFX7 | ENSG00000181827 | regulatory factor X7 | 0,651 | Other | other |
| RGCC | ENSG00000102760 | regulator of cell cycle | -1,083 | Cytoplasm | other |
| RGL1 | ENSG00000143344 | ral guanine nucleotide dissociation stimulator like 1 | 0,664 | Cytoplasm | other |
| RGMB | ENSG00000174136 | repulsive guidance molecule BMP co-receptor b | 1,236 | Plasma Membrane | other |
| RGPD3 (includes others) | ENSG00000169629 | RANBP2 like and GRIP domain containing 3 | 1,333 | Nucleus | enzyme |
| RHOV | ENSG00000104140 | ras homolog family member V | 0,892 | Plasma Membrane | enzyme |
| RIC1 | ENSG00000107036 | RIC1 homolog, RAB6A GEF complex partner 1 | 0,966 | Cytoplasm | other |
| RICTOR | ENSG00000164327 | RPTOR independent companion of MTOR complex 2 | 1,580 | Cytoplasm | other |
| RIMKLB | ENSG00000166532 | ribosomal modification protein rimK like family member B | 1,032 | Cytoplasm | enzyme |
| RING1 | ENSG00000226788 | ring finger protein 1 | 0,747 | Nucleus | transcription regulator |
| RINT1 | ENSG00000135249 | RAD50 interactor 1 | 0,724 | Nucleus | other |
| RIOK3 | ENSG00000101782 | RIO kinase 3 | 1,023 | Cytoplasm | kinase |
| RIPK1 | ENSG00000137275 | receptor interacting serine/threonine kinase 1 | 0,911 | Plasma Membrane | kinase |
| RIPK2 | ENSG00000104312 | receptor interacting serine/threonine kinase 2 | 2,290 | Plasma Membrane | kinase |
| RIPOR1 | ENSG00000039523 | RHO family interacting cell polarization regulator 1 | 0,758 | Cytoplasm | other |
| RIPOR2 | ENSG00000111913 | RHO family interacting cell polarization regulator 2 | 2,789 | Cytoplasm | other |
| RIPOR3 | ENSG00000042062 | RIPOR family member 3 | 1,449 | Other | other |
| RIT1 | ENSG00000143622 | Ras like without CAAX 1 | 0,637 | Plasma Membrane | enzyme |
| RLF | ENSG00000117000 | RLF zinc finger | 1,144 | Nucleus | transcription regulator |
| RLIM | ENSG00000131263 | ring finger protein, LIM domain interacting | 1,125 | Nucleus | enzyme |
| RMC1 | ENSG00000141452 | regulator of MON1-CCZ1 | 0,783 | Cytoplasm | other |
| RNASE10 | ENSG00000182545 | ribonuclease A family member 10 (inactive) | 1,130 | Extracellular Space | other |
| RNASE4 | ENSG00000258818 | ribonuclease A family member 4 | -0,815 | Extracellular Space | enzyme |
| RNASEH2B | ENSG00000136104 | ribonuclease H2 subunit B | -0,627 | Nucleus | enzyme |
| RNASEK | ENSG00000219200 | ribonuclease K | -0,658 | Other | peptidase |
| RNASET2 | ENSG00000026297 | ribonuclease T2 | -0,760 | Cytoplasm | enzyme |
| RND1 | ENSG00000172602 | Rho family GTPase 1 | 2,559 | Cytoplasm | enzyme |
| RND3 | ENSG00000115963 | Rho family GTPase 3 | 1,239 | Cytoplasm | enzyme |
| RNF103 | ENSG00000239305 | ring finger protein 103 | 0,776 | Cytoplasm | enzyme |
| RNF111 | ENSG00000157450 | ring finger protein 111 | 1,005 | Nucleus | enzyme |
| RNF139 | ENSG00000170881 | ring finger protein 139 | 0,704 | Cytoplasm | enzyme |
| RNF141 | ENSG00000110315 | ring finger protein 141 | -0,671 | Other | enzyme |
| RNF149 | ENSG00000163162 | ring finger protein 149 | 0,681 | Cytoplasm | enzyme |
| RNF169 | ENSG00000166439 | ring finger protein 169 | 0,710 | Nucleus | other |
| RNF19B | ENSG00000116514 | ring finger protein 19B | 1,621 | Cytoplasm | enzyme |
| RNF213 | ENSG00000173821 | ring finger protein 213 | 2,660 | Cytoplasm | enzyme |
| RNF223 | ENSG00000237330 | ring finger protein 223 | 0,689 | Other | other |
| RNF31 | ENSG00000092098 | ring finger protein 31 | 0,749 | Cytoplasm | enzyme |
| RNF41 | ENSG00000181852 | ring finger protein 41 | 0,731 | Cytoplasm | enzyme |
| RNF5 | ENSG00000228907 | ring finger protein 5 | -0,606 | Cytoplasm | enzyme |
| ROGDI | ENSG00000067836 | rogdi atypical leucine zipper | -0,718 | Nucleus | other |
| ROS1 | ENSG00000047936 | ROS proto-oncogene 1, receptor tyrosine kinase | -0,937 | Plasma Membrane | kinase |
| RP11-468E2.1 | ENSG00000254692 | | -0,618 | Other | other |
| RP11-468E2.4 | ENSG00000259529 | | 0,784 | Other | other |
| RP11-75L1.2 | ENSG00000213443 | | 1,246 | Other | other |
| RP11-864I4.1 | ENSG00000255508 | | -0,838 | Other | other |
| RP11_295K31 | ENSG00000250644 | | -1,420 | Other | other |
| RP11_298I35 | ENSG00000259132 | | -1,067 | Other | other |
| RP11_613M108 | ENSG00000256966 | | 0,899 | Other | other |
| RP1_34B204 | ENSG00000217275 | | 3,224 | Other | other |
| RP4_764D21 | ENSG00000235371 | | 1,449 | Other | other |
| RP5_1057I204 | ENSG00000268069 | | -1,125 | Other | other |
| RP9 | ENSG00000164610 | RP9 pre-mRNA splicing factor | 0,840 | Nucleus | other |
| RPL3P2 | ENSG00000227656 | ribosomal protein L3 pseudogene 2 | -1,071 | Other | other |
| RPP14 | ENSG00000163684 | ribonuclease P/MRP subunit p14 | 0,709 | Nucleus | enzyme |
| RPRD1B | ENSG00000101413 | regulation of nuclear pre-mRNA domain containing 1B | 0,614 | Nucleus | other |
| RPS27L | ENSG00000185088 | ribosomal protein S27 like | -0,698 | Cytoplasm | translation regulator |
| RPS6KC1 | ENSG00000136643 | ribosomal protein S6 kinase C1 | 1,021 | Cytoplasm | kinase |
| RPTOR | ENSG00000141564 | regulatory associated protein of MTOR complex 1 | 0,658 | Cytoplasm | other |
| RRAD | ENSG00000166592 | RRAD, Ras related glycolysis inhibitor and calcium channel regulator | 1,629 | Cytoplasm | enzyme |
| RRAGD | ENSG00000025039 | Ras related GTP binding D | 0,608 | Cytoplasm | enzyme |
| RRP12 | ENSG00000052749 | ribosomal RNA processing 12 homolog | 0,716 | Nucleus | other |
| RRP8 | ENSG00000132275 | ribosomal RNA processing 8 | 0,708 | Nucleus | enzyme |
| RSAD2 | ENSG00000134321 | radical S-adenosyl methionine domain containing 2 | 4,604 | Cytoplasm | enzyme |
| RSC1A1 | ENSG00000215695 | regulator of solute carriers 1 | 1,246 | Nucleus | other |
| RSF1 | ENSG00000048649 | remodeling and spacing factor 1 | 0,785 | Nucleus | transcription regulator |
| RSPRY1 | ENSG00000159579 | ring finger and SPRY domain containing 1 | 0,937 | Other | other |
| RSRC2 | ENSG00000111011 | arginine and serine rich coiled-coil 2 | 1,406 | Other | other |
| RTL8A | ENSG00000203950 | retrotransposon Gag like 8A | -0,659 | Other | other |
| RTL9 | ENSG00000243978 | retrotransposon Gag like 9 | 2,400 | Other | other |
| RTP4 | ENSG00000136514 | receptor transporter protein 4 | 3,133 | Plasma Membrane | other |
| RUBCN | ENSG00000145016 | rubicon autophagy regulator | 1,240 | Cytoplasm | other |
| RWDD3 | ENSG00000122481 | RWD domain containing 3 | -0,606 | Other | other |
| RXRB | ENSG00000235712 | retinoid X receptor beta | 2,072 | Nucleus | ligand-dependent nuclear receptor |
| RYBP | ENSG00000163602 | RING1 and YY1 binding protein | 0,736 | Nucleus | transcription regulator |
| S1PR2 | ENSG00000267534 | sphingosine-1-phosphate receptor 2 | 0,852 | Plasma Membrane | G-protein coupled receptor |
| SAA2 | ENSG00000134339 | serum amyloid A2 | 1,682 | Extracellular Space | other |
| SAMD4A | ENSG00000020577 | sterile alpha motif domain containing 4A | 1,657 | Cytoplasm | translation regulator |

| | | | | | |
|---|---|---|---|---|---|
| SAMD8 | ENSG00000156671 | sterile alpha motif domain containing 8 | 0,955 | Cytoplasm | enzyme |
| SAMD9 | ENSG00000205413 | sterile alpha motif domain containing 9 | 2,294 | Cytoplasm | other |
| SAMD9L | ENSG00000177409 | sterile alpha motif domain containing 9 like | 3,229 | Extracellular Space | other |
| SAMHD1 | ENSG00000101347 | SAM and HD domain containing deoxynucleoside triphosphate triphosphohydrolase 1 | 2,554 | Nucleus | enzyme |
| SAPCD2 | ENSG00000186193 | suppressor APC domain containing 2 | -0,606 | Nucleus | other |
| SAT1 | ENSG00000130066 | spermidine/spermine N1-acetyltransferase 1 | 1,232 | Cytoplasm | enzyme |
| SATB2 | ENSG00000119042 | SATB homeobox 2 | 1,216 | Nucleus | transcription regulator |
| SBNO2 | ENSG00000064932 | strawberry notch homolog 2 | 1,607 | Other | transcription regulator |
| SCAF4 | ENSG00000156304 | SR-related CTD associated factor 4 | 0,703 | Nucleus | other |
| SCAF8 | ENSG00000213079 | SR-related CTD associated factor 8 | 1,001 | Nucleus | other |
| SCAND1 | ENSG00000171222 | SCAN domain containing 1 | -0,623 | Nucleus | transcription regulator |
| SCD | ENSG00000099194 | stearoyl-CoA desaturase | -0,711 | Cytoplasm | enzyme |
| SCGN | ENSG00000079689 | secretagogin, EF-hand calcium binding protein | -1,337 | Cytoplasm | other |
| SCN3A | ENSG00000153253 | sodium voltage-gated channel alpha subunit 3 | 2,467 | Plasma Membrane | ion channel |
| SCRN2 | ENSG00000141295 | secernin 2 | -0,668 | Extracellular Space | other |
| SCRN3 | ENSG00000144306 | secernin 3 | -0,767 | Other | other |
| SCYL2 | ENSG00000136021 | SCY1 like pseudokinase 2 | 0,757 | Cytoplasm | other |
| SDC1 | ENSG00000115884 | syndecan 1 | -0,624 | Plasma Membrane | enzyme |
| SDC4 | ENSG00000124145 | syndecan 4 | 0,889 | Plasma Membrane | other |
| SDCBP2 | ENSG00000125775 | syndecan binding protein 2 | 0,656 | Cytoplasm | other |
| SDE2 | ENSG00000143751 | SDE2 telomere maintenance homolog | 0,809 | Nucleus | other |
| SDHB | ENSG00000117118 | succinate dehydrogenase complex iron sulfur subunit B | -0,643 | Cytoplasm | enzyme |
| SDSL | ENSG00000139410 | serine dehydratase like | 0,710 | Cytoplasm | enzyme |
| SEC14L2 | ENSG00000100003 | SEC14 like lipid binding 2 | -0,665 | Cytoplasm | transporter |
| SEC16B | ENSG00000120341 | SEC16 homolog B, endoplasmic reticulum export factor | 1,573 | Nucleus | other |
| SEC23B | ENSG00000101310 | SEC23 homolog B, COPII coat complex component | 0,775 | Extracellular Space | transporter |
| SEC24A | ENSG00000113615 | SEC24 homolog A, COPII coat complex component | 1,229 | Cytoplasm | transporter |
| SEC24D | ENSG00000150961 | SEC24 homolog D, COPII coat complex component | 0,650 | Cytoplasm | transporter |
| SECTM1 | ENSG00000141574 | secreted and transmembrane 1 | 1,945 | Extracellular Space | other |
| SELE | ENSG00000007908 | selectin E | 1,389 | Plasma Membrane | transmembrane receptor |
| SEMA3A | ENSG00000075213 | semaphorin 3A | 1,766 | Extracellular Space | other |
| SEMA3D | ENSG00000153993 | semaphorin 3D | 1,574 | Extracellular Space | other |
| SEMA3E | ENSG00000170381 | semaphorin 3E | -0,621 | Extracellular Space | other |
| SEMA4F | ENSG00000135622 | ssemaphorin 4F | -0,694 | Plasma Membrane | other |
| SEMA7A | ENSG00000138623 | semaphorin 7A (John Milton Hagen blood group) | 1,389 | Plasma Membrane | transmembrane receptor |
| SENP6 | ENSG00000112701 | SUMO specific peptidase 6 | 0,639 | Cytoplasm | peptidase |
| SEPSECS | ENSG00000109618 | Sep (O-phosphoserine) tRNA:Sec (selenocysteine) tRNA synthase | 1,428 | Cytoplasm | enzyme |
| SERGEF | ENSG00000129158 | secretion regulating guanine nucleotide exchange factor | -0,890 | Nucleus | other |
| SERPINA10 | ENSG00000140093 | serpin family A member 10 | 2,005 | Extracellular Space | other |
| SERPINA5 | ENSG00000188488 | serpin family A member 5 | -0,712 | Extracellular Space | other |
| SERPINA6 | ENSG00000170099 | serpin family A member 6 | 1,621 | Extracellular Space | other |
| SERPINB2 | ENSG00000197632 | serpin family B member 2 | 1,349 | Extracellular Space | other |
| SERPINB9 | ENSG00000170542 | serpin family B member 9 | 1,642 | Cytoplasm | other |
| SERTAD1 | ENSG00000197019 | SERTA domain containing 1 | 1,136 | Nucleus | transcription regulator |
| SERTAD2 | ENSG00000179833 | SERTA domain containing 2 | 1,812 | Cytoplasm | transcription regulator |
| SERTAD3 | ENSG00000167565 | SERTA domain containing 3 | 0,706 | Nucleus | transcription regulator |
| SESN2 | ENSG00000130766 | sestrin 2 | 1,506 | Cytoplasm | enzyme |
| SETD1B | ENSG00000139718 | SET domain containing 1B, histone lysine methyltransferase | 0,704 | Nucleus | enzyme |
| SETD2 | ENSG00000181555 | SET domain containing 2, histone lysine methyltransferase | 0,975 | Cytoplasm | enzyme |
| SETD5 | ENSG00000168137 | SET domain containing 5 | 1,158 | Nucleus | enzyme |
| SETDB1 | ENSG00000143379 | SET domain bifurcated histone lysine methyltransferase 1 | 0,956 | Nucleus | enzyme |
| SETX | ENSG00000107290 | senataxin | 0,608 | Nucleus | enzyme |
| SFSWAP | ENSG00000061936 | splicing factor SWAP | 0,790 | Nucleus | other |
| SFTA2 | ENSG00000196260 | surfactant associated 2 | -0,888 | Other | other |
| SFXN3 | ENSG00000107819 | sideroflexin 3 | -0,699 | Cytoplasm | transporter |
| SGK1 | ENSG00000118515 | serum/glucocorticoid regulated kinase 1 | 0,713 | Cytoplasm | kinase |
| SGMS2 | ENSG00000164023 | sphingomyelin synthase 2 | 0,728 | Plasma Membrane | enzyme |
| SH2B3 | ENSG00000111252 | SH2B adaptor protein 3 | 1,063 | Plasma Membrane | other |
| SH3D21 | ENSG00000214193 | SH3 domain containing 21 | -0,662 | Cytoplasm | other |
| SH3RF1 | ENSG00000154447 | SH3 domain containing ring finger 1 | 0,763 | Cytoplasm | enzyme |
| SH3TC1 | ENSG00000125089 | SH3 domain and tetratricopeptide repeats 1 | 2,077 | Extracellular Space | other |
| SH3YL1 | ENSG00000035115 | SH3 and SYLF domain containing 1 | -0,731 | Plasma Membrane | other |
| SHFL | ENSG00000130813 | shiftless antiviral inhibitor of ribosomal frameshifting | 2,243 | Other | other |
| SHOX2 | ENSG00000168779 | short stature homeobox 2 | 0,666 | Nucleus | transcription regulator |
| SHROOM2 | ENSG00000146950 | shroom family member 2 | 0,927 | Plasma Membrane | ion channel |
| SIK1/SIK1B | ENSG00000142178 | salt inducible kinase 1 | 0,918 | Nucleus | kinase |
| SIK3 | ENSG00000160584 | SIK family kinase 3 | 1,003 | Cytoplasm | kinase |
| SIM2 | ENSG00000159263 | SIM bHLH transcription factor 2 | 1,272 | Nucleus | transcription regulator |
| SIPA1L1 | ENSG00000197555 | signal induced proliferation associated 1 like 1 | 0,683 | Cytoplasm | other |
| SIPA1L2 | ENSG00000116991 | signal induced proliferation associated 1 like 2 | 2,667 | Other | other |
| SIRT1 | ENSG00000096717 | sirtuin 1 | 0,941 | Nucleus | transcription regulator |
| SIX4 | ENSG00000100625 | SIX homeobox 4 | 1,716 | Nucleus | transcription regulator |
| SKIL | ENSG00000136603 | SKI like proto-oncogene | 0,860 | Nucleus | transcription regulator |
| SKIV2L | ENSG00000223493 | Ski2 like RNA helicase | 0,790 | Nucleus | enzyme |
| SLAIN1 | ENSG00000139737 | SLAIN motif family member 1 | -0,683 | Other | other |
| SLC12A7 | ENSG00000113504 | solute carrier family 12 member 7 | 1,328 | Plasma Membrane | transporter |
| SLC15A3 | ENSG00000110446 | solute carrier family 15 member 3 | 4,134 | Cytoplasm | transporter |
| SLC16A6 | ENSG00000108932 | solute carrier family 16 member 6 | 1,099 | Plasma Membrane | transporter |
| SLC16A9 | ENSG00000165449 | solute carrier family 16 member 9 | 1,718 | Other | other |
| SLC19A2 | ENSG00000117479 | solute carrier family 19 member 2 | 0,946 | Plasma Membrane | transporter |
| SLC1A3 | ENSG00000079215 | solute carrier family 1 member 3 | 2,495 | Plasma Membrane | transporter |
| SLC20A1 | ENSG00000144136 | solute carrier family 20 member 1 | 0,901 | Plasma Membrane | transporter |
| SLC22A1 | ENSG00000175003 | solute carrier family 22 member 1 | 1,406 | Plasma Membrane | transporter |
| SLC25A1 | ENSG00000100075 | solute carrier family 25 member 1 | -0,618 | Other | transporter |
| SLC25A25 | ENSG00000148339 | solute carrier family 25 member 25 | 1,937 | Cytoplasm | transporter |
| SLC25A28 | ENSG00000155287 | solute carrier family 25 member 28 | 1,699 | Cytoplasm | transporter |
| SLC25A30 | ENSG00000174032 | solute carrier family 25 member 30 | 1,167 | Cytoplasm | other |
| SLC25A32 | ENSG00000164933 | solute carrier family 25 member 32 | 0,722 | Cytoplasm | transporter |
| SLC25A33 | ENSG00000171612 | solute carrier family 25 member 33 | 0,738 | Cytoplasm | transporter |
| SLC25A45 | ENSG00000162241 | solute carrier family 25 member 45 | 0,874 | Cytoplasm | transporter |
| SLC25A51 | ENSG00000122696 | solute carrier family 25 member 51 | 1,323 | Cytoplasm | other |
| SLC27A3 | ENSG00000143554 | solute carrier family 27 member 3 | -0,675 | Cytoplasm | transporter |
| SLC28A3 | ENSG00000197506 | solute carrier family 28 member 3 | 1,059 | Plasma Membrane | transporter |
| SLC2A6 | ENSG00000160326 | solute carrier family 2 member 6 | 1,323 | Plasma Membrane | transporter |
| SLC30A7 | ENSG00000162695 | solute carrier family 30 member 7 | 0,680 | Cytoplasm | transporter |
| SLC31A2 | ENSG00000136867 | solute carrier family 31 member 2 | 1,541 | Plasma Membrane | transporter |
| SLC35G2 | ENSG00000168917 | solute carrier family 35 member G2 | 0,826 | Cytoplasm | other |
| SLC3A1 | ENSG00000138079 | solute carrier family 3 member 1 | -1,183 | Plasma Membrane | transporter |
| SLC3A2 | ENSG00000168003 | solute carrier family 3 member 2 | 1,206 | Plasma Membrane | transporter |

| | | | | | |
|---|---|---|---|---|---|
| SLC41A2 | ENSG00000136052 | solute carrier family 41 member 2 | 0,763 | Plasma Membrane | transporter |
| SLC43A2 | ENSG00000167703 | solute carrier family 43 member 2 | 1,096 | Plasma Membrane | transporter |
| SLC44A3 | ENSG00000143036 | solute carrier family 44 member 3 | -0,656 | Plasma Membrane | transporter |
| SLC44A4 | ENSG00000204385 | solute carrier family 44 member 4 | -0,859 | Plasma Membrane | transporter |
| SLC5A5 | ENSG00000105641 | solute carrier family 5 member 5 | 0,829 | Plasma Membrane | transporter |
| SLC66A3 | ENSG00000162976 | solute carrier family 66 member 3 | -0,618 | Other | other |
| SLC7A5P1 | ENSG00000260727 | solute carrier family 7 member 5 pseudogene 1 | 0,912 | Other | other |
| SLC8A2 | ENSG00000118160 | solute carrier family 8 member A2 | 0,940 | Cytoplasm | transporter |
| SLC8B1 | ENSG00000089060 | solute carrier family 8 member B1 | 1,315 | Plasma Membrane | transporter |
| SLC9A3R2 | ENSG00000065054 | SLC9A3 regulator 2 | -0,635 | Plasma Membrane | transporter |
| SLC9A8 | ENSG00000197818 | solute carrier family 9 member A8 | 0,720 | Cytoplasm | transporter |
| SLCO3A1 | ENSG00000176463 | solute carrier organic anion transporter family member 3A1 | 0,733 | Plasma Membrane | transporter |
| SLCO5A1 | ENSG00000137571 | solute carrier organic anion transporter family member 5A1 | 1,087 | Plasma Membrane | transporter |
| SLFN5 | ENSG00000166750 | schlafen family member 5 | 2,308 | Nucleus | enzyme |
| SLIT2 | ENSG00000145147 | slit guidance ligand 2 | 0,980 | Extracellular Space | other |
| SLMAP | ENSG00000163681 | sarcolemma associated protein | 0,764 | Plasma Membrane | other |
| SLTM | ENSG00000137776 | SAFB like transcription modulator | 0,658 | Nucleus | other |
| SLU7 | ENSG00000164609 | SLU7 homolog, splicing factor | 0,602 | Nucleus | enzyme |
| SMAD7 | ENSG00000101665 | SMAD family member 7 | 1,550 | Nucleus | transcription regulator |
| SMARCA2 | ENSG00000080503 | SWI/SNF related, matrix associated, actin dependent regulator of chromatin, subfamily a, member 2 | -0,632 | Nucleus | transcription regulator |
| SMARCA5 | ENSG00000153147 | SWI/SNF related, matrix associated, actin dependent regulator of chromatin, subfamily a, member 5 | 0,608 | Nucleus | transcription regulator |
| SMCHD1 | ENSG00000101596 | structural maintenance of chromosomes flexible hinge domain containing 1 | 1,614 | Nucleus | enzyme |
| SMDT1 | ENSG00000272901 | single-pass membrane protein with aspartate rich tail 1 | -0,687 | Extracellular Space | other |
| SMG1 | ENSG00000157106 | SMG1 nonsense mediated mRNA decay associated PI3K related kinase | 0,878 | Cytoplasm | kinase |
| SMG1P5 | ENSG00000183604 | SMG1 pseudogene 5 | 0,918 | Other | other |
| SMG7 | ENSG00000116698 | SMG7 nonsense mediated mRNA decay factor | 0,717 | Nucleus | other |
| SMIM11A | ENSG00000205670 | small integral membrane protein 11A | -0,661 | Plasma Membrane | other |
| SMIM14 | ENSG00000163683 | small integral membrane protein 14 | 0,754 | Cytoplasm | other |
| SMIM20 | ENSG00000250317 | small integral membrane protein 20 | -0,693 | Cytoplasm | other |
| SMIM30 | ENSG00000214194 | small integral membrane protein 30 | -1,791 | Other | other |
| SMPDL3B | ENSG00000130768 | sphingomyelin phosphodiesterase acid like 3B | -0,734 | Extracellular Space | enzyme |
| SMTNL1 | ENSG00000214872 | smoothelin like 1 | 1,396 | Cytoplasm | other |
| SMURF1 | ENSG00000198742 | SMAD specific E3 ubiquitin protein ligase 1 | 1,169 | Cytoplasm | enzyme |
| SNAI1 | ENSG00000124216 | snail family transcriptional repressor 1 | 1,031 | Nucleus | transcription regulator |
| SNAPC4 | ENSG00000165684 | small nuclear RNA activating complex polypeptide 4 | 0,870 | Nucleus | transcription regulator |
| SNCA | ENSG00000145335 | synuclein alpha | -0,932 | Cytoplasm | enzyme |
| SNIP1 | ENSG00000163877 | Smad nuclear interacting protein 1 | 1,379 | Nucleus | other |
| SNPH | ENSG00000101298 | syntaphilin | 2,135 | Plasma Membrane | other |
| SNRK | ENSG00000163788 | SNF related kinase | 0,980 | Cytoplasm | kinase |
| SNX16 | ENSG00000104497 | sorting nexin 16 | 1,629 | Cytoplasm | transporter |
| SOCS1 | ENSG00000185338 | suppressor of cytokine signaling 1 | 2,294 | Cytoplasm | other |
| SOCS2 | ENSG00000120833 | suppressor of cytokine signaling 2 | 1,191 | Cytoplasm | other |
| SOCS3 | ENSG00000184557 | suppressor of cytokine signaling 3 | 1,390 | Cytoplasm | phosphatase |
| SOD2 | ENSG00000112096 | superoxide dismutase 2 | 2,018 | Cytoplasm | enzyme |
| SORBS2 | ENSG00000154556 | sorbin and SH3 domain containing 2 | -0,738 | Plasma Membrane | other |
| SORL1 | ENSG00000137642 | sortilin related receptor 1 | -0,610 | Cytoplasm | transporter |
| SOSTDC1 | ENSG00000171243 | sclerostin domain containing 1 | -1,204 | Extracellular Space | growth factor |
| SOX4 | ENSG00000124766 | SRY-box transcription factor 4 | 0,890 | Nucleus | transcription regulator |
| SOX6 | ENSG00000110693 | SRY-box transcription factor 6 | 1,455 | Nucleus | transcription regulator |
| SOX9 | ENSG00000125398 | SRY-box transcription factor 9 | 0,754 | Nucleus | transcription regulator |
| SP100 | ENSG00000067066 | SP100 nuclear antigen | 1,717 | Nucleus | transcription regulator |
| SP110 | ENSG00000135899 | SP110 nuclear body protein | 2,131 | Nucleus | transcription regulator |
| SP140L | ENSG00000185404 | SP140 nuclear body protein like | 1,433 | Other | other |
| SP2 | ENSG00000167182 | Sp2 transcription factor | 0,648 | Nucleus | transcription regulator |
| SPA17 | ENSG00000064199 | sperm autoantigenic protein 17 | -1,051 | Plasma Membrane | other |
| SPAG1 | ENSG00000104450 | sperm associated antigen 1 | 0,895 | Cytoplasm | other |
| SPAG16 | ENSG00000144451 | sperm associated antigen 16 | -0,909 | Cytoplasm | other |
| SPAG9 | ENSG00000008294 | sperm associated antigen 9 | 1,147 | Cytoplasm | other |
| SPATA13 | ENSG00000182957 | spermatogenesis associated 13 | 0,632 | Plasma Membrane | other |
| SPATA2 | ENSG00000158480 | spermatogenesis associated 2 | 1,180 | Extracellular Space | other |
| SPC24 | ENSG00000161888 | SPC24 component of NDC80 kinetochore complex | -0,811 | Cytoplasm | other |
| SPCS2 | ENSG00000118363 | signal peptidase complex subunit 2 | -0,682 | Cytoplasm | other |
| SPDEF | ENSG00000124664 | SAM pointed domain containing ETS transcription factor | -1,174 | Nucleus | transcription regulator |
| SPEN | ENSG00000065526 | spen family transcriptional repressor | 0,864 | Nucleus | transcription regulator |
| SPHK1 | ENSG00000176170 | sphingosine kinase 1 | 1,338 | Cytoplasm | kinase |
| SPINK1 | ENSG00000164266 | serine peptidase inhibitor Kazal type 1 | -0,663 | Extracellular Space | other |
| SPINK9 | ENSG00000204909 | serine peptidase inhibitor Kazal type 9 | 1,074 | Extracellular Space | other |
| SPOCK2 | ENSG00000107742 | SPARC (osteonectin), cwcv and kazal like domains proteoglycan 2 | -0,755 | Extracellular Space | other |
| SPP1 | ENSG00000118785 | secreted phosphoprotein 1 | -0,687 | Extracellular Space | cytokine |
| SPRED1 | ENSG00000166068 | sprouty related EVH1 domain containing 1 | 0,601 | Plasma Membrane | other |
| SPRY2 | ENSG00000136158 | sprouty RTK signaling antagonist 2 | 1,933 | Plasma Membrane | other |
| SPRY4 | ENSG00000187678 | sprouty RTK signaling antagonist 4 | 0,968 | Plasma Membrane | other |
| SPSB1 | ENSG00000171621 | splA/ryanodine receptor domain and SOCS box containing 1 | 0,817 | Cytoplasm | other |
| SPSB3 | ENSG00000162032 | splA/ryanodine receptor domain and SOCS box containing 3 | -0,616 | Cytoplasm | other |
| SPTA1 | ENSG00000163554 | spectrin alpha, erythrocytic 1 | 0,763 | Cytoplasm | other |
| SPTB | ENSG00000070182 | spectrin beta, erythrocytic | 1,394 | Plasma Membrane | other |
| SPTY2D1 | ENSG00000179119 | SPT2 chromatin protein domain containing 1 | 0,609 | Nucleus | other |
| SQSTM1 | ENSG00000161011 | sequestosome 1 | 1,173 | Cytoplasm | transcription regulator |
| SRCAP | ENSG00000080603 | Snf2 related CREBBP activator protein | 0,807 | Cytoplasm | transcription regulator |
| SREK1 | ENSG00000153914 | splicing regulatory glutamic acid and lysine rich protein 1 | 0,808 | Nucleus | other |
| SRFBP1 | ENSG00000151304 | serum response factor binding protein 1 | 0,888 | Nucleus | other |
| SRI | ENSG00000075142 | sorcin | -0,832 | Cytoplasm | transporter |
| SRP9 | ENSG00000143742 | signal recognition particle 9 | -0,642 | Cytoplasm | other |
| SRPK2 | ENSG00000135250 | SRSF protein kinase 2 | 1,058 | Nucleus | kinase |
| SRSF6 | ENSG00000124193 | serine and arginine rich splicing factor 6 | 0,610 | Nucleus | other |
| SSTR2 | ENSG00000180616 | somatostatin receptor 2 | 1,491 | Plasma Membrane | G-protein coupled receptor |
| ST3GAL2 | ENSG00000157350 | ST3 beta-galactoside alpha-2,3-sialyltransferase 2 | 1,201 | Cytoplasm | enzyme |
| ST6GALNAC1 | ENSG00000070526 | ST6 N-acetylgalactosaminide alpha-2,6-sialyltransferase 1 | -0,804 | Cytoplasm | enzyme |
| ST6GALNAC2 | ENSG00000070731 | ST6 N-acetylgalactosaminide alpha-2,6-sialyltransferase 2 | -0,886 | Cytoplasm | enzyme |
| STARD5 | ENSG00000172345 | StAR related lipid transfer domain containing 5 | 0,873 | Cytoplasm | transporter |
| STARD8 | ENSG00000130052 | StAR related lipid transfer domain containing 8 | 1,090 | Cytoplasm | other |
| STAT1 | ENSG00000115415 | signal transducer and activator of transcription 1 | 1,942 | Nucleus | transcription regulator |
| STAT2 | ENSG00000170581 | signal transducer and activator of transcription 2 | 2,297 | Nucleus | transcription regulator |
| STAT4 | ENSG00000138378 | signal transducer and activator of transcription 4 | 0,680 | Nucleus | transcription regulator |
| STAT5A | ENSG00000126561 | signal transducer and activator of transcription 5A | 1,778 | Nucleus | transcription regulator |
| STC2 | ENSG00000113739 | stanniocalcin 2 | 1,060 | Extracellular Space | other |
| STEAP4 | ENSG00000127954 | STEAP4 metalloreductase | -1,319 | Plasma Membrane | enzyme |
| STING1 | ENSG00000184584 | stimulator of interferon response cGAMP interactor 1 | 0,755 | Cytoplasm | other |
| STK10 | ENSG00000072786 | serine/threonine kinase 10 | 1,341 | Cytoplasm | kinase |

| | | | | | |
|---|---|---|---|---|---|
| STK19 | ENSG00000226033 | serine/threonine kinase 19 | 0,906 | Nucleus | kinase |
| STK38L | ENSG00000211455 | serine/threonine kinase 38 like | 1,171 | Cytoplasm | kinase |
| STK40 | ENSG00000196182 | serine/threonine kinase 40 | 0,981 | Cytoplasm | kinase |
| STMN1 | ENSG00000117632 | stathmin 1 | -0,748 | Cytoplasm | other |
| STON2 | ENSG00000140022 | stonin 2 | 1,092 | Cytoplasm | other |
| STRN3 | ENSG00000196792 | striatin 3 | 0,874 | Nucleus | transcription regulator |
| STRN4 | ENSG00000090372 | striatin 4 | 0,772 | Cytoplasm | other |
| STX11 | ENSG00000135604 | syntaxin 11 | 2,120 | Plasma Membrane | transporter |
| STX16 | ENSG00000124222 | syntaxin 16 | 0,716 | Cytoplasm | transporter |
| STX17 | ENSG00000136874 | syntaxin 17 | 0,842 | Plasma Membrane | transporter |
| STX19 | ENSG00000178750 | syntaxin 19 | 2,122 | Other | other |
| STX3 | ENSG00000166900 | syntaxin 3 | 0,984 | Plasma Membrane | transporter |
| STYK1 | ENSG00000060140 | serine/threonine/tyrosine kinase 1 | 2,014 | Cytoplasm | kinase |
| SUCO | ENSG00000094975 | SUN domain containing ossification factor | 0,828 | Other | other |
| SULT1C2 | ENSG00000198203 | sulfotransferase family 1C member 2 | 1,808 | Cytoplasm | enzyme |
| SULT4A1 | ENSG00000130540 | sulfotransferase family 4A member 1 | 0,809 | Cytoplasm | enzyme |
| SUMF1 | ENSG00000144455 | sulfatase modifying factor 1 | -0,971 | Cytoplasm | enzyme |
| SUPT7L | ENSG00000119760 | SPT7 like, STAGA complex subunit gamma | 0,649 | Nucleus | transcription regulator |
| SUPV3L1 | ENSG00000156502 | Suv3 like RNA helicase | 0,952 | Cytoplasm | enzyme |
| SUZ12 | ENSG00000178691 | SUZ12 polycomb repressive complex 2 subunit | 0,737 | Nucleus | enzyme |
| SV2B | ENSG00000185518 | synaptic vesicle glycoprotein 2B | 0,639 | Plasma Membrane | transporter |
| SWI5 | ENSG00000175854 | SWI5 homologous recombination repair protein | -0,822 | Nucleus | other |
| SYDE1 | ENSG00000105137 | synapse defective Rho GTPase homolog 1 | 0,990 | Cytoplasm | other |
| SYNE1 | ENSG00000131018 | spectrin repeat containing nuclear envelope protein 1 | 2,185 | Nucleus | other |
| SYNGR1 | ENSG00000100321 | synaptogyrin 1 | -0,945 | Plasma Membrane | transporter |
| SYNJ1 | ENSG00000159082 | synaptojanin 1 | 1,374 | Cytoplasm | phosphatase |
| SYNPO | ENSG00000171992 | synaptopodin | 2,031 | Cytoplasm | other |
| SYT1 | ENSG00000067715 | synaptotagmin 1 | 0,743 | Cytoplasm | transporter |
| SYT12 | ENSG00000173227 | synaptotagmin 12 | -0,741 | Plasma Membrane | transporter |
| SYT14 | ENSG00000143469 | synaptotagmin 14 | 0,617 | Other | transporter |
| SYTL2 | ENSG00000137501 | synaptotagmin like 2 | 0,929 | Cytoplasm | other |
| TAAR3P | ENSG00000179073 | trace amine associated receptor 3, pseudogene | 1,605 | Plasma Membrane | G-protein coupled receptor |
| TAB3 | ENSG00000157625 | TGF-beta activated kinase 1 (MAP3K7) binding protein 3 | 0,654 | Cytoplasm | other |
| TAF4B | ENSG00000141384 | TATA-box binding protein associated factor 4b | 0,978 | Nucleus | transcription regulator |
| TANC2 | ENSG00000170921 | tetratricopeptide repeat, ankyrin repeat and coiled-coil containing 2 | 0,733 | Nucleus | transcription regulator |
| TANK | ENSG00000136560 | TRAF family member associated NFKB activator | 0,768 | Cytoplasm | other |
| TAOK3 | ENSG00000135090 | TAO kinase 3 | 0,856 | Cytoplasm | kinase |
| TAP1 | ENSG00000168394 | transporter 1, ATP binding cassette subfamily B member | 3,147 | Cytoplasm | transporter |
| TAP2 | ENSG00000237599 | transporter 2, ATP binding cassette subfamily B member | 1,768 | Cytoplasm | transporter |
| TAPBP | ENSG00000231925 | TAP binding protein | 1,640 | Cytoplasm | transporter |
| TAPBPL | ENSG00000139192 | TAP binding protein like | 1,222 | Cytoplasm | other |
| TAPT1 | ENSG00000169762 | transmembrane anterior posterior transformation 1 | 0,764 | Plasma Membrane | G-protein coupled receptor |
| TBC1D10A | ENSG00000099992 | TBC1 domain family member 10A | 0,699 | Plasma Membrane | other |
| TBC1D17 | ENSG00000104946 | TBC1 domain family member 17 | 0,627 | Cytoplasm | other |
| TBC1D22B | ENSG00000065491 | TBC1 domain family member 22B | 1,667 | Other | other |
| TBCEL | ENSG00000154114 | tubulin folding cofactor E like | 0,719 | Other | other |
| TBK1 | ENSG00000183735 | TANK binding kinase 1 | 0,894 | Cytoplasm | kinase |
| TBX3 | ENSG00000135111 | T-box transcription factor 3 | 0,749 | Nucleus | transcription regulator |
| TCEA2 | ENSG00000171703 | transcription elongation factor A2 | -0,695 | Nucleus | transcription regulator |
| TCEANC | ENSG00000176896 | transcription elongation factor A N-terminal and central domain containing | 1,303 | Other | other |
| TCIM | ENSG00000176907 | transcriptional and immune response regulator | 1,530 | Nucleus | other |
| TCN1 | ENSG00000134827 | transcobalamin 1 | -1,182 | Cytoplasm | transporter |
| TCP11L2 | ENSG00000166046 | t-complex 11 like 2 | 1,833 | Other | other |
| TDG | ENSG00000139372 | thymine DNA glycosylase | 0,666 | Nucleus | enzyme |
| TDRD7 | ENSG00000196116 | tudor domain containing 7 | 1,880 | Cytoplasm | other |
| TEC | ENSG00000135605 | tec protein tyrosine kinase | 1,194 | Cytoplasm | kinase |
| TENT4B | ENSG00000121274 | terminal nucleotidyltransferase 4B | 1,232 | Nucleus | enzyme |
| TENT5A | ENSG00000112773 | terminal nucleotidyltransferase 5A | 3,770 | Other | other |
| TENT5C | ENSG00000183508 | terminal nucleotidyltransferase 5C | 0,780 | Extracellular Space | enzyme |
| TEP1 | ENSG00000129566 | telomerase associated protein 1 | 1,602 | Nucleus | enzyme |
| TEPSIN | ENSG00000167302 | TEPSIN adaptor related protein complex 4 accessory protein | 0,796 | Nucleus | other |
| TERF2 | ENSG00000132604 | telomeric repeat binding factor 2 | 0,681 | Nucleus | transcription regulator |
| TESK2 | ENSG00000070759 | testis associated actin remodelling kinase 2 | 1,244 | Nucleus | kinase |
| TET2 | ENSG00000168769 | tet methylcytosine dioxygenase 2 | 1,335 | Nucleus | enzyme |
| TET3 | ENSG00000187605 | tet methylcytosine dioxygenase 3 | 0,982 | Nucleus | enzyme |
| TFE3 | ENSG00000068323 | transcription factor binding to IGHM enhancer 3 | 0,705 | Nucleus | transcription regulator |
| TFRC | ENSG00000072274 | transferrin receptor | -1,073 | Plasma Membrane | transporter |
| TGIF2 | ENSG00000118707 | TGFB induced factor homeobox 2 | 1,283 | Nucleus | transcription regulator |
| TGIF2-RAB5IF | ENSG00000259399 | TGIF2-RAB5IF readthrough | -2,630 | Other | other |
| TGS1 | ENSG00000137574 | trimethylguanosine synthase 1 | 0,912 | Nucleus | enzyme |
| THAP3 | ENSG00000041988 | THAP domain containing 3 | 0,715 | Nucleus | transcription regulator |
| THAP9 | ENSG00000168152 | THAP domain containing 9 | 0,859 | Other | enzyme |
| THBS1 | ENSG00000137801 | thrombospondin 1 | 0,814 | Extracellular Space | other |
| THEMIS2 | ENSG00000130775 | thymocyte selection associated family member 2 | 2,352 | Other | other |
| THRA | ENSG00000126351 | thyroid hormone receptor alpha | -0,849 | Nucleus | ligand-dependent nuclear receptor |
| THRB | ENSG00000151090 | thyroid hormone receptor beta | 0,608 | Nucleus | ligand-dependent nuclear receptor |
| THSD7A | ENSG00000005108 | thrombospondin type 1 domain containing 7A | 2,107 | Cytoplasm | other |
| THUMPD2 | ENSG00000138050 | THUMP domain containing 2 | 0,902 | Other | enzyme |
| TIAM2 | ENSG00000146426 | TIAM Rac1 associated GEF 2 | 0,773 | Cytoplasm | enzyme |
| TICAM1 | ENSG00000127666 | toll like receptor adaptor molecule 1 | 1,939 | Nucleus | other |
| TIFA | ENSG00000145365 | TRAF interacting protein with forkhead associated domain | 1,289 | Cytoplasm | other |
| TIMM23 | ENSG00000265354 | translocase of inner mitochondrial membrane 23 | 0,979 | Cytoplasm | transporter |
| TIMM23B | ENSG00000204152 | translocase of inner mitochondrial membrane 23 homolog B | 0,979 | Cytoplasm | other |
| TINAG | ENSG00000137251 | tubulointerstitial nephritis antigen | 0,760 | Extracellular Space | peptidase |
| TIPARP | ENSG00000163659 | TCDD inducible poly(ADP-ribose) polymerase | 2,863 | Nucleus | enzyme |
| TJAP1 | ENSG00000137221 | tight junction associated protein 1 | 0,629 | Plasma Membrane | other |
| TJP1 | ENSG00000104067 | tight junction protein 1 | 0,973 | Plasma Membrane | other |
| TLDC2 | ENSG00000101342 | TBC/LysM-associated domain containing 2 | 2,833 | Other | other |
| TLE3 | ENSG00000140332 | TLE family member 3, transcriptional corepressor | 0,864 | Nucleus | other |
| TLE4 | ENSG00000106829 | TLE family member 4, transcriptional corepressor | 1,105 | Nucleus | transcription regulator |
| TLK1 | ENSG00000198586 | tousled like kinase 1 | 0,939 | Nucleus | kinase |
| TLK2 | ENSG00000146872 | tousled like kinase 2 | 1,283 | Cytoplasm | kinase |
| TLR3 | ENSG00000164342 | toll like receptor 3 | 1,982 | Plasma Membrane | transmembrane receptor |
| TM4SF4 | ENSG00000169903 | transmembrane 4 L six family member 4 | -0,762 | Plasma Membrane | other |
| TM7SF2 | ENSG00000149809 | transmembrane 7 superfamily member 2 | -0,890 | Cytoplasm | enzyme |
| TMA7 | ENSG00000232112 | translation machinery associated 7 homolog | -0,793 | Other | other |
| TMBIM4 | ENSG00000155957 | transmembrane BAX inhibitor motif containing 4 | -0,803 | Nucleus | other |
| TMBIM6 | ENSG00000139644 | transmembrane BAX inhibitor motif containing 6 | -0,675 | Nucleus | other |
| TMC7 | ENSG00000170537 | transmembrane channel like 7 | 0,830 | Other | other |

| | | | | | |
|---|---|---|---|---|---|
| TMEM107 | ENSG00000179029 | transmembrane protein 107 | -1,004 | Extracellular Space | other |
| TMEM139 | ENSG00000178826 | transmembrane protein 139 | -0,676 | Other | other |
| TMEM140 | ENSG00000146859 | transmembrane protein 140 | 1,530 | Other | other |
| TMEM141 | ENSG00000244187 | transmembrane protein 141 | -0,629 | Other | other |
| TMEM156 | ENSG00000121895 | transmembrane protein 156 | 0,947 | Other | other |
| TMEM160 | ENSG00000130748 | transmembrane protein 160 | -0,617 | Cytoplasm | other |
| TMEM170B | ENSG00000205269 | transmembrane protein 170B | -0,848 | Plasma Membrane | other |
| TMEM171 | ENSG00000157111 | transmembrane protein 171 | 0,670 | Other | other |
| TMEM19 | ENSG00000139291 | transmembrane protein 19 | -0,846 | Other | other |
| TMEM230 | ENSG00000089063 | transmembrane protein 230 | -0,610 | Cytoplasm | other |
| TMEM250 | ENSG00000238227 | transmembrane protein 250 | -0,808 | Nucleus | other |
| TMEM251 | ENSG00000153485 | transmembrane protein 251 | -0,602 | Other | other |
| TMEM256 | ENSG00000205544 | transmembrane protein 256 | -0,867 | Cytoplasm | other |
| TMEM268 | ENSG00000157693 | transmembrane protein 268 | 0,913 | Cytoplasm | other |
| TMEM59 | ENSG00000116209 | transmembrane protein 59 | -0,683 | Plasma Membrane | peptidase |
| TMEM62 | ENSG00000137842 | transmembrane protein 62 | 1,107 | Other | enzyme |
| TMEM86A | ENSG00000151117 | transmembrane protein 86A | 0,813 | Other | other |
| TMEM99 | ENSG00000167920 | transmembrane protein 99 (putative) | -0,671 | Other | other |
| TMPRSS5 | ENSG00000166682 | transmembrane serine protease 5 | -0,978 | Plasma Membrane | peptidase |
| TMSB10/TMSB4X | ENSG00000205542 | thymosin beta 4 X-linked | -0,663 | Cytoplasm | other |
| TMX4 | ENSG00000125827 | thioredoxin related transmembrane protein 4 | -0,603 | Cytoplasm | enzyme |
| TNF | ENSG00000228978 | tumor necrosis factor | 4,760 | Extracellular Space | cytokine |
| TNFAIP2 | ENSG00000185215 | TNF alpha induced protein 2 | 2,566 | Extracellular Space | other |
| TNFAIP3 | ENSG00000118503 | TNF alpha induced protein 3 | 4,307 | Nucleus | enzyme |
| TNFAIP8 | ENSG00000145779 | TNF alpha induced protein 8 | 0,810 | Cytoplasm | other |
| TNFRSF10A | ENSG00000104689 | TNF receptor superfamily member 10a | 0,801 | Plasma Membrane | transmembrane receptor |
| TNFRSF10B | ENSG00000120889 | TNF receptor superfamily member 10b | 1,626 | Plasma Membrane | transmembrane receptor |
| TNFRSF10D | ENSG00000173530 | TNF receptor superfamily member 10d | 1,269 | Plasma Membrane | transmembrane receptor |
| TNFRSF1B | ENSG00000028137 | TNF receptor superfamily member 1B | 1,421 | Plasma Membrane | transmembrane receptor |
| TNFRSF9 | ENSG00000049249 | TNF receptor superfamily member 9 | 2,737 | Plasma Membrane | transmembrane receptor |
| TNFSF10 | ENSG00000121858 | TNF superfamily member 10 | 2,109 | Extracellular Space | cytokine |
| TNFSF13B | ENSG00000102524 | TNF superfamily member 13b | 2,064 | Extracellular Space | cytokine |
| TNFSF15 | ENSG00000181634 | TNF superfamily member 15 | 1,542 | Extracellular Space | cytokine |
| TNFSF18 | ENSG00000120337 | TNF superfamily member 18 | 1,236 | Extracellular Space | cytokine |
| TNFSF9 | ENSG00000125657 | TNF superfamily member 9 | 2,460 | Plasma Membrane | cytokine |
| TNIP1 | ENSG00000145901 | TNFAIP3 interacting protein 1 | 1,013 | Nucleus | other |
| TNKS2 | ENSG00000107854 | tankyrase 2 | 1,240 | Nucleus | enzyme |
| TNNC1 | ENSG00000114854 | troponin C1, slow skeletal and cardiac type | -0,755 | Cytoplasm | other |
| TNNT1 | ENSG00000105048 | troponin T1, slow skeletal type | -0,693 | Cytoplasm | other |
| TNRC6A | ENSG00000090905 | trinucleotide repeat containing adaptor 6A | 0,729 | Nucleus | other |
| TOGARAM1 | ENSG00000198718 | TOG array regulator of axonemal microtubules 1 | 1,409 | Extracellular Space | other |
| TOP2A | ENSG00000131747 | DNA topoisomerase II alpha | -0,770 | Nucleus | enzyme |
| TOPORS | ENSG00000197579 | TOP1 binding arginine/serine rich protein, E3 ubiquitin ligase | 0,686 | Nucleus | enzyme |
| TOR1AIP2 | ENSG00000169905 | torsin 1A interacting protein 2 | 0,788 | Cytoplasm | other |
| TP53BP2 | ENSG00000143514 | tumor protein p53 binding protein 2 | 1,440 | Nucleus | other |
| TP53I11 | ENSG00000175274 | tumor protein p53 inducible protein 11 | -0,815 | Other | other |
| TPD52L1 | ENSG00000111907 | TPD52 like 1 | -0,707 | Cytoplasm | other |
| TPM1 | ENSG00000140416 | tropomyosin 1 | -0,725 | Cytoplasm | other |
| TPPP3 | ENSG00000159713 | tubulin polymerization promoting protein family member 3 | -1,382 | Cytoplasm | other |
| TPRA1 | ENSG00000163870 | transmembrane protein adipocyte associated 1 | 0,668 | Plasma Membrane | G-protein coupled receptor |
| TRA2A | ENSG00000164548 | transformer 2 alpha homolog | 1,149 | Nucleus | other |
| TRA2B | ENSG00000136527 | transformer 2 beta homolog | 0,611 | Nucleus | other |
| TRAF1 | ENSG00000056558 | TNF receptor associated factor 1 | 4,084 | Cytoplasm | other |
| TRAF2 | ENSG00000127191 | TNF receptor associated factor 2 | 0,619 | Cytoplasm | enzyme |
| TRAF3 | ENSG00000131323 | TNF receptor associated factor 3 | 1,535 | Cytoplasm | enzyme |
| TRAF4 | ENSG00000076604 | TNF receptor associated factor 4 | 0,777 | Cytoplasm | other |
| TRAF6 | ENSG00000175104 | TNF receptor associated factor 6 | 1,273 | Cytoplasm | enzyme |
| TRAFD1 | ENSG00000135148 | TRAF-type zinc finger domain containing 1 | 1,297 | Other | other |
| TRANK1 | ENSG00000168016 | tetratricopeptide repeat and ankyrin repeat containing 1 | 3,501 | Nucleus | transcription regulator |
| TRAPPC6A | ENSG00000007255 | trafficking protein particle complex 6A | -0,840 | Cytoplasm | other |
| TRAPPC8 | ENSG00000153339 | trafficking protein particle complex 8 | 0,768 | Cytoplasm | transporter |
| TRERF1 | ENSG00000124496 | transcriptional regulating factor 1 | 1,715 | Nucleus | transcription regulator |
| TREX1 | ENSG00000213689 | three prime repair exonuclease 1 | 1,220 | Nucleus | enzyme |
| TRIB1 | ENSG00000173334 | tribbles pseudokinase 1 | 1,426 | Cytoplasm | kinase |
| TRIM14 | ENSG00000106785 | tripartite motif containing 14 | 1,500 | Cytoplasm | other |
| TRIM16 | ENSG00000221926 | tripartite motif containing 16 | 0,742 | Cytoplasm | transcription regulator |
| TRIM16L | ENSG00000108448 | tripartite motif containing 16 like | 1,232 | Other | other |
| TRIM21 | ENSG00000132109 | tripartite motif containing 21 | 1,981 | Nucleus | enzyme |
| TRIM22 | ENSG00000132274 | tripartite motif containing 22 | 4,040 | Cytoplasm | transcription regulator |
| TRIM25 | ENSG00000121060 | tripartite motif containing 25 | 1,316 | Cytoplasm | transcription regulator |
| TRIM26 | ENSG00000228881 | tripartite motif containing 26 | 1,606 | Cytoplasm | other |
| TRIM29 | ENSG00000137699 | tripartite motif containing 29 | 1,255 | Cytoplasm | transcription regulator |
| TRIM31 | ENSG00000137397 | tripartite motif containing 31 | 0,962 | Cytoplasm | other |
| TRIM36 | ENSG00000152503 | tripartite motif containing 36 | 1,532 | Cytoplasm | enzyme |
| TRIM38 | ENSG00000112343 | tripartite motif containing 38 | 1,571 | Cytoplasm | other |
| TRIM39 | ENSG00000232839 | tripartite motif containing 39 | 0,641 | Cytoplasm | other |
| TRIM39-RPP21 | ENSG00000248167 | TRIM39-RPP21 readthrough | 1,444 | Other | other |
| TRIM5 | ENSG00000132256 | tripartite motif containing 5 | 1,821 | Cytoplasm | enzyme |
| TRIM56 | ENSG00000169871 | tripartite motif containing 56 | 1,320 | Cytoplasm | enzyme |
| TRIM6 | ENSG00000121236 | tripartite motif containing 6 | 0,834 | Cytoplasm | enzyme |
| TRIM69 | ENSG00000185880 | tripartite motif containing 69 | 1,555 | Nucleus | enzyme |
| TRIML2 | ENSG00000179046 | tripartite motif family like 2 | 1,891 | Other | other |
| TRIO | ENSG00000038382 | trio Rho guanine nucleotide exchange factor | 0,968 | Cytoplasm | kinase |
| TRIP12 | ENSG00000153827 | thyroid hormone receptor interactor 12 | 0,759 | Cytoplasm | enzyme |
| TRMT44 | ENSG00000155275 | tRNA methyltransferase 44 homolog | 0,618 | Other | other |
| TRPM7 | ENSG00000092439 | transient receptor potential cation channel subfamily M member 7 | 0,908 | Plasma Membrane | kinase |
| TRPS1 | ENSG00000104447 | transcriptional repressor GATA binding 1 | 1,831 | Nucleus | transcription regulator |
| TRPV3 | ENSG00000167723 | transient receptor potential cation channel subfamily V member 3 | 0,873 | Plasma Membrane | ion channel |
| TRPV4 | ENSG00000111199 | transient receptor potential cation channel subfamily V member 4 | -0,992 | Plasma Membrane | ion channel |
| TRRAP | ENSG00000196367 | transformation/transcription domain associated protein | 0,958 | Nucleus | transcription regulator |
| TSC22D1 | ENSG00000102804 | TSC22 domain family member 1 | 0,841 | Nucleus | transcription regulator |
| TSC22D2 | ENSG00000196428 | TSC22 domain family member 2 | 2,275 | Nucleus | transcription regulator |
| TSPAN1 | ENSG00000117472 | tetraspanin 1 | -0,701 | Cytoplasm | other |
| TSPAN6 | ENSG00000000003 | tetraspanin 6 | -0,623 | Plasma Membrane | other |
| TSPAN8 | ENSG00000127324 | tetraspanin 8 | -1,042 | Plasma Membrane | other |
| TSPAN9 | ENSG00000011105 | tetraspanin 9 | -0,601 | Plasma Membrane | other |
| TSPYL2 | ENSG00000184205 | TSPY like 2 | 1,474 | Nucleus | other |
| TSTD1 | ENSG00000215845 | thiosulfate sulfurtransferase like domain containing 1 | -0,721 | Cytoplasm | enzyme |
| TTBK2 | ENSG00000128881 | tau tubulin kinase 2 | 1,241 | Cytoplasm | kinase |

| | | | | | |
|---|---|---|---|---|---|
| TTC28 | ENSG00000100154 | tetratricopeptide repeat domain 28 | 0,714 | Cytoplasm | other |
| TTC39B | ENSG00000155158 | tetratricopeptide repeat domain 39B | 1,350 | Other | other |
| TTC9B | ENSG00000174521 | tetratricopeptide repeat domain 9B | 2,158 | Other | other |
| TTLL1 | ENSG00000100271 | tubulin tyrosine ligase like 1 | -1,104 | Extracellular Space | enzyme |
| TTLL11 | ENSG00000175764 | tubulin tyrosine ligase like 11 | 1,024 | Cytoplasm | enzyme |
| TTLL4 | ENSG00000135912 | tubulin tyrosine ligase like 4 | 1,019 | Cytoplasm | enzyme |
| TTLL7 | ENSG00000137941 | tubulin tyrosine ligase like 7 | 0,873 | Cytoplasm | enzyme |
| TTPAL | ENSG00000124120 | alpha tocopherol transfer protein like | 0,706 | Other | other |
| TUBA1A | ENSG00000167552 | tubulin alpha 1a | -2,142 | Cytoplasm | other |
| TUBB6 | ENSG00000176014 | tubulin beta 6 class V | -0,636 | Cytoplasm | other |
| TUFT1 | ENSG00000143367 | tuftelin 1 | 1,179 | Cytoplasm | other |
| TXK | ENSG00000074966 | TXK tyrosine kinase | 0,913 | Cytoplasm | kinase |
| TXNIP | ENSG00000265972 | thioredoxin interacting protein | 2,689 | Cytoplasm | other |
| TYMP | ENSG00000025708 | thymidine phosphorylase | 2,224 | Extracellular Space | growth factor |
| TYW5 | ENSG00000162971 | tRNA-yW synthesizing protein 5 | 1,039 | Cytoplasm | enzyme |
| UAP1L1 | ENSG00000197355 | UDP-N-acetylglucosamine pyrophosphorylase 1 like 1 | 0,887 | Other | other |
| UBA6 | ENSG00000033178 | ubiquitin like modifier activating enzyme 6 | 0,757 | Cytoplasm | enzyme |
| UBA7 | ENSG00000182179 | ubiquitin like modifier activating enzyme 7 | 2,309 | Cytoplasm | enzyme |
| UBD | ENSG00000226898 | ubiquitin D | 1,020 | Nucleus | other |
| UBE2L6 | ENSG00000156587 | ubiquitin conjugating enzyme E2 L6 | 2,502 | Cytoplasm | enzyme |
| UBE2Z | ENSG00000159202 | ubiquitin conjugating enzyme E2 Z | 0,825 | Nucleus | enzyme |
| UBN2 | ENSG00000157741 | ubinuclein 2 | 0,959 | Nucleus | other |
| UBR1 | ENSG00000159459 | ubiquitin protein ligase E3 component n-recognin 1 | 1,275 | Cytoplasm | enzyme |
| UBR2 | ENSG00000024048 | ubiquitin protein ligase E3 component n-recognin 2 | 1,428 | Nucleus | enzyme |
| UBR3 | ENSG00000144357 | ubiquitin protein ligase E3 component n-recognin 3 | 0,674 | Other | enzyme |
| UBR4 | ENSG00000127481 | ubiquitin protein ligase E3 component n-recognin 4 | 0,890 | Nucleus | enzyme |
| UBR5 | ENSG00000104517 | ubiquitin protein ligase E3 component n-recognin 5 | 0,621 | Nucleus | enzyme |
| UBXN7 | ENSG00000163960 | UBX domain protein 7 | 1,028 | Nucleus | other |
| UCN2 | ENSG00000145040 | urocortin 2 | 1,081 | Extracellular Space | other |
| UCP2 | ENSG00000175567 | uncoupling protein 2 | -0,965 | Cytoplasm | transporter |
| UGCG | ENSG00000148154 | UDP-glucose ceramide glucosyltransferase | 1,116 | Cytoplasm | enzyme |
| UHRF1BP1 | ENSG00000065060 | UHRF1 binding protein 1 | 1,316 | Other | other |
| UIMC1 | ENSG00000087206 | ubiquitin interaction motif containing 1 | 0,648 | Nucleus | other |
| UNC13A | ENSG00000130477 | unc-13 homolog A | 0,821 | Plasma Membrane | other |
| UNC93B1 | ENSG00000110057 | unc-93 homolog B1, TLR signaling regulator | 0,816 | Cytoplasm | other |
| UNK | ENSG00000132478 | unk zinc finger | 0,726 | Nucleus | transporter |
| UPK1B | ENSG00000114638 | uroplakin 1B | -1,219 | Plasma Membrane | other |
| UPP1 | ENSG00000183696 | uridine phosphorylase 1 | 0,612 | Cytoplasm | enzyme |
| UROD | ENSG00000126088 | uroporphyrinogen decarboxylase | -0,642 | Cytoplasm | enzyme |
| USF1 | ENSG00000158773 | upstream transcription factor 1 | 0,888 | Nucleus | transcription regulator |
| USF3 | ENSG00000176542 | upstream transcription factor family member 3 | 1,252 | Other | other |
| USH1C | ENSG00000006611 | USH1 protein network component harmonin | -0,638 | Plasma Membrane | other |
| USHBP1 | ENSG00000130307 | USH1 protein network component harmonin binding protein 1 | 1,022 | Other | other |
| USP15 | ENSG00000135655 | ubiquitin specific peptidase 15 | 0,850 | Cytoplasm | peptidase |
| USP18 | ENSG00000184979 | ubiquitin specific peptidase 18 | 3,862 | Cytoplasm | peptidase |
| USP19 | ENSG00000172046 | ubiquitin specific peptidase 19 | 0,606 | Cytoplasm | peptidase |
| USP25 | ENSG00000155313 | ubiquitin specific peptidase 25 | 1,552 | Cytoplasm | peptidase |
| USP31 | ENSG00000103404 | ubiquitin specific peptidase 31 | 0,868 | Nucleus | peptidase |
| USP32 | ENSG00000170832 | ubiquitin specific peptidase 32 | 0,699 | Cytoplasm | enzyme |
| USP34 | ENSG00000115464 | ubiquitin specific peptidase 34 | 0,772 | Extracellular Space | peptidase |
| USP36 | ENSG00000055483 | ubiquitin specific peptidase 36 | 1,282 | Nucleus | peptidase |
| USP37 | ENSG00000135913 | ubiquitin specific peptidase 37 | 0,717 | Nucleus | peptidase |
| USP38 | ENSG00000170185 | ubiquitin specific peptidase 38 | 0,687 | Other | peptidase |
| USP42 | ENSG00000106346 | ubiquitin specific peptidase 42 | 0,992 | Nucleus | peptidase |
| USP43 | ENSG00000154914 | ubiquitin specific peptidase 43 | 1,530 | Nucleus | peptidase |
| USP47 | ENSG00000170242 | ubiquitin specific peptidase 47 | 0,865 | Cytoplasm | peptidase |
| USP53 | ENSG00000145390 | ubiquitin specific peptidase 53 | 1,110 | Extracellular Space | enzyme |
| USP54 | ENSG00000166348 | ubiquitin specific peptidase 54 | 0,725 | Other | peptidase |
| USP6NL | ENSG00000148429 | USP6 N-terminal like | 0,913 | Plasma Membrane | other |
| USP9X | ENSG00000124486 | ubiquitin specific peptidase 9 X-linked | 1,027 | Plasma Membrane | peptidase |
| USPL1 | ENSG00000132952 | ubiquitin specific peptidase like 1 | 0,898 | Cytoplasm | peptidase |
| UVRAG | ENSG00000198382 | UV radiation resistance associated | 1,149 | Nucleus | other |
| VAMP2 | ENSG00000220205 | vesicle associated membrane protein 2 | 0,618 | Plasma Membrane | other |
| VAMP5 | ENSG00000168899 | vesicle associated membrane protein 5 | 1,070 | Plasma Membrane | transporter |
| VANGL2 | ENSG00000162738 | VANGL planar cell polarity protein 2 | 0,800 | Plasma Membrane | other |
| VAV3 | ENSG00000134215 | vav guanine nucleotide exchange factor 3 | -0,791 | Extracellular Space | cytokine |
| VCAM1 | ENSG00000162692 | vascular cell adhesion molecule 1 | 1,130 | Plasma Membrane | transmembrane receptor |
| VCPIP1 | ENSG00000175073 | valosin containing protein interacting protein 1 | 0,679 | Cytoplasm | peptidase |
| VCPKMT | ENSG00000100483 | valosin containing protein lysine methyltransferase | 1,388 | Cytoplasm | enzyme |
| VDR | ENSG00000111424 | vitamin D receptor | 1,216 | Nucleus | transcription regulator |
| VEGFA | ENSG00000112715 | vascular endothelial growth factor A | 0,762 | Extracellular Space | growth factor |
| VEGFC | ENSG00000150630 | vascular endothelial growth factor C | 0,875 | Extracellular Space | growth factor |
| VEZF1 | ENSG00000136451 | vascular endothelial zinc finger 1 | 0,944 | Nucleus | transcription regulator |
| VGLL1 | ENSG00000102243 | vestigial like family member 1 | -0,711 | Nucleus | transcription regulator |
| VILL | ENSG00000136059 | villin like | -0,696 | Cytoplasm | other |
| VIM | ENSG00000026025 | vimentin | -0,923 | Cytoplasm | other |
| VKORC1 | ENSG00000167397 | vitamin K epoxide reductase complex subunit 1 | -0,606 | Cytoplasm | enzyme |
| VPS33B | ENSG00000184056 | VPS33B late endosome and lysosome associated | 0,647 | Cytoplasm | transporter |
| VPS52 | ENSG00000223501 | VPS52 subunit of GARP complex | -0,712 | Cytoplasm | other |
| VPS54 | ENSG00000143952 | VPS54 subunit of GARP complex | 0,620 | Extracellular Space | other |
| VTCN1 | ENSG00000134258 | V-set domain containing T cell activation inhibitor 1 | -1,420 | Plasma Membrane | other |
| VWA1 | ENSG00000179403 | von Willebrand factor A domain containing 1 | -0,758 | Extracellular Space | other |
| WAC | ENSG00000095787 | WW domain containing adaptor with coiled-coil | 0,681 | Nucleus | other |
| WARS1 | ENSG00000140105 | tryptophanyl-tRNA synthetase 1 | 1,404 | Cytoplasm | enzyme |
| WASHC4 | ENSG00000136051 | WASH complex subunit 4 | 0,936 | Cytoplasm | other |
| WBP1 | ENSG00000239779 | WW domain binding protein 1 | -1,165 | Cytoplasm | other |
| WDR73 | ENSG00000177082 | WD repeat domain 73 | 0,684 | Extracellular Space | other |
| WDR83OS | ENSG00000105583 | WD repeat domain 83 opposite strand | -0,673 | Other | other |
| WDR91 | ENSG00000105875 | WD repeat domain 91 | 0,618 | Cytoplasm | other |
| WEE1 | ENSG00000166483 | WEE1 G2 checkpoint kinase | 0,996 | Nucleus | kinase |
| WFDC5 | ENSG00000175121 | WAP four-disulfide core domain 5 | 4,147 | Extracellular Space | other |
| WHAMM | ENSG00000156232 | WASP homolog associated with actin, golgi membranes and microtubules | 1,720 | Cytoplasm | other |
| WNK2 | ENSG00000165238 | WNK lysine deficient protein kinase 2 | -0,739 | Cytoplasm | kinase |
| WNT10A | ENSG00000135925 | Wnt family member 10A | 1,252 | Extracellular Space | other |
| WSB1 | ENSG00000109046 | WD repeat and SOCS box containing 1 | 0,647 | Cytoplasm | other |
| WTAP | ENSG00000146457 | WT1 associated protein | 0,813 | Nucleus | other |
| XAF1 | ENSG00000132530 | XIAP associated factor 1 | 3,069 | Nucleus | other |
| XBP1 | ENSG00000100219 | X-box binding protein 1 | 0,660 | Nucleus | transcription regulator |
| XDH | ENSG00000158125 | xanthine dehydrogenase | 0,683 | Cytoplasm | enzyme |

| | | | | | |
|---|---|---|---|---|---|
| XIAP | ENSG00000101966 | X-linked inhibitor of apoptosis | 0,691 | Cytoplasm | enzyme |
| XPO1 | ENSG00000082898 | exportin 1 | 0,687 | Nucleus | transporter |
| XPO6 | ENSG00000169180 | exportin 6 | 1,204 | Cytoplasm | other |
| XRN1 | ENSG00000114127 | 5'-3' exoribonuclease 1 | 2,243 | Cytoplasm | enzyme |
| XRRA1 | ENSG00000166435 | X-ray radiation resistance associated 1 | 0,686 | Cytoplasm | other |
| XXbac-BPG246D15.9 | ENSG00000250264 | | 1,600 | Other | other |
| YAF2 | ENSG00000015153 | YY1 associated factor 2 | 0,845 | Nucleus | transcription regulator |
| YJU2 | ENSG00000105248 | YJU2 splicing factor homolog | 0,645 | Nucleus | other |
| YOD1 | ENSG00000180667 | YOD1 deubiquitinase | 0,993 | Cytoplasm | enzyme |
| YPEL4 | ENSG00000166793 | yippee like 4 | 0,754 | Nucleus | other |
| YRDC | ENSG00000196449 | yrdC N6-threonylcarbamoyltransferase domain containing | 0,905 | Other | other |
| YTHDC1 | ENSG00000083896 | YTH domain containing 1 | 0,931 | Cytoplasm | other |
| YY1AP1 | ENSG00000163374 | YY1 associated protein 1 | 0,813 | Nucleus | other |
| ZBED5 | ENSG00000236287 | zinc finger BED-type containing 5 | 1,078 | Nucleus | transcription regulator |
| ZBED6 | ENSG00000257315 | zinc finger BED-type containing 6 | 0,635 | Nucleus | transcription regulator |
| ZBED9 | ENSG00000248496 | zinc finger BED-type containing 9 | 0,782 | Other | other |
| ZBP1 | ENSG00000124256 | Z-DNA binding protein 1 | 3,676 | Cytoplasm | other |
| ZBTB10 | ENSG00000205189 | zinc finger and BTB domain containing 10 | 2,189 | Nucleus | other |
| ZBTB17 | ENSG00000116809 | zinc finger and BTB domain containing 17 | 1,272 | Nucleus | transcription regulator |
| ZBTB2 | ENSG00000181472 | zinc finger and BTB domain containing 2 | 0,620 | Nucleus | transcription regulator |
| ZBTB20 | ENSG00000181722 | zinc finger and BTB domain containing 20 | 2,292 | Nucleus | transcription regulator |
| ZBTB21 | ENSG00000173276 | zinc finger and BTB domain containing 21 | 1,404 | Nucleus | transcription regulator |
| ZBTB25 | ENSG00000089775 | zinc finger and BTB domain containing 25 | 0,876 | Nucleus | transcription regulator |
| ZBTB38 | ENSG00000177311 | zinc finger and BTB domain containing 38 | 0,713 | Nucleus | transcription regulator |
| ZBTB39 | ENSG00000166860 | zinc finger and BTB domain containing 39 | 0,720 | Nucleus | transcription regulator |
| ZBTB43 | ENSG00000169155 | zinc finger and BTB domain containing 43 | 1,503 | Nucleus | transcription regulator |
| ZBTB48 | ENSG00000204859 | zinc finger and BTB domain containing 48 | 0,986 | Nucleus | transcription regulator |
| ZBTB49 | ENSG00000168826 | zinc finger and BTB domain containing 49 | 1,251 | Nucleus | transcription regulator |
| ZBTB6 | ENSG00000186130 | zinc finger and BTB domain containing 6 | 0,623 | Nucleus | transcription regulator |
| ZC3H11A | ENSG00000058673 | zinc finger CCCH-type containing 11A | 0,657 | Nucleus | other |
| ZC3H12A | ENSG00000163874 | zinc finger CCCH-type containing 12A | 3,034 | Cytoplasm | enzyme |
| ZC3H12C | ENSG00000149289 | zinc finger CCCH-type containing 12C | 2,032 | Other | other |
| ZC3H4 | ENSG00000130749 | zinc finger CCCH-type containing 4 | 0,743 | Nucleus | transcription regulator |
| ZC3HAV1 | ENSG00000105939 | zinc finger CCCH-type containing, antiviral 1 | 3,612 | Plasma Membrane | other |
| ZC4H2 | ENSG00000126970 | zinc finger C4H2-type containing | -0,761 | Nucleus | other |
| ZCCHC2 | ENSG00000141664 | zinc finger CCHC-type containing 2 | 1,700 | Cytoplasm | other |
| ZCCHC8 | ENSG00000033030 | zinc finger CCHC-type containing 8 | 0,737 | Nucleus | other |
| ZDHHC14 | ENSG00000175048 | zinc finger DHHC-type palmitoyltransferase 14 | 0,860 | Cytoplasm | enzyme |
| ZDHHC2 | ENSG00000104219 | zinc finger DHHC-type palmitoyltransferase 2 | -0,619 | Nucleus | enzyme |
| ZEB1 | ENSG00000148516 | zinc finger E-box binding homeobox 1 | 1,608 | Nucleus | transcription regulator |
| ZEB2 | ENSG00000169554 | zinc finger E-box binding homeobox 2 | 2,786 | Nucleus | transcription regulator |
| ZFAND2A | ENSG00000178381 | zinc finger AN1-type containing 2A | 0,657 | Other | other |
| ZFAT | ENSG00000066827 | zinc finger and AT-hook domain containing | 0,709 | Nucleus | transcription regulator |
| ZFHX2 | ENSG00000136367 | zinc finger homeobox 2 | 1,296 | Nucleus | transcription regulator |
| ZFHX3 | ENSG00000140836 | zinc finger homeobox 3 | 0,731 | Nucleus | transcription regulator |
| ZFHX4 | ENSG00000091656 | zinc finger homeobox 4 | 1,018 | Nucleus | transcription regulator |
| ZFP36 | ENSG00000128016 | ZFP36 ring finger protein | 2,766 | Nucleus | transcription regulator |
| ZFP37 | ENSG00000136866 | ZFP37 zinc finger protein | 0,651 | Nucleus | transcription regulator |
| ZFX | ENSG00000005889 | zinc finger protein X-linked | 0,822 | Nucleus | transcription regulator |
| ZFYVE1 | ENSG00000165861 | zinc finger FYVE-type containing 1 | 1,197 | Cytoplasm | other |
| ZFYVE26 | ENSG00000072121 | zinc finger FYVE-type containing 26 | 0,891 | Cytoplasm | other |
| ZG16B | ENSG00000162078 | zymogen granule protein 16B | -0,992 | Cytoplasm | other |
| ZGLP1 | ENSG00000220201 | zinc finger GATA like protein 1 | 0,925 | Nucleus | transcription regulator |
| ZHX2 | ENSG00000178764 | zinc fingers and homeoboxes 2 | 1,365 | Nucleus | transcription regulator |
| ZKSCAN2 | ENSG00000155592 | zinc finger with KRAB and SCAN domains 2 | 1,023 | Nucleus | transcription regulator |
| ZKSCAN4 | ENSG00000187626 | zinc finger with KRAB and SCAN domains 4 | 1,014 | Nucleus | transcription regulator |
| ZKSCAN5 | ENSG00000196652 | zinc finger with KRAB and SCAN domains 5 | 0,674 | Nucleus | transcription regulator |
| ZMYM2 | ENSG00000121741 | zinc finger MYM-type containing 2 | 0,620 | Nucleus | transcription regulator |
| ZMYM5 | ENSG00000132950 | zinc finger MYM-type containing 5 | 1,250 | Nucleus | transcription regulator |
| ZMYND8 | ENSG00000101040 | zinc finger MYND-type containing 8 | 0,735 | Nucleus | transcription regulator |
| ZNF10 | ENSG00000256223 | zinc finger protein 10 | 0,925 | Cytoplasm | transcription regulator |
| ZNF101 | ENSG00000181896 | zinc finger protein 101 | 1,272 | Nucleus | transcription regulator |
| ZNF107 | ENSG00000196247 | zinc finger protein 107 | 0,874 | Nucleus | transcription regulator |
| ZNF131 | ENSG00000172262 | zinc finger protein 131 | 0,742 | Nucleus | transcription regulator |
| ZNF136 | ENSG00000196646 | zinc finger protein 136 | 0,699 | Nucleus | transcription regulator |
| ZNF140 | ENSG00000196387 | zinc finger protein 140 | 1,391 | Nucleus | transcription regulator |
| ZNF16 | ENSG00000170631 | zinc finger protein 16 | 0,852 | Nucleus | transcription regulator |
| ZNF17 | ENSG00000186272 | zinc finger protein 17 | 0,857 | Nucleus | transcription regulator |
| ZNF175 | ENSG00000105497 | zinc finger protein 175 | 0,655 | Nucleus | transcription regulator |
| ZNF184 | ENSG00000096654 | zinc finger protein 184 | 1,189 | Nucleus | transcription regulator |
| ZNF20 | ENSG00000132010 | zinc finger protein 20 | 1,075 | Nucleus | transcription regulator |
| ZNF200 | ENSG00000010539 | zinc finger protein 200 | 1,037 | Nucleus | transcription regulator |
| ZNF212 | ENSG00000170260 | zinc finger protein 212 | 1,022 | Nucleus | transcription regulator |
| ZNF217 | ENSG00000171940 | zinc finger protein 217 | 1,653 | Nucleus | transcription regulator |
| ZNF222 | ENSG00000159885 | zinc finger protein 222 | 1,351 | Nucleus | transcription regulator |
| ZNF225 | ENSG00000256294 | zinc finger protein 225 | 0,888 | Nucleus | transcription regulator |
| ZNF254 | ENSG00000213096 | zinc finger protein 254 | 0,804 | Nucleus | transcription regulator |
| ZNF264 | ENSG00000083844 | zinc finger protein 264 | 0,653 | Nucleus | transcription regulator |
| ZNF267 | ENSG00000185947 | zinc finger protein 267 | 1,398 | Nucleus | transcription regulator |
| ZNF274 | ENSG00000171606 | zinc finger protein 274 | 1,573 | Nucleus | transcription regulator |
| ZNF280C | ENSG00000056277 | zinc finger protein 280C | 0,865 | Nucleus | transcription regulator |
| ZNF280D | ENSG00000137871 | zinc finger protein 280D | 1,036 | Other | other |
| ZNF292 | ENSG00000188994 | zinc finger protein 292 | 1,990 | Nucleus | transcription regulator |
| ZNF296 | ENSG00000170684 | zinc finger protein 296 | 1,088 | Nucleus | transcription regulator |
| ZNF3 | ENSG00000166526 | zinc finger protein 3 | 0,668 | Nucleus | transcription regulator |
| ZNF316 | ENSG00000205903 | zinc finger protein 316 | 0,641 | Other | other |
| ZNF317 | ENSG00000130803 | zinc finger protein 317 | 1,110 | Nucleus | transcription regulator |
| ZNF324 | ENSG00000083812 | zinc finger protein 324 | 1,000 | Nucleus | transcription regulator |
| ZNF329 | ENSG00000181894 | zinc finger protein 329 | 0,687 | Other | other |
| ZNF333 | ENSG00000160961 | zinc finger protein 333 | 0,884 | Nucleus | transcription regulator |
| ZNF335 | ENSG00000198026 | zinc finger protein 335 | 1,241 | Nucleus | transcription regulator |
| ZNF347 | ENSG00000197937 | zinc finger protein 347 | 0,938 | Nucleus | transcription regulator |
| ZNF35 | ENSG00000169981 | zinc finger protein 35 | 0,989 | Nucleus | transcription regulator |
| ZNF394 | ENSG00000160908 | zinc finger protein 394 | 0,809 | Nucleus | transcription regulator |
| ZNF408 | ENSG00000175213 | zinc finger protein 408 | 0,631 | Nucleus | transcription regulator |
| ZNF432 | ENSG00000256087 | zinc finger protein 432 | 0,687 | Nucleus | transcription regulator |
| ZNF433 | ENSG00000197647 | zinc finger protein 433 | 2,183 | Nucleus | transcription regulator |
| ZNF436 | ENSG00000125945 | zinc finger protein 436 | 1,684 | Nucleus | transcription regulator |
| ZNF436-AS1 | ENSG00000249087 | ZNF436 antisense RNA 1 | 0,783 | Other | other |

| | | | | | |
|---|---|---|---|---|---|
| ZNF438 | ENSG00000183621 | zinc finger protein 438 | 0,708 | Nucleus | transcription regulator |
| ZNF44 | ENSG00000197857 | zinc finger protein 44 | 1,484 | Nucleus | transcription regulator |
| ZNF441 | ENSG00000197044 | zinc finger protein 441 | 1,512 | Nucleus | transcription regulator |
| ZNF442 | ENSG00000198342 | zinc finger protein 442 | 2,445 | Nucleus | transcription regulator |
| ZNF451 | ENSG00000112200 | zinc finger protein 451 | 1,067 | Nucleus | enzyme |
| ZNF460 | ENSG00000197714 | zinc finger protein 460 | 0,904 | Nucleus | transcription regulator |
| ZNF462 | ENSG00000148143 | zinc finger protein 462 | 1,123 | Nucleus | transcription regulator |
| ZNF467 | ENSG00000181444 | zinc finger protein 467 | 1,097 | Nucleus | transcription regulator |
| ZNF471 | ENSG00000196263 | zinc finger protein 471 | 0,977 | Nucleus | transcription regulator |
| ZNF473 | ENSG00000142528 | zinc finger protein 473 | 1,395 | Nucleus | other |
| ZNF484 | ENSG00000127081 | zinc finger protein 484 | 0,841 | Nucleus | transcription regulator |
| ZNF503 | ENSG00000165655 | zinc finger protein 503 | 1,818 | Nucleus | other |
| ZNF514 | ENSG00000144026 | zinc finger protein 514 | -0,670 | Nucleus | transcription regulator |
| ZNF529 | ENSG00000186020 | zinc finger protein 529 | 0,788 | Other | other |
| ZNF532 | ENSG00000074657 | zinc finger protein 532 | 0,796 | Nucleus | transcription regulator |
| ZNF543 | ENSG00000178229 | zinc finger protein 543 | 1,338 | Other | other |
| ZNF548 | ENSG00000188785 | zinc finger protein 548 | 0,862 | Other | other |
| ZNF551 | ENSG00000204519 | zinc finger protein 551 | 0,804 | Nucleus | transcription regulator |
| ZNF557 | ENSG00000130544 | zinc finger protein 557 | 0,829 | Nucleus | transcription regulator |
| ZNF562 | ENSG00000171466 | zinc finger protein 562 | 1,287 | Nucleus | transcription regulator |
| ZNF564 | ENSG00000249709 | zinc finger protein 564 | 1,071 | Nucleus | transcription regulator |
| ZNF57 | ENSG00000171970 | zinc finger protein 57 | 0,933 | Other | other |
| ZNF571 | ENSG00000180479 | zinc finger protein 571 | 0,682 | Nucleus | other |
| ZNF585B | ENSG00000245680 | zinc finger protein 585B | 0,901 | Nucleus | other |
| ZNF592 | ENSG00000166716 | zinc finger protein 592 | 0,761 | Nucleus | transcription regulator |
| ZNF608 | ENSG00000168916 | zinc finger protein 608 | 1,051 | Other | other |
| ZNF614 | ENSG00000142556 | zinc finger protein 614 | 0,710 | Other | other |
| ZNF619 | ENSG00000177873 | zinc finger protein 619 | 1,248 | Other | other |
| ZNF620 | ENSG00000177842 | zinc finger protein 620 | 1,571 | Nucleus | transcription regulator |
| ZNF623 | ENSG00000183309 | zinc finger protein 623 | 0,730 | Nucleus | transcription regulator |
| ZNF627 | ENSG00000198551 | zinc finger protein 627 | 1,921 | Other | other |
| ZNF628 | ENSG00000197483 | zinc finger protein 628 | 0,601 | Nucleus | transcription regulator |
| ZNF644 | ENSG00000122482 | zinc finger protein 644 | 0,669 | Nucleus | transcription regulator |
| ZNF646 | ENSG00000167395 | zinc finger protein 646 | 0,803 | Nucleus | transcription regulator |
| ZNF654 | ENSG00000175105 | zinc finger protein 654 | 0,859 | Other | other |
| ZNF662 | ENSG00000182983 | zinc finger protein 662 | 0,626 | Other | other |
| ZNF674 | ENSG00000251192 | zinc finger protein 674 | 0,790 | Other | other |
| ZNF697 | ENSG00000143067 | zinc finger protein 697 | 1,929 | Other | other |
| ZNF699 | ENSG00000196110 | zinc finger protein 699 | 0,772 | Other | other |
| ZNF7 | ENSG00000147789 | zinc finger protein 7 | 0,789 | Nucleus | transcription regulator |
| ZNF707 | ENSG00000181135 | zinc finger protein 707 | 0,790 | Other | other |
| ZNF711 | ENSG00000147180 | zinc finger protein 711 | 0,707 | Nucleus | transcription regulator |
| ZNF75A | ENSG00000162086 | zinc finger protein 75a | 0,684 | Nucleus | transcription regulator |
| ZNF763 | ENSG00000197054 | zinc finger protein 763 | 1,242 | Cytoplasm | transcription regulator |
| ZNF764 | ENSG00000169951 | zinc finger protein 764 | 1,201 | Other | other |
| ZNF77 | ENSG00000175691 | zinc finger protein 77 | 0,915 | Nucleus | transcription regulator |
| ZNF772 | ENSG00000197128 | zinc finger protein 772 | 0,870 | Other | other |
| ZNF773 | ENSG00000152439 | zinc finger protein 773 | 0,982 | Other | other |
| ZNF778 | ENSG00000170100 | zinc finger protein 778 | 1,009 | Nucleus | transcription regulator |
| ZNF787 | ENSG00000142409 | zinc finger protein 787 | 0,888 | Other | other |
| ZNF79 | ENSG00000196152 | zinc finger protein 79 | 1,016 | Nucleus | transcription regulator |
| ZNF800 | ENSG00000048405 | zinc finger protein 800 | 0,829 | Other | other |
| ZNF805 | ENSG00000204524 | zinc finger protein 805 | 1,178 | Other | other |
| ZNF816-ZNF321P | ENSG00000221874 | ZNF816-ZNF321P readthrough | -2,100 | Other | other |
| ZNF823 | ENSG00000197933 | zinc finger protein 823 | 0,757 | Nucleus | transcription regulator |
| ZNF841 | ENSG00000197608 | zinc finger protein 841 | 0,844 | Other | other |
| ZNF92 | ENSG00000146757 | zinc finger protein 92 | 0,873 | Nucleus | transcription regulator |
| ZNFX1 | ENSG00000124201 | zinc finger NFX1-type containing 1 | 2,563 | Nucleus | transcription regulator |
| ZRANB1 | ENSG00000019995 | zinc finger RANBP2-type containing 1 | 0,815 | Nucleus | peptidase |
| ZRSR2 | ENSG00000169249 | zinc finger CCCH-type, RNA binding motif and serine/arginine rich 2 | 0,630 | Nucleus | other |
| ZSCAN12 | ENSG00000158691 | zinc finger and SCAN domain containing 12 | 0,734 | Nucleus | transcription regulator |
| ZSCAN20 | ENSG00000121903 | zinc finger and SCAN domain containing 20 | 0,624 | Nucleus | transcription regulator |
| ZSCAN25 | ENSG00000197037 | zinc finger and SCAN domain containing 25 | 0,617 | Other | other |
| ZSWIM3 | ENSG00000132801 | zinc finger SWIM-type containing 3 | 1,364 | Other | other |
| ZSWIM4 | ENSG00000132003 | zinc finger SWIM-type containing 4 | 1,790 | Other | other |
| ZSWIM6 | ENSG00000130449 | zinc finger SWIM-type containing 6 | 1,168 | Extracellular Space | other |
| ZSWIM8 | ENSG00000214655 | zinc finger SWIM-type containing 8 | 0,890 | Extracellular Space | other |
| ZXDA | ENSG00000198205 | zinc finger X-linked duplicated A | 0,689 | Other | transcription regulator |
| ZZEF1 | ENSG00000074755 | zinc finger ZZ-type and EF-hand domain containing 1 | 0,783 | Other | other |

**Supplementary Table S7. List of differentially expressed genes for the condition "Caco-2_SARS-CoV-2 versus Caco-2_Mock" subjected to IPA Core Analysis.** The number of differentially expressed genes is 128.

| Symbol | ID | Entrez Gene Name | Expr Log Ratio | Location | Type(s) |
|---|---|---|---|---|---|
| ABL2 | ENSG00000143322 | ABL proto-oncogene 2, non-receptor tyrosine kinase | 0,687 | Cytoplasm | kinase |
| AFAP1 | ENSG00000196526 | actin filament associated protein 1 | 0,643 | Cytoplasm | other |
| AFP | ENSG00000081051 | alpha fetoprotein | -1,327 | Extracellular Space | transporter |
| AGT | ENSG00000135744 | angiotensinogen | -0,703 | Extracellular Space | growth factor |
| AHSG | ENSG00000145192 | alpha 2-HS glycoprotein | -0,719 | Extracellular Space | other |
| AKAP12 | ENSG00000131016 | A-kinase anchoring protein 12 | 1,321 | Cytoplasm | transporter |
| AKR1C3 | ENSG00000196139 | aldo-keto reductase family 1 member C3 | -0,940 | Cytoplasm | enzyme |
| ALDH1A1 | ENSG00000165092 | aldehyde dehydrogenase 1 family member A1 | -0,687 | Cytoplasm | enzyme |
| ANKRD1 | ENSG00000148677 | ankyrin repeat domain 1 | 0,901 | Cytoplasm | transcription regulator |
| ARRDC3 | ENSG00000113369 | arrestin domain containing 3 | 3,101 | Plasma Membrane | other |
| ASB2 | ENSG00000100628 | ankyrin repeat and SOCS box containing 2 | 0,910 | Nucleus | transcription regulator |
| ATF3 | ENSG00000162772 | activating transcription factor 3 | 2,334 | Nucleus | transcription regulator |
| AXL | ENSG00000167601 | AXL receptor tyrosine kinase | 0,630 | Plasma Membrane | kinase |
| B4GALNT2 | ENSG00000167080 | beta-1,4-N-acetyl-galactosaminyltransferase 2 | -0,718 | Cytoplasm | enzyme |
| BCL10 | ENSG00000142867 | BCL10 immune signaling adaptor | 0,685 | Cytoplasm | transcription regulator |
| BHLHE40 | ENSG00000134107 | basic helix-loop-helix family member e40 | 1,074 | Nucleus | transcription regulator |
| BIRC3 | ENSG00000023445 | baculoviral IAP repeat containing 3 | 0,857 | Cytoplasm | enzyme |
| CCND1 | ENSG00000110092 | cyclin D1 | 0,856 | Nucleus | transcription regulator |
| CSRNP1 | ENSG00000144655 | cysteine and serine rich nuclear protein 1 | 0,957 | Nucleus | transcription regulator |
| CTD_2278I106 | ENSG00000269307 | | -1,002 | Other | other |
| CTSV | ENSG00000136943 | cathepsin V | -0,634 | Cytoplasm | peptidase |
| CXCL2 | ENSG00000081041 | C-X-C motif chemokine ligand 2 | 2,081 | Extracellular Space | cytokine |
| CYLD | ENSG00000083799 | CYLD lysine 63 deubiquitinase | 1,002 | Nucleus | transcription regulator |
| CYP1A1 | ENSG00000140465 | cytochrome P450 family 1 subfamily A member 1 | 1,247 | Cytoplasm | enzyme |
| DCBLD2 | ENSG00000057019 | discoidin, CUB and LCCL domain containing 2 | 0,611 | Plasma Membrane | other |
| DUSP1 | ENSG00000120129 | dual specificity phosphatase 1 | 1,360 | Nucleus | phosphatase |
| DUSP6 | ENSG00000139318 | dual specificity phosphatase 6 | 1,253 | Cytoplasm | phosphatase |
| DYRK1A | ENSG00000157540 | dual specificity tyrosine phosphorylation regulated kinase 1A | 0,767 | Nucleus | kinase |
| EGR1 | ENSG00000120738 | early growth response 1 | 1,752 | Nucleus | transcription regulator |
| EMP1 | ENSG00000134531 | epithelial membrane protein 1 | 0,886 | Plasma Membrane | other |
| EPHA2 | ENSG00000142627 | EPH receptor A2 | 0,750 | Plasma Membrane | kinase |
| EPHX2 | ENSG00000120915 | epoxide hydrolase 2 | -0,657 | Cytoplasm | enzyme |
| ERRFI1 | ENSG00000116285 | ERBB receptor feedback inhibitor 1 | 0,860 | Cytoplasm | other |
| ETS1 | ENSG00000134954 | ETS proto-oncogene 1, transcription factor | 0,805 | Nucleus | transcription regulator |
| FABP1 | ENSG00000163586 | fatty acid binding protein 1 | -0,646 | Cytoplasm | transporter |
| FBXW10 | ENSG00000171931 | F-box and WD repeat domain containing 10 | 1,016 | Cytoplasm | other |
| FGG | ENSG00000171557 | fibrinogen gamma chain | -0,867 | Extracellular Space | other |
| FOS | ENSG00000170345 | Fos proto-oncogene, AP-1 transcription factor subunit | 1,259 | Nucleus | transcription regulator |
| GEM | ENSG00000164949 | GTP binding protein overexpressed in skeletal muscle | 1,039 | Plasma Membrane | enzyme |
| GJB1 | ENSG00000169562 | gap junction protein beta 1 | -1,392 | Plasma Membrane | transporter |
| GSTA2 | ENSG00000244067 | glutathione S-transferase alpha 2 | -1,119 | Cytoplasm | enzyme |
| H2BC5 | ENSG00000158373 | H2B clustered histone 5 | 1,258 | Nucleus | other |
| H3C14 | ENSG00000203811 | H3 clustered histone 14 | 1,692 | Nucleus | other |
| H3C15 | ENSG00000203852 | H3 clustered histone 15 | 1,692 | Nucleus | other |
| H3C4 | ENSG00000197409 | H3 clustered histone 4 | 1,543 | Nucleus | other |
| HDAC9 | ENSG00000048052 | histone deacetylase 9 | 1,354 | Nucleus | transcription regulator |
| HIVEP2 | ENSG00000010818 | HIVEP zinc finger 2 | 1,575 | Nucleus | transcription regulator |
| ICAM1 | ENSG00000090339 | intercellular adhesion molecule 1 | 0,698 | Plasma Membrane | transmembrane receptor |
| IER2 | ENSG00000160888 | immediate early response 2 | 1,176 | Cytoplasm | transcription regulator |
| IER3 | ENSG00000237155 | immediate early response 3 | 1,439 | Cytoplasm | other |
| IGFBP4 | ENSG00000141753 | insulin like growth factor binding protein 4 | 1,045 | Extracellular Space | other |
| INAFM1 | ENSG00000257704 | InaF motif containing 1 | -2,267 | Other | other |
| ITGA2 | ENSG00000164171 | integrin subunit alpha 2 | 1,130 | Plasma Membrane | transmembrane receptor |
| ITGA6 | ENSG00000091409 | integrin subunit alpha 6 | 0,707 | Plasma Membrane | transmembrane receptor |
| JUN | ENSG00000177606 | Jun proto-oncogene, AP-1 transcription factor subunit | 2,403 | Nucleus | transcription regulator |
| JUNB | ENSG00000171223 | JunB proto-oncogene, AP-1 transcription factor subunit | 1,695 | Nucleus | transcription regulator |
| KLF10 | ENSG00000155090 | Kruppel like factor 10 | 1,092 | Nucleus | transcription regulator |
| KLF6 | ENSG00000067082 | Kruppel like factor 6 | 1,105 | Nucleus | transcription regulator |
| KRT6A | ENSG00000205420 | keratin 6A | 1,829 | Cytoplasm | other |
| LAMA1 | ENSG00000101680 | laminin subunit alpha 1 | -0,972 | Extracellular Space | other |
| LCA5 | ENSG00000135338 | lebercilin LCA5 | 0,728 | Extracellular Space | other |
| LDLR | ENSG00000130164 | low density lipoprotein receptor | 0,605 | Plasma Membrane | transporter |
| MAFF | ENSG00000185022 | MAF bZIP transcription factor F | 0,794 | Nucleus | transcription regulator |
| MDM2 | ENSG00000135679 | MDM2 proto-oncogene | 1,676 | Nucleus | transcription regulator |
| MXD1 | ENSG00000059728 | MAX dimerization protein 1 | 1,581 | Nucleus | transcription regulator |
| MXD4 | ENSG00000123933 | MAX dimerization protein 4 | -0,799 | Nucleus | transcription regulator |
| MYC | ENSG00000136997 | MYC proto-oncogene, bHLH transcription factor | 0,662 | Nucleus | transcription regulator |
| NAIPP4 | ENSG00000251634 | NAIP pseudogene 4 | 1,094 | Other | other |
| NDRG1 | ENSG00000104419 | N-myc downstream regulated 1 | -1,044 | Nucleus | kinase |
| NFKBIA | ENSG00000100906 | NFKB inhibitor alpha | 0,938 | Cytoplasm | transcription regulator |
| NFKBIZ | ENSG00000144802 | NFKB inhibitor zeta | 0,753 | Nucleus | transcription regulator |
| NR1D1 | ENSG00000126368 | nuclear receptor subfamily 1 group D member 1 | 1,717 | Nucleus | ligand-dependent nuclear receptor |

| | | | | | |
|---|---|---|---|---|---|
| NUAK2 | ENSG00000163545 | NUAK family kinase 2 | 1,119 | Nucleus | kinase |
| OAT | ENSG00000065154 | ornithine aminotransferase | -0,830 | Cytoplasm | enzyme |
| PALD1 | ENSG00000107719 | phosphatase domain containing paladin 1 | 0,830 | Cytoplasm | phosphatase |
| PARD6B | ENSG00000124171 | par-6 family cell polarity regulator beta | 1,065 | Plasma Membrane | other |
| PDZK1 | ENSG00000174827 | PDZ domain containing 1 | -0,734 | Plasma Membrane | other |
| PER1 | ENSG00000179094 | period circadian regulator 1 | 1,646 | Nucleus | transcription regulator |
| PHLDA1 | ENSG00000139289 | pleckstrin homology like domain family A member 1 | 0,601 | Cytoplasm | other |
| PHLDA2 | ENSG00000181649 | pleckstrin homology like domain family A member 2 | 0,785 | Cytoplasm | other |
| PI3 | ENSG00000124102 | peptidase inhibitor 3 | -1,440 | Extracellular Space | other |
| PLAU | ENSG00000122861 | plasminogen activator, urokinase | 1,375 | Extracellular Space | peptidase |
| PLK2 | ENSG00000145632 | polo like kinase 2 | 1,357 | Nucleus | kinase |
| PMM1 | ENSG00000100417 | phosphomannomutase 1 | -0,673 | Cytoplasm | enzyme |
| POLR2J2/POLR2J3 | ENSG00000267645 | RNA polymerase II subunit J2 | 3,504 | Other | transcription regulator |
| PPP1R15A | ENSG00000087074 | protein phosphatase 1 regulatory subunit 15A | 1,420 | Cytoplasm | other |
| PRR15L | ENSG00000167183 | proline rich 15 like | -1,284 | Other | other |
| REEP6 | ENSG00000115255 | receptor accessory protein 6 | -0,893 | Plasma Membrane | other |
| RGS2 | ENSG00000116741 | regulator of G protein signaling 2 | 0,706 | Nucleus | enzyme |
| RHOB | ENSG00000143878 | ras homolog family member B | 0,709 | Cytoplasm | enzyme |
| RP11-474G23.1 | ENSG00000273398 | | -0,809 | Other | other |
| SELENOW | ENSG00000178980 | selenoprotein W | 1,254 | Cytoplasm | enzyme |
| SEPHS2 | ENSG00000179918 | selenophosphate synthetase 2 | -0,619 | Cytoplasm | enzyme |
| SERINC5 | ENSG00000164300 | serine incorporator 5 | -0,748 | Plasma Membrane | transporter |
| SERPINA6 | ENSG00000170099 | serpin family A member 6 | -1,180 | Extracellular Space | other |
| SERPINE1 | ENSG00000106366 | serpin family E member 1 | 1,861 | Extracellular Space | other |
| SLC25A25 | ENSG00000148339 | solute carrier family 25 member 25 | 0,651 | Cytoplasm | transporter |
| SLC39A5 | ENSG00000139540 | solute carrier family 39 member 5 | -0,873 | Plasma Membrane | transporter |
| SMIM14 | ENSG00000163683 | small integral membrane protein 14 | -0,715 | Cytoplasm | other |
| SPATA2 | ENSG00000158480 | spermatogenesis associated 2 | 0,636 | Extracellular Space | other |
| SPRED1 | ENSG00000166068 | sprouty related EVH1 domain containing 1 | 0,795 | Plasma Membrane | other |
| SPRY4 | ENSG00000187678 | sprouty RTK signaling antagonist 4 | 0,952 | Plasma Membrane | other |
| TBX3 | ENSG00000135111 | T-box transcription factor 3 | 0,719 | Nucleus | transcription regulator |
| TENT5A | ENSG00000112773 | terminal nucleotidyltransferase 5A | 0,673 | Other | other |
| TIPARP | ENSG00000163659 | TCDD inducible poly(ADP-ribose) polymerase | 1,317 | Nucleus | enzyme |
| TNFAIP3 | ENSG00000118503 | TNF alpha induced protein 3 | 1,049 | Nucleus | enzyme |
| TNFRSF10B | ENSG00000120889 | TNF receptor superfamily member 10b | 0,667 | Plasma Membrane | transmembrane receptor |
| TNS4 | ENSG00000131746 | tensin 4 | 1,831 | Cytoplasm | other |
| TRRAP | ENSG00000196367 | transformation/transcription domain associated protein | 0,769 | Nucleus | transcription regulator |
| TSC22D2 | ENSG00000196428 | TSC22 domain family member 2 | 0,931 | Nucleus | transcription regulator |
| TSPAN8 | ENSG00000127324 | tetraspanin 8 | -0,697 | Plasma Membrane | other |
| TTR | ENSG00000118271 | transthyretin | -0,928 | Extracellular Space | transporter |
| TXNIP | ENSG00000265972 | thioredoxin interacting protein | 0,857 | Cytoplasm | other |
| UBASH3B | ENSG00000154127 | ubiquitin associated and SH3 domain containing B | 0,799 | Other | enzyme |
| VGF | ENSG00000128564 | VGF nerve growth factor inducible | 1,729 | Extracellular Space | growth factor |
| VIM | ENSG00000026025 | vimentin | 0,650 | Cytoplasm | other |
| YOD1 | ENSG00000180667 | YOD1 deubiquitinase | 0,854 | Cytoplasm | enzyme |
| ZBTB20 | ENSG00000181722 | zinc finger and BTB domain containing 20 | 1,666 | Nucleus | transcription regulator |
| ZBTB43 | ENSG00000169155 | zinc finger and BTB domain containing 43 | 0,697 | Nucleus | transcription regulator |
| ZC3H12A | ENSG00000163874 | zinc finger CCCH-type containing 12A | 0,977 | Cytoplasm | enzyme |
| ZFP36 | ENSG00000128016 | ZFP36 ring finger protein | 0,874 | Nucleus | transcription regulator |
| ZNF441 | ENSG00000197044 | zinc finger protein 441 | 1,068 | Nucleus | transcription regulator |
| ZNF442 | ENSG00000198342 | zinc finger protein 442 | 1,317 | Nucleus | transcription regulator |
| ZNF554 | ENSG00000172006 | zinc finger protein 554 | 1,756 | Nucleus | transcription regulator |
| ZNF555 | ENSG00000186300 | zinc finger protein 555 | 1,261 | Other | other |
| ZNF608 | ENSG00000168916 | zinc finger protein 608 | 0,967 | Other | other |
| ZNF627 | ENSG00000198551 | zinc finger protein 627 | 1,271 | Other | other |
| ZNF844 | ENSG00000223547 | zinc finger protein 844 | 1,938 | Other | other |

**Supplementary Table S8. List of differentially expressed genes for the condition "Caco-2_SARS-CoV versus Caco-2_Mock" subjected to IPA Core Analysis.** The number of differentially expressed genes is 656.

| Symbol | ID | Entrez Gene Name | Expr Log Ratio | Location | Type(s) |
|---|---|---|---|---|---|
| ABCC6 | ENSG00000091262 | ATP binding cassette subfamily C member 6 | -0,848 | Plasma Membrane | transporter |
| ABHD4 | ENSG00000100439 | abhydrolase domain containing 4, N-acyl phospholipase B | -0,673 | Cytoplasm | peptidase |
| ABL2 | ENSG00000143322 | ABL proto-oncogene 2, non-receptor tyrosine kinase | 1,002 | Cytoplasm | kinase |
| AC0936164 | ENSG00000234231 | | 1,031 | Other | other |
| AC1045343 | ENSG00000268083 | | 2,609 | Other | other |
| AC240274.1 | ENSG00000271254 | | -0,617 | Other | other |
| ACADS | ENSG00000122971 | acyl-CoA dehydrogenase short chain | -0,619 | Cytoplasm | enzyme |
| ACADSB | ENSG00000196177 | acyl-CoA dehydrogenase short/branched chain | -0,687 | Cytoplasm | enzyme |
| ACHE | ENSG00000087085 | acetylcholinesterase (Cartwright blood group) | 0,819 | Plasma Membrane | enzyme |
| ACOT1 | ENSG00000184227 | acyl-CoA thioesterase 1 | -1,311 | Cytoplasm | enzyme |
| ACSF2 | ENSG00000167107 | acyl-CoA synthetase family member 2 | -0,843 | Cytoplasm | enzyme |
| ACSL1 | ENSG00000151726 | acyl-CoA synthetase long chain family member 1 | -0,629 | Cytoplasm | enzyme |
| ACSL5 | ENSG00000197142 | acyl-CoA synthetase long chain family member 5 | 0,810 | Cytoplasm | enzyme |
| ACSS1 | ENSG00000154930 | acyl-CoA synthetase short chain family member 1 | -0,736 | Cytoplasm | enzyme |
| ADD3 | ENSG00000148700 | adducin 3 | -0,611 | Cytoplasm | other |
| ADD3-AS1 | ENSG00000203876 | ADD3 antisense RNA 1 | -1,026 | Other | other |
| ADH4 | ENSG00000198099 | alcohol dehydrogenase 4 (class II), pi polypeptide | -1,144 | Cytoplasm | enzyme |
| ADH6 | ENSG00000172955 | alcohol dehydrogenase 6 (class V) | -0,970 | Cytoplasm | enzyme |
| AEN | ENSG00000181026 | apoptosis enhancing nuclease | 0,660 | Nucleus | enzyme |
| AFAP1 | ENSG00000196526 | actin filament associated protein 1 | 0,829 | Cytoplasm | other |
| AKAP12 | ENSG00000131016 | A-kinase anchoring protein 12 | 2,045 | Cytoplasm | transporter |
| AKR1C3 | ENSG00000196139 | aldo-keto reductase family 1 member C3 | -1,149 | Cytoplasm | enzyme |
| ALDH1A1 | ENSG00000165092 | aldehyde dehydrogenase 1 family member A1 | -0,697 | Cytoplasm | enzyme |
| ALDH3B2 | ENSG00000132746 | aldehyde dehydrogenase 3 family member B2 | -0,694 | Cytoplasm | enzyme |
| ALDH6A1 | ENSG00000119711 | aldehyde dehydrogenase 6 family member A1 | -1,059 | Cytoplasm | enzyme |
| ALG1L | ENSG00000189366 | ALG1 chitobiosyldiphosphodolichol beta-mannosyltransferase like | -1,315 | Other | other |
| ALOXE3 | ENSG00000179148 | arachidonate lipoxygenase 3 | 1,076 | Extracellular Space | enzyme |
| ALPK2 | ENSG00000198796 | alpha kinase 2 | -0,959 | Nucleus | kinase |
| AMACR | ENSG00000242110 | alpha-methylacyl-CoA racemase | -1,211 | Cytoplasm | enzyme |
| AMER3 | ENSG00000178171 | APC membrane recruitment protein 3 | -0,713 | Other | other |
| AMOT | ENSG00000126016 | angiomotin | -1,304 | Plasma Membrane | other |
| ANKRD1 | ENSG00000148677 | ankyrin repeat domain 1 | 1,043 | Cytoplasm | transcription regulator |
| ANXA9 | ENSG00000143412 | annexin A9 | -1,291 | Plasma Membrane | transmembrane receptor |
| APBB1 | ENSG00000166313 | amyloid beta precursor protein binding family B member 1 | -0,659 | Cytoplasm | transcription regulator |
| APOBEC3C | ENSG00000244509 | apolipoprotein B mRNA editing enzyme catalytic subunit 3C | -0,969 | Other | enzyme |
| APOL1 | ENSG00000100342 | apolipoprotein L1 | 0,764 | Extracellular Space | transporter |
| AQP11 | ENSG00000178301 | aquaporin 11 | -0,715 | Plasma Membrane | transporter |
| AREG | ENSG00000109321 | amphiregulin | 0,667 | Extracellular Space | growth factor |
| ARHGAP29 | ENSG00000137962 | Rho GTPase activating protein 29 | 0,900 | Cytoplasm | other |
| ARHGEF10L | ENSG00000074964 | Rho guanine nucleotide exchange factor 10 like | -0,691 | Cytoplasm | enzyme |
| ARPIN/ARPIN-AP3S2 | ENSG00000242498 | ARPIN-AP3S2 readthrough | -0,893 | Plasma Membrane | other |
| ARRB1 | ENSG00000137486 | arrestin beta 1 | -0,624 | Cytoplasm | other |
| ARRDC3 | ENSG00000113369 | arrestin domain containing 3 | 1,943 | Plasma Membrane | other |
| AS3MT | ENSG00000214435 | arsenite methyltransferase | -0,823 | Cytoplasm | enzyme |
| ASRGL1 | ENSG00000162174 | asparaginase and isoaspartyl peptidase 1 | -0,767 | Cytoplasm | enzyme |
| ATAD2B | ENSG00000119778 | ATPase family AAA domain containing 2B | 0,631 | Nucleus | other |
| ATAD3B | ENSG00000160072 | ATPase family AAA domain containing 3B | 0,629 | Nucleus | other |
| ATF3 | ENSG00000162772 | activating transcription factor 3 | 1,491 | Nucleus | transcription regulator |
| B3GNT9 | ENSG00000237172 | UDP-GlcNAc:betaGal beta-1,3-N-acetylglucosaminyltransferase 9 | -0,721 | Cytoplasm | enzyme |
| B4GALNT2 | ENSG00000167080 | beta-1,4-N-acetyl-galactosaminyltransferase 2 | -1,505 | Cytoplasm | enzyme |
| BAZ2B | ENSG00000123636 | bromodomain adjacent to zinc finger domain 2B | 0,729 | Extracellular Space | other |
| BBS2 | ENSG00000125124 | Bardet-Biedl syndrome 2 | -0,695 | Cytoplasm | other |
| BCL10 | ENSG00000142867 | BCL10 immune signaling adaptor | 0,697 | Cytoplasm | transcription regulator |
| BCL2 | ENSG00000171791 | BCL2 apoptosis regulator | -0,689 | Cytoplasm | transporter |
| BCRP3 | ENSG00000215481 | BCR pseudogene 3 | -0,641 | Other | other |
| BDP1 | ENSG00000145734 | B double prime 1, subunit of RNA polymerase III transcription initiation factor IIIB | 0,661 | Nucleus | transcription regulator |
| BHLHE40 | ENSG00000134107 | basic helix-loop-helix family member e40 | 1,081 | Nucleus | transcription regulator |
| BIRC3 | ENSG00000023445 | baculoviral IAP repeat containing 3 | 0,804 | Cytoplasm | enzyme |
| BNIP3L | ENSG00000104765 | BCL2 interacting protein 3 like | -0,989 | Cytoplasm | other |
| BRPF1 | ENSG00000156983 | bromodomain and PHD finger containing 1 | 0,660 | Nucleus | transporter |
| C11orf71 | ENSG00000180425 | chromosome 11 open reading frame 71 | -1,092 | Other | other |
| C19orf54 | ENSG00000188493 | chromosome 19 open reading frame 54 | -0,646 | Other | other |
| C1orf115 | ENSG00000162817 | chromosome 1 open reading frame 115 | -0,905 | Other | other |
| C21orf91 | ENSG00000154642 | chromosome 21 open reading frame 91 | 0,774 | Other | other |
| C2orf72 | ENSG00000204128 | chromosome 2 open reading frame 72 | -0,767 | Other | other |
| C3 | ENSG00000125730 | complement C3 | 0,814 | Extracellular Space | peptidase |
| C6orf141 | ENSG00000197261 | chromosome 6 open reading frame 141 | 1,357 | Other | other |
| CA8 | ENSG00000178538 | carbonic anhydrase 8 | -0,797 | Cytoplasm | enzyme |
| CABLES2 | ENSG00000149679 | Cdk5 and Abl enzyme substrate 2 | -0,738 | Other | other |
| CACHD1 | ENSG00000158966 | cache domain containing 1 | -0,673 | Other | other |
| CAMSAP2 | ENSG00000118200 | calmodulin regulated spectrin associated protein family member 2 | 0,667 | Cytoplasm | other |
| CAPRIN2 | ENSG00000110888 | caprin family member 2 | 1,130 | Cytoplasm | other |
| CASP6 | ENSG00000138794 | caspase 6 | -0,955 | Cytoplasm | peptidase |
| CCDC183 | ENSG00000213213 | coiled-coil domain containing 183 | -0,884 | Other | other |
| CCDC34 | ENSG00000109881 | coiled-coil domain containing 34 | -0,686 | Other | other |
| CCDC68 | ENSG00000166510 | coiled-coil domain containing 68 | 0,667 | Cytoplasm | other |
| CCDC69 | ENSG00000198624 | coiled-coil domain containing 69 | -0,693 | Other | other |
| CCND1 | ENSG00000110092 | cyclin D1 | 0,979 | Nucleus | transcription regulator |
| CCNL1 | ENSG00000163660 | cyclin L1 | 0,620 | Nucleus | other |
| CD3EAP | ENSG00000117877 | CD3e molecule associated protein | 0,935 | Nucleus | other |
| CDC14B | ENSG00000081377 | cell division cycle 14B | -0,622 | Nucleus | phosphatase |
| CDC42EP1 | ENSG00000128283 | CDC42 effector protein 1 | 0,723 | Extracellular Space | other |
| CDK18 | ENSG00000117266 | cyclin dependent kinase 18 | -0,822 | Cytoplasm | kinase |
| CDK19 | ENSG00000155111 | cyclin dependent kinase 19 | -0,651 | Nucleus | kinase |
| CDKN2C | ENSG00000123080 | cyclin dependent kinase inhibitor 2C | -0,822 | Nucleus | transcription regulator |
| CDX2 | ENSG00000165556 | caudal type homeobox 2 | -0,950 | Nucleus | transcription regulator |

| | | | | | |
|---|---|---|---|---|---|
| CEBPA | ENSG00000245848 | CCAAT enhancer binding protein alpha | -0,892 | Nucleus | transcription regulator |
| CEP170 | ENSG00000143702 | centrosomal protein 170 | 0,930 | Nucleus | other |
| CFLAR | ENSG00000003402 | CASP8 and FADD like apoptosis regulator | 0,784 | Cytoplasm | other |
| CFTR | ENSG00000001626 | CF transmembrane conductance regulator | -0,789 | Plasma Membrane | ion channel |
| CGAS | ENSG00000164430 | cyclic GMP-AMP synthase | 0,610 | Cytoplasm | enzyme |
| CHRNB1 | ENSG00000170175 | cholinergic receptor nicotinic beta 1 subunit | -1,064 | Plasma Membrane | transmembrane receptor |
| CHST13 | ENSG00000180767 | carbohydrate sulfotransferase 13 | -0,707 | Cytoplasm | enzyme |
| CHST9 | ENSG00000154080 | carbohydrate sulfotransferase 9 | -0,872 | Cytoplasm | enzyme |
| CISH | ENSG00000114737 | cytokine inducible SH2 containing protein | 0,672 | Cytoplasm | other |
| CLCF1 | ENSG00000175505 | cardiotrophin like cytokine factor 1 | 0,999 | Extracellular Space | cytokine |
| CLDN3 | ENSG00000165215 | claudin 3 | -1,211 | Plasma Membrane | transmembrane receptor |
| CNTN5 | ENSG00000149972 | contactin 5 | -0,636 | Plasma Membrane | other |
| COL4A1 | ENSG00000187498 | collagen type IV alpha 1 chain | -0,801 | Extracellular Space | other |
| COLEC11 | ENSG00000118004 | collectin subfamily member 11 | 1,330 | Extracellular Space | other |
| CPA6 | ENSG00000165078 | carboxypeptidase A6 | -0,625 | Extracellular Space | peptidase |
| CPEB4 | ENSG00000113742 | cytoplasmic polyadenylation element binding protein 4 | 0,626 | Plasma Membrane | other |
| CPN1 | ENSG00000120054 | carboxypeptidase N subunit 1 | -0,802 | Extracellular Space | peptidase |
| CROT | ENSG00000005469 | carnitine O-octanoyltransferase | -0,642 | Cytoplasm | enzyme |
| CRYL1 | ENSG00000165475 | crystallin lambda 1 | -0,606 | Cytoplasm | enzyme |
| CSRNP1 | ENSG00000144655 | cysteine and serine rich nuclear protein 1 | 0,972 | Nucleus | transcription regulator |
| CTD_2278I106 | ENSG00000269307 | | -1,002 | Other | other |
| CTD_3074O711 | ENSG00000256349 | | -0,703 | Other | other |
| CXCL2 | ENSG00000081041 | C-X-C motif chemokine ligand 2 | 1,796 | Extracellular Space | cytokine |
| CXCL8 | ENSG00000169429 | C-X-C motif chemokine ligand 8 | 1,059 | Extracellular Space | cytokine |
| CXCR4 | ENSG00000121966 | C-X-C motif chemokine receptor 4 | 0,745 | Plasma Membrane | G-protein coupled receptor |
| CXXC5 | ENSG00000171604 | CXXC finger protein 5 | -0,654 | Nucleus | other |
| CYMP | ENSG00000240194 | chymosin, pseudogene | -2,080 | Other | other |
| CYP1A1 | ENSG00000140465 | cytochrome P450 family 1 subfamily A member 1 | 1,299 | Cytoplasm | enzyme |
| CYP27A1 | ENSG00000135929 | cytochrome P450 family 27 subfamily A member 1 | -1,183 | Cytoplasm | enzyme |
| CYP2W1 | ENSG00000073067 | cytochrome P450 family 2 subfamily W member 1 | -0,966 | Cytoplasm | enzyme |
| DACT2 | ENSG00000164488 | dishevelled binding antagonist of beta catenin 2 | -0,736 | Cytoplasm | other |
| DCAF11 | ENSG00000100897 | DDB1 and CUL4 associated factor 11 | -0,657 | Nucleus | other |
| DCBLD2 | ENSG00000057019 | discoidin, CUB and LCCL domain containing 2 | 0,846 | Plasma Membrane | other |
| DCUN1D3 | ENSG00000188215 | defective in cullin neddylation 1 domain containing 3 | 0,606 | Cytoplasm | other |
| DDB2 | ENSG00000134574 | damage specific DNA binding protein 2 | -0,613 | Nucleus | other |
| DDIAS | ENSG00000165490 | DNA damage induced apoptosis suppressor | 0,724 | Cytoplasm | other |
| DDX21 | ENSG00000165732 | DExD-box helicase 21 | 0,652 | Nucleus | enzyme |
| DENND11 | ENSG00000262599 | DENN domain containing 11 | -0,651 | Other | other |
| DHRS3 | ENSG00000162496 | dehydrogenase/reductase 3 | -0,943 | Cytoplasm | enzyme |
| DIO1 | ENSG00000211452 | iodothyronine deiodinase 1 | -0,868 | Cytoplasm | enzyme |
| DLX1 | ENSG00000144355 | distal-less homeobox 1 | 0,930 | Nucleus | transcription regulator |
| DLX2 | ENSG00000115844 | distal-less homeobox 2 | 0,737 | Nucleus | transcription regulator |
| DNAJC15 | ENSG00000120675 | DnaJ heat shock protein family (Hsp40) member C15 | -1,119 | Cytoplasm | other |
| DNMT3B | ENSG00000088305 | DNA methyltransferase 3 beta | -0,696 | Nucleus | enzyme |
| DPYSL3 | ENSG00000113657 | dihydropyrimidinase like 3 | -1,236 | Cytoplasm | enzyme |
| DRICH1 | ENSG00000189269 | aspartate rich 1 | -1,145 | Other | other |
| DST | ENSG00000151914 | dystonin | 0,862 | Plasma Membrane | other |
| DUSP1 | ENSG00000120129 | dual specificity phosphatase 1 | 1,382 | Nucleus | phosphatase |
| DUSP2 | ENSG00000158050 | dual specificity phosphatase 2 | 1,015 | Nucleus | phosphatase |
| DUSP4 | ENSG00000120875 | dual specificity phosphatase 4 | 2,115 | Nucleus | phosphatase |
| DUSP5 | ENSG00000138166 | dual specificity phosphatase 5 | 2,082 | Nucleus | phosphatase |
| DUSP6 | ENSG00000139318 | dual specificity phosphatase 6 | 1,606 | Cytoplasm | phosphatase |
| DUSP7 | ENSG00000164086 | dual specificity phosphatase 7 | 0,747 | Cytoplasm | phosphatase |
| DUSP9 | ENSG00000130829 | dual specificity phosphatase 9 | -1,161 | Nucleus | phosphatase |
| EDN1 | ENSG00000078401 | endothelin 1 | 0,734 | Extracellular Space | cytokine |
| EEF1A1P5 | ENSG00000196205 | eukaryotic translation elongation factor 1 alpha 1 pseudogene 5 | -2,724 | Extracellular Space | other |
| EEF1A1P6 | ENSG00000233476 | eukaryotic translation elongation factor 1 alpha 1 pseudogene 6 | -1,119 | Other | other |
| EFNA1 | ENSG00000169242 | ephrin A1 | -0,764 | Plasma Membrane | other |
| EFNB2 | ENSG00000125266 | ephrin B2 | 0,703 | Plasma Membrane | kinase |
| EGR1 | ENSG00000120738 | early growth response 1 | 1,995 | Nucleus | transcription regulator |
| EGR2 | ENSG00000122877 | early growth response 2 | 1,029 | Nucleus | transcription regulator |
| EGR4 | ENSG00000135625 | early growth response 4 | 0,628 | Nucleus | transcription regulator |
| EHD2 | ENSG00000024422 | EH domain containing 2 | 0,724 | Nucleus | other |
| EHHADH | ENSG00000113790 | enoyl-CoA hydratase and 3-hydroxyacyl CoA dehydrogenase | -1,086 | Cytoplasm | enzyme |
| EHMT2 | ENSG00000224143 | euchromatic histone lysine methyltransferase 2 | -1,500 | Nucleus | transcription regulator |
| ELK3 | ENSG00000111145 | ETS transcription factor ELK3 | 0,952 | Nucleus | transcription regulator |
| EMP1 | ENSG00000134531 | epithelial membrane protein 1 | 1,529 | Plasma Membrane | other |
| EPB41L1 | ENSG00000088367 | erythrocyte membrane protein band 4.1 like 1 | 0,613 | Plasma Membrane | other |
| EPHA2 | ENSG00000142627 | EPH receptor A2 | 1,153 | Plasma Membrane | kinase |
| EPHX2 | ENSG00000120915 | epoxide hydrolase 2 | -0,817 | Cytoplasm | enzyme |
| EPN3 | ENSG00000049283 | epsin 3 | -0,696 | Cytoplasm | other |
| EPO | ENSG00000130427 | erythropoietin | -0,752 | Extracellular Space | cytokine |
| EPS8L2 | ENSG00000177106 | EPS8 like 2 | -0,745 | Cytoplasm | other |
| EPSTI1 | ENSG00000133106 | epithelial stromal interaction 1 | -1,068 | Other | other |
| ERBB3 | ENSG00000065361 | erb-b2 receptor tyrosine kinase 3 | -0,879 | Plasma Membrane | kinase |
| ERRFI1 | ENSG00000116285 | ERBB receptor feedback inhibitor 1 | 1,168 | Cytoplasm | other |
| ESAM | ENSG00000149564 | endothelial cell adhesion molecule | 1,133 | Plasma Membrane | other |
| ESPN | ENSG00000187017 | espin | -1,232 | Cytoplasm | other |
| ESRRA | ENSG00000173153 | estrogen related receptor alpha | 0,618 | Nucleus | ligand-dependent nuclear receptor |
| ETS1 | ENSG00000134954 | ETS proto-oncogene 1, transcription factor | 1,635 | Nucleus | transcription regulator |
| EXOC3L4 | ENSG00000205436 | exocyst complex component 3 like 4 | -1,508 | Other | other |
| F7 | ENSG00000057593 | coagulation factor VII | -1,076 | Plasma Membrane | peptidase |
| FA2H | ENSG00000103089 | fatty acid 2-hydroxylase | 0,714 | Cytoplasm | enzyme |
| FAM114A1 | ENSG00000197712 | family with sequence similarity 114 member A1 | -1,122 | Extracellular Space | other |
| FAM131B | ENSG00000159784 | family with sequence similarity 131 member B | -0,624 | Other | other |
| FAM135A | ENSG00000082269 | family with sequence similarity 135 member A | 0,804 | Other | enzyme |
| FAM151A | ENSG00000162391 | family with sequence similarity 151 member A | -0,853 | Extracellular Space | other |
| FAM20C | ENSG00000177706 | FAM20C golgi associated secretory pathway kinase | -0,603 | Extracellular Space | enzyme |
| FAM3B | ENSG00000183844 | FAM3 metabolism regulating signaling molecule B | -0,781 | Extracellular Space | cytokine |

| | | | | | |
|---|---|---|---|---|---|
| FAM3C2 | ENSG00000174028 | FAM3C pseudogene | 0,622 | Other | other |
| FAM43A | ENSG00000185112 | family with sequence similarity 43 member A | 0,957 | Other | other |
| FAM83G | ENSG00000188522 | family with sequence similarity 83 member G | 0,725 | Other | other |
| FBP1 | ENSG00000165140 | fructose-bisphosphatase 1 | -0,708 | Cytoplasm | phosphatase |
| FBXL20 | ENSG00000108306 | F-box and leucine rich repeat protein 20 | -0,888 | Cytoplasm | other |
| FBXL6 | ENSG00000182325 | F-box and leucine rich repeat protein 6 | 0,652 | Other | enzyme |
| FBXW7 | ENSG00000109670 | F-box and WD repeat domain containing 7 | 0,627 | Nucleus | enzyme |
| FCGRT | ENSG00000104870 | Fc fragment of IgG receptor and transporter | -0,712 | Plasma Membrane | transmembrane receptor |
| FEM1A | ENSG00000141965 | fem-1 homolog A | 0,667 | Nucleus | transcription regulator |
| FERMT1 | ENSG00000101311 | fermitin family member 1 | 0,601 | Plasma Membrane | other |
| FERMT2 | ENSG00000073712 | fermitin family member 2 | 0,611 | Cytoplasm | other |
| FGA | ENSG00000171560 | fibrinogen alpha chain | -0,834 | Extracellular Space | other |
| FGD3 | ENSG00000127084 | FYVE, RhoGEF and PH domain containing 3 | -0,872 | Cytoplasm | other |
| FGD6 | ENSG00000180263 | FYVE, RhoGEF and PH domain containing 6 | 0,629 | Cytoplasm | other |
| FGFR1 | ENSG00000077782 | fibroblast growth factor receptor 1 | 0,762 | Plasma Membrane | kinase |
| FGFR2 | ENSG00000066468 | fibroblast growth factor receptor 2 | -0,890 | Plasma Membrane | kinase |
| FGG | ENSG00000171557 | fibrinogen gamma chain | -0,655 | Extracellular Space | other |
| FHL2 | ENSG00000115641 | four and a half LIM domains 2 | 1,069 | Nucleus | transcription regulator |
| FHL3 | ENSG00000183386 | four and a half LIM domains 3 | 0,643 | Plasma Membrane | other |
| FIG4 | ENSG00000112367 | FIG4 phosphoinositide 5-phosphatase | -0,802 | Cytoplasm | enzyme |
| FIGNL2 | ENSG00000261308 | fidgetin like 2 | -0,896 | Other | other |
| FJX1 | ENSG00000179431 | four-jointed box kinase 1 | 0,868 | Extracellular Space | other |
| FMN1 | ENSG00000248905 | formin 1 | 1,027 | Nucleus | other |
| FOS | ENSG00000170345 | Fos proto-oncogene, AP-1 transcription factor subunit | 1,519 | Nucleus | transcription regulator |
| FOSL1 | ENSG00000175592 | FOS like 1, AP-1 transcription factor subunit | 2,088 | Nucleus | transcription regulator |
| FOXA1 | ENSG00000129514 | forkhead box A1 | -0,667 | Nucleus | transcription regulator |
| FOXP1 | ENSG00000114861 | forkhead box P1 | -0,706 | Nucleus | transcription regulator |
| FRAS1 | ENSG00000138759 | Fraser extracellular matrix complex subunit 1 | -0,745 | Extracellular Space | other |
| FSTL3 | ENSG00000070404 | follistatin like 3 | 0,747 | Extracellular Space | other |
| FZD4 | ENSG00000174804 | frizzled class receptor 4 | -0,775 | Plasma Membrane | G-protein coupled receptor |
| G0S2 | ENSG00000123689 | G0/G1 switch 2 | -1,121 | Cytoplasm | other |
| GAB1 | ENSG00000109458 | GRB2 associated binding protein 1 | -0,769 | Cytoplasm | other |
| GABBR1 | ENSG00000232569 | gamma-aminobutyric acid type B receptor subunit 1 | -0,882 | Plasma Membrane | G-protein coupled receptor |
| GADD45A | ENSG00000116717 | growth arrest and DNA damage inducible alpha | 0,803 | Nucleus | other |
| GADD45B | ENSG00000099860 | growth arrest and DNA damage inducible beta | 1,080 | Cytoplasm | other |
| GALM | ENSG00000143891 | galactose mutarotase | -1,132 | Cytoplasm | enzyme |
| GALNT6 | ENSG00000139629 | polypeptide N-acetylgalactosaminyltransferase 6 | -0,666 | Cytoplasm | enzyme |
| GAS2 | ENSG00000148935 | growth arrest specific 2 | -0,737 | Cytoplasm | other |
| GAS6 | ENSG00000183087 | growth arrest specific 6 | -0,669 | Extracellular Space | growth factor |
| GATA4 | ENSG00000136574 | GATA binding protein 4 | -0,924 | Nucleus | transcription regulator |
| GATM | ENSG00000171766 | glycine amidinotransferase | -0,983 | Cytoplasm | enzyme |
| GCNT1 | ENSG00000187210 | glucosaminyl (N-acetyl) transferase 1 | -1,032 | Cytoplasm | enzyme |
| GCNT3 | ENSG00000140297 | glucosaminyl (N-acetyl) transferase 3, mucin type | 0,901 | Plasma Membrane | enzyme |
| GGT1 | ENSG00000100031 | gamma-glutamyltransferase 1 | -0,652 | Plasma Membrane | enzyme |
| GJB1 | ENSG00000169562 | gap junction protein beta 1 | -1,782 | Plasma Membrane | transporter |
| GLUL | ENSG00000135821 | glutamate-ammonia ligase | -0,681 | Cytoplasm | enzyme |
| GLYCTK | ENSG00000168237 | glycerate kinase | -0,905 | Cytoplasm | kinase |
| GNAI1 | ENSG00000127955 | G protein subunit alpha i1 | 0,713 | Plasma Membrane | enzyme |
| GNG2 | ENSG00000186469 | G protein subunit gamma 2 | -0,877 | Plasma Membrane | enzyme |
| GOLT1A | ENSG00000174567 | golgi transport 1A | 0,748 | Cytoplasm | other |
| GPAT3 | ENSG00000138678 | glycerol-3-phosphate acyltransferase 3 | 1,464 | Cytoplasm | enzyme |
| GPC1 | ENSG00000063660 | glypican 1 | 0,759 | Plasma Membrane | transmembrane receptor |
| GPC5 | ENSG00000179399 | glypican 5 | -0,973 | Plasma Membrane | other |
| GPCPD1 | ENSG00000125772 | glycerophosphocholine phosphodiesterase 1 | 0,770 | Cytoplasm | enzyme |
| GPR160 | ENSG00000173890 | G protein-coupled receptor 160 | -0,905 | Plasma Membrane | G-protein coupled receptor |
| GPRC5A | ENSG00000013588 | G protein-coupled receptor class C group 5 member A | 0,967 | Plasma Membrane | G-protein coupled receptor |
| GPRC5C | ENSG00000170412 | G protein-coupled receptor class C group 5 member C | -0,751 | Plasma Membrane | G-protein coupled receptor |
| GPT2 | ENSG00000166123 | glutamic--pyruvic transaminase 2 | -0,681 | Cytoplasm | enzyme |
| GREB1L | ENSG00000141449 | GREB1 like retinoic acid receptor coactivator | 0,604 | Extracellular Space | other |
| GREM1 | ENSG00000166923 | gremlin 1, DAN family BMP antagonist | 1,429 | Extracellular Space | other |
| GRIK4 | ENSG00000149403 | glutamate ionotropic receptor kainate type subunit 4 | -0,607 | Plasma Membrane | ion channel |
| GSTA2 | ENSG00000244067 | glutathione S-transferase alpha 2 | -1,216 | Cytoplasm | enzyme |
| GSTA4 | ENSG00000170899 | glutathione S-transferase alpha 4 | -1,039 | Cytoplasm | enzyme |
| GSTK1 | ENSG00000197448 | glutathione S-transferase kappa 1 | -0,640 | Cytoplasm | enzyme |
| GTF2IP4 | ENSG00000233369 | general transcription factor IIi pseudogene 4 | -0,934 | Other | other |
| GUCY2C | ENSG00000070019 | guanylate cyclase 2C | -0,660 | Plasma Membrane | kinase |
| GYG2 | ENSG00000056998 | glycogenin 2 | -0,717 | Cytoplasm | enzyme |
| HAPLN3 | ENSG00000140511 | hyaluronan and proteoglycan link protein 3 | -1,034 | Extracellular Space | other |
| HDAC9 | ENSG00000048052 | histone deacetylase 9 | 1,180 | Nucleus | transcription regulator |
| HES4 | ENSG00000188290 | hes family bHLH transcription factor 4 | 0,620 | Other | other |
| HEXIM2 | ENSG00000168517 | HEXIM P-TEFb complex subunit 2 | -0,822 | Nucleus | transcription regulator |
| HFE | ENSG00000010704 | homeostatic iron regulator | -0,888 | Plasma Membrane | transmembrane receptor |
| HGD | ENSG00000113924 | homogentisate 1,2-dioxygenase | -0,860 | Cytoplasm | enzyme |
| HIVEP2 | ENSG00000010818 | HIVEP zinc finger 2 | 1,107 | Nucleus | transcription regulator |
| HK2 | ENSG00000159399 | hexokinase 2 | 0,772 | Cytoplasm | kinase |
| HMGCS2 | ENSG00000134240 | 3-hydroxy-3-methylglutaryl-CoA synthase 2 | -1,539 | Cytoplasm | enzyme |
| HOXB3 | ENSG00000120093 | homeobox B3 | 0,664 | Nucleus | transcription regulator |
| HOXB6 | ENSG00000108511 | homeobox B6 | 0,672 | Nucleus | transcription regulator |
| HOXB8 | ENSG00000120068 | homeobox B8 | 1,651 | Nucleus | transcription regulator |
| HOXB9 | ENSG00000170689 | homeobox B9 | 0,686 | Nucleus | transcription regulator |
| HPN | ENSG00000105707 | hepsin | -0,670 | Plasma Membrane | peptidase |
| HRH1 | ENSG00000196639 | histamine receptor H1 | 1,104 | Plasma Membrane | G-protein coupled receptor |
| HSD3B1 | ENSG00000203857 | hydroxy-delta-5-steroid dehydrogenase, 3 beta- and steroid delta-isomerase 1 | -0,759 | Cytoplasm | enzyme |
| HSPA1L | ENSG00000226704 | heat shock protein family A (Hsp70) member 1 like | -1,179 | Cytoplasm | other |
| HSPE1-MOB4 | ENSG00000270757 | HSPE1-MOB4 readthrough | 0,660 | Other | other |
| ICAM1 | ENSG00000090339 | intercellular adhesion molecule 1 | 0,948 | Plasma Membrane | transmembrane receptor |
| ICAM4 | ENSG00000105371 | intercellular adhesion molecule 4 (Landsteiner-Wiener blood group) | -1,017 | Plasma Membrane | other |
| ID1 | ENSG00000125968 | inhibitor of DNA binding 1, HLH protein | 0,859 | Nucleus | transcription regulator |
| IDH1 | ENSG00000138413 | isocitrate dehydrogenase (NADP(+)) 1 | -0,898 | Cytoplasm | enzyme |

| | | | | | |
|---|---|---|---|---|---|
| IER2 | ENSG00000160888 | immediate early response 2 | 1,264 | Cytoplasm | transcription regulator |
| IER3 | ENSG00000237155 | immediate early response 3 | 1,568 | Cytoplasm | other |
| IER5 | ENSG00000162783 | immediate early response 5 | 0,715 | Nucleus | other |
| IER5L | ENSG00000188483 | immediate early response 5 like | 0,679 | Other | other |
| IFRD1 | ENSG00000006652 | interferon related developmental regulator 1 | 0,654 | Nucleus | other |
| IGF2 | ENSG00000167244 | insulin like growth factor 2 | -0,774 | Extracellular Space | growth factor |
| IGFBP4 | ENSG00000141753 | insulin like growth factor binding protein 4 | 1,393 | Extracellular Space | other |
| IGSF9 | ENSG00000085552 | immunoglobulin superfamily member 9 | -0,607 | Plasma Membrane | other |
| IHH | ENSG00000163501 | Indian hedgehog signaling molecule | -0,675 | Extracellular Space | enzyme |
| IL11 | ENSG00000095752 | interleukin 11 | 2,218 | Extracellular Space | cytokine |
| IL17RE | ENSG00000163701 | interleukin 17 receptor E | -0,835 | Plasma Membrane | other |
| IL18 | ENSG00000150782 | interleukin 18 | 0,610 | Extracellular Space | cytokine |
| IL22RA1 | ENSG00000142677 | interleukin 22 receptor subunit alpha 1 | -1,017 | Plasma Membrane | transmembrane receptor |
| INHBA | ENSG00000122641 | inhibin subunit beta A | 1,171 | Extracellular Space | growth factor |
| INPP1 | ENSG00000151689 | inositol polyphosphate-1-phosphatase | 0,807 | Cytoplasm | phosphatase |
| IQCN | ENSG00000130518 | IQ motif containing N | 0,775 | Cytoplasm | other |
| IRF1 | ENSG00000125347 | interferon regulatory factor 1 | 0,643 | Nucleus | transcription regulator |
| ISG20L2 | ENSG00000143319 | interferon stimulated exonuclease gene 20 like 2 | 0,624 | Nucleus | enzyme |
| ITGA2 | ENSG00000164171 | integrin subunit alpha 2 | 1,741 | Plasma Membrane | transmembrane receptor |
| ITGA3 | ENSG00000005884 | integrin subunit alpha 3 | 1,183 | Plasma Membrane | other |
| ITGA5 | ENSG00000161638 | integrin subunit alpha 5 | 0,809 | Plasma Membrane | transmembrane receptor |
| ITGA6 | ENSG00000091409 | integrin subunit alpha 6 | 1,258 | Plasma Membrane | transmembrane receptor |
| ITGA7 | ENSG00000135424 | integrin subunit alpha 7 | -0,700 | Plasma Membrane | other |
| ITPRID2 | ENSG00000138434 | ITPR interacting domain containing 2 | 0,619 | Plasma Membrane | other |
| IYD | ENSG00000009765 | iodotyrosine deiodinase | -0,626 | Plasma Membrane | enzyme |
| JCAD | ENSG00000165757 | junctional cadherin 5 associated | 0,727 | Plasma Membrane | other |
| JUNB | ENSG00000171223 | JunB proto-oncogene, AP-1 transcription factor subunit | 1,893 | Nucleus | transcription regulator |
| KANK2 | ENSG00000197256 | KN motif and ankyrin repeat domains 2 | -0,787 | Nucleus | transcription regulator |
| KBTBD7 | ENSG00000120696 | kelch repeat and BTB domain containing 7 | -0,619 | Cytoplasm | other |
| KIF12 | ENSG00000136883 | kinesin family member 12 | -0,783 | Cytoplasm | other |
| KIF26B | ENSG00000162849 | kinesin family member 26B | -0,914 | Other | other |
| KIFC3 | ENSG00000140859 | kinesin family member C3 | 0,736 | Cytoplasm | enzyme |
| KLC4 | ENSG00000137171 | kinesin light chain 4 | -1,400 | Other | other |
| KLF10 | ENSG00000155090 | Kruppel like factor 10 | 1,298 | Nucleus | transcription regulator |
| KLF4 | ENSG00000136826 | Kruppel like factor 4 | 1,066 | Nucleus | transcription regulator |
| KLF6 | ENSG00000067082 | Kruppel like factor 6 | 1,567 | Nucleus | transcription regulator |
| KLHDC10 | ENSG00000128607 | kelch domain containing 10 | 0,649 | Nucleus | other |
| KLHDC2 | ENSG00000165516 | kelch domain containing 2 | -0,909 | Cytoplasm | other |
| KLHL13 | ENSG00000003096 | kelch like family member 13 | -0,808 | Cytoplasm | other |
| KLHL18 | ENSG00000114648 | kelch like family member 18 | 0,645 | Other | other |
| KLHL21 | ENSG00000162413 | kelch like family member 21 | 0,620 | Cytoplasm | other |
| KRT17 | ENSG00000128422 | keratin 17 | 1,011 | Cytoplasm | other |
| KRT6A | ENSG00000205420 | keratin 6A | 2,119 | Cytoplasm | other |
| L1CAM | ENSG00000198910 | L1 cell adhesion molecule | 0,619 | Plasma Membrane | other |
| LAMA1 | ENSG00000101680 | laminin subunit alpha 1 | -2,276 | Extracellular Space | other |
| LAMC2 | ENSG00000058085 | laminin subunit gamma 2 | 1,061 | Extracellular Space | other |
| LATS1 | ENSG00000131023 | large tumor suppressor kinase 1 | 0,630 | Nucleus | kinase |
| LDHD | ENSG00000166816 | lactate dehydrogenase D | -0,864 | Cytoplasm | enzyme |
| LDLR | ENSG00000130164 | low density lipoprotein receptor | 1,015 | Plasma Membrane | transporter |
| LGALS2 | ENSG00000100079 | galectin 2 | -0,680 | Cytoplasm | other |
| LHX6 | ENSG00000106852 | LIM homeobox 6 | 0,946 | Nucleus | transcription regulator |
| LIF | ENSG00000128342 | LIF interleukin 6 family cytokine | 1,156 | Extracellular Space | cytokine |
| LINS1 | ENSG00000140471 | lines homolog 1 | 0,874 | Other | other |
| LOC101929141 | ENSG00000250111 | coiled-coil domain-containing protein 144A-like | 1,095 | Other | other |
| LPAR6 | ENSG00000139679 | lysophosphatidic acid receptor 6 | 0,656 | Plasma Membrane | G-protein coupled receptor |
| LRCH1 | ENSG00000136141 | leucine rich repeats and calponin homology domain containing 1 | 0,689 | Cytoplasm | other |
| LRFN4 | ENSG00000173621 | leucine rich repeat and fibronectin type III domain containing 4 | 0,698 | Plasma Membrane | other |
| LRP1 | ENSG00000123384 | LDL receptor related protein 1 | -0,688 | Plasma Membrane | transmembrane receptor |
| LRP2 | ENSG00000081479 | LDL receptor related protein 2 | -0,837 | Plasma Membrane | transporter |
| LRRC24 | ENSG00000254402 | leucine rich repeat containing 24 | -0,689 | Other | other |
| LRRN4 | ENSG00000125872 | leucine rich repeat neuronal 4 | -0,831 | Plasma Membrane | other |
| MAFF | ENSG00000185022 | MAF bZIP transcription factor F | 1,435 | Nucleus | transcription regulator |
| MAFG | ENSG00000197063 | MAF bZIP transcription factor G | 0,774 | Nucleus | transcription regulator |
| MAFK | ENSG00000198517 | MAF bZIP transcription factor K | 0,679 | Nucleus | transcription regulator |
| MAGED1 | ENSG00000179222 | MAGE family member D1 | -0,639 | Plasma Membrane | transcription regulator |
| MANSC1 | ENSG00000111261 | MANSC domain containing 1 | -0,827 | Other | other |
| MAP2K6 | ENSG00000108984 | mitogen-activated protein kinase kinase 6 | -1,021 | Cytoplasm | kinase |
| MATN3 | ENSG00000132031 | matrilin 3 | 0,784 | Extracellular Space | other |
| MCL1 | ENSG00000143384 | MCL1 apoptosis regulator, BCL2 family member | 0,750 | Cytoplasm | transporter |
| MDM2 | ENSG00000135679 | MDM2 proto-oncogene | 1,372 | Nucleus | transcription regulator |
| METTL7A | ENSG00000185432 | methyltransferase like 7A | -1,418 | Cytoplasm | other |
| MINDY1 | ENSG00000143409 | MINDY lysine 48 deubiquitinase 1 | -0,959 | Cytoplasm | peptidase |
| MMP1 | ENSG00000196611 | matrix metallopeptidase 1 | 1,530 | Extracellular Space | peptidase |
| MMP11 | ENSG00000099953 | matrix metallopeptidase 11 | -0,914 | Extracellular Space | peptidase |
| MOCS1 | ENSG00000124615 | molybdenum cofactor synthesis 1 | -0,610 | Cytoplasm | other |
| MON1A | ENSG00000164077 | MON1 homolog A, secretory trafficking associated | 0,733 | Cytoplasm | other |
| MOSMO | ENSG00000185716 | modulator of smoothened | 0,855 | Other | other |
| MPC2 | ENSG00000143158 | mitochondrial pyruvate carrier 2 | -0,954 | Plasma Membrane | other |
| MPP1 | ENSG00000130830 | membrane palmitoylated protein 1 | -0,618 | Plasma Membrane | kinase |
| MPZL2 | ENSG00000149573 | myelin protein zero like 2 | -0,621 | Plasma Membrane | other |
| MSRB1 | ENSG00000198736 | methionine sulfoxide reductase B1 | -0,913 | Nucleus | enzyme |
| MST1R | ENSG00000164078 | macrophage stimulating 1 receptor | 0,955 | Plasma Membrane | kinase |
| MTF1 | ENSG00000188786 | metal regulatory transcription factor 1 | 1,171 | Nucleus | transcription regulator |
| MTMR9 | ENSG00000104643 | myotubularin related protein 9 | 0,734 | Cytoplasm | phosphatase |
| MTPAP | ENSG00000107951 | mitochondrial poly(A) polymerase | 0,623 | Cytoplasm | enzyme |
| MUC13 | ENSG00000173702 | mucin 13, cell surface associated | 1,180 | Extracellular Space | other |
| MVD | ENSG00000167508 | mevalonate diphosphate decarboxylase | -0,719 | Cytoplasm | enzyme |
| MXD1 | ENSG00000059728 | MAX dimerization protein 1 | 1,335 | Nucleus | transcription regulator |
| MXD4 | ENSG00000123933 | MAX dimerization protein 4 | -1,159 | Nucleus | transcription regulator |

| | | | | | |
|---|---|---|---|---|---|
| MYBL1 | ENSG00000185697 | MYB proto-oncogene like 1 | 0,678 | Nucleus | transcription regulator |
| MYBPH | ENSG00000133055 | myosin binding protein H | -1,279 | Cytoplasm | other |
| MYC | ENSG00000136997 | MYC proto-oncogene, bHLH transcription factor | 1,081 | Nucleus | transcription regulator |
| MYCN | ENSG00000134323 | MYCN proto-oncogene, bHLH transcription factor | -0,995 | Nucleus | transcription regulator |
| MYO15B | ENSG00000266714 | myosin XVB | 1,047 | Other | other |
| MYO7A | ENSG00000137474 | myosin VIIA | -0,693 | Cytoplasm | enzyme |
| MYORG | ENSG00000164976 | myogenesis regulating glycosidase (putative) | -0,739 | Nucleus | other |
| MYSM1 | ENSG00000162601 | Myb like, SWIRM and MPN domains 1 | 0,786 | Nucleus | enzyme |
| N4BP3 | ENSG00000145911 | NEDD4 binding protein 3 | 0,751 | Cytoplasm | other |
| NABP1 | ENSG00000173559 | nucleic acid binding protein 1 | 0,894 | Nucleus | other |
| NAV2 | ENSG00000166833 | neuron navigator 2 | -1,046 | Nucleus | other |
| NAV3 | ENSG00000067798 | neuron navigator 3 | 1,512 | Nucleus | other |
| NBPF10 (includes others) | ENSG00000270231 | NBPF member 19 | 0,913 | Other | other |
| NBR1 | ENSG00000188554 | NBR1 autophagy cargo receptor | -0,701 | Cytoplasm | other |
| NCAPD2P1 | ENSG00000230356 | non-SMC condensin I complex subunit D2 pseudogene 1 | -0,640 | Other | other |
| NCEH1 | ENSG00000144959 | neutral cholesterol ester hydrolase 1 | 0,805 | Plasma Membrane | enzyme |
| NDNF | ENSG00000173376 | neuron derived neurotrophic factor | -0,724 | Extracellular Space | other |
| NDRG1 | ENSG00000104419 | N-myc downstream regulated 1 | -1,137 | Nucleus | kinase |
| NEU4 | ENSG00000204099 | neuraminidase 4 | -1,052 | Cytoplasm | enzyme |
| NFAT5 | ENSG00000102908 | nuclear factor of activated T cells 5 | 0,606 | Nucleus | transcription regulator |
| NFKB2 | ENSG00000077150 | nuclear factor kappa B subunit 2 | 0,931 | Nucleus | transcription regulator |
| NFKBIE | ENSG00000146232 | NFKB inhibitor epsilon | 0,809 | Nucleus | transcription regulator |
| NFKBIZ | ENSG00000144802 | NFKB inhibitor zeta | 0,674 | Nucleus | transcription regulator |
| NIP7 | ENSG00000132603 | nucleolar pre-rRNA processing protein NIP7 | 0,676 | Nucleus | other |
| NIPSNAP1 | ENSG00000184117 | nipsnap homolog 1 | -0,852 | Cytoplasm | enzyme |
| NKX1-2 | ENSG00000229544 | NK1 homeobox 2 | -0,836 | Nucleus | transcription regulator |
| NLE1 | ENSG00000073536 | notchless homolog 1 | 0,668 | Nucleus | enzyme |
| NOCT | ENSG00000151014 | nocturnin | 1,031 | Nucleus | transcription regulator |
| NOL4L | ENSG00000197183 | nucleolar protein 4 like | -0,775 | Other | other |
| NOTCH3 | ENSG00000074181 | notch receptor 3 | -0,653 | Plasma Membrane | transcription regulator |
| NPIPA9 | ENSG00000233024 | nuclear pore complex interacting protein family, member A9 | 1,231 | Other | other |
| NPIPB13 | ENSG00000198064 | nuclear pore complex interacting protein family, member B13 | 1,739 | Nucleus | other |
| NPTXR | ENSG00000221890 | neuronal pentraxin receptor | -0,729 | Plasma Membrane | transmembrane receptor |
| NR1D1 | ENSG00000126368 | nuclear receptor subfamily 1 group D member 1 | 1,680 | Nucleus | ligand-dependent nuclear receptor |
| NR4A1 | ENSG00000123358 | nuclear receptor subfamily 4 group A member 1 | 0,691 | Nucleus | ligand-dependent nuclear receptor |
| NRXN3 | ENSG00000021645 | neurexin 3 | -0,693 | Plasma Membrane | transporter |
| NSRP1 | ENSG00000126653 | nuclear speckle splicing regulatory protein 1 | 0,636 | Nucleus | other |
| NSUN5 | ENSG00000130305 | NOP2/Sun RNA methyltransferase 5 | 0,680 | Nucleus | enzyme |
| NSUN7 | ENSG00000179299 | NOP2/Sun RNA methyltransferase family member 7 | -0,906 | Other | other |
| NTN4 | ENSG00000074527 | netrin 4 | -0,679 | Extracellular Space | other |
| NUAK2 | ENSG00000163545 | NUAK family kinase 2 | 0,847 | Nucleus | kinase |
| NUFIP2 | ENSG00000108256 | nuclear FMR1 interacting protein 2 | 0,658 | Cytoplasm | other |
| NUTM2D | ENSG00000214562 | NUT family member 2D | -0,872 | Other | other |
| OAT | ENSG00000065154 | ornithine aminotransferase | -1,097 | Cytoplasm | enzyme |
| OSGEPL1 | ENSG00000128694 | O-sialoglycoprotein endopeptidase like 1 | -0,753 | Cytoplasm | peptidase |
| OSR2 | ENSG00000164920 | odd-skipped related transciption factor 2 | -0,664 | Nucleus | transcription regulator |
| P2RY2 | ENSG00000175591 | purinergic receptor P2Y2 | -0,755 | Plasma Membrane | G-protein coupled receptor |
| P2RY6 | ENSG00000171631 | pyrimidinergic receptor P2Y6 | -0,699 | Plasma Membrane | G-protein coupled receptor |
| PAH | ENSG00000171759 | phenylalanine hydroxylase | -0,717 | Cytoplasm | enzyme |
| PAIP2B | ENSG00000124374 | poly(A) binding protein interacting protein 2B | -0,704 | Other | translation regulator |
| PAK1 | ENSG00000149269 | p21 (RAC1) activated kinase 1 | -0,832 | Cytoplasm | kinase |
| PAK6 | ENSG00000137843 | p21 (RAC1) activated kinase 6 | -0,636 | Cytoplasm | kinase |
| PALD1 | ENSG00000107719 | phosphatase domain containing paladin 1 | 1,075 | Cytoplasm | phosphatase |
| PAQR8 | ENSG00000170915 | progestin and adipoQ receptor family member 8 | -0,714 | Plasma Membrane | other |
| PARD6B | ENSG00000124171 | par-6 family cell polarity regulator beta | 0,927 | Plasma Membrane | other |
| PBX1 | ENSG00000185630 | PBX homeobox 1 | -0,918 | Nucleus | transcription regulator |
| PBX2 | ENSG00000224952 | PBX homeobox 2 | -0,988 | Nucleus | transcription regulator |
| PBXIP1 | ENSG00000163346 | PBX homeobox interacting protein 1 | -1,134 | Nucleus | transcription regulator |
| PCYT1B | ENSG00000102230 | phosphate cytidylyltransferase 1, choline, beta | -0,709 | Cytoplasm | enzyme |
| PDCD4 | ENSG00000150593 | programmed cell death 4 | -1,245 | Nucleus | other |
| PDE6A | ENSG00000132915 | phosphodiesterase 6A | -0,652 | Plasma Membrane | enzyme |
| PDGFB | ENSG00000100311 | platelet derived growth factor subunit B | 0,701 | Extracellular Space | growth factor |
| PDZD3 | ENSG00000172367 | PDZ domain containing 3 | -0,921 | Plasma Membrane | transporter |
| PDZK1 | ENSG00000174827 | PDZ domain containing 1 | -1,267 | Plasma Membrane | other |
| PER2 | ENSG00000132326 | period circadian regulator 2 | 0,735 | Nucleus | transcription regulator |
| PHC2 | ENSG00000134686 | polyhomeotic homolog 2 | 0,631 | Nucleus | other |
| PHEX | ENSG00000102174 | phosphate regulating endopeptidase homolog X-linked | -0,814 | Cytoplasm | peptidase |
| PHGDH | ENSG00000092621 | phosphoglycerate dehydrogenase | -0,892 | Cytoplasm | enzyme |
| PHLDA1 | ENSG00000139289 | pleckstrin homology like domain family A member 1 | 0,782 | Cytoplasm | other |
| PHLDA2 | ENSG00000181649 | pleckstrin homology like domain family A member 2 | 1,421 | Cytoplasm | other |
| PHYHIPL | ENSG00000165443 | phytanoyl-CoA 2-hydroxylase interacting protein like | -0,676 | Cytoplasm | other |
| PIGZ | ENSG00000119227 | phosphatidylinositol glycan anchor biosynthesis class Z | -0,766 | Cytoplasm | enzyme |
| PIK3C2B | ENSG00000133056 | phosphatidylinositol-4-phosphate 3-kinase catalytic subunit type 2 beta | -0,640 | Cytoplasm | kinase |
| PIM3 | ENSG00000198355 | Pim-3 proto-oncogene, serine/threonine kinase | 0,908 | Cytoplasm | kinase |
| PLA2G12B | ENSG00000138308 | phospholipase A2 group XIIB | -1,078 | Extracellular Space | enzyme |
| PLAUR | ENSG00000011422 | plasminogen activator, urokinase receptor | 1,134 | Plasma Membrane | transmembrane receptor |
| PLD6 | ENSG00000179598 | phospholipase D family member 6 | 0,844 | Cytoplasm | enzyme |
| PLEC | ENSG00000178209 | plectin | 0,879 | Cytoplasm | other |
| PLEK2 | ENSG00000100558 | pleckstrin 2 | 0,647 | Plasma Membrane | other |
| PLEKHG3 | ENSG00000126822 | pleckstrin homology and RhoGEF domain containing G3 | 0,720 | Other | other |
| PLEKHG6 | ENSG00000008323 | pleckstrin homology and RhoGEF domain containing G6 | -0,832 | Cytoplasm | other |
| PLEKHN1 | ENSG00000187583 | pleckstrin homology domain containing N1 | 0,709 | Cytoplasm | other |
| PLK2 | ENSG00000145632 | polo like kinase 2 | 1,560 | Nucleus | kinase |
| PLK3 | ENSG00000173846 | polo like kinase 3 | 1,073 | Nucleus | kinase |
| PLPP1 | ENSG00000067113 | phospholipid phosphatase 1 | -0,900 | Plasma Membrane | phosphatase |
| PLXNC1 | ENSG00000136040 | plexin C1 | -1,106 | Plasma Membrane | transmembrane receptor |
| PMM1 | ENSG00000100417 | phosphomannomutase 1 | -0,960 | Cytoplasm | enzyme |
| PPP1R15A | ENSG00000087074 | protein phosphatase 1 regulatory subunit 15A | 1,531 | Cytoplasm | other |
| PPP1R3G | ENSG00000219607 | protein phosphatase 1 regulatory subunit 3G | -0,681 | Cytoplasm | other |

| | | | | | |
|---|---|---|---|---|---|
| PPP2R2B | ENSG00000156475 | protein phosphatase 2 regulatory subunit Bbeta | -0,878 | Cytoplasm | phosphatase |
| PRR15L | ENSG00000167183 | proline rich 15 like | -1,147 | Other | other |
| PRR7 | ENSG00000131188 | proline rich 7, synaptic | 0,827 | Plasma Membrane | other |
| PRRG2 | ENSG00000126460 | proline rich and Gla domain 2 | -0,678 | Extracellular Space | other |
| PRSS16 | ENSG00000112812 | serine protease 16 | -0,771 | Extracellular Space | peptidase |
| PRUNE1 | ENSG00000143363 | prune exopolyphosphatase 1 | -0,609 | Nucleus | enzyme |
| PSD4 | ENSG00000125637 | pleckstrin and Sec7 domain containing 4 | -0,688 | Cytoplasm | other |
| PSPH | ENSG00000146733 | phosphoserine phosphatase | -0,630 | Cytoplasm | phosphatase |
| PTGS2 | ENSG00000073756 | prostaglandin-endoperoxide synthase 2 | 1,307 | Cytoplasm | enzyme |
| PXMP4 | ENSG00000101417 | peroxisomal membrane protein 4 | -0,844 | Cytoplasm | other |
| QPRT | ENSG00000103485 | quinolinate phosphoribosyltransferase | -0,842 | Cytoplasm | enzyme |
| RAB3D | ENSG00000105514 | RAB3D, member RAS oncogene family | -0,718 | Cytoplasm | enzyme |
| RAB5B | ENSG00000111540 | RAB5B, member RAS oncogene family | -0,736 | Cytoplasm | enzyme |
| RASA3 | ENSG00000185989 | RAS p21 protein activator 3 | 1,189 | Plasma Membrane | ion channel |
| RASA4 | ENSG00000105808 | RAS p21 protein activator 4 | -0,971 | Cytoplasm | other |
| RASAL2 | ENSG00000075391 | RAS protein activator like 2 | 0,791 | Extracellular Space | other |
| RASGRF2 | ENSG00000113319 | Ras protein specific guanine nucleotide releasing factor 2 | 0,600 | Cytoplasm | other |
| RASL11A | ENSG00000122035 | RAS like family 11 member A | -0,825 | Nucleus | other |
| RASL12 | ENSG00000103710 | RAS like family 12 | -0,657 | Other | enzyme |
| RASSF6 | ENSG00000169435 | Ras association domain family member 6 | 0,902 | Other | other |
| RBBP8NL | ENSG00000130701 | RBBP8 N-terminal like | -1,227 | Extracellular Space | other |
| RBM47 | ENSG00000163694 | RNA binding motif protein 47 | -0,704 | Nucleus | other |
| RCL1 | ENSG00000120158 | RNA terminal phosphate cyclase like 1 | 0,638 | Nucleus | enzyme |
| REEP1 | ENSG00000068615 | receptor accessory protein 1 | -0,655 | Cytoplasm | other |
| REEP6 | ENSG00000115255 | receptor accessory protein 6 | -0,852 | Plasma Membrane | other |
| RELB | ENSG00000104856 | RELB proto-oncogene, NF-kB subunit | 0,806 | Nucleus | transcription regulator |
| REV3L | ENSG00000009413 | REV3 like, DNA directed polymerase zeta catalytic subunit | 0,604 | Nucleus | enzyme |
| REXO1 | ENSG00000079313 | RNA exonuclease 1 homolog | 0,630 | Nucleus | transcription regulator |
| RGN | ENSG00000130988 | regucalcin | -1,302 | Nucleus | enzyme |
| RGS2 | ENSG00000116741 | regulator of G protein signaling 2 | 1,576 | Nucleus | enzyme |
| RHBG | ENSG00000132677 | Rh family B glycoprotein (gene/pseudogene) | -0,935 | Plasma Membrane | transporter |
| RHOB | ENSG00000143878 | ras homolog family member B | 1,037 | Cytoplasm | enzyme |
| RHOBTB2 | ENSG00000008853 | Rho related BTB domain containing 2 | -0,706 | Cytoplasm | enzyme |
| RIMKLA | ENSG00000177181 | ribosomal modification protein rimK like family member A | -0,627 | Cytoplasm | enzyme |
| RIMS4 | ENSG00000101098 | regulating synaptic membrane exocytosis 4 | -0,781 | Plasma Membrane | other |
| RIN1 | ENSG00000174791 | Ras and Rab interactor 1 | 0,877 | Cytoplasm | other |
| RMI2 | ENSG00000175643 | RecQ mediated genome instability 2 | -1,048 | Nucleus | other |
| RNASE1 | ENSG00000129538 | ribonuclease A family member 1, pancreatic | -0,960 | Extracellular Space | enzyme |
| RNASE4 | ENSG00000258818 | ribonuclease A family member 4 | -0,860 | Extracellular Space | enzyme |
| RND1 | ENSG00000172602 | Rho family GTPase 1 | -0,686 | Cytoplasm | enzyme |
| RND3 | ENSG00000115963 | Rho family GTPase 3 | 0,727 | Cytoplasm | enzyme |
| RNF32 | ENSG00000105982 | ring finger protein 32 | -0,798 | Cytoplasm | other |
| ROM1 | ENSG00000149489 | retinal outer segment membrane protein 1 | -1,084 | Plasma Membrane | other |
| RPL23AP42 | ENSG00000234851 | ribosomal protein L23a pseudogene 42 | 2,892 | Other | other |
| RRP9 | ENSG00000114767 | ribosomal RNA processing 9, U3 small nucleolar RNA binding protein | 0,672 | Nucleus | other |
| RSC1A1 | ENSG00000215695 | regulator of solute carriers 1 | 1,609 | Nucleus | other |
| RUNX1 | ENSG00000159216 | RUNX family transcription factor 1 | 0,984 | Nucleus | transcription regulator |
| RWDD2A | ENSG00000013392 | RWD domain containing 2A | -0,639 | Other | other |
| RXRA | ENSG00000186350 | retinoid X receptor alpha | -0,620 | Nucleus | ligand-dependent nuclear receptor |
| SACS | ENSG00000151835 | sacsin molecular chaperone | 0,646 | Plasma Membrane | other |
| SAV1 | ENSG00000151748 | salvador family WW domain containing protein 1 | 0,655 | Cytoplasm | other |
| SBK1 | ENSG00000188322 | SH3 domain binding kinase 1 | -0,637 | Other | kinase |
| SCGN | ENSG00000079689 | secretagogin, EF-hand calcium binding protein | -0,832 | Cytoplasm | other |
| SCRN2 | ENSG00000141295 | secernin 2 | -0,897 | Extracellular Space | other |
| SDC2 | ENSG00000169439 | syndecan 2 | -0,697 | Plasma Membrane | other |
| SELENBP1 | ENSG00000143416 | selenium binding protein 1 | -0,800 | Cytoplasm | other |
| SELENOW | ENSG00000178980 | selenoprotein W | 0,708 | Cytoplasm | enzyme |
| SEMA4G | ENSG00000095539 | semaphorin 4G | -0,633 | Plasma Membrane | other |
| SEPHS2 | ENSG00000179918 | selenophosphate synthetase 2 | -0,928 | Cytoplasm | enzyme |
| SERINC5 | ENSG00000164300 | serine incorporator 5 | -1,119 | Plasma Membrane | transporter |
| SERPINA10 | ENSG00000140093 | serpin family A member 10 | -1,120 | Extracellular Space | other |
| SERPINA4 | ENSG00000100665 | serpin family A member 4 | -0,761 | Extracellular Space | other |
| SERPINA5 | ENSG00000188488 | serpin family A member 5 | -0,819 | Extracellular Space | other |
| SERPINA6 | ENSG00000170099 | serpin family A member 6 | -0,930 | Extracellular Space | other |
| SERPINE1 | ENSG00000106366 | serpin family E member 1 | 2,045 | Extracellular Space | other |
| SERPINF2 | ENSG00000167711 | serpin family F member 2 | -1,185 | Extracellular Space | other |
| SERTAD1 | ENSG00000197019 | SERTA domain containing 1 | 0,679 | Nucleus | transcription regulator |
| SESN1 | ENSG00000080546 | sestrin 1 | -0,845 | Nucleus | other |
| SESN3 | ENSG00000149212 | sestrin 3 | -0,855 | Extracellular Space | other |
| SETX | ENSG00000107290 | senataxin | 0,837 | Nucleus | enzyme |
| SFN | ENSG00000175793 | stratifin | 0,938 | Cytoplasm | other |
| SGSM3 | ENSG00000100359 | small G protein signaling modulator 3 | -0,688 | Plasma Membrane | other |
| SH2B3 | ENSG00000111252 | SH2B adaptor protein 3 | 0,840 | Plasma Membrane | other |
| SH2D3A | ENSG00000125731 | SH2 domain containing 3A | 1,142 | Cytoplasm | other |
| SH2D5 | ENSG00000189410 | SH2 domain containing 5 | 1,637 | Plasma Membrane | other |
| SH3BGRL | ENSG00000131171 | SH3 domain binding glutamate rich protein like | -0,629 | Cytoplasm | other |
| SH3RF1 | ENSG00000154447 | SH3 domain containing ring finger 1 | 0,642 | Cytoplasm | enzyme |
| SHB | ENSG00000107338 | SH2 domain containing adaptor protein B | 0,668 | Cytoplasm | other |
| SHLD2P3 | ENSG00000189014 | shieldin complex subunit 2 pseudogene 3 | -0,858 | Other | other |
| SLC16A3 | ENSG00000141526 | solute carrier family 16 member 3 | 0,725 | Plasma Membrane | transporter |
| SLC16A4 | ENSG00000168679 | solute carrier family 16 member 4 | -0,628 | Plasma Membrane | transporter |
| SLC19A3 | ENSG00000135917 | solute carrier family 19 member 3 | -0,712 | Plasma Membrane | transporter |
| SLC22A1 | ENSG00000175003 | solute carrier family 22 member 1 | 0,856 | Plasma Membrane | transporter |
| SLC25A19 | ENSG00000125454 | solute carrier family 25 member 19 | 0,630 | Cytoplasm | transporter |
| SLC25A25 | ENSG00000148339 | solute carrier family 25 member 25 | 0,984 | Cytoplasm | transporter |
| SLC25A32 | ENSG00000164933 | solute carrier family 25 member 32 | 0,672 | Cytoplasm | transporter |
| SLC25A42 | ENSG00000181035 | solute carrier family 25 member 42 | -0,921 | Cytoplasm | transporter |
| SLC26A2 | ENSG00000155850 | solute carrier family 26 member 2 | -1,024 | Plasma Membrane | transporter |
| SLC29A4 | ENSG00000164638 | solute carrier family 29 member 4 | -1,320 | Plasma Membrane | transporter |

| | | | | | |
|---|---|---|---|---|---|
| SLC2A1 | ENSG00000117394 | solute carrier family 2 member 1 | 0,787 | Plasma Membrane | transporter |
| SLC2A11 | ENSG00000133460 | solute carrier family 2 member 11 | -0,775 | Plasma Membrane | transporter |
| SLC30A1 | ENSG00000170385 | solute carrier family 30 member 1 | 0,641 | Plasma Membrane | transporter |
| SLC30A7 | ENSG00000162695 | solute carrier family 30 member 7 | 0,641 | Cytoplasm | transporter |
| SLC39A5 | ENSG00000139540 | solute carrier family 39 member 5 | -0,734 | Plasma Membrane | transporter |
| SLC3A1 | ENSG00000138079 | solute carrier family 3 member 1 | -0,823 | Plasma Membrane | transporter |
| SLC40A1 | ENSG00000138449 | solute carrier family 40 member 1 | -0,798 | Plasma Membrane | transporter |
| SLC4A7 | ENSG00000033867 | solute carrier family 4 member 7 | 0,846 | Plasma Membrane | transporter |
| SLC4A8 | ENSG00000050438 | solute carrier family 4 member 8 | -0,921 | Plasma Membrane | transporter |
| SLC7A11 | ENSG00000151012 | solute carrier family 7 member 11 | 0,680 | Plasma Membrane | transporter |
| SLC9A2 | ENSG00000115616 | solute carrier family 9 member A2 | 0,681 | Plasma Membrane | transporter |
| SLC9A3R1 | ENSG00000109062 | SLC9A3 regulator 1 | -0,703 | Plasma Membrane | other |
| SLCO2B1 | ENSG00000137491 | solute carrier organic anion transporter family member 2B1 | -0,757 | Plasma Membrane | transporter |
| SMIM14 | ENSG00000163683 | small integral membrane protein 14 | -1,217 | Cytoplasm | other |
| SMTNL2 | ENSG00000188176 | smoothelin like 2 | -1,135 | Other | other |
| SNAPC4 | ENSG00000165684 | small nuclear RNA activating complex polypeptide 4 | 0,743 | Nucleus | transcription regulator |
| SNX33 | ENSG00000173548 | sorting nexin 33 | -0,673 | Cytoplasm | other |
| SOAT2 | ENSG00000167780 | sterol O-acyltransferase 2 | -1,060 | Cytoplasm | enzyme |
| SOCS3 | ENSG00000184557 | suppressor of cytokine signaling 3 | 1,454 | Cytoplasm | phosphatase |
| SORBS2 | ENSG00000154556 | sorbin and SH3 domain containing 2 | -1,425 | Plasma Membrane | other |
| SOSTDC1 | ENSG00000171243 | sclerostin domain containing 1 | -0,605 | Extracellular Space | growth factor |
| SOWAHA | ENSG00000198944 | sosondowah ankyrin repeat domain family member A | -0,692 | Other | other |
| SOWAHC | ENSG00000198142 | sosondowah ankyrin repeat domain family member C | 0,735 | Nucleus | transcription regulator |
| SPATA2 | ENSG00000158480 | spermatogenesis associated 2 | 0,767 | Extracellular Space | other |
| SPEN | ENSG00000065526 | spen family transcriptional repressor | 0,614 | Nucleus | transcription regulator |
| SPIRE1 | ENSG00000134278 | spire type actin nucleation factor 1 | 0,623 | Cytoplasm | other |
| SPRED1 | ENSG00000166068 | sprouty related EVH1 domain containing 1 | 0,681 | Plasma Membrane | other |
| SPRY1 | ENSG00000164056 | sprouty RTK signaling antagonist 1 | 0,694 | Cytoplasm | other |
| SPRY2 | ENSG00000136158 | sprouty RTK signaling antagonist 2 | 0,814 | Plasma Membrane | other |
| SPRY4 | ENSG00000187678 | sprouty RTK signaling antagonist 4 | 1,532 | Plasma Membrane | other |
| SPTLC3 | ENSG00000172296 | serine palmitoyltransferase long chain base subunit 3 | -1,628 | Cytoplasm | enzyme |
| SPTSSB | ENSG00000196542 | serine palmitoyltransferase small subunit B | -0,615 | Cytoplasm | enzyme |
| SRC | ENSG00000197122 | SRC proto-oncogene, non-receptor tyrosine kinase | -0,701 | Cytoplasm | kinase |
| STC2 | ENSG00000113739 | stanniocalcin 2 | 0,754 | Extracellular Space | other |
| STK39 | ENSG00000198648 | serine/threonine kinase 39 | 0,850 | Nucleus | kinase |
| SULT1C2 | ENSG00000198203 | sulfotransferase family 1C member 2 | -0,791 | Cytoplasm | enzyme |
| SULT1C4 | ENSG00000198075 | sulfotransferase family 1C member 4 | -0,607 | Cytoplasm | enzyme |
| SULT1E1 | ENSG00000109193 | sulfotransferase family 1E member 1 | -0,613 | Cytoplasm | enzyme |
| SUOX | ENSG00000139531 | sulfite oxidase | -0,747 | Cytoplasm | enzyme |
| TAF1A | ENSG00000143498 | TATA-box binding protein associated factor, RNA polymerase I subunit A | 0,676 | Nucleus | transcription regulator |
| TAGLN | ENSG00000149591 | transgelin | 0,699 | Cytoplasm | other |
| TAP2 | ENSG00000204267 | transporter 2, ATP binding cassette subfamily B member | 0,723 | Cytoplasm | transporter |
| TBC1D2 | ENSG00000095383 | TBC1 domain family member 2 | 1,175 | Cytoplasm | other |
| TBX3 | ENSG00000135111 | T-box transcription factor 3 | 0,986 | Nucleus | transcription regulator |
| TDRKH | ENSG00000182134 | tudor and KH domain containing | -0,662 | Cytoplasm | other |
| TENT5A | ENSG00000112773 | terminal nucleotidyltransferase 5A | 0,746 | Other | other |
| TESK2 | ENSG00000070759 | testis associated actin remodelling kinase 2 | -0,797 | Nucleus | kinase |
| TGFB3 | ENSG00000119699 | transforming growth factor beta 3 | -0,672 | Extracellular Space | growth factor |
| TGM2 | ENSG00000198959 | transglutaminase 2 | 0,934 | Cytoplasm | enzyme |
| THAP8 | ENSG00000161277 | THAP domain containing 8 | -0,607 | Other | other |
| THRA | ENSG00000126351 | thyroid hormone receptor alpha | -0,650 | Nucleus | ligand-dependent nuclear receptor |
| THYN1 | ENSG00000151500 | thymocyte nuclear protein 1 | -0,644 | Nucleus | other |
| TINAGL1 | ENSG00000142910 | tubulointerstitial nephritis antigen like 1 | 1,019 | Extracellular Space | transporter |
| TIPARP | ENSG00000163659 | TCDD inducible poly(ADP-ribose) polymerase | 0,993 | Nucleus | enzyme |
| TJP3 | ENSG00000105289 | tight junction protein 3 | -1,147 | Cytoplasm | other |
| TM4SF1 | ENSG00000169908 | transmembrane 4 L six family member 1 | 1,099 | Plasma Membrane | other |
| TM7SF2 | ENSG00000149809 | transmembrane 7 superfamily member 2 | -0,864 | Cytoplasm | enzyme |
| TMC6 | ENSG00000141524 | transmembrane channel like 6 | 0,728 | Cytoplasm | transporter |
| TMEM135 | ENSG00000166575 | transmembrane protein 135 | -0,657 | Cytoplasm | other |
| TMEM139 | ENSG00000178826 | transmembrane protein 139 | -0,771 | Other | other |
| TMEM141 | ENSG00000244187 | transmembrane protein 141 | -0,685 | Other | other |
| TMEM14A | ENSG00000096092 | transmembrane protein 14A | -0,756 | Cytoplasm | other |
| TMEM37 | ENSG00000171227 | transmembrane protein 37 | -1,264 | Plasma Membrane | ion channel |
| TMEM45B | ENSG00000151715 | transmembrane protein 45B | -0,769 | Extracellular Space | other |
| TMEM60 | ENSG00000135211 | transmembrane protein 60 | -0,730 | Other | other |
| TMPRSS2 | ENSG00000184012 | transmembrane serine protease 2 | -1,042 | Plasma Membrane | peptidase |
| TNFAIP3 | ENSG00000118503 | TNF alpha induced protein 3 | 1,305 | Nucleus | enzyme |
| TNFRSF10A | ENSG00000104689 | TNF receptor superfamily member 10a | 0,752 | Plasma Membrane | transmembrane receptor |
| TNFRSF10B | ENSG00000120889 | TNF receptor superfamily member 10b | 0,769 | Plasma Membrane | transmembrane receptor |
| TNFRSF12A | ENSG00000006327 | TNF receptor superfamily member 12A | 0,776 | Plasma Membrane | transmembrane receptor |
| TNS4 | ENSG00000131746 | tensin 4 | 1,867 | Cytoplasm | other |
| TPD52L1 | ENSG00000111907 | TPD52 like 1 | -0,708 | Cytoplasm | other |
| TRIB1 | ENSG00000173334 | tribbles pseudokinase 1 | 0,743 | Cytoplasm | kinase |
| TRIM8 | ENSG00000171206 | tripartite motif containing 8 | 0,610 | Nucleus | other |
| TRPC6 | ENSG00000137672 | transient receptor potential cation channel subfamily C member 6 | 0,696 | Plasma Membrane | ion channel |
| TRPV3 | ENSG00000167723 | transient receptor potential cation channel subfamily V member 3 | -0,645 | Plasma Membrane | ion channel |
| TSC22D2 | ENSG00000196428 | TSC22 domain family member 2 | 0,781 | Nucleus | transcription regulator |
| TTBK2 | ENSG00000128881 | tau tubulin kinase 2 | 0,747 | Cytoplasm | kinase |
| TUBB3 | ENSG00000258947 | tubulin beta 3 class III | 0,931 | Cytoplasm | other |
| TXNIP | ENSG00000265972 | thioredoxin interacting protein | 1,076 | Cytoplasm | other |
| TYMSOS | ENSG00000176912 | TYMS opposite strand | -0,930 | Nucleus | enzyme |
| UBASH3B | ENSG00000154127 | ubiquitin associated and SH3 domain containing B | 0,796 | Other | enzyme |
| UGDH | ENSG00000109814 | UDP-glucose 6-dehydrogenase | -0,773 | Nucleus | enzyme |
| UGT2A3 | ENSG00000135220 | UDP glucuronosyltransferase family 2 member A3 | -0,845 | Other | enzyme |
| ULBP2 | ENSG00000131015 | UL16 binding protein 2 | 0,740 | Plasma Membrane | transmembrane receptor |
| ULK1 | ENSG00000177169 | unc-51 like autophagy activating kinase 1 | -0,685 | Cytoplasm | kinase |
| UNC93A | ENSG00000112494 | unc-93 homolog A | -0,696 | Plasma Membrane | other |
| UPB1 | ENSG00000100024 | beta-ureidopropionase 1 | -0,611 | Cytoplasm | enzyme |
| UPP1 | ENSG00000183696 | uridine phosphorylase 1 | 0,929 | Cytoplasm | enzyme |

| | | | | | |
|---|---|---|---|---|---|
| UPRT | ENSG00000094841 | uracil phosphoribosyltransferase homolog | -0,744 | Nucleus | enzyme |
| USH1C | ENSG00000006611 | USH1 protein network component harmonin | -0,710 | Plasma Membrane | other |
| USP53 | ENSG00000145390 | ubiquitin specific peptidase 53 | 0,735 | Extracellular Space | enzyme |
| VAV3 | ENSG00000134215 | vav guanine nucleotide exchange factor 3 | -1,002 | Extracellular Space | cytokine |
| VCPKMT | ENSG00000100483 | valosin containing protein lysine methyltransferase | 0,651 | Cytoplasm | enzyme |
| VGLL3 | ENSG00000206538 | vestigial like family member 3 | 0,610 | Other | other |
| VIM | ENSG00000026025 | vimentin | 0,949 | Cytoplasm | other |
| VPS45 | ENSG00000136631 | vacuolar protein sorting 45 homolog | -1,015 | Cytoplasm | transporter |
| VSIR | ENSG00000107738 | V-set immunoregulatory receptor | 0,628 | Plasma Membrane | other |
| WBP1 | ENSG00000239779 | WW domain binding protein 1 | -0,959 | Cytoplasm | other |
| WNK4 | ENSG00000126562 | WNK lysine deficient protein kinase 4 | 1,186 | Plasma Membrane | kinase |
| XKRX | ENSG00000182489 | XK related X-linked | -0,898 | Other | other |
| YOD1 | ENSG00000180667 | YOD1 deubiquitinase | 0,785 | Cytoplasm | enzyme |
| YPEL5 | ENSG00000119801 | yippee like 5 | -0,960 | Nucleus | other |
| YRDC | ENSG00000196449 | yrdC N6-threonylcarbamoyltransferase domain containing | 0,926 | Other | other |
| ZBTB20 | ENSG00000181722 | zinc finger and BTB domain containing 20 | 0,969 | Nucleus | transcription regulator |
| ZC3H12A | ENSG00000163874 | zinc finger CCCH-type containing 12A | 0,967 | Cytoplasm | enzyme |
| ZC3H12C | ENSG00000149289 | zinc finger CCCH-type containing 12C | 0,718 | Other | other |
| ZFP36 | ENSG00000128016 | ZFP36 ring finger protein | 1,187 | Nucleus | transcription regulator |
| ZFP36L1 | ENSG00000185650 | ZFP36 ring finger protein like 1 | 0,637 | Nucleus | transcription regulator |
| ZMYM3 | ENSG00000147130 | zinc finger MYM-type containing 3 | -0,719 | Nucleus | other |
| ZNF142 | ENSG00000115568 | zinc finger protein 142 | 0,651 | Nucleus | transcription regulator |
| ZNF165 | ENSG00000197279 | zinc finger protein 165 | 0,835 | Nucleus | transcription regulator |
| ZNF239 | ENSG00000196793 | zinc finger protein 239 | 0,730 | Nucleus | transcription regulator |
| ZNF358 | ENSG00000198816 | zinc finger protein 358 | -0,601 | Nucleus | transcription regulator |
| ZNF362 | ENSG00000160094 | zinc finger protein 362 | -1,177 | Other | other |
| ZNF385A | ENSG00000161642 | zinc finger protein 385A | -0,859 | Nucleus | other |
| ZNF430 | ENSG00000118620 | zinc finger protein 430 | 0,787 | Nucleus | transcription regulator |
| ZNF441 | ENSG00000197044 | zinc finger protein 441 | 0,848 | Nucleus | transcription regulator |
| ZNF473 | ENSG00000142528 | zinc finger protein 473 | 0,623 | Nucleus | other |
| ZNF485 | ENSG00000198298 | zinc finger protein 485 | 0,821 | Nucleus | transcription regulator |
| ZNF556 | ENSG00000172000 | zinc finger protein 556 | 0,774 | Other | other |
| ZNF598 | ENSG00000167962 | zinc finger protein 598, E3 ubiquitin ligase | 0,647 | Extracellular Space | enzyme |
| ZNF710 | ENSG00000140548 | zinc finger protein 710 | 0,633 | Nucleus | transcription regulator |
| ZNF844 | ENSG00000223547 | zinc finger protein 844 | 1,432 | Other | other |
| ZRSR2P1 | ENSG00000212643 | ZRSR2 pseudogene 1 | 1,117 | Nucleus | other |

**Supplementary Table S9. List of Canonical Pathways for the condition "Calu-3_SARS-CoV-2 ".** The IPA-based Core Analysis was performed on the basis of differentially expressed genes dissected for a given condition by GENEVESTIGATOR-based analysis. Only statistically significant Canonical Pathways are included (n = 275). The data are taken as the basis for R-based visualization by Venn diagrams shown in Figure 5.

| Ingenuity Canonical Pathways | -log(p-valu | Molecules |
|---|---|---|
| Superpathway of Cholesterol Biosynthesis | 1,56E01 | ACAA2,ACAT2,CYP51A1,DHCR24,DHCR7,EBP,FDFT1,FDPS,HADHB,HMGCR,HMGCS1,HSD17B7,IDI1,LBR,LSS,MSMO1,MVD,MVK,NSDHL,PMVK,SC5D,SQLE,TM7SF2 |
| Dendritic Cell Maturation | 1,46E01 | ATF4,B2M,CD40,CD83,COL11A2,CREB1,CREB5,CREBBP,FSCN1,HLA-A,HLA-B,HLA-C,HLA-DMA,HLA-DMB,HLA-DOB,HLA-DQA1,HLA-DQB1,HLA-DRA,HLA-DRB1,HLA-DRB3,HLA-DRB4,HLA-E,HLA-F,ICAM1,[illegible] |
| Neuroinflammation Signaling Pathway | 1,46E01 | ACVR1C,AGER,ATF4,B2M,BDNF,BIRC2,BIRC3,BIRC5,CASP1,CASP8,CCL2,CCL5,CD40,CFLAR,CHP1,CREB1,CREB5,CREBBP,CRP,CSF1R,CX3CL1,CXCL10,CXCL8,FAS,FOS,GABRE,GABRP,GJB1,GRIN3A,[illegible] |
| Antigen Presentation Pathway | 1,33E01 | B2M,CD74,CIITA,HLA-A,HLA-B,HLA-C,HLA-DMA,HLA-DMB,HLA-DOB,HLA-DPA1,HLA-DPB1,HLA-DQA1,HLA-DQB1,HLA-DRA,HLA-DRB1,HLA-DRB3,HLA-DRB4,HLA-E,HLA-F,NLRC5,PSMB8,PSMB9,TAP1,TAP2,[illegible] |
| Role of Pattern Recognition Receptors in Recognition of Bacteria and Viruses | 1,2E01 | C3,C5,CASP1,CCL5,CLEC7A,CREB1,CXCL8,DDX58,EIF2AK2,IFIH1,IFNA1/IFNA13,IFNB1,IL11,IL12A,IL15,IL1A,IL1B,IL6,IRF7,LIF,LTB,MAPK3,MYD88,NFKB1,NFKB2,NLRP3,NOD1,NOD2,OAS1,OAS2,OAS3,[illegible] |
| Cholesterol Biosynthesis I | 1,18E01 | CYP51A1,DHCR24,DHCR7,EBP,FDFT1,HSD17B7,LBR,LSS,MSMO1,NSDHL,SC5D,SQLE,TM7SF2 |
| Cholesterol Biosynthesis II (via 24,25-dihydrolanosterol) | 1,18E01 | CYP51A1,DHCR24,DHCR7,EBP,FDFT1,HSD17B7,LBR,LSS,MSMO1,NSDHL,SC5D,SQLE,TM7SF2 |
| Cholesterol Biosynthesis III (via Desmosterol) | 1,18E01 | CYP51A1,DHCR24,DHCR7,EBP,FDFT1,HSD17B7,LBR,LSS,MSMO1,NSDHL,SC5D,SQLE,TM7SF2 |
| Activation of IRF by Cytosolic Pattern Recognition Receptors | 1,04E01 | ADAR,CD40,CREBBP,DDX58,DHX58,IFIH1,IFIT2,IFNA1/IFNA13,IFNA7,IFNB1,IKBKE,IL6,IRF7,IRF9,ISG15,JUN,NFKB1,NFKB2,NFKBIA,NFKBIB,NFKBID,NFKBIE,RIPK1,SIKE1,STAT1,STAT2,TNF,TRAF3,ZBP1 |
| MSP-RON Signaling In Macrophages Pathway | 1,03E01 | ATF4,CIITA,CREB1,CREB5,CREBBP,ETV3,F12,FOS,HLA-DMA,HLA-DMB,HLA-DOB,HLA-DPA1,HLA-DPB1,HLA-DQA1,HLA-DQB1,HLA-DRA,HLA-DRB1,HLA-DRB3,HLA-DRB4,IKBKE,IL12A,ITGAM,JAK2,JUN,[illegible] |
| Interferon Signaling | 1,02E01 | BAK1,IFI35,IFI6,IFIT1,IFIT3,IFITM1,IFITM2,IFITM3,IFNA1/IFNA13,IFNB1,IRF1,IRF9,ISG15,JAK2,MX1,OAS1,PSMB8,SOCS1,STAT1,STAT2,TAP1 |
| Role of Macrophages, Fibroblasts and Endothelial Cells in Rheumatoid Arthritis | 9,97E00 | ATF4,C5,CCL2,CCL5,CEBPB,CEBPD,CEBPZ,CHP1,CREB1,CREB5,CREBBP,CSF1,CXCL8,F2RL1,FGF2,FN1,FOS,FZD4,FZD5,ICAM1,IKBKE,IL15,IL18R1,IL1A,IL1B,IL1RN,IL32,IL6,IL7,IRAK1,IRAK2,JAK2,JUN,[illegible] |
| Death Receptor Signaling | 9,53E00 | ACTA2,ARHGDIB,BIRC2,BIRC3,CASP10,CASP7,CASP8,CFLAR,DAXX,FAS,GAS2,IKBKE,MAP4K4,NFKB1,NFKB2,NFKBIA,NFKBIB,NFKBID,NFKBIE,PARP10,PARP12,PARP14,PARP3,PARP8,PARP9,RIPK1,TIPARP,[illegible] |
| PD-1, PD-L1 cancer immunotherapy pathway | 9,4E00 | B2M,CD274,FBXO38,HLA-A,HLA-B,HLA-C,HLA-DMA,HLA-DMB,HLA-DOB,HLA-DPA1,HLA-DPB1,HLA-DQA1,HLA-DQB1,HLA-DRA,HLA-DRB1,HLA-DRB3,HLA-DRB4,HLA-E,HLA-F,IL12A,IL2RG,JAK2,LATS1,LATS2,[illegible] |
| Tumor Microenvironment Pathway | 9,03E00 | BAD,CCL2,CD274,CFLAR,CSF1,CSF3,CXCL8,FAS,FGF2,FN1,FOS,FOXO1,HLA-A,HLA-B,HLA-C,HLA-E,HLA-F,ICAM1,IDO1,IGF2,IL1B,IL6,ITGA5,JAK2,JUN,LEPR,LGALS9,MAPK3,MMP1,MMP25,MMP7,MYC,NFKB1,[illegible] |
| TREM1 Signaling | 8,88E00 | CASP1,CCL2,CD40,CD83,CIITA,CXCL3,CXCL8,ICAM1,IL1B,IL6,IRAK1,ITGA5,ITGB1,JAK2,LAT2,MAPK3,MYD88,NFKB1,NFKB2,NLRC5,NLRP3,NOD1,NOD2,PLCG2,STAT5A,TLR1,TLR2,TLR3,TLR5,TNF |
| Systemic Lupus Erythematosus In B Cell Signaling Pathway | 8,33E00 | BAD,BCL10,BCL2L11,CCND3,CD40,CHP1,CXCL8,FOS,FOXO1,IFIH1,IFIT2,IFIT3,IFNA1/IFNA13,IFNA7,IFNB1,IL11,IL12A,IL15,IL1A,IL1B,IL6,IRAK1,IRF7,IRF9,ISG15,ISG20,JAK2,JUN,LIF,LTB,LYN,MAP4K4,MAPK3,[illegible] |
| Acute Phase Response Signaling | 8,19E00 | AGT,C1R,C1S,C2,C3,C4A/C4B,C4BPB,C5,CEBPB,CFB,CRP,FN1,FOS,IKBKE,IL1A,IL1B,IL1RN,IL6,IRAK1,ITIH2,JAK2,JUN,MAP2K6,MAPK3,MYD88,NFKB1,NFKB2,NFKBIA,NFKBIB,NFKBID,NFKBIE,NGFR,NR3C1,[illegible] |
| T Cell Exhaustion Signaling Pathway | 8,17E00 | ACVR1C,BCL6,CD274,FOS,FOXO1,HLA-A,HLA-B,HLA-C,HLA-DMA,HLA-DMB,HLA-DOB,HLA-DPA1,HLA-DPB1,HLA-DQA1,HLA-DQB1,HLA-DRA,HLA-DRB1,HLA-DRB3,HLA-DRB4,HLA-E,HLA-F,IFNA1/IFNA13,IFNA7,[illegible] |
| Type I Diabetes Mellitus Signaling | 8,17E00 | CASP8,FAS,HLA-A,HLA-B,HLA-C,HLA-DMA,HLA-DMB,HLA-DOB,HLA-DQA1,HLA-DQB1,HLA-DRA,HLA-DRB1,HLA-E,HLA-F,IKBKE,IL12A,IL1B,IRAK1,IRF1,JAK2,MAP2K6,MYD88,NFKB1,NFKB2,NFKBIA,NFKBIB,[illegible] |
| Coronavirus Pathogenesis Pathway | 8,14E00 | ACE2,AGT,ATF4,BCL2L11,CASP1,CASP8,CCL2,CCL5,CXCL8,DDIT3,DDX58,E2F6,E2F7,EEF1A1,EEF1A2,FOS,IFNA1/IFNA13,IFNB1,IL1B,IL6,IRF7,IRF9,JUN,MAPK3,NFKB1,NFKB2,NFKBIA,NFKBIB,NFKBID,NFKBIE,[illegible] |
| Th1 Pathway | 8,11E00 | CD274,CD40,GATA3,HLA-A,HLA-B,HLA-DMA,HLA-DMB,HLA-DOB,HLA-DPA1,HLA-DPB1,HLA-DQA1,HLA-DQB1,HLA-DRA,HLA-DRB1,HLA-DRB3,HLA-DRB4,ICAM1,ICOSLG/LOC102723996,IFNA1/IFNA13,IL10RA,[illegible] |
| Crosstalk between Dendritic Cells and Natural Killer Cells | 8,06E00 | ACTA2,CD40,CD69,CD83,FAS,FSCN1,HLA-A,HLA-B,HLA-C,HLA-DRA,HLA-DRB1,HLA-DRB3,HLA-DRB4,HLA-E,HLA-F,IFNA1/IFNA13,IFNB1,IL12A,IL15,IL15RA,IL2RG,IL3RA,IL6,LTB,MICB,NECTIN2,NFKB1,NFKB2,[illegible] |
| Hepatic Fibrosis Signaling Pathway | 7,76E00 | ACTA2,ACVR1C,AGT,ATF4,CACNA1D,CCL2,CCL5,CD40,CEBPB,COL11A2,CREB1,CREB5,CREBBP,CXCL8,DIRAS3,EDN1,EDNRA,FGF2,FOS,FOXO1,FZD4,FZD5,GLIS2,ICAM1,IKBKE,IL1A,IL1B,IL1RN,IRAK1,[illegible] |
| Th1 and Th2 Activation Pathway | 7,61E00 | ACVR1C,CCR1,CCR4,CD274,CD40,GATA3,HAVCR1,HLA-A,HLA-B,HLA-DMA,HLA-DMB,HLA-DOB,HLA-DPA1,HLA-DPB1,HLA-DQA1,HLA-DQB1,HLA-DRA,HLA-DRB1,HLA-DRB3,HLA-DRB4,ICAM1,[illegible] |
| NF-κB Signaling | 7,37E00 | AZI2,BCL10,[illegible] |
| Altered T Cell and B Cell Signaling in Rheumatoid Arthritis | 7,33E00 | CD40,CSF1,FAS,[illegible] |
| Role of RIG1-like Receptors in Antiviral Innate Immunity | 7,24E00 | CASP10,CASP8,[illegible] |
| Aryl Hydrocarbon Receptor Signaling | 6,88E00 | AHR,AHRR,[illegible] |
| Myc Mediated Apoptosis Signaling | 6,84E00 | ADRB2,BBC3,[illegible] |
| Senescence Pathway | 6,84E00 | ACVR1C,[illegible] |
| Necroptosis Signaling Pathway | 6,56E00 | BIRC2,BIRC3,[illegible] |
| Hepatic Fibrosis / Hepatic Stellate Cell Activation | 6,39E00 | ACTA2,AGT,[illegible] |
| Role of PKR in Interferon Induction and Antiviral Response | 6,36E00 | ATF3,CASP1,[illegible] |
| Osteoarthritis Pathway | 6,34E00 | AGER,ANKH,[illegible] |
| TNFR2 Signaling | 6,24E00 | BIRC2,BIRC3,[illegible] |
| T Helper Cell Differentiation | 5,99E00 | BCL6,CD40,[illegible] |
| TWEAK Signaling | 5,95E00 | BIRC2,BIRC3,[illegible] |
| IL-17A Signaling in Fibroblasts | 5,95E00 | CCL2,CEBPB,[illegible] |
| VDR/RXR Activation | 5,94E00 | CASR,CCL5,[illegible] |
| Th2 Pathway | 5,67E00 | ACVR1C,CCR1,[illegible] |
| Apoptosis Signaling | 5,66E00 | BAD,BAK1,[illegible] |
| Toll-like Receptor Signaling | 5,62E00 | EIF2AK2,FOS,[illegible] |
| Communication between Innate and Adaptive Immune Cells | 5,56E00 | B2M,CCL5,CD40,[illegible] |
| Role of IL-17A in Arthritis | 5,51E00 | CCL2,CCL20,[illegible] |
| Zymosterol Biosynthesis | 5,45E00 | CYP51A1,[illegible] |
| CD40 Signaling | 5,25E00 | CD40,FOS,[illegible] |
| IL-6 Signaling | 5,18E00 | CEBPB,CRP,[illegible] |
| Induction of Apoptosis by HIV1 | 5,17E00 | BAK1,BBC3,[illegible] |
| Mevalonate Pathway I | 5,13E00 | ACAA2,ACAT2,[illegible] |
| Graft-versus-Host Disease Signaling | 5,11E00 | FAS,HLA-A,[illegible] |
| Superpathway of Geranylgeranyldiphosphate Biosynthesis I (via Mevalonate) | 4,86E00 | ACAA2,ACAT2,[illegible] |
| Molecular Mechanisms of Cancer | 4,84E00 | ARHGEF11,[illegible] |
| IL-9 Signaling | 4,82E00 | BCL3,IL2RG,[illegible] |
| HMGB1 Signaling | 4,74E00 | AGER,CCL2,[illegible] |
| Regulation Of The Epithelial Mesenchymal Transition By Growth Factors Pathway | 4,74E00 | CDH2,EGR1,[illegible] |
| LXR/RXR Activation | 4,72E00 | ABCG1,AGT,[illegible] |
| PPAR Signaling | 4,72E00 | CITED2,[illegible] |
| PKCθ Signaling in T Lymphocytes | 4,65E00 | BCL10,[illegible] |
| IL-12 Signaling and Production in Macrophages | 4,63E00 | APOE,APOL1,[illegible] |
| Tec Kinase Signaling | 4,6E00 | ACTA2,DIRAS3,[illegible] |
| Role of Hypercytokinemia/hyperchemokinemia in the Pathogenesis of Influenza | 4,56E00 | CCL2,CCL5,[illegible] |
| Lymphotoxin β Receptor Signaling | 4,43E00 | BIRC2,CREBBP,[illegible] |
| Role of Osteoblasts, Osteoclasts and Chondrocytes in Rheumatoid Arthritis | 4,37E00 | BAD,BIRC2,[illegible] |
| Production of Nitric Oxide and Reactive Oxygen Species in Macrophages | 4,33E00 | APOE,APOL1,[illegible] |
| iNOS Signaling | 4,28E00 | CREBBP,FOS,[illegible] |
| Atherosclerosis Signaling | 4,27E00 | APOE,APOL1,[illegible] |
| iCOS-iCOSL Signaling in T Helper Cells | 4,23E00 | BAD,CD40,CHP1,[illegible] |
| TNFR1 Signaling | 4,22E00 | BIRC2,BIRC3,[illegible] |
| OX40 Signaling Pathway | 4,2E00 | B2M,HLA-A,[illegible] |
| Complement System | 4,15E00 | C1R,C1S,C2,C3,[illegible] |
| PFKFB4 Signaling Pathway | 4,14E00 | ATF4,CREB1,[illegible] |
| Virus Entry via Endocytic Pathways | 4,09E00 | ACTA2,AP1S2,[illegible] |
| IL-17A Signaling in Gastric Cells | 4,07E00 | CCL20,CCL5,[illegible] |
| Prolactin Signaling | 4,07E00 | CEBPB,CREBBP,[illegible] |
| RAR Activation | 4,05E00 | AKR1B10,[illegible] |
| Glucocorticoid Receptor Signaling | 4 | ADRB2,AGT,[illegible] |
| PEDF Signaling | 3,98E00 | ARHGAP22,[illegible] |
| Endocannabinoid Cancer Inhibition Pathway | 3,95E00 | ATF3,ATF4,BAD,[illegible] |
| Erythropoietin Signaling | 3,9E00 | BAD,BIRC2,[illegible] |
| Hepatic Cholestasis | 3,84E00 | CXCL8,[illegible] |
| Xenobiotic Metabolism AHR Signaling Pathway | 3,73E00 | AHR,AHRR,[illegible] |
| p38 MAPK Signaling | 3,72E00 | ATF4,CREB1,[illegible] |
| NRF2-mediated Oxidative Stress Response | 3,68E00 | ABCC4,ACTA2,[illegible] |
| Angiopoietin Signaling | 3,68E00 | ANGPT2,BAD,[illegible] |
| Protein Kinase A Signaling | 3,67E00 | AKAP7,[illegible] |
| IL-10 Signaling | 3,66E00 | BLVRB,CCR1,[illegible] |
| ILK Signaling | 3,63E00 | ACTA2,ACTN2,[illegible] |
| 4-1BB Signaling in T Lymphocytes | 3,6E00 | IKBKE,JUN,[illegible] |
| Antioxidant Action of Vitamin C | 3,57E00 | CSF2RA,GSTO2,[illegible] |
| IL-15 Production | 3,52E00 | ABL2,CLK1,[illegible] |
| CD28 Signaling in T Helper Cells | 3,52E00 | ACTR2,ARPC5,[illegible] |
| Inflammasome pathway | 3,51E00 | CASP1,CASP8,[illegible] |
| Role of IL-17F in Allergic Inflammatory Airway Diseases | 3,48E00 | ATF4,CCL2,[illegible] |
| Role of NFAT in Regulation of the Immune Response | 3,47E00 | CHP1,FOS,[illegible] |
| Huntington's Disease Signaling | 3,45E00 | ATF4,ATP5PB,[illegible] |
| Estrogen-Dependent Breast Cancer Signaling | 3,31E00 | ATF4,CREB1,[illegible] |
| MIF-mediated Glucocorticoid Regulation | 3,31E00 | CD74,MAPK3,[illegible] |
| Granulocyte Adhesion and Diapedesis | 3,31E00 | C5,CCL2,CCL20,[illegible] |
| IL-17A Signaling in Airway Cells | 3,26E00 | CCL20,CXCL1,[illegible] |
| JAK/Stat Signaling | 3,26E00 | CDKN1A,CEBPB,[illegible] |
| Xenobiotic Metabolism Signaling | 3,24E00 | AHR,AHRR,[illegible] |
| PI3K Signaling in B Lymphocytes | 3,2E00 | ATF3,ATF4,[illegible] |
| Cardiac Hypertrophy Signaling (Enhanced) | 3,17E00 | ACVR1C,[illegible] |
| p53 Signaling | 3,16E00 | BBC3,BIRC5,[illegible] |
| Retinoic acid Mediated Apoptosis Signaling | 3,15E00 | CASP8,CFLAR,[illegible] |
| IL-8 Signaling | 3,15E00 | ANGPT2,ARRB2,[illegible] |
| NF-κB Activation by Viruses | 3,15E00 | EIF2AK2,IKBKE,[illegible] |
| Differential Regulation of Cytokine Production in Macrophages and T Helper Cells by IL-17A and I | 3,13E00 | CCL2,CCL5,[illegible] |
| Small Cell Lung Cancer Signaling | 3,12E00 | BIRC2,CDKN2B,[illegible] |
| MSP-RON Signaling In Cancer Cells Pathway | 3,09E00 | ACTA2,ATF4,[illegible] |
| B Cell Development | 3,06E00 | CD40,HLA-A,[illegible] |
| Unfolded protein response | 3,04E00 | ATF4,CEBPB,[illegible] |
| B Cell Activating Factor Signaling | 3,03E00 | FOS,IKBKE,JUN,[illegible] |
| FAT10 Cancer Signaling Pathway | 3,03E00 | ACVR1C,[illegible] |
| RANK Signaling in Osteoclasts | 3,01E00 | BIRC2,BIRC3,[illegible] |
| IL-7 Signaling Pathway | 3 | BAD,BAK1,BCL6,[illegible] |
| PI3K/AKT Signaling | 2,98E00 | BAD,CDKN1A,[illegible] |
| Tryptophan Degradation III (Eukaryotic) | 2,97E00 | ACAA2,ACAT2,[illegible] |
| Caveolar-mediated Endocytosis Signaling | 2,96E00 | ACTA2,B2M,[illegible] |
| MIF Regulation of Innate Immunity | 2,92E00 | CD74,FOS,JUN,[illegible] |
| MSP-RON Signaling Pathway | 2,87E00 | ACTA2,CCL2,[illegible] |
| IL-17 Signaling | 2,85E00 | CCL2,CEBPB,[illegible] |
| Endothelin-1 Signaling | 2,85E00 | CASP1,CASP10,[illegible] |
| Role of JAK1 and JAK3 in γc Cytokine Signaling | 2,84E00 | IL15,IL15RA,[illegible] |
| IL-1 Signaling | 2,8E00 | FOS,GNA13,[illegible] |
| Role of Cytokines in Mediating Communication between Immune Cells | 2,75E00 | CSF3,CXCL8,[illegible] |
| Neurotrophin/TRK Signaling | 2,74E00 | ATF4,BDNF,[illegible] |
| Autoimmune Thyroid Disease Signaling | 2,73E00 | CD40,FAS,[illegible] |
| IL-23 Signaling Pathway | 2,72E00 | IL1B,IL23A,JAK2,[illegible] |
| STAT3 Pathway | 2,72E00 | CDKN1A,FGF2,[illegible] |
| Ceramide Signaling | 2,69E00 | BAD,CERK,[illegible] |
| Agranulocyte Adhesion and Diapedesis | 2,68E00 | ACTA2,C5,CCL2,[illegible] |
| B Cell Receptor Signaling | 2,66E00 | ATF4,BAD,[illegible] |
| Pathogenesis of Multiple Sclerosis | 2,63E00 | CCL5,CCR1,[illegible] |
| Coronavirus Replication Pathway | 2,62E00 | ACE2,BAG3,[illegible] |
| April Mediated Signaling | 2,61E00 | FOS,IKBKE,JUN,[illegible] |
| Phagosome Maturation | 2,54E00 | ATP6V0E2,B2M,[illegible] |
| BEX2 Signaling Pathway | 2,53E00 | BAD,BAK1,[illegible] |
| IL-4 Signaling | 2,51E00 | HLA-A,HLA-B,[illegible] |
| Estrogen Biosynthesis | 2,51E00 | AKR1C3,[illegible] |
| P2Y Purigenic Receptor Signaling Pathway | 2,5E00 | ATF4,CREB1,[illegible] |
| Pancreatic Adenocarcinoma Signaling | 2,49E00 | BAD,BIRC5,[illegible] |
| Thrombopoietin Signaling | 2,46E00 | FOS,IRS2,JAK2,[illegible] |
| Allograft Rejection Signaling | 2,45E00 | B2M,CD40,FAS,[illegible] |
| Role of Tissue Factor in Cancer | 2,44E00 | ARRB2,CSF1,[illegible] |
| Xenobiotic Metabolism PXR Signaling Pathway | 2,42E00 | ALDH16A1,[illegible] |
| FAT10 Signaling Pathway | 2,4E00 | MAP1LC3B,[illegible] |
| Role of PI3K/AKT Signaling in the Pathogenesis of Influenza | 2,39E00 | CCL5,[illegible] |
| Clathrin-mediated Endocytosis Signaling | 2,38E00 | ACTA2,ACTR2,[illegible] |
| Type II Diabetes Mellitus Signaling | 2,38E00 | CACNA1D,[illegible] |
| Ketolysis | 2,38E00 | ACAA2,ACAT2,[illegible] |
| Colorectal Cancer Metastasis Signaling | 2,35E00 | BAD,BIRC5,[illegible] |
| Macropinocytosis Signaling | 2,35E00 | ANKFY1,CSF1,[illegible] |
| Tight Junction Signaling | 2,35E00 | ACTA2,[illegible] |
| Renin-Angiotensin Signaling | 2,34E00 | AGT,CCL2,[illegible] |
| Differential Regulation of Cytokine Production in Intestinal Epithelial Cells by IL-17A and IL-17F | 2,33E00 | CCL2,CCL5,[illegible] |
| BAG2 Signaling Pathway | 2,33E00 | ANXA2,CDKN1A,[illegible] |
| Relaxin Signaling | 2,3E00 | CREB1,CTH,[illegible] |
| Growth Hormone Signaling | 2,3E00 | FOS,IGF2,JAK2,[illegible] |
| ERK/MAPK Signaling | 2,29E00 | ATF4,BAD,[illegible] |
| LPS-stimulated MAPK Signaling | 2,28E00 | CREB1,FOS,[illegible] |
| Neuropathic Pain Signaling In Dorsal Horn Neurons | 2,27E00 | BDNF,CREB1,[illegible] |
| Acute Myeloid Leukemia Signaling | 2,27E00 | BAD,CSF1R,[illegible] |
| Calcium-induced T Lymphocyte Apoptosis | 2,25E00 | CAPN2,CHP1,[illegible] |
| Sperm Motility | 2,24E00 | ABL2,CLK1,[illegible] |
| Semaphorin Neuronal Repulsive Signaling Pathway | 2,23E00 | CSPG5,DPYSL2,[illegible] |
| TR/RXR Activation | 2,22E00 | [illegible] |
| Sirtuin Signaling Pathway | 2,21E00 | ACSS2,ATP5PB,[illegible] |
| PPARα/RXRα Activation | 2,21E00 | ACVR1C,BCL3,[illegible] |
| Role of JAK1, JAK2 and TYK2 in Interferon Signaling | 2,2E00 | IFNA1/IFNA13,[illegible] |
| Adipogenesis pathway | 2,17E00 | BMP2,CEBPB,[illegible] |
| 3-phosphoinositide Biosynthesis | 2,17E00 | ATP1A1,[illegible] |
| Sumoylation Pathway | 2,17E00 | ARHGDIB,CBX4,[illegible] |
| Melatonin Degradation II | 2,17E00 | IL4I1,MAOA,[illegible] |
| IL-3 Signaling | 2,16E00 | BAD,CHP1,FOS,[illegible] |
| Prostate Cancer Signaling | 2,16E00 | ATF4,BAD,[illegible] |
| Systemic Lupus Erythematosus In T Cell Signaling Pathway | 2,16E00 | ATF4,B2M,BCL6,[illegible] |
| Ketogenesis | 2,16E00 | ACAA2,ACAT2,[illegible] |
| Superpathway of Inositol Phosphate Compounds | 2,16E00 | ATP1A1,[illegible] |
| Inhibition of ARE-Mediated mRNA Degradation Pathway | 2,15E00 | AGO4,CNOT4,[illegible] |
| Protein Ubiquitination Pathway | 2,14E00 | ANAPC10,[illegible] |
| fMLP Signaling in Neutrophils | 2,14E00 | ACTR2,ARPC5,[illegible] |
| Hypoxia Signaling in the Cardiovascular System | 2,11E00 | ATF4,CREB1,[illegible] |
| Noradrenaline and Adrenaline Degradation | 2,1E00 | ADH5,ADH6,[illegible] |
| Tryptophan Degradation X (Mammalian, via Tryptamine) | 2,08E00 | AKR1B10,[illegible] |
| Neuregulin Signaling | 2,07E00 | AREG,BAD,BTC,[illegible] |
| IL-15 Signaling | 2,05E00 | CXCL8,GNB1L,[illegible] |
| Airway Pathology in Chronic Obstructive Pulmonary Disease | 2,04E00 | CCL2,CCL20,[illegible] |
| Glutaryl-CoA Degradation | 2,03E00 | ACAA2,ACAT2,[illegible] |
| Natural Killer Cell Signaling | 2 | B2M,COL11A2,[illegible] |
| Germ Cell-Sertoli Cell Junction Signaling | 1,98E00 | ACTA2,ACTN2,[illegible] |
| Corticotropin Releasing Hormone Signaling | 1,98E00 | ARPC5,ATF4,[illegible] |
| Putrescine Degradation III | 1,98E00 | ALDH1A2,[illegible] |
| Endoplasmic Reticulum Stress Pathway | 1,98E00 | ATF4,CASP7,[illegible] |
| Hematopoiesis from Multipotent Stem Cells | 1,97E00 | CSF1,CSF3,IL15,[illegible] |
| FXR/RXR Activation | 1,97E00 | AGT,APOE,[illegible] |
| CD27 Signaling in Lymphocytes | 1,97E00 | CASP8,FOS,[illegible] |
| LPS/IL-1 Mediated Inhibition of RXR Function | 1,96E00 | ABCC4,ABCG1,[illegible] |
| HIF1α Signaling | 1,95E00 | ADM,CDKN1A,[illegible] |
| Signaling by Rho Family GTPases | 1,93E00 | ACTA2,ACTR2,[illegible] |
| Integrin Signaling | 1,91E00 | ACTA2,ACTN2,[illegible] |
| TGF-β Signaling | 1,9E00 | ACVR1C,BMP2,[illegible] |
| Regulation of IL-2 Expression in Activated and Anergic T Lymphocytes | 1,89E00 | BCL10,CHP1,[illegible] |
| Eicosanoid Signaling | 1,88E00 | AKR1C3,[illegible] |
| ERK5 Signaling | 1,88E00 | ATF4,BAD,[illegible] |
| Sertoli Cell-Sertoli Cell Junction Signaling | 1,87E00 | ACTA2,ACTN2,[illegible] |
| ATM Signaling | 1,85E00 | ATF4,CCNB1,[illegible] |
| Cell Cycle: G2/M DNA Damage Checkpoint Regulation | 1,85E00 | AURKA,CCNB1,[illegible] |
| White Adipose Tissue Browning Pathway | 1,84E00 | ANGPT2,ATF4,[illegible] |
| BMP signaling pathway | 1,83E00 | BMP2,BMP8B,[illegible] |
| Thyroid Cancer Signaling | 1,83E00 | BDNF,CXCL8,[illegible] |
| FAK Signaling | 1,82E00 | ACTA2,[illegible] |
| Epoxysqualene Biosynthesis | 1,82E00 | FDFT1,SQLE |
| β-alanine Degradation I | 1,82E00 | ABAT,ALDH6A1 |
| 4-hydroxyproline Degradation I | 1,82E00 | ALDH4A1,[illegible] |
| Sulfate Activation for Sulfonation | 1,82E00 | PAPSS1,[illegible] |
| IGF-1 Signaling | 1,82E00 | BAD,FOS,[illegible] |
| Lysine Degradation II | 1,81E00 | AADAT,AASS,[illegible] |
| Bile Acid Biosynthesis, Neutral Pathway | 1,81E00 | [illegible] |
| Salvage Pathways of Pyrimidine Ribonucleotides | 1,81E00 | AK4,APOBEC3F,[illegible] |
| UVA-Induced MAPK Signaling | 1,81E00 | FOS,JUN,[illegible] |
| Apelin Pancreas Signaling Pathway | 1,8E00 | DDIT3,ERN1,[illegible] |
| Circadian Rhythm Signaling | 1,8E00 | ATF4,CREB1,[illegible] |
| PDGF Signaling | 1,78E00 | ABL2,CAV1,[illegible] |
| Estrogen Receptor Signaling | 1,78E00 | AGT,ATF4,[illegible] |
| Adrenomedullin signaling pathway | 1,78E00 | ADM,BAD,C3,[illegible] |
| Role of MAPK Signaling in the Pathogenesis of Influenza | 1,77E00 | CCL2,CCL5,[illegible] |
| D-myo-inositol-5-phosphate Metabolism | 1,77E00 | ATP1A1,[illegible] |
| Wnt/Ca+ pathway | 1,76E00 | ATF4,CREB1,[illegible] |
| Phosphatidylethanolamine Biosynthesis II | 1,76E00 | CHKA,CHKB,[illegible] |
| Cytotoxic T Lymphocyte-mediated Apoptosis of Target Cells | 1,71E00 | B2M,CASP7,[illegible] |
| Role of JAK2 in Hormone-like Cytokine Signaling | 1,71E00 | HLTF,IRS2,JAK2,[illegible] |
| ErbB Signaling | 1,7E00 | AREG,BTC,[illegible] |
| Phagosome Formation | 1,69E00 | CLEC7A,[illegible] |
| HGF Signaling | 1,68E00 | CDKN1A,ELF1,[illegible] |
| Role of NFAT in Cardiac Hypertrophy | 1,67E00 | CACNA1D,[illegible] |
| Kinetochore Metaphase Signaling Pathway | 1,67E00 | ANAPC10,[illegible] |
| 14-3-3-mediated Signaling | 1,67E00 | BAD,FOS,[illegible] |
| GADD45 Signaling | 1,64E00 | CCNB1,CCND3,[illegible] |
| Reelin Signaling in Neurons | 1,61E00 | ACTR2,APOE,[illegible] |
| D-myo-inositol (1,4,5,6)-Tetrakisphosphate Biosynthesis | 1,6E00 | ATP1A1,[illegible] |
| D-myo-inositol (3,4,5,6)-tetrakisphosphate Biosynthesis | 1,6E00 | ATP1A1,[illegible] |
| PXR/RXR Activation | 1,59E00 | CYP2B6,FOXO1,[illegible] |
| PTEN Signaling | 1,58E00 | BAD,BCL2L11,[illegible] |
| Role of JAK family kinases in IL-6-type Cytokine Signaling | 1,55E00 | IL6,JAK2,MAPK3,[illegible] |
| Tryptophan Degradation to 2-amino-3-carboxymuconate Semialdehyde | 1,55E00 | IDO1,KMO,[illegible] |
| Pyridoxal 5'-phosphate Salvage Pathway | 1,54E00 | CDK18,DYRK1A,[illegible] |
| Maturity Onset Diabetes of Young (MODY) Signaling | 1,53E00 | ABCC9,APOE,[illegible] |
| Adenosine Nucleotides Degradation II | 1,53E00 | ADAT3,NT5C3A,[illegible] |
| Gα12/13 Signaling | 1,52E00 | CDH2,CDH24,[illegible] |
| Aldosterone Signaling in Epithelial Cells | 1,51E00 | AHCY,DNAJB4,[illegible] |
| Gαq Signaling | 1,51E00 | CHP1,DIRAS3,[illegible] |
| Calcium Signaling | 1,51E00 | ACTA2,ATF4,[illegible] |
| Axonal Guidance Signaling | 1,47E00 | ACE2,ACTR2,[illegible] |
| FLT3 Signaling in Hematopoietic Progenitor Cells | 1,47E00 | ATF4,BAD,[illegible] |
| T Cell Receptor Signaling | 1,46E00 | BCL10,FOS,[illegible] |
| Leptin Signaling in Obesity | 1,45E00 | FOXO1,JAK2,[illegible] |
| Phospholipase C Signaling | 1,41E00 | AHNAK,[illegible] |
| Isoleucine Degradation I | 1,41E00 | ACAA2,ACADSB,[illegible] |
| Rac Signaling | 1,4E00 | ACTR2,ARPC5,[illegible] |
| Cdc42 Signaling | 1,38E00 | ACTR2,ARPC5,[illegible] |
| Methylglyoxal Degradation I | 1,38E00 | GLO1,HAGHL |
| 4-aminobutyrate Degradation I | 1,38E00 | ABAT,SUCLG2 |
| 3-phosphoinositide Degradation | 1,36E00 | ATP1A1,[illegible] |
| Androgen Signaling | 1,35E00 | CACNA1D,[illegible] |
| Superpathway of Serine and Glycine Biosynthesis I | 1,35E00 | PSAT1,PSPH,[illegible] |
| Xenobiotic Metabolism General Signaling Pathway | 1,34E00 | ABCA2,AHR,[illegible] |
| Polyamine Regulation in Colon Cancer | 1,34E00 | MAX,MXD1,MYC,[illegible] |
| Chronic Myeloid Leukemia Signaling | 1,33E00 | BAD,CDKN1A,[illegible] |
| Sphingosine-1-phosphate Signaling | 1,31E00 | ASAH1,CASP1,[illegible] |
| Amyotrophic Lateral Sclerosis Signaling | 1,31E00 | BIRC2,BIRC3,[illegible] |
| UVB-Induced MAPK Signaling | 1,3E00 | BAD,FOS,[illegible] |
| HER-2 Signaling in Breast Cancer | 1,3E00 | AREG,BAD,[illegible] |
| VEGF Family Ligand-Receptor Interactions | 1,3E00 | FOS,MAPK3,[illegible] |

**Supplementary Table S10. List of Canonical Pathways for the condition "Calu-3_SARS-CoV".** The IPA-based Core Analysis was performed on the basis of differentially expressed genes dissected for a given condition by GENEVESTIGATOR-based analysis. Only statistically significant Canonical Pathways are included (n = 227). The data are taken as the basis for R-based visualization by Venn diagrams shown in Figure 5.

| Ingenuity Canonical Pathways | -log(p-value) | Molecules |
|---|---|---|
| Neuroinflammation Signaling Pathway | 1,8E01 | ACVR1,ACVR2A,ATF4,B2M,BDNF,BIRC2,BIRC3,BIRC6,CALB1,CASP1,CASP8,CCL2,CCL5,CD40,CFLAR,CREB1,CREB5,CREBBP,CSF1R,CX3CL1,CXCL10,CXCL8,FAS,FOS,GABRE,GABRP,GJB1,GLS,GRIN2A,GRINA,HLA-A,HLA-B,HLA-C,HLA-DOB,HLA-DQA1,HLA-E,HLA-F,HMOX1,ICAM1,IFNB1,IFNGR2,IKBKE,IL12A,[illegible] |
| TNFR2 Signaling | 1,42E01 | BIRC2,BIRC3,FOS,IKBKE,JUN,MAP2K4,MAP3K1,NFKB1,NFKB2,NFKBIA,NFKBIB,NFKBID,NFKBIE,TANK,TBK1,TNF,TNFAIP3,TNFRSF1B,TRAF1,TRAF2,XIAP |
| Death Receptor Signaling | 1,36E01 | ARHGDIB,BIRC2,BIRC3,CASP10,CASP7,CASP8,CFLAR,DAXX,FAS,IKBKE,MAP2K4,MAP4K4,NFKB1,NFKB2,NFKBIA,NFKBIB,NFKBID,NFKBIE,PARP10,PARP12,PARP14,PARP3,PARP8,PARP9,RIPK1,TANK,TBK1,TIPARP,TNF,TNFRSF10A,TNFRSF10B,TNFRSF1B,TNFSF10,TNFSF15,TNKS2,TRAF2,XIAP |
| Activation of IRF by Cytosolic Pattern Recognition Receptors | 1,35E01 | ADAR,CD40,CREBBP,DDX58,DHX58,IFIH1,IFIT2,IFNB1,IKBKE,IL6,IRF7,IRF9,ISG15,JUN,MAP2K4,NFKB1,NFKB2,NFKBIA,NFKBIB,NFKBID,NFKBIE,RIPK1,STAT1,STAT2,TANK,TBK1,TNF,TRAF3,TRAF6,ZBP1 |
| Dendritic Cell Maturation | 1,33E01 | ATF4,B2M,CD40,CD83,COL11A2,COL1A1,CREB1,CREB5,CREBBP,CSF2,FSCN1,HLA-A,HLA-B,HLA-C,HLA-DOB,HLA-DQA1,HLA-DRB4,HLA-E,HLA-F,ICAM1,IFNB1,IKBKE,IL12A,IL15,IL1A,IL1B,IL1RN,IL32,IL6,IRF8,JAK2,LTB,MAP2K4,MYD88,NFKB1,NFKB2,NFKBIA,NFKBIB,NFKBID,NFKBIE,NGFR,PIK3C2A,PIK3CA,PIK3R1,[illegible] |
| Systemic Lupus Erythematosus In B Cell Signaling Pathway | 1,23E01 | BCL10,BCL2L11,CD40,CSF2,CXCL8,FOS,FOXO1,FOXO3,FOXO4,GAB1,IFIH1,IFIT2,IFIT3,IFNAR2,IFNB1,IFNGR2,IGHE,IL11,IL12A,IL15,IL1A,IL1B,IL6,IL6R,IL6ST,IRF7,IRF9,ISG15,ISG20,JAK2,JUN,LIF,LTB,LYN,MAP4K4,MCL1,MYD88,NFAT5,NFATC2,NFKB1,NFKB2,PIK3AP1,PIK3C2A,PIK3CA,PIK3R1,PIK3R3,PLAAT4,[illegible] |
| Interferon Signaling | 1,19E01 | IFI35,IFI6,IFIT1,IFIT3,IFITM1,IFITM2,IFITM3,IFNAR2,IFNB1,IFNGR2,IRF1,IRF9,ISG15,JAK2,MX1,OAS1,PSMB8,SOCS1,STAT1,STAT2,TAP1 |
| Role of RIG1-like Receptors in Antiviral Innate Immunity | 1,16E01 | CASP10,CASP8,CREBBP,DDX58,DHX58,EP300,IFIH1,IFNB1,IKBKE,IRF7,NFKB1,NFKB2,NFKBIA,NFKBIB,NFKBID,NFKBIE,RIPK1,TANK,TBK1,TRAF2,TRAF3,TRAF6,TRIM25 |
| Role of Macrophages, Fibroblasts and Endothelial Cells in Rheumatoid Arthritis | 1,09E01 | ATF4,C5,CCL2,CCL5,CEBPB,CEBPD,CREB1,CREB5,CREBBP,CSF1,CSF2,CXCL8,DKK3,FGF2,FOS,FZD4,FZD5,ICAM1,IKBKE,IL15,IL18R1,IL1A,IL1B,IL1RN,IL32,IL6,IL6R,IL6ST,IL7,IRAK2,JAK2,JUN,LRP1,LTB,MAP2K4,MYD88,NFAT5,NFATC2,NFKB1,NFKBIA,NFKBIB,NFKBID,NFKBIE,NGFR,PDGFA,PDGFD,PGF,[illegible] |
| Role of Pattern Recognition Receptors in Recognition of Bacteria and Viruses | 1,06E01 | C3,C5,CASP1,CCL5,CREB1,CSF2,CXCL8,DDX58,EIF2AK2,IFIH1,IFNB1,IL11,IL12A,IL15,IL1A,IL1B,IL6,IRF7,LIF,LTB,MAP2K4,MYD88,NFKB1,NFKB2,NLRP3,NOD2,OAS1,OAS2,OAS3,PIK3C2A,PIK3CA,PIK3R1,PIK3R3,PLCG2,PRKCE,PTX3,RIPK2,TICAM1,TLR3,TNF,TNFSF10,TNFSF13B,TNFSF15,TNFSF9,TRAF6 |
| Tumor Microenvironment Pathway | 1,01E01 | BRAF,CCL2,CD274,CFLAR,COL1A1,CSF1,CSF2,CSF3,CXCL8,FAS,FGF18,FGF19,FGF2,FOS,FOXO1,FOXO3,FOXO4,HLA-A,HLA-B,HLA-C,HLA-E,HLA-F,ICAM1,IDO1,IGF2,IL1B,IL6,IL6R,ITGA5,JAK2,JUN,LGALS9,MMP7,NFKB1,NFKB2,PDGFA,PDGFD,PGF,PIK3C2A,PIK3CA,PIK3R1,PIK3R3,PLAU,PTGS2,SPP1,TNF,[illegible] |
| Coronavirus Pathogenesis Pathway | 9,33E00 | ACE2,ATF4,BCL2L11,CASP1,CASP8,CCL2,CCL5,CXCL8,DDIT3,DDX58,E2F3,E2F6,E2F7,EEF1A1,EIF2AK3,EP300,FOS,IFNAR2,IFNB1,IL1B,IL6,IRF7,IRF9,JUN,MAP2K4,NFKB1,NFKB2,NFKBIA,NFKBIB,NFKBID,NFKBIE,NLRP3,PTGS2,RBL2,RIPK1,RPS27L,STAT1,STAT2,STING1,TBK1,TRAF3,TRIM25 |
| NF-κB Signaling | 9,32E00 | AZI2,BCL10,BMP2,BRAF,CASP8,CD40,CREBBP,EGFR,EIF2AK2,EP300,FGFR1,FGFR3,IL1A,IL1B,IL1RN,MAP3K1,MAP3K8,MAP4K4,MYD88,NFKB1,NFKB2,NFKBIA,NFKBIB,NFKBID,NFKBIE,NGFR,PELI1,PIK3C2A,PIK3CA,PIK3R1,PIK3R3,PLCG2,PRKACB,RELB,RIPK1,TAB3,TANK,TBK1,TLR3,TNF,TNFAIP3,[illegible] |
| Role of PKR in Interferon Induction and Antiviral Response | 8,61E00 | ATF3,CASP1,CASP8,DDX58,EIF2AK2,FAS,FOS,HSPA1A/HSPA1B,HSPA1L,HSPA6,IFIH1,IFNAR2,IFNB1,IFNGR2,IKBKE,IL1B,IRF1,IRF9,JUN,MAP2K4,MYD88,NFKB1,NFKB2,NFKBIA,NFKBIB,NFKBID,NFKBIE,PDGFA,PDGFD,STAT1,STAT2,TLR3,TNF,TRAF3,TRAF6 |
| TNFR1 Signaling | 8,42E00 | BIRC2,BIRC3,CASP7,CASP8,FOS,IKBKE,JUN,MAP2K4,MAP3K1,NFKB1,NFKB2,NFKBIA,NFKBIB,NFKBID,NFKBIE,RIPK1,TANK,TNF,TNFAIP3,TRAF2,XIAP |
| Hepatic Fibrosis Signaling Pathway | 8,28E00 | ACVR1,ACVR2A,ATF4,BRAF,BRD4,CCL2,CCL5,CD40,CDKN1B,CEBPB,COL11A2,COL1A1,CREB1,CREB5,CREBBP,CXCL8,DIRAS3,EDN1,FGF2,FGFR1,FOS,FOXO1,FOXO4,FZD4,FZD5,ICAM1,IKBKE,IL1A,IL1B,IL1RN,IRAK2,IRS2,ITGA5,JAK2,JUN,MAP2K4,MYD88,MYL6B,NFKB1,NFKB2,NFKBIA,NFKBIB,NFKBID,NFKBIE,[illegible] |
| Type I Diabetes Mellitus Signaling | 8,19E00 | CASP8,FAS,HLA-A,HLA-B,HLA-C,HLA-DOB,HLA-DQA1,HLA-E,HLA-F,IFNGR2,IKBKE,IL12A,IL1B,IRF1,JAK2,MAP2K4,MYD88,NFKB1,NFKB2,NFKBIA,NFKBIB,NFKBID,NFKBIE,NGFR,RIPK1,SOCS1,SOCS2,SOCS3,STAT1,TNF,TNFRSF1B,TRAF2,TRAF6 |
| Lymphotoxin β Receptor Signaling | 7,87E00 | BIRC2,CREBBP,CXCL1,EP300,IKBKE,LTB,NFKB1,NFKB2,NFKBIA,NFKBID,PIK3C2A,PIK3CA,PIK3R1,PIK3R3,RELB,TRAF1,TRAF2,TRAF3,TRAF4,TRAF6,VCAM1 |
| B Cell Activating Factor Signaling | 7,67E00 | FOS,IKBKE,JUN,MAP2K4,MAP3K1,NFAT5,NFATC2,NFKB1,NFKB2,NFKBIA,NFKBIB,NFKBID,NFKBIE,TNFSF13B,TRAF1,TRAF2,TRAF3,TRAF6 |
| Necroptosis Signaling Pathway | 7,63E00 | BIRC2,BIRC3,CAPN8,CASP1,CASP10,CASP8,CFLAR,CYLD,EIF2AK2,FAS,IFNB1,IRF9,JMJD7-PLA2G4B,MLKL,NGFR,NLRP3,PELI1,PLA2G4C,PLA2G4F,PLA2G6,PPP3CC,RBCK1,RBL2,RIPK1,RNF31,STAT1,STAT2,TICAM1,TIMM23,TIMM23B,TLR3,TNF,TNFRSF10A,TNFRSF10B,TNFRSF1B,TNFSF10,TNIP1,TRAF2,[illegible] |
| Regulation Of The Epithelial Mesenchymal Transition By Growth Factors Pathway | 7,6E00 | BRAF,DOCK10,EGFR,EGR1,FGF18,FGF19,FGF2,FGFR1,FGFR3,FOS,FOXO1,FRS2,GAB1,IKBKE,IL6,IL6R,JAK2,JUN,LATS1,LATS2,LTB,MAP2K4,MAP3K1,NFKB1,NFKB2,NGFR,PARD6B,PDGFA,PDGFD,PIK3C2A,PIK3CA,PIK3R1,PIK3R3,SMURF1,SNAI1,TNF,TNFRSF1B,TNFSF10,TNFSF13B,TNFSF15,TNFSF18,[illegible] |
| CD40 Signaling | 7,46E00 | CD40,FOS,ICAM1,IKBKE,JUN,MAP2K4,NFKB1,NFKB2,NFKBIA,NFKBIB,NFKBID,NFKBIE,PIK3C2A,PIK3CA,PIK3R1,PIK3R3,PTGS2,TANK,TNFAIP3,TRAF1,TRAF2,TRAF3,TRAF6 |
| MSP-RON Signaling In Macrophages Pathway | 7,4E00 | ATF4,CIITA,CREB1,CREB5,CREBBP,ETV3,FOS,HLA-DOB,HLA-DPB1,HLA-DQA1,HLA-DRB4,IFNGR2,IKBKE,IL12A,ITGAM,JAK2,JUN,KLK6,KLK7,NFKB1,NFKB2,NFKBIZ,PIK3C2A,PIK3CA,PIK3R1,PIK3R3,PTGS2,SBNO2,SOCS1,SOCS3,STAT1,TNF |
| IL-6 Signaling | 7,25E00 | CEBPB,COL1A1,CXCL8,FOS,IKBKE,IL1A,IL1B,IL1RN,IL6,IL6R,IL6ST,JAK2,JUN,MAP2K4,MAP4K4,MCL1,NFKB1,NFKB2,NFKBIA,NFKBIB,NFKBID,NFKBIE,NGFR,PIK3C2A,PIK3CA,PIK3R1,PIK3R3,SOCS1,SOCS3,TNF,TNFRSF1B,TRAF2,TRAF6,VEGFA |
| Antigen Presentation Pathway | 7,22E00 | B2M,CIITA,HLA-A,HLA-B,HLA-C,HLA-DOB,HLA-DPB1,HLA-DQA1,HLA-DRB4,HLA-E,HLA-F,NLRC5,PSMB8,PSMB9,TAP1,TAP2,TAPBP |
| TWEAK Signaling | 7,19E00 | BIRC2,BIRC3,CASP7,CASP8,IKBKE,NFKB1,NFKB2,NFKBIA,NFKBIB,NFKBID,NFKBIE,RIPK1,TRAF1,TRAF2,TRAF3,XIAP |
| Production of Nitric Oxide and Reactive Oxygen Species in Macrophages | 7,07E00 | APOE,APOL1,ARG2,CAT,CREBBP,CYBA,DIRAS3,FOS,IFNGR2,IKBKE,IRF1,IRF8,JAK2,JUN,LPA,LYZ,MAP2K4,MAP3K1,MAP3K2,MAP3K8,MAP3K9,NFKB1,NFKB2,NFKBIA,NFKBIB,NFKBID,NFKBIE,NGFR,PCYOX1,PIK3C2A,PIK3CA,PIK3R1,PIK3R3,PLCG2,PPP1R12A,PPP1R7,PPP2R2A,PRKCE,RHOV,RND1,RND3,STAT1,[illegible] |
| April Mediated Signaling | 7,02E00 | FOS,IKBKE,JUN,MAP2K4,MAP3K1,NFAT5,NFATC2,NFKB1,NFKB2,NFKBIA,NFKBIB,NFKBID,NFKBIE,TRAF1,TRAF2,TRAF3,TRAF6 |
| Th1 and Th2 Activation Pathway | 7,01E00 | ACVR1,ACVR2A,BHLHE41,CCR1,CCR4,CD274,CD40,CHD4,GATA3,HAVCR1,HLA-A,HLA-B,HLA-DOB,HLA-DPB1,HLA-DQA1,HLA-DRB4,ICAM1,ICOSLG/LOC102723996,IFNGR2,IL10RA,IL12A,IL18R1,IL6,IL6R,IRF1,JAK2,JUN,LGALS9,NFATC2,NFIL3,NFKB1,NOTCH1,PIK3C2A,PIK3CA,PIK3R1,PIK3R3,SOCS1,SOCS3,STAT1,[illegible] |
| Hepatic Fibrosis / Hepatic Stellate Cell Activation | 6,84E00 | CCL2,CCL5,CD40,COL11A2,COL12A1,COL16A1,COL1A1,COL4A1,COL7A1,CSF1,CXCL3,CXCL8,CXCL9,EDN1,EGFR,FAS,FGF2,FGFR1,ICAM1,IFNAR2,IFNGR2,IGF2,IL10RA,IL1A,IL1B,IL6,IL6R,KLF6,MYH10,MYL6B,NFKB1,NFKB2,NGFR,PDGFA,PDGFD,PGF,SMAD7,STAT1,TNF,TNFRSF1B,VCAM1,VEGFA,VEGFC |
| Protein Ubiquitination Pathway | 6,77E00 | B2M,BAG1,BIRC2,BIRC3,BIRC6,DNAJB1,DNAJB4,DNAJB5,DNAJC22,DNAJC25,DNAJC27,DNAJC6,FBXW7,HLA-A,HLA-B,HLA-C,HLA-E,HLA-F,HSCB,HSPA1A/HSPA1B,HSPA1L,HSPA4L,HSPA6,HSPB8,HSPH1,NEDD4,NEDD4L,PSMB8,PSMB9,PSME2,SMURF1,TAP1,TAP2,TRAF6,UBD,UBE2L6,UBE2Z,UBR1,UBR2,USP15,[illegible] |
| Senescence Pathway | 6,66E00 | ACVR1,ACVR2A,ATF3,BRAF,CAPN8,CAT,CBX7,CCNB2,CDKN1A,CDKN1B,CDKN2B,CEBPB,CGAS,CREBBP,CXCL8,E2F3,E2F6,E2F7,ELF1,ELF3,EP300,ETS2,FOXO3,FOXO4,GADD45A,GADD45B,GATA4,IFNB1,IKBKE,IL1A,IL6,ITSN2,JUN,MAP2K4,MAPK6,NFAT5,NFATC2,NFKB1,NFKB2,PIK3C2A,PIK3CA,PIK3R1,PIK3R3,[illegible] |
| Th1 Pathway | 6,64E00 | CD274,CD40,GATA3,HLA-A,HLA-B,HLA-DOB,HLA-DPB1,HLA-DQA1,HLA-DRB4,ICAM1,ICOSLG/LOC102723996,IFNGR2,IL10RA,IL12A,IL18R1,IL6,IL6R,IRF1,JAK2,LGALS9,NFATC2,NFIL3,NFKB1,NOTCH1,PIK3C2A,PIK3CA,PIK3R1,PIK3R3,SOCS1,SOCS3,STAT1,STAT4 |
| Induction of Apoptosis by HIV1 | 6,63E00 | BBC3,BIRC2,BIRC3,CASP8,DAXX,FAS,IKBKE,MAP2K4,NFKB1,NFKB2,NFKBIA,NFKBIB,NFKBID,NFKBIE,NGFR,RIPK1,TNF,TNFRSF1B,TRAF1,TRAF2,XIAP |
| PPAR Signaling | 6,52E00 | CITED2,CREBBP,EP300,FOS,IKBKE,IL1A,IL1B,IL1RN,JUN,MAP4K4,NFKB1,NFKB2,NFKBIA,NFKBIB,NFKBID,NFKBIE,NGFR,NR2F1,NRIP1,PDGFA,PDGFD,PPARGC1A,PTGS2,SCAND1,STAT5A,TNF,TNFRSF1B,TRAF2,TRAF6 |
| VDR/RXR Activation | 6,44E00 | CALB1,CASR,CCL5,CDKN1A,CDKN1B,CEBPB,CSF2,CXCL10,EP300,FOXO1,GADD45A,HES1,HR,HSD17B2,IL12A,KLF4,KLK6,MXD1,NCOA3,PDGFA,PRKCE,RXRB,SPP1,VDR |
| PD-1, PD-L1 cancer immunotherapy pathway | 6,42E00 | B2M,CBLB,CD274,CDKN1B,FBXO38,HLA-A,HLA-B,HLA-C,HLA-DOB,HLA-DPB1,HLA-DQA1,HLA-DRB4,HLA-E,HLA-F,IFNGR2,IL12A,JAK2,LATS1,LATS2,NGFR,PDCD4,PIK3C2A,PIK3CA,PIK3R1,PIK3R3,RASGRP1,STAT5A,TNF,TNFRSF1B |
| Acute Phase Response Signaling | 6,37E00 | C1S,C3,C4A/C4B,C5,CEBPB,CFB,FOS,HMOX1,IKBKE,IL1A,IL1B,IL1RN,IL6,IL6R,IL6ST,JAK2,JUN,MAP2K4,MAP3K1,MYD88,NFKB1,NFKB2,NFKBIA,NFKBIB,NFKBID,NFKBIE,NGFR,NR3C1,PIK3CA,PIK3R1,PIK3R3,RIPK1,SAA2,SOCS1,SOCS2,SOCS3,SOD2,TNF,TNFRSF1B,TRAF2,TRAF6 |
| IL-17A Signaling in Fibroblasts | 6,33E00 | CCL2,CEBPB,CEBPD,FOS,IKBKE,IL6,JUN,NFKB1,NFKB2,NFKBIA,NFKBIB,NFKBID,NFKBIE,NFKBIZ,TRAF6 |
| TREM1 Signaling | 6,17E00 | CASP1,CCL2,CD40,CD83,CIITA,CSF2,CXCL3,CXCL8,ICAM1,IL1B,IL6,ITGA5,JAK2,MYD88,NFKB1,NFKB2,NLRC5,NLRP3,NOD2,PLCG2,STAT5A,TLR3,TNF |
| iNOS Signaling | 6,14E00 | CREBBP,FOS,IFNGR2,IKBKE,IRAK2,IRF1,JAK2,JUN,MYD88,NFKB1,NFKB2,NFKBIA,NFKBIB,NFKBID,NFKBIE,STAT1,TRAF6 |
| IL-10 Signaling | 6,14E00 | ARG2,CCR1,FOS,HMOX1,IKBKE,IL10RA,IL1A,IL1B,IL1RN,IL6,JUN,MAP2K4,MAP4K4,NFKB1,NFKB2,NFKBIA,NFKBIB,NFKBID,NFKBIE,SOCS3,TNF,TRAF6 |
| Myc Mediated Apoptosis Signaling | 6,11E00 | ADRB2,BBC3,BCL2L11,CASP8,CFLAR,CREBBP,FAS,IKBKE,MCL1,NFKB1,NFKB2,NGFR,PMAIP1,PRKACB,PRKAG1,TNF,TNFRSF1B,TRAF2 |
| Toll-like Receptor Signaling | 6,06E00 | EIF2AK2,FOS,IL12A,IL1A,IL1B,IL1RN,IRAK2,JUN,MAP2K4,MAP3K1,MAP4K4,MYD88,NFKB1,NFKB2,NFKBIA,TICAM1,TLR3,TNF,TNFAIP3,TRAF1,TRAF4,TRAF6,UBD |
| Hepatic Cholestasis | 5,97E00 | CSF2,CXCL8,CYP3A5,FGF19,HNF4A,IKBKE,IL11,IL12A,IL15,IL1A,IL1B,IL1RN,IL6,IRAK2,JUN,LIF,LTB,MAP2K4,MYD88,NFKB1,NFKB2,NFKBIA,NFKBIB,NFKBID,NFKBIE,NGFR,NR5A2,PPRC1,PRKACB,PRKAG1,PRKCE,RARA,SLCO3A1,TNF,TNFRSF1B,TNFSF10,TNFSF13B,TNFSF15,TNFSF9,TRAF2,TRAF6 |
| Osteoarthritis Pathway | 5,93E00 | ANXA2,ATF4,BMP2,CASP1,CASP10,CASP7,CASP8,CASR,CEBPB,CREB1,CREB5,CREBBP,CXCL8,ELF3,FGF18,FGF2,FGFR1,FGFR3,FOXO3,FZD4,FZD5,HES1,IL1B,ITGA5,LRP1,MTF1,NAMPT,NFKB1,NFKB2,NOTCH1,PGF,PPARGC1A,PRKAG1,PTGS2,S1PR2,SDC4,SIK3,SIRT1,SMAD7,SOX9,SPHK1,SPP1,TNF,[illegible] |
| IL-9 Signaling | 5,88E00 | BCL3,CISH,IRS2,NFKB1,NFKB2,PIK3C2A,PIK3CA,PIK3R1,PIK3R3,SOCS2,SOCS3,STAT1,STAT5A,TNF |
| p38 MAPK Signaling | 5,88E00 | ATF4,BORCS8-MEF2B,CREB1,CREB5,CREBBP,DAXX,DDIT3,DUSP1,DUSP10,FAS,H3-3A/H3-3B,H3C14,IL1A,IL1B,IL1RN,IRAK2,JMJD7-PLA2G4B,MAP2K4,MEF2A,MEF2D,PLA2G4C,PLA2G4F,PLA2G6,RPS6KC1,STAT1,TIFA,TNF,TNFRSF1B,TRAF2,TRAF6 |
| Crosstalk between Dendritic Cells and Natural Killer Cells | 5,86E00 | CD40,CD69,CD83,CSF2,FAS,FSCN1,HLA-A,HLA-B,HLA-C,HLA-DRB4,HLA-E,HLA-F,IFNB1,IL12A,IL15,IL15RA,IL6,LTB,MICB,NFKB1,NFKB2,TLR3,TNF,TNFRSF1B,TNFSF10 |
| RANK Signaling in Osteoclasts | 5,86E00 | BIRC2,BIRC3,FOS,IKBKE,JUN,MAP2K4,MAP3K1,MAP3K2,MAP3K8,MAP3K9,NFATC2,NFKB1,NFKB2,NFKBIA,NFKBIB,NFKBID,NFKBIE,PIK3C2A,PIK3CA,PIK3R1,PIK3R3,PPP3CC,TRAF2,TRAF6,XIAP |
| IL-17A Signaling in Gastric Cells | 5,82E00 | CCL20,CCL5,CXCL1,CXCL10,CXCL11,CXCL8,EGFR,FOS,JUN,MAP2K4,NFKB1,TNF |
| Role of Osteoblasts, Osteoclasts and Chondrocytes in Rheumatoid Arthritis | 5,66E00 | BIRC2,BIRC3,BMP2,COL1A1,CSF1,CSF1R,CSF2,DKK3,FOS,FOXO1,FZD4,FZD5,IKBKE,IL11,IL18R1,IL1A,IL1B,IL1RN,IL6,IL7,ITGA5,JUN,LRP1,MAP2K4,NFAT5,NFATC2,NFKB1,NFKBIA,NFKBIB,NFKBID,NFKBIE,NGFR,PIK3C2A,PIK3CA,PIK3R1,PIK3R3,PPP3CC,SMURF1,SPP1,TNF,TNFRSF1B,TRAF2,TRAF6,WNT10A,XIAP |
| Sumoylation Pathway | 5,63E00 | ARHGDIB,CBX4,CREBBP,DAXX,DIRAS3,EP300,FAS,FOS,ISG20,JUN,MAP2K4,NFKB1,NFKB2,NFKBIA,NR3C1,PML,RHOV,RND1,RND3,SENP6,SIRT1,SNCA,SP100,TDG,XIAP,ZEB1,ZNF217 |
| Role of IL-17A in Arthritis | 5,55E00 | CCL2,CCL20,CCL5,CXCL1,CXCL3,CXCL8,MAP2K4,NFKB1,NFKB2,NFKBIA,NFKBIB,NFKBID,NFKBIE,PIK3C2A,PIK3CA,PIK3R1,PIK3R3,PTGS2 |
| Glucocorticoid Receptor Signaling | 5,48E00 | ADRB2,ARG2,ARID1A,ARID2,BAG1,BCL2L11,CCL2,CCL5,CDKN1A,CREB1,CREBBP,CSF2,CXCL3,CXCL8,DUSP1,EGFR,EP300,FBP1,FOS,FOXO3,HSPA1A/HSPA1B,HSPA1L,HSPA6,ICAM1,IFNB1,IFNLR1,IKBKE,IL10RA,IL15RA,IL18R1,IL1A,IL1B,IL1RN,IL22RA1,IL6,IL6R,IL6ST,IL7R,JAK2,JMJD7-PLA2G4B,JUN,KRT6B,[illegible] |
| T Cell Exhaustion Signaling Pathway | 5,42E00 | ACVR1,ACVR2A,BCL6,CD274,FOS,FOXO1,FOXP1,HLA-A,HLA-B,HLA-C,HLA-DOB,HLA-DPB1,HLA-DQA1,HLA-DRB4,HLA-E,HLA-F,IFNAR2,IL10RA,IL12A,IL6,IL6R,IRF9,JAK2,JUN,LGALS9,MAP2K4,NFAT5,NFATC2,PIK3C2A,PIK3CA,PIK3R1,PIK3R3,PLCG2,PPP2R2A,STAT1,STAT2,STAT4,VEGFA |
| Small Cell Lung Cancer Signaling | 5,41E00 | BIRC2,CDKN1B,CDKN2B,IKBKE,NFKB1,NFKB2,NFKBIA,NFKBIB,NFKBID,NFKBIE,PIK3C2A,PIK3CA,PIK3R1,PIK3R3,PTGS2,RXRB,TRAF1,TRAF2,TRAF3,TRAF4,TRAF6 |
| 4-1BB Signaling in T Lymphocytes | 5,24E00 | IKBKE,JUN,MAP2K4,NFKB1,NFKB2,NFKBIA,NFKBIB,NFKBID,NFKBIE,TNFRSF9,TNFSF9,TRAF1,TRAF2 |
| HMGB1 Signaling | 5,22E00 | CCL2,CSF2,CXCL8,DIRAS3,FOS,ICAM1,IFNGR2,IL11,IL12A,IL15,IL1A,IL1B,IL6,JUN,KAT6A,LIF,LTB,MAP2K4,NFKB1,NFKB2,NGFR,PIK3C2A,PIK3CA,PIK3R1,PIK3R3,RHOV,RND1,RND3,SELE,TNF,TNFRSF1B,TNFSF10,TNFSF13B,TNFSF15,TNFSF9,VCAM1 |
| Atherosclerosis Signaling | 5,17E00 | ALOXE3,APOE,APOL1,CCL2,CD40,COL11A2,COL1A1,CSF1,CXCL8,ICAM1,IL1A,IL1B,IL1RN,IL6,LPA,LYZ,NFKB1,NFKB2,PCYOX1,PDGFA,PDGFD,PLA2G4C,PLA2G4F,PLA2G6,PLA2R1,PLAAT4,PNPLA3,SELE,TNF,VCAM1 |
| Role of IL-17F in Allergic Inflammatory Airway Diseases | 5,14E00 | ATF4,CCL2,CREB1,CREB5,CREBBP,CSF2,CXCL1,CXCL10,CXCL8,IL11,IL1B,IL6,NFKB1,NFKB2,TRAF6 |
| Circadian Rhythm Signaling | 5,07E00 | ATF4,BHLHE40,BHLHE41,CREB1,CREB5,CREBBP,CRY1,CRY2,GRIN2A,GRINA,NR1D1,PER1,PER2 |
| Retinoic acid Mediated Apoptosis Signaling | 4,82E00 | CASP8,CFLAR,IFNB1,IRF1,PARP10,PARP12,PARP14,PARP3,PARP8,PARP9,RARA,RXRB,TIPARP,TNFRSF10A,TNFRSF10B,TNFRSF10D,TNFSF10,TNKS2 |
| IL-12 Signaling and Production in Macrophages | 4,75E00 | APOE,APOL1,CD40,CEBPB,EP300,FOS,IKBKE,IL12A,IRF1,IRF8,JUN,LPA,LYZ,MAP2K4,MAP3K8,MYD88,NFKB1,NFKB2,NFKBIB,PCYOX1,PIK3C2A,PIK3CA,PIK3R1,PIK3R3,PRKCE,REL,STAT1,STAT4,TNF,TRAF6 |
| Erythropoietin Signaling | 4,74E00 | BIRC2,BIRC3,CSF2,CXCL8,FOS,FOXO3,IL11,IL12A,IL15,IL1A,IL1B,IL6,IRS2,JAK2,JUN,LIF,LTB,NFKB1,NFKB2,NFKBIA,NFKBIB,NFKBID,NFKBIE,NOS3,PIK3C2A,PIK3CA,PIK3R1,PIK3R3,PRKCE,STAT5A,TNF,TNFSF10,TNFSF13B,TNFSF15,TNFSF9,XIAP |
| Communication between Innate and Adaptive Immune Cells | 4,7E00 | B2M,CCL5,CD40,CD83,CSF2,CXCL10,CXCL8,HLA-A,HLA-B,HLA-C,HLA-DRB4,HLA-E,HLA-F,IFNB1,IGHE,IL12A,IL15,IL1A,IL1B,IL1RN,IL6,TLR3,TNF,TNFSF13B |
| Altered T Cell and B Cell Signaling in Rheumatoid Arthritis | 4,7E00 | CD40,CSF1,CSF2,FAS,HLA-A,HLA-B,HLA-DOB,HLA-DQA1,IL12A,IL15,IL1A,IL1B,IL1RN,IL6,LTB,NFKB1,NFKB2,RELB,SPP1,TLR3,TNF,TNFSF13B,TRAF3 |
| LXR/RXR Activation | 4,69E00 | ABCA1,ABCG1,APOE,APOL1,ARG2,C3,C4A/C4B,CCL2,FDFT1,GC,IL1A,IL1B,IL1RN,IL6,LPA,LYZ,MYLIP,NFKB1,NFKB2,NGFR,PCYOX1,PTGS2,RXRB,SAA2,SCD,TLR3,TNF,TNFRSF1B |
| Differential Regulation of Cytokine Production in Macrophages and T Helper Cells by IL-17A and I | 4,67E00 | CCL2,CCL5,CSF2,CSF3,CXCL1,IL12A,IL1B,IL6,TNF |
| T Helper Cell Differentiation | 4,64E00 | BCL6,CD40,GATA3,HLA-A,HLA-B,HLA-DOB,HLA-DQA1,ICOSLG/LOC102723996,IFNGR2,IL10RA,IL12A,IL18R1,IL6,IL6R,IL6ST,NGFR,STAT1,STAT4,TNF,TNFRSF1B |
| Th2 Pathway | 4,56E00 | ACVR1,ACVR2A,BHLHE41,CCR1,CCR4,CD40,CHD4,GATA3,HAVCR1,HLA-A,HLA-B,HLA-DOB,HLA-DPB1,HLA-DQA1,HLA-DRB4,ICAM1,ICOSLG/LOC102723996,IL12A,JAK2,JUN,NFATC2,NFKB1,NOTCH1,PIK3C2A,PIK3CA,PIK3R1,PIK3R3,SOCS3,STAT4,STAT5A |
| MIF Regulation of Innate Immunity | 4,44E00 | FOS,JMJD7-PLA2G4B,JUN,MAP2K4,NFKB1,NFKB2,NFKBIA,NFKBIB,NFKBID,NFKBIE,PLA2G4C,PLA2G4F,PLA2G6,PTGS2 |
| B Cell Receptor Signaling | 4,4E00 | ATF4,BCL10,BCL6,CREB1,CREB5,CREBBP,DAPP1,EGR1,FOXO1,GAB1,IGHE,IKBKE,JUN,LYN,MAP2K4,MAP3K1,MAP3K2,MAP3K8,MAP3K9,NFAT5,NFATC2,NFKB1,NFKB2,NFKBIA,NFKBIB,NFKBID,NFKBIE,PIK3AP1,PIK3C2A,PIK3CA,PIK3R1,PIK3R3,PLCG2,POU2F2,PPP3CC,SYNJ1,VAV3 |
| IL-17A Signaling in Airway Cells | 4,4E00 | CCL20,CXCL1,CXCL3,IKBKE,IL6,JAK2,MAP2K4,NFKB1,NFKB2,NFKBIA,NFKBIB,NFKBID,NFKBIE,PIK3C2A,PIK3CA,PIK3R1,PIK3R3,TRAF6 |
| CD27 Signaling in Lymphocytes | 4,39E00 | CASP8,FOS,IKBKE,JUN,MAP2K4,MAP3K1,MAP3K2,MAP3K8,MAP3K9,NFKB1,NFKB2,NFKBIA,NFKBIB,NFKBID,NFKBIE,TRAF2 |
| Apoptosis Signaling | 4,39E00 | BCL2L11,BIRC2,BIRC3,BIRC6,CAPN8,CASP10,CASP7,CASP8,FAS,IKBKE,MAP2K4,MAP4K4,MCL1,NFKB1,NFKB2,NFKBIA,NFKBIB,NFKBID,NFKBIE,PLCG2,PRKCE,TNF,TNFRSF1B,XIAP |
| RAR Activation | 4,35E00 | ADH1C,AKR1B10,ARID1A,ARID2,BMP2,CITED2,CREBBP,DUSP1,EP300,FOS,JAK2,JUN,MAP2K4,MAP3K1,NFKB1,NFKB2,NR2F1,NRIP1,PIK3CA,PIK3R1,PIK3R3,PML,PNRC1,PPARGC1A,PRKACB,PRKAG1,PRKCE,RARA,RDH5,REL,RELB,RXRB,SCAND1,SMAD7,SMARCA2,STAT5A,TNIP1,VEGFA |
| Role of Hypercytokinemia/hyperchemokinemia in the Pathogenesis of Influenza | 4,32E00 | CCL2,CCL5,CCR1,CXCL10,CXCL8,IFNB1,IFNL1,IL12A,IL15,IL1A,IL1B,IL1RN,IL6,TNF |
| ERK5 Signaling | 4,19E00 | ATF4,BORCS8-MEF2B,CREB1,CREB5,CREBBP,EGFR,FOS,FOSL1,FOXO3,GAB1,GNA13,IL6ST,LIF,MAP3K2,MAP3K8,MEF2A,MEF2D,RPS6KC1,SGK1 |
| MIF-mediated Glucocorticoid Regulation | 4,17E00 | JMJD7-PLA2G4B,NFKB1,NFKB2,NFKBIA,NFKBIB,NFKBID,NFKBIE,NR3C1,PLA2G4C,PLA2G4F,PLA2G6,PTGS2 |
| Unfolded protein response | 4,07E00 | ATF4,CEBPB,CEBPD,DDIT3,EIF2AK3,ERN1,ERO1B,HSPA1A/HSPA1B,HSPA1L,HSPA6,HSPH1,INSIG1,NFE2L2,PPP1R15A,TRAF2,XBP1 |
| PI3K Signaling in B Lymphocytes | 4,02E00 | ATF3,ATF4,BCL10,C3,CD40,CREB1,DAPP1,FOS,FOXO3,IKBKE,IRS2,JUN,LYN,NFAT5,NFATC2,NFKB1,NFKB2,NFKBIA,NFKBIB,NFKBID,NFKBIE,PIK3AP1,PIK3CA,PIK3R1,PLCG2,PLCH1,PLEKHA4,PPP3CC,VAV3 |
| JAK/Stat Signaling | 4,02E00 | CDKN1A,CEBPB,CISH,FOS,IL6,JAK2,JUN,NFKB1,NFKB2,PIK3C2A,PIK3CA,PIK3R1,PIK3R3,SOCS1,SOCS2,SOCS3,STAT1,STAT2,STAT4,STAT5A |
| Prolactin Signaling | 3,94E00 | CEBPB,CREBBP,EP300,FOS,IRF1,JAK2,JUN,NMI,NR3C1,PIK3C2A,PIK3CA,PIK3R1,PIK3R3,PLCG2,PRKCE,SOCS1,SOCS2,SOCS3,STAT1,STAT5A |
| Cardiac Hypertrophy Signaling (Enhanced) | 3,89E00 | ACVR1,ACVR2A,ADRA2A,ADRB2,AKAP13,BORCS8-MEF2B,CSF2,CXCL8,EDN1,EP300,FGF18,FGF19,FGF2,FGFR1,FGFR3,FZD4,FZD5,GATA4,GNA13,HAND1,HDAC9,IFNLR1,IKBKE,IL10RA,IL11,IL12A,IL15,IL15RA,IL18R1,IL1A,IL1B,IL22RA1,IL6,IL6R,IL6ST,IL7R,ITGA5,JAK2,JUN,LIF,LTB,MAP2K4,MAP3K1,MAP3K2,[illegible] |
| Adipogenesis pathway | 3,86E00 | BMP2,CDK5,CEBPB,CEBPD,DDIT3,DGKD,EGR2,FBXW7,FGF2,FGFR1,FGFR3,FOXO1,FZD4,FZD5,HDAC9,KAT6A,KLF5,KMT2B,NOCT,NR1D2,PER2,RPS6KC1,SETDB1,SIRT1,SOX9,TNF,TXNIP,XBP1 |
| IL-15 Production | 3,84E00 | ABL2,CLK1,CLK2,CLK4,CSF1R,DYRK1A,EGFR,EPHA4,FGFR1,FGFR3,FLT3LG,FRK,IFNB1,IL15,IL6,IRF1,JAK2,LYN,MAP2K4,NFKB1,NFKB2,ROS1,STAT1,STYK1,TEC,TXK |
| SAPK/JNK Signaling | 3,79E00 | CRKL,DAXX,DUSP10,DUSP4,DUSP8,GAB1,GADD45A,GNA13,JUN,MAP2K4,MAP3K1,MAP3K2,MAP3K9,MAP4K3,MAP4K4,MAP4K5,MAPK8IP2,PIK3C2A,PIK3CA,PIK3R1,PIK3R3,RIPK1,TRAF2 |
| Differential Regulation of Cytokine Production in Intestinal Epithelial Cells by IL-17A and IL-17F | 3,64E00 | CCL2,CCL5,CSF2,CSF3,CXCL1,IL12A,IL1A,IL1B,TNF |
| Tec Kinase Signaling | 3,64E00 | DIRAS3,FAS,FOS,FRK,GNA13,GNB4,GNG12,ITGA5,JAK2,LYN,MAP2K4,NFKB1,NFKB2,PIK3C2A,PIK3CA,PIK3R1,PIK3R3,PLCG2,PRKCE,RHOV,RND1,RND3,STAT1,STAT2,STAT4,STAT5A,TEC,TNF,TNFRSF10A,TNFRSF10B,TNFSF10,TXK,VAV3 |
| p53 Signaling | 3,62E00 | BBC3,CDKN1A,DRAM1,EP300,FAS,GADD45A,GADD45B,HDAC9,JUN,MDM4,PIK3C2A,PIK3CA,PIK3R1,PIK3R3,PMAIP1,PML,SIRT1,THBS1,TNFRSF10A,TNFRSF10B,TP53BP2,TRIM29 |
| IL-23 Signaling Pathway | 3,57E00 | CSF2,IL1B,JAK2,NFKB1,NFKB2,NFKBIA,PIK3C2A,PIK3CA,PIK3R1,PIK3R3,SOCS3,STAT4,TNF |
| Natural Killer Cell Signaling | 3,53E00 | B2M,COL11A2,COL1A1,HLA-A,HLA-B,HLA-C,HLA-E,HLA-F,HSPA1A/HSPA1B,HSPA1L,HSPA6,IL12A,IL15,IL18R1,JAK2,MAP3K1,MAP3K2,MAP3K8,MAP3K9,MICB,MYD88,NFAT5,NFATC2,NFKB1,NFKB2,PIK3C2A,PIK3CA,PIK3R1,PIK3R3,PLCG2,PVR,RAET1L,STAT4,TNFSF10,TRAF6,VAV3 |
| ILK Signaling | 3,52E00 | ACTN2,ATF4,BMP2,CREB1,CREB5,CREBBP,DIRAS3,FERMT2,FOS,IRS2,ITGB8,JUN,MAP2K4,MYH10,MYL6B,NFKB1,NFKB2,PGF,PIK3C2A,PIK3CA,PIK3R1,PIK3R3,PPP1R12A,PPP2R2A,PTGS2,RHOV,RICTOR,RND1,RND3,SNAI1,TMSB10/TMSB4X,TNF,VEGFA,VEGFC,VIM |
| Role of JAK1, JAK2 and TYK2 in Interferon Signaling | 3,48E00 | IFNAR2,IFNB1,IFNGR2,JAK2,NFKB1,NFKB2,SOCS1,STAT1,STAT2 |
| Molecular Mechanisms of Cancer | 3,42E00 | ARHGEF4,BBC3,BCL2L11,BIRC2,BIRC3,BMP2,BRAF,CASP10,CASP7,CASP8,CDK12,CDK17,CDK18,CDK5,CDK8,CDKN1A,CDKN1B,CDKN2B,CFLAR,CREBBP,DAXX,DIRAS3,E2F3,E2F6,E2F7,EP300,FAS,FOS,FOXO1,FZD4,FZD5,GAB1,GNA13,ITGA5,JAK2,JUN,LRP1,MAP2K4,NFKB1,NFKB2,NFKBIA,NFKBIB,NFKBID,[illegible] |
| PEDF Signaling | 3,39E00 | ARHGAP22,BDNF,CASP7,CASP8,CFLAR,FAS,IKBKE,NFKB1,NFKB2,NFKBIA,NFKBIB,NFKBID,NFKBIE,PIK3C2A,PIK3CA,PIK3R1,PIK3R3,SOD2,ZEB1 |
| Aryl Hydrocarbon Receptor Signaling | 3,37E00 | AHR,AHRR,ALDH7A1,CDKN1A,CDKN1B,CYP1A1,CYP1B1,DHFR,EP300,FAS,FOS,IL1A,IL1B,IL6,JUN,MGST2,NCOA3,NCOA7,NFE2L2,NFIX,NFKB1,NFKB2,NR2F1,NRIP1,RARA,RBL2,RXRB,TNF |
| Inflammasome pathway | 3,37E00 | CASP1,CASP8,IL1B,MYD88,NFKB1,NFKB2,NLRP3,NOD2 |
| Pancreatic Adenocarcinoma Signaling | 3,34E00 | CDKN1A,CDKN1B,CDKN2B,E2F3,E2F6,E2F7,EGFR,HBEGF,HMOX1,JAK2,MAP2K4,NFKB1,NFKB2,NOTCH1,PGF,PIK3C2A,PIK3CA,PIK3R1,PIK3R3,PTGS2,STAT1,VEGFA,VEGFC |
| Role of JAK family kinases in IL-6-type Cytokine Signaling | 3,33E00 | IL6,IL6R,IL6ST,JAK2,MAP2K4,SOCS1,SOCS3,STAT1,STAT5A |
| OX40 Signaling Pathway | 3,29E00 | B2M,HLA-A,HLA-B,HLA-C,HLA-DOB,HLA-DPB1,HLA-DQA1,HLA-DRB4,HLA-E,HLA-F,JUN,MAP2K4,NFKB1,NFKB2,NFKBIA,NFKBIB,NFKBID,NFKBIE,TRAF2,TRAF3 |
| HOTAIR Regulatory Pathway | 3,23E00 | AGO2,AGO3,BRD4,CDKN1A,COL1A1,CREBBP,EP300,H3-3A/H3-3B,H3C14,ICAM1,IRF1,KMT2A,KMT2C,LAMTOR5,MMP7,MRTFA,MTF2,NFKB1,NFKB2,NFKBIA,PIK3C2A,PIK3CA,PIK3R1,PIK3R3,SETDB1,SPP1,SUZ12,VIM,WNT10A,XIAP |
| Endoplasmic Reticulum Stress Pathway | 3,2E00 | ATF4,CASP7,DDIT3,EIF2AK3,ERN1,TAOK3,TRAF2,XBP1 |
| Graft-versus-Host Disease Signaling | 3,17E00 | FAS,HLA-A,HLA-B,HLA-C,HLA-DOB,HLA-DQA1,HLA-E,HLA-F,IL1A,IL1B,IL1RN,IL6,TNF |
| IL-1 Signaling | 3,16E00 | FOS,GNA13,GNB4,GNG12,IKBKE,IL1A,IRAK2,JUN,MAP2K4,MAP3K1,MYD88,NFKB1,NFKB2,NFKBIA,NFKBIB,NFKBID,NFKBIE,PRKACB,PRKAG1,TRAF6 |
| PKCθ Signaling in T Lymphocytes | 3,13E00 | BCL10,CACNG4,FOS,HLA-A,HLA-B,HLA-DOB,HLA-DQA1,IKBKE,JUN,MAP2K4,MAP3K1,MAP3K2,MAP3K8,MAP3K9,NFAT5,NFATC2,NFKB1,NFKB2,NFKBIA,NFKBIB,NFKBID,NFKBIE,PIK3C2A,PIK3CA,PIK3R1,PIK3R3,PLCG2,PPP3CC,VAV3 |
| Type II Diabetes Mellitus Signaling | 3,07E00 | CACNG4,CEBPB,IKBKE,IRS2,MAFA,MAP2K4,MAP3K1,NFKB1,NFKB2,NFKBIA,NFKBIB,NFKBID,NFKBIE,NGFR,PIK3C2A,PIK3CA,PIK3R1,PIK3R3,PRKAG1,PRKCE,SLC27A3,SOCS1,SOCS2,SOCS3,TNF,TNFRSF1B,TRAF2 |
| STAT3 Pathway | 3,06E00 | CDKN1A,CISH,EGFR,FGF2,FGFR1,FGFR3,IFNLR1,IL10RA,IL15RA,IL18R1,IL1A,IL1B,IL22RA1,IL6R,IL6ST,IL7R,JAK2,MAP2K4,MAP3K9,NDUFA13,NGFR,PIM1,SOCS1,SOCS2,SOCS3,VEGFA |
| NGF Signaling | 3,06E00 | ATF4,CREB1,CREB5,CREBBP,GAB1,IKBKE,MAP2K4,MAP3K1,MAP3K2,MAP3K8,MAP3K9,NFKB1,NFKB2,NGFR,PIK3C2A,PIK3CA,PIK3R1,PIK3R3,PLCG2,RPS6KC1,TRAF4,TRAF6,TRIO |
| Pathogenesis of Multiple Sclerosis | 3,04E00 | CCL5,CCR1,CXCL10,CXCL11,CXCL9 |
| Apelin Pancreas Signaling Pathway | 3 | DDIT3,EIF2AK3,ERN1,MAP2K4,NFKB1,NFKB2,PIK3C2A,PIK3CA,PIK3R1,PIK3R3,PRKACB,PRKAG1 |
| Role of NFAT in Regulation of the Immune Response | 2,95E00 | BORCS8-MEF2B,CSNK1G1,FOS,GATA4,GNA13,GNB4,GNG12,HLA-A,HLA-B,HLA-DOB,HLA-DQA1,IKBKE,JUN,LYN,MEF2A,MEF2D,NFAT5,NFATC2,NFKB1,NFKB2,NFKBIA,NFKBIB,NFKBID,NFKBIE,PIK3C2A,PIK3CA,PIK3R1,PIK3R3,PLCG2,PPP3CC,RCAN1,XPO1 |
| NRF2-mediated Oxidative Stress Response | 2,93E00 | ATF4,BACH1,CAT,CREBBP,DNAJA4,DNAJB1,DNAJB4,DNAJB5,DNAJC6,EIF2AK3,EP300,EPHX1,FOS,FOSL1,HERPUD1,HMOX1,HSPB8,JUN,JUNB,JUND,MAFF,MAFG,MAP2K4,MAP3K1,MGST2,NFE2L2,PIK3C2A,PIK3CA,PIK3R1,PIK3R3,PRKCE,SOD2,SQSTM1 |
| FAT10 Signaling Pathway | 2,92E00 | NUB1,PSME2,SQSTM1,TNF,UBA6,UBD,UBE2Z |
| Huntington's Disease Signaling | 2,85E00 | ATF4,ATP5PF,BDNF,CAPN8,CASP1,CASP10,CASP7,CASP8,CDK5,CREB1,CREB5,CREBBP,DNAJB1,EGFR,EP300,GLS,GNB4,GNG12,HDAC9,HSPA1A/HSPA1B,HSPA1L,HSPA6,IFT57,JUN,MAP2K4,NAPA,PIK3C2A,PIK3CA,PIK3R1,PIK3R3,POLR2J,PRKCE,PSME2,SDHB,SGK1,SNCA,STX16,UBD,VAMP2 |
| Airway Pathology in Chronic Obstructive Pulmonary Disease | 2,85E00 | CCL2,CCL20,CSF2,CXCL1,CXCL3,CXCL8,FGF18,FGF19,FGF2,IL11,IL12A,IL15,IL1A,IL1B,IL6,LCN10,LIF,LTB,TNF,TNFSF10,TNFSF13B,TNFSF15,TNFSF9 |
| Role of Oct4 in Mammalian Embryonic Stem Cell Pluripotency | 2,81E00 | ETS2,FBXO15,FOXA2,KDM5B,MEF2A,NR2F1,NR5A2,PHC3,RARA,RXRB,SPP1,TDRD7 |
| Th17 Activation Pathway | 2,81E00 | AHR,CCL20,CSF2,IL12A,IL1B,IL6,IL6R,IRAK2,JAK2,MYD88,NFAT5,NFATC2,NFKB1,NFKB2,PTGER2,PTGER4,SOCS3,STAT4,TRAF6 |
| FAT10 Cancer Signaling Pathway | 2,81E00 | ACVR1,ACVR2A,EEF1A1,IKBKE,IL6,NFKB1,NFKB2,NFKBIA,NGFR,TNF,TNFRSF1B,UBD |
| FXR/RXR Activation | 2,8E00 | APOE,APOL1,BAAT,C3,C4A/C4B,CREBBP,FBP1,FGF19,FOXA2,FOXA3,FOXO1,GC,HNF4A,IL1A,IL1B,IL1RN,MAP2K4,NR5A2,PCYOX1,PPARGC1A,RARA,SAA2,SDC1,TNF |
| IL-8 Signaling | 2,8E00 | BRAF,CXCL1,CXCL8,DIRAS3,EGFR,FOS,GNA13,GNB4,GNG12,HBEGF,HMOX1,ICAM1,IKBKE,IRAK2,ITGAM,JUN,MAP2K4,MAP4K4,NFKB1,NFKBIB,PGF,PIK3C2A,PIK3CA,PIK3R1,PIK3R3,PRKCE,PTGS2,RHOV,RND1,RND3,TRAF6,VCAM1,VEGFA,VEGFC |
| Salvage Pathways of Pyrimidine Ribonucleotides | 2,8E00 | ACVR2A,APOBEC3F,APOBEC3G,BRAF,CDA,CDK18,CDK5,CDK8,CMPK2,DYRK1A,EIF2AK2,MAP2K4,MAP3K8,MAP3K9,MAPK6,NEK2,PIM1,PRKCE,SGK1,UPP1 |
| UVA-Induced MAPK Signaling | 2,8E00 | EGFR,FOS,JUN,MAP2K4,PARP10,PARP12,PARP14,PARP3,PARP8,PARP9,PIK3C2A,PIK3CA,PIK3R1,PIK3R3,PLCG2,PLCH1,RPS6KC1,STAT1,TIPARP,TNKS2 |
| PI3K/AKT Signaling | 2,79E00 | CDKN1A,CDKN1B,FOXO1,FOXO3,GAB1,GDF15,IFNLR1,IKBKE,IL10RA,IL15RA,IL18R1,IL22RA1,IL6R,IL6ST,IL7R,ITGA5,JAK2,MAP3K8,MCL1,NFKB1,NFKB2,NFKBIA,NFKBIB,NFKBID,NFKBIE,NOS3,PIK3CA,PIK3R1,PIK3R3,PPP2R2A,PTGS2,SYNJ1 |
| IL-7 Signaling Pathway | 2,78E00 | BCL6,CDKN1B,FOXO1,FOXO3,FOXO4,IL7,IL7R,JUN,LYN,MCL1,PIK3C2A,PIK3CA,PIK3R1,PIK3R3,SOCS1,STAT1,STAT5A |
| MSP-RON Signaling In Cancer Cells Pathway | 2,76E00 | ATF4,BRAF,CREB1,CREB5,CREBBP,EGFR,ELF1,ELF3,ETS2,FOS,JUN,KLK6,KLK7,NFKB1,NFKB2,NFKBIA,PGF,PIK3C2A,PIK3CA,PIK3R1,PIK3R3,PLAUR,VEGFA,VEGFC,VIM |
| ERK/MAPK Signaling | 2,72E00 | ATF4,BCAR1,BRAF,CREB1,CREB5,CREBBP,CRKL,DUSP1,DUSP2,DUSP4,ELF1,ELF3,ETS2,FOS,H3-3A/H3-3B,H3C14,ITGA5,JMJD7-PLA2G4B,KSR1,PIK3C2A,PIK3CA,PIK3R1,PIK3R3,PLA2G4C,PLA2G4F,PLA2G6,PLCG2,PPP1R12A,PPP1R7,PPP2R2A,PRKACB,PRKAG1,PRKCE,STAT1 |
| Granulocyte Adhesion and Diapedesis | 2,71E00 | C5,CCL2,CCL20,CCL22,CCL5,CLDN10,CLDN16,CSF3,CX3CL1,CXCL1,CXCL10,CXCL11,CXCL2,CXCL3,CXCL8,CXCL9,HRH1,ICAM1,IL1A,IL1B,IL1RN,ITGAM,MMP7,NGFR,SDC1,SDC4,SELE,TNF,TNFRSF1B,VCAM1 |
| Pyridoxal 5'-phosphate Salvage Pathway | 2,71E00 | ACVR2A,BRAF,CDK18,CDK5,CDK8,DYRK1A,EIF2AK2,MAP2K4,MAP3K8,MAP3K9,MAPK6,NEK2,PIM1,PRKCE,SGK1 |
| PDGF Signaling | 2,7E00 | ABL2,CRKL,EIF2AK2,FOS,JAK2,JUN,MAP2K4,MAP3K1,PDGFA,PDGFD,PIK3C2A,PIK3CA,PIK3R1,PIK3R3,PLCG2,SPHK1,STAT1,SYNJ1 |
| CD28 Signaling in T Helper Cells | 2,7E00 | BCL10,FOS,HLA-A,HLA-B,HLA-DOB,HLA-DQA1,IKBKE,JUN,MAP2K4,MAP3K1,NFAT5,NFATC2,NFKB1,NFKB2,NFKBIA,NFKBIB,NFKBID,NFKBIE,PIK3C2A,PIK3CA,PIK3R1,PIK3R3,PPP3CC |
| Endocannabinoid Cancer Inhibition Pathway | 2,68E00 | ATF3,ATF4,CASP1,CASP10,CASP7,CASP8,CDKN1A,CDKN1B,CREB1,CREB5,CREBBP,DDIT3,GNA13,MAP2K4,NOS3,PGF,PIK3C2A,PIK3CA,PIK3R1,PIK3R3,PRKACB,PRKAG1,RPTOR,VEGFA,VEGFC,VIM |
| IL-17 Signaling | 2,66E00 | CCL2,CEBPB,CXCL1,CXCL10,CXCL11,CXCL8,IL6,JAK2,JUN,MAP2K4,NFKB1,PIK3C2A,PIK3CA,PIK3R1,PIK3R3,PTGS2,TRAF6 |
| Role of Cytokines in Mediating Communication between Immune Cells | 2,66E00 | CSF2,CSF3,CXCL8,IFNB1,IFNL1,IL12A,IL15,IL1A,IL1B,IL1RN,IL32,IL6,TNF |
| Sirtuin Signaling Pathway | 2,63E00 | ABCA1,ARG2,ATG2B,ATP5PF,BCL2L11,CRTC2,CXCL8,FOXO1,FOXO3,FOXO4,GADD45A,GADD45B,GLS,H1-0,H1-3,H1-4,H3-3A/H3-3B,H3C14,IDH2,JUN,MAPK6,NAMPT,NDRG1,NDUFA1,NDUFA13,NDUFA2,NDUFB1,NEDD4,NFE2L2,NFKB1,NFKB2,NOS3,PGK1,PPARGC1A,RBBP8,RPTOR,SDHB,SIRT1,SMARCA5,SOD2,[illegible] |
| Estrogen-Dependent Breast Cancer Signaling | 2,61E00 | ATF4,CREB1,CREB5,CREBBP,EGFR,FOS,HSD17B14,HSD17B2,JUN,NFKB1,NFKB2,PIK3C2A,PIK3CA,PIK3R1,PIK3R3,STAT5A |
| Hypoxia Signaling in the Cardiovascular System | 2,61E00 | ATF4,BIRC6,CREB1,CREB5,CREBBP,EDN1,EP300,JUN,NFKBIA,NFKBIB,NFKBID,NFKBIE,NOS3,UBE2L6,UBE2Z,VEGFA |
| Role of Tissue Factor in Cancer | 2,59E00 | ARRB1,CCN1,CSF1,CSF2,CXCL1,CXCL8,EGFR,EGR1,FRK,GNA13,HBEGF,IL1B,JAK2,LYN,PIK3C2A,PIK3CA,PIK3R1,PIK3R3,PLAUR,STAT5A,VEGFA,VEGFC |
| Ceramide Signaling | 2,59E00 | CNKSR1,FOS,JUN,KSR1,MAP2K4,MAP3K1,NFKB1,NFKB2,NGFR,PIK3C2A,PIK3CA,PIK3R1,PIK3R3,PPP2R2A,S1PR2,SPHK1,TNF,TNFRSF1B |
| Antioxidant Action of Vitamin C | 2,59E00 | CSF2,HMOX1,IKBKE,JAK2,MAP2K4,NFKB1,NFKB2,NFKBIA,NFKBIB,NFKBID,NFKBIE,PLA2G4C,PLA2G4F,PLA2G6,PLA2R1,PLAAT4,PLCG2,PLCH1,PNPLA3,STAT5A,TNF |
| Protein Kinase A Signaling | 2,58E00 | AKAP10,AKAP13,AKAP17A,AKAP7,ATF4,BRAF,CDKN3,CREB1,CREB5,CREBBP,DUSP1,DUSP10,DUSP15,DUSP16,DUSP2,DUSP4,DUSP5,DUSP8,EYA1,GNA13,GNB4,GNG12,H1-0,H1-3,H1-4,H3-3A/H3-3B,H3C14,MAP3K1,MTMR3,MYH10,MYL6B,NFAT5,NFATC2,NFKB1,NFKB2,NFKBIA,NFKBIB,NFKBID,NFKBIE,NGFR,[illegible] |
| NF-κB Activation by Viruses | 2,49E00 | EIF2AK2,IKBKE,MAP3K1,NFKB1,NFKB2,NFKBIA,NFKBIB,NFKBID,NFKBIE,PIK3C2A,PIK3CA,PIK3R1,PIK3R3,PRKCE,RIPK1,TRAF2 |
| Neurotrophin/TRK Signaling | 2,49E00 | ATF4,BDNF,CREB1,CREB5,CREBBP,FOS,FRS2,GAB1,JUN,MAP2K4,NGFR,PIK3C2A,PIK3CA,PIK3R1,PIK3R3,SPRY2 |
| iCOS-iCOSL Signaling in T Helper Cells | 2,49E00 | CD40,HLA-A,HLA-B,HLA-DOB,HLA-DQA1,ICOSLG/LOC102723996,IKBKE,NFAT5,NFATC2,NFKB1,NFKB2,NFKBIA,NFKBIB,NFKBID,NFKBIE,PIK3C2A,PIK3CA,PIK3R1,PIK3R3,PLEKHA4,PPP3CC |
| ATM Signaling | 2,48E00 | ATF4,CCNB2,CDKN1A,CREB1,CREB5,CREBBP,GADD45A,GADD45B,JUN,MAP2K4,MDM4,NFKBIA,PPM1D,PPP2R2A,RBBP8,TLK1,TLK2,TRRAP,ZEB1 |
| FGF Signaling | 2,42E00 | ATF4,CREB1,CREB5,CREBBP,CRKL,FGF18,FGF19,FGF2,FGFR1,FGFR3,FRS2,GAB1,MAP3K1,PIK3C2A,PIK3CA,PIK3R1,PIK3R3 |
| Prostate Cancer Signaling | 2,42E00 | ATF4,CDKN1A,CDKN1B,CREB1,CREB5,CREBBP,FOXO1,NFKB1,NFKB2,NFKBIA,NFKBIB,NFKBID,NFKBIE,NKX3-1,PIK3C2A,PIK3CA,PIK3R1,PIK3R3 |
| HGF Signaling | 2,4E00 | CDKN1A,CRKL,ELF1,ELF3,ETS2,FOS,GAB1,IL6,ITGA5,JUN,MAP2K4,MAP3K1,MAP3K2,MAP3K8,MAP3K9,PIK3C2A,PIK3CA,PIK3R1,PIK3R3,PLCG2,PRKCE,PTGS2 |
| Relaxin Signaling | 2,39E00 | BRAF,CREB1,CTH,FOS,GNA13,GNB4,GNG12,JUN,NFKB1,NFKB2,NFKBIA,NFKBIB,NFKBID,NFKBIE,NOS3,PDE10A,PDE4B,PDE4D,PIK3C2A,PIK3CA,PIK3R1,PIK3R3,PRKACB,PRKAG1,SMPDL3B,VEGFA |
| Growth Hormone Signaling | 2,38E00 | FOS,IGF2,JAK2,PIK3C2A,PIK3CA,PIK3R1,PIK3R3,PLCG2,PRKCE,RPS6KC1,SOCS1,SOCS2,SOCS3,STAT1,STAT5A |
| T Cell Receptor Signaling | 2,38E00 | BCL10,FOS,IKBKE,JUN,MAP2K4,MAP3K1,NFAT5,NFATC2,NFKB1,NFKB2,NFKBIA,PIK3C2A,PIK3CA,PIK3R1,PIK3R3,PPP3CC,RASGRP1,TEC,TXK,VAV3 |
| Hematopoiesis from Multipotent Stem Cells | 2,36E00 | CSF1,CSF2,CSF3,IL15,IL7 |
| BEX2 Signaling Pathway | 2,32E00 | BBC3,CDKN1A,JUN,LMO2,MAP2,MAP2K4,NFKB1,NFKB2,NFKBIA,NGFR,PGF,PLXNC1,PPP2R2A,SPP1,VEGFA,VEGFC |
| Inhibition of ARE-Mediated mRNA Degradation Pathway | 2,32E00 | AGO2,AGO3,CNOT4,DCP1A,EDC3,LTB,MAPK6,NGFR,PAPOLG,PPP2R2A,PRKACB,PRKAG1,PSME2,TNF,TNFRSF1B,TNFSF10,TNFSF13B,TNFSF15,TNFSF18,TNFSF9,XRN1,ZFP36 |
| PFKFB4 Signaling Pathway | 2,3E00 | ATF4,CREB1,CREB5,CREBBP,FBP1,FGF2,MAP2K4,NCOA3,PRKACB,PRKAG1,XDH |
| Estrogen Receptor Signaling | 2,29E00 | ARG2,ATF4,CDK8,CDKN1A,CREB1,CREB5,CREBBP,EGFR,EP300,FOS,FOXO1,FOXO3,FOXO4,GNA13,HES1,IGF2,JAK2,JUN,MED12L,MED13,MED13L,MED15,MMP7,MYL6B,NCOA3,NFKB1,NFKB2,NOS3,NOTCH1,NR3C1,NRIP1,PGF,PIK3C2A,PIK3CA,PIK3R1,PIK3R3,PLCG2,PLCH1,PPARGC1A,PPP1R12A,PRKACB,[illegible] |
| Cytotoxic T Lymphocyte-mediated Apoptosis of Target Cells | 2,28E00 | B2M,CASP7,CASP8,FAS,HLA-A,HLA-B,HLA-C,HLA-E,HLA-F |
| Maturity Onset Diabetes of Young (MODY) Signaling | 2,23E00 | APOE,APOL1,APOL2,APOL3,APOL4,APOL6,CREBBP,EP300,FOXA2,HNF4A,NFKB1,NFKB2,UCP2 |
| Angiopoietin Signaling | 2,15E00 | FOXO1,IKBKE,NFKB1,NFKB2,NFKBIA,NFKBIB,NFKBID,NFKBIE,NOS3,PIK3C2A,PIK3CA,PIK3R1,PIK3R3,STAT5A,TNIP1 |
| Regulation of IL-2 Expression in Activated and Anergic T Lymphocytes | 2,11E00 | BCL10,FOS,IKBKE,JUN,MAP2K4,MAP3K1,NFAT5,NFATC2,NFKB1,NFKB2,NFKBIA,NFKBIB,NFKBID,NFKBIE,PLCG2,PPP3CC,VAV3 |
| Colorectal Cancer Metastasis Signaling | 2,11E00 | ARRB1,BRAF,DIRAS3,EGFR,FOS,FZD4,FZD5,GNB4,GNG12,IL6,IL6R,IL6ST,JAK2,JUN,LRP1,MAP2K4,MMP7,NFKB1,NFKB2,PGF,PIK3C2A,PIK3CA,PIK3R1,PIK3R3,PRKACB,PRKAG1,PTGER2,PTGER4,PTGS2,RHOV,RND1,RND3,STAT1,TLR3,TNF,VEGFA,VEGFC,WNT10A |
| LPS-stimulated MAPK Signaling | 2,1E00 | CREB1,FOS,IKBKE,JUN,MAP2K4,NFKB1,NFKB2,NFKBIA,NFKBIB,NFKBID,NFKBIE,PIK3C2A,PIK3CA,PIK3R1,PIK3R3,PRKCE |
| Role of JAK1 and JAK3 in γc Cytokine Signaling | 2,1E00 | IL15,IL15RA,IL7,IL7R,IRS2,JAK2,PIK3C2A,PIK3CA,PIK3R1,PIK3R3,SOCS1,SOCS3,STAT1,STAT5A |
| TR/RXR Activation | 2,05E00 | AKR1C1/AKR1C2,BCL3,DIO2,DIO3,EP300,NCOA3,PIK3C2A,PIK3CA,PIK3R1,PIK3R3,PPARGC1A,RXRB,SYT12,THRA,THRB,UCP2 |
| Glycine Degradation (Creatine Biosynthesis) | 2 | GAMT,GATM |
| Role of PI3K/AKT Signaling in the Pathogenesis of Influenza | 1,99E00 | CCL5,CRKL,IFNB1,NFKB1,NFKB2,NFKBIA,NFKBIB,NFKBID,NFKBIE,PIK3C2A,PIK3CA,PIK3R1,PIK3R3 |
| Apelin Endothelial Signaling Pathway | 1,98E00 | BORCS8-MEF2B,CCL2,FOS,GNA13,ICAM1,JUN,KLF2,MAP2K4,MEF2A,MEF2D,NFKB1,NFKB2,NOS3,PIK3C2A,PIK3CA,PIK3R1,PIK3R3,PRKAG1,PRKCE,VCAM1 |
| MSP-RON Signaling Pathway | 1,94E00 | CCL2,CSF1,IL12A,ITGAM,JAK2,KLK6,KLK7,PIK3C2A,PIK3CA,PIK3R1,PIK3R3,TNF |
| Sphingosine-1-phosphate Signaling | 1,9E00 | ASAH1,CASP1,CASP10,CASP7,CASP8,DIRAS3,GNA13,PDGFA,PDGFD,PIK3C2A,PIK3CA,PIK3R1,PIK3R3,PLCG2,PLCH1,RHOV,RND1,RND3,S1PR2,SPHK1 |
| UVB-Induced MAPK Signaling | 1,89E00 | EGFR,FOS,H3-3A/H3-3B,H3C14,JUN,MAP2K4,PIK3C2A,PIK3CA,PIK3R1,PIK3R3,PRKCE |
| Xenobiotic Metabolism Signaling | 1,87E00 | AHR,AHRR,ALDH7A1,CAT,CHST2,CITED2,CREBBP,CYP1A1,CYP1B1,CYP2B6,CYP3A5,EIF2AK3,EP300,HMOX1,HS2ST1,IL1A,IL1B,IL4I1,IL6,MAOB,MAP2K4,MAP3K1,MAP3K2,MAP3K8,MAP3K9,MGST2,NFE2L2,NFKB1,NFKB2,NRIP1,PIK3C2A,PIK3CA,PIK3R1,PIK3R3,PPARGC1A,PPP2R2A,PRKCE,SCAND1,SULT1C2,[illegible] |
| Renin-Angiotensin Signaling | 1,86E00 | CCL2,CCL5,FOS,JAK2,JUN,MAP2K4,MAP3K1,NFKB1,NFKB2,PIK3C2A,PIK3CA,PIK3R1,PIK3R3,PLCG2,PRKACB,PRKAG1,PRKCE,PTGER2,STAT1,TNF |
| TGF-β Signaling | 1,84E00 | ACVR1,ACVR2A,BMP2,CREBBP,EP300,FOS,HNF4A,INHBA,IRF7,JUN,MAP2K4,RNF111,SMAD7,SMURF1,TFE3,TRAF6,VDR |
| Aldosterone Signaling in Epithelial Cells | 1,83E00 | DNAJB1,DNAJB4,DNAJB5,DNAJC22,DNAJC25,DNAJC27,DNAJC6,DUSP1,HSCB,HSPA1A/HSPA1B,HSPA1L,HSPA4L,HSPA6,HSPB8,HSPH1,NEDD4,PIK3C2A,PIK3CA,PIK3R1,PIK3R3,PIP5K1A,PLCG2,PLCH1,PRKCE,SGK1 |
| Cholecystokinin/Gastrin-mediated Signaling | 1,82E00 | BCAR1,BORCS8-MEF2B,DIRAS3,EGFR,EPHA4,FOS,GNA13,IL1A,IL1B,IL1RN,JUN,MAP2K4,MEF2A,MEF2D,PRKCE,PTGS2,RHOV,RND1,RND3,TNF |
| Acute Myeloid Leukemia Signaling | 1,82E00 | BRAF,CSF1R,FLT3LG,IDH1,IDH2,MAP2K4,NFKB1,NFKB2,PIK3C2A,PIK3CA,PIK3R1,PIK3R3,PIM1,PML,RARA,STAT5A |
| IGF-1 Signaling | 1,82E00 | CCN1,FOS,FOXO1,FOXO3,IGFBP2,IRS2,JAK2,JUN,NEDD4,PIK3C2A,PIK3CA,PIK3R1,PIK3R3,PRKACB,PRKAG1,SOCS1,SOCS2,SOCS3 |
| Cardiac Hypertrophy Signaling | 1,8E00 | ADRA2A,ADRB2,BORCS8-MEF2B,CREB1,CREBBP,DIRAS3,EP300,GATA4,GNA13,GNB4,GNG12,HAND1,IL6,IL6R,JUN,MAP2K4,MAP3K1,MAP3K2,MAP3K8,MAP3K9,MEF2A,MEF2D,MYL6B,PIK3C2A,PIK3CA,PIK3R1,PIK3R3,PLCG2,PLCH1,PPP3CC,PRKACB,PRKAG1,RHOV,RND1,RND3 |
| PPARα/RXRα Activation | 1,8E00 | ABCA1,ACVR1,ACVR2A,BCL3,CREBBP,EP300,HELZ2,IKBKE,IL1B,IL6,JAK2,JUN,MAP2K4,MAP4K4,NCOA3,NFKB1,NFKB2,NFKBIA,NFKBIB,NFKBID,NFKBIE,NR2F1,PLCG2,PLCH1,PPARGC1A,PRKACB,PRKAG1,TGS1,TRAF6 |
| Amyotrophic Lateral Sclerosis Signaling | 1,79E00 | ALS2,BIRC2,BIRC3,CAPN8,CASP1,CASP7,CAT,GRIN2A,GRINA,PGF,PIK3C2A,PIK3CA,PIK3R1,PIK3R3,VEGFA,VEGFC,XIAP |
| Transcriptional Regulatory Network in Embryonic Stem Cells | 1,77E00 | GATA4,GATA6,H3-3A/H3-3B,H4-16,H4C8,HAND1,HNF4A,KAT6A,MEIS1,SKIL,ZFHX3 |
| Regulation of the Epithelial-Mesenchymal Transition Pathway | 1,77E00 | BRAF,EGFR,EGR1,FGF18,FGF19,FGF2,FGFR1,FGFR3,FRS2,FZD4,FZD5,GAB1,JAK2,LOX,MAP2K4,NFKB1,NFKB2,NOTCH1,PARD6B,PDGFD,PIK3C2A,PIK3CA,PIK3R1,PIK3R3,SMURF1,SNAI1,WNT10A,ZEB1,ZEB2 |
| Role of JAK2 in Hormone-like Cytokine Signaling | 1,76E00 | IRS2,JAK2,SH2B3,SOCS1,SOCS2,SOCS3,STAT1,STAT5A |
| Clathrin-mediated Endocytosis Signaling | 1,74E00 | APOE,APOL1,ARRB1,CD2AP,FGF18,FGF19,FGF2,HIP1R,ITGA5,ITGB8,LPA,LYZ,PCYOX1,PDGFA,PDGFD,PGF,PIK3C2A,PIK3CA,PIK3R1,PIK3R3,PPP3CC,RAB4A,STON2,SYNJ1,TFRC,UBD,USP9X,VEGFA,VEGFC |
| Macropinocytosis Signaling | 1,74E00 | ANKFY1,CSF1,CSF1R,ITGA5,ITGB8,PDGFA,PDGFD,PIK3C2A,PIK3CA,PIK3R1,PIK3R3,PLCG2,PRKCE,USP6NL |
| Wnt/Ca+ pathway | 1,72E00 | ATF4,CREB1,CREB5,CREBBP,FZD4,FZD5,NFAT5,NFATC2,NFKB1,NFKB2,PLCG2,PLCH1 |
| VEGF Family Ligand-Receptor Interactions | 1,71E00 | FOS,JMJD7-PLA2G4B,NOS3,PGF,PIK3C2A,PIK3CA,PIK3R1,PIK3R3,PLA2G4C,PLA2G4F,PLA2G6,PLCG2,PRKCE,VEGFA,VEGFC |
| Gα12/13 Signaling | 1,67E00 | BORCS8-MEF2B,GNA13,IKBKE,JUN,MAP2K4,MAP3K1,MEF2A,MEF2D,MYL6B,NFKB1,NFKB2,NFKBIA,NFKBIB,NFKBID,NFKBIE,PIK3C2A,PIK3CA,PIK3R1,PIK3R3,TEC,VAV3 |
| Xenobiotic Metabolism AHR Signaling Pathway | 1,67E00 | AHR,AHRR,ALDH7A1,CREBBP,CYP1A1,CYP1B1,EP300,IL1A,IL1B,IL6,MGST2,NFKB1,NFKB2,NRIP1,TNF |
| Thrombopoietin Signaling | 1,67E00 | FOS,IRS2,JAK2,JUN,PIK3C2A,PIK3CA,PIK3R1,PIK3R3,PLCG2,PRKCE,STAT1,STAT5A |
| Hematopoiesis from Pluripotent Stem Cells | 1,66E00 | CSF1,CSF2,CSF3,CXCL8,IL11,IL12A,IL1A,IL6,IL7,LIF |
| Superpathway of Cholesterol Biosynthesis | 1,66E00 | ACAA2,ACAT2,DHCR7,FDFT1,FDPS,PMVK,TM7SF2 |
| Endothelin-1 Signaling | 1,65E00 | BRAF,CASP1,CASP10,CASP7,CASP8,EDN1,FOS,GAB1,GNA13,HMOX1,JUN,MAPK6,NOS3,PIK3C2A,PIK3CA,PIK3R1,PIK3R3,PLA2G4C,PLA2G4F,PLA2G6,PLA2R1,PLAAT4,PLCG2,PLCH1,PNPLA3,PRKCE,PTGER2,PTGS2 |
| HIF1α Signaling | 1,64E00 | ADM,BRAF,CDKN1A,CREBBP,EDN1,EGLN3,EP300,FGF2,HMOX1,HSPA1A/HSPA1B,HSPA1L,HSPA6,IGF2,IL6,IL6R,JUN,MAP2K4,MMP7,PGF,PIK3C2A,PIK3CA,PIK3R1,PIK3R3,PLCG2,PPP3CC,PRKCE,SAT1,VEGFA,VEGFC,VIM |
| AMPK Signaling | 1,62E00 | ADRA2A,ADRB2,ARID1A,ARID2,ATF4,CDKN1A,CFTR,CREB1,CREB5,CREBBP,CRTC2,EP300,FOXO1,FOXO3,FOXO4,HNF4A,IRS2,NOS3,PIK3C2A,PIK3CA,PIK3R1,PIK3R3,PPARGC1A,PPM1D,PPM1K,PPP2R2A,PRKACB,PRKAG1,RPTOR,SIRT1,SMARCA2 |
| Sperm Motility | 1,6E00 | ABL2,CLK1,CLK2,CLK4,CSF1R,DYRK1A,EGFR,EPHA4,FGFR1,FGFR3,FLT3LG,FRK,JAK2,LYN,MAP2K4,PDE4B,PDE4D,PLA2G4C,PLA2G4F,PLA2G6,PLA2R1,PLAAT4,PLCG2,PLCH1,PNPLA3,PRKACB,PRKAG1,PRKCE,ROS1,STYK1,TEC,TXK |
| Glioma Signaling | 1,59E00 | CDKN1A,CDKN2B,E2F3,E2F6,E2F7,EGFR,IDH1,IDH2,IGF2,PDGFA,PDGFD,PIK3C2A,PIK3CA,PIK3R1,PIK3R3,PLCG2,PRKCE,RBL2 |
| PXR/RXR Activation | 1,57E00 | CYP2B6,CYP3A5,FOXO1,FOXO3,HNF4A,IL6,NR3C1,PPARGC1A,PRKACB,PRKAG1,SCD,TNF |
| Role of BRCA1 in DNA Damage Response | 1,56E00 | ARID1A,ARID2,BRIP1,CDKN1A,E2F3,E2F6,E2F7,FANCM,GADD45A,RBBP8,RBL2,SMARCA2,STAT1,UIMC1 |
| LPS/IL-1 Mediated Inhibition of RXR Function | 1,55E00 | ABCA1,ABCG1,ALDH7A1,APOE,CAT,CHST2,CRAT,CYP2B6,CYP3A5,HS2ST1,IL1A,IL1B,IL1RN,IL4I1,JUN,MAOB,MAP2K4,MAP3K1,MGST2,MYD88,NGFR,NR5A2,PPARGC1A,RARA,SLC27A3,SULT1C2,SULT4A1,TNF,TNFRSF1B,TRAF2,TRAF6,XPO1 |
| Complement System | 1,55E00 | C1S,C3,C4A/C4B,C5,CFB,CFH,CFI,ITGAM |
| Thyronamine and Iodothyronamine Metabolism | 1,55E00 | DIO2,DIO3 |
| Thyroid Hormone Metabolism I (via Deiodination) | 1,55E00 | DIO2,DIO3 |
| 14-3-3-mediated Signaling | 1,54E00 | CDKN1B,EDC3,FOS,FOXO1,JUN,MAP2K4,PIK3C2A,PIK3CA,PIK3R1,PIK3R3,PLCG2,PLCH1,PRKCE,SNCA,SRPK2,TNF,TRAF2,TUBA1A,TUBB6,VIM |
| Agranulocyte Adhesion and Diapedesis | 1,54E00 | C5,CCL2,CCL20,CCL22,CCL5,CLDN10,CLDN16,CX3CL1,CXCL1,CXCL10,CXCL11,CXCL2,CXCL3,CXCL8,CXCL9,HRH1,ICAM1,IL1A,IL1B,IL1RN,ITGA5,MMP7,MYH10,MYL6B,SDC4,SELE,TNF,VCAM1 |
| PTEN Signaling | 1,51E00 | BCAR1,BCL2L11,CDKN1A,CDKN1B,CNKSR3,EGFR,FGFR1,FGFR3,FOXO1,FOXO3,FOXO4,IKBKE,ITGA5,MAGI3,NFKB1,NFKB2,NGFR,PIK3CA,PIK3R1,PIK3R3,SYNJ1 |
| Neuregulin Signaling | 1,49E00 | AREG,BTC,CDK5,CDKN1B,CRKL,EGFR,ERBIN,EREG,ERRFI1,HBEGF,ITGA5,PIK3R1,PIK3R3,PLCG2,PRKCE,RNF41,STAT5A |
| Corticotropin Releasing Hormone Signaling | 1,48E00 | ATF4,BDNF,BORCS8-MEF2B,BRAF,CACNG4,CREB1,CREB5,CREBBP,CTH,FOS,JUN,JUND,MEF2A,MEF2D,NOS3,NR4A1,PLCG2,PRKACB,PRKAG1,PRKCE,PTGS2,VEGFA |
| Bupropion Degradation | 1,47E00 | CYP1A1,CYP1B1,CYP2B6,CYP2J2,CYP3A5,CYP4B1 |
| Role of IL-17A in Psoriasis | 1,46E00 | CCL20,CXCL1,CXCL3,CXCL8 |
| IL-15 Signaling | 1,45E00 | CSF2,CXCL8,IL15,IL15RA,JAK2,NFKB1,NFKB2,PIK3C2A,PIK3CA,PIK3R1,PIK3R3,RPTOR,STAT5A |
| Granzyme A Signaling | 1,45E00 | CREBBP,EP300,H1-0,H1-3,H1-4 |
| Salvage Pathways of Pyrimidine Deoxyribonucleotides | 1,41E00 | APOBEC3G,CDA,TYMP |
| GDNF Family Ligand-Receptor Interactions | 1,41E00 | CREB1,DOK4,FOS,FRS2,GAB1,IRS2,JUN,MAP2K4,PIK3C2A,PIK3CA,PIK3R1,PIK3R3,PLCG2 |
| Apelin Liver Signaling Pathway | 1,39E00 | COL11A2,COL1A1,EDN1,FAS,MAP2K4,TNF |
| Ephrin A Signaling | 1,38E00 | BCAR1,EFNA1,EPHA4,NGFR,PIK3C2A,PIK3CA,PIK3R1,PIK3R3,VAV3 |
| Superpathway of Inositol Phosphate Compounds | 1,37E00 | ATP1A1,DUSP1,DUSP10,DUSP15,DUSP16,DUSP2,DUSP5,DUSP8,IPPK,ITPKC,MTMR9,NOCT,NUDT1,PI4K2B,PIK3C2A,PIK3CA,PIK3R1,PIK3R3,PIP5K1A,PLCG2,PLCH1,PPFIA1,PPM1K,PPP1R12A,PPP1R7,PPTC7,SOCS3,SYNJ1 |
| IL-4 Signaling | 1,36E00 | HLA-A,HLA-B,HLA-DOB,HLA-DQA1,JAK2,NFAT5,NFATC2,NR3C1,PIK3C2A,PIK3CA,PIK3R1,PIK3R3,SOCS1,SYNJ1 |
| BMP signaling pathway | 1,36E00 | BMP2,CREB1,CREBBP,FST,JUN,MAP2K4,NFKB1,NFKB2,PRKACB,PRKAG1,SMAD7,SMURF1,SOSTDC1,XIAP |
| 3-phosphoinositide Biosynthesis | 1,36E00 | ATP1A1,DUSP1,DUSP10,DUSP15,DUSP16,DUSP2,DUSP5,DUSP8,MTMR9,NOCT,NUDT1,PI4K2B,PIK3C2A,PIK3CA,PIK3R1,PIK3R3,PIP5K1A,PPFIA1,PPM1K,PPP1R12A,PPP1R7,PPTC7,SOCS3,SYNJ1 |
| Gαq Signaling | 1,35E00 | DIRAS3,GNB4,GNG12,HMOX1,HRH1,IKBKE,NFATC2,NFKB1,NFKB2,NFKBIA,NFKBIB,NFKBID,NFKBIE,PIK3C2A,PIK3CA,PIK3R1,PIK3R3,PLCG2,PPP3CC,PRKCE,RHOV,RND1,RND3 |
| EGF Signaling | 1,34E00 | EGFR,FOS,JUN,MAP2K4,MAP3K1,PIK3C2A,PIK3CA,PIK3R1,PIK3R3,STAT1 |
| ErbB Signaling | 1,32E00 | AREG,BTC,EGFR,EREG,FOS,FOXO1,HBEGF,JUN,MAP2K4,PIK3C2A,PIK3CA,PIK3R1,PIK3R3,PLCG2,PRKCE |
| Estrogen Biosynthesis | 1,31E00 | CYP1A1,CYP1B1,CYP2B6,CYP2J2,CYP3A5,CYP4B1,HSD17B14,HSD17B2 |
| Human Embryonic Stem Cell Pluripotency | 1,3E00 | ACVR1,BDNF,BMP2,FGF2,FGFR1,FGFR3,FOXO1,FZD4,FZD5,INHBA,PDGFA,PDGFD,PIK3C2A,PIK3CA,PIK3R1,PIK3R3,S1PR2,SMAD7,SPHK1,WNT10A |
| P2Y Purigenic Receptor Signaling Pathway | 1,3E00 | ATF4,CREB1,CREB5,CREBBP,FOS,GNB4,GNG12,JUN,NFKB1,NFKB2,PIK3C2A,PIK3CA,PIK3R1,PIK3R3,PLCG2,PLCH1,PRKACB,PRKAG1,PRKCE |

**Supplementary Table S11. List of Canonical Pathways for the condition "Caco-2_SARS-CoV-2 ".** The IPA-based Core Analysis was performed on the basis of differentially expressed genes dissected for a given condition by GENEVESTIGATOR-based analysis. Only statistically significant Canonical Pathways are included (n = 147). The data are taken as the basis for R-based visualization by Venn diagrams shown in Figure 5.

| Ingenuity Canonical Pathways | -log(p-value) | Molecules |
|---|---|---|
| TNFR2 Signaling | 6,23E00 | BIRC3,FOS,JUN,NFKBIA,TNFAIP3 |
| MSP-RON Signaling In Cancer Cells Pathway | 6,11E00 | CCND1,ETS1,FOS,ITGA6,JUN,MYC,NFKBIA,VIM |
| Aryl Hydrocarbon Receptor Signaling | 5,89E00 | ALDH1A1,CCND1,CYP1A1,FOS,GSTA2,JUN,MDM2,MYC |
| Acute Phase Response Signaling | 5,15E00 | AGT,AHSG,FGG,FOS,JUN,NFKBIA,SERPINE1,TTR |
| Hepatic Fibrosis Signaling Pathway | 5,12E00 | AGT,CCND1,FOS,ICAM1,ITGA2,ITGA6,JUN,MYC,NFKBIA,RHOB,SERPINE1 |
| TNFR1 Signaling | 5,09E00 | BIRC3,FOS,JUN,NFKBIA,TNFAIP3 |
| Glucocorticoid Receptor Signaling | 5,02E00 | AGT,DUSP1,FGG,FOS,ICAM1,JUN,KRT6A,MYC,NFKBIA,PLAU,POLR2J2/POLR2J3,SERPINE1 |
| ERK/MAPK Signaling | 4,79E00 | DUSP1,DUSP6,ETS1,FOS,H3C14,ITGA2,ITGA6,MYC |
| Sumoylation Pathway | 4,64E00 | ETS1,FOS,JUN,MDM2,NFKBIA,RHOB |
| CD40 Signaling | 4,53E00 | FOS,ICAM1,JUN,NFKBIA,TNFAIP3 |
| IL-17A Signaling in Fibroblasts | 4,4E00 | FOS,JUN,NFKBIA,NFKBIZ |
| HGF Signaling | 4,27E00 | CCND1,ETS1,FOS,ITGA2,ITGA6,JUN |
| Molecular Mechanisms of Cancer | 4,13E00 | BIRC3,CCND1,FOS,ITGA2,ITGA6,JUN,MDM2,MYC,NFKBIA,RHOB |
| Coronavirus Pathogenesis Pathway | 3,74E00 | AGT,CCND1,FOS,JUN,NFKBIA,SERPINE1 |
| p53 Signaling | 3,68E00 | CCND1,HDAC9,JUN,MDM2,TNFRSF10B |
| HOTAIR Regulatory Pathway | 3,58E00 | H3C14,ICAM1,MDM2,MYC,NFKBIA,VIM |
| Erythropoietin Signaling | 3,4E00 | BIRC3,CCND1,FOS,JUN,MDM2,NFKBIA |
| Tumor Microenvironment Pathway | 3,36E00 | CCND1,FOS,ICAM1,JUN,MYC,PLAU |
| Cell Cycle: G1/S Checkpoint Regulation | 3,29E00 | CCND1,HDAC9,MDM2,MYC |
| Hepatic Fibrosis / Hepatic Stellate Cell Activation | 3,24E00 | AGT,ICAM1,IGFBP4,KLF6,LAMA1,SERPINE1 |
| Regulation Of The Epithelial Mesenchymal Transition By Growth Factors Pathway | 3,21E00 | EGR1,ETS1,FOS,JUN,PARD6B,VIM |
| ILK Signaling | 3,19E00 | CCND1,FOS,JUN,MYC,RHOB,VIM |
| Circadian Rhythm Signaling | 3,11E00 | BHLHE40,NR1D1,PER1 |
| Toll-like Receptor Signaling | 3,08E00 | FOS,JUN,NFKBIA,TNFAIP3 |
| Coagulation System | 3,03E00 | FGG,PLAU,SERPINE1 |
| Thyroid Cancer Signaling | 3,02E00 | CCND1,FOS,JUN,MYC |
| PI3K Signaling in B Lymphocytes | 3 | ATF3,BCL10,FOS,JUN,NFKBIA |
| PDGF Signaling | 2,88E00 | ABL2,FOS,JUN,MYC |
| April Mediated Signaling | 2,86E00 | FOS,JUN,NFKBIA |
| RANK Signaling in Osteoclasts | 2,83E00 | BIRC3,FOS,JUN,NFKBIA |
| B Cell Activating Factor Signaling | 2,83E00 | FOS,JUN,NFKBIA |
| Regulation of IL-2 Expression in Activated and Anergic T Lymphocytes | 2,81E00 | BCL10,FOS,JUN,NFKBIA |
| MIF Regulation of Innate Immunity | 2,8E00 | FOS,JUN,NFKBIA |
| Death Receptor Signaling | 2,77E00 | BIRC3,NFKBIA,TIPARP,TNFRSF10B |
| Necroptosis Signaling Pathway | 2,75E00 | AXL,BIRC3,CYLD,MDM2,TNFRSF10B |
| iNOS Signaling | 2,71E00 | FOS,JUN,NFKBIA |
| ATM Signaling | 2,69E00 | JUN,MDM2,NFKBIA,TRRAP |
| HMGB1 Signaling | 2,66E00 | FOS,ICAM1,JUN,RHOB,SERPINE1 |
| Chronic Myeloid Leukemia Signaling | 2,6E00 | CCND1,HDAC9,MDM2,MYC |
| Tec Kinase Signaling | 2,57E00 | FOS,ITGA2,ITGA6,RHOB,TNFRSF10B |
| Neuregulin Signaling | 2,57E00 | ERRFI1,ITGA2,ITGA6,MYC |
| Signaling by Rho Family GTPases | 2,55E00 | FOS,ITGA2,ITGA6,JUN,RHOB,VIM |
| T Cell Receptor Signaling | 2,55E00 | BCL10,FOS,JUN,NFKBIA |
| UVB-Induced MAPK Signaling | 2,53E00 | FOS,H3C14,JUN |
| CDK5 Signaling | 2,52E00 | EGR1,ITGA2,ITGA6,LAMA1 |
| CD27 Signaling in Lymphocytes | 2,51E00 | FOS,JUN,NFKBIA |
| PI3K/AKT Signaling | 2,44E00 | CCND1,ITGA2,ITGA6,MDM2,NFKBIA |
| B Cell Receptor Signaling | 2,43E00 | BCL10,EGR1,ETS1,JUN,NFKBIA |
| NRF2-mediated Oxidative Stress Response | 2,4E00 | FOS,GSTA2,JUN,JUNB,MAFF |
| Role of PKR in Interferon Induction and Antiviral Response | 2,38E00 | ATF3,FOS,JUN,NFKBIA |
| Senescence Pathway | 2,38E00 | ATF3,CCND1,ETS1,JUN,MDM2,SERPINE1 |
| RAR Activation | 2,35E00 | AKR1C3,ALDH1A1,DUSP1,FOS,JUN |
| LXR/RXR Activation | 2,35E00 | AGT,AHSG,LDLR,TTR |
| IL-15 Production | 2,35E00 | ABL2,AXL,DYRK1A,EPHA2 |
| CD28 Signaling in T Helper Cells | 2,35E00 | BCL10,FOS,JUN,NFKBIA |
| IL-8 Signaling | 2,3E00 | CCND1,FOS,ICAM1,JUN,RHOB |
| Thrombopoietin Signaling | 2,29E00 | FOS,JUN,MYC |
| ErbB2-ErbB3 Signaling | 2,26E00 | CCND1,JUN,MYC |
| Polyamine Regulation in Colon Cancer | 2,19E00 | MXD1,MYC |
| IL-10 Signaling | 2,17E00 | FOS,JUN,NFKBIA |
| Small Cell Lung Cancer Signaling | 2,15E00 | CCND1,MYC,NFKBIA |
| Role of Macrophages, Fibroblasts and Endothelial Cells in Rheumatoid Arthritis | 2,11E00 | CCND1,FOS,ICAM1,JUN,MYC,NFKBIA |
| Estrogen-Dependent Breast Cancer Signaling | 2,1E00 | CCND1,FOS,JUN |
| Regulation of Cellular Mechanics by Calpain Protease | 2,1E00 | CCND1,ITGA2,ITGA6 |
| Hypoxia Signaling in the Cardiovascular System | 2,1E00 | JUN,MDM2,NFKBIA |
| Endocannabinoid Cancer Inhibition Pathway | 2,09E00 | ATF3,CCND1,MYC,VIM |
| IL-17A Signaling in Gastric Cells | 2,08E00 | FOS,JUN |
| NF-κB Activation by Viruses | 2,07E00 | ITGA2,ITGA6,NFKBIA |
| Estrogen-mediated S-phase Entry | 2,05E00 | CCND1,MYC |
| IL-7 Signaling Pathway | 2,04E00 | CCND1,JUN,MYC |
| BEX2 Signaling Pathway | 2,02E00 | CCND1,JUN,NFKBIA |
| Estrogen Receptor Signaling | 2,02E00 | AGT,CCND1,FOS,JUN,MYC,TRRAP |
| Renal Cell Carcinoma Signaling | 2,01E00 | ETS1,FOS,JUN |
| Prolactin Signaling | 2 | FOS,JUN,MYC |
| Actin Nucleation by ARP-WASP Complex | 2 | ITGA2,ITGA6,RHOB |
| PKCθ Signaling in T Lymphocytes | 1,97E00 | BCL10,FOS,JUN,NFKBIA |
| LPS-stimulated MAPK Signaling | 1,97E00 | FOS,JUN,NFKBIA |
| TR/RXR Activation | 1,95E00 | AKR1C3,LDLR,MDM2 |
| HER-2 Signaling in Breast Cancer | 1,95E00 | CCND1,MDM2,PARD6B |
| Xenobiotic Metabolism AHR Signaling Pathway | 1,94E00 | ALDH1A1,CYP1A1,GSTA2 |
| Colorectal Cancer Metastasis Signaling | 1,88E00 | CCND1,FOS,JUN,MYC,RHOB |
| Glioblastoma Multiforme Signaling | 1,88E00 | CCND1,MDM2,MYC,RHOB |
| 4-1BB Signaling in T Lymphocytes | 1,87E00 | JUN,NFKBIA |
| CXCR4 Signaling | 1,86E00 | EGR1,FOS,JUN,RHOB |
| Prostate Cancer Signaling | 1,86E00 | CCND1,MDM2,NFKBIA |
| IL-1 Signaling | 1,84E00 | FOS,JUN,NFKBIA |
| Retinoate Biosynthesis I | 1,82E00 | AKR1C3,ALDH1A1 |
| Wnt/β-catenin Signaling | 1,81E00 | CCND1,JUN,MDM2,MYC |
| TWEAK Signaling | 1,8E00 | BIRC3,NFKBIA |
| TGF-β Signaling | 1,8E00 | FOS,JUN,SERPINE1 |
| Cdc42 Signaling | 1,79E00 | FOS,ITGA2,ITGA6,JUN |

| | | |
|---|---|---|
| Bladder Cancer Signaling | 1,78E00 | CCND1,MDM2,MYC |
| UVA-Induced MAPK Signaling | 1,77E00 | FOS,JUN,TIPARP |
| Systemic Lupus Erythematosus In B Cell Signaling Pathway | 1,74E00 | BCL10,CCND1,FOS,JUN,MYC |
| Regulation of Actin-based Motility by Rho | 1,71E00 | ITGA2,ITGA6,RHOB |
| IGF-1 Signaling | 1,7E00 | FOS,IGFBP4,JUN |
| PPAR Signaling | 1,69E00 | FOS,JUN,NFKBIA |
| Production of Nitric Oxide and Reactive Oxygen Species in Macrophages | 1,69E00 | FOS,JUN,NFKBIA,RHOB |
| Telomerase Signaling | 1,67E00 | ETS1,HDAC9,MYC |
| Estrogen Biosynthesis | 1,67E00 | AKR1C3,CYP1A1 |
| Agranulocyte Adhesion and Diapedesis | 1,66E00 | CXCL2,ICAM1,ITGA2,ITGA6 |
| Arginine Degradation I (Arginase Pathway) | 1,66E00 | OAT |
| Sirtuin Signaling Pathway | 1,65E00 | DUSP6,H3C14,JUN,MYC,NDRG1 |
| Protein Kinase A Signaling | 1,64E00 | AKAP12,DUSP1,DUSP6,H3C14,NFKBIA,UBASH3B |
| BAG2 Signaling Pathway | 1,63E00 | MDM2,MYC |
| MSP-RON Signaling In Macrophages Pathway | 1,61E00 | FOS,JUN,NFKBIZ |
| Neuroinflammation Signaling Pathway | 1,6E00 | BIRC3,FOS,GJB1,ICAM1,JUN |
| Apelin Endothelial Signaling Pathway | 1,59E00 | FOS,ICAM1,JUN |
| Role of Tissue Factor in Cancer | 1,58E00 | EGR1,FGG,ITGA6 |
| HIF1α Signaling | 1,57E00 | JUN,MDM2,SERPINE1,VIM |
| Proline Biosynthesis II (from Arginine) | 1,57E00 | OAT |
| Renin-Angiotensin Signaling | 1,56E00 | AGT,FOS,JUN |
| p38 MAPK Signaling | 1,56E00 | DUSP1,H3C14,MYC |
| Cholecystokinin/Gastrin-mediated Signaling | 1,55E00 | FOS,JUN,RHOB |
| Rac Signaling | 1,53E00 | ITGA2,ITGA6,JUN |
| Melanoma Signaling | 1,51E00 | CCND1,MDM2 |
| Myc Mediated Apoptosis Signaling | 1,51E00 | MDM2,MYC |
| UVC-Induced MAPK Signaling | 1,49E00 | FOS,JUN |
| FXR/RXR Activation | 1,49E00 | AGT,AHSG,TTR |
| IL-6 Signaling | 1,49E00 | FOS,JUN,NFKBIA |
| Role of Osteoblasts, Osteoclasts and Chondrocytes in Rheumatoid Arthritis | 1,49E00 | BIRC3,FOS,JUN,NFKBIA |
| Arginine Biosynthesis IV | 1,49E00 | OAT |
| Arginine Degradation VI (Arginase 2 Pathway) | 1,49E00 | OAT |
| Selenocysteine Biosynthesis II (Archaea and Eukaryotes) | 1,49E00 | SEPHS2 |
| GDP-mannose Biosynthesis | 1,49E00 | PMM1 |
| 14-3-3-mediated Signaling | 1,48E00 | FOS,JUN,VIM |
| P2Y Purigenic Receptor Signaling Pathway | 1,48E00 | FOS,JUN,MYC |
| Sperm Motility | 1,46E00 | ABL2,AXL,DYRK1A,EPHA2 |
| EIF2 Signaling | 1,45E00 | ATF3,CCND1,MYC,PPP1R15A |
| LPS/IL-1 Mediated Inhibition of RXR Function | 1,44E00 | ALDH1A1,FABP1,GSTA2,JUN |
| EGF Signaling | 1,43E00 | FOS,JUN |
| Phagosome Formation | 1,43E00 | ITGA2,ITGA6,RHOB |
| cAMP-mediated signaling | 1,43E00 | AKAP12,DUSP1,DUSP6,RGS2 |
| Androgen Signaling | 1,41E00 | CCND1,JUN,POLR2J2/POLR2J3 |
| PTEN Signaling | 1,41E00 | CCND1,ITGA2,ITGA6 |
| Hereditary Breast Cancer Signaling | 1,37E00 | CCND1,HDAC9,POLR2J2/POLR2J3 |
| Endometrial Cancer Signaling | 1,36E00 | CCND1,MYC |
| Retinoic acid Mediated Apoptosis Signaling | 1,36E00 | TIPARP,TNFRSF10B |
| PCP pathway | 1,36E00 | JUN,JUNB |
| D-myo-inositol (1,4,5,6)-Tetrakisphosphate Biosynthesis | 1,36E00 | DUSP1,EPHX2,PALD1 |
| D-myo-inositol (3,4,5,6)-tetrakisphosphate Biosynthesis | 1,36E00 | DUSP1,EPHX2,PALD1 |
| Induction of Apoptosis by HIV1 | 1,35E00 | BIRC3,NFKBIA |
| IL-2 Signaling | 1,35E00 | FOS,JUN |
| Activation of IRF by Cytosolic Pattern Recognition Receptors | 1,33E00 | JUN,NFKBIA |
| Citrulline Biosynthesis | 1,32E00 | OAT |
| PXR/RXR Activation | 1,3E00 | ALDH1A1,GSTA2 |
| Relaxin Signaling | 1,3E00 | FOS,JUN,NFKBIA |

**Supplementary Table S12. List of Canonical Pathways for the condition "Caco-2_SARS-CoV".** The IPA-based Core Analysis was performed on the basis of differentially expressed genes dissected for a given condition by GENEVESTIGATOR-based analysis. Only statistically significant Canonical Pathway are included (n = 177). The data are taken as the basis for R-based visualization by Venn diagrams shown in Figure 5.

| **Ingenuity Canonical Pathways** | **-log(p-value)** | **Molecules** |
|---|---|---|
| Molecular Mechanisms of Cancer | 7,46E00 | BCL2,BIRC3,CASP6,CCND1,CDK18,CDK19,CDKN2C,CFLAR,FOS,FZD4,GAB1,GNAI1,IHH,ITGA2,ITGA3,ITGA5,ITGA6,LRP1,MAP2K6,MDM2,MYC,NFKB2,NFKBIE,PAK1,PAK6,PIK3C2B,RASGRF2,RHOB,RHOBTB2,RND1,RND3,SRC,TGFB3 |
| Tec Kinase Signaling | 6,39E00 | FOS,GNAI1,GNG2,ITGA2,ITGA3,ITGA5,ITGA6,NFKB2,PAK1,PAK6,PIK3C2B,RHOB,RHOBTB2,RND1,RND3,SRC,TNFRSF10A,TNFRSF10B,VAV3 |
| ERK/MAPK Signaling | 5,97E00 | DUSP1,DUSP2,DUSP4,DUSP6,DUSP9,ELK3,ETS1,FOS,ITGA2,ITGA3,ITGA5,ITGA6,MYC,MYCN,PAK1,PAK6,PIK3C2B,PLA2G12B,PPP2R2B,SRC |
| Tumor Microenvironment Pathway | 5,63E00 | BCL2,CCND1,CFLAR,CXCL8,CXCR4,FOS,ICAM1,IGF2,ITGA5,MMP1,MMP11,MYC,NFKB2,PDGFB,PIK3C2B,PTGS2,SLC2A1,TGFB3 |
| Aryl Hydrocarbon Receptor Signaling | 5,58E00 | ALDH1A1,ALDH3B2,ALDH6A1,CCND1,CYP1A1,FOS,GSTA2,GSTA4,GSTK1,MDM2,MYC,NFKB2,RXRA,SRC,TGFB3,TGM2 |
| Hepatic Fibrosis Signaling Pathway | 5,54E00 | BCL2,CCND1,CXCL8,EDN1,FGFR1,FOS,FZD4,GNAI1,ICAM1,IL18,ITGA2,ITGA3,ITGA5,ITGA6,MAP2K6,MMP1,MYC,NFKB2,NFKBIE,PDCD4,PDGFB,PIK3C2B,RHOB,RHOBTB2,RND1,RND3,SERPINE1,TGFB3 |
| HMGB1 Signaling | 4,78E00 | CLCF1,CXCL8,FOS,ICAM1,IL11,IL18,LIF,MAP2K6,NFKB2,PIK3C2B,RHOB,RHOBTB2,RND1,RND3,SERPINE1,TGFB3 |
| Role of Tissue Factor in Cancer | 4,66E00 | ARRB1,CXCL8,EGR1,F7,FGA,FGG,ITGA3,ITGA6,MMP1,PAK1,PIK3C2B,PLAUR,SRC |
| Germ Cell-Sertoli Cell Junction Signaling | 4,59E00 | EPN3,ITGA2,ITGA3,ITGA6,MAP2K6,MYO7A,PAK1,PAK6,PIK3C2B,RHOB,RHOBTB2,RND1,RND3,SRC,TGFB3,TUBB3 |
| Coagulation System | 4,36E00 | F7,FGA,FGG,PLAUR,SERPINA5,SERPINE1,SERPINF2 |
| PI3K/AKT Signaling | 4,17E00 | BCL2,CCND1,GAB1,IL17RE,IL22RA1,ITGA2,ITGA3,ITGA5,ITGA6,MCL1,MDM2,NFKB2,NFKBIE,PPP2R2B,PTGS2,SFN |
| TNFR1 Signaling | 4,17E00 | BIRC3,CASP6,FOS,NFKB2,NFKBIE,PAK1,PAK6,TNFAIP3 |
| Hepatic Fibrosis / Hepatic Stellate Cell Activation | 4,15E00 | BCL2,COL4A1,CXCL8,EDN1,FGFR1,FGFR2,ICAM1,IGF2,IGFBP4,KLF6,LAMA1,MMP1,NFKB2,PDGFB,SERPINE1,TGFB3 |
| Regulation Of The Epithelial Mesenchymal Transition By Growth Factors Pathway | 4,09E00 | CLDN3,EGR1,ETS1,FGFR1,FGFR2,FOS,GAB1,LATS1,MAP2K6,MMP1,NFKB2,PARD6B,PDGFB,PIK3C2B,TGFB3,VIM |
| p53 Signaling | 4,04E00 | BCL2,CASP6,CCND1,GADD45A,GADD45B,HDAC9,MDM2,PIK3C2B,SFN,TNFRSF10A,TNFRSF10B |
| MSP-RON Signaling In Cancer Cells Pathway | 4 | CCND1,ELK3,ETS1,FOS,ITGA6,MST1R,MYC,NFKB2,PIK3C2B,PLAUR,SFN,SRC,VIM |
| Erythropoietin Signaling | 3,96E00 | BIRC3,CCND1,CLCF1,CXCL8,EPO,FOS,IL11,IL18,LIF,MDM2,NFKB2,NFKBIE,PIK3C2B,SRC,TGFB3 |
| TR/RXR Activation | 3,94E00 | AKR1C3,DIO1,FGA,LDLR,MDM2,PIK3C2B,RXRA,SLC16A3,SLC2A1,THRA |
| HGF Signaling | 3,86E00 | CCND1,ELK3,ETS1,FOS,GAB1,ITGA2,ITGA3,ITGA5,ITGA6,PAK1,PIK3C2B,PTGS2 |
| Death Receptor Signaling | 3,61E00 | BCL2,BIRC3,CASP6,CFLAR,GAS2,NFKB2,NFKBIE,TIPARP,TNFRSF10A,TNFRSF10B |
| Xenobiotic Metabolism CAR Signaling Pathway | 3,54E00 | ALDH1A1,ALDH3B2,ALDH6A1,CHST13,CYP1A1,GSTA2,GSTA4,GSTK1,MAP2K6,PPP2R2B,RXRA,SRC,SULT1C2,SULT1C4,SULT1E1 |
| Ephrin Receptor Signaling | 3,54E00 | CXCR4,EFNA1,EFNB2,EPHA2,GNAI1,GNG2,ITGA2,ITGA3,ITGA5,ITGA6,PAK1,PAK6,PDGFB,SDC2,SRC |
| Colorectal Cancer Metastasis Signaling | 3,54E00 | ARRB1,CCND1,FOS,FZD4,GNG2,LRP1,MMP1,MMP11,MYC,NFKB2,PIK3C2B,PTGS2,RHOB,RHOBTB2,RND1,RND3,SRC,TGFB3 |
| Cardiac Hypertrophy Signaling (Enhanced) | 3,42E00 | CLCF1,CXCL8,EDN1,FGFR1,FGFR2,FZD4,GATA4,GNAI1,GNG2,HDAC9,IL11,IL17RE,IL18,IL22RA1,ITGA2,ITGA3,ITGA5,ITGA6,LIF,MAP2K6,MYC,NFAT5,NFKB2,PDE6A,PIK3C2B,PLD6,PTGS2,TGFB3 |
| CD40 Signaling | 3,36E00 | FOS,ICAM1,MAP2K6,NFKB2,NFKBIE,PIK3C2B,PTGS2,TNFAIP3 |
| Osteoarthritis Pathway | 3,31E00 | CASP6,CXCL8,FGFR1,FZD4,GREM1,IHH,ITGA2,ITGA3,ITGA5,ITGA6,LRP1,MATN3,MMP1,MTF1,NFKB2,PTGS2 |
| IL-8 Signaling | 3,28E00 | BCL2,CCND1,CXCL8,FOS,GNAI1,GNG2,ICAM1,PIK3C2B,PLD6,PTGS2,RHOB,RHOBTB2,RND1,RND3,SRC |
| Regulation of Actin-based Motility by Rho | 3,21E00 | ITGA2,ITGA3,ITGA5,ITGA6,PAK1,PAK6,RHOB,RHOBTB2,RND1,RND3 |
| Role of IL-17A in Arthritis | 3,14E00 | CXCL8,MAP2K6,MMP1,NFKB2,NFKBIE,PIK3C2B,PTGS2 |
| Senescence Pathway | 3,11E00 | ATF3,CCND1,CGAS,CXCL8,ELK3,ETS1,GADD45A,GADD45B,GATA4,MAP2K6,MDM2,NFAT5,NFKB2,PIK3C2B,PPP2R2B,SERPINE1,TGFB3,ZFP36L1 |
| Small Cell Lung Cancer Signaling | 3,1E00 | BCL2,CCND1,MYC,NFKB2,NFKBIE,PIK3C2B,PTGS2,RXRA |
| Signaling by Rho Family GTPases | 3,09E00 | CDC42EP1,FOS,GNAI1,GNG2,ITGA2,ITGA3,ITGA5,ITGA6,NFKB2,PAK1,PAK6,PIK3C2B,RHOB,RHOBTB2,RND1,RND3,VIM |
| CXCR4 Signaling | 3,07E00 | CXCR4,EGR1,FOS,GNAI1,GNG2,PAK1,PAK6,PIK3C2B,RHOB,RHOBTB2,RND1,RND3,SRC |
| Atherosclerosis Signaling | 3,05E00 | ALOXE3,APOL1,CXCL8,CXCR4,ICAM1,IL18,MMP1,NFKB2,PDGFB,PLA2G12B,TNFRSF12A |
| ILK Signaling | 3,02E00 | CCND1,FERMT2,FOS,MAP2K6,MYC,NFKB2,PIK3C2B,PPP2R2B,PTGS2,RHOB,RHOBTB2,RND1,RND3,VIM |
| Agranulocyte Adhesion and Diapedesis | 2,98E00 | CLDN3,CXCL2,CXCL8,CXCR4,GNAI1,HRH1,ICAM1,IL18,ITGA2,ITGA3,ITGA5,ITGA6,MMP1,MMP11 |
| TNFR2 Signaling | 2,87E00 | BIRC3,FOS,NFKB2,NFKBIE,TNFAIP3 |
| Semaphorin Signaling in Neurons | 2,86E00 | DPYSL3,PAK1,PAK6,RHOB,RHOBTB2,RND1,RND3 |
| Axonal Guidance Signaling | 2,79E00 | CXCR4,EFNA1,EFNB2,EPHA2,FZD4,GNAI1,GNG2,ITGA2,ITGA3,ITGA5,ITGA6,L1CAM,MMP1,MMP11,MYSM1,NFAT5,NTN4,PAK1,PAK6,PDGFB,PIK3C2B,PLXNC1,RND1,SDC2,SEMA4G,TUBB3 |
| LPS/IL-1 Mediated Inhibition of RXR Function | 2,76E00 | ACSL1,ACSL5,ALDH1A1,ALDH3B2,ALDH6A1,CHST13,GSTA2,GSTA4,GSTK1,HMGCS2,IL18,RXRA,SULT1C2,SULT1C4,SULT1E1 |
| Renal Cell Carcinoma Signaling | 2,75E00 | ETS1,FOS,GAB1,PAK1,PAK6,PDGFB,PIK3C2B,SLC2A1 |
| Ethanol Degradation II | 2,74E00 | ACSL1,ACSS1,ADH4,ADH6,ALDH1A1 |
| Actin Nucleation by ARP-WASP Complex | 2,72E00 | ITGA2,ITGA3,ITGA5,ITGA6,RHOB,RHOBTB2,RND1,RND3 |
| HIF1α Signaling | 2,71E00 | EDN1,EPO,HK2,HSPA1L,IGF2,MAP2K6,MDM2,MMP1,MMP11,PDGFB,PIK3C2B,SERPINE1,SLC2A1,VIM |
| Systemic Lupus Erythematosus In B Cell Signaling Pathway | 2,7E00 | BCL10,BCL2,CCND1,CLCF1,CXCL8,FOS,GAB1,IL11,IL18,LIF,MCL1,MYC,NFAT5,NFKB2,PIK3C2B,SRC,TGFB3 |
| IL-15 Production | 2,67E00 | ABL2,EPHA2,ERBB3,FGFR1,FGFR2,IRF1,MAP2K6,MST1R,NFKB2,SRC |
| Glioblastoma Multiforme Signaling | 2,62E00 | CCND1,FZD4,IGF2,MDM2,MYC,PDGFB,PIK3C2B,RHOB,RHOBTB2,RND1,RND3,SRC |
| Sumoylation Pathway | 2,62E00 | CEBPA,ETS1,FOS,MDM2,NFKB2,RHOB,RHOBTB2,RND1,RND3 |
| Xenobiotic Metabolism AHR Signaling Pathway | 2,59E00 | ALDH1A1,ALDH3B2,ALDH6A1,CYP1A1,GSTA2,GSTA4,GSTK1,NFKB2 |
| Myc Mediated Apoptosis Signaling | 2,59E00 | BCL2,CFLAR,MCL1,MDM2,MYC,NFKB2 |
| RhoGDI Signaling | 2,58E00 | GNAI1,GNG2,ITGA2,ITGA3,ITGA5,ITGA6,PAK1,PAK6,RHOB,RHOBTB2,RND1,RND3,SRC |
| TWEAK Signaling | 2,57E00 | BIRC3,CASP6,NFKB2,NFKBIE,TNFRSF12A |
| IL-17A Signaling in Fibroblasts | 2,57E00 | FOS,MMP1,NFKB2,NFKBIE,NFKBIZ |
| Neuregulin Signaling | 2,56E00 | AREG,ERBB3,ERRFI1,ITGA2,ITGA3,ITGA5,ITGA6,MYC,SRC |
| Integrin Signaling | 2,56E00 | ITGA2,ITGA3,ITGA5,ITGA6,ITGA7,PAK1,PAK6,PDGFB,PIK3C2B,RHOB,RHOBTB2,RND1,RND3,SRC |
| Regulation of the Epithelial-Mesenchymal Transition Pathway | 2,52E00 | CLDN3,EGR1,ETS1,FGFR1,FGFR2,FZD4,GAB1,MAP2K6,NFKB2,NOTCH3,PARD6B,PIK3C2B,TGFB3 |
| Paxillin Signaling | 2,48E00 | ITGA2,ITGA3,ITGA5,ITGA6,ITGA7,PAK1,PAK6,PIK3C2B,SRC |
| Role of Osteoblasts, Osteoclasts and Chondrocytes in Rheumatoid Arthritis | 2,47E00 | BCL2,BIRC3,FOS,FZD4,IL11,IL18,ITGA5,LRP1,MAP2K6,MMP1,NFAT5,NFKBIE,PIK3C2B,SRC |
| Acute Myeloid Leukemia Signaling | 2,46E00 | CCND1,CEBPA,IDH1,MAP2K6,MYC,NFKB2,PIK3C2B,RUNX1 |
| Phospholipase C Signaling | 2,46E00 | GNG2,HDAC9,ITGA2,ITGA3,ITGA5,ITGA6,NFAT5,NFKB2,PLA2G12B,PLD6,RHOB,RHOBTB2,RND1,RND3,SRC,TGM2 |
| ERK5 Signaling | 2,4E00 | FOS,FOSL1,GAB1,LIF,MYC,SFN,SRC |
| Caveolar-mediated Endocytosis Signaling | 2,37E00 | ITGA2,ITGA3,ITGA5,ITGA6,ITGA7,RAB5B,SRC |
| Protein Kinase A Signaling | 2,36E00 | ADD3,AKAP12,CDC14B,DUSP1,DUSP2,DUSP4,DUSP5,DUSP6,DUSP9,GNAI1,GNG2,IHH,NFAT5,NFKB2,NFKBIE,PDE6A,PLD6,PTGS2,SFN,TGFB3,UBASH3B |
| Adipogenesis pathway | 2,35E00 | CEBPA,EGR2,FBXW7,FGFR1,FGFR2,FZD4,HDAC9,NOCT,PER2,TXNIP |
| Acetate Conversion to Acetyl-CoA | 2,35E00 | ACSL1,ACSS1 |
| STAT3 Pathway | 2,33E00 | BCL2,CISH,FGFR1,FGFR2,IL17RE,IL22RA1,MYC,PDGFB,SOCS3,SRC |
| Gαq Signaling | 2,31E00 | GNG2,HRH1,NFKB2,NFKBIE,PIK3C2B,PLD6,RGS2,RHOB,RHOBTB2,RND1,RND3 |
| Bile Acid Biosynthesis, Neutral Pathway | 2,3E00 | AKR1C3,AMACR,CYP27A1 |
| HOTAIR Regulatory Pathway | 2,27E00 | DNMT3B,FOXA1,ICAM1,IRF1,MDM2,MMP1,MMP11,MYC,NFKB2,PIK3C2B,VIM |
| Semaphorin Neuronal Repulsive Signaling Pathway | 2,24E00 | DPYSL3,ITGA2,ITGA3,ITGA5,ITGA6,MAP2K6,PAK1,PAK6,PIK3C2B,RND1 |
| Glucocorticoid Receptor Signaling | 2,24E00 | BCL2,CEBPA,CXCL8,DUSP1,FBP1,FGG,FOS,HSPA1L,ICAM1,IL17RE,IL22RA1,KRT17,KRT6A,MMP1,MYC,NFAT5,NFKBIE,PIK3C2B,PLA2G12B,PTGS2,SERPINE1,SRC,TGFB3 |
| Role of PKR in Interferon Induction and Antiviral Response | 2,23E00 | ATF3,FOS,HSPA1L,IL18,IRF1,MAP2K6,NFKB2,NFKBIE,PDGFB |
| MIF Regulation of Innate Immunity | 2,22E00 | FOS,NFKB2,NFKBIE,PLA2G12B,PTGS2 |
| Cholecystokinin/Gastrin-mediated Signaling | 2,2E00 | FOS,IL18,MAP2K6,PTGS2,RHOB,RHOBTB2,RND1,RND3,SRC |
| Retinoic acid Mediated Apoptosis Signaling | 2,19E00 | CFLAR,IRF1,RXRA,TIPARP,TNFRSF10A,TNFRSF10B |
| D-myo-inositol (1,4,5,6)-Tetrakisphosphate Biosynthesis | 2,18E00 | DUSP1,DUSP2,DUSP5,EPHX2,FIG4,MTMR9,NOCT,PALD1,PRUNE1,SOCS3 |
| D-myo-inositol (3,4,5,6)-tetrakisphosphate Biosynthesis | 2,18E00 | DUSP1,DUSP2,DUSP5,EPHX2,FIG4,MTMR9,NOCT,PALD1,PRUNE1,SOCS3 |
| Endothelin-1 Signaling | 2,17E00 | CASP6,EDN1,FOS,GAB1,GNAI1,GUCY2C,MYC,PIK3C2B,PLA2G12B,PLD6,PTGS2,SRC |
| LXR/RXR Activation | 2,16E00 | APOL1,C3,FGA,IL18,LDLR,NFKB2,PTGS2,RXRA,SERPINF2 |
| Rac Signaling | 2,16E00 | ITGA2,ITGA3,ITGA5,ITGA6,NFKB2,PAK1,PAK6,PIK3C2B,SH3RF1 |
| NRF2-mediated Oxidative Stress Response | 2,15E00 | DNAJC15,FOS,FOSL1,GSTA2,GSTA4,GSTK1,JUNB,MAFF,MAFG,MAFK,MAP2K6,PIK3C2B |
| 3-phosphoinositide Biosynthesis | 2,15E00 | DUSP1,DUSP2,DUSP5,EPHX2,FIG4,MTMR9,NOCT,PALD1,PIK3C2B,PRUNE1,SOCS3 |
| Xenobiotic Metabolism Signaling | 2,15E00 | ALDH1A1,ALDH3B2,ALDH6A1,CHST13,CYP1A1,GSTA2,GSTA4,GSTK1,MAP2K6,NFKB2,PIK3C2B,PPP2R2B,RXRA,SULT1C2,SULT1C4,SULT1E1 |
| Role of Macrophages, Fibroblasts and Endothelial Cells in Rheumatoid Arthritis | 2,14E00 | CCND1,CEBPA,CXCL8,FOS,FZD4,ICAM1,IL18,LRP1,MAP2K6,MMP1,MYC,NFAT5,NFKBIE,PDGFB,PIK3C2B,SOCS3,SRC |
| Serine Biosynthesis | 2,13E00 | PHGDH,PSPH |
| RAR Activation | 2,07E00 | ADH4,AKR1C3,ALDH1A1,DHRS3,DUSP1,FOS,MMP1,NFKB2,RELB,RXRA,SRC,TGFB3 |
| FAK Signaling | 2,06E00 | ITGA2,ITGA3,ITGA5,ITGA6,PAK1,PAK6,PIK3C2B,SRC |
| FXR/RXR Activation | 2,05E00 | APOL1,C3,CYP27A1,FBP1,FGA,FOXA1,IL18,RXRA,SERPINF2 |
| IL-6 Signaling | 2,05E00 | CXCL8,FOS,IL18,MAP2K6,MCL1,NFKB2,NFKBIE,PIK3C2B,SOCS3 |
| Granulocyte Adhesion and Diapedesis | 2,04E00 | CLDN3,CXCL2,CXCL8,CXCR4,GNAI1,HRH1,ICAM1,IL18,MMP1,MMP11,SDC2 |
| Extrinsic Prothrombin Activation Pathway | 2,03E00 | F7,FGA,FGG |
| PAK Signaling | 2,01E00 | ITGA2,ITGA3,ITGA5,ITGA6,PAK1,PAK6,PDGFB,PIK3C2B |
| Ephrin A Signaling | 2,01E00 | EFNA1,EPHA2,PAK1,PIK3C2B,VAV3 |
| Neuroinflammation Signaling Pathway | 1,97E00 | BCL2,BIRC3,CFLAR,CXCL8,FOS,GABBR1,GJB1,GLUL,ICAM1,IL18,NFAT5,NFKB2,PIK3C2B,PLA2G12B,PTGS2,TGFB3 |
| Cell Cycle: G1/S Checkpoint Regulation | 1,96E00 | CCND1,CDKN2C,HDAC9,MDM2,MYC,TGFB3 |
| Pancreatic Adenocarcinoma Signaling | 1,94E00 | BCL2,CCND1,MDM2,NFKB2,PIK3C2B,PLD6,PTGS2,TGFB3 |
| RANK Signaling in Osteoclasts | 1,91E00 | BIRC3,FOS,MAP2K6,NFKB2,NFKBIE,PIK3C2B,SRC |
| IL-12 Signaling and Production in Macrophages | 1,9E00 | APOL1,FOS,IL18,IRF1,MST1R,NFKB2,PIK3C2B,RXRA,TGFB3 |
| 3-phosphoinositide Degradation | 1,9E00 | DUSP1,DUSP2,DUSP5,EPHX2,FIG4,MTMR9,NOCT,PALD1,PRUNE1,SOCS3 |
| Phagosome Formation | 1,9E00 | ITGA2,ITGA3,ITGA5,ITGA6,PIK3C2B,RHOB,RHOBTB2,RND1,RND3 |
| Acute Phase Response Signaling | 1,9E00 | C3,FGA,FGG,FOS,IL18,MAP2K6,NFKB2,NFKBIE,SERPINE1,SERPINF2,SOCS3 |
| IL-9 Signaling | 1,89E00 | CISH,NFKB2,PIK3C2B,SOCS3 |
| Regulation of IL-2 Expression in Activated and Anergic T Lymphocytes | 1,89E00 | BCL10,FOS,NFAT5,NFKB2,NFKBIE,TGFB3,VAV3 |
| D-myo-inositol-5-phosphate Metabolism | 1,89E00 | DUSP1,DUSP2,DUSP5,EPHX2,FIG4,MTMR9,NOCT,PALD1,PRUNE1,SOCS3 |
| IL-10 Signaling | 1,87E00 | FOS,IL18,MAP2K6,NFKB2,NFKBIE,SOCS3 |
| PTEN Signaling | 1,84E00 | BCL2,CCND1,FGFR1,FGFR2,ITGA2,ITGA3,ITGA5,ITGA6,NFKB2 |
| MIF-mediated Glucocorticoid Regulation | 1,84E00 | NFKB2,NFKBIE,PLA2G12B,PTGS2 |
| Retinoate Biosynthesis I | 1,84E00 | ADH4,AKR1C3,ALDH1A1,DHRS3 |
| Superpathway of Serine and Glycine Biosynthesis I | 1,83E00 | PHGDH,PSPH |
| GADD45 Signaling | 1,82E00 | CCND1,GADD45A,GADD45B |
| Valine Degradation I | 1,82E00 | ACADSB,ALDH6A1,EHHADH |
| Oxidative Ethanol Degradation III | 1,82E00 | ACSL1,ACSS1,ALDH1A1 |
| Ephrin B Signaling | 1,82E00 | CXCR4,EFNB2,GNAI1,GNG2,PAK1,VAV3 |
| Glioma Invasiveness Signaling | 1,79E00 | PIK3C2B,PLAUR,RHOB,RHOBTB2,RND1,RND3 |
| ErbB Signaling | 1,79E00 | AREG,ERBB3,FOS,MAP2K6,PAK1,PAK6,PIK3C2B |
| Sphingosine-1-phosphate Signaling | 1,77E00 | CASP6,GNAI1,PDGFB,PIK3C2B,RHOB,RHOBTB2,RND1,RND3 |
| Regulation of Cellular Mechanics by Calpain Protease | 1,76E00 | CCND1,ITGA2,ITGA3,ITGA5,ITGA6,SRC |
| Fatty Acid α-oxidation | 1,76E00 | ALDH1A1,ALOXE3,PTGS2 |
| Production of Nitric Oxide and Reactive Oxygen Species in Macrophages | 1,76E00 | APOL1,FOS,IRF1,NFKB2,NFKBIE,PIK3C2B,PPP2R2B,RHOB,RHOBTB2,RND1,RND3 |
| Amyotrophic Lateral Sclerosis Signaling | 1,72E00 | BCL2,BIRC3,GLUL,GRIK4,PAK1,PIK3C2B,RAB5B |
| Cell Cycle Regulation by BTG Family Proteins | 1,72E00 | CCND1,HOXB9,NOCT,PPP2R2B |
| Endocannabinoid Cancer Inhibition Pathway | 1,72E00 | ATF3,CASP6,CCND1,GNAI1,MAP2K6,MYC,PIK3C2B,SRC,VIM |
| NF-κB Activation by Viruses | 1,71E00 | ITGA2,ITGA3,ITGA6,NFKB2,NFKBIE,PIK3C2B |
| Neurotrophin/TRK Signaling | 1,71E00 | FOS,GAB1,MAP2K6,PIK3C2B,SPRY1,SPRY2 |
| Xenobiotic Metabolism PXR Signaling Pathway | 1,71E00 | ALDH1A1,ALDH3B2,ALDH6A1,CHST13,GSTA2,GSTA4,GSTK1,RXRA,SULT1C2,SULT1C4,SULT1E1 |
| Leukocyte Extravasation Signaling | 1,69E00 | CLDN3,CXCR4,GNAI1,ICAM1,ITGA2,ITGA3,MMP1,MMP11,PIK3C2B,SRC,VAV3 |
| Sertoli Cell-Sertoli Cell Junction Signaling | 1,68E00 | CLDN3,EPN3,ITGA2,ITGA3,ITGA5,ITGA6,MYO7A,SRC,TGFB3,TJP3,TUBB3 |
| G-Protein Coupled Receptor Signaling | 1,66E00 | DUSP1,DUSP4,DUSP6,DUSP9,GABBR1,GNAI1,HRH1,NFKB2,NFKBIE,PDE6A,PIK3C2B,PLD6,RGS2,SRC |
| IL-7 Signaling Pathway | 1,66E00 | BCL2,CCND1,MCL1,MYC,PIK3C2B,SLC2A1 |
| Apoptosis Signaling | 1,66E00 | BCL2,BIRC3,CASP6,GAS2,MCL1,NFKB2,NFKBIE |
| Inhibition of Matrix Metalloproteases | 1,64E00 | LRP1,MMP1,MMP11,SDC2 |
| Thyroid Cancer Signaling | 1,63E00 | CCND1,CXCL8,CXCR4,FOS,MYC,PIK3C2B |
| Superpathway of Inositol Phosphate Compounds | 1,61E00 | DUSP1,DUSP2,DUSP5,EPHX2,FIG4,MTMR9,NOCT,PALD1,PIK3C2B,PRUNE1,SOCS3 |
| April Mediated Signaling | 1,6E00 | FOS,NFAT5,NFKB2,NFKBIE |
| Relaxin Signaling | 1,6E00 | FOS,GNAI1,GNG2,GUCY2C,NFKB2,NFKBIE,PDE6A,PIK3C2B,PLD6 |
| Coronavirus Pathogenesis Pathway | 1,6E00 | BCL2,CCND1,CXCL8,FOS,NFKB2,NFKBIE,PTGS2,SERPINE1,TMPRSS2 |
| Ethanol Degradation IV | 1,6E00 | ACSL1,ACSS1,ALDH1A1 |
| Chronic Myeloid Leukemia Signaling | 1,59E00 | CCND1,HDAC9,MDM2,MYC,NFKB2,PIK3C2B,TGFB3 |
| P2Y Purigenic Receptor Signaling Pathway | 1,58E00 | FOS,GNAI1,GNG2,MYC,NFKB2,P2RY2,P2RY6,PIK3C2B |
| B Cell Activating Factor Signaling | 1,57E00 | FOS,NFAT5,NFKB2,NFKBIE |
| Induction of Apoptosis by HIV1 | 1,56E00 | BCL2,BIRC3,CXCR4,NFKB2,NFKBIE |
| Glutamine Biosynthesis I | 1,55E00 | GLUL |
| Methylglyoxal Degradation VI | 1,55E00 | LDHD |
| Sulfite Oxidation IV | 1,55E00 | SUOX |
| PPAR Signaling | 1,55E00 | FOS,IL18,NFKB2,NFKBIE,PDGFB,PTGS2,RXRA |
| LPS-stimulated MAPK Signaling | 1,54E00 | FOS,MAP2K6,NFKB2,NFKBIE,PAK1,PIK3C2B |
| T Cell Receptor Signaling | 1,53E00 | BCL10,FOS,NFAT5,NFKB2,PIK3C2B,SHB,VAV3 |
| Role of Pattern Recognition Receptors in Recognition of Bacteria and Viruses | 1,53E00 | C3,CLCF1,CXCL8,IL11,IL18,LIF,NFKB2,PIK3C2B,TGFB3 |
| NF-κB Signaling | 1,53E00 | BCL10,FGFR1,FGFR2,IL18,MAP2K6,NFKB2,NFKBIE,PIK3C2B,RELB,TNFAIP3 |
| HER-2 Signaling in Breast Cancer | 1,52E00 | AREG,CCND1,ERBB3,MDM2,PARD6B,PIK3C2B |
| BAG2 Signaling Pathway | 1,5E00 | HSPA1L,MDM2,MYC,NFKB2 |
| HIPPO signaling | 1,5E00 | AMOT,LATS1,PPP2R2B,RASSF6,SAV1,SFN |
| PDGF Signaling | 1,48E00 | ABL2,FOS,MYC,PDGFB,PIK3C2B,SRC |
| IL-23 Signaling Pathway | 1,47E00 | NFKB2,PIK3C2B,RUNX1,SOCS3 |
| Glioma Signaling | 1,46E00 | CCND1,CDKN2C,IDH1,IGF2,MDM2,PDGFB,PIK3C2B |
| Sirtuin Signaling Pathway | 1,46E00 | CXCL8,DUSP6,EPO,ESRRA,GADD45A,GADD45B,HMGCS2,LDHD,MYC,MYCN,NDRG1,NFKB2,RRP9,SLC2A1 |
| iNOS Signaling | 1,44E00 | FOS,IRF1,NFKB2,NFKBIE |
| Hepatic Cholestasis | 1,44E00 | CLCF1,CXCL8,CYP27A1,IL11,IL18,LIF,NFKB2,NFKBIE,RXRA,TGFB3 |
| B Cell Receptor Signaling | 1,44E00 | BCL10,EGR1,ETS1,GAB1,MAP2K6,NFAT5,NFKB2,NFKBIE,PIK3C2B,VAV3 |
| Dermatan Sulfate Biosynthesis (Late Stages) | 1,41E00 | CHST13,SULT1C2,SULT1C4,SULT1E1 |
| FAT10 Cancer Signaling Pathway | 1,41E00 | CXCR4,HOXB9,NFKB2,TGFB3 |
| PFKFB4 Signaling Pathway | 1,41E00 | FBP1,HK2,MAP2K6,TGFB3 |
| Serotonin Degradation | 1,41E00 | ADH4,ADH6,ALDH1A1,SULT1C4,UGT2A3 |
| MSP-RON Signaling In Macrophages Pathway | 1,41E00 | FOS,MST1R,NFKB2,NFKBIZ,PIK3C2B,PTGS2,SOCS3 |
| PI3K Signaling in B Lymphocytes | 1,4E00 | ATF3,BCL10,C3,FOS,NFAT5,NFKB2,NFKBIE,VAV3 |
| Nur77 Signaling in T Lymphocytes | 1,38E00 | BCL2,ESRRA,HDAC9,NR4A1,RXRA |
| Ovarian Cancer Signaling | 1,38E00 | ARRB1,BCL2,CCND1,EDN1,FZD4,PIK3C2B,PTGS2,SRC |
| Prostate Cancer Signaling | 1,37E00 | BCL2,CCND1,MDM2,NFKB2,NFKBIE,PIK3C2B |
| Glucose and Glucose-1-phosphate Degradation | 1,37E00 | HK2,RGN |
| IL-1 Signaling | 1,36E00 | FOS,GNAI1,GNG2,MAP2K6,NFKB2,NFKBIE |
| Chondroitin Sulfate Biosynthesis (Late Stages) | 1,35E00 | CHST13,SULT1C2,SULT1C4,SULT1E1 |

| | | |
|---|---|---|
| GM-CSF Signaling | 1,34E00 | CCND1,CISH,ETS1,PIK3C2B,RUNX1 |
| Superpathway of Cholesterol Biosynthesis | 1,33E00 | HMGCS2,MVD,TM7SF2 |
| Hematopoiesis from Pluripotent Stem Cells | 1,32E00 | CXCL8,EPO,IL11,LIF |
| Airway Pathology in Chronic Obstructive Pulmonary Disease | 1,32E00 | CLCF1,CXCL8,IL11,IL18,LIF,MMP1,TGFB3 |
| Growth Hormone Signaling | 1,32E00 | CEBPA,FOS,IGF2,PIK3C2B,SOCS3 |
| Fatty Acid Activation | 1,3E00 | ACSL1,ACSL5 |

**Supplementary Table S13. List of Upstream Regulators for the condition "Calu-3_SARS-CoV-2".** The IPA-based Core Analysis was performed on the basis of differentially expressed genes discerned for a given condition by GENEVESTIGATOR-based analysis. Only statistically significant Upstream Regulators are included (n = 5280). The data are taken as the basis for R-based comparisons by Venn diagrams shown in Figure 5.

| Upstream Regulator | Molecule Type | Predicted Activation State | p-value of overlap | Target Molecules in Dataset |
|---|---|---|---|---|
| lipopolysaccharide | chemical drug | Activated | 4,36E-79 | [illegible] |
| IFNG | cytokine | Activated | 4,64E-75 | [illegible] |
| poly rI:rC-RNA | biologic drug | Activated | 8,10E-73 | [illegible] |
| Interferon alpha | group | Activated | 1,57E-71 | [illegible] |
| TNF | cytokine | Activated | 1,17E-67 | [illegible] |
| IFNA2 | cytokine | Activated | 1,28E-64 | [illegible] |
| MAPK1 | kinase | Inhibited | 1,66E-63 | [illegible] |
| dexamethasone | chemical drug | Inhibited | 6,67E-62 | [illegible] |
| STAT3 | transcription regulator | Activated | 1,02E-60 | [illegible] |
| beta-estradiol | chemical - endogenous mammalian | | 1,70E-60 | [illegible] |
| IRF7 | transcription regulator | Activated | 9,42E-50 | [illegible] |
| IFNL1 | cytokine | Activated | 2,46E-49 | [illegible] |
| resiquimod | chemical drug | Activated | 1,84E-48 | [illegible] |
| STAT1 | transcription regulator | Activated | 6,78E-47 | [illegible] |
| IL1B | cytokine | Activated | 9,84E-47 | [illegible] |
| NKX2-3 | transcription regulator | Inhibited | 1,47E-46 | [illegible] |
| TGFB1 | growth factor | Activated | 3,69E-42 | [illegible] |
| NFkB (complex) | complex | Activated | 5,24E-42 | [illegible] |
| PRL | cytokine | Activated | 3,24E-40 | [illegible] |
| IFNB1 | cytokine | Activated | 2,63E-38 | [illegible] |
| IRF1 | transcription regulator | Activated | 2,81E-38 | [illegible] |
| SB203580 | chemical - kinase inhibitor | Inhibited | 3,30E-38 | [illegible] |
| OSM | cytokine | Activated | 2,15E-36 | [illegible] |
| TLR3 | transmembrane receptor | Activated | 4,99E-34 | [illegible] |
| IL1RN | cytokine | Inhibited | 6,13E-34 | [illegible] |
| CD40LG | cytokine | Activated | 1,31E-33 | [illegible] |
| NR3C1 | ligand-dependent nuclear receptor | Inhibited | 1,75E-33 | [illegible] |
| TP53 | transcription regulator | Activated | 1,01E-32 | [illegible] |
| Ifnar | group | Activated | 1,48E-32 | [illegible] |
| IRF3 | transcription regulator | Activated | 2,06E-32 | [illegible] |
| ESR1 | ligand-dependent nuclear receptor | Inhibited | 2,48E-32 | [illegible] |
| IFNAR1 | transmembrane receptor | Activated | 3,61E-32 | [illegible] |
| RELA | transcription regulator | Activated | 4,44E-32 | [illegible] |
| Immunoglobulin | complex | Activated | 9,63E-32 | [illegible] |
| camptothecin | chemical drug | Activated | 1,32E-31 | [illegible] |
| IFN Beta | group | Activated | 7,89E-31 | [illegible] |
| CSF2 | cytokine | Activated | 1,93E-30 | [illegible] |
| FOXO3 | transcription regulator | | 1,46E-29 | [illegible] |
| filgrastim | biologic drug | Inhibited | 2,01E-29 | [illegible] |

| | | | | |
|---|---|---|---|---|
| 30s Proteasome | complex | | 2.29E-10 | [illegible] |
| CpG ODN 1668 | chemical reagent | Activated | 2.47E-10 | [illegible] |
| USP18 | peptidase | Inhibited | 2.52E-10 | [illegible] |
| TNFRSF1A | transmembrane receptor | Activated | 2.68E-10 | [illegible] |
| SATB1 | transcription regulator | | 2.70E-10 | [illegible] |
| BTK | kinase | | 2.86E-10 | [illegible] |
| homocysteine | chemical - endogenous mammalian | Activated | 3.01E-10 | [illegible] |
| TLR2 | transmembrane receptor | Activated | 3.01E-10 | [illegible] |
| CCL5 | cytokine | Activated | 3.23E-10 | [illegible] |
| EPAS1 | transcription regulator | Activated | 3.27E-10 | [illegible] |
| CDKN1A | kinase | Activated | 3.47E-10 | [illegible] |
| FGF | growth factor | Activated | 3.52E-10 | [illegible] |
| TLR7/8 | group | Activated | 3.52E-10 | [illegible] |
| laminaran | chemical drug | Activated | 3.52E-10 | [illegible] |
| FN1 | enzyme | Activated | 3.62E-10 | [illegible] |
| CSF3 | cytokine | | 3.63E-10 | [illegible] |
| CD24 | other | | 3.72E-10 | [illegible] |
| CERK | kinase | | 4.27E-10 | [illegible] |
| SUMO3 | other | Inhibited | 4.27E-10 | [illegible] |
| TRAF2 | enzyme | Activated | 4.58E-10 | [illegible] |
| ATP-gamma-S | chemical reagent | | 4.71E-10 | [illegible] |
| cytarabine | chemical drug | Activated | 4.71E-10 | [illegible] |
| SMPD1 | enzyme | Activated | 4.82E-10 | [illegible] |
| Irgm1 | other | | 5.05E-10 | [illegible] |
| Ro31-8220 | chemical - kinase inhibitor | Inhibited | 5.05E-10 | [illegible] |
| IL3 | cytokine | Activated | 5.13E-10 | [illegible] |
| tamoxifen | chemical drug | | 5.19E-10 | [illegible] |
| CD36 | transmembrane receptor | Activated | 5.22E-10 | [illegible] |
| lovastatin | chemical drug | | 5.58E-10 | [illegible] |
| EZH2 | transcription regulator | | 5.78E-10 | [illegible] |
| miR-17-5p (and other miRNAs w/seed AAAGUGC) | mature microRNA | Inhibited | 5.95E-10 | [illegible] |
| [illegible] (and other miRNAs w/seed CAUUGCA) | mature microRNA | Inhibited | 6.20E-10 | [illegible] |
| TAL1 | transcription regulator | Inhibited | 6.29E-10 | [illegible] |
| AGN194204 | chemical drug | | 6.29E-10 | [illegible] |
| RPTOR | other | Inhibited | 6.53E-10 | [illegible] |
| IFIH1 | enzyme | Activated | 6.60E-10 | [illegible] |
| BAPTA-AM | chemical reagent | Inhibited | 6.60E-10 | [illegible] |
| [illegible] | [illegible] | [illegible] | [illegible] | [illegible] |

| | | | | |
|---|---|---|---|---|
| CXCR4 | G-protein coupled receptor | Activated | 8,41E-07 | [illegible] |
| carbon monoxide | chemical - endogenous mammalian | Inhibited | 8,41E-07 | [illegible] |
| rifampin | chemical drug | | 8,60E-07 | [illegible] |
| S100A8 | other | Activated | 8,72E-07 | [illegible] |
| lipoteichoic acid | chemical - endogenous non-mammalian | Activated | 8,91E-07 | [illegible] |
| BMP7 | growth factor | | 9,31E-07 | [illegible] |
| MAP2K4 | kinase | Activated | 9,33E-07 | [illegible] |
| MEF2C | transcription regulator | | 9,33E-07 | [illegible] |
| miR-199a-5p (and other miRNAs w/seed CCAGUGU) | mature microRNA | Inhibited | 9,36E-07 | [illegible] |
| OSCAR | other | Activated | 9,48E-07 | [illegible] |
| miR-4757-5p (and other miRNAs w/seed GGGAAGG) | mature microRNA | Inhibited | 9,83E-07 | [illegible] |
| miR-128-3p (and other miRNAs w/seed CACAGUG) | mature microRNA | Inhibited | 1,01E-06 | [illegible] |
| L-methionine | chemical - endogenous mammalian | | 1,02E-06 | [illegible] |
| NFKBIB | transcription regulator | | 1,02E-06 | [illegible] |
| Am 580 | chemical toxicant | Activated | 1,02E-06 | [illegible] |
| miR-92a-3p (and other miRNAs w/seed AUUGCAC) | mature microRNA | Inhibited | 1,02E-06 | [illegible] |
| MAC | complex | Activated | 1,03E-06 | [illegible] |
| ANGPT2 | growth factor | Activated | 1,05E-06 | [illegible] |
| TLR5 | transmembrane receptor | Activated | 1,06E-06 | [illegible] |
| mevalonic acid | chemical - endogenous mammalian | | 1,06E-06 | [illegible] |
| PRKD1 | kinase | Activated | 1,08E-06 | [illegible] |
| miR-363-3p (and other miRNAs w/seed AAUUGCC) | mature microRNA | Inhibited | 1,10E-06 | [illegible] |
| ITGB1 | transmembrane receptor | Activated | 1,11E-06 | [illegible] |
| [illegible] | chemical drug | Activated | 1,12E-06 | [illegible] |
| miR-605-3p (miRNAs w/seed GAAGGCA) | mature microRNA | Inhibited | 1,14E-06 | [illegible] |
| arachidonic acid | chemical - endogenous mammalian | | 1,18E-06 | [illegible] |
| miR-4514 (and other miRNAs w/seed CAGGCAG) | mature microRNA | | 1,19E-06 | [illegible] |
| silibinin | chemical drug | | 1,20E-06 | [illegible] |
| 2-deoxyglucose | chemical drug | | 1,20E-06 | [illegible] |
| losartan potassium | chemical drug | | 1,21E-06 | [illegible] |
| LYN | kinase | | 1,28E-06 | [illegible] |
| SR 144528 | chemical reagent | | 1,28E-06 | [illegible] |
| [illegible] | transcription regulator | Activated | 1,30E-06 | [illegible] |
| PPARD | ligand-dependent nuclear receptor | | 1,30E-06 | [illegible] |
| miR-6829-5p (miRNAs w/seed GGGCUGC) | mature microRNA | | 1,33E-06 | [illegible] |
| CARD9 | other | Activated | 1,33E-06 | [illegible] |
| miR-302c-3p (and other miRNAs w/seed AGUGCUU) | mature microRNA | Inhibited | 1,35E-06 | [illegible] |
| RELB | transcription regulator | Activated | 1,36E-06 | [illegible] |
| caffeine | chemical drug | | 1,40E-06 | [illegible] |
| isobutylmethylxanthine | chemical toxicant | | 1,42E-06 | [illegible] |
| Pdgf (complex) | complex | Activated | 1,45E-06 | [illegible] |
| CEBPD | transcription regulator | Activated | 1,46E-06 | [illegible] |
| celecoxib | chemical drug | | 1,47E-06 | [illegible] |
| potassium chloride | chemical drug | Activated | 1,49E-06 | [illegible] |
| ETS1 | transcription regulator | Activated | 1,50E-06 | [illegible] |
| NFKB2 | transcription regulator | Activated | 1,51E-06 | [illegible] |
| miR-221-5p (and other miRNAs w/seed CCUGGCA) | mature microRNA | Inhibited | 1,51E-06 | [illegible] |
| SN-38 | chemical drug | Activated | 1,54E-06 | [illegible] |
| HNRNPA2B1 | other | | 1,56E-06 | [illegible] |
| TSC22D3 | transcription regulator | | 1,57E-06 | [illegible] |
| NOTCH1 | transcription regulator | Activated | 1,58E-06 | [illegible] |
| ABT-737 | chemical drug | Activated | 1,58E-06 | [illegible] |
| ADRB3 | G-protein coupled receptor | Activated | 1,58E-06 | [illegible] |
| SOCS6 | other | | 1,58E-06 | [illegible] |
| PPP1R15A | other | | 1,58E-06 | [illegible] |
| rotenone | chemical toxicant | | 1,61E-06 | [illegible] |
| SB 216763 | chemical toxicant | | 1,65E-06 | [illegible] |
| parthenolide | chemical drug | | 1,66E-06 | [illegible] |
| let-7c-1-3p (and other miRNAs w/seed UGUACAA) | mature microRNA | Inhibited | 1,67E-06 | [illegible] |
| IFNA8 | cytokine | Activated | 1,68E-06 | [illegible] |
| BTC | growth factor | Activated | 1,69E-06 | [illegible] |
| IFNA16 | cytokine | Activated | 1,69E-06 | [illegible] |
| VHL | transcription regulator | Activated | 1,70E-06 | [illegible] |
| IL33 | cytokine | Activated | 1,70E-06 | [illegible] |
| TCF | group | | 1,72E-06 | [illegible] |
| miR-936 (miRNAs w/seed CAGUAGA) | mature microRNA | Inhibited | 1,75E-06 | [illegible] |
| OPA1 | enzyme | Inhibited | 1,76E-06 | [illegible] |
| PNEP | other | | 1,76E-06 | [illegible] |
| HDAC3 | transcription regulator | | 1,79E-06 | [illegible] |
| Bay 11-7082 | chemical - kinase inhibitor | Inhibited | 1,80E-06 | [illegible] |
| miR-6512-3p (and other miRNAs w/seed UCCAGCC) | mature microRNA | Inhibited | 1,82E-06 | [illegible] |
| TRIB3 | kinase | Inhibited | 1,83E-06 | [illegible] |
| sterol | chemical - endogenous mammalian | | 1,83E-06 | [illegible] |
| mir-15 | microRNA | | 1,85E-06 | [illegible] |
| gemcitabine | chemical drug | Activated | 1,85E-06 | [illegible] |
| miR-1233-5p (and other miRNAs w/seed GUGGGAG) | mature microRNA | | 1,86E-06 | [illegible] |
| miR-669c-3p (and other miRNAs w/seed ACACACA) | mature microRNA | Inhibited | 1,97E-06 | [illegible] |
| aldosterone | chemical - endogenous mammalian | Activated | 2,02E-06 | [illegible] |
| miR-635 (and other miRNAs w/seed CUUGGGC) | mature microRNA | | 2,06E-06 | [illegible] |
| PPARGC1A | transcription regulator | | 2,09E-06 | [illegible] |
| H89 | chemical - kinase inhibitor | Inhibited | 2,14E-06 | [illegible] |
| NRG2 | growth factor | | 2,19E-06 | [illegible] |
| STK11 | kinase | | 2,20E-06 | [illegible] |
| SRC (family) | group | Activated | 2,24E-06 | [illegible] |
| eicosapentaenoic acid | chemical drug | | 2,28E-06 | [illegible] |
| EIF4E | translation regulator | Activated | 2,28E-06 | [illegible] |
| [illegible] | chemical reagent | | 2,30E-06 | [illegible] |
| gossypol | chemical drug | | 2,30E-06 | [illegible] |
| C-miR146a | chemical reagent | | 2,30E-06 | [illegible] |
| CBX5 | transcription regulator | | 2,31E-06 | [illegible] |
| MAP2K1/2 | group | Activated | 2,36E-06 | [illegible] |
| ciglitazone | chemical drug | | 2,36E-06 | [illegible] |
| fenretinide | chemical drug | | 2,36E-06 | [illegible] |
| E2F1 | transcription regulator | Activated | 2,37E-06 | [illegible] |
| [illegible] | chemical drug | | 2,38E-06 | [illegible] |
| miR-493-5p (miRNAs w/seed UGUACAU) | mature microRNA | Inhibited | 2,41E-06 | [illegible] |
| aldesleukin | biologic drug | Activated | 2,41E-06 | [illegible] |
| NfkB-RelA | complex | Activated | 2,43E-06 | [illegible] |
| COMMD1 | transporter | | 2,43E-06 | [illegible] |
| piceatannol | chemical - endogenous non-mammalian | | 2,43E-06 | [illegible] |
| tosylphenylalanyl chloromethyl ketone | chemical - protease inhibitor | Inhibited | 2,43E-06 | [illegible] |
| CASP1 | peptidase | | 2,45E-06 | [illegible] |
| mir-26 | microRNA | Inhibited | 2,45E-06 | [illegible] |
| IL12 (family) | group | Activated | 2,49E-06 | [illegible] |
| pioglitazone | chemical drug | | 2,50E-06 | [illegible] |
| androgen | chemical drug | | 2,50E-06 | [illegible] |
| NAMPT | cytokine | Activated | 2,52E-06 | [illegible] |
| RIPK2 | kinase | Activated | 2,53E-06 | [illegible] |
| DACH1 | transcription regulator | Inhibited | 2,54E-06 | [illegible] |
| LCN2 | transporter | | 2,70E-06 | [illegible] |
| miR-486-3p (and other miRNAs w/seed GGGGCAG) | mature microRNA | Inhibited | 2,73E-06 | [illegible] |
| IL32 | cytokine | Activated | 2,85E-06 | [illegible] |
| miR-5694 (miRNAs w/seed AGAUCAU) | mature microRNA | Inhibited | 2,88E-06 | [illegible] |
| RASL6 | other | Inhibited | 2,96E-06 | [illegible] |
| miR-503-5p (miRNAs w/seed AGCAGCG) | mature microRNA | Inhibited | 3,03E-06 | [illegible] |
| miR-3106-5p (and other miRNAs w/seed AGGGGGC) | mature microRNA | Inhibited | 3,06E-06 | [illegible] |
| ORMDL3 | other | Activated | 3,09E-06 | [illegible] |
| cis-urocanic acid | chemical drug | Activated | 3,09E-06 | [illegible] |
| MAPK13 | kinase | | 3,09E-06 | [illegible] |
| PTPN3 | phosphatase | | 3,09E-06 | [illegible] |
| selumetinib | chemical drug | | 3,12E-06 | [illegible] |
| IRAK3 | kinase | | 3,12E-06 | [illegible] |
| STAT5B | transcription regulator | | 3,13E-06 | [illegible] |
| HDAC4 | transcription regulator | | 3,18E-06 | [illegible] |
| miR-130a-3p (and other miRNAs w/seed UGUAGGG) | mature microRNA | | 3,26E-06 | [illegible] |
| dimethyl sulfoxide | chemical drug | Activated | 3,28E-06 | [illegible] |
| miR-4722-3p (and other miRNAs w/seed CCUGCCA) | mature microRNA | | 3,33E-06 | [illegible] |
| 4-phenylbutyric acid | chemical - endogenous mammalian | | 3,44E-06 | [illegible] |
| TXN | enzyme | Activated | 3,47E-06 | [illegible] |
| miR-6815-5p (and other miRNAs w/seed AGGUGGC) | mature microRNA | Inhibited | 3,58E-06 | [illegible] |
| 3M-011 | chemical reagent | Activated | 3,58E-06 | [illegible] |
| PDPK1 | kinase | | 3,58E-06 | [illegible] |
| imipramine blue | chemical drug | Activated | 3,58E-06 | [illegible] |
| Pdgfr | group | Activated | 3,61E-06 | [illegible] |
| RPL22 | translation regulator | Inhibited | 3,61E-06 | [illegible] |
| PTGER1 | G-protein coupled receptor | | 3,61E-06 | [illegible] |
| bempedoic acid | chemical drug | Inhibited | 3,61E-06 | [illegible] |
| DICER1 | enzyme | Inhibited | 3,62E-06 | [illegible] |
| [illegible] | complex | Inhibited | 3,66E-06 | [illegible] |
| L-type Calcium Channel | complex | | 3,66E-06 | [illegible] |
| BAP1 | peptidase | | 3,69E-06 | [illegible] |
| DPP-23 | chemical reagent | Activated | 3,69E-06 | [illegible] |
| miR-3907 (miRNAs w/seed GGUGCUC) | mature microRNA | | 3,70E-06 | [illegible] |
| [illegible] | cytokine | | 3,71E-06 | [illegible] |
| nifedipine | chemical drug | Inhibited | 3,71E-06 | [illegible] |
| HDL cholesterol | complex | | 3,81E-06 | [illegible] |
| XDH | enzyme | | 3,81E-06 | [illegible] |
| ARRB2 | other | Inhibited | 3,81E-06 | [illegible] |
| NORAD | other | | 3,84E-06 | [illegible] |
| PLAUR | transmembrane receptor | | 3,84E-06 | [illegible] |
| miR-520d-5p (and other miRNAs w/seed UACAAAG) | mature microRNA | | 3,89E-06 | [illegible] |
| CD14 | transmembrane receptor | Activated | 3,93E-06 | [illegible] |
| LTA | cytokine | Activated | 3,93E-06 | [illegible] |
| APOA1 | transporter | Inhibited | 3,93E-06 | [illegible] |
| IRG1 | enzyme | | 3,97E-06 | [illegible] |
| CXCL2 | cytokine | Activated | 3,99E-06 | [illegible] |
| fingolimod | chemical drug | Inhibited | 4,00E-06 | [illegible] |
| SERCA | group | Inhibited | 4,07E-06 | [illegible] |
| CLEC12A | other | Activated | 4,07E-06 | [illegible] |
| Gm38886 | other | Activated | 4,07E-06 | [illegible] |
| IL6R | transmembrane receptor | Activated | 4,22E-06 | [illegible] |
| Sos | group | | 4,26E-06 | [illegible] |
| DCAF1 | kinase | Inhibited | 4,28E-06 | [illegible] |
| XIAP | enzyme | | 4,29E-06 | [illegible] |
| HMGB2 | transcription regulator | | 4,29E-06 | [illegible] |
| miR-328-5p (and other miRNAs w/seed GGGGGGC) | mature microRNA | Inhibited | 4,52E-06 | [illegible] |
| NGF | growth factor | | 4,66E-06 | [illegible] |
| mono-(2-ethylhexyl)phthalate | chemical toxicant | | 4,76E-06 | [illegible] |
| miR-5008-5p (miRNAs w/seed GAGGCCC) | mature microRNA | Inhibited | 4,90E-06 | [illegible] |
| MED1 | transcription regulator | | 5,11E-06 | [illegible] |
| vismodegib | chemical drug | | 5,16E-06 | [illegible] |
| BAK1 | other | Activated | 5,16E-06 | [illegible] |
| miR-3612 (and other miRNAs w/seed GGAGGCA) | mature microRNA | Inhibited | 5,20E-06 | [illegible] |
| ARNT | transcription regulator | | 5,21E-06 | [illegible] |
| miR-6967-5p (and other miRNAs w/seed AGGGAGG) | mature microRNA | | 5,24E-06 | [illegible] |
| diphtheria toxin | chemical - endogenous non-mammalian | | 5,37E-06 | [illegible] |
| mir-155 | microRNA | Inhibited | 5,37E-06 | [illegible] |
| IL12B | cytokine | Activated | 5,38E-06 | [illegible] |
| miR-12182-5p (and other miRNAs w/seed GUGGGGU) | mature microRNA | Inhibited | 5,40E-06 | [illegible] |
| miR-491-5p (and other miRNAs w/seed GUGGGGA) | mature microRNA | Inhibited | 5,58E-06 | [illegible] |
| EZR | other | | 5,66E-06 | [illegible] |
| ARRB1 | other | | 5,66E-06 | [illegible] |
| PLAT | peptidase | Activated | 5,66E-06 | [illegible] |
| PD184352 | chemical drug | | 5,72E-06 | [illegible] |
| tetrandrine | chemical drug | | 5,72E-06 | [illegible] |
| cannabidiol | chemical drug | | 5,72E-06 | [illegible] |
| miR-376c-5p (and other miRNAs w/seed UAAUACA) | mature microRNA | Inhibited | 5,75E-06 | [illegible] |
| miR-5006-5p (miRNAs w/seed UGCCAGG) | mature microRNA | | 5,77E-06 | [illegible] |
| STAT | group | Activated | 5,81E-06 | [illegible] |
| Calcineurin protein(s) | complex | | 5,83E-06 | [illegible] |
| miR-1270 (and other miRNAs w/seed UGGAGAU) | mature microRNA | | 6,03E-06 | [illegible] |
| HMOX1 | enzyme | | 6,07E-06 | [illegible] |
| tanespimycin | chemical drug | | 6,07E-06 | [illegible] |
| CDK9 | kinase | | 6,16E-06 | [illegible] |
| miR-6559-3p (miRNAs w/seed CUGAGGC) | mature microRNA | Inhibited | 6,21E-06 | [illegible] |
| ATF6 | transcription regulator | | 6,24E-06 | [illegible] |
| 1-methyl-4-phenyl-1,2,3,6-tetrahydropyridine | chemical toxicant | Activated | 6,27E-06 | [illegible] |
| MIF | cytokine | Activated | 6,33E-06 | [illegible] |
| miR-6509-5p (and other miRNAs w/seed GGGAGGA) | mature microRNA | | 6,40E-06 | [illegible] |
| ELAVL1 | other | Activated | 6,60E-06 | [illegible] |
| [illegible] | biologic drug | Activated | 6,87E-06 | [illegible] |
| caffeic acid phenethyl ester | chemical drug | Inhibited | 6,91E-06 | [illegible] |
| TIMP3 | other | Inhibited | 6,96E-06 | [illegible] |
| 9,10-dimethyl-1,2-benzanthracene | chemical toxicant | Activated | 6,96E-06 | [illegible] |
| F2R | G-protein coupled receptor | Activated | 7,00E-06 | [illegible] |
| PTH | other | Activated | 7,18E-06 | [illegible] |
| SAV1 | other | | 7,26E-06 | [illegible] |
| MSR1 | transmembrane receptor | | 7,26E-06 | [illegible] |
| LY6E | other | Inhibited | 7,26E-06 | [illegible] |
| TP53COR1 | other | Activated | 7,26E-06 | [illegible] |
| lapatinib | chemical drug | | 7,26E-06 | [illegible] |
| miR-8065 (miRNAs w/seed GUAGGAA) | mature microRNA | Inhibited | 7,28E-06 | [illegible] |
| SP2509 | chemical reagent | | 7,29E-06 | [illegible] |
| miR-548an (miRNAs w/seed AAAGGCA) | mature microRNA | | 7,30E-06 | [illegible] |
| PROCR | other | | 7,32E-06 | [illegible] |
| ozone | chemical toxicant | Activated | 7,32E-06 | [illegible] |
| ISGF3 | complex | Activated | 7,49E-06 | [illegible] |
| manumycin A | chemical reagent | | 7,49E-06 | [illegible] |
| miR-145-5p (and other miRNAs w/seed UCCAGUU) | mature microRNA | | 7,50E-06 | [illegible] |
| LRP6 | transmembrane receptor | | 7,68E-06 | [illegible] |
| YAP1 | transcription regulator | | 8,04E-06 | [illegible] |
| enterotoxin B | biologic drug | Activated | 8,06E-06 | [illegible] |
| HDAC5 | transcription regulator | | 8,06E-06 | [illegible] |
| DYSF | other | | 8,06E-06 | [illegible] |
| KRT17 | other | | 8,32E-06 | [illegible] |
| SMARCB1 | transcription regulator | Activated | 8,37E-06 | [illegible] |
| miR-607 (miRNAs w/seed UUCAAAU) | mature microRNA | | 8,50E-06 | [illegible] |
| tocilizumab | biologic drug | Inhibited | 8,58E-06 | [illegible] |
| SAA | group | Activated | 8,70E-06 | [illegible] |
| CARM1 | transcription regulator | Activated | 8,70E-06 | [illegible] |
| pristane | chemical toxicant | Activated | 8,70E-06 | [illegible] |
| IL36A | cytokine | Activated | 8,75E-06 | [illegible] |
| bafilomycin A1 | chemical drug | | 8,75E-06 | [illegible] |
| formaldehyde | chemical - endogenous mammalian | Activated | 8,75E-06 | [illegible] |
| miR-485-5p (and other miRNAs w/seed GAGGCUG) | mature microRNA | | 8,96E-06 | [illegible] |
| miR-5002-5p (miRNAs w/seed AUUUGGU) | mature microRNA | Inhibited | 8,96E-06 | [illegible] |
| IFNA17 | cytokine | Activated | 8,98E-06 | [illegible] |
| ST14 | peptidase | Activated | 8,98E-06 | [illegible] |
| nickel chloride | chemical toxicant | | 8,98E-06 | [illegible] |
| HDAC1 | transcription regulator | | 9,05E-06 | [illegible] |
| inosine | chemical - endogenous mammalian | | 9,65E-06 | [illegible] |
| mir-181 | microRNA | Inhibited | 9,82E-06 | [illegible] |
| clozapine | chemical drug | | 9,82E-06 | [illegible] |
| BTNL2 | transmembrane receptor | | 9,85E-06 | [illegible] |
| L-glutamic acid | chemical - endogenous mammalian | | 1,01E-05 | [illegible] |
| ERBB3 | kinase | | 1,01E-05 | [illegible] |
| dexamethasone phosphate | chemical drug | | 1,01E-05 | [illegible] |
| IL36B | cytokine | Activated | 1,01E-05 | [illegible] |
| IL37 | cytokine | Inhibited | 1,01E-05 | [illegible] |
| miR-4520-3p (miRNAs w/seed UGGACAG) | mature microRNA | | 1,02E-05 | [illegible] |
| N-Ac-Leu-Leu-norleucinal | chemical - protease inhibitor | | 1,04E-05 | [illegible] |
| PAX3 | transcription regulator | | 1,06E-05 | [illegible] |
| MRTFB | transcription regulator | Activated | 1,06E-05 | [illegible] |
| miR-6511a-5p (and other miRNAs w/seed AGGCAGA) | mature microRNA | | 1,07E-05 | [illegible] |
| zVAD-FMK | chemical - protease inhibitor | | 1,08E-05 | [illegible] |
| n-3 fatty acids | chemical drug | | 1,08E-05 | [illegible] |
| GW9662 | chemical reagent | | 1,09E-05 | [illegible] |
| TGFBR2 | kinase | | 1,11E-05 | [illegible] |
| Ap1 | complex | Activated | 1,14E-05 | [illegible] |
| CLEC4D | other | | 1,15E-05 | [illegible] |
| GSK3B | kinase | | 1,17E-05 | [illegible] |
| let-7f-2-3p (and other miRNAs w/seed UAUACAG) | mature microRNA | Inhibited | 1,18E-05 | [illegible] |
| baicalein | chemical drug | | 1,19E-05 | [illegible] |
| MAP3K3 | kinase | Activated | 1,19E-05 | [illegible] |
| miR-6851 (and other miRNAs w/seed GGGGUGG) | mature microRNA | Inhibited | 1,24E-05 | [illegible] |
| Notch | group | | 1,24E-05 | [illegible] |
| etanercept | biologic drug | Inhibited | 1,31E-05 | [illegible] |
| RNASEL | enzyme | | 1,31E-05 | [illegible] |
| TIRAP | other | | 1,31E-05 | [illegible] |
| TNFSF14 | cytokine | Activated | 1,31E-05 | [illegible] |
| vitamin K3 | chemical drug | Activated | 1,31E-05 | [illegible] |
| CXCL8 | cytokine | Activated | 1,32E-05 | [illegible] |
| miR-203a-5p (and other miRNAs w/seed AGUGGUC) | mature microRNA | | 1,32E-05 | [illegible] |
| EIF4EBP1 | translation regulator | | 1,32E-05 | [illegible] |
| ADIPOQ | other | | 1,33E-05 | [illegible] |
| CD44 | other | | 1,33E-05 | [illegible] |
| miR-1-3p (and other miRNAs w/seed GGAAUGU) | mature microRNA | Inhibited | 1,34E-05 | [illegible] |
| amphetamine | chemical drug | Inhibited | 1,35E-05 | [illegible] |
| Lymphotoxin | complex | Activated | 1,35E-05 | [illegible] |
| miR-27b-5p (miRNAs w/seed GAGCUUA) | mature microRNA | | 1,39E-05 | [illegible] |
| miR-6893-5p (miRNAs w/seed UACUGUG) | mature microRNA | Inhibited | 1,39E-05 | [illegible] |
| diethylstilbestrol | chemical drug | | 1,42E-05 | [illegible] |
| miR-6135 (and other miRNAs w/seed GGUCUAG) | mature microRNA | Inhibited | 1,43E-05 | [illegible] |
| HMGA1 | transcription regulator | | 1,46E-05 | [illegible] |
| FBXO42 | other | Activated | 1,46E-05 | [illegible] |
| TRG | other | | 1,46E-05 | [illegible] |
| UBA7 | enzyme | | 1,46E-05 | [illegible] |
| CTH | enzyme | | 1,46E-05 | [illegible] |
| SR1078 | chemical reagent | | 1,46E-05 | [illegible] |
| TGFB2 | growth factor | | 1,48E-05 | [illegible] |
| 5-hydroxytryptamine | chemical - endogenous mammalian | Activated | 1,48E-05 | [illegible] |
| miR-4436b-3p (and other miRNAs w/seed AGGGCAG) | mature microRNA | Inhibited | 1,51E-05 | [illegible] |
| Gcg | other | | 1,55E-05 | [illegible] |
| miR-185-3p (and other miRNAs w/seed GGGGCUG) | mature microRNA | Inhibited | 1,55E-05 | [illegible] |
| miR-3127-3p (and other miRNAs w/seed CCCCUUC) | mature microRNA | | 1,60E-05 | [illegible] |
| GLI2 | transcription regulator | | 1,60E-05 | [illegible] |
| MAP2K6 | kinase | Activated | 1,62E-05 | [illegible] |
| FOSL1 | transcription regulator | Inhibited | 1,62E-05 | [illegible] |
| lipid A | chemical toxicant | Activated | 1,69E-05 | [illegible] |
| 15(S)-HETE | chemical - endogenous mammalian | | 1,71E-05 | [illegible] |
| PLC | group | Activated | 1,71E-05 | [illegible] |
| HCAR2 | G-protein coupled receptor | | 1,71E-05 | [illegible] |
| PADI2 | enzyme | | 1,71E-05 | [illegible] |
| PLA2G4A | enzyme | | 1,71E-05 | [illegible] |
| testosterone | chemical - endogenous mammalian | | 1,72E-05 | [illegible] |
| LRPAP1 | other | | 1,72E-05 | [illegible] |
| P2RY2 | G-protein coupled receptor | Activated | 1,72E-05 | [illegible] |
| Fcer1 | complex | Activated | 1,73E-05 | [illegible] |
| miR-6752-3p (miRNAs w/seed CCCUGCC) | mature microRNA | Inhibited | 1,77E-05 | [illegible] |
| miR-363-5p (and other miRNAs w/seed GGGUGGA) | mature microRNA | | 1,77E-05 | [illegible] |
| miR-939-3p (miRNAs w/seed CCUGGGC) | mature microRNA | Inhibited | 1,77E-05 | [illegible] |
| SOX4 | transcription regulator | | 1,78E-05 | [illegible] |
| EIF2B1 | translation regulator | | 1,81E-05 | [illegible] |
| MAP3K1 | kinase | Activated | 1,81E-05 | [illegible] |
| MMP1 | peptidase | Activated | 1,81E-05 | [illegible] |
| miR-23a-3p (and other miRNAs w/seed UCACAUU) | mature microRNA | Inhibited | 1,82E-05 | [illegible] |
| miR-12183-5p (and other miRNAs w/seed CUUCUUC) | mature microRNA | Inhibited | 1,85E-05 | [illegible] |
| Ngf | group | Activated | 1,88E-05 | [illegible] |
| miR-6721-5p (miRNAs w/seed GGGCAGG) | mature microRNA | Inhibited | 1,91E-05 | [illegible] |
| NPC2 | transporter | | 1,94E-05 | [illegible] |
| TANK | other | | 1,97E-05 | [illegible] |
| NOS2 | enzyme | Activated | 1,97E-05 | [illegible] |
| KDM3B | enzyme | | 1,98E-05 | [illegible] |
| TRAF3IP2 | other | Activated | 1,98E-05 | [illegible] |
| prazosin | chemical drug | Inhibited | 1,98E-05 | [illegible] |
| [illegible] | chemical reagent | Inhibited | 2,00E-05 | [illegible] |
| DDB2 | other | | 2,00E-05 | [illegible] |
| miR-570-3p (miRNAs w/seed GAAAACA) | mature microRNA | Inhibited | 2,00E-05 | [illegible] |
| tetrachlorodibenzodioxin | chemical toxicant | Activated | 2,00E-05 | [illegible] |
| HLX | transcription regulator | Inhibited | 2,01E-05 | [illegible] |
| GJA1 | transporter | | 2,01E-05 | [illegible] |
| SOD2 | enzyme | | 2,02E-05 | [illegible] |
| miR-6868-5p (miRNAs w/seed GGGAAAA) | mature microRNA | Inhibited | 2,03E-05 | [illegible] |
| miR-6804-5p (miRNAs w/seed UGGACGG) | mature microRNA | | 2,05E-05 | [illegible] |
| emodin | chemical drug | | 2,06E-05 | [illegible] |
| RETN | other | | 2,09E-05 | [illegible] |
| miR-141-3p (and other miRNAs w/seed AACACUG) | mature microRNA | Inhibited | 2,10E-05 | [illegible] |
| CNTF | cytokine | | 2,11E-05 | [illegible] |
| zinc | chemical drug | Activated | 2,11E-05 | [illegible] |
| FGF19 | growth factor | | 2,12E-05 | [illegible] |
| BCR (complex) | complex | Activated | 2,14E-05 | [illegible] |
| miR-6433b-5p (miRNAs w/seed UGUCCCA) | mature microRNA | Inhibited | 2,15E-05 | [illegible] |
| miR-4802-3p (and other miRNAs w/seed ACAUGGA) | mature microRNA | Inhibited | 2,20E-05 | [illegible] |
| DDX5 | enzyme | Inhibited | 2,27E-05 | [illegible] |
| SMARCA4 | transcription regulator | Inhibited | 2,29E-05 | [illegible] |
| isoproterenol | chemical drug | Activated | 2,34E-05 | [illegible] |
| miR-1273h-5p (and other miRNAs w/seed UGGGAGG) | mature microRNA | Inhibited | 2,34E-05 | [illegible] |
| HOTAIR | other | | 2,37E-05 | [illegible] |
| VitaminD3-VDR-RXR | complex | | 2,37E-05 | [illegible] |
| PRC2 | complex | | 2,37E-05 | [illegible] |
| IFNGR1 | transmembrane receptor | Activated | 2,37E-05 | [illegible] |
| IL12A | cytokine | Activated | 2,37E-05 | [illegible] |
| CRP | other | | 2,37E-05 | [illegible] |
| salinosporamide A | chemical drug | | 2,39E-05 | [illegible] |
| KIF3A | enzyme | Activated | 2,39E-05 | [illegible] |
| cuprizone | chemical toxicant | | 2,39E-05 | [illegible] |
| F3 | transmembrane receptor | Activated | 2,43E-05 | [illegible] |
| ICAM1 | transmembrane receptor | Activated | 2,43E-05 | [illegible] |
| DCN | other | | 2,43E-05 | [illegible] |
| TO-901317 | chemical reagent | | 2,44E-05 | [illegible] |
| miR-138-5p (miRNAs w/seed GCUGGUG) | mature microRNA | Inhibited | 2,53E-05 | [illegible] |
| WBP2 | transcription regulator | | 2,54E-05 | [illegible] |
| ANKRD42 | transcription regulator | Activated | 2,54E-05 | [illegible] |
| patulin | chemical toxicant | | 2,54E-05 | [illegible] |
| poly(dA-dT) | chemical reagent | Activated | 2,54E-05 | [illegible] |
| TCIM | other | Activated | 2,54E-05 | [illegible] |
| TRIM8 | other | | 2,54E-05 | [illegible] |
| ADCYAP1R1 | G-protein coupled receptor | Activated | 2,54E-05 | [illegible] |
| Immunoglobulin Lambda Light Chain | group | | 2,54E-05 | [illegible] |
| dacarbazine | chemical drug | Activated | 2,54E-05 | [illegible] |
| manidipine | chemical drug | | 2,54E-05 | [illegible] |
| N-nitro-L-arginine methyl ester | chemical drug | | 2,54E-05 | [illegible] |
| NFE2L2 | transcription regulator | | 2,58E-05 | [illegible] |
| CCND1 | transcription regulator | | 2,57E-05 | [illegible] |
| PTPN11 | phosphatase | | 2,61E-05 | [illegible] |
| (+)-MK-801 | chemical drug | | 2,61E-05 | [illegible] |
| tert-butyl-hydroquinone | chemical reagent | Activated | 2,64E-05 | [illegible] |
| miR-144-3p (miRNAs w/seed ACAGUAU) | mature microRNA | Inhibited | 2,72E-05 | [illegible] |
| PPP2CA | phosphatase | | 2,73E-05 | [illegible] |
| TGFB3 | growth factor | Activated | 2,73E-05 | [illegible] |
| TET2 | enzyme | | 2,76E-05 | [illegible] |
| KITLG | growth factor | Activated | 2,82E-05 | [illegible] |
| NfkB1-RelA | complex | Activated | 2,83E-05 | [illegible] |
| MST1 | growth factor | | 2,83E-05 | [illegible] |
| MTA3 | transcription regulator | | 2,83E-05 | [illegible] |

| | | | | |
|---|---|---|---|---|
| PLK2 | kinase | Activated | 2.83E-06 | CD40,CITED2,CMPK2,CXCL10,EDN1,IFIT3,IFNB1,IL15,IL15RA,IL1B,IRF1,PLAT |
| linoleic acid | chemical - endogenous mammalian | | 2.83E-06 | [illegible],BAK1,EDN1,FASN,ICAM1,LEPR,NFKBIA,NOS3,SERPINE1,SNCA,TNF,UCP2 |
| TRPS1 | transcription regulator | | 2.84E-06 | BMP2,CCND2,CDC20,CDC25C,CENPF,ENPP1,FN1,PTTG1,RAMP1,RANBP3L,RUNX2,SERPIND1,SNAI1,TGFB2,TOP2A,TPX2,VDR,ZEB1 |
| miR-3082-3p (and other miRNAs w/seed ACAUGGC) | mature microRNA | Inhibited | 2.86E-06 | [illegible] |
| miR-3116 (miRNAs w/seed [illegible]) | mature microRNA | | 2.87E-06 | [illegible] |
| tempol | chemical drug | | 2.87E-06 | CAT,CXCL2,CXCL8,F3,GCH1,ICAM1,NOS3,PLAUR,PTGS2,RRAD,SERPINE1,SOD1,SOD2,SPP1,UCP2 |
| NR5A2 | ligand-dependent nuclear receptor | | 2.87E-06 | [illegible] |
| berberine | chemical drug | | 2.87E-06 | [illegible] |
| miR-1226-5p (and other miRNAs w/seed [illegible]) | mature microRNA | | 2.88E-06 | [illegible] |
| [illegible] | [illegible] | [illegible] | [illegible] | [illegible] |

| | | | | |
|---|---|---|---|---|
| CEBG5 | transcription regulator | | 1.73E-04 | ASAH1,CASP1,DDIT3,IL1B,TNF |
| RNU1-1 | other | Activated | 1.73E-04 | CXCL10,FGF2,HBEGF,IDO1,IFNB1 |
| FLT4 | transmembrane receptor | | 1.73E-04 | IL1B,IL6,NOS3,SOCS1,TNF |
| ZNF395 | other | Activated | 1.73E-04 | IFI16,IFI44,IFIT1,IFIT2,MEF2C |
| NLRP11 | other | Inhibited | 1.73E-04 | CXCL8,IFIT1,IFIT2,IFNB1,NFKB1 |
| GMFG | growth factor | | 1.73E-04 | IFNB1,IL6,ITGA5,ITGB1,TNF |
| STK38 | kinase | | 1.73E-04 | CCL20,CXCL2,CXCL3,IL6,TNF |
| XPA | other | | 1.73E-04 | FOS,KIN,PTGS1,PTGS2,TNF |
| coxsackievirus A21 | biologic drug | Activated | 1.73E-04 | CD274,CXCL10,CXCL11,CXCL9,DDX58 |
| EGTA acetoxymethyl ester | chemical reagent | Inhibited | 1.73E-04 | CXCL8,PTGS2,SERPINB2,TNF,VEGFA |
| 6,7-dinitroquinoxaline-2,3-dione | chemical reagent | Activated | 1.73E-04 | EGR1,FOS,FOSB,JUN,JUNB |
| pyridoxamine | chemical - endogenous mammalian | | 1.73E-04 | CRP,CXCL8,DDIT3,IL6,SERPINE1 |
| 6-cyano-7-nitroquinoxaline-2,3-dione | chemical reagent | Inhibited | 1.73E-04 | BDNF,EGR1,FOS,JUN,JUNB |
| PK11007 | chemical reagent | Activated | 1.73E-04 | BBC3,CDKN1A,DDIT3,PMAIP1,XBP1 |
| CV 3988 | chemical reagent | | 1.73E-04 | CCL2,ICAM1,IL6,ITGAV,ITGB1 |
| quisqualic acid | chemical - endogenous non-mammalian | | 1.73E-04 | BDNF,EGR1,FOS,JUNB,NTRK2 |
| miR-892b (miRNAs w/seed ACUGGCU) | mature microRNA | | 1.73E-04 | ACHE,ACSS2,[illegible] |
| SBDS | other | | 1.74E-04 | AKR1C1,AKR1C2,AKR1C3,CHAC1,CRYM,CTH,DDIT3,EGR1,FA2H,FOS,GABRE,GADD45B,HSPA5,HSPA6,IFI16,INHBE,MAFF,MAOA,MT2A,MYD88,PAK3,PPP1R15A,SPP1,TNC,TRIB3 |
| TAC1 | other | Activated | 1.74E-04 | CASP1,CCL2,CXCL10,CXCL3,CXCL8,FOS,FOSB,IFNB1,IL1A,IL1B,IL1RN,IL6,KITLG,MME,NFKB1,PLAT,PRKACA,PTGS2,SERPINB2,SERPINE1,SOX13,TNF,TNFRSF11B,TNFSF15 |
| doxycycline | chemical drug | Activated | 1.74E-04 | [illegible] |
| FCGR2B | transmembrane receptor | | 1.77E-04 | CCL5,CCND2,CD40,CXCL10,ICAM1,IFI27,IFNB1,IL1A,MX1,RASGRP1,SOCS1,SOCS3,TNF,VEGFA |
| miR-3551-5p (and other miRNAs w/seed CUUCACC) | mature microRNA | | 1.78E-04 | [illegible] |
| miR-4755-3p (miRNAs w/seed GCCAGGC) | mature microRNA | | 1.78E-04 | [illegible] |
| miR-6777-3p (miRNAs w/seed CCACUCU) | mature microRNA | Inhibited | 1.81E-04 | [illegible] |
| [illegible] | mature microRNA | Inhibited | 1.82E-04 | [illegible] |
| miR-6131 (miRNAs w/seed UGGAGAA) | mature microRNA | Inhibited | 1.82E-04 | [illegible] |
| [illegible] | mature microRNA | Inhibited | 1.82E-04 | [illegible] |
| [illegible] | chemical drug | Activated | 1.82E-04 | CD40,CDH2,CXCL8,ICAM1,IL1B,IL6,SPP1,TNF |
| [illegible] | chemical drug | | 1.82E-04 | BIRC2,BIRC3,BIRC5,CFLAR,DDIT3,PTGS2,TNFRSF10B,TRAF1 |
| SELENOS | other | | 1.82E-04 | CAV1,CEBPB,DDIT3,FASN,HSPA5,IL6,TNF,XBP1 |
| TCF/LEF | group | | 1.82E-04 | BIRC5,FN1,ITGB1,JUN,MYC,PTGS2,RUNX2,VIM |
| AIMP1 | cytokine | Activated | 1.82E-04 | CCL2,CCL5,CXCL2,CXCL8,ICAM1,IL12A,IL1B,TNF |
| [illegible] | transcription regulator | | 1.82E-04 | BBC3,BCL6,CDKN1A,FAS,MMP7,MYC,PDK1,TNFRSF10A |
| [illegible] | microRNA | | 1.82E-04 | [illegible] |
| INSIG2 | other | Activated | 1.82E-04 | FASN,FDFT1,FDPS,[illegible],HMGCR,INSIG1,SCD,SREBF2 |
| NRP1 | transmembrane receptor | | 1.82E-04 | ACTA2,CD40,HLA-DQB1,IL1B,IL6,MCL1,RCAN1,TNF |
| IL18R1 | transmembrane receptor | | 1.82E-04 | IL12A,IL1A,IL1B,IL6,SOCS1,SOCS3,STAT1,TNF |
| [illegible] | [illegible] | [illegible] | [illegible] | [illegible] |

| | | | | |
|---|---|---|---|---|
| IL6R | transmembrane receptor | | 6.42E-04 | AREG,BCL3,IL6,JUNB,MYC,SELL |
| LUM | other | Activated | 6.42E-04 | CDKN1A,CXCL2,FAS,IL1B,IL6,TNF |
| PRKRA | other | | 6.42E-04 | DCAF6,DICER1,IRF1,NKX3-1,PROS1,RET |
| Igkv1-117 | other | | 6.42E-04 | CDKN1A,FOSL2,JUN,JUNB,MYC,RELB |
| CXCL16 | cytokine | | 6.42E-04 | CCL2,CD68,CXCL8,FN1,SAA2,TNF |
| TRAF1 | other | | 6.42E-04 | BCL2L11,IFNB1,IL6,NFKB2,TNF,TRAF1 |
| COP1 | enzyme | | 6.42E-04 | CDKN1A,CEBPB,FAS,ICAM1,PPARGC1A,TRIB3 |
| pubchem compound 11368867 | chemical reagent | | 6.42E-04 | CXCL10,CXCL2,IL1B,IL23A,IL6,TNFSF13B |
| mastoparan | chemical toxicant | Inhibited | 6.42E-04 | CXCL10,IFNB1,IL12A,IL1B,IL6,TNF |
| poly(U)RNA | chemical reagent | | 6.42E-04 | CCL2,CXCL8,IFNB1,IL6,MX1,TNF |
| triamcinolone hexacetonide | chemical drug | | 6.42E-04 | HMGB1,ICAM1,IL1A,IL1B,TNF |
| coconut oil | chemical drug | | 6.42E-04 | CXCL3,MAP2,PTGS2,SREBF2,TNF |
| bis(3',5')-cyclic diguanylic acid | chemical - endogenous non-mammalian | Activated | 6.42E-04 | IFNB1,IL10,IL1RA,IL6,TNF |
| FIRRE | other | Activated | 6.42E-04 | CSF1,CSF3,IL1A,IL1B,TNF |
| CACNA1C | ion channel | | 6.42E-04 | ANXA2,CAV1,ENO1,PRKACA,PTGS2 |
| iguratimod | chemical drug | Inhibited | 6.42E-04 | CSF3,CXCL2,CXCL3,IL6,TNF |
| REV1 | enzyme | Inhibited | 6.42E-04 | BBC3,IL6,PMAIP1,SESN2,TNFRSF10B |
| GNE | kinase | Inhibited | 6.42E-04 | ASNS,CHAC1,DDIT3,JDP2,TRIB3 |
| TSC22D1 | transcription regulator | Activated | 6.42E-04 | CXCL8,FN1,IL1B,IL6,SERPINE1 |
| SOX4 | other | | 6.42E-04 | ANGPT2,IL1B,KLF2,KLF4,SOX9 |
| PTBP3 | other | | 6.42E-04 | CDH2,FN1,VIM,ZEB1,ZEB2 |
| MARCO | transmembrane receptor | | 6.42E-04 | CXCL3,IL1B,IL6,NFKB1,TNF |
| PROK1 | growth factor | | 6.42E-04 | CXCL8,IL11,MCL1,PTGS2,RCAN1 |
| ISG15 | other | Inhibited | 6.42E-04 | DDX58,IFI6,IFITM3,MX1,OAS1 |
| CLIC4 | ion channel | | 6.42E-04 | CCL5,CXCL2,IL1B,IL6,TNF |
| C4A/C4B | other | | 6.42E-04 | C4A/C4B,C5,CXCL10,IL1B,TNF |
| IL18RAP | transmembrane receptor | | 6.42E-04 | CXCL8,IL1A,IL32,IL6,PTGS2 |
| Pilge2os2 | other | Activated | 6.42E-04 | CCL5,CXCL10,IFNB1,PELI1,TRAF1 |
| 15-E2-isoketal modified phosphatidylethanolamine | chemical reagent | Activated | 6.42E-04 | CCL2,CXCL8,DDIT3,HSPA5,ICAM1 |
| AP20187 | chemical reagent | | 6.42E-04 | ATF4,BDNF,CEBPB,EGR1,FOS |
| thenoyltrifluoroacetone | chemical reagent | | 6.42E-04 | AGT,BIRC3,CXCL8,PDK1,SERPINE1 |
| perifosine | chemical drug | | 6.42E-04 | BAK1,CDKN1A,CFLAR,MCL1,TNFRSF10B |
| ubiquinone 9 | chemical - endogenous mammalian | Inhibited | 6.42E-04 | HSPA1A/HSPA1B,IL1A,IL1B,IL6,TNF |
| 4-coumaric acid | chemical - endogenous mammalian | | 6.42E-04 | CCNB1,CDKN1A,FGF2,MYC,PTGS2 |
| 2-mercaptoethanol | chemical - endogenous mammalian | | 6.42E-04 | ATF3,HERPUD1,HSPA5,IDO1,KIF3C |
| gliotoxin | chemical toxicant | | 6.42E-04 | BIRC3,BIRC5,CCL20,ISR3,TNF |
| RWJ-67657 | chemical - kinase inhibitor | Inhibited | 6.42E-04 | ACTA2,CXCL8,IL1B,PTGS2,TNF |
| borrelia burgdorferi strain B31 peptidoglycan | chemical reagent | Activated | 6.42E-04 | CXCL8,IL1A,IL1B,IL6,TNF |
| vinpocetine | chemical drug | Inhibited | 6.42E-04 | CXCL3,CXCL8,ICAM1,IL1B,TNF |
| miR-6729-3p (miRNAs w/seed CAUCCCC) | mature microRNA | Inhibited | 6.44E-04 | [illegible] |
| miR-1276 (miRNAs w/seed AGUACUG) | mature microRNA | Inhibited | 6.44E-04 | [illegible] |
| bevacizumab | biologic drug | | 6.44E-04 | ANGPT2,CDH2,DICER1,FN1,NRP1,VEGFA,VIM,ZEB1 |
| Foxo | group | Activated | 6.44E-04 | BCL2L11,CDKN1A,CDKN2B,GADD45A,IL7R,PMAIP1,RICTOR,TRIB3 |
| Integrin | complex | Activated | 6.44E-04 | AHR,FOS,JUN,MMP1,MYC,SOCS3,TGFB2,VEGFA |
| Angiotensin II receptor type 1 | group | Activated | 6.44E-04 | ATP1A1,CCL2,CXCL10,CXCL8,EDN1,NOS3,SERPINE1,VEGFA |
| [illegible] | [illegible] | [illegible] | [illegible] | [illegible] |

| | | | | |
|---|---|---|---|---|
| diallyl trisulfide | chemical - endogenous non-mammalian | | 1.26E-03 | ABCC4,CCNB1,CDC25C,CDKN1A,FAS,FOS,GSR,JUN,NFE2L2 |
| miR-5583-5p (and other miRNAs w/seed AACUAAU) | mature microRNA | | 1.26E-03 | [illegible] |
| miR-6513-5p (miRNAs w/seed UUGGGAU) | mature microRNA | | 1.26E-03 | [illegible] |
| miR-6420 (miRNAs w/seed GUUGGGA) | mature microRNA | | 1.27E-03 | [illegible] |
| miR-6726-5p (and other miRNAs w/seed UGGGCCC) | mature microRNA | | 1.28E-03 | [illegible] |
| miR-5193 (miRNAs w/seed CCUCCUC) | mature microRNA | Inhibited | 1.28E-03 | [illegible] |
| miR-190a-5p (and other miRNAs w/seed GAUAUGU) | mature microRNA | Inhibited | 1.29E-03 | [illegible] |
| CST5 | other | | 1.29E-03 | [illegible] |
| miR-5100-5p (miRNAs w/seed UCUGGAG) | mature microRNA | | 1.29E-03 | [illegible] |
| fingolimod phosphate | chemical - endogenous mammalian | | 1.29E-03 | CCL2,ERN1,HAS2,IL6,SERPINE1 |
| Cyp4a14 | enzyme | Activated | 1.29E-03 | CXCL10,CXCL3,IL1A,IL1B,TNF |
| SENP7 | peptidase | Inhibited | 1.29E-03 | BIRC2,CDH2,CXCL8,DHFR,VIM |
| Tlr13 | other | Activated | 1.29E-03 | CXCL8,IL1B,IL6,NFKB1,TNF |
| S100a7a | other | | 1.29E-03 | CXCL1,CXCL8,IL1,IL1A,MKI67 |
| HS6ST1 | enzyme | Activated | 1.29E-03 | CXCL8,FGF2,IL6,SPRY2,VEGFA |
| FALEC | other | | 1.29E-03 | BTG2,CDKN1A,CYFIP2,FAS,PIDD1 |
| LPA | other | | 1.29E-03 | CXCL8,ICAM1,NR2C1,SERPINB2,SERPINE1 |
| ATF7 | transcription regulator | Activated | 1.29E-03 | DDIT3,DUSP1,DUSP10,DUSP6,DUSP8 |
| TAPBP | transporter | | 1.29E-03 | HLA-A,HLA-B,HLA-E,TAP1,TAP2 |
| RBCK1 | transcription regulator | | 1.29E-03 | ATP1A1,CCNB1,ICAM1,MYC,TNFAIP3 |
| kurasoponin | chemical - endogenous non-mammalian | Inhibited | 1.29E-03 | IL1B,IL6,NFKBIA,SOCS3,TNF |
| Dusb1 (includes others) | other | Activated | 1.29E-03 | OAS1,OAS2,OAS3,OASL,RNASEL |
| ULBP1 | transmembrane receptor | | 1.29E-03 | CD69,CD83,HLA-A,TNF,TNFRSF9 |
| CDTI-2 | chemical drug | | 1.29E-03 | CCNB1,CCND3,MCL1,PLK1,PRKACA |
| raclopride | chemical drug | | 1.29E-03 | BDNF,FOS,NR4A1,NR4A2,NR4A3 |
| vadimezan | chemical drug | Activated | 1.29E-03 | CCL5,CXCL10,IFNB1,NFKB1,TNF |
| mechlorethamine | chemical drug | Activated | 1.29E-03 | ATF3,CD40,IL1B,NFE2L2,PIK3R3 |
| domoic acid | chemical toxicant | Activated | 1.29E-03 | CEBPB,FOS,IL1B,PTGS2,TNF |
| latrunculin A | chemical toxicant | | 1.29E-03 | ACTA2,DSTN,HAS2,IL6,TNF |
| magnesium sulfate | chemical drug | | 1.29E-03 | ADM,HMGB1,IL6,NFKBIA,TNF |
| levofloxacin | chemical drug | | 1.29E-03 | CDKN1A,CXCL8,HAS2,IL1A,IL6 |
| alpha-tocopherol succinate | chemical drug | Inhibited | 1.29E-03 | EGR1,FGF2,IL1A,TNF,VEGFA |
| TBP | transcription regulator | | 1.30E-03 | ASNS,BDP1,CDKN1A,EDNRA,ETS2,FN1,FOS,GATA4,HLA-A,HMGCR,ID3,IGF2,MYC,NFKBIA,PTGS2,TNF,TNFAIP3,XDH |
| monophosphoryl lipid A | chemical drug | Activated | 1.31E-03 | CXCL10,IFNB1,IL1A,IL1B,IL1RN,IL6,TNF |
| amphotericin B | chemical drug | | 1.31E-03 | CCL2,CXCL8,IL1B,IL1RN,IL6,SPP1,TNF |
| cetuximab | biologic drug | | 1.31E-03 | BIRC3,BNIP3L,FN1,PLAUR,TNFRSF10A,TNFRSF10B,XBP1 |
| Nos | group | | 1.31E-03 | ADH5,ARC,BDNF,EGR1,FOS,GCH1,VEGFA |
| 1'-acetoxychavicol acetate | chemical reagent | | 1.31E-03 | BIRC2,BIRC3,BIRC5,CFLAR,ICAM1,MYC,PTGS2 |
| IFNL2 | other | Activated | 1.31E-03 | CXCL10,IFIT1,IFNL1,IFNL2,IRF7,MX1,RSAD2 |
| FOXO1 | transcription regulator | | 1.31E-03 | BCL2L11,CDKN1A,CDKN2B,DLX2,FST,NCOA3,SERPINE1 |
| Ifit | complex | Activated | 1.31E-03 | ATF4,CDK11A,DDIT3,HSPA5,MYC,PPP1R15A,VEGFA |
| AZD8055 | chemical drug | | 1.31E-03 | BCL2L11,CCL5,CXCL10,FOXO1,MCL1,SNAI1,TNF |
| SMC3 | other | Activated | 1.31E-03 | HLA-DQA1,HLA-DQB1,HLA-DRA,HLA-DRB1,IRS2,MYC,SOX4 |
| CAPN3 | peptidase | Activated | 1.31E-03 | CCL5,DUSP1,GFI1,GSTZ1,NCF2,PTGS2,SOD2 |
| BDKRB2 | G-protein coupled receptor | | 1.31E-03 | EDN1,FOXO1,ICAM1,ITGAM,NOS3,PTGS2,SNCA |

[illegible]

| | | | | |
|---|---|---|---|---|
| miR-7155-5p (miRNAs w/seed CUGGGGU) | mature microRNA | Inhibited | 2.63E-03 | [illegible] |
| miR-6813-3p (miRNAs w/seed ACCUUGG) | mature microRNA | | 2.63E-03 | [illegible] |
| NFATC3 | transcription regulator | Activated | 2.64E-03 | [illegible] |
| FLT3LG | cytokine | | 2.64E-03 | [illegible] |
| miR-3666-3p (miRNAs w/seed CUCAGGA) | mature microRNA | | 2.65E-03 | [illegible] |
| miR-1260a (and other miRNAs w/seed UCCCACC) | mature microRNA | Inhibited | 2.67E-03 | [illegible] |
| miR-10400-3p (and other miRNAs w/seed UGGGCUC) | mature microRNA | | 2.69E-03 | [illegible] |
| ERBB4 | kinase | | 2.73E-03 | [illegible] |
| MOG | other | | 2.73E-03 | [illegible] |
| VEGFB | growth factor | | 2.73E-03 | [illegible] |
| FGF10 | growth factor | | 2.73E-03 | [illegible] |
| EHMT2 | transcription regulator | | 2.73E-03 | [illegible] |
| miR-8064 (miRNAs w/seed GCACACU) | mature microRNA | Inhibited | 2.73E-03 | [illegible] |
| miR-1301-3p (and other miRNAs w/seed UGCAGCU) | mature microRNA | Inhibited | 2.75E-03 | [illegible] |
| miR-6540 (miRNAs w/seed UAGUCCU) | mature microRNA | | 2.75E-03 | [illegible] |
| miR-5006-3p (miRNAs w/seed UUGGAUU) | mature microRNA | Inhibited | 2.75E-03 | [illegible] |
| miR-147 (and other miRNAs w/seed UGUGCGG) | mature microRNA | | 2.75E-03 | [illegible] |
| miR-10a-5p (and other miRNAs w/seed ACCCUGU) | mature microRNA | Inhibited | 2.76E-03 | [illegible] |
| miR-3065-3p (miRNAs w/seed CAGCACC) | mature microRNA | Inhibited | 2.76E-03 | [illegible] |
| miR-6822-3p (miRNAs w/seed GGCUCUA) | mature microRNA | Inhibited | 2.76E-03 | [illegible] |
| miR-6868b (miRNAs w/seed UGCAUGU) | mature microRNA | | 2.78E-03 | [illegible] |
| miR-690-5p (miRNAs w/seed CAUGGAU) | mature microRNA | Inhibited | 2.79E-03 | [illegible] |
| mir-135 | microRNA | | 2.79E-03 | [illegible] |
| cyclophosphamide | chemical drug | | 2.84E-03 | [illegible] |
| GSK J4 | chemical reagent | | 2.84E-03 | [illegible] |
| miR-873-5p (and other miRNAs w/seed CAGGAAC) | mature microRNA | | 2.86E-03 | [illegible] |
| MYCN | transcription regulator | Inhibited | 2.86E-03 | [illegible] |
| Ras homolog | group | | 2.86E-03 | [illegible] |
| Aldose Reductase | group | Activated | 2.86E-03 | [illegible] |
| SIGIRR | transmembrane receptor | Inhibited | 2.86E-03 | [illegible] |
| PARG | enzyme | | 2.86E-03 | [illegible] |
| MED12 | transcription regulator | | 2.86E-03 | [illegible] |
| SPZ1 | transcription regulator | | 2.86E-03 | [illegible] |
| SP100 | transcription regulator | | 2.86E-03 | [illegible] |
| IDO1 | enzyme | | 2.86E-03 | [illegible] |
| SMARCE1 | transcription regulator | | 2.86E-03 | [illegible] |
| RNASE1 | enzyme | Activated | 2.86E-03 | [illegible] |
| CCL4 | cytokine | Activated | 2.86E-03 | [illegible] |
| ADORA3 | G-protein coupled receptor | | 2.86E-03 | [illegible] |
| 2-mercaptoacetate | chemical drug | | 2.86E-03 | [illegible] |
| [illegible] | chemical drug | | 2.86E-03 | [illegible] |
| W7 | chemical reagent | Inhibited | 2.86E-03 | [illegible] |
| [illegible] | chemical - endogenous mammalian | Inhibited | 2.86E-03 | [illegible] |
| [illegible] | chemical drug | | 2.86E-03 | [illegible] |
| miR-3565 (miRNAs w/seed GGGCAUA) | mature microRNA | Inhibited | 2.86E-03 | [illegible] |
| miR-3622b-5p (miRNAs w/seed GGCAUGG) | mature microRNA | | 2.86E-03 | [illegible] |
| fluocinolone acetonide | chemical drug | | 2.86E-03 | [illegible] |
| fish oils | chemical drug | | 2.86E-03 | [illegible] |
| KAT2B | transcription regulator | Activated | 2.86E-03 | [illegible] |
| hydroxyurea | chemical drug | Activated | 2.86E-03 | [illegible] |
| tilamilerone | chemical drug | | 2.86E-03 | [illegible] |
| miR-4759 (miRNAs w/seed AGGACUA) | mature microRNA | Inhibited | 2.89E-03 | [illegible] |
| miR-2052 (miRNAs w/seed GUUUUGA) | mature microRNA | Inhibited | 2.89E-03 | [illegible] |
| FOXA1 | transcription regulator | | 2.89E-03 | [illegible] |
| miR-101a-3p (miRNAs w/seed UACAGUA) | mature microRNA | Inhibited | 2.91E-03 | [illegible] |
| miR-4480 (miRNAs w/seed GCCAAGU) | mature microRNA | Inhibited | 2.92E-03 | [illegible] |
| pexidartinib | chemical drug | | 2.93E-03 | [illegible] |
| miR-6886-3p (miRNAs w/seed GUCUCUC) | mature microRNA | Inhibited | 2.95E-03 | [illegible] |
| [illegible] | [illegible] | [illegible] | [illegible] | [illegible] |

| | | | | |
|---|---|---|---|---|
| miR-544-5p (miRNAs w/seed CUUGUUA) | mature microRNA | | 5.48E-03 | [illegible] |
| miR-6717-5p (miRNAs w/seed GGCGAUG) | mature microRNA | | 5.48E-03 | [illegible] |
| miR-7064-3p (and other miRNAs w/seed AGGGCCC) | mature microRNA | Inhibited | 5.51E-03 | [illegible] |
| clofibrate | chemical drug | | 5.53E-03 | [illegible] |
| miR-6512 (miRNAs w/seed AGGGCCU) | mature microRNA | Inhibited | 5.54E-03 | [illegible] |
| miR-6263 (miRNAs w/seed AGCCUGA) | mature microRNA | | 5.57E-03 | [illegible] |
| miR-593-3p (miRNAs w/seed GUCUCUG) | mature microRNA | Inhibited | 5.58E-03 | [illegible] |
| miR-30c-3p (and other miRNAs w/seed UGGGAUG) | mature microRNA | Inhibited | 5.61E-03 | [illegible] |
| PTK2 | kinase | Activated | 5.62E-03 | [illegible] |
| pravastatin | chemical drug | | 5.62E-03 | [illegible] |
| miR-532-5p (and other miRNAs w/seed AUGCCUU) | mature microRNA | Inhibited | 5.64E-03 | [illegible] |
| miR-3678-5p (miRNAs w/seed CCGUACA) | mature microRNA | | 5.66E-03 | [illegible] |
| miR-6800-5p (miRNAs w/seed UAGGUGA) | mature microRNA | | 5.70E-03 | [illegible] |
| miR-6791-3p (and other miRNAs w/seed GCCUCCU) | mature microRNA | | 5.70E-03 | [illegible] |
| miR-6508a-5p (and other miRNAs w/seed UGCCAUG) | mature microRNA | | 5.71E-03 | [illegible] |
| miR-4708-5p (miRNAs w/seed GAGAUGC) | mature microRNA | Inhibited | 5.72E-03 | [illegible] |
| miR-374c-3p (miRNAs w/seed ACUUAGC) | mature microRNA | Inhibited | 5.74E-03 | [illegible] |
| EED | transcription regulator | Inhibited | 5.74E-03 | [illegible] |
| pituitary adenylate cyclase-activating polypeptide | biologic drug | | 5.74E-03 | [illegible] |
| miR-589-5p (and other miRNAs w/seed GAGAACC) | mature microRNA | | 5.80E-03 | [illegible] |
| miR-7848-3p (miRNAs w/seed UACCCUC) | mature microRNA | Inhibited | 5.85E-03 | [illegible] |
| miR-3072 (and other miRNAs w/seed GCCCCCU) | mature microRNA | Inhibited | 5.87E-03 | [illegible] |
| collagen type I (family) | group | | 5.90E-03 | [illegible] |
| choline fenofibrate | chemical drug | | 5.90E-03 | [illegible] |
| GRN | growth factor | | 5.90E-03 | [illegible] |
| imipramine | chemical drug | | 5.90E-03 | [illegible] |
| miR-888-3p (miRNAs w/seed ACUGACA) | mature microRNA | Inhibited | 5.91E-03 | [illegible] |
| miR-3925-5p (miRNAs w/seed AGAGAAC) | mature microRNA | | 5.94E-03 | [illegible] |
| KDM2B | enzyme | | 5.96E-03 | [illegible] |
| GNRH | group | Activated | 5.96E-03 | [illegible] |
| IPT88 | other | Activated | 5.96E-03 | [illegible] |
| TSLP | cytokine | | 5.96E-03 | [illegible] |
| ethylene glycol tetraacetic acid | chemical reagent | Inhibited | 5.96E-03 | [illegible] |
| miR-509-3p (miRNAs w/seed GAUUGGU) | mature microRNA | Inhibited | 5.97E-03 | [illegible] |
| miR-6818-3p (miRNAs w/seed UGUCUCU) | mature microRNA | Inhibited | 5.99E-03 | [illegible] |
| miR-4787-5p (and other miRNAs w/seed CGGGGGU) | mature microRNA | | 6.00E-03 | [illegible] |
| miR-4744 (miRNAs w/seed CUAAAGA) | mature microRNA | | 6.01E-03 | [illegible] |
| miR-6084 (miRNAs w/seed UCCGCCA) | mature microRNA | | 6.01E-03 | [illegible] |
| LRP6 | transmembrane receptor | Inhibited | 6.04E-03 | [illegible] |
| honokiol | chemical - endogenous non-mammalian | | 6.04E-03 | [illegible] |
| PDCD1 | phosphatase | | 6.05E-03 | [illegible] |
| SNAI2 | transcription regulator | | 6.08E-03 | [illegible] |
| miR-2114-3p (and other miRNAs w/seed GAGCCUC) | mature microRNA | | 6.08E-03 | [illegible] |
| NKX3-1 | transcription regulator | | 6.10E-03 | [illegible] |
| miR-3922-5p (miRNAs w/seed CAAGGCC) | mature microRNA | | 6.15E-03 | [illegible] |
| CPD1 | enzyme | | 6.20E-03 | [illegible] |
| miR-6300 (and other miRNAs w/seed GGGAGCU) | mature microRNA | Inhibited | 6.21E-03 | [illegible] |
| miR-185-5p (and other miRNAs w/seed GGAGAGA) | mature microRNA | | 6.22E-03 | [illegible] |
| miR-3165 (miRNAs w/seed AUGCUAG) | mature microRNA | | 6.23E-03 | [illegible] |
| miR-760-3p (and other miRNAs w/seed GGCUCUG) | mature microRNA | Inhibited | 6.29E-03 | [illegible] |
| miR-4794 (and other miRNAs w/seed CUGGCUA) | mature microRNA | | 6.32E-03 | [illegible] |
| gemfibrozil | chemical drug | | 6.38E-03 | [illegible] |
| miR-4169-3p (miRNAs w/seed GCCCCUC) | mature microRNA | | 6.43E-03 | [illegible] |
| miR-5008-3p (and other miRNAs w/seed CUGUGCU) | mature microRNA | Inhibited | 6.44E-03 | [illegible] |
| miR-4318 (miRNAs w/seed ACUGUGG) | mature microRNA | Inhibited | 6.46E-03 | [illegible] |
| prostaglandin E1 | chemical - endogenous mammalian | | 6.47E-03 | [illegible] |
| artesunic acid | chemical drug | | 6.47E-03 | [illegible] |
| SLC29A1 | transporter | | 6.47E-03 | [illegible] |
| ITGB4 | transmembrane receptor | Activated | 6.47E-03 | [illegible] |
| MNT | transcription regulator | Inhibited | 6.47E-03 | [illegible] |
| HNRNPU | transporter | | 6.47E-03 | [illegible] |
| CLEC4E | other | Activated | 6.47E-03 | [illegible] |
| IL1RL1 | transmembrane receptor | | 6.47E-03 | [illegible] |
| NRTN | growth factor | | 6.47E-03 | [illegible] |
| CEP-1347 | chemical drug | | 6.47E-03 | [illegible] |
| triflusal | chemical drug | | 6.47E-03 | [illegible] |
| pirfenidone | chemical drug | | 6.47E-03 | [illegible] |
| diethylmaleate | chemical toxicant | | 6.47E-03 | [illegible] |
| Cpd X | chemical reagent | | 6.47E-03 | [illegible] |
| biotin | chemical drug | | 6.50E-03 | FADS1,FADS2,FASN,MMP1 |
| alloxan | chemical reagent | | 6.50E-03 | CCL2,CXCL8,ICAM1,TNF |
| pervanadate | chemical reagent | | 6.50E-03 | IRF1,MX1,NFKBIA,PSMB9 |
| flavokawain B | chemical - endogenous non-mammalian | | 6.50E-03 | [illegible] |
| sesamol | chemical - endogenous mammalian | Inhibited | 6.50E-03 | CD68,IL6,MMP1,TNF |
| nicotinic acetylcholine receptor | complex | | 6.50E-03 | FOS,JUN,JUNB,PNMT |
| Tir11 | transmembrane receptor | Activated | 6.50E-03 | CXCL8,IL1B,IL6,TNF |
| PDCD10 | other | Inhibited | 6.50E-03 | KLF2,KLF4,PROCR,VEGFA |
| USP25 | peptidase | | 6.50E-03 | IFNB1,IL6,TNF,USP25 |
| ST6GALNAC1 | enzyme | | 6.50E-03 | B2M,CDH2,SNAI1,VIM |
| TRIM29 | transcription regulator | | 6.50E-03 | CDKN1A,PMAIP1,SNAI1,ZEB1 |
| BCAR4 | other | | 6.50E-03 | CDH2,HK2,PFKFB3,VIM |
| SLC5A8 | transporter | | 6.50E-03 | [illegible] |
| TP73-AS1 | other | | 6.50E-03 | HMGB1,IL1B,IL6,TNF |
| FNIP1 | other | | 6.50E-03 | [illegible] |
| ZC3HAV1 | other | | 6.50E-03 | CXCL10,IFNA1/IFNA13,IL6,TNF |
| APC (complex) | complex | | 6.50E-03 | CCNB1,PLK1,TRIB3,UBE2C |
| sphingomyelinase | group | | 6.50E-03 | JUN,MMP1,MYC,PTGS2 |
| lefunomide | chemical drug | | 6.50E-03 | IFIT3,IL6,ISG15,TNF |
| HLA-DQ | complex | | 6.50E-03 | FOS,IL1B,IL6,TNF |
| ASCL2 | transcription regulator | | 6.50E-03 | CCNB1,CTSD,CXCL10,EGR1 |
| SERPINC1 | enzyme | | 6.50E-03 | F3,IL6,MAPK3,TNF |
| MEFV | other | | 6.50E-03 | CASP1,CXCL8,IL1B,LYZ |
| IFNGR | complex | | 6.50E-03 | FN1,IRF1,SOCS3,TNF |
| HMGCR | enzyme | | 6.50E-03 | BIRC5,HMGCR,IL1B,TAZ |
| SERPINA4 | other | | 6.50E-03 | BCL2L11,CCL2,MYC,NOS3 |
| mir-320 | microRNA | | 6.50E-03 | AQP4,BIRC5,MCL1,TFRC |
| PIK3C3 | kinase | | 6.50E-03 | CD40,HLA-A,HLA-DQB1,IGF2 |
| LPAR2 | G-protein coupled receptor | | 6.50E-03 | CXCL8,FN1,IL6,PTGS2 |
| CUL7 | enzyme | | 6.50E-03 | BIRC5,CDH2,VIM,ZEB1 |
| PPM1A | phosphatase | | 6.50E-03 | CDKN1A,ITGA5,ITGAV,ITGB1 |
| MLLT3 | transcription regulator | | 6.50E-03 | EDN1,MYC,SCNN1A,SGK1 |
| PLA2G2A | enzyme | | 6.50E-03 | CD69,CXCL8,IL6,PTGS2 |
| MED13L | other | | 6.50E-03 | ANKRD1,KITLG,LIF,MYC |
| cardiolin | chemical - endogenous non-mammalian | | 6.50E-03 | BIRC5,CFLAR,IL1B,TNFRSF10B |
| KLK81 | peptidase | | 6.50E-03 | F3,ICAM1,KLF4,SIRT1 |
| USP21 | peptidase | | 6.50E-03 | GATA3,IFNB1,IL6,ISG15 |
| HLA-B | transmembrane receptor | | 6.50E-03 | EDN1,IL6,OAS2,XBP1 |
| F2RL3 | other | | 6.50E-03 | CXCL2,IL1B,IL6,TNF |
| BP-1-102 | chemical reagent | Inhibited | 6.50E-03 | IL1RN,IL6,NFIL3,TNF |
| benzoquinone | chemical - endogenous mammalian | | 6.50E-03 | APOL1,GBP1,IRF1,TNF |
| fluphenazine | chemical drug | | 6.50E-03 | FOS,NR4A1,NR4A2,NR4A3 |
| tomatosmin | chemical reagent | | 6.50E-03 | IL1B,MYD88,PTGS2,TNF |
| ISRIB | chemical reagent | Inhibited | 6.50E-03 | ATF4,CCL20,IL6,PMAIP1 |
| diazepam | chemical drug | | 6.50E-03 | CXCL8,FOS,NTRK2,PER1 |
| eugenol | chemical - endogenous non-mammalian | | 6.50E-03 | CDKN1A,CXCL8,IL6,PTGS2 |
| retroinverso ERG inhibitory peptide 2 | chemical reagent | | 6.50E-03 | [illegible] |
| retroinverso ERG inhibitory peptide 1 | chemical reagent | | 6.50E-03 | [illegible] |
| dBET6 | chemical reagent | | 6.50E-03 | ARL4C,DUSP4,IRX3,PDE4B |
| miR-506-3p inhibitor | chemical reagent | | 6.50E-03 | SERPINE1,SNAI1,VIM,ZEB1 |
| miR-545-5p (miRNAs w/seed CAGUAAA) | mature microRNA | Inhibited | 6.56E-03 | [illegible] |
| LTBR | transmembrane receptor | | 6.57E-03 | [illegible] |
| miR-1296-3p (and other miRNAs w/seed AGUGGGG) | mature microRNA | Inhibited | 6.58E-03 | [illegible] |
| miR-296-3p (miRNAs w/seed AGGGUUG) | mature microRNA | Inhibited | 6.62E-03 | [illegible] |
| miR-218-2-3p (and other miRNAs w/seed AUGGUUC) | mature microRNA | Inhibited | 6.62E-03 | [illegible] |
| RICTOR | other | Activated | 6.70E-03 | [illegible] |
| miR-3120 (and other miRNAs w/seed ACAGCAA) | mature microRNA | Inhibited | 6.71E-03 | [illegible] |
| miR-3680 (miRNAs w/seed CCUGGAC) | mature microRNA | | 6.79E-03 | [illegible] |
| miR-6881-3p (miRNAs w/seed UCCUCUU) | mature microRNA | Inhibited | 6.84E-03 | [illegible] |
| miR-1-5p (and other miRNAs w/seed CAUACUU) | mature microRNA | Inhibited | 6.85E-03 | [illegible] |
| miR-5189-3p (and other miRNAs w/seed GCCAACC) | mature microRNA | Inhibited | 6.86E-03 | [illegible] |
| miR-136-5p (miRNAs w/seed CUCCAUU) | mature microRNA | Inhibited | 6.90E-03 | [illegible] |
| miR-4477b (miRNAs w/seed UUAAGGA) | mature microRNA | | 6.91E-03 | [illegible] |
| PTP4A1 | phosphatase | | 6.94E-03 | [illegible] |
| miR-383-5p (miRNAs w/seed AGAUCAG) | mature microRNA | | 6.96E-03 | [illegible] |
| miR-3934-3p (miRNAs w/seed GCUCAGG) | mature microRNA | | 6.98E-03 | [illegible] |
| ANGPT1 | growth factor | | 7.01E-03 | [illegible] |
| TXNRD1 | enzyme | | 7.01E-03 | [illegible] |
| CDK2 | kinase | | 7.01E-03 | [illegible] |
| TAF4 | transcription regulator | | 7.04E-03 | [illegible] |
| mir-210 | microRNA | | 7.04E-03 | [illegible] |
| CCN2 | growth factor | Activated | 7.06E-03 | [illegible] |
| methylmercury | chemical toxicant | | 7.07E-03 | [illegible] |
| miR-3618 (miRNAs w/seed GUCUACA) | mature microRNA | Inhibited | 7.13E-03 | [illegible] |
| miR-3912-5p (miRNAs w/seed UGUCCAU) | mature microRNA | Inhibited | 7.15E-03 | [illegible] |
| miR-5580-5p (and other miRNAs w/seed GCUGGCU) | mature microRNA | Inhibited | 7.15E-03 | [illegible] |
| miR-495-3p (and other miRNAs w/seed UCAUACA) | mature microRNA | Inhibited | 7.16E-03 | [illegible] |
| miR-3141 (miRNAs w/seed AGGGCGG) | mature microRNA | | 7.16E-03 | [illegible] |
| miR-548ao-5p (and other miRNAs w/seed GAAGUAA) | mature microRNA | Inhibited | 7.23E-03 | [illegible] |
| miR-4664-3p (miRNAs w/seed GUUGCAU) | mature microRNA | Inhibited | 7.24E-03 | [illegible] |
| miR-3605-5p (and other miRNAs w/seed GAGGAUG) | mature microRNA | | 7.24E-03 | [illegible] |
| carboplatin | chemical drug | | 7.26E-03 | [illegible] |
| miR-1302 (and other miRNAs w/seed UGCCAGC) | mature microRNA | Inhibited | 7.26E-03 | [illegible] |
| miR-4515 (miRNAs w/seed GGACUGG) | mature microRNA | Inhibited | 7.26E-03 | [illegible] |
| Cbp | group | | 7.30E-03 | [illegible] |
| JUN/JUNB/JUND | group | | 7.30E-03 | [illegible] |
| RNF20 | enzyme | | 7.30E-03 | [illegible] |
| LINC00963 | other | Activated | 7.30E-03 | [illegible] |
| WNT7A | cytokine | | 7.30E-03 | [illegible] |
| SAFB2 | other | Inhibited | 7.30E-03 | [illegible] |
| USP18 | peptidase | | 7.30E-03 | [illegible] |
| ACE | peptidase | | 7.30E-03 | [illegible] |
| 1-methyl-4-phenylpyridinium | chemical toxicant | | 7.30E-03 | [illegible] |
| diosgenin | chemical - endogenous non-mammalian | | 7.30E-03 | [illegible] |
| 3-O-glucosyl-1,2-diacylglycerol | chemical - endogenous non-mammalian | | 7.32E-03 | CXCL3,IL6,TNF |
| behenic acid | chemical - endogenous mammalian | | 7.32E-03 | IL1B,IL6,TNF |
| treprostinil | chemical drug | | 7.32E-03 | IL1B,IL6,TNF |
| ITPR | group | | 7.32E-03 | CYP1B1,IL1B,MAPK3 |
| chitinase | group | | 7.32E-03 | CCL2,CCL8,CXCL8 |
| tylophorine | chemical drug | | 7.32E-03 | JUN,PTGS2,TNF |
| nebivolol | chemical drug | | 7.32E-03 | NOS3,PTGS1,PTGS2 |
| CACTIN | other | | 7.32E-03 | CCL5,CXCL8,TNF |
| PFKFB4 | kinase | | 7.32E-03 | NCOA3,TKT,XDH |
| PDE8A | enzyme | | 7.32E-03 | CYP51A1,FDPS,HMGCR |
| ZXDC | transcription regulator | | 7.32E-03 | CCL2,HLA-DRA,ITGAM |
| ROBO3 | transmembrane receptor | | 7.32E-03 | IFNB1,IL6,IRF8 |
| TNIP3 | other | | 7.32E-03 | CXCL8,IL6,TNF |
| USP28 | peptidase | | 7.32E-03 | CDKN1A,GATA4,PLK3 |
| UBE1 | other | | 7.32E-03 | DDIT3,HSPA5,XBP1 |
| ATP6V0D2 | transporter | | 7.32E-03 | IFNB1,IL6,TNF |
| ABRAXAS2 | other | | 7.32E-03 | CASP1,IL1B,JAK2 |
| LINC00657 | other | | 7.32E-03 | CDH2,VIM,ZEB1 |
| B3GNT2 | enzyme | | 7.32E-03 | IL1B,IL6,TNF |
| ZFP64 | transcription regulator | | 7.32E-03 | IFNB1,IL6,TNF |
| CIRBP | translation regulator | | 7.32E-03 | CXCL2,CXCL3,IL6 |
| RNF122 | enzyme | | 7.32E-03 | IFNB1,IL6,TNF |
| EHD2 | other | | 7.32E-03 | CAV1,CAV2,CAVIN1 |
| CUBDC2 | other | | 7.32E-03 | LDHA,NR3C1,SLC2A3 |
| LRRC19 | other | | 7.32E-03 | CXCL8,IL6,TNF |
| Eif2 | complex | | 7.32E-03 | EIF2AK2,HSPA5,PTGS2 |
| P-TEFb | complex | | 7.32E-03 | BBC3,CDKN1A,CXCL8 |
| nintedanib | chemical drug | | 7.32E-03 | DDIT3,FN1,RND3 |
| danusertib | chemical drug | | 7.32E-03 | CCNB1,CDKN1A,GADD45A |
| RAB7 | group | | 7.32E-03 | CXCL10,IFNB1,MYC |
| EIF3I | translation regulator | | 7.32E-03 | MYC,PTGS2,SERPINE1 |
| cerotic acid | chemical - endogenous mammalian | | 7.32E-03 | CD40,IL1A,IL1B |
| PF-4691502 | chemical drug | | 7.32E-03 | BBC3,PMAIP1,VIM |
| orexin | chemical drug | | 7.32E-03 | HSPA5,TNF,XBP1 |
| ORM1 | other | | 7.32E-03 | IL1RN,IL6,TNF |
| MEP1A | peptidase | | 7.32E-03 | CXCL8,IL1B,IL6 |
| HSPB6 | other | | 7.32E-03 | CCL2,CXCL8,ICAM1 |
| BAG2 | other | | 7.32E-03 | IL1B,IL6,TNF |
| IL17B | cytokine | | 7.32E-03 | CDKN1A,CSF3,IL6 |
| A4GALT | enzyme | | 7.32E-03 | IL6,IRF1,TNF |
| CD163 | transmembrane receptor | | 7.32E-03 | IL1B,IL6,TNF |
| RNF139 | enzyme | | 7.32E-03 | FASN,FDFT1,HMGCR |
| APOD | transporter | | 7.32E-03 | IL1B,IL6,TNF |
| NLRC3 | other | | 7.32E-03 | IL1B,IL6,TNF |
| PLAGL2 | transcription regulator | | 7.32E-03 | IGF2,LDHA,NCF2 |
| Ripk3 | kinase | | 7.32E-03 | IL1B,IL6,TNF |
| WARS1 | enzyme | | 7.32E-03 | FGF2,IL6,NFKBIA |
| SKL8023 | chemical reagent | | 7.32E-03 | IL1B,PTGS2,TNF |
| VSNL1 | other | | 7.32E-03 | CREB1,ITGA5,ITGAV |
| CFL1 | other | | 7.32E-03 | DSTN,ICAM1,JUN |
| ERO1A | enzyme | | 7.32E-03 | CSF3,CXCL2,CXCL3 |
| CST8 | peptidase | | 7.32E-03 | CASP4,CEBPB,IL1B |
| HSPH1 | other | | 7.32E-03 | BCL6,MYC,SNCA |
| PLLL-32 | chemical reagent | | 7.32E-03 | CCL2,CCL22,IL6 |
| NEK2 | kinase | | 7.32E-03 | CDKN1A,FAS,GADD45A |
| PPP1CC | phosphatase | | 7.32E-03 | BDNF,IL6,TNF |
| HS6ST2 | enzyme | | 7.32E-03 | CXCL8,FGF2,IL6 |
| MAD1L1 | other | | 7.32E-03 | MXD3,MXI1,MYC |
| CAY10397 | chemical reagent | | 7.32E-03 | IL1B,PTGS2,TNF |
| N-hydroxy-2,2-diphenylacetamide | chemical reagent | | 7.32E-03 | EDN1,IL6,TNF |
| (+)-epicatechin | chemical drug | | 7.32E-03 | IL1B,PTGS2,TNF |
| GSU-C012 | chemical reagent | | 7.32E-03 | HSPA5,KLF6,SIRT1 |
| 1-(2-hydroxy-5-methylphenyl)-3-phenyl-1,3-propanedione | chemical reagent | | 7.32E-03 | CEBPD,CREB1,DDIT3 |
| ITF2096 | chemical reagent | | 7.32E-03 | IL1B,IL6,TNF |
| abrocitinib | chemical drug | | 7.32E-03 | MCL1,MYC,SOCS3 |
| methylamine | chemical - endogenous mammalian | | 7.32E-03 | FN1,IL1B,IL6 |
| hoechst 33342 | chemical reagent | | 7.32E-03 | EPC1,FOS,EPRV1 |
| PTIO | chemical reagent | | 7.32E-03 | CAV1,ITGAM,OPRK1 |
| iboterric acid | chemical toxicant | | 7.32E-03 | BDNF,FOS,JUN |
| peginterferon | biologic drug | | 7.32E-03 | DDX58,ISG15,USP18 |
| dihydrosphingosine 1-phosphate | chemical - endogenous mammalian | | 7.32E-03 | EPAS1,IL6,TNF |
| MCC950 | chemical reagent | | 7.32E-03 | CXCL10,IL1B,IL6 |
| tramadol | chemical drug | | 7.32E-03 | BDNF,FOS,NOS3 |
| ethyl linoleate | chemical - endogenous mammalian | | 7.32E-03 | FADS2,PTGS2,SCSD |
| bupropion | chemical drug | | 7.32E-03 | TNF,TNFRSF10A,TNFRSF10B |
| 1,1-diethyl-2-hydroxy-2-nitrosohydrazine | chemical toxicant | | 7.32E-03 | GADD45A,IFNB1,NFKBIA |
| MR-409 | chemical reagent | | 7.32E-03 | IL1B,IL6,TNF |
| AC063837.1 | other | | 7.32E-03 | CCL2,CCL5,IL6 |
| miR-29a-5p inhibitor | chemical reagent | | 7.32E-03 | EIF2AK2,MX1,OAS1 |
| miR-206 inhibitor | chemical reagent | | 7.32E-03 | EIF2AK2,MX1,OAS1 |
| minocycline-LR | chemical toxicant | | 7.32E-03 | IL6,MYC,TNF |
| galanthamine | chemical drug | | 7.32E-03 | CHRNA7,IGF2,TNF |
| CX-4268 | chemical - kinase inhibitor | | 7.32E-03 | IFNA1/IFNA13,IFNB1,STING1 |
| oxamic acid | chemical - endogenous non-mammalian | | 7.32E-03 | IL1B,INHBA,TNF |
| Vi capsular polysaccharide | chemical drug | | 7.32E-03 | CXCL8,IL6,TNF |
| thymoleasin | chemical toxicant | | 7.32E-03 | CD69,CDKN1A,SOD2 |
| miR-369-5p (miRNAs w/seed GAUCGAC) | mature microRNA | | 7.33E-03 | [illegible] |
| CRKL | kinase | Activated | 7.35E-03 | [illegible] |
| EPHA3 | kinase | | 7.35E-03 | [illegible] |
| TNFAIP2 | other | Activated | 7.35E-03 | [illegible] |
| GNA13 | enzyme | | 7.35E-03 | [illegible] |
| SP2 | transcription regulator | | 7.35E-03 | [illegible] |
| GABBR1 | G-protein coupled receptor | | 7.35E-03 | [illegible] |
| GTF2I | transcription regulator | | 7.35E-03 | [illegible] |
| CRTC1 | transcription regulator | Activated | 7.35E-03 | [illegible] |
| EDNRA | transmembrane receptor | Activated | 7.35E-03 | [illegible] |
| Z-IIE1 | chemical reagent | | 7.35E-03 | [illegible] |
| N-ethyl-N-nitrosourea | chemical toxicant | | 7.35E-03 | [illegible] |
| amlodipine | chemical drug | | 7.35E-03 | [illegible] |
| oxalic acid | chemical reagent | Activated | 7.35E-03 | [illegible] |
| 24-hydroxycholesterol | chemical - endogenous mammalian | | 7.35E-03 | [illegible] |
| NR1D1 | ligand-dependent nuclear receptor | | 7.37E-03 | [illegible] |
| PRKN | enzyme | | 7.37E-03 | [illegible] |
| TNFRSF9 | transmembrane receptor | | 7.37E-03 | [illegible] |
| pentoxifol | chemical drug | | 7.38E-03 | [illegible] |
| miR-3150-5p (miRNAs w/seed CUGGCCA) | mature microRNA | | 7.38E-03 | [illegible] |
| mir-36 | microRNA | | 7.40E-03 | [illegible] |
| FBXW7 | enzyme | | 7.40E-03 | [illegible] |
| PTHLH | other | Activated | 7.40E-03 | [illegible] |
| miR-3063-3p (and other miRNAs w/seed GUGGACA) | mature microRNA | | 7.44E-03 | [illegible] |
| miR-1258-5p (and other miRNAs w/seed GGAUGAG) | mature microRNA | Inhibited | 7.48E-03 | [illegible] |
| miR-1266-5p (and other miRNAs w/seed CUCAGGG) | mature microRNA | | 7.49E-03 | [illegible] |
| miR-6893-3p (miRNAs w/seed GAGAGUG) | mature microRNA | | 7.58E-03 | [illegible] |
| miR-6821-3p (and other miRNAs w/seed GACCUCU) | mature microRNA | Inhibited | 7.58E-03 | [illegible] |
| miR-374b-3p (miRNAs w/seed UUAGCAG) | mature microRNA | Inhibited | 7.58E-03 | [illegible] |
| ABCB4 | transporter | | 7.60E-03 | [illegible] |
| CDK5 | kinase | | 7.60E-03 | [illegible] |
| CDC42 | enzyme | | 7.60E-03 | [illegible] |
| MEIS1 | transcription regulator | Activated | 7.60E-03 | [illegible] |
| fisetin | chemical toxicant | | 7.60E-03 | [illegible] |
| CHADL | other | | 7.62E-03 | [illegible] |
| HOXB13 | transcription regulator | | 7.62E-03 | [illegible] |
| NFIL3 | transcription regulator | Inhibited | 7.62E-03 | [illegible] |
| NPPA | other | | 7.62E-03 | [illegible] |
| ketoconazole | chemical drug | | 7.62E-03 | [illegible] |
| miR-3157-5p (miRNAs w/seed UCAGCCA) | mature microRNA | Inhibited | 7.62E-03 | [illegible] |
| miR-5681b (miRNAs w/seed GGUAUUG) | mature microRNA | | 7.64E-03 | [illegible] |
| ROR2 | kinase | | 7.64E-03 | [illegible] |
| GCG | other | Activated | 7.64E-03 | [illegible] |
| tannic acid | chemical toxicant | | 7.69E-03 | CFB,FASN,IL6,PTGS2,TGFBR1 |
| ZFAS1 | other | | 7.69E-03 | CDH2,KLF2,VIM,ZEB1,ZEB2 |
| HOXA-AS2 | other | Inhibited | 7.69E-03 | [illegible] |
| C9orf72 | other | | 7.69E-03 | [illegible] |
| PBRM1 | other | | 7.69E-03 | [illegible] |
| SARM1 | transmembrane receptor | | 7.69E-03 | CCL5,IFNB1,IL6,TNF,XAF1 |
| G2638 | chemical reagent | | 7.69E-03 | BBC3,IRAK1,NFKBIA,VIM,ZEB1 |
| SLC7A5 | transporter | | 7.69E-03 | [illegible] |
| MED14 | transcription regulator | | 7.69E-03 | DUSP1,IRF8,ISG15,LAD1,NR0C1 |
| DRD1 | G-protein coupled receptor | | 7.69E-03 | BDNF,EGR1,FOS,FOSB,JUNB |
| VLDLR | transporter | | 7.69E-03 | [illegible] |
| AFP | transporter | | 7.69E-03 | [illegible] |
| ENTPD1 | enzyme | Inhibited | 7.69E-03 | [illegible] |
| Ifnr (includes others) | cytokine | Activated | 7.69E-03 | CD40,CD83,DAXX,IRF1,TLR3 |
| sevoflurane | chemical drug | | 7.69E-03 | FOS,IL1B,IL6,PER2,TNF |
| 2-aminoethoxydiphenylborane | chemical reagent | Inhibited | 7.69E-03 | [illegible] |
| phospholipid | chemical - endogenous mammalian | | 7.69E-03 | CCL5,CXCL10,CXCL3,F3,PTGS2 |
| theophylline | chemical drug | | 7.69E-03 | CXCL8,FOS,HS3ST1,IL6,LRP1 |
| tyrphostin AG 1296 | chemical - kinase inhibitor | | 7.69E-03 | CD38,ICAM1,ITGAM,JUN,PTGS2 |
| canrenoate potassium | chemical drug | | 7.69E-03 | [illegible] |
| miR-1277-3p (and other miRNAs w/seed ACGUAGA) | mature microRNA | | 7.78E-03 | [illegible] |
| SPI8 | transcription regulator | | 7.81E-03 | [illegible] |
| GRIN3A | ion channel | Inhibited | 7.85E-03 | [illegible] |
| CCR2 | G-protein coupled receptor | Activated | 7.85E-03 | [illegible] |
| miR-6530 (miRNAs w/seed CCAGCAG) | mature microRNA | | 7.85E-03 | [illegible] |
| Hsp70 | group | | 7.87E-03 | [illegible] |
| cytochalasin D | chemical toxicant | | 7.87E-03 | [illegible] |
| miR-630 (miRNAs w/seed GUAUUCU) | mature microRNA | Inhibited | 7.90E-03 | [illegible] |
| hemin | chemical - endogenous mammalian | | 7.91E-03 | [illegible] |
| miR-486-5p (miRNAs w/seed CCAGAUA) | mature microRNA | | 7.92E-03 | [illegible] |
| SLC26A13 | transporter | | 7.96E-03 | [illegible] |
| miR-6734-3p (miRNAs w/seed CCUUCCC) | mature microRNA | | 8.00E-03 | [illegible] |
| miR-941 (miRNAs w/seed ACCCGGC) | mature microRNA | Inhibited | 8.01E-03 | [illegible] |
| miR-3160-5p (miRNAs w/seed GCUUUCU) | mature microRNA | | 8.03E-03 | [illegible] |
| miR-22-5p (and other miRNAs w/seed GUUCUUC) | mature microRNA | Inhibited | 8.13E-03 | [illegible] |
| miR-590-3p (miRNAs w/seed AAUUUUA) | mature microRNA | | 8.21E-03 | [illegible] |
| GHRL | growth factor | | 8.22E-03 | [illegible] |
| miR-6978-5p (and other miRNAs w/seed AGGGAAG) | mature microRNA | | 8.24E-03 | [illegible] |
| FGFR2 | kinase | | 8.29E-03 | [illegible] |
| miR-4701-5p (and other miRNAs w/seed UGGCCAC) | mature microRNA | | 8.31E-03 | [illegible] |
| miR-6799-5p (miRNAs w/seed GGGAGGU) | mature microRNA | | 8.31E-03 | [illegible] |
| miR-6771-3p (miRNAs w/seed AAACCCC) | mature microRNA | Inhibited | 8.43E-03 | [illegible] |
| miR-6802-3p (miRNAs w/seed UCACCCC) | mature microRNA | Inhibited | 8.44E-03 | [illegible] |
| miR-3064-5p (and other miRNAs w/seed CCAGGGC) | mature microRNA | | 8.46E-03 | [illegible] |
| miR-3191-3p (miRNAs w/seed GGGGACG) | mature microRNA | Inhibited | 8.46E-03 | [illegible] |
| acetovanillone | chemical drug | Inhibited | 8.48E-03 | [illegible] |
| dorsomorphin | chemical - kinase inhibitor | | 8.57E-03 | [illegible] |
| PTPN6 | phosphatase | | 8.57E-03 | [illegible] |
| CLEC11A | growth factor | | 8.57E-03 | [illegible] |
| di(2-ethylhexyl) phthalate | chemical toxicant | Activated | 8.57E-03 | [illegible] |
| miR-3924 (miRNAs w/seed UAUGUAU) | mature microRNA | Inhibited | 8.57E-03 | [illegible] |
| miR-4714-3p (miRNAs w/seed CAACCUA) | mature microRNA | Inhibited | 8.61E-03 | [illegible] |
| miR-548aj-5p (and other miRNAs w/seed GCAAAAG) | mature microRNA | Inhibited | 8.64E-03 | [illegible] |
| miR-153-5p (miRNAs w/seed CAUUUUU) | mature microRNA | Inhibited | 8.71E-03 | [illegible] |
| miR-4668 (miRNAs w/seed GUGGGAC) | mature microRNA | | 8.73E-03 | [illegible] |
| miR-3117-3p (miRNAs w/seed UAGGACU) | mature microRNA | Inhibited | 8.76E-03 | [illegible] |
| miR-675-3p (miRNAs w/seed UGUAUGC) | mature microRNA | | 8.77E-03 | [illegible] |
| miR-6789-5p (miRNAs w/seed UAGGGGC) | mature microRNA | Inhibited | 8.77E-03 | [illegible] |
| miR-7113-5p (and other miRNAs w/seed CCAGGGA) | mature microRNA | | 8.79E-03 | [illegible] |
| miR-4786-3p (miRNAs w/seed GAAGCCA) | mature microRNA | | 8.79E-03 | [illegible] |
| miR-6809-5p (miRNAs w/seed GGCAAGG) | mature microRNA | Inhibited | 8.83E-03 | [illegible] |
| miR-6826-3p (miRNAs w/seed UCCCCUC) | mature microRNA | Inhibited | 8.86E-03 | [illegible] |
| miR-4270 (and other miRNAs w/seed CAGGGAG) | mature microRNA | | 8.87E-03 | [illegible] |
| CYB5R4 | enzyme | | 8.88E-03 | [illegible] |
| dasatinib | chemical drug | | 8.88E-03 | [illegible] |
| TRAP | enzyme | | 8.88E-03 | [illegible] |
| mir-218 | microRNA | | 8.88E-03 | [illegible] |
| mir-486 | microRNA | | 8.88E-03 | [illegible] |
| MLX | transcription regulator | | 8.88E-03 | [illegible] |
| DHX9 | enzyme | Activated | 8.88E-03 | [illegible] |
| LCP2 | other | | 8.88E-03 | [illegible] |
| RPA1 | other | | 8.88E-03 | [illegible] |
| farnesol | chemical reagent | Activated | 8.88E-03 | [illegible] |
| biochanin A | chemical toxicant | | 8.88E-03 | [illegible] |
| leptomycin B | chemical drug | | 8.88E-03 | [illegible] |
| betaine | chemical - endogenous mammalian | Inhibited | 8.88E-03 | [illegible] |
| chitin | chemical - endogenous non-mammalian | Activated | 8.88E-03 | [illegible] |
| 7-ketocholesterol | chemical - endogenous mammalian | | 8.88E-03 | [illegible] |
| miR-3584-5p (miRNAs w/seed GGAGGAG) | mature microRNA | | 8.88E-03 | [illegible] |
| miR-192-5p (and other miRNAs w/seed UGACCUA) | mature microRNA | Inhibited | 8.98E-03 | [illegible] |
| miR-5010-3p (and other miRNAs w/seed UUUGUGU) | mature microRNA | | 9.00E-03 | [illegible] |
| miR-196a-5p (and other miRNAs w/seed AGGUAGU) | mature microRNA | | 9.01E-03 | [illegible] |
| miR-4733-5p (miRNAs w/seed AUCCCAA) | mature microRNA | | 9.01E-03 | [illegible] |
| USP22 | peptidase | Activated | 9.03E-03 | [illegible] |
| carbamylcholine | chemical drug | Activated | 9.03E-03 | [illegible] |
| propofol | chemical drug | | 9.03E-03 | [illegible] |
| miR-591 (miRNAs w/seed GACCAUG) | mature microRNA | Inhibited | 9.10E-03 | [illegible] |
| miR-5699-5p (miRNAs w/seed GCCCCAA) | mature microRNA | Inhibited | 9.11E-03 | [illegible] |
| streptozocin | chemical drug | | 9.12E-03 | [illegible] |
| miR-6875-3p (miRNAs w/seed UUCUUCC) | mature microRNA | Inhibited | 9.14E-03 | [illegible] |
| miR-6505-3p (and other miRNAs w/seed GACUUCU) | mature microRNA | Inhibited | 9.18E-03 | [illegible] |
| miR-582-5p (miRNAs w/seed UACAGUU) | mature microRNA | Inhibited | 9.18E-03 | [illegible] |
| miR-4776-3p (miRNAs w/seed UUGCCUA) | mature microRNA | Inhibited | 9.20E-03 | [illegible] |

| | | | | |
|---|---|---|---|---|
| miR-1282 (miRNAs w/seed CGUUUGC) | mature microRNA | | 9.23E-03 | AHNAK,ANKH,ATP5CKMT,BMP8B,CDKN2B,CHD9,CYSLTR1,DAB2IP,DENND1D,EMP2,EYA1,FOSL2 [illegible] |
| miR-877-3p (miRNAs w/seed CCUCUUC) | mature microRNA | Inhibited | 9.26E-03 | ACKR4,ACP7,ACTR2,ADH6,AGER,AK4,ANKS4B [illegible] |
| miR-3189-3p (miRNAs w/seed CCUUGGG) | mature microRNA | Inhibited | 9.29E-03 | ACP2,ADAR,ADRA2A,AKR7A2,ANXA2,AP3B2 [illegible] |
| GATA6 | transcription regulator | | 9.33E-03 | ACO1,ACTA2,C1R,C4A/C4B,CAV1,CDKN1A,COL4A1,CRYL1,EDN1,FGF2,FH,GATA4 [illegible] |
| DGCR8 | enzyme | | 9.37E-03 | ACTA2,BDNF,CNP,EGR1,EGR2,FOS,ID4,MYC,NGFR,PDCD4,SMOC1,TCF7L2,TNC |
| TEAD2 | transcription regulator | Activated | 9.37E-03 | ADM,ARID5B,EDN1,EDNRA,FGFR4,FLIP1L,GPR37,LIMCH1,MCM6,NFKBIZ,PDGFRL,SLFN5,TPM1 |
| beta-naphthoflavone | chemical toxicant | | 9.37E-03 | AKR1C1/AKR1C2,APOE,CRP,CYP1A1,CYP1B1,CYP2U1,CYP2W1,FOS,MAP2,NR4A1,SAA1,SOCS2,UGT1A10 (includes others) |
| RUNX3 | transcription regulator | | 9.38E-03 | BIRC3,BCL2L11,BIRC5,CDKN1A,CFLAR,CIITA,COL4A1,COMMD3-BMI1,CSF1R,CXCL8,EXT1,FGF2,IL1B,ITGAE,ITGAM,ITGB3,LIFR,MYC,RESP1,SGPP1,SPP1,STC2,TGFB2,TLCD4,TNF,VIM |
| miR-323-5p (and other miRNAs w/seed GGUGGUC) | mature microRNA | Inhibited | 9.39E-03 | ABCF1,ACAA2,ALDH7A1,ANTXR2,APOBEC3G [illegible] |
| miR-6788-3p (miRNAs w/seed UCGCCAC) | mature microRNA | | 9.43E-03 | ARRDC3,CD274,CHD2,CLEC18A/CLEC18C,COQ10B [illegible] |
| PRKCQ | kinase | | 9.46E-03 | CD69,CDKN1A,FOSL1,GATA3,IL6,ITGB1,MMP1,NFKB2,PRKCE,TNF |
| miR-6524a-3p (miRNAs w/seed GAGACAG) | mature microRNA | | 9.50E-03 | AADAT,ACE2,ACP7,ACTA2,ADM,AK4,ANGPT2 [illegible] |
| calpain | complex | | 9.56E-03 | AQP4,CCL5,CREB1,DDIT3,HSPA5,IL6,NFKBIA,NTRK2 |
| Smad | complex | | 9.56E-03 | ANGPTL4,CDKN1A,CDKN2B,ID3,MCL1,MYC,SERPINE1,VEGFA |
| TNFRSF11B | transmembrane receptor | | 9.56E-03 | BMP2,IL11,IL6,ITGAV,RUNX2,SDC1,SPP1,TNFRSF11B |
| BTRC | enzyme | | 9.56E-03 | BST2,CDKN1A,CHD1,CXCL8,IL1B,IL6,SNAI1,TRAF1 |
| UBE3A | enzyme | Inhibited | 9.56E-03 | ARC,CDKN1A,IL1B,NR4A2,PML,RNF2,SERPINE1,SOX9 |
| NMU | other | | 9.56E-03 | BTG2,CDCA3,DIO2,FOSB,IL6,MKI67,NMU,SPAG5 |
| EGLN3 | enzyme | | 9.56E-03 | ADM,BCL2L11,IL1A,IL1B,LDHA,NOS3,TNFRSF11B,VEGFA |
| PCYT1A | enzyme | Activated | 9.56E-03 | CDK19,CXCL2,EPHA7,FST,GSN,KLF4,SCD,SIX4 |
| miR-6882-5p (miRNAs w/seed ACAAGUC) | mature microRNA | Inhibited | 9.62E-03 | ABCF1,AGTPBP1,ANKRD18B,AP1S2,ARMCX1 [illegible] |
| miR-154-3p (and other miRNAs w/seed AUCAUAC) | mature microRNA | | 9.62E-03 | ABTB2,ACYP1,ALG14,ANKRD18B,ANXA4 [illegible] |
| miR-6438 (miRNAs w/seed ACAGGCU) | mature microRNA | | 9.68E-03 | ABCD1,ABHD2,ADAR,ANGPT2,ANKFY1 [illegible] |
| GC-GCR dimer | complex | | 9.69E-03 | ADRB2,AGT,BCL2L11,DUSP1,FBP1,IL1RN,NFKBIA,PC,PIK3R1 |
| trabectedin | chemical drug | | 9.69E-03 | CDKN1A,DHRS2,G6PD,GADD45A,H1-10,LGMN,NXF1,OAT,SAT1 |
| urethane | chemical toxicant | | 9.69E-03 | CDKN1A,CDKN2B,CYP1A1,IL12A,IL1B,MYC,PTGS1,PTGS2,TEP1 |
| dithiothreitol | chemical reagent | Activated | 9.69E-03 | ATF3,DDIT3,ERO1B,GADD45A,HSPA5,IFNB1,MYC,TLR2,VEGFA |
| plerixafor | chemical drug | | 9.69E-03 | CCL22,CDA,IL6,KITLG,KLF4,MMP1,NFE2L2,NOS3,PDK1 |
| miR-4782-5p (and other miRNAs w/seed UCUGGAU) | mature microRNA | | 9.73E-03 | ABCA3,ABCC9,ACO1,ACTL6A,ADORA1 [illegible] |
| miR-506-3p (and other miRNAs w/seed GGGAGAG) | mature microRNA | | 9.78E-03 | ABAT,ABHD14B,ACO1,ADH6,ADM2,ADORA1 [illegible] |
| miR-6755-3p (miRNAs w/seed GUUGUCA) | mature microRNA | | 9.80E-03 | ACE2,AKAP7,ALCAM,ALDH1A1,AN0,ANGPT2 [illegible] |
| Erk5 | kinase | Activated | 9.80E-03 | ACTA2,C1R,CFB,COL4A1,COL8A1,IL1A,IL6,KLF4,LOXL2,MYC,NBN,PDGFA,PLAT,SPTA1,TNFSF10,TNFSF15,ZNF280 |
| miR-6510-5p (miRNAs w/seed AGCAGGG) | mature microRNA | | 9.81E-03 | ABCA2,ABHD14B,AFF4,AIF1L,AK4,ALCAM [illegible] |
| miR-4279 (miRNAs w/seed UCUCCUC) | mature microRNA | Inhibited | 9.85E-03 | A4GALT,ABRAXAS2,ADM,ADORA1,AGER [illegible] |
| miR-3157-3p (miRNAs w/seed UGCCCUA) | mature microRNA | Inhibited | 9.87E-03 | ABCC4,ABHD14B,ACP7,ADAR,AHCY,AHR [illegible] |
| miR-7704 (miRNAs w/seed GGGGUCG) | mature microRNA | | 9.87E-03 | ABHD14B,ABTB1,ACP2,ADM2,ADORA1,AIF1L [illegible] |
| miR-1260-3p (miRNAs w/seed CAUUUUC) | mature microRNA | Inhibited | 9.88E-03 | ACVR1C,ANKRD12,ARID5B,ARL4A,ARRDC3 [illegible] |
| budesonide | chemical drug | | 9.88E-03 | BCL2L11,CCL5,CDKN1A,CSF3,CXCL10 [illegible] |
| miR-6873-5p (miRNAs w/seed AGAGGGA) | mature microRNA | Inhibited | 9.98E-03 | ABHD15,ABHD2,ACP7,ACTR1B,ADM2 [illegible] |
| miR-3115 (miRNAs w/seed UAUGGGU) | mature microRNA | Inhibited | 1.00E-02 | AADAT,ACVR1C,AK4,ALDH1A2,ANGPT2 [illegible] |
| miR-8073 (miRNAs w/seed UGUGAUU) | mature microRNA | | 1.00E-02 | ACYP1,ADAMDEC1,AGAP6 (includes others) [illegible] |
| miR-6803-3p (miRNAs w/seed CCCUCGC) | mature microRNA | | 1.01E-02 | ABCD1,ABTB1,AEN,AKR1C1/AKR1C2 [illegible] |
| H1-6 | other | | 1.01E-02 | ARPC5,ATP6V0E2,B2M,B4GALT5,BCL3,CYT [illegible] |
| H1FI | other | | 1.01E-02 | ARPC5,ATP6V0E2,B2M,B4GALT5,BCL3,CYT [illegible] |
| miR-6749-3p (miRNAs w/seed UCCUCCC) | mature microRNA | | 1.01E-02 | AADAT,ACO1,ADGRF4,AGER,AIF1L,AKR1C [illegible] |
| miR-576-5p (miRNAs w/seed GAGUGUG) | mature microRNA | | 1.01E-02 | ABTB2,ACE2,ACTR1B,AQP4,ARC,ARID3A [illegible] |
| PRKACA | kinase | | 1.02E-02 | ANTXR2,BCL2L11,BDNF,CCND3,CEBPB [illegible] |
| N-acetylsphingosine | chemical reagent | Activated | 1.02E-02 | CAT,CDKN1A,CEBPB,CXCL2,CXCL8,FOS [illegible] |
| miR-4453 (and other miRNAs w/seed AGCUUGG) | mature microRNA | | 1.02E-02 | ABHD14B,ACO1,AKR1B10,APOL3,BCL2L14 [illegible] |
| miR-3097-5p (and other miRNAs w/seed ACAGGUG) | mature microRNA | | 1.02E-02 | AADAT,ABCD1,ACADSB,ACYP1,ADM2,AIF1L [illegible] |
| miR-218-5p (and other miRNAs w/seed UGUGCUU) | mature microRNA | Inhibited | 1.02E-02 | ABHD15,ABRAXAS2,AFF4,AIF1L,ANKRD1 [illegible] |
| miR-637 (and other miRNAs w/seed CUGGGGG) | mature microRNA | Inhibited | 1.03E-02 | ABCA2,ABHD14B,ABHD2,ABTB2,ADM2 [illegible] |
| miR-3685 (miRNAs w/seed UUCCUAC) | mature microRNA | | 1.03E-02 | ATP5CKMT,BAZ1A,BCL2L13,C18orf25,CAV [illegible] |
| miR-577 (miRNAs w/seed AGAUAAA) | mature microRNA | | 1.03E-02 | ADK,AQP4,ARL6IP5,ARMC3,BDP1,BLZF1 [illegible] |
| miR-6739-3p (miRNAs w/seed UUGUUCU) | mature microRNA | Inhibited | 1.04E-02 | ABRAXAS2,AGT,AKR1B10,ARHGDIB,ARL14 [illegible] |
| miR-6895-5p (and other miRNAs w/seed AGGAGGC) | mature microRNA | Inhibited | 1.05E-02 | ACP2,ACP7,ADM2,AK4,AKAP7,ANGPTL4 [illegible] |
| miR-6427 (miRNAs w/seed CUGAAUA) | mature microRNA | Inhibited | 1.05E-02 | AFF4,ARRDC4,ART3,BDNF,CASP7,CASP8 [illegible] |
| miR-682 (miRNAs w/seed UGCAGUC) | mature microRNA | Inhibited | 1.05E-02 | ABCD1,ACSS2,AGAP6 (includes others),ALD [illegible] |
| epoprostenol | chemical - endogenous mammalian | | 1.05E-02 | EGR1,FOS,MMP1,PTGS2,SERPINE1,SPP1 |
| CSF2RB | transmembrane receptor | Activated | 1.05E-02 | FOS,IL6,ITGAM,JUN,JUNB,MYC |
| resatorvid | chemical drug | Inhibited | 1.05E-02 | CAT,CCL2,CXCL8,IL1B,IL6,TNF |
| UNC93B1 | other | Activated | 1.05E-02 | CXCL10,IFNB1,IFNL1,IL1B,IL6,TNF |
| GADL3 | other | | 1.05E-02 | BTG2,CDKN1A,EPHX1,FAS,LIF,SAT1 |
| MESP1 | transcription regulator | Activated | 1.05E-02 | GATA4,MEF2C,RASGRP3,SNAI1,ZEB1,ZEB2 |
| PURA | transcription regulator | | 1.05E-02 | ACTA2,CASP8,CDKN1A,DHFR,ITGAM,MYC |
| GPNMB | enzyme | | 1.05E-02 | FOS,IL6,ITGA5,ITGB1,NRP1,TNF |
| TGIF1 | transcription regulator | Activated | 1.05E-02 | CXCL1,CXCL2,CXCL3,CXCL8,IL1B,IL6 |
| [illegible] | | | | |

| | | | |
|---|---|---|---|
| Rab5 | group | 1.60E-02 | ADRB2,IL6 |
| glemanserin | chemical drug | 1.60E-02 | NR4A1,NR4A3 |
| doxmetin | chemical - endogenous non-mammalian | 1.60E-02 | CYP1A1,TNF |
| DF 203 | chemical drug | 1.60E-02 | CYP1A1,CYP1B1 |
| Mitochondrial complex 1 | complex | 1.60E-02 | MAOA,PPARGC1A |
| NPS | other | 1.60E-02 | FOS,ITGAM |
| SFTPA2 | other | 1.60E-02 | HSPA5,XBP1 |
| T-type Calcium Channel | complex | 1.60E-02 | ANKH,ENPP1 |
| Rpn11a-ps4 | other | 1.60E-02 | NFKB2,NFKBIA |
| Rkx1 | enzyme | 1.60E-02 | CCNB1,PTTG1 |
| EIF4B | translation regulator | 1.60E-02 | ISG15,MCL1 |
| TRIM50 | other | 1.60E-02 | IFIT2,IFNB1 |
| FAM168A | other | 1.60E-02 | CCNB1,NFKBIA |
| UPK3A | other | 1.60E-02 | UPK1B,UPK3B |
| FBXO25 | enzyme | 1.60E-02 | EGR1,FOS |
| NMRAL1 | other | 1.60E-02 | MX1,TNF |
| POPDC2 | other | 1.60E-02 | IL1B,TNF |
| ARL16 | other | 1.60E-02 | CCL5,IFNB1 |
| LYST | transporter | 1.60E-02 | IFNB1,TNF |
| HeD | other | 1.60E-02 | PDZD2,PROM2 |
| MARCHF2 | enzyme | 1.60E-02 | TFRC,TNFRSF10A |
| SSF29 | other | 1.60E-02 | DDIT3,HSPA5 |
| TRIM71 | enzyme | 1.60E-02 | IFNB1,MYC |
| SMYD5 | other | 1.60E-02 | CXCL10,TNF |
| PHOSPHO1 | enzyme | 1.60E-02 | PSPH,SHMT1 |
| ZNF385B | other | 1.60E-02 | FAS,PERP |
| MLXIP | transcription regulator | 1.60E-02 | ARRDC4,TXNIP |
| NCEH1 | enzyme | 1.60E-02 | DDIT3,HSPA5 |
| ARL11 | other | 1.60E-02 | IL6,TNF |
| SENP6 | peptidase | 1.60E-02 | BIRC3,CXCL8 |
| PACS2 | other | 1.60E-02 | HLA-C,HSPA5 |
| Pfk | group | 1.60E-02 | CXCL10,IFNB1 |
| Cm-Cel Receptor | complex | 1.60E-02 | JAK2,MYC |
| MAP3K21 | kinase | 1.60E-02 | SNAI1,VIM |
| AFAP1L2 | other | 1.60E-02 | CXCL8,IL6 |
| ECD | transcription regulator | 1.60E-02 | ATF4,HSPA5 |
| soy isoflavones | chemical drug | 1.60E-02 | MKI67,PTGS2 |
| [illegible] | [illegible] | [illegible] | [illegible] |

| | | | | |
|---|---|---|---|---|
| SCUBE3 | other | | 1,90E-02 | FGFR4,SERPINE1,SNAI1,VIM |
| NPC1L1 | transporter | | 1,90E-02 | FOXO1,HMGCR,HMGCS1,SREBF2 |
| UACA | other | | 1,90E-02 | CEBPD,DDIT3,HSPA5,IL6 |
| IL22RA1 | transmembrane receptor | | 1,90E-02 | CCL20,CXCL8,HBEGF,SOD2 |
| PPP1CA | phosphatase | | 1,90E-02 | CDH2,FOS,RUNX2,SPP1 |
| MCAM | other | | 1,90E-02 | ATF3,CD24,FN1,VIM |
| TKT | enzyme | | 1,90E-02 | BIRC5,IL6,MCL1,TNF |
| PDYN | transporter | | 1,90E-02 | ARC,NRP1,OPRK1,TNF |
| DEK | transcription regulator | | 1,90E-02 | BIRC3,CXCL8,MCL1,NFKBIA |
| niflumic acid | chemical drug | | 1,90E-02 | CFLAR,CXCL8,FAS,IL6 |
| ziprasidone | chemical drug | | 1,90E-02 | BDNF,HMGCR,HMGCS1,SCD |
| tyrphostin AG 127 | chemical - kinase inhibitor | | 1,90E-02 | IL1B,IRAK1,PTGS2,TNF |
| I-BOP | chemical reagent | | 1,90E-02 | CCL2,ICAM1,MMP1,PTGS2 |
| L-N6-(1-iminoethyl)-lysine | chemical reagent | | 1,90E-02 | FMR1,ITGAM,PTGS2,VEGFA |
| miR-548aw (miRNAs w/seed UGCAAAA) | mature microRNA | | 1,90E-02 | [illegible] |
| miR-3921 (and other miRNAs w/seed CUCUGAG) | mature microRNA | | 1,92E-02 | [illegible] |
| miR-4451 (miRNAs w/seed GGUAGAG) | mature microRNA | Inhibited | 1,92E-02 | [illegible] |
| miR-3177-5p (miRNAs w/seed CUGUACA) | mature microRNA | Inhibited | 1,93E-02 | [illegible] |
| BMPR2 | kinase | Activated | 1,93E-02 | CXCL8,EPHA4,FN1,ICAM1,RUNX2,SNAI1,SPP1,TNFRSF11B |
| ELOVL6 | enzyme | | 1,95E-02 | FADS1,FADS2,FASN,FOXO1,PPARGC1A,RICTOR,SCD,TRIB3 |
| REN | peptidase | | 1,95E-02 | AGT,CYP2J2,F3,FGF2,FOS,ICAM1,IL6,TNF |
| SFN | other | | 1,95E-02 | BCL6,BTG2,CDH2,DSG2,F3,SOX1,VIM,ZEB1 |
| arsenic | chemical toxicant | | 1,95E-02 | BIRC3,DDIT3,ERN1,FOS,KEAP1,NOS3,SPINK1,TRAF1 |
| miR-4433b-3p (miRNAs w/seed AGGAGUG) | mature microRNA | | 1,95E-02 | [illegible] |
| GLIS2 | transcription regulator | | 1,95E-02 | C3,CXCL10,LTBP2,SERPINE1,SNAI1,VIM |
| FZD8 | G-protein coupled receptor | | 1,95E-02 | ACTA2,BIRC5,BMP2,FN1,GATA4,VIM |
| HOTAIRM1 | other | | 1,95E-02 | CFH,GRB10,KCNQ1,KLF2,MCTP1,SPINK1 |
| mir-154 | microRNA | | 1,95E-02 | BCL2L11,BIRC5,FZD4,KLF10,MYC,PLAUR |
| [illegible] | transcription regulator | | 1,95E-02 | CDKN1A,FOXO1,GATA3,MYC,SOCS1,SOCS3 |
| PDK4 | kinase | | 1,95E-02 | IL1B,IL6,IRF8,PC,PPARGC1A,TNF |
| indirubin | chemical drug | | 1,95E-02 | BIRC2,BIRC3,BIRC5,CYP1A1,JUN,MYC |
| propionic acid | chemical - endogenous mammalian | | 1,95E-02 | CCL22,ICAM1,IL1A,IL1B,RASGRP2,SMOC1 |
| magnolol | chemical - endogenous non-mammalian | | 1,95E-02 | CDKN1A,CXCL8,FOS,IL6,PTGS2,TNF |
| lisinopril | biologic drug | Inhibited | 1,95E-02 | FOS,IL1B,JUN,PDGFA,TNF,VEGFA |
| TGAL copolymer | biologic drug | | 1,95E-02 | BDNF,CCL5,CXCL8,IL1B,IL1RN,TNF |
| glycyrrhetinic acid | chemical drug | | 1,95E-02 | ATP1A1,BIRC3,ICAM1,IL6,MYD88,TRIM5 |
| irinotecan | chemical drug | Activated | 1,95E-02 | FAS,IL1B,IL6,PMAIP1,SNAI1,TNF |
| iloprost | chemical drug | | 1,95E-02 | ANGPT2,CCL2,CCL5,CXCL8,IL1B,TNF |
| tauroursodeoxycholic acid | chemical - endogenous mammalian | Inhibited | 1,95E-02 | BAD,DDIT3,HSPA5,IL1B,IL6,TNF |
| miR-4735-5p (miRNAs w/seed CUAAUUU) | mature microRNA | Inhibited | 1,96E-02 | [illegible] |
| miR-3658 (miRNAs w/seed AUUCCCU) | mature microRNA | Inhibited | 1,96E-02 | [illegible] |
| miR-448-3p (and other miRNAs w/seed UGCAUAU) | mature microRNA | Inhibited | 1,96E-02 | [illegible] |
| miR-106a-3p (and other miRNAs w/seed UGCAAUG) | mature microRNA | | 1,97E-02 | [illegible] |
| miR-7158-3p (miRNAs w/seed UGAACUA) | mature microRNA | | 1,97E-02 | [illegible] |
| AURK | group | Inhibited | 1,97E-02 | AEN,BBC3,BTG2,CDKN1A,CYFIP2,NYNRIN,PHLDB3,RRAD,SESN2,TNFRSF10A,TNFRSF10B,ZNF79 |
| ANLN | other | Inhibited | 1,97E-02 | AEN,BBC3,BTG2,CDKN1A,CYFIP2,NYNRIN,PHLDB3,RRAD,SESN2,TNFRSF10A,TNFRSF10B,ZNF79 |
| miR-186 (and other miRNAs w/seed GGACGGA) | mature microRNA | | 1,98E-02 | [illegible] |
| miR-4797-3p (miRNAs w/seed CUCAGUA) | mature microRNA | Inhibited | 1,98E-02 | [illegible] |
| miR-4768-3p (miRNAs w/seed CCGGUGC) | mature microRNA | | 1,98E-02 | [illegible] |
| miR-6999-3p (miRNAs w/seed CACUACC) | mature microRNA | Inhibited | 1,98E-02 | [illegible] |
| miR-876-5p (and other miRNAs w/seed GAGGCUU) | mature microRNA | | 1,98E-02 | [illegible] |
| YBX1 | transcription regulator | | 1,99E-02 | ACTA2,APOL2,CCL5,CDKN1A,CXCL2,F3,FOS,HLA-DQB1,HLA-DRA,KLF4,MYCL,NFE2L2,PDK1,PLXNA2,PMAIP1,PTGS2,SLC2A3,SNAI1,STC2,TRIM16 |
| miR-2392 (miRNAs w/seed AGGAUGG) | mature microRNA | | 1,99E-02 | [illegible] |
| SUZ12 | enzyme | | 1,99E-02 | [illegible] |
| miR-3126-5p (and other miRNAs w/seed GAGGGAC) | mature microRNA | | 2,00E-02 | [illegible] |
| alvespimycin | chemical drug | | 2,00E-02 | FASN,FDFT1,HMGCR,HSPA1A/HSPA1B,MCL1,SCD,SREBF2 |
| HTATIP2 | transcription regulator | | 2,00E-02 | ANGPT2,CDH2,FOS,MYC,SNAI1,SPP1,VEGFA |
| MGAT5 | enzyme | | 2,00E-02 | CDH2,ITGA5,ITGB1,MYC,SNAI1,TGFBR1,TNF |
| CETP | enzyme | | 2,00E-02 | APOE,DDIT3,HERPUD1,HMGCR,HSPA5,PLIN2,TNF |
| pifithrin-alpha | chemical reagent | | 2,00E-02 | BBC3,CDKN1A,CYP1A1,FAS,TNFRSF10A,TNFRSF10B,XAF1 |
| [illegible] | chemical reagent | | 2,00E-02 | CD38,CDKN1A,CSF1R,F3,IRF1,PLAT,TGFB2 |
| beta-glycerophosphoric acid | chemical - endogenous mammalian | Activated | 2,00E-02 | BMP2,CCND2,CDKN1A,PTGS2,RUNX2,SPP1,TNFRSF11B |
| 9Z,11E-octadecadienoic acid | chemical - endogenous mammalian | | 2,00E-02 | FADS1,FASN,IL10RA,IL12A,NFAT5,PTGS2,UCP2 |
| miR-4450 (and other miRNAs w/seed GGGGAUU) | mature microRNA | Inhibited | 2,01E-02 | [illegible] |
| miR-197-5p (and other miRNAs w/seed GGGUAGA) | mature microRNA | | 2,02E-02 | [illegible] |
| miR-3109-3p (and other miRNAs w/seed AGGGCCA) | mature microRNA | | 2,04E-02 | [illegible] |
| miR-1261-3p (miRNAs w/seed GCUUUGC) | mature microRNA | Inhibited | 2,04E-02 | [illegible] |
| miR-548as (and other miRNAs w/seed AAAACCA) | mature microRNA | | 2,04E-02 | [illegible] |
| miR-6886-3p (miRNAs w/seed GCCCUUC) | mature microRNA | Inhibited | 2,04E-02 | [illegible] |
| miR-7064-3p (and other miRNAs w/seed UGCAGCC) | mature microRNA | | 2,07E-02 | [illegible] |
| miR-1306-3p (miRNAs w/seed CGUUGGC) | mature microRNA | | 2,07E-02 | [illegible] |
| miR-4777-3p (miRNAs w/seed UACCUCA) | mature microRNA | Inhibited | 2,08E-02 | [illegible] |
| miR-4323 (miRNAs w/seed AGCCCCA) | mature microRNA | Inhibited | 2,08E-02 | [illegible] |
| RETNLB | other | Activated | 2,09E-02 | ACTA2,CCL2,CCL5,CCR1,E2F7,FAP,FN1,IL1B,IL6,IRS2,LMNB1,MYC,NR4A1,SOCS3,TLR2,TNNC1,UBE2C |
| phenylephrine | chemical drug | Activated | 2,10E-02 | [illegible] |
| TSC1 | other | | 2,10E-02 | [illegible] |
| miR-1185-5p (and other miRNAs w/seed GAGGAUA) | mature microRNA | Inhibited | 2,11E-02 | [illegible] |
| miR-6868-3p (miRNAs w/seed GACCCCU) | mature microRNA | Inhibited | 2,12E-02 | [illegible] |
| miR-320c (and other miRNAs w/seed AAAGCUG) | mature microRNA | | 2,12E-02 | [illegible] |
| miR-3909 (and other miRNAs w/seed GUCCUCU) | mature microRNA | | 2,13E-02 | [illegible] |
| miR-1908 (and other miRNAs w/seed AAAGGCC) | mature microRNA | Inhibited | 2,16E-02 | [illegible] |
| miR-888-5p (miRNAs w/seed ACUCAAA) | mature microRNA | Inhibited | 2,16E-02 | [illegible] |
| miR-3915 (miRNAs w/seed UGAGGAA) | mature microRNA | Inhibited | 2,19E-02 | [illegible] |
| miR-297a-5p (and other miRNAs w/seed UGUAUGU) | mature microRNA | Inhibited | 2,19E-02 | [illegible] |
| miR-4713-3p (miRNAs w/seed GGGAUCC) | mature microRNA | | 2,22E-02 | [illegible] |
| miR-1343-5p (and other miRNAs w/seed GGGGAGC) | mature microRNA | | 2,22E-02 | [illegible] |
| FOXA2 | transcription regulator | | 2,23E-02 | [illegible] |
| GAB2 | other | | 2,23E-02 | CD69,FOS,GSN,IL6,MCL1,MUC5B,SERPINE1,TNF,ZEB1 |
| CLN3 | other | | 2,23E-02 | ASAH1,CTSD,GAA,LGMN,LIPA,NPC2,PPT1,PSAP,SIAE |
| CHRNA7 | transmembrane receptor | Inhibited | 2,23E-02 | CDKN1A,CXCL10,CXCL2,CXCL3,IL1B,IL1RN,IL6,ITGAM,TNF |
| IGF2BP1 | translation regulator | | 2,23E-02 | BIRC2,CDKN1A,CYFIP2,DDIT3,GADD45A,IGF2,MYC,SOX9,TNC |
| PAK1 | kinase | | 2,23E-02 | CCNB1,CDKN1A,CTSD,F3,FN1,FOSL1,ITGAM,MYC,VEGFA |
| bile acid | chemical - endogenous mammalian | | 2,23E-02 | CAV1,DIO2,EPHX1,FABP6,HNF4A,IL23A,PTGS2,TNF,TNFRSF10B |
| miR-610 (miRNAs w/seed GAGCUAA) | mature microRNA | | 2,24E-02 | [illegible] |
| miR-191-5p (and other miRNAs w/seed AACGGAA) | mature microRNA | Inhibited | 2,26E-02 | [illegible] |
| miR-1249-3p (and other miRNAs w/seed CGCCCUU) | mature microRNA | | 2,26E-02 | [illegible] |
| miR-4765 (miRNAs w/seed GAGUGAU) | mature microRNA | Inhibited | 2,26E-02 | [illegible] |
| miR-6834-3p (miRNAs w/seed AUGUCCC) | mature microRNA | Inhibited | 2,26E-02 | [illegible] |
| miR-1266-3p (miRNAs w/seed AUCUGGG) | mature microRNA | | 2,26E-02 | [illegible] |
| CDK4 | kinase | | 2,27E-02 | [illegible] |
| miR-597-3p (and other miRNAs w/seed GGUUCUC) | mature microRNA | Inhibited | 2,28E-02 | [illegible] |
| miR-2861 (and other miRNAs w/seed GGGCCUG) | mature microRNA | Inhibited | 2,28E-02 | [illegible] |
| miR-655-5p (miRNAs w/seed GAGGUUA) | mature microRNA | Inhibited | 2,28E-02 | [illegible] |
| miR-349 (and other miRNAs w/seed AGCCCUG) | mature microRNA | Inhibited | 2,29E-02 | [illegible] |
| miR-8086 (miRNAs w/seed GCUAGUC) | mature microRNA | | 2,29E-02 | [illegible] |
| BRD2 | kinase | Activated | 2,29E-02 | CD47,CNOT4,IL1B,IL6,IL7R,MYC,PKIB,RAB24,SPTA1,STAT4,TNF,VAV3 |
| candesartan | chemical drug | Inhibited | 2,29E-02 | ADM,AGT,CREB1,FN1,FOS,FOSL2,ICAM1,IL6,NOX2,SERPINE1,SPP1,TNF |
| PRDM5 | transcription regulator | | 2,30E-02 | [illegible] |
| miR-890 (miRNAs w/seed ACUUGGA) | mature microRNA | | 2,31E-02 | [illegible] |
| miR-151-3p (and other miRNAs w/seed UAGACUG) | mature microRNA | Inhibited | 2,32E-02 | [illegible] |
| miR-196-3p (and other miRNAs w/seed CAGUGGG) | mature microRNA | | 2,32E-02 | [illegible] |
| miR-6862-5p (miRNAs w/seed UAGCCAA) | mature microRNA | | 2,32E-02 | [illegible] |
| E2f | group | Inhibited | 2,34E-02 | [illegible] |
| miR-520g-5p (miRNAs w/seed CUAGAGG) | mature microRNA | Inhibited | 2,34E-02 | [illegible] |
| miR-4781-5p (miRNAs w/seed AGCGGGG) | mature microRNA | | 2,35E-02 | [illegible] |
| hexachlorobenzene | chemical toxicant | | 2,36E-02 | [illegible] |
| MMP3 | peptidase | | 2,37E-02 | [illegible] |
| NFATC1 | transcription regulator | Activated | 2,37E-02 | [illegible] |
| miR-1268 (miRNAs w/seed CCUUCUU) | mature microRNA | | 2,37E-02 | [illegible] |
| miR-568 (miRNAs w/seed UGUAUAA) | mature microRNA | Inhibited | 2,37E-02 | [illegible] |
| miR-3127-5p (miRNAs w/seed UCAGGGC) | mature microRNA | Inhibited | 2,37E-02 | [illegible] |
| miR-758-5p (miRNAs w/seed AUGGUUG) | mature microRNA | | 2,38E-02 | [illegible] |
| miR-6526 (and other miRNAs w/seed GGGGGAU) | mature microRNA | Inhibited | 2,38E-02 | [illegible] |
| IL26 | cytokine | | 2,38E-02 | BTG2,CCL2,CCL5,CSF3,CXCL8,FGF2,FOSB,GATA3,IL12A,IL1B,IL23A,IL6,TNF,TSLP |
| USF2 | transcription regulator | | 2,38E-02 | [illegible] |
| mir-133 | microRNA | | 2,38E-02 | [illegible] |
| FASN | enzyme | | 2,38E-02 | ABCG1,ACTA2,AMOTL2,CAV1,CDH2,CXCL8,DDIT3,FASN,FOXO1,IL1B,IL6,PTGS2,SCD,TNF,VIM |
| COLQ | other | | 2,38E-02 | [illegible] |
| miR-4513 (and other miRNAs w/seed GACUGAC) | mature microRNA | | 2,38E-02 | [illegible] |
| miR-4762-3p (miRNAs w/seed CUGUAUU) | mature microRNA | Inhibited | 2,39E-02 | [illegible] |
| crizotinib | chemical drug | | 2,40E-02 | [illegible] |
| [illegible] | other | | 2,40E-02 | [illegible] |
| SP4 | transcription regulator | | 2,40E-02 | [illegible] |
| [illegible] | chemical - kinase inhibitor | | 2,40E-02 | [illegible] |
| EPOR | transmembrane receptor | | 2,40E-02 | JUN,JUNB,MYC,SLC4A1,SOCS1,SOCS3,TFRC,TNF |
| HOXA7 | transcription regulator | Activated | 2,40E-02 | [illegible] |
| silymarin | chemical drug | Inhibited | 2,40E-02 | CAT,GSR,ICAM1,IL6,LTB,PPARGC1A,TNF,TNFSF10 |
| miR-139-3p (miRNAs w/seed GGAGACG) | mature microRNA | | 2,40E-02 | [illegible] |
| miR-3688a-5p (and other miRNAs w/seed GUGAUAU) | mature microRNA | Inhibited | 2,40E-02 | [illegible] |
| miR-4796-3p (miRNAs w/seed AAAGUGG) | mature microRNA | Inhibited | 2,41E-02 | [illegible] |
| miR-639 (miRNAs w/seed UCGCUGC) | mature microRNA | Inhibited | 2,41E-02 | [illegible] |
| xanthine | chemical - endogenous mammalian | | 2,41E-02 | CD69,DUSP1,DUSP10,ID3,PLAUR |
| mocetinostat | chemical drug | | 2,41E-02 | BDNF,CXCL10,IL1B,MCL1,TNF |
| DANCR | other | | 2,41E-02 | CDH2,RUNX2,SNAI1,VIM,ZEB1 |
| Collagen Alpha1 | group | | 2,41E-02 | CDH2,CDKN1A,IL1RN,PLAUR,PTGS2 |
| SCAVENGER receptor CLASS A | group | | 2,41E-02 | CCL5,CXCL8,IL12A,IL6,TNF |
| TOLLIP | other | | 2,41E-02 | IFNB1,IL6,NFKB1,RELB,TNF |
| CBX2 | transcription regulator | | 2,41E-02 | AHR,CDKN1A,GATA4,LHX9,SOX9 |
| [illegible] | other | | 2,41E-02 | ACTA2,FOS,MMP1,TGFB2,VEGFA |
| XRCC5 | enzyme | | 2,41E-02 | APOE,FASN,NFKB1,SEMA3A,XDH |
| MGAT3 | enzyme | | 2,41E-02 | CDH2,DAG1,ITGA5,NOTCH3,SNAI1 |
| PTPRE | phosphatase | | 2,41E-02 | CXCL2,CXCL3,INHBA,SLFN12L,STAT5A |
| TRAF4 | other | | 2,41E-02 | CCL5,IFNB1,IL6,MYC,STEAP4 |
| RIPK3 | kinase | | 2,41E-02 | IL1A,IL1B,IL6,MLKL,TNF |
| RBP3 | transporter | | 2,41E-02 | ATF4,DDIT3,IL22RA1,RIPK1,TNF |
| NPFF | other | | 2,41E-02 | ADRB2,CEBPB,ID3,PIK3R1,SREBF2 |
| FPR2 | G-protein coupled receptor | | 2,41E-02 | CCL2,CSF1,CXCL8,IL12A,IL6 |
| ID4 | transcription regulator | | 2,41E-02 | BBC3,CDKN1A,CDKN2B,VEGFA,VIM |
| coomassie brilliant blue | chemical drug | | 2,41E-02 | IL1B,IL6,LIF,PTGS2,TNF |
| [illegible] | chemical drug | | 2,41E-02 | CYP2B6,HK2,IL1A,MYC,NAMPT |
| CpdX-D3 | chemical reagent | Inhibited | 2,41E-02 | IL1B,IL6,PTGS2,TNF,TSLP |
| N-acetylglucosamine | chemical - endogenous mammalian | | 2,41E-02 | IL6,PTGS2,TGFBR1,TNF,TNFRSF10B |
| miR-662 (miRNAs w/seed CCCACGU) | mature microRNA | | 2,41E-02 | [illegible] |
| miR-5588-3p (miRNAs w/seed AGUCCCA) | mature microRNA | Inhibited | 2,41E-02 | [illegible] |
| miR-514a-3p (and other miRNAs w/seed UUGACAC) | mature microRNA | Inhibited | 2,41E-02 | [illegible] |
| miR-4268 (miRNAs w/seed GCUCCUC) | mature microRNA | Inhibited | 2,43E-02 | [illegible] |
| miR-4780 (miRNAs w/seed CCCUAGA) | mature microRNA | | 2,43E-02 | [illegible] |
| LATS2 | kinase | | 2,43E-02 | BBC3,CCNB1,CD24,CDKN1A,CYP24A1,GATA3,LATS2,PLK1,SNAI1,TAZ |
| EIF3E | other | | 2,43E-02 | CD40,CXCL1,FARP1,FGF2,LIG1,SAA1,SMC2,SNAI1,TNC,ZEB2 |
| CEACAM1 | transporter | | 2,43E-02 | ANG,ANGPT2,CD40,CSF3,CXCL8,ICAM1,IL2RG,NOTCH3,VEGFA,VEGFC |
| ENG | transmembrane receptor | | 2,43E-02 | ACTA2,FN1,ITGA5,MMP1,MYC,NOS3,SERPINE1,SIRT1,SMAD6,TGFBR1 |
| anandamide | chemical - endogenous mammalian | | 2,43E-02 | [illegible] |
| CYP1A1 | enzyme | | 2,44E-02 | [illegible] |
| miR-550a-3-5p (and other miRNAs w/seed UGUGCCU) | mature microRNA | | 2,46E-02 | [illegible] |
| miR-6804-3p (miRNAs w/seed GCACCUG) | mature microRNA | Inhibited | 2,46E-02 | [illegible] |
| miR-6501-3p (miRNAs w/seed CAGAGCA) | mature microRNA | | 2,47E-02 | [illegible] |
| miR-6918-5p (and other miRNAs w/seed GCUGAGG) | mature microRNA | Inhibited | 2,47E-02 | [illegible] |
| miR-5093 (miRNAs w/seed GGAAAUG) | mature microRNA | | 2,49E-02 | [illegible] |
| miR-3562-3p (and other miRNAs w/seed UUCAGAU) | mature microRNA | | 2,50E-02 | [illegible] |
| FOLR1 | transporter | | 2,50E-02 | [illegible] |
| miR-6113 (and other miRNAs w/seed CAGAGGA) | mature microRNA | Inhibited | 2,51E-02 | [illegible] |
| miR-4457 (miRNAs w/seed CACAAGG) | mature microRNA | | 2,51E-02 | [illegible] |
| shikonin | chemical drug | | 2,52E-02 | ABCG1,CAT,CCL5,CXCL8,FASN,IL1B,TNF |
| paroxetine | chemical drug | | 2,52E-02 | [illegible] |
| miR-6126 (miRNAs w/seed CGGAAGG) | mature microRNA | | 2,53E-02 | [illegible] |
| miR-3152-5p (miRNAs w/seed UUGCCUC) | mature microRNA | | 2,54E-02 | [illegible] |
| miR-6872-3p (miRNAs w/seed CCAUGCC) | mature microRNA | | 2,54E-02 | [illegible] |
| miR-3473g (and other miRNAs w/seed AAAAGUG) | mature microRNA | | 2,54E-02 | [illegible] |
| miR-3681 (and other miRNAs w/seed GACCUGG) | mature microRNA | Inhibited | 2,54E-02 | [illegible] |
| [illegible] | group | | 2,56E-02 | BMP2,IRAK1,ITGB1,RUNX2,SERPINE1,SPP1 |
| RHOJ | enzyme | | 2,56E-02 | CAV1,GSN,MYH9,PLAT,PTN,RND3 |
| IL31 | other | Activated | 2,56E-02 | CCL2,CCNB1,JUNB,SOCS1,SOCS2,SOCS3 |
| CHEK1 | kinase | | 2,56E-02 | BCL2L11,BUB1B,CDKN1A,MCL1,MICB,MYC |
| SMURF1 | enzyme | | 2,56E-02 | CXCL10,IRF1,NR4A1,RUNX2,SMAD6,TRAF4 |
| NT5E | phosphatase | | 2,56E-02 | IL1B,IL6,MKI67,SERPINE1,SPP1,TNF |
| ACTB | other | | 2,56E-02 | AURKA,CCNB2,DSTN,IFI6,MYLK,STMN1 |
| [illegible] | kinase | | 2,56E-02 | CFLAR,GATA4,SERPINE1,TNF,TNFSF10,TNNC1 |
| thioridazine | chemical drug | | 2,56E-02 | CDKN1A,DEPP1,LSS,MCL1,SERPINA3,SLC2A3 |
| lycopene | chemical drug | | 2,56E-02 | CCND1,CD69,CDKN1A,KLF2,MMP7,PTGS2 |
| dichlororibofuranosylbenzimidazole | chemical toxicant | | 2,56E-02 | CDKN1A,CTSD,CXCL8,CYP1A1,CYP24A1,MYC |
| miR-142-5p (and other miRNAs w/seed AUAAAGU) | mature microRNA | | 2,56E-02 | [illegible] |
| miR-6786-5p (miRNAs w/seed CGGUGGG) | mature microRNA | | 2,56E-02 | [illegible] |
| NEDD9 | other | | 2,56E-02 | [illegible] |
| MEF2A | transcription regulator | | 2,57E-02 | [illegible] |
| GABPA | transcription regulator | | 2,57E-02 | [illegible] |
| miR-6165 (miRNAs w/seed AGCAGGA) | mature microRNA | Inhibited | 2,58E-02 | [illegible] |
| miR-5579-5p (miRNAs w/seed AUGGUAC) | mature microRNA | | 2,58E-02 | [illegible] |
| miR-6522 (and other miRNAs w/seed GACUCUG) | mature microRNA | | 2,58E-02 | [illegible] |
| miR-449c-3p (and other miRNAs w/seed AGCCACA) | mature microRNA | | 2,58E-02 | [illegible] |
| miR-7113-3p (miRNAs w/seed CUCCCUG) | mature microRNA | | 2,60E-02 | [illegible] |
| miR-2113 (miRNAs w/seed UUUGUGC) | mature microRNA | | 2,60E-02 | [illegible] |
| miR-5681a (and other miRNAs w/seed GAAAGGG) | mature microRNA | Inhibited | 2,60E-02 | [illegible] |
| miR-642a-3p (and other miRNAs w/seed GACACAU) | mature microRNA | Inhibited | 2,61E-02 | [illegible] |
| miR-4680-3p (miRNAs w/seed CUGAAUU) | mature microRNA | | 2,62E-02 | [illegible] |
| miR-4465-5p (miRNAs w/seed GAUUGUU) | mature microRNA | | 2,63E-02 | [illegible] |
| miR-1183 (miRNAs w/seed ACUGUAG) | mature microRNA | Inhibited | 2,63E-02 | [illegible] |
| cholic acid | chemical - endogenous mammalian | | 2,63E-02 | [illegible] |
| miR-6727-5p (miRNAs w/seed UCGGGGC) | mature microRNA | | 2,67E-02 | [illegible] |
| SETD2 | enzyme | | 2,68E-02 | ANG,CAV1,CDH2,CITED2,GNA13,JUN,MEF2C,QKI,SERPINE1 |
| sildenafil | chemical drug | | 2,68E-02 | BDNF,CEBPB,CFLAR,NOS3,PDE5A,PTGS2,TNF,VEGFA,VEGFC |
| methylnitrosourea | chemical toxicant | | 2,68E-02 | [illegible] |
| sodium orthovanadate | chemical reagent | | 2,68E-02 | [illegible] |
| andrographolide | chemical drug | | 2,68E-02 | CYP1A1,CYP2B6,FN1,GATA3,ICAM1,IL1B,IL6,PTGS2,TNF |
| choline | chemical - endogenous mammalian | | 2,68E-02 | BBC3,CDKN1A,COL8A1,DDIT3,ERRFI1,IGF2,PEMT,PTGS2,SPP1 |
| phosphate | chemical - endogenous mammalian | Activated | 2,69E-02 | [illegible] |
| trichloroethylene | chemical toxicant | | 2,69E-02 | [illegible] |
| miR-3195 (miRNAs w/seed GCGCCGG) | mature microRNA | | 2,69E-02 | [illegible] |
| miR-4263 (miRNAs w/seed UUCUAAG) | mature microRNA | | 2,69E-02 | [illegible] |
| miR-651-3p (miRNAs w/seed AAGGAAA) | mature microRNA | | 2,71E-02 | [illegible] |
| miR-3672 (and other miRNAs w/seed UGAGACU) | mature microRNA | | 2,72E-02 | [illegible] |
| miR-5590-5p (miRNAs w/seed UGCCAUA) | mature microRNA | Inhibited | 2,72E-02 | [illegible] |
| miR-3928-5p (and other miRNAs w/seed GAAGCUC) | mature microRNA | | 2,74E-02 | [illegible] |
| miR-5571-5p (miRNAs w/seed AAUUCUC) | mature microRNA | | 2,76E-02 | [illegible] |
| miR-4264 (miRNAs w/seed GGAGUCU) | mature microRNA | | 2,76E-02 | [illegible] |
| miR-556-3p (miRNAs w/seed UAUACC) | mature microRNA | | 2,76E-02 | [illegible] |
| PPIF | enzyme | | 2,77E-02 | [illegible] |
| CALCA | other | | 2,77E-02 | [illegible] |
| miR-4446-5p (miRNAs w/seed UUUCCCU) | mature microRNA | | 2,77E-02 | [illegible] |
| miR-4432 (miRNAs w/seed AAGACUC) | mature microRNA | | 2,78E-02 | [illegible] |
| miR-6776-5p (miRNAs w/seed CUGGGUG) | mature microRNA | | 2,78E-02 | [illegible] |
| miR-3108-3p (and other miRNAs w/seed GUCUGGG) | mature microRNA | | 2,79E-02 | [illegible] |
| miR-6827-5p (miRNAs w/seed GGGAGCC) | mature microRNA | Inhibited | 2,79E-02 | [illegible] |
| miR-6757-5p (miRNAs w/seed AGGGAUG) | mature microRNA | | 2,82E-02 | [illegible] |
| miR-3200-5p (miRNAs w/seed AUCUGAG) | mature microRNA | | 2,84E-02 | [illegible] |
| TCL1A | transcription regulator | | 2,85E-02 | [illegible] |
| cinnamaldehyde | chemical toxicant | | 2,85E-02 | [illegible] |
| aflatoxin B1 | chemical - endogenous non-mammalian | | 2,85E-02 | CD40,CYP1A1,IRS2,STAT5A |
| PARP | group | | 2,85E-02 | APOE,CDKN1A,SNAI1,VIM |
| ATP6AP2 | transporter | | 2,85E-02 | AGT,FN1,IL6,PTGS2 |
| LINC01139 | other | | 2,85E-02 | ANGPTL4,EGR1,LDHA,SLC16A3 |
| ganetespib | chemical drug | | 2,85E-02 | HLA-A,HLA-DQA1,MYC,STAT1 |
| CAVIN1 | transcription regulator | | 2,85E-02 | CDKN1A,IL6,MYC,TNF |
| cercosporin | chemical - endogenous non-mammalian | | 2,85E-02 | ACTA2,BIRC5,FN1,MYC |
| ADCY7 | enzyme | | 2,85E-02 | IRAK1,IRAK2,TLR1,TNF |
| TYRO3 | kinase | | 2,85E-02 | EGR2,IL1B,IL6,TNF |
| HSP90AA1 | enzyme | | 2,85E-02 | BIRC5,CFLAR,FN1,TCF12 |
| PDK2 | kinase | | 2,85E-02 | IL1B,IL6,IRF8,TNF |
| MIR320 | group | | 2,85E-02 | EDN1,ETS2,FN1,TFRC |
| mir-185 | microRNA | | 2,85E-02 | FDFT1,HMGCR,NTRK2,SREBF2 |
| RAMP1 | G-protein coupled receptor | | 2,85E-02 | IL6,TNF,VEGFA,VEGFC |
| UGDH | enzyme | | 2,85E-02 | FADS2,HAS2,SPRY2,TNC |
| HOXB3 | transcription regulator | | 2,85E-02 | CDCA3,EFNA1,HOXB7,HOXB8 |
| SETD7 | enzyme | | 2,85E-02 | CXCL10,MAFA,MYC,TNF |
| NPAT | transcription regulator | | 2,85E-02 | H2AC6,H2BC21,H2BC5,H3C14 |
| TEF | transcription regulator | | 2,85E-02 | AQP4,BCL2L14,MYLK,SCNN1A |
| AFF1 | transcription regulator | | 2,85E-02 | EPHA7,MYC,PTPRF,RPTOR |
| SH2B1 | other | | 2,85E-02 | CDH2,FOSL1,MYC,PLAUR |
| SLC4A1 | transporter | | 2,85E-02 | IL23A,IL6,STAT1,TNF |
| TP53BP2 | other | | 2,85E-02 | BBC3,FAS,VIM,ZEB1 |
| PCBP2 | other | | 2,85E-02 | IFNB1,MYC,STAT1,STAT2 |
| HDAC8 | transcription regulator | | 2,85E-02 | BBC3,CDKN1A,IFNB1,SOCS1 |
| diltiazem | chemical drug | | 2,85E-02 | EDN1,FOS,JUN,NOS3 |
| 2-hydroxy-1-naphthylaldehyde isonicotinoyl hydrazone | chemical reagent | | 2,85E-02 | CCNB1,CCND3,CDKN1A,GADD45A |
| [illegible] | chemical drug | | 2,85E-02 | BAK1,CDKN1A,EDN1,NOS3 |
| clomipramine | chemical drug | | 2,85E-02 | DEPP1,LSS,SERPINA3,SLC2A3 |
| (5-(4-N-methyl-N(2-pyridyl)amino)ethoxy)benzyl thiazolidine-2,4-dione | chemical reagent | | 2,85E-02 | CTSS,ITGAM,PC,SCD |
| tin mesoporphyrin | chemical drug | Activated | 2,85E-02 | ICAM1,IL1B,IL6,TNF |
| 2,4-dinitrobenzenesulfonic acid | chemical reagent | | 2,85E-02 | CTH,ICAM1,IL6,TNF |
| polymyxin B | biologic drug | | 2,85E-02 | CXCL8,IL1B,IL6,TNF |
| perillyl alcohol | chemical drug | | 2,85E-02 | CCNB1,CDKN1A,IL1B,NFKBIA |
| dydrogesterone | chemical drug | | 2,85E-02 | AKR1C1/AKR1C2,AKR1C3,HSD17B2,TNF |
| miR-802-5p (miRNAs w/seed CAGUAAC) | mature microRNA | | 2,86E-02 | [illegible] |
| miR-4788-5p (miRNAs w/seed UCGGUAU) | mature microRNA | | 2,86E-02 | [illegible] |
| zoledronic acid | chemical drug | | 2,86E-02 | [illegible] |
| semaxanib | chemical drug | | 2,86E-02 | AHR,CD24,CYP1B1,F3,IL6,NFE2L2,NOS3,NR4A2,PTGS2,S100P |
| miR-8063 (miRNAs w/seed CAAAAUC) | mature microRNA | | 2,89E-02 | [illegible] |
| miR-548d-3p (and other miRNAs w/seed AAAAACC) | mature microRNA | | 2,89E-02 | [illegible] |
| CTCF | transcription regulator | | 2,91E-02 | [illegible] |
| CASP3 | peptidase | | 2,92E-02 | BIRC5,CAPN2,CASP4,CDKN1A,IFNB1,MCL1,RUNX2,TNF |
| ZNF281 | transcription regulator | | 2,92E-02 | CCL2,CXCL2,GADD45A,GADD45G,IL11,IL6,PTGS1,PTGS2 |
| PRKCI | kinase | | 2,92E-02 | CXCL2,CXCL3,FASN,HK2,IL1A,IL6,PLK1,PTGS2 |
| mir-132 | microRNA | | 2,92E-02 | ARHGEF11,BDNF,CCL2,NR4A2,SOX4,STAT4,TNF,ZEB2 |
| mir-31 | microRNA | | 2,92E-02 | EGLN3,ITGA5,KRT17,NOS3,PRKCE,SATB2,SPP1,STMN1 |
| SFRP4 | transmembrane receptor | | 2,92E-02 | AREG,BIRC5,BTC,EREG,FN1,MYC,PTGS2,VIM |
| ferulic acid | chemical - endogenous non-mammalian | | 2,92E-02 | CAT,CDKN1A,CSF3,DPYSL2,IL1B,MKI67,MMP1,TNF |
| glycyrrhizic acid | chemical drug | | 2,92E-02 | ADRB2,AGER,CD40,EDN1,HMGB1,NOS3,PTGS2,TNF |
| miR-548ae-3p (miRNAs w/seed AAAACCC) | mature microRNA | | 2,92E-02 | [illegible] |
| miR-4278 (miRNAs w/seed UAGGGGG) | mature microRNA | Inhibited | 2,92E-02 | [illegible] |
| miR-5100 (miRNAs w/seed UCAGAUC) | mature microRNA | | 2,93E-02 | [illegible] |
| miR-4444 (miRNAs w/seed UCGAGUU) | mature microRNA | Inhibited | 2,93E-02 | [illegible] |
| miR-1269b-5p (and other miRNAs w/seed ACCCAGA) | mature microRNA | Inhibited | 2,94E-02 | [illegible] |
| miR-378b (and other miRNAs w/seed UGGACUU) | mature microRNA | | 2,94E-02 | [illegible] |
| miR-498-5p (miRNAs w/seed UUCAAGC) | mature microRNA | | 2,95E-02 | [illegible] |
| miR-8084 (miRNAs w/seed AAUACUA) | mature microRNA | | 2,96E-02 | [illegible] |
| miR-136-3p (and other miRNAs w/seed UCUGGGC) | mature microRNA | Inhibited | 2,97E-02 | [illegible] |
| UCHL1 | peptidase | | 2,98E-02 | [illegible] |
| miR-361-5p (miRNAs w/seed UAUCAGA) | mature microRNA | | 2,98E-02 | [illegible] |
| miR-5195-5p (miRNAs w/seed ACCCCUA) | mature microRNA | Inhibited | 2,99E-02 | [illegible] |
| 1,2-dioleoyloxy-3-(trimethylammonium)propane | chemical reagent | | 3,00E-02 | IFNB1,IL1B,IRF7 |
| ganciclovir | chemical drug | | 3,00E-02 | ARC,CDKN1A,TNF |
| [illegible] | chemical drug | | 3,00E-02 | IL1B,IL6,TNF |
| GPIIB-IIIA | complex | | 3,00E-02 | BCL3,CD40,PLAUR |
| Cobra Venom Factor | chemical - endogenous non-mammalian | | 3,00E-02 | CRP,FGF2,TGFB2 |
| [illegible] | chemical - endogenous non-mammalian | | 3,00E-02 | IL1B,IL6,PTGS2 |
| norethindrone acetate | chemical drug | | 3,00E-02 | F3,PLAT,SERPINE1 |
| Fcgr2 | group | | 3,00E-02 | AGER,CCL5,IL6 |
| mGLUR Group I | group | | 3,00E-02 | ARC,BDNF,FMR1 |
| CYP3A | group | | 3,00E-02 | HMGCS1,SQLE,SREBF2 |
| [illegible] | chemical drug | | 3,00E-02 | ARC,FOS,IGF2 |
| N-hexanoylhomoserine lactone | chemical - endogenous non-mammalian | | 3,00E-02 | CSF1,IL1A,IL1B |
| TLR2/TLR6 | group | | 3,00E-02 | IL23A,IL6,TNF |
| glycitein | chemical drug | | 3,00E-02 | IL1B,IL6,TNF |
| MN1 | other | | 3,00E-02 | BCL2L11,CYP24A1,TNFRSF11B |
| KLF17 | transcription regulator | | 3,00E-02 | CDKN1A,FN1,VIM |
| CLEC4A | transmembrane receptor | | 3,00E-02 | IL1B,IL6,TNF |
| CLEC4D | other | | 3,00E-02 | IL1B,IL6,TNF |
| CCN6 | growth factor | | 3,00E-02 | ICAM1,SNAI1,ZEB1 |
| [illegible] | enzyme | | 3,00E-02 | CXCL10,IFNB1,TLR3 |
| dynasore | chemical reagent | | 3,00E-02 | IFNB1,IL6,VEGFA |
| peptide T | biologic drug | | 3,00E-02 | ICAM1,ITGAV,TNF |
| RABGEF1 | enzyme | | 3,00E-02 | CXCL10,IFNB1,IL6 |
| [illegible] | enzyme | | 3,00E-02 | DDIT3,HSPA5,MAP1LC3B |
| SMPD2 | enzyme | | 3,00E-02 | IL1B,IL6,TNF |
| HSPB2 | other | | 3,00E-02 | CCL2,CXCL8,ICAM1 |
| FGF13 | growth factor | | 3,00E-02 | CDKN1A,HSPA5,VEGFA |
| PPP4C | phosphatase | | 3,00E-02 | CCL5,IFNB1,ISG15 |
| CNGB3 | ion channel | | 3,00E-02 | CAPN2,DDIT3,HSPA5 |
| [illegible] | transcription regulator | | 3,00E-02 | FGF2,TOP2A,VEGFA |
| CXCL5 | cytokine | | 3,00E-02 | ITGAM,SNAI1,TNF |
| ABCD2 | transporter | | 3,00E-02 | IL1B,PTGS2,TNF |
| PROK2 | other | | 3,00E-02 | FOS,IL1B,VEGFA |
| RALB | enzyme | | 3,00E-02 | BIRC5,CDKN1A,IFNB1 |
| BCL2L12 | other | | 3,00E-02 | BBC3,CDKN1A,TNFRSF10A |
| HAS1 | enzyme | | 3,00E-02 | CCNB1,CDC25C,HAS2 |
| mir-302 | microRNA | | 3,00E-02 | IL6,KPNA2,TNF |
| NUCB2 | other | | 3,00E-02 | FOS,IL6,PTGS2 |
| Irs3 | other | | 3,00E-02 | EGR1,FASN,IRS2 |
| [illegible] | transporter | | 3,00E-02 | DDIT3,HSPA5,XBP1 |
| MAGED1 | transcription regulator | | 3,00E-02 | CDKN1A,JUN,TNF |
| ETV5 | transcription regulator | | 3,00E-02 | MMP1,MYC,SPP1 |
| RNF111 | enzyme | | 3,00E-02 | CDKN2B,PMEPA1,SERPINE1 |
| PRSS8 | peptidase | | 3,00E-02 | IL1A,IL1B,TSLP |
| RYK | kinase | | 3,00E-02 | DLX2,FN1,RUNX2 |
| F13A1 | enzyme | | 3,00E-02 | IL1B,IL6,TNF |
| PTPRR | phosphatase | | 3,00E-02 | FOS,FOSL1,JUN |
| SRPK1 | kinase | | 3,00E-02 | CXCL10,MCL1,VEGFA |
| GRM5 | G-protein coupled receptor | | 3,00E-02 | FOS,SOD2,TNF |
| DYNLL1 | other | | 3,00E-02 | BCL2L11,CDKN1A,FOS |
| FSTL3 | other | | 3,00E-02 | CDKN1A,FST,SIRT1 |
| CCL15 | cytokine | | 3,00E-02 | CXCL8,ICAM1,IL6 |
| CFH | other | | 3,00E-02 | CRP,CXCL2,TNF |
| CTSK | peptidase | | 3,00E-02 | CTSV,IL6,PTGS2 |
| PPID | enzyme | | 3,00E-02 | SNAI1,VIM,ZEB1 |
| YWHAB | other | | 3,00E-02 | DUSP1,OPRK1,SCNN1A |
| RNF17 | other | | 3,00E-02 | MXD3,MXI1,SERPINA3 |
| TNFRSF14 | transmembrane receptor | | 3,00E-02 | F2RL1,MMP1,TNF |
| HIVEP2 | transcription regulator | | 3,00E-02 | FOS,GATA3,MYC |
| 19,20-epoxydocosapentaenoic acid | chemical - endogenous mammalian | | 3,00E-02 | ACTA2,SNAI1,ZEB2 |
| SERPIND1 | other | | 3,00E-02 | EGR1,IL1B,IL6 |
| SP1 | transcription regulator | | 3,00E-02 | ACTA2,FN1,ICAM1 |
| Ifna4 | other | | 3,00E-02 | IRF7,ISG15,TRIM25 |
| Hrg | other | | 3,00E-02 | ANGPT2,CCL2,CCL22 |
| [illegible] | other | | 3,00E-02 | CEACAM1,REL,VEGFA |
| VTX-2337 | chemical drug | | 3,00E-02 | IL1B,IL6,TNF |
| CXXC4 | other | | 3,00E-02 | CDKN1A,MMP7,TET2 |
| ZYX | other | | 3,00E-02 | FOS,ITGA5,ITGB1 |
| SAR-20347 | chemical - kinase inhibitor | | 3,00E-02 | IL23A,IL6,TNF |
| fenebrutinib | chemical drug | | 3,00E-02 | MCL1,MYC,POU2F2 |
| MIA-659 | chemical reagent | | 3,00E-02 | IL1B,NFKB2,PTGS2 |
| MIA-313 | chemical reagent | | 3,00E-02 | IL1B,NFKB2,PTGS2 |
| COG112 peptide | chemical reagent | | 3,00E-02 | CXCL2,CXCL3,TNF |
| ambroxol | chemical drug | | 3,00E-02 | CFTR,ICAM1,TNF |
| olmesartan medoxomil | chemical drug | | 3,00E-02 | DHFR,IL6,PTGS2 |
| XCT790 | chemical reagent | | 3,00E-02 | CDKN1A,MAOB,SOX9 |
| ferric nitrilotriacetate | chemical toxicant | | 3,00E-02 | ID3,KLF4,PTGS2 |

| | | | | |
|---|---|---|---|---|
| famotidine | chemical drug | | 3.00E-02 | CXCL2,ICAM1,TNF |
| emricasan | chemical drug | | 3.00E-02 | ACTA2,CXCL2,CXCL3 |
| 10-(6'-ubiquinonyl)decyltriphenylphosphonium | chemical drug | | 3.00E-02 | IL1B,IL6,NFE2L2 |
| papaverine | chemical drug | | 3.00E-02 | BDNF,CAV1,NTRK2 |
| catechol | chemical - endogenous mammalian | | 3.00E-02 | FOS,JUN,TNF |
| propyl gallate | chemical toxicant | | 3.00E-02 | CXCL8,PTGS2,TNF |
| FC-99 | chemical reagent | | 3.00E-02 | CD40,CD69,TNFSF13B |
| apamin | chemical toxicant | | 3.00E-02 | FOS,JUN,NOS3 |
| scopoletin | chemical - endogenous non-mammalian | | 3.00E-02 | FGF2,IL6,NOS3 |
| [illegible] | chemical reagent | | 3.00E-02 | ATF4,DDIT3,ERN1 |
| [illegible] | chemical reagent | | 3.00E-02 | ATF4,CASP8,ERN1 |
| N-retinylidene-N-retinylethanolamine | chemical - endogenous mammalian | | 3.00E-02 | AGER,DRAM1,VEGFA |
| palmitoylethanolamide | chemical drug | | 3.00E-02 | IL1B,TNF,TNFRSF1B |
| [illegible] | chemical drug | | 3.00E-02 | BCL2L11,CDKN1A,PMAIP1 |
| ginsenoside Rg1 | chemical - endogenous non-mammalian | | 3.00E-02 | BIRC5,NOS3,PTGS2 |
| miR-330-3p (and other miRNAs w/seed CAAAGCA) | mature microRNA | | 3.03E-02 | [illegible] |
| miR-5190 (miRNAs w/seed CAGUGAC) | mature microRNA | | 3.03E-02 | [illegible] |
| GHR | transmembrane receptor | | 3.04E-02 | ACTA2,CDKN1A,IGFBP4,IL1B,IL6,MOGAT1,MYC,NFKB1,SOCS2,SOCS3,SPP1,TNF |
| miR-6885-3p (miRNAs w/seed UUUGCUU) | mature microRNA | | 3.04E-02 | [illegible] |
| oxaliplatin | chemical drug | | 3.06E-02 | ANXA2,BAD,BBC3,BCL2L11,BIRC5,CDKN1A,CFLAR,FAS,GDF15,MMP7,PMAIP1,SAT1,STAT1,VIM |
| miR-3607-3p (miRNAs w/seed [illegible]) | mature microRNA | Inhibited | 3.07E-02 | [illegible] |
| miR-[illegible] (miRNAs w/seed [illegible]) | mature microRNA | | 3.08E-02 | [illegible] |
| miR-3691-3p (miRNAs w/seed CCAAGUC) | mature microRNA | | 3.08E-02 | [illegible] |
| miR-6767-3p (miRNAs w/seed CACGUGC) | mature microRNA | | 3.08E-02 | [illegible] |
| miR-4281 (miRNAs w/seed GGUCCCG) | mature microRNA | Inhibited | 3.08E-02 | [illegible] |
| miR-6868-5p (miRNAs w/seed CUGGCAG) | mature microRNA | Inhibited | 3.09E-02 | [illegible] |
| miR-2115-3p (miRNAs w/seed AUCAGAA) | mature microRNA | | 3.10E-02 | [illegible] |
| miR-3668 (miRNAs w/seed AUGUAGA) | mature microRNA | | 3.10E-02 | [illegible] |
| miR-1265 (miRNAs w/seed AGGAUGU) | mature microRNA | | 3.10E-02 | [illegible] |
| miR-26b-3p (and other miRNAs w/seed CUGUUCU) | mature microRNA | Inhibited | 3.12E-02 | [illegible] |
| E2F6 | transcription regulator | | 3.13E-02 | CDKN1A,DHFR,DIRAS3,DUSP10,MYC,SERPINB2,SERPINE1 |
| SOX6 | transcription regulator | | 3.13E-02 | CDKN1A,FGFR3,MYC,MYL9,RUNX2,SOCS3,TNNC1 |
| HEXIM1 | transcription regulator | | 3.13E-02 | ACE2,BBC3,CDKN1A,FGF2,LOXL2,TGFB2,VIM |
| miR-6886-3p (miRNAs w/seed [illegible]) | mature microRNA | | 3.14E-02 | [illegible] |
| miR-1226-5p (miRNAs w/seed [illegible]) | mature microRNA | | 3.14E-02 | [illegible] |
| RARG | ligand-dependent nuclear receptor | | 3.15E-02 | [illegible] |
| miR-[illegible] (miRNAs w/seed [illegible]) | mature microRNA | Inhibited | 3.16E-02 | [illegible] |
| [illegible] | [illegible] | [illegible] | [illegible] | [illegible] |

| | | | | |
|---|---|---|---|---|
| TPSG1 | peptidase | | 4,39E-02 | CXCL3,IL6 |
| COL3A1 | other | | 4,39E-02 | FN1,IL1RN |
| EMP2 | other | | 4,39E-02 | CAV1,ITGA5 |
| BAG4 | other | | 4,39E-02 | IL1B,IL6 |
| PURB | transcription regulator | | 4,39E-02 | ACTA2,MYC |
| 17alpha-hydroxyprogesterone caproate | chemical drug | | 4,39E-02 | PTGS2,VEGFA |
| GSTA4 | enzyme | | 4,39E-02 | CCNB1,CDC25C |
| CLIP1 | other | | 4,39E-02 | IL6,TNF |
| LDAF2 | other | | 4,39E-02 | BBC3,CDKN1A |
| LYZ | enzyme | | 4,39E-02 | IL1B,LYZ |
| PKC delta V1-1 peptide 8-17 | chemical - kinase inhibitor | | 4,39E-02 | CCL2,CXCL3 |
| HAO1 | enzyme | | 4,39E-02 | HSPA5,PPARGC1A |
| CASP7 | peptidase | | 4,39E-02 | CASP4,IFNB1 |
| MADD | other | | 4,39E-02 | MLPH,TNF |
| riociguat | chemical drug | | 4,39E-02 | SERPINE1,SPP1 |
| 9-benzyl-8-hydroxy-2-(2-methoxyethoxy)adenine | chemical reagent | | 4,39E-02 | IL6,IRF7 |
| SGCA | other | | 4,39E-02 | SGCB,SSPN |
| TRIM8 | enzyme | | 4,39E-02 | IFIT2,IFNB1 |
| PBK | kinase | | 4,39E-02 | BIRC3,NFKB1 |
| CARD8 | other | | 4,39E-02 | BIRC2,IL1B |
| KLF16 | transcription regulator | | 4,39E-02 | CDKN1A,CYP1A1 |
| SGI 1776 | chemical drug | | 4,39E-02 | MCL1,MYC |
| CEACAM6 | other | | 4,39E-02 | IL1B,IL6 |
| SERPINB3 | other | | 4,39E-02 | IL6,LRP1 |
| CEACAM21 | other | | 4,39E-02 | IL1B,IL6 |
| DOK2 | other | | 4,39E-02 | MYC,TNF |
| IFI30 | enzyme | | 4,39E-02 | CTSS,HLA-A |
| EIF2B1 | translation regulator | | 4,39E-02 | ATF4,DDIT3 |
| STK16 | kinase | | 4,39E-02 | SERPINE1,VEGFA |
| MID2 | other | | 4,39E-02 | IFIT2,IFNB1 |
| GZMA | peptidase | | 4,39E-02 | CCL2,CXCL8 |
| EID2 | other | | 4,39E-02 | CDKN1A,CDKN2B |
| PPP1R1A | phosphatase | | 4,39E-02 | ERN1,HSPA5 |
| MKNK2 | kinase | | 4,39E-02 | IFIT2,ISG15 |
| MLST8 | other | | 4,39E-02 | IFIT2,ISG15 |
| FCAR | transmembrane receptor | | 4,39E-02 | IL6,TNF |
| TOR3A | other | | 4,39E-02 | FOS,MYC |
| DSG1 | other | | 4,39E-02 | TNF,TSLP |
| IFNGR2 | transmembrane receptor | | 4,39E-02 | GBP5,IRF1 |
| STAG2 | other | | 4,39E-02 | CD69,TNF |
| RGS3 | enzyme | | 4,39E-02 | MYC,SNAI1 |
| GC | transporter | | 4,39E-02 | CYP24A1,CYP27B1 |
| PDZD2 | other | | 4,39E-02 | BAD,CDKN1A |
| Defb2 | other | | 4,39E-02 | CD40,TNFRSF9 |
| CLIC1 | ion channel | | 4,39E-02 | IL1B,TNF |
| KRT5 | other | | 4,39E-02 | ICAM1,VEGFA |
| Trim30a/Trim30d | other | | 4,39E-02 | IL6,TNF |
| Pkhd1 | other | | 4,39E-02 | ANG,VEGFA |
| USP27X | peptidase | | 4,39E-02 | CGAS,IFNB1 |
| CEACAM7 | other | | 4,39E-02 | IL1B,IL6 |
| ELOA | transcription regulator | | 4,39E-02 | ATF3,CDKN1A |
| YWHAE | other | | 4,39E-02 | FOSL1,MMP1 |
| BTG1 | transcription regulator | | 4,39E-02 | JUN,NR3C1 |
| CPB2 | enzyme | | 4,39E-02 | IL6,MMP1 |
| IL20RA | transmembrane receptor | | 4,39E-02 | CXCL8,ICAM1 |
| Mal | other | | 4,39E-02 | ICAM1,TNF |
| URMC-099 | chemical reagent | | 4,39E-02 | IL6,TNF |
| VEZF1 | transcription regulator | | 4,39E-02 | EDN1,USP18 |
| CASP8AP2 | transcription regulator | | 4,39E-02 | CFLAR,MCL1 |
| RAN | enzyme | | 4,39E-02 | MCL1,TNF |
| TCP1 | other | | 4,39E-02 | BBC3,CDKN1A |
| CASP10 | peptidase | | 4,39E-02 | IL6,TNF |
| RING1 | transcription regulator | | 4,39E-02 | FOS,JUN |
| Acp5 | phosphatase | | 4,39E-02 | IL1B,TNF |
| CRIP2 | other | | 4,39E-02 | CXCL8,IL6 |
| Irs4 | other | | 4,39E-02 | EGR1,IRS2 |
| SRSF6 | other | | 4,39E-02 | NR3C1,VEGFA |
| NKRF | transcription regulator | | 4,39E-02 | CXCL8,IFNB1 |
| TIGAR | enzyme | | 4,39E-02 | CCL2,TNFAIP3 |
| BUB1B | kinase | | 4,39E-02 | CDC20,PTTG1 |
| UIMC1 | other | | 4,39E-02 | BBC3,CDKN1A |
| CNTFR | transmembrane receptor | | 4,39E-02 | CXCL8,IL1A |
| proTAME | chemical reagent | | 4,39E-02 | CCNB1,PTTG1 |
| dinitrophenyl-R group | chemical reagent | | 4,39E-02 | IL6,TGM2 |
| AZD-1208 | chemical drug | | 4,39E-02 | CD38,SELL |
| mercaptodecenol 4 | chemical reagent | | 4,39E-02 | CCL2,CCL20 |
| mercaptodecenol 6 | chemical reagent | | 4,39E-02 | CCL2,CCL20 |
| P2pal-18S | chemical reagent | | 4,39E-02 | IL6,TNF |
| C20-D3-vitamin A | chemical drug | | 4,39E-02 | C3,CFB |
| beta2 adrenergic receptor agonist | chemical drug | | 4,39E-02 | CDKN1A,TNF |
| N6-carboxymethyl-lysine bovine serum albumin | chemical reagent | | 4,39E-02 | CCL20,SERPINB2 |
| temocapril | chemical reagent | | 4,39E-02 | NOS3,PTGS2 |
| nordihydrocapsiate | chemical reagent | | 4,39E-02 | CD69,ICAM1 |
| methoxyacetic acid | chemical toxicant | | 4,39E-02 | PDE4D,PLK1 |
| nitrous oxide | chemical drug | | 4,39E-02 | FOS,TNF |
| mecamylamine | chemical drug | | 4,39E-02 | BDNF,FOS |
| amantadine | chemical drug | | 4,39E-02 | DDC,FOS |
| pinacidil | chemical drug | | 4,39E-02 | CXCL2,IL1B |
| filipin | chemical - endogenous non-mammalian | | 4,39E-02 | CXCL8,SERPINE1 |
| astemizole | chemical drug | | 4,39E-02 | CCL2,IL6 |
| ODN1411 | chemical reagent | | 4,39E-02 | CXCL10,TNF |
| beta-lapachone | chemical drug | | 4,39E-02 | NOS3,TNFRSF10B |
| phosphorylcholine | chemical - endogenous mammalian | | 4,39E-02 | IL1B,TNF |
| triphenyltin | chemical toxicant | | 4,39E-02 | IL1B,IL6 |
| albendazole | chemical drug | | 4,39E-02 | ICAM1,VEGFA |
| 10-hydroxy docosahexaenoic acid | chemical - endogenous mammalian | | 4,39E-02 | IL6,TNF |
| cycloserine | chemical drug | | 4,39E-02 | BDNF,FOS |
| pegaptanib | biologic drug | | 4,39E-02 | FGF2,IL6 |
| oseltamivir | chemical drug | | 4,39E-02 | IL6,TNF |
| K-ATP Channel | complex | | 4,39E-02 | PPARGC1A,SCNN1A |
| di-2-pyridylketone 4-cyclohexyl-4-methyl-3-thiosemicarbazone | chemical drug | | 4,39E-02 | ERRFI1,NDRG1 |
| [illegible] | chemical drug | | 4,39E-02 | JUNB,NGFR |
| minodronate | chemical drug | | 4,39E-02 | CCL2,ICAM1 |
| fosbretabulin | chemical drug | | 4,39E-02 | CCNB1,CSF3 |
| ketotifen | chemical drug | | 4,39E-02 | IL6,TNF |
| modafinil | chemical drug | | 4,39E-02 | BDNF,FOS |
| AX15839 | chemical - kinase inhibitor | | 4,39E-02 | CXCL8,IL6 |
| 10-decarbamoylmitomycin C | chemical toxicant | | 4,39E-02 | CDKN1A,GADD45A |
| [illegible] | chemical drug | | 4,39E-02 | IL6,LIF |
| idronoxil | chemical drug | | 4,39E-02 | CDKN1A,CFLAR |
| MLN-4760 | chemical - protease inhibitor | | 4,39E-02 | CXCL2,CXCL3 |
| 4-amino-6-hydrazino-7-beta-D-ribofuranosyl-7H-pyrrolo[2,3-d]pyrimidine-5-carboxamide | chemical reagent | | 4,39E-02 | BIRC5,MCL1 |
| TTI-101 | chemical drug | | 4,39E-02 | ACTA2,SERPINE1 |
| MitoBloCK-6 | chemical drug | | 4,39E-02 | ASNS,PSPH |
| 5,10,15,20-tetrakis(4-sulfonatophenyl)porphyrinato iron (III) | chemical reagent | | 4,39E-02 | HMGB1,ICAM1 |
| BRP110 | chemical reagent | | 4,39E-02 | DDC,GCH1 |
| yoda1 | chemical reagent | | 4,39E-02 | CD69,IL6 |
| potassium tetraperoxochromate | chemical reagent | | 4,39E-02 | IL1B,TNF |
| aripiprazole | chemical drug | | 4,39E-02 | BDNF,TNF |
| N(1)-guanyl-1,7-diaminoheptane | chemical reagent | | 4,39E-02 | CD83,MVK |
| NC9 | chemical reagent | | 4,39E-02 | ITGB4,LATS1 |
| actinomycin | chemical - endogenous non-mammalian | | 4,39E-02 | IGFBP4,SAA1 |
| bacitracin | biologic drug | | 4,39E-02 | IL23A,TNF |
| glutathione ethyl ester | biologic drug | | 4,39E-02 | MCL1,MMP1 |
| tenidap | chemical drug | | 4,39E-02 | IL6,PTGS2 |
| (indol-3-yl)pyruvic acid | chemical - endogenous non-mammalian | | 4,39E-02 | CYP1A1,IL1B |
| ellipticine | chemical reagent | | 4,39E-02 | CD40,IL1B |
| physostigmine | chemical drug | | 4,39E-02 | ACHE,ITGAM |
| strychnine | chemical drug | | 4,39E-02 | IL1B,TNF |
| aluminum | chemical drug | | 4,39E-02 | IL1B,RUNX2 |
| SR 31747 | chemical drug | | 4,39E-02 | IL6,TNF |
| danazol | chemical drug | | 4,39E-02 | CXCL8,IL6 |
| epiallopregnanolone | chemical - endogenous mammalian | | 4,39E-02 | PTGS2,TNF |
| L-alpha-hydroxyglutarate | chemical - endogenous mammalian | | 4,39E-02 | IL7R,SELL |
| miR-650a-1-3p (and other miRNAs w/seed UUGGGAA) | mature microRNA | | 4,41E-02 | [illegible] |
| EOMES | transcription regulator | | 4,43E-02 | [illegible] |
| miR-138-1-3p (miRNAs w/seed CUACUUC) | mature microRNA | | 4,44E-02 | [illegible] |
| miR-6889-5p (and other miRNAs w/seed AGAGGAA) | mature microRNA | Inhibited | 4,45E-02 | [illegible] |
| miR-3064a-3p (and other miRNAs w/seed UCUGCCA) | mature microRNA | Inhibited | 4,45E-02 | [illegible] |
| miR-580-3p (miRNAs w/seed UGAGAAU) | mature microRNA | | 4,45E-02 | [illegible] |
| miR-1264 (and other miRNAs w/seed AAGUCUU) | mature microRNA | | 4,46E-02 | [illegible] |
| 2-methoxyestradiol | chemical - endogenous mammalian | | 4,48E-02 | AREG,ATF3,BCL2L11,CAV1,F3,IL6,ITGB8,MCL1,PTGS2,TNF |
| miR-6793-5p (miRNAs w/seed GUGGGUU) | mature microRNA | Inhibited | 4,48E-02 | [illegible] |
| miR-10400-5p (and other miRNAs w/seed GGCGGCG) | mature microRNA | Inhibited | 4,50E-02 | [illegible] |
| Hedgehog | group | | 4,50E-02 | BDNF,COMMD3-BMI1,GATA4,GATA6,IGF2,KLF4,MAFA,MYC,MYCL,SOX9,SPP1 |
| TFE3 | transcription regulator | Activated | 4,50E-02 | ELOVL3,FASN,HK2,INSIG1,IRS2,KLF2,MT-ND1,MYH9,PPARGC1A,SCD,SERPINE1 |
| miR-487a-5p (and other miRNAs w/seed UGGUUAU) | mature microRNA | Inhibited | 4,51E-02 | [illegible] |
| miR-4764-5p (miRNAs w/seed GGAUGUG) | mature microRNA | Inhibited | 4,52E-02 | [illegible] |
| sodium chloride | chemical - endogenous mammalian | | 4,52E-02 | [illegible] |
| miR-28-3p (and other miRNAs w/seed ACUAGAU) | mature microRNA | Inhibited | 4,55E-02 | [illegible] |
| miR-1322 (miRNAs w/seed AUGAUGC) | mature microRNA | Inhibited | 4,58E-02 | [illegible] |
| miR-6070 (miRNAs w/seed CGGUUCC) | mature microRNA | | 4,60E-02 | [illegible] |
| miR-6869 (miRNAs w/seed GUGUCCG) | mature microRNA | | 4,61E-02 | [illegible] |
| miR-6877-5p (miRNAs w/seed GGGCCGA) | mature microRNA | | 4,61E-02 | [illegible] |
| miR-6776-3p (miRNAs w/seed AACCACC) | mature microRNA | | 4,62E-02 | [illegible] |
| miR-4423-3p (miRNAs w/seed UAGGCAC) | mature microRNA | Inhibited | 4,63E-02 | [illegible] |
| picrotoxin | chemical toxicant | | 4,63E-02 | ADRA2A,ADRB2,CYP1A1,FOS,OPRK1,PDE4D,PRKCE |
| IL17C | cytokine | Activated | 4,63E-02 | CCL20,CXCL8,IL1A,IL1B,IL23A,IL6,TNF |
| miR-1263 (miRNAs w/seed GAGAAGA) | mature microRNA | | 4,65E-02 | [illegible] |
| miR-219a-3p (miRNAs w/seed GAAUUGC) | mature microRNA | | 4,66E-02 | [illegible] |
| miR-5196-3p (miRNAs w/seed CAUCCUC) | mature microRNA | | 4,66E-02 | [illegible] |
| miR-6511 (miRNAs w/seed AAGAACU) | mature microRNA | | 4,67E-02 | [illegible] |
| TFAP2C | transcription regulator | | 4,67E-02 | [illegible] |
| cadmium | chemical toxicant | Activated | 4,67E-02 | [illegible] |
| miR-1247-5p (miRNAs w/seed CCCGUCC) | mature microRNA | | 4,69E-02 | [illegible] |
| miR-4768-5p (miRNAs w/seed CGGUCCC) | mature microRNA | Inhibited | 4,70E-02 | [illegible] |
| miR-6529-3p (and other miRNAs w/seed CUGUGCC) | mature microRNA | | 4,72E-02 | [illegible] |
| miR-6843-3p (and other miRNAs w/seed UGGUCUC) | mature microRNA | | 4,73E-02 | [illegible] |
| miR-23a-5p (and other miRNAs w/seed GGGUUCC) | mature microRNA | | 4,73E-02 | [illegible] |
| FMR1 | translation regulator | | 4,74E-02 | [illegible] |
| phosphatidylinositol | chemical - endogenous mammalian | | 4,77E-02 | CXCL8,MMP1,TNF |
| dopamine D1 receptor | group | | 4,77E-02 | BDNF,FOS,FOSB |
| CXCL6 | cytokine | | 4,77E-02 | IL1B,IL6,TNF |
| Cd209e | other | | 4,77E-02 | IL1B,IL6,TNF |
| Syk | group | | 4,77E-02 | CCL2,IL6,TNF |
| doramapimod | chemical - kinase inhibitor | | 4,77E-02 | HAS2,IL6,TNF |
| blinatumomab | biologic drug | | 4,77E-02 | CCL2,CXCL8,IL6 |
| rh-endostatin | chemical drug | | 4,77E-02 | FAS,JUN,MAP1LC3B |
| PRCP | peptidase | | 4,77E-02 | KLF2,KLF4,NOS3 |
| ISX | transcription regulator | | 4,77E-02 | ALDH1A2,BCO1,SNAI1 |
| MRPS18B | other | | 4,77E-02 | ACTA2,FUT4,VIM |
| PIMREG | other | | 4,77E-02 | IL6,SOCS3,TNF |
| EIF2A | translation regulator | | 4,77E-02 | ATF4,DDIT3,HSPA5 |
| ZNF652 | other | | 4,77E-02 | TCF12,TGFB2,VIM |
| SH2D5 | other | | 4,77E-02 | BIRC5,IL6,MCL1 |
| AKIRIN2 | other | | 4,77E-02 | DUSP1,IL6,MYC |
| PTK | group | | 4,77E-02 | IL6,NR3C1,TNF |
| CC2D1A | transcription regulator | | 4,77E-02 | CCL5,IFNB1,IL6 |
| CXCL17 | cytokine | | 4,77E-02 | IL12A,IL23A,TNF |
| KMT5B | enzyme | | 4,77E-02 | DUSP1,MT2A,SGK1 |
| SFRP5 | transmembrane receptor | | 4,77E-02 | CCL2,IL1B,TNF |
| EHT-1864 | chemical reagent | | 4,77E-02 | ACTA2,FN1,FOXO1 |
| saracatinib | chemical drug | | 4,77E-02 | IL1B,NLRP3,PLCG2 |
| valine | chemical - endogenous mammalian | | 4,77E-02 | CD83,CDKN1A,SOD2 |
| LAX1 | other | | 4,77E-02 | IL6,LAT2,TNF |
| sotrastaurin | chemical drug | | 4,77E-02 | CD69,NFKBIA,PRKCQ |
| catumaxomab | biologic drug | | 4,77E-02 | CD40,CD69,TNFSF10 |
| nadifloxacin | chemical drug | | 4,77E-02 | CXCL8,IL1A,IL6 |
| Sfk | group | | 4,77E-02 | CDCP1,ICAM1,IL1B |
| Collagen type III | complex | | 4,77E-02 | ITGA5,ITGAV,ITGB1 |
| PDGFD | growth factor | | 4,77E-02 | MMP1,PTGS2,VEGFA |
| idelalisib | chemical drug | | 4,77E-02 | MCL1,MYC,SELL |
| IER3 | other | | 4,77E-02 | BIRC2,BIRC3,MCL1 |
| SELL | transmembrane receptor | | 4,77E-02 | IL1B,IL6,TNF |
| PDE6B | enzyme | | 4,77E-02 | CAT,FOS,NFE2L2 |
| EZH1 | enzyme | | 4,77E-02 | IFNB1,IL6,TNF |
| AGR2 | other | | 4,77E-02 | AREG,EGR1,FOS |
| PTPRB | phosphatase | | 4,77E-02 | ANGPT2,EDN1,FOXO1 |
| DAB2 | other | | 4,77E-02 | FOS,HMGCR,LRP2 |
| ENO1 | enzyme | | 4,77E-02 | EDN1,MYC,PTGS2 |
| RBX1 | enzyme | | 4,77E-02 | BIRC5,CCNB1,MCL1 |
| TDO2 | enzyme | | 4,77E-02 | AHR,PTGS2,SLC1A4 |
| CYP4F2 | enzyme | | 4,77E-02 | IL6,NCF2,VEGFA |
| P2RY1 | G-protein coupled receptor | | 4,77E-02 | FN1,FOS,IL6 |
| TPSD1 | peptidase | | 4,77E-02 | CXCL8,IL6,PTGS2 |
| MGLL | enzyme | | 4,77E-02 | IL1A,IL6,TNF |
| PIP5K1C | kinase | | 4,77E-02 | CD274,ERRFI1,PIP5K1A |
| MAD2L2 | enzyme | | 4,77E-02 | CDH2,EGR1,VIM |
| CHGA | other | | 4,77E-02 | BIRC5,HSPA5,IL1B |
| TNKS | enzyme | | 4,77E-02 | MCL1,MYC,SIRT1 |
| ERBIN | other | | 4,77E-02 | CDKN1A,CDKN2B,SERPINE1 |
| CCR4 | G-protein coupled receptor | | 4,77E-02 | CCL22,CXCL3,FOS |
| NKX3-2 | transcription regulator | | 4,77E-02 | FGFR3,RUNX2,SOX9 |
| TRPA1 | transporter | | 4,77E-02 | CXCL3,IL1B,IL6 |
| MS4A1 | other | | 4,77E-02 | CD69,MCL1,MYC |
| ITGB8 | other | | 4,77E-02 | APOE,CDKN1A,ITGB5 |
| FOXN3 | transcription regulator | | 4,77E-02 | CDH2,CFAP36,GCK |
| GNMT | enzyme | | 4,77E-02 | ABCG1,CYP1A1,NPC2 |
| Pde4d | enzyme | | 4,77E-02 | CTSV,PTGS2,TNF |
| PDE4D | enzyme | | 4,77E-02 | BCL2L11,FN1,PLAT |
| RHOC | enzyme | | 4,77E-02 | ATF3,CDKN1A,DDIT3 |
| BC-1215 | chemical reagent | | 4,77E-02 | TRAF1,TRAF3,TRAF6 |
| CB-5083 | chemical drug | | 4,77E-02 | BIK,DDIT3,HSPA5 |
| nicorandil | chemical drug | | 4,77E-02 | ATF3,JUN,NOS3 |
| dimercaptopropanol | chemical drug | | 4,77E-02 | CRYZ,CXCL2,TNF |
| aminoglutethimide | chemical drug | | 4,77E-02 | HMGCR,HSD17B7,SPP1 |
| trimethyltin | chemical reagent | | 4,77E-02 | FOS,IL6,TNF |
| hydroxychloroquine | chemical drug | | 4,77E-02 | CD69,CFLAR,IL6 |
| nigericin | chemical drug | | 4,77E-02 | CASP1,EIF2AK2,IL1B |
| zimelidine | chemical drug | | 4,77E-02 | ASAH1,DEPP1,SERPINA3 |
| poly rI:rC-RNA HMW | chemical reagent | | 4,77E-02 | EIF2AK2,IFNB1,IL6 |
| butylidenephthalide | chemical reagent | | 4,77E-02 | CDH2,SNAI1,VIM |
| diacerein | chemical drug | | 4,77E-02 | IL1B,IL6,TNF |
| polyphosphate | chemical - endogenous mammalian | | 4,77E-02 | BMP2,ICAM1,SPP1 |
| BAY601 | chemical reagent | | 4,77E-02 | IL1B,IL6,TNF |
| [illegible] | chemical reagent | | 4,77E-02 | CD40,CD83,CEBPB |
| cyclosporin | biologic drug | | 4,77E-02 | CXCL8,ICAM1,LAMA3 |
| geranylgeraniol | chemical - endogenous mammalian | | 4,77E-02 | HMGCR,MCL1,MMP1 |
| atropine | chemical drug | | 4,77E-02 | FOS,PTGS2,VEGFA |
| vinorelbine | chemical drug | | 4,77E-02 | CCL8,PMAIP1,TYMP |
| tyrphostin AG825 | chemical - kinase inhibitor | | 4,77E-02 | CDKN1A,LMO4,SNAI1 |
| phosphatidic acid | chemical - endogenous mammalian | | 4,77E-02 | IGF2,MYC,PTGS2 |
| 12-hydroxyeicosatetraenoic acid | chemical - endogenous mammalian | | 4,77E-02 | ICAM1,PTGS2,VEGFA |
| noladin ether | chemical - endogenous mammalian | | 4,77E-02 | CYP27A1,IL6,NFKB1 |
| S-allyl-L-cysteine | chemical - endogenous non-mammalian | | 4,77E-02 | ACTA2,FASN,PTGS2 |
| Muscarinic cholinergic receptor | group | | 4,77E-02 | FMR1,FOS,JUN |
| miR-6716-5p (miRNAs w/seed GGGAAUG) | mature microRNA | Inhibited | 4,77E-02 | [illegible] |
| miR-7109-3p (miRNAs w/seed AAGCCUC) | mature microRNA | | 4,78E-02 | [illegible] |
| miR-6770-3p (miRNAs w/seed UGGCGGC) | mature microRNA | | 4,79E-02 | [illegible] |
| miR-6828-3p (miRNAs w/seed UCUGCUC) | mature microRNA | Inhibited | 4,81E-02 | [illegible] |
| miR-3110-5p (and other miRNAs w/seed UCUGCCU) | mature microRNA | | 4,81E-02 | [illegible] |
| miR-6502-3p (miRNAs w/seed AGACCAU) | mature microRNA | | 4,81E-02 | [illegible] |
| ANXA7 | ion channel | | 4,81E-02 | [illegible] |
| miR-181a-1-3p (and other miRNAs w/seed CCAUCGA) | mature microRNA | Inhibited | 4,84E-02 | [illegible] |
| POMC | other | Activated | 4,84E-02 | [illegible] |
| miR-6884-5p (and other miRNAs w/seed GGAGGAU) | mature microRNA | | 4,84E-02 | [illegible] |
| miR-7110-3p (miRNAs w/seed CUCUCUC) | mature microRNA | Inhibited | 4,84E-02 | [illegible] |
| miR-764 (miRNAs w/seed CAGGUGC) | mature microRNA | | 4,86E-02 | [illegible] |
| miR-647 (miRNAs w/seed UGGCUGC) | mature microRNA | | 4,86E-02 | [illegible] |
| bisphenol A | chemical - endogenous mammalian | | 4,91E-02 | [illegible] |
| miR-548q (miRNAs w/seed CUGGUGC) | mature microRNA | | 4,91E-02 | [illegible] |
| miR-578 (and other miRNAs w/seed UUCUUGU) | mature microRNA | | 4,91E-02 | [illegible] |
| miR-4486 (miRNAs w/seed CUGGGCG) | mature microRNA | Inhibited | 4,92E-02 | [illegible] |
| miR-1227-5p (miRNAs w/seed UGGGGCC) | mature microRNA | | 4,92E-02 | [illegible] |
| BMS-690514 | chemical drug | | 4,93E-02 | BBC3,BCL2L11,CCNB1,CCND3,CTSH,DDIT3,MCL1,MCM6 |
| HSD11B1 | enzyme | | 4,93E-02 | AGT,ANGPTL4,CCL2,FASN,IL6,PPARGC1A,TNF,UCP2 |
| CR1L | other | Inhibited | 4,93E-02 | C3,COL4A1,COL8A1,IL1B,PTGER4,SERPINE1,TNC,TNF |
| miR-3669 (miRNAs w/seed GGGACCU) | mature microRNA | | 4,93E-02 | [illegible] |
| NPY | other | Activated | 4,94E-02 | [illegible] |
| miR-3614-3p (miRNAs w/seed AGCCUUC) | mature microRNA | | 4,94E-02 | [illegible] |
| miR-4428 (miRNAs w/seed AAGGAGA) | mature microRNA | | 4,94E-02 | [illegible] |
| miR-20a-3p (miRNAs w/seed CUGCAUU) | mature microRNA | Inhibited | 4,96E-02 | [illegible] |
| miR-6506-3p (miRNAs w/seed CGUAUCA) | mature microRNA | Inhibited | 4,98E-02 | [illegible] |
| miR-376a-3p (and other miRNAs w/seed CUGGACU) | mature microRNA | Inhibited | 4,98E-02 | [illegible] |
| miR-33-3p (and other miRNAs w/seed AAUGUUU) | mature microRNA | | 5,00E-02 | [illegible] |

**Supplementary Table S14. List of Upstream Regulators for the condition "Calu-3_SARS-CoV".** The IPA-based Core Analysis was performed on the basis of differentially expressed genes discerned for a given condition by GENEVESTIGATOR-based analysis. Only statistically significant Upstream Regulators are included (n = [illegible]). The data are taken as the basis for R-based visualization by Venn diagrams shown in Figure [illegible].

| Upstream Regulator | Molecule Type | Predicted Activation State | p-value of overlap | Target Molecules in Dataset |
|---|---|---|---|---|
| interferon alpha | group | Activated | 2,61E-74 | [illegible] |
| lipopolysaccharide | chemical drug | Activated | 2,27E-73 | [illegible] |
| poly rI:rC-RNA | biologic drug | Activated | 4,68E-73 | [illegible] |
| TNF | cytokine | Activated | 1,24E-63 | [illegible] |
| IFNG | cytokine | Activated | 1,74E-63 | [illegible] |
| IFNA2 | cytokine | Activated | 2,61E-61 | [illegible] |
| IFNL1 | cytokine | Activated | [illegible] | [illegible] |
| IL1B | cytokine | Activated | [illegible] | [illegible] |
| resiquimod | chemical drug | Activated | [illegible] | [illegible] |
| MAPK1 | kinase | Inhibited | [illegible] | [illegible] |
| STAT1 | transcription regulator | Activated | [illegible] | [illegible] |
| IRF7 | transcription regulator | Activated | [illegible] | [illegible] |
| STAT3 | transcription regulator | Activated | [illegible] | [illegible] |
| NFkB (complex) | complex | Activated | [illegible] | [illegible] |
| CD40LG | cytokine | Activated | [illegible] | [illegible] |
| NKX2-3 | transcription regulator | Inhibited | [illegible] | [illegible] |
| IRF1 | transcription regulator | Activated | [illegible] | [illegible] |
| RELA | transcription regulator | Activated | [illegible] | [illegible] |
| PRL | cytokine | Activated | [illegible] | [illegible] |
| IFNB1 | cytokine | Activated | [illegible] | [illegible] |
| SB203580 | chemical - kinase inhibitor | Inhibited | [illegible] | [illegible] |
| dexamethasone | chemical drug | Inhibited | [illegible] | [illegible] |
| TLR3 | transmembrane receptor | Activated | [illegible] | [illegible] |
| IRF3 | transcription regulator | Activated | [illegible] | [illegible] |
| IFN Beta | group | Activated | [illegible] | [illegible] |
| beta-estradiol | chemical - endogenous mammalian | Activated | [illegible] | [illegible] |
| EIF2AK2 | kinase | Activated | [illegible] | [illegible] |
| Ifnar | group | Activated | [illegible] | [illegible] |
| IL10RA | cytokine | Inhibited | [illegible] | [illegible] |
| Salmonella enterica serotype abortus equi lipopolysaccharide | chemical toxicant | Activated | [illegible] | [illegible] |
| NFKB1 | transcription regulator | Activated | [illegible] | [illegible] |
| [illegible] | [illegible] | [illegible] | [illegible] | [illegible] |

| | | | | |
|---|---|---|---|---|
| carrageenan | chemical drug | Activated | 3.24E-10 | [illegible] |
| LDB1 | transcription regulator | | 3.26E-10 | [illegible] |
| IFNK | cytokine | Activated | 3.30E-10 | [illegible] |
| TMPRSS2-ERG | fusion gene/product | | 3.43E-10 | [illegible] |
| Go 6976 | chemical - kinase inhibitor | Inhibited | 3.46E-10 | [illegible] |
| PLX5622 | chemical drug | Activated | 3.86E-10 | [illegible] |
| docosahexaenoic acid | chemical drug | Inhibited | 3.97E-10 | [illegible] |
| MET | kinase | Activated | 4.00E-10 | [illegible] |
| hyaluronic acid | chemical - endogenous mammalian | Activated | 4.22E-10 | [illegible] |
| ERBB2 | kinase | Activated | 4.44E-10 | [illegible] |
| CEBPA | transcription regulator | Activated | 4.45E-10 | [illegible] |
| mir-8 | microRNA | Inhibited | 4.49E-10 | [illegible] |
| [illegible] | other | Activated | 4.72E-10 | [illegible] |
| zymosan | chemical - endogenous non-mammalian | Activated | 4.90E-10 | [illegible] |
| miR-302a-3p (and other miRNAs w/seed AGUGCUU) | mature microRNA | Inhibited | 5.14E-10 | [illegible] |
| CD28 | transmembrane receptor | Activated | 5.21E-10 | [illegible] |
| laminarin | chemical drug | Activated | 5.33E-10 | [illegible] |
| VEGFA | growth factor | Activated | 5.34E-10 | [illegible] |
| ATP | chemical - endogenous mammalian | Activated | 5.54E-10 | [illegible] |
| WNT5A | cytokine | | 5.65E-10 | [illegible] |
| Histone h3 | group | | 5.79E-10 | [illegible] |
| CEBPB | transcription regulator | Activated | 6.06E-10 | [illegible] |
| FSH | complex | Activated | 6.08E-10 | [illegible] |
| okadaic acid | chemical toxicant | Activated | 6.23E-10 | [illegible] |
| D-glucose | chemical - endogenous mammalian | Activated | 6.79E-10 | [illegible] |
| [illegible] | cytokine | | 7.03E-10 | [illegible] |
| miR-124-3p (and other miRNAs w/seed AAGGCAC) | mature microRNA | Inhibited | 7.17E-10 | [illegible] |
| arsenite | chemical toxicant | Activated | 7.24E-10 | [illegible] |
| cyclic AMP | chemical - endogenous mammalian | Activated | 7.47E-10 | [illegible] |
| ATP-gamma-S | chemical reagent | | 7.59E-10 | [illegible] |
| RAF1 | kinase | Activated | 8.09E-10 | [illegible] |
| PIK3R1 | kinase | Activated | 8.13E-10 | [illegible] |
| platelet activating factor | chemical - endogenous mammalian | Activated | 8.14E-10 | [illegible] |
| CAMP | other | Activated | 8.32E-10 | [illegible] |
| CD36 | transmembrane receptor | Activated | 8.43E-10 | [illegible] |
| NLRC5 | transcription regulator | Activated | 8.47E-10 | [illegible] |
| staurosporine | chemical - kinase inhibitor | | 8.71E-10 | [illegible] |
| SAMHD1 | enzyme | Inhibited | 8.73E-10 | [illegible] |
| POU5F1 | transcription regulator | Activated | 8.73E-10 | [illegible] |
| CREM | transcription regulator | Activated | 8.86E-10 | [illegible] |
| SQSTM1 | transcription regulator | | 8.90E-10 | [illegible] |
| PPARG | ligand-dependent nuclear receptor | | 9.10E-10 | [illegible] |
| Fcer1 | complex | Activated | 9.20E-10 | [illegible] |
| 2-amino-5-phosphonovaleric acid | chemical - other | Inhibited | 9.24E-10 | [illegible] |
| IL18 | cytokine | Activated | 9.34E-10 | [illegible] |
| Tlr | group | Activated | 9.43E-10 | [illegible] |
| EGR2 | transcription regulator | | 1.18E-09 | [illegible] |
| ZBED2 | other | Inhibited | 1.29E-09 | [illegible] |
| miR-182-5p (and other miRNAs w/seed UUGGCAA) | mature microRNA | Inhibited | 1.29E-09 | [illegible] |
| 1-methyl-4-phenyl-1,2,3,6-tetrahydropyridine | chemical toxicant | Activated | 1.40E-09 | [illegible] |
| ATF4 | transcription regulator | Activated | 1.40E-09 | [illegible] |
| ADRA1A | G-protein coupled receptor | | 1.41E-09 | [illegible] |
| miR-503-5p (miRNAs w/seed AGCAGCG) | mature microRNA | Inhibited | 1.43E-09 | [illegible] |
| JAG2 | growth factor | | 1.49E-09 | [illegible] |
| ARHGAP21 | other | | 1.55E-09 | [illegible] |
| DDIT3 | transcription regulator | Activated | 1.58E-09 | [illegible] |
| SP110 | transcription regulator | Inhibited | 1.65E-09 | [illegible] |
| CpG oligonucleotide | chemical drug | Activated | 1.79E-09 | [illegible] |
| genistein | chemical drug | Inhibited | 1.80E-09 | [illegible] |
| methylprednisolone | chemical drug | | 1.82E-09 | [illegible] |
| romidepsin | biologic drug | | 2.04E-09 | [illegible] |
| RPS6KA5 | kinase | | 2.11E-09 | [illegible] |
| mezerein | chemical - endogenous non-mammalian | | 2.11E-09 | [illegible] |
| NOD1 | other | Activated | 2.14E-09 | [illegible] |
| NGF | growth factor | Activated | 2.23E-09 | [illegible] |
| TRAF6 | enzyme | Activated | 2.30E-09 | [illegible] |
| lysophosphatidylinositol | chemical - endogenous mammalian | Activated | 2.44E-09 | [illegible] |
| ADRA1B | G-protein coupled receptor | | 2.44E-09 | [illegible] |
| mitoxantrone | chemical drug | | 2.58E-09 | [illegible] |
| Pka | complex | Activated | 2.60E-09 | [illegible] |
| miR-1283 (miRNAs w/seed CUACAAA) | mature microRNA | Inhibited | 2.71E-09 | [illegible] |
| [illegible] | [illegible] | [illegible] | [illegible] | [illegible] |

| | | | | |
|---|---|---|---|---|
| RBM5 | other | | 1,03E-06 | [illegible] |
| advanced glycation end-products | chemical - endogenous mammalian | | 1,03E-06 | [illegible] |
| sulforafan | chemical drug | | 1,04E-06 | [illegible] |
| LMNA | other | | 1,07E-06 | [illegible] |
| LYN | kinase | | 1,10E-06 | [illegible] |
| CCN1 | other | Activated | 1,10E-06 | [illegible] |
| FOXP3 | transcription regulator | | 1,12E-06 | [illegible] |
| pirinixic acid | chemical toxicant | | 1,12E-06 | [illegible] |
| PRKD1 | kinase | Activated | 1,14E-06 | [illegible] |
| RNASEL | enzyme | | 1,14E-06 | [illegible] |
| ROR1 | kinase | | 1,14E-06 | [illegible] |
| CTNNB1 | transcription regulator | Activated | 1,16E-06 | [illegible] |
| 12-(3-adamantan-1-yl-ureido) dodecanoic acid | chemical reagent | Inhibited | 1,18E-06 | [illegible] |
| CLEC12A | other | Activated | 1,19E-06 | [illegible] |
| EGFL7 | other | | 1,19E-06 | [illegible] |
| ICAM1 | transmembrane receptor | Activated | 1,19E-06 | [illegible] |
| MALP-2s | chemical reagent | Activated | 1,20E-06 | [illegible] |
| hydrochloric acid | chemical - endogenous mammalian | | 1,20E-06 | [illegible] |
| 8-bromo-cAMP | chemical reagent | Activated | 1,22E-06 | [illegible] |
| mir-17 | microRNA | Inhibited | 1,23E-06 | [illegible] |
| miR-432 (and other miRNAs w/seed CUUGGAG) | mature microRNA | Inhibited | 1,24E-06 | [illegible] |
| fulvestrant | chemical drug | | 1,26E-06 | [illegible] |
| miR-128-3p (and other miRNAs w/seed CACAGUG) | mature microRNA | Inhibited | 1,29E-06 | [illegible] |
| CDKN2A | transcription regulator | | 1,30E-06 | [illegible] |
| leukotriene C4 | chemical - endogenous mammalian | Activated | 1,30E-06 | [illegible] |
| fontolizumab | biologic drug | Inhibited | 1,30E-06 | [illegible] |
| PPP2R5C | other | Inhibited | 1,30E-06 | [illegible] |
| diethylstilbestrol | chemical drug | | 1,31E-06 | [illegible] |
| SP2509 | chemical reagent | | 1,31E-06 | [illegible] |
| Y 27632 | chemical drug | Inhibited | 1,35E-06 | [illegible] |
| minocycline | chemical drug | Inhibited | 1,35E-06 | [illegible] |
| miR-4261 (miRNAs w/seed CUGAGAA) | mature microRNA | Inhibited | 1,35E-06 | [illegible] |
| miR-4718 (miRNAs w/seed GCUGUAC) | mature microRNA | Inhibited | 1,39E-06 | [illegible] |
| IKZF3 | transcription regulator | Inhibited | 1,46E-06 | [illegible] |
| IL12A | cytokine | Activated | 1,47E-06 | [illegible] |
| [illegible] | chemical drug | Activated | 1,47E-06 | [illegible] |
| berberine | chemical drug | | 1,48E-06 | [illegible] |
| CD44 | other | Activated | 1,50E-06 | [illegible] |
| KITLG | growth factor | Activated | 1,51E-06 | [illegible] |
| miR-146a-5p (and other miRNAs w/seed GAGAACU) | mature microRNA | Inhibited | 1,54E-06 | [illegible] |
| XDH | enzyme | | 1,55E-06 | [illegible] |
| TANK | other | | 1,55E-06 | [illegible] |
| EGLN1 | enzyme | | 1,56E-06 | [illegible] |
| ISGF3 | complex | Activated | 1,56E-06 | [illegible] |
| YOD1 | enzyme | | 1,56E-06 | [illegible] |
| [illegible] | enzyme | | 1,56E-06 | [illegible] |
| IL1R2 | transmembrane receptor | Inhibited | 1,56E-06 | [illegible] |
| S100A7 | other | | 1,56E-06 | [illegible] |
| J11-Cl | chemical reagent | Inhibited | 1,56E-06 | [illegible] |
| miR-22-3p (miRNAs w/seed AGCUGCC) | mature microRNA | Inhibited | 1,57E-06 | [illegible] |
| 9,10-dimethyl-1,2-benzanthracene | chemical toxicant | Activated | 1,59E-06 | [illegible] |
| IFNA17 | cytokine | Activated | 1,60E-06 | [illegible] |
| F10 | peptidase | Activated | 1,60E-06 | [illegible] |
| nickel chloride | chemical toxicant | | 1,60E-06 | [illegible] |
| mir-181 | microRNA | Inhibited | 1,62E-06 | [illegible] |
| miR-767 (and other miRNAs w/seed GCACCAU) | mature microRNA | Inhibited | 1,62E-06 | [illegible] |
| MAP2K6 | kinase | Activated | 1,74E-06 | [illegible] |
| hydrocortisone | chemical - endogenous mammalian | | 1,74E-06 | [illegible] |
| Fibrinogen | complex | Activated | 1,80E-06 | [illegible] |
| ACKR1 | G-protein coupled receptor | | 1,80E-06 | [illegible] |
| SOCS6 | other | Activated | 1,80E-06 | [illegible] |
| VTN | other | | 1,80E-06 | [illegible] |
| eplerenone | chemical drug | | 1,80E-06 | [illegible] |
| YY1 | transcription regulator | | 1,82E-06 | [illegible] |
| miR-326-5p (and other miRNAs w/seed [illegible]) | mature microRNA | Inhibited | 1,85E-06 | [illegible] |
| TGFB2 | growth factor | Activated | 1,85E-06 | [illegible] |
| FASLG | cytokine | | 1,87E-06 | [illegible] |
| S-nitrosoglutathione | chemical toxicant | | 1,87E-06 | [illegible] |
| ATM | kinase | | 1,90E-06 | [illegible] |
| silibinin | chemical drug | Inhibited | 1,90E-06 | [illegible] |
| miR-2682-5p (and other miRNAs w/seed AGGCAGU) | mature microRNA | Inhibited | 1,94E-06 | [illegible] |
| IFNL3 | cytokine | Activated | 1,98E-06 | [illegible] |
| IRAK3 | kinase | | 1,98E-06 | [illegible] |
| methylnitronitrosoguanidine | chemical toxicant | Activated | 1,98E-06 | [illegible] |
| hemozoin | chemical - endogenous non-mammalian | Activated | 1,98E-06 | [illegible] |
| fludarabine | chemical drug | | 1,98E-06 | [illegible] |
| ibrutinib | chemical drug | Inhibited | 2,01E-06 | [illegible] |
| VDR | transcription regulator | | 2,02E-06 | [illegible] |
| ritonavir | chemical drug | | 2,13E-06 | [illegible] |
| miR-1275 (and other miRNAs w/seed [illegible]) | mature microRNA | Inhibited | 2,16E-06 | [illegible] |
| CXCR4 | G-protein coupled receptor | Activated | 2,16E-06 | [illegible] |
| WBP2 | transcription regulator | | 2,18E-06 | [illegible] |
| SRC (family) | group | Activated | 2,19E-06 | [illegible] |
| niacinamide | chemical - endogenous mammalian | | 2,20E-06 | [illegible] |
| CLEC4D | other | | 2,21E-06 | [illegible] |
| CD38 | enzyme | | 2,22E-06 | [illegible] |
| EPO | cytokine | Activated | 2,26E-06 | [illegible] |
| miR-3680 (and other miRNAs w/seed [illegible]) | mature microRNA | Inhibited | 2,27E-06 | [illegible] |
| STAT | group | Activated | 2,31E-06 | [illegible] |
| CASR | G-protein coupled receptor | | 2,60E-06 | [illegible] |
| miR-26a-5p (and other miRNAs w/seed UCAAGUA) | mature microRNA | Inhibited | 2,64E-06 | [illegible] |
| PI-103 | chemical - kinase inhibitor | | 2,66E-06 | [illegible] |
| LRBA | other | | 2,66E-06 | [illegible] |
| S1PR3 | G-protein coupled receptor | | 2,66E-06 | [illegible] |
| miR-190a-5p (and other miRNAs w/seed GAUAUGU) | mature microRNA | Inhibited | 2,67E-06 | [illegible] |
| LIPE | enzyme | | 2,69E-06 | [illegible] |
| SELPLG | other | Activated | 2,69E-06 | [illegible] |
| DPP4 | peptidase | | 2,69E-06 | [illegible] |
| SELP | transmembrane receptor | Activated | 2,77E-06 | [illegible] |
| KDM1A | enzyme | | 2,79E-06 | [illegible] |
| miR-141-3p (and other miRNAs w/seed AACACUG) | mature microRNA | Inhibited | 2,80E-06 | [illegible] |
| ciglitazone | chemical drug | | 2,84E-06 | [illegible] |
| nobiletin | chemical toxicant | Inhibited | 2,84E-06 | [illegible] |
| LEP | growth factor | Activated | 2,88E-06 | [illegible] |
| L-triiodothyronine | chemical - endogenous mammalian | | 2,90E-06 | [illegible] |
| aldesleukin | biologic drug | Activated | 2,92E-06 | [illegible] |
| MAP2K4 | kinase | Activated | 2,98E-06 | [illegible] |
| BRD4 | kinase | Activated | 2,98E-06 | [illegible] |
| isoproterenol | chemical drug | Activated | 3,03E-06 | [illegible] |
| NS-398 | chemical reagent | Inhibited | 3,09E-06 | [illegible] |
| indican | chemical - endogenous mammalian | | 3,16E-06 | [illegible] |
| mir-132 | microRNA | | 3,16E-06 | [illegible] |
| celecoxib | chemical drug | | 3,22E-06 | [illegible] |
| lactosylceramide | chemical - endogenous mammalian | | 3,26E-06 | [illegible] |
| cis-urocanic acid | chemical drug | Activated | 3,26E-06 | [illegible] |
| PTAFR | G-protein coupled receptor | | 3,26E-06 | [illegible] |
| ACVR1C | kinase | Activated | 3,26E-06 | [illegible] |
| NfkB1-RelA | complex | Activated | 3,38E-06 | [illegible] |
| PLK2 | kinase | Activated | 3,38E-06 | [illegible] |
| ACE2 | peptidase | | 3,38E-06 | [illegible] |
| BAG1 | other | | 3,38E-06 | [illegible] |
| KN-62 | chemical - kinase inhibitor | Inhibited | 3,38E-06 | [illegible] |
| geranylgeranyl pyrophosphate | chemical - endogenous mammalian | | 3,38E-06 | [illegible] |
| F3 | transmembrane receptor | Activated | 3,48E-06 | [illegible] |
| miR-142-3p (and other miRNAs w/seed GUAGUGU) | mature microRNA | Inhibited | 3,52E-06 | [illegible] |
| AZD8055 | chemical drug | | 3,62E-06 | [illegible] |
| miR-217-5p (and other miRNAs w/seed ACUGCAU) | mature microRNA | Inhibited | 3,65E-06 | [illegible] |
| C6 | complex | | 3,65E-06 | [illegible] |
| NEK7 | kinase | | 3,65E-06 | [illegible] |
| PSMB9 | peptidase | | 3,65E-06 | [illegible] |
| ATG16L1 | enzyme | Inhibited | 3,65E-06 | [illegible] |
| ING4 | transcription regulator | | 3,65E-06 | [illegible] |
| C7 | other | | 3,65E-06 | [illegible] |
| methotrexate | chemical drug | Inhibited | 3,73E-06 | [illegible] |
| 3-deazaneplanocin | chemical drug | Activated | 3,76E-06 | [illegible] |
| NR1H2 | ligand-dependent nuclear receptor | | 3,76E-06 | [illegible] |
| miR-669c-3p (and other miRNAs w/seed ACACACA) | mature microRNA | Inhibited | 3,79E-06 | [illegible] |
| TGFB3 | growth factor | Activated | 3,90E-06 | [illegible] |
| emodin | chemical drug | | 3,97E-06 | [illegible] |
| heme | chemical - endogenous mammalian | Inhibited | 3,97E-06 | [illegible] |
| ITGA5 | transmembrane receptor | | 4,04E-06 | [illegible] |
| IL24 | cytokine | Activated | 4,04E-06 | [illegible] |
| miR-205-5p (and other miRNAs w/seed CCUUCAU) | mature microRNA | Inhibited | 4,11E-06 | [illegible] |
| bafilomycin A1 | chemical drug | | 4,17E-06 | [illegible] |
| SN-38 | chemical drug | Activated | 4,17E-06 | [illegible] |
| [illegible] | chemical reagent | Inhibited | 4,20E-06 | [illegible] |
| CD82 | other | | 4,20E-06 | [illegible] |
| ARF6 | transporter | | 4,20E-06 | [illegible] |
| C9 | other | | 4,20E-06 | [illegible] |
| SPP1 | cytokine | Activated | 4,43E-06 | [illegible] |
| SOX2 | transcription regulator | | 4,49E-06 | [illegible] |
| CXCL8 | cytokine | Activated | 4,54E-06 | [illegible] |
| miR-3167-5p (miRNAs w/seed [illegible]) | mature microRNA | Inhibited | 4,54E-06 | [illegible] |
| sirolimus | chemical drug | Inhibited | 4,63E-06 | [illegible] |
| [illegible] | other | | 4,76E-06 | [illegible] |
| morphine | chemical drug | | 4,84E-06 | [illegible] |
| miR-2117 (miRNAs w/seed GUUCUCU) | mature microRNA | Inhibited | 4,87E-06 | [illegible] |
| ZBTB16 | transcription regulator | | 4,88E-06 | [illegible] |
| miR-608-3p (miRNAs w/seed [illegible]) | mature microRNA | Inhibited | 4,92E-06 | [illegible] |
| APEX1 | enzyme | Activated | 4,93E-06 | [illegible] |
| KRT17 | other | | 4,93E-06 | [illegible] |
| proteasome inhibitor PSI | chemical - protease inhibitor | | 4,93E-06 | [illegible] |
| miR-6800-3p (miRNAs w/seed ACCUCUC) | mature microRNA | Inhibited | 4,98E-06 | [illegible] |
| AGN194204 | chemical drug | | 5,02E-06 | [illegible] |
| CBX7 | other | Inhibited | 5,10E-06 | [illegible] |
| BIRC2 | enzyme | | 5,10E-06 | [illegible] |
| PDCD4 | other | Inhibited | 5,10E-06 | [illegible] |
| TREM2 | transmembrane receptor | | 5,10E-06 | [illegible] |
| helenalin | chemical - endogenous non-mammalian | Inhibited | 5,10E-06 | [illegible] |
| [illegible] | chemical - endogenous non-mammalian | | 5,10E-06 | [illegible] |
| miR-6802-3p (and other miRNAs w/seed ACAUGGA) | mature microRNA | Inhibited | 5,10E-06 | [illegible] |
| tetrachlorodibenzodioxin | chemical toxicant | Activated | 5,14E-06 | [illegible] |
| nifedipine | chemical drug | Inhibited | 5,14E-06 | [illegible] |
| PPP2CA | phosphatase | | 5,20E-06 | [illegible] |
| miR-539-3p (miRNAs w/seed [illegible]) | mature microRNA | Inhibited | 5,26E-06 | [illegible] |
| GATA3 | transcription regulator | | 5,32E-06 | [illegible] |
| A2M | transporter | | 5,37E-06 | [illegible] |
| N-[N-(3,5-difluorophenacetyl-L-Ala)]-S-phenylglycine t-butyl ester | chemical - protease inhibitor | Inhibited | 5,37E-06 | [illegible] |
| N-nitro-L-arginine methyl ester | chemical drug | | 5,47E-06 | [illegible] |
| CASP8 | peptidase | | 5,48E-06 | [illegible] |
| DCN | other | | 5,48E-06 | [illegible] |
| miR-6881-5p (miRNAs w/seed [illegible]) | mature microRNA | Inhibited | 5,50E-06 | [illegible] |
| PROCR | other | | 5,51E-06 | [illegible] |
| TWIST1 | transcription regulator | | 5,51E-06 | [illegible] |
| CD86 | transmembrane receptor | | 5,54E-06 | [illegible] |
| miR-1-3p (and other miRNAs w/seed GGAAUGU) | mature microRNA | Inhibited | 5,54E-06 | [illegible] |
| Pkg | group | | 5,66E-06 | [illegible] |
| IL17a dimer | complex | Activated | 5,66E-06 | [illegible] |
| FGF4 | growth factor | | 5,66E-06 | [illegible] |
| CX-5461 | chemical drug | Activated | 5,66E-06 | [illegible] |
| [illegible] | chemical - endogenous non-mammalian | Inhibited | 5,66E-06 | [illegible] |
| [illegible] | chemical drug | | 5,66E-06 | [illegible] |
| RET | kinase | | 5,67E-06 | [illegible] |
| ZBTB17 | transcription regulator | | 5,70E-06 | [illegible] |
| GLI3 | transcription regulator | Activated | 5,76E-06 | [illegible] |
| lipoteichoic acid | chemical - endogenous non-mammalian | Activated | 5,76E-06 | [illegible] |
| miR-3120 (and other miRNAs w/seed ACAGCAA) | mature microRNA | Inhibited | 5,91E-06 | [illegible] |
| KDM5B | transcription regulator | | 6,21E-06 | [illegible] |
| infliximab | biologic drug | Inhibited | 6,36E-06 | [illegible] |
| ATF2 | transcription regulator | Activated | 6,46E-06 | [illegible] |
| PRNP | other | | 6,51E-06 | [illegible] |
| cyclophosphamide | chemical drug | Activated | 6,51E-06 | [illegible] |
| BMP6 | growth factor | Activated | 6,67E-06 | [illegible] |
| CBX8 | transcription regulator | | 6,74E-06 | [illegible] |
| Ap1 | complex | Activated | 6,84E-06 | [illegible] |
| TYROBP | transmembrane receptor | Activated | 6,89E-06 | [illegible] |
| dopamine | chemical - endogenous mammalian | Activated | 6,98E-06 | [illegible] |
| NORAD | other | Activated | 7,02E-06 | [illegible] |
| Tcf | transcription regulator | | 7,08E-06 | [illegible] |
| zinc | chemical drug | | 7,12E-06 | [illegible] |
| miR-6516-3p (miRNAs w/seed UCAUGUA) | mature microRNA | Inhibited | 7,14E-06 | [illegible] |
| EBI3 | cytokine | Activated | 7,30E-06 | [illegible] |
| MEF2D | transcription regulator | Activated | 7,30E-06 | [illegible] |
| HOTAIR | other | | 7,40E-06 | [illegible] |
| Vitamin D3-VDR-RXR | complex | | 7,48E-06 | [illegible] |
| RIPK1 | kinase | Activated | 7,48E-06 | [illegible] |
| epothilone B | chemical drug | | 7,52E-06 | [illegible] |
| LY96 | transmembrane receptor | | 7,52E-06 | [illegible] |
| FCGR1A | transmembrane receptor | | 7,52E-06 | [illegible] |
| etanercept | biologic drug | Inhibited | 7,52E-06 | [illegible] |
| Fc gamma receptor | group | | 7,52E-06 | [illegible] |
| CORT | other | Inhibited | 7,52E-06 | [illegible] |
| miR-3691-5p (miRNAs w/seed [illegible]) | mature microRNA | Inhibited | 7,60E-06 | [illegible] |
| miR-875-5p (and other miRNAs w/seed AUACCUC) | mature microRNA | Inhibited | 7,60E-06 | [illegible] |
| patulin | chemical toxicant | | 7,60E-06 | [illegible] |
| FIRRE | other | Activated | 7,60E-06 | [illegible] |
| TRIM8 | other | | 7,60E-06 | [illegible] |
| PROK1 | growth factor | | 7,60E-06 | [illegible] |
| ADCYAP1R1 | G-protein coupled receptor | Activated | 7,60E-06 | [illegible] |
| 15-E2-isoketal modified phosphatidylethanolamine | chemical reagent | Activated | 7,60E-06 | [illegible] |
| dacarbazine | chemical drug | Activated | 7,60E-06 | [illegible] |
| [illegible] | chemical drug | Inhibited | 7,60E-06 | [illegible] |
| vinpocetine | chemical drug | Inhibited | 7,60E-06 | [illegible] |
| [illegible] (miRNAs w/seed [illegible]) | mature microRNA | Inhibited | 7,68E-06 | [illegible] |
| TAF4 | transcription regulator | | 7,82E-06 | [illegible] |
| GW9662 | chemical reagent | | 7,92E-06 | [illegible] |
| ETS1 | transcription regulator | Activated | 8,00E-06 | [illegible] |
| miR-122b-3p (and other miRNAs w/seed AACACCA) | mature microRNA | Inhibited | 8,02E-06 | [illegible] |
| GNA15 | enzyme | | 8,17E-06 | [illegible] |
| glutathione | chemical - endogenous mammalian | | 8,19E-06 | [illegible] |
| Lh | complex | Activated | 8,43E-06 | [illegible] |
| NR3C2 | ligand-dependent nuclear receptor | Activated | 8,59E-06 | [illegible] |
| JAK inhibitor I | chemical - kinase inhibitor | Inhibited | 8,73E-06 | [illegible] |
| carbon monoxide | chemical - endogenous mammalian | | 8,98E-06 | [illegible] |
| 3M-001 | chemical drug | Activated | 9,01E-06 | [illegible] |
| CAMK4 | kinase | Activated | 9,01E-06 | [illegible] |
| ARRB2 | other | Inhibited | 9,01E-06 | [illegible] |
| IFNW1 | cytokine | Activated | 9,26E-06 | [illegible] |
| S1PR1 | G-protein coupled receptor | | 9,26E-06 | [illegible] |
| [illegible] | chemical reagent | Activated | 9,26E-06 | [illegible] |
| (1S,2R)-NCL-1 | chemical reagent | Activated | 9,26E-06 | [illegible] |
| miR-218-1-3p (miRNAs w/seed [illegible]) | mature microRNA | Inhibited | 9,36E-06 | [illegible] |
| dexamethasone phosphate | chemical drug | | 9,48E-06 | [illegible] |
| Ikk | group | | 9,48E-06 | [illegible] |
| IL36B | cytokine | Activated | 9,48E-06 | [illegible] |
| USP18 | peptidase | | 9,48E-06 | [illegible] |
| MAP2K7 | kinase | | 9,64E-06 | [illegible] |
| luteolin | chemical drug | | 9,64E-06 | [illegible] |
| FGFR1 | kinase | Activated | 9,67E-06 | [illegible] |
| ATF1 | transcription regulator | | 9,89E-06 | [illegible] |
| CHRM1 | G-protein coupled receptor | Activated | 1,00E-05 | [illegible] |
| ELK4 | transcription regulator | | 1,00E-05 | [illegible] |
| CRY2 | enzyme | | 1,00E-05 | [illegible] |
| RPS6KA4 | kinase | | 1,00E-05 | [illegible] |
| PTGER1 | G-protein coupled receptor | | 1,00E-05 | [illegible] |
| HSPA1A/HSPA1B | enzyme | | 1,00E-05 | [illegible] |
| SIRPA | phosphatase | Inhibited | 1,00E-05 | [illegible] |
| verteporfin | chemical drug | Inhibited | 1,00E-05 | [illegible] |
| brefeldin A | chemical - endogenous non-mammalian | | 1,01E-05 | [illegible] |
| nitroprusside | chemical drug | | 1,01E-05 | [illegible] |
| enterotoxin B | biologic drug | Activated | 1,03E-05 | [illegible] |
| PTPN11 | phosphatase | | 1,03E-05 | [illegible] |
| (+)-MK-801 | chemical drug | | 1,03E-05 | [illegible] |
| caffeine | chemical drug | | 1,03E-05 | [illegible] |
| lenalidomide | chemical drug | Activated | 1,06E-05 | [illegible] |
| miR-144-3p (miRNAs w/seed ACAGUAU) | mature microRNA | Inhibited | 1,09E-05 | [illegible] |
| [illegible] (miRNAs w/seed [illegible]) | mature microRNA | Inhibited | 1,09E-05 | [illegible] |
| [illegible] (miRNAs w/seed AGAUCAU) | mature microRNA | Inhibited | 1,11E-05 | [illegible] |
| 6-mercaptopurine | chemical drug | | 1,11E-05 | [illegible] |
| belinostat | chemical drug | | 1,11E-05 | [illegible] |
| RNASEH2B | enzyme | | 1,11E-05 | [illegible] |
| FAT1 | other | Activated | 1,11E-05 | [illegible] |
| PARP14 | enzyme | | 1,11E-05 | [illegible] |
| B4GALT6 | enzyme | Activated | 1,11E-05 | [illegible] |
| N4BP1 | other | | 1,11E-05 | [illegible] |
| TRAF1 | other | | 1,11E-05 | [illegible] |
| TPCA-1 | chemical - kinase inhibitor | Inhibited | 1,11E-05 | [illegible] |
| let-7a-2-3p (and other miRNAs w/seed UGUACAG) | mature microRNA | Inhibited | 1,12E-05 | [illegible] |
| HLX | transcription regulator | Inhibited | 1,12E-05 | [illegible] |
| JQ1 | chemical reagent | | 1,14E-05 | [illegible] |
| [illegible] | chemical reagent | Activated | 1,15E-05 | [illegible] |
| L-alpha-lysophosphatidylcholine, palmitoyl | chemical - endogenous mammalian | | 1,16E-05 | [illegible] |
| styrene | chemical toxicant | Activated | 1,16E-05 | [illegible] |
| BPI | transporter | | 1,16E-05 | [illegible] |
| SENP6 | peptidase | Inhibited | 1,16E-05 | [illegible] |
| ACOD1 | enzyme | | 1,16E-05 | [illegible] |
| PELI2 | enzyme | Activated | 1,16E-05 | [illegible] |
| grepafloxacin | chemical drug | Inhibited | 1,16E-05 | [illegible] |
| [illegible] (miRNAs w/seed [illegible]) | mature microRNA | Inhibited | 1,16E-05 | [illegible] |
| ERBB3 | kinase | | 1,17E-05 | [illegible] |
| HDAC3 | transcription regulator | | 1,18E-05 | [illegible] |
| molybdenum disulfide | chemical reagent | | 1,18E-05 | [illegible] |
| INSIG1 | other | | 1,19E-05 | [illegible] |
| TAZ | enzyme | | 1,19E-05 | [illegible] |
| miR-548aj-5p (and other miRNAs w/seed [illegible]) | mature microRNA | Inhibited | 1,24E-05 | [illegible] |
| miR-21-5p (and other miRNAs w/seed AGCUUAU) | mature microRNA | Inhibited | 1,26E-05 | [illegible] |
| HDAC1 | transcription regulator | | 1,26E-05 | [illegible] |
| tempol | chemical drug | | 1,26E-05 | [illegible] |
| cilostazol | chemical drug | | 1,26E-05 | [illegible] |
| [illegible] (miRNAs w/seed [illegible]) | mature microRNA | Inhibited | 1,29E-05 | [illegible] |
| RUNX3 | transcription regulator | | 1,30E-05 | [illegible] |
| gemcitabine | chemical drug | Activated | 1,30E-05 | [illegible] |
| [illegible] | transporter | Inhibited | 1,30E-05 | [illegible] |
| amphetamine | chemical drug | Activated | 1,30E-05 | [illegible] |
| histamine | chemical - endogenous mammalian | | 1,30E-05 | [illegible] |
| ITGB2 | transmembrane receptor | | 1,39E-05 | [illegible] |
| IL1RAP | transmembrane receptor | Activated | 1,40E-05 | [illegible] |
| [illegible] | other | | 1,40E-05 | [illegible] |
| silicon dioxide | chemical drug | Activated | 1,40E-05 | [illegible] |
| [illegible] | other | | 1,46E-05 | [illegible] |
| [illegible] (miRNAs w/seed [illegible]) | mature microRNA | Inhibited | 1,47E-05 | [illegible] |
| BTNL2 | transmembrane receptor | | 1,48E-05 | [illegible] |
| NTRK2 | kinase | | 1,48E-05 | [illegible] |
| RARB | ligand-dependent nuclear receptor | | 1,48E-05 | [illegible] |
| rotenone | chemical toxicant | | 1,49E-05 | [illegible] |
| TRPV4 | ion channel | Activated | 1,53E-05 | [illegible] |
| ceruletide | biologic drug | Activated | 1,53E-05 | [illegible] |
| kaempferol | chemical toxicant | Inhibited | 1,53E-05 | [illegible] |
| [illegible] | enzyme | Activated | 1,60E-05 | [illegible] |
| PPP1R15A | other | | 1,60E-05 | [illegible] |
| edaravone | chemical drug | | 1,60E-05 | [illegible] |
| carbon tetrachloride | chemical toxicant | Activated | 1,61E-05 | [illegible] |
| 17-alpha-ethinylestradiol | chemical drug | | 1,63E-05 | [illegible] |
| PTK2 | kinase | Activated | 1,65E-05 | [illegible] |
| ARRB1 | other | | 1,65E-05 | [illegible] |
| NFYA | transcription regulator | | 1,66E-05 | [illegible] |
| [illegible] | chemical drug | | 1,68E-05 | [illegible] |
| miR-6733-3p (and other miRNAs w/seed [illegible]) | mature microRNA | Inhibited | 1,68E-05 | [illegible] |
| CTLA4 | transmembrane receptor | | 1,69E-05 | [illegible] |
| STAT5B | transcription regulator | | 1,74E-05 | [illegible] |
| miR-6730-3p (miRNAs w/seed [illegible]) | mature microRNA | Inhibited | 1,77E-05 | [illegible] |
| LPL | enzyme | | 1,77E-05 | [illegible] |
| [illegible] | translation regulator | | 1,77E-05 | [illegible] |
| miR-96-5p (and other miRNAs w/seed UUGGCAC) | mature microRNA | Inhibited | 1,81E-05 | [illegible] |
| [illegible] | enzyme | | 1,82E-05 | [illegible] |
| FCGR2B | transmembrane receptor | | 1,82E-05 | [illegible] |
| CGS 21680 | chemical reagent | | 1,82E-05 | [illegible] |
| pristane | chemical toxicant | Activated | 1,82E-05 | [illegible] |
| MRTFB | transcription regulator | Activated | 1,84E-05 | [illegible] |
| ARID1A | transcription regulator | | 1,86E-05 | [illegible] |
| miR-4727-3p (miRNAs w/seed [illegible]) | mature microRNA | Inhibited | 1,87E-05 | [illegible] |
| miR-3686 (and other miRNAs w/seed [illegible]) | mature microRNA | Inhibited | 1,88E-05 | [illegible] |
| miR-4672 (miRNAs w/seed UACACAG) | mature microRNA | Inhibited | 1,88E-05 | [illegible] |
| ANXA1 | enzyme | | 1,88E-05 | [illegible] |
| baicalein | chemical drug | | 1,88E-05 | [illegible] |
| [illegible] (and other miRNAs w/seed [illegible]) | mature microRNA | Inhibited | 1,91E-05 | [illegible] |
| ADIPOQ | other | | 1,93E-05 | [illegible] |
| BMP2 | growth factor | Activated | 2,01E-05 | [illegible] |
| NPC1 | transporter | | 2,03E-05 | [illegible] |
| TAC1 | other | Activated | 2,03E-05 | [illegible] |
| RXRA | ligand-dependent nuclear receptor | | 2,04E-05 | [illegible] |
| leucine | chemical - endogenous mammalian | | 2,04E-05 | [illegible] |
| vismodegib | chemical drug | | 2,04E-05 | [illegible] |
| suramin | chemical drug | | 2,04E-05 | [illegible] |
| N(G)-monomethyl-D-arginine | chemical - endogenous mammalian | | 2,04E-05 | [illegible] |
| miR-365-3p (and other miRNAs w/seed AAUGCCC) | mature microRNA | Inhibited | 2,08E-05 | [illegible] |
| entinostat | chemical drug | Activated | 2,13E-05 | [illegible] |
| IL1R1 | transmembrane receptor | Activated | 2,13E-05 | [illegible] |
| capsaicin | chemical drug | Activated | 2,13E-05 | [illegible] |
| CDKN2B-AS1 | other | Activated | 2,13E-05 | [illegible] |
| PLCE1 | enzyme | Activated | 2,13E-05 | [illegible] |
| ebselen | chemical drug | Inhibited | 2,13E-05 | [illegible] |
| N-(3-oxododecanoyl)-homoserine lactone | chemical reagent | | 2,13E-05 | [illegible] |
| [illegible] | chemical drug | Inhibited | 2,13E-05 | [illegible] |
| ERN1 | kinase | | 2,16E-05 | [illegible] |
| GLI1 | transcription regulator | Activated | 2,23E-05 | [illegible] |
| [illegible] (and other miRNAs w/seed [illegible]) | mature microRNA | Inhibited | 2,24E-05 | [illegible] |
| FOSL1 | transcription regulator | | 2,29E-05 | [illegible] |
| carbonyl cyanide m-chlorophenyl hydrazone | chemical toxicant | Activated | 2,32E-05 | [illegible] |
| miR-4714-3p (miRNAs w/seed [illegible]) | mature microRNA | Inhibited | 2,33E-05 | [illegible] |
| resolvin D1 | chemical - endogenous mammalian | Inhibited | 2,38E-05 | [illegible] |
| [illegible] | enzyme | Inhibited | 2,38E-05 | [illegible] |
| andrographolide | chemical drug | | 2,38E-05 | [illegible] |
| pioglitazone | chemical drug | | 2,41E-05 | [illegible] |
| CpG ODN 1826 | chemical reagent | Activated | 2,41E-05 | [illegible] |

| | | | | |
|---|---|---|---|---|
| MST1 | growth factor | | 2,41E-05 | FAS,FOS,GPC3,IL10,IL1RN,IL6,IRF8,PTGS2,SOX4,SPP1,TNF |
| RCAN1 | transcription regulator | Inhibited | 2,41E-05 | BDNF,CXCL8,FOS,ICAM1,IL6,NOS3,PTGS2,RCAN1,SELE,TNF,VCAM1 |
| XIAP | enzyme | | 2,41E-05 | CDKN1A,CDKN1B,CSF1,CSF3,CXCL1,CXCL8,IL6,PTGS2,SELE,SOD2,TNF |
| HMGB2 | transcription regulator | | 2,41E-05 | CCL20,CEBPB,CSF3,CXCL1,CXCL2,CXCL8,IFNB1,IL1B,IL6,INHBA,VEGFC |
| CD38 | transmembrane receptor | Activated | 2,42E-05 | BCL10,CBLB,CCL20,CD69,CSF2,FAS,FOS,IL6,JUN,MAP3K8,NFKB1,NFKBIA,NFKBIB,PIK3R1,PTGS2,SOCS1,SPHK1,TNF,TNFRSF1B |
| LCN2 | transporter | Activated | 2,47E-05 | BMP2,CCL20,CCL22,CCL5,CCR1,COL1A1,CXCL10,CXCL2,CXCL3,FAS,HMOX1,IL10,IL1A,IL1B,IL1RN,IL6,PPARGC1A,RARA,SCD,SOD2,SPP1,TNF,VIM |
| cinnamaldehyde | chemical toxicant | | 2,47E-05 | CAT,CLK2,CXCL8,EGR1,FOS,HMOX1,HSPA1A/HSPA1B,IFNB1,IL1B,INSIG1,JUND,NFE2L2,NFKB1,ODC1,PTGS2,SELE,SOD2,SQSTM1,TNF,TRAF6,TRIM16,TXNIP,XIAP |
| Hsp70 | group | | 2,48E-05 | CD40,CD83,CXCL3,CXCL8,ICAM1,IL10,IL1B,IL6,MLKL,NFKBIA,NOD2,NPC1,SELE,SNCA,TNF,VCAM1 |
| miR-638-3p (miRNAs w/seed CUAGUAA) | mature microRNA | Inhibited | 2,48E-05 | AKAP10,ARRDC3,C18orf25,C21orf91,CD69,EGFL6,EREG,EYA1,FAM133B,FAS,GORAB,HDX,HIRA,HLA-F,HSPBAP1,IDO1,IL7,KLK7,KMO,LCOR,LRIG2,MAPK8,MAST4,MASTL,MBNL3,MTPN,RGL3,NBPF10 (includes others),NEDD4,NHSL2,NPC1,NR4A3,PAPSS1,PARD [illegible] |
| POMC | other | Activated | 2,49E-05 | CDKN1B,CYP1B1,DUSP1,FGF2,FOS,FOSL1,FOSL2,HMOX1,ICAM1,IGF2,IL6,JUN,JUNB,NOS3,NR4A1,NR4A2,NR4A3,PPARGC1A,SELE,SLC9A3R2,SOD2,TFE3,TNF,USP10,VCAM1 |
| CEBPD | transcription regulator | Activated | 2,49E-05 | C3,CCL20,CCND3,CDKN1A,CDKN1B,CEBPB,CEBPD,CSF1R,CTSV,CXCL1,CXCL3,CXCL8,CYP1A1,DDIT3,FBXW7,FOS,IKBKE,IL1B,IL6,ITGAM,PDGFA,PTGS2,TNF,VEGFC,XBP1 |
| DACH1 | transcription regulator | Inhibited | 2,54E-05 | CDKN1A,CXCL3,CXCL8,EGR1,FGF2,FGFR1,FOS,ID2,IL6,JUN,KLF4,PTGS2,STC2,TNFAIP3 |
| FCER1G | transmembrane receptor | Activated | 2,54E-05 | CCL5,CD69,CSF2,CXCL8,GATA3,IDO1,IFIT2,IL1B,IL1RN,IL6,MX1,PTGS2,RSAD2,TNF |
| Collagen(s) | complex | | 2,58E-05 | ATF4,BCL3,C3,C4A/C4B,CCN1,CD83,CD68,DDIT3,HERPUD1,ITGAM,NCOA6,PLA2G6,PPP1R15A,SNAI1,THBS1 |
| miR-27b-5p (miRNAs w/seed GAGCUUA) | mature microRNA | Inhibited | 2,57E-05 | ABCA5,AKAP17A,AKAP7,ARHGEF26,ARRDC3,ATP6IP1,BCL2L14,BLZF1,BMT2,BRD4,BRPF3,BTC,C6,CASP8,CCDC88A,CDR2,CHAC1,CNOT4,CNTNAP1,CRIM1,CRY2,CX3CL1,CXCL1,CXCL10,DHRS2,DUSP16,EIF4E3,EPHX1,FBXL12,FBXO11,FNIP2,FOXP2,GLCCI [illegible] |
| glycine | chemical - endogenous mammalian | | 2,64E-05 | ATF4,BDNF,CDKN1A,FOS,IL1B,ITGAM,ODC1,TNF,VEGFA |
| PLC | group | Activated | 2,64E-05 | CCL2,CXCL10,CXCL8,EGR3,FOS,NR4A1,ODC1,PTGS2,TNF |
| PKNOX2 | transcription regulator | Activated | 2,64E-05 | BBC3,BTG2,CDKN1A,EGR1,FAS,GADD45A,MDM4,STAT1,VIM |
| PADI2 | enzyme | | 2,64E-05 | ADAMTS9,CXCL2,CXCL3,CXCL8,IL6,PTGS2,SNAI1,TNFRSF1B,VIM |
| PAEP | other | | 2,64E-05 | CCL5,CD274,CD40,CXCL1,EDN1,GATA3,HBEGF,MICB,VEGFA |
| NCR2 | transmembrane receptor | Activated | 2,64E-05 | CCL20,CSF2,NR4A1,NR4A2,NR4A3,PTGS2,RCAN1,THBS1,TNF |
| melphalan | chemical drug | | 2,64E-05 | BBC3,BCL2L11,CDKN1A,IFNB1,MCL1,MICB,TNF,TNFRSF10B,XAF1 |
| salinosporamide A | chemical drug | Inhibited | 2,67E-05 | BIRC2,BIRC3,CFLAR,ICAM1,PTGS2,SNAI1,TNFRSF10B,TRAF1,VEGFA,XIAP |
| ORMDL3 | other | Activated | 2,67E-05 | ADAM8,CCL20,CXCL10,CXCL11,CXCL8,IFNB1,OAS1,OAS2,OAS3,TNFSF13B |
| MAPK12 | kinase | | 2,67E-05 | FOS,IFNB1,IL12A,IL1B,ITGB8,JUN,MAP3K8,PPARGC1A,TNF,VDR |
| GADD45A | other | | 2,67E-05 | BIRC2,CDKN1A,CXCL2,CXCL3,FOSB,IL1B,IL6,JUN,MMP7,NEK2 |
| ketamine | chemical drug | | 2,67E-05 | ARC,BDNF,CXCL8,FOS,HMOX1,KLF6,NOS3,NR4A1,PTGS2,TNF |
| TNFRSF8 | transmembrane receptor | Activated | 2,73E-05 | AHNAK,CASP1,CCL22,CDKN1A,CXCL8,FAS,GBP1,GBP2,IFI16,JUNB,MAP4K3,NFKB2,ODC1,RELB,TNF,TNFSF10,TRAF1,TRAF2 |
| fingolimod phosphate | chemical - endogenous mammalian | | 2,77E-05 | CCL2,ERN1,HAS2,IL6,SPHK1,THBS1 |
| PADI4 | enzyme | | 2,77E-05 | BBC3,CDKN1A,GADD45A,IL6,OSGIN1,TNF |
| ARID3B | transcription regulator | Activated | 2,77E-05 | BCL2L11,CASP10,CASP7,TNF,TNFRSF1B,TNFSF10 |
| cyanidin 3-O-glucoside | chemical - endogenous non-mammalian | | 2,77E-05 | ABCG1,CCL5,IL1B,IL6,PTGS2,TNF |
| IL36 | cytokine | Activated | 2,77E-05 | CCL20,CXCL8,ICAM1,IL1B,IL6,TNF |
| FEM1A | transcription regulator | Inhibited | 2,77E-05 | CD68,CXCL10,CXCL2,IL1B,IL6,TNF |
| LPA | other | | 2,77E-05 | CXCL8,ICAM1,NR3C1,SELE,SERPINB2,VCAM1 |
| ATF7 | transcription regulator | Activated | 2,77E-05 | DDIT3,DUSP1,DUSP10,DUSP5,DUSP6,SELE |
| BLM | enzyme | | 2,77E-05 | CDKN1A,IFI44L,IFIT1,ISG20,MX1,OAS1 |
| raclopride | chemical drug | | 2,77E-05 | BDNF,FOS,NR4A1,NR4A2,NR4A3,SDHB |
| AGI-1067 | chemical drug | Inhibited | 2,77E-05 | CCL2,EGR1,IL1B,IL6,TNF,VCAM1 |
| levofloxacin | chemical drug | | 2,77E-05 | CDKN1A,CSF2,CXCL8,HAS2,IL1A,IL6 |
| huperzine A | chemical drug | | 2,77E-05 | ACHE,CCL2,IL1B,IL6,NGFR,TNF |
| alpha-tocopherol succinate | chemical drug | Inhibited | 2,77E-05 | EGR1,FGF2,IL1A,TNF,VCAM1,VEGFA |
| EPHB1 | kinase | Activated | 2,77E-05 | EGR1,EGR2,FOS,IL6ST,JUN,JUNB,PTGS2 |
| teriflunomide | chemical drug | | 2,77E-05 | CASP8,FAS,ICAM1,IL10RA,IL1B,IL6,TNF |
| HOXA-AS2 | other | | 2,77E-05 | BRAF,CDKN1A,DDIT3,EGFR,KLF2,PLK3,TNFSF10 |
| APOL1 | transporter | | 2,77E-05 | CASP1,CXCL2,IFNGR2,IL1B,MUC13,NLRP3,USO |
| WWP2 | enzyme | | 2,77E-05 | CCL5,EGR2,IFNB1,IL6,SMAD7,TNF,VIM |
| nandrolone | chemical drug | | 2,77E-05 | CAT,HMOX1,IL6,NGFR,NOS3,SOD2,VEGFA |
| miR-376a-3p (miRNAs w/seed AUCAUAGA) | mature microRNA | Inhibited | 2,86E-05 | [illegible] |
| BCL2 | transporter | | 2,91E-05 | [illegible] |
| miR-376a-5p (and other miRNAs w/seed UAUACA) | mature microRNA | Inhibited | 3,00E-05 | [illegible] |
| 4-phenylbutyric acid | chemical - endogenous mammalian | | 3,00E-05 | [illegible] |
| CARD9 | other | Activated | 3,02E-05 | C3,CCL5,CCR1,CSF3,CXCL10,CXCL2,CXCL3,IL1A,IL1B,IL6,NPTX1,TNF |
| IDR-1002 | chemical reagent | | 3,02E-05 | CCL2,CCL20,CCL5,CXCL1,CXCL2,CXCL3,CXCL8,IL6,ITGAM,NLRP3,PTGER2,TNF |
| RGS10 | enzyme | | 3,02E-05 | [illegible] |
| SHC1 | other | | 3,06E-05 | [illegible] |
| RBPJ | transcription regulator | | 3,09E-05 | [illegible] |
| miR-6065 (miRNAs w/seed GUAGGAA) | mature microRNA | Inhibited | 3,10E-05 | [illegible] |
| ITGB1 | transmembrane receptor | | 3,11E-05 | [illegible] |
| miR-4719 (miRNAs w/seed CACAAAU) | mature microRNA | Inhibited | 3,13E-05 | [illegible] |
| MYOD1 | transcription regulator | | 3,14E-05 | [illegible] |
| GSK3B | kinase | | 3,19E-05 | [illegible] |
| deoxycholate | chemical - endogenous mammalian | | 3,27E-05 | [illegible] |
| PLK1 | kinase | | 3,27E-05 | [illegible] |
| HDAC3 (includes others) | other | Activated | 3,27E-05 | [illegible] |
| MAP2K5 | kinase | | 3,27E-05 | [illegible] |
| TIMP3 | other | Inhibited | 3,27E-05 | CEBPB,CREBBP,EGR2,FAS,FOS,IL6,JUN,KLF4,NFKB1,SKIL,SMAD7,STAT1,THBS1,TNF,VIM,ZEB2 |
| miR-4460 (miRNAs w/seed UAGUGGU) | mature microRNA | Inhibited | 3,28E-05 | [illegible] |
| AGER | transmembrane receptor | | 3,31E-05 | [illegible] |
| 8-bromoguanosine 3',5'-cyclic monophosphate | chemical - kinase inhibitor | | 3,37E-05 | BMP2,CDKN1A,CYP3A5,EDN1,FOS,HMOX1,ITGAM,JUNB,NOS3,PLAUR,SNAI1,VCAM1,VEGFA |
| ACVRL1 | kinase | | 3,37E-05 | CDKN1A,CXCL8,EFNA1,EFNB2,GDF15,ICAM1,PLAU,PLAUR,SELE,SMAD7,STAT1,VCAM1,VEGFA |
| miR-326-5p (and other miRNAs w/seed GGGGGGC) | mature microRNA | Inhibited | 3,43E-05 | [illegible] |
| miR-155-3p (miRNAs w/seed UCCUACA) | mature microRNA | Inhibited | 3,43E-05 | [illegible] |
| IFNGR1 | transmembrane receptor | Activated | 3,44E-05 | BCL6,CCL20,CCL5,CXCL10,CXCL3,FAS,ICAM1,IDO1,IL1A,IL1B,IL6,IRF1,IRF7,MX1,TNF |
| CRP | other | | 3,44E-05 | CCL2,CCL5,CD40,CXCL8,EGR1,IL1B,IL1RN,IL6,IL6R,ITGAM,NOS3,PSMB8,PSMB9,TNF,TNFSF10 |
| N-acetylsphingosine | chemical reagent | Activated | 3,44E-05 | CAT,CDKN1A,CEBPB,COL1A1,CXCL2,CXCL8,EIF2AK3,FOS,JUN,PTGS2,RARA,SOD2,SPHK1,TNF,UCP2 |
| SCD | enzyme | Inhibited | 3,49E-05 | [illegible] |
| PP1 | chemical - kinase inhibitor | Inhibited | 3,49E-05 | BCL2L11,CDKN1B,CXCL8,FOS,HMOX1,IL1A,IL1B,IL1RN,JUN,ODC1,PTGS2,TJP1,TNF,VCAM1 |
| PLAUR | transmembrane receptor | | 3,49E-05 | CCL2,CCL5,CXCL3,EIF2AK3,FOSL1,ITGAM,JUN,JUNB,PLAU,PLAUR,SNAI1,SOCS1,TNF,VIM |
| AGTR1 | G-protein coupled receptor | | 3,49E-05 | ACE2,CYBA,DUSP1,EDN1,EGFR,FOS,HMOX1,IL6,JUN,NOS3,PTGS2,SGK1,TXNIP,XDH |
| THBS1 | other | | 3,49E-05 | CD69,COL1A1,COL4A1,EGR1,IL1B,IL6,ITGAM,LRP1,PLAU,THBS1,TNF,TNFRSF10A,TNFRSF10B,VEGFA |
| H-7 | chemical - kinase inhibitor | Inhibited | 3,49E-05 | AREG,COL1A1,CYP1A1,EGR1,FAS,FOS,IFIT2,ISG15,ITGA5,JUNB,PIM1,SELE,SOD2,VDR |
| PC-SPES | chemical drug | | 3,61E-05 | [illegible] |
| RASSF1 | other | | 3,63E-05 | [illegible] |
| phorbol esters | chemical - other | Activated | 3,63E-05 | [illegible] |
| VCP | enzyme | | 3,69E-05 | CCL2,CCL5,CXCL8,DDIT3,DUSP1,ERCC6,HERPUD1,NFKB2,SYNE1,TNFSF13B,VCAM1 |
| PLK4 | kinase | Activated | 3,69E-05 | CD40,CITED2,CMPK2,CXCL10,EDN1,IFIT2,IFNB1,IL15,IL15RA,IL1B,IRF1 |
| ceramide | chemical - endogenous mammalian | | 3,69E-05 | ABCA12,CASP1,CFLAR,COL1A1,CXCL8,FOS,ICAM1,IL1B,JUN,PTGS2,SLC5A5 |
| miR-1300 (miRNAs w/seed UCCUGAG) | mature microRNA | Inhibited | 3,72E-05 | [illegible] |
| miR-521 (miRNAs w/seed UAGUGAG) | mature microRNA | Inhibited | 3,76E-05 | [illegible] |
| miR-548an (miRNAs w/seed AAAGGCA) | mature microRNA | Inhibited | 3,82E-05 | [illegible] |
| let-7f-2-3p (and other miRNAs w/seed UAUACAG) | mature microRNA | Inhibited | 3,86E-05 | [illegible] |
| NCOR2 | transcription regulator | | 3,89E-05 | [illegible] |
| EIF4E | translation regulator | Activated | 3,94E-05 | [illegible] |
| FOXO4 | transcription regulator | | 4,09E-05 | [illegible] |
| PARP1 | enzyme | | 4,10E-05 | [illegible] |
| TET2 | enzyme | | 4,15E-05 | [illegible] |
| lenalidomide | chemical drug | | 4,15E-05 | BIRC2,BIRC3,CFLAR,DDIT3,PTGS2,TNFRSF10B,TRAF1,XIAP |
| AIMP1 | cytokine | Activated | 4,15E-05 | CCL2,CCL5,CXCL2,CXCL8,ICAM1,IL12A,IL1B,TNF |
| IL18R1 | transmembrane receptor | | 4,15E-05 | IL12A,IL1A,IL1B,IL6,SOCS1,SOCS3,STAT1,TNF |
| BTC | growth factor | Activated | 4,15E-05 | CCL20,CXCL8,ERBB3,HAS2,IL6,IRS2,ITGAM,PTGS2 |
| [illegible] | [illegible] | [illegible] | [illegible] | [illegible] |

| | | | | |
|---|---|---|---|---|
| lithium chloride | chemical drug | | 1,52E-04 | [illegible] |
| diethylnitrosamine | chemical toxicant | | 1,53E-04 | [illegible] |
| miR-183-5p (miRNAs w/seed AUGGCAC) | mature microRNA | Inhibited | 1,53E-04 | [illegible] |
| miR-145-5p (and other miRNAs w/seed UCCAGUU) | mature microRNA | Inhibited | 1,54E-04 | [illegible] |
| miR-133a-3p (miRNAs w/seed [illegible]) | mature microRNA | Inhibited | 1,55E-04 | [illegible] |
| NRIP1 | transcription regulator | Activated | 1,55E-04 | [illegible] |
| miR-567 (miRNAs w/seed [illegible]) | mature microRNA | Inhibited | 1,55E-04 | [illegible] |
| LGR4 | transmembrane receptor | | 1,57E-04 | [illegible] |
| miR-4753-5p (miRNAs w/seed AAGGCCA) | mature microRNA | Inhibited | 1,58E-04 | [illegible] |
| Pro-inflammatory Cytokine | group | Activated | 1,61E-04 | [illegible] |
| CRH | cytokine | Activated | 1,61E-04 | [illegible] |
| AIRE | transcription regulator | Inhibited | 1,62E-04 | [illegible] |
| crocidolite asbestos | chemical toxicant | Activated | 1,62E-04 | [illegible] |
| clodronic acid | chemical drug | | 1,62E-04 | [illegible] |
| [illegible] | biologic drug | | 1,64E-04 | [illegible] |
| ZBTB20 | transcription regulator | | 1,66E-04 | [illegible] |
| miR-138-1-3p (miRNAs w/seed CUACUUC) | mature microRNA | Inhibited | 1,66E-04 | [illegible] |
| miR-3547 (and other miRNAs w/seed GAGCACC) | mature microRNA | Inhibited | 1,70E-04 | [illegible] |
| miR-199a-3p (and other miRNAs w/seed CAGUAGU) | mature microRNA | Inhibited | 1,71E-04 | [illegible] |
| miR-3171 (miRNAs w/seed GAUGUAU) | mature microRNA | Inhibited | 1,71E-04 | [illegible] |
| F630028O10Rik | other | Inhibited | 1,73E-04 | [illegible] |
| ANKRD17 | other | Activated | 1,73E-04 | [illegible] |
| rhesus theta-defensin 1 | chemical - endogenous mammalian | | 1,73E-04 | [illegible] |
| SELE | transmembrane receptor | | 1,73E-04 | [illegible] |
| LUM | other | Activated | 1,73E-04 | [illegible] |
| Igkv1-117 | other | | 1,73E-04 | [illegible] |
| CTTN | other | Activated | 1,73E-04 | [illegible] |
| linuron | chemical reagent | | 1,73E-04 | [illegible] |
| poly(U)RNA | chemical reagent | | 1,73E-04 | [illegible] |
| miR-223-3p (miRNAs w/seed GUCAGUU) | mature microRNA | Inhibited | 1,80E-04 | [illegible] |
| mir-204 | microRNA | | 1,81E-04 | [illegible] |
| Gcg | other | | 1,81E-04 | [illegible] |
| miR-876-3p (miRNAs w/seed GGUGGUU) | mature microRNA | Inhibited | 1,82E-04 | [illegible] |
| prostaglandin A1 | chemical - endogenous non-mammalian | | 1,84E-04 | [illegible] |
| CaMKII | complex | Activated | 1,84E-04 | [illegible] |
| NOX1 | enzyme | | 1,84E-04 | [illegible] |
| cuprizone | chemical toxicant | | 1,84E-04 | [illegible] |
| [illegible] | chemical drug | | 1,84E-04 | [illegible] |
| miR-3687 (and other miRNAs w/seed CCGGACA) | mature microRNA | Inhibited | 1,85E-04 | [illegible] |
| YAP1 | transcription regulator | Activated | 1,85E-04 | [illegible] |
| TO-901317 | chemical reagent | | 1,86E-04 | [illegible] |
| TFRC | transporter | Inhibited | 1,88E-04 | [illegible] |
| ATF6 | transcription regulator | | 1,91E-04 | [illegible] |
| GFI2 | transcription regulator | | 1,91E-04 | [illegible] |
| miR-3907 (miRNAs w/seed GGUGCUC) | mature microRNA | Inhibited | 1,92E-04 | [illegible] |
| miR-4731-5p (miRNAs w/seed GCUGGGG) | mature microRNA | Inhibited | 1,93E-04 | [illegible] |
| miR-584-3p (miRNAs w/seed CAGUUCC) | mature microRNA | Inhibited | 1,93E-04 | [illegible] |
| MACROH2A1 | other | | 1,93E-04 | [illegible] |
| MTOR | kinase | Activated | 1,95E-04 | [illegible] |
| KLF4 | transcription regulator | Activated | 1,96E-04 | [illegible] |
| miR-3682-5p (miRNAs w/seed UACUUCU) | mature microRNA | Inhibited | 1,96E-04 | [illegible] |
| miR-548au-3p (miRNAs w/seed GGCAGUU) | mature microRNA | Inhibited | 1,97E-04 | [illegible] |
| miR-1262-5p (miRNAs w/seed GAAGGAA) | mature microRNA | Inhibited | 2,01E-04 | [illegible] |
| Sykb | group | | 2,03E-04 | [illegible] |
| bis(3',5')-cyclic diguanylic acid | chemical - endogenous non-mammalian | Activated | 2,03E-04 | [illegible] |
| poly(dG-dC) | chemical reagent | Activated | 2,03E-04 | [illegible] |
| iguratimod | chemical drug | Inhibited | 2,03E-04 | [illegible] |
| HLA-DQ | complex | | 2,03E-04 | [illegible] |
| REV1 | enzyme | Inhibited | 2,03E-04 | [illegible] |
| MARCO | transmembrane receptor | | 2,03E-04 | [illegible] |
| ISG15 | other | Inhibited | 2,03E-04 | [illegible] |
| CLIC4 | ion channel | | 2,03E-04 | [illegible] |
| C4A/C4B | other | | 2,03E-04 | [illegible] |
| IL18RAP | transmembrane receptor | | 2,03E-04 | [illegible] |
| KLKB1 | peptidase | | 2,03E-04 | [illegible] |
| [illegible] | other | Activated | 2,03E-04 | [illegible] |
| cepharanthine | chemical reagent | Inhibited | 2,03E-04 | [illegible] |
| BC-1215 | chemical reagent | Inhibited | 2,03E-04 | [illegible] |
| Immunoglobulin Lambda Light Chain | group | | 2,03E-04 | [illegible] |
| AP20187 | chemical reagent | | 2,03E-04 | [illegible] |
| perifosine | chemical drug | | 2,03E-04 | [illegible] |
| ubiquinone 9 | chemical - endogenous mammalian | Inhibited | 2,03E-04 | [illegible] |
| [illegible] | chemical reagent | Inhibited | 2,03E-04 | [illegible] |
| polyphosphate | chemical - endogenous mammalian | | 2,03E-04 | [illegible] |
| borrelia burgdorferi strain B31 peptidoglycan | chemical reagent | Activated | 2,03E-04 | [illegible] |
| miR-10a-5p (and other miRNAs w/seed ACCCUGU) | mature microRNA | Inhibited | 2,05E-04 | [illegible] |
| PEBP1 | other | | 2,06E-04 | [illegible] |
| everolimus | chemical drug | | 2,06E-04 | [illegible] |
| miR-4477b (miRNAs w/seed UUAAGGA) | mature microRNA | Inhibited | 2,07E-04 | [illegible] |
| ADM | other | | 2,10E-04 | [illegible] |
| miR-6529-5p (and other miRNAs w/seed GGGAGGA) | mature microRNA | Inhibited | 2,12E-04 | [illegible] |
| Pdgf Ab | complex | Activated | 2,13E-04 | [illegible] |
| HCAR2 | G-protein coupled receptor | | 2,13E-04 | [illegible] |
| CPA3 | enzyme | Inhibited | 2,13E-04 | [illegible] |
| IL17R | complex | | 2,13E-04 | [illegible] |
| hemoglobin | complex | Activated | 2,13E-04 | [illegible] |
| PLCG2 | enzyme | | 2,13E-04 | [illegible] |
| PLA2G4A | enzyme | Activated | 2,13E-04 | [illegible] |
| MAPKAPK3 | kinase | | 2,13E-04 | [illegible] |
| superoxide | chemical - endogenous mammalian | | 2,13E-04 | [illegible] |
| sertraline | chemical drug | | 2,13E-04 | [illegible] |
| miR-6999-3p (miRNAs w/seed CACUACC) | mature microRNA | Inhibited | 2,13E-04 | [illegible] |
| GAS6 | growth factor | | 2,14E-04 | [illegible] |
| MALT1 | peptidase | Activated | 2,14E-04 | [illegible] |
| CTSS | peptidase | | 2,14E-04 | [illegible] |
| DTX1 | transcription regulator | | 2,14E-04 | [illegible] |
| CTSB | peptidase | Activated | 2,14E-04 | [illegible] |
| miR-4777-3p (miRNAs w/seed UACCUCA) | mature microRNA | Inhibited | 2,15E-04 | [illegible] |
| miR-3681-3p (miRNAs w/seed AUCAUGG) | mature microRNA | Inhibited | 2,16E-04 | [illegible] |
| miR-361-3p (miRNAs w/seed CCCCCAG) | mature microRNA | Inhibited | 2,17E-04 | [illegible] |
| Ahr-aryl hydrocarbon-Arnt | complex | Activated | 2,18E-04 | [illegible] |
| Collagen Alpha1 | group | Activated | 2,18E-04 | [illegible] |
| RALA | enzyme | | 2,18E-04 | [illegible] |
| ST14 | peptidase | Activated | 2,18E-04 | [illegible] |
| dimethyl fumarate | chemical drug | | 2,18E-04 | [illegible] |
| coomassie brilliant blue | chemical drug | | 2,18E-04 | [illegible] |
| picryl chloride | chemical toxicant | | 2,18E-04 | [illegible] |
| pentobarbital | chemical drug | | 2,18E-04 | [illegible] |
| spermidine | chemical - endogenous mammalian | | 2,18E-04 | [illegible] |
| spermine | chemical - endogenous mammalian | | 2,18E-04 | [illegible] |
| auranofin | chemical drug | | 2,18E-04 | [illegible] |
| growth factor | group | | 2,18E-04 | [illegible] |
| miR-587 (miRNAs w/seed UUCCAUA) | mature microRNA | Inhibited | 2,18E-04 | [illegible] |
| POU2F1 | transcription regulator | | 2,18E-04 | [illegible] |
| miR-141-5p (and other miRNAs w/seed AUCUUCC) | mature microRNA | Inhibited | 2,19E-04 | [illegible] |
| IKZF3 | transcription regulator | Inhibited | 2,20E-04 | [illegible] |
| mir-10 | microRNA | | 2,20E-04 | [illegible] |
| COL18A1 | other | Inhibited | 2,20E-04 | [illegible] |
| tert-butyl-hydroquinone | chemical reagent | Activated | 2,22E-04 | [illegible] |
| Notch | group | | 2,24E-04 | [illegible] |
| ITCH | enzyme | | 2,25E-04 | [illegible] |
| miR-3169 (miRNAs w/seed AGGACUG) | mature microRNA | Inhibited | 2,25E-04 | [illegible] |
| miR-6734-3p (miRNAs w/seed CCUUCCC) | mature microRNA | Inhibited | 2,29E-04 | [illegible] |
| testosterone | chemical - endogenous mammalian | | 2,32E-04 | [illegible] |
| ursodeoxycholic acid | chemical - endogenous mammalian | | 2,34E-04 | [illegible] |
| BCL2L1 | other | | 2,34E-04 | [illegible] |
| oxaliplatin | chemical drug | | 2,34E-04 | [illegible] |
| lomustine | chemical drug | | 2,34E-04 | [illegible] |
| N-cor | group | | 2,36E-04 | [illegible] |
| losartan potassium | chemical drug | | 2,36E-04 | [illegible] |
| JAK1/2 | group | Activated | 2,37E-04 | [illegible] |
| PPRC1 | transcription regulator | Activated | 2,38E-04 | [illegible] |
| quinolinic acid | chemical - endogenous mammalian | Activated | 2,38E-04 | [illegible] |
| n-3 fatty acids | chemical drug | | 2,38E-04 | [illegible] |
| HDAC4 | transcription regulator | | 2,38E-04 | [illegible] |
| miR-2277-3p (miRNAs w/seed GACAGCG) | mature microRNA | Inhibited | 2,41E-04 | [illegible] |
| miR-4271 (and other miRNAs w/seed GGGGAAG) | mature microRNA | Inhibited | 2,48E-04 | [illegible] |
| daidzein | chemical drug | | 2,49E-04 | [illegible] |
| miR-5700 (miRNAs w/seed AAUGCAU) | mature microRNA | Inhibited | 2,49E-04 | [illegible] |
| miR-6509-3p (miRNAs w/seed UCCACUG) | mature microRNA | Inhibited | 2,52E-04 | [illegible] |
| miR-3063-5p (and other miRNAs w/seed GGAGAUC) | mature microRNA | Inhibited | 2,53E-04 | [illegible] |
| PPARD | ligand-dependent nuclear receptor | | 2,54E-04 | [illegible] |
| arsenic trioxide | chemical drug | Activated | 2,55E-04 | [illegible] |
| colistin | biologic drug | | 2,55E-04 | [illegible] |
| NANOG | transcription regulator | | 2,60E-04 | [illegible] |
| Rock | group | | 2,63E-04 | [illegible] |
| iron | chemical - endogenous mammalian | | 2,63E-04 | [illegible] |
| pituitary adenylate cyclase-activating polypeptide | biologic drug | | 2,68E-04 | [illegible] |
| mir-30 (includes others) | microRNA | Inhibited | 2,68E-04 | [illegible] |
| miR-648 (miRNAs w/seed AGUGUGC) | mature microRNA | Inhibited | 2,68E-04 | [illegible] |
| metronidazole | chemical drug | | 2,72E-04 | [illegible] |
| trans-cinnamaldehyde | chemical drug | | 2,74E-04 | [illegible] |
| CXCR2 | G-protein coupled receptor | | 2,74E-04 | [illegible] |
| pifithrin-alpha | chemical reagent | | 2,74E-04 | [illegible] |
| cannabidiol | chemical drug | | 2,74E-04 | [illegible] |
| miR-18a-5p (and other miRNAs w/seed AAGGUGC) | mature microRNA | Inhibited | 2,78E-04 | [illegible] |
| adenosine | chemical - endogenous mammalian | | 2,78E-04 | [illegible] |
| RNF2 | transcription regulator | | 2,78E-04 | [illegible] |
| PTGES | enzyme | | 2,78E-04 | [illegible] |
| miR-221-5p (and other miRNAs w/seed CCUGGCA) | mature microRNA | Inhibited | 2,82E-04 | [illegible] |
| miR-6499-3p (miRNAs w/seed GCAGUGU) | mature microRNA | Inhibited | 2,82E-04 | [illegible] |
| miR-423-5p (and other miRNAs w/seed GAGGGGC) | mature microRNA | Inhibited | 2,83E-04 | [illegible] |
| miR-3102-3p (and other miRNAs w/seed AGCACCC) | mature microRNA | Inhibited | 2,87E-04 | [illegible] |
| alitretinoin | chemical drug | | 2,90E-04 | [illegible] |
| SRF | transcription regulator | | 2,91E-04 | [illegible] |
| ASPSCR1-TFE3 | fusion gene/product | Activated | 2,91E-04 | [illegible] |
| KDM2B | enzyme | | 2,92E-04 | [illegible] |
| TWIST2 | transcription regulator | | 2,92E-04 | [illegible] |
| dimethyl sulfoxide | chemical drug | Activated | 2,98E-04 | [illegible] |
| miR-146a-3p (and other miRNAs w/seed CUCUGAA) | mature microRNA | Inhibited | 3,02E-04 | [illegible] |
| CCR1 | G-protein coupled receptor | | 3,03E-04 | [illegible] |
| hydroxypropyl-beta-cyclodextrin | chemical drug | | 3,03E-04 | [illegible] |
| EGR3 | transcription regulator | | 3,04E-04 | [illegible] |
| IL15RA | transmembrane receptor | | 3,04E-04 | [illegible] |
| Fgf | group | Activated | 3,06E-04 | [illegible] |
| MMP14 | peptidase | | 3,06E-04 | [illegible] |
| LRP6 | transmembrane receptor | Inhibited | 3,06E-04 | [illegible] |
| SCAP | other | | 3,23E-04 | [illegible] |
| 3,4,5,3',4'-pentachlorobiphenyl | chemical toxicant | Activated | 3,23E-04 | [illegible] |
| benzo(a)pyrene | chemical toxicant | | 3,24E-04 | [illegible] |
| miR-548ax-5p (and other miRNAs w/seed GAAGUAA) | mature microRNA | Inhibited | 3,28E-04 | [illegible] |
| MOG | other | | 3,28E-04 | [illegible] |
| pyridaben | chemical toxicant | | 3,28E-04 | [illegible] |
| PELP1 | other | | 3,28E-04 | [illegible] |
| miR-5003-5p (miRNAs w/seed CACAACA) | mature microRNA | Inhibited | 3,31E-04 | [illegible] |
| miR-600 (miRNAs w/seed CUUACAG) | mature microRNA | Inhibited | 3,31E-04 | [illegible] |
| MNT | transcription regulator | Inhibited | 3,34E-04 | [illegible] |
| DPP-23 | chemical reagent | Activated | 3,34E-04 | [illegible] |
| metyrapone | chemical drug | | 3,34E-04 | [illegible] |
| idarubicin | chemical drug | Activated | 3,34E-04 | [illegible] |
| MMP9 | peptidase | | 3,36E-04 | [illegible] |
| TBX3 | transcription regulator | | 3,36E-04 | [illegible] |
| DYSF | other | | 3,38E-04 | [illegible] |
| miR-4668 (and other miRNAs w/seed AGGGGCA) | mature microRNA | Inhibited | 3,38E-04 | [illegible] |
| kanamycin A | chemical drug | | 3,40E-04 | [illegible] |
| IL11 | cytokine | | 3,41E-04 | [illegible] |
| vitamin D | chemical drug | | 3,41E-04 | [illegible] |
| miR-103-3p (and other miRNAs w/seed GCAGCAU) | mature microRNA | Inhibited | 3,43E-04 | [illegible] |
| KDM3B | enzyme | | 3,44E-04 | [illegible] |
| NAMPT | cytokine | Activated | 3,44E-04 | [illegible] |
| TNC | other | | 3,44E-04 | [illegible] |
| ELANE | peptidase | | 3,44E-04 | [illegible] |
| U73122 | chemical reagent | Inhibited | 3,44E-04 | [illegible] |
| Tnfsf18 | other | | 3,46E-04 | [illegible] |
| RLIM | enzyme | | 3,46E-04 | [illegible] |
| APP | transporter | | 3,46E-04 | [illegible] |
| CNB-001 | chemical reagent | Activated | 3,46E-04 | [illegible] |
| sevoflurane | chemical drug | | 3,46E-04 | [illegible] |
| orlistat | chemical drug | Activated | 3,46E-04 | [illegible] |
| ONECUT1 | transcription regulator | | 3,49E-04 | [illegible] |
| miR-6509-5p (miRNAs w/seed UUAGGUA) | mature microRNA | Inhibited | 3,55E-04 | [illegible] |
| miR-4771 (miRNAs w/seed GCAGACU) | mature microRNA | Inhibited | 3,57E-04 | [illegible] |
| miR-3117-3p (miRNAs w/seed UAGGACU) | mature microRNA | Inhibited | 3,58E-04 | [illegible] |
| miR-3127-3p (and other miRNAs w/seed CCCCUUC) | mature microRNA | Inhibited | 3,60E-04 | [illegible] |
| miR-6511a-5p (and other miRNAs w/seed AGGCAGA) | mature microRNA | Inhibited | 3,61E-04 | [illegible] |
| miR-4762-5p (miRNAs w/seed CAGGCAA) | mature microRNA | Inhibited | 3,62E-04 | [illegible] |
| miR-548o-3p (and other miRNAs w/seed CAAAACU) | mature microRNA | Inhibited | 3,64E-04 | [illegible] |
| LEF1 | transcription regulator | Activated | 3,65E-04 | [illegible] |
| MED1 | transcription regulator | Activated | 3,65E-04 | [illegible] |
| TCF12 | transcription regulator | | 3,67E-04 | [illegible] |
| miR-6866a-3p (miRNAs w/seed AUACAAU) | mature microRNA | Inhibited | 3,71E-04 | [illegible] |
| monophosphoryl lipid A | chemical drug | Activated | 3,72E-04 | [illegible] |
| amphotericin B | chemical drug | | 3,72E-04 | [illegible] |
| nystatin | chemical drug | | 3,72E-04 | [illegible] |
| cetuximab | biologic drug | | 3,72E-04 | [illegible] |
| Nos | group | | 3,72E-04 | [illegible] |
| IFNL2 | other | Activated | 3,72E-04 | [illegible] |
| ZBTB33 | transcription regulator | | 3,72E-04 | [illegible] |
| mir-160 | microRNA | | 3,72E-04 | [illegible] |
| BDKRB2 | G-protein coupled receptor | | 3,72E-04 | [illegible] |
| NRP1 | transmembrane receptor | | 3,72E-04 | [illegible] |
| dexmedetomidine | chemical drug | | 3,72E-04 | [illegible] |
| lauric acid | chemical - endogenous mammalian | Activated | 3,72E-04 | [illegible] |
| MIR17HG | other | Inhibited | 3,75E-04 | [illegible] |
| ERG | transcription regulator | Activated | 3,75E-04 | [illegible] |
| miR-548e-5p (miRNAs w/seed AAAAGCA) | mature microRNA | Inhibited | 3,76E-04 | [illegible] |
| miR-515-5p (and other miRNAs w/seed UCUCCAA) | mature microRNA | Inhibited | 3,77E-04 | [illegible] |
| H1-6 | other | | 3,81E-04 | [illegible] |
| H1f1 | other | | 3,81E-04 | [illegible] |
| BSG | transporter | | 3,82E-04 | [illegible] |
| TEAD4 | transcription regulator | Activated | 3,86E-04 | [illegible] |
| PIN1 | enzyme | | 3,86E-04 | [illegible] |
| ADCY | group | | 3,86E-04 | [illegible] |
| NFAT (complex) | complex | | 3,86E-04 | [illegible] |
| PDGF-AA | complex | | 3,86E-04 | [illegible] |
| JUN/JUNB/JUND | group | | 3,86E-04 | [illegible] |
| MAP3K3 | kinase | | 3,86E-04 | [illegible] |
| STK4 | kinase | | 3,86E-04 | [illegible] |
| SAP30 | other | Inhibited | 3,86E-04 | [illegible] |
| oxazolone | chemical reagent | Activated | 3,86E-04 | [illegible] |
| SB 290157 | chemical reagent | | 3,86E-04 | [illegible] |
| miR-6776-3p (miRNAs w/seed AACCACC) | mature microRNA | Inhibited | 4,02E-04 | [illegible] |
| miR-548at-5p (miRNAs w/seed AAAGUUA) | mature microRNA | Inhibited | 4,03E-04 | [illegible] |
| galactosylceramide-alpha | chemical reagent | Activated | 4,06E-04 | [illegible] |
| miR-8086 (miRNAs w/seed GCUAGUC) | mature microRNA | Inhibited | 4,06E-04 | [illegible] |
| PD-0325901 | chemical drug | | 4,10E-04 | [illegible] |
| ZBED6 | transcription regulator | | 4,10E-04 | [illegible] |
| daunorubicin | chemical drug | | 4,10E-04 | [illegible] |
| bardoxolone | chemical drug | | 4,10E-04 | [illegible] |
| ADAM17 | peptidase | Activated | 4,11E-04 | [illegible] |
| phenylephrine | chemical drug | Activated | 4,11E-04 | [illegible] |
| miR-376a-3p (miRNAs w/seed UUAGCAG) | mature microRNA | Inhibited | 4,14E-04 | [illegible] |
| miR-888-5p (miRNAs w/seed ACUCAAA) | mature microRNA | Inhibited | 4,14E-04 | [illegible] |
| SIRT6 | enzyme | | 4,17E-04 | [illegible] |
| [illegible] | chemical drug | Activated | 4,17E-04 | [illegible] |
| miR-12130-5p (and other miRNAs w/seed GUGGGGU) | mature microRNA | Inhibited | 4,20E-04 | [illegible] |
| taurocholic acid | chemical - endogenous mammalian | | 4,20E-04 | [illegible] |
| CLEC7A | transmembrane receptor | Activated | 4,20E-04 | [illegible] |
| B2M | transmembrane receptor | | 4,20E-04 | [illegible] |
| TGFBR2 | kinase | | 4,23E-04 | [illegible] |
| MMP3 | peptidase | | 4,24E-04 | [illegible] |
| STAT5a/b | group | Activated | 4,26E-04 | [illegible] |
| PPARA | ligand-dependent nuclear receptor | | 4,27E-04 | [illegible] |
| miR-3140-5p (miRNAs w/seed CCUGAAU) | mature microRNA | Inhibited | 4,28E-04 | [illegible] |
| THRB | ligand-dependent nuclear receptor | Inhibited | 4,29E-04 | [illegible] |
| miR-570-3p (miRNAs w/seed GAAAACA) | mature microRNA | Inhibited | 4,33E-04 | [illegible] |
| FGFR2 | kinase | | 4,36E-04 | [illegible] |
| EPHA5 | kinase | Inhibited | 4,36E-04 | [illegible] |
| TXNIP | other | | 4,38E-04 | [illegible] |
| dimethylnitrosamine | chemical toxicant | | 4,38E-04 | [illegible] |
| miR-30c-1-3p (and other miRNAs w/seed UGGGAGA) | mature microRNA | Inhibited | 4,42E-04 | [illegible] |
| miR-452-5p (and other miRNAs w/seed ACUGUUU) | mature microRNA | Inhibited | 4,48E-04 | [illegible] |
| miR-6856-3p (miRNAs w/seed ACAGCCC) | mature microRNA | Inhibited | 4,50E-04 | [illegible] |
| levodopa | chemical - endogenous mammalian | Activated | 4,62E-04 | [illegible] |
| miR-6901-5p (miRNAs w/seed GCAGGAA) | mature microRNA | Inhibited | 4,63E-04 | [illegible] |
| miR-10396-5p (and other miRNAs w/seed CUGCAGG) | mature microRNA | Inhibited | 4,65E-04 | [illegible] |
| miR-7706 (miRNAs w/seed AUAGCUC) | mature microRNA | Inhibited | 4,78E-04 | [illegible] |
| miR-136-5p (miRNAs w/seed CUCCAUU) | mature microRNA | Inhibited | 4,80E-04 | [illegible] |
| miR-4665-5p (miRNAs w/seed ACCGGGG) | mature microRNA | Inhibited | 4,81E-04 | [illegible] |
| miR-12201-5p (and other miRNAs w/seed GGAAGGA) | mature microRNA | Inhibited | 4,85E-04 | [illegible] |
| miR-4489 (miRNAs w/seed GGGGCUA) | mature microRNA | Inhibited | 4,88E-04 | [illegible] |
| miR-2355-5p (miRNAs w/seed UCCCCAG) | mature microRNA | Inhibited | 4,88E-04 | [illegible] |
| miR-6825-5p (and other miRNAs w/seed GGGGAGG) | mature microRNA | Inhibited | 4,91E-04 | [illegible] |
| PDLIM2 | other | | 4,91E-04 | [illegible] |
| trinitrobenzenesulfonic acid | chemical reagent | Activated | 4,91E-04 | [illegible] |
| NOX4 | enzyme | Activated | 4,95E-04 | [illegible] |
| carbamylcholine | chemical drug | Activated | 4,95E-04 | [illegible] |
| propofol | chemical drug | | 4,95E-04 | [illegible] |
| acetaminophen | chemical drug | Activated | 4,95E-04 | [illegible] |
| Cyp4a14 | enzyme | Activated | 4,96E-04 | [illegible] |
| Tlr13 | other | Activated | 4,96E-04 | [illegible] |
| FANCA | other | | 4,96E-04 | [illegible] |
| S100a7a | other | Activated | 4,96E-04 | [illegible] |
| HS6ST1 | enzyme | Activated | 4,96E-04 | [illegible] |
| COPA | transporter | | 4,96E-04 | [illegible] |
| TAPBP | transporter | | 4,96E-04 | [illegible] |
| [illegible] | chemical - endogenous non-mammalian | Inhibited | 4,96E-04 | [illegible] |
| DIABLO | other | Inhibited | 4,96E-04 | [illegible] |
| ULBP1 | transmembrane receptor | | 4,96E-04 | [illegible] |
| vadimezan | chemical drug | Activated | 4,96E-04 | [illegible] |
| mechlorethamine | chemical drug | Activated | 4,96E-04 | [illegible] |
| domoic acid | chemical toxicant | Activated | 4,96E-04 | [illegible] |
| palmitoyl-Cys((RS)-2,3-di(palmitoyloxy)-propyl)-Ala-Gly-OH | chemical reagent | Activated | 4,96E-04 | [illegible] |
| theaflavin | chemical - endogenous non-mammalian | | 4,96E-04 | [illegible] |
| miR-495-3p (and other miRNAs w/seed AACAAAC) | mature microRNA | Inhibited | 5,00E-04 | [illegible] |
| miR-6762-5p (miRNAs w/seed UAGGCAG) | mature microRNA | Inhibited | 5,00E-04 | [illegible] |
| maneb | chemical toxicant | | 5,06E-04 | [illegible] |
| puromycin | chemical - endogenous non-mammalian | | 5,07E-04 | [illegible] |
| ssRNA40 | chemical reagent | Activated | 5,07E-04 | [illegible] |
| [illegible] | chemical drug | | 5,07E-04 | [illegible] |
| [illegible] | chemical toxicant | | 5,07E-04 | [illegible] |
| leptomycin B | chemical drug | | 5,07E-04 | [illegible] |
| miR-548a-3p (miRNAs w/seed [illegible]) | mature microRNA | Inhibited | 5,13E-04 | [illegible] |
| [illegible] | chemical drug | | 5,16E-04 | [illegible] |
| 1-palmitoyl-2-oleoylglycero-3-phosphoglycerol | chemical reagent | | 5,16E-04 | [illegible] |
| MARCHF3 | other | Inhibited | 5,16E-04 | [illegible] |
| SPINDOC | other | Inhibited | 5,16E-04 | [illegible] |
| sGC | complex | Activated | 5,16E-04 | [illegible] |
| Tlr12 | other | Activated | 5,16E-04 | [illegible] |
| IL31RA | transmembrane receptor | | 5,16E-04 | [illegible] |
| FGA | other | | 5,16E-04 | [illegible] |
| SPIN1 | other | Activated | 5,16E-04 | [illegible] |
| ARL8B | enzyme | | 5,16E-04 | [illegible] |
| PLAA | other | | 5,16E-04 | [illegible] |
| polyinosine-polycytidylic acid/polyethylenimine formulation | chemical reagent | | 5,16E-04 | [illegible] |
| sulfamethoxazole/trimethoprim | chemical drug | | 5,16E-04 | [illegible] |
| 3-hydroxybutyric acid | chemical - endogenous mammalian | | 5,16E-04 | [illegible] |
| tranexamic acid | chemical drug | Inhibited | 5,16E-04 | [illegible] |
| roflumilast | chemical drug | | 5,16E-04 | [illegible] |
| paxilline | chemical - endogenous non-mammalian | | 5,16E-04 | [illegible] |
| sappanone A | chemical - endogenous non-mammalian | Inhibited | 5,16E-04 | [illegible] |
| allosamidin | chemical reagent | | 5,16E-04 | [illegible] |
| miR-377-3p (miRNAs w/seed UCACACA) | mature microRNA | Inhibited | 5,23E-04 | [illegible] |
| TGFBR1 | kinase | Activated | 5,24E-04 | [illegible] |
| miR-132-3p (and other miRNAs w/seed AACAGUC) | mature microRNA | Inhibited | 5,26E-04 | [illegible] |
| miR-4653-3p (miRNAs w/seed GGAGUUA) | mature microRNA | Inhibited | 5,29E-04 | [illegible] |
| miR-636 (and other miRNAs w/seed GUGCGGC) | mature microRNA | Inhibited | 5,31E-04 | [illegible] |
| miR-6732-3p (miRNAs w/seed AACCCUG) | mature microRNA | Inhibited | 5,33E-04 | [illegible] |
| miR-101a-3p (miRNAs w/seed UACAGUA) | mature microRNA | Inhibited | 5,37E-04 | [illegible] |
| IKK (complex) | complex | Activated | 5,43E-04 | [illegible] |
| PLAAT4 | enzyme | Activated | 5,43E-04 | [illegible] |
| sodium orthovanadate | chemical reagent | | 5,43E-04 | [illegible] |
| miR-150-5p (and other miRNAs w/seed UCCCAAC) | mature microRNA | Inhibited | 5,47E-04 | [illegible] |
| miR-4752 (miRNAs w/seed UGUGGAU) | mature microRNA | Inhibited | 5,53E-04 | [illegible] |
| MTPN | transcription regulator | Activated | 5,54E-04 | [illegible] |
| PRKAA | group | | 5,55E-04 | [illegible] |
| SERPINA1 | other | | 5,55E-04 | [illegible] |
| miR-216a-5p (and other miRNAs w/seed [illegible]) | mature microRNA | Inhibited | 5,58E-04 | [illegible] |
| miR-106-5p (and other miRNAs w/seed CAAAGUG) | mature microRNA | Inhibited | 5,58E-04 | [illegible] |
| miR-4525 (and other miRNAs w/seed GGGGGAU) | mature microRNA | Inhibited | 5,59E-04 | [illegible] |
| miR-130b-5p (and other miRNAs w/seed CUCUUUC) | mature microRNA | Inhibited | 5,60E-04 | [illegible] |
| THRA | ligand-dependent nuclear receptor | | 5,61E-04 | [illegible] |
| geldanamycin | chemical - endogenous non-mammalian | Inhibited | 5,67E-04 | [illegible] |
| leukotriene B4 | chemical - endogenous mammalian | Activated | 5,67E-04 | [illegible] |
| GABA | chemical - endogenous mammalian | Inhibited | 5,67E-04 | [illegible] |
| miR-6843-5p (miRNAs w/seed CCCCCGG) | mature microRNA | Inhibited | 5,72E-04 | [illegible] |
| LPIN1 | phosphatase | | 5,72E-04 | [illegible] |
| PRKDC | kinase | | 5,72E-04 | [illegible] |
| RWJ 60,212 | chemical reagent | Inhibited | 5,72E-04 | [illegible] |
| bardoxolone methyl | chemical drug | | 5,72E-04 | [illegible] |
| miR-34a-3p (and other miRNAs w/seed AUCAGCA) | mature microRNA | Inhibited | 5,75E-04 | [illegible] |
| ZNF217 | transcription regulator | Inhibited | 5,80E-04 | [illegible] |
| miR-6815-5p (and other miRNAs w/seed AGGUGGC) | mature microRNA | Inhibited | 5,90E-04 | [illegible] |
| miR-6756-5p (and other miRNAs w/seed GGGUGGG) | mature microRNA | Inhibited | 5,91E-04 | [illegible] |
| inosine | chemical - endogenous mammalian | | 5,92E-04 | [illegible] |
| miR-5585-5p (miRNAs w/seed AUCCAGC) | mature microRNA | Inhibited | 5,94E-04 | [illegible] |
| misoprostol | chemical drug | | 6,03E-04 | [illegible] |
| C1q | complex | | 6,03E-04 | [illegible] |
| IL1RL2 | transmembrane receptor | Activated | 6,03E-04 | [illegible] |
| UNC93B1 | other | Activated | 6,03E-04 | [illegible] |
| PFKFB3 | kinase | | 6,03E-04 | [illegible] |
| H1-2 | other | | 6,03E-04 | [illegible] |
| TGIF1 | transcription regulator | Activated | 6,03E-04 | [illegible] |
| IGHE | other | Activated | 6,03E-04 | [illegible] |
| IFT57 | other | Activated | 6,03E-04 | [illegible] |

| | | | | |
|---|---|---|---|---|
| CDK6 | kinase | | 6,03E-04 | DHFR,IL1B,IL6,NFKBIZ,RBL2,SOCS3,VEGFA |
| VIPR1 | G-protein coupled receptor | | 6,03E-04 | CCL5,CD40,CXCL10,CXCL2,CXCL3,IRF1,TNF |
| MAML1 | transcription regulator | | 6,03E-04 | CXCL8,EGR1,HES1,IL1A,IL1B,IL7R,RND3 |
| RHOB | enzyme | | 6,03E-04 | CDKN1A,CXCL8,EGFR,ICAM1,IL6,NFKB1,PTGS2 |
| 101.10 peptide | chemical reagent | Inhibited | 6,03E-04 | IFNB1,IL1B,IL1RN,IL6,OXTR,PTGS2,TNF |
| cyclopiazonic acid | chemical - endogenous non-mammalian | Activated | 6,03E-04 | CXCL2,DDIT3,EGR1,FAS,FOS,IL6,TNF |
| concanamycin A | chemical drug | | 6,03E-04 | BST2,DAXX,ICAM1,IFNB1,NFKBIA,SELE,VCAM1 |
| erythromycin | chemical drug | Inhibited | 6,03E-04 | CSF2,CXCL8,ICAM1,IL1B,IL6,TLR3,TNF |
| CCL11 | cytokine | Activated | 6,09E-04 | CCL2,CDKN1B,CXCL11,CXCL2,CXCL3,CXCL8,CXCL9,FAS,ICAM1,IL1B,IL6,ITGAM,PDGFA,TNF,VEGFC |
| MAX | transcription regulator | Inhibited | 6,11E-04 | BCL6,CDKN1A,CDKN1B,CDKN2B,DDIT3,DHFR,DLEU1,DUSP2,EDN1,FADS2,GADD45A,GADD45B,KLF10,KLF6,LRRN3,MTF2,ODC1,RBBP8,[illegible],TXNIP |
| NCOA3 | transcription regulator | Activated | 6,11E-04 | AHR,ANKRD1,CCN1,CDKN1A,CYP1A1,CYP2B6,DDIT3,FGF2,HES1,HMOX1,IRF1,LAMP3,NCOA3,NFKBIA,PARD6B,PGK1,PTGS2,VEGFA,XBP1,XDH |
| miR-6507-3p (miRNAs w/seed AAAGUCC) | mature microRNA | Inhibited | 6,16E-04 | [illegible] |
| miR-6777-3p (miRNAs w/seed CCACUCU) | mature microRNA | Inhibited | 6,20E-04 | [illegible] |
| L-histidine | chemical - endogenous mammalian | Inhibited | 6,30E-04 | ATF3,ATF4,CEBPB,EGR1,PPP1R15A,SOD2 |
| PP2A | complex | | 6,30E-04 | ARC,FOXO3,LYN,PIM1,PIM3,PTGS2 |
| STAT1/3/5 dimer | complex | Activated | 6,30E-04 | BCL3,CISH,MT2A,PLAU,SOCS2,SOCS3 |
| PLZ | chemical drug | | 6,30E-04 | ACHE,BDNF,FAS,PMAIP1,PTGS2,TNF |
| SRT1720 | chemical drug | | 6,30E-04 | CXCL8,HAS2,HMMR,IL1B,IL6,TNF |
| LILRB4 | other | Inhibited | 6,30E-04 | CXCL10,CXCL11,IL1A,IL1B,IL6,TNF |
| RB1CC1 | other | | 6,30E-04 | CCL5,CDKN1A,CDKN2B,CXCL10,SKIL,SMAD7 |
| PIK3R2 | kinase | | 6,30E-04 | CXCL10,FOXO1,IRF7,ISG15,PIK3R1,PLAU |
| OGG1 | enzyme | | 6,30E-04 | CCL20,CXCL2,IL1A,IL1B,IL6,TNF |
| [illegible] N-oxide | chemical - endogenous mammalian | | 6,30E-04 | CXCL8,HMOX1,ICAM1,IL6,NFE2L2,TNF |
| midostaurin | chemical drug | | 6,30E-04 | BCL2L11,CCL2,HMOX1,MCL1,PTGS2,VIM |
| 4-methylnitrosamino-1-(3-pyridyl)-1-butanone | chemical toxicant | Activated | 6,30E-04 | CDKN1A,EGFR,FOS,IKBKE,IL6,JUN,JUNB,LOX,NFE2L2,NFKB1,PTGS2,SPP1 |
| miR-208a-3p (and other miRNAs w/seed UAAGACG) | mature microRNA | Inhibited | 6,50E-04 | [illegible] |
| apigenin | chemical - endogenous non-mammalian | | 6,50E-04 | CASP8,CCL2,CDKN1A,CDKN1B,CXCL3,CXCL8,CYP1A1,FOS,HMOX1,ICAM1,IFNB1,IL1B,IL6,PTGS2,SELE,SOD2,TNF,TNFRSF10B,VCAM1 |
| EHF | transcription regulator | | 6,61E-04 | ANGPTL4,CCL20,CDKN1B,CSF2,CYP1A1,EREG,GPR68,[illegible],HMOX1,IL1RN,IL6,KLF5,KLK6,KLK7,LYN,PLAUR,SAA2,SPDEF,TNFRSF10B,VEGFA,VEGFC |
| miR-4456 (and other miRNAs w/seed CUGGUGG) | mature microRNA | Inhibited | 6,70E-04 | [illegible] |
| miR-203a-5p (and other miRNAs w/seed AGUGGUC) | mature microRNA | Inhibited | 6,72E-04 | [illegible] |
| miR-499-5p (and other miRNAs w/seed UAAGACU) | mature microRNA | Inhibited | 6,78E-04 | [illegible] |
| miR-638 (miRNAs w/seed GGGAUCG) | mature microRNA | Inhibited | 6,78E-04 | [illegible] |
| miR-3162-5p (miRNAs w/seed UUGCCUC) | mature microRNA | Inhibited | 6,79E-04 | [illegible] |
| [illegible] (and other miRNAs w/seed GGCGGCG) | mature microRNA | Inhibited | 6,80E-04 | [illegible] |
| [illegible] (and other miRNAs w/seed [illegible]) | mature microRNA | Inhibited | 6,86E-04 | [illegible] |
| miR-5588-3p (miRNAs w/seed AGUCCCA) | mature microRNA | Inhibited | 6,94E-04 | [illegible] |
| [illegible] (and other miRNAs w/seed CUCAAAA) | mature microRNA | Inhibited | 6,99E-04 | [illegible] |
| C5AR1 | G-protein coupled receptor | | 6,99E-04 | C3,C5,CCL5,CD40,COL1A1,CSF3,CXCL10,CXCL8,DDIT3,GULP1,IL1B,IL6,TNF |
| PTGER2 | G-protein coupled receptor | | 7,02E-04 | [illegible] |
| arginine | chemical - endogenous mammalian | | 7,10E-04 | CXCL10,DDIT3,EGR1,GCH1,PIK3R1,PPARGC1A,PTGS2,SCD,SOD2,TNF,XBP1 |
| rolipram | chemical drug | | 7,10E-04 | AREG,BCL2L11,BDNF,CCL2,DUSP1,IL1B,IL6,PDE4B,PTGS2,SYNPO,TNF |
| phorbol 12,13-dibutyrate | chemical - endogenous non-mammalian | Activated | 7,10E-04 | CDKN1A,CSF2,CXCL8,FOS,MAP3K8,PPP1R12A,PRKCE,PTGS2,SPRY2,SPRY4,TNF |
| IL22 | cytokine | Activated | 7,11E-04 | [illegible] |
| miR-2052 (miRNAs w/seed GUUUUGA) | mature microRNA | Inhibited | 7,13E-04 | [illegible] |
| miR-6852-5p (and other miRNAs w/seed CCUGGGG) | mature microRNA | Inhibited | 7,13E-04 | [illegible] |
| NFATC3 | transcription regulator | Activated | 7,14E-04 | ABCA3,ATF3,BMP2,CASP10,CD69,COL1A1,EDN1,EGR2,EGR3,FOS,FOSB,FOXA2,IFNB1,IL6,JUN,PTGS2,TNF |
| ITGB3 | transmembrane receptor | | 7,14E-04 | [illegible] |
| sulindac sulfide | chemical drug | Activated | 7,14E-04 | [illegible] |
| [illegible] (and other miRNAs w/seed [illegible]) | mature microRNA | Inhibited | 7,15E-04 | [illegible] |
| miR-383-5p (miRNAs w/seed GAUCAGA) | mature microRNA | Inhibited | 7,16E-04 | [illegible] |
| [illegible] (and other miRNAs w/seed GGAGGAG) | mature microRNA | Inhibited | 7,18E-04 | [illegible] |
| [illegible] (and other miRNAs w/seed GGGAAAG) | mature microRNA | Inhibited | 7,20E-04 | [illegible] |
| [illegible] (and other miRNAs w/seed [illegible]) | mature microRNA | Inhibited | 7,32E-04 | [illegible] |
| sulindac | chemical drug | | 7,34E-04 | [illegible] |
| NR5A2 | ligand-dependent nuclear receptor | | 7,38E-04 | [illegible] |
| Ras homolog | group | | 7,40E-04 | ABCA1,CDKN1A,EGR1,FOS,NOS3,PLAU,PTGS2,TJP1 |
| [illegible] | enzyme | Activated | 7,40E-04 | CCL2,CCL20,CCL5,CXCL10,CXCL8,IL6,IL7,TNF |
| CCL4 | cytokine | Activated | 7,40E-04 | CCL2,CCR1,CD40,IFNB1,IL12A,IL6,TNF,XBP1 |
| SIN3B | transcription regulator | | 7,40E-04 | BCL6,DDIT3,DHFR,GADD45B,HES1,KLF10,KLF6,TXNIP |
| [illegible] | group | | 7,40E-04 | COL1A1,E2F3,JUN,LOX,LRP1,[illegible],ZEB1,ZEB2 |
| LIPG | enzyme | Activated | 7,40E-04 | ABCA1,CCL2,CSF1,IL12A,IL1B,IL6,TNF,VEGFA |
| A-Fos | chemical reagent | | 7,40E-04 | ARC,BDNF,CCL20,CSF2,EGR3,[illegible],ODC1,TNF |
| ST6GAL1 | enzyme | | 7,40E-04 | C3,C4A/C4B,CDKN1A,CDKN1B,EGFR,IL1A,IL1B,TNF |
| PD 169316 | chemical - kinase inhibitor | | 7,40E-04 | ABCA1,BIRC3,CDKN1A,MAPK8,PPARGC1A,PTGS2,SNAI1,VIM |
| chrysin | chemical - endogenous non-mammalian | Inhibited | 7,40E-04 | CCL2,CXCL10,HMOX1,ICAM1,IFNB1,IL6R,IL6ST,SELE |
| BDNF | growth factor | Activated | 7,45E-04 | [illegible] |
| miR-424-3p (miRNAs w/seed [illegible]) | mature microRNA | Inhibited | 7,46E-04 | [illegible] |
| HISTONE | group | | 7,67E-04 | AHR,CIITA,CSF2,[illegible],FGF2,FMR1,IL6,NFATC2,NOS2,NOS3 |
| [illegible] | peptidase | Activated | 7,67E-04 | ACO1,C3,C4A/C4B,C5,CXCL2,CXCL3,DDIT3,IL1B,ITGAM,PPARGC1A |
| KNG1 | other | Activated | 7,67E-04 | BDKRB2,COL1A1,CXCL8,EGFR,FGF2,FOS,IL1B,IL6,NOS3,PTGS2 |
| SMARCB1 | transcription regulator | Activated | 7,71E-04 | [illegible] |
| [illegible] | [illegible] | [illegible] | [illegible] | [illegible] |

[illegible]

| | | | | |
|---|---|---|---|---|
| glycochenodeoxycholate | chemical - endogenous mammalian | | 1,72E-03 | BIRC2,BIRC3,NOD,TNFRSF10A,TNFRSF10B,XIAP |
| MIR3 | other | | 1,72E-03 | COL1A1,GDF15,JUN,VIM,ZEB1,ZEB2 |
| mocetinostat | chemical drug | | 1,72E-03 | BDNF,BEST1,CXCL10,IL1B,MCL1,TNF |
| belnacasan | chemical drug | | 1,72E-03 | CASP1,CXCL3,IL18,NLRP3,SNCA,TNF |
| MFN2 | enzyme | | 1,72E-03 | ATF4,CXCL1,DDIT3,IL1B,PPP1R15A,XBP1 |
| N(2)-(gamma-D-glutamyl)-meso-2,2'-diaminopimelic acid | chemical reagent | Activated | 1,72E-03 | CASP1,CCL5,CXCL8,ICAM1,IL1A,IL1B |
| PSMB10 | peptidase | | 1,72E-03 | GATA3,IL1A,IL6,NFKB2,NFKBIA,TNFAIP3 |
| CSF3R | transmembrane receptor | | 1,72E-03 | CDKN1B,CISH,FOS,ITGAM,LYZ,SELE |
| BMX | kinase | | 1,72E-03 | CXCL8,FOS,HSPA1A/HSPA1B,IL6,PPARGC1A,TNFRSF1B |
| ADAM15 | peptidase | | 1,72E-03 | CCL5,EGFR,IFNB1,IL6,TNF,VEGFA |
| ID4 | transcription regulator | | 1,72E-03 | BBC3,CDKN1A,CDKN1B,CDKN2B,VEGFA,VIM |
| clasto-lactacystin beta-lactone | chemical - protease inhibitor | Activated | 1,72E-03 | ATF3,ATF4,DDIT3,MAPK8,PRKCE,TP53BP2 |
| pyruvic acid | chemical - endogenous mammalian | Activated | 1,72E-03 | FOS,NR4A1,TNFRSF10A,TNFRSF10B,TNFSF10,VEGFA |
| anti-benzo(a)pyrene-diol-epoxide | chemical toxicant | Activated | 1,72E-03 | BCL2L11,CDKN1A,GADD45B,IL6,JUN,TNF |
| D-tubocurarine | chemical drug | | 1,72E-03 | DHFR,EGR1,IL6,KMT2C,RBBP8,TNF |
| loxoribine | chemical drug | | 1,72E-03 | CXCL8,IFNB1,IL6,PTGS2,SDC1,TNF |
| buthionine sulfoximine | chemical drug | Activated | 1,72E-03 | CD274,FOS,HBEGF,HMOX1,NFKBIA,PTGS2 |
| [illegible] | chemical toxicant | | 1,72E-03 | CXCL3,DDIT3,FOS,FXYD2,HMOX1,PTGS2 |
| puromycin aminonucleoside | chemical reagent | | 1,74E-03 | [illegible] |
| miR-369-5p (miRNAs w/seed AAUCGAC) | mature microRNA | Inhibited | 1,75E-03 | [illegible] |
| miR-191-5p (and other miRNAs w/seed AACGGAA) | mature microRNA | Inhibited | 1,76E-03 | [illegible] |
| GATA4 | transcription regulator | | 1,78E-03 | [illegible] |
| miR-1262 (and other miRNAs w/seed UGGGUGA) | mature microRNA | Inhibited | 1,79E-03 | [illegible] |
| miR-943 (miRNAs w/seed UGACUGU) | mature microRNA | Inhibited | 1,80E-03 | [illegible] |
| RHOA | enzyme | | 1,82E-03 | CDKN1A,CDKN1B,CDKN2B,CEBPB,COL1A1,CXCL8,FOS,GDF15,ICAM1,IL1B,IL6,JUN,NFKB1,NFKBIB,NOS3,PLAU,RND3,SOX9,VCAM1 |
| miR-1343-5p (and other miRNAs w/seed GGGGAGC) | mature microRNA | Inhibited | 1,82E-03 | [illegible] |
| miR-5107-3p (and other miRNAs w/seed AACCUGU) | mature microRNA | Inhibited | 1,83E-03 | [illegible] |
| miR-6716-3p (miRNAs w/seed CCGAACU) | mature microRNA | Inhibited | 1,84E-03 | [illegible] |
| ABL1 | kinase | | 1,86E-03 | BCL2L11,BMP2,C3,CDKN1A,CDKN1B,CSF1,DHFR,FAS,GADD45A,IER3,VEGFA |
| histone deacetylase | complex | | 1,86E-03 | CCN1,JUNB,NDRG2,NOTCH1,PER1,PLAU,PPARGC1A,SSTR2,THBS1,TNF,TNFRSF10A |
| RBL1 | transcription regulator | | 1,86E-03 | BCL2L11,CDKN1A,CDKN1B,DHFR,DUSP10,FAS,FGF2,FOS,HES1,MCM5,NEK2,NOTCH1,PPARGC1A,RBL2,THBS1,TNF,ZEB1 |
| PLG | peptidase | Activated | 1,86E-03 | BDNF,CCL2,CCN1,CD40,COL1A1,CSF1,EDN1,GULP1,IL1A,IL1B,IL6,MFGE8,PIP5K1A,PLAU,PRKCE,PTGS2,TNF |
| aflatoxin B1 | chemical - endogenous non-mammalian | | 1,87E-03 | CD40,CYP1A1,CYP3A5,IRS2,STAT5A |
| Stat1-Stat2 | complex | Activated | 1,87E-03 | EIF2AK2,IRF1,IRF7,OAS1,TLR3 |
| PARP | group | | 1,87E-03 | APOE,CDKN1A,SLC9A5,SNAI1,VIM |
| IL10RB | transmembrane receptor | | 1,87E-03 | CXCL8,ICAM1,MX1,SOCS3,TNF |
| ANG | enzyme | | 1,87E-03 | CCL5,CDKN1A,CXCL10,CXCL8,IL6 |
| DCLK1 | kinase | | 1,87E-03 | NOTCH1,SNAI1,SNCA,ZEB1,ZEB2 |
| ITGA4 | transmembrane receptor | | 1,87E-03 | IL1B,IL1RN,ITGA5,TNF,TNFAIP3 |
| NCK1 | kinase | | 1,87E-03 | ATF4,CD69,DDIT3,FOS,PPP1R15A |
| NCF4 | enzyme | | 1,87E-03 | CCR1,FUT4,IL1B,IL6,TNF |
| IL9R | transmembrane receptor | | 1,87E-03 | AREG,BCL3,IL6,JUNB,PIM1 |
| PDE4B | enzyme | | 1,87E-03 | CCL5,CXCL10,CXCL11,DUSP1,TNF |
| GPC1 | transmembrane receptor | Activated | 1,87E-03 | CX3CL1,EFNA1,GNAI3,PTGS2,VEGFA |
| COP1 | enzyme | | 1,87E-03 | CDKN1A,CEBPB,FAS,ICAM1,PPARGC1A |
| HDAC8 | transcription regulator | | 1,87E-03 | BBC3,BMF,CDKN1A,IFNB1,SOCS1 |
| CYBA | enzyme | Activated | 1,87E-03 | CDKN1A,IL6,SOD2,TNF,VEGFA |
| pubchem compound 11368987 | chemical reagent | | 1,87E-03 | CXCL10,CXCL2,IL1B,IL6,TNFRSF10B |
| butylhydroxybutylnitrosamine | chemical toxicant | | 1,87E-03 | AREG,BTC,CDKN1A,CYP1A1,HBEGF |
| cortistatin A | chemical reagent | Activated | 1,87E-03 | BHLHE40,CDKN1B,FOSL2,IRF1,IRF8 |
| acetic acid | chemical - endogenous mammalian | Activated | 1,87E-03 | EGR1,FOS,JUN,JUNB,TNF |
| mesalamine | chemical drug | | 1,87E-03 | CD274,HMOX1,ODC1,PTGS2,SOD2 |
| polymyxin B | biologic drug | Inhibited | 1,87E-03 | CXCL8,IL1B,IL6,TNF,VCAM1 |
| miR-3913-3p (miRNAs w/seed GACAUCA) | mature microRNA | Inhibited | 1,87E-03 | [illegible] |
| miR-545-5p (miRNAs w/seed CAGUAAA) | mature microRNA | Inhibited | 1,88E-03 | [illegible] |
| flutamide | chemical drug | | 1,88E-03 | CCN1,CXCL2,CYP1A1,CYP1B1,CYP3A5,CYP4B1,FOS,FOXO1,GAS6,IL6,IL6ST,NOS3,NR3C1,NR4A1,ODC1,SERPINA5,SLCO3A1,TNF,TPD52L1,VIM |
| ACTL6A | other | Inhibited | 1,90E-03 | BBC3,BCL2L11,CCNB2,CDKN1A,CDKN1B,ETS2,FAS,HMOX1,MCL1 |
| CHADL | other | | 1,90E-03 | CDKN1A,FAS,IL6,KLF2,PDGFA,PTX3,SOX9,STEAP4,VCAM1 |
| SND1 | enzyme | | 1,90E-03 | ANGPTL4,APOBEC3G,DHFR,ERBB3,IDH2,NR2F1,PTGS2,SMURF1,SOX4 |
| epoxomicin | chemical - protease inhibitor | Activated | 1,90E-03 | BCL2L11,CFLAR,CXCL8,DDIT3,FOXO1,IRS2,OPTN,RICTOR,SQSTM1 |
| corticosterone | chemical - endogenous mammalian | | 1,91E-03 | [illegible] |
| miR-5580-3p (miRNAs w/seed ACCCCCU) | mature microRNA | Inhibited | 1,91E-03 | [illegible] |
| miR-4278 (miRNAs w/seed UAGGGGG) | mature microRNA | Inhibited | 1,91E-03 | [illegible] |
| 1,25-dihydroxyvitamin D | chemical drug | Activated | 1,92E-03 | CSF1,CXCL8,EGR1,FGFR1,FST,GLS,IGF2,IL6,SOCS1,TLR3,TNF,VDR |
| TLR8 | transmembrane receptor | Activated | 1,92E-03 | CCL2,CD40,CD83,CXCL10,CXCL2,CXCL8,IFNB1,IL12A,IL1B,IL6,TNF,VDR |
| PRKACA | kinase | | 1,92E-03 | BCL2L11,BDNF,CDKN1B,CEBPB,DIO2,HSPA1A/HSPA1B,MYH10,OXTR,PRKACB,SDC1,SOCS3,VCAM1 |
| D-glucose | chemical - endogenous mammalian | Activated | 1,92E-03 | CD69,COL1A1,CXCL10,CXCL2,ICAM1,IL1A,IL1B,IL6,ITGAM,SCD,THBS1,TNF |
| TEAD3 | transcription regulator | Activated | 1,93E-03 | ADM,AMOTL2,ARID5B,CCN1,EDN1,FILIP1L,LATS2,NFKBIZ,NUAK1,SLFN5,THBS1,TNNC1,TPM1 |
| GW501516 | chemical drug | | 1,93E-03 | [illegible] |
| methapyrilene | chemical drug | | 1,94E-03 | [illegible] |
| mibolerone | chemical drug | Inhibited | 1,94E-03 | [illegible] |
| miR-520d-5p (and other miRNAs w/seed UACAAAG) | mature microRNA | Inhibited | 1,94E-03 | [illegible] |
| miR-4464 (miRNAs w/seed CAGACUG) | mature microRNA | Inhibited | 1,96E-03 | [illegible] |
| miR-500a-5p (miRNAs w/seed AAUCCUU) | mature microRNA | Inhibited | 1,96E-03 | [illegible] |
| SPARC | other | | 1,96E-03 | [illegible] |
| miR-1913 (miRNAs w/seed CUGCCCC) | mature microRNA | Inhibited | 1,98E-03 | [illegible] |
| miR-3109-3p (and other miRNAs w/seed AGGGCCA) | mature microRNA | Inhibited | 1,99E-03 | [illegible] |
| miR-6809-3p (and other miRNAs w/seed AGCCACA) | mature microRNA | Inhibited | 2,00E-03 | [illegible] |
| TCOF1 | transporter | | 2,00E-03 | [illegible] |
| E2F4 | transcription regulator | | 2,01E-03 | [illegible] |
| miR-7106-5p (miRNAs w/seed UGUGGCC) | mature microRNA | Inhibited | 2,01E-03 | [illegible] |
| IL31 | other | Activated | 2,01E-03 | CCL2,CDKN1B,CISH,JUNB,SOCS1,SOCS2,SOCS3 |
| HNRNPU | transporter | | 2,01E-03 | AREG,CCL2,IL1B,IL6,SPP1,TNF,VCAM1 |
| CLEC4E | other | Activated | 2,01E-03 | AREG,CSF1,CXCL2,CXCL3,IL1B,IL6,TNF |
| chlorogenic acid | chemical drug | | 2,01E-03 | CCL2,HMOX1,IL6,NFATC2,PTGS2,TNF,VCAM1 |
| miR-548am-3p (and other miRNAs w/seed AAAAACU) | mature microRNA | Inhibited | 2,02E-03 | [illegible] |
| Cbp | group | | 2,03E-03 | CCL5,CSF2,CSF3,CXCL2,IL1A,IL1B,IL6,TNF |
| LINC00963 | other | | 2,03E-03 | DDIT3,EGFR,FGF19,FOXO3,ICAM1,IL4I1,IL6,TNF |
| UCN | other | | 2,03E-03 | CCL5,CXCL3,FOS,ICAM1,IL1B,IL6,PLA2G5,TNF |
| HOXA5 | transcription regulator | Activated | 2,03E-03 | CDKN1A,COL1A1,CXCL8,EGR1,GADD45B,IGF2,KLF10,SAT1 |
| CYP7A1 | enzyme | | 2,03E-03 | ABCA1,ABCG1,FDFT1,FGF19,IL1B,IL6,PTGS2,TNF |
| bisindolylmaleimide iv | chemical - kinase inhibitor | Inhibited | 2,03E-03 | CXCL8,EGR1,FGF2,FOS,IL6,ITGAM,NR4A1,TNF |
| miR-1178-5p (miRNAs w/seed AGGGUCA) | mature microRNA | Inhibited | 2,06E-03 | [illegible] |
| miR-3144-3p (miRNAs w/seed UAUACCU) | mature microRNA | Inhibited | 2,06E-03 | [illegible] |
| miR-6813-3p (miRNAs w/seed ACCUUGG) | mature microRNA | Inhibited | 2,07E-03 | [illegible] |
| nocodazole | chemical reagent | | 2,08E-03 | [illegible] |
| miR-1226-3p (miRNAs w/seed CACCAGC) | mature microRNA | Inhibited | 2,08E-03 | [illegible] |
| ARNTL | transcription regulator | | 2,13E-03 | [illegible] |
| CUX1 | transcription regulator | | 2,13E-03 | [illegible] |
| miR-4423-5p (miRNAs w/seed GUUGCCU) | mature microRNA | Inhibited | 2,14E-03 | [illegible] |
| miR-4473 (miRNAs w/seed UAGUGCU) | mature microRNA | Inhibited | 2,14E-03 | [illegible] |
| miR-4479 (miRNAs w/seed GCGCGGC) | mature microRNA | Inhibited | 2,15E-03 | [illegible] |
| miR-4364 (miRNAs w/seed CUCAGUC) | mature microRNA | Inhibited | 2,16E-03 | [illegible] |
| miR-4777-5p (miRNAs w/seed UCUAGAU) | mature microRNA | Inhibited | 2,16E-03 | [illegible] |
| miR-6888-5p (miRNAs w/seed AGGAGAU) | mature microRNA | Inhibited | 2,16E-03 | [illegible] |
| [illegible] | transcription regulator | | 2,17E-03 | ANGPTL4,CCL2,CCL5,CDKN1A,ERN1,HSPA1A/HSPA1B,ITGB2,JAK2,SPP1,ZFP36 |
| diclofenac | chemical drug | | 2,17E-03 | CDKN1A,FOS,GDF15,IL1A,IL6,ITGAM,PTGS2,TNF,VEGFA,VEGFC |
| FUS-DDIT3 | fusion gene/product | | 2,17E-03 | COL6A1,CXCL8,IL1B,IL6,KLF2,LOX,NAMPT,PTX3,SERPINB2,VIM |
| indole-3-carbinol | chemical drug | | 2,17E-03 | CDKN1A,CDKN1B,CRYZ,CYP3A5,FOS,GATA3,GDF15,NFE2L2,SPP1,TRAF1 |
| miR-3939 (miRNAs w/seed ACGCGCA) | mature microRNA | Inhibited | 2,19E-03 | [illegible] |
| miR-6860 (miRNAs w/seed GCAGCUC) | mature microRNA | Inhibited | 2,19E-03 | [illegible] |
| PTP4A1 | phosphatase | Activated | 2,22E-03 | [illegible] |
| EGLN | group | Inhibited | 2,22E-03 | [illegible] |
| PRKCB | kinase | Activated | 2,22E-03 | CD40,CD83,CDKN1A,EGR1,FGF2,FOS,ICAM1,IL6,IRS2,JUN,PPARGC1A,PTGS2,RELB,SOD2 |
| ZAP70 | kinase | | 2,24E-03 | [illegible] |
| pravastatin | chemical drug | | 2,24E-03 | ABCA1,CCL2,CD40,CYP2B6,GPAT4,HMOX1,ICAM1,IL1B,IL6,NOS3,PGF,SDC4,TNF,VEGFA |
| miR-548p (miRNAs w/seed AGCAAAA) | mature microRNA | Inhibited | 2,26E-03 | [illegible] |
| miR-6795-3p (miRNAs w/seed CCCUCUC) | mature microRNA | Inhibited | 2,27E-03 | [illegible] |
| miR-6762-3p (miRNAs w/seed GGCUGCU) | mature microRNA | Inhibited | 2,28E-03 | [illegible] |
| miR-8063 (miRNAs w/seed CAAAAUC) | mature microRNA | Inhibited | 2,30E-03 | [illegible] |
| conjugated linoleic acid | chemical drug | | 2,30E-03 | ANGPTL4,CXCL8,DUSP1,FOS,GDF15,IL10RA,IL1RN,PPARGC1A,PTGS2,SOCS3,TNF |
| benzyl isothiocyanate | chemical - endogenous non-mammalian | | 2,30E-03 | BCL2L11,CDKN1B,EGFR,FOS,ICAM1,MCL1,PTGS2,ROS1,SELE,VCAM1,ZBTB10 |
| semaxinib | chemical drug | | 2,30E-03 | AHR,CD24,CYP1B1,HMOX1,IL6,NFE2L2,NOS3,NR4A2,PTGS2,SELE,THBS1 |
| eflornithine | chemical drug | Activated | 2,30E-03 | C3,CDKN1A,CDKN1B,CDKN2B,DHFR,HLA-B,IL32,LGALS3BP,ODC1,PRKAG1,SOD2 |
| miR-190a-3p (miRNAs w/seed UAUAUAU) | mature microRNA | Inhibited | 2,31E-03 | [illegible] |
| miR-762 (and other miRNAs w/seed GGGCUGG) | mature microRNA | Inhibited | 2,31E-03 | [illegible] |
| GHR | transmembrane receptor | | 2,32E-03 | CDKN1A,CDKN1B,CISH,COL1A1,IGFBP2,IL1B,IL6,MOGAT1,NFKB1,SOCS2,SOCS3,SPP1,TNF |
| linoleic acid | chemical - endogenous mammalian | | 2,33E-03 | ABCA1,ANGPTL4,DDIT3,DUSP1,EDN1,ICAM1,IL1B,IL6,NEDD4L,NFKBIA,NOS3,PTGS2,SCD,UCP2,VCAM1,VEGFA |
| TEAD1 | transcription regulator | Activated | 2,33E-03 | ADM,AMOTL2,ARID5B,CCN1,CDKN1A,CDKN1B,EDN1,FILIP1L,LATS2,NFKBIZ,NUAK1,PTGS2,SLFN5,THBS1,TNNC1,TPM1 |
| LGALS1 | other | Activated | 2,33E-03 | AHR,CD69,CDKN1A,CDKN1B,CXCL2,DHCR7,GPCPD1,HBEGF,IFNGR2,IL1A,IL1B,IL6,INSIG1,PTX3,TFRC,TNF |
| HDAC4 | transcription regulator | | 2,33E-03 | COL1A1,ICAM1,IFNB1,IL6,JUN,JUNB,MYOG,NR3C1,PLAU,TNF,VCAM1,VIM |
| KDR | kinase | | 2,33E-03 | ANXA2,CREBBP,HMOX1,NOTCH1,NR4A1,PIM1,PTGS2,S1PR2,SELE,TJP1,VEGFA,XBP1 |
| GSK2816126 | chemical drug | | 2,33E-03 | [illegible] |
| vinorelbine | chemical drug | Activated | 2,36E-03 | CCL5,CDKN1A,CDKN1B,EGR1,FAS,IL1B,JUN,MAP2K6,MCL1,PMAIP1,PTGS2,SOD2 |
| miR-593-5p (and other miRNAs w/seed GGCACCA) | mature microRNA | Inhibited | 2,36E-03 | [illegible] |
| miR-6757-5p (and other miRNAs w/seed GGCCUCU) | mature microRNA | Inhibited | 2,36E-03 | [illegible] |
| NR1H4 | ligand-dependent nuclear receptor | | 2,40E-03 | [illegible] |
| miR-203a-3p (miRNAs w/seed UGAAAUG) | mature microRNA | Inhibited | 2,40E-03 | [illegible] |
| miR-296-5p (miRNAs w/seed GGGCCCC) | mature microRNA | Inhibited | 2,40E-03 | [illegible] |
| miR-7152-5p (miRNAs w/seed UUCCUGU) | mature microRNA | Inhibited | 2,40E-03 | [illegible] |
| miR-3185 (miRNAs w/seed GAAGAAG) | mature microRNA | Inhibited | 2,45E-03 | [illegible] |
| miR-5008 (miRNAs w/seed AAUGUGA) | mature microRNA | Inhibited | 2,46E-03 | [illegible] |
| miR-766-3p (miRNAs w/seed CUCCAGC) | mature microRNA | Inhibited | 2,47E-03 | [illegible] |
| NFATC1 | transcription regulator | Activated | 2,47E-03 | [illegible] |
| C1QA | other | | 2,47E-03 | C3,C4A/C4B,CD274,CXCL10,EGR1,IL6,MAP2,TNF,VIM |
| PRKCI | kinase | | 2,47E-03 | CXCL2,CXCL3,ELF3,FOXA2,HMOX1,IL1A,IL6,PLS1,PTGS2 |
| urethane | chemical toxicant | | 2,47E-03 | CDKN1A,CDKN1B,CDKN2B,CYP1A1,HMOX1,IL12A,IL1B,PTGS2,TEP1 |
| miR-571 (miRNAs w/seed GAGUUGG) | mature microRNA | Inhibited | 2,48E-03 | [illegible] |
| miR-2682-3p (and other miRNAs w/seed GCCUCUU) | mature microRNA | Inhibited | 2,48E-03 | [illegible] |
| miR-5582-5p (miRNAs w/seed AGGCACA) | mature microRNA | Inhibited | 2,48E-03 | [illegible] |
| HDAC5 | transcription regulator | | 2,48E-03 | [illegible] |
| [illegible] | chemical drug | | 2,50E-03 | ANXA2,BIRC3,C4A/C4B,ELF3,IER3,LXN,PRTFDC1,PSMB8,RFTN1,SPP1,STING1,TMSB10/TMSB4X,TSPAN8,UPP1,VIM |
| miR-4325 (miRNAs w/seed UGCACUU) | mature microRNA | Inhibited | 2,50E-03 | [illegible] |
| DIO2 | enzyme | | 2,51E-03 | [illegible] |
| miR-5681b (miRNAs w/seed GGUAUUG) | mature microRNA | Inhibited | 2,52E-03 | [illegible] |
| miR-6515 (miRNAs w/seed GGACUGG) | mature microRNA | Inhibited | 2,53E-03 | [illegible] |
| miR-501-3p (and other miRNAs w/seed AUGCACC) | mature microRNA | Inhibited | 2,58E-03 | [illegible] |
| miR-6527 (and other miRNAs w/seed GGUCUGC) | mature microRNA | Inhibited | 2,59E-03 | [illegible] |
| miR-6305 (miRNAs w/seed CUAGACA) | mature microRNA | Inhibited | 2,59E-03 | [illegible] |
| miR-548av-3p (miRNAs w/seed AAACUGC) | mature microRNA | Inhibited | 2,61E-03 | [illegible] |
| CDKN1A | kinase | | 2,62E-03 | [illegible] |
| 1'-acetoxychavicol acetate | chemical reagent | Inhibited | 2,62E-03 | BIRC2,BIRC3,CFLAR,ICAM1,PTGS2,XIAP |
| FOXO1 | transcription regulator | | 2,62E-03 | BCL2L11,CDKN1A,CDKN2B,DLX2,FST,NCOA3 |
| SPTLC2 | enzyme | Inhibited | 2,62E-03 | ATF4,COL1A1,DDIT3,EIF2AK3,THBS1,XBP1 |
| CRKL | kinase | Activated | 2,62E-03 | CXCL10,IFNB1,IL15,IL15RA,ISG20,TLR3 |
| SPHK2 | kinase | | 2,62E-03 | CCL5,CDKN1A,IL6,LYN,STAT4,TNF |
| TNFAIP2 | other | Activated | 2,62E-03 | CITED2,IFNB1,IL15RA,IRF1,ISG20,SLCO3A1 |
| GABBR1 | G-protein coupled receptor | | 2,62E-03 | ATF4,BMP2,CASR,IL6,SPP1,TNF |
| GTF2I | transcription regulator | | 2,62E-03 | CAT,CDKN1A,CDKN1B,FOS,SIRT1,THBS1 |
| RGS1 | enzyme | Activated | 2,62E-03 | CITED2,CXCL2,IFNB1,IL15,IL15RA,INHBA |
| U18666A | chemical reagent | | 2,62E-03 | ABCA1,DDIT3,IL6,NPC1,TNF,XBP1 |
| cobalt | chemical toxicant | | 2,62E-03 | FGF2,ICAM1,IL6,IREB2,POR,TNF |
| miR-4716-5p (miRNAs w/seed CCAUGUU) | mature microRNA | Inhibited | 2,62E-03 | [illegible] |
| miR-1277-5p (miRNAs w/seed AAUAUAU) | mature microRNA | Inhibited | 2,62E-03 | [illegible] |
| miR-6786-3p (miRNAs w/seed GACGCCC) | mature microRNA | Inhibited | 2,62E-03 | [illegible] |
| miR-3684 (miRNAs w/seed UAGACCU) | mature microRNA | Inhibited | 2,63E-03 | [illegible] |
| miR-6652-5p (miRNAs w/seed GGGGACU) | mature microRNA | Inhibited | 2,63E-03 | [illegible] |
| CHD4 | enzyme | | 2,65E-03 | [illegible] |
| phenylbutazone | chemical drug | | 2,66E-03 | ANXA2,C4A/C4B,IDO1,IER3,LXN,PRTFDC1,PSMB8,PTGS2,SPP1,STING1,TMSB10/TMSB4X,TSPAN8,UPP1,VIM |
| miR-6893-3p (miRNAs w/seed CCCUCUC) | mature microRNA | Inhibited | 2,68E-03 | [illegible] |
| miR-652-5p (miRNAs w/seed AACCCUA) | mature microRNA | Inhibited | 2,68E-03 | [illegible] |
| miR-3121-5p (miRNAs w/seed CCUUUGC) | mature microRNA | Inhibited | 2,72E-03 | [illegible] |
| desoxycorticosterone acetate | chemical drug | | 2,72E-03 | CD68,CYBA,EDN1,EGFR,PTGS2,SELE,SPP1,VCAM1 |
| plumbagin | chemical toxicant | | 2,72E-03 | BIRC2,BIRC3,CFLAR,IL6,PRKCE,PTGS2,TNFRSF10A,TNFRSF10B |
| Smad | complex | Activated | 2,72E-03 | ANGPTL4,CDKN1A,CDKN2B,COL1A1,ID3,MCL1,SMAD7,VEGFA |
| NODAL | growth factor | | 2,72E-03 | CCL5,CD40,CDKN1B,CXCL10,FOXO3,IL12A,TNF,XIAP |
| BTRC | enzyme | | 2,72E-03 | BST2,CDKN1A,CHD1,CXCL8,IL1B,IL6,SNAI1,TRAF1 |
| LRPAP1 | other | | 2,72E-03 | CXCL8,DDIT3,IL1B,IL6,LRP1,PTGS2,SORL1,TNF |
| COMMD1 | transporter | | 2,72E-03 | BIRC3,EGLN3,NFKBIA,PGK1,STAT1,STC2,TFRC,VEGFA |
| BTG2 | transcription regulator | | 2,72E-03 | BTG2,CAT,CXCL1,CXCL3,HMOX1,IL1B,RARA,SOD2 |
| miR-606 (miRNAs w/seed AACUACU) | mature microRNA | Inhibited | 2,72E-03 | [illegible] |
| [illegible] | chemical drug | | 2,73E-03 | [illegible] |
| [illegible] | enzyme | | 2,73E-03 | [illegible] |
| [illegible] | transmembrane receptor | Activated | 2,73E-03 | CCL5,CSF1,CSF2,IL1RN,IRF8,SPP1,STAT1,TNF,TXK,XDH |
| amiodarone | chemical drug | | 2,73E-03 | ASAH1,CXCL8,CYP1A1,FADS2,HMOX1,ICAM1,NDRG2,PTGS2,SCD,SOD2,TNF |
| PML-RARA | fusion gene/product | | 2,73E-03 | CDKN1A,EGR1,FOS,HMOX1,JUN,PSMB8,PSMB9,RARA,TNF,VCAM1 |
| miR-6807-5p (miRNAs w/seed UGGGGAU) | mature microRNA | Inhibited | 2,75E-03 | [illegible] |
| miR-3162-3p (miRNAs w/seed CCCUACC) | mature microRNA | Inhibited | 2,76E-03 | [illegible] |
| CD3 group | group | | 2,77E-03 | CBLB,CDKN1B,EGR3,GATA3,GBP2,IL1B,KLF2,ODC1,PLA2G4C,PLA2G6,SCD,TFRC,TNF |
| [illegible] | chemical drug | | 2,77E-03 | [illegible] |
| miR-1285-3p (and other miRNAs w/seed CUGGGCA) | mature microRNA | Inhibited | 2,78E-03 | [illegible] |
| miR-6833-5p (miRNAs w/seed UAUGCCC) | mature microRNA | Inhibited | 2,78E-03 | [illegible] |
| miR-924 (miRNAs w/seed GAGUCUU) | mature microRNA | Inhibited | 2,78E-03 | [illegible] |
| PPARGC1A | transcription regulator | | 2,79E-03 | [illegible] |
| miR-6863-3p (miRNAs w/seed GUUGCAA) | mature microRNA | Inhibited | 2,80E-03 | [illegible] |
| miR-6504 (miRNAs w/seed GUGACAA) | mature microRNA | Inhibited | 2,81E-03 | [illegible] |
| 11,12-epoxyeicosatrienoic acid | chemical - endogenous mammalian | | 2,82E-03 | CDKN1B,DUSP1,EGFR,ICAM1,NFKBIA,SELE,VCAM1 |
| ERVW-1 | other | Activated | 2,82E-03 | CCL5,CDKN1A,CXCL1,CXCL8,IL6,[illegible],XBP1 |
| CDK1 | kinase | | 2,82E-03 | CDKN1A,CDKN1B,[illegible],JUN,MAP2,XBP1,XIAP |
| MPZ | other | | 2,82E-03 | ATF4,DDIT3,ERO1B,FDFT1,JUN,PPP1R15A,SOX4 |
| TRAP | enzyme | | 2,82E-03 | C3,CSF3,IL1A,IL1B,IL6,[illegible],TNF |
| mir-486 | microRNA | Inhibited | 2,82E-03 | ANKRD12,ARID4B,FOXO1,FOXP1,GABRE,PIK3AP1,RFFL |
| [illegible] | enzyme | Activated | 2,82E-03 | APOE,CCL5,CXCL10,IFNB1,IL6,TNF,XAF1 |
| RBP4 | other | | 2,82E-03 | CASP1,IL1B,IL6,MYD88,NLRP3,RARA,TNF |
| RND3 | enzyme | | 2,82E-03 | CDKN1A,CDKN1B,HES1,NOTCH1,PTGS2,VEGFA,VIM |
| NAB2 | transcription regulator | | 2,82E-03 | BCAR1,CDKN1A,FGF2,FOS,PLAU,RRAD,TNFSF10 |
| Usp17la (includes others) | peptidase | Activated | 2,82E-03 | CCL20,CISH,CXCL8,IL1B,IL6,KLF4,TNF |
| zinc sulfate | chemical drug | | 2,82E-03 | C3,HMOX1,IDO1,IL12A,IL1B,IL6,TNF |
| [illegible] | chemical - endogenous mammalian | | 2,82E-03 | APOE,CDKN1A,CFH,ICAM1,IL1B,IL6,VEGFA |
| miR-22-5p (and other miRNAs w/seed GUUCUUC) | mature microRNA | Inhibited | 2,83E-03 | [illegible] |
| mir-19 | microRNA | Inhibited | 2,84E-03 | ABCA1,BCL2L11,CDKN1A,E2F3,FZD4,HIPK3,MYLIP,SUZ12,THBS1,TNF,TNFAIP3 |
| CSF1R | kinase | Activated | 2,84E-03 | CDKN1A,CDKN1B,CSF1,CSF1R,FOS,HMOX1,IL1A,IL6,JUN,JUNB,VCAM1 |
| EPHA4 | kinase | Inhibited | 2,84E-03 | ABCA12,APOBEC3G,DUSP6,FOSL1,FOXA2,IRF1,IRF7,KLK7,NT5E,SLC20A1,SOX9 |
| phenacetin | chemical drug | | 2,84E-03 | ANXA2,BIRC3,ELF3,IER3,PSMB8,RFTN1,SPP1,TMSB10/TMSB4X,TSPAN8,UPP1,VIM |
| Hsp27 | group | | 2,84E-03 | CASP8,CD274,CDKN1A,CSF1,CSF1R,CXCL8,FOS,GCH1,IL6,IL6R,NGFR,PTGS2 |
| OLR1 | transmembrane receptor | | 2,84E-03 | CCL2,CD68,CYBA,HAS2,ICAM1,IL6,ITGAM,NOS3,RARA,SPP1,TNF,VCAM1 |
| herbimycin | chemical - kinase inhibitor | Inhibited | 2,84E-03 | CDKN1B,CXCL8,DUSP1,FOS,ICAM1,IL1B,IL6,JUNB,PLAU,PTGS2,SOD2,TNF |
| EWSR1-FLI1 | fusion gene/product | Inhibited | 2,87E-03 | [illegible] |
| miR-6895-3p (miRNAs w/seed GAUCUCA) | mature microRNA | Inhibited | 2,88E-03 | [illegible] |
| miR-3126-3p (and other miRNAs w/seed CUUUCCU) | mature microRNA | Inhibited | 2,90E-03 | [illegible] |
| miR-383-5p (miRNAs w/seed AGAUCAG) | mature microRNA | Inhibited | 2,91E-03 | [illegible] |
| vitamin E | chemical drug | | 2,92E-03 | CAT,CCL2,CDKN1A,EGR1,IL1A,IL1B,KLF6,NFIL3,NOS3,PTGS2,SPP1,TNF,UCP2,VCAM1,VEGFA |
| GHRL | growth factor | | 2,92E-03 | ABCG1,BDNF,DDIT3,EDN1,FOS,FOXO1,IL1B,IL6,PPARGC1A,PTGS2,SCD,SIRT1,SPP1,TNF,UCP2 |
| miR-6738-3p (miRNAs w/seed UUCUGCC) | mature microRNA | Inhibited | 2,95E-03 | [illegible] |
| miR-1207-5p (and other miRNAs w/seed GGCAGGG) | mature microRNA | Inhibited | 2,96E-03 | [illegible] |
| miR-1185-5p (and other miRNAs w/seed GAGGAUA) | mature microRNA | Inhibited | 2,96E-03 | [illegible] |
| [illegible] | mature microRNA | Inhibited | 2,97E-03 | [illegible] |
| miR-6793-3p (miRNAs w/seed CCCCAAC) | mature microRNA | Inhibited | 2,98E-03 | [illegible] |
| SUZ12 | enzyme | | 2,99E-03 | [illegible] |
| miR-3612 (and other miRNAs w/seed GGAGGCA) | mature microRNA | Inhibited | 3,02E-03 | [illegible] |
| miR-518c-5p (miRNAs w/seed CUCUGGA) | mature microRNA | Inhibited | 3,03E-03 | [illegible] |
| alloxan | chemical reagent | | 3,05E-03 | CCL2,CXCL8,ICAM1,TNF |
| pervanadate | chemical reagent | | 3,05E-03 | IRF1,MX1,NFKBIA,PSMB9 |
| flavokawain B | chemical - endogenous non-mammalian | | 3,05E-03 | BBC3,BCL2L11,TNFRSF10B,XIAP |
| triamcinolone hexacetonide | chemical drug | | 3,05E-03 | ICAM1,IL1A,IL1B,TNF |
| coconut oil | chemical drug | | 3,05E-03 | CXCL3,MAP2,PTGS2,TNF |
| Tlr11 | transmembrane receptor | Activated | 3,05E-03 | CXCL8,IL1B,IL6,TNF |
| USP25 | peptidase | | 3,05E-03 | IFNB1,IL6,TNF,USP25 |
| TRIM29 | transcription regulator | | 3,05E-03 | CDKN1A,PMAIP1,SNAI1,ZEB1 |
| tofacitinib | chemical drug | | 3,05E-03 | IFIT3,IL6,ISG15,TNF |
| [illegible] | chemical drug | Inhibited | 3,05E-03 | CSF2,CXCL8,IL1A,IL6 |
| [illegible] | enzyme | | 3,05E-03 | CAT,FOS,HMOX1,NFE2L2 |
| MEFV | other | | 3,05E-03 | CASP1,CXCL8,IL1B,LYZ |
| AGR2 | other | | 3,05E-03 | AREG,EGFR,EGR1,FOS |
| IFNGR | complex | | 3,05E-03 | COL1A1,IRF1,SOCS3,TNF |
| SERPINA4 | other | | 3,05E-03 | BCL2L11,CCL2,NOS3,VCAM1 |
| [illegible] | other | | 3,05E-03 | IL1B,KLF2,KLF4,SOX9 |
| P2RY1 | G-protein coupled receptor | | 3,05E-03 | FOS,IL6,SELE,VCAM1 |
| PLA2G2A | enzyme | | 3,05E-03 | CD69,CXCL8,IL6,PTGS2 |
| [illegible] | chemical - endogenous non-mammalian | | 3,05E-03 | CFLAR,IL1B,TNFRSF10B,XIAP |
| CCR4 | G-protein coupled receptor | | 3,05E-03 | CCL22,CSF2,CXCL3,FOS |
| USP21 | peptidase | | 3,05E-03 | GATA3,IFNB1,IL6,ISG15 |
| HLA-B | transmembrane receptor | | 3,05E-03 | EDN1,IL6,OAS2,XBP1 |
| GNMT | enzyme | | 3,05E-03 | ABCA1,ABCG1,CYP1A1,NPC1 |
| PILRA | other | | 3,05E-03 | CXCL2,IL1B,IL6,TNF |
| BP-1-102 | chemical reagent | Inhibited | 3,05E-03 | IL1RN,IL6,NFIL3,TNF |
| benzoquinone | chemical - endogenous mammalian | | 3,05E-03 | APOL1,GBP1,IRF1,TNF |
| dimercaptopropanol | chemical drug | | 3,05E-03 | CRYZ,CXCL2,SELE,TNF |
| 4-coumaric acid | chemical - endogenous mammalian | | 3,05E-03 | CDKN1A,FGF2,ODC1,PTGS2 |
| fluphenazine | chemical drug | | 3,05E-03 | FOS,NR4A1,NR4A2,NR4A3 |
| 2-mercaptoethanol | chemical - endogenous mammalian | | 3,05E-03 | ATF3,HERPUD1,HMOX1,IDO1 |
| gliotoxin | chemical toxicant | | 3,05E-03 | BIRC3,CCL20,IER3,TNF |
| RWJ 67657 | chemical - kinase inhibitor | | 3,05E-03 | CXCL8,IL1B,PTGS2,TNF |
| ISRIB | chemical reagent | Inhibited | 3,05E-03 | ATF4,CCL20,IL6,PMAIP1 |
| diacerein | chemical drug | | 3,05E-03 | IL1B,IL6,IL6R,TNF |
| eugenol | chemical - endogenous non-mammalian | | 3,05E-03 | CDKN1A,CXCL8,IL6,PTGS2 |
| 12-hydroxyeicosatetraenoic acid | chemical - endogenous mammalian | | 3,05E-03 | ICAM1,PTGS2,VCAM1,VEGFA |
| Muscarinic cholinergic receptor | group | | 3,05E-03 | CCN1,FMR1,FOS,JUN |
| miR-7153-3p (miRNAs w/seed ACCAUGG) | mature microRNA | Inhibited | 3,06E-03 | [illegible] |
| miR-381-3p (and other miRNAs w/seed AUACAAG) | mature microRNA | Inhibited | 3,10E-03 | [illegible] |
| miR-328-3p (and other miRNAs w/seed UGGCCCU) | mature microRNA | Inhibited | 3,11E-03 | [illegible] |
| STUB1 | enzyme | | 3,12E-03 | CDKN1A,CTSS,FOXO1,GATA4,IFNB1,IL6,JUNB,NFKB1,NFKB2,NOS3,RBL2,RIPK1,SNCA,VEGFA |
| miR-6867-3p (miRNAs w/seed CCCUCCU) | mature microRNA | Inhibited | 3,13E-03 | [illegible] |
| nitroglycerin | chemical drug | | 3,14E-03 | CALCA,CXCL8,HMOX1,IL6,PLAUR |
| ZFAS1 | other | | 3,14E-03 | KLF2,NKD2,VIM,ZEB1,ZEB2 |
| T 0070907 | chemical reagent | | 3,14E-03 | CDKN1A,COL1A1,HMOX1,PTGS2,TNF |
| ABCA4 | transporter | | 3,14E-03 | C3,CFB,CXCL10,IFNB1,TLR3 |
| PPP1R15B | phosphatase | Inhibited | 3,14E-03 | ATF4,CHAC1,DDIT3,PPP1R15A,SESN2 |
| FFAR1 | G-protein coupled receptor | | 3,14E-03 | CYP1A1,FOXA2,IL1B,IRS2,PPARGC1A |
| SARM1 | transmembrane receptor | | 3,14E-03 | CCL5,IFNB1,IL6,TNF,XAF1 |
| ruxolitinib | chemical drug | | 3,14E-03 | CD274,COL1A1,IFNL1,JAK2,MCL1 |
| [illegible] | chemical reagent | | 3,14E-03 | BBC3,EGFR,NFKBIA,VIM,ZEB1 |
| GPR132 | G-protein coupled receptor | | 3,14E-03 | CSF2,EGR1,ICAM1,IL6,SELE |
| DRD1 | G-protein coupled receptor | | 3,14E-03 | BDNF,EGR1,FOS,FOSB,JUNB |
| APOH | transporter | | 3,14E-03 | CD40,CD83,NFKB1,SELE,TNF |
| CEBPG | transcription regulator | | 3,14E-03 | ADH1C,CXCL8,DDIT3,IDH1,IL6 |
| VLDLR | transporter | | 3,14E-03 | ABCA1,FOS,IL6,TNF,TNFAIP3 |
| IL18BP | other | | 3,14E-03 | CIITA,CXCL8,IL6,TNF,VEGFA |
| Ifna (includes others) | cytokine | Activated | 3,14E-03 | CD40,CD83,DAXX,IRF1,TLR3 |
| 2-aminoethoxydiphenylborane | chemical reagent | Inhibited | 3,14E-03 | CYP1A1,FGF2,PTGS2,SERPINB2,VEGFA |
| clioquinol | chemical drug | Activated | 3,14E-03 | CDKN1A,CDKN1B,FOS,JUN,KDM5B |
| manumycin A | chemical reagent | | 3,14E-03 | CXCL8,IL1B,PTGS2,TNF,VEGFA |
| rosmarinic acid | chemical - endogenous non-mammalian | | 3,14E-03 | BDNF,CXCL8,IL6,PTGS2,TNF |
| Z-DEVD-FMK | chemical - protease inhibitor | | 3,14E-03 | CXCL2,CXCL3,MCL1,PDZD2,SPHK1 |
| theophylline | chemical drug | | 3,14E-03 | CXCL8,FOS,IL6,LRP1,THBS1 |
| allopregnanolone | chemical - endogenous mammalian | | 3,14E-03 | FOS,GRIN2A,IL6,PTGS2,TNF |
| canrenoate potassium | chemical drug | | 3,14E-03 | CDKN1A,EDN1,PTGS2,SIRT1,SPP1 |
| miR-617 (miRNAs w/seed GACUUCC) | mature microRNA | Inhibited | 3,15E-03 | [illegible] |
| caspase | group | | 3,17E-03 | CDKN1A,CDKN1B,EGFR,HERPUD1,IFNB1,IL1A,MCL1,THBS1,TNF |
| MAPK11 | kinase | Activated | 3,17E-03 | FAS,FOS,HMOX1,IL6,PPARGC1A,PTGS2,SLC9A5,UBR2,VDR |
| VAV1 | transcription regulator | | 3,17E-03 | CD69,CDKN1B,FOS,IL6,JUN,PTGS2,REL,TNF,VEGFA |
| STS | enzyme | | 3,17E-03 | CD69,CEBPD,HSPA1A/HSPA1B,IGFBP2,IL1B,IL6,TNF,UCP2,XBP1 |
| NUP98-HOXA9 | fusion gene/product | | 3,17E-03 | EYA1,HOXB5,HOXB6,MARCHF7,NR4A2,PTGS2,SORBS2,TLE4,VANGL2 |
| miR-6734-5p (miRNAs w/seed UGAGGGG) | mature microRNA | Inhibited | 3,19E-03 | [illegible] |
| miR-6515-3p (miRNAs w/seed CUCUUCA) | mature microRNA | Inhibited | 3,20E-03 | [illegible] |
| miR-6810-5p (miRNAs w/seed UGGGGAC) | mature microRNA | Inhibited | 3,22E-03 | [illegible] |
| miR-17-2-3p (and other miRNAs w/seed CUGCACU) | mature microRNA | Inhibited | 3,23E-03 | [illegible] |
| ZEB1 | transcription regulator | | 3,26E-03 | [illegible] |
| sodium chloride | chemical - endogenous mammalian | | 3,26E-03 | [illegible] |
| Ccl2 | cytokine | Activated | 3,29E-03 | CCL5,CSF1,CTSV,CXCL10,CXCL2,CXCL3,IL1B,IL6,KLF4,SDC4,[illegible],TNF,VCAM1 |
| miR-3154 (miRNAs w/seed AGAAGGG) | mature microRNA | Inhibited | 3,30E-03 | [illegible] |
| MITF | transcription regulator | | 3,37E-03 | [illegible] |
| NPM1 | transcription regulator | | 3,40E-03 | CCL2,CSF1,CXCL2,CXCL3,HOXB5,IL6,IL6ST,SOD2,TNF,VEGFA |
| RNASE2 | enzyme | Activated | 3,40E-03 | CCL2,CCL22,CCL5,CD83,CSF1,CXCL10,CXCL8,IL6,IL7,TNF |
| PLP1 | other | | 3,40E-03 | ATF3,ATF4,CCL2,CCL20,CDKN1B,CXCL2,DDIT3,NOTCH1,PPP1R15A,TNF |
| FOSB | transcription regulator | | 3,40E-03 | BDNF,CCR4,CDK5,CEBPB,FOS,FOSB,FOSL1,INHBA,MT2A,SERPINB2 |
| ELF4 | transcription regulator | | 3,40E-03 | CDKN1A,CXCL2,CXCL8,DUSP1,DUSP6,KLF2,KLF4,LYZ,RCAN1,SPP1 |
| IL2RA | transmembrane receptor | | 3,40E-03 | BCL6,CD69,CXCL10,FAS,IL1B,IL6,LTB,SOCS3,STAT1,TNF |
| doxycycline | chemical drug | Activated | 3,40E-03 | [illegible] |
| LTBR | transmembrane receptor | | 3,41E-03 | CCL20,CSF2,ICAM1,IFNB1,IL6,LYZ,MFGE8,NFKB2,NFKBIA,TNF,TNFSF13B,VCAM1 |
| monocrotaline | chemical toxicant | | 3,41E-03 | BCL6,CDKN1B,GADD45A,HMOX1,ID3,IL1B,IL6,NOS3,PDGFA,SIRT1,SPP1,TNF |
| PSMB11 | peptidase | Inhibited | 3,44E-03 | [illegible] |
| miR-6801-3p (and other miRNAs w/seed CCCUGGC) | mature microRNA | Inhibited | 3,44E-03 | [illegible] |
| miR-6764-3p (and other miRNAs w/seed CUCUGGU) | mature microRNA | Inhibited | 3,46E-03 | [illegible] |
| Laminin (complex) | complex | | 3,46E-03 | BIRC3,CCL2,COL1A1,EGFR,ETS2,IGF2,ODAM,PLAU,PTGS2,SDC4,VIM |
| ABCB6 | transporter | | 3,46E-03 | [illegible] |
| EPHA3 | kinase | Inhibited | 3,46E-03 | ABCA12,APOBEC3G,DUSP6,FOSL1,FOXA2,IRF1,IRF7,KLK7,NT5E,SLC20A1,SOX9 |
| beta-carotene | chemical - endogenous mammalian | | 3,46E-03 | [illegible] |
| Esrra | transcription regulator | | 3,46E-03 | [illegible] |
| miR-6882-3p (miRNAs w/seed GCUGCCU) | mature microRNA | Inhibited | 3,46E-03 | [illegible] |
| MKNK1 | kinase | Activated | 3,50E-03 | [illegible] |
| EHMT1 | transcription regulator | | 3,50E-03 | [illegible] |
| MRTFA | transcription regulator | Activated | 3,50E-03 | [illegible] |
| [illegible] (miRNAs w/seed GCCCCAA) | mature microRNA | Inhibited | 3,50E-03 | [illegible] |
| miR-1278 (miRNAs w/seed AGUACUG) | mature microRNA | Inhibited | 3,54E-03 | [illegible] |
| [illegible] | mature microRNA | Inhibited | 3,56E-03 | [illegible] |
| miR-621 (miRNAs w/seed GCUAGCA) | mature microRNA | Inhibited | 3,56E-03 | [illegible] |
| SIRT2 | transcription regulator | | 3,58E-03 | CDKN1B,CXCL10,DHCR7,FDFT1,IFIT3,ISG15,NEDD4,PMVK |
| Trp53cor1 | other | Activated | 3,58E-03 | BBC3,CDKN1A,FOXA2,IL1B,IL6,MCL1,NPTX1,SSTR2 |
| MTA3 | transcription regulator | | 3,58E-03 | [illegible] |
| SUPT16H | transcription regulator | | 3,58E-03 | [illegible] |
| K-252 | chemical drug | | 3,58E-03 | BDNF,CXCL8,ITGAM,JUN,NDRG1,SOD2,SPP1,VEGFA |
| [illegible] | mature microRNA | Inhibited | 3,60E-03 | [illegible] |
| [illegible] | mature microRNA | Inhibited | 3,60E-03 | [illegible] |
| [illegible] | mature microRNA | Inhibited | 3,62E-03 | [illegible] |
| [illegible] | mature microRNA | Inhibited | 3,62E-03 | [illegible] |
| [illegible] | mature microRNA | Inhibited | 3,63E-03 | [illegible] |
| miR-1276 (miRNAs w/seed AAAGAGC) | mature microRNA | Inhibited | 3,63E-03 | [illegible] |
| miR-548g-3p (miRNAs w/seed AAACUGU) | mature microRNA | Inhibited | 3,65E-03 | [illegible] |
| CSHL1 | growth factor | | 3,65E-03 | BCL6,BMP2,CISH,CYP2B6,CYP3A5,FOS,FOXA2,HNF4A,IRF1,PIK3R1,SLC22A1,SOCS2,SOCS3,SSTR2 |
| [illegible] | mature microRNA | Inhibited | 3,67E-03 | [illegible] |
| miR-3064-3p (miRNAs w/seed UGCCACA) | mature microRNA | Inhibited | 3,68E-03 | [illegible] |
| [illegible] | mature microRNA | Inhibited | 3,70E-03 | [illegible] |
| [illegible] | mature microRNA | Inhibited | 3,71E-03 | [illegible] |
| [illegible] | mature microRNA | Inhibited | 3,72E-03 | [illegible] |
| [illegible] | mature microRNA | Inhibited | 3,76E-03 | [illegible] |
| ascorbic acid | chemical - endogenous mammalian | | 3,76E-03 | [illegible] |
| miR-218-5p (and other miRNAs w/seed UGUGCUU) | mature microRNA | Inhibited | 3,77E-03 | [illegible] |
| [illegible] | mature microRNA | Inhibited | 3,80E-03 | [illegible] |
| [illegible] | mature microRNA | Inhibited | 3,80E-03 | [illegible] |
| [illegible] | mature microRNA | Inhibited | 3,80E-03 | [illegible] |
| fluoride | chemical - endogenous mammalian | Activated | 3,83E-03 | DDIT3,FAS,FOS,GADD45A,VCAM1,XBP1 |
| CSF2RB | transmembrane receptor | Activated | 3,83E-03 | FOS,IL6,ITGAM,JUN,JUNB,PIM1 |
| resatorvid | chemical drug | Inhibited | 3,83E-03 | CAT,CCL2,CXCL8,IL1B,IL6,TNF |
| PMP22 | other | | 3,83E-03 | ABCA1,APOE,JUN,NGFR,NOTCH1,PRX |
| TNFRSF25 | transmembrane receptor | | 3,83E-03 | CCL2,CSF3,CXCL8,IL6,SELE,TNF |
| TNFRSF12A | transmembrane receptor | | 3,83E-03 | CCL2,CCL5,CXCL10,CXCL8,IL6,PPARGC1A |
| S1PR3 | G-protein coupled receptor | | 3,83E-03 | FOS,ICAM1,IL1B,JUN,PTGS2,VCAM1 |
| CH-223191 | chemical reagent | | 3,83E-03 | AHR,COL1A1,CSF2,CYP1A1,NT5E,PTGS2 |
| farnesyl pyrophosphate | chemical - endogenous mammalian | | 3,83E-03 | ABCA1,ICAM1,LIPG,NOS3,PTGS2,VCAM1 |
| [illegible] | mature microRNA | Inhibited | 3,83E-03 | [illegible] |
| [illegible] | mature microRNA | Inhibited | 3,85E-03 | [illegible] |
| [illegible] | mature microRNA | Inhibited | 3,85E-03 | [illegible] |
| geraniol | chemical - endogenous non-mammalian | | 3,86E-03 | [illegible] |
| Aldose Reductase | group | Activated | 3,86E-03 | CSF2,CXCL8,ICAM1,IL1B,IL6,ITGAM,TNF |

| | | | | |
|---|---|---|---|---|
| SIGIRR | transmembrane receptor | Inhibited | 3.86E-03 | CXCL2,CXCL3,IL1A,IL1B,IL6,NFKB1,TNF |
| CpG ODN 2395 | chemical reagent | Activated | 3.86E-03 | BCL6,CD40,HMOX1,ICHE,IL1B,IL6,TNF |
| PARG | enzyme | | 3.86E-03 | GDF15,IL1B,LGALS3BP,NFATS,PTGS2,SOCS2,TNF |
| HOXA11 | transcription regulator | | 3.86E-03 | CDKN1B,COL1A1,CTSV,EGFR,KLF2,PDGFA,SPP1 |
| trametinib | chemical drug | | 3.86E-03 | CD274,IFIT1,ISG15,MX1,TNF,TNFAIP3,TNFSF10 |
| MED12 | transcription regulator | | 3.86E-03 | ANKRD1,CREB5,EGR2,EREG,GDF15,LIF,SLC16A6 |
| GDF15 | growth factor | | 3.86E-03 | CDKN1A,GDF15,IL6,PLAU,PLAUR,RND3,TNF |
| TRIM21 | enzyme | Inhibited | 3.86E-03 | CXCL10,IFNB1,IL1B,IL6,IRF7,TNF,TRIM25 |
| acetaldehyde | chemical - endogenous mammalian | | 3.86E-03 | CEBPB,COL1A1,FOS,IL6,JUN,SIRT1,TNF |
| retinol | chemical drug | | 3.86E-03 | CSF2,CXCL8,IL6,JUN,RARA,SAA2,SLC5A5 |
| eicosanoid7634 | chemical - endogenous mammalian | | 3.86E-03 | APOE,CDKN1A,CFH,ICAM1,IL1B,IL6,VEGFA |
| butaprost | chemical drug | | 3.86E-03 | IL1B,IL6,PTGER2,PTGS2,SIRT1,SOCS3,TNF |
| 5-hydroxytryptamine | chemical - endogenous mammalian | Activated | 3.88E-03 | ARC,BDNF,CCL2,CXCL8,CYP1B1,EGR1,FGF2,FOS,HBEGF,ICAM1,IL1A,IL1B,IL6,NOS3,NR3C1,NR4A1,PTGS2,SERPINB2,THBS1,TNF,UCP2,VCAM1 |
| SIRT3 | enzyme | | 3.88E-03 | CAT,DIO2,IL1B,IL6,PPARGC1A,RICTOR,RPTOR,SCD,SOD2,TFRC,TNF,VEGFA,VIM |
| miR-3174 (miRNAs w/seed AGUGAGU) | mature microRNA | Inhibited | 3.90E-03 | [illegible] |
| miR-448-3p (and other miRNAs w/seed UGCAUAU) | mature microRNA | Inhibited | 3.92E-03 | [illegible] |
| SOD2 | enzyme | | 3.93E-03 | ATP1A1,CAT,CDKN1A,DDIT3,GCH1,HMOX1,IL1B,IL6,IL7,MAP2,MMP7,NCALD,NOS3,ODC1,PTGS2,SLC19A2,SOD2,TNF,TRIM14,UCP2 |
| MSTN | growth factor | | 3.93E-03 | AKIRIN1,BMP2,CDKN1A,CTSV,CXCL1,FOS,IGF2,IL6,INHBA,KLF10,MEF2A,PPARGC1A,SPP1,VEGFA,VIM |
| MYRF | transcription regulator | | 3.93E-03 | AMOTL2,APBZ1,BCL2L11,CEACAM1,CFI,GDF15,JUNB,OTUD1,PARP10,PLAUR,PWWP3B,RASSF6,SDSL,TENT5A,UAP1L1 |
| fenamic acid | chemical reagent | | 3.93E-03 | [illegible] |
| miR-150-5p (and other miRNAs w/seed CUCCCAA) | mature microRNA | Inhibited | 3.96E-03 | [illegible] |
| miR-376-5p (and other miRNAs w/seed GGUAGAC) | mature microRNA | Inhibited | 3.97E-03 | [illegible] |
| miR-891a (miRNAs w/seed GCAACUU) | mature microRNA | Inhibited | 4.01E-03 | [illegible] |
| mir-193 | microRNA | Inhibited | 4.01E-03 | CCL2,CD2AP,MCL1,NOTCH1,PIK3R3,PLAU,STMN1,SYNPO,VEGFA |
| PRKCZ | kinase | Activated | 4.01E-03 | ABCA1,CXCL8,HMOX1,JUN,NFKBIA,NOS3,PHGDH,VCAM1,VEGFA |
| NOSTRIN | transcription regulator | Inhibited | 4.01E-03 | CCL5,IL6,ITGA5,NFKB1,NOS3,PGF,PLAU,SELE,THBS1 |
| BECN1 | other | Activated | 4.01E-03 | BCL2L11,BMF,CCL5,IFNB1,IL1B,IL6,SOD2,SPP1,TNF |
| linsidomine | chemical drug | | 4.01E-03 | ANXA2,EGR1,FMR1,HMOX1,NR4A1,PDGFA,PTGS2,TNF,VEGFA |
| KN-93 | chemical - kinase inhibitor | Inhibited | 4.01E-03 | ABCA1,BIRC3,CD83,CFLAR,FOS,HAS2,MCL1,NR4A1,NR4A2 |
| BMP7 | growth factor | | 4.02E-03 | ACVR1,ADORA1,BMP2,CCL2,CDKN1A,CEBPB,CEBPD,COL1A1,CXCL8,EDN1,EGFR,EREG,ICAM1,IGF2,IL1B,IL1RN,IL6,ITGA6,KLF4,NDRG1,PIK3R1,PPARGC1A,RNF111,SMAD7,SOCS3,SOSTDC1,SPP1,VEGFA,VIM,ZEB2 |
| 3-O-glucosyl-1,2-diacylglycerol | chemical - endogenous non-mammalian | | 4.04E-03 | CXCL3,IL6,TNF |
| behenic acid | chemical - endogenous mammalian | | 4.04E-03 | IL1B,IL6,TNF |
| chitinase | group | | 4.04E-03 | CCL2,CCL5,CXCL8 |
| tylophorine | chemical drug | | 4.04E-03 | JUN,PTGS2,TNF |
| CACTIN | other | | 4.04E-03 | CCL5,CXCL8,TNF |
| NPSR1 | G-protein coupled receptor | | 4.04E-03 | GADD45A,NFKB1,NR4A1 |
| PIEZO1 | ion channel | | 4.04E-03 | CD69,IL6,VCAM1 |
| ROBO3 | transmembrane receptor | | 4.04E-03 | IFNB1,IL6,IRF8 |
| ATF6B | transcription regulator | | 4.04E-03 | DDIT3,RCAN1,XBP1 |
| TNIP3 | other | | 4.04E-03 | CXCL8,IL6,TNF |
| USP28 | peptidase | | 4.04E-03 | CDKN1A,GATA4,PLK3 |
| FOX2 | transporter | | 4.04E-03 | ACO1,IREB2,TFRC |
| ATP6V0D2 | transporter | | 4.04E-03 | IFNB1,IL6,TNF |
| ABRAXAS2 | other | | 4.04E-03 | CASP1,IL1B,JAK2 |
| B3GNT2 | enzyme | | 4.04E-03 | IL1B,IL6,TNF |
| ZFP64 | transcription regulator | | 4.04E-03 | IFNB1,IL6,TNF |
| CYP2C8 | enzyme | | 4.04E-03 | IL1B,IL6,SELE |
| CIRBP | translation regulator | | 4.04E-03 | CXCL2,CXCL3,IL6 |
| RNF123 | enzyme | | 4.04E-03 | IFNB1,IL6,TNF |
| LRRC19 | other | | 4.04E-03 | CXCL8,IL6,TNF |
| CDCA3 | other | | 4.04E-03 | CDKN1A,CDKN1B,CDKN2B |
| P-TEFb | complex | | 4.04E-03 | BBC3,CDKN1A,CXCL8 |
| proanthocyanidin derivative | chemical - other | | 4.04E-03 | CYP1A1,JUN,MFGE8 |
| NCF2 | enzyme | | 4.04E-03 | SELE,SOD2,VCAM1 |
| cerotic acid | chemical - endogenous mammalian | | 4.04E-03 | CD40,IL1A,IL1B |
| PF-4981502 | chemical drug | | 4.04E-03 | BBC3,PMAIP1,VIM |
| GRPR | G-protein coupled receptor | | 4.04E-03 | BDNF,CDKN1A,CDKN1B |
| mir-590 | microRNA | | 4.04E-03 | IL6,IL6R,NFKB1 |
| CALCR | G-protein coupled receptor | | 4.04E-03 | CDKN1A,CDKN1B,FOS |
| OPAM1 | other | | 4.04E-03 | IL1RN,IL6,TNF |
| GCLC | enzyme | | 4.04E-03 | FOS,JUN,JUNB |
| MEP1A | peptidase | | 4.04E-03 | CXCL8,IL1B,IL6 |
| lanatoside C | chemical - endogenous non-mammalian | | 4.04E-03 | NUAK1,TNFRSF10A,TNFRSF10B |
| HSPB6 | other | | 4.04E-03 | CCL2,CXCL8,ICAM1 |
| BAG2 | other | | 4.04E-03 | IL1B,IL6,TNF |
| IL17B | cytokine | | 4.04E-03 | CDKN1A,CSF3,IL6 |
| A4GALT | enzyme | | 4.04E-03 | IL6,IRF1,TNF |
| APOD | transporter | | 4.04E-03 | IL1B,IL6,TNF |
| TMSB10/TMSB4X | other | | 4.04E-03 | FAS,MMP7,TMSB10/TMSB4X |
| NLRC3 | other | | 4.04E-03 | IL1B,IL6,TNF |
| Rps6ka6 | kinase | | 4.04E-03 | IL1B,IL6,TNF |

[illegible]

| | | | | |
|---|---|---|---|---|
| KT5720 | chemical - kinase inhibitor | | 7,33E-03 | ADORA2A,CDKN1B,HMOX1,NFKBIA,OXTR |
| KMT2A-AFF1 | fusion gene/product | | 7,33E-03 | BCL6,FUT4,ITGAE,MEIS1,SLC43A2 |
| ethylenediaminetetraacetic acid | chemical drug | | 7,33E-03 | CXCL8,EGR1,IL1B,NOS3,TNF |
| icatibant | biologic drug | | 7,33E-03 | BDKRB2,ICAM1,NOS3,NOTCH1,PTGS2 |
| poly-L-lysine | chemical reagent | Activated | 7,33E-03 | CCL2,CXCL8,IL1RN,IL6,ITGAM |
| adefovir dipivoxil | chemical drug | | 7,33E-03 | CEBPB,COL1A1,CREBBP,IL6,TNF |
| cyclomaltodextrin | chemical drug | Inhibited | 7,33E-03 | CSF3,CXCL3,IL1B,IL6,TNF |
| miR-4730 (miRNAs w/seed UGGCGGA) | mature microRNA | Inhibited | 7,36E-03 | [illegible] |
| miR-6866-5p (miRNAs w/seed UACAUGU) | mature microRNA | Inhibited | 7,41E-03 | [illegible] |
| GFI1B | transcription regulator | | 7,41E-03 | CDKN1A,FOXO1,GATA3,OGA,SOCS1,SOCS3 |
| batimastat | chemical - protease inhibitor | | 7,41E-03 | CASP1,CEBPB,CXCL3,FGF2,SDC4,VEGFA |
| pimozide | chemical drug | | 7,41E-03 | [illegible],CSF1R,EREG,IRF8,MCL1,PIM1 |
| pyruvaldehyde | chemical - endogenous mammalian | | 7,41E-03 | ARG2,HBEGF,HMOX1,ICAM1,NOS3,SELE |
| lisinopril | biologic drug | Inhibited | 7,41E-03 | FOS,IL1B,JUN,PDGFA,TNF,VEGFA |
| TGAL copolymer | biologic drug | | 7,41E-03 | BDNF,CCL5,CXCL8,IL1B,IL1RN,TNF |
| irinotecan | chemical drug | Activated | 7,41E-03 | FAS,IL1B,IL6,PMAIP1,SNAI1,TNF |
| crizotinib | chemical drug | | 7,44E-03 | AREG,CD274,EGFR,PTP4A1,ROS1,SNAI1,VIM,ZEB1 |
| NFIL3 | transcription regulator | Inhibited | 7,44E-03 | CDKN1A,FAS,GADD45A,GADD45B,PTGS2,SCD,TNF,TNFSF10 |
| PHB | transcription regulator | | 7,44E-03 | CXCL8,IGF2,IL1B,JUN,KPNA4,NFKBIA,PDGFA,TNF |
| adenosine | chemical - endogenous non-mammalian | | 7,44E-03 | FOS,HMOX1,IL1B,IL6,MAP2,SMAD7,SNCA,TNF |
| deoxynivalenol | chemical toxicant | Activated | 7,44E-03 | CXCL8,ERN1,ICAM1,IL1B,IL6,TJP1,TNF,XBP1 |
| carbenoxolone | chemical toxicant | Inhibited | 7,44E-03 | BCL2L11,COL1A1,CYBA,DSPP,IL6,NOS3,PTGS2,SPP1 |
| miR-208a-5p (and other miRNAs w/seed AGCUUUU) | mature microRNA | Inhibited | 7,46E-03 | [illegible] |
| NFYB | transcription regulator | | 7,47E-03 | [illegible] |
| TNIP1 | other | Inhibited | 7,47E-03 | CCL20,CCR1,CXCL2,CXCL3,HLA-A,HLA-B,IFNB1,IL1B,RELB,TNF |
| isoprenaline | chemical drug | | 7,47E-03 | FOS,IER5,KBTBD8,KLF5,NR4A1,NR4A2,NR4A3,PER1,TNF,ZFYVE26 |
| propranolol | chemical drug | Inhibited | 7,47E-03 | CYP1A1,DIO2,DUSP1,FOS,ICAM1,IL6,NFKBIA,PPARGC1A,PTGS2,VEGFA |
| miR-6436b-3p (and other miRNAs w/seed AGGGCAG) | mature microRNA | Inhibited | 7,48E-03 | [illegible] |
| miR-376a-3p (and other miRNAs w/seed ACAUAGA) | mature microRNA | Inhibited | 7,52E-03 | [illegible] |
| ARNT2 | transcription regulator | Activated | 7,54E-03 | [illegible] |
| miR-152-5p (miRNAs w/seed GGUUCUC) | mature microRNA | Inhibited | 7,54E-03 | [illegible] |
| HAND2 | transcription regulator | | 7,56E-03 | ACE2,ACTN2,CCL2,COL1A1,CXCL2,FAP,HAND1,IL11,IL6,NFATC2,PTGS2,SIK1/SIK1B,TNNC1,TPM1 |
| TBP | transcription regulator | | 7,56E-03 | BDP1,CDKN1A,COL1A1,ETS2,FOS,GATA4,HLA-A,IER3,IGF2,NFKBIA,PTGS2,TNF,TNFAIP3,XDH |
| miR-6835-5p (and other miRNAs w/seed GGGGGUA) | mature microRNA | Inhibited | 7,59E-03 | [illegible] |
| TERC | other | | 7,61E-03 | CASP1,CCL5,CDKN1A,CDKN1B,CRY1,EGR1,H1-0,NR1D1,THRA |
| sildenafil | chemical drug | | 7,61E-03 | BDNF,CEBPB,CFLAR,HMOX1,NOS3,PTGS2,TNF,VEGFA,VEGFC |
| choline | chemical - endogenous mammalian | Inhibited | 7,61E-03 | BBC3,CDKN1A,DDIT3,ERRFI1,HMOX1,IGF2,OXTR,PTGS2,SPP1 |
| Erk5 | kinase | Activated | 7,76E-03 | CFB,COL1A1,COL4A1,IL1A,IL6,KLF4,LOX,PDGFA,PLAU,SELE,SPTA1,SRSF6,TNFSF10,TNFSF15,ZNF296 |
| miR-591-5p (and other miRNAs w/seed GUGGGGA) | mature microRNA | Inhibited | 7,81E-03 | [illegible] |
| miR-6874-5p (and other miRNAs w/seed UGGAGCU) | mature microRNA | Inhibited | 7,81E-03 | [illegible] |
| FGF8 | growth factor | | 7,82E-03 | [illegible] |
| NLRP3 | other | | 7,82E-03 | [illegible] |
| CALCA | other | | 7,82E-03 | AHNAK,AKAP13,CALCA,CARD14,CCL2,CXCL8,CYBA,EDN1,FOS,GNG12,IL6,JUNB,MME,NDRG2,ODC1,PHLDB2,SLC43A2,TNF |
| miR-3678-5p (miRNAs w/seed CCGUACA) | mature microRNA | Inhibited | 7,83E-03 | [illegible] |
| CEBPE | transcription regulator | | 7,84E-03 | CDKN1B,CEBPB,CSF1R,CTSV,IL12A,IL1RN,IL6,ITGAM,LYZ,PTGS2,SERPINB2,TNF |
| NKX3-1 | transcription regulator | | 7,84E-03 | BTG2,CASP7,CTSV,ETV3,FOS,FOXO3,PIK3R1,PIM1,SDC1,SDC4,SOX4,VAV3 |
| MMP1 | peptidase | Activated | 7,84E-03 | CDKN1A,CXCL8,EGR1,FOS,IFNB1,JUN,NFKB1,NFKBIA,PDGFA,PLAU,PTGS2,TNF |
| NEDD9 | other | | 7,88E-03 | [illegible] |
| miR-154-3p (and other miRNAs w/seed AUCAUAC) | mature microRNA | Inhibited | 7,94E-03 | [illegible] |
| miR-187-5p (miRNAs w/seed GCUACAA) | mature microRNA | Inhibited | 7,94E-03 | [illegible] |
| miR-301a-5p (and other miRNAs w/seed CUCUGAC) | mature microRNA | Inhibited | 7,94E-03 | [illegible] |
| miR-6802-3p (miRNAs w/seed UCACCCC) | mature microRNA | Inhibited | 8,09E-03 | [illegible] |
| miR-4482 (miRNAs w/seed UGAAUUC) | mature microRNA | Inhibited | 8,09E-03 | [illegible] |
| miR-5088-5p (miRNAs w/seed AGGGCUC) | mature microRNA | Inhibited | 8,11E-03 | [illegible] |
| miR-592 (and other miRNAs w/seed UGUGUCA) | mature microRNA | Inhibited | 8,17E-03 | [illegible] |
| miR-4451 (miRNAs w/seed GGUAGAG) | mature microRNA | Inhibited | 8,18E-03 | [illegible] |
| miR-6878-3p (and other miRNAs w/seed UGGCCUC) | mature microRNA | Inhibited | 8,19E-03 | [illegible] |
| miR-541-3p (and other miRNAs w/seed GGUGGGC) | mature microRNA | Inhibited | 8,20E-03 | [illegible] |
| thyroid hormone | chemical - endogenous mammalian | | 8,27E-03 | [illegible] |
| miR-342-3p (and other miRNAs w/seed CUCCCAU) | mature microRNA | Inhibited | 8,27E-03 | [illegible] |
| miR-4445-3p (and other miRNAs w/seed ACGGCAA) | mature microRNA | Inhibited | 8,28E-03 | [illegible] |
| miR-31-3p (and other miRNAs w/seed GCUAUGC) | mature microRNA | Inhibited | 8,29E-03 | [illegible] |
| miR-409-5p (and other miRNAs w/seed GGUUACC) | mature microRNA | Inhibited | 8,30E-03 | [illegible] |
| AR | ligand-dependent nuclear receptor | | 8,37E-03 | [illegible] |
| miR-6857-3p (miRNAs w/seed GACUGAG) | mature microRNA | Inhibited | 8,37E-03 | [illegible] |
| miR-3156-3p (miRNAs w/seed UCCCACU) | mature microRNA | Inhibited | 8,37E-03 | [illegible] |
| miR-5008-3p (and other miRNAs w/seed CUGUGCU) | mature microRNA | Inhibited | 8,37E-03 | [illegible] |
| miR-671-3p (and other miRNAs w/seed CCGGUUC) | mature microRNA | Inhibited | 8,39E-03 | [illegible] |
| MEN1 | transcription regulator | | 8,43E-03 | CASP8,CCNB2,CDKN1A,CDKN1B,CDKN2B,FOS,IGFBP2,IRS2,MAFA,MEIS1,PTPRZ1 |
| NUP98-DDX10 | fusion gene/product | | 8,43E-03 | EGR1,GADD45B,HOXB6,LYZ,MCL1,MEIS1,NDRG2,PTGS2,SOX4,STYK1,ZFHX3 |
| miR-650a-5p (and other miRNAs w/seed UUUGCGA) | mature microRNA | Inhibited | 8,45E-03 | [illegible] |
| miR-181a-1-3p (and other miRNAs w/seed CCAUCGA) | mature microRNA | Inhibited | 8,45E-03 | [illegible] |
| miR-3925-3p (miRNAs w/seed CUCCAGU) | mature microRNA | Inhibited | 8,47E-03 | [illegible] |
| miR-660 (miRNAs w/seed UACACAU) | mature microRNA | Inhibited | 8,50E-03 | [illegible] |
| miR-483-3p (miRNAs w/seed CACUCCU) | mature microRNA | Inhibited | 8,54E-03 | [illegible] |
| KCNE3 | ion channel | | 8,63E-03 | [illegible] |
| risperidone | chemical drug | | 8,73E-03 | CXCL10,FADS2,FOS,NGFR,NR4A1,NR4A2,NR4A3 |
| shikonin | chemical drug | | 8,73E-03 | ABCA1,ABCG1,CAT,CCL5,CXCL8,IL1B,TNF |
| IPMK | kinase | Activated | 8,73E-03 | CCN1,FOS,FOSB,JUN,JUNB,PER1,TNF |
| UHRF2 | enzyme | | 8,73E-03 | BCL2L11,CBX7,CRIP2,FOXO1,FOXO3,NFKBIB,TRAF3 |
| vasoactive intestinal peptide | biologic drug | | 8,73E-03 | CCL2,CSF1,CXCL8,FOS,IL12A,MYD88,VEGFA |
| Mi2 | other | Inhibited | 8,73E-03 | ATF3,ETS2,FOS,IL1B,IL6,JUN,TNF |
| paroxetine | chemical drug | | 8,73E-03 | ABCA1,ADM,BDNF,IL1B,MAP2,TNF,UCN2 |
| miR-938 (miRNAs w/seed GCCCUUA) | mature microRNA | Inhibited | 8,80E-03 | [illegible] |
| miR-6729-3p (miRNAs w/seed CAUCCCC) | mature microRNA | Inhibited | 8,90E-03 | [illegible] |
| miR-1269a (and other miRNAs w/seed UGGACUG) | mature microRNA | Inhibited | 8,91E-03 | [illegible] |
| miR-5089-3p (and other miRNAs w/seed UGCUACU) | mature microRNA | Inhibited | 8,91E-03 | [illegible] |
| miR-4363 (miRNAs w/seed UUCUAAG) | mature microRNA | Inhibited | 8,92E-03 | [illegible] |
| KDM3A | transcription regulator | Activated | 8,92E-03 | ADM,ARHGEF26,CCL2,CCN1,EDN1,FSTL3,GDF15,HMOX1,KLF5,NKX3-1 |
| HOXC6 | transcription regulator | | 8,92E-03 | CASP1,ERRFI1,GAS2L3,MME,NFKB2,RFX7,SIPA1L1,SLC1A3,TNFAIP6,VCAM1 |
| ibuprofen | chemical drug | | 8,92E-03 | COL12A1,GDF15,IL1B,IL6,ITGAM,NGFR,PTGS2,TNF,TNFRSF10B,VEGFA |
| miR-3473g (and other miRNAs w/seed AAAAGUG) | mature microRNA | Inhibited | 8,98E-03 | [illegible] |
| miR-4651 (and other miRNAs w/seed GGGGUGG) | mature microRNA | Inhibited | 8,99E-03 | [illegible] |
| miR-6862-5p (and other miRNAs w/seed GGGCAUG) | mature microRNA | Inhibited | 9,03E-03 | [illegible] |
| miR-4267 (miRNAs w/seed CCAGCUC) | mature microRNA | Inhibited | 9,05E-03 | [illegible] |
| FGF10 | growth factor | | 9,11E-03 | AHNAK,ANXA2,CEBPB,CTSH,CYP1B1,LMO7,PTGS2,SCD,SNAI1,SOX9,SPRY2,TSPAN8 |
| JAG1 | growth factor | Activated | 9,11E-03 | CDKN1A,CDKN2B,COL1A1,DUSP1,DUSP10,DUSP2,DUSP5,DUSP6,HES1,SNAI1,SPP1,VIM |
| miR-8088 (miRNAs w/seed CUCGGUA) | mature microRNA | Inhibited | 9,11E-03 | [illegible] |
| miR-3656 (miRNAs w/seed AAGGGGU) | mature microRNA | Inhibited | 9,16E-03 | [illegible] |
| miR-5585-5p (miRNAs w/seed GAAGUAC) | mature microRNA | Inhibited | 9,17E-03 | [illegible] |
| miR-4300 (and other miRNAs w/seed GGGAGCU) | mature microRNA | Inhibited | 9,21E-03 | [illegible] |
| miR-593-3p (miRNAs w/seed GUCUCUG) | mature microRNA | Inhibited | 9,21E-03 | [illegible] |
| SYK/ZAP | group | | 9,24E-03 | CD69,FOXO1,H3-3A/H3-3B,ICOSLG/LOC102723996,IL6,PTGS2,TNF,VCAM1,VEGFC |
| NCSTN | peptidase | | 9,24E-03 | CD68,CEBPB,CEBPD,CSF1R,EGFR,HES1,IRF8,NOTCH1,VIM |
| mercuric chloride | chemical toxicant | | 9,24E-03 | CDKN1A,DDIT3,GADD45A,HBEGF,HMOX1,HSPA1A/HSPA1B,JUN,JUNB,SPP1 |
| KDM5B | enzyme | | 9,26E-03 | CCL5,CD40,IL6,NGFR,NOS3,OASL,SNAI1,VIM |
| dithiothreitol | chemical reagent | | 9,26E-03 | ATF3,DDIT3,ERO1B,GADD45A,HMOX1,IFNB1,SELE,VEGFA |
| sphingosylphosphocholine | chemical - endogenous mammalian | | 9,26E-03 | CCL2,FOS,ICAM1,JUN |
| ganglioside | chemical - endogenous mammalian | | 9,26E-03 | ICAM1,IL6,PTGS2,TNF |
| 2',3'-dialdehyde ATP | chemical reagent | | 9,26E-03 | CXCL10,IL6,PTGS2,TNF |
| [illegible] | chemical drug | | 9,26E-03 | ANGPTL4,ATF3,BCL2L11,CXCL3 |
| R 59022 | chemical - kinase inhibitor | | 9,26E-03 | CCL2,CD69,CXCL8,IL6 |
| L-lysine | chemical - endogenous mammalian | | 9,26E-03 | CXCL2,DDIT3,IL6,SOD2 |
| carbonyl iron | chemical reagent | | 9,26E-03 | HNF4A,IL1B,IL6,TNF |
| Ck2 | complex | | 9,26E-03 | ATF4,FOS,NGFR,ZFP36 |
| cytidylyl-3'-5'-guanosine | chemical reagent | | 9,26E-03 | BCL2L11,HLA-DRB4,IL1B,TNF |
| IRF3 dimer | complex | | 9,26E-03 | CCL5,IFIT2,IFNB1,ISG15 |
| bacterial lipopolysaccharides | chemical - other | | 9,26E-03 | BCL3,CYP2J2,IL1B,TNF |
| bafilomycin A | chemical reagent | | 9,26E-03 | CD40,CXCL10,IL6,TNF |
| RASSF6 | other | Activated | 9,26E-03 | BBC3,BTG2,CASP7,CDKN1A |
| LRRK2 | kinase | | 9,26E-03 | IL1B,SNCA,SQSTM1,TNF |
| MEMO1 | other | | 9,26E-03 | FOS,JUN,SNAI1,STC2 |
| HUWE1 | transcription regulator | | 9,26E-03 | ABCG1,CDKN1A,CDKN2B,ZBTB17 |
| SMAD1/5 | group | | 9,26E-03 | CEBPB,ID3,PDGFA,PLAUR |
| Ferritin | complex | | 9,26E-03 | CCL5,ICAM1,IL1B,NFKBIA |
| SPTAN1 | other | | 9,26E-03 | CDKN1A,ITGA5,SPTA1,SPTB |
| pictilisib | chemical drug | | 9,26E-03 | ANGPTL4,BCL2L11,CXCL8,TNFSF10 |
| UNC5B | transmembrane receptor | | 9,26E-03 | CCL22,CXCL2,IL6,TNF |
| SERPINB2 | other | | 9,26E-03 | CASP10,CASP7,CASP8,SERPINB2 |
| THBD | transmembrane receptor | | 9,26E-03 | CXCL3,IL1B,IL6,TNF |
| TRG | other | | 9,26E-03 | AREG,CXCL1,IL1B,NT5E |
| ZIC2 | transcription regulator | | 9,26E-03 | APOE,CDKN1B,DRD1,EPHA4 |
| mir-217 | microRNA | | 9,26E-03 | HMOX1,KLK7,NR4A2,SIRT1 |
| mir-378 | microRNA | | 9,26E-03 | CRAT,FOXO1,FOXO3,MED13 |
| GUSB | enzyme | | 9,26E-03 | CCN1,CEBPD,CTSV,EGR2 |
| RALBP1 | enzyme | | 9,26E-03 | CDKN1B,COL4A1,IL6,TNF |
| ALOX15B | enzyme | | 9,26E-03 | CDKN1B,KLF4,NKX3-1,VEGFA |
| IL4I1 | enzyme | Inhibited | 9,26E-03 | CCL2,CXCL1,CXCL8,IL1A |
| ERP29 | transporter | | 9,26E-03 | PARD3,SNAI1,TJP1,ZEB2 |
| TSG101 | transcription regulator | | 9,26E-03 | CDKN1A,FAM98B,NR3C1,STMN1 |
| COCH | other | | 9,26E-03 | CXCL2,IL1B,IL6,TNF |
| MC1R | G-protein coupled receptor | | 9,26E-03 | NR4A1,NR4A2,NR4A3,TNF |
| IL22RA1 | transmembrane receptor | | 9,26E-03 | CCL20,CXCL8,HBEGF,SOD2 |
| RECK | other | | 9,26E-03 | CDKN1A,CDKN1B,EGFR,SPRY2 |
| IL13RA2 | transmembrane receptor | | 9,26E-03 | CSF2,IL18R1,TNF,VCAM1 |
| DEK | transcription regulator | | 9,26E-03 | BIRC3,CXCL8,MCL1,NFKBIA |
| HA900 | chemical reagent | | 9,26E-03 | CCL2,CCL5,CXCL3,SOCS3 |
| niflumic acid | chemical drug | | 9,26E-03 | CFLAR,CXCL8,FAS,IL6 |
| 7,8-dihydro-7,8-dihydroxybenzo(a)pyrene 9,10-oxide | chemical toxicant | | 9,26E-03 | EGFR,JUN,PTGS2,VEGFA |
| 1,1-bis(3'-indolyl)-1-(4-trifluoromethylphenyl)methane | chemical reagent | | 9,26E-03 | ATF3,CDKN1A,GDF15,TNFSF10 |
| prodigiosin | chemical toxicant | | 9,26E-03 | BBC3,CDKN1A,DDIT3,TNFRSF10B |
| SR1078 | chemical reagent | | 9,26E-03 | ATF4,DDIT3,IL6,TNF |
| pentazocine | chemical drug | | 9,26E-03 | CAT,CDKN1B,HMOX1,NFE2L2 |
| miR-3973 (miRNAs w/seed CAAAGUA) | mature microRNA | Inhibited | 9,27E-03 | [illegible] |
| miR-219b-5p (and other miRNAs w/seed GAUGUCC) | mature microRNA | Inhibited | 9,27E-03 | [illegible] |
| miR-6505-3p (and other miRNAs w/seed GACUUCU) | mature microRNA | Inhibited | 9,28E-03 | [illegible] |
| delta-12-prostaglandin J2 | chemical - endogenous mammalian | | 9,31E-03 | CFLAR,CSF2,TNF |
| beta-glucan | chemical drug | | 9,31E-03 | IL12A,IL1B,IL1RN |
| teniposide | chemical drug | | 9,31E-03 | CCL5,CDKN1A,FAS |
| [Lys15,Arg16,Leu27]VIP(1-7)GRF(8-27) | chemical reagent | | 9,31E-03 | CCL2,CXCL8,IL6 |
| AOPEP | peptidase | | 9,31E-03 | IL1B,IL6,TNF |
| ADRA1 | group | | 9,31E-03 | EGR1,FOS,JUNB |
| dienogest | chemical drug | | 9,31E-03 | CXCL8,HSD17B7P2,PTGS2 |
| rintatolimod | chemical drug | | 9,31E-03 | IFNB1,IL1A,IL6 |
| ATPase | group | | 9,31E-03 | CD40,ICAM1,VCAM1 |
| edratide | biologic drug | | 9,31E-03 | EGR2,EGR3,FOXO3 |
| IFN alpha receptor | complex | | 9,31E-03 | IRF7,MICB,MX1 |
| beta-sitosterol | chemical reagent | | 9,31E-03 | PTGS2,SELE,VCAM1 |
| CpG ODN M362 | chemical reagent | | 9,31E-03 | IL12A,IL6,TNF |
| olmesartan | chemical drug | | 9,31E-03 | CYBA,ICAM1,UCP2 |
| SMPD3 | enzyme | | 9,31E-03 | FOXO1,ICAM1,VCAM1 |
| Gpcr | group | | 9,31E-03 | EGFR,FOS,JUN |
| BCAS2 | other | | 9,31E-03 | BBC3,CDKN1A,PMAIP1 |
| TRIM56 | other | | 9,31E-03 | IFIT2,IFNB1,MYD88 |
| PYHIN1 | other | | 9,31E-03 | CDKN1A,IL6,TNF |
| G6PC3 | phosphatase | | 9,31E-03 | ATF4,CYBA,DDIT3 |
| GATM | enzyme | | 9,31E-03 | CXCL3,IL6,TNF |
| [illegible] | transcription regulator | | 9,31E-03 | CFLAR,PTGS2,XIAP |
| benzenesulfonic acid | chemical reagent | | 9,31E-03 | IL1A,IL6,TNF |
| IgG2b | complex | | 9,31E-03 | IL1B,IL6,TNF |
| Mucin | group | | 9,31E-03 | CD80,CXCL8,PTGS2 |
| TRAFD1 | other | | 9,31E-03 | IFNB1,IL6,TNF |
| DCB 3503 | chemical reagent | | 9,31E-03 | IL1B,IL6,TNF |
| STX11 | transporter | | 9,31E-03 | IL1B,IL6,TNF |
| AHSG | other | | 9,31E-03 | CXCL8,IL6,TNF |
| ERN2 | kinase | | 9,31E-03 | DDIT3,ICAM1,XBP1 |
| NCK2 | kinase | | 9,31E-03 | ATF4,DDIT3,PPP1R15A |
| LOC102724788/PRODH | enzyme | | 9,31E-03 | PTGS2,TNFRSF10B,TNFSF10 |
| PPP1R15B | phosphatase | | 9,31E-03 | BBC3,CDKN1A,LATS2 |
| APAF1 | other | | 9,31E-03 | DDIT3,IFNB1,VIM |
| PROS1 | other | | 9,31E-03 | CXCL8,IFNB1,IL6 |
| FGG | other | | 9,31E-03 | IL1B,IL6,TNF |
| PILRB | other | | 9,31E-03 | IL1B,IL6,TNF |
| [illegible] (includes others) | other | | 9,31E-03 | IL1B,IL6,TNF |
| EIF2AK1 | kinase | | 9,31E-03 | IL1B,IL6,TNF |
| PFN1 | other | | 9,31E-03 | ANXA2,SQSTM1,VCAM1 |
| KIDINS220 | transcription regulator | | 9,31E-03 | CD69,EGR1,FOS |
| RPS3 | enzyme | | 9,31E-03 | BCL2L11,CXCL8,NFKBIA |
| CCL20 | cytokine | | 9,31E-03 | CXCL2,CXCL3,TNF |
| ADAM8 | peptidase | | 9,31E-03 | CCL5,CXCL10,TNF |
| PRMT2 | enzyme | | 9,31E-03 | IL6,SNAI1,TNF |
| LILRB3 | transmembrane receptor | | 9,31E-03 | CSF2,IL1B,IL6 |
| TNFAIP8 | other | | 9,31E-03 | CXCL3,IL1B,IL6 |
| RAB7A | enzyme | | 9,31E-03 | EGR1,FOS,VIM |
| DGKA | kinase | | 9,31E-03 | CD69,IL1B,WEE1 |
| ODN-A151 | chemical reagent | | 9,31E-03 | IFNB1,TNF,TNFSF10 |
| Mas1 | transmembrane receptor | | 9,31E-03 | CXCL2,PDGFA,TNF |
| GCLM | enzyme | | 9,31E-03 | FOS,JUN,JUNB |
| HG-9-91-01 | chemical reagent | | 9,31E-03 | IL1B,IL6,TNF |
| CC-122 | chemical drug | | 9,31E-03 | DDX58,IFIT3,IRF7 |
| MI-773 | chemical drug | | 9,31E-03 | BBC3,BCL2L11,CDKN1A |
| pubchem compound 11683566 | chemical reagent | | 9,31E-03 | IL1B,IL6,TNFSF13B |
| 2-cyclohexen-1-one | chemical - endogenous non-mammalian | | 9,31E-03 | ICAM1,PTGS2,VCAM1 |
| mevalonolactone | chemical reagent | | 9,31E-03 | ICAM1,NOS3,PTGS2 |
| cimetidine | chemical drug | | 9,31E-03 | FAS,GDF15,SELE |
| SD-282 | chemical - kinase inhibitor | | 9,31E-03 | CSF2,CXCL8,TNF |
| butylated hydroxytoluene | chemical toxicant | | 9,31E-03 | FOS,JUN,PTGS2 |
| PD 150606 | chemical - protease inhibitor | | 9,31E-03 | AHR,CD69,CDKN1B |
| ethacrynic acid | chemical drug | | 9,31E-03 | AKR1C1/AKR1C2,HMOX1,MCL1 |
| phenol derivative | chemical toxicant | | 9,31E-03 | HMOX1,NFE2L2,USP47 |
| [illegible] | chemical drug | | 9,31E-03 | ICAM1,VCAM1,VEGFA |
| FT671 | chemical - protease inhibitor | | 9,31E-03 | BBC3,CDKN1A,RPS27L |
| retinaldehyde | chemical - endogenous mammalian | | 9,31E-03 | BMP2,EGFR,IL6 |
| aclarubicin | chemical drug | | 9,31E-03 | MCL1,PTGS2,TNFRSF10B |
| methyllycaconitine | chemical toxicant | | 9,31E-03 | FOS,GCH1,IL6 |
| acetyl-11-keto-beta-boswellic acid | chemical reagent | | 9,31E-03 | DDIT3,TNF,TNFRSF10B |
| Hg2+ | chemical - endogenous non-mammalian | | 9,31E-03 | CYP1A1,FXYD2,HMOX1 |
| miR-558 (and other miRNAs w/seed GAGCUGC) | mature microRNA | Inhibited | 9,32E-03 | [illegible] |
| miR-433-3p (miRNAs w/seed UCAUGAU) | mature microRNA | Inhibited | 9,33E-03 | [illegible] |
| miR-212-5p (miRNAs w/seed CCUUGGC) | mature microRNA | Inhibited | 9,37E-03 | [illegible] |
| miR-298-3p (miRNAs w/seed AGGGUUG) | mature microRNA | Inhibited | 9,44E-03 | [illegible] |
| miR-651 (miRNAs w/seed AGCAAGA) | mature microRNA | Inhibited | 9,45E-03 | [illegible] |
| miR-1306-3p (miRNAs w/seed CGUUGGC) | mature microRNA | Inhibited | 9,46E-03 | [illegible] |
| miR-1245b-5p (and other miRNAs w/seed GGAGGGA) | mature microRNA | Inhibited | 9,46E-03 | [illegible] |
| miR-31-5p (and other miRNAs w/seed GGCAAGA) | mature microRNA | Inhibited | 9,47E-03 | [illegible] |
| miR-6780a-3p (miRNAs w/seed UCCUCUG) | mature microRNA | Inhibited | 9,48E-03 | [illegible] |
| miR-4455 (miRNAs w/seed GGGUGUG) | mature microRNA | Inhibited | 9,50E-03 | [illegible] |
| miR-4475 (miRNAs w/seed AAGGGAC) | mature microRNA | Inhibited | 9,50E-03 | [illegible] |
| miR-548x-3p (miRNAs w/seed AAGAACC) | mature microRNA | Inhibited | 9,50E-03 | [illegible] |
| mir-27 | microRNA | | 9,56E-03 | ABCA1,BMP2,EDN1,EGFR,FBXW7,FOXO1,IL1B,IL6,MAP2K4,PTGS2,SPRY2,TNF,ZBTB10 |
| C5AR1 | G-protein coupled receptor | Activated | 9,56E-03 | C3,C5,CD40,COL1A1,CSF3,CXCL2,CXCL3,CXCL8,EIF2AK3,ERN1,IL1B,IL6,TNF |
| SIN3A | transcription regulator | | 9,56E-03 | ARID4B,BCL6,BDNF,COL1A1,CYP1A1,DDIT3,GADD45B,KLF10,KLF6,PTGS2,RBL2,SMAD7,TXNIP |
| miR-892b (miRNAs w/seed ACUGGCU) | mature microRNA | Inhibited | 9,74E-03 | [illegible] |
| miR-876-5p (miRNAs w/seed GGCCCCA) | mature microRNA | Inhibited | 9,74E-03 | [illegible] |
| miR-3191-5p (miRNAs w/seed UCUCUGG) | mature microRNA | Inhibited | 9,79E-03 | [illegible] |
| miR-450b-3p (and other miRNAs w/seed UUGGGGA) | mature microRNA | Inhibited | 9,81E-03 | [illegible] |
| NRF1 | transcription regulator | | 9,83E-03 | ATP1A1,CCL20,CXCL8,FMR1,HMOX1,IL1A,IL1RN,NCALD,PPRC1,PVR,SNAI1,TJP1,VIM,ZEB1 |
| RARG | ligand-dependent nuclear receptor | | 9,83E-03 | ABCA1,AREG,BMP2,COL4A1,DUSP1,EGR1,EGR2,FOS,GATA4,HAS2,HOXB8,IL6,JUN,KLF4,NR2F1,RARA,UBA7 |
| miR-653-5p (miRNAs w/seed UGAAACA) | mature microRNA | Inhibited | 9,87E-03 | [illegible] |
| 2-bromoethylamine | chemical reagent | | 9,88E-03 | ANXA2,C4A/C4B,ELF3,IER3,LXN,SPP1,STING1,TM4SF1,TSPAN8,UPP1,VIM |
| miR-543-3p (and other miRNAs w/seed AACAUUC) | mature microRNA | Inhibited | 9,88E-03 | [illegible] |
| rifampin | chemical drug | | 9,88E-03 | [illegible] |
| miR-5190 (miRNAs w/seed CAGUGAC) | mature microRNA | Inhibited | 9,88E-03 | [illegible] |
| Srebp | group | | 9,90E-03 | CDKN1A,FOS,IDH1,NPC1,SLC25A1,UCP2 |
| trastuzumab | biologic drug | | 9,90E-03 | BNIP3L,CEBPB,CXCL8,IL6,PDCD4,TOP2A |
| Fgfr | group | | 9,90E-03 | CDKN1B,FGFR1,FOS,HMOX1,MCL1,SPP1 |
| artesunic acid | chemical drug | | 9,90E-03 | CCL2,EGFR,IL1B,MMP7,PTGS2,TNF |
| HNF1A-AS1 | other | Inhibited | 9,90E-03 | H2AC6,H2BC12,H2BC21,H2BC5,NCAPG,NEK2 |
| NUMB | other | Inhibited | 9,90E-03 | BIRC3,CDKN1A,CFLAR,EGFR,HES1,IFI16 |
| SLC29A1 | transporter | | 9,90E-03 | CDKN1A,FAS,GADD45A,ITGA5,PPM1D,RPS27L |
| TEK | kinase | Activated | 9,90E-03 | CDKN1A,IL1A,IL6,TJP1,TNF,VEGFA |
| CCNH | transcription regulator | | 9,90E-03 | CDKN1A,COMMD3-BMI1,FGF19,GATA4,HAND1,UCP2 |
| [illegible] | other | | 9,90E-03 | DDIT3,ERN1,ERO1B,HERPUD1,HMOX1,XBP1 |
| EPHA2 | kinase | | 9,90E-03 | EGFR,FGF2,SELE,SLIT2,TJP1,VCAM1 |
| RARRES3 | transmembrane receptor | Inhibited | 9,90E-03 | CCL5,CCND2,CXCL2,IL1B,IL6,TNF |
| GSK3A | kinase | | 9,90E-03 | CFLAR,GATA4,SQSTM1,TNF,TNFSF10,TNNC1 |
| IL1RL1 | transmembrane receptor | | 9,90E-03 | CXCL10,CXCL3,CXCL8,IL6,ITGAM,TNF |
| MST1R | kinase | | 9,90E-03 | CXCL3,FOS,JUN,ROS1,TNF,VIM |
| NRTN | growth factor | | 9,90E-03 | EGR1,EGR2,EGR4,FOSB,IL6,TNF |
| ACTN4 | transcription regulator | Activated | 9,90E-03 | ANGPTL4,CCL2,CXCL8,FOS,IL1B,SNAI1 |
| triflusal | chemical drug | Inhibited | 9,90E-03 | CSF1,CSF2,CSF3,CXCL8,PTGS2,TNF |
| lycopene | chemical drug | | 9,90E-03 | CD69,CDKN1A,KLF2,MMP7,PTGS2,SLC22A1 |
| dichlororibofuranosylbenzimidazole | chemical toxicant | | 9,90E-03 | CDKN1A,CDKN1B,CXCL8,CYP1A1,CYP3A5,SLC5A5 |
| miR-1226-5p (and other miRNAs w/seed UGAGGGC) | mature microRNA | Inhibited | 9,90E-03 | [illegible] |
| miR-6124a-3p (miRNAs w/seed GAGACAG) | mature microRNA | Inhibited | 9,91E-03 | [illegible] |
| miR-708-5p (and other miRNAs w/seed AGGAGCU) | mature microRNA | Inhibited | 9,94E-03 | [illegible] |
| miR-2116-5p (miRNAs w/seed GUUCUUA) | mature microRNA | Inhibited | 9,98E-03 | [illegible] |
| miR-6070 (miRNAs w/seed CGGUUCC) | mature microRNA | Inhibited | 1,00E-02 | [illegible] |
| miR-2110 (and other miRNAs w/seed UGGGGAA) | mature microRNA | Inhibited | 1,00E-02 | [illegible] |
| miR-8082 (miRNAs w/seed AGUGAUU) | mature microRNA | Inhibited | 1,01E-02 | [illegible] |
| miR-214-3p (and other miRNAs w/seed CAGCAGG) | mature microRNA | Inhibited | 1,01E-02 | [illegible] |
| CLOCK | transcription regulator | Activated | 1,02E-02 | [illegible] |
| miR-4530 (miRNAs w/seed CCAGCAG) | mature microRNA | Inhibited | 1,02E-02 | [illegible] |
| miR-4513 (and other miRNAs w/seed GACUGAC) | mature microRNA | Inhibited | 1,03E-02 | [illegible] |
| miR-4446-3p (miRNAs w/seed AGGGCUG) | mature microRNA | Inhibited | 1,03E-02 | [illegible] |
| miR-5007-5p (miRNAs w/seed AGAGUCU) | mature microRNA | Inhibited | 1,04E-02 | [illegible] |
| sorbitol | chemical - endogenous mammalian | | 1,04E-02 | CCN1,CDKN1A,ERRFI1,SGK1,SLC1A3 |
| ginsenoside Rb1 | chemical - endogenous non-mammalian | | 1,04E-02 | CXCL8,FOS,HBEGF,PPARGC1A,SCD |
| Ras | group | | 1,04E-02 | BMP2,CCL2,FOS,IL6,PTGS2 |
| [illegible] | phosphatase | Inhibited | 1,04E-02 | CCL2,CDKN1A,CEBPD,IFI35,PSMB9 |
| SCAVENGER receptor CLASS A | group | | 1,04E-02 | CCL5,CXCL8,IL12A,IL6,TNF |
| TOLLIP | other | | 1,04E-02 | IFNB1,IL6,NFKB1,RELB,TNF |
| TAC4 | other | Activated | 1,04E-02 | IL1B,IL6,TNF,TNFSF10,XBP1 |
| SRSF3 | other | Activated | 1,04E-02 | CXCL8,E2F7,JUN,PDCD4,VEGFA |
| LPAR1 | G-protein coupled receptor | Activated | 1,04E-02 | CXCL8,IL6,KLF5,PTGS2,SPHK1 |
| PIAS4 | transcription regulator | Inhibited | 1,04E-02 | CXCL2,IFNB1,IL1B,IRF1,ITGB8 |
| PTPRE | phosphatase | | 1,04E-02 | CXCL2,CXCL3,INHBA,STAT5A,TSC22D1 |
| SAT1 | enzyme | | 1,04E-02 | CDKN1A,DHFR,IGFBP2,PPARGC1A,PRKAG1 |
| deoxycorticosterone acetate/potassium chloride/sodium chloride | chemical reagent | | 1,04E-02 | CXCL2,HNF4A,IL6,THRB,VDR |
| RIPK3 | kinase | | 1,04E-02 | IL1A,IL1B,IL6,MLKL,TNF |
| RBP3 | transporter | | 1,04E-02 | ATF4,DDIT3,IL20RA,RIPK1,TNF |
| FPR2 | G-protein coupled receptor | | 1,04E-02 | CCL2,CSF1,CXCL8,IL12A,IL6 |
| salmeterol | chemical drug | | 1,04E-02 | CFTR,DUSP1,IL6,SOCS3,TNF |
| meclizine | chemical drug | | 1,04E-02 | CYP2B6,CYP3A4,IL1A,NAMPT,PPRC1 |
| beta-1,3-glucan | chemical drug | Activated | 1,04E-02 | FGF2,IL6,PDGFA,TNF,VEGFA |
| miR-6076 (and other miRNAs w/seed GCAUGAC) | mature microRNA | Inhibited | 1,05E-02 | [illegible] |
| miR-1268a (and other miRNAs w/seed GCUGAGC) | mature microRNA | Inhibited | 1,05E-02 | [illegible] |
| di(2-ethylhexyl) phthalate | chemical toxicant | Activated | 1,06E-02 | CCL2,CEACAM1,CXCL1,CXCL8,EGFR,EGR1,FOS,FOSB,IRAK2,JUN,MYD88,ZFP36 |
| miR-6078 (miRNAs w/seed CGCCUGA) | mature microRNA | Inhibited | 1,06E-02 | [illegible] |
| KL | enzyme | | 1,06E-02 | EGR1,ICAM1,IL6,IRF7,MMP7,SIRT1,SOD2,TNF,VDR,VEGFA |
| S100A4 | other | | 1,06E-02 | CCL5,CDKN1A,CSF3,CXCL3,EFNA1,PLAU,SNAI1,THBS1,VCAM1,VIM |
| SRSF2 | transcription regulator | | 1,06E-02 | ATF4,BAAT,COL1A1,DDIT3,EP300,FOXA2,PPP1R15A,SRSF6,TNFRSF10A,VIM |
| 4-hydroxynonenal | chemical toxicant | | 1,06E-02 | AKR1C1/AKR1C2,BAG3,HSPA1A/HSPA1B,ICAM1,IL1B,IL6,JUN,MME,PTGS2,TNF |
| ouabain | chemical drug | Activated | 1,06E-02 | ATP1A1,ATP4A,BDNF,CDKN1A,CSF2,DDIT3,FOS,TNFRSF10A,TNFRSF10B,VIM |
| L-ornithine | chemical - endogenous mammalian | | 1,06E-02 | ARG2,CDKN1A |
| diphosphoryl lipid A | chemical toxicant | | 1,06E-02 | IL1B,TNF |
| 1,4-glucan | chemical - endogenous mammalian | | 1,06E-02 | CXCL8,TNF |
| arbutin | chemical drug | | 1,06E-02 | CDKN1A,PTGS2 |
| syringin | chemical - endogenous non-mammalian | | 1,06E-02 | IL1B,TNF |
| 7beta-hydroxycholesterol | chemical - endogenous mammalian | | 1,06E-02 | NR4A1,NR4A2 |
| Gelonin | chemical toxicant | | 1,06E-02 | MCL1,XIAP |
| chaetocin | chemical reagent | | 1,06E-02 | CDKN1A,TNFRSF10B |
| Plc beta | group | | 1,06E-02 | IL6,NFKB1 |
| MEK1/2 | group | | 1,06E-02 | DUSP1,FOS |
| PLA2 | group | | 1,06E-02 | FOS,TNF |
| Rab5 | group | | 1,06E-02 | ADRB2,IL6 |
| Max-Myc | complex | | 1,06E-02 | CFLAR,ODC1 |
| [illegible] | chemical drug | | 1,06E-02 | NR4A1,NR4A3 |
| diosmetin | chemical - endogenous non-mammalian | | 1,06E-02 | CYP1A1,TNF |
| DF 203 | chemical drug | | 1,06E-02 | CYP1A1,CYP1B1 |
| NPS | other | | 1,06E-02 | FOS,ITGAM |
| METTL21C | enzyme | | 1,06E-02 | MEF2A,MEF2D |
| [illegible] | other | | 1,06E-02 | NFKB2,NFKBIA |
| TCHP | other | | 1,06E-02 | SQSTM1,VEGFA |
| EIF4B | translation regulator | | 1,06E-02 | ISG15,MCL1 |
| ZFAT | transcription regulator | | 1,06E-02 | FOXO1,SQSTM1 |
| TRIM50 | other | | 1,06E-02 | IFIT2,IFNB1 |
| MTNR1A | G-protein coupled receptor | | 1,06E-02 | DIO2,DIO3 |
| FBXO25 | enzyme | | 1,06E-02 | EGR1,FOS |
| NMRAL1 | other | | 1,06E-02 | MX1,TNF |
| POPDC2 | other | | 1,06E-02 | IL1B,TNF |
| ARL16 | other | | 1,06E-02 | CCL5,IFNB1 |
| LYST | transporter | | 1,06E-02 | IFNB1,TNF |
| [illegible] | other | | 1,06E-02 | PGDS,PROM2 |
| MARCHF2 | enzyme | | 1,06E-02 | TFRC,TNFRSF10A |
| [illegible] | chemical drug | | 1,06E-02 | EGFR,TNFSF10 |
| GPR4 | G-protein coupled receptor | | 1,06E-02 | ICAM1,VCAM1 |
| SMYD5 | other | | 1,06E-02 | CXCL10,TNF |
| BATF2 | other | | 1,06E-02 | CCN1,JUN |
| MTNR1B | G-protein coupled receptor | | 1,06E-02 | DIO2,DIO3 |
| MLXIP | transcription regulator | | 1,06E-02 | ARRDC4,TXNIP |
| ARL11 | other | | 1,06E-02 | IL6,TNF |
| SENP6 | peptidase | | 1,06E-02 | BIRC2,CXCL8 |
| Pik | group | | 1,06E-02 | CXCL10,IFNB1 |
| MAP3K21 | kinase | | 1,06E-02 | SNAI1,VIM |
| AFAP1L2 | other | | 1,06E-02 | CXCL8,IL6 |
| ganglioside GM3 (d18:1/18:0) | chemical - endogenous mammalian | | 1,06E-02 | IL6,TNF |
| ganglioside GM3 (d18:1/20:0) | chemical - endogenous mammalian | | 1,06E-02 | IL6,TNF |
| ganglioside GM3 (d18:1/22:0) | chemical - endogenous mammalian | | 1,06E-02 | IL6,TNF |
| Par | group | | 1,06E-02 | CCL20,IL6 |
| IKKA/B | group | | 1,06E-02 | IL1B,TNF |
| diaziquone | chemical drug | | 1,06E-02 | CD40,IL1B |
| RPL19 | other | | 1,06E-02 | CXCL10,CXCL8 |
| pimasertib | chemical drug | | 1,06E-02 | BBC3,BCL2L11 |
| SH3GLB1 | enzyme | | 1,06E-02 | MCL1,SQSTM1 |
| AZD4547 | chemical drug | | 1,06E-02 | CXCL1,TNF |
| Raet1b | other | | 1,06E-02 | HLA-A,TNF |
| MAPK8IP2 | other | | 1,06E-02 | BDNF,FOS |
| IVNS1ABP | other | | 1,06E-02 | PML,SOD2 |
| CELA1 | peptidase | | 1,06E-02 | HLA-A,HSPA1A/HSPA1B |
| TRIM6 | enzyme | | 1,06E-02 | IFIT2,IFNB1 |
| cyproconazole | chemical drug | | 1,06E-02 | CYP2B6,GADD45B |
| NMI | other | | 1,06E-02 | FOS,JUN |
| mir-630 | microRNA | | 1,06E-02 | CDKN1B,DICER1 |
| TPPP3 | other | | 1,06E-02 | ADRB2,TPPP3 |
| lynestrenol | chemical drug | | 1,06E-02 | ABCA1,APOE |
| [illegible] | other | | 1,06E-02 | HLA-A,TNF |
| POLDIP3 | other | | 1,06E-02 | CDKN1A,ISG15 |
| IL1RAPL1 | transmembrane receptor | | 1,06E-02 | IL6,TNF |
| GATAD2B | transcription regulator | | 1,06E-02 | CXCL8,PTGS2 |
| DCTN4 | other | | 1,06E-02 | CCL5,CXCL10 |
| CCT6 | other | | 1,06E-02 | BBC3,CDKN1A |
| AZU1 | peptidase | | 1,06E-02 | CX3CL1,TNF |
| SLC7A11 | transporter | | 1,06E-02 | IL1B,TNF |
| RPS6KA2 | kinase | | 1,06E-02 | EGR1,TRAF3 |

| | | | |
|---|---|---|---|
| DDX24 | enzyme | 1,00E-02 | BBC3,CDKN1A |
| GABRG2 | ion channel | 1,00E-02 | FOS,GPHN |
| ZFP36L1 | transcription regulator | 1,00E-02 | TNF,VEGFA |
| CRADD | other | 1,00E-02 | CCL2,IL6 |
| ATP8A | transporter | 1,00E-02 | VEGFA,VEGFC |
| IMPACT | other | 1,00E-02 | ATF4,DDIT3 |
| PGAM1 | phosphatase | 1,00E-02 | CDKN1A,RBBP8 |
| GPR32 | G-protein coupled receptor | 1,00E-02 | CXCL8,IL6 |
| TMEM98 | other | 1,00E-02 | CXCL8,IL6 |
| CD6B | other | 1,00E-02 | IL1B,TNF |
| RAMP3 | G-protein coupled receptor | 1,00E-02 | FOS,VIM |
| KHDRBS2 | other | 1,00E-02 | CDKN1A,STAT1 |
| OAS3 | other | 1,00E-02 | IFIT1,OAS1 |
| 3-hydroxydodecanoic acid | chemical - endogenous mammalian | 1,00E-02 | CXCL8,TNF |
| 6-n-octylaminouracil | chemical - kinase inhibitor | 1,00E-02 | CXCL8,TNF |
| FGF5 | growth factor | 1,00E-02 | CBF3,FGFR1 |
| CD300E | other | 1,00E-02 | IL6,TNF |
| selumetinib | chemical drug | 1,00E-02 | SPP1,TNF |
| DDT | enzyme | 1,00E-02 | IL6,TNF |
| CDGBP1 | transcription regulator | 1,00E-02 | CDKN1A,PMR1 |
| zebracolib | chemical drug | 1,00E-02 | MCL1,XIAP |
| panatuzumab | biologic drug | 1,00E-02 | HES1,NOTCH1 |
| TMPRSS4 | peptidase | 1,00E-02 | ITGA5,PLAU |
| IL17D | cytokine | 1,00E-02 | CXCL8,IL6 |
| HUS1 | kinase | 1,00E-02 | BBC3,BCL2L11 |
| EYA4 | phosphatase | 1,00E-02 | CXCL10,IFNB1 |
| ATB-346 | chemical drug | 1,00E-02 | IL1B,TNF |
| polysaccharide TA-1 | chemical - endogenous non-mammalian | 1,00E-02 | IL6,TNF |
| BAI | chemical - kinase inhibitor | 1,00E-02 | CFLAR,MCL1 |
| verbolin A | chemical reagent | 1,00E-02 | FAS,TNFRSF10B |
| bimekizumab | biologic drug | 1,00E-02 | CXCL8,IL6 |
| minalrestat | chemical drug | 1,00E-02 | FGF2,PRKCE |
| 2,2-(2-chlorophenyl-4'-chlorophenyl)-1,1-dichloroethene | chemical - endogenous mammalian | 1,00E-02 | AHR,CYP1A1 |
| buformin | chemical drug | 1,00E-02 | ATF4,XBP1 |
| neocuproine | chemical reagent | 1,00E-02 | CXCL1,VEGFA |
| telomestatin | chemical - endogenous non-mammalian | 1,00E-02 | CDKN1A,CDKN1B |
| primaquine | chemical drug | 1,00E-02 | CYP1A1,GCH1 |

[illegible]

| | | | |
|---|---|---|---|
| KLF17 | transcription regulator | 1.72E-02 | CDKN1A,TJP1,VIM |
| CLEC4A | transmembrane receptor | 1.72E-02 | IL1B,IL6,TNF |
| CLEC4D | other | 1.72E-02 | IL1B,IL6,TNF |
| CCN6 | growth factor | 1.72E-02 | ICAM1,SNAI1,ZEB1 |
| TRPM7 | kinase | 1.72E-02 | NOS3,TRPM7,VCAM1 |
| RDH8 | enzyme | 1.72E-02 | CXCL10,IFNB1,TLR3 |
| dynasore | chemical reagent | 1.72E-02 | IFNB1,IL6,VEGFA |
| ghrelin | biologic drug | 1.72E-02 | EGR1,FOS,SCD |
| RABGEF1 | enzyme | 1.72E-02 | CXCL10,IFNB1,IL6 |
| SMPD2 | enzyme | 1.72E-02 | IL1B,IL6,TNF |
| HSPB2 | other | 1.72E-02 | CCL2,CXCL8,ICAM1 |
| FGF13 | growth factor | 1.72E-02 | CDKN1A,HSPA4,VEGFA |
| PPP6C | phosphatase | 1.72E-02 | CCL5,IFNB1,ISG15 |
| DLX4 | transcription regulator | 1.72E-02 | FGF2,TOP2A,VEGFA |
| CXCL6 | cytokine | 1.72E-02 | ITGAM,SNAI1,TNF |
| ABCG2 | transporter | 1.72E-02 | IL1B,PTGS2,TNF |
| PROK2 | other | 1.72E-02 | FOS,IL1B,VEGFA |
| RALB | enzyme | 1.72E-02 | CDKN1A,CDKN1B,IFNB1 |
| BCL2L12 | other | 1.72E-02 | BBC3,CDKN1A,TNFRSF10A |
| CASP4 | peptidase | 1.72E-02 | IFNB1,IL1A,IL1B |
| SOX18 | transcription regulator | 1.72E-02 | IL6ST,PROX1,VCAM1 |
| NUCB2 | other | 1.72E-02 | FOS,IL6,PTGS2 |
| AMBN | growth factor | 1.72E-02 | CDKN1A,CDKN1B,TNFRSF1B |
| MAGED1 | transcription regulator | 1.72E-02 | CDKN1A,JUN,TNF |
| RNF111 | enzyme | 1.72E-02 | CDKN2B,FOXA2,SMAD7 |
| F13A1 | enzyme | 1.72E-02 | IL1B,IL6,TNF |
| PTPRR | phosphatase | 1.72E-02 | FOS,FOSL1,JUN |
| SRPK1 | kinase | 1.72E-02 | CXCL10,MCL1,VEGFA |
| GRM5 | G-protein coupled receptor | 1.72E-02 | FOS,SOD2,TNF |
| DNAJA3 | other | 1.72E-02 | CXCL8,EGFR,VEGFA |
| DYNLL1 | other | 1.72E-02 | BCL2L11,CDKN1A,FOS |
| FZD6 | G-protein coupled receptor | 1.72E-02 | SPP1,TJP1,VEGFA |
| CARD14 | other | 1.72E-02 | BCL10,CXCL2,PIK3CA |
| CCL15 | cytokine | 1.72E-02 | CXCL8,ICAM1,IL6 |
| CLIM | transporter | 1.72E-02 | ATF4,DDIT3,IRS2 |
| CTSK | peptidase | 1.72E-02 | CTSV,IL6,PTGS2 |
| PPIG | enzyme | 1.72E-02 | SNAI1,VIM,ZEB1 |

[illegible]

| | | | |
|---|---|---|---|
| sesamol | chemical - endogenous mammalian | 2.78E-02 | CD69,IL6,TNF |
| nicotinic acetylcholine receptor | complex | 2.78E-02 | FOS,JUN,JUNB |
| Stat1 dimer | complex | 2.78E-02 | EIF2AK2,IRF1,PIM1 |
| doramapimod | chemical - kinase inhibitor | 2.78E-02 | HAS2,IL6,TNF |
| blinatumomab | biologic drug | 2.78E-02 | CCL2,CXCL8,IL6 |
| PRCP | peptidase | 2.78E-02 | KLF2,KLF4,NOS3 |
| PDCD10 | other | 2.78E-02 | KLF2,KLF4,VEGFA |
| PIM1EG | other | 2.78E-02 | IL6,SOCS3,TNF |
| EIF2A | translation regulator | 2.78E-02 | ATF4,DDIT3,XIAP |
| ST6GALNAC1 | enzyme | 2.78E-02 | B2M,SNAI1,VIM |
| PTK | group | 2.78E-02 | IL6,NR3C1,TNF |
| CC2D1A | transcription regulator | 2.78E-02 | CCL5,IFNB1,IL6 |
| SLC5A8 | transporter | 2.78E-02 | TNFRSF10A,TNFRSF10B,TNFSF10 |
| TP73-AS1 | other | 2.78E-02 | IL1B,IL6,TNF |
| KMT5B | enzyme | 2.78E-02 | DUSP1,MT2A,SGK1 |
| ADAM21 | peptidase | 2.78E-02 | FAM43A,GLS,TPPP3 |
| SFRP5 | transmembrane receptor | 2.78E-02 | CCL2,IL1B,TNF |
| FNIP1 | other | 2.78E-02 | PPARGC1A,TNNC1,TNNT1 |
| ZC3HAV1 | other | 2.78E-02 | CXCL10,IL6,TNF |
| saracatinib | chemical drug | 2.78E-02 | IL1B,NLRP3,PLCG2 |
| valine | chemical - endogenous mammalian | 2.78E-02 | CD83,CDKN1A,SOD2 |
| catumaxomab | biologic drug | 2.78E-02 | CD40,CD69,TNFSF10 |
| IER3 | other | 2.78E-02 | BIRC2,BIRC3,MCL1 |
| SELL | transmembrane receptor | 2.78E-02 | IL1B,IL6,TNF |
| EZH1 | enzyme | 2.78E-02 | IFNB1,IL6,TNF |
| DUSP10 | phosphatase | 2.78E-02 | IL6,KLF5,TNF |
| CCNA2 | other | 2.78E-02 | CDKN1A,CDKN1B,DHFR |
| mir-296 | microRNA | 2.78E-02 | CA9,COL1A1,IL1B |
| TSC22D1 | transcription regulator | 2.78E-02 | CXCL8,IL1B,IL6 |
| PTBP3 | other | 2.78E-02 | VIM,ZEB1,ZEB2 |
| P2RY4 | G-protein coupled receptor | 2.78E-02 | ICAM1,SELE,VCAM1 |
| TPSD1 | peptidase | 2.78E-02 | CXCL8,IL6,PTGS2 |
| TBXA2R | G-protein coupled receptor | 2.78E-02 | ICAM1,PTGS2,VCAM1 |
| PIK3C3 | kinase | 2.78E-02 | CD40,HLA-A,IGF2 |
| MGLL | enzyme | 2.78E-02 | IL1A,IL6,TNF |
| PIP5K1C | kinase | 2.78E-02 | CD274,ERRFI1,PIP5K1A |
| LPAR2 | G-protein coupled receptor | 2.78E-02 | CXCL8,IL6,PTGS2 |
| FKBP1A | enzyme | 2.78E-02 | CDKN1A,SGC1,XIAP |
| TRPA1 | transporter | 2.78E-02 | CXCL3,IL1B,IL6 |
| MS4A1 | other | 2.78E-02 | CD69,MCL1,XIAP |
| GLP1R | G-protein coupled receptor | 2.78E-02 | ABCA1,FOS,IL6 |
| NPAS2 | transcription regulator | 2.78E-02 | CRY1,PER1,PER2 |
| KLRB1 | transmembrane receptor | 2.78E-02 | CCL5,CSF2,TNF |
| CLEC2A | other | 2.78E-02 | CCL5,CSF2,CXCL10 |
| Eda | other | 2.78E-02 | AREG,EGFR,WNT10A |
| Pde4d | enzyme | 2.78E-02 | CTSV,PTGS2,TNF |
| RHOC | enzyme | 2.78E-02 | ATF3,CDKN1A,DDIT3 |
| triacetyl-3-hydroxyphenyladenosine | chemical reagent | 2.78E-02 | ABCA1,ABCG1,TNF |
| nicorandil | chemical drug | 2.78E-02 | ATF3,JUN,NOS3 |
| trimethylin | chemical reagent | 2.78E-02 | FOS,IL6,TNF |
| hydroxychloroquine | chemical drug | 2.78E-02 | CD69,CFLAR,IL6 |
| PQQ cofactor | chemical - endogenous non-mammalian | 2.78E-02 | HMOX1,NFE2L2,PPARGC1A |
| nigericin | chemical drug | 2.78E-02 | CASP1,EIF2AK2,IL1B |
| poly rI:rC-RNA HMW | chemical reagent | 2.78E-02 | EIF2AK2,IFNB1,IL6 |
| diazepam | chemical drug | 2.78E-02 | CXCL8,FOS,PER1 |
| KI16425 | chemical reagent | 2.78E-02 | PLPP3,PTGS2,SPHK1 |
| BAY601 | chemical reagent | 2.78E-02 | IL1B,IL6,TNF |
| dBET6 | chemical reagent | 2.78E-02 | ARL4C,DUSP4,PDE4B |
| miR-508-3p inhibitor | chemical reagent | 2.78E-02 | SNAI1,VIM,ZEB1 |
| Boc-D-FMK | chemical reagent | 2.78E-02 | CDKN1B,CXCL8,JAK2 |
| muramide | chemical reagent | 2.78E-02 | CD40,CD83,CEBPB |
| atropine | chemical drug | 2.78E-02 | FOS,PTGS2,VEGFA |
| [illegible] | [illegible] | [illegible] | [illegible] |

| | | | | |
|---|---|---|---|---|
| miR-8055 (miRNAs w/seed UUUGAGC) | mature microRNA | Inhibited | 3.21E-02 | [illegible] |
| miR-498-5p (miRNAs w/seed UUCAAGC) | mature microRNA | Inhibited | 3.22E-02 | [illegible] |
| miR-6852-3p (miRNAs w/seed UUCUGUU) | mature microRNA | Inhibited | 3.22E-02 | [illegible] |
| NEUROD1 | transcription regulator | Inhibited | 3.22E-02 | [illegible] |
| folic acid | chemical - endogenous mammalian | | 3.22E-02 | [illegible] |
| miR-6879 (miRNAs w/seed CUGUGAU) | mature microRNA | Inhibited | 3.24E-02 | [illegible] |
| miR-126a-1-3p (miRNAs w/seed CGGGUUA) | mature microRNA | Inhibited | 3.27E-02 | [illegible] |
| miR-509-5p (and other miRNAs w/seed ACUCCAG) | mature microRNA | Inhibited | 3.28E-02 | [illegible] |
| miR-6540 (miRNAs w/seed UAGUCCU) | mature microRNA | Inhibited | 3.28E-02 | [illegible] |
| miR-1914-5p (miRNAs w/seed CCUGUGC) | mature microRNA | Inhibited | 3.28E-02 | [illegible] |
| miR-324-3p (miRNAs w/seed CCACUGC) | mature microRNA | Inhibited | 3.28E-02 | [illegible] |
| POU2AF1 | transcription regulator | | 3.29E-02 | [illegible] |
| miR-12185-3p (and other miRNAs w/seed CAGCCAC) | mature microRNA | Inhibited | 3.30E-02 | [illegible] |
| ZNF106 | other | | 3.31E-02 | [illegible] |
| ILK | kinase | | 3.31E-02 | [illegible] |
| CDK2 | kinase | | 3.31E-02 | [illegible] |
| miR-651-3p (miRNAs w/seed AAGGAAA) | mature microRNA | Inhibited | 3.31E-02 | [illegible] |
| PD173074 | chemical reagent | | 3.32E-02 | [illegible] |
| RETNLB | other | Activated | 3.32E-02 | [illegible] |
| miR-3618 (miRNAs w/seed GUCUACA) | mature microRNA | Inhibited | 3.33E-02 | [illegible] |
| miR-3924 (miRNAs w/seed UAUGUAU) | mature microRNA | Inhibited | 3.34E-02 | [illegible] |
| miR-1270 (and other miRNAs w/seed UGGAGAU) | mature microRNA | Inhibited | 3.36E-02 | [illegible] |
| miR-574-3p (miRNAs w/seed ACGCUCA) | mature microRNA | Inhibited | 3.36E-02 | [illegible] |
| miR-3667-3p (miRNAs w/seed CCUUCCU) | mature microRNA | Inhibited | 3.38E-02 | [illegible] |
| miR-5092 (miRNAs w/seed AUCCACG) | mature microRNA | Inhibited | 3.39E-02 | [illegible] |
| miR-19b-1-5p (and other miRNAs w/seed GUUUUGC) | mature microRNA | Inhibited | 3.40E-02 | [illegible] |
| miR-3680-3p (miRNAs w/seed UUUGCAU) | mature microRNA | Inhibited | 3.43E-02 | [illegible] |
| miR-451a (and other miRNAs w/seed AACCGUU) | mature microRNA | Inhibited | 3.45E-02 | [illegible] |
| miR-6849-3p (miRNAs w/seed CCAGCCU) | mature microRNA | Inhibited | 3.46E-02 | [illegible] |
| PIK3CA | kinase | | 3.46E-02 | [illegible] |
| miR-606 (miRNAs w/seed GGGUGUU) | mature microRNA | Inhibited | 3.47E-02 | [illegible] |
| miR-3200-3p (miRNAs w/seed ACCUUGC) | mature microRNA | Inhibited | 3.48E-02 | [illegible] |
| miR-4445-5p (miRNAs w/seed GAUUGUU) | mature microRNA | Inhibited | 3.48E-02 | [illegible] |
| miR-589-5p (and other miRNAs w/seed GAGAACC) | mature microRNA | Inhibited | 3.48E-02 | [illegible] |
| miR-6865-3p (miRNAs w/seed UCGGCCG) | mature microRNA | Inhibited | 3.50E-02 | [illegible] |
| miR-3689d (and other miRNAs w/seed GGAGGUG) | mature microRNA | Inhibited | 3.50E-02 | [illegible] |
| miR-873-3p (miRNAs w/seed GAGACUG) | mature microRNA | Inhibited | 3.51E-02 | [illegible] |
| miR-6511a-3p (and other miRNAs w/seed CUCACCA) | mature microRNA | Inhibited | 3.52E-02 | [illegible] |
| Ubiquitin | group | | 3.52E-02 | [illegible] |
| Nuclear factor 1 | group | | 3.52E-02 | [illegible] |
| Rhox5 | transcription regulator | | 3.52E-02 | [illegible] |
| DRD2 | G-protein coupled receptor | | 3.52E-02 | [illegible] |
| ramipril | chemical drug | | 3.52E-02 | [illegible] |
| bufalin | chemical reagent | | 3.52E-02 | [illegible] |
| miR-4290 (miRNAs w/seed GCCCUCC) | mature microRNA | Inhibited | 3.53E-02 | [illegible] |
| miR-3922-5p (miRNAs w/seed CAAGGCC) | mature microRNA | Inhibited | 3.53E-02 | [illegible] |
| miR-4767-5p (and other miRNAs w/seed GGGAAGG) | mature microRNA | Inhibited | 3.53E-02 | [illegible] |
| miR-5194-5p (miRNAs w/seed CUCCAAA) | mature microRNA | Inhibited | 3.53E-02 | [illegible] |
| miR-5684 (miRNAs w/seed ACUCUAG) | mature microRNA | Inhibited | 3.54E-02 | [illegible] |
| miR-1268 (miRNAs w/seed GCAGGAC) | mature microRNA | Inhibited | 3.55E-02 | [illegible] |
| 1,4-bis[2-(3,5-dichloropyridyloxy)]benzene | chemical toxicant | Activated | 3.55E-02 | [illegible] |
| miR-3164-3p (miRNAs w/seed AAGUCUC) | mature microRNA | Inhibited | 3.57E-02 | [illegible] |
| miR-6516-5p (miRNAs w/seed UUGCAGU) | mature microRNA | Inhibited | 3.57E-02 | [illegible] |
| methylmercury | chemical toxicant | | 3.58E-02 | [illegible] |
| hydroxyurea | chemical drug | Activated | 3.58E-02 | [illegible] |
| azoxymethane | chemical toxicant | Activated | 3.58E-02 | [illegible] |
| HOXA10 | transcription regulator | | 3.59E-02 | [illegible] |
| miR-1262 (miRNAs w/seed CCUUUGC) | mature microRNA | Inhibited | 3.60E-02 | [illegible] |
| miR-614 (miRNAs w/seed AACGCCU) | mature microRNA | Inhibited | 3.62E-02 | [illegible] |
| miR-6886-3p (miRNAs w/seed GCCCUUC) | mature microRNA | Inhibited | 3.64E-02 | [illegible] |
| MXD1 | transcription regulator | | 3.65E-02 | [illegible] |
| miR-6863-3p (miRNAs w/seed GUUCAUU) | mature microRNA | Inhibited | 3.65E-02 | [illegible] |
| miR-4795 (miRNAs w/seed GAGUCGG) | mature microRNA | Inhibited | 3.66E-02 | [illegible] |
| miR-4756-3p (miRNAs w/seed CAGAGAU) | mature microRNA | Inhibited | 3.66E-02 | [illegible] |
| telmisartan | chemical drug | | 3.66E-02 | [illegible] |
| miR-4774-5p (miRNAs w/seed CUGGUAU) | mature microRNA | Inhibited | 3.69E-02 | [illegible] |
| miR-4773 (miRNAs w/seed AGAACAG) | mature microRNA | Inhibited | 3.70E-02 | [illegible] |
| miR-3920 (miRNAs w/seed CUGAUUA) | mature microRNA | Inhibited | 3.70E-02 | [illegible] |
| C5A | other | | 3.70E-02 | [illegible] |
| miR-224-3p (and other miRNAs w/seed AAAUGGU) | mature microRNA | Inhibited | 3.70E-02 | [illegible] |
| miR-4327 (miRNAs w/seed GCUUGCA) | mature microRNA | Inhibited | 3.70E-02 | [illegible] |
| nitrofen | chemical toxicant | Inhibited | 3.70E-02 | [illegible] |
| MEX3A | other | Inhibited | 3.70E-02 | [illegible] |
| NSD2 | enzyme | Activated | 3.70E-02 | [illegible] |
| SAA1 | transporter | | 3.70E-02 | [illegible] |
| miR-376b-5p (and other miRNAs w/seed UAUAAUA) | mature microRNA | Inhibited | 3.71E-02 | [illegible] |
| miR-298 (miRNAs w/seed GCAGAAG) | mature microRNA | Inhibited | 3.72E-02 | [illegible] |
| miR-7110-3p (miRNAs w/seed CUCUCUC) | mature microRNA | Inhibited | 3.73E-02 | [illegible] |
| miR-302c-5p (miRNAs w/seed UUAACAU) | mature microRNA | Inhibited | 3.74E-02 | [illegible] |
| miR-3614-3p (miRNAs w/seed AGCCUAC) | mature microRNA | Inhibited | 3.74E-02 | [illegible] |
| miR-6744 (miRNAs w/seed CUAAAGA) | mature microRNA | Inhibited | 3.74E-02 | [illegible] |
| MM-589 | chemical reagent | | 3.75E-02 | [illegible] |
| PTC-209 | chemical reagent | | 3.75E-02 | [illegible] |
| miR-5586-3p (miRNAs w/seed AGACUGA) | mature microRNA | Inhibited | 3.76E-02 | [illegible] |
| AKT inhibitor VIII | chemical reagent | | 3.76E-02 | [illegible] |
| particulate matter | chemical reagent | | 3.76E-02 | [illegible] |
| GPBAR1 | G-protein coupled receptor | | 3.76E-02 | [illegible] |
| [illegible] | group | | 3.76E-02 | [illegible] |
| RYR2 | ion channel | | 3.76E-02 | [illegible] |
| YWHAQ | other | | 3.76E-02 | [illegible] |
| CTSG | peptidase | | 3.76E-02 | [illegible] |
| TRPV1 | ion channel | | 3.76E-02 | [illegible] |
| mir-373 | microRNA | | 3.76E-02 | [illegible] |
| CCL21 | cytokine | | 3.76E-02 | [illegible] |
| HIF3A | transcription regulator | | 3.76E-02 | [illegible] |
| MED16 | transcription regulator | | 3.76E-02 | [illegible] |
| HBP1 | transcription regulator | | 3.76E-02 | [illegible] |
| BCAR1 | enzyme | | 3.76E-02 | [illegible] |
| LMX1A | transcription regulator | | 3.76E-02 | [illegible] |
| PIM3 | kinase | | 3.76E-02 | [illegible] |
| UGCG | enzyme | | 3.76E-02 | [illegible] |
| PEX5L | ion channel | | 3.76E-02 | [illegible] |
| PPP5C | phosphatase | | 3.76E-02 | [illegible] |
| OPRM1 | G-protein coupled receptor | | 3.76E-02 | [illegible] |
| DUSP14 | phosphatase | | 3.76E-02 | [illegible] |
| MIA-602 | chemical reagent | | 3.76E-02 | [illegible] |
| RV 538 | chemical reagent | | 3.76E-02 | [illegible] |
| isoboldine | chemical reagent | | 3.76E-02 | [illegible] |
| gambogic acid | chemical - endogenous non-mammalian | Inhibited | 3.76E-02 | [illegible] |
| S-equol | chemical drug | | 3.76E-02 | [illegible] |
| miR-138-3p (miRNAs w/seed GGAGACG) | mature microRNA | | 3.76E-02 | [illegible] |
| DLL4 | other | | 3.77E-02 | [illegible] |
| RAC2 | enzyme | | 3.77E-02 | [illegible] |
| acetovanillone | chemical drug | | 3.77E-02 | [illegible] |
| NCOR-LXR-Oxysterol-RXR-9 cis RA | complex | | 3.79E-02 | [illegible] |
| NMU | other | | 3.79E-02 | [illegible] |
| miR-3162-3p (and other miRNAs w/seed GUGUUAG) | mature microRNA | Inhibited | 3.79E-02 | [illegible] |
| miR-4329 (miRNAs w/seed CUGAGAC) | mature microRNA | Inhibited | 3.79E-02 | [illegible] |
| Hsp90 | group | | 3.80E-02 | [illegible] |
| mir-29 | microRNA | Inhibited | 3.80E-02 | [illegible] |
| TCL1A | transcription regulator | | 3.80E-02 | [illegible] |
| miR-3200-5p (miRNAs w/seed AUCUGAG) | mature microRNA | Inhibited | 3.82E-02 | [illegible] |
| miR-6499-5p (miRNAs w/seed CGGGCGC) | mature microRNA | Inhibited | 3.84E-02 | [illegible] |
| miR-218-2-3p (and other miRNAs w/seed AUGGUUC) | mature microRNA | Inhibited | 3.85E-02 | [illegible] |
| miR-1247-5p (miRNAs w/seed CCCGUCC) | mature microRNA | Inhibited | 3.85E-02 | [illegible] |
| miR-1909-3p (and other miRNAs w/seed GCAGGGG) | mature microRNA | Inhibited | 3.86E-02 | [illegible] |
| miR-122-3p (miRNAs w/seed ACGCCAU) | mature microRNA | Inhibited | 3.86E-02 | [illegible] |
| miR-4712-3p (miRNAs w/seed AUGAGAG) | mature microRNA | Inhibited | 3.86E-02 | [illegible] |
| miR-1237-3p (and other miRNAs w/seed CCUUCUG) | mature microRNA | Inhibited | 3.87E-02 | [illegible] |
| miR-767-3p (miRNAs w/seed CUGCUCA) | mature microRNA | Inhibited | 3.87E-02 | [illegible] |
| HDAC2 | transcription regulator | | 3.90E-02 | [illegible] |
| miR-3160-3p (miRNAs w/seed GAGCUGA) | mature microRNA | Inhibited | 3.90E-02 | [illegible] |
| miR-4421 (and other miRNAs w/seed CCUGUCU) | mature microRNA | Inhibited | 3.92E-02 | [illegible] |
| miR-507 (and other miRNAs w/seed UUUGCAC) | mature microRNA | Inhibited | 3.92E-02 | [illegible] |
| miR-887-5p (miRNAs w/seed UUGGGAG) | mature microRNA | Inhibited | 3.92E-02 | [illegible] |
| miR-6770-5p (miRNAs w/seed GAGAAGG) | mature microRNA | Inhibited | 3.93E-02 | [illegible] |
| miR-3189-3p (miRNAs w/seed CCUUGGG) | mature microRNA | Inhibited | 3.93E-02 | [illegible] |
| CHEK1 | kinase | | 3.93E-02 | [illegible] |
| PTPN2 | phosphatase | | 3.93E-02 | [illegible] |
| SMURF1 | enzyme | | 3.93E-02 | [illegible] |
| ITGB4 | transmembrane receptor | Activated | 3.93E-02 | [illegible] |
| NT5E | phosphatase | | 3.93E-02 | [illegible] |
| KCNIP3 | transcription regulator | | 3.93E-02 | [illegible] |
| KLRC4-KLRK1/KLRK1 | transmembrane receptor | | 3.93E-02 | [illegible] |
| MUC4 | other | | 3.93E-02 | [illegible] |
| EMD | other | | 3.93E-02 | [illegible] |
| pirfenidone | chemical drug | | 3.93E-02 | [illegible] |
| vitamin K2 | chemical drug | | 3.93E-02 | [illegible] |
| CpdX | chemical reagent | | 3.93E-02 | [illegible] |
| vanadate | chemical - other | Activated | 3.93E-02 | [illegible] |
| miR-1229-3p (miRNAs w/seed UCUCACC) | mature microRNA | Inhibited | 3.96E-02 | [illegible] |
| miR-1970 (and other miRNAs w/seed GUGUCAC) | mature microRNA | Inhibited | 3.96E-02 | [illegible] |
| miR-6785-3p (miRNAs w/seed CAUCGCC) | mature microRNA | Inhibited | 3.97E-02 | [illegible] |
| CIP2A | other | | 3.97E-02 | [illegible] |
| miR-6812-3p (miRNAs w/seed CGCUCAU) | mature microRNA | Inhibited | 3.99E-02 | [illegible] |
| Rb | group | | 4.00E-02 | [illegible] |
| miR-1914-3p (and other miRNAs w/seed GAGGGGU) | mature microRNA | Inhibited | 4.01E-02 | [illegible] |
| miR-3938 (miRNAs w/seed AUUCCCU) | mature microRNA | Inhibited | 4.01E-02 | [illegible] |
| miR-4707-3p (miRNAs w/seed GCCCGCC) | mature microRNA | Inhibited | 4.03E-02 | [illegible] |
| miR-7155-5p (miRNAs w/seed CUGGGGU) | mature microRNA | Inhibited | 4.04E-02 | [illegible] |
| miR-1243 (miRNAs w/seed ACUGGAU) | mature microRNA | Inhibited | 4.04E-02 | [illegible] |
| miR-3150a-3p (and other miRNAs w/seed UGGGGAG) | mature microRNA | Inhibited | 4.08E-02 | [illegible] |
| miR-656-5p (miRNAs w/seed CCCUAUC) | mature microRNA | Inhibited | 4.08E-02 | [illegible] |
| miR-8076 (miRNAs w/seed AUAUGGA) | mature microRNA | Inhibited | 4.08E-02 | [illegible] |
| miR-6760-5p (miRNAs w/seed AGGGAGA) | mature microRNA | Inhibited | 4.08E-02 | [illegible] |
| BNIP3L | other | | 4.11E-02 | [illegible] |
| miR-7975 (miRNAs w/seed UCCUAGU) | mature microRNA | Inhibited | 4.11E-02 | [illegible] |
| miR-7162-3p (miRNAs w/seed CUGAGGU) | mature microRNA | Inhibited | 4.11E-02 | [illegible] |
| Stat3-Stat3 | complex | | 4.12E-02 | [illegible] |
| grape seed extract | chemical drug | | 4.12E-02 | [illegible] |
| PTH1R | G-protein coupled receptor | | 4.12E-02 | [illegible] |
| PRLR | transmembrane receptor | | 4.12E-02 | [illegible] |
| 12(S)-hydroxyeicosatetraenoic acid | chemical - endogenous non-mammalian | | 4.12E-02 | [illegible] |
| 15-epi-lipoxin A4 | chemical - endogenous mammalian | | 4.12E-02 | [illegible] |
| UDP | chemical - endogenous mammalian | | 4.12E-02 | [illegible] |
| Vn | complex | | 4.12E-02 | [illegible] |
| L-phenylalanine | chemical - endogenous mammalian | | 4.12E-02 | [illegible] |
| ganglioside GM3 | chemical - endogenous mammalian | | 4.12E-02 | [illegible] |
| ganglioside GD1a | chemical - endogenous mammalian | | 4.12E-02 | [illegible] |
| GCS-100 | chemical drug | | 4.12E-02 | [illegible] |
| 2,5-bis(5-hydroxymethyl-2-thienyl)furan | chemical reagent | | 4.12E-02 | [illegible] |
| 2-amino-5-azotoluene | chemical toxicant | | 4.12E-02 | [illegible] |
| REL/RELA/RELB | group | | 4.12E-02 | [illegible] |
| ibudilast | chemical drug | | 4.12E-02 | [illegible] |
| tectorigenin | chemical - endogenous non-mammalian | | 4.12E-02 | [illegible] |
| CYTOR | other | | 4.12E-02 | [illegible] |
| GPR84 | G-protein coupled receptor | | 4.12E-02 | [illegible] |
| PPT1 | enzyme | | 4.12E-02 | [illegible] |
| kremezin | chemical drug | | 4.12E-02 | [illegible] |
| SLC39A14 | transporter | | 4.12E-02 | [illegible] |
| IL-2R | complex | | 4.12E-02 | [illegible] |
| C-21 | chemical drug | | 4.12E-02 | [illegible] |
| PNU282987 | chemical reagent | | 4.12E-02 | [illegible] |
| S100A10 | other | | 4.12E-02 | [illegible] |
| hydrogel | chemical drug | | 4.12E-02 | [illegible] |
| AZI2 | other | | 4.12E-02 | [illegible] |
| LITAF | transcription regulator | | 4.12E-02 | [illegible] |
| HDAC10 | transcription regulator | | 4.12E-02 | [illegible] |
| BACE | enzyme | | 4.12E-02 | [illegible] |
| [illegible] | other | | 4.12E-02 | [illegible] |
| SLC25A4 | transporter | | 4.12E-02 | [illegible] |
| CD5L | transmembrane receptor | | 4.12E-02 | [illegible] |
| PLCD1 | enzyme | | 4.12E-02 | [illegible] |
| CYSLTR1 | G-protein coupled receptor | | 4.12E-02 | [illegible] |
| DNM1L | enzyme | | 4.12E-02 | [illegible] |
| EFEMP1 | enzyme | | 4.12E-02 | [illegible] |
| USP2 | peptidase | | 4.12E-02 | [illegible] |
| ARAF | kinase | | 4.12E-02 | [illegible] |
| DGKZ | kinase | | 4.12E-02 | [illegible] |
| SDC2 | other | | 4.12E-02 | [illegible] |
| RNF41 | enzyme | | 4.12E-02 | [illegible] |
| GHSR | G-protein coupled receptor | | 4.12E-02 | [illegible] |
| CLTC | other | | 4.12E-02 | [illegible] |
| MAP3K10 | kinase | | 4.12E-02 | [illegible] |
| TBL1X | transcription regulator | | 4.12E-02 | [illegible] |
| PLA2G5 | enzyme | | 4.12E-02 | [illegible] |
| [illegible] | other | | 4.12E-02 | [illegible] |
| PBX2 | transcription regulator | | 4.12E-02 | [illegible] |
| SLC19A1 | transporter | | 4.12E-02 | [illegible] |
| CAPN2 | peptidase | | 4.12E-02 | [illegible] |
| NCR3 | transmembrane receptor | | 4.12E-02 | [illegible] |
| SERPINH1 | other | | 4.12E-02 | [illegible] |
| TBL1XR1 | transcription regulator | | 4.12E-02 | [illegible] |
| L-lactic acid | chemical - endogenous mammalian | | 4.12E-02 | [illegible] |
| mycothiazole | chemical toxicant | | 4.12E-02 | [illegible] |
| latrunculin A | chemical toxicant | | 4.12E-02 | [illegible] |
| epoxyeicosatrienoic acid analog B | chemical reagent | | 4.12E-02 | [illegible] |
| epoxyeicosatrienoic acid analog A | chemical reagent | | 4.12E-02 | [illegible] |
| [illegible] | chemical drug | | 4.12E-02 | [illegible] |
| buserelin | biologic drug | | 4.12E-02 | [illegible] |
| dihematoporphyrin ether | chemical drug | | 4.12E-02 | [illegible] |
| miR-485-3p (and other miRNAs w/seed UCAUACA) | mature microRNA | Inhibited | 4.12E-02 | [illegible] |
| bisphenol A | chemical - endogenous mammalian | Activated | 4.14E-02 | [illegible] |
| miR-941 (miRNAs w/seed ACCCGGC) | mature microRNA | Inhibited | 4.14E-02 | [illegible] |
| ADORA2A | G-protein coupled receptor | | 4.16E-02 | [illegible] |
| miR-7848-3p (miRNAs w/seed UACCCUC) | mature microRNA | Inhibited | 4.18E-02 | [illegible] |
| Hif1 | complex | | 4.18E-02 | [illegible] |
| cadmium | chemical toxicant | Activated | 4.19E-02 | [illegible] |
| MTORC1 | complex | | 4.21E-02 | [illegible] |
| CD276 | enzyme | | 4.21E-02 | [illegible] |
| miR-30c-3p (and other miRNAs w/seed UGGGAUG) | mature microRNA | Inhibited | 4.21E-02 | [illegible] |
| miR-4435 (miRNAs w/seed UGGCCAG) | mature microRNA | Inhibited | 4.23E-02 | [illegible] |
| RORA | ligand-dependent nuclear receptor | | 4.23E-02 | [illegible] |
| miR-1287-5p (miRNAs w/seed GCUGGAU) | mature microRNA | Inhibited | 4.25E-02 | [illegible] |
| TFAP4 | transcription regulator | | 4.26E-02 | [illegible] |
| PRKN | enzyme | | 4.26E-02 | [illegible] |
| TNFRSF9 | transmembrane receptor | | 4.26E-02 | [illegible] |
| paricalcitol | chemical drug | | 4.26E-02 | [illegible] |
| miR-3921 (and other miRNAs w/seed CUCUGAG) | mature microRNA | Inhibited | 4.27E-02 | [illegible] |
| RUVBL1 | transcription regulator | | 4.27E-02 | [illegible] |
| Hbb-b2 | other | | 4.27E-02 | [illegible] |
| hexamethylene bisacetamide | chemical reagent | | 4.27E-02 | [illegible] |
| miR-5011-3p (and other miRNAs w/seed UGCAUGG) | mature microRNA | Inhibited | 4.28E-02 | [illegible] |
| NKX3-1 | transcription regulator | | 4.28E-02 | [illegible] |
| miR-6830-3p (miRNAs w/seed GUCUUUC) | mature microRNA | Inhibited | 4.29E-02 | [illegible] |
| miR-4758-3p (miRNAs w/seed GCCCCAC) | mature microRNA | Inhibited | 4.30E-02 | [illegible] |
| E2F3 | transcription regulator | | 4.30E-02 | [illegible] |
| miR-4699-5p (miRNAs w/seed GAAGAUU) | mature microRNA | Inhibited | 4.30E-02 | [illegible] |
| miR-3605-5p (and other miRNAs w/seed GAGGAUG) | mature microRNA | Inhibited | 4.30E-02 | [illegible] |
| miR-6772-5p (miRNAs w/seed GGGUGUA) | mature microRNA | Inhibited | 4.30E-02 | [illegible] |
| miR-6831-3p (miRNAs w/seed GACUAAC) | mature microRNA | Inhibited | 4.34E-02 | [illegible] |
| miR-3190-5p (miRNAs w/seed CUGGCCA) | mature microRNA | Inhibited | 4.38E-02 | [illegible] |
| miR-655-5p (miRNAs w/seed GAGGUUA) | mature microRNA | Inhibited | 4.42E-02 | [illegible] |
| miR-380-3p (and other miRNAs w/seed AUGUAGU) | mature microRNA | Inhibited | 4.44E-02 | [illegible] |
| miR-4765 (miRNAs w/seed GAGUGAU) | mature microRNA | Inhibited | 4.44E-02 | [illegible] |
| miR-383-3p (miRNAs w/seed CAGCACU) | mature microRNA | Inhibited | 4.44E-02 | [illegible] |
| SOX4 | transcription regulator | | 4.47E-02 | [illegible] |
| miR-320e (miRNAs w/seed AAGCUGG) | mature microRNA | Inhibited | 4.49E-02 | [illegible] |
| miR-1227-3p (miRNAs w/seed GUGCCAC) | mature microRNA | Inhibited | 4.50E-02 | [illegible] |
| miR-7154-5p (miRNAs w/seed UCAUGAA) | mature microRNA | Inhibited | 4.50E-02 | [illegible] |
| miR-8063 (miRNAs w/seed GGCGAUU) | mature microRNA | Inhibited | 4.51E-02 | [illegible] |
| miR-1231 (miRNAs w/seed UGUCUGG) | mature microRNA | Inhibited | 4.51E-02 | [illegible] |
| [illegible] | other | Activated | 4.51E-02 | [illegible] |
| RAG2 | enzyme | | 4.51E-02 | [illegible] |
| SPRY1 | other | | 4.51E-02 | [illegible] |
| curdlan | chemical - endogenous non-mammalian | | 4.51E-02 | [illegible] |
| miR-4703-3p (miRNAs w/seed GUAGUUG) | mature microRNA | Inhibited | 4.56E-02 | [illegible] |
| miR-6881-3p (miRNAs w/seed UCCUCUU) | mature microRNA | Inhibited | 4.56E-02 | [illegible] |
| tetracycline | chemical drug | | 4.60E-02 | [illegible] |
| PLA2G10 | enzyme | | 4.60E-02 | [illegible] |
| ETV5 | transcription regulator | | 4.60E-02 | [illegible] |
| miR-140-3p (and other miRNAs w/seed ACCACAG) | mature microRNA | Inhibited | 4.62E-02 | [illegible] |
| miR-550a-3-5p (and other miRNAs w/seed UGUGCCU) | mature microRNA | Inhibited | 4.63E-02 | [illegible] |
| miR-6501-3p (miRNAs w/seed CAGAGCA) | mature microRNA | Inhibited | 4.64E-02 | [illegible] |
| miR-339-5p (and other miRNAs w/seed CCCUGUC) | mature microRNA | Inhibited | 4.68E-02 | [illegible] |
| miR-337-3p (and other miRNAs w/seed CAGCUCC) | mature microRNA | Inhibited | 4.68E-02 | [illegible] |
| miR-3158-3p (miRNAs w/seed AGGGCUU) | mature microRNA | Inhibited | 4.69E-02 | [illegible] |
| miR-425-3p (miRNAs w/seed UCGGGAA) | mature microRNA | Inhibited | 4.69E-02 | [illegible] |
| miR-203a-5p (and other miRNAs w/seed GUGGUUC) | mature microRNA | Inhibited | 4.70E-02 | [illegible] |
| miR-6809-5p (and other miRNAs w/seed AGAGGAA) | mature microRNA | Inhibited | 4.70E-02 | [illegible] |
| miR-6126 (miRNAs w/seed CAUUAUA) | mature microRNA | Inhibited | 4.71E-02 | [illegible] |
| miR-6864-3p (miRNAs w/seed AAAUGGA) | mature microRNA | Inhibited | 4.74E-02 | [illegible] |
| SB-431542 | chemical reagent | | 4.75E-02 | [illegible] |
| miR-6878-5p (miRNAs w/seed GGGAGAA) | mature microRNA | Inhibited | 4.76E-02 | [illegible] |
| miR-194-5p (miRNAs w/seed GUAACAG) | mature microRNA | Inhibited | 4.76E-02 | [illegible] |
| SPIB | transcription regulator | Activated | 4.77E-02 | [illegible] |
| fluoxetine | chemical drug | | 4.77E-02 | [illegible] |
| SETDB1 | enzyme | | 4.78E-02 | [illegible] |
| ITGAV | transmembrane receptor | | 4.78E-02 | [illegible] |
| NPPB | other | | 4.78E-02 | [illegible] |
| SSRP1 | transcription regulator | | 4.78E-02 | [illegible] |
| GDF11 | growth factor | | 4.78E-02 | [illegible] |
| vitamin A | chemical - endogenous mammalian | | 4.78E-02 | [illegible] |
| miR-3606-5p (miRNAs w/seed UAGUGAA) | mature microRNA | Inhibited | 4.78E-02 | [illegible] |
| miR-5581-3p (miRNAs w/seed UCCAUGC) | mature microRNA | Inhibited | 4.78E-02 | [illegible] |
| miR-7109-3p (miRNAs w/seed AAGCCUC) | mature microRNA | Inhibited | 4.78E-02 | [illegible] |
| miR-6869 (miRNAs w/seed GUGUCCG) | mature microRNA | Inhibited | 4.80E-02 | [illegible] |
| miR-7058-5p (and other miRNAs w/seed GUGGAGG) | mature microRNA | Inhibited | 4.80E-02 | [illegible] |
| miR-3160-5p (miRNAs w/seed GCUUUCU) | mature microRNA | Inhibited | 4.80E-02 | [illegible] |
| cyclooxygenase | group | Activated | 4.82E-02 | [illegible] |
| STAP2 | other | Activated | 4.82E-02 | [illegible] |
| mir-218 | microRNA | | 4.82E-02 | [illegible] |
| KLF10 | transcription regulator | | 4.82E-02 | [illegible] |
| GAS5 | other | | 4.82E-02 | [illegible] |
| AURKB | kinase | Inhibited | 4.82E-02 | [illegible] |
| PKNOX1 | transcription regulator | | 4.82E-02 | [illegible] |
| LCP2 | other | | 4.82E-02 | [illegible] |
| STAU1 | transporter | | 4.82E-02 | [illegible] |
| BID | other | | 4.82E-02 | [illegible] |
| HULC | other | | 4.82E-02 | [illegible] |
| acrolein | chemical toxicant | | 4.82E-02 | [illegible] |
| zymosan A | chemical - endogenous non-mammalian | Activated | 4.82E-02 | [illegible] |
| biochanin A | chemical toxicant | Inhibited | 4.82E-02 | [illegible] |
| miR-3064-5p (and other miRNAs w/seed CUGGCUG) | mature microRNA | Inhibited | 4.82E-02 | [illegible] |
| TCF7 | transcription regulator | | 4.82E-02 | [illegible] |
| ZBTB7B | transcription regulator | | 4.82E-02 | [illegible] |
| miR-576-3p (miRNAs w/seed AGAUGUG) | mature microRNA | Inhibited | 4.84E-02 | [illegible] |
| miR-516b-5p (miRNAs w/seed UCUGGAG) | mature microRNA | Inhibited | 4.84E-02 | [illegible] |
| miR-6883-5p (miRNAs w/seed GAACUCU) | mature microRNA | Inhibited | 4.86E-02 | [illegible] |
| [illegible] | chemical - endogenous mammalian | | 4.86E-02 | [illegible] |
| Ginkgo biloba | chemical drug | | 4.86E-02 | [illegible] |
| FFAR4 | G-protein coupled receptor | | 4.86E-02 | [illegible] |
| SGPP1 | phosphatase | Inhibited | 4.86E-02 | [illegible] |
| TRAF3IP3 | other | | 4.86E-02 | [illegible] |
| FH | enzyme | | 4.86E-02 | [illegible] |
| TNFAIP8L2 | other | | 4.86E-02 | [illegible] |
| elastase | group | | 4.86E-02 | [illegible] |
| IDH2 | enzyme | | 4.86E-02 | [illegible] |
| PECAM1 | other | | 4.86E-02 | [illegible] |
| APOA4 | transporter | | 4.86E-02 | [illegible] |
| CBLB | enzyme | | 4.86E-02 | [illegible] |
| CBX2 | transcription regulator | | 4.86E-02 | [illegible] |
| XRCC6 | enzyme | | 4.86E-02 | [illegible] |
| MGAT3 | enzyme | | 4.86E-02 | [illegible] |
| TRAF4 | other | | 4.86E-02 | [illegible] |
| NHLH2 | transcription regulator | | 4.86E-02 | [illegible] |
| LAMC1 | other | | 4.86E-02 | [illegible] |
| NPFF | other | | 4.86E-02 | [illegible] |
| GRIN1 | ion channel | | 4.86E-02 | [illegible] |
| HNRNPD | transcription regulator | | 4.86E-02 | [illegible] |
| DMH1 | chemical reagent | | 4.86E-02 | [illegible] |
| CpdX-D3 | chemical reagent | | 4.86E-02 | [illegible] |
| diacylglycerol | chemical - endogenous mammalian | | 4.86E-02 | [illegible] |
| E64d | chemical - protease inhibitor | | 4.86E-02 | [illegible] |
| N-acetylglucosamine | chemical - endogenous mammalian | | 4.86E-02 | [illegible] |
| manganese | chemical - endogenous mammalian | | 4.86E-02 | [illegible] |
| USP22 | peptidase | | 4.86E-02 | [illegible] |
| LATS2 | kinase | | 4.86E-02 | [illegible] |
| anandamide | chemical - endogenous mammalian | | 4.86E-02 | [illegible] |
| miR-210-5p (and other miRNAs w/seed GCCACUG) | mature microRNA | Inhibited | 4.86E-02 | [illegible] |
| miR-147-5p (and other miRNAs w/seed GGAAACA) | mature microRNA | Inhibited | 4.86E-02 | [illegible] |
| miR-3166 (and other miRNAs w/seed GUGACUU) | mature microRNA | Inhibited | 4.87E-02 | [illegible] |
| miR-1973 (miRNAs w/seed CCGUGCA) | mature microRNA | Inhibited | 4.87E-02 | [illegible] |
| miR-6035 (miRNAs w/seed UGGACCU) | mature microRNA | Inhibited | 4.88E-02 | [illegible] |
| miR-132-5p (miRNAs w/seed CCGUGGC) | mature microRNA | Inhibited | 4.88E-02 | [illegible] |
| miR-6918-5p (and other miRNAs w/seed GCUGAGG) | mature microRNA | Inhibited | 4.88E-02 | [illegible] |
| miR-4786-5p (miRNAs w/seed GAGACCA) | mature microRNA | Inhibited | 4.88E-02 | [illegible] |
| miR-326-3p (and other miRNAs w/seed CACUGCC) | mature microRNA | Inhibited | 4.90E-02 | [illegible] |
| miR-6768-5p (miRNAs w/seed ACACAGG) | mature microRNA | Inhibited | 4.98E-02 | [illegible] |
| miR-6737-5p (and other miRNAs w/seed UGGGGUG) | mature microRNA | Inhibited | 4.98E-02 | [illegible] |

**SupplementaryTable S15. List of Upstream Regulators for the condition "Caco-2_SARS-CoV-2".** The IPA-based Core Analysis was performed on the basis of differentially expressedgenes dissected for a given conditionbyGENEVESTIGATOR-basedanalysis.Only statisticallysignificant UpstreamRegulators are included (n = 3639). The data are taken as the basis for R-based visualization by Venn diagrams shown in Figure 5.

| Upstream Regulator | Molecule Type | Predicted Activation State | p-value of overlap | Target Molecules in Dataset |
|---|---|---|---|---|
| PDGF BB | complex | Activated | 6,53E-28 | [illegible] |
| TNF | cytokine | Activated | 1,59E-24 | [illegible] |
| PD98059 | chemical - kinase inhibitor | Inhibited | 2,58E-24 | [illegible] |
| U0126 | chemical - kinase inhibitor | Inhibited | 6,32E-24 | [illegible] |
| beta-estradiol | chemical - endogenous mammalian | Activated | 1,94E-23 | [illegible] |
| ERK | group | Activated | 4,46E-23 | [illegible] |
| IRAK4 | kinase | | 1,02E-22 | ATF3,BIRC3,CSRNP1,CXCL2,EGR1,ERRFI1,FOS,GEM,IER2,JUN,NFKBIA,NFKBIZ,PHLDA1,SLC25A25,TNFAIP3,ZC3H12A,ZFP36 |
| HGF | growth factor | Activated | 1,33E-22 | [illegible] |
| dexamethasone | chemical drug | | 4,05E-22 | [illegible] |
| LY294002 | chemical - kinase inhibitor | Inhibited | 7,88E-22 | [illegible] |
| EGF | growth factor | Activated | 1,57E-21 | [illegible] |
| NR3C1 | ligand-dependent nuclear receptor | | 2,18E-21 | [illegible] |
| NFkB (complex) | complex | Activated | 2,00E-20 | [illegible] |
| IL1B | cytokine | Activated | 5,16E-20 | [illegible] |
| RELA | transcription regulator | Activated | 7,53E-20 | [illegible] |
| GPER1 | G-protein coupled receptor | Activated | 1,17E-19 | ATF3,BHLHE40,CCND1,DUSP1,EGR1,ERRFI1,FOS,IER2,JUN,MYC,NFKBIA,PPP1R15A,TNFAIP3,ZFP36 |
| triamcinolone acetonide | chemical drug | Inhibited | 3,27E-19 | [illegible] |
| Mek | group | Activated | 1,74E-18 | [illegible] |
| F2 | peptidase | Activated | 2,72E-18 | [illegible] |
| forskolin | chemical toxicant | Activated | 9,57E-18 | [illegible] |
| 4-hydroxytamoxifen | chemical drug | Activated | 1,35E-17 | ATF3,BHLHE40,CCND1,CYP1A1,DUSP1,EGR1,ERRFI1,FOS,IER2,IER3,JUN,JUNB,MYC,NFKBIA,PDZK1,PPP1R15A,TNFAIP3,ZFP36 |
| IGF1 | growth factor | Activated | 1,37E-17 | [illegible] |
| E. coli B5 lipopolysaccharide | chemical - endogenous non-mammalian | Activated | 2,66E-17 | [illegible] |
| paclitaxel | chemical drug | | 4,06E-17 | [illegible] |
| trovafloxacin | chemical drug | Activated | 6,85E-17 | BHLHE40,BIRC3,CXCL2,ERRFI1,FOS,ICAM1,JUNB,LDLR,MDM2,NFKBIA,PHLDA1,SERPINE1,TNFAIP3 |
| HRAS | enzyme | | 7,93E-17 | [illegible] |
| TP53 | transcription regulator | Activated | 1,41E-16 | [illegible] |
| GnRH-A | chemical reagent | Activated | 1,52E-16 | [illegible] |
| IL1A | cytokine | Activated | 2,06E-16 | [illegible] |
| cycloheximide | chemical reagent | | 2,17E-16 | [illegible] |
| RAF1 | kinase | Activated | 3,37E-16 | [illegible] |
| lysophosphatidic acid | chemical - other | Activated | 3,63E-16 | [illegible] |
| JUN | transcription regulator | | 4,73E-16 | [illegible] |
| SP600125 | chemical - kinase inhibitor | Inhibited | 4,84E-16 | [illegible] |
| NRG1 | growth factor | Activated | 9,15E-16 | [illegible] |
| CREB1 | transcription regulator | Activated | 1,24E-15 | [illegible] |
| IL2 | cytokine | Activated | 1,56E-15 | [illegible] |
| actinomycin D | biologic drug | | 1,70E-15 | [illegible] |
| tetradecanoylphorbol acetate | chemical drug | Activated | 1,87E-15 | [illegible] |
| AGT | growth factor | Activated | 2,90E-15 | [illegible] |
| TP63 | transcription regulator | Activated | 3,05E-15 | [illegible] |
| Raf | group | | 3,76E-15 | [illegible] |
| lipopolysaccharide | chemical drug | Activated | 4,19E-15 | [illegible] |
| TGFB1 | growth factor | Activated | 6,00E-15 | [illegible] |
| OSM | cytokine | Activated | 8,90E-15 | [illegible] |
| poly rI:rC-RNA | biologic drug | Activated | 1,06E-14 | [illegible] |
| hydrogen peroxide | chemical - endogenous mammalian | Activated | 1,15E-14 | [illegible] |
| FOS | transcription regulator | | 1,58E-14 | [illegible] |
| thapsigargin | chemical toxicant | Activated | 2,52E-14 | [illegible] |
| mifepristone | chemical drug | | 2,66E-14 | [illegible] |
| IL6 | cytokine | Activated | 3,16E-14 | [illegible] |
| IGF2 | growth factor | | 3,98E-14 | [illegible] |
| ERBB2 | kinase | Activated | 4,40E-14 | [illegible] |
| FADD | other | | 8,01E-14 | [illegible] |
| IFNG | cytokine | Activated | 9,14E-14 | [illegible] |
| ERK1/2 | group | Activated | 1,28E-13 | [illegible] |
| methylprednisolone | chemical drug | | 1,35E-13 | [illegible] |
| MYC | transcription regulator | | 1,38E-13 | [illegible] |
| curcumin | chemical drug | Inhibited | 1,92E-13 | [illegible] |
| Ca2+ | chemical - endogenous mammalian | Activated | 1,93E-13 | [illegible] |
| CREM | transcription regulator | | 2,13E-13 | [illegible] |
| PRKCD | kinase | Activated | 2,23E-13 | [illegible] |
| Growth hormone | group | Activated | 2,72E-13 | [illegible] |
| FGF2 | growth factor | Activated | 2,91E-13 | [illegible] |
| MAP3K1 | kinase | Activated | 4,11E-13 | [illegible] |
| N-acetyl-L-cysteine | chemical drug | Inhibited | 4,30E-13 | [illegible] |
| anisomycin | chemical - endogenous non-mammalian | Activated | 5,00E-13 | [illegible] |
| tretinoin | chemical - endogenous mammalian | | 5,38E-13 | [illegible] |
| IKBKB | kinase | | 5,80E-13 | [illegible] |
| deferoxamine | chemical drug | Activated | 6,73E-13 | [illegible] |
| NFKB1 | transcription regulator | Activated | 6,85E-13 | [illegible] |
| FOXO3 | transcription regulator | | 7,23E-13 | [illegible] |
| bicuculline | chemical - endogenous non-mammalian | Activated | 7,32E-13 | [illegible] |
| MAPK8 | kinase | Activated | 8,97E-13 | [illegible] |
| TGFA | growth factor | Activated | 1,09E-12 | [illegible] |
| SMARCA4 | transcription regulator | | 1,12E-12 | [illegible] |
| Tgf beta | group | Activated | 1,12E-12 | [illegible] |
| estrogen receptor | group | | 1,16E-12 | [illegible] |
| progesterone | chemical - endogenous mammalian | | 1,19E-12 | [illegible] |
| FSH | complex | Activated | 1,22E-12 | [illegible] |
| dalfampridine | chemical drug | Activated | 1,26E-12 | [illegible] |
| Jnk | group | Activated | 1,34E-12 | [illegible] |
| Vegf | group | Activated | 1,48E-12 | [illegible] |
| Pkc(s) | group | Activated | 1,87E-12 | [illegible] |
| tunicamycin | chemical - endogenous non-mammalian | | 1,99E-12 | [illegible] |
| L-triiodothyronine | chemical - endogenous mammalian | | 2,05E-12 | [illegible] |
| prostaglandin E2 | chemical - endogenous mammalian | Activated | 2,98E-12 | [illegible] |
| epigallocatechin-gallate | chemical drug | | 3,33E-12 | [illegible] |
| P38 MAPK | group | Activated | 3,73E-12 | [illegible] |
| salmonella minnesota R595 lipopolysaccharides | chemical - endogenous non-mammalian | Activated | 3,91E-12 | [illegible] |
| INSR | kinase | Activated | 4,10E-12 | [illegible] |
| CEBPB | transcription regulator | | 5,01E-12 | [illegible] |
| ESR1 | ligand-dependent nuclear receptor | | 5,85E-12 | [illegible] |
| NFKBIA | transcription regulator | | 5,87E-12 | [illegible] |
| kainic acid | chemical toxicant | Activated | 6,22E-12 | [illegible] |
| EGFR | kinase | Activated | 6,36E-12 | [illegible] |
| INS | other | Activated | 7,88E-12 | [illegible] |
| SMAD4 | transcription regulator | | 8,73E-12 | [illegible] |
| Pdgf (complex) | complex | Activated | 1,13E-11 | [illegible] |
| Sb202190 | chemical - kinase inhibitor | Inhibited | 1,21E-11 | [illegible] |
| PI3K (complex) | complex | | 1,22E-11 | [illegible] |
| gentamicin | chemical drug | Activated | 1,24E-11 | [illegible] |
| cisplatin | chemical drug | | 1,41E-11 | [illegible] |
| resveratrol | chemical drug | | 1,49E-11 | [illegible] |
| bucladesine | chemical toxicant | Activated | 1,69E-11 | [illegible] |
| ATF3 | transcription regulator | | 1,75E-11 | [illegible] |
| D-glucose | chemical - endogenous mammalian | Activated | 1,90E-11 | [illegible] |
| ADRB | group | | 2,63E-11 | [illegible] |
| S100A8 | other | | 2,97E-11 | [illegible] |
| CG | complex | Activated | 3,09E-11 | [illegible] |
| 2-amino-5-phosphonovaleric acid | chemical - other | Inhibited | 3,11E-11 | [illegible] |
| MYD88 | other | Activated | 3,84E-11 | [illegible] |
| romidepsin | biologic drug | | 4,12E-11 | [illegible] |
| NDRG1 | kinase | | 4,12E-11 | [illegible] |
| NGF | growth factor | | 4,87E-11 | [illegible] |
| PDX1 | transcription regulator | | 5,12E-11 | [illegible] |
| arsenite | chemical toxicant | | 5,23E-11 | [illegible] |
| RAC1 | enzyme | Activated | 5,75E-11 | [illegible] |
| doxorubicin | chemical drug | Activated | 5,80E-11 | [illegible] |
| EGR1 | transcription regulator | | 5,84E-11 | [illegible] |
| F7 | peptidase | | 6,34E-11 | [illegible] |
| SP1 | transcription regulator | Activated | 6,36E-11 | [illegible] |
| CREBBP | transcription regulator | | 7,89E-11 | [illegible] |
| Alpha catenin | group | | 8,30E-11 | [illegible] |
| estrogen | chemical drug | | 8,97E-11 | [illegible] |
| ZFP36 | transcription regulator | Inhibited | 9,28E-11 | [illegible] |
| FOXL2 | transcription regulator | Activated | 9,98E-11 | [illegible] |
| nitric oxide | chemical - endogenous mammalian | | 1,07E-10 | [illegible] |
| PRKCA | kinase | Activated | 1,08E-10 | [illegible] |
| SRC | kinase | Activated | 1,08E-10 | [illegible] |
| ethanol | chemical - endogenous mammalian | | 1,16E-10 | [illegible] |
| CXCL12 | cytokine | Activated | 1,18E-10 | [illegible] |
| Mt1 | other | | 1,18E-10 | [illegible] |
| MAPK9 | kinase | | 1,43E-10 | [illegible] |
| calphostin C | chemical - kinase inhibitor | Inhibited | 1,52E-10 | [illegible] |
| calcitriol | chemical drug | | 1,57E-10 | [illegible] |
| trichostatin A | chemical drug | | 1,71E-10 | [illegible] |
| CDK19 | kinase | | 1,77E-10 | [illegible] |
| EIF2AK2 | kinase | | 2,17E-10 | [illegible] |
| CHUK | kinase | Activated | 2,27E-10 | [illegible] |
| simvastatin | chemical drug | | 2,41E-10 | [illegible] |
| Ins1 | other | Activated | 2,60E-10 | [illegible] |
| SP3 | transcription regulator | | 2,70E-10 | [illegible] |
| TLR7 | transmembrane receptor | Activated | 2,86E-10 | [illegible] |
| Ap1 | complex | Activated | 3,04E-10 | [illegible] |
| STAT3 | transcription regulator | | 3,20E-10 | [illegible] |
| IgG | complex | | 3,39E-10 | [illegible] |
| LDL | complex | Activated | 3,47E-10 | [illegible] |
| FAS | transmembrane receptor | Inhibited | 3,77E-10 | [illegible] |
| TNFSF11 | cytokine | Activated | 3,82E-10 | [illegible] |
| arachidonic acid | chemical - endogenous mammalian | Activated | 3,90E-10 | [illegible] |
| EP300 | transcription regulator | | 4,48E-10 | [illegible] |
| glutamine | chemical - endogenous mammalian | | 4,71E-10 | [illegible] |
| carrageenan | chemical drug | Activated | 4,74E-10 | [illegible] |
| SMAD3 | transcription regulator | Activated | 4,86E-10 | [illegible] |
| PRKCE | kinase | Activated | 5,36E-10 | [illegible] |
| MAPK1 | kinase | Activated | 5,52E-10 | [illegible] |
| APOE | transporter | | 5,64E-10 | [illegible] |
| EIF2AK3 | kinase | Activated | 5,66E-10 | [illegible] |
| TERT | enzyme | | 5,87E-10 | [illegible] |
| MAP2K1/2 | group | Activated | 6,02E-10 | [illegible] |
| 15-deoxy-delta-12,14 -PGJ 2 | chemical - endogenous mammalian | | 6,13E-10 | [illegible] |
| MMP1 | peptidase | Activated | 6,50E-10 | [illegible] |
| DETA-NONOate | chemical reagent | | 6,50E-10 | [illegible] |
| EPAS1 | transcription regulator | | 6,86E-10 | [illegible] |
| leukotriene D4 | chemical - endogenous mammalian | Activated | 7,57E-10 | [illegible] |
| CDK9 | kinase | | 7,57E-10 | [illegible] |
| HMGA1 | transcription regulator | Activated | 7,86E-10 | [illegible] |
| SU6656 | chemical toxicant | Inhibited | 8,64E-10 | [illegible] |
| Z-LLL-CHO | chemical - protease inhibitor | | 8,85E-10 | [illegible] |
| REL | transcription regulator | Activated | 8,90E-10 | [illegible] |
| CD40LG | cytokine | Activated | 8,90E-10 | [illegible] |
| dihydrotestosterone | chemical - endogenous mammalian | | 9,64E-10 | [illegible] |
| PP2/AG1879 tyrosine kinase inhibitor | chemical - kinase inhibitor | | 1,05E-09 | [illegible] |
| CSF2 | cytokine | Activated | 1,09E-09 | [illegible] |
| MAP2K4 | kinase | Activated | 1,17E-09 | [illegible] |
| SB203580 | chemical - kinase inhibitor | Inhibited | 1,30E-09 | [illegible] |
| AKT1 | kinase | Activated | 1,44E-09 | [illegible] |
| SOX2 | transcription regulator | | 1,47E-09 | [illegible] |
| EDN1 | cytokine | Activated | 1,49E-09 | [illegible] |
| NEDD9 | other | | 1,60E-09 | [illegible] |
| ESR2 | ligand-dependent nuclear receptor | | 1,63E-09 | [illegible] |
| wortmannin | chemical - kinase inhibitor | Inhibited | 1,76E-09 | [illegible] |
| COL18A1 | other | Inhibited | 1,77E-09 | [illegible] |
| thioacetamide | chemical toxicant | | 1,87E-09 | [illegible] |
| Insulin | group | Activated | 2,14E-09 | [illegible] |
| RASSF1 | other | | 2,17E-09 | [illegible] |
| GNAQ | enzyme | | 2,18E-09 | [illegible] |
| EPHB1 | kinase | Activated | 2,29E-09 | [illegible] |
| BCL3 | transcription regulator | | 2,32E-09 | [illegible] |
| C5 | cytokine | Activated | 2,36E-09 | [illegible] |
| KRAS | enzyme | | 2,49E-09 | [illegible] |
| sphingosine-1-phosphate | chemical - endogenous mammalian | Activated | 2,63E-09 | [illegible] |
| Hdac | group | Inhibited | 2,64E-09 | [illegible] |
| PLAUR | transmembrane receptor | | 2,81E-09 | [illegible] |
| AGTR1 | G-protein coupled receptor | | 2,81E-09 | [illegible] |
| S100A9 | other | | 3,18E-09 | [illegible] |
| raloxifene | chemical drug | | 3,39E-09 | [illegible] |
| Mt2 | other | Inhibited | 3,40E-09 | [illegible] |
| cyclic AMP | chemical - endogenous mammalian | | 3,76E-09 | [illegible] |
| HDAC3 | transcription regulator | | 3,84E-09 | [illegible] |
| HIF1A | transcription regulator | | 3,85E-09 | [illegible] |
| Ngf | group | | 4,32E-09 | [illegible] |
| carbon tetrachloride | chemical toxicant | | 4,59E-09 | [illegible] |
| F3 | transmembrane receptor | Activated | 4,87E-09 | [illegible] |
| troglitazone | chemical drug | | 4,92E-09 | [illegible] |
| RNA polymerase II | complex | | 5,12E-09 | [illegible] |
| trans-hydroxytamoxifen | chemical drug | | 6,15E-09 | [illegible] |
| THPO | cytokine | | 6,56E-09 | [illegible] |
| F2R | G-protein coupled receptor | Activated | 6,56E-09 | [illegible] |
| ITGB1 | transmembrane receptor | | 6,58E-09 | [illegible] |
| vorinostat | chemical drug | | 6,93E-09 | [illegible] |
| palmitic acid | chemical - endogenous mammalian | | 7,18E-09 | [illegible] |
| HDAC1 | transcription regulator | | 7,22E-09 | [illegible] |
| EPCAM | other | | 7,37E-09 | [illegible] |
| HDAC6 | transcription regulator | Activated | 7,64E-09 | [illegible] |
| thyroid hormone | chemical - endogenous mammalian | | 7,73E-09 | [illegible] |
| TICAM1 | other | Activated | 8,64E-09 | [illegible] |
| triptolide | chemical drug | Inhibited | 8,64E-09 | [illegible] |
| 5-fluorouracil | chemical drug | Activated | 8,65E-09 | [illegible] |
| herbimycin | chemical - kinase inhibitor | Inhibited | 8,91E-09 | [illegible] |
| camptothecin | chemical drug | Activated | 9,01E-09 | [illegible] |
| 5-N-ethylcarboxamido adenosine | chemical reagent | | 9,20E-09 | [illegible] |
| STAT5A | transcription regulator | | 9,67E-09 | [illegible] |
| TP73 | transcription regulator | | 1,03E-08 | [illegible] |
| PAF1 | other | Activated | 1,03E-08 | [illegible] |
| MAPK14 | kinase | | 1,08E-08 | [illegible] |
| NCOA1 | transcription regulator | | 1,12E-08 | [illegible] |
| LEP | growth factor | | 1,16E-08 | [illegible] |
| methylnitronitrosoguanidine | chemical toxicant | Activated | 1,17E-08 | [illegible] |
| FOSL1 | transcription regulator | | 1,18E-08 | [illegible] |
| cocaine | chemical drug | Activated | 1,27E-08 | [illegible] |
| Tnf (family) | group | Activated | 1,28E-08 | [illegible] |
| MAP2K1 | kinase | Activated | 1,34E-08 | [illegible] |
| SERCA | group | | 1,40E-08 | [illegible] |
| 6,7-dinitroquinoxaline-2,3-dione | chemical reagent | | 1,40E-08 | [illegible] |
| 6-cyano-7-nitroquinoxaline-2,3-dione | chemical reagent | | 1,40E-08 | [illegible] |
| PTEN | phosphatase | | 1,44E-08 | [illegible] |

| | | | | |
|---|---|---|---|---|
| KLF6 | transcription regulator | | 1,45E-08 | ATF3,CXCL2,CYP1A1,MYC,NFKBIA,NR1D1,PLAU,TNFAIP3 |
| rottlerin | chemical toxicant | | 1,45E-08 | BIRC3,CCND1,ICAM1,JUN,MDM2,RGS2,TNFRSF10B,ZFP36 |
| fenretinide | chemical drug | | 1,45E-08 | ANKRD1,ATF3,CCND1,JUN,MYC,NFKBIA,PPP1R15A,TNFRSF10B |
| Gsk3 | group | | 1,60E-08 | CCND1,FOS,ICAM1,JUN,LDLR,MYC,TNFAIP3,TNFRSF10B |
| APP | other | | 1,60E-08 | ARRDC3,AXL,CCND1,CTSV,CXCL2,DYRK1A,EGR1,FOS,H2BC5,ICAM1,ITGA2,ITGA6,JUN,JUNB,LDLR,MAFF,MYC,NR1D1,PLAU,SERPINE1,TNFRSF10B,TTR |
| PGR | ligand-dependent nuclear receptor | | 1,63E-08 | AKR1C3,BIRC3,CCND1,FOS,ICAM1,IER3,ITGA6,MYC,NDRG1,NFKBIA,PLAU,PLK2,TIPARP |
| cytokine | group | Activated | 1,65E-08 | CCND1,DUSP1,EGR1,FOS,ICAM1,ITGA6,JUN,MYC,PLAU,SERPINE1 |
| NRAS | enzyme | | 1,65E-08 | AKAP12,ANKRD1,CCND1,CXCL2,DUSP6,EGR1,ETS1,ITGA2,PLAU,RHOB,SPRY4 |
| crocidolite asbestos | chemical toxicant | | 1,80E-08 | CCND1,CXCL2,FOS,JUN,JUNB,MYC |
| DUSP1 | phosphatase | | 1,87E-08 | CXCL2,DUSP1,DUSP6,ICAM1,IER3,JUN,MXD1,SERPINE1,ZFP36 |
| PPARG | ligand-dependent nuclear receptor | | 1,94E-08 | AGT,CCND1,CXCL2,ETS1,FABP1,HIVEP2,ICAM1,JUN,KLF6,LDLR,NDRG1,NR1D1,PMM1,RHOB,SERPINE1,VIM |
| platelet activating factor | chemical - endogenous mammalian | | 2,08E-08 | EGR1,FOS,ICAM1,NFKBIA,PLAU,SERPINE1,VIM |
| H1-2 | other | | 2,12E-08 | CCND1,IER3,MDM2,MYC,PHLDA2 |
| collagenase | group | Activated | 2,21E-08 | CXCL2,EGR1,ICAM1,IER3,JUN,JUNB |
| etoposide | chemical drug | | 2,24E-08 | ATF3,BIRC3,CCND1,DUSP1,ICAM1,IER3,JUN,MDM2,MYC,TNFAIP3,TNFRSF10B |
| DDIT3 | transcription regulator | | 2,35E-08 | ANKRD1,ATF3,ITGA2,NDRG1,PER1,PPP1R15A,TNFRSF10B,TXNIP,VIM |
| mycophenolic acid | chemical drug | | 2,60E-08 | AKAP12,ATF3,BIRC3,DUSP1,ETS1,LDLR,SERPINE1,TNFAIP3 |
| Fgf | group | | 2,68E-08 | CCND1,FOS,JUN,JUNB,PER1,SPRY4 |
| Cdk | group | Activated | 2,68E-08 | CXCL2,DUSP1,MYC,PHLDA1,RGS2,TXNIP |
| atorvastatin | chemical drug | | 2,74E-08 | DUSP1,EGR1,FOS,ICAM1,IER3,JUN,JUNB,LDLR,RHOB,SERPINE1,ZFP36 |
| amphetamine | chemical drug | | 2,85E-08 | ATF3,EGR1,FOS,ICAM1,JUN,JUNB,RGS2,TNFAIP3 |
| TLR3 | transmembrane receptor | Activated | 3,04E-08 | ATF3,CXCL2,DUSP1,FOS,ICAM1,IER3,JUNB,NFKBIZ,PHLDA1,PLK2,RHOB,SERPINE1 |
| MAP2K7 | kinase | | 3,07E-08 | ATF3,BIRC3,CCND1,JUNB,MYC,PLAU,RHOB |
| luteolin | chemical drug | | 3,07E-08 | CCND1,CYP1A1,FOS,ICAM1,JUN,MYC,TNFRSF10B |
| NFYA | transcription regulator | Activated | 3,13E-08 | CYP1A1,EGR1,ICAM1,JUN,JUNB,PPP1R15A,RHOB,TXNIP |
| MAPK3 | kinase | Activated | 3,13E-08 | CCND1,EGR1,FOS,JUN,JUNB,MYC,RGS2,TNFRSF10B |
| tamoxifen | chemical drug | | 3,22E-08 | CCND1,FABP1,FOS,IER3,IGFBP4,JUN,JUNB,LDLR,MAFF,MDM2,MYC,PDZK1,SERPINA6 |
| AHR | ligand-dependent nuclear receptor | | 3,33E-08 | CCND1,CYP1A1,DUSP6,FOS,HDAC9,ITGA6,JUN,JUNB,MYC,PMM1,PPP1R15A,SERPINE1,TIPARP,VIM |
| cyclosporin A | biologic drug | | 3,53E-08 | AGT,ATF3,BIRC3,CCND1,EGR1,FOS,ICAM1,IER3,JUN,LDLR,MYC,NFKBIA,SERPINE1,TSPAN8,VIM |
| Akt | group | | 3,55E-08 | ANKRD1,BHLHE40,CCND1,ETS1,FOS,IER3,JUN,MDM2,MYC,NDRG1,NFKBIA,RHOB,SERPINE1,TIPARP |
| FGF1 | growth factor | | 3,86E-08 | AFP,CCND1,DUSP6,EGR1,FOS,ICAM1,JUN,MYC,TTR |
| IL17A | cytokine | Activated | 3,89E-08 | CCND1,CXCL2,FOS,ICAM1,JUN,MYC,NFKBIZ,PI3,PLAU,TNFAIP3,VIM,ZC3H12A |
| NGFR | transmembrane receptor | | 3,90E-08 | CCND1,FOS,JUN,LDLR,RHOB,SERPINE1 |
| PRKCB | kinase | Activated | 3,92E-08 | CCND1,EGR1,FOS,ICAM1,JUN,MYC,SERPINE1 |
| Salmonella enterica serotype abortus equi lipopolysaccharide | chemical toxicant | Activated | 4,00E-08 | ABL2,BIRC3,CXCL2,EGR1,GEM,MAFF,NFKBIZ,PHLDA1,PHLDA2,PLAU |
| IL4 | cytokine | Activated | 4,11E-08 | BHLHE40,CCND1,CXCL2,EPHA2,FOS,ICAM1,IER2,IER3,IGFBP4,ITGA2,JUN,JUNB,KLF6,MYC,NDRG1,NFKBIA,PLAU,SERPINE1,SLC25A25,VIM,ZFP36,ZNF608 |
| indirubin | chemical drug | | 4,12E-08 | BIRC3,CCND1,CYP1A1,JUN,MYC |
| LIF | cytokine | | 4,22E-08 | AFP,AHSG,CCND1,CXCL2,EGR1,FGG,FOS,JUN,JUNB,KLF10,MYC |
| R5020 | chemical reagent | Activated | 4,47E-08 | BIRC3,CCND1,IER3,MYC,NFKBIA,RGS2,TIPARP,TXNIP |
| Creb | group | Activated | 4,77E-08 | ABL2,ATF3,CCND1,CTSV,DUSP1,EGR1,FOS,IER3,JUN,JUNB,LDLR,PER1,RGS2 |
| TCF | group | Activated | 4,87E-08 | ALDH1A1,CCND1,EGR1,FOS,JUNB,MYC,SERPINE1,TSPAN8 |
| ELK1 | transcription regulator | Activated | 4,97E-08 | EGR1,FOS,JUN,JUNB,TIPARP,TNFRSF10B,ZFP36 |
| CD40 | transmembrane receptor | Activated | 5,08E-08 | BIRC3,CCND1,DUSP1,DUSP6,FOS,ICAM1,JUN,MYC,NFKBIA,SERPINE1,TNFAIP3 |
| xanthohumol | chemical drug | | 5,53E-08 | BIRC3,CCND1,FOS,ICAM1,MYC,TNFRSF10B |
| ARRB2 | other | | 5,53E-08 | CXCL2,EGR1,JUNB,MYC,NFKBIZ,SERPINE1 |
| MST1R | kinase | Activated | 5,57E-08 | CCND1,FOS,JUN,MYC,VIM |
| SIN3A | transcription regulator | | 5,58E-08 | CYP1A1,KLF10,KLF6,MYC,PMM1,SERPINE1,TXNIP |
| FOXO1 | transcription regulator | | 5,74E-08 | BIRC3,CCND1,CTSV,EGR1,FOS,ICAM1,IER3,JUN,JUNB,MYC,PPP1R15A,SERPINE1,SLC25A25,TXNIP,UBASH3B |
| ATF2 | transcription regulator | | 5,77E-08 | ATF3,CCND1,DUSP1,EGR1,FOS,JUN,PLAU,RHOB |
| erlotinib | chemical drug | | 5,77E-08 | AXL,EGR1,ICAM1,IGFBP4,NFKBIA,PLAU,SERPINE1,TNFRSF10B |
| okadaic acid | chemical toxicant | | 6,27E-08 | CCND1,CXCL2,EGR1,FOS,JUN,JUNB,MYC,NDRG1 |
| RBCK1 | transcription regulator | Activated | 6,46E-08 | CCND1,ICAM1,MYC,TNFAIP3 |
| aplidine | biologic drug | | 6,46E-08 | DUSP1,FOS,JUN,JUNB |
| CSF3 | cytokine | | 6,53E-08 | BIRC3,CXCL2,DUSP6,EGR1,ETS1,FOS,ICAM1,JUN,MYC,TNFAIP3 |
| CP-55940 | chemical reagent | | 6,54E-08 | JUN,JUNB,MDM2,MYC,NFKBIA,TNFAIP3 |
| GNRH | group | Activated | 6,54E-08 | ATF3,DUSP1,EGR1,FOS,JUN,VGF |
| RAS | group | Activated | 6,59E-08 | CCND1,EGR1,FOS,JUN,LDLR,MYC,PLAU,SERPINE1,VIM |
| genistein | chemical drug | Inhibited | 6,86E-08 | AKR1C3,CCND1,CTSV,CXCL2,CYP1A1,DUSP1,FOS,ICAM1,JUN,JUNB,LDLR,MYC,PER1,SERPINE1 |
| CTNNB1 | transcription regulator | | 6,91E-08 | AFP,ALDH1A1,BHLHE40,CCND1,CXCL2,CYP1A1,EGR1,FOS,GJB1,ITGA6,JUN,MYC,OAT,PHLDA2,PLAU,SELENOW,SERPINE1,SPRY4,TNFAIP3,TSPAN8,VIM |
| AR | ligand-dependent nuclear receptor | | 6,93E-08 | AKR1C3,ALDH1A1,ANKRD1,CCND1,DYRK1A,EGR1,ITGA2,ITGA6,MYC,NDRG1,PER1,PLAU,RHOB,SERPINE1,TSPAN8,VIM |
| MET | kinase | Activated | 7,03E-08 | CCND1,CTSV,FOS,JUN,MDM2,MYC,NFKBIZ,PLK2,VIM |
| tributyrin | chemical drug | | 7,78E-08 | AKAP12,ATF3,BIRC3,DUSP1,ETS1,NDRG1,TNFAIP3 |
| 25-hydroxycholesterol | chemical reagent | | 7,78E-08 | ATF3,FOS,JUN,JUNB,LDLR,MAFF,NFKBIA |
| resiquimod | chemical drug | | 7,91E-08 | ATF3,CXCL2,HIVEP2,ICAM1,IER3,IGFBP4,JUN,NFKBIA,NFKBIZ,PLAU,TNFAIP3,ZC3H12A |
| DACH1 | transcription regulator | Inhibited | 9,01E-08 | EGR1,FOS,IER2,JUN,SERPINE1,TNFAIP3 |
| carbamylcholine | chemical drug | | 9,01E-08 | CCND1,EGR1,FOS,GEM,JUN,RGS2 |
| WT1 | transcription regulator | | 9,07E-08 | CCND1,CTSV,DYRK1A,EGR1,ETS1,JUNB,MDM2,MYC,NDRG1,PHLDA1,SERPINE1 |
| Ras homolog | group | | 9,65E-08 | CCND1,EGR1,FOS,MYC,PLAU |
| EDN3 | other | Activated | 9,65E-08 | CCND1,EGR1,FOS,ITGA2,LAMA1 |
| IL1 | group | Activated | 9,73E-08 | BHLHE40,CXCL2,CYP1A1,DUSP1,EGR1,FOS,ICAM1,JUN,KLF6,LDLR,MYC,SERPINE1,TNFAIP3 |
| quercetin | chemical drug | | 1,02E-07 | CYP1A1,EGR1,FOS,ICAM1,JUN,LDLR,MYC,PLAU,TNFRSF10B |
| propranolol | chemical drug | | 1,05E-07 | CYP1A1,DUSP1,FOS,ICAM1,MYC,NFKBIA |
| H-7 | chemical - kinase inhibitor | Inhibited | 1,05E-07 | CYP1A1,EGR1,FOS,JUNB,MYC,SERPINE1 |
| TNFSF10 | cytokine | | 1,09E-07 | BIRC3,FOS,JUN,NFKBIA,PLAU,PPP1R15A,TNFAIP3,TNFRSF10B |
| HNF1A | transcription regulator | | 1,10E-07 | AFP,AGT,AHSG,AKR1C3,CCND1,DUSP6,FABP1,GJB1,LDLR,NR1D1,SERPINE1,TSC22D2,TTR,ZBTB20,ZNF442 |
| sphingosylphosphocholine | chemical - endogenous mammalian | | 1,16E-07 | DUSP6,FOS,ICAM1,JUN |
| MEMO1 | other | | 1,16E-07 | CCND1,FOS,JUN,MYC |
| RTKN | other | | 1,16E-07 | BIRC3,FOS,TNFAIP3,VIM |
| TAZ | enzyme | | 1,18E-07 | ANKRD1,AXL,DUSP1,KLF6,LDLR,MYC,PLK2,SERPINE1 |
| PLAU | peptidase | | 1,18E-07 | CCND1,ICAM1,MDM2,PHLDA1,PLAU,SERPINE1,VIM |
| DICER1 | enzyme | | 1,23E-07 | AFP,CCND1,EGR1,FOS,ICAM1,ITGA2,ITGA6,JUN,MYC,PLAU,SERPINE1,VIM |
| salinosporamide A | chemical drug | | 1,24E-07 | BIRC3,CCND1,ICAM1,MYC,TNFRSF10B |
| RPS6KA5 | kinase | Activated | 1,24E-07 | DUSP1,FOS,JUN,JUNB,MYC |
| ionomycin | chemical reagent | Activated | 1,26E-07 | DUSP1,EGR1,ETS1,FOS,GEM,ICAM1,LDLR,MYC,PPP1R15A,RGS2 |
| decitabine | chemical drug | | 1,31E-07 | AFP,AKAP12,ANKRD1,BIRC3,CCND1,CYP1A1,DUSP1,DYRK1A,GJB1,ITGA2,JUNB,KLF6,LDLR,MYC,NDRG1,NFKBIA,PLK2,TRRAP,VIM |
| TLR9 | transmembrane receptor | Activated | 1,33E-07 | ATF3,CCND1,CXCL2,DUSP1,EGR1,ICAM1,IER3,MYC,NFKBIZ,PHLDA1,SERPINE1 |
| Laminin (complex) | complex | | 1,41E-07 | BIRC3,CCND1,ITGA6,PLAU,SERPINE1,VIM |
| D-galactosamine | chemical - endogenous mammalian | Activated | 1,41E-07 | CXCL2,FOS,ICAM1,JUN,JUNB,MYC |
| hydrocortisone | chemical - endogenous mammalian | | 1,46E-07 | ANKRD1,CCND1,ICAM1,IGFBP4,ITGA2,NFKBIA,SERPINE1,TNFAIP3,VIM |
| VEGFA | growth factor | Activated | 1,48E-07 | CCND1,CTSV,CXCL2,EGR1,ETS1,FOS,ICAM1,IGFBP4,ITGA2,JUNB,PLAU,SERPINE1 |
| RET | kinase | | 1,55E-07 | CCND1,CTSV,DUSP6,EGR1,FOS,ITGA6,PLAU,REEP6,VGF |
| BRD7 | transcription regulator | | 1,58E-07 | CYP1A1,MDM2,NDRG1,SERPINE1,VIM |
| evodiamine | chemical - endogenous non-mammalian | Inhibited | 1,58E-07 | BIRC3,CCND1,CYP1A1,ICAM1,MYC |
| lysophosphatidylcholine | chemical - other | Activated | 1,59E-07 | EGR1,ICAM1,JUN,LDLR,PLAU,RGS2,SERPINE1 |
| ETS2 | transcription regulator | | 1,59E-07 | DUSP6,FOS,ICAM1,JUN,JUNB,MYC,PLAU |
| MEF2D | transcription regulator | | 1,59E-07 | CCND1,FOS,HDAC9,JUN,JUNB,VIM,ZFP36 |
| silibinin | chemical drug | | 1,59E-07 | CCND1,EGR1,MYC,NFKBIA,PLAU,TNFRSF10B,VIM |
| caffeic acid phenethyl ester | chemical drug | | 1,71E-07 | BIRC3,CCND1,EGR1,ICAM1,KLF6,NFKBIA,RHOB,VIM |
| KLHL21 | other | | 1,72E-07 | DUSP1,NFKBIA,NFKBIZ |
| GPC3 | other | | 1,72E-07 | CCND1,LDLR,MYC |
| STRAP | other | | 1,72E-07 | JUN,MYC,SERPINE1 |
| 2-amino-3-phosphonopropionic acid | chemical - endogenous mammalian | | 1,72E-07 | EGR1,FOS,JUNB |
| acetic acid | chemical - endogenous mammalian | | 1,92E-07 | EGR1,FOS,JUN,JUNB |
| phenylephrine | chemical drug | Activated | 1,93E-07 | ANKRD1,DUSP1,EGR1,FOS,JUN,MYC,RGS2 |
| NR5A2 | ligand-dependent nuclear receptor | | 1,97E-07 | AFP,CCND1,FOS,ICAM1,JUN,JUNB,LDLR,MYC |
| zerumbone | chemical - endogenous non-mammalian | | 1,99E-07 | BIRC3,CCND1,ICAM1,MYC,TNFRSF10B |
| butyric acid | chemical - endogenous mammalian | | 2,11E-07 | ALDH1A1,CCND1,CYP1A1,DUSP1,FOS,HDAC9,ICAM1,MDM2,MXD1,MYC,NFKBIA,PLAU,SERPINE1,TNFRSF10B |
| pirinixic acid | chemical toxicant | | 2,16E-07 | AFP,AHSG,AKR1C3,CCND1,CYP1A1,FABP1,JUNB,KLF10,LDLR,MYC,NFKBIA,NR1D1,PHLDA1,SERPINE1 |
| MAP3K8 | kinase | Activated | 2,18E-07 | ARRDC3,BIRC3,CXCL2,DUSP6,FOS,IER3,JUN,JUNB,TXNIP |
| JAK2 | kinase | Activated | 2,30E-07 | AKAP12,AKR1C3,CCND1,EGR1,FOS,ICAM1,MYC,NFKBIZ,PLK2 |
| Y 27632 | chemical drug | Inhibited | 2,32E-07 | AXL,FOS,ICAM1,MYC,PLAU,SERPINE1,VIM |
| CSF1 | cytokine | Activated | 2,41E-07 | AXL,CCND1,CXCL2,DUSP1,EGR1,FOS,JUN,JUNB,MYC,PLAU,TNFAIP3 |
| dibutyl phthalate | chemical toxicant | Activated | 2,48E-07 | FOS,JUN,JUNB,MYC,SERPINE1 |
| tosylphenylalanyl chloromethyl ketone | chemical - protease inhibitor | | 2,48E-07 | BIRC3,CXCL2,EPHX2,ICAM1,MYC |
| hyaluronic acid | chemical - endogenous mammalian | | 2,60E-07 | CXCL2,FOS,ICAM1,JUN,NFKBIA,PLAU,SERPINE1,TNFAIP3 |
| ADRA1B | G-protein coupled receptor | | 2,78E-07 | CCND1,CTSV,DUSP1,EGR1,FOS,JUN |
| ATP | chemical - endogenous mammalian | Activated | 2,78E-07 | CCND1,CXCL2,DUSP1,FOS,ICAM1,MYC,SERPINE1,TXNIP |
| calcimycin | chemical reagent | Activated | 2,78E-07 | ATF3,EGR1,FOS,ICAM1,JUN,JUNB,MYC,SERPINE1 |
| CCL5 | cytokine | | 2,78E-07 | CXCL2,DUSP1,DUSP6,EMP1,FOS,IER2,ZFP36 |
| NFATC2 | transcription regulator | Activated | 2,79E-07 | AXL,CXCL2,IER3,JUN,MDM2,MYC,NFKBIA,NFKBIZ,PLK2,RGS2 |
| aldosterone | chemical - endogenous mammalian | | 2,86E-07 | AGT,DUSP1,EGR1,FOS,ICAM1,NFKBIA,PER1,RGS2,SERPINE1 |
| ST3-Hel2A-2 | chemical reagent | Activated | 3,01E-07 | ATF3,FOS,IER3,PHLDA1 |
| (+)-MK-801 | chemical drug | | 3,04E-07 | CCND1,EGR1,FOS,ICAM1,JUN,JUNB,NFKBIA |
| TAF4B | transcription regulator | | 3,05E-07 | IER3,ITGA6,JUN,NFKBIA,TNFAIP3 |
| BAPTA-AM | chemical reagent | Inhibited | 3,15E-07 | ATF3,CYP1A1,EGR1,FOS,ICAM1,JUN |
| GH1 | growth factor | Activated | 3,17E-07 | ATF3,EGR1,FOS,JUN,JUNB,LDLR,VIM,ZFP36 |
| FN1 | enzyme | Activated | 3,48E-07 | BIRC3,CCND1,CXCL2,FOS,MYC,NFKBIA,PLAU,SERPINE1,TNFRSF10B,VIM |
| NOTCH1 | transcription regulator | | 3,52E-07 | ANKRD1,ASB2,BIRC3,CCND1,FOS,ICAM1,IGFBP4,ITGA6,MYC,OAT,SERPINE1 |
| BDNF | growth factor | | 3,56E-07 | BHLHE40,DUSP1,DUSP6,EGR1,FOS,JUN,KLF10,PALD1,TIPARP,TXNIP,VGF,VIM |
| USF1 | transcription regulator | | 3,61E-07 | AGT,AKAP12,DUSP1,LDLR,SERPINE1,TNFAIP3,TXNIP |
| oleic acid | chemical - endogenous mammalian | | 3,62E-07 | DUSP1,EGR1,FABP1,FOS,ICAM1,LDLR,NFKBIA,SERPINE1 |
| ANGPT2 | growth factor | | 3,79E-07 | EPHX2,ETS1,ICAM1,PHLDA1,PLAU,RHOB,SERPINE1,TNFRSF10B,TXNIP,VIM |
| Ige | complex | Activated | 3,79E-07 | ASB2,CXCL2,DUSP6,EGR1,ERRFI1,ICAM1,IER3,JUNB,MYC,NFKBIA,SPRED1,VIM |
| Immunoglobulin | complex | | 3,81E-07 | AGT,BCL10,BIRC3,CXCL2,DUSP1,DUSP6,EGR1,FOS,ICAM1,IER3,IGFBP4,MAFF,MYC,NFKBIA,PLAU,RGS2,SERPINA6 |
| ZBTB17 | transcription regulator | Activated | 3,93E-07 | BHLHE40,CCND1,EGR1,IER3,JUN,LDLR,ZFP36 |
| PLK1 | kinase | | 4,02E-07 | ANKRD1,EPHA2,IER3,MDM2,MYC,VIM |
| ADRA1A | G-protein coupled receptor | | 4,02E-07 | CTSV,CYP1A1,DUSP1,EGR1,FOS,JUN |
| 9,10-dimethyl-1,2-benzanthracene | chemical toxicant | Activated | 4,02E-07 | CCND1,CXCL2,CYP1A1,EPHA2,JUN,SERPINE1 |
| staurosporine | chemical - kinase inhibitor | Inhibited | 4,38E-07 | CCND1,CYP1A1,FOS,ICAM1,KLF10,MYC,PLAU,SERPINE1 |
| TCF4 | transcription regulator | | 4,43E-07 | BHLHE40,CCND1,CSRNP1,FOS,IGFBP4,JUN,MYC,PDZK1,PLAU,PLK2,SERINC5,TENT5A,TXNIP |
| CIC | transcription regulator | Inhibited | 4,49E-07 | CCND1,ITGA2,MAFF,SPRY4 |
| ITGA3 | other | | 4,49E-07 | CTSV,ITGA2,PLAU,SERPINE1 |
| fulvestrant | chemical drug | | 4,54E-07 | AXL,CCND1,EGR1,FOS,ICAM1,IER3,JUN,KLF10,LDLR,MYC,PLK2 |
| 5-O-mycolyl-beta-araf-(1->2)-5-O-mycolyl-alpha-araf-(1->1')-glycerol | chemical - endogenous non-mammalian | Activated | 5,03E-07 | ATF3,CXCL2,DUSP1,ICAM1,IER3,NFKBIZ,TNFAIP3 |
| ETS1 | transcription regulator | Activated | 5,30E-07 | CCND1,EGR1,ETS1,ICAM1,JUNB,MDM2,MYC,PHLDA2,PLAU,SERPINE1 |
| selumetinib | chemical drug | | 5,43E-07 | CCND1,FOS,ITGA2,JUN,JUNB |
| PIK3R1 | kinase | | 5,45E-07 | CCND1,FOS,ICAM1,MYC,PLAU,SERPINE1,TXNIP |
| reactive oxygen species | chemical toxicant | | 5,60E-07 | ETS1,JUN,KLF6,LAMA1,MDM2,MYC,SERPINE1,TNFAIP3 |
| NFKBIB | transcription regulator | | 5,69E-07 | CTSV,CXCL2,EGR1,ICAM1,NFKBIA,PLAU |
| tanespimycin | chemical drug | Inhibited | 6,32E-07 | AXL,BIRC3,CCND1,JUN,MYC,PLAU,SERPINE1,TNFAIP3 |
| carbonyl cyanide m-chlorophenyl hydrazone | chemical toxicant | | 6,35E-07 | AGT,CTSV,ERRFI1,NFKBIZ,PHLDA1,SERPINE1 |
| FGF19 | growth factor | | 6,38E-07 | AFP,ALDH1A1,CCND1,CXCL2,EGR1,FOS,RGS2 |
| CSF2RA | transmembrane receptor | | 6,46E-07 | FOS,JUN,JUNB,MYC |
| TLE1 | transcription regulator | | 6,46E-07 | ATF3,DUSP1,EGR1,FOS |
| RPS6KA4 | kinase | | 6,46E-07 | DUSP1,FOS,JUN,JUNB |
| PPP5C | phosphatase | | 6,46E-07 | CCND1,DUSP1,MDM2,MYC |
| LLGL2 | other | | 6,49E-07 | ANKRD1,AXL,DUSP1,NDRG1,SERPINE1 |
| ADIPOQ | other | | 6,73E-07 | CCND1,CXCL2,DUSP1,EGR1,FOS,ICAM1,LDLR,NFKBIA,TNFAIP3 |
| MIB2 | transcription regulator | | 6,84E-07 | CYLD,NFKBIA,TNFAIP3 |
| EIF3I | translation regulator | | 6,84E-07 | CCND1,MYC,SERPINE1 |
| GCLC | enzyme | | 6,84E-07 | FOS,JUN,JUNB |
| ACP1 | phosphatase | | 6,84E-07 | EPHA2,FOS,MYC |
| atipamezole | chemical drug | | 6,84E-07 | EGR1,FOS,JUNB |
| di(2-ethylhexyl) phthalate | chemical toxicant | | 7,08E-07 | EGR1,FABP1,FOS,JUN,MYC,ZFP36 |
| gefitinib | chemical drug | | 7,40E-07 | ALDH1A1,CCND1,CXCL2,ITGA2,MAFF,RHOB,SPRY4,TNFRSF10B,VIM |
| IL33 | cytokine | Activated | 7,40E-07 | AXL,BIRC3,CCND1,CXCL2,EPHX2,ICAM1,JUNB,NFKBIA,NFKBIZ,TNFAIP3,ZC3H12A |
| HNF4A | transcription regulator | | 7,43E-07 | AFP,AGT,AHSG,AKR1C3,ALDH1A1,CCND1,CYLD,CYP1A1,DUSP6,EGR1,EPHA2,FABP1,GJB1,H2BC5,H3C14,ITGA6,JUN,JUNB,LDLR,MDM2,MYC,NDRG1,NR1D1,NUAK2,PDZK1,SERPINE1,SPATA2,TENT5A,TTR,TXNIP,ZFP36 |
| RHOA | enzyme | | 7,44E-07 | AGT,CCND1,FOS,ICAM1,JUN,PLAU,RHOB |
| MYB | transcription regulator | | 7,76E-07 | BHLHE40,BIRC3,CCND1,CXCL2,ICAM1,JUN,MYC,VIM,ZC3H12A |
| MEF2C | transcription regulator | | 8,96E-07 | ATF3,CXCL2,FOS,HDAC9,JUN,JUNB,VIM,ZFP36 |
| PRKCZ | kinase | | 9,09E-07 | CCND1,ETS1,JUN,MYC,NFKBIA |
| AG490 | chemical - kinase inhibitor | | 9,31E-07 | AGT,AKR1C3,BIRC3,CXCL2,FOS,ICAM1,MYC |
| acetaminophen | chemical drug | | 9,33E-07 | CCND1,CTSV,CXCL2,FABP1,GJB1,ICAM1,JUN,MYC,TTR |
| VDR | transcription regulator | | 9,97E-07 | AGT,CCND1,EGR1,FGG,ICAM1,IER3,JUN,LDLR,MYC,TXNIP |
| SPP1 | cytokine | | 1,02E-06 | CXCL2,FOS,ICAM1,ITGA2,MYC,PLAU,SELENOW,SERPINE1,VIM |
| NUPR1 | transcription regulator | Activated | 1,03E-06 | ABL2,AKAP12,ATF3,ETS1,H3C15,KLF6,MXD1,MYC,NDRG1,NR1D1,PHLDA1,PPP1R15A,SERPINE1,TSC22D2 |
| glucocorticoid | chemical drug | | 1,03E-06 | AFP,AGT,CXCL2,DUSP1,ICAM1,MYC,NFKBIA,PER1,SERPINA6,SERPINE1,TXNIP |
| HLX | transcription regulator | | 1,07E-06 | ANKRD1,EGR1,ICAM1,JUN,MYC |
| TAB1 | enzyme | | 1,07E-06 | BIRC3,JUN,NFKBIA,RGS2,SERPINE1 |
| cadmium chloride | chemical toxicant | | 1,07E-06 | CCND1,FOS,JUN,MYC,PLAU,VIM |
| PTGS2 | enzyme | | 1,12E-06 | BIRC3,CCND1,DUSP1,EGR1,FOS,ICAM1,ITGA6,MYC,TNFRSF10B |
| CD36 | transmembrane receptor | Activated | 1,15E-06 | AXL,FABP1,ICAM1,IGFBP4,PLAU,SERPINE1,TNFRSF10B |
| IKBKG | kinase | | 1,18E-06 | BIRC3,CXCL2,DUSP6,FOS,ICAM1,JUNB,NFKBIA,TNFAIP3 |
| mitomycin C | chemical drug | | 1,18E-06 | ATF3,CCND1,EGR1,JUN,MDM2,PPP1R15A |
| KLF4 | transcription regulator | | 1,21E-06 | ALDH1A1,CCND1,CYP1A1,DUSP1,EPHA2,FABP1,LAMA1,MYC,SERPINE1,TBX3,VIM |
| topotecan | chemical drug | | 1,22E-06 | AFP,AKAP12,ATF3,EGR1,ITGA2,JUNB,MDM2,MYC,PLK2 |
| 1'-acetoxychavicol acetate | chemical reagent | Inhibited | 1,22E-06 | BIRC3,CCND1,ICAM1,MYC |
| TCF/LEF | group | | 1,22E-06 | CCND1,JUN,MYC,VIM |
| prazosin | chemical drug | Inhibited | 1,22E-06 | DUSP1,EGR1,FOS,MYC |
| gamma-tocotrienol | chemical drug | | 1,22E-06 | CCND1,JUN,NFKBIA,TNFAIP3 |
| PC-SPES | chemical drug | | 1,24E-06 | CCND1,GJB1,GSTA2,PPP1R15A,SELENOW,SEPHS2,VIM |
| PLAT | peptidase | Activated | 1,25E-06 | CCND1,FOS,JUN,MYC,SERPINE1 |
| fisetin | chemical toxicant | | 1,25E-06 | BIRC3,CCND1,ICAM1,MYC,VIM |
| CXCR4 | G-protein coupled receptor | Activated | 1,30E-06 | AXL,CCND1,EGR1,JUNB,MYC,TNFRSF10B |
| allopurinol | chemical drug | | 1,33E-06 | BIRC3,FABP1,IER3,MYC,TSPAN8,TXNIP,VIM |
| bee venom | chemical - endogenous non-mammalian | | 1,33E-06 | ICAM1,JUN,JUNB,NFKBIA,PPP1R15A,RGS2,TNFAIP3 |
| LGALS3 | other | | 1,33E-06 | CCND1,CTSV,CXCL2,DUSP6,ICAM1,ITGA6,MYC |
| mir-8 | microRNA | | 1,39E-06 | ABL2,AFP,ATF3,CCND1,ERRFI1,ETS1,MYC,VIM,ZFP36 |
| bortezomib | chemical drug | | 1,39E-06 | ATF3,BIRC3,CCND1,CXCL2,ICAM1,MDM2,MYC,PPP1R15A,TNFRSF10B,VIM |
| N-nitro-L-arginine methyl ester | chemical drug | | 1,42E-06 | AGT,ATF3,CXCL2,ICAM1,LDLR,NFKBIA,SERPINE1 |
| formaldehyde | chemical - endogenous mammalian | Activated | 1,45E-06 | CYP1A1,EGR1,FOS,JUN,JUNB |
| Pka | complex | | 1,46E-06 | DUSP1,EGR1,FOS,JUN,JUNB,KLF6,LDLR,RGS2 |
| dopamine | chemical - endogenous mammalian | Activated | 1,46E-06 | ATF3,CSRNP1,EGR1,FOS,JUN,LDLR,PER1,PHLDA2 |
| bexarotene | chemical drug | Inhibited | 1,52E-06 | AKR1C3,AXL,CCND1,DUSP1,EPHA2,ICAM1,IER2,KLF10,MAFF |
| vancomycin | biologic drug | | 1,52E-06 | BIRC3,CCND1,CXCL2,FOS,IER3,JUNB,MYC,TSPAN8,VIM |
| nicotine | chemical drug | Activated | 1,52E-06 | CCND1,EGR1,FOS,ICAM1,JUNB,LDLR,SERPINE1,TTR,ZFP36 |
| CAV1 | transmembrane receptor | | 1,54E-06 | AFP,CCND1,EGR1,FOS,ICAM1,MDM2,SERPINE1,VIM |
| BRD4 | kinase | | 1,54E-06 | BHLHE40,CCND1,CXCL2,FOS,ICAM1,MYC,PLAU,TNFRSF10B |
| rotenone | chemical toxicant | | 1,58E-06 | AGT,BIRC3,CCND1,CXCL2,FOS,TXNIP |
| methylselenic acid | chemical reagent | | 1,61E-06 | ATF3,BIRC3,CCND1,JUN,JUNB,MXD1,MYC,NDRG1,RHOB,TNFAIP3 |
| CSF2RB | transmembrane receptor | | 1,62E-06 | FOS,JUN,JUNB,MYC |
| PLCG1 | enzyme | | 1,62E-06 | CCND1,EGR1,FOS,SERPINE1 |
| IL2RB | transmembrane receptor | | 1,62E-06 | ETS1,FOS,JUN,MYC |
| S1PR3 | G-protein coupled receptor | | 1,62E-06 | ETS1,FOS,ICAM1,JUN |
| GFAP | other | | 1,62E-06 | ITGA2,ITGA6,LAMA1,VIM |
| exenatide | biologic drug | | 1,67E-06 | AGT,FOS,ICAM1,PER1,TXNIP |
| phorbol 12,13-dibutyrate | chemical - endogenous non-mammalian | Activated | 1,67E-06 | FOS,ITGA2,MDM2,SERPINE1,SPRY4 |
| ADRA1 | group | | 1,70E-06 | EGR1,FOS,JUNB |
| ZMIZ2 | transcription regulator | | 1,70E-06 | CCND1,JUN,MYC |
| RAB7A | enzyme | | 1,70E-06 | EGR1,FOS,VIM |
| GCLM | enzyme | | 1,70E-06 | FOS,JUN,JUNB |
| MUC1 | other | | 1,73E-06 | ALDH1A1,CCND1,MYC,NFKBIA,PLAU,VIM |
| lomustine | chemical drug | | 1,73E-06 | BIRC3,IER3,MYC,TNFRSF10B,TSPAN8,VIM |
| CEBPA | transcription regulator | | 1,76E-06 | AGT,AKAP12,AKR1C3,CCND1,FOS,ICAM1,IER3,IGFBP4,JUN,JUNB,MYC,RGS2,SERPINE1 |
| TREM1 | transmembrane receptor | Activated | 1,79E-06 | ABL2,ATF3,CXCL2,EGR1,GEM,MAFF,NFKBIA,PHLDA1,PHLDA2 |
| cigarette smoke | chemical toxicant | Activated | 1,85E-06 | CCND1,CXCL2,CYP1A1,EGR1,FOS,ICAM1,JUN,MAFF,NFKBIA,SERPINE1 |
| Interferon alpha | group | | 1,89E-06 | AFP,ATF3,AXL,CCND1,CXCL2,DUSP6,FOS,ICAM1,IGFBP4,MDM2,MYC,NFKBIA,SPRY4 |
| POLR2A | enzyme | | 2,07E-06 | CCND1,FOS,MYC,NFKBIA,TNFAIP3,VIM |
| TBP | transcription regulator | | 2,07E-06 | FOS,IER3,LDLR,MYC,NFKBIA,TNFAIP3 |
| glutathione | chemical - endogenous mammalian | | 2,07E-06 | ATF3,CCND1,MYC,NDRG1,SERPINE1,TNFRSF10B |
| pCPT-cAMP | chemical - kinase inhibitor | | 2,11E-06 | EGR1,FOS,JUN,JUNB |
| Integrin | complex | | 2,11E-06 | CCND1,FOS,JUN,MYC |
| DDX17 | enzyme | | 2,11E-06 | CCND1,JUN,MDM2,MYC |
| silicon dioxide | chemical drug | | 2,11E-06 | EGR1,FOS,JUN,JUNB |
| Sos | group | | 2,12E-06 | AKR1C3,FOS,ITGA6,KLF6,MXD1,PHLDA1,PLAU,PLK2,TIPARP |
| PSEN1 | peptidase | | 2,41E-06 | AXL,CCND1,CXCL2,DUSP1,DYRK1A,EGR1,EMP1,FOS,H2BC5,MYC,TIPARP,TNFRSF10B |
| TFAP4 | transcription regulator | | 2,52E-06 | DYRK1A,EGR1,FOS,MDM2,VIM |
| CARM1 | transcription regulator | | 2,52E-06 | EPHX2,FOS,ICAM1,IGFBP4,MYC |
| H89 | chemical - kinase inhibitor | | 2,55E-06 | CCND1,DUSP1,EGR1,FOS,MYC,NFKBIA,PLAU,SERPINE1 |
| Mapk | group | Activated | 2,56E-06 | CCND1,EGR1,FOS,JUN,JUNB,MYC,PER1 |
| EZH2 | transcription regulator | | 2,67E-06 | ABL2,ATF3,BIRC3,CCND1,CXCL2,EGR1,ICAM1,MYC,NDRG1,NFKBIA,PER1,TBX3 |
| JUNB | transcription regulator | | 2,68E-06 | ATF3,CCND1,CXCL2,DUSP1,JUNB,PLAU,SERPINE1,TBX3 |
| SNAI1 | transcription regulator | | 2,68E-06 | AFAP1,ANKRD1,AXL,CCND1,FOS,ITGA2,SERPINE1,VIM |
| N(6)-(3-iodobenzyl)-5'-N-methylcarboxamidoadenosine | chemical drug | | 2,71E-06 | CCND1,FOS,MYC,RGS2 |
| S100B | other | | 2,71E-06 | FOS,JUN,MDM2,MYC |

| | | | | |
|---|---|---|---|---|
| berberine | chemical drug | | 2,89E-06 | BIRC3,CCND1,CYP1A1,ICAM1,LDLR,MDM2,VIM |
| GLI3 | transcription regulator | Activated | 2,92E-06 | CCND1,DUSP1,EGR1,JUNB,NFKBIZ,TNFAIP3 |
| ID1 | transcription regulator | | 2,92E-06 | ALDH1A1,CCND1,EGR1,ICAM1,MYC,VIM |
| sulindac sulfide | chemical drug | | 2,92E-06 | ATF3,CCND1,CYP1A1,EGR1,PLAU,TNFRSF10B |
| Pam3-Cys-Ser-Lys4 | chemical reagent | | 2,95E-06 | CXCL2,CYLD,ICAM1,IER3,JUN,NFKBIA,NFKBIZ,RGS2 |
| Histone h3 | group | | 3,02E-06 | AFP,AKAP12,ATF3,CCND1,EGR1,FOS,ICAM1,IGFBP4,JUN,MDM2,NFKBIA,TBX3,TNFAIP3 |
| peptidoglycan | chemical - endogenous non-mammalian | Activated | 3,07E-06 | CXCL2,CYLD,DUSP1,EGR1,FOS,ICAM1,JUNB |
| NCOA2 | transcription regulator | Activated | 3,07E-06 | CCND1,CYP1A1,EGR1,LDLR,NFKBIA,NR1D1,PARD6B |
| SPARC | other | | 3,07E-06 | AKR1C3,BHLHE40,CYP1A1,H3C4,NDRG1,SERPINE1,VIM |
| ATM | kinase | | 3,17E-06 | CCND1,DUSP1,JUN,SERPINE1,TNFAIP3,TNFRSF10B |
| PTHLH | other | | 3,17E-06 | CCND1,FOS,ITGA6,PLAU,RGS2,SERPINE1 |
| 2-deoxyglucose | chemical drug | | 3,17E-06 | CCND1,FOS,JUN,MYC,TNFRSF10B,TXNIP |
| medroxyprogesterone acetate | chemical drug | | 3,24E-06 | CCND1,CYP1A1,ETS1,ICAM1,JUN,JUNB,MYC,PLAU |
| NORAD | other | Activated | 3,25E-06 | BHLHE40,IGFBP4,JUN,JUNB,RHOB |
| TLR7/8 | group | Activated | 3,25E-06 | GEM,NFKBIA,NFKBIZ,PPP1R15A,TNFAIP3 |
| benzyl isothiocyanate | chemical - endogenous non-mammalian | | 3,25E-06 | CCND1,FOS,ICAM1,MYC,SERPINE1 |
| bisindolylmaleimide I | chemical - kinase inhibitor | | 3,26E-06 | AGT,BIRC3,EGR1,FOS,ICAM1,NFKBIA,SERPINE1 |
| quisqualic acid | chemical - endogenous non-mammalian | | 3,39E-06 | EGR1,FOS,JUNB |
| Calcineurin protein(s) | complex | | 3,44E-06 | CTSV,DUSP1,EGR1,FOS,RGS2,SERPINE1 |
| AGER | transmembrane receptor | | 3,44E-06 | CCND1,CXCL2,EGR1,ICAM1,SERPINE1,TXNIP |
| PI3K (family) | group | | 3,46E-06 | BIRC3,FOS,ITGA6,MYC,SERPINE1,TNFRSF10B,TXNIP |
| POU5F1 | transcription regulator | | 3,46E-06 | AFP,BCL10,BIRC3,CCND1,DUSP1,EPHA2,FABP1,MYC,TBX3,TNFRSF10B,VIM |
| CSF1R | kinase | | 3,67E-06 | CCND1,FOS,JUN,JUNB,MYC |
| phenacetin | chemical drug | | 3,67E-06 | BIRC3,IER3,MYC,TSPAN8,VIM |
| pentoxifylline | chemical drug | | 3,67E-06 | CCND1,FOS,ICAM1,NFKBIA,TNFRSF10B |
| isobutylmethylxanthine | chemical toxicant | | 3,81E-06 | AGT,CCND1,EGR1,FOS,JUN,JUNB,MYC,SERPINE1,ZC3H12A |
| BMP2 | growth factor | | 3,81E-06 | BHLHE40,CCND1,EPHX2,ETS1,FOS,JUN,JUNB,KLF10,MYC |
| ERBB3 | kinase | | 3,89E-06 | AXL,CCND1,DUSP1,EGR1,IER2,NDRG1,SERPINE1 |
| NTRK2 | kinase | | 4,03E-06 | CCND1,EGR1,FOS,MYC,SERPINE1,VIM |
| BMP4 | growth factor | | 4,10E-06 | CCND1,JUN,JUNB,KLF10,LAMA1,MXD1,MYC,SERPINE1,TBX3 |
| KL | enzyme | | 4,14E-06 | AXL,CCND1,EGR1,ICAM1,MYC |
| pituitary adenylate cyclase-activating polypeptide | biologic drug | | 4,14E-06 | CCND1,DUSP6,EGR1,ERRFI1,PLK2 |
| 11,12-epoxyeicosatrienoic acid | chemical - endogenous mammalian | | 4,25E-06 | DUSP1,EPHX2,ICAM1,NFKBIA |
| TP53COR1 | other | | 4,25E-06 | BIRC3,MDM2,PLK2,TNFRSF10B |
| CSNK2A1 | kinase | | 4,25E-06 | CCND1,DUSP6,VIM,ZFP36 |
| sulfasalazine | chemical drug | | 4,25E-06 | CCND1,ICAM1,IER3,NFKBIA |
| lapatinib | chemical drug | | 4,25E-06 | FOS,ITGA2,JUN,JUNB |
| SRF | transcription regulator | Activated | 4,33E-06 | AKAP12,ANKRD1,CYLD,EGR1,ETS1,FOS,IER2,ITGA6,JUN,JUNB,LDLR |
| nitrofurantoin | chemical drug | | 4,46E-06 | CCND1,CTSV,FABP1,GJB1,IER3,MYC,NFKBIA,TTR |
| methyl methanesulfonate | chemical toxicant | | 4,65E-06 | ATF3,EGR1,JUN,MDM2,VIM |
| baicalein | chemical drug | | 4,65E-06 | CCND1,CYP1A1,FOS,ICAM1,JUN |
| MAPK7 | kinase | | 4,70E-06 | CCND1,CXCL2,DUSP1,JUN,LDLR,VIM |
| PRL | cytokine | Activated | 4,82E-06 | AKR1C3,CCND1,CTSV,EGR1,FOS,IER3,JUN,MYC,PHLDA2,VIM |
| STAT5B | transcription regulator | | 4,97E-06 | AKR1C3,ANKRD1,BIRC3,CCND1,FOS,GJB1,MDM2,MYC,PMM1,ZFP36 |
| SIRT1 | transcription regulator | | 5,08E-06 | AFP,AGT,BIRC3,CCND1,CYP1A1,ICAM1,LDLR,MYC,NR1D1,PER1,SERPINE1,TNFRSF10B |
| Ro31-8220 | chemical - kinase inhibitor | Inhibited | 5,20E-06 | DUSP1,EGR1,ICAM1,MDM2,SERPINE1 |
| ADRB3 | G-protein coupled receptor | | 5,22E-06 | EGR1,FOS,JUN,JUNB |
| AXIN1 | other | | 5,22E-06 | CCND1,CSRNP1,FOS,MDM2 |
| SIN3B | transcription regulator | | 5,22E-06 | KLF10,KLF6,MYC,TXNIP |
| epirubicin | chemical drug | | 5,22E-06 | CCND1,DUSP1,MYC,TNFRSF10B |
| GSK3B | kinase | | 5,45E-06 | BIRC3,CCND1,FOS,MYC,NFKBIZ,SERPINE1,TNFRSF10B |
| MMP9 | peptidase | Activated | 5,46E-06 | CCND1,CXCL2,FOS,ICAM1,MYC,VIM |
| IFI16 | transcription regulator | | 5,46E-06 | CCND1,CYP1A1,ICAM1,LDLR,MDM2,NFKBIA |
| PTPN11 | phosphatase | | 5,46E-06 | CCND1,EGR1,FOS,JUN,MDM2,MXD4 |
| SFTPA1 | transporter | Inhibited | 5,46E-06 | CXCL2,EGR1,FOS,MYC,PHLDA1,SERPINE1 |
| ciglitazone | chemical drug | | 5,46E-06 | AGT,CCND1,ICAM1,MXD1,NFKBIA,SERPINE1 |
| androgen | chemical drug | | 5,56E-06 | AKR1C3,CCND1,CTSV,ETS1,FOS,JUN,MYC,NFKBIA |
| 5-azacytidine | chemical drug | | 5,56E-06 | DUSP1,EPHA2,KLF6,MAFF,MYC,PLAU,RHOB,TNFRSF10B |
| PTH | other | | 5,80E-06 | BHLHE40,CCND1,CXCL2,DUSP1,FOS,JUN,PLAU,RGS2 |
| N-acetylsphingosine | chemical reagent | Activated | 5,81E-06 | CXCL2,FOS,GSTA2,JUN,SERPINE1 |
| nicotinic acetylcholine receptor | complex | | 5,91E-06 | FOS,JUN,JUNB |
| AKIRIN2 | other | | 5,91E-06 | CCND1,DUSP1,MYC |
| thenoyltrifluoroacetone | chemical reagent | | 5,91E-06 | AGT,BIRC3,SERPINE1 |
| Muscarinic cholinergic receptor | group | | 5,91E-06 | FOS,JUN,RGS2 |
| PARP1 | enzyme | | 6,07E-06 | CCND1,FOS,ICAM1,JUN,MDM2,MYC,TNFRSF10B |
| mir-17 | microRNA | Inhibited | 6,32E-06 | BIRC3,CCND1,CYLD,MDM2,TNFAIP3,TXNIP |
| ETS | group | | 6,36E-06 | EGR1,FOS,ICAM1,SERPINE1 |
| MAPK8IP1 | other | | 6,36E-06 | CCND1,FOS,JUN,PLAU |
| ACVR1C | kinase | | 6,36E-06 | CCND1,FOS,JUNB,SERPINE1 |
| L-buthionine (SR)-sulfoximine | chemical drug | | 6,36E-06 | NFKBIA,SERPINE1,TNFAIP3,TNFRSF10B |
| LRP6 | transmembrane receptor | | 6,48E-06 | ALDH1A1,CCND1,FOS,MYC,VIM |
| SPHK1 | kinase | Activated | 6,48E-06 | BIRC3,CCND1,EGR1,JUN,SERPINE1 |
| SB 216763 | chemical toxicant | | 6,48E-06 | BHLHE40,CCND1,MYC,PLAU,RGS2 |
| C11orf95-RELA | fusion gene/product | Activated | 6,48E-06 | BIRC3,CXCL2,ICAM1,NFKBIA,TNFAIP3 |
| LIPE | enzyme | | 6,76E-06 | ALDH1A1,ANKRD1,ATF3,EGR1,FOS,ITGA6,PER1 |
| MACROH2A1 | other | | 6,78E-06 | ASB2,EGR1,ETS1,PLAU,SERPINE1,TBX3 |
| MTPN | transcription regulator | | 6,78E-06 | AHSG,CCND1,FOS,JUN,MYC,SERPINE1 |
| STAT1 | transcription regulator | | 7,16E-06 | AGT,AXL,CCND1,CXCL2,EGR1,FOS,ICAM1,JUN,MDM2,MYC,TNFRSF10B |
| PROC | peptidase | | 7,20E-06 | AGT,CXCL2,ICAM1,SERPINE1,TNFAIP3 |
| ADRA1D | G-protein coupled receptor | | 7,20E-06 | CTSV,DUSP1,EGR1,FOS,JUN |
| HNRNPK | transcription regulator | | 7,20E-06 | AGT,ITGA6,LDLR,MYC,ZBTB20 |
| quinolinic acid | chemical - endogenous mammalian | Activated | 7,20E-06 | EGR1,FOS,JUN,JUNB,MYC |
| omeprazole | chemical drug | | 7,20E-06 | AGT,CYP1A1,EGR1,FOS,ITGA6 |
| LDLR | transporter | | 7,26E-06 | CCND1,DUSP1,EGR1,ICAM1,IER3,LDLR,MYC,PLAU,PPP1R15A |
| apigenin | chemical - endogenous non-mammalian | | 7,28E-06 | CCND1,CYP1A1,FOS,ICAM1,MDM2,TNFRSF10B |
| ADCYAP1 | other | | 7,52E-06 | CXCL2,EGR1,EPHA2,FOS,IER3,LAMA1,MYC,NFKBIA,RHOB |
| YY1 | transcription regulator | | 7,56E-06 | ANKRD1,EGR1,FOS,LAMA1,LDLR,MDM2,MYC,NFKBIZ,PPP1R15A,VIM |
| TFPI2 | other | | 7,66E-06 | ATF3,CCND1,CXCL2,JUNB |
| fluoxetine | chemical drug | | 7,80E-06 | EGR1,FABP1,FOS,MYC,SERPINA6,VGF |
| ITGA5 | transmembrane receptor | | 7,98E-06 | CCND1,FOS,JUN,JUNB,PLAU |
| 2-(4-amino-1-isopropyl-1H-pyrazolo[3,4-d]pyrimidin-3-yl)-1H-indol-5-ol | chemical reagent | | 8,78E-06 | ANKRD1,ATF3,CCND1,EGR1,ITGA2,KLF6,PPP1R15A,TXNIP |
| NLRX1 | other | | 8,82E-06 | AGT,BCL10,BIRC3,NFKBIA,TNFAIP3 |
| CCN1 | other | Activated | 8,94E-06 | CCND1,CXCL2,ITGA2,MYC,PLAU,SERPINE1 |
| dexamethasone phosphate | chemical drug | Activated | 9,15E-06 | DUSP1,ERRFI1,NFKBIA,TIPARP |
| Ciap | group | | 9,15E-06 | CCND1,CXCL2,MDM2,MYC |
| IPMK | kinase | | 9,15E-06 | FOS,JUN,JUNB,PER1 |
| HOXA5 | transcription regulator | Activated | 9,15E-06 | EGR1,IER2,KLF10,MDM2 |
| bisindolylmaleimide iv | chemical - kinase inhibitor | | 9,15E-06 | EGR1,FOS,ITGA2,MYC |
| Ni2+ | chemical reagent | | 9,15E-06 | BIRC3,CXCL2,ICAM1,NDRG1 |
| IRS1 | enzyme | Activated | 9,20E-06 | CCND1,EGR1,FOS,IGFBP4,LDLR,MYC,NFKBIA |
| IL1RN | cytokine | | 9,20E-06 | ATF3,CYLD,HDAC9,ICAM1,IGFBP4,KLF6,SERPINE1 |
| saikosaponin A | chemical reagent | | 9,42E-06 | FOS,JUN,JUNB |
| SNW1 | transcription regulator | | 9,42E-06 | CCND1,PLAU,SERPINE1 |
| deoxycholate | chemical - endogenous mammalian | Activated | 9,74E-06 | CCND1,EGR1,LDLR,PLAU,TNFRSF10B |
| methotrexate | chemical drug | | 9,82E-06 | CCND1,CTSV,DUSP1,FABP1,ICAM1,MDM2,MYC,TTR,ZBTB43 |
| WNT1 | cytokine | | 1,02E-05 | CCND1,EGR1,FOS,JUN,JUNB,MYC,SERPINE1 |
| FGFR2 | kinase | | 1,02E-05 | AFP,CCND1,ICAM1,LAMA1,MYC,TSPAN8,TTR |
| AREG | growth factor | | 1,02E-05 | CCND1,EGR1,FOS,H2BC5,JUN,PLAU |
| ERBB4 | kinase | | 1,02E-05 | CCND1,DUSP1,EGR1,IGFBP4,JUN,MYC |
| nitroprusside | chemical drug | | 1,07E-05 | DUSP6,EGR1,FOS,MDM2,MYC |
| indomethacin | chemical drug | | 1,08E-05 | BIRC3,CCND1,FOS,IER3,LDLR,MYC,TNFRSF10B,TSPAN8,VIM |
| calcipotriene | chemical drug | | 1,08E-05 | FOS,JUN,JUNB,PLAU |
| NfkB-RelA | complex | | 1,08E-05 | AGT,ICAM1,MYC,NFKBIA |
| P2RY2 | G-protein coupled receptor | | 1,08E-05 | CCND1,ICAM1,JUN,SERPINE1 |
| piceatannol | chemical - endogenous non-mammalian | | 1,08E-05 | CCND1,ICAM1,MYC,TNFRSF10B |
| kaempferol | chemical toxicant | | 1,08E-05 | CYP1A1,ICAM1,JUN,PLAU |
| tert-butyl-hydroquinone | chemical reagent | Activated | 1,09E-05 | ATF3,CYP1A1,FOS,JUN,JUNB,SERPINE1 |
| 8-bromo-cAMP | chemical reagent | | 1,16E-05 | CCND1,DUSP1,EGR1,FOS,JUN,LDLR,MYC,SERPINE1 |
| tacrolimus | chemical drug | | 1,23E-05 | CCND1,CXCL2,EGR1,FOS,IGFBP4,JUN,JUNB,MYC,NFKBIA |
| phorbol esters | chemical - other | Activated | 1,24E-05 | DUSP1,EGR1,FOS,JUN,JUNB,PLAU |
| BAG1 | other | Activated | 1,28E-05 | CCND1,FOS,JUN,PLAU |
| AZGP1 | transporter | | 1,28E-05 | CCND1,EGR1,MYC,VIM |
| KN-62 | chemical - kinase inhibitor | | 1,28E-05 | ATF3,CCND1,EGR1,PPP1R15A |
| amino acids | chemical - endogenous mammalian | | 1,28E-05 | FOS,JUN,JUNB,MYC |
| linolenic acid | chemical - endogenous mammalian | | 1,28E-05 | FABP1,ICAM1,NFKBIA,SERPINE1 |
| methyl-beta-cyclodextrin | chemical drug | | 1,28E-05 | CCND1,LDLR,MYC,SERPINE1 |
| Rock | group | | 1,29E-05 | CCND1,ETS1,FOS,ICAM1,MYC |
| TRAF3 | enzyme | | 1,32E-05 | BIRC3,CXCL2,ICAM1,NDRG1,NFKBIA,TNFAIP3 |
| methapyrilene | chemical drug | | 1,35E-05 | ATF3,CCND1,CTSV,FABP1,GJB1,MYC,TTR |
| SOCS3 | phosphatase | Inhibited | 1,40E-05 | ATF3,CCND1,EGR1,FOS,ICAM1,JUN |
| Ck2 | complex | | 1,41E-05 | FOS,MYC,ZFP36 |
| YBX3 | transcription regulator | | 1,41E-05 | CCND1,JUN,MYC |
| ICAM1 | transmembrane receptor | Activated | 1,42E-05 | CXCL2,FOS,ICAM1,MYC,VIM |
| JAG1 | growth factor | Activated | 1,42E-05 | CCND1,DUSP1,DUSP6,MYC,VIM |
| IRF8 | transcription regulator | Activated | 1,49E-05 | ATF3,EGR1,FOS,ICAM1,KLF6,MDM2,MYC |
| MAC | complex | | 1,49E-05 | AKR1C3,CXCL2,EGR1,ZFP36 |
| CTSB | peptidase | | 1,49E-05 | CCND1,CTSV,CXCL2,MYC |
| ECSIT | transcription regulator | | 1,49E-05 | IER3,JUNB,NFKBIA,TNFAIP3 |
| ozone | chemical toxicant | | 1,49E-05 | CXCL2,FOS,JUN,JUNB |
| ceramide | chemical - endogenous mammalian | | 1,49E-05 | FOS,ICAM1,JUN,MYC |
| rosiglitazone | chemical drug | | 1,51E-05 | AGT,AHSG,CCND1,CYP1A1,EPHX2,ETS1,FABP1,FOS,ICAM1,NR1D1,RGS2,SERPINE1 |
| miR-6873-3p (miRNAs w/seed UCUCUCU) | mature microRNA | | 1,54E-05 | CCND1,CXCL2,DUSP6,EMP1,FGG,GJB1,JUN,MAFF,POLR2J2/POLR2J3,SEPHS2,SERINC5,SERPINE1,SMIM14,TBX3,TENT5A,ZNF627,ZNF844 |
| TAF4 | transcription regulator | | 1,59E-05 | ABL2,CCND1,ETS1,ITGA6,MAFF,MXD1 |
| SYVN1 | transporter | Activated | 1,63E-05 | AXL,BHLHE40,CCND1,DUSP1,EPHA2,ITGA6,TNS4 |
| lactacystin | chemical - protease inhibitor | Activated | 1,68E-05 | ATF3,CCND1,CYP1A1,DUSP1,LDLR,MDM2,NFKBIA,TNFRSF10B |
| FGFR1 | kinase | Activated | 1,68E-05 | CCND1,FOS,JUN,MYC,PLAU,VIM |
| OGT | enzyme | | 1,68E-05 | CCND1,FOS,JUN,MDM2,MYC,VIM |
| NCOR2 | transcription regulator | | 1,69E-05 | BIRC3,CCND1,FOS,JUN,MYC |
| HDAC2 | transcription regulator | | 1,70E-05 | EGR1,FABP1,MDM2,MYC,NFKBIA,PLAU,RHOB |
| sulforafan | chemical drug | | 1,70E-05 | ALDH1A1,CCND1,FOS,ICAM1,NFKBIA,TNFRSF10B,VIM |
| TRADD | other | Activated | 1,73E-05 | CXCL2,ICAM1,NFKBIA,TNFAIP3 |
| hydrochloric acid | chemical - endogenous mammalian | | 1,73E-05 | CXCL2,FOS,ICAM1,JUN |
| olanzapine | chemical drug | | 1,79E-05 | ATF3,DCBLD2,FOS,LDLR,RGS2,TSPAN8 |
| GRP | growth factor | | 2,00E-05 | CCND1,FOS,JUN,MYC |
| PHB | transcription regulator | | 2,00E-05 | CCND1,JUN,MDM2,NFKBIA |
| IRAK3 | kinase | | 2,00E-05 | CXCL2,NFKBIA,TNFAIP3,VIM |
| fludarabine | chemical drug | | 2,00E-05 | CCND1,CXCL2,MDM2,TNFRSF10B |
| DAB2IP | other | | 2,00E-05 | EGR1,MYC,VIM |
| Igkv1-117 | other | | 2,00E-05 | JUN,JUNB,MYC |
| FUS | transcription regulator | | 2,00E-05 | ATF3,CCND1,ICAM1 |
| MAPKAPK2 | kinase | Activated | 2,00E-05 | CXCL2,ICAM1,NFKBIA,PLAU,ZFP36 |
| fenofibrate | chemical drug | | 2,01E-05 | CCND1,FABP1,FGG,ICAM1,JUN,LDLR,PDZK1,SERPINE1,TXNIP |
| ELAVL1 | other | | 2,08E-05 | ATF3,DUSP1,FOS,MYC,NFKBIA,SLC39A5,VIM,ZFP36 |
| PTGES | enzyme | | 2,18E-05 | EGR1,ITGA6,MYC,SERPINE1,VIM |
| CDKN1A | kinase | | 2,20E-05 | CCND1,CXCL2,DUSP1,MDM2,MYC,NFKBIA,PLK2,SERPINE1,VIM |
| POMC | other | | 2,25E-05 | DUSP1,FOS,ICAM1,JUN,JUNB,LDLR |
| CEBPD | transcription regulator | | 2,25E-05 | AGT,CCND1,CTSV,CYP1A1,FOS,MYC |
| PRKDC | kinase | | 2,30E-05 | BIRC3,CCND1,MDM2,NR1D1 |
| FGFR4 | kinase | | 2,30E-05 | CCND1,FOS,PLAU,SERPINE1 |
| dithiothreitol | chemical reagent | | 2,30E-05 | ATF3,CCND1,MYC,PHLDA1 |
| bardoxolone methyl | chemical drug | | 2,30E-05 | BIRC3,CCND1,MYC,TNFRSF10B |
| TFAP2A | transcription regulator | | 2,52E-05 | BIRC3,CXCL2,DCBLD2,GEM,PLAU,SERPINE1 |
| RBPJ | transcription regulator | | 2,52E-05 | CCND1,DUSP1,ETS1,FOS,JUN,MYC,OAT |
| levodopa | chemical - endogenous mammalian | | 2,62E-05 | AXL,CCND1,CYLD,DUSP1,DUSP6,FOS,IER3,JUNB,KLF6,MXD1,PER1,PLK2,SMIM14,VGF |
| APEX1 | enzyme | Activated | 2,63E-05 | EGR1,FOS,JUNB,RGS2 |
| NF1 | other | | 2,63E-05 | CCND1,MXD1,MYC,TNFRSF10B |
| bufalin | chemical reagent | | 2,63E-05 | CCND1,EGR1,FOS,TNFRSF10B |
| E2F1 | transcription regulator | | 2,66E-05 | CCND1,DUSP1,EGR1,FOS,ICAM1,MDM2,MYC,NFKBIA,PLK2,SERPINE1,VIM,ZFP36 |
| THRB | ligand-dependent nuclear receptor | | 2,68E-05 | BIRC3,CCND1,CXCL2,FOS,JUN,LDLR,MYC,PER1,SERPINE1 |
| SNHG20 | other | | 2,74E-05 | CCND1,MYC,VIM |
| OIP5-AS1 | other | | 2,74E-05 | CCND1,KLF10,MYC |
| PIAS2 | transcription regulator | | 2,74E-05 | FOS,ITGA2,LDLR |
| CHRM3 | G-protein coupled receptor | | 2,74E-05 | EGR1,FOS,ITGA2 |
| DRD1 | G-protein coupled receptor | | 2,74E-05 | EGR1,FOS,JUNB |
| VLDLR | transporter | | 2,74E-05 | FOS,SERPINE1,TNFAIP3 |
| AFP | transporter | | 2,74E-05 | EGR1,FOS,TNFRSF10B |
| ELK3 | transcription regulator | | 2,74E-05 | EGR1,FOS,SERPINE1 |
| clioquinol | chemical drug | | 2,74E-05 | CCND1,FOS,JUN |
| clonidine | chemical drug | | 2,74E-05 | EGR1,FOS,MYC |
| OSMR | transmembrane receptor | | 2,76E-05 | BIRC3,CXCL2,FOS,JUNB,ZFP36 |
| IL6ST | transmembrane receptor | | 2,76E-05 | CCND1,EGR1,JUN,JUNB,MYC |
| sodium arsenite | chemical drug | | 2,76E-05 | CCND1,EGR1,FOS,JUN,TNFRSF10B |
| STAT6 | transcription regulator | Activated | 2,92E-05 | CXCL2,EGR1,EPHA2,FABP1,IER2,NFKBIA,SERPINE1,SLC25A25,VIM,ZFP36,ZNF608 |
| potassium chloride | chemical drug | | 2,96E-05 | ATF3,EGR1,FOS,JUN,MYC,TXNIP |
| IFNAR1 | transmembrane receptor | | 2,98E-05 | ATF3,AXL,CXCL2,DUSP6,MYC,SERPINE1,TNFAIP3 |
| GPS2 | transcription regulator | Inhibited | 2,98E-05 | CCND1,HIVEP2,MYC,NFKBIA,RHOB |
| napabucasin | chemical drug | | 2,98E-05 | ALDH1A1,AXL,ITGA2,ITGA6,MYC |
| Sn50 peptide | chemical toxicant | Inhibited | 2,98E-05 | BIRC3,CXCL2,ICAM1,MYC,SERPINE1 |
| Stat3-Stat3 | complex | | 2,99E-05 | FGG,ICAM1,JUN,MYC |
| grape seed extract | chemical drug | | 2,99E-05 | CCND1,MYC,PLAU,VIM |
| ROR1 | kinase | | 2,99E-05 | AXL,NDRG1,SERPINE1,VIM |
| vitamin K3 | chemical drug | | 2,99E-05 | EGR1,FOS,KLF6,MYC |
| lonaprisan | chemical drug | | 3,11E-05 | CCND1,MYC |
| MSK1/2 | group | | 3,11E-05 | DUSP1,FOS |
| FBXO25 | enzyme | | 3,11E-05 | EGR1,FOS |
| RPRD1B | other | | 3,11E-05 | CCND1,MYC |
| CCDC88C | other | | 3,11E-05 | CCND1,MYC |
| CTNNβ-LEF1 | complex | | 3,11E-05 | CCND1,MYC |
| OMG | G-protein coupled receptor | | 3,11E-05 | FOS,MYC |
| NMB | other | | 3,11E-05 | FOS,JUN |
| RAMP3 | G-protein coupled receptor | | 3,11E-05 | FOS,VIM |
| nitrosonifedipine | chemical reagent | | 3,11E-05 | AGT,ICAM1 |
| inostamycin | chemical reagent | | 3,11E-05 | CCND1,TNFRSF10B |
| (S)-flurbiprofen | chemical drug | | 3,11E-05 | CCND1,JUNB |
| GLI2 | transcription regulator | | 3,12E-05 | CCND1,CXCL2,JUNB,MYC,PI3,VIM |
| CCN2 | growth factor | | 3,12E-05 | AFP,CCND1,EGR1,JUN,MYC,SERPINE1 |
| PDGFB | growth factor | Activated | 3,22E-05 | EGR1,FOS,ICAM1,MYC,PHLDA1 |
| Go 6976 | chemical - kinase inhibitor | Inhibited | 3,22E-05 | CCND1,CXCL2,ETS1,FOS,NFKBIA |
| MED1 | transcription regulator | Activated | 3,23E-05 | BHLHE40,CCND1,CYP1A1,MDM2,MYC,NFKBIA,TBX3 |
| VIP | other | | 3,29E-05 | CCND1,CXCL2,FOS,MYC,NFKBIA,VIM |
| CD24 | other | | 3,29E-05 | ATF3,FOS,ICAM1,JUN,PLAU,PPP1R15A |
| BCL2 | transporter | | 3,29E-05 | CCND1,FOS,ICAM1,MDM2,NDRG1,NFKBIA |
| ITGAV | transmembrane receptor | | 3,39E-05 | FOS,LDLR,PLAU,SERPINE1 |
| ALOX15 | enzyme | | 3,39E-05 | CXCL2,ICAM1,JUN,RGS2 |
| honokiol | chemical - endogenous non-mammalian | | 3,39E-05 | CCND1,FOS,ICAM1,JUNB |
| TSH | complex | Activated | 3,47E-05 | CCND1,EGR1,FOS,JUNB,MYC |
| CD3E | transmembrane receptor | | 3,47E-05 | BCL10,FOS,JUN,MYC,NFKBIA |
| methylmercury | chemical toxicant | | 3,47E-05 | CYP1A1,EGR1,MDM2,RHOB,TXNIP |
| triamterene | chemical drug | | 3,47E-05 | BIRC3,IER3,MYC,TSPAN8,VIM |
| miR-155-5p (miRNAs w/seed UAAUGCU) | mature microRNA | Inhibited | 3,54E-05 | ATF3,BCL10,BIRC3,CCND1,CXCL2,ETS1,FOS,HIVEP2,LCA5,MDM2,PALD1,SERPINE1,SPRED1,TIPARP |
| TNFRSF1A | transmembrane receptor | | 3,64E-05 | BIRC3,CXCL2,ICAM1,IER3,JUN,PLAU,SERPINE1 |
| L-histidine | chemical - endogenous mammalian | | 3,64E-05 | ATF3,EGR1,PPP1R15A |
| RB1CC1 | other | | 3,64E-05 | CCND1,MYC,SERPINE1 |
| SR 144528 | chemical reagent | | 3,64E-05 | EGR1,ICAM1,NFKBIA |
| pyrrolidine dithiocarbamate | chemical reagent | | 3,65E-05 | BIRC3,CXCL2,ICAM1,MYC,NFKBIA,SERPINE1 |
| puromycin aminonucleoside | chemical reagent | | 3,65E-05 | CTSV,IER3,MYC,TSPAN8,TTR,VIM |
| IL13 | cytokine | | 3,68E-05 | ATF3,CTSV,CXCL2,EGR1,FGG,ICAM1,JUN,KLF6,PHLDA1,PLAU,SERPINE1 |
| SHC1 | other | | 3,73E-05 | EGR1,FOS,ICAM1,PHLDA1,SERPINE1 |
| VCAN | other | | 3,78E-05 | CXCL2,ICAM1,IGFBP4,MDM2,NFKBIA,PLAU,RGS2 |
| SOX17 | transcription regulator | | 3,82E-05 | AFP,CCND1,FOS,LAMA1 |
| ANXA2 | other | | 3,82E-05 | JUN,MDM2,MYC,TNFRSF10B |
| ARRB1 | other | | 3,82E-05 | EGR1,JUNB,MYC,SERPINE1 |
| CDC42 | enzyme | | 3,82E-05 | ATF3,CCND1,FOS,JUN |
| green tea polyphenol | chemical drug | | 3,82E-05 | FOS,ICAM1,JUN,PLAU |
| miR-200b-3p (and other miRNAs w/seed AAUACUG) | mature microRNA | Inhibited | 3,89E-05 | ATF3,DCBLD2,DUSP1,ERRFI1,ETS1,GEM,JUN,KLF10,KLF6,LCA5,MXD4,PMM1,TSC22D2,VIM |

| | | | | |
|---|---|---|---|---|
| CpG oligonucleotide | chemical drug | | 3,93E-05 | CXCL2,DUSP1,EGR1,ICAM1,MYC,NFKBIZ,SERPINE1 |
| KCNE3 | ion channel | | 4,01E-05 | AKR1C3,CSRNP1,DUSP6,PER1,PHLDA1 |
| Map3k7 | kinase | Activated | 4,01E-05 | AFP,BHLHE40,JUN,NFKBIA,NFKBIZ |
| 3,3'-diindolylmethane | chemical drug | | 4,01E-05 | ATF3,CCND1,NFKBIA,TNFRSF10B,VIM |
| diethylnitrosamine | chemical toxicant | | 4,04E-05 | AFP,CCND1,JUN,MYC,OAT,PHLDA1 |
| metribolone | chemical reagent | | 4,07E-05 | AKAP12,CCND1,EGR1,ERRFI1,FOS,ITGA6,LDLR,MYC,NDRG1,REEP6,TXNIP |
| PML | transcription regulator | | 4,09E-05 | CCND1,FABP1,LDLR,MAFF,NDRG1,TNFAIP3,VIM |
| TAL1 | transcription regulator | | 4,17E-05 | CYLD,ETS1,ICAM1,JUN,MYC,NFKBIZ,TNFAIP3,ZC3H12A |
| PRKAG3 | other | | 4,25E-05 | ANKRD1,EGR1,HIVEP2,ITGA6,JUNB,TSPAN8,ZFP36 |
| IKK (complex) | complex | | 4,30E-05 | BIRC3,CCND1,ICAM1,MYC |
| SETD2 | enzyme | | 4,30E-05 | JUN,LAMA1,RHOB,SERPINE1 |
| SPDEF | transcription regulator | | 4,30E-05 | BIRC3,ITGA6,PLAU,SERPINE1,VIM |
| Collagen type I (complex) | complex | | 4,30E-05 | CCND1,ITGA2,MYC,PLAU,VIM |
| fenamic acid | chemical reagent | | 4,30E-05 | BIRC3,IER3,MYC,TSPAN8,VIM |
| testosterone | chemical - endogenous mammalian | | 4,36E-05 | ANKRD1,CCND1,CTSV,EGR1,FOS,JUN,NDRG1,NFKBIA,OAT,PLAU |
| EPO | cytokine | Activated | 4,38E-05 | EGR1,FABP1,FOS,JUN,JUNB,LDLR,MYC,NFKBIA,TXNIP |
| TRAF6 | enzyme | Activated | 4,61E-05 | BIRC3,CXCL2,CYLD,ICAM1,TNFAIP3 |
| bromobenzene | chemical toxicant | | 4,61E-05 | CTSV,FOS,JUN,MYC,PPP1R15A |
| CDKN2A | transcription regulator | | 4,62E-05 | CCND1,DUSP1,EGR1,FOS,JUN,MDM2,MYC,SERPINE1,VIM |
| L-glutamic acid | chemical - endogenous mammalian | Activated | 4,69E-05 | CCND1,EGR1,FOS,JUN,JUNB,PER1 |
| CHRM1 | G-protein coupled receptor | | 4,71E-05 | EGR1,FOS,JUN |
| ELK4 | transcription regulator | | 4,71E-05 | EGR1,FOS,JUNB |
| gambogic acid | chemical - endogenous non-mammalian | | 4,71E-05 | BIRC3,CCND1,MYC |
| ACVRL1 | kinase | | 4,82E-05 | ICAM1,MYC,PLAU,SERPINE1 |
| amiodarone | chemical drug | | 4,82E-05 | CYP1A1,FABP1,ICAM1,LDLR |
| PML-RARA | fusion gene/product | | 4,82E-05 | EGR1,FOS,JUN,MYC |
| gemfibrozil | chemical drug | | 4,94E-05 | CCND1,LDLR,MYC,NFKBIA,SERPINE1 |
| HTT | transcription regulator | | 4,96E-05 | AGT,AKAP12,ATF3,BHLHE40,CCND1,DUSP6,EGR1,FOS,HIVEP2,JUN,JUNB,LDLR,PER1,VGF |
| pioglitazone | chemical drug | | 5,13E-05 | AHSG,CCND1,EGR1,FOS,ICAM1,LDLR,SERPINE1 |
| linoleic acid | chemical - endogenous mammalian | | 5,28E-05 | DUSP1,FABP1,ICAM1,LDLR,NFKBIA |
| GNA14 | enzyme | | 5,28E-05 | AKAP12,ANKRD1,ATF3,KLF6,PHLDA1 |
| mir-34 | microRNA | Inhibited | 5,28E-05 | AXL,BIRC3,CCND1,EMP1,MYC |
| FBXW7 | enzyme | | 5,28E-05 | JUN,LDLR,MYC,RHOB,SERPINE1 |
| lipoxin A4 | chemical - endogenous mammalian | | 5,38E-05 | CCND1,CYP1A1,ICAM1,SERPINE1 |
| TRH | other | | 5,38E-05 | DUSP1,FOS,JUN,JUNB |
| XDH | enzyme | | 5,38E-05 | DUSP1,PLAU,SERPINE1,VIM |
| CAMK4 | kinase | | 5,38E-05 | ATF3,FOS,JUN,JUNB |
| GNB2 | enzyme | | 5,38E-05 | DUSP1,IER3,PLAU,PLK2 |
| CDKN1B | kinase | | 5,42E-05 | CCND1,EGR1,EPHA2,MYC,PALD1,SERPINE1 |
| norepinephrine | chemical - endogenous mammalian | Activated | 5,52E-05 | DUSP1,EGR1,FOS,GEM,NFKBIA,PER1,SERPINE1 |
| miR-10394-3p (and other miRNAs w/seed GGGCGCG) | mature microRNA | Inhibited | 5,61E-05 | H3C14,H3C15,IER2,IER3,JUN,JUNB,NUAK2,PARD6B,RHOB,TXNIP,VGF |
| TRAF3IP2 | other | | 5,64E-05 | AGT,CXCL2,ICAM1,NFKBIZ,ZC3H12A |
| tyrphostin AG 1478 | chemical - kinase inhibitor | | 5,64E-05 | CCND1,FOS,ICAM1,ITGA2,PLAU |
| tetrachlorodibenzodioxin | chemical toxicant | | 5,96E-05 | CCND1,CTSV,CYP1A1,FOS,HDAC9,JUN,MYC,PMM1,SERPINE1,TIPARP |
| MEG3 | other | | 5,98E-05 | JUN,MDM2,VIM |
| dihydroartemisinin | chemical drug | | 5,98E-05 | CCND1,MDM2,MYC |
| PIAS4 | transcription regulator | | 5,98E-05 | CXCL2,FABP1,SERPINE1 |
| HOXA4 | transcription regulator | | 5,98E-05 | ANKRD1,CCND1,MYC |
| MVP | other | | 5,98E-05 | CXCL2,ICAM1,JUN |
| pentobarbital | chemical drug | | 5,98E-05 | FOS,JUN,JUNB |
| noscapine | chemical drug | | 5,98E-05 | BIRC3,CCND1,ICAM1 |
| growth factor | group | | 5,98E-05 | DUSP1,FOS,MYC |
| Cd2+ | chemical toxicant | | 5,98E-05 | CCND1,FOS,MYC |
| TWIST2 | transcription regulator | | 5,98E-05 | CCND1,DUSP6,KLF6,VIM |
| ursolic acid | chemical drug | | 6,03E-05 | ATF3,CCND1,FOS,MYC,TNFRSF10B |
| BCL6 | transcription regulator | | 6,19E-05 | CCND1,DUSP6,HDAC9,JUNB,KLF6,LDLR,MYC,SERPINE1 |
| JQ1 | chemical reagent | | 6,23E-05 | BIRC3,CCND1,FOS,MYC,TNFAIP3,TNFRSF10B |
| GW3965 | chemical reagent | | 6,23E-05 | DUSP1,IER3,LDLR,NFKBIA,NR1D1,PPP1R15A |
| aspirin | chemical drug | Inhibited | 6,38E-05 | AKAP12,CCND1,EMP1,ICAM1,IER3,ITGA2,ITGA6 |
| NOD2 | other | | 6,43E-05 | CXCL2,ICAM1,IER3,NFKBIA,TNFAIP3 |
| cadmium | chemical toxicant | | 6,43E-05 | CCND1,CYP1A1,JUN,MYC,PLAU |
| GNB1 | enzyme | | 6,64E-05 | DUSP1,IER3,PLAU,PLK2 |
| NR1D1 | ligand-dependent nuclear receptor | | 6,64E-05 | CTSV,EGR1,FOS,NR1D1 |
| MIF | cytokine | | 6,83E-05 | CCND1,CXCL2,DUSP1,FOS,ICAM1,JUN |
| Il3 | cytokine | Activated | 6,85E-05 | BHLHE40,EGR1,FOS,MDM2,MYC |
| PF4 | cytokine | | 6,85E-05 | BIRC3,ICAM1,NFKBIA,NFKBIZ,TNFAIP3 |
| TLR4 | transmembrane receptor | Activated | 6,93E-05 | ATF3,BCL10,CXCL2,ICAM1,MYC,NFKBIA,NFKBIZ,PLK2,TNFAIP3,ZC3H12A |
| miR-101-3p (and other miRNAs w/seed ACAGUAC) | mature microRNA | Inhibited | 6,96E-05 | DCBLD2,DUSP1,EMP1,FOS,ITGA2,JUNB,KLF6,MXD1,PHLDA1,RGS2,SMIM14,SPATA2,SPRED1,TENT5A |
| BRCA1 | transcription regulator | | 7,10E-05 | CCND1,CYP1A1,EGR1,MDM2,MYC,PHLDA1,VIM |
| PRNP | other | | 7,29E-05 | CCND1,EGR1,MDM2,MYC,NFKBIA |
| cyclophosphamide | chemical drug | | 7,29E-05 | ATF3,FOS,IER3,MYC,TSPAN8 |
| cerivastatin | chemical drug | | 7,29E-05 | ICAM1,JUN,MYC,PLAU,SERPINE1 |
| ENG | transmembrane receptor | | 7,34E-05 | ITGA6,MYC,PLAU,SERPINE1 |
| ABL1 | kinase | | 7,34E-05 | CCND1,IER3,MDM2,MYC |
| histone deacetylase | complex | | 7,34E-05 | CCND1,JUNB,PER1,PLAU |
| propofol | chemical drug | | 7,34E-05 | EGR1,FOS,GJB1,ZFP36 |
| lonafarnib | chemical drug | | 7,44E-05 | BIRC3,CCND1,TNFRSF10B |
| TIFA | other | | 7,44E-05 | ICAM1,NFKBIA,TNFAIP3 |
| 48s | complex | | 7,44E-05 | CCND1,MYC,PPP1R15A |
| Betacatenin/TCF | complex | | 7,44E-05 | CCND1,JUN,MYC |
| SP2 | transcription regulator | | 7,44E-05 | CCND1,FOS,SERPINE1 |
| GTF2I | transcription regulator | | 7,44E-05 | CCND1,FOS,MYC |
| FOXP3 | transcription regulator | | 7,61E-05 | DUSP6,EGR1,FOS,IGFBP4,JUN,TNFAIP3,TTR |
| heparin | chemical - endogenous mammalian | | 7,76E-05 | CCND1,DUSP1,FOS,ICAM1,VIM |
| GATA3 | transcription regulator | | 7,79E-05 | CCND1,ETS1,FOS,ICAM1,MDM2,MYC,TBX3,VIM |
| celecoxib | chemical drug | | 7,81E-05 | CCND1,FOS,ICAM1,MDM2,SERPINE1,TNFRSF10B |
| PP1 | chemical - kinase inhibitor | | 8,10E-05 | FOS,JUN,MYC,SERPINE1 |
| isoprenaline | chemical drug | | 8,10E-05 | EPHX2,FOS,PER1,PLK2 |
| MAP3K7 | kinase | | 8,25E-05 | BIRC3,CXCL2,JUN,RGS2,SERPINE1 |
| HMOX1 | enzyme | | 8,52E-05 | CCND1,CXCL2,CYP1A1,FOS,ICAM1,ZFP36 |
| Lh | complex | | 8,71E-05 | AKAP12,AXL,DUSP1,FOS,GEM,PER1,RHOB,TNFAIP3 |
| PD173074 | chemical reagent | | 8,76E-05 | CCND1,DUSP6,LAMA1,MXD1,MYC |
| KDM3A | transcription regulator | | 8,92E-05 | CCND1,ETS1,MYC,SERPINE1 |
| IRS2 | enzyme | | 8,92E-05 | CCND1,EGR1,FOS,MYC |
| epoprostenol | chemical - endogenous mammalian | | 9,12E-05 | EGR1,FOS,SERPINE1 |
| cyclopiazonic acid | chemical - endogenous non-mammalian | | 9,12E-05 | CXCL2,EGR1,FOS |
| 2-acetylaminofluorene | chemical toxicant | | 9,12E-05 | AFP,CCND1,CYP1A1 |
| myricetin | chemical - endogenous non-mammalian | | 9,12E-05 | CCND1,CYP1A1,MYC |
| CXCL8 | cytokine | | 9,29E-05 | CXCL2,ICAM1,NFKBIA,TNFRSF10B,ZFP36 |
| MAP3K14 | kinase | Activated | 9,29E-05 | BIRC3,CXCL2,ICAM1,NFKBIA,TNFAIP3 |
| GAST | other | Activated | 9,29E-05 | CCND1,EGR1,FOS,JUN,NFKBIA |
| dehydroisoandrosterone | chemical - endogenous mammalian | | 9,29E-05 | CCND1,CYP1A1,ICAM1,MYC,NFKBIA |
| NIBAN2 | transcription regulator | | 9,31E-05 | CCND1,MYC |
| exendin(9-39) amide | biologic drug | | 9,31E-05 | AGT,FOS |
| potassium channel | group | | 9,31E-05 | FOS,JUN |
| KLHL2 | other | | 9,31E-05 | CCND1,JUN |
| ADI PEG20 | biologic drug | | 9,31E-05 | AXL,MYC |
| CSNK1E | kinase | | 9,31E-05 | PER1,SERPINE1 |
| SRGAP1 | other | | 9,31E-05 | CCND1,MYC |
| ELL | transcription regulator | | 9,31E-05 | FOS,MYC |
| ASS1 | enzyme | | 9,31E-05 | AXL,MYC |
| SGI 1776 | chemical drug | | 9,31E-05 | CCND1,MYC |
| AHNAK | other | | 9,31E-05 | CCND1,MYC |
| TOR2A | other | | 9,31E-05 | FOS,MYC |
| TRAF7 | enzyme | | 9,31E-05 | JUN,MYC |
| RING1 | transcription regulator | | 9,31E-05 | FOS,JUN |
| HMGN2 | other | | 9,31E-05 | IER3,PHLDA2 |
| CYP2C9 | enzyme | | 9,31E-05 | CCND1,DUSP1 |
| ATAD2 | enzyme | | 9,31E-05 | CCND1,MYC |
| (E)-1-(2-nitrovinyl)naphthalene | chemical reagent | | 9,31E-05 | CCND1,MYC |
| (E)-9-(2-nitrovinyl)anthracene | chemical reagent | | 9,31E-05 | CCND1,MYC |
| di-2-pyridylketone 4-cyclohexyl-4-methyl-3-thiosemicarbazone | chemical drug | | 9,31E-05 | ERRFI1,NDRG1 |
| tarenflurbil | chemical drug | | 9,31E-05 | CCND1,JUNB |
| metformin | chemical drug | | 9,32E-05 | AHSG,ATF3,FOS,ITGA6,MYC,NFKBIA,SERPINE1 |
| verapamil | chemical drug | | 9,79E-05 | FOS,JUN,LDLR,TXNIP |
| CNTF | cytokine | | 1,04E-04 | EGR1,FOS,JUNB,VIM,ZFP36 |
| TRAF2 | enzyme | Activated | 1,04E-04 | BIRC3,CYLD,ICAM1,NDRG1,TNFAIP3 |
| TSC2 | other | Inhibited | 1,05E-04 | ATF3,CCND1,EGR1,FOS,PLAU,ZFP36 |
| ATF4 | transcription regulator | | 1,06E-04 | ANKRD1,ATF3,CXCL2,JUN,NDRG1,PPP1R15A,TNFRSF10B |
| NAMPT | cytokine | | 1,07E-04 | AXL,CXCL2,EPHA2,JUN |
| leukotriene C4 | chemical - endogenous mammalian | | 1,10E-04 | EGR1,FOS,ICAM1 |
| triclosan | chemical drug | | 1,10E-04 | FOS,JUN,PLAU |
| PPP1R1B | phosphatase | | 1,10E-04 | CCND1,FOS,MYC |
| CHD1 | enzyme | | 1,10E-04 | CXCL2,ICAM1,IER3 |
| THY1 | other | | 1,10E-04 | AXL,ETS1,VIM |
| CCAT2 | other | | 1,10E-04 | CCND1,MYC,VIM |
| NEDD4 | enzyme | | 1,10E-04 | DUSP6,ERRFI1,MDM2 |
| ethidium | chemical drug | | 1,10E-04 | CCND1,PLAU,SERPINE1 |
| THRA | ligand-dependent nuclear receptor | | 1,14E-04 | CCND1,CXCL2,FOS,JUN,LDLR,SERPINE1 |
| HDAC4 | transcription regulator | | 1,14E-04 | ATF3,DYRK1A,FOS,HDAC9,JUN,RGS2 |
| cobalt chloride | chemical reagent | | 1,17E-04 | BHLHE40,CYP1A1,DUSP1,MDM2,NDRG1 |
| ZMPSTE24 | peptidase | | 1,17E-04 | ATF3,CTSV,CXCL2,ICAM1 |
| bleomycin | biologic drug | | 1,21E-04 | CXCL2,FABP1,ICAM1,LAMA1,MYC,SERPINE1,TNFRSF10B |
| SOD1 | enzyme | | 1,23E-04 | CCND1,FOS,ICAM1,JUN,JUNB,TNFAIP3,TNFRSF10B,VGF |
| LYN | kinase | | 1,24E-04 | EGR1,ETS1,MYC,NFKBIA,NFKBIZ |
| histamine | chemical - endogenous mammalian | | 1,24E-04 | CXCL2,FOS,ICAM1,JUN,LDLR |
| miR-4281 (miRNAs w/seed GGUCCCG) | mature microRNA | | 1,27E-04 | BIRC3,DUSP1,GEM,GJB1,LDLR,MAFF,REEP6,SERPINE1,TNFRSF10B |
| HOTAIR | other | | 1,28E-04 | ABL2,ICAM1,MYC,NFKBIA |
| IFNLR1 | transmembrane receptor | | 1,28E-04 | ATF3,CXCL2,MYC,TNFAIP3 |
| BRAF | kinase | | 1,29E-04 | BIRC3,CCND1,EGR1,ICAM1,MYC,SPRY4,TNFRSF10B |
| NR3C2 | ligand-dependent nuclear receptor | Activated | 1,29E-04 | AGT,EGR1,ICAM1,JUNB,RGS2,SERPINE1 |
| miR-4753-5p (miRNAs w/seed AAGGCCA) | mature microRNA | Inhibited | 1,31E-04 | ATF3,DCBLD2,DYRK1A,EPHX2,KLF6,MDM2,NFKBIZ,RGS2,SERINC5,SLC25A25,SMIM14,TNFAIP3,TNFRSF10B,TXNIP,ZBTB20 |
| GLIS2 | transcription regulator | | 1,32E-04 | CCND1,SERPINE1,VIM |
| CRBN | enzyme | | 1,32E-04 | JUN,MYC,OAT |
| Endothelin | group | | 1,32E-04 | FOS,JUN,MYC |
| mir-154 | microRNA | | 1,32E-04 | CCND1,KLF10,MYC |
| S1PR2 | G-protein coupled receptor | | 1,32E-04 | FOS,ICAM1,JUN |
| RPS6KA3 | kinase | | 1,32E-04 | FOS,MYC,TNFRSF10B |
| lisinopril | biologic drug | | 1,32E-04 | FOS,JUN,LDLR |
| acadesine | chemical drug | | 1,32E-04 | EGR1,LDLR,OAT |
| chenodeoxycholic acid | chemical - endogenous mammalian | | 1,38E-04 | AGT,EGR1,FGG,LDLR,TNFRSF10B |
| SBDS | other | | 1,38E-04 | AKR1C3,EGR1,FOS,MAFF,PPP1R15A |
| IKBKE | kinase | | 1,38E-04 | BIRC3,CCND1,CXCL2,MYC,NFKBIA |
| TGFBR1 | kinase | | 1,38E-04 | BHLHE40,CCND1,SERPINE1,VIM,ZFP36 |
| IL3 | cytokine | Activated | 1,38E-04 | BIRC3,CCND1,EGR1,FOS,ICAM1,JUN,JUNB,MDM2,MYC |
| MTA1 | transcription regulator | | 1,39E-04 | CCND1,EMP1,EPHA2,MYC |
| K+ | chemical - endogenous mammalian | | 1,39E-04 | ATF3,EGR1,FOS,JUN |
| SOCS1 | other | Inhibited | 1,45E-04 | CCND1,CXCL2,DUSP1,FOS,ICAM1,JUN |
| SP110 | transcription regulator | | 1,45E-04 | ATF3,CYLD,ETS1,MYC,TBX3,TXNIP |
| NCOA3 | transcription regulator | | 1,45E-04 | ANKRD1,CCND1,CYP1A1,NFKBIA,PARD6B |
| PKD1 | ion channel | | 1,50E-04 | ALDH1A1,AXL,CXCL2,HDAC9,IER2,MYC,TTR |
| TGFBR2 | kinase | | 1,50E-04 | BHLHE40,CCND1,CXCL2,ICAM1,JUN,SERPINE1,TXNIP |
| AVP | other | | 1,51E-04 | ATF3,CCND1,EGR1,FOS |
| RARG | ligand-dependent nuclear receptor | | 1,53E-04 | DUSP1,EGR1,FOS,JUN,MYC |
| GATA4 | transcription regulator | | 1,55E-04 | ANKRD1,CXCL2,FABP1,FOS,JUN,MYC,VIM |
| prostaglandin E1 | chemical - endogenous mammalian | | 1,56E-04 | FOS,ICAM1,MYC |
| NOD1 | other | | 1,56E-04 | CXCL2,CYLD,DUSP1 |
| CTBP2 | transcription regulator | | 1,56E-04 | CCND1,MDM2,SPRY4 |
| KCNIP3 | transcription regulator | | 1,56E-04 | CCND1,FOS,TNFAIP3 |
| MNT | transcription regulator | | 1,56E-04 | KLF10,KLF6,TXNIP |
| PDCD4 | other | | 1,56E-04 | JUN,MYC,PLAU |
| TREM2 | transmembrane receptor | | 1,56E-04 | CXCL2,NFKBIA,TNFAIP3 |
| ACTN4 | transcription regulator | | 1,56E-04 | CCND1,FOS,SERPINE1 |
| SCP2 | transporter | | 1,56E-04 | FABP1,LDLR,PDZK1 |
| metyrapone | chemical drug | | 1,56E-04 | CYP1A1,FOS,ICAM1 |
| PS-1145 | chemical - kinase inhibitor | | 1,56E-04 | ICAM1,NFKBIA,TNFAIP3 |
| peroxynitrite | chemical toxicant | | 1,56E-04 | FOS,ICAM1,RGS2 |
| IFIH1 | enzyme | | 1,64E-04 | CXCL2,EGR1,NFKBIA,PHLDA1 |
| 2-bromoethylamine | chemical reagent | | 1,64E-04 | IER3,MYC,TSPAN8,VIM |
| IL11RA | transmembrane receptor | | 1,64E-04 | FOS,ICAM1,JUNB,MYC |
| TXN | enzyme | | 1,64E-04 | CYP1A1,FOS,ICAM1,NFKBIA |
| CCL2 | cytokine | | 1,70E-04 | CCND1,FOS,ICAM1,SERPINE1,ZC3H12A |
| lovastatin | chemical drug | | 1,75E-04 | ATF3,CCND1,LDLR,MYC,RHOB,SERPINE1 |
| SQSTM1 | transcription regulator | | 1,77E-04 | CXCL2,JUN,NFKBIA,PLK2 |
| BSG | transporter | | 1,77E-04 | CCND1,PLAU,SERPINE1,VIM |
| RETN | other | | 1,77E-04 | FOS,ICAM1,LDLR,SERPINE1 |
| elaidic acid | chemical - endogenous mammalian | | 1,79E-04 | ALDH1A1,FABP1,LDLR,SERPINA6,TTR |
| ERG | transcription regulator | | 1,80E-04 | BIRC3,ETS1,ICAM1,ITGA6,NDRG1,PLAU,VIM |
| SAV1 | other | | 1,83E-04 | CCND1,CXCL2,VIM |
| STAP2 | other | | 1,83E-04 | CCND1,CXCL2,MYC |
| mir-486 | microRNA | | 1,83E-04 | DCBLD2,EMP1,UBASH3B |
| S1PR1 | G-protein coupled receptor | | 1,83E-04 | CCND1,ICAM1,JUNB |
| farnesol | chemical reagent | | 1,83E-04 | ATF3,PHLDA1,PHLDA2 |
| 7-ketocholesterol | chemical - endogenous mammalian | | 1,83E-04 | CYP1A1,EGR1,ICAM1 |
| TEX11 | other | | 1,86E-04 | CCND1,FOS |
| DACT3 | other | | 1,86E-04 | CCND1,MYC |
| proanthocyanidin derivative | chemical - other | | 1,86E-04 | CYP1A1,JUN |
| RAB11FIP3 | other | | 1,86E-04 | FOS,JUN |
| CDC25B | phosphatase | | 1,86E-04 | CCND1,MYC |
| PHLDA1 | other | | 1,86E-04 | ITGA2,ITGA6 |
| SNRK | kinase | | 1,86E-04 | CCND1,MYC |
| CFL1 | other | | 1,86E-04 | ICAM1,JUN |
| SLC9A3R2 | transporter | | 1,86E-04 | CCND1,MYC |
| carboxyamido-triazole | chemical drug | | 1,86E-04 | CCND1,FOS |
| ibotenic acid | chemical toxicant | | 1,86E-04 | FOS,JUN |
| C25-140 | chemical reagent | | 1,86E-04 | ICAM1,TNFAIP3 |
| yohimbine | chemical drug | | 1,86E-04 | EGR1,FOS |
| KMT2A | transcription regulator | | 1,88E-04 | EGR1,ETS1,IGFBP4,KLF6,MYC |
| Notch | group | | 1,89E-04 | AFP,AGT,ANKRD1,DUSP1,MYC,PLAU |
| miR-100-3p (miRNAs w/seed AAGCUUG) | mature microRNA | | 1,90E-04 | AHSG,BHLHE40,CXCL2,ERRFI1,MDM2,NDRG1,OAT,PARD6B,PDZK1,ZBTB43 |
| DGCR8 | enzyme | | 1,91E-04 | CCND1,EGR1,FOS,MYC |
| FGF21 | growth factor | | 1,91E-04 | EGR1,FOS,LDLR,SERPINE1 |
| TNFAIP3 | enzyme | | 1,91E-04 | BIRC3,ICAM1,NFKBIA,TNFAIP3 |
| MAP2K5 | kinase | | 1,91E-04 | CCND1,FOS,JUN,LDLR |
| TIMP3 | other | Inhibited | 1,91E-04 | FOS,JUN,SERPINE1,VIM |
| candesartan | chemical drug | | 1,91E-04 | AGT,FOS,ICAM1,SERPINE1 |
| valsartan | chemical drug | | 1,91E-04 | AGT,FOS,ICAM1,SERPINE1 |
| YAP1 | transcription regulator | Activated | 1,94E-04 | ANKRD1,CCND1,EGR1,FOS,ICAM1,MYC,SERPINE1,VIM |
| Histone h4 | group | | 1,96E-04 | AGT,AKAP12,CCND1,FOS,ICAM1,TNFAIP3 |
| methamphetamine | chemical drug | | 1,97E-04 | EGR1,FOS,ICAM1,JUN,MYC |
| PRKAA1 | kinase | | 2,03E-04 | ATF3,BIRC3,IGFBP4,NFKBIA,OAT,RGS2 |
| GFI1 | transcription regulator | | 2,03E-04 | ETS1,ICAM1,JUN,MAFF,MYC,NFKBIA |
| daidzein | chemical drug | | 2,07E-04 | AKR1C3,CCND1,CYP1A1,LDLR,MYC |
| Aldose Reductase | group | | 2,12E-04 | CCND1,ICAM1,MYC |
| Collagen type IV | complex | | 2,12E-04 | MYC,PLAU,SERPINE1 |
| SP100 | transcription regulator | | 2,12E-04 | FOS,ICAM1,PLAU |
| GDF15 | growth factor | | 2,12E-04 | CCND1,PLAU,SERPINE1 |
| SMARCE1 | transcription regulator | | 2,12E-04 | CYLD,FOS,MYC |
| A-Fos | chemical reagent | | 2,12E-04 | DUSP6,IER3,MYC |
| PCDH11Y | other | | 2,12E-04 | CCND1,JUN,MYC |
| PD 169316 | chemical - kinase inhibitor | | 2,12E-04 | BIRC3,CCND1,VIM |
| 2-mercaptoacetate | chemical drug | | 2,12E-04 | CXCL2,ICAM1,PLAU |
| PD 153035 | chemical drug | | 2,12E-04 | CCND1,FOS,ITGA6 |
| acetaldehyde | chemical - endogenous mammalian | | 2,12E-04 | FOS,JUN,MYC |
| IL15 | cytokine | | 2,15E-04 | CCND1,CXCL2,DYRK1A,ETS1,JUN,JUNB,MYC,NFKBIA,TNFRSF10B,ZFP36 |

| | | | | |
|---|---|---|---|---|
| RARA | ligand-dependent nuclear receptor | | 2,16E-04 | ALDH1A1,CCND1,DUSP1,EGR1,FOS,JUN,MXD4,MYC,TNFRSF10B |
| NCOR1 | transcription regulator | | 2,18E-04 | AFP,CCND1,DUSP1,NDRG1,NR1D1 |
| 26s Proteasome | complex | | 2,18E-04 | BIRC3,ICAM1,JUNB,MYC,TXNIP,VIM |
| SMAD7 | transcription regulator | | 2,18E-04 | HDAC9,ICAM1,ITGA6,MYC,NFKBIA,SERPINE1 |
| MYCN | transcription regulator | | 2,18E-04 | ALDH1A1,CCND1,ITGA2,MDM2,NDRG1,SERPINE1,TNFRSF10B,VIM |
| IGFBP2 | other | | 2,22E-04 | CCND1,IGFBP4,ITGA6,MYC |
| ADM | other | | 2,22E-04 | AGT,CXCL2,FOS,ICAM1 |
| STK11 | kinase | | 2,29E-04 | ATF3,CCND1,FGG,JUNB,MYC,NR1D1,SERPINE1,TNFAIP3 |
| IL5 | cytokine | | 2,34E-04 | DUSP6,EGR1,ICAM1,IER2,IER3,MYC,NDRG1,VIM |
| miR-4302 (miRNAs w/seed CAGUGUG) | mature microRNA | Inhibited | 2,37E-04 | ATF3,DYRK1A,ICAM1,KLF6,NFKBIA,PI3,POLR2J2/POLR2J3,SPATA2,YOD1,ZNF441,ZNF627,ZNF844 |
| L-methionine | chemical - endogenous mammalian | | 2,39E-04 | CXCL2,IGFBP4,NFKBIA,RHOB |
| NKX3-1 | transcription regulator | | 2,39E-04 | CTSV,ETS1,FOS,MYC |
| salirasib | chemical drug | | 2,39E-04 | ATF3,BHLHE40,PLAU,SERPINE1 |
| TEAD4 | transcription regulator | | 2,39E-04 | CCND1,JUN,JUNB,NFKBIZ,PLK2 |
| clofibrate | chemical drug | | 2,39E-04 | CCND1,CYP1A1,FABP1,LDLR,MYC |
| NFE2L2 | transcription regulator | | 2,44E-04 | AGT,ATF3,CXCL2,FABP1,ICAM1,MAFF,OAT,SERPINA6,SERPINE1,TTR |
| MAPK12 | kinase | | 2,45E-04 | CCND1,FOS,JUN |
| PTAFR | G-protein coupled receptor | | 2,45E-04 | CXCL2,MYC,VIM |
| imiquimod | chemical drug | | 2,51E-04 | BCL10,CXCL2,FOS,ICAM1,JUNB |
| IL10 | cytokine | | 2,52E-04 | CTSV,CXCL2,DUSP1,FOS,HDAC9,ICAM1,IGFBP4,JUN,PDZK1,ZFP36 |
| CD3 | complex | Activated | 2,55E-04 | BIRC3,CCND1,DYRK1A,ETS1,FOS,ICAM1,JUN,JUNB,MYC,NFKBIA,TNFAIP3,ZFP36 |
| oblimersen | biologic drug | Activated | 2,57E-04 | ATF3,DUSP1,KLF10,MAFF |
| CAT | enzyme | | 2,57E-04 | AGT,SERPINE1,TNFRSF10B,VIM |
| SFRP1 | transmembrane receptor | | 2,57E-04 | CCND1,MYC,SERPINE1,VIM |
| IL2RG | transmembrane receptor | | 2,57E-04 | ETS1,FOS,JUN,MYC |
| Ccl2 | cytokine | | 2,57E-04 | CTSV,CXCL2,ETS1,TBX3 |
| enalapril | biologic drug | | 2,57E-04 | AGT,IER3,MYC,SERPINE1 |
| colchicine | chemical drug | | 2,57E-04 | ATF3,FOS,JUN,MDM2 |
| benzo(a)pyrene | chemical toxicant | | 2,60E-04 | CYP1A1,FOS,IER3,JUN,MAFF,MDM2,SERPINE1 |
| alitretinoin | chemical drug | | 2,60E-04 | CCND1,DUSP1,FABP1,FOS,PLAU,VIM |
| phosphate | chemical - endogenous mammalian | | 2,63E-04 | AHSG,AXL,DUSP6,EGR1,SPRY4 |
| TCR | complex | Activated | 2,69E-04 | BIRC3,CCND1,EGR1,FOS,JUN,JUNB,MYC,NFKBIA,TNFAIP3 |
| MKNK1 | kinase | Activated | 2,75E-04 | ETS1,FOS,MYC,TXNIP,VIM |
| miR-34a-3p (miRNAs w/seed AAUCAGC) | mature microRNA | Inhibited | 2,75E-04 | ANKRD1,DUSP1,EMP1,ETS1,FOS,ITGA6,JUN,MDM2,MXD1,TXNIP |
| PTPRJ | phosphatase | | 2,75E-04 | CXCL2,FOS,NFKBIZ,ZC3H12A |
| PTPN6 | phosphatase | | 2,75E-04 | CCND1,ETS1,MYC,VIM |
| LEF1 | transcription regulator | Activated | 2,78E-04 | CCND1,GEM,MXD1,MYC,PHLDA1,VIM |
| trans-cinnamaldehyde | chemical drug | | 2,80E-04 | EGR1,MDM2,SERPINE1 |
| Pkg | group | | 2,80E-04 | EGR1,FOS,JUN |
| Fibrin | complex | | 2,80E-04 | ICAM1,PLAU,SERPINE1 |
| NADPH oxidase | complex | | 2,80E-04 | FOS,JUN,SERPINE1 |
| CTR9 | other | | 2,80E-04 | CYP1A1,IGFBP4,MYC |
| WDR5 | transcription regulator | | 2,80E-04 | MDM2,MYC,VIM |
| HTATIP2 | transcription regulator | | 2,80E-04 | CCND1,FOS,MYC |
| IL2RA | transmembrane receptor | | 2,80E-04 | FOS,JUN,MYC |
| CX-5461 | chemical drug | | 2,80E-04 | IER3,MDM2,PLK2 |
| PD184352 | chemical drug | | 2,80E-04 | CCND1,EGR1,FOS |
| pifithrin alpha | chemical reagent | | 2,80E-04 | CYP1A1,MDM2,TNFRSF10B |
| lipoarabinomannan | chemical - endogenous non-mammalian | | 2,80E-04 | CXCL2,ICAM1,IER3 |
| diallyl trisulfide | chemical - endogenous non-mammalian | | 2,80E-04 | FOS,JUN,MDM2 |
| STAT4 | transcription regulator | | 2,82E-04 | CXCL2,ERRFI1,IER3,MYC,NDRG1,PER1,SERPINE1 |
| PAX3-FOXO1 | fusion gene/product | | 2,82E-04 | AKAP12,AXL,CCND1,DUSP1,IER3,MDM2,TSC22D2 |
| miR-7110-3p (miRNAs w/seed CUCUCUC) | mature microRNA | | 2,83E-04 | CCND1,CXCL2,DUSP6,EMP1,FABP1,GJB1,JUN,MAFF,MDM2,POLR2J2/POLR2J3,PRR15L,SERINC5,SMIM14,TENT5A,ZNF608,ZNF844 |
| Nfat (family) | group | | 2,88E-04 | ATF3,CCND1,ICAM1,KLF6,SERPINE1 |
| hexachlorobenzene | chemical toxicant | | 2,95E-04 | IER3,MYC,TSPAN8,VIM |
| SERPINF1 | other | | 2,95E-04 | CCND1,MYC,NFKBIA,VIM |
| GNRH1 | other | | 2,95E-04 | EGR1,FOS,JUN,JUNB |
| TNFRSF1B | transmembrane receptor | | 3,01E-04 | CXCL2,ICAM1,JUN,SERPINE1,TNFAIP3 |
| RXRA | ligand-dependent nuclear receptor | | 3,08E-04 | ALDH1A1,CCND1,FABP1,FGG,FOS,IER3,NR1D1,TNFRSF10B |
| EGR2 | transcription regulator | | 3,08E-04 | BHLHE40,EGR1,FOS,GJB1,JUN,MYC |
| RNF187 | enzyme | | 3,08E-04 | CCND1,JUN |
| Gpcr | group | | 3,08E-04 | FOS,JUN |
| sGC | complex | | 3,08E-04 | EGR1,FOS |
| FZD7 | G-protein coupled receptor | | 3,08E-04 | CCND1,MYC |
| MCF2 | other | | 3,08E-04 | CCND1,ITGA2 |
| rigosertib | chemical drug | | 3,08E-04 | CCND1,MYC |
| RBM14 | transcription regulator | | 3,08E-04 | CCND1,MYC |
| PSAP | enzyme | | 3,08E-04 | BIRC3,CCND1 |
| KIDINS220 | transcription regulator | | 3,08E-04 | EGR1,FOS |
| NME3 | kinase | | 3,08E-04 | ITGA2,VIM |
| SH2B2 | other | | 3,08E-04 | FOS,MYC |
| Muc1 | transmembrane receptor | | 3,08E-04 | CCND1,CXCL2 |
| CLDN2 | other | | 3,08E-04 | CCND1,ITGA2 |
| scoparone | chemical - endogenous non-mammalian | | 3,08E-04 | CCND1,MYC |
| dimaprit | chemical drug | | 3,08E-04 | ICAM1,LDLR |
| butylated hydroxytoluene | chemical toxicant | | 3,08E-04 | FOS,JUN |
| TCF12 | transcription regulator | | 3,15E-04 | ASB2,JUN,KLF6,VGF,ZC3H12A |
| trichloroethylene | chemical toxicant | | 3,15E-04 | EGR1,MDM2,RHOB,TXNIP |
| benzene | chemical toxicant | | 3,15E-04 | EGR1,MDM2,RHOB,TXNIP |
| isoproterenol | chemical drug | | 3,18E-04 | AGT,ATF3,CYP1A1,FOS,JUN,JUNB |
| Ikb | group | | 3,19E-04 | CCND1,ICAM1,TNFAIP3 |
| NFAT (complex) | complex | | 3,19E-04 | ATF3,ICAM1,SERPINE1 |
| PDGF-AA | complex | | 3,19E-04 | FOS,LDLR,VIM |
| RNF20 | enzyme | | 3,19E-04 | FOS,MYC,RHOB |
| MAP3K3 | kinase | | 3,19E-04 | FOS,JUN,TNFAIP3 |
| IGFBP5 | other | | 3,19E-04 | BHLHE40,CCND1,SERPINE1 |
| RACK1 | enzyme | | 3,19E-04 | FOS,MDM2,MYC |
| TGFB1I1 | transcription regulator | | 3,19E-04 | FABP1,FOS,SERPINE1 |
| LCAT | enzyme | | 3,19E-04 | CCND1,LDLR,MYC |
| THZ2 | chemical - kinase inhibitor | | 3,19E-04 | CCND1,MDM2,MYC |
| chelerythrine | chemical drug | | 3,19E-04 | ICAM1,MDM2,NFKBIA |
| diosgenin | chemical - endogenous non-mammalian | | 3,19E-04 | CCND1,MDM2,SERPINE1 |
| sirolimus | chemical drug | | 3,23E-04 | CCND1,EGR1,FOS,ICAM1,JUN,JUNB,MYC,PLAU,SERPINE1,VIM,ZFP36 |
| UCP1 | transporter | | 3,29E-04 | ANKRD1,ASB2,CTSV,MYC,REEP6,SERPINE1 |
| miR-188-3p (miRNAs w/seed UCCCACA) | mature microRNA | Inhibited | 3,31E-04 | ABL2,ARRDC3,CSRNP1,EMP1,H3C14,H3C15,IER3,IGFBP4,ITGA2,JUN,MAFF,SMIM14,ZBTB20,ZNF441,ZNF627,ZNF844 |
| PTK2 | kinase | | 3,37E-04 | BIRC3,CCND1,FOS,LDLR |
| KLF5 | transcription regulator | | 3,37E-04 | CCND1,DUSP1,LAMA1,MYC |
| XBP1 | transcription regulator | | 3,57E-04 | AFP,CXCL2,ETS1,ICAM1,MYC,PHLDA2,TTR |
| propylthiouracil | chemical drug | | 3,58E-04 | AKR1C3,CXCL2,ITGA6,JUN,PHLDA1 |
| fatty acid | chemical - endogenous mammalian | | 3,58E-04 | AHSG,LDLR,NFKBIA,SERPINE1,TNFRSF10B |
| adenosine | chemical - endogenous mammalian | | 3,59E-04 | FOS,ICAM1,SERPINE1,TXNIP |
| IL1R1 | transmembrane receptor | | 3,59E-04 | CXCL2,EGR1,FOS,ICAM1 |
| diphenyleneiodonium | chemical reagent | | 3,59E-04 | AGT,CXCL2,FOS,SERPINE1 |
| phenylbutazone | chemical drug | | 3,59E-04 | CCND1,IER3,TSPAN8,VIM |
| thioctic acid | chemical drug | | 3,59E-04 | FOS,ICAM1,JUN,MYC |
| BIRC3 | enzyme | | 3,61E-04 | BIRC3,ICAM1,NFKBIA |
| plumbagin | chemical toxicant | | 3,61E-04 | BIRC3,CCND1,TNFRSF10B |
| SLPI | other | | 3,61E-04 | CCND1,ICAM1,NFKBIA |
| BCL2L11 | other | | 3,61E-04 | CCND1,CXCL2,HDAC9 |
| TWIST1 | transcription regulator | | 3,62E-04 | ALDH1A1,AXL,ETS1,FOS,IGFBP4,VIM |
| NR1I3 | ligand-dependent nuclear receptor | | 3,74E-04 | ALDH1A1,ICAM1,MAFF,MDM2,MYC |
| mir-181 | microRNA | | 3,83E-04 | CYLD,DUSP6,ICAM1,KLF6 |
| N-acetylmuramyl-L-alanyl-D-isoglutamine | chemical - endogenous non-mammalian | | 3,83E-04 | CXCL2,ETS1,ICAM1,NFKBIA |
| miR-337-5p (miRNAs w/seed AACGGCU) | mature microRNA | | 3,98E-04 | AGT,EMP1,EPHX2,H3C14,H3C15,ZFP36 |
| NfkB1-RelA | complex | | 4,06E-04 | CCND1,ICAM1,MYC |
| CHI3L1 | enzyme | | 4,06E-04 | BHLHE40,EMP1,SERPINE1 |
| CDC73 | other | | 4,06E-04 | CCND1,IGFBP4,MYC |
| MTA2 | transcription regulator | | 4,06E-04 | JUN,MDM2,MYC |
| XIAP | enzyme | | 4,06E-04 | CCND1,MYC,SERPINE1 |
| CD9 | other | | 4,06E-04 | ITGA2,ITGA6,MYC |
| KAT2A | enzyme | | 4,06E-04 | CCND1,JUN,TNFRSF10B |
| ACE2 | peptidase | | 4,06E-04 | AGT,CXCL2,SERPINE1 |
| HMGB2 | transcription regulator | | 4,06E-04 | CCND1,CXCL2,PHLDA1 |
| PRKAA2 | kinase | | 4,07E-04 | EGR1,IGFBP4,NFKBIA,OAT,VIM |
| ciprofloxacin | chemical drug | | 4,07E-04 | CTSV,ICAM1,LDLR,SPRED1,VIM |
| miR-5003-5p (miRNAs w/seed CACAACA) | mature microRNA | | 4,21E-04 | ANKRD1,CCND1,ERRFI1,ETS1,GSTA2,IER2,KRT6A,NDRG1,PDZK1,ZBTB43,ZNF554 |
| ETV6-RUNX1 | fusion gene/product | | 4,25E-04 | ICAM1,IER3,KLF6,MDM2,MYC,PPP1R15A,TNFRSF10B,ZBTB20 |
| PROCR | other | | 4,55E-04 | CCND1,ICAM1,MYC |
| NTF3 | growth factor | | 4,55E-04 | FOS,JUN,VGF |
| DTX1 | transcription regulator | | 4,55E-04 | DUSP6,EGR1,SPRY4 |
| WR 1065 | chemical drug | | 4,55E-04 | MDM2,MYC,TNFRSF10B |
| miR-4269 (and other miRNAs w/seed CAGGCAC) | mature microRNA | | 4,56E-04 | AGT,AHSG,BHLHE40,EGR1,EMP1,GSTA2,LDLR,MAFF,REEP6,RHOB,SERINC5,SMIM14,TENT5A,ZNF608 |
| 3-deazaneplanocin | chemical drug | | 4,60E-04 | KLF6,MYC,PLAU,PPP1R15A |
| F2RL1 | G-protein coupled receptor | | 4,60E-04 | CXCL2,DUSP6,FOS,TXNIP |
| DDX5 | enzyme | | 4,60E-04 | CCND1,FOS,JUN,MYC |
| gentamicin C | chemical drug | Activated | 4,60E-04 | BIRC3,IER3,MYC,VIM |
| Tcf 1/3/4 | group | | 4,60E-04 | AHSG,TTR |
| vanillyl-N-nonylamide | chemical drug | | 4,60E-04 | ATF3,CCND1 |
| LINC00842 | other | | 4,60E-04 | SERPINE1,VIM |
| ZMIZ1 | transcription regulator | | 4,60E-04 | MYC,SERPINE1 |
| Crnde | other | | 4,60E-04 | CCND1,MYC |
| BPIFB1 | other | | 4,60E-04 | CCND1,NFKBIA |
| ghrelin | biologic drug | | 4,60E-04 | EGR1,FOS |
| PLSCR1 | enzyme | | 4,60E-04 | CCND1,MYC |
| SLC7A11-AS1 | other | | 4,60E-04 | CCND1,JUN |
| EGFL7 | other | | 4,60E-04 | ICAM1,NFKBIA |
| PTPRR | phosphatase | | 4,60E-04 | FOS,JUN |
| CARD14 | other | | 4,60E-04 | BCL10,CXCL2 |
| TRIM33 | transcription regulator | | 4,60E-04 | MYC,SERPINE1 |
| F2RL3 | G-protein coupled receptor | | 4,60E-04 | EGR1,FOS |
| STX2 | transporter | | 4,60E-04 | CCND1,MYC |
| YWHAB | other | | 4,60E-04 | CCND1,DUSP1 |
| HIVEP2 | transcription regulator | | 4,60E-04 | FOS,MYC |
| RASA1 | transporter | | 4,60E-04 | FOS,PLAU |
| CIMO | chemical reagent | | 4,60E-04 | CCND1,ICAM1 |
| famotidine | chemical drug | | 4,60E-04 | CXCL2,ICAM1 |
| dibenzoylmethane | chemical toxicant | | 4,60E-04 | CCND1,CYP1A1 |
| saquinavir | chemical drug | | 4,60E-04 | MYC,NFKBIA |
| catechol | chemical - endogenous mammalian | | 4,60E-04 | FOS,JUN |
| apamin | chemical toxicant | | 4,60E-04 | FOS,JUN |
| TGFB3 | growth factor | Activated | 4,78E-04 | BHLHE40,ETS1,FOS,JUN,SERPINE1 |
| BMP7 | growth factor | | 4,78E-04 | ICAM1,IGFBP4,NDRG1,RGS2,SERPINE1,VIM |
| BSCL2 | other | | 4,88E-04 | AKR1C3,ANKRD1,CCND1,IER3 |
| NRG2 | growth factor | | 4,88E-04 | EGR1,IER2,IER3,JUNB |
| SNAI2 | transcription regulator | | 4,88E-04 | ANKRD1,AXL,CCND1,VIM |
| E2F3 | transcription regulator | | 5,08E-04 | AKR1C3,CCND1,H3C4,MYC,SERPINE1,TENT5A |
| choline fenofibrate | chemical drug | | 5,08E-04 | NFKBIA,NR1D1,SERPINE1 |
| REN | peptidase | | 5,08E-04 | AGT,FOS,ICAM1 |
| PRKAR2B | kinase | | 5,08E-04 | BIRC3,EGR1,FOS |
| CCR1 | G-protein coupled receptor | | 5,08E-04 | CTSV,FOS,VIM |
| GRN | growth factor | | 5,08E-04 | CCND1,CXCL2,ICAM1 |
| imipramine | chemical drug | | 5,08E-04 | EGR1,FABP1,FOS |
| arsenic | chemical toxicant | | 5,08E-04 | BIRC3,EPHX2,FOS |
| MYOD1 | transcription regulator | | 5,18E-04 | AGT,ANKRD1,ASB2,DUSP1,JUNB,KLF6,SELENOW |
| diphtheria toxin | chemical - endogenous non-mammalian | | 5,18E-04 | DUSP6,FOS,PLAU,SPRED1,TNFAIP3 |
| Tlr | group | Activated | 5,18E-04 | DUSP1,ICAM1,NFKBIA,TNFAIP3,ZC3H12A |
| TGFB2 | growth factor | | 5,18E-04 | ALDH1A1,BHLHE40,EGR1,FOS,SERPINE1 |
| 5-hydroxytryptamine | chemical - endogenous mammalian | Activated | 5,18E-04 | EGR1,FOS,ICAM1,MYC,SERPINE1 |
| azoxymethane | chemical toxicant | | 5,18E-04 | AGT,CCND1,CXCL2,MYC |
| NR5A1 | ligand-dependent nuclear receptor | | 5,48E-04 | AFP,ETS1,GSTA2,VIM |
| HOXA9 | transcription regulator | | 5,55E-04 | ALDH1A1,IER2,JUN,JUNB,MYC,VIM |
| ROCK1 | kinase | | 5,64E-04 | CCND1,ETS1,ICAM1 |
| SUB1 | transcription regulator | | 5,64E-04 | CCND1,FOS,MDM2 |
| HOXB13 | transcription regulator | | 5,64E-04 | CCND1,MYC,NFKBIA |
| S100A6 | transporter | | 5,64E-04 | ATF3,CXCL2,TNFAIP3 |
| EPOR | transmembrane receptor | | 5,64E-04 | JUN,JUNB,MYC |
| NPPA | other | | 5,64E-04 | CCND1,DUSP1,EGR1 |
| stearic acid | chemical - endogenous mammalian | | 5,64E-04 | ATF3,ICAM1,NFKBIA |
| MRTFA | transcription regulator | Activated | 5,71E-04 | ANKRD1,EGR1,FOS,ICAM1,ITGA6,SERPINE1 |
| KLF2 | transcription regulator | Inhibited | 5,71E-04 | CXCL2,JUNB,MYC,NDRG1,NFKBIA,SERPINE1 |
| GnRH analog | biologic drug | | 5,76E-04 | ATF3,BHLHE40,DUSP1,FOS,JUN,NDRG1,NFKBIA,PHLDA2 |
| caffeine | chemical drug | | 5,80E-04 | EGR1,FOS,JUNB,VIM |
| HNF1B | transcription regulator | | 6,03E-04 | AFP,BHLHE40,DUSP1,PLAU,TTR |
| miR-6826-3p (miRNAs w/seed UCCCCUC) | mature microRNA | Inhibited | 6,07E-04 | BIRC3,EMP1,H2BC5,H3C14,H3C15,ICAM1,ITGA2,JUNB,KLF6,LCA5,NUAK2,PALD1,PHLDA1,PRR15L,RGS2,TNFAIP3,ZNF627 |
| CCND1 | transcription regulator | Activated | 6,11E-04 | AFAP1,CCND1,EPHA2,ERRFI1,IER3,MAFF,MYC,RGS2 |
| MSTN | growth factor | | 6,13E-04 | CTSV,FOS,KLF10,VIM |
| OGA | enzyme | | 6,20E-04 | CCND1,HIVEP2,IGFBP4,ITGA2,JUN,MDM2,PLAU,RHOB,VIM |
| miR-1911-5p (miRNAs w/seed GAGUACC) | mature microRNA | Inhibited | 6,23E-04 | AKAP12,ATF3,AXL,BIRC3,CCND1,EGR1,FABP1,JUNB |
| KITLG | growth factor | | 6,24E-04 | AXL,CXCL2,EGR1,FOS,LDLR,NFKBIA,TXNIP |
| GC-GCR dimer | complex | | 6,24E-04 | AGT,DUSP1,NFKBIA |
| TCF7L1 | transcription regulator | | 6,24E-04 | ATF3,JUN,TBX3 |
| leucine | chemical - endogenous mammalian | | 6,24E-04 | ATF3,FOS,PPP1R15A |
| C1QA | other | | 6,24E-04 | EGR1,SERPINE1,VIM |
| SFRP4 | transmembrane receptor | | 6,24E-04 | CCND1,MYC,VIM |
| urethane | chemical toxicant | | 6,24E-04 | CCND1,CYP1A1,MYC |
| miR-6883-3p (miRNAs w/seed UCCCUAU) | mature microRNA | Inhibited | 6,31E-04 | BCL10,BHLHE40,BIRC3,CYP1A1,ETS1,ITGA2,MAFF,TBX3,ZNF441 |
| patulin | chemical toxicant | | 6,42E-04 | ATF3,CYP1A1 |
| C1orf61 | other | | 6,42E-04 | CCND1,VIM |
| ATE1 | enzyme | | 6,42E-04 | CCND1,JUN |
| sphingomyelinase | group | | 6,42E-04 | JUN,MYC |
| RAP1GAP | other | | 6,42E-04 | CCND1,FOS |
| AGR2 | other | | 6,42E-04 | EGR1,FOS |
| CBR3-AS1 | other | | 6,42E-04 | CCND1,VIM |
| DAB2 | other | | 6,42E-04 | CCND1,FOS |
| PDE3A | enzyme | | 6,42E-04 | CCND1,DUSP1 |
| MAD2L2 | enzyme | | 6,42E-04 | EGR1,VIM |
| MED13L | other | | 6,42E-04 | ANKRD1,MYC |
| AP20187 | chemical reagent | | 6,42E-04 | EGR1,FOS |
| nicorandil | chemical drug | | 6,42E-04 | ATF3,JUN |
| temsirolimus | chemical drug | | 6,42E-04 | CCND1,MYC |
| volinanserin | chemical drug | | 6,42E-04 | EGR1,FOS |
| gliotoxin | chemical toxicant | | 6,42E-04 | BIRC3,IER3 |
| butylidenephthalide | chemical reagent | | 6,42E-04 | AXL,VIM |
| diazepam | chemical drug | | 6,42E-04 | FOS,PER1 |
| manidipine | chemical drug | | 6,42E-04 | FOS,JUN |
| miR-508-3p inhibitor | chemical reagent | | 6,42E-04 | SERPINE1,VIM |
| cyclosporin | biologic drug | | 6,42E-04 | CCND1,ICAM1 |
| RIPK2 | kinase | | 6,47E-04 | CXCL2,FABP1,ICAM1,LDLR |
| JAK1 | kinase | | 6,47E-04 | FOS,ICAM1,JUNB,TNS4 |
| SB-431542 | chemical reagent | | 6,50E-04 | CCND1,CYP1A1,FOS,MYC,SERPINE1 |
| CLOCK | transcription regulator | | 6,53E-04 | BHLHE40,CCND1,ICAM1,JUNB,NR1D1,PER1,SERINC5 |
| PELP1 | other | | 6,74E-04 | CCND1,FOS,MYC,NDRG1,PI3 |
| CDK4/6 | group | | 6,83E-04 | AFAP1,CCND1,EPHA2,ERRFI1 |
| ITGB3 | transmembrane receptor | | 6,83E-04 | FOS,LDLR,PLAU,SERPINE1 |
| entinostat | chemical drug | | 6,83E-04 | CCND1,DUSP1,SERPINE1,TNFRSF10B |
| H2AZ1 | other | | 6,88E-04 | AFP,CCND1,TTR |
| VAV1 | transcription regulator | | 6,88E-04 | CCND1,FOS,JUN |
| PTX3 | other | | 6,88E-04 | CXCL2,FOS,JUN |
| ACKR3 | G-protein coupled receptor | | 6,88E-04 | AKR1C3,AXL,CCND1 |
| ramipril | chemical drug | | 6,88E-04 | AGT,ITGA2,LAMA1 |
| proteasome inhibitor PSI | chemical - protease inhibitor | | 6,88E-04 | CXCL2,CYP1A1,TNFRSF10B |
| vinblastine | chemical drug | | 6,88E-04 | CYP1A1,FOS,IGFBP4 |
| lithium chloride | chemical drug | | 6,99E-04 | CCND1,FOS,MYC,PER1,TNFRSF10B |
| STAT5a/b | group | | 7,25E-04 | CCND1,DUSP1,EMP1,FOS,MYC |
| Fc gamma receptor | group | | 7,56E-04 | CCND1,ICAM1,TNFAIP3 |

| | | | | |
|---|---|---|---|---|
| ALDH1A2 | enzyme | | 7,56E-04 | ALDH1A1,ATF3,IGFBP4 |
| PRKG1 | kinase | | 7,56E-04 | CCND1,FOS,SERPINE1 |
| EIF2AK4 | kinase | | 7,56E-04 | ATF3,EGR1,PPP1R15A |
| PRLR | transmembrane receptor | | 7,56E-04 | AKR1C3,GJB1,MYC |
| UXT | transcription regulator | | 7,56E-04 | NFKBIA,TNFAIP3,TNFRSF10B |
| irbesartan | chemical drug | | 7,56E-04 | FOS,ICAM1,SERPINE1 |
| KN 93 | chemical - kinase inhibitor | | 7,56E-04 | CCND1,ETS1,FOS |
| RNF31 | enzyme | | 7,98E-04 | CCND1,CXCL2,IGFBP4,PDZK1 |
| LCK | kinase | | 7,98E-04 | EGR1,FOS,JUN,MYC |
| KLF11 | transcription regulator | | 8,06E-04 | AGT,ITGA2,MYC,PLAU,SERPINE1 |
| GJA1 | transporter | | 8,29E-04 | CCND1,ICAM1,VIM |
| ZBTB7A | transcription regulator | | 8,29E-04 | FOS,MDM2,MYC |
| SSRP1 | transcription regulator | | 8,29E-04 | EGR1,MDM2,PLAU |
| PDPK1 | kinase | | 8,29E-04 | CCND1,MYC,SERPINE1 |
| VTN | other | | 8,29E-04 | BHLHE40,PLAU,SERPINE1 |
| taurine | chemical - endogenous mammalian | | 8,29E-04 | AGT,AXL,VIM |
| SMARCA5 | transcription regulator | | 8,35E-04 | EPHA2,IGFBP4,PHLDA1,PLK2,ZNF442 |
| PTTG1 | transcription regulator | | 8,40E-04 | CCND1,MDM2,MYC,VIM |
| Vhl | complex | | 8,53E-04 | BIRC3,CCND1 |
| L-type Calcium Channel | complex | | 8,53E-04 | EGR1,FOS |
| ARHGEF1 | other | | 8,53E-04 | CCND1,FOS |
| EFEMP1 | enzyme | | 8,53E-04 | SERPINE1,VIM |
| LPA | other | | 8,53E-04 | ICAM1,SERPINE1 |
| ARAF | kinase | | 8,53E-04 | CCND1,FOS |
| WNT7B | other | | 8,53E-04 | CCND1,MYC |
| PROKR1 | G-protein coupled receptor | | 8,53E-04 | MYC,VIM |
| PBX2 | transcription regulator | | 8,53E-04 | FOS,PLAU |
| SERPINH1 | other | | 8,53E-04 | SERPINE1,VIM |
| MM-401 | chemical reagent | | 8,53E-04 | KLF6,MYC |
| R59949 | chemical - kinase inhibitor | | 8,53E-04 | FOS,MYC |
| buserelin | biologic drug | | 8,53E-04 | EGR1,FOS |
| artemisinin | chemical drug | | 8,53E-04 | CCND1,MDM2 |
| cordycepin | chemical drug | | 8,53E-04 | FOS,MYC |
| N,N-dimethylarginine | chemical - endogenous mammalian | | 8,53E-04 | ICAM1,SERPINE1 |
| tosyllysine chloromethyl ketone | chemical - protease inhibitor | | 8,53E-04 | CYP1A1,NFKBIA |
| PITX2 | transcription regulator | | 8,64E-04 | CCND1,EGR1,MYC,TBX3,VIM |
| filgrastim | biologic drug | | 8,75E-04 | ATF3,BIRC3,JUN,KLF6,LDLR,MAFF,PHLDA2,VIM,ZBTB20 |
| Calcineurin A | group | | 9,05E-04 | CCND1,FOS,JUN |
| daporinad | chemical drug | | 9,05E-04 | ALDH1A1,BHLHE40,SERPINE1 |
| CDK5 | kinase | | 9,05E-04 | CXCL2,JUN,TNFAIP3 |
| bryostatin 1 | chemical drug | | 9,05E-04 | BIRC3,ICAM1,MYC |
| CD28 | transmembrane receptor | Activated | 9,06E-04 | BHLHE40,CCND1,CXCL2,FOS,JUN,MYC,NFKBIA,TNFAIP3 |
| dinoprost | chemical - endogenous mammalian | | 9,27E-04 | CCND1,FOS,JUN,MYC |
| MAP2K6 | kinase | | 9,27E-04 | CCND1,JUN,LDLR,PLAU |
| RELB | transcription regulator | | 9,27E-04 | BIRC3,CCND1,MYC,NFKBIA |
| captopril | chemical drug | | 9,27E-04 | AGT,EPHX2,IER3,TSPAN8 |
| GNA12 | enzyme | | 9,73E-04 | FOS,ITGA6,MYC,VIM |
| GCG | other | | 9,73E-04 | FOS,IGFBP4,PDZK1,TENT5A |
| 1-methyl-4-phenyl-1,2,3,6-tetrahydropyridine | chemical toxicant | | 9,73E-04 | EGR1,EPHX2,FOS,JUN |
| ING1 | transcription regulator | | 9,86E-04 | AFP,MDM2,ZFP36 |
| diclofenac | chemical drug | | 9,86E-04 | CCND1,FOS,MYC |
| sodium orthovanadate | chemical reagent | | 9,86E-04 | CCND1,NFKBIA,SERPINE1 |
| KIN001-043 | chemical - kinase inhibitor | | 9,86E-04 | CCND1,MYC,TNS4 |
| indole-3-carbinol | chemical drug | | 9,86E-04 | CCND1,FOS,GSTA2 |
| SN-38 | chemical drug | | 9,86E-04 | BIRC3,MYC,TNFRSF10B |
| BCR (complex) | complex | | 9,89E-04 | CCND1,EGR1,FOS,JUNB,MYC |
| NR4A1 | ligand-dependent nuclear receptor | | 1,01E-03 | ATF3,CCND1,ICAM1,LDLR,MDM2,NFKBIA,SERPINE1 |
| MDM2 | transcription regulator | | 1,02E-03 | ATF3,CCND1,ETS1,MDM2 |
| VHL | transcription regulator | | 1,06E-03 | ALDH1A1,BHLHE40,CCND1,IER3,VIM |
| N-Ac-Leu-Leu-norleucinal | chemical - protease inhibitor | | 1,07E-03 | CCND1,ICAM1,IER3,NFKBIA |
| CRNDE | other | | 1,07E-03 | ABL2,DUSP6,EPHA2 |
| arginine | chemical - endogenous mammalian | | 1,07E-03 | AXL,EGR1,MYC |
| EIF4EBP1 | translation regulator | | 1,07E-03 | CCND1,EGR1,MYC |
| NLRP12 | other | | 1,07E-03 | CXCL2,ICAM1,JUN |
| GAPDH | enzyme | | 1,07E-03 | DUSP1,GEM,TXNIP |
| IGF1R | transmembrane receptor | Activated | 1,07E-03 | ANKRD1,EGR1,FOS,IGFBP4,MYC,PHLDA2,PLAU |
| ONECUT1 | transcription regulator | | 1,07E-03 | ABL2,AFP,GJB1,NFKBIA,NUAK2,OAT,TTR |
| L-cysteine | chemical - endogenous mammalian | | 1,09E-03 | FOS,MYC |
| Agtr1b | G-protein coupled receptor | | 1,09E-03 | AGT,FOS |
| 4-nonylphenol | chemical toxicant | | 1,09E-03 | FOS,MYC |
| C8 | complex | | 1,09E-03 | CXCL2,ICAM1 |
| Bcl9-Cbp/p300-Ctnnb1-Lef/Tcf | complex | | 1,09E-03 | CCND1,MYC |
| BLVRA | enzyme | | 1,09E-03 | JUN,TNFRSF10B |
| CTDSP1 | phosphatase | | 1,09E-03 | MYC,VIM |
| Ferritin | complex | | 1,09E-03 | ICAM1,NFKBIA |
| bicyclol | chemical drug | | 1,09E-03 | AFP,MYC |
| CD244 | transmembrane receptor | | 1,09E-03 | EGR1,FOS |
| GLRX | enzyme | | 1,09E-03 | CXCL2,ICAM1 |
| PSMB9 | peptidase | | 1,09E-03 | NFKBIA,TNFAIP3 |
| ATG16L1 | enzyme | | 1,09E-03 | CXCL2,NFKBIA |
| ING4 | transcription regulator | | 1,09E-03 | MYC,NFKBIA |
| CD160 | transmembrane receptor | | 1,09E-03 | FOS,JUN |
| SCUBE3 | other | | 1,09E-03 | SERPINE1,VIM |
| MCAM | other | | 1,09E-03 | ATF3,VIM |
| MPL | transmembrane receptor | | 1,09E-03 | FOS,MYC |
| C7 | other | | 1,09E-03 | CXCL2,ICAM1 |
| BRMS1 | transcription regulator | | 1,09E-03 | GJB1,PLAU |
| USP17L2 (includes others) | peptidase | | 1,09E-03 | ALDH1A1,MYC |
| DEK | transcription regulator | | 1,09E-03 | BIRC3,NFKBIA |
| asbestos | chemical toxicant | | 1,09E-03 | JUN,JUNB |
| capsazepine | chemical toxicant | | 1,09E-03 | FOS,TNFRSF10B |
| CITCO | chemical reagent | | 1,09E-03 | ALDH1A1,GSTA2 |
| acrylamide | chemical toxicant | | 1,09E-03 | FOS,JUN |
| cyclopentenone | chemical reagent | | 1,09E-03 | CCND1,ICAM1 |
| RP 73401 | chemical toxicant | | 1,09E-03 | CXCL2,EGR1 |
| JMF3086 | chemical reagent | | 1,09E-03 | CCND1,CXCL2 |
| I-BOP | chemical reagent | | 1,09E-03 | CCND1,ICAM1 |
| miR-133a-3p (and other miRNAs w/seed UUGGUCC) | mature microRNA | | 1,10E-03 | AFAP1,BCL10,BIRC3,CTSV,DUSP1,GEM,MAFF,NDRG1,PRR15L,SEPHS2,SMIM14,TENT5A,TNFRSF10B |
| ZEB1 | transcription regulator | | 1,12E-03 | ALDH1A1,PLAU,SERPINE1,VIM |
| fingolimod | chemical drug | Inhibited | 1,12E-03 | AXL,CXCL2,ICAM1,SERPINE1 |
| miR-126a-3p (and other miRNAs w/seed CGUACCG) | mature microRNA | | 1,13E-03 | ITGA6,PLK2,PMM1,SPRED1,TNFRSF10B |
| FOXM1 | transcription regulator | | 1,13E-03 | CCND1,FOS,JUN,MYC,VIM |
| mibolerone | chemical drug | | 1,13E-03 | CCND1,JUN,KLF10,LDLR,MYC |
| KDM1A | enzyme | | 1,14E-03 | AFP,AGT,IGFBP4,MYC,SERPINE1,ZBTB20 |
| miR-181a-5p (and other miRNAs w/seed ACAUUCA) | mature microRNA | Inhibited | 1,15E-03 | BHLHE40,DUSP6,ETS1,KLF6,PHLDA1,PLAU,SPRY4,TSC22D2,TSPAN8,YOD1,ZNF441,ZNF627,ZNF844 |
| CCAR2 | peptidase | | 1,16E-03 | CCND1,MDM2,NR1D1 |
| PLP1 | other | | 1,16E-03 | ATF3,CXCL2,PPP1R15A |
| CTF1 | cytokine | | 1,16E-03 | AGT,FOS,JUNB |
| ALOX5 | enzyme | | 1,16E-03 | CCND1,FABP1,MYC |
| TANK | other | | 1,16E-03 | CXCL2,NFKBIZ,ZC3H12A |
| FOSB | transcription regulator | | 1,16E-03 | CCND1,FOS,SERPINE1 |
| ELF4 | transcription regulator | | 1,16E-03 | CXCL2,DUSP1,MDM2 |
| histone deacetylase inhibitor | chemical drug | | 1,16E-03 | MYC,PHLDA2,TXNIP |
| IL12 (complex) | complex | | 1,17E-03 | FOS,ICAM1,ITGA6,JUN,MYC,PLAU |
| NFKB2 | transcription regulator | | 1,17E-03 | BIRC3,CCND1,FOS,MYC |
| ascorbic acid | chemical - endogenous mammalian | | 1,17E-03 | IGFBP4,ITGA2,NDRG1,SERPINE1 |
| CD44 | other | | 1,20E-03 | BIRC3,CCND1,JUN,MYC,PLAU,VIM |
| PPARA | ligand-dependent nuclear receptor | | 1,21E-03 | CCND1,CYP1A1,FABP1,FGG,FOS,MYC,NFKBIA,NR1D1,PHLDA1 |
| TCF3 | transcription regulator | | 1,22E-03 | ASB2,ATF3,CTSV,JUN,KLF6,MYC,SERINC5,ZC3H12A |
| GMNN | transcription regulator | | 1,23E-03 | DUSP1,DYRK1A,EPHA2,FABP1 |
| miR-4688 (and other miRNAs w/seed AGGGGCA) | mature microRNA | Inhibited | 1,24E-03 | AFAP1,ASB2,CCND1,CYP1A1,H3C14,H3C15,IER3,INAFM1,MAFF,MXD4,PER1,POLR2J2/POLR2J3,RHOB,SERINC5,SPATA2,SPRY4,UBASH3B,VGF,ZC3H12A,ZFP36 |
| STAT | group | | 1,26E-03 | AGT,FOS,JUNB |
| LHCGR | G-protein coupled receptor | | 1,26E-03 | CTSV,DUSP6,IGFBP4 |
| SERPINE1 | other | | 1,26E-03 | CXCL2,FOS,PLAU |
| FGFR3 | kinase | | 1,26E-03 | CCND1,DUSP6,FOS |
| TLR5 | transmembrane receptor | | 1,26E-03 | ATF3,CXCL2,ICAM1 |
| PAK2 | kinase | | 1,26E-03 | DUSP1,ETS1,MDM2 |
| mevalonic acid | chemical - endogenous mammalian | | 1,26E-03 | ICAM1,LDLR,SERPINE1 |
| tempol | chemical drug | | 1,26E-03 | CXCL2,ICAM1,SERPINE1 |
| ethylene glycol tetraacetic acid | chemical reagent | | 1,26E-03 | BIRC3,EGR1,FOS |
| thalidomide | chemical drug | | 1,26E-03 | BIRC3,ICAM1,SERPINE1 |
| SPIB | transcription regulator | Activated | 1,28E-03 | ATF3,EGR1,GEM,KLF6 |
| CFTR | ion channel | | 1,28E-03 | EPHX2,NFKBIA,NFKBIZ,TNFAIP3 |
| phenobarbital | chemical drug | | 1,28E-03 | CCND1,CYP1A1,GSTA2,MDM2 |
| ERN1 | kinase | | 1,32E-03 | ATF3,CXCL2,ETS1,JUN,TNFRSF10B |
| ID2 | transcription regulator | | 1,32E-03 | CCND1,DUSP1,ICAM1,IGFBP4,KLF6,MYC |
| CDH1 | other | | 1,34E-03 | CCND1,JUN,MYC,VIM |
| miR-141-3p (and other miRNAs w/seed AACACUG) | mature microRNA | Inhibited | 1,34E-03 | ABL2,BHLHE40,CXCL2,EPHA2,ITGA6,SMIM14,TNFAIP3,TTR,TXNIP,UBASH3B,VIM,ZNF627 |
| NSUN6 | enzyme | | 1,35E-03 | AXL,NDRG1,SERPINE1 |
| FCGR2A | transmembrane receptor | | 1,35E-03 | CXCL2,ICAM1,SERPINE1 |
| MEOX2 | transcription regulator | | 1,35E-03 | CXCL2,ICAM1,TNFAIP3 |
| PRKN | enzyme | | 1,35E-03 | CCND1,MYC,PLK2 |
| sorafenib | chemical drug | | 1,35E-03 | CCND1,PPP1R15A,VIM |
| CGS 21680 | chemical reagent | | 1,35E-03 | EGR1,FOS,RGS2 |
| parthenolide | chemical drug | | 1,35E-03 | ANKRD1,CXCL2,TNFRSF10B |
| ethionine | chemical toxicant | | 1,35E-03 | IER3,MYC,TSPAN8 |
| ACOX1 | enzyme | | 1,36E-03 | CTSV,KLF6,OAT,PHLDA1,RGS2 |
| L-threonine | chemical - endogenous mammalian | | 1,36E-03 | CCND1,MYC |
| NAP1L1 | other | | 1,36E-03 | AFP,TTR |
| CAMK2N2 | other | | 1,36E-03 | CCND1,MDM2 |
| LINC01139 | other | | 1,36E-03 | BHLHE40,EGR1 |
| FAT1 | other | | 1,36E-03 | JUN,PLAU |
| Shc | group | | 1,36E-03 | FOS,MYC |
| BCO2 | enzyme | | 1,36E-03 | ARRDC3,NR1D1 |
| Sox2ot | other | | 1,36E-03 | ALDH1A1,VIM |
| cercosporin | chemical - endogenous non-mammalian | | 1,36E-03 | CCND1,MYC |
| AMBP | transporter | | 1,36E-03 | CXCL2,PLAU |
| NCK1 | kinase | | 1,36E-03 | FOS,PPP1R15A |
| HCRT | other | | 1,36E-03 | EGR1,FOS |
| IL9R | transmembrane receptor | | 1,36E-03 | JUNB,MYC |
| SETD7 | enzyme | | 1,36E-03 | CCND1,MYC |
| NPAT | transcription regulator | | 1,36E-03 | H2BC5,H3C14 |
| N4BP1 | other | | 1,36E-03 | JUN,JUNB |
| RANBP9 | other | | 1,36E-03 | FOS,JUN |
| KLB | enzyme | | 1,36E-03 | EGR1,FOS |
| EXT1 | enzyme | | 1,36E-03 | CCND1,MYC |
| INCB054329 | chemical drug | | 1,36E-03 | CCND1,MYC |
| diltiazem | chemical drug | | 1,36E-03 | FOS,JUN |
| calmidazolium | chemical drug | | 1,36E-03 | EGR1,FOS |
| tiron | chemical reagent | | 1,36E-03 | AGT,DUSP1 |
| SU1498 | chemical - kinase inhibitor | | 1,36E-03 | ETS1,ICAM1 |
| SS18-SSX1 | fusion gene/product | | 1,36E-03 | CCND1,MDM2 |
| BQ-788 | biologic drug | | 1,36E-03 | CXCL2,LAMA1 |
| miR-495-3p (and other miRNAs w/seed AACAAAC) | mature microRNA | | 1,36E-03 | ARRDC3,BCL10,CXCL2,IER2,JUNB,MDM2,SMIM14 |
| lithium | chemical drug | | 1,40E-03 | EGR1,FOS,JUN,MDM2 |
| homocysteine | chemical - endogenous mammalian | | 1,40E-03 | ATF3,CCND1,NDRG1,PHLDA1 |
| IL27 | cytokine | | 1,40E-03 | EGR1,FOS,ICAM1,MYC,VIM |
| ciprofibrate | chemical drug | | 1,40E-03 | CTSV,NFKBIA,OAT,PDZK1,PHLDA1 |
| miR-5579-3p (miRNAs w/seed UAGCUUA) | mature microRNA | | 1,40E-03 | ALDH1A1,ARRDC3,EPHX2,GSTA2,KLF6,MXD1,REEP6,SMIM14,YOD1 |
| miR-27a-3p (and other miRNAs w/seed UCACAGU) | mature microRNA | Inhibited | 1,42E-03 | AFAP1,AHSG,CSRNP1,GEM,IER3,LDLR,PARD6B,PLAU,PLK2,SLC25A25,SMIM14,TSC22D2,ZNF608,ZNF627 |
| NOX4 | enzyme | | 1,46E-03 | AGT,ICAM1,SERPINE1 |
| pimagedine | chemical drug | | 1,46E-03 | ICAM1,MDM2,SERPINE1 |
| haloperidol | chemical drug | | 1,46E-03 | EGR1,FOS,LDLR,PER1 |
| MAPT | other | | 1,52E-03 | CCND1,CXCL2,FOS,GEM,H2BC5,JUN,PLAU,SERPINE1 |
| TFRC | transporter | | 1,52E-03 | ATF3,FOS,JUN,PPP1R15A |
| tazemetostat | chemical drug | | 1,53E-03 | AFP,ANKRD1,CCND1,DYRK1A,MYC,PLK2,TRRAP |
| T3-TR-RXR | complex | | 1,57E-03 | AKR1C3,LDLR,MDM2 |
| PGF | growth factor | | 1,57E-03 | ATF3,EGR1,SERPINE1 |
| HSPA9 | other | | 1,57E-03 | MDM2,MYC,VIM |
| THBS1 | other | | 1,57E-03 | EGR1,PLAU,TNFRSF10B |
| 4-methylnitrosoamino-1-(3-pyridinyl)-1-butanone | chemical toxicant | | 1,57E-03 | FOS,JUN,JUNB |
| valproic acid | chemical drug | | 1,57E-03 | AKR1C3,ATF3,CCND1,CYP1A1,FABP1,ICAM1,JUN,LDLR,SERPINE1 |
| halofuginone | chemical drug | | 1,58E-03 | AKAP12,BHLHE40,EGR1,EMP1,SELENOW |
| MAX | transcription regulator | | 1,59E-03 | KLF10,KLF6,MYC,TXNIP |
| cinnamaldehyde | chemical toxicant | | 1,59E-03 | DUSP6,EGR1,FOS,TXNIP |
| let-7 | microRNA | | 1,59E-03 | CCND1,DUSP6,MYC,SERPINE1,VIM,ZFP36 |
| miR-3613-3p (miRNAs w/seed CAAAAAA) | mature microRNA | Inhibited | 1,59E-03 | BCL10,DCBLD2,EGR1,KLF6,MDM2,MXD1,PARD6B,POLR2J2/POLR2J3,SERINC5,TSC22D2,YOD1,ZNF844 |
| miR-4446-5p (miRNAs w/seed UUUCCCU) | mature microRNA | | 1,59E-03 | ARRDC3,B4GALNT2,CYP1A1,EGR1,GEM,ITGA2,RHOB,SMIM14,TENT5A,TNFAIP3,TSPAN8,TXNIP |
| PBRM1 | other | | 1,66E-03 | IGFBP4,MDM2 |
| G2535 | chemical reagent | | 1,66E-03 | NFKBIA,VIM |
| GPR132 | G-protein coupled receptor | | 1,66E-03 | EGR1,ICAM1 |
| CCL1 | cytokine | | 1,66E-03 | ITGA2,SERPINE1 |
| RLIM | enzyme | | 1,66E-03 | SERPINE1,VIM |
| PTCSC3 | other | | 1,66E-03 | CCND1,MYC |
| PIKFYVE | kinase | | 1,66E-03 | ALDH1A1,ATF3 |
| ENTPD1 | enzyme | | 1,66E-03 | CXCL2,SERPINE1 |
| CNB-001 | chemical reagent | | 1,66E-03 | ATF3,PPP1R15A |
| Ncoa6 | transcription regulator | | 1,66E-03 | FOS,SERPINE1 |
| UBR5 | enzyme | | 1,66E-03 | CCND1,MDM2 |
| 7-(4-fluorobenzylamino)-1,3,4,8-tetrahydropyrrolo[4,3,2-de]quinolin-8(1H)-one | chemical reagent | | 1,66E-03 | CCND1,MDM2 |
| pterostilbene | chemical drug | | 1,66E-03 | CCND1,PLAU |
| triptorelin | biologic drug | | 1,66E-03 | FOS,JUN |
| tyrphostin AG 1296 | chemical - kinase inhibitor | | 1,66E-03 | ICAM1,JUN |
| Cu2+ | chemical - endogenous mammalian | | 1,66E-03 | CCND1,CYP1A1 |
| ATF1 | transcription regulator | | 1,68E-03 | EGR1,FOS,JUN |
| chloropromazine | chemical drug | | 1,68E-03 | FABP1,FOS,LDLR |
| S-nitrosoglutathione | chemical toxicant | | 1,68E-03 | BIRC3,ICAM1,NFKBIA |
| GDF2 | growth factor | | 1,72E-03 | FOS,ICAM1,MYC,NFKBIA |
| BCR-ABL1 | fusion gene/product | | 1,72E-03 | CCND1,MXD4,MYC,TNFRSF10B |
| miR-709 (and other miRNAs w/seed GAGGCAG) | mature microRNA | Inhibited | 1,74E-03 | AHSG,BCL10,ETS1,FABP1,GJB1,H2BC5,INAFM1,LDLR,MDM2,MYC,NR1D1,NUAK2,PALD1,PARD6B,PI3,POLR2J2/POLR2J3,TENT5A,ZC3H12A,ZNF627,ZNF844 |
| AGN194204 | chemical drug | | 1,74E-03 | ATF3,DUSP1,EMP1,KLF6,MYC,PHLDA1 |
| S100A4 | other | | 1,80E-03 | MDM2,PLAU,VIM |
| HBB | transporter | | 1,80E-03 | CXCL2,ICAM1,SERPINE1 |
| SRSF2 | transcription regulator | | 1,80E-03 | CCND1,PPP1R15A,VIM |
| retinoid | chemical drug | | 1,80E-03 | DUSP1,ITGA6,MYC |
| lipid | chemical - endogenous mammalian | | 1,80E-03 | AGT,FABP1,LDLR |
| 4-hydroxynonenal | chemical toxicant | | 1,80E-03 | ICAM1,JUN,SERPINE1 |
| mir-30 (includes others) | microRNA | | 1,80E-03 | ITGA2,SERPINE1,VIM |
| ouabain | chemical drug | | 1,80E-03 | FOS,TNFRSF10B,VIM |
| 17-alpha-ethinylestradiol | chemical drug | | 1,82E-03 | ARRDC3,FOS,JUN,LDLR,MDM2 |
| TGM2 | enzyme | Activated | 1,86E-03 | BIRC3,CCND1,CSRNP1,ITGA6,MYC,PHLDA1 |
| DIO2 | enzyme | | 1,88E-03 | ALDH1A1,ATF3,EGR1,FOS,PHLDA2 |
| diethylstilbestrol | chemical drug | Activated | 1,88E-03 | CCND1,DUSP6,EGR1,EMP1,FOS,MDM2,MXD4 |
| SP2509 | chemical reagent | | 1,88E-03 | AFP,ANKRD1,CCND1,DYRK1A,MYC,PLK2,TRRAP |
| miR-3614-3p (miRNAs w/seed AGCCUUC) | mature microRNA | | 1,90E-03 | ATF3,DCBLD2,EMP1,NDRG1,SEPHS2,SERINC5,SMIM14,TNFAIP3,YOD1,ZNF627 |
| ANGPT1 | growth factor | | 1,92E-03 | CCND1,DUSP1,ICAM1 |
| CDK2 | kinase | | 1,92E-03 | FOS,MYC,TNFAIP3 |
| miR-19b-3p (and other miRNAs w/seed GUGCAAA) | mature microRNA | Inhibited | 1,98E-03 | ARRDC3,CCND1,CXCL2,CYLD,H3C14,H3C15,KLF10,KLF6,LDLR,MXD1,RHOB,SPATA2,TENT5A,TNFAIP3,ZBTB20 |
| embelin | chemical - endogenous non-mammalian | | 1,98E-03 | CCND1,MYC |
| SOX2-OT | other | | 1,98E-03 | ALDH1A1,VIM |
| DEPTOR | other | | 1,98E-03 | ICAM1,VIM |
| CST3 | other | | 1,98E-03 | FOS,NFKBIA |
| CCAT1 | other | | 1,98E-03 | MYC,VIM |
| UBA1 | enzyme | | 1,98E-03 | BIRC3,CCND1 |
| SH3KBP1 | other | | 1,98E-03 | MYC,SERPINE1 |

| | | | | |
|---|---|---|---|---|
| mir-379 | microRNA | | 1,98E-03 | SPRY4,TXNIP |
| CCNT1 | transcription regulator | | 1,98E-03 | CCND1,MYC |
| PAX4 | transcription regulator | | 1,98E-03 | MYC,VIM |
| C9 | other | | 1,98E-03 | CXCL2,ICAM1 |
| pentylenetetrazol | chemical drug | | 1,98E-03 | FOS,JUN |
| bisphenol A diglycidyl ether | chemical reagent | | 1,98E-03 | ICAM1,SERPINE1 |
| D-sphingosine | chemical - endogenous mammalian | | 1,98E-03 | CXCL2,SERPINE1 |
| baclofen | chemical drug | | 1,98E-03 | FOS,PER1 |
| putrescine | chemical - endogenous mammalian | | 1,98E-03 | FOS,JUN |
| trientine | chemical drug | | 1,98E-03 | IGFBP4,SERPINE1 |
| NAD+ | chemical - endogenous mammalian | | 1,98E-03 | ANKRD1,TXNIP |
| miR-3679-3p (miRNAs w/seed UUCCCCC) | mature microRNA | Inhibited | 2,00E-03 | CSRNP1,CTSV,EGR1,EMP1,ETS1,GEM,H3C14,H3C15,ITGA2,NDRG1,PARD6B,SPRY4,TNFRSF10B,ZFP36,ZNF608 |
| miR-3691-5p (miRNAs w/seed GUGGAUG) | mature microRNA | | 2,05E-03 | AFAP1,ETS1,FABP1,MXD1,NFKBIA,PARD6B,PI3,SELENOW,SMIM14,UBASH3B,ZNF442,ZNF627 |
| HBEGF | growth factor | | 2,05E-03 | BHLHE40,CCND1,EGR1 |
| TXNIP | other | | 2,05E-03 | CCND1,ICAM1,TXNIP |
| TBK1 | kinase | Activated | 2,10E-03 | CXCL2,ICAM1,NFKBIZ,ZC3H12A |
| miR-3121-3p (miRNAs w/seed AAAUAGA) | mature microRNA | Inhibited | 2,17E-03 | ANKRD1,BCL10,CXCL2,EGR1,GEM,JUNB,PPP1R15A |
| Ifn gamma | complex | | 2,18E-03 | AGT,ICAM1,IGFBP4,MYC |
| flutamide | chemical drug | | 2,18E-03 | CXCL2,CYP1A1,FOS,VIM |
| RUNX1-RUNX1T1 | fusion gene/product | | 2,18E-03 | DUSP6,ITGA2,MDM2,UBASH3B |
| D-fructose | chemical - endogenous mammalian | | 2,19E-03 | AGT,CXCL2,ICAM1 |
| streptozocin | chemical drug | | 2,21E-03 | ATF3,FOS,ICAM1,SERPINE1,TXNIP,VIM |
| miR-1268a (and other miRNAs w/seed GGGCGUG) | mature microRNA | Inhibited | 2,22E-03 | AFAP1,H2BC5,H3C14,H3C15,MAFF,MXD1,POLR2J2/POLR2J3,SMIM14,TNFRSF10B,TNS4,ZNF554,ZNF844 |
| miR-3156-3p (miRNAs w/seed UCCCACU) | mature microRNA | Inhibited | 2,22E-03 | ABL2,ARRDC3,H3C14,H3C15,JUN,MAFF,PER1,SMIM14,UBASH3B,ZBTB20,ZNF441,ZNF627 |
| miR-204-5p (and other miRNAs w/seed UCCCUUU) | mature microRNA | | 2,24E-03 | AFAP1,B4GALNT2,EGR1,EMP1,MDM2,MYC,PRR15L,SPRED1,TBX3,TENT5A,TSPAN8,TXNIP |
| miR-3681-5p (and other miRNAs w/seed AGUGGAU) | mature microRNA | | 2,25E-03 | CSRNP1,DCBLD2,FABP1,MXD1,NUAK2,PI3,UBASH3B,YOD1,ZNF627 |
| TO-901317 | chemical reagent | | 2,26E-03 | CCND1,IGFBP4,ITGA6,LDLR,NFKBIA,NR1D1 |
| eicosapentenoic acid | chemical drug | | 2,26E-03 | FOS,JUN,LDLR,SERPINE1 |
| roscovitine | chemical drug | | 2,33E-03 | CCND1,MDM2,MYC |
| SMAD1 | transcription regulator | | 2,33E-03 | CXCL2,ICAM1,MYC |
| vincristine | chemical drug | | 2,33E-03 | EGR1,JUN,MDM2 |
| NR1I2 | ligand-dependent nuclear receptor | | 2,33E-03 | ALDH1A1,CYP1A1,EGR1,FABP1,JUN |
| mono-(2-ethylhexyl)phthalate | chemical toxicant | Activated | 2,33E-03 | CYP1A1,FOS,IER2,JUNB,LDLR |
| digoxin | chemical drug | | 2,33E-03 | NDRG1,TNFRSF10B |
| desoxycorticosterone | chemical - endogenous mammalian | | 2,33E-03 | FOS,SERPINE1 |
| Pdgfr | group | | 2,33E-03 | EGR1,VIM |
| trypsin | group | | 2,33E-03 | FOS,ICAM1 |
| BCO1 | enzyme | | 2,33E-03 | ARRDC3,NR1D1 |
| harmine | chemical - endogenous non-mammalian | | 2,33E-03 | CYP1A1,FOS |
| CSNK2B | kinase | | 2,33E-03 | CCND1,MYC |
| FGF23 | growth factor | | 2,33E-03 | EGR1,PDZK1 |
| EN1 | transcription regulator | | 2,33E-03 | ALDH1A1,CCND1 |
| CRY2 | enzyme | | 2,33E-03 | FOS,PER1 |
| BCAR1 | enzyme | | 2,33E-03 | CCND1,EGR1 |
| SDC1 | enzyme | | 2,33E-03 | CXCL2,JUNB |
| PTGER1 | G-protein coupled receptor | | 2,33E-03 | FOS,ICAM1 |
| SPINT2 | other | | 2,33E-03 | CXCL2,PLAU |
| CRY1 | enzyme | | 2,33E-03 | FOS,PER1 |
| HSPA1A/HSPA1B | enzyme | | 2,33E-03 | CXCL2,FOS |
| aristolochic acid I | chemical toxicant | | 2,33E-03 | FABP1,FOS |
| 1,2-dimethylhydrazine | chemical toxicant | | 2,33E-03 | CCND1,MYC |
| hydroquinone | chemical - endogenous mammalian | | 2,33E-03 | FOS,JUN |
| SKF-38393 | chemical reagent | | 2,33E-03 | EGR1,FOS |
| N,N-dimethylsphingosine | chemical reagent | | 2,33E-03 | FOS,ICAM1 |
| astressin 2B | biologic drug | | 2,33E-03 | CXCL2,FOS |
| verteporfin | chemical drug | | 2,33E-03 | CXCL2,VIM |
| miR-202-5p (miRNAs w/seed UCCUAUG) | mature microRNA | | 2,34E-03 | ARRDC3,ERRFI1,NDRG1,PDZK1,TENT5A,UBASH3B,VIM,ZNF442 |
| GATA2 | transcription regulator | | 2,38E-03 | CCND1,CXCL2,ETS1,ICAM1,ITGA2,MYC,PLK2,SERPINA6 |
| miR-4696 (miRNAs w/seed GCAAGAC) | mature microRNA | | 2,40E-03 | BCL10,DCBLD2,EMP1,FABP1,H3C14,H3C15,MDM2,PRR15L,ZNF627 |
| miR-4476 (and other miRNAs w/seed AGGAAGG) | mature microRNA | Inhibited | 2,45E-03 | AFAP1,AHSG,BHLHE40,CCND1,GJB1,IGFBP4,INAFM1,LDLR,PLAU,SPATA2,SPRED1,SPRY4,TRRAP,TSC22D2,ZBTB43 |
| PRMT5 | enzyme | | 2,47E-03 | CCND1,MYC,PER1 |
| APLN | other | | 2,47E-03 | EGR1,FOS,MYC |
| PPRC1 | transcription regulator | | 2,47E-03 | ERRFI1,NFKBIZ,PHLDA1 |
| zVAD-FMK | chemical - protease inhibitor | | 2,47E-03 | BIRC3,FOS,MDM2 |
| RPTOR | other | | 2,52E-03 | CCND1,DUSP6,JUN,MYC |
| N-cor | group | | 2,61E-03 | CCND1,CXCL2,HIVEP2,RHOB |
| SCARB1 | transporter | | 2,62E-03 | CXCL2,ICAM1,LDLR |
| EGR3 | transcription regulator | | 2,62E-03 | BHLHE40,JUN,MYC |
| MYBL2 | transcription regulator | | 2,62E-03 | BIRC3,CCND1,MYC |
| AIP | transcription regulator | | 2,62E-03 | CYP1A1,DUSP1,RGS2 |
| bicalutamide | chemical drug | | 2,62E-03 | FOS,TNFRSF10B,VIM |
| MRTFB | transcription regulator | | 2,66E-03 | ANKRD1,EGR1,ITGA6,NFKBIZ,SERPINE1 |
| lenalidomide | chemical drug | | 2,66E-03 | CCND1,H3C4,MYC,NFKBIZ,TNFRSF10B,VIM |
| NR1H2 | ligand-dependent nuclear receptor | | 2,71E-03 | AGT,CXCL2,LDLR,NFKBIA |
| xanthine | chemical - endogenous mammalian | | 2,71E-03 | DUSP1,PLAU |
| glycochenodeoxycholate | chemical - endogenous mammalian | | 2,71E-03 | BIRC3,TNFRSF10B |
| Rac | group | | 2,71E-03 | CCND1,FOS |
| IDH2 | enzyme | | 2,71E-03 | EGR1,VIM |
| PSMB10 | peptidase | | 2,71E-03 | NFKBIA,TNFAIP3 |
| PTBP1 | enzyme | | 2,71E-03 | LDLR,MYC |
| F10 | peptidase | | 2,71E-03 | EGR1,ICAM1 |
| SRSF3 | other | | 2,71E-03 | CCND1,JUN |
| TRAF4 | other | | 2,71E-03 | CCND1,MYC |
| ILF3 | transcription regulator | | 2,71E-03 | FOS,PLAU |
| HNRNPD | transcription regulator | | 2,71E-03 | FOS,LDLR |
| ST14 | peptidase | | 2,71E-03 | CXCL2,ICAM1 |
| ebselen | chemical drug | | 2,71E-03 | DUSP1,ICAM1 |
| dimethyl fumarate | chemical drug | | 2,71E-03 | JUN,VIM |
| pyruvic acid | chemical - endogenous mammalian | | 2,71E-03 | FOS,TNFRSF10B |
| salmeterol | chemical drug | | 2,71E-03 | DUSP1,RGS2 |
| nickel chloride | chemical toxicant | | 2,71E-03 | JUN,NDRG1 |
| anti-benzo(a)pyrene-diol-epoxide | chemical toxicant | | 2,71E-03 | CCND1,JUN |
| buthionine sulfoximine | chemical drug | | 2,71E-03 | FOS,NFKBIA |
| CD86 | transmembrane receptor | | 2,78E-03 | ICAM1,NFKBIA,TNFAIP3 |
| PRKD1 | kinase | | 2,78E-03 | CCND1,CXCL2,TNFAIP3 |
| miR-30c-5p (and other miRNAs w/seed GUAAACA) | mature microRNA | Inhibited | 2,89E-03 | ASB2,BCL10,H3C4,ITGA2,ITGA6,JUN,JUNB,KLF10,SMIM14,TENT5A,TNFRSF10B,VIM,YOD1,ZNF608 |
| fluticasone propionate | chemical drug | | 2,94E-03 | CXCL2,EMP1,PHLDA1,PLAU,SERPINE1 |
| IFN Beta | group | Activated | 2,94E-03 | ATF3,DUSP1,MYC,SERPINE1,SPRY4 |
| SMARCB1 | transcription regulator | | 2,94E-03 | CCND1,FOS,GJB1,MDM2,MYC |
| ROCK2 | kinase | | 2,94E-03 | ICAM1,PI3,SERPINE1 |
| TSC22D3 | transcription regulator | | 2,94E-03 | CCND1,DUSP1,ICAM1 |
| methimazole | chemical drug | | 2,94E-03 | IGFBP4,JUN,PHLDA1 |
| IKZF1 | transcription regulator | | 3,00E-03 | AXL,CXCL2,EMP1,MYC,NFKBIZ,TXNIP |
| NFATC1 | transcription regulator | | 3,00E-03 | CCND1,CYP1A1,ICAM1,MYC |
| RARB | ligand-dependent nuclear receptor | | 3,01E-03 | CXCL2,EGR1,ETS1,ICAM1,JUN |
| SRC (family) | group | | 3,11E-03 | CYP1A1,EGR1,ICAM1 |
| IDH1 | enzyme | | 3,11E-03 | EGR1,PPP1R15A,VIM |
| CCN5 | growth factor | | 3,11E-03 | JUN,SERPINE1,VIM |
| TNFSF12 | cytokine | | 3,11E-03 | CCND1,CXCL2,ICAM1,NFKBIA |
| cyclic GMP | chemical - endogenous mammalian | | 3,12E-03 | FOS,SERPINE1 |
| ADP | chemical - endogenous mammalian | | 3,12E-03 | FOS,SERPINE1 |
| Nos | group | | 3,12E-03 | EGR1,FOS |
| THRAP3 | transcription regulator | | 3,12E-03 | CCND1,TXNIP |
| IGFBP7 | transporter | | 3,12E-03 | CCND1,MYC |
| PCSK9 | peptidase | | 3,12E-03 | ICAM1,LDLR |
| AIMP1 | cytokine | | 3,12E-03 | CXCL2,ICAM1 |
| PNN | other | | 3,12E-03 | JUN,MYC |
| APBB1 | transcription regulator | | 3,12E-03 | CCND1,MYC |
| mir-199 | microRNA | | 3,12E-03 | AXL,DYRK1A |
| GHRHR | G-protein coupled receptor | | 3,12E-03 | CCND1,FOS |
| PER2 | transcription regulator | | 3,12E-03 | CCND1,PER1 |
| HOXB4 | transcription regulator | | 3,12E-03 | JUNB,MYC |
| GNA13 | enzyme | | 3,12E-03 | EGR1,FOS |
| TGFBR3 | kinase | | 3,12E-03 | ALDH1A1,SERPINE1 |
| EDNRA | transmembrane receptor | | 3,12E-03 | FOS,ICAM1 |
| TOPBP1 | other | | 3,12E-03 | MDM2,MYC |
| Mn(III)-tetrakis-(4-benzoic acid) porphyrin | chemical reagent | | 3,12E-03 | AGT,SERPINE1 |
| cyproterone acetate | chemical drug | | 3,12E-03 | CCND1,MYC |
| 24-hydroxycholesterol | chemical - endogenous mammalian | | 3,12E-03 | AGT,LDLR |
| uric acid | chemical - endogenous mammalian | | 3,21E-03 | AGT,CXCL2,ICAM1,MDM2 |
| miR-512-3p (miRNAs w/seed AGUGCUG) | mature microRNA | | 3,22E-03 | DUSP1,FABP1,GJB1,H3C14,H3C15,IER3,REEP6,SERPINA6,TNFRSF10B,ZNF441 |
| GHR | transmembrane receptor | | 3,28E-03 | CCND1,IGFBP4,MYC |
| FOXO4 | transcription regulator | | 3,43E-03 | CCND1,IER3,SERPINE1,TXNIP |
| KAT5 | transcription regulator | | 3,43E-03 | AFP,CCND1,MYC,TTR |
| plicamycin | chemical drug | | 3,43E-03 | AXL,IGFBP4,MDM2,SERPINE1 |
| PRDM1 | transcription regulator | Inhibited | 3,43E-03 | AFP,CXCL2,FGG,FOS,MYC,TTR |
| EIF2S1 | translation regulator | | 3,46E-03 | CCND1,PHLDA1,PPP1R15A |
| PTPN1 | phosphatase | | 3,46E-03 | CXCL2,DUSP1,NFKBIA |
| iron | chemical - endogenous mammalian | | 3,46E-03 | CCND1,NDRG1,PPP1R15A |
| miR-4786-5p (miRNAs w/seed GAGACCA) | mature microRNA | | 3,52E-03 | CYP1A1,ETS1,KLF10,NUAK2,PARD6B,REEP6,SEPHS2,SERINC5,TENT5A,YOD1 |
| prostaglandin A2 | chemical - endogenous non-mammalian | | 3,55E-03 | CCND1,ICAM1 |
| glycerol | chemical - endogenous mammalian | | 3,55E-03 | AGT,LDLR |
| BMP10 | growth factor | | 3,55E-03 | ICAM1,NFKBIA |
| BLNK | other | | 3,55E-03 | JUN,MDM2 |
| MAPK10 | kinase | | 3,55E-03 | FOS,JUN |
| CXCL2 | cytokine | | 3,55E-03 | CXCL2,ICAM1 |
| PRDX2 | enzyme | | 3,55E-03 | CCND1,ICAM1 |
| MAML1 | transcription regulator | | 3,55E-03 | CCND1,EGR1 |
| BCL10 | transcription regulator | | 3,55E-03 | CXCL2,TNFAIP3 |
| BHLHA15 | transcription regulator | | 3,55E-03 | ARRDC3,GJB1 |
| RHOB | enzyme | | 3,55E-03 | ICAM1,RHOB |
| 4-nitroquinoline-1-oxide | chemical toxicant | | 3,55E-03 | EGR1,MDM2 |
| concanamycin A | chemical drug | | 3,55E-03 | ICAM1,NFKBIA |
| farnesyl pyrophosphate | chemical - endogenous mammalian | | 3,55E-03 | ICAM1,SERPINE1 |
| reserpine | chemical drug | | 3,55E-03 | EGR1,FOS |
| mannose | chemical - endogenous mammalian | | 3,55E-03 | FOS,ICAM1 |
| miR-6772-3p (miRNAs w/seed UGCUCCU) | mature microRNA | | 3,56E-03 | DUSP6,ERRFI1,ETS1,GJB1,ICAM1,POLR2J2/POLR2J3,SEPHS2,SLC39A5,TIPARP,TSPAN8,UBASH3B |
| HDL | complex | | 3,64E-03 | ATF3,ICAM1,LDLR |
| DCN | other | | 3,64E-03 | DUSP1,ICAM1,SERPINE1 |
| carboplatin | chemical drug | | 3,64E-03 | IER3,MYC,VIM |
| 3-methylcholanthrene | chemical toxicant | | 3,64E-03 | CCND1,CYP1A1,FOS |
| SYK | kinase | | 3,66E-03 | AKAP12,BIRC3,CCND1,ICAM1 |
| GNA15 | enzyme | | 3,78E-03 | AKAP12,CXCL2,MYC,RGS2 |
| miR-4716-3p (and other miRNAs w/seed AGGGGGA) | mature microRNA | Inhibited | 3,81E-03 | AFAP1,ARRDC3,CCND1,CSRNP1,DYRK1A,EPHA2,FABP1,IER3,KLF6,MDM2,MXD1,PRR15L,SERINC5,SPATA2,SPRY4,TBX3,TNFAIP3,VGF,ZBTB20,ZNF442 |
| WWTR1 | transcription regulator | | 3,83E-03 | CXCL2,ICAM1,SERPINE1 |
| EBI3 | cytokine | | 3,83E-03 | FOS,ICAM1,MYC |
| miR-1-3p (and other miRNAs w/seed GGAAUGU) | mature microRNA | | 3,89E-03 | AKAP12,AXL,EMP1,ETS1,MXD1,MXD4,OAT,SLC25A25,SMIM14,SPRED1,TNS4,TTR |
| miR-3157-5p (miRNAs w/seed UCAGCCA) | mature microRNA | Inhibited | 3,95E-03 | ARRDC3,BIRC3,CCND1,CSRNP1,LDLR,MAFF,MYC,NDRG1,NFKBIZ,PALD1,POLR2J2/POLR2J3,SMIM14,UBASH3B |
| NFkB (family) | group | | 4,01E-03 | CCND1,TNFAIP3 |
| Smad2/3-Smad4 | complex | | 4,01E-03 | MYC,SERPINE1 |
| GIP | other | | 4,01E-03 | FOS,RGS2 |
| PSMB8 | peptidase | | 4,01E-03 | NFKBIA,TNFAIP3 |
| PPP2R5C | other | | 4,01E-03 | BIRC3,TNFAIP3 |
| GPI | enzyme | | 4,01E-03 | CCND1,VIM |
| MAZ | transcription regulator | | 4,01E-03 | FGG,MYC |
| DNAJB6 | transcription regulator | | 4,01E-03 | VGF,VIM |
| SMURF2 | enzyme | | 4,01E-03 | JUN,SERPINE1 |
| DAXX | transcription regulator | | 4,01E-03 | CCND1,SERPINE1 |
| KLK5 | peptidase | | 4,01E-03 | CXCL2,ICAM1 |
| premarin | chemical drug | | 4,01E-03 | MYC,SERPINE1 |
| 3-aminobenzamide | chemical toxicant | | 4,01E-03 | ICAM1,MDM2 |
| 7-nitroindazole | chemical reagent | | 4,01E-03 | FOS,NFKBIA |
| decursin | chemical - endogenous non-mammalian | | 4,01E-03 | CCND1,MYC |
| nimodipine | chemical drug | | 4,01E-03 | FOS,MYC |
| zearalenone | chemical toxicant | | 4,01E-03 | CCND1,MYC |
| glutamyl-Se-methylselenocysteine | chemical - endogenous mammalian | | 4,01E-03 | EGR1,JUN |
| losartan potassium | chemical drug | | 4,02E-03 | AGT,EGR1,FOS,SERPINE1 |
| PAX5 | transcription regulator | | 4,03E-03 | CCND1,TXNIP,VIM |
| BTK | kinase | | 4,15E-03 | EMP1,ETS1,JUN,MDM2 |
| GNAS | enzyme | | 4,23E-03 | FOS,MYC,VGF |
| PIM1 | kinase | | 4,23E-03 | EPHA2,MDM2,MYC |
| CD14 | transmembrane receptor | | 4,23E-03 | CXCL2,ICAM1,TNFAIP3 |
| ZEB2 | transcription regulator | | 4,23E-03 | AXL,CCND1,VIM |
| CCL11 | cytokine | | 4,23E-03 | CXCL2,ICAM1,ITGA2 |
| IFNA1/IFNA13 | cytokine | | 4,23E-03 | CYP1A1,GEM,MYC |
| miR-585-5p (miRNAs w/seed UAGCACA) | mature microRNA | Inhibited | 4,31E-03 | AXL,DUSP6,H3C4,ITGA2,LCA5,MDM2,SPRED1,TENT5A,VIM,ZBTB43 |
| miR-3934-3p (miRNAs w/seed GCUCAGG) | mature microRNA | Inhibited | 4,36E-03 | CSRNP1,CYP1A1,INAFM1,KRT6A,LDLR,MAFF,MYC,NFKBIZ,NUAK2,PALD1,RHOB,TNS4,TRRAP |
| miR-4765 (miRNAs w/seed GAGUGAU) | mature microRNA | | 4,38E-03 | ARRDC3,DCBLD2,ETS1,FABP1,IGFBP4,RGS2,SMIM14,ZBTB43 |
| CBX5 | transcription regulator | | 4,41E-03 | AKR1C3,PRR15L,TSPAN8,TXNIP |
| emodin | chemical drug | | 4,44E-03 | CYP1A1,ICAM1,VIM |
| heme | chemical - endogenous mammalian | | 4,44E-03 | CXCL2,ICAM1,PER1 |
| 15(S)-HETE | chemical - endogenous mammalian | | 4,49E-03 | EGR1,JUN |
| glycine | chemical - endogenous mammalian | | 4,49E-03 | CCND1,FOS |
| mannitol | chemical - endogenous mammalian | | 4,49E-03 | ICAM1,JUN |
| NMDA Receptor | complex | | 4,49E-03 | ATF3,FOS |
| Pdgf Ab | complex | | 4,49E-03 | CCND1,FOS |
| PLC | group | | 4,49E-03 | FOS,MYC |
| PKNOX2 | transcription regulator | | 4,49E-03 | EGR1,VIM |
| PI-103 | chemical - kinase inhibitor | | 4,49E-03 | CCND1,TNFRSF10B |
| PADI2 | enzyme | | 4,49E-03 | CXCL2,VIM |
| LYL1 | transcription regulator | | 4,49E-03 | EGR1,ITGA2 |
| DEF6 | other | | 4,49E-03 | FOS,VIM |
| ARHGDIG | other | | 4,49E-03 | DUSP6,RGS2 |
| HOTAIRM1 | other | | 4,49E-03 | EPHX2,LAMA1 |
| MDM4 | transcription regulator | | 4,49E-03 | MDM2,VIM |
| PDGFRA | kinase | | 4,49E-03 | FOS,MYC |
| PLA2G4A | enzyme | | 4,49E-03 | ICAM1,MYC |
| CD22 | transmembrane receptor | | 4,49E-03 | ETS1,MYC |
| XPO1 | transporter | | 4,49E-03 | CCND1,ICAM1 |
| CXCL3 | cytokine | | 4,49E-03 | ICAM1,ITGA6 |
| MAPKAPK3 | kinase | | 4,49E-03 | CXCL2,ZFP36 |
| YY2 | transcription regulator | | 4,49E-03 | FOS,MYC |
| quinpirole | chemical reagent | | 4,49E-03 | FOS,JUNB |
| methylphenidate | chemical drug | | 4,49E-03 | EGR1,FOS |
| acyclic retinoid | chemical drug | | 4,49E-03 | CCND1,MYC |
| miR-4645-3p (miRNAs w/seed GACAGUA) | mature microRNA | Inhibited | 4,50E-03 | ARRDC3,DCBLD2,EMP1,IER3,JUN,PDZK1,SPATA2,SPRED1,SPRY4,TSC22D2,ZNF441 |
| dextran sulfate | chemical drug | | 4,51E-03 | AGT,CCND1,CXCL2,ICAM1,MYC,SERPINE1 |
| 1,4-bis[2-(3,5-dichloropyridyloxy)]benzene | chemical toxicant | | 4,54E-03 | CCND1,ICAM1,MAFF,MDM2 |
| TLR2 | transmembrane receptor | | 4,57E-03 | ATF3,CXCL2,CYLD,DUSP1,ICAM1 |
| miR-6729-3p (miRNAs w/seed CAUCCCC) | mature microRNA | Inhibited | 4,76E-03 | ATF3,ERRFI1,ETS1,IGFBP4,KLF10,LDLR,MAFF,RHOB,SERINC5,UBASH3B,ZFP36 |
| AMPK | complex | | 4,82E-03 | EGR1,OAT,SERPINE1,VIM |
| POU2F1 | transcription regulator | | 4,82E-03 | CCND1,CYP1A1,ICAM1,TNFAIP3 |
| miR-6861-3p (miRNAs w/seed GGACCUC) | mature microRNA | Inhibited | 4,85E-03 | ABL2,AKAP12,CYP1A1,EMP1,IGFBP4,MDM2,MXD1,PMM1,ZBTB20 |
| sunitinib | chemical drug | | 4,88E-03 | CYP1A1,ICAM1,ITGA6 |
| IL11 | cytokine | | 4,88E-03 | FOS,ICAM1,JUNB |
| HES1 | transcription regulator | | 4,88E-03 | DUSP1,MDM2,OAT |
| clozapine | chemical drug | | 4,88E-03 | EGR1,FOS,LDLR |
| ARNT | transcription regulator | | 4,96E-03 | BHLHE40,CYP1A1,SERPINE1,VIM |
| miR-6752-5p (and other miRNAs w/seed GGGGGUG) | mature microRNA | | 4,96E-03 | ABL2,AFAP1,AGT,ALDH1A1,ANKRD1,ATF3,DCBLD2,ETS1,FABP1,GJB1,JUNB,KLF6,LDLR,REEP6,RHOB,SERINC5,SMIM14,SPATA2,SPRY4,TNS4,ZBTB43,ZC3H12A |
| Srebp | group | | 5,00E-03 | FOS,LDLR |
| trastuzumab | biologic drug | | 5,00E-03 | CCND1,MYC |
| TMSB4 | group | | 5,00E-03 | ITGA2,SERPINE1 |
| carfilzomib | biologic drug | | 5,00E-03 | ATF3,MYC |
| PTPN2 | phosphatase | | 5,00E-03 | CCND1,PPP1R15A |
| BAP1 | peptidase | | 5,00E-03 | ATF3,CXCL2 |

| | | | | |
|---|---|---|---|---|
| DBP | transcription regulator | | 5,00E-03 | AGT,PER1 |
| MUC4 | other | | 5,00E-03 | CCND1,VIM |
| EMD | other | | 5,00E-03 | EGR1,IER3 |
| diethylmaleate | chemical toxicant | | 5,00E-03 | NFKBIA,TNFAIP3 |
| helenalin | chemical - endogenous non-mammalian | | 5,00E-03 | NFKBIA,TNFRSF10B |
| vanadate | chemical - other | | 5,00E-03 | CCND1,JUN |
| dichlororibofuranosylbenzimidazole | chemical toxicant | | 5,00E-03 | CYP1A1,MYC |
| SPI1 | transcription regulator | Inhibited | 5,01E-03 | CCND1,DUSP6,FOS,JUN,MYC,VIM |
| miR-4800-5p (miRNAs w/seed GUGGACC) | mature microRNA | Inhibited | 5,07E-03 | AXL,EMP1,H3C14,H3C15,MAFF,MXD1,SEPHS2,TXNIP,ZBTB20 |
| FLI1 | transcription regulator | | 5,11E-03 | CCND1,EGR1,MDM2 |
| carbon monoxide | chemical - endogenous mammalian | | 5,11E-03 | EGR1,ICAM1,SERPINE1 |
| PSMB11 | peptidase | | 5,11E-03 | CCND1,CTSV,LAMA1,RHOB |
| miR-6731-3p (miRNAs w/seed CUAUUCC) | mature microRNA | | 5,11E-03 | CCND1,GJB1,RGS2,SERPINE1,VIM,YOD1,ZNF441 |
| miR-193a-5p (miRNAs w/seed GGGUCUU) | mature microRNA | Inhibited | 5,13E-03 | CYP1A1,FGG,IGFBP4,LDLR,SPRY4,TENT5A,TNS4,TSC22D2,ZNF627,ZNF844 |
| miR-517a-3p (and other miRNAs w/seed UCGUGCA) | mature microRNA | | 5,18E-03 | AFAP1,JUN,KLF10,LDLR,SMIM14,UBASH3B |
| miR-148b-5p (and other miRNAs w/seed AGUUCUG) | mature microRNA | Inhibited | 5,19E-03 | BHLHE40,CSRNP1,CXCL2,ETS1,JUN,KLF10,LCA5,RGS2,TSC22D2 |
| miR-3168 (miRNAs w/seed AGUUCUA) | mature microRNA | Inhibited | 5,20E-03 | CXCL2,ERRFI1,GEM,JUN,KLF10 |
| 3M-002 | chemical reagent | Inhibited | 5,25E-03 | AGT,CCND1,SPRY4,TXNIP |
| ID3 | transcription regulator | | 5,31E-03 | DUSP1,ICAM1,IGFBP4,KLF6,MYC |
| Fcer1 | complex | | 5,34E-03 | DUSP6,FOS,JUN |
| HOXD10 | transcription regulator | | 5,34E-03 | ICAM1,RHOB,SERPINE1 |
| USF2 | transcription regulator | | 5,34E-03 | AGT,MYC,SERPINE1 |
| N-methyl-D-aspartate | chemical drug | | 5,34E-03 | EGR1,FOS,MYC |
| glucosamine | chemical - endogenous mammalian | | 5,34E-03 | AGT,ICAM1,TXNIP |
| MIR17HG | other | | 5,40E-03 | CCND1,CYLD,TBX3,TNFAIP3 |
| E. coli B4 lipopolysaccharide | chemical toxicant | | 5,45E-03 | CXCL2,ICAM1,NFKBIA,TNFAIP3,TTR,TXNIP |
| puromycin | chemical - endogenous non-mammalian | | 5,54E-03 | CYP1A1,SERPINE1 |
| cyclooxygenase | group | | 5,54E-03 | FOS,RGS2 |
| MPZ | other | | 5,54E-03 | JUN,PPP1R15A |
| CDK5R1 | kinase | | 5,54E-03 | FOS,SERPINE1 |
| KLF10 | transcription regulator | | 5,54E-03 | CCND1,SERPINE1 |
| AURKB | kinase | | 5,54E-03 | ATF3,IER3 |
| IAPP | other | | 5,54E-03 | FOS,MYC |
| lapatinib/pazopanib | chemical drug | | 5,54E-03 | EGR1,PHLDA1 |
| PIM2 | kinase | | 5,54E-03 | CCND1,MYC |
| LY6E | other | | 5,54E-03 | NFKBIA,SERPINE1 |
| RND3 | enzyme | | 5,54E-03 | CCND1,VIM |
| DUSP5 | phosphatase | | 5,54E-03 | EGR1,ZFP36 |
| PTK2B | kinase | | 5,54E-03 | FOS,JUN |
| NAB2 | transcription regulator | | 5,54E-03 | FOS,PLAU |
| pevonedistat | chemical drug | | 5,54E-03 | RHOB,TNFRSF10B |
| HNRNPA1 | enzyme | | 5,54E-03 | FGG,MYC |
| AS1842856 | chemical reagent | | 5,54E-03 | ICAM1,PLAU |
| NCD-38 | chemical reagent | | 5,54E-03 | ATF3,PPP1R15A |
| (1S,2R)-NCL-1 | chemical reagent | | 5,54E-03 | ATF3,PPP1R15A |
| salvin | chemical toxicant | | 5,54E-03 | CCND1,EGR1 |
| miR-148a-3p (and other miRNAs w/seed CAGUGCA) | mature microRNA | Inhibited | 5,56E-03 | ABL2,ARRDC3,DUSP1,DYRK1A,EMP1,ERRFI1,ETS1,KLF6,LDLR,MAFF,MXD1,TXNIP,UBASH3B,ZFP36 |
| 9-hydroxy-(S)-10,12-octadecadienoic acid | chemical - endogenous mammalian | | 5,60E-03 | SERPINE1 |
| pelargonidin | chemical - endogenous non-mammalian | | 5,60E-03 | CYP1A1 |
| 2'-adenylic acid | chemical - endogenous mammalian | | 5,60E-03 | TXNIP |
| peptide YY 3-36 | biologic drug | | 5,60E-03 | FOS |
| G protein alpha | group | | 5,60E-03 | FOS |
| growth factor receptor | group | | 5,60E-03 | FOS |
| Ahr-aryl hydrocarbon | complex | | 5,60E-03 | MDM2 |
| 2,2',4,4',5,5'-hexabromodiphenyl ether | chemical reagent | | 5,60E-03 | CYP1A1 |
| 5,7-dimethoxyflavone | chemical - endogenous mammalian | | 5,60E-03 | CYP1A1 |
| DE 71 | chemical reagent | | 5,60E-03 | CYP1A1 |
| Ahr-aryl hydrocarbon-RelA | complex | | 5,60E-03 | MYC |
| NR2F1-AS1 | other | | 5,60E-03 | CCND1 |
| G-Actin | group | | 5,60E-03 | JUN |
| tandospirone | chemical drug | | 5,60E-03 | PER1 |
| PKR dimer | complex | | 5,60E-03 | ATF3 |
| GSK 189254 | chemical drug | | 5,60E-03 | FOS |
| CLTA | other | | 5,60E-03 | LDLR |
| (-)-OSU 6162 | chemical drug | | 5,60E-03 | FOS |
| GJC2 | transporter | | 5,60E-03 | GJB1 |
| PMM2 | enzyme | | 5,60E-03 | ICAM1 |
| NCK | group | | 5,60E-03 | CCND1 |
| pridopidine | chemical drug | | 5,60E-03 | FOS |
| FBXO31 | enzyme | | 5,60E-03 | CCND1 |
| NLRP2 | other | | 5,60E-03 | ICAM1 |
| FBXO22 | enzyme | | 5,60E-03 | MDM2 |
| SRF-ELK1 | complex | | 5,60E-03 | FOS |
| WFDC1 | other | | 5,60E-03 | ICAM1 |
| KCNJ12 | ion channel | | 5,60E-03 | CCND1 |
| CALCOCO1 | transcription regulator | | 5,60E-03 | CCND1 |
| URGCP | other | | 5,60E-03 | CCND1 |
| RND2 | enzyme | | 5,60E-03 | RHOB |
| N-arachidonylglycine | chemical - endogenous mammalian | | 5,60E-03 | AXL |
| TMEM184A | other | | 5,60E-03 | DUSP1 |
| DEPDC1 | transcription regulator | | 5,60E-03 | TNFAIP3 |
| NACC2 | transcription regulator | | 5,60E-03 | MDM2 |
| MYADM | other | | 5,60E-03 | ICAM1 |
| MACROD1 | enzyme | | 5,60E-03 | NFKBIA |
| CCDC22 | other | | 5,60E-03 | BIRC3 |
| CCDC62 | transcription regulator | | 5,60E-03 | CCND1 |
| KCTD10 | ion channel | | 5,60E-03 | RHOB |
| harman | chemical - endogenous non-mammalian | | 5,60E-03 | CYP1A1 |
| FGF14 | growth factor | | 5,60E-03 | FOS |
| TRPM3 | ion channel | | 5,60E-03 | EGR1 |
| PLAC1 | other | | 5,60E-03 | CCND1 |
| RNF181 | enzyme | | 5,60E-03 | BCL10 |
| FANK1 | transcription regulator | | 5,60E-03 | JUN |
| TCF4-CTNNβ | complex | | 5,60E-03 | MYC |
| hispidulin | chemical drug | | 5,60E-03 | EGR1 |
| NNZ 2566 | biologic drug | | 5,60E-03 | ATF3 |
| vasopressins | biologic drug | | 5,60E-03 | FOS |
| black cohosh extract | chemical drug | | 5,60E-03 | SERPINE1 |
| Integrin alpha 5 beta 1 | complex | | 5,60E-03 | CCND1 |
| mediator | complex | | 5,60E-03 | FOS |
| Mapk kinase | group | | 5,60E-03 | CCND1 |
| Rho gdi | group | | 5,60E-03 | RHOB |
| GLIPR1 | other | | 5,60E-03 | MYC |
| NSMCE3 | other | | 5,60E-03 | MYC |
| ARHGEF17 | other | | 5,60E-03 | FOS |
| PRCC | other | | 5,60E-03 | SERPINE1 |
| MEDI-547 | biologic drug | | 5,60E-03 | EPHA2 |
| Tenascin | group | | 5,60E-03 | CCND1 |
| DYNLRB1 | other | | 5,60E-03 | SERPINE1 |
| RBMS1 | other | | 5,60E-03 | MYC |
| AHRR | transcription regulator | | 5,60E-03 | CYP1A1 |
| KIFAP3 | other | | 5,60E-03 | FOS |
| MED7 | transcription regulator | | 5,60E-03 | TNFAIP3 |
| PPY | other | | 5,60E-03 | FOS |
| CDC45 | other | | 5,60E-03 | MYC |
| GPHA2 | other | | 5,60E-03 | FOS |
| dodecylbenzenesulfonic acid | chemical reagent | | 5,60E-03 | PI3 |
| SLC52A1 | transporter | | 5,60E-03 | ICAM1 |
| ADCY2 | enzyme | | 5,60E-03 | CCND1 |
| CLTB | other | | 5,60E-03 | LDLR |
| mir-363 | microRNA | | 5,60E-03 | MYC |
| mir-1301 | microRNA | | 5,60E-03 | KLF6 |
| TIMELESS | other | | 5,60E-03 | PER1 |
| NPVF | other | | 5,60E-03 | FOS |
| RAB22A | enzyme | | 5,60E-03 | VGF |
| BORCS8-MEF2B | transcription regulator | | 5,60E-03 | JUN |
| SLC30A9 | transporter | | 5,60E-03 | CYP1A1 |
| CNOT9 | other | | 5,60E-03 | MYC |
| CAMK2 | group | | 5,60E-03 | BIRC3 |
| EPSTI1 | other | | 5,60E-03 | NFKBIA |
| TOB2 | other | | 5,60E-03 | CCND1 |
| SLC1A2 | transporter | | 5,60E-03 | FOS |
| GTPBP4 | enzyme | | 5,60E-03 | CCND1 |
| GPHB5 | other | | 5,60E-03 | FOS |
| SCN7A | ion channel | | 5,60E-03 | FOS |
| RNF7 | enzyme | | 5,60E-03 | JUN |
| NAPB | transporter | | 5,60E-03 | CYP1A1 |
| ANKS1A | other | | 5,60E-03 | FOS |
| NKX2-8 | transcription regulator | | 5,60E-03 | AFP |
| PTPRU | phosphatase | | 5,60E-03 | CCND1 |
| MED21 | transcription regulator | | 5,60E-03 | TNFAIP3 |
| PMS2 | enzyme | | 5,60E-03 | CCND1 |
| Sin3b | transcription regulator | | 5,60E-03 | MYC |
| EPGN | growth factor | | 5,60E-03 | FOS |
| RSL1D1 | other | | 5,60E-03 | PLAU |
| TAX1BP3 | transcription regulator | | 5,60E-03 | FOS |
| Tnxa-ps1 | other | | 5,60E-03 | DUSP1 |
| PRDM2 | transcription regulator | | 5,60E-03 | MYC |
| RAB5B | enzyme | | 5,60E-03 | FOS |
| CDK20 | kinase | | 5,60E-03 | CCND1 |
| GDF10 | growth factor | | 5,60E-03 | SERPINE1 |
| cytarabine/filgrastim/fludarabine phosphate | biologic drug | | 5,60E-03 | FOS |
| ETS1-AS1 | other | | 5,60E-03 | ETS1 |
| PTOV1 | other | | 5,60E-03 | CCND1 |
| PRDX6 | enzyme | | 5,60E-03 | PLAU |
| INHBE | growth factor | | 5,60E-03 | JUNB |
| EXOSC10 | kinase | | 5,60E-03 | FOS |
| PPP2R3A | phosphatase | | 5,60E-03 | MYC |
| ZBTB14 | transcription regulator | | 5,60E-03 | MYC |
| RPS20 | other | | 5,60E-03 | MYC |
| CHRM2 | G-protein coupled receptor | | 5,60E-03 | EGR1 |
| Flt1 | kinase | | 5,60E-03 | ATF3 |
| molibresib | chemical drug | | 5,60E-03 | TNFRSF10B |
| oligomycin A | chemical - endogenous non-mammalian | | 5,60E-03 | TXNIP |
| CML advanced glycation end products | chemical reagent | | 5,60E-03 | EGR1 |
| dihydrotanshinone I | chemical reagent | | 5,60E-03 | TNFRSF10B |
| NMS-873 | chemical reagent | | 5,60E-03 | MYC |
| RPL21 | other | | 5,60E-03 | MYC |
| SLC25A5 | transporter | | 5,60E-03 | TNFRSF10B |
| RPLP0 | other | | 5,60E-03 | CCND1 |
| renin-angiotensin-aldosterone system blocking agent | chemical drug | | 5,60E-03 | AGT |
| NBI 27914 | chemical drug | | 5,60E-03 | FOS |
| alpha-cyano-4-hydroxycinnamate | chemical reagent | | 5,60E-03 | AGT |
| SB202474 | chemical - kinase inhibitor | | 5,60E-03 | CYP1A1 |
| impromidine | chemical drug | | 5,60E-03 | LDLR |
| guanfacine | chemical drug | | 5,60E-03 | FOS |
| amlexanox | chemical drug | | 5,60E-03 | CCND1 |
| sterigmatocystin | chemical toxicant | | 5,60E-03 | CYP1A1 |
| ondansetron | chemical drug | | 5,60E-03 | FOS |
| cyclothiazide | chemical drug | | 5,60E-03 | EGR1 |
| SU6657 | chemical - kinase inhibitor | | 5,60E-03 | MYC |
| flurothyl | chemical drug | | 5,60E-03 | FOS |
| L 733060 | chemical reagent | | 5,60E-03 | FOS |
| SC-19220 | chemical reagent | | 5,60E-03 | ICAM1 |
| sulfaphenazole | chemical drug | | 5,60E-03 | CCND1 |
| remikiren | chemical drug | | 5,60E-03 | AGT |
| selfotel | chemical drug | | 5,60E-03 | FOS |
| (R)-5-(2-azetidnylmethoxy)-2-chloropyridine | chemical drug | | 5,60E-03 | FOS |
| Ro 20-1724 | chemical reagent | | 5,60E-03 | CCND1 |
| BTG3-AS1 | other | | 5,60E-03 | ATF3 |
| GS-626510 | chemical reagent | | 5,60E-03 | MYC |
| pubchem compound 353999 | chemical reagent | | 5,60E-03 | MYC |
| triazolam | chemical drug | | 5,60E-03 | PER1 |
| thiabendazole | chemical drug | | 5,60E-03 | CYP1A1 |
| arachidonyl-2-chloroethylamide | chemical reagent | | 5,60E-03 | EGR1 |
| naphthalene-1-2-dione | chemical toxicant | | 5,60E-03 | CYP1A1 |
| trans-7,8-dihydroxy-7,8-dihydrobenzo(a)pyrene | chemical toxicant | | 5,60E-03 | CYP1A1 |
| anthracene | chemical toxicant | | 5,60E-03 | CYP1A1 |
| vitamin K1 | chemical - endogenous mammalian | | 5,60E-03 | AFP |
| sodium 2-((6Z,15Z)-20-hydroxyicosa-6,15-dienamido)acetate | chemical reagent | | 5,60E-03 | AGT |
| ACS-1 | chemical reagent | | 5,60E-03 | MYC |
| 2-amino-3-methylimidazo(4,5-f)quinoline | chemical toxicant | | 5,60E-03 | CYP1A1 |
| ONO-8711 | chemical drug | | 5,60E-03 | FOS |
| anti-miR-302d | chemical reagent | | 5,60E-03 | CCND1 |
| AP-1 decoy | chemical reagent | | 5,60E-03 | DUSP1 |
| anti-miR-302c inhibitor | chemical reagent | | 5,60E-03 | CCND1 |
| beta-penta-O-galloyl-glucose | chemical - endogenous non-mammalian | | 5,60E-03 | CCND1 |
| BRD4-NUTM1 | fusion gene/product | | 5,60E-03 | MYC |
| ZBTB16-RARA | fusion gene/product | | 5,60E-03 | DUSP6 |
| SU11333 | chemical - kinase inhibitor | | 5,60E-03 | MYC |
| CTOP | chemical reagent | | 5,60E-03 | FOS |
| PF-4800567 | chemical - kinase inhibitor | | 5,60E-03 | MYC |
| chymostatin | chemical - protease inhibitor | | 5,60E-03 | AGT |
| NCB-0846 | chemical - kinase inhibitor | | 5,60E-03 | JUN |
| Ac-DEVD-CHO | chemical reagent | | 5,60E-03 | FOS |
| ibutamoren | chemical drug | | 5,60E-03 | FOS |
| SMAD3-EP300 | complex | | 5,60E-03 | SERPINE1 |
| 3-methoxyapigenin | chemical - endogenous non-mammalian | | 5,60E-03 | MYC |
| TEI-F00806 | chemical - protease inhibitor | | 5,60E-03 | AGT |
| berberrubine | chemical - endogenous non-mammalian | | 5,60E-03 | LDLR |
| columbamine | chemical - endogenous non-mammalian | | 5,60E-03 | LDLR |
| 2-methoxy-5-(2',3',4'-trimethoxyphenyl)-2,4,6-cycloheptatrien-1-one | chemical reagent | | 5,60E-03 | JUN |
| harmaline | chemical drug | | 5,60E-03 | CYP1A1 |
| LY 354740 | chemical drug | | 5,60E-03 | FOS |
| droxidopa | chemical drug | | 5,60E-03 | FOS |
| S(-)-carbidopa | chemical drug | | 5,60E-03 | FOS |
| 3'-adenylic acid | chemical - endogenous mammalian | | 5,60E-03 | TXNIP |
| 2'-fucosyllactose | chemical - endogenous mammalian | | 5,60E-03 | ICAM1 |
| sarcosine | chemical - endogenous mammalian | | 5,60E-03 | FOS |
| allose | chemical - endogenous mammalian | | 5,60E-03 | TXNIP |
| estramustine | chemical drug | | 5,60E-03 | CYP1A1 |
| miR-6867-3p (and other miRNAs w/seed UCUCCCU) | mature microRNA | Inhibited | 5,69E-03 | DUSP6,EGR1,EMP1,ETS1,GJB1,H3C14,H3C15,MAFF,NDRG1,PALD1,PLK2,SPATA2,TRRAP |
| miR-5107-3p (and other miRNAs w/seed AACCUGU) | mature microRNA | Inhibited | 5,70E-03 | BIRC3,ERRFI1,H3C14,H3C15,ITGA2,ITGA6,PDZK1,SPRED1 |
| geldanamycin | chemical - endogenous non-mammalian | | 5,73E-03 | CCND1,CYP1A1,JUN,MDM2,MYC,TRRAP |
| PRMT1 | enzyme | | 5,83E-03 | CCND1,SERPINE1,VIM |
| CUX1 | transcription regulator | | 5,83E-03 | CXCL2,MYC,VIM |
| sulindac | chemical drug | | 5,83E-03 | CCND1,CYP1A1,MYC |
| miR-5131 (and other miRNAs w/seed GGCGCCC) | mature microRNA | Inhibited | 5,85E-03 | CCND1,H3C14,H3C15,IER2,JUNB,PHLDA2,RHOB,ZBTB43,ZNF554 |
| RUNX3 | transcription regulator | | 5,87E-03 | CCND1,MYC,SPRED1,VIM |
| MECP2 | transcription regulator | | 5,87E-03 | EGR1,JUNB,MYC,VIM |
| miR-223-3p (miRNAs w/seed GUCAGUU) | mature microRNA | Inhibited | 5,95E-03 | ARRDC3,CCND1,CTSV,DUSP6,MDM2,RGS2,RHOB,SPRED1,TENT5A,VIM |
| miR-520d-5p (and other miRNAs w/seed UACAAAG) | mature microRNA | | 5,97E-03 | BCL10,JUN,OAT,PHLDA2,TENT5A,TSPAN8,UBASH3B |
| CD 437 | chemical drug | | 6,01E-03 | FOS,JUN,MYC,NFKBIA,TNFRSF10B |
| NANOG | transcription regulator | | 6,04E-03 | EGR1,FOS,LAMA1,TBX3 |
| MBD2 | transcription regulator | | 6,08E-03 | BIRC3,OAT,PLAU |

| | | | | |
|---|---|---|---|---|
| hydroxyurea | chemical drug | | 6,08E-03 | CCND1,MDM2,MYC |
| Stat5 dimer | complex | | 6,10E-03 | CCND1,MYC |
| LGR5 | transmembrane receptor | | 6,10E-03 | AKR1C3,OAT |
| trametinib | chemical drug | | 6,10E-03 | MYC,TNFAIP3 |
| MED12 | transcription regulator | | 6,10E-03 | ANKRD1,MYC |
| SPZ1 | transcription regulator | | 6,10E-03 | CCND1,VIM |
| ACKR1 | G-protein coupled receptor | | 6,10E-03 | ICAM1,TNFAIP3 |
| PER1 | transcription regulator | | 6,10E-03 | CCND1,PER1 |
| CDK8 | kinase | | 6,10E-03 | CXCL2,MYC |
| CYP2J2 | enzyme | | 6,10E-03 | ICAM1,NFKBIA |
| PARK7 | enzyme | | 6,10E-03 | ICAM1,VIM |
| W7 | chemical reagent | | 6,10E-03 | BIRC3,FOS |
| pilocarpine | chemical drug | | 6,10E-03 | FOS,JUNB |
| nickel | chemical toxicant | | 6,10E-03 | ICAM1,SERPINE1 |
| IRF3 | transcription regulator | | 6,14E-03 | BIRC3,ICAM1,JUNB,TNFAIP3,VIM |
| dimethyl sulfoxide | chemical drug | | 6,20E-03 | CYP1A1,FOS,JUN,MYC |
| miR-519a-3p (and other miRNAs w/seed AAGUGCA) | mature microRNA | Inhibited | 6,21E-03 | DCBLD2,FABP1,H2BC5,JUNB,KLF10,LDLR,MXD1,NR1D1,SMIM14,TIPARP,TNFRSF10B |
| miR-214-3p (and other miRNAs w/seed CAGCAGG) | mature microRNA | | 6,28E-03 | ERRFI1,ETS1,IER3,INAFM1,MAFF,NUAK2,PALD1,PER1,PI3,PMM1,PRR15L,RHOB,SLC25A25,TENT5A,ZBTB20 |
| GATA1 | transcription regulator | | 6,32E-03 | CCND1,EGR1,ETS1,ITGA6,JUNB,MYC |
| WNT3A | cytokine | | 6,32E-03 | CCND1,JUN,MYC,TBX3,TXNIP,VIM |
| vitamin E | chemical drug | | 6,34E-03 | EGR1,KLF6,SERPINE1 |
| CIP2A | other | | 6,34E-03 | IER3,LAMA1,MYC |
| cytarabine | chemical drug | | 6,34E-03 | ICAM1,MDM2,TNFRSF10B |
| WNT5A | cytokine | | 6,37E-03 | CCND1,CXCL2,MYC,VIM |
| IFNB1 | cytokine | | 6,42E-03 | AXL,BHLHE40,CXCL2,CYP1A1,FOS,MYC |
| miR-4684-3p (miRNAs w/seed GUUGCAA) | mature microRNA | | 6,44E-03 | GSTA2,LAMA1,LCA5,PARD6B,RHOB,SELENOW,SEPHS2,SPRED1,TENT5A |
| miR-8064 (miRNAs w/seed GCACACU) | mature microRNA | Inhibited | 6,51E-03 | AGT,IER2,JUN,PARD6B,SERPINE1,TENT5A,YOD1,ZFP36,ZNF627 |
| miR-4493 (miRNAs w/seed GAAGGCC) | mature microRNA | Inhibited | 6,51E-03 | BIRC3,ICAM1,INAFM1,KLF6,NDRG1,NUAK2,SERPINE1,SPATA2,TNS4,ZFP36,ZNF627,ZNF844 |
| TNFSF13B | cytokine | | 6,60E-03 | ICAM1,JUN,MYC |
| CaMKII | complex | | 6,69E-03 | BIRC3,FOS |
| EIF4G2 | translation regulator | | 6,69E-03 | JUN,TBX3 |
| CCK | other | | 6,69E-03 | EGR1,FOS |
| NFIX | transcription regulator | | 6,69E-03 | CCND1,SERPINE1 |
| MAP3K5 | kinase | | 6,69E-03 | CCND1,JUN |
| GADD45A | other | | 6,69E-03 | CXCL2,JUN |
| CCN3 | growth factor | | 6,69E-03 | ITGA2,SERPINE1 |
| PTPN3 | phosphatase | | 6,69E-03 | MYC,SERPINE1 |
| PDGFC | growth factor | | 6,69E-03 | CCND1,MYC |
| HSP90AB1 | enzyme | | 6,69E-03 | ATF3,MYC |
| ketamine | chemical drug | | 6,69E-03 | FOS,KLF6 |
| selenomethylselenocysteine | chemical - endogenous mammalian | | 6,69E-03 | EGR1,JUN |
| GLI1 | transcription regulator | | 6,77E-03 | CCND1,PHLDA2,RHOB,TNFRSF10B,VIM |
| MYRF | transcription regulator | | 6,88E-03 | AKR1C3,JUNB,TENT5A |
| EFNA1 | other | | 6,88E-03 | DCBLD2,DUSP6,EPHA2 |
| gemcitabine | chemical drug | | 6,88E-03 | CCND1,EGR1,MYC |
| miR-12185-3p (and other miRNAs w/seed CAGCCAC) | mature microRNA | Inhibited | 6,96E-03 | ARRDC3,CXCL2,DYRK1A,MYC,NDRG1,PALD1,PLK2,POLR2J2/POLR2J3,RHOB,SELENOW,ZBTB43 |
| miR-561-5p (miRNAs w/seed UCAAGGA) | mature microRNA | Inhibited | 7,02E-03 | BIRC3,DCBLD2,MXD1,PLK2,PRR15L,RGS2,RHOB,SPRED1,TNS4,TXNIP,YOD1 |
| APC | enzyme | | 7,07E-03 | CCND1,CXCL2,MXD1,MYC |
| miR-145-5p (and other miRNAs w/seed UCCAGUU) | mature microRNA | Inhibited | 7,15E-03 | B4GALNT2,BCL10,DUSP6,DYRK1A,MDM2,MYC,SERPINE1,SLC25A25,ZFP36 |
| DKK1 | growth factor | | 7,15E-03 | CCND1,MYC,TBX3 |
| clopidogrel | chemical drug | | 7,15E-03 | ATF3,MYC,PPP1R15A |
| SUZ12 | enzyme | | 7,26E-03 | ABL2,MYC,PLAU,TBX3 |
| miR-4671-5p (miRNAs w/seed CCGAAGA) | mature microRNA | | 7,28E-03 | EPHA2,IER3,LDLR,REEP6,SPRY4,TRRAP |
| zidovudine | chemical drug | | 7,30E-03 | BCL10,MYC |
| pregna-4,17-diene-3,16-dione | chemical - endogenous non-mammalian | | 7,30E-03 | CCND1,MYC |
| FAM3B | cytokine | | 7,30E-03 | IER3,IGFBP4 |
| Pde4 | group | | 7,30E-03 | ICAM1,LDLR |
| MGP | other | | 7,30E-03 | AFP,TTR |
| TF | transporter | | 7,30E-03 | CCND1,NFKBIA |
| EYA1 | phosphatase | | 7,30E-03 | MYC,TBX3 |
| TNFAIP6 | other | | 7,30E-03 | CXCL2,VIM |
| CNR2 | G-protein coupled receptor | | 7,30E-03 | CCND1,EGR1 |
| DYRK1A | kinase | | 7,30E-03 | CCND1,SERPINE1 |
| Sch-23390 | chemical drug | | 7,30E-03 | EGR1,FOS |
| epicatechin gallate | chemical drug | | 7,30E-03 | ICAM1,MDM2 |
| 2,4-dinitrofluorobenzene | chemical toxicant | | 7,30E-03 | CXCL2,ICAM1 |
| 9Z,11E-octadecadienoic acid | chemical - endogenous mammalian | | 7,30E-03 | FABP1,LDLR |
| NFATC3 | transcription regulator | | 7,44E-03 | ATF3,FOS,JUN |
| PKM | kinase | | 7,44E-03 | CCND1,EGR1,MYC |
| GDNF | growth factor | | 7,44E-03 | CCND1,EGR1,ITGA6 |
| MAP2K3 | kinase | | 7,44E-03 | ICAM1,PLAU,SERPINE1 |
| IL22 | cytokine | | 7,44E-03 | CCND1,CXCL2,MYC,PI3 |
| ADORA2A | G-protein coupled receptor | | 7,44E-03 | CXCL2,DUSP1,FOS,NFKBIA |
| CASR | G-protein coupled receptor | Activated | 7,44E-03 | CCND1,EGR1,IER3,TXNIP |
| miR-2355-5p (miRNAs w/seed UCCCCAG) | mature microRNA | Inhibited | 7,48E-03 | ARRDC3,ASB2,AXL,CTSV,EMP1,EPHA2,ERRFI1,ETS1,KLF6,LAMA1,RHOB,SLC25A25,SMIM14,TNFAIP3,ZNF608 |
| miR-605-3p (miRNAs w/seed GAAGGCA) | mature microRNA | Inhibited | 7,57E-03 | AFAP1,ALDH1A1,ARRDC3,BIRC3,DCBLD2,DUSP1,EPHA2,JUN,NFKBIZ,NUAK2,SERPINE1,UBASH3B,ZNF627,ZNF844 |
| HIPK2 | kinase | | 7,73E-03 | EGR1,MDM2,VIM |
| miR-3190-5p (miRNAs w/seed CUGGCCA) | mature microRNA | Inhibited | 7,77E-03 | AKR1C3,ASB2,ATF3,CXCL2,ETS1,H3C14,H3C15,LDLR,PHLDA2,PLAU,POLR2J2/POLR2J3,SMIM14,ZNF555 |
| miR-7854-3p (miRNAs w/seed GAGGUGA) | mature microRNA | | 7,77E-03 | AKR1C3,ARRDC3,BCL10,DUSP6,EGR1,H3C14,H3C15,INAFM1,NUAK2,PRR15L,REEP6,TTR,ZBTB43 |
| miR-6894-3p (miRNAs w/seed UGCCUGC) | mature microRNA | Inhibited | 7,84E-03 | AFAP1,BHLHE40,BIRC3,EMP1,ERRFI1,GJB1,H2BC5,MDM2,NDRG1,TXNIP,ZNF608 |
| ADCY | group | | 7,93E-03 | FOS,PER1 |
| JUN/JUNB/JUND | group | | 7,93E-03 | FOS,JUN |
| KISS1 | other | | 7,93E-03 | EGR1,FOS |
| UCN | other | | 7,93E-03 | FOS,ICAM1 |
| WNT7A | cytokine | | 7,93E-03 | SPRY4,VIM |
| SAFB2 | other | | 7,93E-03 | FOS,JUN |
| COL4A3 | other | | 7,93E-03 | ITGA2,LAMA1 |
| VIM | other | | 7,93E-03 | AXL,PLAU |
| NTRK1 | kinase | | 7,93E-03 | EGR1,FOS |
| ACE | peptidase | | 7,93E-03 | AGT,SERPINE1 |
| HEY1 | transcription regulator | | 7,93E-03 | MDM2,OAT |
| THZ1 | chemical - kinase inhibitor | | 7,93E-03 | BIRC3,MYC |
| bilirubin | chemical - endogenous mammalian | | 7,93E-03 | CCND1,GSTA2 |
| SOX11 | transcription regulator | | 8,02E-03 | CCND1,EGR1,FGG,IER2 |
| molybdenum disulfide | chemical reagent | | 8,02E-03 | AKR1C3,DCBLD2,DUSP6,PHLDA1 |
| PIK3CA | kinase | | 8,03E-03 | CCND1,SERPINE1,VIM |
| SOX1 | transcription regulator | | 8,03E-03 | DUSP1,EPHA2,FABP1 |
| E2F2 | transcription regulator | | 8,03E-03 | CCND1,MYC,SERPINE1 |
| HSF1 | transcription regulator | | 8,04E-03 | BCL10,ICAM1,JUN,LDLR,TTR |
| miR-4752 (miRNAs w/seed UGUGGAU) | mature microRNA | Inhibited | 8,33E-03 | ETS1,MXD1,NFKBIA,SELENOW,SMIM14,TENT5A,UBASH3B,ZNF442,ZNF627 |
| Tcf7 | transcription regulator | | 8,34E-03 | GEM,KLF6,LDLR,MXD1,PHLDA1 |
| ritonavir | chemical drug | | 8,42E-03 | CYP1A1,ICAM1,LDLR,MYC |
| miR-4523 (miRNAs w/seed ACCGAGA) | mature microRNA | | 8,50E-03 | BCL10,H3C14,H3C15,KRT6A,NDRG1 |
| miR-130a-3p (and other miRNAs w/seed AGUGCAA) | mature microRNA | Inhibited | 8,54E-03 | ARRDC3,BHLHE40,CXCL2,CYLD,DCBLD2,FABP1,H2BC5,HIVEP2,KLF6,LDLR,MAFF,MXD1,SPRED1 |
| leukotriene B4 | chemical - endogenous mammalian | | 8,59E-03 | CXCL2,JUN |
| CHD7 | enzyme | | 8,59E-03 | MDM2,TNFRSF10B |
| calpain | complex | | 8,59E-03 | CCND1,NFKBIA |
| Smad | complex | | 8,59E-03 | MYC,SERPINE1 |
| E2F5 | transcription regulator | | 8,59E-03 | MYC,SERPINE1 |
| GHRH | other | | 8,59E-03 | CCND1,FOS |
| SOX6 | transcription regulator | | 8,59E-03 | CCND1,MYC |
| LRPAP1 | other | | 8,59E-03 | LDLR,SERPINE1 |
| UBE3A | enzyme | | 8,59E-03 | MDM2,SERPINE1 |
| HEXIM1 | transcription regulator | | 8,59E-03 | CCND1,VIM |
| COMMD1 | transporter | | 8,59E-03 | BIRC3,NFKBIA |
| TRPV4 | ion channel | | 8,59E-03 | CXCL2,FOS |
| SDCBP | enzyme | | 8,59E-03 | ITGA6,VIM |
| SSTR2 | G-protein coupled receptor | | 8,59E-03 | FOS,VIM |
| PCYT1A | enzyme | | 8,59E-03 | AXL,CXCL2 |
| UCN-01 | chemical drug | | 8,59E-03 | CCND1,RHOB |
| miR-3186-3p (and other miRNAs w/seed CACGCGG) | mature microRNA | Inhibited | 8,65E-03 | H3C14,H3C15,ICAM1,LDLR,MXD1 |
| miR-450b-5p (miRNAs w/seed UUUGCAA) | mature microRNA | | 8,94E-03 | BCL10,ERRFI1,GEM,LCA5,PHLDA2,SEPHS2,SMIM14,ZBTB20 |
| STK40 | kinase | | 8,97E-03 | DUSP6,JUN,SPRY4 |
| minocycline | chemical drug | | 8,97E-03 | ATF3,FOS,ICAM1 |
| nifedipine | chemical drug | | 8,97E-03 | AGT,FOS,LDLR |
| miR-200a-5p (and other miRNAs w/seed AUCUUAC) | mature microRNA | | 9,12E-03 | CTSV,KLF6,RGS2,SEPHS2,ZNF441,ZNF627 |
| corticosterone | chemical - endogenous mammalian | | 9,26E-03 | CCND1,FOS,JUN,MYC |
| CNR1 | G-protein coupled receptor | | 9,26E-03 | ERRFI1,FOS,LAMA1,PLAU |
| thyroid hormone receptor | group | | 9,27E-03 | FOS,MYC |
| DCAF1 | kinase | | 9,27E-03 | KLF10,TXNIP |
| OXT | other | | 9,27E-03 | FOS,RGS2 |
| MST1 | growth factor | | 9,27E-03 | CCND1,FOS |
| CXCL10 | cytokine | | 9,27E-03 | FOS,ITGA2 |
| RCAN1 | transcription regulator | | 9,27E-03 | FOS,ICAM1 |
| MTA3 | transcription regulator | | 9,27E-03 | MDM2,MYC |
| HDAC9 | transcription regulator | | 9,27E-03 | JUN,RGS2 |
| SUPT16H | transcription regulator | | 9,27E-03 | EGR1,PLAU |
| NOS1 | enzyme | | 9,27E-03 | EGR1,FOS |
| PSMD10 | transcription regulator | | 9,27E-03 | CCND1,ICAM1 |
| HSPD1 | enzyme | | 9,27E-03 | CXCL2,ICAM1 |
| diazoxide | chemical drug | | 9,27E-03 | EGR1,MYC |
| K-252 | chemical drug | | 9,27E-03 | JUN,NDRG1 |
| calyculin A | chemical toxicant | | 9,27E-03 | CCND1,EGR1 |
| arotinoid acid | chemical toxicant | | 9,27E-03 | DUSP1,FOS |
| cyanocobalamin | chemical - endogenous mammalian | | 9,27E-03 | JUN,PER1 |
| RBL1 | transcription regulator | | 9,29E-03 | CCND1,FOS,MYC |
| UCHL1 | peptidase | | 9,29E-03 | HDAC9,LDLR,SERPINE1 |
| miR-524-3p (and other miRNAs w/seed AAGGCGC) | mature microRNA | Inhibited | 9,30E-03 | AFAP1,EGR1,H3C14,H3C15,PHLDA2 |
| FGF7 | growth factor | | 9,63E-03 | CCND1,PLAU,SERPINE1 |
| ROR2 | kinase | | 9,63E-03 | CCND1,MDM2,SPRY4 |
| Bay 11-7082 | chemical - kinase inhibitor | | 9,63E-03 | CCND1,CXCL2,ICAM1 |
| CDX2 | transcription regulator | | 9,70E-03 | ETS1,JUN,MYC,PARD6B |
| miR-4504 (miRNAs w/seed GUGACAA) | mature microRNA | | 9,83E-03 | ABL2,AHSG,ATF3,ERRFI1,ETS1,MDM2,PMM1,TNFRSF10B,YOD1 |
| miR-532-5p (and other miRNAs w/seed AUGCCUU) | mature microRNA | Inhibited | 9,85E-03 | AHSG,ANKRD1,CXCL2,ITGA2,LCA5,PLK2,RHOB,ZNF627 |
| HDAC5 | transcription regulator | | 9,97E-03 | JUN,KLF6,MYC |
| TBX5 | transcription regulator | | 9,97E-03 | ANKRD1,CXCL2,VIM |
| DYSF | other | | 9,97E-03 | ANKRD1,CTSV,ERRFI1 |
| picrotoxin | chemical toxicant | | 9,98E-03 | CYP1A1,FOS |
| UTP | chemical - endogenous mammalian | | 9,98E-03 | ICAM1,SERPINE1 |
| p70 S6k | group | | 9,98E-03 | CCND1,VIM |
| AMH | growth factor | | 9,98E-03 | CXCL2,IER3 |
| vemurafenib | chemical drug | | 9,98E-03 | DUSP6,TNFRSF10B |
| NCL | other | | 9,98E-03 | MDM2,MYC |
| mir-148 | microRNA | | 9,98E-03 | EGR1,LDLR |
| BGN | other | | 9,98E-03 | CCND1,SERPINE1 |
| MAP2K2 | kinase | | 9,98E-03 | FOS,MYC |
| CPE | peptidase | | 9,98E-03 | LDLR,MYC |
| isoflurane | chemical drug | | 9,98E-03 | FOS,ICAM1 |
| miR-1255b-5p (and other miRNAs w/seed GGAUGAG) | mature microRNA | Inhibited | 1,01E-02 | ANKRD1,EMP1,EPHA2,ETS1,H2BC5,IGFBP4,ITGA2,MDM2,PI3,PLAU,TIPARP |
| miR-4433b-3p (miRNAs w/seed AGGAGUG) | mature microRNA | | 1,01E-02 | CCND1,CSRNP1,DUSP1,GSTA2,IGFBP4,ITGA2,MDM2,SMIM14,TIPARP,VIM |
| NR1H3 | ligand-dependent nuclear receptor | | 1,01E-02 | ATF3,CXCL2,LDLR,NFKBIA,PLAU |
| RETNLB | other | | 1,03E-02 | FABP1,LAMA1,MYC |
| PIN1 | enzyme | | 1,03E-02 | CCND1,MDM2,MYC |
| SOX3 | transcription regulator | | 1,03E-02 | DUSP1,EPHA2,FABP1 |
| nelfinavir | chemical drug | | 1,03E-02 | ATF3,PPP1R15A,TNFRSF10B |
| miR-4677-5p (miRNAs w/seed UGUUCUU) | mature microRNA | Inhibited | 1,04E-02 | AGT,AKAP12,BIRC3,CXCL2,CYP1A1,NFKBIA,NFKBIZ,YOD1 |
| miR-452-3p (miRNAs w/seed UCAUCUG) | mature microRNA | | 1,04E-02 | ETS1,FABP1,ITGA6,JUN,KLF10,LAMA1,LDLR,MDM2,UBASH3B |
| miR-4503 (miRNAs w/seed UUAAGCA) | mature microRNA | | 1,05E-02 | CTSV,EGR1,FABP1,KLF10,PDZK1,SPRED1,TENT5A,ZNF554 |
| miR-3926 (miRNAs w/seed GGCCAAA) | mature microRNA | | 1,05E-02 | IGFBP4,KLF6,OAT,PHLDA2,PMM1,PRR15L,SPRY4,ZNF441,ZNF555,ZNF844 |
| miR-129-5p (and other miRNAs w/seed UUUUUGC) | mature microRNA | Inhibited | 1,05E-02 | ARRDC3,BCL10,EMP1,ETS1,ZBTB20 |
| miR-4687-5p (miRNAs w/seed AGCCCUC) | mature microRNA | Inhibited | 1,05E-02 | ANKRD1,ASB2,BHLHE40,DUSP1,EGR1,IGFBP4,KLF6,RHOB,SERINC5 |
| miR-4301 (miRNAs w/seed CCCACUA) | mature microRNA | Inhibited | 1,06E-02 | DUSP1,H3C14,H3C15,JUN,MAFF,NFKBIZ,SMIM14,TENT5A,ZNF554 |
| estradiol benzoate | chemical drug | | 1,07E-02 | FOS,GJB1,PLAU |
| JUND | transcription regulator | | 1,07E-02 | AGT,CCND1,PLAU |
| cholesterol | chemical - endogenous mammalian | | 1,07E-02 | AGT,ATF3,CXCL2,ICAM1,LDLR |
| NCF1 | enzyme | | 1,07E-02 | ICAM1,SERPINE1 |
| HMGN1 | transcription regulator | | 1,07E-02 | FOS,TTR |
| USP7 | peptidase | | 1,07E-02 | FABP1,MDM2 |
| ADA | enzyme | | 1,07E-02 | CXCL2,SERPINE1 |
| TAF6 | transcription regulator | | 1,07E-02 | ATF3,JUN |
| MALP-2s | chemical reagent | | 1,07E-02 | CXCL2,CYLD |
| apomorphine | chemical drug | | 1,07E-02 | EGR1,FOS |
| phorbol 12,13-didecanoate | chemical toxicant | | 1,07E-02 | DUSP1,MYC |
| progestin | chemical drug | | 1,07E-02 | CCND1,NFKBIA |
| miR-1237-3p (and other miRNAs w/seed CCUUCUG) | mature microRNA | | 1,08E-02 | AHSG,ETS1,H2BC5,H3C14,H3C15,MAFF,REEP6,SPRED1,SPRY4 |
| miR-5008-5p (miRNAs w/seed GAGGCCC) | mature microRNA | Inhibited | 1,08E-02 | AFAP1,ETS1,FOS,H3C14,H3C15,KLF6,MAFF,PER1,RHOB,SPATA2,TNFAIP3,TNS4,UBASH3B,ZC3H12A |
| miR-8088 (miRNAs w/seed CUCGGUA) | mature microRNA | | 1,08E-02 | ANKRD1,EMP1,H2BC5,REEP6,ZNF555,ZNF844 |
| miR-3692-3p (miRNAs w/seed UUCCACA) | mature microRNA | Inhibited | 1,08E-02 | ARRDC3,FOS,JUNB,SPRED1,TIPARP,ZNF441,ZNF608,ZNF844 |
| miR-6888-5p (miRNAs w/seed AGGAGAU) | mature microRNA | | 1,09E-02 | ALDH1A1,BCL10,GSTA2,IER2,IGFBP4,SERPINA6,TENT5A,TXNIP,ZFP36 |
| miR-567 (miRNAs w/seed GUAUGUU) | mature microRNA | | 1,10E-02 | ALDH1A1,ARRDC3,EMP1,ERRFI1,GSTA2,LCA5,SMIM14 |
| Rb | group | | 1,10E-02 | CCND1,CYP1A1,MYC |
| MBD3 | other | | 1,10E-02 | CCND1,MDM2,MYC |
| C3 | peptidase | | 1,10E-02 | CXCL2,JUN,TTR |
| FLT1 | kinase | | 1,10E-02 | FOS,ICAM1,TNFRSF10B |
| miR-34a-5p (and other miRNAs w/seed GGCAGUG) | mature microRNA | Inhibited | 1,11E-02 | ANKRD1,ATF3,AXL,CCND1,FABP1,FOS,ICAM1,MAFF,MYC,NDRG1,NFKBIA,PARD6B,POLR2J2/POLR2J3,SMIM14,TENT5A,UBASH3B,ZFP36,ZNF844 |
| miR-6800-3p (miRNAs w/seed ACCUCUC) | mature microRNA | | 1,11E-02 | ABL2,BHLHE40,CXCL2,PLAU,PMM1,SMIM14,TIPARP,ZBTB43 |
| PTGER4 | G-protein coupled receptor | | 1,11E-02 | ARRDC3,EGR1,ICAM1,MAFF |
| miR-3940-3p (miRNAs w/seed AGCCCGG) | mature microRNA | Inhibited | 1,11E-02 | ATF3,H3C14,H3C15,IER2,KLF6,SLC25A25,VGF |
| cysteinyl-leukotriene | chemical - endogenous mammalian | | 1,12E-02 | FOS |
| L-leucine | chemical - endogenous mammalian | | 1,12E-02 | CXCL2 |
| isopropyl thiogalactoside | chemical reagent | | 1,12E-02 | CCND1 |
| ginsenoside Rh2 | chemical - endogenous non-mammalian | | 1,12E-02 | FOS |
| Cop9 Signalosome | complex | | 1,12E-02 | JUN |
| CERT1 | kinase | | 1,12E-02 | CCND1 |
| chartreusin | chemical reagent | | 1,12E-02 | TNFRSF10B |
| Pak | group | | 1,12E-02 | MDM2 |
| Cofilin | group | | 1,12E-02 | MYC |
| PLA2 | group | | 1,12E-02 | FOS |
| 2,2',4,4'-tetrabromodiphenyl ether | chemical reagent | | 1,12E-02 | CYP1A1 |
| hexadecanedioic acid | chemical - endogenous mammalian | | 1,12E-02 | FABP1 |
| diosmetin | chemical - endogenous non-mammalian | | 1,12E-02 | CYP1A1 |
| DF 203 | chemical drug | | 1,12E-02 | CYP1A1 |
| NPS | other | | 1,12E-02 | FOS |
| nitrosomethylurethane | chemical reagent | | 1,12E-02 | CCND1 |
| canthaxanthin | chemical - endogenous non-mammalian | | 1,12E-02 | CYP1A1 |
| Rps15a-ps4 | other | | 1,12E-02 | NFKBIA |
| Rab11 | group | | 1,12E-02 | ITGA6 |
| USP42 | peptidase | | 1,12E-02 | MDM2 |
| RBMS3 | other | | 1,12E-02 | MYC |
| FAM168A | other | | 1,12E-02 | NFKBIA |
| ADRM1 | other | | 1,12E-02 | NFKBIA |
| REM2 | enzyme | | 1,12E-02 | CCND1 |
| OSI-027 | chemical drug | | 1,12E-02 | CCND1 |
| TRIM71 | enzyme | | 1,12E-02 | MYC |
| GPR4 | G-protein coupled receptor | | 1,12E-02 | ICAM1 |
| BCAS3 | other | | 1,12E-02 | MYC |
| USP36 | peptidase | | 1,12E-02 | MYC |
| BATF2 | other | | 1,12E-02 | JUN |
| VENTX | transcription regulator | | 1,12E-02 | CCND1 |
| MLXIP | transcription regulator | | 1,12E-02 | TXNIP |
| FKRP | other | | 1,12E-02 | LAMA1 |
| MRGPRX1 | G-protein coupled receptor | | 1,12E-02 | EGR1 |
| ETS-ELK1 | complex | | 1,12E-02 | CCND1 |
| Gm-Csf Receptor | complex | | 1,12E-02 | MYC |
| MAP3K21 | kinase | | 1,12E-02 | VIM |

| | | | | |
|---|---|---|---|---|
| RYR3 | ion channel | | 1,12E-02 | ATF3 |
| INTERLEUKIN | group | | 1,12E-02 | FOS |
| EIF4H | translation regulator | | 1,12E-02 | CCND1 |
| RNMT | enzyme | | 1,12E-02 | CCND1 |
| NOSIP | other | | 1,12E-02 | MYC |
| dibromoacetic acid | chemical toxicant | | 1,12E-02 | MYC |
| MCF2L | other | | 1,12E-02 | CCND1 |
| MAPK8IP2 | other | | 1,12E-02 | FOS |
| CSDE1 | enzyme | | 1,12E-02 | MYC |
| ATP1A1 | transporter | | 1,12E-02 | LDLR |
| MMP10 | peptidase | | 1,12E-02 | CXCL2 |
| PTPRF | phosphatase | | 1,12E-02 | CCND1 |
| HLTF | transcription regulator | | 1,12E-02 | SERPINE1 |
| DAPK3 | kinase | | 1,12E-02 | CCND1 |
| ZNF224 | transcription regulator | | 1,12E-02 | TNFAIP3 |
| RAC3 | enzyme | | 1,12E-02 | CCND1 |
| PTPA | phosphatase | | 1,12E-02 | MYC |
| AMD1 | enzyme | | 1,12E-02 | MYC |
| LAMA1 | other | | 1,12E-02 | PLAU |
| STAM | other | | 1,12E-02 | MYC |
| mir-331 | microRNA | | 1,12E-02 | MYC |
| mir-605 | microRNA | | 1,12E-02 | MDM2 |
| mir-105 | microRNA | | 1,12E-02 | MYC |
| CENPX | other | | 1,12E-02 | MYC |
| RPL15 | other | | 1,12E-02 | MYC |
| CCNDBP1 | other | | 1,12E-02 | CCND1 |
| NASP | other | | 1,12E-02 | CCND1 |
| ARHGEF28 | other | | 1,12E-02 | FOS |
| FGB | other | | 1,12E-02 | ICAM1 |
| GAD1 | enzyme | | 1,12E-02 | FOS |
| VANGL1 | other | | 1,12E-02 | JUN |
| NEK4 | kinase | | 1,12E-02 | MDM2 |
| FBXO4 | enzyme | | 1,12E-02 | CCND1 |
| BCLAF1 | transcription regulator | | 1,12E-02 | CCND1 |
| TIAM2 | enzyme | | 1,12E-02 | VIM |
| RPS6KA2 | kinase | | 1,12E-02 | EGR1 |
| Hnrpfl | other | | 1,12E-02 | AGT |
| POU4F3 | transcription regulator | | 1,12E-02 | FOS |
| GABRG2 | ion channel | | 1,12E-02 | FOS |
| ILKAP | phosphatase | | 1,12E-02 | CCND1 |
| ING2 | transcription regulator | | 1,12E-02 | SERPINE1 |
| WASHC1 | other | | 1,12E-02 | MYC |
| TH | enzyme | | 1,12E-02 | FOS |
| RPA2 | other | | 1,12E-02 | MYC |
| silvestrol | chemical - endogenous non-mammalian | | 1,12E-02 | JUN |
| RPS25 | other | | 1,12E-02 | MYC |
| DPH1 | other | | 1,12E-02 | CCND1 |
| STAMBP | enzyme | | 1,12E-02 | MYC |
| GRAP | other | | 1,12E-02 | FOS |
| alirocumab | biologic drug | | 1,12E-02 | LDLR |
| FRAT1 | other | | 1,12E-02 | JUN |
| TRIP11 | transcription regulator | | 1,12E-02 | CYP1A1 |
| UBE2V1 | transcription regulator | | 1,12E-02 | FOS |
| PPP2R5A | phosphatase | | 1,12E-02 | MYC |
| RAMAC | other | | 1,12E-02 | MYC |
| TMPRSS4 | peptidase | | 1,12E-02 | PLAU |
| CRTAP | other | | 1,12E-02 | SERPINE1 |
| Bhlhe41 | transcription regulator | | 1,12E-02 | PER1 |
| RNF11 | enzyme | | 1,12E-02 | SERPINE1 |
| STYX | phosphatase | | 1,12E-02 | MYC |
| RPS4Y1 | other | | 1,12E-02 | VIM |
| ZFYVE16 | other | | 1,12E-02 | SERPINE1 |
| sorafenib derivative SC-1 | chemical reagent | | 1,12E-02 | CCND1 |
| verticillin A | chemical reagent | | 1,12E-02 | TNFRSF10B |
| LY3039478 | chemical drug | | 1,12E-02 | VIM |
| 2,2-(2-chlorophenyl-4'-chlorophenyl)-1,1-dichloroethene | chemical - endogenous mammalian | | 1,12E-02 | CYP1A1 |
| 1-(3-chlorophenyl)piperazine | chemical drug | | 1,12E-02 | FOS |
| 3-iodobenzylguanidine | chemical drug | | 1,12E-02 | MYC |
| NPC 15437 | chemical - kinase inhibitor | | 1,12E-02 | FOS |
| amsacrine | chemical drug | | 1,12E-02 | JUN |
| phenoxyacetic acid | chemical reagent | | 1,12E-02 | MYC |
| palytoxin | chemical toxicant | | 1,12E-02 | DUSP6 |
| alpha-endosulfan | chemical toxicant | | 1,12E-02 | CYP1A1 |
| phosphorylethanolamine | chemical - endogenous mammalian | | 1,12E-02 | CCND1 |
| primaquine | chemical drug | | 1,12E-02 | CYP1A1 |
| sarpogrelate | chemical drug | | 1,12E-02 | SERPINE1 |
| methiothepin | chemical drug | | 1,12E-02 | SERPINE1 |
| sibutramine | chemical drug | | 1,12E-02 | SERPINE1 |
| imidapril | chemical drug | | 1,12E-02 | AGT |
| neratinib | chemical drug | | 1,12E-02 | CCND1 |
| butyrine | chemical - endogenous mammalian | | 1,12E-02 | MYC |
| chlordiazepoxide | chemical drug | | 1,12E-02 | FOS |
| GYKI 52466 | chemical drug | | 1,12E-02 | FOS |
| dibenzo(a,l)pyrene | chemical toxicant | | 1,12E-02 | CYP1A1 |
| chalcone-24 | chemical reagent | | 1,12E-02 | BIRC3 |
| (S)-2-((6Z,15Z)-20- hydroxyicosa-6,15-dienamido)succinate | chemical reagent | | 1,12E-02 | AGT |
| benzo(a)pyrene-7,8-dione | chemical toxicant | | 1,12E-02 | CYP1A1 |
| butylbenzyl phthalate | chemical toxicant | | 1,12E-02 | MYC |
| nisoldipine | chemical drug | | 1,12E-02 | FOS |
| AGI-1095 | chemical reagent | | 1,12E-02 | EGR1 |
| lavendustin A | chemical - kinase inhibitor | | 1,12E-02 | CYP1A1 |
| sodium fluoride | chemical drug | | 1,12E-02 | ICAM1 |
| EWSR1-WT1 | fusion gene/product | | 1,12E-02 | EGR1 |
| procaterol | chemical drug | | 1,12E-02 | ICAM1 |
| aprotinin | biologic drug | | 1,12E-02 | PLAU |
| enalaprilat | chemical - endogenous mammalian | | 1,12E-02 | LDLR |
| ropinirole | chemical drug | | 1,12E-02 | EGR1 |
| indolo(3,2-b)carbazole | chemical reagent | | 1,12E-02 | CYP1A1 |
| tegaserod | chemical drug | | 1,12E-02 | FOS |
| kenpaullone | chemical reagent | | 1,12E-02 | TNFRSF10B |
| veratridine | chemical toxicant | | 1,12E-02 | FOS |
| teleocidins | chemical toxicant | | 1,12E-02 | FOS |
| tyrphostin B56 | chemical - kinase inhibitor | | 1,12E-02 | ICAM1 |
| 5-oxo-6-8-11-14-(e,z,z,z)-eicosatetraenoic acid | chemical - endogenous mammalian | | 1,12E-02 | MYC |
| cyclosporin H | chemical reagent | | 1,12E-02 | EGR1 |
| 5-thio-D-glucose | chemical reagent | | 1,12E-02 | FOS |
| 3-O-methylglucose | chemical reagent | | 1,12E-02 | TXNIP |
| tibolone | chemical drug | | 1,12E-02 | SERPINE1 |
| flunisolide | chemical drug | | 1,12E-02 | ICAM1 |
| digitoxin | chemical drug | | 1,12E-02 | TNFRSF10B |
| 1-phenazinecarboxylic acid | chemical - endogenous non-mammalian | | 1,12E-02 | ICAM1 |
| miR-493-5p (miRNAs w/seed UGUACAU) | mature microRNA | Inhibited | 1,13E-02 | CTSV,HIVEP2,KRT6A,LCA5,NFKBIA,TSC22D2,ZNF441,ZNF627 |
| miR-6887-3p (miRNAs w/seed CCCCUCC) | mature microRNA | Inhibited | 1,13E-02 | CSRNP1,EMP1,ERRFI1,ETS1,GJB1,H3C14,H3C15,JUN,JUNB,KLF6,LDLR,MAFF,PALD1,PHLDA1,PLAU,PRR15L,RHOB,TNFAIP3 |
| miR-6815-5p (and other miRNAs w/seed AGGUGGC) | mature microRNA | Inhibited | 1,14E-02 | BCL10,CXCL2,GJB1,H3C14,H3C15,IGFBP4,KLF6,NFKBIZ,NUAK2,REEP6,SERINC5,TNFAIP3,YOD1,ZNF844 |
| taurocholic acid | chemical - endogenous mammalian | | 1,15E-02 | ATF3,EGR1 |
| crizotinib | chemical drug | | 1,15E-02 | SERPINE1,VIM |
| SH3TC2 | other | | 1,15E-02 | EGR1,GJB1 |
| ACTL6A | other | | 1,15E-02 | CCND1,MYC |
| MEL S3 | chemical - kinase inhibitor | | 1,15E-02 | EGR1,VIM |
| CFLAR | other | | 1,15E-02 | FOS,TNFRSF10B |
| ketoconazole | chemical drug | | 1,15E-02 | CYP1A1,GSTA2 |
| letrozole | chemical drug | | 1,15E-02 | CCND1,MYC |
| hemozoin | chemical - endogenous non-mammalian | | 1,15E-02 | CXCL2,ICAM1 |
| succinic acid | chemical - endogenous mammalian | | 1,15E-02 | AGT,IGFBP4 |
| miR-6794-3p (miRNAs w/seed UCACUCU) | mature microRNA | | 1,15E-02 | CYP1A1,KLF6,NDRG1,PLK2,PRR15L,SMIM14,ZBTB43,ZNF441 |
| miR-6516-3p (miRNAs w/seed UCAUGUA) | mature microRNA | Inhibited | 1,15E-02 | ARRDC3,H2BC5,PARD6B,SMIM14,TNFRSF10B,ZBTB20,ZNF441,ZNF555,ZNF627,ZNF844 |
| miR-510-3p (miRNAs w/seed UUGAAAC) | mature microRNA | | 1,16E-02 | MYC,OAT,SLC25A25,TSC22D2,ZNF627 |
| miR-512-5p (miRNAs w/seed ACUCAGC) | mature microRNA | | 1,19E-02 | ABL2,AKR1C3,BHLHE40,ETS1,MYC,NDRG1,NFKBIZ,SMIM14 |
| miR-6732-3p (miRNAs w/seed AACCCUG) | mature microRNA | Inhibited | 1,20E-02 | ABL2,ANKRD1,ATF3,CCND1,LCA5,MDM2,OAT,SLC25A25 |
| ALKBH5 | enzyme | | 1,22E-02 | AXL,LAMA1,RGS2 |
| 3,4,5,3',4'-pentachlorobiphenyl | chemical toxicant | | 1,22E-02 | CYP1A1,JUN,TIPARP |
| AICAR | chemical - endogenous mammalian | | 1,22E-02 | CCND1,MDM2,SERPINE1 |
| indican | chemical - endogenous mammalian | | 1,22E-02 | AGT,ICAM1 |
| tosedostat | chemical drug | | 1,22E-02 | ATF3,PPP1R15A |
| CRTC2 | other | | 1,22E-02 | CCND1,MYC |
| RPSA | translation regulator | | 1,22E-02 | DUSP1,ITGA6 |
| SIAH2 | transcription regulator | | 1,22E-02 | AKR1C3,NDRG1 |
| PRKCI | kinase | | 1,22E-02 | CCND1,CXCL2 |
| GNAI2 | enzyme | | 1,22E-02 | BHLHE40,CCND1 |
| LTB4R | G-protein coupled receptor | | 1,22E-02 | CXCL2,LDLR |
| oltipraz | chemical drug | | 1,22E-02 | AGT,CYP1A1 |
| trabectedin | chemical drug | | 1,22E-02 | CCND1,OAT |
| R-WIN 55,212 | chemical reagent | | 1,22E-02 | CCND1,ICAM1 |
| miR-3192-5p (miRNAs w/seed CUGGGAG) | mature microRNA | Inhibited | 1,23E-02 | AFAP1,AGT,ATF3,ETS1,MXD1,NUAK2,PMM1,POLR2J2/POLR2J3,REEP6,RHOB,TNS4,VGF,YOD1,ZNF627 |
| miR-6789-5p (miRNAs w/seed UAGGGGC) | mature microRNA | Inhibited | 1,25E-02 | CYP1A1,H3C14,H3C15,IER3,POLR2J2/POLR2J3,SERINC5,UBASH3B,ZBTB43 |
| maslinic acid | chemical - endogenous non-mammalian | | 1,25E-02 | CCND1,ETS1,JUNB |
| miR-3908 (miRNAs w/seed AGCAAUG) | mature microRNA | | 1,26E-02 | CTSV,KLF10,LAMA1,MXD4,TIPARP |
| miR-3691-3p (miRNAs w/seed CCAAGUC) | mature microRNA | | 1,26E-02 | ETS1,FABP1,POLR2J2/POLR2J3,RHOB,SEPHS2,SPRED1,UBASH3B,ZBTB20 |
| 10E,12Z-octadecadienoic acid | chemical - endogenous mammalian | | 1,26E-02 | ATF3,LDLR,PLK2,PPP1R15A |
| miR-6748-3p (miRNAs w/seed CCUGUCC) | mature microRNA | Inhibited | 1,28E-02 | ABL2,ANKRD1,BIRC3,CCND1,EGR1,H3C14,H3C15,MAFF,PALD1,SERINC5,SLC25A25 |
| prednisolone | chemical drug | | 1,28E-02 | BIRC3,CYP1A1,DUSP1,TNFAIP3,TXNIP |
| miR-5571-5p (miRNAs w/seed AAUUCUC) | mature microRNA | | 1,28E-02 | BIRC3,FABP1,H3C14,H3C15,MDM2 |
| FEV | transcription regulator | | 1,29E-02 | FOS,ICAM1,VIM |
| NR2E1 | ligand-dependent nuclear receptor | | 1,30E-02 | CCND1,MYC |
| FECH | enzyme | | 1,30E-02 | CCND1,FABP1 |
| UPF2 | other | | 1,30E-02 | TNFAIP3,TSPAN8 |
| SRSF1 | other | | 1,30E-02 | KLF6,VIM |
| PRKAR1A | kinase | | 1,30E-02 | CCND1,VIM |
| MECOM | transcription regulator | | 1,30E-02 | ITGA6,SERPINE1 |
| KNG1 | other | | 1,30E-02 | CCND1,FOS |
| DRD2 | G-protein coupled receptor | | 1,30E-02 | FOS,JUN |
| VAV2 | transcription regulator | | 1,30E-02 | FOS,VIM |
| Hmgn3 | other | | 1,30E-02 | AHSG,TTR |
| clodronic acid | chemical drug | | 1,30E-02 | FOS,ICAM1 |
| 4-tert-octylphenol | chemical toxicant | | 1,30E-02 | EMP1,MYC |
| butylated hydroxyanisol | chemical toxicant | | 1,30E-02 | FOS,JUN |
| miR-3146 (miRNAs w/seed AUGCUAG) | mature microRNA | | 1,31E-02 | ETS1,GEM,ITGA6,NDRG1,TENT5A,TSC22D2,YOD1 |
| miR-6840-5p (miRNAs w/seed CCCCCGG) | mature microRNA | Inhibited | 1,33E-02 | H3C14,H3C15,IER2,MAFF,PER1,PRR15L,RHOB,TNS4,VGF |
| NPC1 | transporter | | 1,33E-02 | CCND1,EGR1,LDLR |
| miR-544-3p (and other miRNAs w/seed UUCUGCA) | mature microRNA | Inhibited | 1,35E-02 | ANKRD1,ATF3,DYRK1A,EPHX2,KLF6,KRT6A,PLK2,SPRED1,TENT5A,TSC22D2,ZC3H12A |
| miR-582-5p (miRNAs w/seed UACAGUU) | mature microRNA | Inhibited | 1,35E-02 | ANKRD1,BCL10,CXCL2,ITGA2,ITGA6,PARD6B,YOD1,ZNF627 |
| miR-4648 (miRNAs w/seed GUGGGAC) | mature microRNA | | 1,37E-02 | CCND1,ITGA6,KRT6A,MXD1,PI3,RHOB,SERINC5,SERPINA6,SPRY4,ZNF844 |
| LCN2 | transporter | | 1,37E-02 | CXCL2,LDLR,VIM |
| miR-552-5p (miRNAs w/seed UUUAACC) | mature microRNA | | 1,37E-02 | ERRFI1,H3C14,H3C15 |
| niacinamide | chemical - endogenous mammalian | | 1,37E-02 | ICAM1,JUNB,MYC |
| deferasirox | chemical drug | | 1,38E-02 | FOS,JUN |
| TIRAP | other | | 1,38E-02 | CXCL2,ICAM1 |
| TNFSF14 | cytokine | | 1,38E-02 | BIRC3,ICAM1 |
| PTH1R | G-protein coupled receptor | | 1,38E-02 | CCND1,DUSP1 |
| NF2 | other | | 1,38E-02 | CCND1,MYC |
| SOX10 | transcription regulator | | 1,38E-02 | FOS,ITGA6 |
| SMAD5 | transcription regulator | | 1,38E-02 | ICAM1,SERPINE1 |
| TNFSF15 | cytokine | | 1,38E-02 | BIRC3,CXCL2 |
| linsidomine | chemical drug | | 1,38E-02 | EGR1,MYC |
| betulinic acid | chemical drug | | 1,38E-02 | CCND1,TNFRSF10B |
| miR-4634 (miRNAs w/seed GGCGCGA) | mature microRNA | | 1,39E-02 | ABL2,H3C14,H3C15,ITGA2,REEP6,ZNF844 |
| miR-17-5p (and other miRNAs w/seed AAAGUGC) | mature microRNA | Inhibited | 1,40E-02 | ABL2,CCND1,KLF10,LDLR,PARD6B,SMIM14,TNFRSF10B,TXNIP,VIM,YOD1,ZBTB43 |
| miR-6798-5p (miRNAs w/seed CAGGGGG) | mature microRNA | Inhibited | 1,41E-02 | AFAP1,CCND1,EPHA2,FABP1,H3C14,H3C15,IER3,JUNB,MXD1,NDRG1,NR1D1,PRR15L,RHOB,SPRY4,TNS4,ZFP36 |
| ZFHX3 | transcription regulator | | 1,42E-02 | AFP,CCND1,RGS2 |
| 4-phenylbutyric acid | chemical - endogenous mammalian | | 1,42E-02 | AHSG,CCND1,MXD1 |
| hemin | chemical - endogenous mammalian | | 1,42E-02 | CCND1,HDAC9,JUN |
| miR-6853-5p (miRNAs w/seed GCGUGGG) | mature microRNA | | 1,44E-02 | AHSG,AXL,ICAM1,PALD1,POLR2J2/POLR2J3,REEP6,TNFRSF10B,TNS4,ZNF844 |
| SREBF1 | transcription regulator | | 1,45E-02 | BHLHE40,LDLR,OAT,SERPINE1,VGF |
| miR-4700-3p (miRNAs w/seed ACAGGAC) | mature microRNA | | 1,45E-02 | AKR1C3,DCBLD2,EPHX2,IGFBP4,KLF10,KLF6,OAT,PLAU,TNFRSF10B |
| SAMSN1 | other | | 1,46E-02 | CXCL2,NFKBIZ,ZC3H12A |
| nocodazole | chemical reagent | | 1,46E-02 | CCND1,FOS,MDM2 |
| WNT4 | cytokine | | 1,47E-02 | CCND1,PARD6B |
| MARK2 | kinase | | 1,47E-02 | CXCL2,NFKBIZ |
| AHCY | enzyme | | 1,47E-02 | AFP,ICAM1 |
| GDF11 | growth factor | | 1,47E-02 | FOS,ICAM1 |
| vitamin A | chemical - endogenous mammalian | | 1,47E-02 | CYP1A1,OAT |
| eplerenone | chemical drug | | 1,47E-02 | ICAM1,SERPINE1 |
| 6-hydroxydopamine | chemical toxicant | | 1,50E-02 | FOS,JUN,MDM2 |
| PD 0325901 | chemical drug | | 1,55E-02 | MYC,TBX3 |
| oleanolic acid | chemical - endogenous non-mammalian | | 1,55E-02 | CCND1,ITGA6 |
| RAB1B | other | | 1,55E-02 | GEM,JUN |
| OSCAR | other | | 1,55E-02 | CXCL2,ICAM1 |
| ABCB4 | transporter | | 1,55E-02 | AFP,SERPINE1 |
| SKP2 | other | | 1,55E-02 | CCND1,VIM |
| GAB2 | other | | 1,55E-02 | FOS,SERPINE1 |
| EZR | other | | 1,55E-02 | ATF3,MYC |
| mir-126 | microRNA | | 1,55E-02 | PLK2,SPRED1 |
| PAK1 | kinase | | 1,55E-02 | CCND1,MYC |
| EWSR1 | other | | 1,55E-02 | CCND1,FOS |
| MEIS1 | transcription regulator | | 1,55E-02 | FOS,TSC22D2 |
| daunorubicin | chemical drug | | 1,55E-02 | MDM2,MYC |
| bardoxolone | chemical drug | | 1,55E-02 | CCND1,NFKBIA |
| miR-4768-5p (and other miRNAs w/seed UUCUCUC) | mature microRNA | Inhibited | 1,57E-02 | ATF3,CCND1,JUN,MAFF,SMIM14,TBX3,TENT5A,ZNF441,ZNF627,ZNF844 |
| miR-6749-3p (miRNAs w/seed UCCUCCC) | mature microRNA | Inhibited | 1,57E-02 | ATF3,BIRC3,CCND1,EMP1,ERRFI1,FABP1,FOS,GJB1,IER3,PALD1,PLK2,RHOB,SLC25A25,SPRED1,SPRY4,ZC3H12A |
| miR-1252-5p (miRNAs w/seed GAAGGAA) | mature microRNA | | 1,57E-02 | BHLHE40,GJB1,IGFBP4,ITGA2,MYC,OAT,PARD6B,PI3,PRR15L,SPRY4,TBX3,ZNF627 |
| miR-936 (miRNAs w/seed CAGUAGA) | mature microRNA | Inhibited | 1,59E-02 | ABL2,AFAP1,ANKRD1,EGR1,ICAM1,SERINC5,SPRED1,ZBTB43,ZNF441 |
| miR-6740-3p (miRNAs w/seed GUCUUCU) | mature microRNA | | 1,59E-02 | AFP,CCND1,DUSP1,H2BC5,PHLDA1,PRR15L,SERPINE1,SPRY4,ZNF627 |
| LMNA | other | | 1,59E-02 | AFP,JUN,MAFF,MXD4,ZFP36 |
| PDLIM2 | other | | 1,59E-02 | CCND1,PHLDA1,TXNIP |
| miR-224-5p (miRNAs w/seed AAGUCAC) | mature microRNA | | 1,59E-02 | AHSG,ATF3,GEM,KLF10,MAFF,TIPARP,ZBTB43 |
| miR-3194-3p (miRNAs w/seed GCUCUGC) | mature microRNA | Inhibited | 1,59E-02 | ANKRD1,ARRDC3,ASB2,ATF3,ETS1,H3C14,H3C15,IGFBP4,MXD1,SEPHS2,SPRED1 |
| miR-31-3p (and other miRNAs w/seed GCUAUGC) | mature microRNA | | 1,62E-02 | ARRDC3,BHLHE40,KLF10,KLF6,MXD4,TENT5A |
| miR-490-5p (miRNAs w/seed CAUGGAU) | mature microRNA | Inhibited | 1,63E-02 | ETS1,FOS,KRT6A,RGS2,SLC25A25,TNFRSF10B,ZNF627 |
| DSCAM | other | | 1,64E-02 | MYC,VGF,VIM |
| NLRP3 | other | | 1,64E-02 | CXCL2,MDM2,VIM |
| GW9662 | chemical reagent | | 1,64E-02 | FABP1,FOS,NR1D1 |
| GSTP1 | enzyme | | 1,64E-02 | ITGA2,JUN,PER1,SERPINE1 |
| resolvin D1 | chemical - endogenous mammalian | | 1,64E-02 | CXCL2,JUN |
| TERC | other | | 1,64E-02 | EGR1,NR1D1 |
| CYBB | enzyme | | 1,64E-02 | DUSP1,ICAM1 |
| IL36A | cytokine | | 1,64E-02 | CXCL2,NFKBIZ |
| IL17F | cytokine | | 1,64E-02 | CXCL2,ICAM1 |
| bafilomycin A1 | chemical drug | | 1,64E-02 | NFKBIA,RHOB |
| methylnitrosourea | chemical toxicant | | 1,64E-02 | EGR1,JUN |
| FUS-DDIT3 | fusion gene/product | | 1,64E-02 | SERPINE1,VIM |
| andrographolide | chemical drug | | 1,64E-02 | CYP1A1,ICAM1 |
| naringenin | chemical - endogenous non-mammalian | | 1,64E-02 | CYP1A1,LDLR |
| miR-509-5p (and other miRNAs w/seed ACUCCAG) | mature microRNA | | 1,64E-02 | AKAP12,EMP1,NUAK2,REEP6,SLC25A25,ZNF627,ZNF844 |
| miR-583 (miRNAs w/seed AAAGAGG) | mature microRNA | | 1,64E-02 | AFP,ATF3,DCBLD2,NDRG1,PRR15L,SMIM14,YOD1 |
| miR-6757-3p (miRNAs w/seed ACACUGG) | mature microRNA | | 1,67E-02 | ABL2,AGT,ARRDC3,DUSP1,GEM,SMIM14,UBASH3B,ZNF608,ZNF627 |

| | | | | |
|---|---|---|---|---|
| docosahexaenoic acid | chemical drug | | 1,67E-02 | FOS,LDLR,MYC,SERPINE1 |
| Rp-8-Br-cGMPS | chemical - kinase inhibitor | | 1,67E-02 | FOS |
| sangivamycin | chemical - endogenous non-mammalian | | 1,67E-02 | JUN |
| thioguanine | chemical drug | | 1,67E-02 | MYC |
| glycodeoxycholic acid | chemical - endogenous mammalian | | 1,67E-02 | TNFRSF10B |
| pregnenolone sulfate | chemical - endogenous mammalian | | 1,67E-02 | EGR1 |
| IGF receptor | group | | 1,67E-02 | ICAM1 |
| 1-palmitoyl-2-arachidonoyl-phosphatidylcholine | chemical - endogenous mammalian | | 1,67E-02 | CCND1 |
| AMOT | other | | 1,67E-02 | ANKRD1 |
| ANTXR2 | transmembrane receptor | | 1,67E-02 | VIM |
| benzo(a)pyrene 7,8-dihydrodiol | chemical toxicant | | 1,67E-02 | CYP1A1 |
| 14,15-DHET | chemical - endogenous mammalian | | 1,67E-02 | EPHX2 |
| DUB | group | | 1,67E-02 | LDLR |
| myosin-light-chain kinase | group | | 1,67E-02 | CCND1 |
| FKHR | group | | 1,67E-02 | CCND1 |
| lipoxygenase | group | | 1,67E-02 | RGS2 |
| PLC gamma | group | | 1,67E-02 | MYC |
| Stat1-Stat3 | complex | | 1,67E-02 | FOS |
| 1,2,5,6-dibenzanthracene | chemical toxicant | | 1,67E-02 | CYP1A1 |
| diosmin | chemical drug | | 1,67E-02 | CYP1A1 |
| Esr1-Estrogen-Sp1 | complex | | 1,67E-02 | FOS |
| sethoxydim | chemical toxicant | | 1,67E-02 | MYC |
| cetylpyridinium | chemical drug | | 1,67E-02 | FOS |
| Corticosteroid-GCR | complex | | 1,67E-02 | AGT |
| prochloraz | chemical reagent | | 1,67E-02 | CYP1A1 |
| INTS11 | other | | 1,67E-02 | EGR1 |
| NSD3 | enzyme | | 1,67E-02 | CCND1 |
| Jun-ATF2 | complex | | 1,67E-02 | CCND1 |
| LZTS2 | other | | 1,67E-02 | MYC |
| RFFL | enzyme | | 1,67E-02 | MDM2 |
| PUM1 | other | | 1,67E-02 | LAMA1 |
| NOP58 | enzyme | | 1,67E-02 | CCND1 |
| CHD5 | enzyme | | 1,67E-02 | VIM |
| SPRED2 | cytokine | | 1,67E-02 | VIM |
| H1-3 | other | | 1,67E-02 | MYC |
| ZNF451 | enzyme | | 1,67E-02 | SERPINE1 |
| PRR7 | other | | 1,67E-02 | JUN |
| CNOT1 | other | | 1,67E-02 | MYC |
| GPC6 | transmembrane receptor | | 1,67E-02 | CCND1 |
| NDUFAF3 | other | | 1,67E-02 | CCND1 |
| SENP8 | peptidase | | 1,67E-02 | MDM2 |
| MYLIP | enzyme | | 1,67E-02 | LDLR |
| C1QL1 | other | | 1,67E-02 | FOS |
| PSMD14 | peptidase | | 1,67E-02 | CCND1 |
| ZNF300 | transcription regulator | | 1,67E-02 | MYC |
| Ribosomal Subunit 80S | complex | | 1,67E-02 | EGR1 |
| PVT1 | other | | 1,67E-02 | MYC |
| NAA30 | enzyme | | 1,67E-02 | TNFRSF10B |
| HDGF | growth factor | | 1,67E-02 | SERPINE1 |
| AMOTL1 | other | | 1,67E-02 | ANKRD1 |
| ASH1L | transcription regulator | | 1,67E-02 | TNFAIP3 |
| PTPN23 | phosphatase | | 1,67E-02 | VIM |
| OLA1 | enzyme | | 1,67E-02 | CCND1 |
| Trk Receptor | group | | 1,67E-02 | DUSP6 |
| EIF4F | complex | | 1,67E-02 | MYC |
| UBE2D3 | enzyme | | 1,67E-02 | CCND1 |
| ATP1A2 | transporter | | 1,67E-02 | FOS |
| defibrotide | biologic drug | | 1,67E-02 | SERPINE1 |
| Gap | group | | 1,67E-02 | FOS |
| SERTAD1 | transcription regulator | | 1,67E-02 | CCND1 |
| RRAD | enzyme | | 1,67E-02 | CCND1 |
| MAT2B | enzyme | | 1,67E-02 | CCND1 |
| EMP2 | other | | 1,67E-02 | ITGA6 |
| PURB | transcription regulator | | 1,67E-02 | MYC |
| YARS1 | enzyme | | 1,67E-02 | NFKBIA |
| SPTBN1 | other | | 1,67E-02 | FOS |
| AGAP2 | enzyme | | 1,67E-02 | CCND1 |
| U2AF2 | other | | 1,67E-02 | CCND1 |
| BPTF | transcription regulator | | 1,67E-02 | MYC |
| riociguat | chemical drug | | 1,67E-02 | SERPINE1 |
| mir-497 | microRNA | | 1,67E-02 | CCND1 |
| mir-383 | microRNA | | 1,67E-02 | MYC |
| MAPK15 | kinase | | 1,67E-02 | JUN |
| STRN | other | | 1,67E-02 | VIM |
| SLC1A1 | transporter | | 1,67E-02 | FOS |
| RPS14 | translation regulator | | 1,67E-02 | MYC |
| PBK | kinase | | 1,67E-02 | BIRC3 |
| PRDX4 | enzyme | | 1,67E-02 | ICAM1 |
| apitolisib | chemical drug | | 1,67E-02 | NDRG1 |
| HSPB3 | other | | 1,67E-02 | ICAM1 |
| PUM2 | other | | 1,67E-02 | LAMA1 |
| KLF16 | transcription regulator | | 1,67E-02 | CYP1A1 |
| aurora kinase inhibitor III | chemical - kinase inhibitor | | 1,67E-02 | FOS |
| FOXO6 | transcription regulator | | 1,67E-02 | MYC |
| RALGDS | other | | 1,67E-02 | CCND1 |
| NCOA5 | other | | 1,67E-02 | MYC |
| SCN1B | ion channel | | 1,67E-02 | FOS |
| DOK2 | other | | 1,67E-02 | MYC |
| MGAT4A | enzyme | | 1,67E-02 | CCND1 |
| SLC3A2 | transporter | | 1,67E-02 | ANKRD1 |
| EIF2B1 | translation regulator | | 1,67E-02 | CCND1 |
| STK16 | kinase | | 1,67E-02 | SERPINE1 |
| MED26 | transcription regulator | | 1,67E-02 | MYC |
| sulfurbutylether-beta-cyclodextrin | chemical reagent | | 1,67E-02 | LDLR |
| PTTG1IP | other | | 1,67E-02 | CCND1 |
| DLST | enzyme | | 1,67E-02 | JUN |
| GFRA2 | transmembrane receptor | | 1,67E-02 | EGR1 |
| SRPK2 | kinase | | 1,67E-02 | CCND1 |
| ADCY6 | enzyme | | 1,67E-02 | ATF3 |
| GCNT1 | enzyme | | 1,67E-02 | FOS |
| RGS3 | enzyme | | 1,67E-02 | MYC |
| SIGLEC10 | other | | 1,67E-02 | ETS1 |
| LSS | enzyme | | 1,67E-02 | LDLR |
| KPNB1 | transporter | | 1,67E-02 | TNFRSF10B |
| SLC12A6 | transporter | | 1,67E-02 | VIM |
| KRT5 | other | | 1,67E-02 | ICAM1 |
| CTAG1A/CTAG1B | other | | 1,67E-02 | CCND1 |
| RASSF7 | other | | 1,67E-02 | CCND1 |
| ELOA | transcription regulator | | 1,67E-02 | ATF3 |
| CDK5R2 | other | | 1,67E-02 | FOS |
| BTG1 | transcription regulator | | 1,67E-02 | JUN |
| INHBC | growth factor | | 1,67E-02 | JUNB |
| STAM2 | other | | 1,67E-02 | MYC |
| PES1 | other | | 1,67E-02 | CCND1 |
| RBBP8 | enzyme | | 1,67E-02 | CCND1 |
| CNPY2 | other | | 1,67E-02 | LDLR |
| ERC1 | other | | 1,67E-02 | NFKBIA |
| IL20RA | transmembrane receptor | | 1,67E-02 | ICAM1 |
| ARHGEF11 | other | | 1,67E-02 | FOS |
| Miat | other | | 1,67E-02 | ICAM1 |
| 8-butyl-10-[(3-methoxy-5-pyrrol-2-ylidenepyrrol-2-ylidene)methyl]-11-azabicyclo[7.2.1]dodeca-1(12),9-diene | chemical reagent | | 1,67E-02 | TNFRSF10B |
| EEF1A1 | translation regulator | | 1,67E-02 | MYC |
| Hspa1b | other | | 1,67E-02 | FOS |
| Irs4 | other | | 1,67E-02 | EGR1 |
| RPL23A | other | | 1,67E-02 | MYC |
| H1-1 | other | | 1,67E-02 | FOS |
| TIGAR | enzyme | | 1,67E-02 | TNFAIP3 |
| RDH16 | enzyme | | 1,67E-02 | CCND1 |
| NOLC1 | transcription regulator | | 1,67E-02 | MDM2 |
| (+)-R-3-(4-chlorophenyl)-3-(1-hydroxymethylcyclopropylmethoxy)-2-(4-nitrobenzyl)-2,3-dihydroisoindol-1-one | chemical reagent | | 1,67E-02 | MDM2 |
| BCKDHA | enzyme | | 1,67E-02 | MYC |
| capivasertib | chemical drug | | 1,67E-02 | VIM |
| umbralisib | chemical drug | | 1,67E-02 | MYC |
| mir-297 | microRNA | | 1,67E-02 | JUNB |
| singlet oxygen | chemical - endogenous mammalian | | 1,67E-02 | ICAM1 |
| compound 48/80 | chemical reagent | | 1,67E-02 | LDLR |
| nordihydrocapsiate | chemical reagent | | 1,67E-02 | ICAM1 |
| nitrous oxide | chemical drug | | 1,67E-02 | FOS |
| mecamylamine | chemical drug | | 1,67E-02 | FOS |
| amantadine | chemical drug | | 1,67E-02 | FOS |
| prinaberel | chemical drug | | 1,67E-02 | CXCL2 |
| filipin | chemical - endogenous non-mammalian | | 1,67E-02 | SERPINE1 |
| 2,2-dimethylbutyric acid | chemical drug | | 1,67E-02 | MYC |
| p-chloroamphetamine | chemical drug | | 1,67E-02 | FOS |
| beta-lapachone | chemical drug | | 1,67E-02 | TNFRSF10B |
| oxotremorine | chemical drug | | 1,67E-02 | FOS |
| albendazole | chemical drug | | 1,67E-02 | ICAM1 |
| ethyl ether | chemical reagent | | 1,67E-02 | FOS |
| rocuronium | chemical drug | | 1,67E-02 | CCND1 |
| cycloserine | chemical drug | | 1,67E-02 | FOS |
| minodronate | chemical drug | | 1,67E-02 | ICAM1 |
| oenothein B | chemical - endogenous non-mammalian | | 1,67E-02 | CXCL2 |
| devazepide | chemical drug | | 1,67E-02 | FOS |
| tamsulosin | chemical drug | | 1,67E-02 | FOS |
| modafinil | chemical drug | | 1,67E-02 | FOS |
| 3-nitrobenzanthrone | chemical toxicant | | 1,67E-02 | CYP1A1 |
| betamethasone valerate | chemical drug | | 1,67E-02 | CCND1 |
| salsalate | chemical drug | | 1,67E-02 | NFKBIA |
| ethyl gallate | chemical - endogenous non-mammalian | | 1,67E-02 | ICAM1 |
| taprostene | chemical reagent | | 1,67E-02 | DUSP1 |
| MLN-4760 | chemical - protease inhibitor | | 1,67E-02 | CXCL2 |
| phloretin | chemical - endogenous non-mammalian | | 1,67E-02 | TXNIP |
| TTI-101 | chemical drug | | 1,67E-02 | SERPINE1 |
| mycophenolate mofetil | chemical drug | | 1,67E-02 | ICAM1 |
| lanthanum chloride | chemical reagent | | 1,67E-02 | AXL |
| nickel subsulfide | chemical toxicant | | 1,67E-02 | NDRG1 |
| sodium iodide | chemical drug | | 1,67E-02 | ICAM1 |
| 5,10,15,20-tetrakis(4-sulfonatophenyl)porphyrinato iron (III) | chemical reagent | | 1,67E-02 | ICAM1 |
| senexin B | chemical reagent | | 1,67E-02 | CXCL2 |
| C66 | chemical reagent | | 1,67E-02 | ICAM1 |
| 3,4-methylenedioxyamphetamine | chemical drug | | 1,67E-02 | JUN |
| actinomycin | chemical - endogenous non-mammalian | | 1,67E-02 | IGFBP4 |
| glutathione diethyl ester | chemical reagent | | 1,67E-02 | PLAU |
| ODN-BW006 | chemical reagent | | 1,67E-02 | ICAM1 |
| GNE-317 | chemical - kinase inhibitor | | 1,67E-02 | NDRG1 |
| R191 | chemical - kinase inhibitor | | 1,67E-02 | MYC |
| (indol-3-yl)pyruvic acid | chemical - endogenous non-mammalian | | 1,67E-02 | CYP1A1 |
| R-etodolac | chemical drug | | 1,67E-02 | CCND1 |
| muscarine | chemical toxicant | | 1,67E-02 | EGR1 |
| 3,7-dimethyl-1-propargylxanthine | chemical reagent | | 1,67E-02 | FOS |
| AVI-4126 | biologic drug | | 1,67E-02 | MYC |
| mizoribine | chemical drug | | 1,67E-02 | CCND1 |
| D,L-propargylglycine | chemical reagent | | 1,67E-02 | AGT |
| CH6953755 | chemical - kinase inhibitor | | 1,67E-02 | ANKRD1 |
| raffinose | chemical - endogenous mammalian | | 1,67E-02 | ICAM1 |
| prostratin | chemical - endogenous non-mammalian | | 1,67E-02 | ICAM1 |
| oleandrin | chemical drug | | 1,67E-02 | TNFRSF10B |
| ponasterone A | chemical reagent | | 1,67E-02 | CCND1 |
| Mn2+ | chemical - endogenous mammalian | | 1,67E-02 | ICAM1 |
| EHF | transcription regulator | | 1,68E-02 | CYP1A1,SERPINE1,TNFRSF10B |
| miR-3118 (and other miRNAs w/seed GUGACUG) | mature microRNA | | 1,70E-02 | ALDH1A1,ATF3,B4GALNT2,H2BC5,PHLDA2,SERPINE1,TSC22D2,ZNF627,ZNF844 |
| miR-6809-3p (miRNAs w/seed UUCUCUU) | mature microRNA | | 1,70E-02 | ATF3,DUSP6,FABP1,MDM2,SMIM14,ZNF441,ZNF555,ZNF627,ZNF844 |
| miR-6069 (miRNAs w/seed GGCUAGG) | mature microRNA | | 1,70E-02 | ANKRD1,BCL10,CYP1A1,GSTA2,KLF6,TENT5A,ZBTB43 |
| miR-5008-3p (and other miRNAs w/seed CUGUGCU) | mature microRNA | | 1,71E-02 | AHSG,ANKRD1,ERRFI1,H3C14,H3C15,IGFBP4,INAFM1,KLF10,SPRED1,ZNF441 |
| miR-542-3p (miRNAs w/seed GUGACAG) | mature microRNA | | 1,71E-02 | ABL2,DCBLD2,ERRFI1,ETS1,PMM1,SMIM14,SPRED1,TNFRSF10B,YOD1 |
| Ap2 | group | | 1,73E-02 | AFP,ICAM1 |
| 8-bromoguanosine 3',5'-cyclic monophosphate | chemical - kinase inhibitor | | 1,73E-02 | FOS,JUNB |
| PRDM16 | transcription regulator | | 1,73E-02 | AGT,SERPINE1 |
| CASP1 | peptidase | | 1,73E-02 | CXCL2,VIM |
| TFDP1 | transcription regulator | | 1,73E-02 | CCND1,MYC |
| IGFBP3 | other | | 1,73E-02 | ICAM1,SERPINE1 |
| GTF2B | transcription regulator | | 1,73E-02 | NFKBIA,TNFAIP3 |
| mercuric chloride | chemical toxicant | | 1,73E-02 | JUN,JUNB |
| CX3CR1 | G-protein coupled receptor | | 1,73E-02 | EGR1,EMP1,FOS,ICAM1 |
| miR-1257 (miRNAs w/seed GUGAAUG) | mature microRNA | Inhibited | 1,76E-02 | DCBLD2,H3C14,H3C15,KLF10,MDM2,NFKBIZ,TIPARP,ZNF844 |
| SMAD2 | transcription regulator | | 1,78E-02 | AFP,SERPINE1,VIM |
| miR-193a-3p (and other miRNAs w/seed ACUGGCC) | mature microRNA | | 1,80E-02 | CCND1,CXCL2,ETS1,MDM2,PHLDA2,PLAU,POLR2J2/POLR2J3,PRR15L,SLC39A5,SMIM14,ZNF608 |
| miR-4755-5p (and other miRNAs w/seed UUCCCUU) | mature microRNA | | 1,80E-02 | ARRDC3,B4GALNT2,CYP1A1,EGR1,EMP1,SMIM14,TNFAIP3,TSPAN8,TXNIP |
| miR-4668-3p (miRNAs w/seed AAAAUCC) | mature microRNA | | 1,82E-02 | BCL10,GJB1,SLC39A5 |
| CpG ODN 2006 | chemical reagent | | 1,82E-02 | BIRC3,ICAM1,NFKBIZ |
| PIAS1 | transcription regulator | | 1,83E-02 | CCND1,MDM2 |
| RAD21 | transcription regulator | | 1,83E-02 | MYC,VIM |
| alvocidib | chemical drug | | 1,83E-02 | BIRC3,CCND1 |
| miR-891a-5p (miRNAs w/seed GCAACGA) | mature microRNA | Inhibited | 1,84E-02 | BHLHE40,CYLD,DYRK1A,PARD6B,PHLDA2 |
| miR-5581-3p (miRNAs w/seed UCCAUGC) | mature microRNA | | 1,86E-02 | AGT,DYRK1A,EGR1,EPHX2,FOS,JUN,NFKBIA,NFKBIZ |
| miR-3148 (miRNAs w/seed GGAAAAA) | mature microRNA | Inhibited | 1,90E-02 | ARRDC3,BCL10,DCBLD2,ETS1,KLF6,MXD1,SMIM14,VIM |
| LEPR | transmembrane receptor | | 1,90E-02 | FOS,LDLR,SERPINE1,VGF |
| RB1 | transcription regulator | | 1,92E-02 | CCND1,CXCL2,EGR1,FOS,MYC,NFKBIA,SERPINE1 |
| nitrofen | chemical toxicant | | 1,92E-02 | EGR1,ITGA6 |
| NSD2 | enzyme | | 1,92E-02 | FOS,JUN |
| TSLP | cytokine | | 1,92E-02 | ICAM1,MYC |
| GDF9 | growth factor | | 1,92E-02 | ITGA6,PLAU |
| NQO1 | enzyme | | 1,92E-02 | BIRC3,CCND1 |
| SPRY2 | other | | 1,92E-02 | ETS1,FOS |
| IGHM | transmembrane receptor | | 1,92E-02 | JUN,MYC |
| MMP2 | peptidase | | 1,92E-02 | CCND1,MYC |
| ADORA2B | G-protein coupled receptor | | 1,92E-02 | CXCL2,SERPINE1 |
| IL9 | cytokine | | 1,92E-02 | JUNB,MYC |
| cilostazol | chemical drug | | 1,92E-02 | ICAM1,SERPINE1 |
| steroid | chemical - endogenous mammalian | | 1,92E-02 | FOS,MYC |
| S-adenosylmethionine | chemical - endogenous mammalian | | 1,92E-02 | FOS,PLAU |
| RBL2 | other | | 1,92E-02 | CCND1,KLF10,MYC |
| NR0B2 | ligand-dependent nuclear receptor | | 1,92E-02 | AGT,EGR1,SERPINE1 |
| miR-1471 (miRNAs w/seed CCCGCGU) | mature microRNA | | 1,94E-02 | AGT,CCND1,RHOB,VGF |
| miR-6822-3p (miRNAs w/seed GGCUCUA) | mature microRNA | Inhibited | 1,95E-02 | AKAP12,ATF3,DYRK1A,H3C14,H3C15,H3C4,PARD6B,SEPHS2,TNFAIP3,TXNIP,UBASH3B |
| miR-3152-3p (and other miRNAs w/seed GUGUUAG) | mature microRNA | Inhibited | 1,96E-02 | ATF3,ETS1,TENT5A,TSC22D2,ZC3H12A,ZNF554 |
| SHH | peptidase | | 1,97E-02 | CCND1,MYC,SERPINE1,TRRAP |
| miR-1180-3p (miRNAs w/seed UUCCGGC) | mature microRNA | | 1,97E-02 | ETS1,H2BC5,NDRG1,SPRY4,VGF |
| miR-769-5p (miRNAs w/seed GAGACCU) | mature microRNA | | 1,98E-02 | ICAM1,KLF10,PARD6B,PLAU,SERINC5,TENT5A,ZBTB43 |
| miR-589-5p (and other miRNAs w/seed GAGAACC) | mature microRNA | | 2,00E-02 | EGR1,ERRFI1,MXD1,NUAK2,SEPHS2,SPRY4,TBX3 |
| miR-4432 (miRNAs w/seed AAGACUC) | mature microRNA | | 2,01E-02 | AFP,MYC,OAT,PARD6B,PHLDA2,SMIM14 |
| IL18 | cytokine | | 2,01E-02 | ATF3,ICAM1,JUN,NFKBIZ |
| CYLD | transcription regulator | | 2,02E-02 | JUN,SERPINE1 |
| NR2F1 | ligand-dependent nuclear receptor | | 2,02E-02 | AGT,EGR1 |
| paricalcitol | chemical drug | | 2,02E-02 | AGT,ICAM1 |

| | | | | |
|---|---|---|---|---|
| fluvastatin | chemical drug | | 2,02E-02 | CCND1,SERPINE1 |
| INSIG1 | other | | 2,02E-02 | CXCL2,LDLR,NFKBIZ |
| NR1H4 | ligand-dependent nuclear receptor | | 2,04E-02 | FABP1,LDLR,MYC,TXNIP |
| miR-455-5p (and other miRNAs w/seed AUGUGCC) | mature microRNA | | 2,05E-02 | ARRDC3,ETS1,MXD1,OAT,PARD6B,PRR15L,ZNF441,ZNF627 |
| INHA | growth factor | | 2,08E-02 | IGFBP4,MYC,SERPINE1 |
| CGA | other | | 2,08E-02 | CCND1,CTSV,MXD4 |
| miR-4681 (miRNAs w/seed ACGGGAA) | mature microRNA | | 2,08E-02 | AHSG,ARRDC3,LDLR,NDRG1,NUAK2,PHLDA2 |
| miR-1226-3p (miRNAs w/seed CACCAGC) | mature microRNA | | 2,08E-02 | ABL2,B4GALNT2,EMP1,FOS,PLAU,POLR2J2/POLR2J3,REEP6,SERPINE1,TNS4 |
| NKX2-1 | transcription regulator | | 2,08E-02 | AGT,IER3,PARD6B,SERPINE1 |
| EIF3E | other | | 2,11E-02 | CCND1,PLAU |
| CEACAM1 | transporter | | 2,11E-02 | ALDH1A1,ICAM1 |
| LG100268 | chemical reagent | | 2,11E-02 | LDLR,NDRG1 |
| miR-3687 (and other miRNAs w/seed CCGGACA) | mature microRNA | | 2,14E-02 | ATF3,DYRK1A,LDLR,REEP6,TBX3 |
| IRF4 | transcription regulator | | 2,15E-02 | BIRC3,MYC,RHOB,ZBTB20 |
| IRF1 | transcription regulator | | 2,15E-02 | CCND1,CXCL2,MYC,TBX3 |
| MMP3 | peptidase | | 2,18E-02 | EGR1,NDRG1,VIM |
| let-7d-3p (miRNAs w/seed UAUACGA) | mature microRNA | | 2,19E-02 | BCL10,CTSV,KLF6,ZNF627 |
| miR-4477a (miRNAs w/seed UAUUAAG) | mature microRNA | | 2,20E-02 | AHSG,ALDH1A1,BHLHE40,GEM,PHLDA2 |
| miR-526a-5p (and other miRNAs w/seed UCUAGAG) | mature microRNA | Inhibited | 2,20E-02 | ATF3,AXL,CYLD,MXD1,NFKBIZ,NUAK2,TNFAIP3 |
| conjugated linoleic acid | chemical drug | | 2,22E-02 | DUSP1,FOS |
| LGR4 | transmembrane receptor | | 2,22E-02 | OAT,VIM |
| memantine | chemical drug | | 2,22E-02 | FOS,NFKBIA |
| miR-6818-3p (miRNAs w/seed UGUCUCU) | mature microRNA | Inhibited | 2,22E-02 | ANKRD1,CXCL2,CYP1A1,IGFBP4,JUN,RGS2,TTR,UBASH3B |
| 13(S)-hydroxyoctadecadienoic acid | chemical - endogenous mammalian | | 2,22E-02 | SERPINE1 |
| CGP 74514A | chemical - kinase inhibitor | | 2,22E-02 | CCND1 |
| 6-dimethylaminopurine | chemical - endogenous mammalian | | 2,22E-02 | ICAM1 |
| adenine | chemical - endogenous mammalian | | 2,22E-02 | FABP1 |
| PD 180970 | chemical - kinase inhibitor | | 2,22E-02 | MYC |
| L-alanine | chemical - endogenous mammalian | | 2,22E-02 | CCND1 |
| ribose | chemical - endogenous mammalian | | 2,22E-02 | ICAM1 |
| inecalcitol | chemical drug | | 2,22E-02 | CCND1 |
| Osteocalcin | group | | 2,22E-02 | CCND1 |
| chondroitin sulfate B | chemical - endogenous mammalian | | 2,22E-02 | ICAM1 |
| colfosceril palmitate | chemical - endogenous mammalian | | 2,22E-02 | ICAM1 |
| darbepoetin alfa | biologic drug | | 2,22E-02 | ICAM1 |
| SHP | group | | 2,22E-02 | EGR1 |
| Rsk | group | | 2,22E-02 | CXCL2 |
| tyrosine kinase | group | | 2,22E-02 | CYP1A1 |
| Stat dimer | complex | | 2,22E-02 | FOS |
| Esr1-Esr1-estrogen-estrogen | complex | | 2,22E-02 | MYC |
| CC8490 | chemical drug | | 2,22E-02 | IER3 |
| 1-methyl-2-pyrrolidinone | chemical drug | | 2,22E-02 | FOS |
| aluminum hydroxide | chemical drug | | 2,22E-02 | ICAM1 |
| benoxaprofen | chemical drug | | 2,22E-02 | CYP1A1 |
| tylophorine | chemical drug | | 2,22E-02 | JUN |
| NPSR1 | G-protein coupled receptor | | 2,22E-02 | MYC |
| CINP | other | | 2,22E-02 | CCND1 |
| PDE8A | enzyme | | 2,22E-02 | LDLR |
| GPR65 | G-protein coupled receptor | | 2,22E-02 | MYC |
| DNAJB4 | other | | 2,22E-02 | CCND1 |
| AMOTL2 | other | | 2,22E-02 | ANKRD1 |
| GLIPR2 | other | | 2,22E-02 | VIM |
| LINC00887 | other | | 2,22E-02 | VIM |
| ENTPD2 | enzyme | | 2,22E-02 | SERPINE1 |
| GJB6 | transporter | | 2,22E-02 | GJB1 |
| SLCO1C1 | transporter | | 2,22E-02 | ALDH1A1 |
| CIRBP | translation regulator | | 2,22E-02 | CXCL2 |
| endothelin receptor | group | | 2,22E-02 | FOS |
| Slfn1 | enzyme | | 2,22E-02 | CCND1 |
| ORAI3 | other | | 2,22E-02 | CCND1 |
| MUCL1 | other | | 2,22E-02 | CCND1 |
| SLC39A4 | transporter | | 2,22E-02 | CCND1 |
| XRN2 | enzyme | | 2,22E-02 | MYC |
| Sapk | group | | 2,22E-02 | FOS |
| VLDL | complex | | 2,22E-02 | SERPINE1 |
| PDLIM1 | transcription regulator | | 2,22E-02 | VIM |
| BIIB021 | chemical drug | | 2,22E-02 | MDM2 |
| Casein | group | | 2,22E-02 | ICAM1 |
| Nc2 | complex | | 2,22E-02 | MYC |
| RAB7 | group | | 2,22E-02 | MYC |
| ZNF467 | transcription regulator | | 2,22E-02 | FOS |
| bitertanol | chemical drug | | 2,22E-02 | CYP1A1 |
| N-docosahexaenoylethanolamine | chemical - endogenous mammalian | | 2,22E-02 | JUN |
| PF-4691502 | chemical drug | | 2,22E-02 | VIM |
| KCNJ2 | ion channel | | 2,22E-02 | FOS |
| TLCD3A | other | | 2,22E-02 | CCND1 |
| ADRA2B | G-protein coupled receptor | | 2,22E-02 | CCND1 |
| ARTN | growth factor | | 2,22E-02 | ALDH1A1 |
| PSME3 | peptidase | | 2,22E-02 | MDM2 |
| G0S2 | other | | 2,22E-02 | MYC |
| ARID1B | transcription regulator | | 2,22E-02 | FOS |
| mir-338 | microRNA | | 2,22E-02 | CCND1 |
| mir-138 | microRNA | | 2,22E-02 | VIM |
| mir-32 | microRNA | | 2,22E-02 | MDM2 |
| TOPORS | enzyme | | 2,22E-02 | MDM2 |
| CALCR | G-protein coupled receptor | | 2,22E-02 | FOS |
| LIMK1 | kinase | | 2,22E-02 | MYC |
| TIAM1 | other | | 2,22E-02 | JUN |
| SEPTIN9 | enzyme | | 2,22E-02 | CCND1 |
| CALM1 (includes others) | other | | 2,22E-02 | TNFAIP3 |
| HUNK | kinase | | 2,22E-02 | MYC |
| GALR1 | G-protein coupled receptor | | 2,22E-02 | CCND1 |
| TCF20 | transcription regulator | | 2,22E-02 | MYC |
| lanatoside C | chemical - endogenous non-mammalian | | 2,22E-02 | TNFRSF10B |
| HSPB6 | other | | 2,22E-02 | ICAM1 |
| GIPC1 | other | | 2,22E-02 | SERPINE1 |
| NME2 | kinase | | 2,22E-02 | MYC |
| UBTF | transcription regulator | | 2,22E-02 | CCND1 |
| SOS1 | other | | 2,22E-02 | PLAU |
| CHRM4 | G-protein coupled receptor | | 2,22E-02 | EGR1 |
| ABI1 | other | | 2,22E-02 | EGR1 |
| Cyp2c70 | enzyme | | 2,22E-02 | LDLR |
| CTNNAL1 | other | | 2,22E-02 | CCND1 |
| CCKAR | G-protein coupled receptor | | 2,22E-02 | EGR1 |
| ADRA2A | G-protein coupled receptor | | 2,22E-02 | CYP1A1 |
| WARS1 | enzyme | | 2,22E-02 | NFKBIA |
| ARHGEF2 | other | | 2,22E-02 | CCND1 |
| FOXH1 | transcription regulator | | 2,22E-02 | ALDH1A1 |
| LETMD1 | other | | 2,22E-02 | MDM2 |
| SERPINB7 | other | | 2,22E-02 | ICAM1 |
| AKAP13 | other | | 2,22E-02 | FOS |
| chelidonine | chemical - endogenous non-mammalian | | 2,22E-02 | PHLDA1 |
| S3I-1757 | chemical reagent | | 2,22E-02 | CCND1 |
| COL1A2 | other | | 2,22E-02 | SERPINE1 |
| CRLF1 | other | | 2,22E-02 | ICAM1 |
| PLAC8 | other | | 2,22E-02 | CCND1 |
| ERO1A | enzyme | | 2,22E-02 | CXCL2 |
| HSPH1 | other | | 2,22E-02 | MYC |
| ARF1 | enzyme | | 2,22E-02 | CCND1 |
| TOP2A | enzyme | | 2,22E-02 | MYC |
| PODXL | kinase | | 2,22E-02 | VIM |
| RPL5 | other | | 2,22E-02 | MYC |
| TBPL1 | transcription regulator | | 2,22E-02 | FOS |
| MAD1L1 | other | | 2,22E-02 | MYC |
| 3',4'-dimethoxyflavone | chemical - endogenous non-mammalian | | 2,22E-02 | CYP1A1 |
| LY5 | chemical reagent | | 2,22E-02 | CCND1 |
| OSU-CG12 | chemical reagent | | 2,22E-02 | KLF6 |
| benfur | chemical reagent | | 2,22E-02 | MDM2 |
| SRT2183 | chemical reagent | | 2,22E-02 | LDLR |
| abrocitinib | chemical drug | | 2,22E-02 | MYC |
| 5,6,7,8-tetrahydrobiopterin | chemical - endogenous mammalian | | 2,22E-02 | MYC |
| apicularen A | chemical reagent | | 2,22E-02 | NFKBIA |
| acetoacetic acid | chemical - endogenous mammalian | | 2,22E-02 | CCND1 |
| ketanserin | chemical drug | | 2,22E-02 | FOS |
| NSC719239 | chemical drug | | 2,22E-02 | ITGA2 |
| hoechst 33342 | chemical reagent | | 2,22E-02 | FOS |
| tipifarnib | chemical drug | | 2,22E-02 | TNFRSF10B |
| KP-SD-1 | chemical - kinase inhibitor | | 2,22E-02 | CCND1 |
| clomiphene | chemical drug | | 2,22E-02 | LDLR |
| bilobalide | chemical - endogenous non-mammalian | | 2,22E-02 | MYC |
| liarozole | chemical drug | | 2,22E-02 | PI3 |
| alpha-methylhydrocinnamic acid | chemical - endogenous mammalian | | 2,22E-02 | MYC |
| phenylhydrazine | chemical toxicant | | 2,22E-02 | SELENOW |
| glabridin | chemical - endogenous non-mammalian | | 2,22E-02 | FOS |
| pentamidine | chemical drug | | 2,22E-02 | TNFRSF10B |
| tramadol | chemical drug | | 2,22E-02 | FOS |
| lomefloxacin | chemical drug | | 2,22E-02 | MDM2 |
| cromakalim | chemical drug | | 2,22E-02 | FOS |
| bupropion | chemical drug | | 2,22E-02 | TNFRSF10B |
| nitrendipine | chemical drug | | 2,22E-02 | LDLR |
| salicylamide | chemical drug | | 2,22E-02 | CYP1A1 |
| 4-methylcatechol | chemical - endogenous mammalian | | 2,22E-02 | JUN |
| methylpiperidinyl-3b-hydroxycholenamide | chemical reagent | | 2,22E-02 | LDLR |
| 1,1-diethyl-2-hydroxy-2-nitrosohydrazine | chemical toxicant | | 2,22E-02 | NFKBIA |
| MS645 | chemical reagent | | 2,22E-02 | MYC |
| CCI-007 | chemical reagent | | 2,22E-02 | MYC |
| WZB117 | chemical reagent | | 2,22E-02 | PI3 |
| Phe-Pro-Arg-chloromethyl ketone | chemical - protease inhibitor | | 2,22E-02 | ICAM1 |
| microcystin-LR | chemical toxicant | | 2,22E-02 | MYC |
| bisindolylmaleimide II | chemical - kinase inhibitor | | 2,22E-02 | JUN |
| resiniferatoxin | chemical drug | | 2,22E-02 | FOS |
| galangin | chemical - endogenous non-mammalian | | 2,22E-02 | ICAM1 |
| nor-binaltorphimine | chemical reagent | | 2,22E-02 | FOS |
| colcemid | chemical - endogenous non-mammalian | | 2,22E-02 | CYP1A1 |
| palmatine | chemical toxicant | | 2,22E-02 | CYP1A1 |
| tyrphostin AG 18 | chemical - kinase inhibitor | | 2,22E-02 | AKR1C3 |
| iodixanol | chemical drug | | 2,22E-02 | SERPINE1 |
| L-asparagine | chemical - endogenous mammalian | | 2,22E-02 | CCND1 |
| N-butyldeoxynojirimycin | chemical drug | | 2,22E-02 | ICAM1 |
| Collagen type VI | complex | | 2,22E-02 | CCND1 |
| trehalose | chemical - endogenous mammalian | | 2,22E-02 | VIM |
| cholesterol ester | chemical - endogenous mammalian | | 2,22E-02 | LDLR |
| pyocyanin | chemical reagent | | 2,22E-02 | ICAM1 |
| Co2+ | chemical - endogenous mammalian | | 2,22E-02 | NDRG1 |
| Pb2+ | chemical reagent | | 2,22E-02 | CYP1A1 |
| miR-6728-3p (miRNAs w/seed CUCUGCU) | mature microRNA | | 2,26E-02 | AHSG,ASB2,ATF3,CXCL2,EPHX2,ERRFI1,MAFF,MXD1,TTR |
| miR-3571 (and other miRNAs w/seed ACACACU) | mature microRNA | Inhibited | 2,28E-02 | CCND1,CXCL2,FABP1,FOS,KLF6,TENT5A,TNFAIP3,ZNF627 |
| miR-221-3p (and other miRNAs w/seed GCUACAU) | mature microRNA | Inhibited | 2,28E-02 | ALDH1A1,ATF3,DYRK1A,ETS1,FOS,ICAM1,TENT5A,TIPARP,ZFP36 |
| miR-4317 (miRNAs w/seed CAUUGCC) | mature microRNA | | 2,29E-02 | ATF3,B4GALNT2,LCA5,NDRG1,POLR2J2/POLR2J3,ZFP36,ZNF627 |
| MSX2 | transcription regulator | | 2,32E-02 | CCND1,VIM |
| CD2 | transmembrane receptor | | 2,32E-02 | CCND1,ICAM1 |
| PEBP1 | other | | 2,32E-02 | CXCL2,TNFRSF10B |
| PTGS1 | enzyme | | 2,32E-02 | ATF3,GEM |
| EFNA4 | kinase | | 2,32E-02 | DCBLD2,DUSP6 |
| CCR5 | G-protein coupled receptor | | 2,32E-02 | CXCL2,FOS |
| FASLG | cytokine | | 2,32E-02 | EGR1,FOS |
| ibuprofen | chemical drug | | 2,32E-02 | CCND1,TNFRSF10B |
| everolimus | chemical drug | | 2,32E-02 | CCND1,MYC |
| SAHM1 | chemical reagent | | 2,32E-02 | BHLHE40,MYC |
| LDB1 | transcription regulator | | 2,32E-02 | ABL2,NDRG1,SPRED1,TNFAIP3,TXNIP |
| miR-1973 (miRNAs w/seed CCGUGCA) | mature microRNA | | 2,32E-02 | AFAP1,DYRK1A,MAFF,PHLDA2,SEPHS2,ZNF844 |
| miR-12201-5p (and other miRNAs w/seed GGAAGGA) | mature microRNA | Inhibited | 2,32E-02 | ABL2,BHLHE40,EPHX2,ERRFI1,GJB1,H2BC5,IER3,IGFBP4,JUNB,SPRY4,TNS4,TRRAP,ZNF441 |
| miR-875-3p (miRNAs w/seed CUGGAAA) | mature microRNA | | 2,33E-02 | ERRFI1,ETS1,KLF6,MXD4,NDRG1,PRR15L,VIM,ZFP36,ZNF627 |
| INHBA | growth factor | | 2,34E-02 | AKR1C3,ANKRD1,JUNB,SERPINE1 |
| colistin | biologic drug | | 2,35E-02 | CCND1,FOS,JUNB |
| LMO2 | transcription regulator | | 2,35E-02 | ABL2,NDRG1,SPRED1,TNFAIP3,TXNIP |
| RRP1B | transcription regulator | | 2,40E-02 | CCND1,MDM2,ZNF554 |
| SOX4 | transcription regulator | | 2,42E-02 | EMP1,FOS,IGFBP4,VIM |
| PXR ligand-PXR-Retinoic acid-RXRα | complex | | 2,42E-02 | ALDH1A1,GSTA2 |
| SLC27A2 | transporter | | 2,42E-02 | ALDH1A1,FABP1 |
| EED | transcription regulator | | 2,42E-02 | MYC,TBX3 |
| RPS6KB1 | kinase | | 2,42E-02 | CCND1,SERPINE1 |
| SELPLG | other | | 2,42E-02 | CXCL2,FOS |
| DPP4 | peptidase | | 2,42E-02 | CXCL2,PLAU |
| ARHGAP21 | other | | 2,42E-02 | CXCL2,NFKBIZ |
| EFNA3 | kinase | | 2,42E-02 | DCBLD2,DUSP6 |
| fasudil | chemical drug | | 2,42E-02 | ICAM1,JUN |
| beta-carotene | chemical - endogenous mammalian | | 2,42E-02 | ARRDC3,NR1D1 |
| miR-454-5p (miRNAs w/seed CCCUAUC) | mature microRNA | | 2,42E-02 | CYP1A1,DUSP6,EMP1,PRR15L,TNFAIP3,ZNF441 |
| miR-1970 (and other miRNAs w/seed GUGUCAC) | mature microRNA | Inhibited | 2,44E-02 | ASB2,DCBLD2,EMP1,FOS,LDLR,MXD1,TNFRSF10B |
| miR-6076 (and other miRNAs w/seed GCAUGAC) | mature microRNA | Inhibited | 2,44E-02 | EMP1,ETS1,KLF6,RHOB,TSC22D2,ZBTB43,ZNF627 |
| miR-4722-5p (miRNAs w/seed GCAGGAG) | mature microRNA | | 2,45E-02 | FABP1,INAFM1,MDM2,NDRG1,NUAK2,PARD6B,POLR2J2/POLR2J3,TNFAIP3,ZNF442,ZNF844 |
| kanamycin A | chemical drug | | 2,46E-02 | CCND1,FOS,JUNB |
| miR-5135 (and other miRNAs w/seed GGUCUAG) | mature microRNA | | 2,51E-02 | CSRNP1,DYRK1A,ERRFI1,MXD1,PER1,PHLDA1,TSPAN8 |
| SKIL | transcription regulator | | 2,53E-02 | AFP,SERPINE1 |
| TNC | other | | 2,53E-02 | FOS,MYC |
| ELANE | peptidase | | 2,53E-02 | CXCL2,FOS |
| ANXA1 | enzyme | | 2,53E-02 | CXCL2,PLAU |
| CITED2 | transcription regulator | | 2,53E-02 | DUSP1,TNFAIP3 |
| ILK | kinase | | 2,53E-02 | CCND1,ITGA6 |
| SST | other | | 2,53E-02 | CCND1,FOS |
| NME1 | kinase | | 2,53E-02 | CCND1,SERPINE1 |
| U73122 | chemical reagent | | 2,53E-02 | AGT,ICAM1 |
| miR-192-5p (and other miRNAs w/seed UGACCUA) | mature microRNA | | 2,54E-02 | ATF3,CXCL2,DYRK1A,EMP1,GSTA2,KLF10,SERPINE1,SMIM14 |
| miR-411-5p (and other miRNAs w/seed AGUAGAC) | mature microRNA | Inhibited | 2,54E-02 | ARRDC3,DUSP1,IER3,SPRY4,TXNIP |
| GATA6 | transcription regulator | | 2,54E-02 | AFP,DCBLD2,LAMA1,MYC |
| miR-503-3p (and other miRNAs w/seed GAGUAUU) | mature microRNA | Inhibited | 2,56E-02 | ARRDC3,EMP1,MDM2,SPRED1,ZNF441,ZNF555 |
| miR-1291 (and other miRNAs w/seed UGGCUCU) | mature microRNA | Inhibited | 2,56E-02 | AKAP12,ATF3,H3C14,H3C15,H3C4,TXNIP |
| miR-4664-3p (miRNAs w/seed UUCCGGU) | mature microRNA | | 2,57E-02 | CCND1,ITGA6,NDRG1,SPRY4,VGF |
| EOMES | transcription regulator | | 2,57E-02 | AFAP1,ITGA2,RGS2 |
| miR-199a-3p (and other miRNAs w/seed CAGUAGU) | mature microRNA | Inhibited | 2,60E-02 | DCBLD2,EGR1,FOS,ITGA6,MDM2,PALD1,ZBTB43,ZNF441 |
| miR-500a-5p (miRNAs w/seed AAUCCUU) | mature microRNA | | 2,61E-02 | EMP1,MXD1,OAT,UBASH3B,ZBTB20 |
| miR-6752-3p (miRNAs w/seed CCCUGCC) | mature microRNA | | 2,61E-02 | AFAP1,CXCL2,EGR1,ERRFI1,FABP1,GJB1,H3C14,H3C15,ITGA6,PLAU,POLR2J2/POLR2J3,PRR15L,SERINC5,ZNF554 |
| miR-6124 (miRNAs w/seed GGAAAAG) | mature microRNA | | 2,63E-02 | BCL10,BHLHE40,GSTA2,PDZK1 |
| miR-4290 (miRNAs w/seed GCCCUCC) | mature microRNA | | 2,63E-02 | ASB2,BIRC3,CCND1,EMP1,EPHA2,ERRFI1,ETS1,GJB1,MAFF,NDRG1,PRR15L,RHOB,SERINC5 |
| miR-3686 (and other miRNAs w/seed UCUGUAA) | mature microRNA | Inhibited | 2,63E-02 | AKAP12,CCND1,H2BC5,KLF6,MDM2,SMIM14,VIM,YOD1,ZNF608 |
| miR-3117-3p (miRNAs w/seed UAGGACU) | mature microRNA | | 2,63E-02 | ALDH1A1,CYLD,ETS1,KRT6A,PI3,ZBTB43,ZNF441 |
| RBM5 | other | | 2,64E-02 | BIRC3,ICAM1 |
| A2M | transporter | | 2,64E-02 | CCND1,PPP1R15A |
| EFNA5 | kinase | | 2,64E-02 | DCBLD2,DUSP6 |
| PCGF2 | transcription regulator | | 2,64E-02 | MYC,VIM |
| dimethylnitrosamine | chemical toxicant | | 2,64E-02 | ICAM1,VIM |
| acetovanillone | chemical drug | | 2,64E-02 | DUSP1,SERPINE1 |
| miR-4713-5p (miRNAs w/seed UCUCCCA) | mature microRNA | Inhibited | 2,66E-02 | BIRC3,DYRK1A,EGR1,EMP1,H3C14,H3C15,IER2,LDLR,MAFF,PALD1,PLK2,SMIM14,TENT5A |
| sodium bisulfide | chemical reagent | | 2,75E-02 | AGT,ICAM1 |

| | | | | |
|---|---|---|---|---|
| PRKAA | group | | 2,75E-02 | DUSP1,VIM |
| mir-146 | microRNA | | 2,75E-02 | DUSP1,MYC |
| MED13 | transcription regulator | | 2,75E-02 | ANKRD1,MYC |
| MEF2A | transcription regulator | | 2,75E-02 | FOS,JUN |
| RIPK1 | kinase | | 2,75E-02 | CXCL2,MDM2 |
| CRP | other | | 2,75E-02 | EGR1,SERPINE1 |
| PRKACA | kinase | | 2,75E-02 | CCND1,MYC |
| Pam3-Cys | chemical toxicant | | 2,75E-02 | CXCL2,SERPINE1 |
| temozolomide | chemical drug | | 2,75E-02 | NFKBIA,TNFRSF10B |
| miR-153-3p (miRNAs w/seed UGCAUAG) | mature microRNA | Inhibited | 2,75E-02 | GEM,IER3,LDLR,PARD6B,SMIM14,SPRED1,TBX3,ZNF441,ZNF555 |
| metronidazole | chemical drug | | 2,75E-02 | CCND1,FOS,JUNB |
| 1,2-dioctanoyl-sn-glycerol | chemical reagent | | 2,77E-02 | JUN |
| L-aspartic acid | chemical - endogenous mammalian | | 2,77E-02 | CCND1 |
| p38 Sapk | group | | 2,77E-02 | CCND1 |
| ILX-23-7553 | chemical drug | | 2,77E-02 | CCND1 |
| L-alpha-lysophosphatidylcholine, palmitoyl | chemical - endogenous mammalian | | 2,77E-02 | ICAM1 |
| C9orf116 | other | | 2,77E-02 | MDM2 |
| styrene | chemical toxicant | | 2,77E-02 | EGR1 |
| Hbb | other | | 2,77E-02 | ICAM1 |
| ATPase | group | | 2,77E-02 | ICAM1 |
| MNK1/2 | group | | 2,77E-02 | MYC |
| Cpla2 | group | | 2,77E-02 | MYC |
| Erm | group | | 2,77E-02 | ICAM1 |
| Ho | group | | 2,77E-02 | ICAM1 |
| 1,2,3,4,6,7,8-heptachlorodibenzofuran | chemical toxicant | | 2,77E-02 | CYP1A1 |
| olmesartan | chemical drug | | 2,77E-02 | ICAM1 |
| DGKE | kinase | | 2,77E-02 | ICAM1 |
| SMPD3 | enzyme | | 2,77E-02 | ICAM1 |
| DRD3 | G-protein coupled receptor | | 2,77E-02 | FOS |
| MELK | kinase | | 2,77E-02 | MDM2 |
| MARCHF3 | other | | 2,77E-02 | NFKBIA |
| KLF8 | transcription regulator | | 2,77E-02 | CCND1 |
| BCAS2 | other | | 2,77E-02 | MDM2 |
| GPR17 | G-protein coupled receptor | | 2,77E-02 | FOS |
| SSBP2 | transcription regulator | | 2,77E-02 | MYC |
| BPI | transporter | | 2,77E-02 | ICAM1 |
| MTF2 | transcription regulator | | 2,77E-02 | TBX3 |
| FOXK2 | transcription regulator | | 2,77E-02 | SERPINE1 |
| Ftx | other | | 2,77E-02 | JUN |
| PYHIN1 | other | | 2,77E-02 | MDM2 |
| UTP18 | other | | 2,77E-02 | MYC |
| ZDHHC2 | enzyme | | 2,77E-02 | VIM |
| SNHG3 | other | | 2,77E-02 | MYC |
| CDH16 | enzyme | | 2,77E-02 | CCND1 |
| PCA3 | other | | 2,77E-02 | NDRG1 |
| RPE65 | enzyme | | 2,77E-02 | FOS |
| GPR68 | G-protein coupled receptor | | 2,77E-02 | DUSP1 |
| harmol | chemical - endogenous non-mammalian | | 2,77E-02 | CYP1A1 |
| Cathepsin | group | | 2,77E-02 | CCND1 |
| 7S NGF | complex | | 2,77E-02 | FOS |
| MAP4K1 | kinase | | 2,77E-02 | BIRC3 |
| Atf | group | | 2,77E-02 | JUN |
| GRAP2 | other | | 2,77E-02 | MYC |
| ZIC1 | transcription regulator | | 2,77E-02 | CCND1 |
| FAF1 | other | | 2,77E-02 | VIM |
| ERN2 | kinase | | 2,77E-02 | ICAM1 |
| PUF60 | other | | 2,77E-02 | MYC |
| TYMS | enzyme | | 2,77E-02 | MYC |
| NCK2 | kinase | | 2,77E-02 | PPP1R15A |
| RNF34 | enzyme | | 2,77E-02 | MDM2 |
| LOC102724788/PRODH | enzyme | | 2,77E-02 | TNFRSF10B |
| mir-190 | microRNA | | 2,77E-02 | VIM |
| MIR4269 | microRNA | | 2,77E-02 | MYC |
| SFRP2 | transmembrane receptor | | 2,77E-02 | MYC |
| MRAS | enzyme | | 2,77E-02 | VIM |
| ADAR | enzyme | | 2,77E-02 | FOS |
| APAF1 | other | | 2,77E-02 | VIM |
| LSP1 | other | | 2,77E-02 | ITGA6 |
| MAPKAPK5 | kinase | | 2,77E-02 | TNFAIP3 |
| UTS2 | other | | 2,77E-02 | FOS |
| PENK | other | | 2,77E-02 | FOS |
| FLOT1 | other | | 2,77E-02 | CCND1 |
| ARHGEF25 | other | | 2,77E-02 | JUN |
| RAP1GDS1 | other | | 2,77E-02 | MYC |
| PORCN | enzyme | | 2,77E-02 | CCND1 |
| BRCA2 | transcription regulator | | 2,77E-02 | SERPINE1 |
| RPS3 | enzyme | | 2,77E-02 | NFKBIA |
| MYCT1 | other | | 2,77E-02 | MXD4 |
| CCL22 | cytokine | | 2,77E-02 | CXCL2 |
| BCAR3 | other | | 2,77E-02 | CCND1 |
| CEL | enzyme | | 2,77E-02 | CCND1 |
| PRMT2 | enzyme | | 2,77E-02 | CCND1 |
| SENP6 | peptidase | | 2,77E-02 | ICAM1 |
| Tmsb4x (includes others) | other | | 2,77E-02 | ICAM1 |
| ACOD1 | enzyme | | 2,77E-02 | TNFAIP3 |
| CMA1 | peptidase | | 2,77E-02 | AGT |
| VCAM1 | transmembrane receptor | | 2,77E-02 | CCND1 |
| PELI2 | enzyme | | 2,77E-02 | CXCL2 |
| CDC27 | other | | 2,77E-02 | CCND1 |
| GNL2 | enzyme | | 2,77E-02 | MYC |
| CDH13 | other | | 2,77E-02 | CCND1 |
| Nrg1 | other | | 2,77E-02 | FOS |
| KRT10 | other | | 2,77E-02 | CCND1 |
| Foxe3 | transcription regulator | | 2,77E-02 | TTR |
| Mcpt1 | peptidase | | 2,77E-02 | AGT |
| H1f4 | other | | 2,77E-02 | MYC |
| ILF2 | transcription regulator | | 2,77E-02 | FOS |
| LGH447 | chemical drug | | 2,77E-02 | MYC |
| CC-122 | chemical drug | | 2,77E-02 | MYC |
| MI-773 | chemical drug | | 2,77E-02 | MDM2 |
| U 50488H | chemical reagent | | 2,77E-02 | FOS |
| gabapentin | chemical drug | | 2,77E-02 | FOS |
| 2-cyclohexen-1-one | chemical - endogenous non-mammalian | | 2,77E-02 | ICAM1 |
| LY379196 | chemical - kinase inhibitor | | 2,77E-02 | EGR1 |
| silicon phthalocyanine | chemical drug | | 2,77E-02 | CCND1 |
| mevalonolactone | chemical reagent | | 2,77E-02 | ICAM1 |
| lindane | chemical drug | | 2,77E-02 | FOS |
| triiodothyronine, reverse | chemical - endogenous mammalian | | 2,77E-02 | EGR1 |
| ranitidine | chemical drug | | 2,77E-02 | CXCL2 |
| tranexamic acid | chemical drug | | 2,77E-02 | PLAU |
| 2,3,7,8-tetrachlorodibenzofuran | chemical toxicant | | 2,77E-02 | CYP1A1 |
| Collagen type V | complex | | 2,77E-02 | ITGA2 |
| etidronic acid | chemical drug | | 2,77E-02 | FOS |
| roflumilast | chemical drug | | 2,77E-02 | CXCL2 |
| paxilline | chemical - endogenous non-mammalian | | 2,77E-02 | TNFRSF10B |
| orantinib | chemical drug | | 2,77E-02 | ICAM1 |
| opioid | chemical drug | | 2,77E-02 | PLAU |
| H-8 | chemical toxicant | | 2,77E-02 | MYC |
| 4-aminophenol | chemical - endogenous mammalian | | 2,77E-02 | VIM |
| butylphen | chemical toxicant | | 2,77E-02 | TNFRSF10B |
| sodium azide | chemical toxicant | | 2,77E-02 | TXNIP |
| FT671 | chemical - protease inhibitor | | 2,77E-02 | MDM2 |
| dexfenfluramine | chemical drug | | 2,77E-02 | FOS |
| aclarubicin | chemical drug | | 2,77E-02 | TNFRSF10B |
| cannabinoid drugs | chemical drug | | 2,77E-02 | ICAM1 |
| phytol | chemical - endogenous non-mammalian | | 2,77E-02 | FABP1 |
| methyllycaconitine | chemical toxicant | | 2,77E-02 | FOS |
| phloroglucinol | chemical drug | | 2,77E-02 | FOS |
| 5-hydroxydecanoic acid | chemical - endogenous mammalian | | 2,77E-02 | FOS |
| 5-hydroxytryptophan | chemical - endogenous mammalian | | 2,77E-02 | FOS |
| acetyl-11-keto-beta-boswellic acid | chemical reagent | | 2,77E-02 | TNFRSF10B |
| Hg2+ | chemical - endogenous non-mammalian | | 2,77E-02 | CYP1A1 |
| miR-6726-3p (miRNAs w/seed UCGCCCU) | mature microRNA | | 2,81E-02 | PLAU,PRR15L,SLC25A25,SMIM14,TRRAP |
| melatonin | chemical - endogenous mammalian | | 2,81E-02 | FOS,JUNB,VIM |
| obeticholic acid | chemical drug | | 2,86E-02 | CXCL2,ICAM1 |
| miR-675-3p (miRNAs w/seed UGUAUGC) | mature microRNA | | 2,87E-02 | CYLD,ERRFI1,GSTA2,ITGA2,ITGA6,ZNF627 |
| miR-3977 (miRNAs w/seed UGCUUCA) | mature microRNA | | 2,87E-02 | ANKRD1,B4GALNT2,ERRFI1,ETS1,MXD1,SEPHS2 |
| miR-202-3p (and other miRNAs w/seed GAGGUAU) | mature microRNA | | 2,89E-02 | CCND1,DCBLD2,DUSP1,LAMA1,MXD1,PARD6B,SEPHS2,ZNF441 |
| cholecalciferol | chemical - endogenous mammalian | | 2,94E-02 | FOS,JUN,SERPINE1 |
| Hsp27 | group | | 2,97E-02 | CCND1,FOS |
| MEN1 | transcription regulator | | 2,97E-02 | CCND1,FOS |
| FYN | kinase | | 2,97E-02 | DUSP1,FOS |
| NOS3 | enzyme | | 2,97E-02 | ATF3,ICAM1 |
| NUP98-DDX10 | fusion gene/product | | 2,97E-02 | EGR1,MYC |
| miR-6817-3p (miRNAs w/seed CUCUCUG) | mature microRNA | Inhibited | 3,00E-02 | CXCL2,DUSP6,FGG,FOS,JUN,NUAK2,POLR2J2/POLR2J3,TENT5A,ZBTB43 |
| EIF4E | translation regulator | | 3,02E-02 | CCND1,IGFBP4,MYC,NFKBIA |
| miR-140-5p (and other miRNAs w/seed AGUGGUU) | mature microRNA | Inhibited | 3,03E-02 | CTSV,KLF6,NR1D1,PARD6B,SPRED1,TSC22D2,YOD1,ZNF441 |
| miR-92a-3p (and other miRNAs w/seed AUUGCAC) | mature microRNA | Inhibited | 3,04E-02 | ARRDC3,CCND1,DUSP1,INAFM1,ITGA6,KLF6,MDM2,TENT5A,UBASH3B |
| miR-291a-3p (and other miRNAs w/seed AAGUGCU) | mature microRNA | | 3,04E-02 | CCND1,FABP1,HIVEP2,MXD1,SMIM14,TIPARP,TNFRSF10B,TXNIP,YOD1,ZBTB43 |
| miR-4251 (miRNAs w/seed CUGAGAA) | mature microRNA | Inhibited | 3,05E-02 | ABL2,ATF3,EGR1,FOS,MXD1,MXD4,PER1,TXNIP |
| miR-7844-5p (miRNAs w/seed AAACUAG) | mature microRNA | | 3,06E-02 | RGS2,SMIM14,VIM,ZNF608 |
| nilotinib | chemical drug | | 3,09E-02 | MDM2,MYC |
| IL24 | cytokine | | 3,09E-02 | CCND1,PPP1R15A |
| LTBR | transmembrane receptor | | 3,09E-02 | ICAM1,NFKBIA |
| miR-6902-3p (miRNAs w/seed CAUGUGA) | mature microRNA | | 3,09E-02 | ARRDC3,EMP1,SERINC5,TNFRSF10B,ZNF627 |
| Ifn | group | | 3,13E-02 | FOS,ICAM1,SERPINE1 |
| miR-4751 (miRNAs w/seed GAGGACC) | mature microRNA | | 3,17E-02 | ATF3,EGR1,H3C14,H3C15,INAFM1,KLF6,MDM2,POLR2J2/POLR2J3,PRR15L |
| Hsp70 | group | | 3,20E-02 | ICAM1,NFKBIA |
| Hedgehog | group | | 3,20E-02 | CCND1,MYC |
| AURK | group | | 3,20E-02 | MDM2,TNFRSF10B |
| ANLN | other | | 3,20E-02 | MDM2,TNFRSF10B |
| miR-6529-3p (and other miRNAs w/seed CUGUGCC) | mature microRNA | | 3,22E-02 | AHSG,CYP1A1,DYRK1A,ETS1,FABP1,IER3,PALD1,ZBTB43,ZNF441 |
| miR-6753-5p (miRNAs w/seed ACCAGGG) | mature microRNA | Inhibited | 3,23E-02 | AFAP1,ETS1,LDLR,SERINC5,SPRED1,SPRY4,TIPARP,TNFAIP3,TNFRSF10B,UBASH3B,ZFP36 |
| miR-891a-3p (miRNAs w/seed GUGGCAC) | mature microRNA | Inhibited | 3,26E-02 | BCL10,FOS,ICAM1,ITGA6,TNFRSF10B,TSC22D2,UBASH3B,ZBTB43,ZNF844 |
| 20-hydroxyeicosatetraenoic acid | chemical - endogenous mammalian | | 3,32E-02 | EPHX2 |
| adenosine dialdehyde | chemical reagent | | 3,32E-02 | ICAM1 |
| 8-hydroxyguanine | chemical - endogenous mammalian | | 3,32E-02 | CXCL2 |
| histidinol | chemical - endogenous mammalian | | 3,32E-02 | EGR1 |
| nitrite | chemical - endogenous mammalian | | 3,32E-02 | ICAM1 |
| L-glutamine | chemical - endogenous mammalian | | 3,32E-02 | DUSP1 |
| Iberiotoxin | chemical reagent | | 3,32E-02 | ICAM1 |
| norethindrone acetate | chemical drug | | 3,32E-02 | SERPINE1 |
| Neurotrophin | group | | 3,32E-02 | FOS |
| Dynamin | group | | 3,32E-02 | FOS |
| F Actin | complex | | 3,32E-02 | CTSV |
| tacedinaline | chemical drug | | 3,32E-02 | FOS |
| obatoclax | chemical drug | | 3,32E-02 | ATF3 |
| DPPA3 | other | | 3,32E-02 | AFP |
| Beta Arrestin | group | | 3,32E-02 | FOS |
| EIF4A3 | enzyme | | 3,32E-02 | CCND1 |
| CCAR1 | transcription regulator | | 3,32E-02 | RGS2 |
| KCNK2 | ion channel | | 3,32E-02 | ICAM1 |
| KLF17 | transcription regulator | | 3,32E-02 | VIM |
| ANKHD1/ANKHD1-EIF4EBP3 | other | | 3,32E-02 | CCND1 |
| PDZK1IP1 | other | | 3,32E-02 | PDZK1 |
| RGMB | other | | 3,32E-02 | CXCL2 |
| CCN6 | growth factor | | 3,32E-02 | ICAM1 |
| AQP11 | transporter | | 3,32E-02 | MYC |
| lumefantrine | chemical drug | | 3,32E-02 | VIM |
| taspoglutide | biologic drug | | 3,32E-02 | FABP1 |
| LDL-cholesterol | complex | | 3,32E-02 | ICAM1 |
| GSK690693 | chemical drug | | 3,32E-02 | EPHA2 |
| peptide T | biologic drug | | 3,32E-02 | ICAM1 |
| RABGEF1 | enzyme | | 3,32E-02 | VGF |
| PXN | other | | 3,32E-02 | CCND1 |
| PRKACB | kinase | | 3,32E-02 | MYC |
| TFPI | other | | 3,32E-02 | ITGA2 |
| PRKD2 | kinase | | 3,32E-02 | ITGA2 |
| CSRP3 | other | | 3,32E-02 | TXNIP |
| HSPB2 | other | | 3,32E-02 | ICAM1 |
| BAD | other | | 3,32E-02 | CCND1 |
| OPRK1 | G-protein coupled receptor | | 3,32E-02 | FOS |
| RBBP4 | enzyme | | 3,32E-02 | MYC |
| CLDN1 | other | | 3,32E-02 | VIM |
| PROK2 | other | | 3,32E-02 | FOS |
| entolimod | biologic drug | | 3,32E-02 | CXCL2 |
| Pzp | other | | 3,32E-02 | LDLR |
| GNB5 | enzyme | | 3,32E-02 | TNFRSF10B |
| mir-637 | microRNA | | 3,32E-02 | CCND1 |
| NUCB2 | other | | 3,32E-02 | FOS |
| SNIP1 | other | | 3,32E-02 | CCND1 |
| Irs3 | other | | 3,32E-02 | EGR1 |
| Nisch | other | | 3,32E-02 | FOS |
| MAGED1 | transcription regulator | | 3,32E-02 | JUN |
| ETV3 | transcription regulator | | 3,32E-02 | MYC |
| AATF | transcription regulator | | 3,32E-02 | JUN |
| RNF111 | enzyme | | 3,32E-02 | SERPINE1 |
| OTUD7B | peptidase | | 3,32E-02 | ICAM1 |
| TRPC3 | ion channel | | 3,32E-02 | CCND1 |
| GRM5 | G-protein coupled receptor | | 3,32E-02 | FOS |
| DYNLL1 | other | | 3,32E-02 | FOS |
| SIX3 | transcription regulator | | 3,32E-02 | CCND1 |
| CCL15 | cytokine | | 3,32E-02 | ICAM1 |
| OXSR1 | kinase | | 3,32E-02 | MDM2 |
| CFH | other | | 3,32E-02 | CXCL2 |
| STK38 | kinase | | 3,32E-02 | CXCL2 |
| TDG | enzyme | | 3,32E-02 | MYC |
| GPS1 | other | | 3,32E-02 | FOS |
| ECT2 | other | | 3,32E-02 | CCND1 |
| CTSK | peptidase | | 3,32E-02 | CTSV |
| TFF2 | other | | 3,32E-02 | CCND1 |
| PPID | enzyme | | 3,32E-02 | VIM |
| XPA | other | | 3,32E-02 | FOS |
| BARD1 | transcription regulator | | 3,32E-02 | CYP1A1 |
| SERPIND1 | other | | 3,32E-02 | EGR1 |
| MTUS1 | other | | 3,32E-02 | VIM |
| SF1 | transcription regulator | | 3,32E-02 | ICAM1 |
| SRGN | other | | 3,32E-02 | VIM |
| OMP-52M51 | biologic drug | | 3,32E-02 | MYC |
| NET1 | other | | 3,32E-02 | SERPINE1 |
| equol | chemical - endogenous non-mammalian | | 3,32E-02 | MYC |
| CXXC4 | other | | 3,32E-02 | CCND1 |
| ZYX | other | | 3,32E-02 | FOS |
| fenebrutinib | chemical drug | | 3,32E-02 | MYC |
| COG112 peptide | chemical reagent | | 3,32E-02 | CXCL2 |

| | | | | |
|---|---|---|---|---|
| gelam honey | chemical reagent | | 3,32E-02 | NFKBIA |
| enzastaurin | chemical drug | | 3,32E-02 | TNFRSF10B |
| ambroxol | chemical drug | | 3,32E-02 | ICAM1 |
| emricasan | chemical drug | | 3,32E-02 | CXCL2 |
| 2-morpholin-4-yl-6-thianthren-1-yl-pyran-4-one | chemical reagent | | 3,32E-02 | TNFRSF10B |
| pyridoxamine | chemical - endogenous mammalian | | 3,32E-02 | SERPINE1 |
| chlorambucil | chemical drug | | 3,32E-02 | TNFRSF10B |
| halothane | chemical drug | | 3,32E-02 | FOS |
| eprosartan | chemical drug | | 3,32E-02 | SERPINE1 |
| PK11007 | chemical reagent | | 3,32E-02 | MDM2 |
| juglone | chemical toxicant | | 3,32E-02 | EGR1 |
| benz[a]anthracene | chemical toxicant | | 3,32E-02 | CYP1A1 |
| ruboxistaurin | chemical drug | | 3,32E-02 | EGR1 |
| JWH-015 | chemical reagent | | 3,32E-02 | ICAM1 |
| curcuminoids | chemical drug | | 3,32E-02 | ICAM1 |
| EPZ005687 | chemical - protease inhibitor | | 3,32E-02 | MYC |
| GSI34 | chemical reagent | | 3,32E-02 | CCND1 |
| PDZ1i | chemical reagent | | 3,32E-02 | ITGA6 |
| Z-VEID-FMK | chemical reagent | | 3,32E-02 | CCND1 |
| trigonelline | chemical - endogenous mammalian | | 3,32E-02 | AGT |
| alpha-hydroxyglutarate | chemical - endogenous mammalian | | 3,32E-02 | VIM |
| edelfosine | chemical drug | | 3,32E-02 | ATF3 |
| CV 3988 | chemical reagent | | 3,32E-02 | ICAM1 |
| remifentanil | chemical drug | | 3,32E-02 | FOS |
| 3',4',7-trihydroxyisoflavone | chemical - endogenous non-mammalian | | 3,32E-02 | CCND1 |
| 8-chlorophenylthio-adenosine 3',5'-cyclic monophosphate | chemical - kinase inhibitor | | 3,32E-02 | BIRC3,DUSP1 |
| H2AB3 (includes others) | other | | 3,32E-02 | ATF3,FOS |
| TEAD2 | transcription regulator | | 3,32E-02 | CCND1,NFKBIZ |
| beta-naphthoflavone | chemical toxicant | | 3,32E-02 | CYP1A1,FOS |
| E2f | group | | 3,33E-02 | ITGA6,MYC,SERPINE1 |
| miR-423-5p (and other miRNAs w/seed GAGGGGC) | mature microRNA | Inhibited | 3,35E-02 | AFAP1,ASB2,EGR1,GJB1,INAFM1,KRT6A,MAFF,PER1,REEP6,SELENOW,SERINC5,SPATA2,SPRY4,TNS4,VGF,ZFP36,ZNF608 |
| miR-4327 (miRNAs w/seed GCUUGCA) | mature microRNA | | 3,36E-02 | AGT,DYRK1A,PARD6B,TENT5A,ZNF627,ZNF844 |
| miR-4453 (and other miRNAs w/seed AGCUUGG) | mature microRNA | | 3,39E-02 | AXL,BHLHE40,MDM2,PDZK1,ZBTB43 |
| miR-3187-3p (miRNAs w/seed UGGCCAU) | mature microRNA | Inhibited | 3,39E-02 | CXCL2,EGR1,KLF6,MDM2,PLK2,TIPARP,ZNF441 |
| miR-656-3p (miRNAs w/seed AUAUUAU) | mature microRNA | Inhibited | 3,40E-02 | DUSP1,JUNB,RGS2,ZFP36 |
| miR-489-5p (miRNAs w/seed GUCGUAU) | mature microRNA | | 3,40E-02 | ETS1,FOS,RHOB |
| RUNX2 | transcription regulator | | 3,40E-02 | CCND1,PLAU,SERPINE1 |
| ZBTB20 | transcription regulator | | 3,44E-02 | AFP,NFKBIA |
| panobinostat | chemical drug | | 3,44E-02 | MYC,VIM |
| mir-183 | microRNA | | 3,44E-02 | EGR1,VIM |
| RTN4 | other | | 3,44E-02 | JUN,RHOB |
| miR-6505-5p (miRNAs w/seed UGGAAUA) | mature microRNA | | 3,48E-02 | ARRDC3,ATF3,CSRNP1,MXD1,NR1D1,SMIM14,TTR |
| miR-6771-3p (miRNAs w/seed AAACCCC) | mature microRNA | Inhibited | 3,50E-02 | EMP1,EPHX2,POLR2J2/POLR2J3,PPP1R15A,SPATA2,TNFRSF10B,TSC22D2,ZBTB43 |
| miR-660-3p (miRNAs w/seed CCUCCUG) | mature microRNA | | 3,54E-02 | CTSV,DYRK1A,EGR1,EMP1,FABP1,GJB1,MAFF,PMM1,PRR15L,SMIM14,UBASH3B,ZNF554 |
| bromodeoxyuridine | chemical drug | | 3,56E-02 | IGFBP4,SERPINE1 |
| MTORC1 | complex | | 3,56E-02 | CCND1,MYC |
| CD274 | enzyme | | 3,56E-02 | CXCL2,ICAM1 |
| miR-4325 (miRNAs w/seed UGCACUU) | mature microRNA | Inhibited | 3,60E-02 | ANKRD1,ARRDC3,DYRK1A,GEM,MDM2,PLK2,SPRED1 |
| TMPRSS2-ERG | fusion gene/product | | 3,61E-02 | MYC,PHLDA1,VIM |
| miR-432 (and other miRNAs w/seed CUUGGAG) | mature microRNA | | 3,61E-02 | ASB2,AXL,GSTA2,MXD1,NR1D1,SERPINA6,SERPINE1,TNS4 |
| miR-2392 (miRNAs w/seed AGGAUGG) | mature microRNA | | 3,61E-02 | BHLHE40,CCND1,GSTA2,INAFM1,NUAK2,POLR2J2/POLR2J3,TBX3,YOD1,ZC3H12A |
| miR-4520-3p (miRNAs w/seed UGGACAG) | mature microRNA | | 3,64E-02 | AFAP1,CYP1A1,FABP1,H3C14,H3C15,MAFF,PI3,SLC25A25,TIPARP |
| GRB2 | other | | 3,69E-02 | AFP,PLAU |
| CEBPE | transcription regulator | | 3,69E-02 | CTSV,MYC |
| LPL | enzyme | | 3,69E-02 | ICAM1,LDLR |
| EPHB4 | kinase | | 3,69E-02 | IGFBP4,MYC |
| SAFB | other | | 3,69E-02 | FOS,JUN |
| 3-nitropropionic acid | chemical toxicant | | 3,69E-02 | JUN,MYC |
| Am 580 | chemical toxicant | | 3,69E-02 | DUSP1,ITGA6 |
| miR-582-3p (miRNAs w/seed AACUGGU) | mature microRNA | | 3,69E-02 | CTSV,SERINC5,TENT5A,TSC22D2,ZBTB20,ZBTB43,ZNF627 |
| miR-642a-3p (and other miRNAs w/seed GACACAU) | mature microRNA | | 3,69E-02 | AGT,ANKRD1,ASB2,CYLD,EGR1,KLF6,SELENOW |
| miR-4734 (miRNAs w/seed CUGCGGG) | mature microRNA | | 3,73E-02 | CCND1,LCA5,NDRG1,SLC25A25,TNS4,ZNF627 |
| miR-5088-3p (miRNAs w/seed CCCUUCU) | mature microRNA | Inhibited | 3,80E-02 | CCND1,DUSP6,GJB1,H3C14,H3C15,IER2,KLF10,RGS2 |
| miR-8074 (miRNAs w/seed UAUGGCG) | mature microRNA | Inhibited | 3,80E-02 | ANKRD1,KLF6,MXD1,MYC,RGS2,TENT5A |
| MOG | other | | 3,81E-02 | CXCL2,ICAM1 |
| TSHZ3 | transcription regulator | | 3,81E-02 | IER2,TXNIP |
| FGF10 | growth factor | | 3,81E-02 | LDLR,TSPAN8 |
| CD5 | transmembrane receptor | | 3,81E-02 | ICAM1,LDLR |
| EHMT2 | transcription regulator | | 3,81E-02 | MDM2,MYC |
| miR-1292-3p (miRNAs w/seed CGCGCCC) | mature microRNA | Inhibited | 3,84E-02 | CCND1,CSRNP1,IER2,MAFF,POLR2J2/POLR2J3 |
| miR-1825 (miRNAs w/seed CCAGUGC) | mature microRNA | Inhibited | 3,85E-02 | ABL2,ARRDC3,ETS1,KLF10,KLF6,LCA5,PMM1,ZNF627 |
| alloxan | chemical reagent | | 3,86E-02 | ICAM1 |
| L-proline | chemical - endogenous mammalian | | 3,86E-02 | CCND1 |
| pervanadate | chemical reagent | | 3,86E-02 | NFKBIA |
| dopamine D1 receptor | group | | 3,86E-02 | FOS |
| flavokawain B | chemical - endogenous non-mammalian | | 3,86E-02 | TNFRSF10B |
| triamcinolone hexacetonide | chemical drug | | 3,86E-02 | ICAM1 |
| rh-endostatin | chemical drug | | 3,86E-02 | JUN |
| GPRC6A | G-protein coupled receptor | | 3,86E-02 | EGR1 |
| ISX | transcription regulator | | 3,86E-02 | CCND1 |
| MRPS18B | other | | 3,86E-02 | VIM |
| ST6GALNAC1 | enzyme | | 3,86E-02 | VIM |
| ZNF652 | other | | 3,86E-02 | VIM |
| SH2D5 | other | | 3,86E-02 | CCND1 |
| BCAR4 | other | | 3,86E-02 | VIM |
| SLC5A8 | transporter | | 3,86E-02 | TNFRSF10B |
| KMT5B | enzyme | | 3,86E-02 | DUSP1 |
| TCIM | other | | 3,86E-02 | ICAM1 |
| iguratimod | chemical drug | | 3,86E-02 | CXCL2 |
| sotrastaurin | chemical drug | | 3,86E-02 | NFKBIA |
| olaparib | chemical drug | | 3,86E-02 | TNFRSF10B |
| Sfk | group | | 3,86E-02 | ICAM1 |
| Collagen type III | complex | | 3,86E-02 | ITGA2 |
| HLA-DQ | complex | | 3,86E-02 | FOS |
| JDP2 | transcription regulator | | 3,86E-02 | JUN |
| penfluridol | chemical drug | | 3,86E-02 | ITGA6 |
| HNRNPR | other | | 3,86E-02 | PLK2 |
| ASCL2 | transcription regulator | | 3,86E-02 | EGR1 |
| idelalisib | chemical drug | | 3,86E-02 | MYC |
| IER3 | other | | 3,86E-02 | BIRC3 |
| PRKG2 | kinase | | 3,86E-02 | FOS |
| DVL1 | other | | 3,86E-02 | MYC |
| PDE6B | enzyme | | 3,86E-02 | FOS |
| REV1 | enzyme | | 3,86E-02 | TNFRSF10B |
| CCN4 | other | | 3,86E-02 | CCND1 |
| MEFV | other | | 3,86E-02 | MDM2 |
| BANP | other | | 3,86E-02 | CCND1 |
| IFNGR | complex | | 3,86E-02 | LAMA1 |
| WNK1 | kinase | | 3,86E-02 | SERPINE1 |
| SERPINA4 | other | | 3,86E-02 | MYC |
| PHIP | other | | 3,86E-02 | CCND1 |
| SNCB | other | | 3,86E-02 | MDM2 |
| ENO1 | enzyme | | 3,86E-02 | MYC |
| NUP107 | other | | 3,86E-02 | FOS |
| TSC22D1 | transcription regulator | | 3,86E-02 | SERPINE1 |
| RBX1 | enzyme | | 3,86E-02 | RHOB |
| EGR4 | transcription regulator | | 3,86E-02 | EGR1 |
| DLC1 | other | | 3,86E-02 | SERPINE1 |
| BANCR | other | | 3,86E-02 | CCND1 |
| GNAO1 | enzyme | | 3,86E-02 | FOS |
| P2RY1 | G-protein coupled receptor | | 3,86E-02 | FOS |
| PTBP3 | other | | 3,86E-02 | VIM |
| P2RY4 | G-protein coupled receptor | | 3,86E-02 | ICAM1 |
| TBXA2R | G-protein coupled receptor | | 3,86E-02 | ICAM1 |
| KHDRBS1 | transcription regulator | | 3,86E-02 | CCND1 |
| PIP5K1C | kinase | | 3,86E-02 | ERRFI1 |
| TP53INP1 | other | | 3,86E-02 | MDM2 |
| CUL7 | enzyme | | 3,86E-02 | VIM |
| FKBP1A | enzyme | | 3,86E-02 | MDM2 |
| OXTR | G-protein coupled receptor | | 3,86E-02 | FOS |
| FKBP5 | enzyme | | 3,86E-02 | ICAM1 |
| CUL1 | enzyme | | 3,86E-02 | MYC |
| PDK1 | kinase | | 3,86E-02 | VIM |
| CLIC4 | ion channel | | 3,86E-02 | CXCL2 |
| DMTF1 | transcription regulator | | 3,86E-02 | MDM2 |
| MLLT3 | transcription regulator | | 3,86E-02 | MYC |
| SHCBP1 | other | | 3,86E-02 | CCND1 |
| TNKS | enzyme | | 3,86E-02 | MYC |
| ERBIN | other | | 3,86E-02 | SERPINE1 |
| casticin | chemical - endogenous non-mammalian | | 3,86E-02 | TNFRSF10B |
| ADCYAP1R1 | G-protein coupled receptor | | 3,86E-02 | FOS |
| CCR4 | G-protein coupled receptor | | 3,86E-02 | FOS |
| PGK1 | kinase | | 3,86E-02 | VIM |
| CAPNS1 | peptidase | | 3,86E-02 | CCND1 |
| MS4A1 | other | | 3,86E-02 | MYC |
| GLP1R | G-protein coupled receptor | | 3,86E-02 | FOS |
| KLKB1 | peptidase | | 3,86E-02 | ICAM1 |
| NPAS2 | transcription regulator | | 3,86E-02 | PER1 |
| FUBP1 | transcription regulator | | 3,86E-02 | MYC |
| H4C3 | other | | 3,86E-02 | AFP |
| GNMT | enzyme | | 3,86E-02 | CYP1A1 |
| TNIK | kinase | | 3,86E-02 | CCND1 |
| PILRA | other | | 3,86E-02 | CXCL2 |
| Pde4d | enzyme | | 3,86E-02 | CTSV |
| RHOC | enzyme | | 3,86E-02 | ATF3 |
| VGF | growth factor | | 3,86E-02 | FOS |
| 15-E2-isoketal modified phosphatidylethanolamine | chemical reagent | | 3,86E-02 | ICAM1 |
| N-methyl mesoporphyrin IX | chemical reagent | | 3,86E-02 | MYC |
| Gm26313 | other | | 3,86E-02 | PLK2 |
| perifosine | chemical drug | | 3,86E-02 | TNFRSF10B |
| dimercaptopropanol | chemical drug | | 3,86E-02 | CXCL2 |
| aminoglutethimide | chemical drug | | 3,86E-02 | LDLR |
| trimethyltin | chemical reagent | | 3,86E-02 | FOS |
| 4-coumaric acid | chemical - endogenous mammalian | | 3,86E-02 | MYC |
| fluphenazine | chemical drug | | 3,86E-02 | FOS |
| 2-mercaptoethanol | chemical - endogenous mammalian | | 3,86E-02 | ATF3 |
| anethole | chemical - endogenous non-mammalian | | 3,86E-02 | PLAU |
| CHS-828 | chemical drug | | 3,86E-02 | ALDH1A1 |
| bergamottin | chemical - endogenous non-mammalian | | 3,86E-02 | CYP1A1 |
| polyphosphate | chemical - endogenous mammalian | | 3,86E-02 | ICAM1 |
| ferric chloride | chemical toxicant | | 3,86E-02 | PLAU |
| retroinverso ERG inhibitory peptide 2 | chemical reagent | | 3,86E-02 | NDRG1 |
| retroinverso ERG inhibitory peptide 1 | chemical reagent | | 3,86E-02 | NDRG1 |
| phytanic acid | chemical - endogenous mammalian | | 3,86E-02 | FABP1 |
| atropine | chemical drug | | 3,86E-02 | FOS |
| phosphatidic acid | chemical - endogenous mammalian | | 3,86E-02 | MYC |
| non-esterified fatty acid | chemical - endogenous mammalian | | 3,86E-02 | LDLR |
| 12-hydroxyeicosatetraenoic acid | chemical - endogenous mammalian | | 3,86E-02 | ICAM1 |
| acolbifene | chemical drug | | 3,86E-02 | LDLR |
| vinpocetine | chemical drug | | 3,86E-02 | ICAM1 |
| arsenic trioxide | chemical drug | | 3,88E-02 | CCND1,MYC,NFKBIA,TNFAIP3,TNFRSF10B |
| docetaxel | chemical drug | | 3,89E-02 | ITGA2,PLAU,TNFRSF10B |
| miR-6764-3p (and other miRNAs w/seed CUCUGGU) | mature microRNA | | 3,91E-02 | ITGA6,POLR2J2/POLR2J3,SERPINA6,SMIM14,TIPARP,ZNF608,ZNF627,ZNF844 |
| imatinib | chemical drug | | 3,92E-02 | CCND1,MDM2,MYC,TNFAIP3,TNFRSF10B |
| dorsomorphin | chemical - kinase inhibitor | | 3,94E-02 | EGR1,FABP1 |
| BIRC5 | other | | 3,94E-02 | MDM2,VIM |
| mir-24 | microRNA | | 3,94E-02 | MYC,SERPINE1 |
| CBL | transcription regulator | | 3,94E-02 | CXCL2,FOS |
| E2F4 | transcription regulator | | 3,96E-02 | CCND1,H3C4,MYC,SERPINE1 |
| miR-5190 (miRNAs w/seed CAGUGAC) | mature microRNA | | 4,03E-02 | AFAP1,B4GALNT2,BCL10,DCBLD2,SLC25A25,SMIM14,ZNF608 |
| miR-614 (miRNAs w/seed AACGCCU) | mature microRNA | | 4,04E-02 | KLF10,MDM2,TBX3 |
| IL32 | cytokine | | 4,07E-02 | ICAM1,VIM |
| EFNA2 | kinase | | 4,07E-02 | DCBLD2,DUSP6 |
| Pln | other | | 4,07E-02 | AHSG,VIM |
| miR-4757-5p (and other miRNAs w/seed GGCCUCU) | mature microRNA | Inhibited | 4,08E-02 | AFAP1,CXCL2,DYRK1A,GEM,GJB1,H3C14,H3C15,IER2,KLF6,POLR2J2/POLR2J3 |
| miR-5088-5p (miRNAs w/seed AGGGCUC) | mature microRNA | Inhibited | 4,10E-02 | CSRNP1,ETS1,IER3,IGFBP4,KRT6A,MXD4,SPATA2,TNS4,ZC3H12A,ZNF627 |
| WBP2 | transcription regulator | | 4,11E-02 | CXCL2,EGR1,JUN |
| miR-4691-5p (and other miRNAs w/seed UCCUCCA) | mature microRNA | Inhibited | 4,12E-02 | BCL10,CCND1,CSRNP1,CTSV,EMP1,FABP1,GJB1,PLK2,RGS2,SLC25A25,SPRED1,SPRY4,TENT5A,ZNF627 |
| miR-548at-5p (miRNAs w/seed AAAGUUA) | mature microRNA | | 4,14E-02 | ARRDC3,BIRC3,MYC,SERPINA6,SPRED1 |
| miR-4717-3p (miRNAs w/seed CACAUGG) | mature microRNA | | 4,16E-02 | BIRC3,DUSP6,FOS,PDZK1,SMIM14,ZNF441,ZNF627 |
| mir-204 | microRNA | | 4,20E-02 | CXCL2,MYC |
| ELL2 | transcription regulator | | 4,20E-02 | MDM2,MYC |
| APOA1 | transporter | | 4,20E-02 | ATF3,ICAM1 |
| Gcg | other | | 4,20E-02 | FOS,OAT |
| miR-369-5p (miRNAs w/seed GAUCGAC) | mature microRNA | | 4,27E-02 | ETS1,GJB1,ZNF627 |
| miR-18a-5p (and other miRNAs w/seed AAGGUGC) | mature microRNA | Inhibited | 4,28E-02 | CCND1,FGG,ITGA2,KLF6,PARD6B,TNFAIP3,ZNF627,ZNF844 |
| miR-296-3p (miRNAs w/seed AGGGUUG) | mature microRNA | Inhibited | 4,32E-02 | ICAM1,JUNB,NR1D1,ZBTB20,ZNF627,ZNF844 |
| miR-292b-5p (and other miRNAs w/seed CUCAAAA) | mature microRNA | | 4,32E-02 | ANKRD1,BCL10,FABP1,POLR2J2/POLR2J3,SERINC5,SMIM14 |
| 1810019D21Rik | other | | 4,33E-02 | ITGA2,PLAU |
| telmisartan | chemical drug | | 4,33E-02 | AGT,ICAM1 |
| pravastatin | chemical drug | | 4,33E-02 | ICAM1,LDLR |
| IL12 (family) | group | | 4,34E-02 | ATF3,MYC,NFKBIZ |
| miR-4717-5p (and other miRNAs w/seed AGGCCAC) | mature microRNA | | 4,35E-02 | ARRDC3,BCL10,EPHX2,ETS1,PLK2,RGS2,SMIM14,TNFAIP3,ZBTB20,ZNF555 |
| miR-4474-5p (miRNAs w/seed UAGUCUC) | mature microRNA | | 4,36E-02 | BCL10,ETS1,MXD1,SERINC5,TBX3,ZNF441 |
| miR-31-5p (and other miRNAs w/seed GGCAAGA) | mature microRNA | | 4,39E-02 | AGT,DCBLD2,DYRK1A,EMP1,H2BC5,KRT6A,NFKBIZ,PRR15L,SEPHS2,SPRY4,ZNF554 |
| 15-epi-lipoxin A4 | chemical - endogenous mammalian | | 4,40E-02 | TNFAIP3 |
| UDP | chemical - endogenous mammalian | | 4,40E-02 | SERPINE1 |
| L-phenylalanine | chemical - endogenous mammalian | | 4,40E-02 | TTR |
| ganglioside GM3 | chemical - endogenous mammalian | | 4,40E-02 | ICAM1 |
| heparan sulfate | chemical - endogenous mammalian | | 4,40E-02 | CXCL2 |
| N-Cadherin | group | | 4,40E-02 | CCND1 |
| meldonium | chemical drug | | 4,40E-02 | LDLR |
| fingolimod phosphate | chemical - endogenous mammalian | | 4,40E-02 | SERPINE1 |
| rhodamine 6G | chemical toxicant | | 4,40E-02 | CCND1 |
| 2,5-bis(5-hydroxymethyl-2-thienyl)furan | chemical reagent | | 4,40E-02 | MDM2 |
| 2-amino-5-azotoluene | chemical toxicant | | 4,40E-02 | MYC |
| citrinin | chemical toxicant | | 4,40E-02 | JUNB |
| Cyclin A | group | | 4,40E-02 | MYC |
| tolfenamic acid | chemical drug | | 4,40E-02 | ATF3 |
| TFIIA | complex | | 4,40E-02 | FOS |
| DBH-AS1 | other | | 4,40E-02 | CCND1 |
| UCA1 | other | | 4,40E-02 | CCND1 |
| LINC00461 | other | | 4,40E-02 | CCND1 |
| Retinoic acid-RAR-RXR | complex | | 4,40E-02 | DUSP1 |
| MRE11 | enzyme | | 4,40E-02 | MYC |
| 1,3-bis(4-hydroxyphenyl)-4-methyl-5-[4-(2-piperidinylethoxy)phenol]-1H-pyrazole | chemical reagent | | 4,40E-02 | CCND1 |
| tectorigenin | chemical - endogenous non-mammalian | | 4,40E-02 | ICAM1 |
| VTCN1 | other | | 4,40E-02 | CCND1 |
| CYTOR | other | | 4,40E-02 | MYC |
| SENP7 | peptidase | | 4,40E-02 | VIM |
| PON2 | enzyme | | 4,40E-02 | CXCL2 |
| NELFCD | other | | 4,40E-02 | JUNB |
| DUOXA1 | other | | 4,40E-02 | VIM |
| SULT2B1 | enzyme | | 4,40E-02 | MYC |
| ZNF382 | transcription regulator | | 4,40E-02 | MYC |

| | | | | |
|---|---|---|---|---|
| LTBP4 | growth factor | | 4,40E-02 | MYC |
| CYSLTR2 | G-protein coupled receptor | | 4,40E-02 | EGR1 |
| IL-2R | complex | | 4,40E-02 | FOS |
| Lefty | group | | 4,40E-02 | ETS1 |
| PDS5B | other | | 4,40E-02 | MYC |
| IL26 | cytokine | | 4,40E-02 | ICAM1 |
| STAG1 | other | | 4,40E-02 | MYC |
| parathyroid hormone-related protein | biologic drug | | 4,40E-02 | FOS |
| hydrogel | chemical drug | | 4,40E-02 | VIM |
| HDAC10 | transcription regulator | | 4,40E-02 | TXNIP |
| evofosfamide | chemical drug | | 4,40E-02 | CCND1 |
| BAG6 | enzyme | | 4,40E-02 | SERPINE1 |
| FANCA | other | | 4,40E-02 | EGR1 |
| HNRNPC | other | | 4,40E-02 | MYC |
| SLC25A4 | transporter | | 4,40E-02 | BIRC3 |
| MAP3K12 | kinase | | 4,40E-02 | JUN |
| PLCD1 | enzyme | | 4,40E-02 | VIM |
| S100a7a | other | | 4,40E-02 | CCND1 |
| CXCR1 | G-protein coupled receptor | | 4,40E-02 | CCND1 |
| mir-326 | microRNA | | 4,40E-02 | ETS1 |
| SMARCA1 | transcription regulator | | 4,40E-02 | MYC |
| RGS19 | enzyme | | 4,40E-02 | MYC |
| GAL3ST1 | enzyme | | 4,40E-02 | ITGA6 |
| CYSLTR1 | G-protein coupled receptor | | 4,40E-02 | FOS |
| FEM1A | transcription regulator | | 4,40E-02 | CXCL2 |
| ponesimod | chemical drug | | 4,40E-02 | SERPINE1 |
| CYP4A11 | enzyme | | 4,40E-02 | AGT |
| DLG4 | kinase | | 4,40E-02 | FOS |
| KSR1 | kinase | | 4,40E-02 | MYC |
| USP2 | peptidase | | 4,40E-02 | SERPINE1 |
| MAOA | enzyme | | 4,40E-02 | CCND1 |
| MFGE8 | other | | 4,40E-02 | CCND1 |
| DES | other | | 4,40E-02 | ANKRD1 |
| APCS | other | | 4,40E-02 | LDLR |
| DGKZ | kinase | | 4,40E-02 | CCND1 |
| RBM38 | other | | 4,40E-02 | MDM2 |
| ATF7 | transcription regulator | | 4,40E-02 | DUSP1 |
| CSNK2A2 | kinase | | 4,40E-02 | ITGA6 |
| PLP2 | transporter | | 4,40E-02 | ICAM1 |
| LASP1 | transporter | | 4,40E-02 | VIM |
| RASSF10 | other | | 4,40E-02 | JUN |
| GRB10 | other | | 4,40E-02 | FOS |
| TBL1X | transcription regulator | | 4,40E-02 | CXCL2 |
| NEU3 | enzyme | | 4,40E-02 | EGR1 |
| AQP7 | transporter | | 4,40E-02 | MYC |
| PIWIL2 | enzyme | | 4,40E-02 | CCND1 |
| RGS16 | enzyme | | 4,40E-02 | FABP1 |
| TSPO | transmembrane receptor | | 4,40E-02 | ICAM1 |
| SULF1 | enzyme | | 4,40E-02 | CCND1 |
| CCDC80 | other | | 4,40E-02 | CCND1 |
| GALP | other | | 4,40E-02 | FOS |
| farnesyl transferase | complex | | 4,40E-02 | KLF6 |
| teasaponin | chemical - endogenous non-mammalian | | 4,40E-02 | NFKBIA |
| DIABLO | other | | 4,40E-02 | BIRC3 |
| ACTA2 | other | | 4,40E-02 | VIM |
| CCL7 | cytokine | | 4,40E-02 | ICAM1 |
| H3C6 | other | | 4,40E-02 | VGF |
| VIPR2 | G-protein coupled receptor | | 4,40E-02 | JUNB |
| ethyl 2-{[(6,7-dimethyl-3-oxo-1,2,3,4-tetrahydro-2-quinoxalinyl)acetyl]amino}-4,5-dimethyl-3-thiophenecarboxylate | chemical reagent | | 4,40E-02 | MDM2 |
| GADD45GIP1 | other | | 4,40E-02 | GSTA2 |
| PCBP1 | translation regulator | | 4,40E-02 | MYC |
| TBL1XR1 | transcription regulator | | 4,40E-02 | CXCL2 |
| proteasome inhibitor | chemical drug | | 4,40E-02 | MYC |
| intralipid | chemical drug | | 4,40E-02 | FOS |
| naproxen | chemical drug | | 4,40E-02 | CCND1 |
| raclopride | chemical drug | | 4,40E-02 | FOS |
| BAY 61-3606 | chemical - kinase inhibitor | | 4,40E-02 | CXCL2 |
| itraconazole | chemical drug | | 4,40E-02 | CYP1A1 |
| NCX-4040 | chemical drug | | 4,40E-02 | CYP1A1 |
| coumestrol | chemical toxicant | | 4,40E-02 | FOS |
| GW 5074 | chemical - kinase inhibitor | | 4,40E-02 | SERPINE1 |
| mechlorethamine | chemical drug | | 4,40E-02 | ATF3 |
| domoic acid | chemical toxicant | | 4,40E-02 | FOS |
| pranlukast | chemical drug | | 4,40E-02 | EGR1 |
| myxothiazol | chemical toxicant | | 4,40E-02 | TXNIP |
| vandetanib | chemical drug | | 4,40E-02 | CCND1 |
| citalopram | chemical drug | | 4,40E-02 | FOS |
| rabeprazole | chemical drug | | 4,40E-02 | ICAM1 |
| venlafaxine | chemical drug | | 4,40E-02 | FOS |
| carnosol | chemical - endogenous non-mammalian | | 4,40E-02 | ICAM1 |
| AGI-1067 | chemical drug | | 4,40E-02 | EGR1 |
| 5-nitro-2-(3-phenylpropylamino)benzoic acid | chemical reagent | | 4,40E-02 | CCND1 |
| magnesium sulfate | chemical drug | | 4,40E-02 | NFKBIA |
| N-methyl-3,4-methylenedioxyamphetamine | chemical drug | | 4,40E-02 | FOS |
| tyrphostin | chemical drug | | 4,40E-02 | CXCL2 |
| dehydrocostus lactone | chemical - endogenous non-mammalian | | 4,40E-02 | MYC |
| palmitoyl-Cys((RS)-2,3-di(palmitoyloxy)-propyl)-Ala-Gly-OH | chemical reagent | | 4,40E-02 | ICAM1 |
| dihematoporphyrin ether | chemical drug | | 4,40E-02 | DUSP1 |
| theaflavin | chemical - endogenous non-mammalian | | 4,40E-02 | ICAM1 |
| pyrogallol | chemical toxicant | | 4,40E-02 | JUN |
| alpha-tocopherol succinate | chemical drug | | 4,40E-02 | EGR1 |
| miR-4757-3p (miRNAs w/seed AUGACGU) | mature microRNA | | 4,40E-02 | ANKRD1,LCA5,MXD4,SPRED1 |
| miR-3124-5p (miRNAs w/seed UCGCGGG) | mature microRNA | | 4,42E-02 | IER2,NUAK2,RHOB |
| miR-3654 (miRNAs w/seed ACUGGAC) | mature microRNA | | 4,45E-02 | FABP1,REEP6,SPATA2,TBX3,ZNF554 |
| S-nitroso-N-acetyl-DL-penicillamine | chemical reagent | | 4,46E-02 | LDLR,MYC |
| PPP2CA | phosphatase | | 4,46E-02 | CCND1,MYC |
| CHD4 | enzyme | | 4,46E-02 | JUN,MDM2 |
| capsaicin | chemical drug | | 4,46E-02 | CCND1,FOS |
| miR-3547 (and other miRNAs w/seed GAGCACC) | mature microRNA | | 4,50E-02 | AHSG,ASB2,ERRFI1,FABP1,PALD1,PRR15L,SLC25A25,ZFP36 |
| miR-7158-5p (miRNAs w/seed GCUCAAU) | mature microRNA | | 4,57E-02 | ERRFI1,ETS1,IER3,SEPHS2 |
| TET2 | enzyme | | 4,58E-02 | BHLHE40,ITGA2,MAFF |
| TNFRSF8 | transmembrane receptor | | 4,60E-02 | JUNB,MYC |
| HIC1 | transcription regulator | | 4,60E-02 | CCND1,EPHA2 |
| vitamin D | chemical drug | | 4,60E-02 | CYP1A1,NFKBIA |
| miR-504-5p (and other miRNAs w/seed GACCCUG) | mature microRNA | | 4,62E-02 | B4GALNT2,H3C14,H3C15,IER2,MDM2,PRR15L,SLC25A25,SPRY4,UBASH3B |
| miR-4781-3p (miRNAs w/seed AUGUUGG) | mature microRNA | | 4,64E-02 | FABP1,PARD6B,SPRED1,VIM,YOD1 |
| miR-6824-5p (miRNAs w/seed UAGGGGA) | mature microRNA | Inhibited | 4,65E-02 | H3C14,H3C15,IGFBP4,LDLR,NR1D1,POLR2J2/POLR2J3,SERINC5,ZNF442 |
| mir-223 | microRNA | | 4,66E-02 | CCND1,EMP1,ICAM1 |
| miR-3176 (and other miRNAs w/seed CUGGCCU) | mature microRNA | | 4,66E-02 | AKR1C3,ATF3,CCND1,ETS1,H3C14,H3C15,PALD1,POLR2J2/POLR2J3,SEPHS2,SMIM14 |
| miR-16-5p (and other miRNAs w/seed AGCAGCA) | mature microRNA | Inhibited | 4,67E-02 | ABL2,AFAP1,AKAP12,CCND1,CSRNP1,FABP1,ITGA2,JUN,LCA5,NUAK2,PARD6B,PMM1,PRR15L,SPRED1,TSC22D2,ZBTB43 |
| miR-4761-5p (miRNAs w/seed CAAGGUG) | mature microRNA | | 4,67E-02 | AXL,KLF6,MXD1,NFKBIZ,POLR2J2/POLR2J3,SERINC5,SMIM14 |
| miR-187-3p (miRNAs w/seed CGUGUCU) | mature microRNA | Inhibited | 4,69E-02 | ABL2,ARRDC3,BCL10,ETS1,NFKBIZ |
| miR-1893 (and other miRNAs w/seed GCGCGGG) | mature microRNA | Inhibited | 4,73E-02 | H3C14,H3C15,JUN,MXD1,VGF |
| miR-6512-3p (and other miRNAs w/seed UCCAGCC) | mature microRNA | Inhibited | 4,74E-02 | ANKRD1,ATF3,CYP1A1,DUSP6,FABP1,KLF10,KLF6,MDM2,NUAK2,PI3,PLK2 |
| Smad2/3 | group | | 4,74E-02 | MXD1,SERPINE1 |
| E2F6 | transcription regulator | | 4,74E-02 | MYC,SERPINE1 |
| C5AR1 | G-protein coupled receptor | | 4,74E-02 | CXCL2,SERPINE1 |
| RHO | G-protein coupled receptor | | 4,74E-02 | FOS,TNFAIP3 |
| seocalcitol | chemical drug | | 4,74E-02 | ANKRD1,MYC |
| miR-6084 (miRNAs w/seed UCCGCCA) | mature microRNA | Inhibited | 4,78E-02 | ABL2,AFAP1,ETS1,H3C14,H3C15 |
| miR-548aj-5p (and other miRNAs w/seed GCAAAAG) | mature microRNA | | 4,79E-02 | ALDH1A1,DUSP6,KLF10,MXD1,PRR15L,SPRED1 |
| miR-187-5p (miRNAs w/seed GCUACAA) | mature microRNA | | 4,83E-02 | ALDH1A1,ETS1,GEM,MXD1,ZFP36 |
| miR-377-3p (miRNAs w/seed UCACACA) | mature microRNA | | 4,85E-02 | AFP,ATF3,ETS1,ITGA6,LAMA1,MDM2,RGS2,SMIM14,VGF,ZNF627 |
| STING1 | other | | 4,87E-02 | CXCL2,PPP1R15A |
| NPY | other | | 4,87E-02 | FOS,PER1 |
| NRIP1 | transcription regulator | | 4,87E-02 | CCND1,CYP1A1 |
| BACH2 | transcription regulator | | 4,87E-02 | JUN,JUNB |
| miR-4762-5p (miRNAs w/seed CAAAUCU) | mature microRNA | | 4,88E-02 | KLF10,LCA5,MYC,SMIM14,TSPAN8 |
| miR-12202-3p (and other miRNAs w/seed CUUCUCU) | mature microRNA | Inhibited | 4,88E-02 | AFP,ANKRD1,BHLHE40,CCND1,LCA5,SERPINE1,TENT5A,ZNF844 |
| miR-3551-5p (and other miRNAs w/seed CUUCACC) | mature microRNA | | 4,88E-02 | ANKRD1,ARRDC3,B4GALNT2,SPATA2,SPRY4,TRRAP,ZNF627 |
| miR-5193 (miRNAs w/seed CCUCCUC) | mature microRNA | | 4,91E-02 | AFAP1,AHSG,ARRDC3,ATF3,CCND1,CSRNP1,EGR1,EMP1,FABP1,GJB1,NDRG1,PALD1,PRR15L |
| IFNA2 | cytokine | | 4,92E-02 | GEM,MDM2,MYC,TNFRSF10B |
| ganglioside | chemical - endogenous mammalian | | 4,93E-02 | ICAM1 |
| 16,16-dimethylprostaglandin E2 | chemical - endogenous mammalian | | 4,93E-02 | FOS |
| triciribine | chemical drug | | 4,93E-02 | ATF3 |
| L-lysine | chemical - endogenous mammalian | | 4,93E-02 | CXCL2 |
| geneticin | chemical toxicant | | 4,93E-02 | MDM2 |
| cholesterol sulfate | chemical - endogenous mammalian | | 4,93E-02 | NR1D1 |
| ganglioside GD3 | chemical - endogenous mammalian | | 4,93E-02 | ICAM1 |
| bazedoxifene | chemical drug | | 4,93E-02 | MYC |
| Pde | group | | 4,93E-02 | FOS |
| BMS-387032 | chemical drug | | 4,93E-02 | CCND1 |
| CRTC3 | other | | 4,93E-02 | RGS2 |
| HTR7 | G-protein coupled receptor | | 4,93E-02 | FOS |
| SNHG7 | other | | 4,93E-02 | VIM |
| FBXO42 | other | | 4,93E-02 | ICAM1 |
| AFF4 | transcription regulator | | 4,93E-02 | MYC |
| TACSTD2 | other | | 4,93E-02 | CCND1 |
| CPEB4 | other | | 4,93E-02 | ZBTB20 |
| KDM4B | enzyme | | 4,93E-02 | VIM |
| LRRC32 | other | | 4,93E-02 | VIM |
| HUWE1 | transcription regulator | | 4,93E-02 | MYC |
| TPSAB1/TPSB2 | peptidase | | 4,93E-02 | CXCL2 |
| tryptase | group | | 4,93E-02 | TNFAIP3 |
| nicotiflorin | chemical reagent | | 4,93E-02 | ICAM1 |
| PRRX1 | transcription regulator | | 4,93E-02 | FOS |
| UNC5B | transmembrane receptor | | 4,93E-02 | CXCL2 |
| AGTRAP | G-protein coupled receptor | | 4,93E-02 | FOS |
| AKAP12 | transporter | | 4,93E-02 | CCND1 |
| KHSRP | enzyme | | 4,93E-02 | LDLR |
| PDGF (family) | group | | 4,93E-02 | VIM |
| DLX1 | transcription regulator | | 4,93E-02 | SERPINE1 |
| FGF3 | growth factor | | 4,93E-02 | PLAU |
| CTNND1 | other | | 4,93E-02 | VIM |
| ZIC2 | transcription regulator | | 4,93E-02 | CCND1 |
| TPM3 | other | | 4,93E-02 | TNFRSF10B |
| PI3 | other | | 4,93E-02 | NFKBIA |
| AGRP | other | | 4,93E-02 | FOS |
| mir-873 | microRNA | | 4,93E-02 | TNFAIP3 |
| MED15 | transcription regulator | | 4,93E-02 | CCND1 |
| HGS | other | | 4,93E-02 | FOS |
| DKK2 | other | | 4,93E-02 | CCND1 |
| Ccl6 | cytokine | | 4,93E-02 | CTSV |
| GUSB | enzyme | | 4,93E-02 | CTSV |
| PRKCG | kinase | | 4,93E-02 | CCND1 |
| salinomycin | chemical - endogenous non-mammalian | | 4,93E-02 | CCND1 |
| genipin | chemical - endogenous non-mammalian | | 4,93E-02 | CCND1 |
| WNT5B | other | | 4,93E-02 | CCND1 |
| PTGES3 | enzyme | | 4,93E-02 | MYC |
| UBQLN1 | other | | 4,93E-02 | VIM |
| CDKN2B | transcription regulator | | 4,93E-02 | SERPINE1 |
| CCND2 | other | | 4,93E-02 | CCND1 |
| MSH2 | enzyme | | 4,93E-02 | CCND1 |
| RPL11 | other | | 4,93E-02 | MYC |
| NELFA | other | | 4,93E-02 | JUNB |
| DIAPH1 | other | | 4,93E-02 | EGR1 |
| EIF3A | other | | 4,93E-02 | NDRG1 |
| TSG101 | transcription regulator | | 4,93E-02 | MDM2 |
| USP37 | peptidase | | 4,93E-02 | MYC |
| PINK1 | kinase | | 4,93E-02 | CCND1 |
| GPR183 | G-protein coupled receptor | | 4,93E-02 | MYC |
| ST6GAL1 | enzyme | | 4,93E-02 | BIRC3 |
| COCH | other | | 4,93E-02 | CXCL2 |
| PPP1CA | phosphatase | | 4,93E-02 | FOS |
| HP | peptidase | | 4,93E-02 | FOS |
| TKT | enzyme | | 4,93E-02 | CCND1 |
| ANPEP | peptidase | | 4,93E-02 | NDRG1 |
| NELFE | other | | 4,93E-02 | JUNB |
| RECK | other | | 4,93E-02 | CCND1 |
| muscimol | chemical drug | | 4,93E-02 | FOS |
| 7,8-dihydro-7,8-dihydroxybenzo(a)pyrene 9,10-oxide | chemical toxicant | | 4,93E-02 | JUN |
| 1,1-bis(3'-indolyl)-1-(4-trifluoromethyl-phenyl)methane | chemical reagent | | 4,93E-02 | ATF3 |
| prodigiosin | chemical toxicant | | 4,93E-02 | TNFRSF10B |
| ketorolac | chemical drug | | 4,93E-02 | NFKBIA |
| miR-182 inhibitor | chemical reagent | | 4,93E-02 | NFKBIA |
| BQ 123 | biologic drug | | 4,93E-02 | CXCL2 |
| hexamethoxyflavone | chemical toxicant | | 4,93E-02 | ICAM1 |
| iodine | chemical - endogenous mammalian | | 4,93E-02 | ICAM1 |
| oleoylethanolamide | chemical - endogenous mammalian | | 4,93E-02 | FOS |
| 5-iodotubercidin | chemical - kinase inhibitor | | 4,93E-02 | CCND1 |
| lead acetate | chemical toxicant | | 4,93E-02 | CCND1 |
| miR-3667-3p (miRNAs w/seed CCUUCCU) | mature microRNA | | 4,94E-02 | ANKRD1,CTSV,ETS1,ICAM1,NDRG1,OAT,SELENOW,SMIM14,TENT5A,ZBTB43 |
| miR-29b-1-5p (miRNAs w/seed CUGGUUU) | mature microRNA | | 4,97E-02 | CTSV,NR1D1,NUAK2,SERPINE1,SPRED1 |
| HNRNPA2B1 | other | | 4,98E-02 | CTSV,MYC,UBASH3B |
| morphine | chemical drug | | 4,98E-02 | CCND1,FOS,JUNB |
| miR-378b (and other miRNAs w/seed UGGACUU) | mature microRNA | | 4,99E-02 | AXL,H3C14,H3C15,MXD1,NFKBIZ,PARD6B,TTR |

**Supplementary Table S16. List of Upstream Regulators for the condition "Caco-2_SARS-CoV".**

The IPA-based Core Analysis was performed on the basis of differentially expressed genes disclosed for a given condition by GENEVESTIGATOR-based analysis. Only statistically significant Upstream Regulators are included (n = 3736). The data are taken as the basis for R-based visualization by Venn diagrams shown in Figure 5.

| Upstream Regulator | Molecule Type | Predicted Activation State | p-value of overlap | Target Molecules in Dataset |
|---|---|---|---|---|
| PDGF BB | complex | Activated | 8,77E-32 | [illegible] |
| U0126 | chemical - kinase inhibitor | Inhibited | 4,55E-31 | [illegible] |
| PD98059 | chemical - kinase inhibitor | Inhibited | 2,14E-26 | [illegible] |
| beta-estradiol | chemical - endogenous mammalian | Activated | 6,76E-26 | [illegible] |
| TP63 | transcription regulator | Activated | 8,21E-26 | [illegible] |
| EGF | growth factor | Activated | 8,80E-25 | [illegible] |
| dexamethasone | chemical drug | Inhibited | 2,01E-24 | [illegible] |
| IL1B | cytokine | Activated | 3,96E-24 | [illegible] |
| TNF | cytokine | Activated | 4,18E-24 | [illegible] |
| TGFB1 | growth factor | Activated | 6,40E-24 | [illegible] |
| NFkB (complex) | complex | Activated | 2,08E-22 | [illegible] |
| Mek | group | Activated | 3,20E-22 | [illegible] |
| HGF | growth factor | Activated | 8,45E-22 | [illegible] |
| NRG1 | growth factor | Activated | 1,04E-21 | [illegible] |
| ERK | group | Activated | 1,27E-21 | [illegible] |
| tetradecanoylphorbol acetate | chemical drug | Activated | 3,61E-21 | [illegible] |
| paclitaxel | chemical drug | | 2,77E-20 | [illegible] |
| NR3C1 | ligand-dependent nuclear receptor | | 3,51E-20 | [illegible] |
| TP53 | transcription regulator | Activated | 1,35E-19 | [illegible] |
| HRAS | enzyme | | 2,00E-19 | [illegible] |
| Vegf | group | Activated | 9,17E-19 | [illegible] |
| IL2 | cytokine | Activated | 2,29E-18 | [illegible] |
| ESR1 | ligand-dependent nuclear receptor | | 2,79E-18 | [illegible] |
| CG | complex | Activated | 1,11E-17 | [illegible] |
| F2 | peptidase | Activated | 1,14E-17 | [illegible] |
| Raf | group | | 1,33E-17 | [illegible] |
| Salmonella enterica serotype abortus equi lipopolysaccharide | chemical toxicant | Activated | 1,61E-17 | [illegible] |
| IGF2 | growth factor | | 1,50E-17 | [illegible] |
| IGF1 | growth factor | Activated | 2,04E-17 | [illegible] |
| 4-hydroxytamoxifen | chemical drug | Activated | 2,34E-17 | [illegible] |
| [illegible] | [illegible] | [illegible] | [illegible] | [illegible] |

| | | | | |
|---|---|---|---|---|
| methylnitronitrosoguanidine | chemical toxicant | Activated | 1.08E-06 | CCND1,CEBPA,EGR1,GADD45A,IRF1,KLF10,MDM2,SERPINE1 |
| fludarabine | chemical drug | | 1.08E-06 | CCND1,CXCL2,DDB2,IRF1,MCL1,MDM2,TNFRSF10A,TNFRSF10B |
| IL33 | cytokine | Activated | 1.11E-06 | AREG,ARRB1,BIRC3,CCND1,CISH,CXCL2,CXCL8,DUSP2,EGR2,EPHX2,G0S2,GADD45B,ICAM1,ITGA5,JUNB,NAB1,NFKB2,NFKBIE,NFKBIZ,PDGFB,RELB,SLC7A11,SOCS3,TNFAIP3,ZC3H12A |
| methylselenic acid | chemical reagent | Inhibited | 1.11E-06 | ADD3,ATF3,BIRC3,CCND1,CDC14B,CFLAR,FOXA1,GADD45A,ID1,IRF1,JUNB,KLF4,MCL1,MXD1,MYC,NDRG1,RHOB,RND3,TMPRSS2,TNFAIP3,UGDH,ULBP2,ZNF165 |
| hydrocortisone | chemical - endogenous mammalian | | 1.13E-06 | ACHE,ANKRD1,CCND1,CEBPA,CXCL8,CXCR4,GADD45B,GLUL,ICAM1,IGFBP4,INHBA,ITGA2,PTGS2,SERPINE1,STC2,TNFAIP3,VIM |
| S100A8 | other | | 1.19E-06 | ACSS1,AKAP12,ALDH1A1,AREG,ATF3,BCL2,BHLHE40,CCND1,CXCL2,CXCL8,EFNA1,FOSL1,GPRC5A,ICAM1,MMP1,PHLDA1,PLK3,SOCS3,TXNIP |
| CARM1 | transcription regulator | Activated | 1.21E-06 | C3,CDK2,EPHX2,FOS,GADD45A,ICAM1,IGFBP4,MYC,STC2 |
| CEBPA | transcription regulator | | 1.23E-06 | ACSL1,ADH6,AKAP12,AKR1C3,BCL2,C3,CCND1,CEBPA,CXCL8,CXCR4,DDX21,EFNB2,EGR2,FOS,G0S2,GADD45A,ICAM1,ID1,IER3,IGFBP6,JUNB,KLF4,KRT17,MYC,MYCN,PTGS2,RGS2,RIN1,SERPINE1,SOCS3,THRA,TNFRSF10A,TRIB1 |
| ERBB4 | kinase | | 1.26E-06 | CCND1,DUSP1,EFNB2,EGR1,GADD45A,GPC1,IGF2,IGFBP4,MYC,PTGS2,RASA3,SLC2A1,SOCS3 |
| FGF19 | growth factor | | 1.26E-06 | ACSL1,ALDH1A1,AREG,CCND1,CXCL2,EGR1,FBP1,FOS,G0S2,GADD45A,GAS2,ID1,RGS2 |
| KLF2 | transcription regulator | | 1.30E-06 | CEBPA,COL4A1,CXCL2,CXCL8,CXCR4,EDN1,EFNA1,GADD45A,GATA4,ID1,IGF2,JUNB,MYC,NDRG1,NOTCH3,PTGS2,SERPINE1,SLC2A1,UGDH |
| SIN3A | transcription regulator | | 1.32E-06 | ALDH1A1,BCL2,CYP1A1,GADD45B,KLF10,KLF4,MYC,PMM1,PTGS2,SERPINE1,TXNIP |
| pyruvic acid | chemical - endogenous mammalian | | 1.33E-06 | BCL2,EPO,FOS,NR4A1,TNFRSF10A,TNFRSF10B |
| Nfat (family) | group | Activated | 1.43E-06 | ATF3,BCL2,CCND1,CFLAR,CXCL8,EGR2,ICAM1,KLF6,PDGFB,PLPP1,PTGS2,RELB,SERPINE1,TUBB3 |
| E2F1 | transcription regulator | | 1.44E-06 | ACSL5,BCL2,CASP6,CCND1,CDKN2C,CEBPA,CFLAR,DDB2,DUSP1,DUSP6,EGR1,EGR2,EGR4,FGFR1,FGFR2,FOS,ICAM1,IGF2,MCL1,MDM2,MMP1,MYC,MYCN,PLK2,REV3L,RHOBTB2,RND3,SERPINE1,SERPINF2,SOAT2,TAP2,UGDH,ULK1,VIM,ZFP36 |
| bardoxolone methyl | chemical drug | | 1.45E-06 | BCL2,BIRC3,CCND1,CFLAR,MYC,PTGS2,TNFRSF10A,TNFRSF10B |
| NCOA3 | transcription regulator | | 1.48E-06 | ANKRD1,BCL2,CCND1,CEBPA,CYP1A1,FGFR2,HK2,IRF1,PARD6B,PTGS2,SLC16A3,SLC2A1,TAGLN |
| Tnf (family) | group | Activated | 1.50E-06 | ATF3,BCL2,BIRC3,CCND1,CFLAR,CXCL8,CYP1A1,ICAM1,INHBA,MYC,PDGFB,PHEX,PLEK2,PTGS2,SERPINE1,SOCS3,TNFAIP3 |
| ENG | transmembrane receptor | | 1.52E-06 | CXCR4,ID1,ITGA5,ITGA6,MMP1,MYC,PAK1,PDGFB,SERPINE1 |
| CXCL12 | cytokine | Activated | 1.58E-06 | AKR1C3,BCL2,CCND1,CXCL8,CXCR4,EGR1,FOS,GAS2,ICAM1,ID1,JUNB,MAPK,MMP11,MYC,NR4A1,PTGS2,SOCS3,TNFRSF10A,TNFRSF10B,VIM |
| PF4 | cytokine | | 1.60E-06 | BIRC3,CEBPA,CXCL8,EPSTI1,ICAM1,INHBA,IRF1,KLF4,MYCN,NFKB2,NFKBIZ,TNFAIP3 |
| miR-1273h-5p (and other miRNAs w/seed UGGGAGG) | mature microRNA | Activated | 1.63E-06 | ABHD4,ACADSB,ALDH3B2,AMER3,ARRB1,APOBEC3C,APOL1,ARPIN,ARPIN-AP3S2,ASDMT,BAZ2B,BCL10,BCL2,[illegible] |
| APP | other | Activated | 1.71E-06 | ACHE,ARRB1,ARRDC3,BCL2,BRIPF1,C3,CCND1,CFLAR,CRYL1,CXCL2,CXCL8,CXCR4,EDN1,EGR1,[illegible] |
| napabucasin | chemical drug | | 1.80E-06 | ALDH1A1,CXCL8,FGFR2,FOXP1,ID1,ITGA2,ITGA6,KLF4,LATS1,MYC,PLAUR |
| wortmannin | chemical - kinase inhibitor | Inhibited | 1.85E-06 | ARRB1,ATF3,BCL2,CCND1,CFLAR,CXCL8,EGR1,ERBB3,FAM3B,FOS,GUCY2C,ICAM1,IRF1,MCL1,MYC,NR4A1,PTGS2,SERPINE1,SOCS3,VIM |
| carrageenan | chemical drug | Activated | 1.85E-06 | BCL10,CXCL2,CXCL8,EGR1,EGR2,FOS,FOSL1,ICAM1,JUNB,PTGS2 |
| [illegible] | other | Activated | 1.89E-06 | BHLHE40,COL4A1,FSTL3,GADD45B,IGFBP4,JUNB,RHOB,TAGLN,TNFRSF12A |
| [illegible] | group | | 1.89E-06 | BCL2,CFLAR,EDN1,NFKBIZ,PLAUR,PPP1R15A,PTGS2,SLC7A11,TNFAIP3 |
| benzyl isothiocyanate | chemical - endogenous non-mammalian | | 1.89E-06 | BCL2,CCND1,FOS,ICAM1,MCL1,[illegible],MYC,PTGS2,SERPINE1 |
| APEX1 | enzyme | Activated | 1.91E-06 | EGR1,EGR2,EGR4,FOS,JUNB,NR4A1,PTGS2,RGS2 |
| BCR-ABL1 | fusion gene/product | | 1.91E-06 | BCL2,CCND1,CDKN2C,CEBPA,CXCR4,GAS2,HK2,ID1,MXD4,MYC,SLC2A1,TNFRSF10A,TNFRSF10B |
| [illegible] | mature microRNA | Activated | 1.96E-06 | [illegible] |
| POU2F1 | transcription regulator | | 1.96E-06 | ACSL1,CCND1,CDX2,CXCL8,CYP1A1,ELK3,GADD45A,GAS6,GSTK1,ICAM1,IDH1,SLC7A11,TMPRSS2,TNFAIP3,TNFRSF10A |
| JQ1 | chemical reagent | | 1.96E-06 | BCL2,BIRC3,CCND1,CFLAR,CFTR,CXCR4,FOS,MCL1,MVD,MYC,MYCN,NFKB2,SLC7A11,TNFAIP3,TNFRSF10B |
| S100A9 | other | Activated | 2.00E-06 | ACSS1,AKAP12,ALDH1A1,AREG,ATF3,BHLHE40,C3,CCND1,CXCL8,EFNA1,FOSL1,GPRC5A,ICAM1,MMP1,PHLDA1,PLK3,SOCS3,TXNIP |
| TCF4 | transcription regulator | | 2.11E-06 | AEN,BHLHE40,CCND1,CEBPA,[illegible] |
| PRKCE | kinase | Activated | 2.13E-06 | BCL2,BIRC3,CCND1,CXCL8,EGR1,FOS,JUNB,LDLR,MYC,PDCD4,PTGS2,RGS2 |
| [illegible] | [illegible] | [illegible] | [illegible] | [illegible] |

| | | | | |
|---|---|---|---|---|
| xanthohumol | chemical drug | | 7.46E-05 | BIRC3,CCND1,FOS,ICAM1,MYC,SRC,TNFRSF10B |
| CTF1 | cytokine | | 7.46E-05 | FOS,GATA4,IRF1,JUNB,MMP1,SOCS3,TAGLN |
| FOSB | transcription regulator | | 7.46E-05 | CCND1,CEBPA,FOS,FOSL1,INHBA,MMP1,SERPINE1 |
| ELF4 | transcription regulator | Activated | 7.46E-05 | CXCL2,CXCL8,DUSP1,DUSP6,KLF4,MDM2,PLEC |
| alvocidib | chemical drug | | 7.46E-05 | AEN,BCL2,BIRC3,CCND1,CFLAR,MCL1,RHOBTB2 |
| sphingosylphosphocholine | chemical - endogenous mammalian | | 7.49E-05 | DUSP6,FOS,ICAM1,TGM2 |
| MEMO1 | other | | 7.49E-05 | CCND1,FOS,MYC,STC2 |
| RTKN | other | | 7.49E-05 | BIRC3,FOS,TNFAIP3,VIM |
| I-BOP | chemical reagent | | 7.49E-05 | CCND1,ICAM1,MMP1,PTGS2 |
| NR5A2 | ligand-dependent nuclear receptor | | 7.50E-05 | BCL2,C3,CCND1,CYP27A1,FOS,GATM,GPT2,ICAM1,JUNB,LDLR,MYC,PLK3 |
| ACOX1 | enzyme | | 7.52E-05 | ACSL1,DIO1,EHHADH,GADD45A,HSD3B1,KLF6,OAT,PAH,PHLDA1,RGN,RGS2,SELENBP1,SLCO2B1,TNFRSF10A |
| GNA12 | enzyme | | 7.56E-05 | BCL2,CXCL8,FOS,ITGA6,MYC,PTGS2,TAGLN,TRPC6,VAV3,VIM |
| CCL5 | cytokine | Activated | 7.56E-05 | CXCL2,CXCL8,DUSP1,DUSP6,EMP1,FOS,IER2,PLAUR,PLEC,ZFP36 |
| NFkB1-RelA | complex | Activated | 7.61E-05 | CCND1,CXCL8,ICAM1,MMP1,MYC,PTGS2 |
| TAF4B | transcription regulator | | 7.61E-05 | IER3,INHBA,IRF1,ITGA6,TGFB3,TNFAIP3 |
| HMGB2 | transcription regulator | | 7.61E-05 | CCND1,CXCL2,CXCL8,INHBA,MMP1,PHLDA1 |
| AZGP1 | transporter | | 7.61E-05 | CCND1,EGR1,HK2,ITGA6,MYC,VIM |
| KN-62 | chemical - kinase inhibitor | | 7.61E-05 | ATF3,CCND1,CXCL8,EGR1,PPP1R15A,PTGS2 |
| benzo(a)pyrene | chemical toxicant | | 7.72E-05 | ABHD4,BCL2,CYP1A1,FOS,GNA11,GPC1,HK2,IER3,INHBA,MAFF,MAFG,MAP2K6,MDM2,MMP1,NR4A1,PTGS2,SELENBP1,SERPINE1 |
| carbon tetrachloride | chemical toxicant | Activated | 7.72E-05 | ATF3,CYP1A1,DUSP1,FGG,FOS,FOXA1,GADD45A,GADD45B,GSTK1,ICAM1,JUNB,MYC,NR4A1,PDGFB,PPP1R15A,PTGS2,RGN,TNFRSF12A |
| PPARA | ligand-dependent nuclear receptor | | 7.76E-05 | ACADS,ACSL1,ACSL5,BCL2,C3,CCND1,CDKN2C,CEBPA,CROT,CYP1A1,CYP27A1,DIO1,EDN1,EHHADH,FGA,FGG,FOS,[illegible],GLUL,GSTK1,HMGCS2,HSD3B1,MVD,MYC,NR1D1,PAH,PHLDA1,PTGS2,SELENBP1 |
| miR-6476 (and other miRNAs w/seed AGGAAGG) | mature microRNA | | 7.82E-05 | ABHD4,[illegible] |
| CpG oligonucleotide | chemical drug | | 7.85E-05 | BCL2,CXCL2,CXCL8,DUSP1,EGR1,FCGRT,ICAM1,IL18,MCL1,MYC,NFKBIZ,PHGDH,PTGS2,SERPINE1,SLC40A1 |
| SHH | peptidase | | 7.87E-05 | BCL2,CCND1,DLX2,FOXA1,GATA4,GPC1,HK2,ID1,IGF2,MYC,MYCN,NR4A1,SERPINE1,SOSTDC1,TGFB3,TUBB3 |
| acetaminophen | chemical drug | Activated | 7.87E-05 | AMACR,BCL2,CCND1,CXCL2,GADD45A,GJB1,GLUL,HMGCS2,ICAM1,IFRD1,IL18,MCL1,MYC,PLAUR,PTGS2,SOCS3 |
| miR-19b-3p (and other miRNAs w/seed GUGCAAA) | mature microRNA | Inhibited | 8.00E-05 | [illegible] |
| miR-199a-5p (and other miRNAs w/seed CCAGUGU) | mature microRNA | | 8.03E-05 | [illegible] |
| NLRX1 | other | Inhibited | 8.05E-05 | BCL10,BIRC3,IRF1,NFKB2,NFKBIE,RELB,SLC2A1,TNFAIP3 |
| PRKD1 | kinase | Activated | 8.05E-05 | CCND1,CFLAR,CXCL2,GATA4,MMP1,NR4A1,PTGS2,TNFAIP3 |
| IL27 | cytokine | Activated | 8.11E-05 | APOL1,CFLAR,CXCL8,EGR1,EGR2,FOS,ICAM1,IL18,IRF1,MCL1,MYC,PTGS2,SOCS3,VIM |
| losartan | chemical drug | | 8.11E-05 | ATF3,BCL2,CCND1,FGFR1,IRF1,KLF4,LDLR,LIF,MCL1,MVD,MYC,PTGS2,RHOB,SERPINE1 |
| TGFBR2 | kinase | | 8.14E-05 | BCL2,BHLHE40,CCND1,CXCL2,GADD45B,GATM,ICAM1,IHH,PDGFB,PTGS2,SERPINE1,SOCS3,TAGLN,TGFB3,TMPRSS2,TNFRSF12A,TXNIP |
| GPS2 | transcription regulator | | 8.26E-05 | CCND1,CXCL8,DHRS3,FAM20C,HIVEP2,ID1,MYC,RHOB,TBC1D2 |
| rotenone | chemical toxicant | | 8.29E-05 | BIRC3,CCND1,CFLAR,CXCL2,FOS,MMP1,PDGFB,PTGS2,TXNIP |
| HDAC5 | transcription regulator | | 8.43E-05 | ACSL1,EGR2,HK2,HMGCS2,KLF6,MYC,MYCN,NR4A1,TAGLN,TUBB3 |
| MAP3K7 | kinase | | 8.43E-05 | BIRC3,CXCL2,CXCL8,GATA4,IRF1,MMP1,PDGFB,PTGS2,RGS2,SERPINE1 |
| fenretinide | chemical drug | | 8.43E-05 | ANKRD1,ATF3,BCL2,CCND1,CFLAR,MYC,PPP1R15A,PTGS2,RXRA,TNFRSF10B |
| DSCAM | other | | 8.63E-05 | CEBPA,KLF4,L1CAM,MYC,NOTCH3,NTN4,PDGFB,PLXNC1,SLC29A4,TUBB3,VIM |
| KLF11 | transcription regulator | | 8.70E-05 | ACSL5,BCL2,CHRNB1,CXCR4,[illegible],GREM1,ITGA2,ITGA3,MMP1,MYC,PDGFB,SERPINE1,TGFB3 |
| NFkB (family) | group | | 8.82E-05 | CCND1,CFLAR,CXCL8,PTGS2,TNFAIP3 |
| Integrin | complex | Activated | 8.82E-05 | CCND1,FOS,MMP1,MYC,SOCS3 |
| PPP1R15B | phosphatase | | 8.82E-05 | BCL2,CCND1,CFLAR,FOS,MYC |
| CHD1 | enzyme | Activated | 8.82E-05 | CXCL2,ICAM1,IER3,LIF,PTGS2 |
| PPP2R5C | other | Inhibited | 8.82E-05 | BCL2,BIRC3,CXCL8,RELB,TNFAIP3 |
| DDX17 | enzyme | | 8.82E-05 | CCND1,FOSL1,MDM2,MYC,NFAT5 |
| premarin | chemical drug | | 8.82E-05 | AREG,BCL2,CXCL8,MYC,SERPINE1 |
| silicon dioxide | chemical drug | Activated | 8.82E-05 | CXCL8,EGR1,FOS,FOSL1,JUNB |
| TCF7L2 | transcription regulator | | 8.83E-05 | ACSL1,CCND1,CDK19,EHHADH,ERBB3,FA2H,FBP1,FGFR2,GAB1,GJB1,GLUL,HMGCS2,ID1,IDH1,LAMC2,MMP1,MYC,PIM3,PLEKHG3,RNASE4,SDC2,STK39,TMEM141 |
| TWIST2 | transcription regulator | | 8.92E-05 | CCND1,DUSP5,DUSP6,[illegible],KLF6,MYCN,VIM |
| NQO1 | enzyme | | 8.92E-05 | BCL2,BIRC3,CCND1,CEBPA,CFLAR,CXCR4,PTGS2 |
| PAK2 | kinase | | 8.92E-05 | DUSP1,ETS1,GADD45A,ID1,MDM2,PLAUR,TAGLN |
| SPP1 | cytokine | | 8.96E-05 | BCL2,CXCL2,CXCL8,FGFR2,FOS,ICAM1,ITGA2,ITGA3,LIF,MMP1,MYC,PTGS2,SELENOW,SERPINE1,[illegible],VIM |
| [illegible] | chemical drug | | 9.01E-05 | AMACR,C3,CASP6,CCND1,EHHADH,FGA,GADD45A,GJB1,HMGCS2,ID1,IER3,IFRD1,MYC,SELENBP1,TNFRSF12A |
| miR-1343-3p (and other miRNAs w/seed [illegible]) | mature microRNA | | 9.06E-05 | [illegible] |
| miR-6806-5p (miRNAs w/seed [illegible]) | mature microRNA | | 9.14E-05 | [illegible] |
| NPSR1 | G-protein coupled receptor | | 9.16E-05 | GADD45A,MYC,NR4A1 |
| HSPB6 | other | | 9.16E-05 | BCL2,CXCL8,ICAM1 |
| [illegible] | chemical - endogenous non-mammalian | | 9.16E-05 | DUSP6,PHLDA1,SPRY2 |
| ACP1 | phosphatase | | 9.16E-05 | EPHA2,FOS,MYC |
| [illegible] | chemical drug | | 9.16E-05 | EGR1,FOS,JUNB |
| [illegible] | chemical drug | | 9.16E-05 | ITGA2,ITGA3,ITGA6 |
| alpha-methylhydrocinnamic acid | chemical - endogenous mammalian | | 9.16E-05 | CISH,MCL1,MYC |
| DGCR8 | enzyme | | 9.31E-05 | CCND1,EGR1,EGR2,ERBB3,FOS,MYC,PDCD4,TAGLN |
| FGF21 | growth factor | | 9.31E-05 | EGR1,FGFR1,FGFR2,FOS,HMGCS2,LDLR,SERPINE1,SLC2A1 |
| TSC22D3 | transcription regulator | | 9.31E-05 | BCL2,CCND1,CEBPA,CXCL8,DUSP1,EGR2,ICAM1,PTGS2 |
| ADRA1A | G-protein coupled receptor | | 9.31E-05 | CYP1A1,DUSP1,EGR1,EGR2,FOS,LIF,NR4A1,PAK1 |
| BCL2L1 | other | | 9.36E-05 | BCL2,CFLAR,CXCL8,EPO,ITGA3,MCL1,TNFRSF10A,TNFRSF10B,TUBB3 |
| miR-4267 (miRNAs w/seed [illegible]) | mature microRNA | | 9.46E-05 | [illegible] |
| [illegible] | other | | 9.55E-05 | ATF3,CCND1,CEBPA,FOS,MYC,SERPINE1 |
| WR 1065 | chemical drug | | 9.55E-05 | DDB2,GADD45A,MDM2,MYC,TNFRSF10B,ZFP36L1 |
| [illegible] | mature microRNA | | 9.62E-05 | [illegible] |
| [illegible] | [illegible] | [illegible] | [illegible] | [illegible] |

| | | | | |
|---|---|---|---|---|
| miR-6455 (miRNAs w/seed GGGUGUC) | mature microRNA | | 5,32E-04 | [illegible] |
| TO-901317 | chemical reagent | | 5,37E-04 | [illegible] |
| miR-6918-5p (and other miRNAs w/seed GCUGAGG) | mature microRNA | | 5,42E-04 | [illegible] |
| TLR7 | transmembrane receptor | | 5,49E-04 | ATF3,BCL2,CCND1,CHST9,CXCL2,CXCL8,FOSL1,GNG2,HIVEP2,ICAM1,IER3,IRF1,JUNB,SPRY4,TNFAIP3,ZC3H12A |
| miR-5010-3p (and other miRNAs w/seed UUUGUGU) | mature microRNA | | 5,57E-04 | [illegible] |
| ZBTB17 | transcription regulator | | 5,59E-04 | BCL2,BHLHE40,CCND1,DDB2,EGR1,IER3,LDLR,NOCT,ZFP36 |
| miR-1231-3p (miRNAs w/seed GCCCUGU) | mature microRNA | | 5,60E-04 | [illegible] |
| triamterene | chemical drug | Activated | 5,77E-04 | BIRC3,FGA,IER3,LAMC2,MYC,TNFRSF12A,UPP1,VIM |
| CBX5 | transcription regulator | | 5,85E-04 | AKR1C3,FOXA1,GCNT3,HOXB6,ITGA7,MUC13,PRR15L,QPRT,SELENBP1,TM4SF1,TXNIP |
| PIAS1 | transcription regulator | | 5,88E-04 | CCND1,CXCL8,GADD45A,MCL1,MDM2,TAGLN |
| histone deacetylase inhibitor | chemical drug | | 5,88E-04 | BCL2,MYC,PHLDA2,SLC9A3R1,TXNIP,ULBP2 |
| IL22 | cytokine | | 5,90E-04 | BCL2,C3,CCND1,CXCL2,CXCL8,FGA,KRT17,MCL1,MMP1,MYC,PTGS2,SOCS3 |
| TNFAIP3 | enzyme | | 5,93E-04 | BCL2,BIRC3,CFLAR,CXCL8,ICAM1,SOCS3,TNFAIP3 |
| miR-8088 (miRNAs w/seed CUCGGUA) | mature microRNA | | 5,93E-04 | [illegible] |
| miR-1285-3p (and other miRNAs w/seed CUGGGCA) | mature microRNA | Activated | 5,96E-04 | [illegible] |
| zinc | chemical drug | | 6,11E-04 | CCND1,IGF2,MAFG,MTF1,PTGS2,SELENBP1,SLC30A1,SLC30A7,TCN2 |
| miR-3064-3p (miRNAs w/seed UGCCACA) | mature microRNA | | 6,18E-04 | [illegible] |
| miR-6826-3p (miRNAs w/seed UCCCCUC) | mature microRNA | | 6,21E-04 | [illegible] |
| calcipotriene | chemical drug | | 6,27E-04 | CXCL8,FOS,FOSL1,JUNB,PTGS2 |
| NfkB-RelA | complex | | 6,27E-04 | BCL2,CXCL8,ICAM1,MYC,PTGS2 |
| BCL2L11 | other | | 6,27E-04 | BCL2,CCND1,CXCL2,HDAC9,MCL1 |
| LRPAP1 | other | | 6,27E-04 | CXCL8,LDLR,LRP1,PTGS2,SERPINE1 |
| kaempferol | chemical toxicant | | 6,27E-04 | BCL2,CYP1A1,ICAM1,MMP1,PTGS2 |
| UCN-01 | chemical drug | | 6,27E-04 | BCL2,CCND1,CXCL8,MCL1,RHOB |
| miR-7851-3p (miRNAs w/seed ACCUGGG) | mature microRNA | | 6,35E-04 | [illegible] |
| miR-6856-3p (miRNAs w/seed ACAGCCC) | mature microRNA | | 6,35E-04 | [illegible] |
| CIP2A | other | Inhibited | 6,39E-04 | BCL2,GADD45A,HK2,IER3,LAMA1,MYC,PLAUR,SLC16A3 |
| miR-3175 (miRNAs w/seed GGGGAGA) | mature microRNA | | 6,39E-04 | [illegible] |
| miR-6777-5p (and other miRNAs w/seed CGGGGAG) | mature microRNA | | 6,44E-04 | [illegible] |
| miR-6788-3p (miRNAs w/seed UCGCCAC) | mature microRNA | | 6,47E-04 | [illegible] |
| phenobarbital | chemical drug | | 6,68E-04 | BCL2,CCND1,CYP1A1,CYP27A1,DIO1,GADD45B,GSTA2,MDM2,SULT1B1 |
| CCN5 | growth factor | | 6,69E-04 | CEBPA,FOXA1,ITGA5,KLF4,LAMC2,SERPINE1,VIM |
| CP-55940 | chemical reagent | | 6,82E-04 | CXCL8,GADD45A,JUNB,MDM2,MYC,TNFAIP3 |
| STAT | group | Activated | 6,82E-04 | CISH,FOS,IRF1,JUNB,MMP1,SOCS3 |
| FGFR3 | kinase | Activated | 6,82E-04 | ANXA8,CCND1,DUSP6,FOS,HK2,IHH |
| mevalonic acid | chemical - endogenous mammalian | | 6,82E-04 | GNA11,ICAM1,LDLR,MMP1,PTGS2,SERPINE1 |
| tempol | chemical drug | Inhibited | 6,82E-04 | CXCL2,CXCL8,ICAM1,PLAUR,PTGS2,SERPINE1 |
| ethylene glycol tetraacetic acid | chemical reagent | | 6,82E-04 | BCL2,BIRC3,CXCL8,EGR1,FAM3B,FOS |
| medroxyprogesterone acetate | chemical drug | | 6,83E-04 | BCL2,CCND1,CXCR4,CYP1A1,DNMT3B,ETS1,[illegible] |
| miR-3173-5p (and other miRNAs w/seed GCCCUGC) | mature microRNA | | 6,85E-04 | [illegible] |
| miR-6729-3p (miRNAs w/seed CAUCCCC) | mature microRNA | | 6,85E-04 | [illegible] |
| TcF | transcription regulator | | 7,03E-04 | BCL2,CISH,CROT,EGR2,ELK3,GGT1,KLF6,[illegible] |
| TNFSF13B | cytokine | Activated | 7,06E-04 | BCL2,CXCL8,DUSP6,ICAM1,MCL1,MYC,[illegible] |
| 3,3'-diindolylmethane | chemical drug | | 7,06E-04 | ATF3,BCL2,CCND1,PTGS2,TAP2,TNFRSF10A,[illegible] |
| TCF/LEF | group | | 7,06E-04 | CCND1,MYC,PTGS2,VIM |
| N-carbobenzoxy-leucine-leucine-norvalinal | chemical - protease inhibitor | | 7,06E-04 | CFLAR,CXCL8,MCL1,RXRA |
| molybdenum disulfide | chemical reagent | | 7,16E-04 | AKR1C3,COL4A1,CXCL8,DCBLD2,DUSP6,[illegible] |
| miR-145-5p (and other miRNAs w/seed UCCAGUU) | mature microRNA | | 7,26E-04 | [illegible] |
| nicotinic acetylcholine receptor | complex | | 7,51E-04 | FOS,ID1,JUNB |
| SH2D5 | other | | 7,51E-04 | CCND1,MCL1,[illegible] |
| AKIRIN2 | other | | 7,51E-04 | CCND1,DUSP1,MYC |
| SLC5A8 | transporter | | 7,51E-04 | BCL2,TNFRSF10A,TNFRSF10B |
| TCIM | other | | 7,51E-04 | CXCL8,ICAM1,PTGS2 |
| sphingomyelinase | group | | 7,51E-04 | MMP1,MYC,PTGS2 |
| PDGFD | growth factor | | 7,51E-04 | CXCR4,MMP1,PTGS2 |
| idelalisib | chemical drug | | 7,51E-04 | MCL1,MYC,SLC2A1 |
| IER3 | other | | 7,51E-04 | BCL2,BIRC3,MCL1 |
| IFNGR | complex | | 7,51E-04 | IRF1,LAMA1,SOCS3 |
| DAB2 | other | | 7,51E-04 | CCND1,FOS,LRP2 |
| RBX1 | enzyme | | 7,51E-04 | BCL2,MCL1,RHOB |
| EGR4 | transcription regulator | | 7,51E-04 | EGR1,EGR2,MYBL1 |
| PDK1 | kinase | | 7,51E-04 | GSTK1,HK2,VIM |
| SF3B2 | other | | 7,51E-04 | HOXB3,HOXB6,HOXB8 |
| MED13L | other | | 7,51E-04 | ANKRD1,LIF,MYC |
| [illegible] | chemical - endogenous non-mammalian | | 7,51E-04 | BCL2,CFLAR,TNFRSF10B |
| [illegible] | other | | 7,51E-04 | MCL1,MYC,ZFP36L1 |
| PHC2 | other | | 7,51E-04 | HOXB3,HOXB6,HOXB8 |
| thenoyltrifluoroacetone | chemical reagent | | 7,51E-04 | BIRC3,CXCL8,SERPINE1 |
| perifosine | chemical drug | | 7,51E-04 | CFLAR,MCL1,TNFRSF10B |
| temsirolimus | chemical drug | | 7,51E-04 | CCND1,MYC,SLC2A1 |
| gliotoxin | chemical toxicant | | 7,51E-04 | BCL2,BIRC3,IER3 |
| cyclosporin | biologic drug | | 7,51E-04 | CCND1,CXCL8,ICAM1 |
| rutin | chemical toxicant | | 7,51E-04 | BCL2,PTGS2,SLCO2B1 |
| phosphatidic acid | chemical - endogenous mammalian | | 7,51E-04 | IGF2,MYC,PTGS2 |
| thyroid hormone receptor | group | | 7,58E-04 | EHHADH,FOS,KRT17,MMP11,MYC |
| MST1 | growth factor | | 7,58E-04 | CCND1,FOS,IL18,MST1R,PTGS2 |
| XIAP | enzyme | Activated | 7,58E-04 | CCND1,CXCL8,MYC,PTGS2,SERPINE1 |
| CD9 | other | | 7,58E-04 | ITGA2,ITGA3,ITGA5,ITGA6,MYC |
| BAG1 | other | | 7,58E-04 | BCL2,CCND1,FOS,GADD45A,PTGS2 |
| methyl-beta-cyclodextrin | chemical drug | | 7,58E-04 | CCND1,CXCL8,LDLR,MYC,SERPINE1 |
| IRF8 | transcription regulator | Activated | 7,62E-04 | ATF3,CEBPA,EGR1,EGR2,FOS,ICAM1,IL18,[illegible] |
| CD38 | enzyme | | 7,68E-04 | [illegible] |
| miR-96-5p (and other miRNAs w/seed UUGGCAC) | mature microRNA | | 7,77E-04 | [illegible] |
| MSTN | growth factor | | 7,79E-04 | FOS,IGF2,INHBA,KLF10,SLC2A1,SRC,ULK1,[illegible] |
| [illegible] | transcription regulator | | 7,79E-04 | AKR1C3,JUNB,KLHL21,PLAUR,[illegible] |
| [illegible] acid | chemical reagent | Activated | 7,79E-04 | BIRC3,FGA,IER3,LAMC2,MYC,TNFRSF12A,[illegible] |
| miR-[illegible] (miRNAs w/seed GUCCCAC) | mature microRNA | | 7,83E-04 | [illegible] |
| [illegible] | enzyme | | 7,87E-04 | CDKN2C,GAS6,NDRG1,PHGDH,SERPINE1,[illegible] |
| TFAP4 | transcription regulator | | 7,87E-04 | EGR1,FOS,ID1,LIF,MDM2,VIM |
| NR1D1 | ligand-dependent nuclear receptor | | 7,87E-04 | EGR1,EHHADH,FOS,NR1D1,SLC2A1,ULK1 |
| ellipticine | chemical toxicant | | 7,87E-04 | FGA,IER3,LAMC2,MYC,TNFRSF10A,UPP1 |
| FOXP3 | transcription regulator | | 7,91E-04 | BCL2,CXCR4,DDB2,DUSP4,DUSP6,EGR1,FOS,[illegible] |
| miR-1471 (miRNAs w/seed CCCGGGU) | mature microRNA | | 7,91E-04 | [illegible] |
| LYN | kinase | | 7,93E-04 | BCL2,CFLAR,EGR1,ETS1,MYC,NFKB2,[illegible] |
| SMO | G-protein coupled receptor | | 7,93E-04 | BCL2,CCND1,DIO1,DLX2,IL11,MYC,RUNX1,[illegible] |
| miR-5006-3p (and other miRNAs w/seed CUGUGCU) | mature microRNA | | 7,96E-04 | [illegible] |
| miR-541-3p (and other miRNAs w/seed GGUGGGC) | mature microRNA | | 7,96E-04 | [illegible] |
| TNFRSF1B | transmembrane receptor | | 8,07E-04 | CFLAR,CXCL2,CXCL8,EFNA1,ICAM1,MMP1,[illegible] |
| miR-[illegible] (miRNAs w/seed CAUCCUC) | mature microRNA | | 8,12E-04 | [illegible] |
| prednisolone | chemical drug | | 8,15E-04 | BCL2,BIRC3,CFLAR,CXCR4,CYP1A1,DUSP1,[illegible] |
| lonafarnib | chemical drug | | 8,19E-04 | CCND1,MYC |
| MEK1/2 | group | | 8,19E-04 | DUSP1,FOS |
| Rab11 | group | | 8,19E-04 | ITGA6,TRPC6 |
| [illegible] | enzyme | | 8,19E-04 | EGR1,FOS |
| RPRD1B | other | | 8,19E-04 | CCND1,MYC |
| [illegible] | other | | 8,19E-04 | CCND1,MYC |
| CTNNB-LEF1 | complex | | 8,19E-04 | CCND1,MYC |
| [illegible] | G-protein coupled receptor | | 8,19E-04 | FOS,MYC |
| NOSIP | other | | 8,19E-04 | BCL2,MYC |
| dibromoacetic acid | chemical toxicant | | 8,19E-04 | IGF2,MYC |
| mir-153 | microRNA | | 8,19E-04 | BCL2,MCL1 |
| mir-331 | microRNA | | 8,19E-04 | MYC,PLAUR |
| GATAD2B | transcription regulator | | 8,19E-04 | CXCL8,PTGS2 |
| RAMP3 | G-protein coupled receptor | | 8,19E-04 | FOS,VIM |
| STAMBP | enzyme | | 8,19E-04 | CXCR4,MYC |
| UBE2V1 | transcription regulator | | 8,19E-04 | BCL2,FOS |
| sorafenib derivative SC-1 | chemical reagent | | 8,19E-04 | CCND1,MCL1 |
| [illegible] | chemical - kinase inhibitor | | 8,19E-04 | CFLAR,MCL1 |
| 3-iodobenzylguanidine | chemical drug | | 8,19E-04 | MYC,MYCN |
| [illegible] | chemical reagent | | 8,19E-04 | CCND1,TNFRSF10B |
| (S)-flurbiprofen | chemical drug | | 8,19E-04 | CCND1,JUNB |
| lavendustin A | chemical - kinase inhibitor | | 8,19E-04 | CYP1A1,EGR1 |
| EWSR1-WT1 | fusion gene/product | | 8,19E-04 | EGR1,[illegible] |
| tetrahydropalmatine | chemical - endogenous non-mammalian | | 8,19E-04 | CXCL8,PTGS2 |
| digitoxin | chemical drug | | 8,19E-04 | TNFRSF10A,TNFRSF10B |
| 1-phenazinecarboxylic acid | chemical - endogenous non-mammalian | | 8,19E-04 | CXCL8,ICAM1 |
| GATA4 | transcription regulator | | 8,32E-04 | ANKRD1,BCL2,CDK2,CXCL2,CXCR4,[illegible] |
| miR-3109-3p (and other miRNAs w/seed AGGGCCA) | mature microRNA | | 8,33E-04 | [illegible] |
| miR-[illegible] (miRNAs w/seed UUGUCCC) | mature microRNA | | 8,33E-04 | [illegible] |
| L-methionine | chemical - endogenous mammalian | | 8,43E-04 | [illegible] |
| EPHB4 | kinase | | 8,43E-04 | [illegible] |
| AKT2 | kinase | | 8,43E-04 | [illegible] |
| NKX3-1 | transcription regulator | | 8,43E-04 | [illegible] |
| HTT | transcription regulator | | 8,43E-04 | [illegible] |
| miR-3160-3p (miRNAs w/seed GAGCUGA) | mature microRNA | | 8,45E-04 | [illegible] |
| miR-[illegible] (miRNAs w/seed CUCUGGA) | mature microRNA | | 8,48E-04 | [illegible] |
| miR-11181-5p (and other miRNAs w/seed UCUGACC) | mature microRNA | | 8,53E-04 | [illegible] |
| VCAN | other | | 8,62E-04 | [illegible] |
| IL7R | transmembrane receptor | | 8,62E-04 | [illegible] |
| miR-6717-5p (and other miRNAs w/seed AGGCCAC) | mature microRNA | | 8,72E-04 | [illegible] |
| miR-[illegible] (and other miRNAs w/seed AACAAAC) | mature microRNA | | 8,85E-04 | [illegible] |
| PELP1 | other | | 8,87E-04 | [illegible] |
| miR-2861 (and other miRNAs w/seed GGGCCUG) | mature microRNA | | 8,88E-04 | [illegible] |
| CEACAM1 | transporter | | 9,03E-04 | ALDH1A1,CXCL8,CXCR4,ICAM1,NOTCH3,[illegible] |
| BACH1 | transcription regulator | | 9,08E-04 | CXCR4,DUSP1,MMP1,SLC40A1,SLC7A11 |
| CSF2RB | transmembrane receptor | | 9,20E-04 | BCL2,FOS,JUNB,MYC |
| PLCG1 | enzyme | | 9,20E-04 | CCND1,EGR1,FOS,SERPINE1 |
| NREP | other | | 9,20E-04 | ITGA3,ITGA5,PDGFB,TAGLN |
| [illegible] (includes others) | other | | 9,20E-04 | DUSP6,PTGS2,RUNX1,SULT1E1 |
| MAPK10 | kinase | | 9,20E-04 | CXCL8,FOS,MMP1,PTGS2 |
| PRDX2 | enzyme | | 9,20E-04 | CCND1,EPO,ICAM1,PTGS2 |
| MAML1 | transcription regulator | | 9,20E-04 | CCND1,CXCL8,EGR1,[illegible] |
| BCL10 | transcription regulator | | 9,20E-04 | CXCL2,CXCL8,NFKB2,TNFAIP3 |
| RHOB | enzyme | | 9,20E-04 | CXCL8,ICAM1,PTGS2,RHOB |
| cyclopiazonic acid | chemical - endogenous non-mammalian | | 9,20E-04 | BCL2,CXCL2,EGR1,FOS |
| 2-acetylaminofluorene | chemical toxicant | | 9,20E-04 | BCL2,CCND1,CYP1A1,NOTCH3 |
| hydrogen sulfide | chemical - endogenous mammalian | | 9,20E-04 | BCL2,CXCL8,PTGS2,TMPRSS2 |
| [illegible] | chemical - kinase inhibitor | | 9,20E-04 | EGR1,FOS,PLAUR,SLC2A1 |
| mannose | chemical - endogenous mammalian | | 9,20E-04 | FOS,ICAM1,MCL1,NR4A1 |
| MYOC | other | | 9,37E-04 | EGR2,EPHA2,[illegible] |
| IKBKE | kinase | Activated | 9,37E-04 | BCL2,BIRC3,CCND1,CXCL2,CXCL8,MYC,[illegible] |
| miR-296-5p (miRNAs w/seed GGGCCCC) | mature microRNA | | 9,40E-04 | [illegible] |
| ICAM1 | transmembrane receptor | | 9,42E-04 | COL4A1,CXCL2,FOS,ICAM1,KLF4,MYC,VIM |
| PLAG1 | transcription regulator | | 9,42E-04 | BCL2,GJB1,IGF2,NPTXR,PLEC,RDM1,TGM2 |
| [illegible] | transmembrane receptor | | 9,42E-04 | BCL2,CXXC5,ETS1,FOS,MYC,[illegible] |
| carboplatin | chemical drug | | 9,42E-04 | FGA,IER3,LAMC2,MYC,TNFRSF10A,UPP1,VIM |
| gemfibrozil | chemical drug | | 9,43E-04 | CCND1,EHHADH,GADD45A,LDLR,MYC,[illegible] |
| miR-[illegible] (miRNAs w/seed ACCCCCU) | mature microRNA | | 9,45E-04 | [illegible] |
| miR-3914 (miRNAs w/seed AGGAACC) | mature microRNA | | 9,45E-04 | [illegible] |
| miR-6767-3p (and other miRNAs w/seed CCUGCCU) | mature microRNA | | 9,56E-04 | [illegible] |
| miR-[illegible] (and other miRNAs w/seed GGGCUGA) | mature microRNA | | 9,71E-04 | [illegible] |
| miR-6833-3p (and other miRNAs w/seed GGAGCUA) | mature microRNA | | 9,72E-04 | [illegible] |
| miR-148a-3p (and other miRNAs w/seed CAGUGCA) | mature microRNA | Inhibited | [illegible] | [illegible] |
| dextran sulfate | chemical drug | | 9,90E-04 | C3,CCND1,CXCL2,CXCL8,EFNB2,FGFR1,[illegible] |
| miR-[illegible] (and other miRNAs w/seed CUCCCUC) | mature microRNA | | 9,96E-04 | [illegible] |
| GLI2 | transcription regulator | | 1,01E-03 | BCL2,CCND1,CXCL2,GATA4,HK2,JUNB,[illegible] |
| miR-6882-3p (miRNAs w/seed GCUGCCU) | mature microRNA | | 1,01E-03 | [illegible] |
| miR-30c-5p (and other miRNAs w/seed GUAAACA) | mature microRNA | Inhibited | 1,01E-03 | [illegible] |
| mir-155 | microRNA | | 1,01E-03 | BCL2,CCND1,CEBPA,CXCL8,DUSP6,[illegible] |
| mir-29 | microRNA | | 1,02E-03 | BCL2,CCND1,DNMT3B,KLF4,MCL1,MYCN,[illegible] |
| tert-butyl-hydroquinone | chemical reagent | Activated | 1,02E-03 | [illegible],ATF3,BCL2,CYP1A1,FOS,FOSL1,[illegible] |
| miR-[illegible] (miRNAs w/seed UAGGGGG) | mature microRNA | | 1,02E-03 | [illegible] |
| FOXA2 | transcription regulator | | 1,02E-03 | C3,CCND1,CDK2,CEBPA,[illegible] |
| T3-TR-RXR | complex | | 1,03E-03 | AKR1C3,FGA,LDLR,MDM2,SLC16A3,SLC2A1 |
| THBS1 | other | | 1,03E-03 | COL4A1,EGR1,ITGA5,LRP1,TNFRSF10A,[illegible] |
| isoprenaline | chemical drug | Activated | 1,03E-03 | [illegible],FOS,IER3,NR4A1,PLK2,SLC2A1 |
| miR-7973 (miRNAs w/seed GUGACCC) | mature microRNA | | 1,03E-03 | [illegible] |
| mir-34 | microRNA | | 1,03E-03 | BCL2,BIRC3,CCND1,EMP1,FOSL1,MYC,MYCN,[illegible] |
| ETS2 | transcription regulator | | 1,03E-03 | CDK2,CYP27A1,DUSP6,FOS,ICAM1,JUNB,[illegible] |
| PTPN6 | phosphatase | | 1,06E-03 | BCL2,CCND1,CXCL8,ETS1,IRF1,MYC,VIM |
| miR-18a-3p (and other miRNAs w/seed CUGCCCU) | mature microRNA | | 1,07E-03 | [illegible] |
| BCL2 | transporter | | 1,08E-03 | BCL2,CCND1,CXCL8,FOS,GADD45A,ICAM1,[illegible] |
| fatty acid | chemical - endogenous mammalian | | 1,08E-03 | BCL2,CXCL8,EPO,LDLR,NR4A1,PTGS2,RXRA,[illegible] |
| miR-[illegible] (and other miRNAs w/seed GGAAGGA) | mature microRNA | | 1,09E-03 | [illegible] |
| miR-[illegible] (and other miRNAs w/seed GGGGGAU) | mature microRNA | | 1,09E-03 | [illegible] |
| morphine | chemical drug | | 1,10E-03 | ARRB1,BCL2,CCND1,CXCL8,EHMT2,ERBB3,[illegible] |
| DSCAML1 | other | | 1,10E-03 | KLF4,MYC,NOTCH3,NT5E,PDGFB,PLXNC1,[illegible] |
| 4-phenylbutyric acid | chemical - endogenous mammalian | | 1,10E-03 | BCL2,CCND1,CFLAR,CXCL8,EHHADH,[illegible] |
| miR-6835-5p (and other miRNAs w/seed GGGGGUA) | mature microRNA | | 1,10E-03 | [illegible] |
| miR-[illegible] (miRNAs w/seed GCUCCUC) | mature microRNA | | 1,11E-03 | [illegible] |
| CD44 | other | | 1,12E-03 | BCL2,BIRC3,CCND1,CDK2,GADD45A,ITGA3,[illegible] |
| miR-665 (and other miRNAs w/seed CCAGGAG) | mature microRNA | | 1,12E-03 | [illegible] |
| miR-[illegible] (and other miRNAs w/seed UGGGAUC) | mature microRNA | | 1,13E-03 | [illegible] |
| [illegible] | enzyme | | 1,13E-03 | AMACR,FBP1,GGT1,HK2,ID1,IDH1,[illegible] |
| NR1I3 | ligand-dependent nuclear receptor | | 1,16E-03 | ALDH1A1,CYP27A1,DIO1,GADD45B,ICAM1,[illegible] |
| IL7 | cytokine | | 1,17E-03 | BCL2,CFLAR,CISH,CXCL2,CXCR4,DUSP6,LIF,[illegible] |
| hexachlorobenzene | chemical toxicant | | 1,17E-03 | FGA,IER3,LAMC2,MYC,TNFRSF10A,UPP1,VIM |
| SERPINF1 | other | | 1,17E-03 | BCL2,CCND1,CEBPA,CXCL8,[illegible],MYC,VIM |
| IL32 | cytokine | | 1,17E-03 | BCL2,CXCL8,ICAM1,MCL1,PTGS2,ULBP2,VIM |
| [illegible] | complex | | 1,18E-03 | BIRC3,CCND1,CFLAR |
| ARHGEF1 | other | | 1,18E-03 | CCND1,FOS,TRPC6 |
| S100a7a | other | | 1,18E-03 | CCND1,CXCL8,IL11 |
| CXCR1 | G-protein coupled receptor | | 1,18E-03 | BCL2,CCND1,CXCL8 |
| SMARCA1 | transcription regulator | | 1,18E-03 | GADD45A,MYC,SFN |
| LPA | other | | 1,18E-03 | CXCL8,ICAM1,SERPINE1 |
| NEU3 | enzyme | | 1,18E-03 | BCL2,EGR1,MMP1 |
| TNFRSF10B | transmembrane receptor | | 1,18E-03 | CFLAR,CXCL8,TNFRSF10B |
| MM-401 | chemical reagent | | 1,18E-03 | BCL2,KLF6,MYC |
| [illegible] | chemical - kinase inhibitor | | 1,18E-03 | CXCL8,FOS,MYC |
| GW 5074 | chemical - kinase inhibitor | | 1,18E-03 | FGFR2,PTGS2,SERPINE1 |
| buserelin | biologic drug | | 1,18E-03 | DUSP6,EGR1,FOS |
| palmitoyl-Cys((RS)-2,3-di(palmitoyloxy)-propyl)-Ala-Gly-OH | chemical reagent | | 1,18E-03 | CXCL8,ICAM1,PTGS2 |
| N,N-dimethylarginine | chemical - endogenous mammalian | | 1,18E-03 | BCL2,ICAM1,SERPINE1 |
| tosyllysine chloromethyl ketone | chemical - protease inhibitor | | 1,18E-03 | CYP1A1,IRF1,PTGS2 |
| [illegible] | enzyme | | 1,18E-03 | CCND1,CEBPA,EGR1,FOS,MYC,SLC2A1 |
| ATF1 | transcription regulator | | 1,18E-03 | BCL2,EGR1,FOS,FOSL1,INHBA,PTGS2 |
| chlorpromazine | chemical drug | | 1,18E-03 | FOS,HPN,IL18,LDLR,NR4A1,TAGLN |
| SAHM1 | chemical reagent | Inhibited | 1,18E-03 | BHLHE40,HES4,HK2,ID1,MYC,NLE1 |
| pCPT-cAMP | chemical - kinase inhibitor | | 1,18E-03 | EGR1,FOS,JUNB,NR4A1 |
| Hmgb1 | transcription regulator | | 1,18E-03 | BCL2,CCND1,CXCL8,TUBB3 |
| p85 (pik3r) | group | | 1,18E-03 | BCL2,MCL1,NDRG1,PTGS2 |
| THY1 | other | | 1,18E-03 | ETS1,GAS6,PDGFB,VIM |
| CCAT2 | other | | 1,18E-03 | BCL2,CCND1,MYC,VIM |
| DAXX | transcription regulator | | 1,18E-03 | BCL2,CCND1,MMP1,SERPINE1 |
| nimesulide | chemical drug | | 1,18E-03 | AREG,CDKN2C,CYP1A1,PTGS2 |
| ethidium | chemical drug | | 1,18E-03 | BCL2,CCND1,MMP1,SERPINE1 |
| SAMSN1 | other | Activated | 1,19E-03 | CXCL2,[illegible],IL18,INHBA,IRF1,NFKBIZ,PTGS2,[illegible] |
| nocodazole | chemical reagent | | 1,19E-03 | CCND1,CFLAR,DUSP6,FOS,[illegible],MCL1,[illegible] |
| miR-[illegible] (miRNAs w/seed GGAUGUG) | mature microRNA | | 1,20E-03 | [illegible] |
| miR-3127-3p (and other miRNAs w/seed CCCCUUC) | mature microRNA | | 1,22E-03 | [illegible] |
| Histone h3 | group | | 1,24E-03 | AKAP12,CCND1,CFLAR,CXCL8,DIO1,FOS,[illegible] |
| miR-[illegible] (and other miRNAs w/seed AGGGCCC) | mature microRNA | | 1,27E-03 | [illegible] |
| taurocholic acid | chemical - endogenous mammalian | Activated | 1,27E-03 | ATF3,CYP27A1,EGR1,GADD45B,PTGS2 |
| erlotinib | chemical drug | | 1,27E-03 | AREG,ERBB3,SERPINE1,TGFB3,VIM |
| SH3TC2 | other | | 1,27E-03 | ACSL1,EGR1,EGR2,GJB1,MVD |
| SUB1 | transcription regulator | | 1,27E-03 | CCND1,FJX1,FOS,GADD45A,MDM2 |
| S100A6 | transporter | Inhibited | 1,27E-03 | ATF3,CXCL2,CXCL8,INHBA,TNFAIP3 |
| PHB | transcription regulator | | 1,27E-03 | BCL2,CCND1,CXCL8,IGF2,MDM2 |
| EPOR | transmembrane receptor | | 1,27E-03 | CISH,JUNB,MYC,SLC40A1,SOCS3 |
| letrozole | chemical drug | | 1,27E-03 | BCL2,CCND1,FOSL1,LIF,MYC |
| CD14 | transmembrane receptor | Activated | 1,30E-03 | CISH,CXCL2,CXCL8,ICAM1,PTGS2,SOCS3,[illegible] |
| Gag | other | | 1,30E-03 | [illegible] |
| miR-6791-3p (and other miRNAs w/seed GCCUCCU) | mature microRNA | Activated | 1,31E-03 | [illegible] |
| TLR2 | transmembrane receptor | Activated | 1,35E-03 | ATF3,CISH,CXCL2,CXCL8,DUSP1,GADD45A,[illegible] |
| PRKAA2 | kinase | | 1,35E-03 | CEBPA,DUSP4,EGR1,FGFR2,GALM,IGFBP4,[illegible] |
| SPARC | other | | 1,35E-03 | AKR1C3,BHLHE40,CYP1A1,NDRG1,[illegible] |
| KL | enzyme | | 1,35E-03 | BCL2,CCND1,EGR1,ICAM1,IHH,MYC |
| SREBF2 | transcription regulator | | 1,35E-03 | CCND1,CEBPA,MST1R,PPP1R15A,[illegible] |
| Hif1 | complex | | 1,35E-03 | BCL2,BNIP3L,CXCR4,[illegible],EPO,SERPINE1,[illegible] |
| miR-[illegible] (miRNAs w/seed UAGAGGC) | mature microRNA | | 1,36E-03 | [illegible] |
| PRDM1 | transcription regulator | | 1,37E-03 | APOBEC3C,CXCL2,CXCR4,EGR2,FGA,FOS,[illegible] |
| miR-[illegible] (miRNAs w/seed CCAGUGC) | mature microRNA | | 1,37E-03 | [illegible] |
| miR-638 (miRNAs w/seed GGGAUCG) | mature microRNA | | 1,38E-03 | [illegible] |
| SOCS3 | phosphatase | Inhibited | 1,38E-03 | ATF3,BCL2,CCND1,EGR1,ESRRA,FOS,ICAM1,[illegible] |
| miR-661 (miRNAs w/seed GCCUGGG) | mature microRNA | | 1,40E-03 | [illegible] |
| miR-615-5p (and other miRNAs w/seed GGGGUCC) | mature microRNA | | 1,40E-03 | [illegible] |
| miR-[illegible] (and other miRNAs w/seed UGGAGCU) | mature microRNA | | 1,40E-03 | [illegible] |
| miR-[illegible] (miRNAs w/seed GGGGCUA) | mature microRNA | | 1,42E-03 | [illegible] |
| miR-[illegible] (and other miRNAs w/seed UGCUCUG) | mature microRNA | | 1,42E-03 | [illegible] |
| miR-342-5p (and other miRNAs w/seed GGGGUGC) | mature microRNA | | 1,44E-03 | [illegible] |
| IL3 | cytokine | Activated | 1,48E-03 | BCL2,BHLHE40,CISH,EGR1,FOS,MCL1,MDM2,[illegible] |
| minocycline | chemical drug | | 1,48E-03 | ATF3,BCL2,CXCL8,FOS,ICAM1,IL18,PDGFB,[illegible] |
| N(6)-(3-iodobenzyl)-5'-N-methylcarboxamidoadenosine | chemical drug | | 1,48E-03 | CCND1,FOS,MYC,RGS2 |
| PADI2 | enzyme | | 1,48E-03 | CXCL2,CXCL8,PTGS2,VIM |
| GLIS2 | transcription regulator | | 1,48E-03 | C3,CCND1,SERPINE1,VIM |
| Endothelin | group | | 1,48E-03 | EDN1,FOS,MYC,PTGS2 |
| PLA2G4A | enzyme | | 1,48E-03 | CXCL8,ICAM1,MYC,PTGS2 |
| S1PR2 | G-protein coupled receptor | | 1,48E-03 | FOS,ICAM1,PDGFB,PTGS2 |
| antimycin A | chemical - endogenous non-mammalian | | 1,48E-03 | BCL2,CFLAR,MMP1,TXNIP |
| pimozide | chemical drug | | 1,48E-03 | BCL2,CCND1,CISH,MCL1 |
| magnolol | chemical - endogenous non-mammalian | | 1,48E-03 | BCL2,CXCL8,FOS,PTGS2 |
| IFN Beta | group | Activated | 1,49E-03 | AREG,ATF3,BCL2,CXCL8,DUSP1,GAS6,IL11,[illegible] |
| ADAMTS18 | peptidase | | 1,49E-03 | FGFR2,ITGA3,LAMA1,LAMC2,TGFB3 |
| ZNF281 | transcription regulator | | 1,49E-03 | CXCL2,CXCR4,GADD45A,IL11,PTGS2 |
| RPSA | translation regulator | | 1,49E-03 | DUSP1,DUSP2,ITGA6,PTGS2,RELB |
| PRKCI | kinase | | 1,49E-03 | CCND1,CXCL2,HK2,PTGS2,SLC2A1 |
| urethane | chemical toxicant | | 1,49E-03 | CCND1,CEBPA,CYP1A1,MYC,PTGS2 |
| [illegible] | chemical reagent | | 1,49E-03 | ATF3,CCND1,GADD45A,MYC,PHLDA1 |
| R-WIN 55,212 | chemical reagent | | 1,49E-03 | BCL2,CASP6,CCND1,CXCL8,ICAM1 |
| miR-634 (miRNAs w/seed ACCAGCA) | mature microRNA | | 1,50E-03 | [illegible] |
| 2-methoxyestradiol | chemical - endogenous mammalian | | 1,51E-03 | AREG,ATF3,CXCR4,ID1,MCL1,PTGS2 |
| CITED2 | transcription regulator | | 1,51E-03 | CDK2,CXCL8,DUSP1,MMP1,TGM2,TNFAIP3 |
| EOMES | transcription regulator | | 1,53E-03 | AFAP1,BCL2,CXCR4,[illegible] |
| CD28 | transmembrane receptor | Activated | 1,54E-03 | BCL2,BHLHE40,CASP6,CCND1,CFLAR,CISH,[illegible] |
| miR-[illegible] (miRNAs w/seed CUGAGGC) | mature microRNA | | 1,54E-03 | [illegible] |
| E. coli B4 lipopolysaccharide | chemical toxicant | Activated | 1,56E-03 | BCL2,CASP6,CXCL2,CXCL8,CYP27A1,[illegible] |
| miR-[illegible] (miRNAs w/seed UCCCCAG) | mature microRNA | | 1,56E-03 | [illegible] |
| PPP2CA | phosphatase | | 1,58E-03 | BCL2,CCND1,IRF1,MYC,PTGS2,SOCS3,UPP1 |
| IL1R1 | transmembrane receptor | | 1,58E-03 | CXCL2,CXCL8,EGR1,FOS,ICAM1,IL18,PTGS2 |
| phenylbutazone | chemical drug | | 1,58E-03 | CCND1,IER3,LAMC2,PTGS2,TNFRSF12A,UPP1 |
| cadmium chloride | chemical toxicant | | 1,58E-03 | CCND1,EPO,FOS,GADD45A,MYC,TGFB3,VIM |
| [illegible] | kinase | | 1,61E-03 | BCL2,CCND1,CXCL8,IHH,LDLR,MMP1,PTGS2,[illegible] |
| RELB | transcription regulator | | 1,61E-03 | BCL2,BIRC3,CCND1,CXCL8,MYC,NFKB2,[illegible] |
| miR-[illegible] (and other miRNAs w/seed GGGGCAG) | mature microRNA | | 1,61E-03 | [illegible] |
| IL10 | cytokine | | 1,61E-03 | BCL2,CFLAR,CXCL2,CXCL8,CXCR4,DUSP1,[illegible] |
| miR-[illegible] (miRNAs w/seed UGUGGGU) | mature microRNA | | 1,62E-03 | [illegible] |
| Rar | group | | 1,63E-03 | BCL2,DIO1,GADD45A,GAS2,MMP1,MMP11,[illegible] |
| miR-138-5p (miRNAs w/seed GCUGGUG) | mature microRNA | | 1,63E-03 | [illegible] |
| miR-3177-3p (miRNAs w/seed GCACGGC) | mature microRNA | | 1,65E-03 | [illegible] |
| miR-[illegible] (and other miRNAs w/seed GGGGGCU) | mature microRNA | | 1,66E-03 | [illegible] |
| miR-137-3p (miRNAs w/seed UAUUGCU) | mature microRNA | | 1,66E-03 | [illegible] |
| RB1 | transcription regulator | | 1,69E-03 | BCL2,CASP6,CCND1,CDK2,CEBPA,CHRNB1,[illegible] |
| RAC2 | enzyme | | 1,70E-03 | BCL2,EFNB2,EGR2,FHL2,FOS,NR4A1 |
| dimethylnitrosamine | chemical toxicant | | 1,70E-03 | COL4A1,ICAM1,IGF2,NR4A1,TGM2,VIM |
| L-cysteine | chemical - endogenous mammalian | | 1,73E-03 | CXCL8,FOS,MYC |
| bazedoxifene | chemical drug | | 1,73E-03 | AREG,BCL2,MYC |
| Ck2 | complex | | 1,73E-03 | FOS,MYC,ZFP36 |
| [illegible] | complex | | 1,73E-03 | CFLAR,CXCL2,ICAM1 |
| KDM4B | enzyme | | 1,73E-03 | PDGFB,ULK1,VIM |
| GLRX | enzyme | | 1,73E-03 | CXCL2,ICAM1,PDGFB |
| [illegible] | peptidase | | 1,73E-03 | NFKB2,RELB,TNFAIP3 |
| [illegible] | other | | 1,73E-03 | ITGA3,MMP1,TNFRSF10B |
| [illegible] | transcription regulator | | 1,73E-03 | BCL2,DNMT3B,SERPINE1 |
| ST6GAL1 | enzyme | | 1,73E-03 | BIRC3,CXCL8,SLC2A1 |
| YBX3 | transcription regulator | | 1,73E-03 | CCND1,ERBB3,MYC |
| MCAM | other | | 1,73E-03 | ATF3,ID1,VIM |
| MPL | transmembrane receptor | | 1,73E-03 | FOS,MYC,SLC2A1 |
| C7 | other | | 1,73E-03 | CFLAR,CXCL2,ICAM1 |
| USP17L2 (includes others) | peptidase | | 1,73E-03 | ALDH1A1,KLF4,MYC |
| DEK | transcription regulator | | 1,73E-03 | BIRC3,CXCL8,MCL1 |
| asbestos | chemical toxicant | | 1,73E-03 | CXCL8,FOSL1,JUNB |
| cyclopentenone | chemical reagent | | 1,73E-03 | CCND1,ICAM1,PTGS2 |
| PD 168393 | chemical - kinase inhibitor | | 1,73E-03 | BCL2,CXCL8,MCL1 |
| [illegible] | chemical reagent | | 1,73E-03 | CCND1,CXCL2,PTGS2 |
| hexamethoxyflavone | chemical toxicant | | 1,73E-03 | CXCL8,ICAM1,PTGS2 |
| TBK1 | kinase | Activated | 1,74E-03 | CXCL2,CXCL8,[illegible],ICAM1,IL18,INHBA,[illegible] |
| Focal adhesion kinase | group | | 1,74E-03 | BCL2,EFNB2,FZD4,ICAM1,TAGLN |
| PTX3 | other | | 1,74E-03 | CXCL2,CXCL8,EGR2,FOS,GATA4 |
| PRKAR1A | kinase | | 1,74E-03 | BCL2,CCND1,ERBB3,GATA4,VIM |
| NF1 | other | | 1,74E-03 | CCND1,MXD1,MYC,PDGFB,TNFRSF10B |
| NUP98-HOXA9 | fusion gene/product | | 1,74E-03 | HOXB3,HOXB6,HOXB8,PTGS2,SORBS2 |
| TGFB3 | growth factor | Activated | 1,74E-03 | BCL2,BHLHE40,ETS1,FOS,IL11,MMP1,PLAUR,[illegible] |
| losartan potassium | chemical drug | | 1,74E-03 | CXCL8,EDN1,EGR1,FOS,IL18,PTGS2,[illegible] |
| heparin | chemical - endogenous mammalian | | 1,75E-03 | AREG,CCND1,DUSP1,FOS,ICAM1,MMP1,[illegible] |
| GCG | other | | 1,75E-03 | ACADS,BCL2,FOS,IGFBP4,PDZK1,PLK3,[illegible] |
| 1-methyl-4-phenyl-1,2,3,6-tetrahydropyridine | chemical toxicant | | 1,75E-03 | BCL2,EGR1,EPHX2,FOS,LIF,NR4A1,PTGS2,[illegible] |
| HES1 | transcription regulator | | 1,75E-03 | BCL2,CEBPA,DUSP1,MDM2,OAT,TAGLN,[illegible] |
| HIC1 | transcription regulator | | 1,75E-03 | CCND1,EPHA2,FHL2,INHBA,LIF,PLEC,[illegible] |
| mitomycin C | chemical drug | | 1,75E-03 | ATF3,CCND1,CXCL8,EGR1,GADD45A,MDM2,[illegible] |
| miR-[illegible] (miRNAs w/seed GCCCGCC) | mature microRNA | | 1,76E-03 | [illegible] |
| miR-[illegible] (and other miRNAs w/seed GGGCGCG) | mature microRNA | | 1,76E-03 | [illegible] |
| miR-155-5p (miRNAs w/seed UAAUGCU) | mature microRNA | Inhibited | 1,79E-03 | [illegible] |
| miR-[illegible] (miRNAs w/seed GGGGUGU) | mature microRNA | | 1,80E-03 | [illegible] |
| miR-[illegible] (and other miRNAs w/seed CCAGGGC) | mature microRNA | Activated | 1,81E-03 | [illegible] |
| Srebp | group | | 1,82E-03 | ACSL1,FOS,IDH1,LDLR |
| prostaglandin E1 | chemical - endogenous mammalian | | 1,82E-03 | FOS,ICAM1,MMP1,MYC |
| NOD1 | other | | 1,82E-03 | CDK2,CXCL2,CXCL8,DUSP1 |
| SLC29A1 | transporter | | 1,83E-03 | GADD45A,ITGA5,MDM2,SESN1 |
| ITGA6 | transmembrane receptor | | 1,83E-03 | BCL2,ITGA2,ITGA3,ITGA7 |
| BAP1 | peptidase | | 1,83E-03 | ATF3,CXCL2,LATS1,LIF |
| MNT | transcription regulator | Inhibited | 1,83E-03 | GADD45B,KLF10,KLF6,TXNIP |
| PDCD4 | other | | 1,83E-03 | MYC,PDCD4,PLAUR,PTGS2 |
| metyrapone | chemical drug | | 1,83E-03 | CXCR4,CYP1A1,FOS,ICAM1 |
| lycopene | chemical drug | | 1,83E-03 | BCL2,CCND1,PTGS2,SLC2A1 |
| Esrra | transcription regulator | | 1,86E-03 | [illegible],ESRRA,FBP1,GALM,HSD3B1,IHH,[illegible] |
| olanzapine | chemical drug | | 1,87E-03 | ATF3,BCL2,DCBLD2,FGFR2,FOS,LDLR,NR4A1,[illegible] |
| WBP2 | transcription regulator | | 1,88E-03 | ALOXE3,ALPK2,COL4A1,CXCL2,CXCL8,EGR1,[illegible] |
| MMP9 | peptidase | Activated | 1,89E-03 | CCND1,CXCL2,CXCR4,FOS,GADD45A,ICAM1,[illegible] |
| TBX3 | transcription regulator | | 1,89E-03 | ANKRD1,CXCL2,CXCL8,CXCR4,GATA4,IL11,[illegible] |
| ciglitazone | chemical drug | | 1,89E-03 | BCL2,CCND1,CFLAR,CXCL8,ICAM1,MXD1,[illegible] |
| miR-[illegible] (miRNAs w/seed GAGGUGA) | mature microRNA | | 1,90E-03 | [illegible] |
| miR-[illegible] (miRNAs w/seed GGGACGG) | mature microRNA | | 1,91E-03 | [illegible] |
| PRKAA | group | | 1,91E-03 | BCL2,DUSP1,DUSP2,MCL1,TMPRSS2,VIM |
| PRC2 | complex | | 1,91E-03 | CDK2,CXCL8,GATA4,HOXB6,PPP2R2B,TBX3 |
| IFNAR1 | transmembrane receptor | | 1,91E-03 | ATF3,CXCL2,LIF,MYC,PTGS2,TNFAIP3 |
| FLI1 | transcription regulator | Activated | 1,92E-03 | CCND1,DDX21,EGR1,MDM2,MMP1,[illegible],SRC |
| miR-[illegible] (and other miRNAs w/seed CUGACCC) | mature microRNA | | 1,96E-03 | [illegible] |
| miR-[illegible] (miRNAs w/seed CAGUGAC) | mature microRNA | | 1,96E-03 | [illegible] |
| n-nitrosomethylbenzylamine | chemical toxicant | | 1,96E-03 | CCND1,DUSP6,GREM1,GSTK1,IDH1,KLF10,[illegible] |
| YBX1 | transcription regulator | Activated | 2,01E-03 | CXCL2,FGFR1,FOS,ID1,KLF4,PDGFB,PTGS2,[illegible] |
| Stat3-Stat3 | complex | | 2,01E-03 | FGA,FOS,ICAM1,MYC,SOCS3 |
| ROR1 | kinase | | 2,01E-03 | CDKN2C,GAS6,NDRG1,SERPINE1,VIM |
| TIRAP | other | | 2,01E-03 | CXCL2,CXCL8,ICAM1,IRF1,PTGS2 |
| NF2 | other | | 2,01E-03 | CCND1,CDK2,GLUL,MYC,PLAUR |
| EIF2AK4 | kinase | | 2,01E-03 | ATF3,EGR1,PHGDH,PPP1R15A,PSPH |

| | | | | |
|---|---|---|---|---|
| PRLR | transmembrane receptor | | 2.01E-03 | AKR1C3,CSH,GJB1,HSD3B1,MYC |
| SMAD5 | transcription regulator | | 2.01E-03 | ICAM1,ID1,PDGFB,RUNX1,SERPINE1 |
| lovastatin | chemical drug | | 2.01E-03 | EGR1,MMP1,MYC,NR4A1,PTGS2 |
| hymecromone | chemical drug | | 2.01E-03 | BCL2,CXCL8,CXCR4,MCL1,TNFRSF10A |
| selenium | chemical drug | | 2.01E-03 | CEBPA,IL18,MSRB1,PTGS2,SELENOW |
| ETS1 | transcription regulator | Activated | 2.02E-03 | CCND1,EGR1,ETS1,HSPA1L,ICAM1,JUNB,MCL1,MDM2,MMP1,MYC,PHLDA2,RUNX1,SERPINE1,SLC7A11,ULBP2 |
| N-Ac-Leu-Leu-norleucinal | chemical - protease inhibitor | | 2.06E-03 | ACHE,CCND1,CEBPA,CXCL8,ICAM1,IER3,MYCN,PTGS2 |
| miR-133a-5p (miRNAs w/seed GCUGGUA) | mature microRNA | | 2.08E-03 | [illegible] |
| miR-6081 (miRNAs w/seed GGAGCAG) | mature microRNA | | 2.10E-03 | [illegible] |
| IL6ST | transmembrane receptor | | 2.11E-03 | CCND1,EGR1,FOS,IRF1,JUNB,MYC,SOCS3 |
| maneb | chemical toxicant | | 2.11E-03 | CYP1A1,EGR4,INHBA,LAMC2,NFAT5,NR4A1,PRR7 |
| CDKN1B | kinase | | 2.11E-03 | BCL2,CCND1,DUSP6,EGR1,EPHA2,MCL1,MYC,PALD1,RUNX1,SERPINE1 |
| miR-363-5p (and other miRNAs w/seed GGGUGGA) | mature microRNA | | 2.12E-03 | [illegible] |
| miR-3150a-3p (and other miRNAs w/seed GAGGAGA) | mature microRNA | Activated | 2.13E-03 | [illegible] |
| LRP6 | transmembrane receptor | | 2.13E-03 | ALDH1A1,CCND1,FOS,HK2,MYC,VIM |
| vinorelbine | chemical drug | | 2.13E-03 | BCL2,EGR1,MCL1,MDM2,MYCN,PTGS2 |
| levodopa | chemical - endogenous mammalian | Activated | 2.14E-03 | [illegible] |
| miR-6433b-3p (miRNAs w/seed AGGAGUG) | mature microRNA | | 2.14E-03 | [illegible] |
| LEF1 | transcription regulator | Activated | 2.15E-03 | BCL2,CCND1,CXCR4,LHX8,MKI1,MYC,NTN4,PHLDA1,PTGS2,SHORT1,SPRY1,VIM |
| N-nitro-L-arginine methyl ester | chemical drug | | 2.15E-03 | ATF3,BCL2,CXCL2,CXCL8,EPO,ICAM1,LDLR,PTGS2,SERPINE1 |
| PPARGC1A | transcription regulator | | 2.15E-03 | [illegible] |
| miR-6884-3p (miRNAs w/seed GUUGCAA) | mature microRNA | | 2.18E-03 | [illegible] |
| miR-548aj-5p (and other miRNAs w/seed GCAAAAG) | mature microRNA | | 2.20E-03 | [illegible] |
| USF1 | transcription regulator | | 2.20E-03 | AKAP12,CEBPA,DUSP1,LDLR,PTGS2,SERPINE1,TNFAIP3,TXNIP |
| ZEB1 | transcription regulator | | 2.20E-03 | ALDH1A1,LAMC2,MMP1,MPZL2,PTGS2,RBM47,SERPINE1,VIM |
| STAT1 | transcription regulator | Activated | 2.20E-03 | [illegible] |
| miR-744-5p (and other miRNAs w/seed GCGGGGC) | mature microRNA | | 2.20E-03 | [illegible] |
| cyclooxygenase | group | | 2.24E-03 | FOS,LIF,PTGS2,RGS2 |
| dacinostat | chemical drug | | 2.24E-03 | BCL2,CFLAR,TNFRSF10A,TNFRSF10B |
| SAV1 | other | | 2.24E-03 | CCND1,COL4A1,CXCL2,VIM |
| dasatinib | chemical drug | | 2.24E-03 | BCL2,ERBB3,MCL1,MYC |
| STAP2 | other | | 2.24E-03 | CCND1,CXCL2,CXCR4,MYC |
| KLF10 | transcription regulator | | 2.24E-03 | BCL2,CCND1,CEBPA,SERPINE1 |
| HOXD3 | transcription regulator | | 2.24E-03 | ITGA5,SERPINE1,TAGLN,TGM2 |
| S1PR1 | G-protein coupled receptor | | 2.24E-03 | CCND1,ICAM1,JUNB,PTGS2 |
| RNDS | enzyme | | 2.24E-03 | BCL2,CCND1,PTGS2,VIM |
| Usp17la (includes others) | peptidase | | 2.24E-03 | CSH,CXCL8,KLF4,MYC |
| [illegible] | [illegible] | [illegible] | [illegible] | [illegible] |

| | | | | |
|---|---|---|---|---|
| lanatoside C | chemical - endogenous non-mammalian | | 4.73E-03 | TNFRSF10A,TNFRSF10B |
| NME2 | kinase | | 4.73E-03 | MYC,PTGS2 |
| CHRM4 | G-protein coupled receptor | | 4.73E-03 | EGR1,ITGA6 |
| RSD1980226 | other | | 4.73E-03 | EDN1,PTGS2 |
| SNRK | kinase | | 4.73E-03 | CCND1,MYC |
| CDC20 | other | | 4.73E-03 | CFLAR,[illegible] |
| SLC9A3R2 | transporter | | 4.73E-03 | CCND1,MYC |
| PODXL | kinase | | 4.73E-03 | MMP1,VIM |
| WTAP | other | | 4.73E-03 | AREG,BCL2 |
| carboxyamido-triazole | chemical drug | | 4.73E-03 | CCND1,FOS |
| hoechst 33342 | chemical reagent | | 4.73E-03 | FOS,SPRY1 |
| lansoprazole | chemical drug | | 4.73E-03 | CXCL8,PTGS2 |
| clomiphene | chemical drug | | 4.73E-03 | LDLR,LIF |
| pentamidine | chemical drug | | 4.73E-03 | MCL1,TNFRSF10B |
| bupropion | chemical drug | | 4.73E-03 | TNFRSF10A,TNFRSF10B |
| CCI-007 | chemical reagent | | 4.73E-03 | BCL2,MYC |
| C26-163 | chemical reagent | | 4.73E-03 | ICAM1,TNFAIP3 |
| yohimbine | chemical drug | | 4.73E-03 | EGR1,FOS |
| galanthamine | chemical drug | | 4.73E-03 | BCL2,IGF2 |
| tyrphostin AG 18 | chemical - kinase inhibitor | | 4.73E-03 | AKR1C3,TAGLN |
| pyocyanin | chemical reagent | | 4.73E-03 | CXCL8,ICAM1 |
| Ca2+ | chemical - endogenous mammalian | | 4.73E-03 | EPO,NDRG1 |
| miR-5585-5p (and other miRNAs w/seed CCAGGCA) | mature microRNA | Activated | 4.73E-03 | [illegible] |
| miR-1225-3p (miRNAs w/seed GAGCCCC) | mature microRNA | Activated | 4.75E-03 | [illegible] |
| SFRP1 | transmembrane receptor | Inhibited | 4.75E-03 | CCND1,MMP1,MYC,SDC2,SERPINE1,VIM |
| pyridaben | chemical toxicant | | 4.75E-03 | EGR4,INHBA,LAMC2,NFAT5,NR4A1,PRRT |
| 3-methylcholanthrene | chemical toxicant | | 4.75E-03 | CCND1,CYP1A1,FOS,NR4A1,PTGS2,SLC7A11 |
| colchicine | chemical drug | | 4.75E-03 | ATF3,FOS,IL18,MCL1,MDM2,PTGS2 |
| NKX2-3 | transcription regulator | | 4.79E-03 | CSRNP1,CXCL8,EDN1,HPE,KLF4,PHGDH,PIM3,PTGS2,REEP1,SLC40A1,TAP2,TENT5A,TIPARP,TRPC6 |
| RARB | ligand-dependent nuclear receptor | | 4.87E-03 | ACSL1,ALDH1A1,EPO,FGFR1,GAS6,MMP11,PDGFB |
| SOX1 | transcription regulator | | 4.87E-03 | CDX2,DUSP1,DUSP4,DUSP6,EPHA2,IER5,INHBA |
| mir-22 | microRNA | | 4.89E-03 | ARRB1,ERBB3,FOSL1,HK2,RGS2 |
| FCGR2A | transmembrane receptor | Activated | 4.89E-03 | CXCL2,CXCL8,ICAM1,PTGS2,SERPINE1 |
| SLC13A1 | transporter | | 4.90E-03 | ACSL5,AKR1C3,ALDH1A1,G0S2,HK2,MVD,PEX11,SULT1E1 |
| diethylnitrosamine | chemical toxicant | Activated | 4.90E-03 | CCND1,HSD3B1,MCL1,MYC,OAT,PAH,PHLDA1,SELENBP1,SLCO2B1 |
| miR-5682 (miRNAs w/seed UAGCACC) | mature microRNA | | 4.96E-03 | [illegible] |
| let-7 | microRNA | | 4.96E-03 | CCND1,DUSP6,FOSL1,HK2,MYC,MYCN,PBX3,PLAUR,PTGS2,SERPINE1,TMPRSS2,TRIB1,VIM,ZFP36 |
| TSC2 | other | Inhibited | 5.01E-03 | ATF3,CCND1,EGR1,FOS,HK2,HMGCS2,MCL1,SLC2A1,TAGLN,ZFP36 |
| miR-6834-3p (miRNAs w/seed AUGUCCC) | mature microRNA | | 5.04E-03 | [illegible] |
| miR-6569-5p (and other miRNAs w/seed GGGCGAG) | mature microRNA | | 5.06E-03 | [illegible] |
| miR-186a-3p (and other miRNAs w/seed CAGUAGU) | mature microRNA | | 5.06E-03 | [illegible] |
| miR-4779 (miRNAs w/seed AGGAGGG) | mature microRNA | | 5.07E-03 | [illegible] |
| CDKN1A | kinase | | 5.09E-03 | BCL2,CCND1,COL4A1,CXCL2,DUSP1,GADD45A,GNA11,INHBA,MDM2,MMP1,MYC,PHGDH,PLK2,SERPINE1,TUBB3,VIM |
| 17-alpha-ethinylestradiol | chemical drug | | 5.15E-03 | ARRDC3,C3,FOS,LDLR,MDM2,PLK3,SESN1,SPN,SLC40A1,TGFB3,TGM2 |
| miR-3190-5p (miRNAs w/seed CUGGCCA) | mature microRNA | | 5.15E-03 | [illegible] |
| CHD7 | enzyme | | 5.23E-03 | MDM2,MYCN,NOTCH3,TNFRSF10B |
| dibutyl phthalate | chemical toxicant | | 5.23E-03 | FOS,JUNB,MYC,SERPINE1 |
| Smad | complex | | 5.23E-03 | ID1,MCL1,MYC,SERPINE1 |
| E2F6 | transcription regulator | | 5.23E-03 | CASP6,MYC,MYCN,SERPINE1 |
| COMMD1 | transporter | | 5.23E-03 | BIRC3,HK2,LGALS3,STC2 |
| SSTR2 | G-protein coupled receptor | | 5.23E-03 | BCL2,FOS,TNFRSF10A,VIM |
| elaidic acid | chemical - endogenous mammalian | Inhibited | 5.23E-03 | ACOT1,ACSL1,ALDH1A1,CEBPA,DHRS3,LDLR,PHGDH,SERPINA6 |
| LCK | kinase | Activated | 5.25E-03 | CISH,EGR1,FOS,GADD45B,IRF1,MYC,RUNX1 |
| ADAM17 | peptidase | | 5.25E-03 | AREG,CCND1,CXCL2,CXCL8,EPHA2,MYCN,SOCS3 |
| miR-4445-3p (miRNAs w/seed AGGGCUG) | mature microRNA | | 5.26E-03 | [illegible] |
| miR-891a-5p (miRNAs w/seed GCAACGA) | mature microRNA | | 5.26E-03 | [illegible] |
| miR-6530 (miRNAs w/seed CCAGCAG) | mature microRNA | | 5.32E-03 | [illegible] |
| miR-4753-5p (miRNAs w/seed AAGGCCA) | mature microRNA | | 5.36E-03 | [illegible] |
| digoxin | chemical drug | | 5.40E-03 | NDRG1,TNFRSF10A,TNFRSF10B |
| desoxycorticosterone | chemical - endogenous mammalian | | 5.40E-03 | FOS,PTGS2,SERPINE1 |
| [illegible] | group | | 5.40E-03 | AREG,EGR1,VIM |
| CSNK2B | kinase | | 5.40E-03 | CCND1,CFLAR,MYC |
| [illegible] | group | | 5.40E-03 | BCL2,DUSP1,ID1 |
| FGF23 | growth factor | | 5.40E-03 | EGR1,IHH,PDZK1 |
| EN1 | transcription regulator | | 5.40E-03 | ALDH1A1,CCND1,TUBB3 |
| TRPV1 | ion channel | | 5.40E-03 | FOS,IL11,PTGS2 |
| HIF3A | transcription regulator | | 5.40E-03 | EDN1,PLAUR,SLC2A1 |
| CSF2RA | transmembrane receptor | | 5.40E-03 | FOS,JUNB,MYC |
| MED16 | transcription regulator | | 5.40E-03 | MMP1,TAGLN,VIM |
| HBP1 | transcription regulator | | 5.40E-03 | CCND1,MYCN,PTGS2 |
| CRY2 | enzyme | | 5.40E-03 | FOS,IHH,PER2 |
| BCAR1 | enzyme | | 5.40E-03 | CCND1,EGR1,SRC |
| RPS6KA4 | kinase | | 5.40E-03 | DUSP1,FOS,JUNB |
| MMP8 | peptidase | | 5.40E-03 | CLDN3,CXCL8,PDCD4 |
| ALCAM | other | | 5.40E-03 | AMOT,BCL2,L1CAM |
| DUSP14 | phosphatase | | 5.40E-03 | IL18,INHBA,NFKBIZ |
| HSPA1A/HSPA1B | enzyme | | 5.40E-03 | CXCL2,CXCL8,FOS |
| cantharidin | chemical drug | | 5.40E-03 | BCL2,MCL1,RXRA |
| N,N-dimethylsphingosine | chemical reagent | | 5.40E-03 | FOS,ICAM1,PTGS2 |
| [illegible] | biologic drug | | 5.40E-03 | CXCL2,FOS,SLC2A1 |
| verteporfin | chemical drug | | 5.40E-03 | COL4A1,CXCL2,VIM |
| miR-6520-3p (miRNAs w/seed UGGACAG) | mature microRNA | | 5.42E-03 | [illegible] |
| miR-3689a (and other miRNAs w/seed GGAGGUG) | mature microRNA | Activated | 5.43E-03 | [illegible] |
| NOX4 | enzyme | | 5.45E-03 | CXCL8,ICAM1,MCL1,SERPINE1,SLC2A1 |
| carbamylcholine | chemical drug | | 5.45E-03 | CCND1,EGR1,FOS,NR4A1,RGS2 |
| histone deacetylase | complex | | 5.45E-03 | BCL2,CCND1,CXCR4,JUNB,TNFRSF10A |
| propofol | chemical drug | | 5.45E-03 | BCL2,EGR1,FOS,GJB1,ZFP36 |
| miR-376a-3p (and other miRNAs w/seed ACAUAGA) | mature microRNA | | 5.53E-03 | [illegible] |
| miR-614 (miRNAs w/seed AACGCCU) | mature microRNA | Inhibited | 5.56E-03 | ADD3,CPEB4,GPCPD1,KLF10,MAFG,MDM2,NOCT,PLK3,RND3,TBX3 |
| miR-3150a-3p (and other miRNAs w/seed UGGGGAG) | mature microRNA | | 5.63E-03 | [illegible] |
| zymosan | chemical - endogenous non-mammalian | Activated | 5.65E-03 | CXCL2,CXCL8,LIF,PTGS2,RARA,SOCS3,THRA |
| [illegible] | biologic drug | | 5.65E-03 | ANXA8,BCL2,CXCL8,IRF1,MMP1,NFKB2,PTGS2 |
| cadmium | chemical toxicant | Activated | 5.65E-03 | C3,CCND1,CYP1A1,EPO,MYC,PTGS2,SLC30A1 |
| miR-124-3p (and other miRNAs w/seed AAGGCAC) | mature microRNA | Inhibited | 5.66E-03 | [illegible] |
| CLEC4D | other | | 5.67E-03 | AREG,FZD4,IGF2,IL11,NOTCH3,TGFB3 |
| PAX5 | transcription regulator | | 5.67E-03 | BCL2,CCND1,HK2,SLC2A1,TXNIP,VIM |
| miR-6808-5p (and other miRNAs w/seed AGGCAGG) | mature microRNA | | 5.72E-03 | [illegible] |
| miR-6720-3p (miRNAs w/seed GCGCCUG) | mature microRNA | | 5.79E-03 | [illegible] |
| miR-4276 (and other miRNAs w/seed UCAGUGA) | mature microRNA | | 5.80E-03 | [illegible] |
| miR-3975 (miRNAs w/seed GAGGCUA) | mature microRNA | | 5.85E-03 | [illegible] |
| SUZ12 | enzyme | Activated | 5.86E-03 | ABL2,FOXA1,GATA4,HOXB8,KLF4,KRT17,LAMC2,MYC,PPP2R2B,TBX3 |
| L-glutamic acid | chemical - endogenous mammalian | Activated | 5.86E-03 | BCL2,CCND1,EGR1,FOS,GADD45B,IL18,JUNB,PTGS2,TAP2 |
| KIT | transmembrane receptor | | 5.86E-03 | BCL2,CISH,CXCR4,ICAM1,IRF1,LIF,MCL1,MYC,SOCS3 |
| miR-187-3p (miRNAs w/seed CGUGUCU) | mature microRNA | Inhibited | 5.97E-03 | [illegible] |
| miR-6370 (and other miRNAs w/seed CAGGGAG) | mature microRNA | | 5.97E-03 | [illegible] |
| PHF1 | transcription regulator | | 6.04E-03 | HOXB6,HOXB8,MDM2,SPN |
| [illegible] | enzyme | | 6.04E-03 | BHLHE40,CXCL8,EMP1,SERPINE1 |
| DCAF1 | kinase | | 6.04E-03 | KLF10,SOCS3,TGM2,TXNIP |
| OXT | other | | 6.04E-03 | FOS,GATA4,PTGS2,RGS2 |
| RCAN1 | transcription regulator | | 6.04E-03 | CXCL8,FOS,ICAM1,PTGS2 |
| AKT3 | kinase | | 6.04E-03 | ERBB3,ETS1,FOSL1,IGF2 |
| HDAC9 | transcription regulator | | 6.04E-03 | BCL2,CEBPA,NR4A1,RGS2 |
| NOS1 | enzyme | | 6.04E-03 | EGR1,EPO,FOS,SLC2A1 |
| K-252 | chemical drug | | 6.04E-03 | CXCL8,MMP1,MYCN,NDRG1 |
| calyculin A | chemical toxicant | | 6.04E-03 | CCND1,EGR1,MCL1,TGM2 |
| amino acids | chemical - endogenous mammalian | | 6.04E-03 | FOS,IGF2,JUNB,MYC |
| linolenic acid | chemical - endogenous mammalian | | 6.04E-03 | ACSL1,EDN1,ICAM1,SERPINE1 |
| zoledronic acid | chemical drug | | 6.07E-03 | BCL2,CXCL8,CXCR4,PAK1,PTGS2 |
| STK40 | kinase | | 6.07E-03 | CDX2,CEBPA,CXCL8,DUSP6,IVD,PTGS2,SPRY4 |
| ZNF217 | transcription regulator | | 6.07E-03 | ERBB3,GATA4,GPRC5A,MPZL2,MYC,MYCN,NRXN3 |
| nifedipine | chemical drug | | 6.07E-03 | DUSP6,EDN1,[illegible],FOS,LDLR,NR4A1,PTGS2 |
| miR-344a-5p (and other miRNAs w/seed CAGGCUC) | mature microRNA | | 6.14E-03 | [illegible] |
| mir-204 | microRNA | | 6.18E-03 | BCL2,CXCL2,CXCL8,MCL1,MYC,TGFB3 |
| PIM1 | kinase | | 6.18E-03 | BCL2,EPHA2,ERBB3,FOSL1,MDM2,MYC |
| RORC | ligand-dependent nuclear receptor | | 6.21E-03 | CISH,CXCL2,GADD45B,HSD3B1,NOCT,RHBG,SELENBP1,SLC26A4,SLC39A8,SULT1E1,USP53 |
| miR-181a-5p (and other miRNAs w/seed ACAUUCA) | mature microRNA | | 6.26E-03 | [illegible] |
| miR-328-3p (and other miRNAs w/seed UGGCCCU) | mature microRNA | | 6.26E-03 | [illegible] |
| miR-1915-3p (and other miRNAs w/seed CCCAGGG) | mature microRNA | | 6.30E-03 | [illegible] |
| miR-6862-3p (and other miRNAs w/seed CUCACCC) | mature microRNA | | 6.32E-03 | [illegible] |
| daidzein | chemical drug | | 6.32E-03 | AKR1C3,BCL2,CCND1,CYP1A1,LDLR,MYC,PTGS2,PXMP4 |
| miR-24-3p (and other miRNAs w/seed GGCUCAG) | mature microRNA | | 6.46E-03 | [illegible] |
| diphtheria toxin | chemical - endogenous non-mammalian | | 6.54E-03 | ACADSB,DUSP6,FOS,GAS6,RELB,SPRED1,TGM2,TNFAIP3,VAV3 |
| Tlr | group | Activated | 6.54E-03 | CISH,CXCL8,DUSP1,ICAM1,IRF1,PTGS2,SOCS3,TNFAIP3,ZC3H12A |
| miR-16-5p (and other miRNAs w/seed AGCAGCA) | mature microRNA | | 6.54E-03 | [illegible] |
| HOXA10 | transcription regulator | | 6.66E-03 | ADH4,ALDH1A1,EGR2,GAS6,HOXB3,ID1,IL11,KLF10,MYCN,NR4A1,PHGDH,RNASE4 |
| miR-6865-3p (and other miRNAs w/seed CUCUCCU) | mature microRNA | | 6.71E-03 | [illegible] |
| PTK2 | kinase | | 6.71E-03 | BIRC3,CCND1,FOS,GATA4,ITGA6,LDLR |
| MAPKAPK2 | kinase | Activated | 6.71E-03 | CXCL2,CXCL8,ICAM1,IL18,ZC3H12C,ZFP36 |
| emodin | chemical drug | | 6.71E-03 | BCL2,CXCL8,CXCR4,CYP1A1,ICAM1,VIM |
| NCOR1 | transcription regulator | | 6.72E-03 | CCND1,CEBPA,DIO1,DUSP1,NDRG1,NR1D1,PTGS2,STK39 |
| 5'-methylthioadenosine | chemical - endogenous mammalian | | 6.73E-03 | CCND1,MMP1,PTGS2 |
| glycochenodeoxycholate | chemical - endogenous mammalian | | 6.73E-03 | BIRC3,TNFRSF10A,TNFRSF10B |
| CD40LG-AS1 | other | | 6.73E-03 | CXCL8,ICAM1,PTGS2 |
| PTBP1 | enzyme | | 6.73E-03 | FGFR1,LDLR,MYC |
| PLCE1 | enzyme | | 6.73E-03 | CXCL2,CXCL8,PTGS2 |
| F10 | peptidase | | 6.73E-03 | CXCL8,EGR1,ICAM1 |
| PIAS4 | transcription regulator | | 6.73E-03 | CXCL2,IRF1,SERPINE1 |
| PTPRE | phosphatase | | 6.73E-03 | CXCL2,IL18,INHBA |
| LAMC1 | other | | 6.73E-03 | EGR2,IGF2,VIM |
| MVP | other | | 6.73E-03 | CXCL2,CXCL8,ICAM1 |
| N-(3-oxododecanoyl)-homoserine lactone | chemical reagent | | 6.73E-03 | CXCL2,CXCL8,PTGS2 |
| auranofin | chemical drug | | 6.73E-03 | C3,CXCL8,PTGS2 |
| MSX2 | transcription regulator | | 6.73E-03 | CCND1,EGR2,IHH,TGFB3,VIM |
| [illegible] | transcription regulator | | 6.73E-03 | G0S2,GPCPD1,HK2,PDCD4,SERPINE1 |
| EPHA4 | kinase | Inhibited | 6.73E-03 | DCBLD2,DUSP4,DUSP6,FOSL1,IRF1 |
| ibuprofen | chemical drug | | 6.73E-03 | CCND1,MMP1,PTGS2,SLC2A1,TNFRSF10B |
| everolimus | chemical drug | | 6.73E-03 | CCND1,MCL1,MYC,NCEH1,SLC2A1 |
| pentoxifylline | chemical drug | | 6.73E-03 | CCND1,FGA,FOS,ICAM1,TNFRSF10B |
| miR-6314 (and other miRNAs w/seed UCUGGGA) | mature microRNA | Activated | 6.75E-03 | [illegible] |
| miR-4323 (miRNAs w/seed AGCCCCA) | mature microRNA | | 6.79E-03 | [illegible] |
| miR-887-3p (miRNAs w/seed UGAACGG) | mature microRNA | | 6.80E-03 | [illegible] |
| lithocholic acid | chemical - endogenous mammalian | | 6.80E-03 | BCL2,LDLR,LRP2,PLAUR |
| CD300LF | other | | 6.80E-03 | CXCL8,IDH1,MPC2,SLC2A1 |
| BMP | group | | 6.80E-03 | ERBB3,GATA4,ID1,IRF1 |
| vemurafenib | chemical drug | | 6.80E-03 | CXCL8,DUSP6,SPRY2,TNFRSF10B |
| PROCR | other | | 6.80E-03 | CCND1,ICAM1,LIF,MYC |
| mir-148 | microRNA | | 6.80E-03 | BCL2,DNMT3B,EGR1,LDLR |
| DTX1 | transcription regulator | | 6.80E-03 | DUSP6,EGR1,MCL1,SPRY4 |
| MAP3K2 | kinase | | 6.80E-03 | AREG,FOS,MYC,TNFRSF10A |
| CTSB | peptidase | | 6.80E-03 | CCND1,CXCL2,MYC,PLAUR |
| VCP | enzyme | | 6.80E-03 | BCL2,CXCL8,DUSP1,NFKB2 |
| ozone | chemical toxicant | | 6.80E-03 | CXCL2,CXCL8,FOS,JUNB |
| isoflurane | chemical drug | | 6.80E-03 | BCL2,EPO,FOS,ICAM1 |
| FGF7 | growth factor | | 6.86E-03 | CCND1,CEBPA,FGFR1,FGFR2,IRF1,SERPINE1,SPRY2 |
| ROR2 | kinase | | 6.86E-03 | BCL2,CCND1,CEBPA,GALM,MDM2,SPN,SPRY4 |
| Bay 11-7082 | chemical - kinase inhibitor | Inhibited | 6.86E-03 | CCND1,CXCL2,CXCL8,ICAM1,MCL1,PTGS2,SH2D6 |
| TNFRSF1A | transmembrane receptor | | 7.12E-03 | BIRC3,CFLAR,CXCL2,CXCL8,EFNA1,ICAM1,IER3,MMP1,SERPINE1,SOCS3,SRC |
| SPI1 | transcription regulator | | 7.16E-03 | CCND1,CEBPA,DDX21,DUSP6,EGR2,FCGRT,FOS,IL18,ITGA6,KLF4,MCL1,MMP1,MYC,NPT,PTGS2,VIM |
| miR-1228-3p (miRNAs w/seed CACACCU) | mature microRNA | | 7.20E-03 | [illegible] |
| NR1H4 | ligand-dependent nuclear receptor | | 7.22E-03 | ACSS1,C3,CEBPA,EDN1,KLF4,LDLR,MYC,NFAT5,PTGS2,RXRA,SULT1E1,TXNIP |
| miR-550-5p (and other miRNAs w/seed GGCACCA) | mature microRNA | | 7.24E-03 | [illegible] |
| miR-6888-5p (miRNAs w/seed AGGAGAU) | mature microRNA | | 7.26E-03 | [illegible] |
| NOTCH3 | transcription regulator | | 7.26E-03 | BCL2,CFLAR,ID1,PBX1,SDC2,TAGLN |
| RNF2 | transcription regulator | | 7.26E-03 | CDX2,GATA4,HOXB3,HOXB6,HOXB8,PHC2 |
| capsaicin | chemical drug | | 7.26E-03 | BCL2,CCND1,CXCL8,FOS,PTGS2,RXRA |
| miR-3547 (and other miRNAs w/seed GAGCACC) | mature microRNA | | 7.36E-03 | [illegible] |
| miR-153-3p (miRNAs w/seed UGCAUAG) | mature microRNA | | 7.37E-03 | [illegible] |
| miR-5588-3p (miRNAs w/seed AGUCCCA) | mature microRNA | | 7.37E-03 | [illegible] |
| miR-3148 (miRNAs w/seed GGAAAAA) | mature microRNA | | 7.42E-03 | [illegible] |
| miR-6866-3p (miRNAs w/seed GACCCCU) | mature microRNA | | 7.43E-03 | [illegible] |
| miR-540-3p (and other miRNAs w/seed GGUCAGA) | mature microRNA | | 7.44E-03 | [illegible] |
| SLC27A2 | transporter | Activated | 7.44E-03 | ACOT1,ACSL1,ALDH1A1,EHHADH,PXMP4 |
| mir-150 | microRNA | | 7.44E-03 | CXCL2,CXCR4,FZD4,NOTCH3,SLC2A1 |
| RPS6KB1 | kinase | | 7.44E-03 | BCL2,CCND1,CFLAR,MCL1,SERPINE1 |
| SELPLG | other | Activated | 7.44E-03 | CXCL2,CXCL8,CXCR4,FOS,PLAUR |
| ARHGAP21 | other | Activated | 7.44E-03 | CXCL2,IL18,IRF1,NFKBIZ,PTGS2 |
| EFNA3 | kinase | Inhibited | 7.44E-03 | DCBLD2,DUSP4,DUSP6,FOSL1,IRF1 |
| HBB | transporter | | 7.44E-03 | CXCL2,CXCL8,ICAM1,LIF,SERPINE1 |
| retinol | chemical drug | | 7.44E-03 | DUSP1,EDN1,ITGA6,MYC,PTGS2 |
| mir-30 (includes others) | microRNA | Inhibited | 7.44E-03 | GADD45A,ITGA2,SERPINE1,STK39,VIM |
| ouabain | chemical drug | | 7.44E-03 | CLDN3,FOS,TNFRSF10A,TNFRSF10B,VIM |
| IL12 (complex) | complex | Activated | 7.45E-03 | CISH,CXCL8,DDB2,FOS,GADD45B,ICAM1,IL18,IRF1,ITGA6,MYC,PDCD4,PTGS2,SOCS3 |
| ERG | transcription regulator | | 7.45E-03 | BIRC3,CHRNB1,CXCL8,CXCR4,ETS1,ICAM1,ITGA6,MMP1,MYCN,NDRG1,PLAUR,TMPRSS2,VIM |
| EIF4E | translation regulator | | 7.45E-03 | BCL2,CCND1,CEBPA,FOSL1,IGFBP4,IL18,MCL1,MYC,PLAUR,SERTAD1,SLCO2B1,UPP1,VSIR |
| miR-184 (and other miRNAs w/seed GGACGGA) | mature microRNA | | 7.47E-03 | [illegible] |
| miR-6316 (miRNAs w/seed ACUGUGG) | mature microRNA | | 7.48E-03 | [illegible] |
| SFTPA1 | transporter | Inhibited | 7.48E-03 | AMOT,CXCL2,EGR1,FOS,MYC,PHLDA1,SERPINE1 |
| SREBF2 | transcription regulator | | 7.48E-03 | ACSL1,CXCL8,IDH1,LDLR,LRP1,MVD,TM7SF2 |
| (+)-MK-801 | chemical drug | | 7.48E-03 | CCND1,EGR1,FOS,ICAM1,JUNB,NPTXR,PTGS2 |
| SP110 | transcription regulator | | 7.49E-03 | ARRB1,ATF3,ETS1,IL18,MCL1,MYC,MYCN,RXRA,TBX3,TXNIP |
| miR-4721 (and other miRNAs w/seed GAGGGCU) | mature microRNA | | 7.51E-03 | [illegible] |
| NKX2-1 | transcription regulator | | 7.53E-03 | AREG,CA8,CEBPA,ESAM,IER3,LRP2,MTF1,MYBPH,PARD6B,RNASE4,SERPINE1,TMEM37 |
| miR-128-3p (and other miRNAs w/seed CACAGUG) | mature microRNA | | 7.53E-03 | [illegible] |
| imatinib | chemical drug | Inhibited | 7.63E-03 | [illegible] |
| miR-718 (miRNAs w/seed UUCCGCC) | mature microRNA | | 7.63E-03 | [illegible] |
| miR-6728-3p (miRNAs w/seed CUCUGCU) | mature microRNA | | 7.66E-03 | [illegible] |
| miR-1268a (and other miRNAs w/seed GGGCGUG) | mature microRNA | | 7.72E-03 | [illegible] |
| ILX-23-7553 | chemical drug | | 7.73E-03 | BCL2,CCND1 |
| L-alpha-lysophosphatidylcholine, palmitoyl | chemical - endogenous mammalian | | 7.73E-03 | CXCL8,ICAM1 |
| [illegible] | group | | 7.73E-03 | NFKB2,RELB |
| dienogest | chemical drug | | 7.73E-03 | CXCL8,PTGS2 |
| styrene | chemical toxicant | | 7.73E-03 | EGR1,HSPA1L |
| Cpla2 | group | | 7.73E-03 | MYC,PTGS2 |
| [illegible] | chemical drug | | 7.73E-03 | BCL2,MCL1 |
| [illegible] | chemical - endogenous non-mammalian | | 7.73E-03 | CXCL8,PTGS2 |
| RAR-RXR | complex | | 7.73E-03 | IRF1,TGM2 |
| MELK | kinase | | 7.73E-03 | MCL1,MDM2 |
| KLF8 | transcription regulator | | 7.73E-03 | CCND1,EPSTI1 |
| BPI | transporter | | 7.73E-03 | CXCL8,ICAM1 |
| MTF2 | transcription regulator | | 7.73E-03 | KLF4,TBX3 |
| ZMIZ2 | transcription regulator | | 7.73E-03 | CCND1,MYC |
| Gata | group | | 7.73E-03 | EDN1,MAPK |
| sGC | complex | | 7.73E-03 | EGR1,FOS |
| [illegible] | other | | 7.73E-03 | BCL2,MYC |
| GPR68 | G-protein coupled receptor | | 7.73E-03 | DUSP1,PTGS2 |
| Vla-4 | complex | | 7.73E-03 | BCL2,PDGFB |
| TAF9 | transcription regulator | | 7.73E-03 | CXCL8,IRF1 |
| carvacrol | chemical - endogenous non-mammalian | | 7.73E-03 | BCL2,PTGS2 |
| CSF | group | | 7.73E-03 | C3,MCL1 |
| Mucin | group | | 7.73E-03 | CXCL8,PTGS2 |
| Pik3r | group | | 7.73E-03 | CXCL8,EPO |
| MAP4K1 | kinase | | 7.73E-03 | BCL2,BIRC3 |
| IL31RA | transmembrane receptor | | 7.73E-03 | CISH,SOCS3 |
| FZD7 | G-protein coupled receptor | | 7.73E-03 | CCND1,MYC |
| MCF2 | other | | 7.73E-03 | CCND1,ITGA2 |
| RNF34 | enzyme | | 7.73E-03 | BCL2,MDM2 |
| LOC102724788/PRODH | enzyme | | 7.73E-03 | PTGS2,TNFRSF10B |
| TLN1 | other | | 7.73E-03 | BCL2,MCL1 |
| APAF1 | other | | 7.73E-03 | BCL2,VIM |
| RBM14 | transcription regulator | | 7.73E-03 | CCND1,MYC |
| WNK4 | kinase | | 7.73E-03 | CFTR,WNK4 |
| PSAP | enzyme | | 7.73E-03 | BIRC3,CCND1 |
| KIDINS220 | transcription regulator | | 7.73E-03 | EGR1,FOS |
| CDH6 | other | | 7.73E-03 | MMP1,PTGS2 |
| NME3 | kinase | | 7.73E-03 | ITGA2,VIM |
| SH2B2 | other | | 7.73E-03 | FOS,MYC |
| SENP6 | peptidase | | 7.73E-03 | CXCL8,ICAM1 |
| PELI2 | enzyme | | 7.73E-03 | CXCL2,CXCL8 |
| [illegible] | transmembrane receptor | | 7.73E-03 | CCND1,CXCL2 |
| GCLM | enzyme | | 7.73E-03 | FOS,JUNB |
| STK17B | kinase | | 7.73E-03 | BCL2,CFLAR |
| 2-cyclohexen-1-one | chemical - endogenous non-mammalian | | 7.73E-03 | ICAM1,PTGS2 |
| mevalonolactone | chemical reagent | | 7.73E-03 | ICAM1,PTGS2 |
| lindane | chemical drug | | 7.73E-03 | BCL2,FOS |
| [illegible] | chemical drug | | 7.73E-03 | BCL2,PTGS2 |
| dimaprit | chemical drug | | 7.73E-03 | ICAM1,LDLR |
| butylated hydroxytoluene | chemical toxicant | | 7.73E-03 | FOS,PTGS2 |
| Collagen type V | complex | | 7.73E-03 | ITGA2,ITGA6 |
| [illegible] | chemical toxicant | | 7.73E-03 | TNFRSF10A,TNFRSF10B |
| cannabinoid drugs | chemical drug | | 7.73E-03 | CXCR4,ICAM1 |
| NADPH | chemical - endogenous mammalian | | 7.73E-03 | EPO,PTGS2 |
| miR-3103-5p (and other miRNAs w/seed GAGGGAG) | mature microRNA | | 7.76E-03 | [illegible] |
| mono-(2-ethylhexyl)phthalate | chemical toxicant | | 7.78E-03 | ACADSB,ACSL1,BCL2,CEBPA,CYP1A1,FOS,HK2,IER2,JUNB,LDLR,LRP1 |
| TNFRSF8 | transmembrane receptor | | 7.89E-03 | CXCL8,CXCR4,JUNB,MYC,NFKB2,RELB |
| TYROBP | transmembrane receptor | | 7.89E-03 | CXCL8,ICAM1,IL18,ITGA6,RBM47,SH2B3 |
| N-acetylmuramyl-L-alanyl-D-isoglutamine | chemical - endogenous non-mammalian | | 7.89E-03 | CXCL2,CXCL8,ETS1,FZD4,ICAM1,PTGS2 |
| BMPR2 | kinase | | 7.91E-03 | CXCL8,EFNB2,ICAM1,ID1 |
| TRIB3 | kinase | | 7.91E-03 | CEBPA,ID1,PSPH,STC2 |
| PRKAR2B | kinase | | 7.91E-03 | BIRC3,EGR1,FOS,NR4A1 |
| GRN | growth factor | | 7.91E-03 | BCL2,CCND1,CXCL2,ICAM1 |
| JAK inhibitor I | chemical - kinase inhibitor | | 7.91E-03 | C3,CXCL2,PTGS2,SOCS3 |
| imipramine | chemical drug | | 7.91E-03 | EGR1,FOS,HPN,TAGLN |
| hydrochloric acid | chemical - endogenous mammalian | | 7.91E-03 | CXCL2,CXCL8,FOS,ICAM1 |
| phorbol 12,13-didecanoate | chemical toxicant | | 7.91E-03 | DUSP1,MCL1,MMP1,MYC |
| SOX3 | transcription regulator | | 8.00E-03 | CDX2,DUSP1,DUSP4,DUSP6,EPHA2,IER5,INHBA |
| delta-9-tetrahydrocannabinol | chemical drug | | 8.00E-03 | CEBPA,CFLAR,CXCR4,DUSP6,FOS,SOCS3,TAP2 |
| miR-550a-3-5p (and other miRNAs w/seed UGUGCCU) | mature microRNA | | 8.06E-03 | [illegible] |
| miR-3150a-5p (and other miRNAs w/seed AACCUCG) | mature microRNA | | 8.09E-03 | [illegible] |
| AMPK | complex | | 8.10E-03 | CXCL8,DUSP4,EGR1,GSTK1,OAT,PTGS2,SERPINE1,SLC2A1,VIM |
| IRF4 | transcription regulator | | 8.16E-03 | ALPK2,BIRC3,CFLAR,CXCR4,ERBB3,IRF1,MYC,PLAUR,RHOB,SLC2A1,ZBTB20,ZFP36L1 |
| IRF1 | transcription regulator | | 8.16E-03 | BCL2,CCND1,CFTR,CXCL2,CXCL8,DST,IL18,IRF1,MYC,PTGS2,TAP2,TBX3 |
| metformin | chemical drug | | 8.16E-03 | ATF3,BCL2,CXCL8,FOS,GADD45B,ITGA6,MYC,NR4A1,PTGS2,SERPINE1,SLC22A1,SLC2A1 |
| NAMPT | cytokine | Activated | 8.20E-03 | CXCL2,CXCL8,EPHA2,ID1,MMP1 |
| NME1 | kinase | | 8.20E-03 | BCL2,CCND1,L1CAM,PTGS2,SERPINE1 |
| cyclic GMP | chemical - endogenous mammalian | | 8.20E-03 | BCL2,FOS,SERPINE1 |
| ADP | chemical - endogenous mammalian | | 8.20E-03 | CXCL8,FOS,SERPINE1 |
| NSD1 | transcription regulator | | 8.20E-03 | KLF6,ZFP36L1,ZMYM3 |
| IGFBP7 | transporter | | 8.20E-03 | CCND1,MYC,PTGS2 |
| AIMP1 | cytokine | | 8.20E-03 | CXCL2,CXCL8,ICAM1 |
| APBB1 | transcription regulator | | 8.20E-03 | CCND1,MYC,TAGLN |
| mir-322 | microRNA | | 8.20E-03 | BCL2,FGFR1,VIM |
| mir-196 | microRNA | | 8.20E-03 | BCL2,FOS,HOXB8 |
| CSK | kinase | | 8.20E-03 | FOS,SRC,TAGLN |
| PER2 | transcription regulator | | 8.20E-03 | CCND1,IHH,PER2 |
| VEGFC | growth factor | | 8.20E-03 | BCL2,CXCR4,PTGS2 |
| BDKRB2 | G-protein coupled receptor | | 8.20E-03 | EDN1,ICAM1,PTGS2 |
| HOXB4 | transcription regulator | | 8.20E-03 | FOSL1,JUNB,MYC |
| NRP1 | transmembrane receptor | | 8.20E-03 | BCL2,ID1,MCL1 |
| SP2 | transcription regulator | | 8.20E-03 | CCND1,FOS,SERPINE1 |
| TGFBR3 | kinase | | 8.20E-03 | ALDH1A1,BCL2,SERPINE1 |
| GTF2I | transcription regulator | | 8.20E-03 | CCND1,FOS,MYC |
| PTGFR | G-protein coupled receptor | | 8.20E-03 | FOS,NR4A1,PTGS2 |
| EDNRA | transmembrane receptor | | 8.20E-03 | FOS,ICAM1,PTGS2 |
| TOPBP1 | other | | 8.20E-03 | GADD45A,MDM2,MYC |
| dexmedetomidine | chemical drug | | 8.20E-03 | CXCL8,FOS,PTGS2 |
| hydroxyflutamide | chemical drug | | 8.20E-03 | BCL2,SULT1E1,UGDH |
| amlodipine | chemical drug | | 8.20E-03 | EDN1,LDLR,PTGS2 |
| Mn(III)-tetrakis (4-benzoic acid) porphyrin | chemical reagent | | 8.20E-03 | BCL2,MCL1,SERPINE1 |
| zinc protoporphyrin IX | chemical - endogenous mammalian | | 8.20E-03 | CCND1,FGFR2,PTGS2 |
| NUP98-KDM5A | fusion gene/product | | 8.20E-03 | BCL2,MCL1,MYC |
| NUP98-NSD1 | fusion gene/product | | 8.20E-03 | BCL2,MCL1,MYC |
| gamma-tocotrienol | chemical drug | | 8.20E-03 | CCND1,IL18,TNFAIP3 |
| miR-4708-3p (miRNAs w/seed GCAAGGC) | mature microRNA | | 8.26E-03 | [illegible] |
| miR-4777-3p (miRNAs w/seed UACCUCA) | mature microRNA | | 8.36E-03 | [illegible] |
| miR-512-5p (miRNAs w/seed ACUCAGC) | mature microRNA | | 8.36E-03 | [illegible] |
| CAMP | other | Activated | 8.52E-03 | BCL2,CXCL8,CXCR4,ICAM1,IL18,MCL1,PTGS2,TNFAIP3 |
| RHO | G-protein coupled receptor | | 8.53E-03 | DPYSL3,FOS,HPE,HK2,PDE6A,TNFAIP3 |
| CSHL1 | growth factor | | 8.53E-03 | CISH,FOS,IRF1,SLC22A1,SLC2A1,SOCS3 |
| miR-4706 (and other miRNAs w/seed GCGGGGA) | mature microRNA | | 8.53E-03 | [illegible] |
| JUND | transcription regulator | | 8.55E-03 | C3,CCND1,FOSL1,IL11,MMP1,NR4A1,PLAUR |
| dehydroisoandrosterone | chemical - endogenous mammalian | | 8.55E-03 | BCL2,CCND1,CYP1A1,ICAM1,MYC,TAP2,TMPRSS2 |
| miR-6732-3p (miRNAs w/seed AACCCUG) | mature microRNA | | 8.55E-03 | [illegible] |
| miR-100-3p (miRNAs w/seed AAGCUUG) | mature microRNA | | 8.60E-03 | [illegible] |
| ERN1 | kinase | | 8.64E-03 | ATF3,CXCL2,ETS1,MCL1,MPZL2,MVD,SPTLC3,TINAGL1,TMPRSS2,TNFRSF10B |
| streptozocin | chemical drug | | 8.69E-03 | ATF3,BCL2,CEBPA,COL4A1,DNMT3B,FOS,GATA4,GREM1,ICAM1,PTGS2,SERPINE1,SLC2A1,TXNIP,VIM |
| miR-1908 (and other miRNAs w/seed AAAGGCC) | mature microRNA | | 8.76E-03 | [illegible] |
| miR-216a-3p (and other miRNAs w/seed CACACUU) | mature microRNA | | 8.76E-03 | [illegible] |
| miR-7110-3p (miRNAs w/seed CUCUCUC) | mature microRNA | | 8.78E-03 | [illegible] |
| miR-1224-3p (miRNAs w/seed CCCACCU) | mature microRNA | | 8.81E-03 | [illegible] |
| miR-4366 (miRNAs w/seed UAGGAGG) | mature microRNA | | 8.89E-03 | [illegible] |
| miR-337-3p (and other miRNAs w/seed CAGCUCC) | mature microRNA | | 8.92E-03 | [illegible] |
| miR-4445-3p (and other miRNAs w/seed ACGGCAA) | mature microRNA | | 8.93E-03 | [illegible] |
| miR-3960 (and other miRNAs w/seed GCGGCGG) | mature microRNA | | 8.94E-03 | [illegible] |
| miR-128-1-5p (and other miRNAs w/seed GGGGCCG) | mature microRNA | | 8.94E-03 | [illegible] |
| ACTL6A | other | | 8.97E-03 | CCND1,MCL1,MYC,SPN |
| ROCK1 | kinase | | 8.97E-03 | CCND1,EDN1,ETS1,ICAM1 |
| [illegible] | other | | 8.97E-03 | G0S2,GPCPD1,HK2,SERPINE1 |
| PTCH1 | transmembrane receptor | | 8.97E-03 | CCND1,GADD45A,IGF2,MYCN |
| CFLAR | other | | 8.97E-03 | CFLAR,CXCL8,FOS,TNFRSF10B |
| EPHA5 | kinase | Inhibited | 9.01E-03 | DCBLD2,DUSP4,DUSP6,FOSL1,IRF1 |
| TXNIP | other | | 9.01E-03 | CCND1,G0S2,ICAM1,PTGS2,TXNIP |
| FLT3 | kinase | | 9.01E-03 | BCL2,ID1,MCL1,MYC,SLC2A1 |
| [illegible] | chemical drug | Inhibited | 9.01E-03 | DUSP1,EDN1,PTGS2,SERPINE1,TAGLN |
| PSEN2 | peptidase | | 9.02E-03 | ABCC6,ACSL1,BCL2,EMP1,FBXW7,FZD4,MTF1,PTGS2 |
| miR-5192 (miRNAs w/seed GGAGAGU) | mature microRNA | | 9.06E-03 | [illegible] |
| Rb | group | | 9.12E-03 | CCND1,CYP1A1,DLX1,DLX2,EPO,FGFR2,MYC |
| ascorbic acid | chemical - endogenous mammalian | | 9.12E-03 | CXCL8,IGFBP4,ITGA2,NDRG1,PTGS2,SERPINE1,SLC2A1 |
| miR-1183-3p (and other miRNAs w/seed GAGGCUC) | mature microRNA | | 9.14E-03 | [illegible] |
| miR-22-3p (miRNAs w/seed AGCUGCC) | mature microRNA | | 9.19E-03 | [illegible] |
| miR-708 (miRNAs w/seed CAGAGUG) | mature microRNA | | 9.20E-03 | [illegible] |
| Fcer1 | complex | Activated | 9.20E-03 | CFLAR,CXCL8,DUSP6,FOS,INHBA,LIF |
| STING1 | other | Activated | 9.20E-03 | CGAS,CXCL2,CXCL8,LIF,PPP1R15A,UPP1 |
| HOXD10 | transcription regulator | | 9.20E-03 | GCNT1,ICAM1,ITGA3,PAK1,RHOB,SERPINE1 |
| NRIP1 | transcription regulator | | 9.20E-03 | AREG,CCND1,CYP1A1,PTGS2,[illegible],SUOX |
| glucosamine | chemical - endogenous mammalian | | 9.20E-03 | BCL2,CEBPA,CXCL8,ICAM1,PTGS2,TXNIP |
| miR-6281 (miRNAs w/seed GGUCCCG) | mature microRNA | | 9.36E-03 | [illegible] |
| miR-6426 (miRNAs w/seed AAGGAGA) | mature microRNA | | 9.36E-03 | [illegible] |
| miR-1268-5p (and other miRNAs w/seed GCUGAGC) | mature microRNA | | 9.36E-03 | [illegible] |
| miR-1254-3p (and other miRNAs w/seed CGGCCUG) | mature microRNA | | 9.36E-03 | [illegible] |
| miR-708-5p (and other miRNAs w/seed AGGAGCU) | mature microRNA | | 9.47E-03 | [illegible] |
| miR-3140-5p (miRNAs w/seed CCUGAAU) | mature microRNA | | 9.57E-03 | [illegible] |
| miR-6872-3p (miRNAs w/seed CCAUGCC) | mature microRNA | Activated | 9.67E-03 | [illegible] |
| miR-4445-5p (miRNAs w/seed GAUGUAU) | mature microRNA | | 9.67E-03 | [illegible] |
| GMNN | transcription regulator | | 9.72E-03 | CDX2,DUSP1,DUSP4,DUSP6,EPHA2,IER5,INHBA |
| TRAF2 | enzyme | | 9.72E-03 | BIRC3,CFLAR,CXCL8,EFNA1,ICAM1,NDRG1,TNFAIP3 |
| miR-4326 (miRNAs w/seed GUUCCUC) | mature microRNA | | 9.76E-03 | [illegible] |
| miR-6810-5p (and other miRNAs w/seed UGGGGAC) | mature microRNA | | 9.81E-03 | [illegible] |
| miR-644a (and other miRNAs w/seed GUGUGGC) | mature microRNA | Activated | 9.86E-03 | [illegible] |
| 1,25-dihydroxyvitamin D | chemical drug | | 9.86E-03 | CXCL8,EGR1,FGFR1,GLUL,IGF2 |
| Wnt | group | | 9.86E-03 | CDX2,CEBPA,GATA4,MYC,SDC2 |
| mir-155 | microRNA | | 9.86E-03 | CXCL8,DUSP1,MYC,PTGS2,RUNX1 |
| MEF2A | transcription regulator | | 9.86E-03 | EDN1,FOS,ITGA7,NR4A1,TGFB3 |
| RIPK1 | kinase | Activated | 9.86E-03 | CFLAR,CXCL2,CXCL8,MDM2,PDGFB |
| prostaglandin A2 | chemical - endogenous non-mammalian | | 9.92E-03 | CCND1,ICAM1,LPAR6 |
| epothilone B | chemical drug | | 9.92E-03 | MCL1,MDM2,SPN |

| | | | | |
|---|---|---|---|---|
| C1q | complex | | 9.92E-03 | CXCL8,GAB6,ICAM1 |
| COL1A1 | other | | 9.92E-03 | CXCR4,EDN1,ICAM1 |
| GADL3 | other | | 9.92E-03 | LIF,PLK2,TMEPF1 |
| HIF1AN | enzyme | | 9.92E-03 | EPO,HK2,SLC2A1 |
| UBD | other | | 9.92E-03 | ACOT1,ACSL1,HOXB9 |
| TGIF1 | transcription regulator | | 9.92E-03 | CXCL2,CXCL8,SOAT2 |
| LY96 | transmembrane receptor | | 9.92E-03 | CXCL2,CXCL8,MMP1 |
| IGFBP1 | other | | 9.92E-03 | BCL2,ITGA2,ITGA5 |
| CXCL2 | cytokine | | 9.92E-03 | CXCL2,ICAM1,IPRD1 |
| IL2RB | transmembrane receptor | | 9.92E-03 | ETS1,FOS,MYC |
| BHLHA15 | transcription regulator | | 9.92E-03 | ARRDC3,DJB1,RAB3D |
| STMN1 | other | | 9.92E-03 | BCL2,CFLAR,EGR1 |
| 4-nitroquinoline-1-oxide | chemical toxicant | | 9.92E-03 | EGR1,MDM2,PTGS2 |
| Hif | complex | | 9.92E-03 | CXCL8,EPO,L1CAM |
| mevastatin | chemical drug | | 9.92E-03 | KLF4,LDLR,PTGS2 |
| indole | chemical - endogenous mammalian | | 9.92E-03 | CXCL8,CYP1A1,TJP3 |
| farnesyl pyrophosphate | chemical - endogenous mammalian | | 9.92E-03 | ICAM1,PTGS2,SERPINE1 |
| caffeic acid | chemical drug | | 9.92E-03 | BCL2,CXCL8,PTGS2 |
| reserpine | chemical drug | | 9.92E-03 | EGR1,FOS,IL18 |
| lithium chloride | chemical drug | | 9.92E-03 | BCL2,CCND1,FOS,GATA4,MYC,NOTCH3,PTGS2,TAGLN,TNFRSF10B |
| miR-6884-5p (and other miRNAs w/seed GUGUGUG) | mature microRNA | | 1.00E-02 | [illegible] |
| miR-6868-3p (miRNAs w/seed UCCUUCU) | mature microRNA | | 1.01E-02 | [illegible] |
| indican | chemical - endogenous mammalian | | 1.01E-02 | CXCR4,ICAM1,PTGS2,SLC2A1 |
| TCF7L1 | transcription regulator | | 1.01E-02 | ATF3,KLF4,SLC26A2,TBX3 |
| leucine | chemical - endogenous mammalian | | 1.01E-02 | ATF3,FOS,NR4A1,PPP1R15A |
| FGFR4 | kinase | | 1.01E-02 | CCND1,FOS,MMP1,SERPINE1 |
| C1QA | other | | 1.01E-02 | C3,EGR1,SERPINE1,VIM |
| SIAH2 | transcription regulator | | 1.01E-02 | AKR1C3,NDRG1,PLK3,TMPRSS2 |
| mir-135 | microRNA | | 1.01E-02 | EDN1,IL11,KLF4,MCL1 |
| LTB4R | G-protein coupled receptor | Activated | 1.01E-02 | CXCL2,CXCL8,LDLR,PTGS2 |
| N(G)-monomethyl-D-arginine | chemical - endogenous mammalian | | 1.01E-02 | EPO,FOS,PLAUR,PTGS2 |
| miR-892a (miRNAs w/seed ACUGGCU) | mature microRNA | | 1.02E-02 | [illegible] |
| miR-2114-5p (miRNAs w/seed AGUCCCU) | mature microRNA | | 1.02E-02 | [illegible] |
| CFTR | ion channel | | 1.04E-02 | CFTR,CXCL8,EPHX2,NFKBIZ,PTGS2,SOCS3,TNFAIP3 |
| fluoxetine | chemical drug | | 1.04E-02 | EGR1,FOS,GNAI1,HPN,MYC,SERPINA3,TAGLN |
| spironolactone | chemical drug | | 1.04E-02 | BCL2,CXCR4,EDN1,ICAM1,PTGS2,SLCO2B1,ULBP2 |
| miR-6761-3p (miRNAs w/seed CGGCUCU) | mature microRNA | | 1.06E-02 | [illegible] |

[illegible]

| | | | | |
|---|---|---|---|---|
| PHLPP1 | enzyme | | 1,86E-02 | CXCL2,IL18,MCL1 |
| CDK5R1 | kinase | | 1,86E-02 | CXCL8,FOS,SERPINE1 |
| mir-101 | microRNA | | 1,86E-02 | DUSP1,PTGS2,RUNX1 |
| mir-218 | microRNA | | 1,86E-02 | FZD4,SRC,VAV3 |
| MLX | transcription regulator | | 1,86E-02 | DIO1,IGF2,TXNIP |
| AURKB | kinase | | 1,86E-02 | ATF3,DDB2,IER3 |
| IAPP | other | | 1,86E-02 | FOS,MVD,MYC |
| LY6E | other | | 1,86E-02 | CXCL8,RELB,SERPINE1 |
| RPA1 | other | | 1,86E-02 | LRRN4,NAV2,TPD52L1 |
| pevonedistat | chemical drug | | 1,86E-02 | CFLAR,RHOB,TNFRSF10B |
| HULC | other | | 1,86E-02 | BCL2,CDKN2C,VIM |
| ammonium chloride | chemical drug | | 1,86E-02 | EDN1,LDLR,ULBP2 |
| EML4-ALK | fusion gene/product | | 1,86E-02 | ERBB3,SERPINE1,TGFB3 |
| farnesol | chemical reagent | | 1,86E-02 | ATF3,PHLDA1,PHLDA2 |
| naloxone | chemical drug | | 1,86E-02 | BCL2,FOS,NR4A1 |
| miR-551b-3p (and other miRNAs w/seed CGACCCA) | mature microRNA | | 1,88E-02 | AEN,BCL2,DENND11,ICAM4,IER3,MAFG,SOCS3,SOWAHC,TMEM141,UPB1,ZFP36 |
| miR-139-5p (miRNAs w/seed CUACAGU) | mature microRNA | | 1,88E-02 | [illegible] |
| miR-3065-3p (miRNAs w/seed CAGCACC) | mature microRNA | | 1,88E-02 | [illegible] |
| miR-3649 (miRNAs w/seed GGGACCU) | mature microRNA | | 1,88E-02 | [illegible] |
| IL12 (family) | group | | 1,88E-02 | ATF3,BCL2,CFLAR,EGR2,IRF1,LIF,MYC,NFKBIZ,ULBP2 |
| LMNA | other | | 1,88E-02 | BCL2,CEBPA,DUSP4,EDN1,FBXW7,GATA4,IER5,IRF1,MAFF,MXD4,NUFIP2,SLC26A2,TUBB3,ZFP36 |
| miR-6772-3p (miRNAs w/seed UGCUCCU) | mature microRNA | | 1,88E-02 | [illegible] |
| CEBPE | transcription regulator | | 1,89E-02 | BCL2,F7,IL18,MYC,PTGS2 |
| LPL | enzyme | Inhibited | 1,89E-02 | BCL2,CXCL8,ICAM1,LDLR,SLC2A1 |
| [illegible] | chemical drug | | 1,89E-02 | ATF3,BHLHE40,SERPINE1,SLC2A1,TGM2 |
| arginine | chemical - endogenous mammalian | | 1,90E-02 | BCL2,EGR1,MYC,PTGS2 |
| CASP1 | peptidase | | 1,90E-02 | CXCL2,IL18,PTGS2,VIM |
| IGFBP3 | other | | 1,90E-02 | BCL2,ICAM1,SDC2,SERPINE1 |
| NLRP12 | other | | 1,90E-02 | CXCL2,CXCL8,CXCR4,ICAM1 |
| rolipram | chemical drug | | 1,90E-02 | AREG,CXCR4,DUSP1,PTGS2 |
| PML-RARA | fusion gene/product | | 1,90E-02 | EGR1,FOS,MYC,RARA |
| exenatide | biologic drug | | 1,90E-02 | FOS,ICAM1,PTGS2,TXNIP |
| miR-6877-5p (miRNAs w/seed GGGCCGA) | mature microRNA | | 1,92E-02 | [illegible] |
| miR-182-5p (and other miRNAs w/seed UUGGCAA) | mature microRNA | | 1,92E-02 | [illegible] |
| CALCA | other | | 1,94E-02 | ADD3,CXCL8,CXCR4,EDN1,ESAM,FOS,JUNB |
| melatonin | chemical - endogenous mammalian | | 1,94E-02 | BCL2,CXCL8,EDN1,FOS,JUNB,PER2,PTGS2,VIM |
| 5-hydroxytryptamine | chemical - endogenous mammalian | Activated | 1,94E-02 | CXCL8,EGR1,FOS,ICAM1,MYC,NR4A1,PTGS2,SERPINE1 |
| miR-4710 (miRNAs w/seed GGUGAGG) | mature microRNA | | 1,96E-02 | [illegible] |
| geldanamycin | chemical - endogenous non-mammalian | Inhibited | 1,96E-02 | BCL2,CCND1,CDKN2C,CFLAR,CXCL8,CYP1A1,FGFR1,IRF1,LIF,MDM2,MYC,PAK1,PDCD4,PTGS2,SNX33 |
| miR-452-3p (miRNAs w/seed UCAUCUG) | mature microRNA | | 1,96E-02 | [illegible] |
| miR-203a-3p (and other miRNAs w/seed UGAAAUG) | mature microRNA | | 1,96E-02 | DUSP6,FOS,IL11,INPP1,KLF4,RASSF6,RGN,RNASE4,SOCS3,SRC,SULT1E1,TPD52L1 |
| miR-3613-3p (miRNAs w/seed CAAAAAA) | mature microRNA | Inhibited | 1,96E-02 | [illegible] |
| SOX11 | transcription regulator | | 1,96E-02 | [illegible] |
| miR-6511a-3p (and other miRNAs w/seed CUCACCA) | mature microRNA | | 1,99E-02 | [illegible] |
| UCP1 | transporter | | 2,00E-02 | ANKRD1,GADD45A,MYC,PHGDH,PSPH,REEP6,SERPINE1,SMTNL2,SOAT2,[illegible] |
| miR-421-3p (and other miRNAs w/seed UCAACAG) | mature microRNA | | 2,00E-02 | [illegible] |
| miR-4335 (miRNAs w/seed UGGACCU) | mature microRNA | | 2,01E-02 | [illegible] |
| HDL | complex | | 2,04E-02 | ATF3,CXCL8,ICAM1,LDLR,PTGS2 |
| FGF10 | growth factor | | 2,04E-02 | FGFR2,HSD3B1,LDLR,PTGS2,SPRY2 |
| CD5 | transmembrane receptor | | 2,04E-02 | BCL2,ICAM1,LDLR,NR4A1,RELB |
| enalapril | biologic drug | | 2,04E-02 | FOS,IER3,MYC,SERPINE1,TNFRSF12A |
| EHF | transcription regulator | | 2,04E-02 | CYP1A1,MMP1,NOTCH3,PLAUR,SERPINE1,TNFRSF10B,ZFP36L1 |
| miR-4767-5p (and other miRNAs w/seed GGGAAGG) | mature microRNA | | 2,04E-02 | [illegible] |
| miR-6886 (miRNAs w/seed AUCUGCU) | mature microRNA | | 2,04E-02 | [illegible] |
| 15-epi-lipoxin A4 | chemical - endogenous mammalian | | 2,06E-02 | CXCL8,TNFAIP3 |
| UDP | chemical - endogenous mammalian | | 2,06E-02 | CXCL8,SERPINE1 |
| saikosaponin A | chemical reagent | | 2,06E-02 | FOS,JUNB |
| rhodamine 6G | chemical toxicant | | 2,06E-02 | BCL2,CCND1 |
| 2,5-bis(5-hydroxymethyl-2-thienyl)furan | chemical reagent | | 2,06E-02 | GADD45A,MDM2 |
| L-type Calcium Channel | complex | | 2,06E-02 | EGR1,FOS |
| SENP7 | peptidase | | 2,06E-02 | CXCL8,VIM |
| GSTK1 | enzyme | | 2,06E-02 | [illegible],IL18 |
| HJV | other | | 2,06E-02 | ID1,SLC40A1 |
| DUOXA1 | other | | 2,06E-02 | TUBB3,VIM |
| SULT2B1 | enzyme | | 2,06E-02 | BCL2,MYC |
| ZNF382 | transcription regulator | | 2,06E-02 | ID1,MYC |
| CYSLTR2 | G-protein coupled receptor | | 2,06E-02 | CXCL8,EGR1 |
| IL-2R | complex | | 2,06E-02 | FOS,PHGDH |
| IL36 | cytokine | | 2,06E-02 | CXCL8,ICAM1 |
| parathyroid hormone-related protein | biologic drug | | 2,06E-02 | FOS,SLC9A3R1 |
| LGI1 | other | | 2,06E-02 | MMP1,MMP11 |
| HDAC10 | transcription regulator | | 2,06E-02 | NR4A1,TXNIP |
| [illegible] | enzyme | | 2,06E-02 | SERPINE1,SESN1 |
| SLC25A4 | transporter | | 2,06E-02 | BIRC3,MCL1 |
| RGS19 | enzyme | | 2,06E-02 | GATA4,MYC |
| GAL3ST1 | enzyme | | 2,06E-02 | ITGA5,ITGA6 |
| CYSLTR1 | G-protein coupled receptor | | 2,06E-02 | BCL2,FOS |
| HS6ST1 | enzyme | | 2,06E-02 | CXCL8,SPRY2 |
| GRIP1 | transcription regulator | | 2,06E-02 | FRAS1,PTGS2 |
| EFEMP1 | enzyme | | 2,06E-02 | SERPINE1,VIM |
| USP2 | peptidase | | 2,06E-02 | CFLAR,SERPINE1 |
| MAOA | enzyme | | 2,06E-02 | BCL2,CCND1 |
| ARAF | kinase | | 2,06E-02 | CCND1,FOS |
| HEYL | transcription regulator | | 2,06E-02 | GATA4,TMPRSS2 |
| ATF7 | transcription regulator | | 2,06E-02 | DUSP1,DUSP6 |
| WNT7B | other | | 2,06E-02 | CCND1,MYC |
| MAP3K10 | kinase | | 2,06E-02 | CXCL8,SH3RF1 |
| GRB10 | other | | 2,06E-02 | FOS,TGM2 |
| TBL1X | transcription regulator | | 2,06E-02 | CXCL2,DIO1 |
| SNW1 | transcription regulator | | 2,06E-02 | CCND1,SERPINE1 |
| PROKR1 | G-protein coupled receptor | | 2,06E-02 | MYC,VIM |
| TSPO | transmembrane receptor | | 2,06E-02 | BCL2,ICAM1 |
| CCDC80 | other | | 2,06E-02 | CCND1,CEBPA |
| DIABLO | other | | 2,06E-02 | BIRC3,CFLAR |
| SERPINH1 | other | | 2,06E-02 | SERPINE1,VIM |
| PCBP1 | translation regulator | | 2,06E-02 | INHBA,MYC |
| TBL1XR1 | transcription regulator | | 2,06E-02 | CXCL2,DIO1 |
| proteasome inhibitor | chemical drug | | 2,06E-02 | CFLAR,MYC |
| naproxen | chemical drug | | 2,06E-02 | CCND1,PTGS2 |
| raclopride | chemical drug | | 2,06E-02 | FOS,NR4A1 |
| NCX-4040 | chemical drug | | 2,06E-02 | CYP1A1,PTGS2 |
| domoic acid | chemical toxicant | | 2,06E-02 | FOS,PTGS2 |
| pranlukast | chemical drug | | 2,06E-02 | BCL2,EGR1 |
| vandetanib | chemical drug | | 2,06E-02 | CCND1,CXCR4 |
| carnosol | chemical - endogenous non-mammalian | | 2,06E-02 | ICAM1,PTGS2 |
| [illegible] | chemical drug | | 2,06E-02 | CFLAR,CXCL2 |
| artemisinin | chemical drug | | 2,06E-02 | CCND1,MDM2 |
| citric acid | chemical - endogenous mammalian | | 2,06E-02 | BCL2,MCL1 |
| alpha-tocopherol succinate | chemical drug | | 2,06E-02 | BCL2,EGR1 |
| cordycepin | chemical drug | | 2,06E-02 | FOS,MYC |
| miR-3158-3p (miRNAs w/seed UCCCACU) | mature microRNA | | 2,06E-02 | [illegible] |
| Bvht | other | | 2,06E-02 | CDK2,ETS1,GATA4,HOXB6,IGF2,VIM |
| PTTG1 | transcription regulator | | 2,06E-02 | BCL2,CCND1,MDM2,MYC,[illegible],VIM |
| TFAP2C | transcription regulator | | 2,06E-02 | CDK2,FBXW7,FMN1,FOXA1,MYC,TNFAIP3 |
| miR-760-3p (and other miRNAs w/seed GGCUCUG) | mature microRNA | | 2,08E-02 | [illegible] |
| miR-370-5p (and other miRNAs w/seed AGGUCAC) | mature microRNA | | 2,08E-02 | [illegible] |
| 3M-001 | chemical drug | | 2,09E-02 | BCL2,CXCL8,MYC,PTGS2 |
| TRH | other | | 2,09E-02 | DUSP1,FOS,JUNB,NR4A1 |
| PLP1 | other | | 2,09E-02 | ATF3,CXCL2,NOTCH3,PPP1R15A |
| GNB2 | enzyme | | 2,09E-02 | DUSP1,IER3,NR4A1,PLK2 |
| CUL4B | other | | 2,09E-02 | CABLES2,COL4A1,RUNX1,ZFP36 |
| RAD21 | transcription regulator | | 2,09E-02 | IGF2,ITGA5,MYC,VIM |
| miR-6850-5p (miRNAs w/seed CAGGCCU) | mature microRNA | | 2,09E-02 | [illegible] |
| miR-4726-3p (and other miRNAs w/seed UACCUUC) | mature microRNA | | 2,09E-02 | [illegible] |
| miR-1262-5p (miRNAs w/seed GAAGGAA) | mature microRNA | | 2,09E-02 | [illegible] |
| miR-1237-3p (and other miRNAs w/seed CCUUCUG) | mature microRNA | Inhibited | 2,10E-02 | [illegible] |
| DNA-methyltransferase | group | | 2,12E-02 | EPHX2,MMP1,PTGS2 |
| Lymphotoxin | complex | | 2,12E-02 | CXCL8,ICAM1,MMP1 |
| TNFRSF18 | transmembrane receptor | | 2,12E-02 | CXCL8,ICAM1,PTGS2 |
| YWHAZ | enzyme | | 2,12E-02 | FOSL1,MCL1,MMP1 |
| FBN1 | other | | 2,12E-02 | INHBA,MMP1,SERPINE1 |
| HDAC7 | transcription regulator | | 2,12E-02 | CCND1,EDN1,NR4A1 |
| NTN1 | growth factor | | 2,12E-02 | CXCL2,L1CAM,PTGS2 |
| SOCS6 | other | | 2,12E-02 | CXCL2,IL18,PTGS2 |
| PPP4R3A | other | | 2,12E-02 | DLX1,DLX2,TUBB3 |
| A-Fos | chemical reagent | | 2,12E-02 | DUSP6,IER3,MYC |
| PARK7 | enzyme | | 2,12E-02 | CXCL8,ICAM1,VIM |
| SCGB1A1 | cytokine | | 2,12E-02 | C3,COL4A1,PTGS2 |
| pilocarpine | chemical drug | | 2,12E-02 | EDN1,FOS,JUNB |
| nickel | chemical toxicant | | 2,12E-02 | CXCL8,ICAM1,SERPINE1 |
| miR-505-3p (miRNAs w/seed UCAACAC) | mature microRNA | | 2,14E-02 | CXCL8,DDIAS,GADD45A,GATM,GSTA2,IDH1,MPP1,RGS2,TPD52L1,YPEL5,ZNF441 |
| miR-4300 (and other miRNAs w/seed GGGAGCU) | mature microRNA | | 2,14E-02 | [illegible] |
| Ifn gamma | complex | | 2,14E-02 | CXCL8,EDN1,ICAM1,IGFBP4,LIF,MYC,SOCS3 |
| miR-196a-3p (miRNAs w/seed GGCAACA) | mature microRNA | | 2,16E-02 | [illegible] |
| miR-338-3p (miRNAs w/seed CCAGCAU) | mature microRNA | | 2,16E-02 | [illegible] |
| cholic acid | chemical - endogenous mammalian | | 2,17E-02 | CDK2,CYP27A1,EHHADH,FBP1,LRP2,TAGLN |
| ATG7 | enzyme | | 2,17E-02 | ACSL1,CXCL8,HK2,MYC,SLC2A1,SOCS3 |
| miR-3612 (and other miRNAs w/seed GGAGGCA) | mature microRNA | | 2,17E-02 | [illegible] |
| ACSL4 | enzyme | | 2,18E-02 | CFLAR,FERMT1,ICAM1,PTGS2,[illegible] |
| BIRC6 | other | | 2,18E-02 | BCL2,ITGA5,MCL1,MDM2,VIM |
| CLEC11A | growth factor | | 2,18E-02 | BCL2,EGR2,FHL2,FOS,NR4A1 |
| miR-4713-3p (miRNAs w/seed GGGAUCC) | mature microRNA | | 2,20E-02 | [illegible] |
| miR-571 (miRNAs w/seed GAGUUGG) | mature microRNA | | 2,26E-02 | [illegible] |
| eicosapentaenoic acid | chemical drug | | 2,26E-02 | FOS,LDLR,MMP1,PTGS2,SERPINE1,SLC2A1,TGFB3 |
| miR-675-5p (and other miRNAs w/seed GGUGCGG) | mature microRNA | | 2,26E-02 | [illegible] |
| 3-methyladenine | chemical toxicant | | 2,26E-02 | BCL2,CXCL2,CXCL8,PTGS2 |
| CERK | kinase | | 2,26E-02 | CLCF1,CXCL2,CXCL8,CXCR4 |
| [illegible] | enzyme | | 2,26E-02 | BCL2,CXCR4,FOS,GADD45A |
| TSLP | cytokine | | 2,26E-02 | CISH,CXCL8,ICAM1,MYC |
| SERPINE1 | other | | 2,26E-02 | CXCL2,CXCL8,FOS,PLAUR |
| SPRY2 | other | | 2,26E-02 | EPO,ETS1,FOS,SLC2A1 |
| SAA1 | transporter | | 2,26E-02 | BCL2,CCND1,CEBPA,CXCL8 |
| MMP2 | peptidase | | 2,26E-02 | CCND1,ITGA3,MMP1,MYC |
| cilostazol | chemical drug | | 2,26E-02 | BCL2,ICAM1,MMP1,SERPINE1 |
| steroid | chemical - endogenous mammalian | | 2,26E-02 | CEBPA,FOS,MYC,SPEN |
| GATA1 | transcription regulator | | 2,30E-02 | BCL2,CCND1,CDKN2C,CEBPA,EGR1,EPO,ETS1,ITGA6,JUNB,MAGED1,MYC,MYCN,PBXIP1,SRC,TGM2 |
| miR-7106-3p (miRNAs w/seed GCUCCCU) | mature microRNA | | 2,30E-02 | [illegible] |
| cerivastatin | chemical drug | | 2,30E-02 | CXCL8,ICAM1,MYC,PLAUR,PTGS2,SERPINE1 |
| [illegible] (and other miRNAs w/seed CUGGUCC) | mature microRNA | | 2,30E-02 | [illegible] |
| miR-1287-5p (miRNAs w/seed GCUGGAU) | mature microRNA | | 2,31E-02 | [illegible] |
| miR-7152-5p (miRNAs w/seed UUCCUGU) | mature microRNA | | 2,31E-02 | [illegible] |
| [illegible] (and other miRNAs w/seed UCUGCUG) | mature microRNA | | 2,32E-02 | [illegible] |
| EFNA2 | kinase | | 2,34E-02 | DCBLD2,DUSP4,DUSP6,FOSL1,RUNX1 |
| miR-6809-3p (miRNAs w/seed GACCCCC) | mature microRNA | | 2,37E-02 | [illegible] |
| ETV5 | transcription regulator | | 2,37E-02 | CCND1,CLDN3,CXCR4,ICAM1,ITGA5,TINAGL1,TJP3 |
| miR-3913-3p (miRNAs w/seed GACAUCA) | mature microRNA | | 2,38E-02 | [illegible] |
| miR-6769a-3p (miRNAs w/seed AGCCCCU) | mature microRNA | Activated | 2,38E-02 | [illegible] |
| CaMKII | complex | | 2,40E-02 | BIRC3,FOS,IRF1 |
| Tnf receptor | group | | 2,40E-02 | C3,CXCL8,SERPINE1 |
| CCK | other | | 2,40E-02 | CFLAR,EGR1,FOS |
| NFIX | transcription regulator | | 2,40E-02 | CCND1,CXCL8,SERPINE1 |
| ITGA2 | transmembrane receptor | | 2,40E-02 | ITGA2,ITGA5,MMP1 |
| KIF3A | enzyme | | 2,40E-02 | IHH,MVD,MYC |
| CXCR3 | G-protein coupled receptor | | 2,40E-02 | BCL2,CFTR,MMP1 |
| TERF2IP | other | | 2,40E-02 | CXCL8,IGF2,SMTNL2 |
| PDGFC | growth factor | | 2,40E-02 | CCND1,MYC,NR4A1 |
| HSP90AB1 | enzyme | | 2,40E-02 | ATF3,CFLAR,MYC |
| alendronic acid | chemical drug | | 2,40E-02 | BCL2,CXCL8,ID1 |
| GLP-1-(7-34)-amide | biologic drug | | 2,40E-02 | BCL2,FOS,NR4A1 |
| wogonin | chemical - endogenous non-mammalian | | 2,40E-02 | BCL2,CFLAR,FBXW7 |
| [illegible] (and other miRNAs w/seed AGGGCAG) | mature microRNA | | 2,42E-02 | [illegible] |
| ZBTB16 | transcription regulator | | 2,45E-02 | [illegible] |
| miR-876-3p (miRNAs w/seed [illegible]) | mature microRNA | Activated | 2,45E-02 | [illegible] |
| miR-6868-5p (miRNAs w/seed CUGGCAG) | mature microRNA | | 2,45E-02 | [illegible] |
| [illegible] (miRNAs w/seed CCUGUUC) | mature microRNA | | 2,45E-02 | [illegible] |
| miR-182-3p (miRNAs w/seed GGUUCUA) | mature microRNA | | 2,47E-02 | [illegible] |
| miR-1343-3p (and other miRNAs w/seed UCCUGGG) | mature microRNA | | 2,48E-02 | [illegible] |
| SAA | group | | 2,48E-02 | CXCL8,ICAM1,MMP1,PTGS2 |
| CYLD | transcription regulator | | 2,48E-02 | CXCL8,NFKB2,RELB,SERPINE1 |
| GNB1 | enzyme | | 2,48E-02 | DUSP1,IER3,NR4A1,PLK2 |
| NR2F1 | ligand-dependent nuclear receptor | | 2,48E-02 | EGR1,EPO,SPRY1,SPRY2 |
| PRKN | enzyme | | 2,48E-02 | CCND1,MCL1,MYC,PLK2 |
| CGS 21680 | chemical reagent | Inhibited | 2,48E-02 | EGR1,FOS,PTGS2,RGS2 |
| bromocriptine | chemical drug | Inhibited | 2,48E-02 | BCL2,GNAI1,IHH,SLC2A1 |
| miR-567 (miRNAs w/seed GUAUGUU) | mature microRNA | | 2,49E-02 | [illegible] |
| miR-1470 (miRNAs w/seed CCCUCCG) | mature microRNA | | 2,49E-02 | [illegible] |
| miR-143-3p (and other miRNAs w/seed GAGAUGA) | mature microRNA | | 2,49E-02 | [illegible] |
| trichloroethylene | chemical toxicant | | 2,50E-02 | EGR1,MDM2,RHOB,SLC9A3R1,TXNIP |
| ZEB2 | transcription regulator | | 2,50E-02 | CCND1,EHMT2,GATM,MMP1,VIM |
| CCL11 | cytokine | | 2,50E-02 | BCL2,CXCL2,CXCL8,ICAM1,ITGA2 |
| benzene | chemical toxicant | | 2,50E-02 | EGR1,MDM2,RHOB,SLC9A3R1,TXNIP |
| CpG ODN 1668 | chemical reagent | Activated | 2,50E-02 | CFLAR,CXCL8,ICAM1,PTGS2,SOCS3 |
| miR-544b (miRNAs w/seed CCUGAGG) | mature microRNA | | 2,52E-02 | [illegible] |
| miR-8063 (miRNAs w/seed GGCGAUU) | mature microRNA | | 2,52E-02 | [illegible] |
| miR-3667-3p (miRNAs w/seed CCUUCCU) | mature microRNA | | 2,52E-02 | [illegible] |
| CLDN7 | other | | 2,53E-02 | C3,CXCL8,FOSL1,MAGED1,MUC13,PHGDH,SOSTDC1,TINAGL1 |
| miR-340 (and other miRNAs w/seed [illegible]) | mature microRNA | | 2,54E-02 | [illegible] |
| miR-3940-3p (miRNAs w/seed AGCCCGG) | mature microRNA | | 2,54E-02 | [illegible] |
| [illegible] (and other miRNAs w/seed GAUCAGG) | mature microRNA | | 2,57E-02 | [illegible] |
| ganglioside | chemical - endogenous mammalian | | 2,58E-02 | ICAM1,PTGS2 |
| novobiocin | chemical drug | | 2,58E-02 | HRH1,SRC |
| cholesterol sulfate | chemical - endogenous mammalian | | 2,58E-02 | FOSL1,NR1D1 |
| NRDE2 | other | | 2,58E-02 | APOL1,IL18 |
| Agtr1a | G-protein coupled receptor | | 2,58E-02 | FOS,PTGS2 |
| atrazine | chemical toxicant | | 2,58E-02 | GATA4,PTGS2 |
| 4-nonylphenol | chemical toxicant | | 2,58E-02 | FOS,MYC |
| Pde | group | | 2,58E-02 | FOS,NR4A1 |
| Bcl9-Cbp/p300-Ctnnb1-Lef/Tcf | complex | | 2,58E-02 | CCND1,MYC |
| BMS-387032 | chemical drug | | 2,58E-02 | CCND1,MCL1 |
| FBXO42 | other | | 2,58E-02 | CXCL8,ICAM1 |
| PHF8 | enzyme | | 2,58E-02 | ID1,TGM2 |
| AFF4 | transcription regulator | | 2,58E-02 | MYC,STC2 |
| CTDSP1 | phosphatase | | 2,58E-02 | MYC,VIM |
| NEK7 | kinase | | 2,58E-02 | EDN1,PTGS2 |
| EGLN2 | enzyme | | 2,58E-02 | CXCL8,EPO |
| LRRC32 | other | | 2,58E-02 | TGFB3,VIM |
| HUWE1 | transcription regulator | | 2,58E-02 | CDKN2C,MYC |
| SMAD1/5 | group | | 2,58E-02 | ID1,PLAUR |
| SPTAN1 | other | | 2,58E-02 | ITGA3,ITGA5 |
| pictilisib | chemical drug | | 2,58E-02 | CXCL8,SLC2A1 |
| CD244 | transmembrane receptor | | 2,58E-02 | EGR1,FOS |
| CTNND1 | other | | 2,58E-02 | CXCL8,VIM |
| PRTN3 | peptidase | | 2,58E-02 | CXCL8,IL18 |
| ATG16L1 | enzyme | | 2,58E-02 | CXCL2,IL18 |
| DKK2 | other | | 2,58E-02 | BCL2,CCND1 |
| GUSB | enzyme | | 2,58E-02 | APBB1,EGR2 |
| salinomycin | chemical - endogenous non-mammalian | | 2,58E-02 | CCND1,MCL1 |
| PTGES3 | enzyme | | 2,58E-02 | MYC,TM4SF1 |
| RPL11 | other | | 2,58E-02 | MYC,PDCD4 |
| CD160 | transmembrane receptor | | 2,58E-02 | FOS,MCL1 |
| USP37 | peptidase | | 2,58E-02 | CEBPA,MYC |
| PINK1 | kinase | | 2,58E-02 | CCND1,SLC2A1 |
| GPR183 | G-protein coupled receptor | | 2,58E-02 | BCL2,MYC |
| COCH | other | | 2,58E-02 | CXCL2,IL18 |
| MC1R | G-protein coupled receptor | | 2,58E-02 | NR4A1,SDC2 |
| SCUBE3 | other | | 2,58E-02 | SERPINE1,VIM |
| TKT | enzyme | | 2,58E-02 | CCND1,MCL1 |
| BRMS1 | transcription regulator | | 2,58E-02 | CXCR4,GJB1 |
| NELFE | other | | 2,58E-02 | CXCL8,JUNB |
| RECK | other | | 2,58E-02 | CCND1,SPRY2 |
| 1,1-bis(3'-indolyl)-1-(4-hydroxyphenyl)methane | chemical reagent | | 2,58E-02 | BCL2,NR4A1 |
| niflumic acid | chemical drug | | 2,58E-02 | CFLAR,CXCL8 |
| benazepril | chemical drug | | 2,58E-02 | BCL2,EDN1 |
| CITCO | chemical reagent | | 2,58E-02 | ALDH1A1,GSTA2 |
| RP 73401 | chemical toxicant | | 2,58E-02 | CXCL2,EGR1 |
| prodigiosin | chemical toxicant | | 2,58E-02 | BCL2,TNFRSF10B |
| ketorolac | chemical drug | | 2,58E-02 | CXCL8,PTGS2 |
| iodine | chemical - endogenous mammalian | | 2,58E-02 | BCL2,ICAM1 |
| tyrphostin AG 127 | chemical - kinase inhibitor | | 2,58E-02 | BCL2,PTGS2 |
| 5-iodotubercidin | chemical - kinase inhibitor | | 2,58E-02 | CCND1,MYCN |
| miR-1236-3p (miRNAs w/seed CUCUUCC) | mature microRNA | | 2,59E-02 | [illegible] |
| miR-4432 (miRNAs w/seed AAGACUC) | mature microRNA | | 2,59E-02 | [illegible] |
| HNRNPA2B1 | other | | 2,59E-02 | ABCC6,ACSL5,ERBB3,ITPRID2,MUC13,MYC,PBX1,UBASH3B,[illegible] |
| miR-659-3p (miRNAs w/seed UUGGUUC) | mature microRNA | | 2,60E-02 | [illegible] |
| RPTOR | other | | 2,61E-02 | CCND1,DUSP6,HK2,MVD,MYC,SLC2A1,SPTLC3 |
| miR-2277-3p (miRNAs w/seed GACAGCG) | mature microRNA | | 2,61E-02 | [illegible] |
| miR-5582-5p (miRNAs w/seed AGGCACA) | mature microRNA | | 2,63E-02 | [illegible] |
| miR-887-5p (miRNAs w/seed UUGGGAG) | mature microRNA | | 2,64E-02 | [illegible] |
| [illegible] (miRNAs w/seed UCGCUUU) | mature microRNA | | 2,64E-02 | [illegible] |
| [illegible] (miRNAs w/seed UUUGCAA) | mature microRNA | | 2,66E-02 | [illegible] |
| 1810019D21Rik | other | Inhibited | 2,67E-02 | AREG,EDN1,ITGA2,MYCN,SLC7A11 |
| miR-551b-5p (miRNAs w/seed AAAUCAA) | mature microRNA | | 2,67E-02 | AMACR,IER3,LPAR6,PLPP1,TPD52L1 |
| miR-3152-5p (miRNAs w/seed UUGCCUC) | mature microRNA | | 2,67E-02 | [illegible] |
| miR-6786-5p (miRNAs w/seed CGGUGGG) | mature microRNA | | 2,69E-02 | [illegible] |
| miR-4759 (miRNAs w/seed AGGACUA) | mature microRNA | | 2,70E-02 | [illegible] |
| [illegible] (and other miRNAs w/seed GUGGACA) | mature microRNA | | 2,70E-02 | [illegible] |
| miR-1304 (miRNAs w/seed CGUGGCC) | mature microRNA | | 2,70E-02 | [illegible] |
| MGP | other | | 2,71E-02 | GATA4,PAH,TAGLN |
| ALDH2 | enzyme | | 2,71E-02 | PHGDH,PSPH,SLC7A11 |
| BMP15 | growth factor | | 2,71E-02 | AREG,ID1,PTGS2 |
| CRHR2 | G-protein coupled receptor | | 2,71E-02 | BCL2,CXCL2,SLC2A1 |
| CNR2 | G-protein coupled receptor | | 2,71E-02 | CCND1,CXCL8,EGR1 |
| FOXP2 | transcription regulator | | 2,71E-02 | CCND1,PDGFB,TAGLN |
| EIF4EBP2 | translation regulator | | 2,71E-02 | CEBPA,EGR1,PTGS2 |
| 2,4-dinitrofluorobenzene | chemical toxicant | | 2,71E-02 | CXCL2,ICAM1,IL18 |
| [illegible] | chemical - endogenous non-mammalian | | 2,71E-02 | CYP27A1,HSD3B1,LDLR |
| 9Z,11E-octadecadienoic acid | chemical - endogenous mammalian | | 2,71E-02 | LDLR,NFAT5,PTGS2 |
| miR-3195 (miRNAs w/seed GCGCCGG) | mature microRNA | | 2,71E-02 | [illegible] |
| [illegible] (miRNAs w/seed [illegible]) | mature microRNA | | 2,75E-02 | [illegible] |
| miR-6721-5p (miRNAs w/seed GGGCAGG) | mature microRNA | | 2,75E-02 | [illegible] |
| MIR17HG | other | | 2,75E-02 | CCND1,FRAS1,FZD4,LRP1,MYCN,TAGLN,TBX3,TNFAIP3 |
| miR-670-5p (miRNAs w/seed UCCCUGA) | mature microRNA | | 2,77E-02 | [illegible] |
| miR-1283 (miRNAs w/seed CUACAAA) | mature microRNA | | 2,78E-02 | [illegible] |
| miR-548o-3p (and other miRNAs w/seed CAAAACU) | mature microRNA | | 2,78E-02 | [illegible] |
| miR-3168 (miRNAs w/seed AGUUCUA) | mature microRNA | | 2,79E-02 | [illegible] |
| miR-4722-5p (miRNAs w/seed GCAGGAG) | mature microRNA | | 2,79E-02 | [illegible] |
| [illegible] (and other miRNAs w/seed UGGGCUC) | mature microRNA | | 2,79E-02 | [illegible] |
| miR-6735-3p (miRNAs w/seed GGCCUGU) | mature microRNA | | 2,81E-02 | [illegible] |
| miR-1263 (miRNAs w/seed [illegible]) | mature microRNA | | 2,84E-02 | [illegible] |
| miR-6762-3p (miRNAs w/seed CCUGGGU) | mature microRNA | | 2,84E-02 | [illegible] |
| CHD4 | enzyme | | 2,86E-02 | [illegible] |
| miR-23a-5p (and other miRNAs w/seed GGGUUCC) | mature microRNA | | 2,86E-02 | [illegible] |
| PTGER2 | G-protein coupled receptor | | 2,86E-02 | AREG,CXCL8,CXCR4,EGR1,ESRRA,PTGS2,SERPINE1 |
| 9-hydroxy-(S)-10,12-octadecadienoic acid | chemical - endogenous mammalian | | 2,88E-02 | SERPINE1 |
| N(6)-methyl-2'-deoxyadenosine | chemical reagent | | 2,88E-02 | SOCS3 |
| acyclovir | chemical drug | | 2,88E-02 | DUSP2 |
| pelargonidin | chemical - endogenous non-mammalian | | 2,88E-02 | CYP1A1 |
| azide | chemical reagent | | 2,88E-02 | SLC2A1 |
| thromboxane A2 | chemical - endogenous mammalian | | 2,88E-02 | PTGS2 |
| 2'-adenylic acid | chemical - endogenous mammalian | | 2,88E-02 | TXNIP |
| [illegible] | chemical - endogenous non-mammalian | | 2,88E-02 | CFLAR |
| ABLIM2 | other | | 2,88E-02 | TAGLN |
| cyanuric acid | chemical reagent | | 2,88E-02 | CXCL8 |
| peptide YY 3-36 | biologic drug | | 2,88E-02 | FOS |
| hydroxamic acid | chemical - other | | 2,88E-02 | CXCR4 |
| LTB4R/LTB4R2 | group | | 2,88E-02 | CXCL8 |
| [illegible] | group | | 2,88E-02 | PTGS2 |
| NDPK | group | | 2,88E-02 | CXCL8 |
| G protein alpha | group | | 2,88E-02 | FOS |
| beta-glucuronidase | group | | 2,88E-02 | PTGS2 |
| Mkp | complex | | 2,88E-02 | NR4A1 |
| glutathione peroxidase | group | | 2,88E-02 | CFLAR |
| MKK3/6 | group | | 2,88E-02 | CXCL8 |
| growth factor receptor | group | | 2,88E-02 | FOS |
| G protein gamma | group | | 2,88E-02 | SRC |
| G protein beta | group | | 2,88E-02 | SRC |
| Ahr-aryl hydrocarbon | complex | | 2,88E-02 | MDM2 |
| 2,2',4,4',5,5'-hexabromodiphenyl ether | chemical reagent | | 2,88E-02 | CYP1A1 |
| 5,7-dimethoxyflavone | chemical - endogenous mammalian | | 2,88E-02 | CYP1A1 |
| DE 71 | chemical reagent | | 2,88E-02 | CYP1A1 |
| [illegible] | chemical reagent | | 2,88E-02 | PTGS2 |
| Ahr-aryl hydrocarbon-RelA | complex | | 2,88E-02 | MYC |
| NR2F1-AS1 | other | | 2,88E-02 | CCND1 |
| PKR dimer | complex | | 2,88E-02 | ATF3 |
| MAP2K4/7 | group | | 2,88E-02 | PTGS2 |
| NF279 | chemical reagent | | 2,88E-02 | CXCL8 |
| sodium thiosulfate | chemical drug | | 2,88E-02 | BCL2 |
| GSK 189254 | chemical drug | | 2,88E-02 | FOS |
| CLTA | other | | 2,88E-02 | LDLR |
| (-)-OSU6162 | chemical drug | | 2,88E-02 | FOS |
| GJC2 | transporter | | 2,88E-02 | GJB1 |
| PMM2 | enzyme | | 2,88E-02 | ICAM1 |
| NCK | group | | 2,88E-02 | CCND1 |
| SEC16B | other | | 2,88E-02 | RGN |
| [illegible] | other | | 2,88E-02 | PTGS2 |
| pridopidine | chemical drug | | 2,88E-02 | FOS |
| FBXO31 | enzyme | | 2,88E-02 | CCND1 |
| EMC2 | other | | 2,88E-02 | TRPC6 |
| NLRP2 | other | | 2,88E-02 | ICAM1 |
| WTIP | transcription regulator | | 2,88E-02 | AREG |
| [illegible] | other | | 2,88E-02 | CYP27A1 |
| RGN | enzyme | | 2,88E-02 | BCL2 |
| FBXO22 | enzyme | | 2,88E-02 | MDM2 |
| SRF-ELK1 | complex | | 2,88E-02 | FOS |
| WFDC1 | other | | 2,88E-02 | ICAM1 |
| ABLIM3 | other | | 2,88E-02 | TAGLN |
| KCNJ12 | ion channel | | 2,88E-02 | CCND1 |
| CALCOCO1 | transcription regulator | | 2,88E-02 | CCND1 |
| URGCP | other | | 2,88E-02 | CCND1 |
| MARCHF4 | enzyme | | 2,88E-02 | TNFRSF10A |
| [illegible] | enzyme | | 2,88E-02 | RHOB |
| FCN1 | other | | 2,88E-02 | CXCL8 |
| procyanidin B2 | chemical - endogenous non-mammalian | | 2,88E-02 | PTGS2 |
| TMEM184A | other | | 2,88E-02 | DUSP1 |
| DEPDC1 | transcription regulator | | 2,88E-02 | TNFAIP3 |
| [illegible] | cytokine | | 2,88E-02 | SOCS3 |
| NACC2 | transcription regulator | | 2,88E-02 | MDM2 |
| MYADM | other | | 2,88E-02 | ICAM1 |
| TRIM17 | enzyme | | 2,88E-02 | MCL1 |
| SLC17A6 | transporter | | 2,88E-02 | NR4A1 |
| NBR2 | other | | 2,88E-02 | SLC2A1 |
| ZNF580 | transcription regulator | | 2,88E-02 | CXCL8 |
| CCDC22 | other | | 2,88E-02 | BIRC3 |
| USP50 | other | | 2,88E-02 | IL18 |
| NLRP6 | other | | 2,88E-02 | IL18 |

| | | | | |
|---|---|---|---|---|
| CCDC82 | transcription regulator | | 2,88E-02 | CCND1 |
| RPS6KA | group | | 2,88E-02 | CXCL8 |
| MPC1 | other | | 2,88E-02 | MPC2 |
| CCDC47 | other | | 2,88E-02 | TRPC6 |
| ZG16B | other | | 2,88E-02 | CXCR4 |
| KCTD10 | ion channel | | 2,88E-02 | RHOB |
| harman | chemical - endogenous non-mammalian | | 2,88E-02 | CYP1A1 |
| FGF16 | growth factor | | 2,88E-02 | FOS |
| EMC6 | other | | 2,88E-02 | TRPC6 |
| TRPM3 | ion channel | | 2,88E-02 | EGR1 |
| Gm13070/Gm2984 | other | | 2,88E-02 | MPC2 |
| PLAC1 | other | | 2,88E-02 | CCND1 |
| RNF181 | enzyme | | 2,88E-02 | BCL10 |
| MARCHF9 | other | | 2,88E-02 | TNFRSF10A |
| METAP2 | peptidase | | 2,88E-02 | BCL2 |
| DHODH | enzyme | | 2,88E-02 | CFLAR |
| TCF4-CTNNB | complex | | 2,88E-02 | MYC |
| nickel sulfate | chemical reagent | | 2,88E-02 | PTGS2 |
| rimantadine | chemical drug | | 2,88E-02 | CXCL8 |
| tandutinib | chemical drug | | 2,88E-02 | ID1 |
| Tyk2 kinase inhibitor | chemical - kinase inhibitor | | 2,88E-02 | PTGS2 |
| alisertib | chemical drug | | 2,88E-02 | MYCN |
| [illegible] | chemical drug | | 2,88E-02 | EGR1 |
| MEF2D-NFATc2-p300 | complex | | 2,88E-02 | NR4A1 |
| mapatumumab | biologic drug | | 2,88E-02 | CFLAR |
| NNZ 2566 | biologic drug | | 2,88E-02 | ATF3 |
| vasopressins | biologic drug | | 2,88E-02 | FOS |
| black cohosh extract | chemical drug | | 2,88E-02 | SERPINE1 |
| Integrin alpha 5 beta 1 | complex | | 2,88E-02 | CCND1 |
| mediator | complex | | 2,88E-02 | FOS |
| Mapk kinase | group | | 2,88E-02 | CCND1 |
| Rho gdi | group | | 2,88E-02 | RHOB |
| CLIPR1 | other | | 2,88E-02 | MYC |
| NSMCE3 | other | | 2,88E-02 | MYC |
| TOMM22 | transporter | | 2,88E-02 | HSD3B1 |
| DLAT | enzyme | | 2,88E-02 | [illegible] |
| binimetinib | chemical drug | | 2,88E-02 | TNFRSF10A |
| cucurbitacin E | chemical - endogenous non-mammalian | | 2,88E-02 | PTGS2 |
| ARHGEF17 | other | | 2,88E-02 | FOS |
| vandetanib | chemical drug | | 2,88E-02 | BCL2 |
| pidilizumab | biologic drug | | 2,88E-02 | CXCR4 |
| [illegible] | translation regulator | | 2,88E-02 | [illegible] |
| IRGM | enzyme | | 2,88E-02 | ULK1 |
| PRCC | other | | 2,88E-02 | SERPINE1 |
| MEDI-547 | biologic drug | | 2,88E-02 | EPHA2 |
| Tenascin | group | | 2,88E-02 | CCND1 |
| DYNLRB1 | other | | 2,88E-02 | SERPINE1 |
| [illegible] | other | | 2,88E-02 | MYC |
| MED26 | other | | 2,88E-02 | MMP1 |
| [illegible] | growth factor | | 2,88E-02 | PTGS2 |
| AHRR | transcription regulator | | 2,88E-02 | CYP1A1 |
| KIFAP3 | other | | 2,88E-02 | FOS |
| NAPA | transporter | | 2,88E-02 | BCL2 |
| MED7 | transcription regulator | | 2,88E-02 | TNFAIP3 |
| KRT1 | other | | 2,88E-02 | IL18 |
| PPY | other | | 2,88E-02 | FOS |
| CDC45 | other | | 2,88E-02 | MYC |
| CHKA | kinase | | 2,88E-02 | TUBB3 |
| [illegible] | other | | 2,88E-02 | FOS |
| PRL-3 inhibitor I | chemical reagent | | 2,88E-02 | ULBP2 |
| SLC52A1 | transporter | | 2,88E-02 | ICAM1 |
| KSR2 | kinase | | 2,88E-02 | CXCL8 |
| ADCY2 | enzyme | | 2,88E-02 | CCND1 |
| CLTB | other | | 2,88E-02 | LDLR |
| CFI | peptidase | | 2,88E-02 | C3 |
| mir-383 | microRNA | | 2,88E-02 | MYC |
| miR-690 (miRNAs w/seed AAGGCUA) | mature microRNA | | 2,88E-02 | CEBPA |
| Mir690 | microRNA | | 2,88E-02 | CEBPA |
| mir-1301 | microRNA | | 2,88E-02 | KLF6 |
| DYNLT3 | other | | 2,88E-02 | BCL2 |
| NPVF | other | | 2,88E-02 | FOS |
| SLC30A9 | transporter | | 2,88E-02 | CYP1A1 |
| CNOT9 | other | | 2,88E-02 | MYC |
| CAMK2 | group | | 2,88E-02 | BIRC3 |
| SIGLEC7 | transmembrane receptor | | 2,88E-02 | PTGS2 |
| SIGLEC9 | other | | 2,88E-02 | PTGS2 |
| TOB2 | other | | 2,88E-02 | CCND1 |
| [illegible] | transporter | | 2,88E-02 | CXCL8 |
| TYMP | growth factor | | 2,88E-02 | CXCL8 |
| SLC1A2 | transporter | | 2,88E-02 | FOS |
| GTPBP4 | enzyme | | 2,88E-02 | CCND1 |
| GPHB5 | other | | 2,88E-02 | FOS |
| SCN7A | ion channel | | 2,88E-02 | FOS |
| NXF3 | transporter | | 2,88E-02 | TGFB3 |
| NAPB | transporter | | 2,88E-02 | CYP1A1 |
| LRRN1 | other | | 2,88E-02 | MYCN |
| aloperine | chemical reagent | | 2,88E-02 | CFLAR |
| ANKS1A | other | | 2,88E-02 | FOS |
| 5-methoxytryptophan | chemical - endogenous mammalian | | 2,88E-02 | PTGS2 |
| venetoclax | chemical drug | | 2,88E-02 | MCL1 |
| [illegible] | enzyme | | 2,88E-02 | NOTCH3 |
| KIF11 | other | | 2,88E-02 | CFLAR |
| LUC7L3 | other | | 2,88E-02 | PTGS2 |
| PTPRU | phosphatase | | 2,88E-02 | CCND1 |
| WBP1 | other | | 2,88E-02 | MCL1 |
| TFF1 | other | | 2,88E-02 | PTGS2 |
| MED21 | transcription regulator | | 2,88E-02 | TNFAIP3 |
| PTCRA | other | | 2,88E-02 | NOTCH3 |
| Pla2g2a | other | | 2,88E-02 | PTGS2 |
| PMS2 | enzyme | | 2,88E-02 | CCND1 |
| [illegible] | transcription regulator | | 2,88E-02 | MYC |
| EPGN | growth factor | | 2,88E-02 | FOS |
| WDR83OS | other | | 2,88E-02 | TRPC6 |
| TAX1BP3 | transcription regulator | | 2,88E-02 | FOS |
| RIT1 | enzyme | | 2,88E-02 | BCL2 |
| CORO2A | other | | 2,88E-02 | DIO1 |
| CFD | peptidase | | 2,88E-02 | C3 |
| ABLIM1 | other | | 2,88E-02 | TAGLN |
| THEM4 | enzyme | | 2,88E-02 | PTGS2 |
| BARX1 | transcription regulator | | 2,88E-02 | L1CAM |
| Tnxa-ps1 | other | | 2,88E-02 | DUSP1 |
| PRDM2 | transcription regulator | | 2,88E-02 | MYC |
| 16:0 ceramide-1-phosphate | chemical - endogenous mammalian | | 2,88E-02 | CXCL8 |
| [illegible] | chemical drug | | 2,88E-02 | CEBPA |
| RAB8B | enzyme | | 2,88E-02 | FOS |
| CDK20 | kinase | | 2,88E-02 | CCND1 |
| ADAM19 | peptidase | | 2,88E-02 | EGR2 |
| GDF10 | growth factor | | 2,88E-02 | SERPINE1 |
| CD164 | other | | 2,88E-02 | CXCR4 |
| cytarabine/filgrastim/fludarabine phosphate | biologic drug | | 2,88E-02 | FOS |
| ETS1-AS1 | other | | 2,88E-02 | ETS1 |
| PRC1 | other | | 2,88E-02 | PTGS2 |
| PTOV1 | other | | 2,88E-02 | CCND1 |
| tetramethylpyrazine | chemical - endogenous non-mammalian | | 2,88E-02 | CXCR4 |
| INHBE | growth factor | | 2,88E-02 | JUNB |
| EXOSC10 | kinase | | 2,88E-02 | FOS |
| PPP2R3A | phosphatase | | 2,88E-02 | MYC |
| TPD52 | other | | 2,88E-02 | BCL2 |
| ZBTB14 | transcription regulator | | 2,88E-02 | MYC |
| RPS20 | other | | 2,88E-02 | MYC |
| CHRM2 | G-protein coupled receptor | | 2,88E-02 | EGR1 |
| [illegible] | kinase | | 2,88E-02 | ATF3 |
| [illegible] | chemical drug | | 2,88E-02 | MCL1 |
| PEX14 | transcription regulator | | 2,88E-02 | PTGS2 |
| [illegible] | chemical drug | | 2,88E-02 | TNFRSF10B |
| spliceostatin A | chemical reagent | | 2,88E-02 | MCL1 |
| oligomycin A | chemical - endogenous non-mammalian | | 2,88E-02 | TXNIP |
| CID-2011756 | chemical reagent | | 2,88E-02 | NR4A1 |
| CML advanced glycation end products | chemical reagent | | 2,88E-02 | EGR1 |
| [illegible] | chemical reagent | | 2,88E-02 | EDN1 |
| dihydrotanshinone I | chemical reagent | | 2,88E-02 | TNFRSF10B |
| NMS-873 | chemical reagent | | 2,88E-02 | MYC |
| RPL31 | other | | 2,88E-02 | MYC |
| SLC25A5 | transporter | | 2,88E-02 | TNFRSF10B |
| RPLP0 | other | | 2,88E-02 | CCND1 |
| 3,4-(methylenedioxy)cinnamic acid | chemical reagent | | 2,88E-02 | MCL1 |
| [illegible] | chemical reagent | | 2,88E-02 | CFLAR |
| diclofenac/hyaluronic acid | chemical drug | | 2,88E-02 | CFLAR |
| RB4 | chemical reagent | | 2,88E-02 | MCL1 |
| RB3 | chemical reagent | | 2,88E-02 | MCL1 |
| [illegible] | chemical reagent | | 2,88E-02 | CFLAR |
| arsenic trichloride | chemical reagent | | 2,88E-02 | GADD45A |
| meayamycin B | chemical reagent | | 2,88E-02 | MCL1 |
| amurensin G | chemical reagent | | 2,88E-02 | CFLAR |
| BCL201 | chemical drug | | 2,88E-02 | MCL1 |
| [illegible] | chemical drug | | 2,88E-02 | CXCL8 |
| NBI-27914 | chemical drug | | 2,88E-02 | FOS |
| [illegible] | chemical - kinase inhibitor | | 2,88E-02 | CYP1A1 |
| [illegible] | chemical reagent | | 2,88E-02 | PTGS2 |
| impromidine | chemical drug | | 2,88E-02 | LDLR |
| guanfacine | chemical drug | | 2,88E-02 | FOS |
| amlexanox | chemical drug | | 2,88E-02 | CCND1 |
| 2,5-dihydroxymethylcinnamade | chemical - kinase inhibitor | | 2,88E-02 | EDN1 |
| LY311727 | chemical reagent | | 2,88E-02 | PTGS2 |
| BN 50730 | chemical reagent | | 2,88E-02 | PTGS2 |
| sterigmatocystin | chemical toxicant | | 2,88E-02 | CYP1A1 |
| ondansetron | chemical drug | | 2,88E-02 | FOS |
| nafoxidine | chemical drug | | 2,88E-02 | C3 |
| cyclothiazide | chemical drug | | 2,88E-02 | EGR1 |
| [illegible] | chemical - kinase inhibitor | | 2,88E-02 | MYC |
| flurothyl | chemical drug | | 2,88E-02 | FOS |
| pustulan | chemical reagent | | 2,88E-02 | CXCL8 |
| trichloroacetic acid | chemical drug | | 2,88E-02 | MCL1 |
| L 733060 | chemical reagent | | 2,88E-02 | FOS |
| 1-(1-glycero)dodeca-1,3,5,7,9-pentaene | chemical toxicant | | 2,88E-02 | PTGS2 |
| methaneselenol | chemical - endogenous non-mammalian | | 2,88E-02 | ULBP2 |
| SC-19220 | chemical reagent | | 2,88E-02 | ICAM1 |
| sulfaphenazole | chemical drug | | 2,88E-02 | CCND1 |
| L-threo-safingol | chemical drug | | 2,88E-02 | CXCL8 |
| [illegible] | chemical drug | | 2,88E-02 | FOS |
| melarsoprol | chemical drug | | 2,88E-02 | BCL2 |
| trichlorfon | chemical toxicant | | 2,88E-02 | ACHE |
| (R)-5-(2-azetidinylmethoxy)-2-chloropyridine | chemical drug | | 2,88E-02 | FOS |
| Ro 20-1724 | chemical reagent | | 2,88E-02 | CCND1 |
| [illegible] | chemical - kinase inhibitor | | 2,88E-02 | PLAUR |
| [illegible] | other | | 2,88E-02 | ATF3 |
| [illegible] | chemical reagent | | 2,88E-02 | MYC |
| [illegible] | chemical reagent | | 2,88E-02 | MYC |
| aspergillus beta-glucan | chemical - endogenous non-mammalian | | 2,88E-02 | CXCL8 |
| pneumocystis beta-glucan | chemical - endogenous non-mammalian | | 2,88E-02 | CXCL8 |
| flunitrazepam | chemical drug | | 2,88E-02 | CXCL8 |
| thiabendazole | chemical drug | | 2,88E-02 | CYP1A1 |
| arachidonyl-2-chloroethylamide | chemical reagent | | 2,88E-02 | EGR1 |
| naphthalene-1,2-dione | chemical toxicant | | 2,88E-02 | CYP1A1 |
| trans-7,8-dihydroxy-7,8-dihydrobenzo(a)pyrene | chemical toxicant | | 2,88E-02 | CYP1A1 |
| anthracene | chemical toxicant | | 2,88E-02 | CYP1A1 |
| tolrestat | chemical drug | | 2,88E-02 | CXCL8 |
| MUC2 | other | | 2,88E-02 | CXCL8 |
| [illegible] | chemical reagent | | 2,88E-02 | MYC |
| 2-amino-3-methylimidazo(4,5-f)quinoline | chemical toxicant | | 2,88E-02 | CYP1A1 |
| Ro 31-7549 | chemical - kinase inhibitor | | 2,88E-02 | PTGS2 |
| bisindolylmaleimide III | chemical - kinase inhibitor | | 2,88E-02 | CFLAR |
| ONO-8711 | chemical drug | | 2,88E-02 | FOS |
| theaflavin monogallate B | chemical - endogenous non-mammalian | | 2,88E-02 | PTGS2 |
| anti-miR-302d | chemical reagent | | 2,88E-02 | CCND1 |
| AP-1 decoy | chemical reagent | | 2,88E-02 | DUSP1 |
| anti-miR-302c inhibitor | chemical reagent | | 2,88E-02 | CCND1 |
| beta-penta-O-galloyl-glucose | chemical - endogenous non-mammalian | | 2,88E-02 | CCND1 |
| potassium iodide | chemical drug | | 2,88E-02 | CXCL8 |
| bis-T-23 | chemical reagent | | 2,88E-02 | COL4A1 |
| ETV6-PDGFRB | fusion gene/product | | 2,88E-02 | ID1 |
| [illegible] | fusion gene/product | | 2,88E-02 | MMP1 |
| BRD4-NUTM1 | fusion gene/product | | 2,88E-02 | MYC |
| ZBTB16-RARA | fusion gene/product | | 2,88E-02 | DUSP6 |
| CRTC1-MAML2 | fusion gene/product | | 2,88E-02 | AREG |
| AuNP@PEG@e14a2 | chemical reagent | | 2,88E-02 | BCL2 |
| miR-126 inhibitor | chemical reagent | | 2,88E-02 | MCL1 |
| anti-miR-32 inhibitor | chemical reagent | | 2,88E-02 | KLF4 |
| PT2399 | chemical reagent | | 2,88E-02 | EPO |
| SU11333 | chemical - kinase inhibitor | | 2,88E-02 | MYC |
| CTOP | chemical reagent | | 2,88E-02 | FOS |
| PF-4800567 | chemical - kinase inhibitor | | 2,88E-02 | MYC |
| N-t-Boc-Phe-D-Leu-Phe-D-Leu-Phe | chemical reagent | | 2,88E-02 | PTGS2 |
| IP 20 | chemical - kinase inhibitor | | 2,88E-02 | HSD3B1 |
| Gly-Gln | chemical reagent | | 2,88E-02 | ACHE |
| Ac-DEVD-CHO | chemical reagent | | 2,88E-02 | FOS |
| [illegible] | chemical drug | | 2,88E-02 | FOS |
| SMAD3-EP300 | complex | | 2,88E-02 | SERPINE1 |
| 3-methoxyapigenin | chemical - endogenous non-mammalian | | 2,88E-02 | MYC |
| berberrubine | chemical - endogenous non-mammalian | | 2,88E-02 | LDLR |
| columbamine | chemical - endogenous non-mammalian | | 2,88E-02 | LDLR |
| harmaline | chemical drug | | 2,88E-02 | CYP1A1 |
| LY 354740 | chemical drug | | 2,88E-02 | FOS |
| droxidopa | chemical drug | | 2,88E-02 | FOS |
| S(-)-carbidopa | chemical drug | | 2,88E-02 | FOS |
| 2-(N-myristoylamino)-1-phenyl-1-propanol | chemical reagent | | 2,88E-02 | MMP1 |
| 10-hydroxydecanoic acid | chemical - endogenous non-mammalian | | 2,88E-02 | IRF1 |
| n-6 docosapentaenoic acid | chemical - other | | 2,88E-02 | PTGS2 |
| long chain alcohol | chemical - endogenous non-mammalian | | 2,88E-02 | PTGS2 |
| 3'-adenylic acid | chemical - endogenous mammalian | | 2,88E-02 | TXNIP |
| poractant alfa | chemical drug | | 2,88E-02 | CXCL8 |
| 2'-fucosyllactose | chemical - endogenous mammalian | | 2,88E-02 | ICAM1 |
| sarcosine | chemical - endogenous mammalian | | 2,88E-02 | FOS |
| allose | chemical - endogenous mammalian | | 2,88E-02 | TXNIP |
| lactulose | chemical - endogenous mammalian | | 2,88E-02 | CXCL8 |
| gestodene | chemical drug | | 2,88E-02 | TGFB3 |
| estramustine | chemical drug | | 2,88E-02 | CYP1A1 |
| [illegible] | chemical toxicant | | 2,88E-02 | BCL2 |
| V2+ | chemical toxicant | | 2,88E-02 | CXCL8 |
| estradiol benzoate | chemical drug | | 2,87E-02 | C3,FOS,GJB1,PAQR8,PTGS2,STK39 |
| GAST | other | | 2,87E-02 | AREG,CCND1,EGR1,FOS,PTGS2,SRC |
| sodium chloride | chemical - endogenous mammalian | | 2,87E-02 | CXCL8,EDN1,FOS,ICAM1,NFAT5,PTGS2 |
| miR-688-5p (miRNAs w/seed [illegible]) | mature microRNA | | 2,87E-02 | [illegible],ETS1,FOS,GPRC5C,HK2,KLHL21,RHOB,THAP6 |
| tributyltin | chemical reagent | | 2,87E-02 | ACSL1,CEBPA,GPRC5A,IDH1,IL11,INHBA,ITGA3,WNK4 |
| miR-6494 (miRNAs w/seed CAGACUG) | mature microRNA | | 2,88E-02 | [illegible] |
| miR-19b-1-5p (and other miRNAs w/seed GUUUUGC) | mature microRNA | Inhibited | 2,89E-02 | ARRDC3,[illegible] |
| miR-6894-3p (miRNAs w/seed UGCCUGC) | mature microRNA | | 2,90E-02 | AFAP1,APOBEC3C,BHLHE40,BIRC3,CDC14B,CDK2,COA5,EFNA1,[illegible] |
| LGR4 | transmembrane receptor | | 2,90E-02 | GLUL,OAT,SLC2A1,VIM |
| RNASEH2A | enzyme | | 2,90E-02 | DDX21,IRF1,NFKB2,PTGS2 |
| M344 | chemical reagent | | 2,90E-02 | APBB1,BRPF1,CXCL8,SERPINE1 |
| 4-methylnitrosamino-1-(3-pyridyl)-1-butanone | chemical toxicant | | 2,90E-02 | BCL2,FOS,JUNB,PTGS2 |
| memantine | chemical drug | | 2,90E-02 | FOS,GLUL,PAK1,RXRA |
| eflornithine | chemical drug | | 2,90E-02 | C3,MYCN,SERPINE1,[illegible] |
| laminaran | chemical drug | | 2,90E-02 | CXCL8,[illegible],INHBA,PTGS2 |
| miR-4701-5p (and other miRNAs w/seed UGGCCAC) | mature microRNA | | [illegible] | ABCC5,ATF3,CCDC69,CDK18,CXCR4,DCBLD2,DUSP6,[illegible] |
| miR-583 (miRNAs w/seed AAAGAGG) | mature microRNA | | [illegible] | [illegible] |
| miR-512-3p (miRNAs w/seed AGUGCUG) | mature microRNA | | [illegible] | [illegible] |
| miR-1268a-5p (and other miRNAs w/seed [illegible]) | mature microRNA | | [illegible] | [illegible] |
| miR-6641 (miRNAs w/seed GCCCAUG) | mature microRNA | Activated | [illegible] | [illegible] |
| arsenic trioxide | chemical drug | | [illegible] | BCL2,BNIP3L,CCND1,CFLAR,CXCL8,[illegible] |
| miR-6875-3p (miRNAs w/seed UUCUUCC) | mature microRNA | | 3,00E-02 | [illegible] |
| miR-5702 (miRNAs w/seed GAGUCAG) | mature microRNA | | 3,00E-02 | [illegible] |
| miR-298 (miRNAs w/seed GCAGAAG) | mature microRNA | Activated | 3,01E-02 | [illegible] |
| miR-6802-5p (miRNAs w/seed UAGGUGG) | mature microRNA | | 3,02E-02 | [illegible] |
| TAL1 | transcription regulator | | 3,02E-02 | BCL2,C3,ETS1,FBXW7,ICAM1,MVD,MYC,NFKBIZ,RUNX1,SPRY2,TNFAIP3,ZC3H12A |
| MBD3 | other | | 3,02E-02 | CCND1,FOSL1,FZD4,MDM2,MYC,[illegible] |
| risperidone | chemical drug | | 3,03E-02 | BCL2,FOS,NR4A1 |
| bosentan | chemical drug | | 3,03E-02 | EDN1,GATA4,ICAM1 |
| RNF20 | enzyme | | 3,03E-02 | FOS,MYC,RHOB |
| IL36B | cytokine | | 3,03E-02 | CXCL8,IL18,NFKBIZ |
| Relaxin | group | | 3,03E-02 | EDN1,GATA4,MMP1 |
| vasoactive intestinal peptide | biologic drug | | 3,03E-02 | BCL2,CXCL8,FOS |
| LINC00963 | other | | 3,03E-02 | BCL2,CXCR4,ICAM1 |
| BMPER | other | | 3,03E-02 | EFNB2,ICAM1,ID1 |
| PLD1 | enzyme | | 3,03E-02 | BCL2,MYC,PTGS2 |
| FOXC1 | transcription regulator | | 3,03E-02 | CXCR4,EFNB2,SERPINE1 |
| RACK1 | enzyme | | 3,03E-02 | FOS,MDM2,MYC |
| bilirubin | chemical - endogenous mammalian | | 3,03E-02 | CCND1,CXCL8,GATA2 |
| chelerythrine | chemical drug | | 3,03E-02 | ICAM1,MCL1,MDM2 |
| poly dA-dT | chemical reagent | | 3,03E-02 | [illegible],CXCL8,IL18 |
| SB 290157 | chemical reagent | | 3,03E-02 | CXCL2,PTGS2,TAGLN |
| sunitinib | chemical drug | | 3,03E-02 | CYP1A1,ICAM1,ITGA3,ITGA6,RUNX1 |
| FOXC2 | transcription regulator | Activated | 3,03E-02 | CEBPA,CXCR4,EFNB2,PTGS2,VIM |
| STUB1 | enzyme | | 3,03E-02 | GATA4,JUNB,NFKB2,RUNX1,TAGLN |
| clozapine | chemical drug | | 3,03E-02 | EGR1,FOS,FOXA1,LDLR,NR4A1 |
| miR-3616-3p (miRNAs w/seed GAGGGCA) | mature microRNA | | [illegible] | [illegible] |
| miR-4785 (miRNAs w/seed GAGUCGG) | mature microRNA | | [illegible] | [illegible] |
| miR-6501-5p (miRNAs w/seed [illegible]) | mature microRNA | | [illegible] | [illegible] |
| miR-3141 (miRNAs w/seed AGGGCGG) | mature microRNA | | [illegible] | [illegible] |
| miR-6756-5p (miRNAs w/seed [illegible]) | mature microRNA | | [illegible] | [illegible] |
| DNMT3B | enzyme | | 3,07E-02 | DNMT3B,FOXA1,GATA4,HDAC9,MYC,PAX6,PIK3C2B,SLC30A1,SLC7A11,YOD1 |
| miR-642a-3p (and other miRNAs w/seed GACACAU) | mature microRNA | | 3,07E-02 | [illegible] |
| miR-4733-5p (miRNAs w/seed AUCCCAA) | mature microRNA | | [illegible] | [illegible] |
| miR-542-3p (miRNAs w/seed GUGACAG) | mature microRNA | | [illegible] | [illegible] |
| miR-6818-3p (miRNAs w/seed UGUCUCU) | mature microRNA | | 3,10E-02 | [illegible] |
| MECP2 | transcription regulator | | 3,11E-02 | EGR1,EHMT2,JUNB,MYC,NAV3,NR4A1,PTGS2,VIM |
| miR-4755-3p (miRNAs w/seed GCCAGGC) | mature microRNA | | [illegible] | [illegible] |
| miR-1900 (and other miRNAs w/seed GCCGCCC) | mature microRNA | | [illegible] | [illegible] |
| PCGEM1 | other | | 3,16E-02 | GOT1,HK2,IDH1,SLC2A1 |
| CSF1R | kinase | | 3,16E-02 | CCND1,FOS,JUNB,MYC |
| PTGS1 | enzyme | | 3,16E-02 | ATF3,CFTR,EDN1,PTGS2 |
| NKX2-5 | transcription regulator | | 3,16E-02 | ANKRD1,BCL2,GATA4,MYCN |
| S-nitrosoglutathione | chemical toxicant | | 3,16E-02 | BIRC3,ICAM1,LIF,PTGS2 |
| cyclopamine | chemical reagent | | 3,16E-02 | BCL2,CCND1,MYCN,PTGS2 |
| L-threonine | chemical - endogenous mammalian | | 3,17E-02 | CCND1,MYC |
| niclosamide | chemical drug | | 3,17E-02 | BCL2,MCL1 |
| anacardic acid | chemical - endogenous non-mammalian | | 3,17E-02 | CXCL8,EDN1 |
| FAT1 | other | | 3,17E-02 | PDCD4,PTGS2 |
| BCO2 | enzyme | | 3,17E-02 | ARRDC3,NR1D1 |
| Sox2ot | other | | 3,17E-02 | ALDH1A1,VIM |
| ganetespib | chemical drug | | 3,17E-02 | BCL2,MYC |
| PF-3084014 | chemical drug | | 3,17E-02 | BCL2,MCL1 |
| [illegible] | chemical - endogenous non-mammalian | | 3,17E-02 | CCND1,MYC |
| IGF2R | transmembrane receptor | | 3,17E-02 | IGF2,TGFB3 |
| TNFRSF17 | transmembrane receptor | | 3,17E-02 | BCL2,ICAM1 |
| ITGA4 | transmembrane receptor | | 3,17E-02 | ITGA5,TNFAIP3 |
| NCK1 | kinase | | 3,17E-02 | FOS,PPP1R15A |
| [illegible] | transcription regulator | | 3,17E-02 | MMP1,PLAUR |
| SETD7 | enzyme | | 3,17E-02 | CCND1,MYC |
| [illegible] | phosphatase | | 3,17E-02 | [illegible],IRF1 |
| HES5 | transcription regulator | | 3,17E-02 | FBXW7,RUNX1 |
| GPC1 | transmembrane receptor | | 3,17E-02 | EFNA1,PTGS2 |
| KLB | enzyme | | 3,17E-02 | EGR1,FOS |
| CXCL13 | cytokine | | 3,17E-02 | MMP1,MYC |
| PCBP2 | other | | 3,17E-02 | CEBPA,MYC |
| STX3 | transporter | | 3,17E-02 | ANKRD1,SULT1C4 |
| EXT1 | enzyme | | 3,17E-02 | CCND1,MYC |
| IQGAP1 | other | | 3,17E-02 | CCND1,CXCR4 |
| HOXA1 | transcription regulator | | 3,17E-02 | EGR2,GATA6 |
| INCB054329 | chemical drug | | 3,17E-02 | CCND1,MYC |
| butyl(hydroxybutyl)nitrosamine | chemical toxicant | | 3,17E-02 | AREG,CYP1A1 |
| diltiazem | chemical drug | | 3,17E-02 | EDN1,FOS |
| allyl isothiocyanate | chemical toxicant | | 3,17E-02 | FOS,PTGS2 |
| calmidazolium | chemical drug | | 3,17E-02 | EGR1,FOS |
| Trolox C | chemical drug | | 3,17E-02 | CXCL8,MCL1 |
| 2-hydroxy-1-naphthylaldehyde isonicotinoyl hydrazone | chemical reagent | | 3,17E-02 | CCND1,GADD45A |
| largazole | chemical - endogenous non-mammalian | | 3,17E-02 | [illegible],HK2 |
| [illegible] | chemical reagent | | 3,17E-02 | DUSP1,PLAUR |
| SU1498 | chemical - kinase inhibitor | | 3,17E-02 | ETS1,ICAM1 |
| tin mesoporphyrin | chemical drug | | 3,17E-02 | ICAM1,[illegible] |
| SS18-SSX1 | fusion gene/product | | 3,17E-02 | CCND1,MDM2 |
| polymyxin B | biologic drug | | 3,17E-02 | CXCL8,LDLR |
| miR-4481 (and other miRNAs w/seed [illegible]) | mature microRNA | | [illegible] | [illegible] |
| miR-5626-3p (and other miRNAs w/seed UAAGGCA) | mature microRNA | | [illegible] | [illegible] |
| miR-6450 (miRNAs w/seed GAAGGCC) | mature microRNA | | [illegible] | [illegible] |
| miR-31-3p (and other miRNAs w/seed GCUAUGC) | mature microRNA | | [illegible] | [illegible] |
| miR-216-5p (miRNAs w/seed [illegible]) | mature microRNA | | 3,20E-02 | [illegible] |
| miR-431-3p (miRNAs w/seed [illegible]) | mature microRNA | | 3,20E-02 | BCL2,DACT2,ETS1,FAM131B,FOXP1,MXD1,PCYT1B,PLD6,SPIRE1,TM4SF1,ULBP2,ZNF362 |
| mir-27 | microRNA | | 3,22E-02 | EDN1,FBXW7,PTGS2,RUNX1,SPRY2 |
| SCD | enzyme | | 3,22E-02 | ATF3,BCL2,CXCL8,GADD45A,SLC2A1 |
| C5AR1 | G-protein coupled receptor | Activated | 3,22E-02 | C3,CXCL2,CXCL8,MMP1,SERPINE1 |
| miR-5580-5p (and other miRNAs w/seed [illegible]) | mature microRNA | | [illegible] | [illegible] |
| miR-5579-3p (miRNAs w/seed [illegible]) | mature microRNA | | [illegible] | [illegible] |
| miR-331-3p (miRNAs w/seed CCCCUGG) | mature microRNA | | [illegible] | [illegible] |
| miR-3571 (and other miRNAs w/seed ACACACU) | mature microRNA | | [illegible] | [illegible] |
| miR-513a-3p (miRNAs w/seed AAUGUCA) | mature microRNA | | [illegible] | [illegible] |
| NFATC1 | transcription regulator | | [illegible] | BCL2,CCND1,CYP1A1,EDN1,ICAM1,MYC,PTGS2 |
| miR-4767 (miRNAs w/seed GCGGGCG) | mature microRNA | | [illegible] | [illegible] |
| miR-365-1-5p (and other miRNAs w/seed GGGACUU) | mature microRNA | | [illegible] | [illegible] |
| ONECUT1 | transcription regulator | | [illegible] | [illegible] |
| FBXO32 | enzyme | | [illegible] | CXCL2,GADD45A,ITGA3,MYCN,OAT,PTGS2 |
| SPIB | transcription regulator | | [illegible] | ATF3,EGR1,EGR2,KLF6,NAV3,P2RY6 |
| miR-1911-5p (miRNAs w/seed GAGUACC) | mature microRNA | Inhibited | [illegible] | [illegible] |
| miR-1184 (miRNAs w/seed CUGCAGC) | mature microRNA | | [illegible] | [illegible] |
| [illegible] | transcription regulator | | [illegible] | AEN,EPHX2,IGFBP4,LPAR6,MPZL2,PHLDA1,PLK2,TUBB3 |
| miR-6798-3p (miRNAs w/seed UACCCCC) | mature microRNA | | [illegible] | [illegible] |
| SLPI | other | | [illegible] | CCND1,ICAM1,MCL1 |
| mir-9 | microRNA | | [illegible] | CXCR4,ETS1,RUNX1 |
| mir-214 | microRNA | | [illegible] | FGFR1,LDLR,PTGS2 |
| SOX6 | transcription regulator | | [illegible] | CCND1,MYC,SOCS3 |
| BTRC | enzyme | | [illegible] | CCND1,CXCL8,GLUL |
| [illegible] | chemical drug | | [illegible] | BCL2,MCL1,MYC |
| HEXIM1 | transcription regulator | | [illegible] | CCND1,SLC2A1,VIM |
| HSD17B4 | enzyme | | [illegible] | ACSL1,IHH,PTGS2 |
| TRPV4 | ion channel | | [illegible] | CXCL2,FOS,SOCS3 |

| | | | | |
|---|---|---|---|---|
| SDCBP | enzyme | | 3.37E-02 | ITGA6,SRC,VIM |
| ceruletide | biologic drug | | 3.37E-02 | CFLAR,CXCL2,PTGS2 |
| miR-584-3p (miRNAs w/seed CAGUUCC) | mature microRNA | | 3.37E-02 | ANXA8,APOL1,ARHGAP29,BHLHE40,C21orf91,CDK19,CISH,CXCR4,DDX21,DUSP6,EHHADH,ELK3,EPO,ETS1,KLHDC2,LRRN4,NBPF10 (includes others),NIPSNAP1,PLK2,PRUNE1,RASAL2,RIN1,RM2,SMIM14,TNFRSF12A,UPRT |
| miR-6819-3p (and other miRNAs w/seed AGCCUCU) | mature microRNA | | 3.40E-02 | ADH6,APBB1,APOBEC3C,C6orf141,CSRNP1,CXCL2,DDIAS,FOXA1,GOLT1A,GPRC5A,GREM1,GSTA4,IL11,IL17RE,IL18,IYD,KLF6,MAFG,MINDY1,NABP3,NDRG1,NSRP1,REEP1,RND3,SCIN,SH2B3,SLC19A3,SOCS3,ZNF430 |
| miR-3665 (miRNAs w/seed GGGCAUA) | mature microRNA | | 3.41E-02 | ABCC6,ALOX15,APBB1,APOL1,ARPIN/ARPIN-AP3S2,AS3MT,ATF3,C21orf91,CCDC69,CISH,CTD_3074O7.11,EFNA1,FZD4,GADD45A,GPAT3,IYD,KBTBD7,MAFF,MDM2,MPP1,MVD,MYBL1,MYBPH,OSGEPL1,PCYT1B,RASAL2,REEP1,RHBG,RRP9,SLC26A2,SULT1C2,TDO2,TRPC6,TTBK2,UPB1,XKRX |
| HDAX | transcription regulator | | 3.41E-02 | GATA4,MYC,SLC2A1,SLC7A11 |
| PTPRC | phosphatase | | 3.41E-02 | CCND1,CXCL8,ICAM1,MCL1 |
| fasudil | chemical drug | | 3.41E-02 | BCL2,ICAM1,TAGLN,TGFB3 |
| beta-carotene | chemical - endogenous mammalian | | 3.41E-02 | ARRDC3,NR1D1,PTGS2,SLC6A2 |
| 4-hydroxynonenal | chemical toxicant | | 3.41E-02 | ICAM1,IL18,PTGS2,SERPINE1 |
| miR-31-5p (and other miRNAs w/seed GGCAAGA) | mature microRNA | | 3.44E-02 | CEBPA,CLCF1,CXCL8,DACT2,DCBLD2,DDX21,DUSP7,EMP1,FHL2,FZD4,GABBR1,GPRC5A,IRF1,ITGA5,JCAD,KLHDC10,KRT17,LRRN4,NFKBIZ,NPTXR,NUFIP2,OSGEPL1,PAQR8,PDGFB,PRR15L,RND3,RWDD2A,SBK1,SEPHS2,SERPINA5,SPN,SPRY4,SRC,SULT1C4,TAF1A,TAP2,TESK2,UPP1,VAV3,ZFP36L1 |
| miR-1973 (miRNAs w/seed CCGUGCA) | mature microRNA | | 3.44E-02 | AFAP1,APOBEC3C,B3GNT9,BAZ2B,BDP1,CXCL8,FAM131B,GATA4,HOXB6,INHBA,KIF26B,MAFF,PCYT1B,PHLDA2,RELB,SEPHS2,TBC1D2,ZNF846 |
| miR-3075 (and other miRNAs w/seed CUGCUGG) | mature microRNA | | 3.46E-02 | ADH6,AMOT,BCL2,BNIP3L,CAPRIN2,CCND1,CISH,CTD_3074O7.11,DDIAS,DRICH1,FEM1A,FOSL1,GUCY2C,HRH1,LIF,LRCH1,MOCS1,MYCN,PER2,PHLDA2,PRUNE1,RASL12,RBBP8NL,REEP6,SCRN2,SERPINA10,SLC26A4,SLCO2B1,SNX33,SORBS2,SPTSSB,TMEM141,TPD52L1,TUBB3,ULBP2,VGLL3,VSIR,YOD1,ZFP36L1,ZNF710 |
| miR-1304-5p (miRNAs w/seed UUGAGGC) | mature microRNA | | 3.47E-02 | ACAD8,ATF3,BCL2,C2orf72,CAPRIN2,CDC14B,CROT,DPYSL3,EFNA1,FAM114A1,GPRC5A,KLF6,MDM2,MYC,NAV3,NBPF10 (includes others),NPIPA8,PIGZ,PLXNC1,RAB3D,SLC26A2,SLC2A11,SOCS3,SPIRE1,SULT1C4,TENT5A,TTBK2,ZFP36L1 |
| miR-1207-3p (and other miRNAs w/seed CAGCUGG) | mature microRNA | | 3.50E-02 | ABHD4,AQP11,B4GALNT2,BHLHE40,C19orf54,CCDC69,CHST13,CHST9,CXCL8,CXCR4,DIO1,DUSP6,EPST11,ESAM,ETS1,F7,GREM1,IRF1,LAMC2,MDM2,MXD4,NPTXR,NR1D1,PBX1,PDE6A,PER2,RCL1,RHBG,SLC25A19,SMTNL2,SOCS3,STC2,TAF1A,TENT5A,TMEM139 |
| ITK | kinase | Activated | 3.52E-02 | CXCL8,FOS,LPAR6,MYC,SLC2A1,SPRY2 |
| miR-636 (miRNAs w/seed GUGCUUG) | mature microRNA | | 3.53E-02 | ACSL1,ALDH3B2,CCND1,CLCF1,CROT,DLX1,DST,FERMT2,GREM1,HK2,ITGA2,KLHL18,PARD6B,RBBP8NL,RND3,SERPINA10,SNX33,SOCS3,SPTLC3,SUOX,TMEM14A,VAV3,ZNF239 |
| miR-4720-5p (and other miRNAs w/seed CUGGCAU) | mature microRNA | | 3.59E-02 | ARPIN/ARPIN-AP3S2,CXCL8,DCUN1D3,DLX1,DST,EPO,FOSL1,FRAS1,GPAT3,GPC5,GREB1L,GREM1,GSTK1,ITGA5,KLF4,LRRN4,MOCS1,MYBL1,NBPF10 (includes others),NKX1-2,NOCT,NRXN3,PCYT1B,PDE6A,REEP1,RGN,RNASE4,SLC30A7,SLC4A7,SOSTDC1,VAV3 |
| miR-8060 (miRNAs w/seed CAUGAAG) | mature microRNA | | 3.59E-02 | ANKRD1,C19orf54,CA8,CCDC69,CDK19,CISH,CNTN6,CXCL8,EPST11,MTMR9,PIGZ,PLEK2,PRR15L,SELENOW,SH3BGRL,SPRY2,SPTLC3,TMEM139,TPD52L1,UNC93A |
| miR-6883-3p (miRNAs w/seed UCCCUAU) | mature microRNA | Inhibited | 3.60E-02 | ALDH6A1,ARPIN/ARPIN-AP3S2,BCL10,BHLHE40,BIRC3,CHST9,CYP1A1,ESAM,ETS1,FOXA1,ITGA2,MAFF,PHGDH,RASSF6,SLC7A11,TBX3,TTBK2,VGLL3,ZNF441 |
| miR-323a-3p (miRNAs w/seed CCAAUAC) | mature microRNA | Inhibited | 3.60E-02 | AEN,ATF3,ERRFI1,[illegible],INHBA,KLHL13,NABP1,NBPF10 (includes others),NOCT,RHOB,RSC1A1,SELENOW,UPB1,VGLL3 |
| HOXA9 | transcription regulator | | 3.61E-02 | ADD3,ALDH1A1,BCL2,CISH,FCGRT,IER3,JUNB,MAFG,MYC,VIM |
| LEPR | transmembrane receptor | Activated | 3.61E-02 | BCL2,COL4A1,EDN1,FOS,GADD45B,GATA4,LDLR,LIF,SERPINE1,SOCS3 |
| miR-3200-5p (miRNAs w/seed AUCUGAG) | mature microRNA | | 3.62E-02 | ATF3,CDC14B,CPM,CTD_3074O7.11,CXCR4,DDIAS,EGR2,FBXL6,FOS,GATM,ID1,MXD4,PBX2,PBXIP1,PLEK2,PTGS2,SDC2,SOSTDC1,SPATA2,TINAGL1,UPB1,ZNF239 |
| miR-3934-3p (miRNAs w/seed GCUCAGG) | mature microRNA | | 3.62E-02 | C19orf54,CSRNP1,CYP1A1,CYP27A1,EPO,FAM114A1,FMN1,FOSL1,GALNT6,GAS2,GLYCTK,GSTA4,IHH,LDHD,LDLR,LIF,MAFF,MOSMO,MTMR9,MYC,NFKBIE,NFKBIZ,NPIPA8,NPIPB13,NPTXR,NSUN7,NUAK2,PALD1,PDE6A,PHC2,PIM3,PLK3,RHOB,SOCS3,TAGLN,TMEM37,TNS4,ZFP36L1 |
| NR4A3 | ligand-dependent nuclear receptor | | 3.63E-02 | BIRC3,CCND1,CEBPA,FBP1,TGFB3 |
| DDX5 | enzyme | | 3.63E-02 | CCND1,FOS,FOSL1,MYC,NFAT5 |
| BCL11B | transcription regulator | | 3.63E-02 | BCL2,CXCL8,MDM2,NCEH1,SPRY1 |
| miR-142-3p (and other miRNAs w/seed GUAGUGU) | mature microRNA | | 3.66E-02 | ACSL1,EGR2,FAM114A1,GAB1,GPCPD1,HSPE1-MOB4,IER3,MOCS1,MPZL2,MTMR9,OSR2,PARD6B,RAB3D,RBM47,RNASE4,SH3BGRL,SPRED1,SULT1C4,TESK2,TIPARP,USH1C,ZBTB20,ZNF430 |
| COMMD3-BMI1 | transcription regulator | | 3.67E-02 | FOXA1,HOXB3,HOXB6,HOXB9 |
| TNC | other | | 3.67E-02 | BCL2,FOS,MMP1,MYC |
| CDK2 | kinase | | 3.67E-02 | CDK2,FOS,MYC,TNFAIP3 |
| miR-561-5p (miRNAs w/seed UCAAGGA) | mature microRNA | | 3.69E-02 | BIRC3,CCDC69,DCBLD2,DDX21,EGR4,FERMT2,GOLT1A,GRIK4,INHBA,METTL7A,MXD1,NCEH1,PLK2,PRR15L,RGS2,RHOB,SESN3,SLC25A19,SLC26A2,SLC30A1,SLC9A3R1,SPRED1,SULT1E1,TMPRSS2,TNFRSF10A,TNS4,TPD52L1,TXNIP,USH1C,XKR6,YOD1 |
| miR-4653-3p (miRNAs w/seed GGAGUUA) | mature microRNA | | 3.71E-02 | ADD3,C21orf91,CCDC69,CHRNB1,CXCL8,CXXC5,ESRRA,FERMT2,FGG,FOSL1,FZD4,GALNT6,GNG2,IL18,MDM2,MYBPH,NPIPA8,NRXN3,PAQR8,PBXIP1,PDGFB,PDZK1,PHYHIPL,PLPP1,RWDD2A,SMTNL2,SPTLC3 |
| Nr1h | group | | 3.72E-02 | ALDH1A1,BCL2,C3,CXCL8,EDN1,NFKB2,NFKBIZ,PTGS2,RGN |
| alitretinoin | chemical drug | | 3.72E-02 | BCL2,CCND1,DUSP1,FOS,IRF1,MMP1,MMP11,RXRA,VIM |
| PYCARD | transcription regulator | | 3.73E-02 | CXCL8,IL18,PTGS2 |
| PSMD10 | transcription regulator | | 3.73E-02 | CCND1,CXCL8,ICAM1 |
| HSPD1 | enzyme | | 3.73E-02 | CXCL2,ICAM1,SOCS3 |
| KAT2A | enzyme | | 3.73E-02 | CCND1,TNFRSF10A,TNFRSF10B |
| diazoxide | chemical drug | | 3.73E-02 | BCL2,EGR1,MYC |
| [illegible] | chemical reagent | | 3.73E-02 | C3,FGA,SOCS3 |
| colistin | biologic drug | | 3.73E-02 | CCND1,CRYL1,FOS,GAS6,JUNB,NAV2,SPIRE1 |
| miR-5585-5p (miRNAs w/seed GAAGUAC) | mature microRNA | | 3.74E-02 | AQP11,CCDC69,CFLAR,CFTR,CPEB4,DLX1,GALNT6,MXD1,OAT,PIGZ,RHOB,SACS,STK38,TMEM37,UGDH,ULBP2,ZNF486 |
| miR-876-3p (miRNAs w/seed GGUGGUU) | mature microRNA | | 3.77E-02 | APOBEC3C,C19orf54,CEBPA,CLDN3,FHL3,GRIK4,HEXIM2,IGF2,ITPRID2,KLHDC10,MCL1,MMP11,MVD,NAV3,NBPF10 (includes others),NFAT5,NSUN7,NUAK2,PCYT1B,RASL12,RBM47,ROM1,SORBS2,SULT1C4,TAF1A,TENT5A,TGFB3,VGLL3,ZNF382,ZNF441 |
| KDM1A | enzyme | | 3.78E-02 | DNMT3B,GATA4,IGFBP4,MPC2,MYC,RIN1,SERPINE1,TJP3,ZBTB20,ZNF165 |
| miR-292-3p (and other miRNAs w/seed AGUGCCG) | mature microRNA | | 3.78E-02 | BRPF1,C19orf54,CFTR,EGR4,EPN3,HSPE1-MOB4,LHX6,PLD6,QPRT,REEP1,RHOBTB2,RND1,RNF32,SLC9A2,SMTNL2,UBASH3B,XKR6,YRDC,ZBTB20,ZNF385A |
| nitroglycerin | chemical drug | | 3.80E-02 | CXCL8,PLAUR |
| tannic acid | chemical toxicant | | 3.80E-02 | BCL2,PTGS2 |
| teriflunomide | chemical drug | | 3.80E-02 | ICAM1,TRPC6 |
| coal tar | chemical drug | | 3.80E-02 | CYP1A1,PTGS2 |
| APOL1 | transporter | | 3.80E-02 | CXCL2,MUC13 |
| T 0070907 | chemical reagent | | 3.80E-02 | CEBPA,PTGS2 |
| TRIM25 | transcription regulator | | 3.80E-02 | MDM2,SPN |
| LTB4R2 | G-protein coupled receptor | | 3.80E-02 | CXCL8,MMP1 |
| PSRM1 | other | | 3.80E-02 | IGFBP4,MDM2 |
| FFAR1 | G-protein coupled receptor | | 3.80E-02 | CYP1A1,SLC2A1 |
| ruxolitinib | chemical drug | | 3.80E-02 | BCL2,MCL1 |
| astragalin | chemical - endogenous non-mammalian | | 3.80E-02 | ICAM1,PTGS2 |
| GPR132 | G-protein coupled receptor | | 3.80E-02 | EGR1,ICAM1 |
| ACVR2A | kinase | | 3.80E-02 | ICAM1,ID1 |
| CCL1 | cytokine | | 3.80E-02 | ITGA2,SERPINE1 |
| RLIM | enzyme | | 3.80E-02 | SERPINE1,VIM |
| DUSP4 | phosphatase | | 3.80E-02 | DUSP1,MMP1 |
| HCK | kinase | | 3.80E-02 | MDM2,SRC |
| PDE3B | enzyme | | 3.80E-02 | SERPINE1,SOCS3 |
| IL1R2 | transmembrane receptor | | 3.80E-02 | CXCL2,CXCL8 |
| PTCSC3 | other | | 3.80E-02 | CCND1,MYC |
| WWP2 | enzyme | | 3.80E-02 | EGR2,VIM |
| RUVBL2 | transcription regulator | | 3.80E-02 | CCND1,CDKN2C |
| SART1 | other | | 3.80E-02 | EPO,SERPINE1 |
| PIKFYVE | kinase | | 3.80E-02 | ALDH1A1,ATF3 |
| CEBPG | transcription regulator | | 3.80E-02 | CXCL8,F7 |
| IL18BP | other | | 3.80E-02 | CXCL8,IL18 |
| JMJD1C | enzyme | | 3.80E-02 | EGR1,ID1 |
| WNT10B | other | | 3.80E-02 | ACAD8,CEBPA |
| UBR5 | enzyme | | 3.80E-02 | CCND1,MDM2 |
| COL17A1 | other | | 3.80E-02 | CXCL8,FZD4 |
| S100A7 | other | | 3.80E-02 | CXCL8,IL11 |
| TAS1R3 | G-protein coupled receptor | | 3.80E-02 | CEBPA,SLC7A11 |
| J11-Cl | chemical reagent | | 3.80E-02 | CXCL2,PTGS2 |
| acriflavine | chemical toxicant | | 3.80E-02 | MYC,SOCS3 |
| oligomycin | chemical - endogenous non-mammalian | | 3.80E-02 | BCL2,PDGFB |
| 2-aminoethoxydiphenylborane | chemical reagent | | 3.80E-02 | CYP1A1,PTGS2 |
| zileuton | chemical drug | | 3.80E-02 | ICAM1,PTGS2 |
| clioquinol | chemical drug | | 3.80E-02 | CCND1,FOS |
| orlistat | chemical drug | | 3.80E-02 | CXCL8,ICAM1 |
| manumycin A | chemical reagent | | 3.80E-02 | CXCL8,PTGS2 |
| lidocaine | chemical drug | | 3.80E-02 | BCL2,FOS |
| kukoamine A | chemical - endogenous non-mammalian | | 3.80E-02 | BCL2,VIM |
| rosmarinic acid | chemical - endogenous non-mammalian | | 3.80E-02 | CXCL8,PTGS2 |
| pterostilbene | chemical drug | | 3.80E-02 | BCL2,CCND1 |
| Z-DEVD-FMK | chemical - protease inhibitor | | 3.80E-02 | CXCL2,MCL1 |
| puerarin | chemical drug | | 3.80E-02 | BCL2,PTGS2 |
| scopolamine | chemical drug | | 3.80E-02 | ACHE,FOS |
| tyrphostin AG 1296 | chemical - kinase inhibitor | | 3.80E-02 | ICAM1,PTGS2 |
| allopregnanolone | chemical - endogenous mammalian | | 3.80E-02 | FOS,PTGS2 |
| canrenoate potassium | chemical drug | | 3.80E-02 | EDN1,PTGS2 |
| Cu2+ | chemical - endogenous mammalian | | 3.80E-02 | CCND1,CYP1A1 |
| miR-6734-5p (and other miRNAs w/seed UGAGGGG) | mature microRNA | | 3.81E-02 | ACOT1,ANXA8,ARRDC3,BDP1,CCDC69,CISH,DENND11,DNMT3B,DUSP2,EMP1,EPB41L1,ETS1,FAM20C,FGFR1,FOXA1,GATA4,GLYCTK,GOLT1A,HES4,HSPA1L,IER5,IGFBP4,IL18,IRF1,ITGA2,ITGA5,IYD,KIFC3,KLHL21,MAFG,MOCS1,NFKB2,NPTXR,PAQR8,PLEKHG3,PRR15L,RAB3D,RAB6B,RASAL2,RASL12,REXO1,RIMS4,RNASE1,SBK1,SERINC5,SPRY1,SPRY4,SRC,STK38,TDO2,THRA,TNFAIP3,VSIR,ZFP36L1 |
| miR-3189 (and other miRNAs w/seed GGGACUG) | mature microRNA | | 3.82E-02 | APOL1,ARPIN/ARPIN-AP3S2,ATAD3B,BCL2,BIRC3,CCND1,CDC14B,DHRS3,DUSP2,EGR1,ERBB3,FHL2,GOLT1A,GREM1,HOXB6,IGF2,IL17RE,IYD,KLHL21,KRT17,MAFF,MAFG,MTMR9,NIPSNAP1,PER2,PHC2,PRUNE1,REEP6,RIMS4,RNASE1,SPN,SLC2A11,SULT1C2,SULT1C4,THAP8,TNFRSF10A,TPD52L1,WBP1,ZFP36L1 |
| PRMT1 | enzyme | | 3.84E-02 | BCL2,CCND1,KLF4,SERPINE1,VIM |
| [illegible] | chemical drug | | 3.84E-02 | EFNB2,HOXB3,MYC,MYCN,TNFRSF10B |
| miR-664a-5p (miRNAs w/seed GGGCUAA) | mature microRNA | | 3.84E-02 | ALDH3B2,C19orf54,C1orf116,CDC14B,CFLAR,CSRNP1,EGR2,ELK3,FAM43A,FBXL20,FERMT2,GALNT6,GLYCTK,GPAT3,GREB1L,IGFBP4,LDHD,LHX6,MAFG,MOCS1,NBPF10 (includes others),PHC2,REXO1,RND1,SBK1,SPEN,TBX3,VPS45,ZFP36L1 |
| miR-877-3p (miRNAs w/seed CCUCUUC) | mature microRNA | | 3.85E-02 | ABHD4,ADH6,BIRC3,C1orf116,C6orf141,CSRNP1,CXCL2,DIO1,DST,ERBB3,ESAM,FAM131B,FSTL3,GADD45B,GNG2,HFE,HOXB6,IER5,IYD,KLF6,NBR1,NRXN3,NSUN7,PAIP2B,PBX2,PMM1,RBM47,RIMS4,ROM1,RWDD2A,SEPHS2,SPRED1,UBASH3B,ZNF142,ZNF430 |
| PRDM5 | transcription regulator | | 3.88E-02 | GADD45B,MYC,NAV2,RUNX1,SOCS3,TINAGL1 |
| puromycin aminonucleoside | chemical reagent | | 3.89E-02 | FGA,IER3,LAMC2,MYC,TNFRSF12A,UPP1,VIM |
| miR-656-5p (miRNAs w/seed GAGGUUA) | mature microRNA | | 3.90E-02 | ALPK2,ARHGEF10L,BCL10,BDP1,CABLES2,CLDN3,ELK3,EPO,GREM1,HFE,HK2,HOXB3,HPN,IL18,IYD,MMP1,MYC,NBPF10 (includes others),PDCD4,PHYHIPL,RAB3D,RAB6B,RHOB,RNASE1,ROM1,SLC19A3,SNX33,TGFB3,TRIB1,UNC93A |
| miR-3065-5p (and other miRNAs w/seed GGAGUGC) | mature microRNA | | 3.92E-02 | ASRGL1,CASP6,CHRNB1,CYP2W1,EFNA1,EPO,FBP1,FOSL1,GAS2,GREM1,IER3,IL18,ISG20L2,MAFF,MXD1,NOCT,NSRP1,PAK1,RBM47,SLC19A3,SMIM14,ZNF430,ZNF486 |
| miR-431-5p (and other miRNAs w/seed GUCUUGC) | mature microRNA | | 3.92E-02 | C19orf54,CGAS,DST,EFNA1,EPB41L1,EPST11,ERBB3,FOXA1,GSTA2,ICAM1,IYD,KLF6,METTL7A,MOCS1,NPIPA8,NRXN3,PHLDA1,PRUNE1,SPN,SLC2A1,SLC4A8,SLCO2B1,ZNF486 |
| miR-6759-5p (miRNAs w/seed CGGGCCU) | mature microRNA | | 3.92E-02 | AFAP1,AMER3,BCL2,CHST13,FAM131B,FEM1A,GABBR1,HOXB6,HPN,IER2,IER3,KLHL21,L1CAM,MAFG,MAPK,PPP1R3C,RASA4,RASL12,RND1,SBK1,SLC29A4,SLC2A11,TMEM37 |
| lipid A | chemical toxicant | Activated | 3.94E-02 | C3,CGAS,CXCL8,ITGA5 |
| RBM5 | other | | 3.94E-02 | BCL2,BIRC3,ICAM1,MCL1 |
| N-[N-(3,5-difluorophenacetyl-L-Ala)]-S-phenylglycine t-butyl ester | chemical - protease inhibitor | | 3.94E-02 | CXCR4,PDGFB,RELB,RND3 |
| miR-6716-5p (miRNAs w/seed GGGAAUG) | mature microRNA | | 3.96E-02 | ACSF2,CA8,CDC14B,DNMT3B,DRICH1,EMP1,FAM131B,FGA,FJX1,GOLT1A,GSTK1,HK2,HOXB6,HSPA1L,IGF2,IGFBP4,MAFF,MXD1,NUAK2,PARD6B,PLD6,PPP2R2B,RBM47,RSC1A1,SBK1,SH3BGRL,SLC30A7,SOAT2,SPRED1,SRC,UPB1,UPRT,ZFP36L1 |
| miR-290a-3p (and other miRNAs w/seed AGUGCCC) | mature microRNA | | 3.96E-02 | AEN,BRPF1,C19orf54,CDC14B,CDK19,CFTR,CISH,CLDN3,EPN3,EPO,ERBB3,ETS1,FAM131B,GATA4,IGSF9,MOCS1,PCYT1B,PLXNC1,PRRG3,QPRT,REEP1,RELB,SERPINA4,SPRY1,TDRKH,TIPARP,YRDC,ZBTB20 |
| miR-526b-5p (miRNAs w/seed UCUUGAG) | mature microRNA | | 3.96E-02 | ACAD8,AMACR,ATF3,CDK2,CHST9,DST,FAM3B,FOXA1,GOLT1A,GPRC5C,IYD,MDM2,MMP1,NPIPA8,PAH,PAQR8,PCYT1B,PXMP4,RAB3D,SLC19A3,SLC2A1,TDRKH,TENT5A,TMEM80,ZFP36L1 |
| miR-921 (miRNAs w/seed UAGUGAG) | mature microRNA | | 3.96E-02 | ADD3,ALDH1A1,APBB1,BCL10,BDP1,C19orf54,C1orf116,CNTN6,ERRFI1,FAM135A,GCNT3,GREM1,IER5L,IYD,KLHL13,KLHL18,MOSMO,NFKBIZ,NPTXR,NSUN7,REEP1,RWDD2A,SULT1C4,VGLL3 |
| miR-1183 (miRNAs w/seed ACUGUAG) | mature microRNA | | 4.00E-02 | AMACR,BIRC3,C21orf91,CCNL1,CHST9,CXCR4,ERRFI1,FERMT1,GNAI1,GPCPD1,GSTA4,MOSMO,NBR1,NFAT5,RNASE1,RSC1A1,RWDD2A,SLC9A2,SPRY1,SPRY2 |
| miR-653 (and other miRNAs w/seed GGUUGCC) | mature microRNA | | 4.00E-02 | APOBEC3C,BDP1,C19orf54,CLCF1,CPM,CXCL8,ERRFI1,FGD3,GPT2,GSTK1,ITGA2,LHX6,MUC13,NBPF10 (includes others),NIPSNAP1,PLXNC1,RASA4,RASSF6,RBBP8NL,RBM47,SOAT2,SPRY2,STC2,TTBK2,ZNF846 |
| kanamycin A | chemical drug | | 4.06E-02 | CCND1,CRYL1,FOS,GAS6,JUNB,NAV2,SPIRE1 |
| ciprofloxacin | chemical drug | | 4.06E-02 | ACSL6,EPO,ICAM1,LDLR,MPP1,SPRED1,VIM |
| miR-3162-5p (miRNAs w/seed UAGGGAG) | mature microRNA | | 4.06E-02 | DCBLD2,DIO1,DLX2,ERBB3,FBP1,FOSL1,GADD45A,GATA4,GREM1,HOXB9,IDH1,IGFBP4,IYD,JCAD,KBTBD7,KIF26B,L1CAM,NDRG1,NSUN7,PAX8,PBX1,PBX2,PBXIP1,PIK3C2B,RGS2,SELENBP1,SEMA4G,SNX33,SPRY1,SPRY4,TAF1A,TIPARP,ZFP36L1,ZNF385A,ZNF710 |
| BMI1 | transcription regulator | | 4.06E-02 | FBXW7,HK2,HOXB9,SPN,VIM |
| hydroxyurea | chemical drug | | 4.06E-02 | CCND1,EDN1,GADD45A,MDM2,MYC |
| miR-4532 (miRNAs w/seed CCCGGGG) | mature microRNA | | 4.06E-02 | ARPIN/ARPIN-AP3S2,BBS2,C19orf54,CHST13,DLX2,EPN3,FAM20C,GPRC5C,HOXB6,IER2,IER5,IER5L,IGF2,KANK2,MAFG,MAPK,MVD,PCYT1B,PLEC,RASA3,RHOB,SBK1,SERINC5,SERPINF2,SH2D3A,SLC26A2,SLC29A4,SOCS3,THRA,TNS4,TRIM8,ZNF382,ZNF385A,ZNF430 |
| miR-185-5p (and other miRNAs w/seed GGAGAGA) | mature microRNA | | 4.07E-02 | ACOT1,ADH4,ADH6,BRPF1,CTD_3074O7.11,CXCL8,CXCR4,DIO1,DLX2,DNMT3B,DUSP7,EFNA1,EHMT2,EPB41L1,EPN3,ESAM,FJX1,FOSL1,FZD4,GATA4,GSTA4,HFE,HK2,HOXB6,IER5,IGFBP4,KANK2,LDLR,LIF,MDM2,MTMR9,NR1D1,PAK6,PBX2,PCYT1B,PRUNE1,RAB3D,RBM47,RNASE1,SBK1,SESN1,SOCS3,SPATA2,TAF1A,TAP2,THRA,TMEM139,TTBK2,USH1C,VGLL3,ZNF385A,ZNF473 |
| JNK1/2 | group | | 4.11E-02 | BCL2,PTGS2,RHOB |
| p70 S6k | group | | 4.11E-02 | CCND1,PDCD4,VIM |
| Pka catalytic subunit | group | | 4.11E-02 | CXCL8,ESRRA,NR4A1 |
| AMH | growth factor | | 4.11E-02 | CXCL2,IER3,IRF1 |
| NOTCH4 | transcription regulator | | 4.11E-02 | EFNB2,SERPINE1,TAGLN |
| NCL | other | | 4.11E-02 | MDM2,MYC,PDCD4 |
| CTSS | peptidase | | 4.11E-02 | COL4A1,ITGA5,ITGA7 |
| BGN | other | | 4.11E-02 | CCND1,CXCL8,SERPINE1 |
| SLC9A3 | ion channel | | 4.11E-02 | CCND1,TAP2,UPP1 |
| CPE | peptidase | | 4.11E-02 | BCL2,LDLR,MYC |
| ESRRB | ligand-dependent nuclear receptor | | 4.11E-02 | BCL2,HK2,MCL1 |
| acetylcholine | chemical - endogenous mammalian | | 4.11E-02 | FOS,MMP1,SOCS3 |
| miR-3944-5p (miRNAs w/seed GUGCAGC) | mature microRNA | | 4.11E-02 | AEN,AFAP1,AMER3,AQP11,BCL2,CA8,CYP2W1,DACT2,DCUN1D3,DDIAS,EGR2,EPN3,ERBB3,F7,FAM114A1,FGA,GLYCTK,GUCY2C,KLHL18,KLHL21,LRCH1,MTF1,MVD,PER2,RASA3,SPATA2,STK38,TENT5A,TGFB3,TMEM37,TTBK2 |
| miR-668-5p (miRNAs w/seed GCGCCUC) | mature microRNA | | 4.14E-02 | CLCF1,DST,ESRRA,FAM20C,FOSL1,GABBR1,GPRC5A,IER3,KIFC3,LIF,MYBPH,NABP3,NSUN5,PLEC,THRA,TRIM8,ZMYM3 |
| miR-5684 (miRNAs w/seed ACUCUAG) | mature microRNA | | 4.14E-02 | CGAS,DCAF11,DNMT3B,GCNT1,GNG2,HIVEP2,HOXB3,KLC4,KLF10,MCL1,MMP1,NABP1,NCEH1,PLK2,RM2,SERPINA6,SLC26A2,ZNF239 |
| miR-769-5p (miRNAs w/seed GAGACCU) | mature microRNA | | 4.15E-02 | ARPIN/ARPIN-AP3S2,C19orf54,CCNL1,DRICH1,EPO,FAM20C,FHL3,GALNT6,ICAM1,ID1,KLF10,LRCH1,PARD6B,PIK3C2B,PRUNE1,RAB3D,RWDD2A,SERINC5,TENT5A,VGLL3,YPEL5 |
| miR-200a-5p (and other miRNAs w/seed AUCUUAC) | mature microRNA | | 4.15E-02 | C11orf71,C21orf91,EHHADH,EPST11,GNG2,GPR180,KLF6,NR4A1,RGS2,SEPHS2,SULT1C2,UGT2A3,ZC3H12C,ZNF430,ZNF441 |
| miR-6886-3p (miRNAs w/seed GCCCUUC) | mature microRNA | | 4.17E-02 | APOL1,C1orf116,CCDC69,ETS1,FERMT1,FMN1,GPC5,GPR180,HFE,ITGA5,ITGA7,MMP11,NABP3,NPTXR,PAQR8,PBX2,PXMP4,RGN,SBK1,SLC25A25,SLC2A1,SNX33,SOCS3,TGFB3,VGLL3,ZBTB20,ZNF430,ZNF596,ZNF710 |
| HOTAIR | other | | 4.22E-02 | ABL2,ICAM1,LAMC2,MYC |
| KLF1 | transcription regulator | | 4.22E-02 | CDKN2C,ICAM4,MPP1,SLC2A1 |
| IL12A | cytokine | | 4.22E-02 | BCL2,CFLAR,ICAM1,SOCS3 |
| MED13 | transcription regulator | | 4.22E-02 | ANKRD1,LIF,MYC,TMEM45B |
| hexamethylene bisacetamide | chemical reagent | | 4.22E-02 | BCL2,CXCL8,DDX21,[illegible] |
| miR-6801-5p (miRNAs w/seed GCAGGAA) | mature microRNA | | 4.22E-02 | ABL2,APOBEC3C,ARPIN/ARPIN-AP3S2,C19orf54,C1orf116,C2orf72,CCDC69,CYP2W1,DLX2,DUSP7,EFNA1,FOXA1,FZD4,GATA4,GPRC5C,HK2,KLHL18,KLHL21,LHX6,LIF,MAFG,MATN3,METTL7A,NBPF10 (includes others),NBR1,NDRG1,NFAT5,NIPSNAP1,NRXN3,NUAK2,PCYT1B,RAB3D,SEPHS2,SLC2A11,TDRKH,THRA,TUBB3,UBASH3B,ULBP2,ULK1,VCPKMT,VPS45,ZC3H12C,ZNF846 |
| miR-6078 (miRNAs w/seed CGCCUGA) | mature microRNA | | 4.23E-02 | AFAP1,CYP2W1,DLX1,DUSP4,EDN1,HAPLN3,KLHDC10,LIF,NDRG1,PER2,RIMS4,SLC9A3R1,SMIM14,SOCS3,SORBS2,TINAGL1,ZMYM3 |
| miR-466b (miRNAs w/seed UGCAUGU) | mature microRNA | | 4.23E-02 | ADH6,BCL2,C21orf91,C6orf141,CAPRIN2,CDK19,DPYSL3,DUSP7,EDN1,FBXW7,FGG,FOXA1,GPCPD1,GSTA2,HFE,IGF2,KLHL13,NABP1,RASAL2,RBM47,SH3BGRL,SULT1E1,TMPRSS2,UGT2A3,ZNF441,ZNF846 |
| miR-6779-3p (miRNAs w/seed GCCUCCC) | mature microRNA | | 4.26E-02 | APOBEC3C,APOL1,AS3MT,C1orf116,C6orf141,CCND1,CDC14B,CHRNB1,CYP2W1,DHRS3,EGR1,ERBB3,ESRRA,FA2H,FAM161A,FSTL3,GALNT6,ICAM4,IER2,IL11,IL17RE,IL18,ISG20L2,KLHL13,KRT17,LDLR,LIF,LRCH1,MAFF,MAFG,MAPK,MMP11,NABP3,NSRP1,NSUN5,PDGFB,PIM3,PLA2G12B,RIMS4,RNASE4,SAV1,SH2D5,SLC19A3,SLC2A11,SMIM14,SNX33,TESK2,THRA,TNFRSF10A,ZFP36,ZNF430,ZNF486,ZNF596 |
| miR-128-1-3p (and other miRNAs w/seed AGCCCUU) | mature microRNA | | 4.26E-02 | ARPIN/ARPIN-AP3S2,BDP1,BNIP3L,C21orf91,CAMSAP2,DCUN1D3,EGR2,GPC1,GPR180,HOXB6,KLHL13,MAFG,MOSMO,NAV3,PARD6B,PXMP4,RASAL2,SACS,SBK1,SLC19A3,SMIM14,SOSTDC1,SPATA2,TMEM141,TRIB1,VAV3,ZBTB20 |
| miR-516a-5p (and other miRNAs w/seed UUCAAAG) | mature microRNA | | 4.27E-02 | APOBEC3C,DDIAS,KLHL13,MAPK,MCL1,METTL7A,NFKBIZ,RHOB,SDC2,SELENOW,SERINC5,SERPINA6,SLC30A7,SOCS3,SPTSSB,THAP8,ULK1,ZBTB20,ZNF430 |
| miR-4728-3p (miRNAs w/seed AUGCUGA) | mature microRNA | | 4.27E-02 | APOBEC3C,ARRDC3,BCL10,BDP1,BIRC3,C1orf116,CEP170,DACT2,DDIAS,DST,DUSP6,FGFR2,FOXA1,LHX6,MPZL2,MXD4,NSRP1,PER2,PLEK2,RASL12,SLC30A7,SPRED1,TMEM80,TNFRSF10A,VCPKMT,VGLL3,YOD1 |
| vitamin E | chemical drug | | 4.29E-02 | BCL2,EGR1,KLF6,PTGS2,SERPINE1 |
| HAND2 | transcription regulator | | 4.29E-02 | CXCL2,CXCR4,GREM1,IL11,PTGS2 |
| levothyroxine | chemical - endogenous mammalian | | 4.29E-02 | BNIP3L,DIO1,FOS,IGFBP6,THRA |
| miR-7846-3p (miRNAs w/seed AGCGGAG) | mature microRNA | | 4.29E-02 | AMER3,CABLES2,EHD2,GYG2,HES4,MAFG,MDM2,METTL7A,NCEH1,PARD6B,RNF32,SMTNL2,SRC,ZFP36L1 |
| miR-6565-3p (miRNAs w/seed GACAGUA) | mature microRNA | | 4.29E-02 | ARRDC3,BRPF1,CHRNB1,DCBLD2,DDIAS,EFNB2,ELK3,EMP1,EPST11,FZD4,IER3,NR4A1,PAH,PDZK1,PHYHIPL,REEP1,RGN,SERPINA6,SPATA2,SPRED1,SPRY4,SPTSSB,TAGLN,TMEM14A,TPD52L1,TSC22D2,UGT2A3,XKRX,YPEL5,ZNF430,ZNF441 |
| miR-6888-3p (and other miRNAs w/seed UCUGUCU) | mature microRNA | | 4.30E-02 | ATAD3B,BDP1,C1orf116,CDK18,CNTN6,DST,ERBB3,ESAM,F7,FERMT1,GAS2,IER5,IL11,MMP1,MSRB1,NBR1,NIPSNAP1,RASGRF2,RASL12,REEP1,RM2,SERTAD1,SHB,SPRY4,VAV3,ZNF382 |
| miR-7158-5p (miRNAs w/seed GCUCAAU) | mature microRNA | | 4.33E-02 | AEN,C19orf54,DST,EHD2,ERRFI1,ETS1,FAM114A1,FBXL6,IER3,SEPHS2,SLC2A11,SOWAHA |
| miR-6863 (miRNAs w/seed AGACGUG) | mature microRNA | | 4.36E-02 | CABLES2,CAPRIN2,CISH,EGR1,FIG4,KLF6,MAPK,OSR2,PAX6,SERINC5,SLC9A3R1,SULT1C2,TMEM37,ZNF710 |
| miR-1296-3p (and other miRNAs w/seed AGUGGGG) | mature microRNA | | 4.36E-02 | ABHD4,CTD_3074O7.11,CYP2W1,EGR2,EPB41L1,FHL3,FOSL1,GOT1,GREB1L,KLHL18,L1CAM,LRRN4,NOTCH3,NPTXR,PALD1,PBX2,PIGZ,RAB3D,RHOB,SBK1,SEMA4G,SERPINA10,SNX33,SPRY4,SRC,TGFB3,THAP8,TINAGL1,TNFRSF12A,USH1C,VPS45,ZC3H12A |
| miR-3077-5p (and other miRNAs w/seed UGCGGAC) | mature microRNA | Activated | 4.37E-02 | ARPIN/ARPIN-AP3S2,BCL2,BNIP3L,ESPN,MOSMO,PBX1,REEP1,SCGN,SNAPC4,TMEM141,TMPRSS2,ZNF382,ZNF385A |
| black raspberry extract | chemical drug | | 4.40E-02 | DUSP6,GREM1,KLF10,MCL1,PHGDH,PLK3,SLC30A1 |
| miR-5189-3p (and other miRNAs w/seed GCCAACC) | mature microRNA | | 4.40E-02 | ACSF2,ACSS1,ARRDC3,C1orf116,COLEC11,DENND11,FA2H,FAM43A,IGFBP4,KLHL21,P2RY2,PALD1,PBX1,RASA4,SH3BGRL,SLC2A11,SLC4A8,UPRT,VPS45,XKRX,ZNF596 |
| miR-4436 (miRNAs w/seed ACUCGUG) | mature microRNA | | 4.46E-02 | ABHD4,ADH6,EGR4,FCGRT,GAS2,HES4,IGF2,PARD6B,RWDD2A,SAV1,SDC2,[illegible] |
| miR-609 (miRNAs w/seed GGGUGUU) | mature microRNA | Activated | 4.46E-02 | ARPIN/ARPIN-AP3S2,C1orf116,CDKN2C,CHST9,DACT2,EHD2,FGFR2,FHL2,GREM1,HEXIM2,IER3,IGF2,INHBA,KBTBD7,MPZL2,MSRB1,NBR1,NFKB2,NSUN5,PRUNE1,RIMS4,SEMA4G,SH2B3,SH3BGRL,SLC26A2,SPRY1,TMEM37,ZNF382 |
| SOX2-OT | other | | 4.47E-02 | ALDH1A1,VIM |
| PP2A | complex | | 4.47E-02 | PIM3,PTGS2 |
| chlorcyclizine | chemical drug | | 4.47E-02 | HPN,TAGLN |
| naringin | chemical - endogenous non-mammalian | | 4.47E-02 | BCL2,CXCL8 |
| highly active antiretroviral therapy | chemical drug | | 4.47E-02 | BCL2,CXCR4 |
| Sfk | group | | 4.47E-02 | BCL2,CXCL8 |
| METTL3 | enzyme | | 4.47E-02 | CISH,SOCS3 |
| HCAR1 | G-protein coupled receptor | | 4.47E-02 | EDN1,SLC16A3 |
| WWC1 | transcription regulator | | 4.47E-02 | PDGFB,VIM |
| S100A12 | other | | 4.47E-02 | CCND1,PDGFB |
| DEPTOR | other | | 4.47E-02 | ICAM1,VIM |
| AFAP1-AS1 | other | | 4.47E-02 | AFAP1,BCL2 |
| PLZ | chemical drug | | 4.47E-02 | ACHE,PTGS2 |
| PELI1 | enzyme | | 4.47E-02 | CFLAR,CXCL8 |
| PAK4 | kinase | | 4.47E-02 | BCL2,VIM |
| CTNNBIP1 | other | | 4.47E-02 | CCND1,TUBB3 |
| NTS | other | | 4.47E-02 | CXCL8,FOS |
| CCAT1 | other | | 4.47E-02 | MYC,VIM |
| UBA1 | enzyme | | 4.47E-02 | BIRC3,CCND1 |
| JUP | other | | 4.47E-02 | BCL2,MYC |
| SH3KBP1 | other | | 4.47E-02 | MYC,SERPINE1 |
| NACC1 | transcription regulator | | 4.47E-02 | BCL2,MATN3 |
| ETV6 | transcription regulator | | 4.47E-02 | ID1,SPRY4 |
| NUMBL | other | | 4.47E-02 | KLF4,MYC |
| HSPB8 | kinase | | 4.47E-02 | CXCL8,ICAM1 |
| PAWR | transcription regulator | | 4.47E-02 | BCL2,TNFRSF10B |
| PAX4 | transcription regulator | | 4.47E-02 | MYC,VIM |
| HSF4 | transcription regulator | | 4.47E-02 | LIF,VIM |
| CDH5 | other | | 4.47E-02 | BCL2,ICAM1 |
| BEX2 | other | | 4.47E-02 | BCL2,PLXNC1 |
| CXCL1 | cytokine | | 4.47E-02 | ICAM1,SOCS3 |
| HNRNPAB | enzyme | | 4.47E-02 | PTGS2,VIM |
| toremifene | chemical drug | | 4.47E-02 | IGF2,LDLR |
| bisphenol A diglycidyl ether | chemical reagent | | 4.47E-02 | ICAM1,SERPINE1 |
| roflumilast N-oxide | chemical - endogenous mammalian | | 4.47E-02 | CXCL8,ICAM1 |
| trientine | chemical drug | | 4.47E-02 | IGFBP4,SERPINE1 |
| zinostatin | biologic drug | | 4.47E-02 | BCL2,MDM2 |
| cucurbitacin B | chemical - endogenous non-mammalian | | 4.47E-02 | BCL2,MYC |
| NAD+ | chemical - endogenous mammalian | | 4.47E-02 | ANKRD1,TXNIP |
| androstenediol | chemical - endogenous mammalian | | 4.47E-02 | BCL2,TMPRSS2 |
| miR-3690 (miRNAs w/seed CCUGGAC) | mature microRNA | | 4.47E-02 | ADD3,APBB1,C19orf54,C1orf116,CCDC183,CCDC69,CDC14B,CFTR,DLX1,DUSP7,ESAM,GALM,GALNT6,GATA4,GRIK4,KLHL18,LIF,MAFF,MAFG,NDNF,SELENOW,SERPINA6,SULT1E1,TAP2,TRIM8,TYMSOS,UPB1 |
| miR-5587-3p (miRNAs w/seed CCCGGGG) | mature microRNA | | 4.48E-02 | BIRC3,CDC42EP1,CHST13,DLX2,FSTL3,GJB1,IER2,IER3,IER5L,IGF2,LDLR,LIF,MAPK,NKX1-2,NPTXR,RBBP8NL,SLC29A4,SMTNL2,TMEM37,UPRT,ZFP36L1,ZNF382,ZNF710 |
| miR-6834 (miRNAs w/seed GGCGCGA) | mature microRNA | | 4.48E-02 | ABL2,APOBEC3C,BRPF1,CCDC69,CDC14B,EHD2,FEM1A,GALNT6,HOXB6,ITGA2,REEP6,SPN,SH2D3A,ZNF430,ZNF846 |
| miR-6125 (miRNAs w/seed CGGAAGG) | mature microRNA | | 4.48E-02 | ATF3,CGAS,CHST9,DACT2,HAPLN3,HOXB9,IL11,KLF4,KLF6,MAFF,NPIPA8,TBC1D2,TMEM37,ZFP36L1,ZNF385A |
| 2-(4-acetoxyphenyl)-2-chloro-N-methylethylamine | chemical reagent | | 4.51E-02 | CXCL8,MMP1,SERPINA6 |
| NCF1 | enzyme | | 4.51E-02 | EDN1,ICAM1,SERPINE1 |
| choline fenofibrate | chemical drug | | 4.51E-02 | ACSL1,NR1D1,SERPINE1 |
| LATS1 | kinase | | 4.51E-02 | ERBB3,FOXA1,LATS1 |
| HMGN1 | transcription regulator | | 4.51E-02 | FGA,FOS,[illegible] |
| REN | peptidase | | 4.51E-02 | ADD3,FOS,ICAM1 |
| BMPR1B | kinase | | 4.51E-02 | ID1,PTGS2,SERPINE1 |
| TAF6 | transcription regulator | | 4.51E-02 | ATF3,GADD45A,IRF1 |
| MALP-2s | chemical reagent | | 4.51E-02 | CXCL2,MCL1,PTGS2 |
| apomorphine | chemical drug | | 4.51E-02 | BCL2,EGR1,FOS |
| arsenic | chemical toxicant | | 4.51E-02 | BIRC3,EPHX2,FOS |
| sterol | chemical - endogenous mammalian | | 4.51E-02 | ACSL1,IDH1,LDLR |
| obeticholic acid | chemical drug | | 4.51E-02 | CEBPA,CXCL2,ICAM1,PTGS2 |
| TCF7 | transcription regulator | | 4.51E-02 | CEBPA,FOS,FOXA1,SDC2 |
| K+ | chemical - endogenous mammalian | | 4.51E-02 | ATF3,EGR1,FOS,NR4A1 |
| tetrodotoxin | chemical drug | | 4.53E-02 | EGR1,HDAC9,ID1,IFRD1,NSUN7,PTGS2,REV3L,TAP2 |
| CX3CL1 | cytokine | | 4.53E-02 | DLX1,DLX2,EFNB2,ERBB3,ICAM1,NABP3,NDNF,TGFB3 |
| miR-580-5p (miRNAs w/seed UUGCACA) | mature microRNA | | 4.54E-02 | ACSL1,AEN,DUSP6,EGR4,FGA,GAB1,HSPE1-MOB4,IDH1,ITGA2,KLHDC10,MDM2,NSRP1,RCL1,RWDD2A,SCIN,SLC19A3,SLC2A11,SLC30A7,SMTNL2,SPRED1,SPRY2,TENT5A,TPD52L1,VGLL3,VIM,ZNF486,ZNF596 |
| miR-6513-5p (miRNAs w/seed UUGGGAU) | mature microRNA | | 4.55E-02 | ALDH3B2,AMER3,C19orf54,EMP1,EPST11,ETS1,FAM114A1,FOXA1,FOXP1,KBTBD7,MAPK,MATN3,NABP1,PDE6A,SOCS3,SOWAHA,TDO2,ULBP2 |
| miR-3190-3p (and other miRNAs w/seed GUGGAAG) | mature microRNA | | 4.60E-02 | APOBEC3C,CISH,CRYL1,DDIAS,DIO1,DPYSL3,FAM131B,GAB1,GLYCTK,HPN,ITGA2,JUNB,MAFG,MTF1,MXD4,NCEH1,NPIPA8,NR4A1,PLPP1,REEP1,RM2,RND1,SDC2,SLC4A7,TBC1D2,TMEM37,VGLL3,ZFP36L1 |
| miR-670-3p (miRNAs w/seed UUCCUCA) | mature microRNA | Inhibited | 4.65E-02 | ACSL1,AMACR,BDP1,BIRC3,C1orf116,C21orf91,COLEC11,CXCL2,DRICH1,DST,EFNB2,EMP1,ETS1,FZD4,GALM,ID1,KLF4,NRXN3,NSRP1,PBX1,PLA2G12B,RELB,RNF32,SLC30A1,SPEN,THAP8,TNFAIP3,TRPC6,UBASH3B,ZFP36L1,ZNF165,ZNF441 |
| TRAP1 | enzyme | | 4.66E-02 | BCL2,GNG2,GPT2,L1CAM,PPP2R2B,PTGS2 |
| POU2F2 | transcription regulator | | 4.66E-02 | CFLAR,FJX1,GATM,MYC,PRR7,TNFRSF12A |
| miR-4744 (miRNAs w/seed CUAAAGA) | mature microRNA | | 4.67E-02 | ADH4,BIRC3,CXCR4,CXXC5,GAS2,GNG2,GPRC5C,LPAR6,MTMR9,PAH,PDZK1,PHEX,REEP1,SDC2,SPRY1,SPTSSB |
| miR-6849-3p (miRNAs w/seed CCAGCCU) | mature microRNA | | 4.68E-02 | ABHD4,ANKRD1,ANXA8,APOBEC3C,ARPIN/ARPIN-AP3S2,ATF3,C19orf54,CA8,CCDC69,CDC14B,CHST9,DDIAS,DST,FAM131B,GNG2,GPC1,GPT2,GSTA4,IL11,KLF10,LIF,MANSC1,METTL7A,MTF1,NABP3,NOCT,PCYT1B,PIK3C2B,PLD6,RAB3D,RNASE4,SERPINA6,SLC30A7,SOCS3,SPTSSB,SULT1C4,SULT1E1,UGDH |
| NR1I2 | ligand-dependent nuclear receptor | | 4.69E-02 | ALDH1A1,CEBPA,CYP1A1,CYP27A1,EGR1,GLUL,HMGCS2,SULT1E1,TDO2 |
| miR-548as-3p (miRNAs w/seed AAAACGA) | mature microRNA | | 4.69E-02 | CLDN3,CXXC5,DCBLD2,GLUL,KLF6,LRCH1,TMPRSS2,ZNF382 |
| miR-135a-3p (and other miRNAs w/seed GUAACCG) | mature microRNA | | 4.69E-02 | FERMT1,ITGA6,KANK2,PLK2,PMM1,SDC2,SPRED1,TNFRSF10B |
| mibolerone | chemical drug | | 4.69E-02 | ACSL1,CCND1,DHRS3,KLF10,LDLR,MMP1,MYC,TMPRSS2 |
| miR-1287-3p (and other miRNAs w/seed UCUAGCC) | mature microRNA | | 4.70E-02 | ABL2,AQP11,BCL10,BCL2,CFTR,EHHADH,GLYCTK,GREM1,IYD,KLHL13,MMP11,MOSMO,NIPSNAP1,PDZK1,PLEK2,SLC26A2,SMIM14,SUOX,TMEM14A,TRIB1,VIM |
| miR-4449 (miRNAs w/seed GUCCGGG) | mature microRNA | | 4.70E-02 | ARRDC3,CPEB4,DENND11,FAM20C,HOXB9,KANK2,LDLR,MAPK,OSGEPL1,RASGRF2,REEP6,SBK1,SERPINF2,SH2B3,SH2D3A,TBX3,TNFRSF10B,ULK1,ZNF385A |
| Proinflammatory Cytokine | group | | 4.77E-02 | EPO,GLUL,ICAM1,NR4A1,SOCS3 |
| mir-15 | microRNA | | 4.77E-02 | BCL2,CCND1,MCL1,PLAUR,PTGS2 |
| miR-3118 (and other miRNAs w/seed GUGACUG) | mature microRNA | | 4.77E-02 | ALDH1A1,ALDH3B2,ATF3,B3GNT9,B4GALNT2,C1orf116,CACHD1,CHST9,DDIAS,DUSP4,EDN1,ESAM,GABBR1,GPAT3,MMP1,MOCS1,MYCN,PCYT1B,PDGFB,PHLDA2,PLD6,SERPINE1,SH2D5,SLC16A4,TSC22D2,XKR6,ZNF846 |
| miR-5091 (miRNAs w/seed CGGAGAC) | mature microRNA | Inhibited | 4.77E-02 | ABL2,BCL2,EPB41L1,FEM1A,GCNT1,GYG2,IER5L,KRT17,L1CAM,LIF,MAPK,MDM2,MXD1,RIMS4,STC2 |
| miR-4774-3p (miRNAs w/seed UUGCCUA) | mature microRNA | | 4.79E-02 | AMER3,ATF3,BNIP3L,C21orf91,DCBLD2,EPST11,FAM114A1,FERMT1,GAS2,GNAI1,GUCY2C,HRH1,IDH1,IER5L,IYD,L1CAM,NDNF,NSUN7,NUAK2,NUFIP2,PDE6A,RASL11A,SOWAHA,SPRY2,TDRKH,XKRX,ZNF441 |
| miR-340-3p (and other miRNAs w/seed CCGUCUC) | mature microRNA | | 4.80E-02 | BCL10,C19orf54,C6orf141,CACHD1,EHD2,GYG2,KLHL21,MANSC1,METTL7A,NABP3,NOCT,NUAK2,PARD6B,PCYT1B,PHC2,PIK3C2B,RASA4,RWDD2A,SLC16A4,SLC29A4,SMIM14,SOCS3,SULT1C4,ZFP36L1,ZNF430,ZNF596,ZNF846 |
| miR-1587 (and other miRNAs w/seed UGGGCUG) | mature microRNA | | 4.80E-02 | C19orf54,CCDC69,CRYL1,CYP1A1,DDIAS,DLX1,EHMT2,FA2H,FAM3B,FOSL1,GNG2,GPRC5A,GREM1,HOXB9,HRH1,IER3,IGFBP4,KLHL21,MANSC1,MOCS1,NCEH1,NIPSNAP1,NPTXR,NSUN5,P2RY2,PBXIP1,PER2,RBBP8NL,REXO1,RHOBTB2,RIMS4,SBK1,SEPHS2,SHB,SLC25A19,SNAPC4,SOCS3,SOWAHA,TAGLN,TDO2,TNFAIP3,TPD52L1,VAV3,ZC3H12A |
| PRMT5 | enzyme | | 4.82E-02 | CCND1,CEBPA,KLF4,MYC |
| APLN | other | | 4.82E-02 | BCL2,EGR1,FOS,MYC |
| NOS3 | enzyme | | 4.82E-02 | ATF3,COL4A1,ICAM1,PTGS2 |
| AIP | other | | 4.82E-02 | ATF3,CCND1,EGR1,FOS |
| KLF15 | transcription regulator | | 4.82E-02 | ACAD8,ACSL1,ACSS1,EHHADH |
| zVAD-FMK | chemical - protease inhibitor | | 4.82E-02 | BIRC3,FOS,MCL1,MDM2 |
| miR-7159-3p (miRNAs w/seed UUCUAUG) | mature microRNA | | 4.82E-02 | ALDH1A1,BCL10,CA8,CCND1,CPEB4,DIO1,HFE,ITPRID2,REEP1,SDC2,SLC3A1,SPRY2 |
| miR-6804-3p (miRNAs w/seed GCACCUG) | mature microRNA | | 4.86E-02 | ATAD3B,B3GNT9,CDC14B,CHRNB1,CSRNP1,ELK3,ETS1,FAM131B,FBXW7,GPAT3,GREM1,ITGA6,IYD,KIF26B,LHX6,LIF,LRCH1,LRRN4,MOCS1,MTF1,MYBPH,NFKBIE,PALD1,PCYT1B,PLK2,REV3L,SERPINA6,TESK2,TINAGL1,TMEM139,VSIR,ZNF846 |
| miR-4285 (miRNAs w/seed CGGCGAG) | mature microRNA | | 4.86E-02 | ID1,IGF2,KLHL18,LDLR,MVD,NDRG1,REXO1,ZNF385A |
| DNMT1 | enzyme | | 4.87E-02 | FOXA1,GATA4,IGF2,MYC,PTGS2,SLC7A11 |
| 6-hydroxydopamine | chemical toxicant | | 4.87E-02 | BCL2,BNIP3L,C3,FOS,MDM2,PTGS2 |
| DLL1 | enzyme | | 4.92E-02 | EFNB2,HOXB6,TAGLN |
| HOXB13 | transcription regulator | | 4.92E-02 | CCND1,CEP170,MYC |
| SND1 | enzyme | | 4.92E-02 | EMP1,ID1,PTGS2 |
| NFIL3 | transcription regulator | | 4.92E-02 | GADD45A,GADD45B,PTGS2 |
| NPC2 | transporter | | 4.92E-02 | AKR1C3,IL18,PTGS2 |
| IRAK3 | kinase | | 4.92E-02 | CXCL2,TNFAIP3,VIM |
| PD 123319 | chemical drug | | 4.92E-02 | PTGS2,SERPINE1,SPRY1 |
| ketoconazole | chemical drug | | 4.92E-02 | CXCL8,CYP1A1,GSTA2 |
| hemozoin | chemical - endogenous non-mammalian | | 4.92E-02 | CXCL2,CXCR4,ICAM1 |
| deoxynivalenol | chemical toxicant | | 4.92E-02 | CLDN3,CXCL8,ICAM1 |
| miR-1914-5p (miRNAs w/seed CCUGUGC) | mature microRNA | | 4.93E-02 | AEN,AMER3,ANKRD1,CLCF1,DDX21,DHRS3,ETS1,GREB1L,GSTA2,IER5,KLHDC10,PAX8,PAQR8,RM2,SEPHS2,SERPINA5,SLC16A3,SLC19A3,SMTNL2,TDO2,TMEM37,TNFRSF12A,WBP1,ZNF430,ZNF596 |
| metronidazole | chemical drug | | 4.94E-02 | CCND1,CRYL1,FOS,GAS6,JUNB,NAV2,SPIRE1 |
| miR-548s (miRNAs w/seed UGGCCAA) | mature microRNA | | 4.96E-02 | ADD3,ADH4,ARPIN/ARPIN-AP3S2,CASP6,CCDC69,CDK18,CHST9,DENND11,EGR1,IYD,LIF,MAFG,MDM2,NSRP1,OAT,PCYT1B,PER2,PHLDA2,PLXNC1,PRR15L,SLC19A3,SLC2A11,SLC4A8,SLCO2B1,TAGLN,TXNIP,UNC93A,USP53,ZNF441 |
| miR-124-5p (miRNAs w/seed GUGUUCA) | mature microRNA | | 5.00E-02 | C1orf116,CABLES2,CHST9,DDIAS,DST,EFNB2,ELK3,F7,GAS2,GREM1,IER3,IGF2,IRF1,IYD,KLHDC10,KLHL13,KLHL18,NABP1,PAQR8,PER2,REV3L,SLC3A1,SOWAHA,SPRY4,SPTLC3,TBX3,TENT5A,TMEM139,TMEM37,USH1C,ZNF486 |

**Supplementary Table S17. Unique and overlapping Canonical Pathways for the compassion of the conditions "Caco-2_SARS-CoV-2" and "Calu-3_SARS-CoV-2".** Comparative analysis was done using VENNY 2.1. Data visualization was done by R-based Venn diagrams shown in Figure 5.

**34 elements included exclusively in "Caco-2_SARS-CoV-2":**
HOTAIR Regulatory Pathway
Cell Cycle: G1/S Checkpoint Regulation
Coagulation System
CDK5 Signaling
ErbB2-ErbB3 Signaling
Regulation of Cellular Mechanics by Calpain Protease
Estrogen-mediated S-phase Entry
Renal Cell Carcinoma Signaling
Actin Nucleation by ARP-WASP Complex
Glioblastoma Multiforme Signaling
CXCR4 Signaling
Retinoate Biosynthesis I
Wnt/β-catenin Signaling
Bladder Cancer Signaling
Regulation of Actin-based Motility by Rho
Telomerase Signaling
Arginine Degradation I (Arginase Pathway)
Apelin Endothelial Signaling Pathway
Proline Biosynthesis II (from Arginine)
Cholecystokinin/Gastrin-mediated Signaling
Melanoma Signaling
UVC-Induced MAPK Signaling
Arginine Biosynthesis IV
Arginine Degradation VI (Arginase 2 Pathway)
Selenocysteine Biosynthesis II (Archaea and Eukaryotes)
GDP-mannose Biosynthesis
EIF2 Signaling
EGF Signaling
cAMP-mediated signaling
Hereditary Breast Cancer Signaling
Endometrial Cancer Signaling
PCP pathway
IL-2 Signaling
Citrulline Biosynthesis

**162 elements included exclusively in "Calu-3_SARS-CoV-2":**
Superpathway of Cholesterol Biosynthesis
Dendritic Cell Maturation
Antigen Presentation Pathway
Role of Pattern Recognition Receptors in Recognition of Bacteria and Viruses
Cholesterol Biosynthesis I
Cholesterol Biosynthesis II (via 24,25-dihydrolanosterol)
Cholesterol Biosynthesis III (via Desmosterol)
Interferon Signaling
PD-1, PD-L1 cancer immunotherapy pathway
TREM1 Signaling
T Cell Exhaustion Signaling Pathway
Type I Diabetes Mellitus Signaling
Th1 Pathway
Crosstalk between Dendritic Cells and Natural Killer Cells
Th1 and Th2 Activation Pathway
NF-κB Signaling
Altered T Cell and B Cell Signaling in Rheumatoid Arthritis
Role of RIG1-like Receptors in Antiviral Innate Immunity
Osteoarthritis Pathway
T Helper Cell Differentiation
VDR/RXR Activation
Th2 Pathway
Apoptosis Signaling
Communication between Innate and Adaptive Immune Cells
Role of IL-17A in Arthritis
Zymosterol Biosynthesis
Mevalonate Pathway I
Graft-versus-Host Disease Signaling
Superpathway of Geranylgeranyldiphosphate Biosynthesis I (via Mevalonate
IL-9 Signaling
IL-12 Signaling and Production in Macrophages
Role of Hypercytokinemia/hyperchemokinemia in the Pathogenesis of Influenza
Lymphotoxin β Receptor Signaling
Atherosclerosis Signaling
iCOS-iCOSL Signaling in T Helper Cells
OX40 Signaling Pathway
Complement System
PFKFB4 Signaling Pathway
Virus Entry via Endocytic Pathways
PEDF Signaling
Hepatic Cholestasis
Angiopoietin Signaling
Antioxidant Action of Vitamin C
Inflammasome pathway
Role of IL-17F in Allergic Inflammatory Airway Diseases
Role of NFAT in Regulation of the Immune Response
Huntington's Disease Signaling
MIF-mediated Glucocorticoid Regulation
Granulocyte Adhesion and Diapedesis
IL-17A Signaling in Airway Cells
JAK/Stat Signaling
Xenobiotic Metabolism Signaling
Cardiac Hypertrophy Signaling (Enhanced)
Differential Regulation of Cytokine Production in Macrophages and T Helper Cells by IL-17A and IL-17F
B Cell Development
Unfolded protein response
FAT10 Cancer Signaling Pathway
Tryptophan Degradation III (Eukaryotic)
Caveolar-mediated Endocytosis Signaling
MSP-RON Signaling Pathway
IL-17 Signaling
Endothelin-1 Signaling
Role of JAK1 and JAK3 in γc Cytokine Signaling
Role of Cytokines in Mediating Communication between Immune Cells
Neurotrophin/TRK Signaling
Autoimmune Thyroid Disease Signaling
IL-23 Signaling Pathway
STAT3 Pathway
Ceramide Signaling
Pathogenesis of Multiple Sclerosis
Coronavirus Replication Pathway
Phagosome Maturation
IL-4 Signaling
Pancreatic Adenocarcinoma Signaling
Allograft Rejection Signaling
Xenobiotic Metabolism PXR Signaling Pathway
FAT10 Signaling Pathway
Role of PI3K/AKT Signaling in the Pathogenesis of Influenza
Clathrin-mediated Endocytosis Signaling
Type II Diabetes Mellitus Signaling
Ketolysis
Macropinocytosis Signaling
Tight Junction Signaling
Differential Regulation of Cytokine Production in Intestinal Epithelial Cells by IL-17A and IL-17F
Growth Hormone Signaling
Neuropathic Pain Signaling In Dorsal Horn Neurons
Acute Myeloid Leukemia Signaling
Calcium-induced T Lymphocyte Apoptosis
Semaphorin Neuronal Repulsive Signaling Pathway
PPARα/RXRα Activation
Role of JAK1, JAK2 and TYK2 in Interferon Signaling
Adipogenesis pathway
3-phosphoinositide Biosynthesis
Melatonin Degradation II
IL-3 Signaling
Systemic Lupus Erythematosus In T Cell Signaling Pathway
Ketogenesis
Superpathway of Inositol Phosphate Compounds
Inhibition of ARE-Mediated mRNA Degradation Pathway
Protein Ubiquitination Pathway
fMLP Signaling in Neutrophils
Noradrenaline and Adrenaline Degradation
Tryptophan Degradation X (Mammalian, via Tryptamine)
IL-15 Signaling
Airway Pathology in Chronic Obstructive Pulmonary Disease
Glutaryl-CoA Degradation
Natural Killer Cell Signaling
Germ Cell-Sertoli Cell Junction Signaling
Corticotropin Releasing Hormone Signaling
Putrescine Degradation III
Endoplasmic Reticulum Stress Pathway
Hematopoiesis from Multipotent Stem Cells
Integrin Signaling
Eicosanoid Signaling
ERK5 Signaling
Sertoli Cell-Sertoli Cell Junction Signaling
Cell Cycle: G2/M DNA Damage Checkpoint Regulation
White Adipose Tissue Browning Pathway
BMP signaling pathway
FAK Signaling
Epoxysqualene Biosynthesis
β-alanine Degradation I
4-hydroxyproline Degradation I
Sulfate Activation for Sulfonation
Lysine Degradation II
Bile Acid Biosynthesis, Neutral Pathway
Salvage Pathways of Pyrimidine Ribonucleotides
Apelin Pancreas Signaling Pathway
Adrenomedullin signaling pathway
Role of MAPK Signaling in the Pathogenesis of Influenza
D-myo-inositol-5-phosphate Metabolism
Wnt/Ca+ pathway
Phosphatidylethanolamine Biosynthesis II
Cytotoxic T Lymphocyte-mediated Apoptosis of Target Cells
Role of JAK2 in Hormone-like Cytokine Signaling
ErbB Signaling
Role of NFAT in Cardiac Hypertrophy
Kinetochore Metaphase Signaling Pathway
GADD45 Signaling
Reelin Signaling in Neurons
Role of JAK family kinases in IL-6-type Cytokine Signaling
Tryptophan Degradation to 2-amino-3-carboxymuconate Semialdehyde
Pyridoxal 5'-phosphate Salvage Pathway
Maturity Onset Diabetes of Young (MODY) Signaling
Adenosine Nucleotides Degradation II
Gα12/13 Signaling
Aldosterone Signaling in Epithelial Cells
Gαq Signaling
Calcium Signaling
Axonal Guidance Signaling
FLT3 Signaling in Hematopoietic Progenitor Cells
Leptin Signaling in Obesity
Phospholipase C Signaling
Isoleucine Degradation I
Methylglyoxal Degradation I
4-aminobutyrate Degradation I
3-phosphoinositide Degradation
Superpathway of Serine and Glycine Biosynthesis I
Xenobiotic Metabolism General Signaling Pathway
Sphingosine-1-phosphate Signaling
Amyotrophic Lateral Sclerosis Signaling
VEGF Family Ligand-Receptor Interactions

**113 common elements in "Caco-2_SARS-CoV-2" and "Calu-3_SARS-CoV-2":**
TNFR2 Signaling
MSP-RON Signaling In Cancer Cells Pathway
Aryl Hydrocarbon Receptor Signaling
Acute Phase Response Signaling
Hepatic Fibrosis Signaling Pathway
TNFR1 Signaling
Glucocorticoid Receptor Signaling
ERK/MAPK Signaling
Sumoylation Pathway
CD40 Signaling
IL-17A Signaling in Fibroblasts
HGF Signaling
Molecular Mechanisms of Cancer
Coronavirus Pathogenesis Pathway
p53 Signaling
Erythropoietin Signaling
Tumor Microenvironment Pathway
Hepatic Fibrosis / Hepatic Stellate Cell Activation
Regulation Of The Epithelial Mesenchymal Transition By Growth Factors Pathway
ILK Signaling
Circadian Rhythm Signaling
Toll-like Receptor Signaling
Thyroid Cancer Signaling
PI3K Signaling in B Lymphocytes
PDGF Signaling
April Mediated Signaling
RANK Signaling in Osteoclasts
B Cell Activating Factor Signaling
Regulation of IL-2 Expression in Activated and Anergic T Lymphocytes
MIF Regulation of Innate Immunity
Death Receptor Signaling
Necroptosis Signaling Pathway
iNOS Signaling
ATM Signaling
HMGB1 Signaling
Chronic Myeloid Leukemia Signaling
Tec Kinase Signaling
Neuregulin Signaling
Signaling by Rho Family GTPases
T Cell Receptor Signaling
UVB-Induced MAPK Signaling
CD27 Signaling in Lymphocytes
PI3K/AKT Signaling
B Cell Receptor Signaling
NRF2-mediated Oxidative Stress Response
Role of PKR in Interferon Induction and Antiviral Response
Senescence Pathway
RAR Activation
LXR/RXR Activation
IL-15 Production
CD28 Signaling in T Helper Cells
IL-8 Signaling
Thrombopoietin Signaling
Polyamine Regulation in Colon Cancer
IL-10 Signaling
Small Cell Lung Cancer Signaling
Role of Macrophages, Fibroblasts and Endothelial Cells in Rheumatoid Arthritis
Estrogen-Dependent Breast Cancer Signaling
Hypoxia Signaling in the Cardiovascular System
Endocannabinoid Cancer Inhibition Pathway
IL-17A Signaling in Gastric Cells
NF-κB Activation by Viruses
IL-7 Signaling Pathway
BEX2 Signaling Pathway
Estrogen Receptor Signaling
Prolactin Signaling
PKCθ Signaling in T Lymphocytes
LPS-stimulated MAPK Signaling
TR/RXR Activation
HER-2 Signaling in Breast Cancer
Xenobiotic Metabolism AHR Signaling Pathway
Colorectal Cancer Metastasis Signaling
4-1BB Signaling in T Lymphocytes
Prostate Cancer Signaling
IL-1 Signaling
TWEAK Signaling
TGF-β Signaling
Cdc42 Signaling
UVA-Induced MAPK Signaling
Systemic Lupus Erythematosus In B Cell Signaling Pathway
IGF-1 Signaling
PPAR Signaling
Production of Nitric Oxide and Reactive Oxygen Species in Macrophages
Estrogen Biosynthesis
Agranulocyte Adhesion and Diapedesis
Sirtuin Signaling Pathway
Protein Kinase A Signaling
BAG2 Signaling Pathway
MSP-RON Signaling In Macrophages Pathway
Neuroinflammation Signaling Pathway
Role of Tissue Factor in Cancer
HIF1α Signaling
Renin-Angiotensin Signaling
p38 MAPK Signaling
Rac Signaling
Myc Mediated Apoptosis Signaling
FXR/RXR Activation
IL-6 Signaling
Role of Osteoblasts, Osteoclasts and Chondrocytes in Rheumatoid Arthritis
14-3-3-mediated Signaling
P2Y Purigenic Receptor Signaling Pathway
Sperm Motility
LPS/IL-1 Mediated Inhibition of RXR Function
Phagosome Formation
Androgen Signaling
PTEN Signaling
Retinoic acid Mediated Apoptosis Signaling
D-myo-inositol (1,4,5,6)-Tetrakisphosphate Biosynthesis
D-myo-inositol (3,4,5,6)-tetrakisphosphate Biosynthesis
Induction of Apoptosis by HIV1
Activation of IRF by Cytosolic Pattern Recognition Receptors
PXR/RXR Activation
Relaxin Signaling

**Supplementary Table S18. Unique and overlapping Canonical Pathways for the compassion of the conditions "Caco-2_SARS-CoV" and "Calu-3_SARS-CoV".** Comparative analysis was done using VENNY 2.1. Data visualization was done by R-based Venn diagrams shown in Figure 5.

**64 elements included exclusively in "Caco-2_SARS-CoV":**

- Germ Cell-Sertoli Cell Junction Signaling
- Coagulation System
- Xenobiotic Metabolism CAR Signaling Pathway
- Ephrin Receptor Signaling
- Regulation of Actin-based Motility by Rho
- Signaling by Rho Family GTPases
- CXCR4 Signaling
- Semaphorin Signaling in Neurons
- Axonal Guidance Signaling
- Renal Cell Carcinoma Signaling
- Ethanol Degradation I
- Actin Nucleation by ARP-WASP Complex
- Glioblastoma Multiforme Signaling
- RhoGDI Signaling
- Integrin Signaling
- Paxillin Signaling
- Phospholipase C Signaling
- Caveolar-mediated Endocytosis Signaling
- Acetate Conversion to Acetyl-CoA
- Bile Acid Biosynthesis, Neutral Pathway
- Semaphorin Neuronal Repulsive Signaling Pathway
- D-myo-inositol (1,4,5,6)-Tetrakisphosphate Biosynthesis
- D-myo-inositol (3,4,5,6)-tetrakisphosphate Biosynthesis
- Rac Signaling
- Serine Biosynthesis
- FAK Signaling
- Extrinsic Prothrombin Activation Pathway
- PAK Signaling
- Cell Cycle: G1/S Checkpoint Regulation
- 3-phosphoinositide Degradation
- Phagosome Formation
- D-myo-inositol-5-phosphate Metabolism
- Retinoate Biosynthesis I
- Superpathway of Serine and Glycine Biosynthesis
- GADD45 Signaling
- Valine Degradation I
- Oxidative Ethanol Degradation II
- Ephrin B Signaling
- Glioma Invasiveness Signaling
- Regulation of Cellular Mechanics by Calpain Protease
- Fatty Acid α-oxidation
- Cell Cycle Regulation by BTG Family Proteins
- Xenobiotic Metabolism PXR Signaling Pathway
- Leukocyte Extravasation Signaling
- Sertoli Cell-Sertoli Cell Junction Signaling
- G-Protein Coupled Receptor Signaling
- Inhibition of Matrix Metalloproteases
- Thyroid Cancer Signaling
- Ethanol Degradation IV
- Chronic Myeloid Leukemia Signaling
- Glutamine Biosynthesis I
- Methylglyoxal Degradation V
- Sulfite Oxidation IV
- HER-2 Signaling in Breast Cancer
- BAG2 Signaling Pathway
- HIPPO signaling
- Dermatan Sulfate Biosynthesis (Late Stages)
- Serotonin Degradation
- Nur77 Signaling in T Lymphocytes
- Ovarian Cancer Signaling
- Glucose and Glucose-1-phosphate Degradation
- Chondroitin Sulfate Biosynthesis (Late Stages)
- GM-CSF Signaling
- Fatty Acid Activation

**114 elements included exclusively in "Calu-3_SARS-CoV":**

- Activation of IRF by Cytosolic Pattern Recognition Receptors
- Dendritic Cell Maturation
- Interferon Signaling
- Role of RIG1-like Receptors in Antiviral Innate Immunity
- Type I Diabetes Mellitus Signaling
- Lymphotoxin β Receptor Signaling
- Necroptosis Signaling Pathway
- Antigen Presentation Pathway
- Th1 and Th2 Activation Pathway
- Protein Ubiquitination Pathway
- Th1 Pathway
- VDR/RXR Activation
- PD-1, PD-L1 cancer immunotherapy pathway
- TREM1 Signaling
- Toll-like Receptor Signaling
- p38 MAPK Signaling
- Crosstalk between Dendritic Cells and Natural Killer Cells
- IL-17A Signaling in Gastric Cells
- T Cell Exhaustion Signaling Pathway
- 4-1BB Signaling in T Lymphocytes
- Role of IL-17F in Allergic Inflammatory Airway Diseases
- Circadian Rhythm Signaling
- Communication between Innate and Adaptive Immune Cells
- Altered T Cell and B Cell Signaling in Rheumatoid Arthritis
- Differential Regulation of Cytokine Production in Macrophages and T Helper Cells by IL-17A and IL-17F
- T Helper Cell Differentiation
- Th2 Pathway
- IL-17A Signaling in Airway Cells
- CD27 Signaling in Lymphocytes
- Role of Hypercytokinemia/hyperchemokinemia in the Pathogenesis of Influenza
- Unfolded protein response
- JAK/Stat Signaling
- Prolactin Signaling
- SAPK/JNK Signaling
- Differential Regulation of Cytokine Production in Intestinal Epithelial Cells by IL-17A and IL-17F
- Natural Killer Cell Signaling
- Role of JAK1, JAK2 and TYK2 in Interferon Signaling
- PEDF Signaling
- Inflammasome pathway
- Role of JAK family kinases in IL-6-type Cytokine Signaling
- OX40 Signaling Pathway
- Endoplasmic Reticulum Stress Pathway
- Graft-versus-Host Disease Signaling
- PKCθ Signaling in T Lymphocytes
- Type II Diabetes Mellitus Signaling
- NGF Signaling
- Pathogenesis of Multiple Sclerosis
- Apelin Pancreas Signaling Pathway
- Role of NFAT in Regulation of the Immune Response
- FAT10 Signaling Pathway
- Huntington's Disease Signaling
- Role of Oct4 in Mammalian Embryonic Stem Cell Pluripotency
- Th17 Activation Pathway
- Salvage Pathways of Pyrimidine Ribonucleotides
- UVA-Induced MAPK Signaling
- Pyridoxal 5'-phosphate Salvage Pathwa
- CD28 Signaling in T Helper Cells
- IL-17 Signaling
- Role of Cytokines in Mediating Communication between Immune Cells
- Estrogen-Dependent Breast Cancer Signaling
- Hypoxia Signaling in the Cardiovascular System
- Ceramide Signaling
- Antioxidant Action of Vitamin C
- iCOS-iCOSL Signaling in T Helper Cells
- ATM Signaling
- FGF Signaling
- Hematopoiesis from Multipotent Stem Cells
- BEX2 Signaling Pathway
- Inhibition of ARE-Mediated mRNA Degradation Pathway
- Estrogen Receptor Signaling
- Cytotoxic T Lymphocyte-mediated Apoptosis of Target Cell
- Maturity Onset Diabetes of Young (MODY) Signaling
- Angiopoietin Signaling
- Role of JAK1 and JAK3 in γc Cytokine Signaling
- Glycine Degradation (Creatine Biosynthesis
- Role of PI3K/AKT Signaling in the Pathogenesis of Influenza
- Apelin Endothelial Signaling Pathway
- MSP-RON Signaling Pathway
- UVB-Induced MAPK Signaling
- Renin-Angiotensin Signaling
- TGF-β Signaling
- Aldosterone Signaling in Epithelial Cells
- IGF-1 Signaling
- Cardiac Hypertrophy Signaling
- PPARα/RXRα Activation
- Transcriptional Regulatory Network in Embryonic Stem Cells
- Role of JAK2 in Hormone-like Cytokine Signaling
- Clathrin-mediated Endocytosis Signaling
- Macropinocytosis Signaling
- Wnt/Ca+ pathway
- VEGF Family Ligand-Receptor Interactions
- Gα12/13 Signaling
- Thrombopoietin Signaling
- AMPK Signaling
- Sperm Motility
- PXR/RXR Activation
- Role of BRCA1 in DNA Damage Response
- Complement System
- Thyronamine and Iodothyronamine Metabolism
- Thyroid Hormone Metabolism I (via Deiodination
- 14-3-3-mediated Signaling
- Corticotropin Releasing Hormone Signaling
- Bupropion Degradation
- Role of IL-17A in Psoriasis
- IL-15 Signaling
- Granzyme A Signaling
- Salvage Pathways of Pyrimidine Deoxyribonucleotide
- GDNF Family Ligand-Receptor Interactions
- Apelin Liver Signaling Pathway
- IL-4 Signaling
- BMP signaling pathway
- EGF Signaling
- Estrogen Biosynthesis
- Human Embryonic Stem Cell Pluripotency

**113 common elements in "Caco-2_SARS-CoV" and "Calu-3_SARS-CoV":**

- Molecular Mechanisms of Cancer
- Tec Kinase Signaling
- ERK/MAPK Signaling
- Tumor Microenvironment Pathway
- Aryl Hydrocarbon Receptor Signaling
- Hepatic Fibrosis Signaling Pathway
- HMGB1 Signaling
- Role of Tissue Factor in Cancer
- PI3K/AKT Signaling
- TNFR1 Signaling
- Hepatic Fibrosis / Hepatic Stellate Cell Activation
- Regulation Of The Epithelial Mesenchymal Transition By Growth Factors Pathway
- p53 Signaling
- MSP-RON Signaling In Cancer Cells Pathway
- Erythropoietin Signaling
- TR/RXR Activation
- HGF Signaling
- Death Receptor Signaling
- Colorectal Cancer Metastasis Signaling
- Cardiac Hypertrophy Signaling (Enhanced
- CD40 Signaling
- Osteoarthritis Pathway
- IL-8 Signaling
- Role of IL-17A in Arthritis
- Senescence Pathway
- Small Cell Lung Cancer Signaling
- Atherosclerosis Signaling
- ILK Signaling
- Agranulocyte Adhesion and Diapedesis
- TNFR2 Signaling
- LPS/IL-1 Mediated Inhibition of RXR Function
- HIF1α Signaling
- Systemic Lupus Erythematosus In B Cell Signaling Pathway
- IL-15 Production
- Sumoylation Pathway
- Xenobiotic Metabolism AHR Signaling Pathway
- Myc Mediated Apoptosis Signaling
- TWEAK Signaling
- IL-17A Signaling in Fibroblasts
- Neuregulin Signaling
- Regulation of the Epithelial-Mesenchymal Transition Pathway
- Role of Osteoblasts, Osteoclasts and Chondrocytes in Rheumatoid Arthritis
- Acute Myeloid Leukemia Signaling
- ERK5 Signaling
- Protein Kinase A Signaling
- Adipogenesis pathway
- STAT3 Pathway
- Gαq Signaling
- HOTAIR Regulatory Pathway
- Glucocorticoid Receptor Signaling
- Role of PKR in Interferon Induction and Antiviral Response
- MIF Regulation of Innate Immunity
- Cholecystokinin/Gastrin-mediated Signaling
- Retinoic acid Mediated Apoptosis Signaling
- Endothelin-1 Signaling
- LXR/RXR Activation
- NRF2-mediated Oxidative Stress Response
- 3-phosphoinositide Biosynthesis
- Xenobiotic Metabolism Signaling
- Role of Macrophages, Fibroblasts and Endothelial Cells in Rheumatoid Arthritis
- RAR Activation
- FXR/RXR Activation
- IL-6 Signaling
- Granulocyte Adhesion and Diapedesis
- Ephrin A Signaling
- Neuroinflammation Signaling Pathway
- Pancreatic Adenocarcinoma Signaling
- RANK Signaling in Osteoclasts
- IL-12 Signaling and Production in Macrophages
- Acute Phase Response Signaling
- IL-9 Signaling
- Regulation of IL-2 Expression in Activated and Anergic T Lymphocytes
- IL-10 Signaling
- PTEN Signaling
- MIF-mediated Glucocorticoid Regulation
- ErbB Signaling
- Sphingosine-1-phosphate Signaling
- Production of Nitric Oxide and Reactive Oxygen Species in Macrophage
- Amyotrophic Lateral Sclerosis Signaling
- Endocannabinoid Cancer Inhibition Pathway
- NF-κB Activation by Viruses
- Neurotrophin/TRK Signaling
- IL-7 Signaling Pathway
- Apoptosis Signaling
- Superpathway of Inositol Phosphate Compounds
- April Mediated Signaling
- Relaxin Signaling
- Coronavirus Pathogenesis Pathway
- P2Y Purigenic Receptor Signaling Pathway
- B Cell Activating Factor Signaling
- Induction of Apoptosis by HIV1
- PPAR Signaling
- LPS-stimulated MAPK Signaling
- T Cell Receptor Signaling
- Role of Pattern Recognition Receptors in Recognition of Bacteria and Viruses
- NF-κB Signaling
- PDGF Signaling
- IL-23 Signaling Pathway
- Glioma Signaling
- Sirtuin Signaling Pathway
- iNOS Signaling
- Hepatic Cholestasis
- B Cell Receptor Signaling
- FAT10 Cancer Signaling Pathway
- PFKFB4 Signaling Pathway
- MSP-RON Signaling In Macrophages Pathway
- PI3K Signaling in B Lymphocytes
- Prostate Cancer Signaling
- IL-1 Signaling
- Superpathway of Cholesterol Biosynthesis
- Hematopoiesis from Pluripotent Stem Cells
- Airway Pathology in Chronic Obstructive Pulmonary Disease
- Growth Hormone Signaling

**Supplementary Table S19. Unique and overlapping Canonical Pathways for the compassion of the conditions "Caco-2_SARS-CoV-2" and "Caco-2_SARS-CoV".** Comparative analysis was done using VENNY 2.1. Data visualization was done by R-based Venn diagrams shown in Figure 5.

**52 elements included exclusively in "Caco-2_SARS-CoV-2":**

Circadian Rhythm Signaling
Toll-like Receptor Signaling
Necroptosis Signaling Pathway
ATM Signaling
UVB-Induced MAPK Signaling
CDK5 Signaling
CD27 Signaling in Lymphocytes
CD28 Signaling in T Helper Cells
Thrombopoietin Signaling
ErbB2-ErbB3 Signaling
Polyamine Regulation in Colon Cance
Estrogen-Dependent Breast Cancer Signaling
Hypoxia Signaling in the Cardiovascular Syster
IL-17A Signaling in Gastric Cells
Estrogen-mediated S-phase Entr
BEX2 Signaling Pathway
Estrogen Receptor Signaling
Prolactin Signaling
PKCθ Signaling in T Lymphocytes
4-1BB Signaling in T Lymphocytes
Wnt/β-catenin Signaling
TGF-β Signaling
Cdc42 Signaling
Bladder Cancer Signaling
UVA-Induced MAPK Signaling
IGF-1 Signaling
Telomerase Signaling
Estrogen Biosynthesis
Arginine Degradation I (Arginase Pathway
Apelin Endothelial Signaling Pathwa
Proline Biosynthesis II (from Arginine
Renin-Angiotensin Signaling
p38 MAPK Signaling
Melanoma Signaling
UVC-Induced MAPK Signaling
Arginine Biosynthesis IV
Arginine Degradation VI (Arginase 2 Pathway
Selenocysteine Biosynthesis II (Archaea and Eukaryotes
GDP-mannose Biosynthesis
14-3-3-mediated Signaling
Sperm Motility
EIF2 Signaling
EGF Signaling
cAMP-mediated signaling
Androgen Signaling
Hereditary Breast Cancer Signaling
Endometrial Cancer Signaling
PCP pathway
IL-2 Signaling
Activation of IRF by Cytosolic Pattern Recognition Receptor
Citrulline Biosynthesis
PXR/RXR Activation

**82 elements included exclusively in "Caco-2_SARS-CoV":**

Germ Cell-Sertoli Cell Junction Signaling
Xenobiotic Metabolism CAR Signaling Pathwa
Ephrin Receptor Signaling
Cardiac Hypertrophy Signaling (Enhanced
Osteoarthritis Pathway
Role of IL-17A in Arthritis
Atherosclerosis Signaling
Semaphorin Signaling in Neurons
Axonal Guidance Signaling
Ethanol Degradation I
RhoGDI Signaling
Integrin Signaling
Regulation of the Epithelial-Mesenchymal Transition Pathwa
Paxillin Signaling
Acute Myeloid Leukemia Signaling
Phospholipase C Signaling
ERK5 Signaling
Caveolar-mediated Endocytosis Signalin
Adipogenesis pathway
Acetate Conversion to Acetyl-CoA
STAT3 Pathway
Gαq Signaling
Bile Acid Biosynthesis, Neutral Pathway
Semaphorin Neuronal Repulsive Signaling Pathwa
Endothelin-1 Signaling
3-phosphoinositide Biosynthesis
Xenobiotic Metabolism Signaling
Serine Biosynthesis
FAK Signaling
Granulocyte Adhesion and Diapedesis
Extrinsic Prothrombin Activation Pathway
PAK Signaling
Ephrin A Signaling
Pancreatic Adenocarcinoma Signaling
IL-12 Signaling and Production in Macrophage
3-phosphoinositide Degradation
IL-9 Signaling
D-myo-inositol-5-phosphate Metabolism
MIF-mediated Glucocorticoid Regulatio
Superpathway of Serine and Glycine Biosynthesis
GADD45 Signaling
Valine Degradation
Oxidative Ethanol Degradation I
Ephrin B Signaling
Glioma Invasiveness Signaling
ErbB Signaling
Sphingosine-1-phosphate Signaling
Fatty Acid α-oxidation
Amyotrophic Lateral Sclerosis Signaling
Cell Cycle Regulation by BTG Family Proteins
Neurotrophin/TRK Signaling
Xenobiotic Metabolism PXR Signaling Pathwa
Leukocyte Extravasation Signaling
Sertoli Cell-Sertoli Cell Junction Signaling
G-Protein Coupled Receptor Signaling
Apoptosis Signaling
Inhibition of Matrix Metalloprotease
Superpathway of Inositol Phosphate Compound
Ethanol Degradation IV
Glutamine Biosynthesis
Methylglyoxal Degradation V
Sulfite Oxidation IV
Role of Pattern Recognition Receptors in Recognition of Bacteria and Viruse
NF-κB Signaling
HIPPO signaling
IL-23 Signaling Pathway
Glioma Signaling
Hepatic Cholestasis
Dermatan Sulfate Biosynthesis (Late Stages
FAT10 Cancer Signaling Pathway
PFKFB4 Signaling Pathway
Serotonin Degradation
Nur77 Signaling in T Lymphocytes
Ovarian Cancer Signaling
Glucose and Glucose-1-phosphate Degradatio
Chondroitin Sulfate Biosynthesis (Late Stages
GM-CSF Signaling
Superpathway of Cholesterol Biosynthesi
Hematopoiesis from Pluripotent Stem Cell
Airway Pathology in Chronic Obstructive Pulmonary Diseas
Growth Hormone Signaling
Fatty Acid Activation

**95 common elements in "Caco-2_SARS-CoV-2" and "Caco-2_SARS-CoV":**

TNFR2 Signaling
MSP-RON Signaling In Cancer Cells Pathway
Aryl Hydrocarbon Receptor Signaling
Acute Phase Response Signaling
Hepatic Fibrosis Signaling Pathway
TNFR1 Signaling
Glucocorticoid Receptor Signaling
ERK/MAPK Signaling
Sumoylation Pathway
CD40 Signaling
IL-17A Signaling in Fibroblasts
HGF Signaling
Molecular Mechanisms of Cancer
Coronavirus Pathogenesis Pathway
p53 Signaling
HOTAIR Regulatory Pathway
Erythropoietin Signaling
Tumor Microenvironment Pathway
Cell Cycle: G1/S Checkpoint Regulation
Hepatic Fibrosis / Hepatic Stellate Cell Activatio
Regulation Of The Epithelial Mesenchymal Transition By Growth Factors Pathwa
ILK Signaling
Coagulation System
Thyroid Cancer Signaling
PI3K Signaling in B Lymphocytes
PDGF Signaling
April Mediated Signaling
RANK Signaling in Osteoclasts
B Cell Activating Factor Signaling
Regulation of IL-2 Expression in Activated and Anergic T Lymphocyte
MIF Regulation of Innate Immunity
Death Receptor Signaling
iNOS Signaling
HMGB1 Signaling
Chronic Myeloid Leukemia Signaling
Tec Kinase Signaling
Neuregulin Signaling
Signaling by Rho Family GTPases
T Cell Receptor Signaling
PI3K/AKT Signaling
B Cell Receptor Signaling
NRF2-mediated Oxidative Stress Response
Role of PKR in Interferon Induction and Antiviral Respons
Senescence Pathway
RAR Activation
LXR/RXR Activation
IL-15 Production
IL-8 Signaling
IL-10 Signaling
Small Cell Lung Cancer Signaling
Role of Macrophages, Fibroblasts and Endothelial Cells in Rheumatoid Arthri
Regulation of Cellular Mechanics by Calpain Proteas
Endocannabinoid Cancer Inhibition Pathway
NF-κB Activation by Viruses
IL-7 Signaling Pathway
Renal Cell Carcinoma Signaling
Actin Nucleation by ARP-WASP Complex
LPS-stimulated MAPK Signaling
TR/RXR Activation
HER-2 Signaling in Breast Cancer
Xenobiotic Metabolism AHR Signaling Pathwa
Colorectal Cancer Metastasis Signaling
Glioblastoma Multiforme Signaling
CXCR4 Signaling
Prostate Cancer Signaling
IL-1 Signaling
Retinoate Biosynthesis
TWEAK Signaling
Systemic Lupus Erythematosus In B Cell Signaling Pathwa
Regulation of Actin-based Motility by Rho
PPAR Signaling
Production of Nitric Oxide and Reactive Oxygen Species in Macrophage
Agranulocyte Adhesion and Diapedesis
Sirtuin Signaling Pathway
Protein Kinase A Signaling
BAG2 Signaling Pathway
MSP-RON Signaling In Macrophages Pathway
Neuroinflammation Signaling Pathway
Role of Tissue Factor in Cancer
HIF1α Signaling
Cholecystokinin/Gastrin-mediated Signalin
Rac Signaling
Myc Mediated Apoptosis Signaling
FXR/RXR Activation
IL-6 Signaling
Role of Osteoblasts, Osteoclasts and Chondrocytes in Rheumatoid Arthrit
P2Y Purigenic Receptor Signaling Pathwa
LPS/IL-1 Mediated Inhibition of RXR Function
Phagosome Formation
PTEN Signaling
Retinoic acid Mediated Apoptosis Signalin
D-myo-inositol (1,4,5,6)-Tetrakisphosphate Biosynthesi
D-myo-inositol (3,4,5,6)-tetrakisphosphate Biosynthesi
Induction of Apoptosis by HIV1
Relaxin Signaling

**Supplementary Table S20. Unique and overlapping Canonical Pathways for the compassion of the conditions "Calu-3_SARS-CoV-2" and "Calu-3_SARS-CoV".** Comparative analysis was done using VENNY 2.1. Data visualization was done by R-based Venn diagrams shown in Figure 5.

**75 elements included exclusively in "Calu-3_SARS-CoV-2"**

Cholesterol Biosynthesis
Cholesterol Biosynthesis II (via 24,25-dihydrolanostero
Cholesterol Biosynthesis III (via Desmosterol
Zymosterol Biosynthesis
Mevalonate Pathway
Superpathway of Geranylgeranyldiphosphate Biosynthesis I (via Mevalonat
Virus Entry via Endocytic Pathways
B Cell Development
Tryptophan Degradation III (Eukaryotic
Caveolar-mediated Endocytosis Signaling
Autoimmune Thyroid Disease Signaling
Coronavirus Replication Pathway
Phagosome Maturation
Allograft Rejection Signaling
Xenobiotic Metabolism PXR Signaling Pathway
Ketolysis
Tight Junction Signaling
BAG2 Signaling Pathway
Neuropathic Pain Signaling In Dorsal Horn Neurons
Calcium-induced T Lymphocyte Apoptosis
Semaphorin Neuronal Repulsive Signaling Pathway
Melatonin Degradation I
IL-3 Signaling
Systemic Lupus Erythematosus In T Cell Signaling Pathway
Ketogenesis
fMLP Signaling in Neutrophils
Noradrenaline and Adrenaline Degradatio
Tryptophan Degradation X (Mammalian, via Tryptamine
Glutaryl-CoA Degradation
Germ Cell-Sertoli Cell Junction Signaling
Putrescine Degradation III
Signaling by Rho Family GTPases
Integrin Signaling
Eicosanoid Signaling
Sertoli Cell-Sertoli Cell Junction Signaling
Cell Cycle: G2/M DNA Damage Checkpoint Regulation
White Adipose Tissue Browning Pathway
Thyroid Cancer Signaling
FAK Signaling
Epoxysqualene Biosynthesis
β-alanine Degradation
4-hydroxyproline Degradation
Sulfate Activation for Sulfonation
Lysine Degradation I
Bile Acid Biosynthesis, Neutral Pathway
Adrenomedullin signaling pathway
Role of MAPK Signaling in the Pathogenesis of Influenza
D-myo-inositol-5-phosphate Metabolism
Phosphatidylethanolamine Biosynthesis I
Phagosome Formation
Role of NFAT in Cardiac Hypertrophy
Kinetochore Metaphase Signaling Pathway
GADD45 Signaling
Reelin Signaling in Neurons
D-myo-inositol (1,4,5,6)-Tetrakisphosphate Biosynthesi
D-myo-inositol (3,4,5,6)-tetrakisphosphate Biosynthesi
Tryptophan Degradation to 2-amino-3-carboxymuconate Semialdehyd
Adenosine Nucleotides Degradation I
Calcium Signaling
Axonal Guidance Signaling
FLT3 Signaling in Hematopoietic Progenitor Cells
Leptin Signaling in Obesity
Phospholipase C Signaling
Isoleucine Degradation
Rac Signaling
Cdc42 Signaling
Methylglyoxal Degradation
4-aminobutyrate Degradation
3-phosphoinositide Degradation
Androgen Signaling
Superpathway of Serine and Glycine Biosynthesis
Xenobiotic Metabolism General Signaling Pathway
Polyamine Regulation in Colon Cance
Chronic Myeloid Leukemia Signaling
HER-2 Signaling in Breast Cancer

**27 elements included exclusively in "Calu-3_SARS-CoV"**

SAPK/JNK Signaling
HOTAIR Regulatory Pathway
NGF Signaling
Role of Oct4 in Mammalian Embryonic Stem Cell Pluripotency
Th17 Activation Pathway
FGF Signaling
Glycine Degradation (Creatine Biosynthesis
Apelin Endothelial Signaling Pathway
Cholecystokinin/Gastrin-mediated Signaling
Cardiac Hypertrophy Signaling
Transcriptional Regulatory Network in Embryonic Stem Cells
Regulation of the Epithelial-Mesenchymal Transition Pathwa
Hematopoiesis from Pluripotent Stem Cells
AMPK Signaling
Glioma Signaling
Role of BRCA1 in DNA Damage Response
Thyronamine and Iodothyronamine Metabolism
Thyroid Hormone Metabolism I (via Deiodination
Bupropion Degradation
Role of IL-17A in Psoriasis
Granzyme A Signaling
Salvage Pathways of Pyrimidine Deoxyribonucleotide
GDNF Family Ligand-Receptor Interactions
Apelin Liver Signaling Pathway
Ephrin A Signaling
EGF Signaling
Human Embryonic Stem Cell Pluripotency

**200 common elements in "Calu-3_SARS-CoV-2" and "Calu-3_SARS-CoV"**

Superpathway of Cholesterol Biosynthesi
Dendritic Cell Maturation
Neuroinflammation Signaling Pathway
Antigen Presentation Pathway
Role of Pattern Recognition Receptors in Recognition of Bacteria and Viruse
Activation of IRF by Cytosolic Pattern Recognition Receptors
MSP-RON Signaling In Macrophages Pathway
Interferon Signaling
Role of Macrophages, Fibroblasts and Endothelial Cells in Rheumatoid Arthriti
Death Receptor Signaling
PD-1, PD-L1 cancer immunotherapy pathway
Tumor Microenvironment Pathway
TREM1 Signaling
Systemic Lupus Erythematosus In B Cell Signaling Pathway
Acute Phase Response Signaling
T Cell Exhaustion Signaling Pathway
Type I Diabetes Mellitus Signaling
Coronavirus Pathogenesis Pathway
Th1 Pathway
Crosstalk between Dendritic Cells and Natural Killer Cell
Hepatic Fibrosis Signaling Pathway
Th1 and Th2 Activation Pathway
NF-κB Signaling
Altered T Cell and B Cell Signaling in Rheumatoid Arthritis
Role of RIG1-like Receptors in Antiviral Innate Immunity
Aryl Hydrocarbon Receptor Signaling
Myc Mediated Apoptosis Signaling
Senescence Pathway
Necroptosis Signaling Pathway
Hepatic Fibrosis / Hepatic Stellate Cell Activation
Role of PKR in Interferon Induction and Antiviral Response
Osteoarthritis Pathway
TNFR2 Signaling
T Helper Cell Differentiation
TWEAK Signaling
IL-17A Signaling in Fibroblasts
VDR/RXR Activation
Th2 Pathway
Apoptosis Signaling
Toll-like Receptor Signaling
Communication between Innate and Adaptive Immune Cells
Role of IL-17A in Arthritis
CD40 Signaling
IL-6 Signaling
Induction of Apoptosis by HIV1
Graft-versus-Host Disease Signaling
Molecular Mechanisms of Cance
IL-9 Signaling
HMGB1 Signaling
Regulation Of The Epithelial Mesenchymal Transition By Growth Factors Pathwa
LXR/RXR Activation
PPAR Signaling
PKCθ Signaling in T Lymphocytes
IL-12 Signaling and Production in Macrophages
Tec Kinase Signaling
Role of Hypercytokinemia/hyperchemokinemia in the Pathogenesis of Influenza
Lymphotoxin β Receptor Signaling
Role of Osteoblasts, Osteoclasts and Chondrocytes in Rheumatoid Arthriti
Production of Nitric Oxide and Reactive Oxygen Species in Macrophage
iNOS Signaling
Atherosclerosis Signaling
iCOS-iCOSL Signaling in T Helper Cells
TNFR1 Signaling
OX40 Signaling Pathway
Complement System
PFKFB4 Signaling Pathway
IL-17A Signaling in Gastric Cells
Prolactin Signaling
RAR Activation
Glucocorticoid Receptor Signaling
PEDF Signaling
Endocannabinoid Cancer Inhibition Pathway
Erythropoietin Signaling
Hepatic Cholestasis
Xenobiotic Metabolism AHR Signaling Pathway
p38 MAPK Signaling
NRF2-mediated Oxidative Stress Response
Angiopoietin Signaling
Protein Kinase A Signaling
IL-10 Signaling
ILK Signaling
4-1BB Signaling in T Lymphocytes
Antioxidant Action of Vitamin C
IL-15 Production
CD28 Signaling in T Helper Cells
Inflammasome pathway
Role of IL-17F in Allergic Inflammatory Airway Diseases
Role of NFAT in Regulation of the Immune Response
Huntington's Disease Signaling
Estrogen-Dependent Breast Cancer Signaling
MIF-mediated Glucocorticoid Regulation
Granulocyte Adhesion and Diapedesis
IL-17A Signaling in Airway Cells
JAK/Stat Signaling
Xenobiotic Metabolism Signaling
PI3K Signaling in B Lymphocytes
Cardiac Hypertrophy Signaling (Enhanced
p53 Signaling
Retinoic acid Mediated Apoptosis Signaling
IL-8 Signaling
NF-κB Activation by Viruses
Differential Regulation of Cytokine Production in Macrophages and T Helper Cells by IL-17A and IL-17
Small Cell Lung Cancer Signaling
MSP-RON Signaling In Cancer Cells Pathway
Unfolded protein response
B Cell Activating Factor Signaling
FAT10 Cancer Signaling Pathway
RANK Signaling in Osteoclasts
IL-7 Signaling Pathway
PI3K/AKT Signaling
MIF Regulation of Innate Immunity
MSP-RON Signaling Pathway
IL-17 Signaling
Endothelin-1 Signaling
Role of JAK1 and JAK3 in γc Cytokine Signaling
IL-1 Signaling
Role of Cytokines in Mediating Communication between Immune Cell
Neurotrophin/TRK Signaling
IL-23 Signaling Pathway
STAT3 Pathway
Ceramide Signaling
Agranulocyte Adhesion and Diapedesis
B Cell Receptor Signaling
Pathogenesis of Multiple Sclerosis
April Mediated Signaling
BEX2 Signaling Pathway
IL-4 Signaling
Estrogen Biosynthesis
P2Y Purigenic Receptor Signaling Pathway
Pancreatic Adenocarcinoma Signaling
Thrombopoietin Signaling
Role of Tissue Factor in Cancer
FAT10 Signaling Pathway
Role of PI3K/AKT Signaling in the Pathogenesis of Influenza
Clathrin-mediated Endocytosis Signaling
Type II Diabetes Mellitus Signaling
Colorectal Cancer Metastasis Signaling
Macropinocytosis Signaling
Renin-Angiotensin Signaling
Differential Regulation of Cytokine Production in Intestinal Epithelial Cells by IL-17A and IL-17
Relaxin Signaling
Growth Hormone Signaling
ERK/MAPK Signaling
LPS-stimulated MAPK Signaling
Acute Myeloid Leukemia Signaling
Sperm Motility
TR/RXR Activation
Sirtuin Signaling Pathway

PPARα/RXRα Activation
Role of JAK1, JAK2 and TYK2 in Interferon Signaling
Adipogenesis pathway
3-phosphoinositide Biosynthesis
Sumoylation Pathway
Prostate Cancer Signaling
Superpathway of Inositol Phosphate Compounds
Inhibition of ARE-Mediated mRNA Degradation Pathway
Protein Ubiquitination Pathway
Hypoxia Signaling in the Cardiovascular System
Neuregulin Signaling
IL-15 Signaling
Airway Pathology in Chronic Obstructive Pulmonary Disease
Natural Killer Cell Signaling
Corticotropin Releasing Hormone Signaling
Endoplasmic Reticulum Stress Pathway
Hematopoiesis from Multipotent Stem Cells
FXR/RXR Activation
CD27 Signaling in Lymphocytes
LPS/IL-1 Mediated Inhibition of RXR Function
HIF1α Signaling
TGF-β Signaling
Regulation of IL-2 Expression in Activated and Anergic T Lymphocyte
ERK5 Signaling
ATM Signaling
BMP signaling pathway
IGF-1 Signaling
Salvage Pathways of Pyrimidine Ribonucleotides
UVA-Induced MAPK Signaling
Apelin Pancreas Signaling Pathway
Circadian Rhythm Signaling
PDGF Signaling
Estrogen Receptor Signaling
Wnt/Ca+ pathway
Cytotoxic T Lymphocyte-mediated Apoptosis of Target Cell
Role of JAK2 in Hormone-like Cytokine Signaling
ErbB Signaling
HGF Signaling
14-3-3-mediated Signaling
PXR/RXR Activation
PTEN Signaling
Role of JAK family kinases in IL-6-type Cytokine Signaling
Pyridoxal 5'-phosphate Salvage Pathway
Maturity Onset Diabetes of Young (MODY) Signaling
Gα12/13 Signaling
Aldosterone Signaling in Epithelial Cells
Gαq Signaling
T Cell Receptor Signaling
Sphingosine-1-phosphate Signaling
Amyotrophic Lateral Sclerosis Signaling
UVB-Induced MAPK Signaling
VEGF Family Ligand-Receptor Interactions

**Supplementary Table S21. Unique and overlapping Upstream Regulators for the compassion of the conditions "Caco-2_SARS-CoV-2" and "Calu-3_SARS-CoV-2".** Comparative analysis was done using VENNY 2.1. Data visualization was done by R-based Venn diagrams shown in Figure 5.

**1319 elements included exclusively in "Caco-2_SARS-CoV-2":**
NGFR
ETS2
GPC3
CIC
ACP1
GFAP
ZMIZ2
N(6)-(3-iodobenzyl)-5'-N-methylcarboxamidoadenosine
CSF1R
phenacetin
11,12-epoxyeicosatrienoic acid
AXIN1
ETS
MAPK8IP1
L-buthionine (SR)-sulfoximine
omeprazole
apigenin
SNW1
AZGP1
YBX3
miR-6873-3p (miRNAs w/seed UCUCUCU)
OGT
HDAC2
PHB
FUS
FGFR4
NF1
OIP5-AS1
CHRM3
ELK3
IL6ST
lonaprisan
RPRD1B
CCDC88C
CTNNβ-LEF1
inostamycin
(S)-flurbiprofen
SOX17
CDKN1B
dihydroartemisinin
isoprenaline
2-acetylaminofluorene
dehydroisoandrosterone
NIBAN2
KLHL2
ADI PEG20
SRGAP1
ASS1
AHNAK
HMGN2
CYP2C9
ATAD2
(E)-1-(2-nitrovinyl)naphthalene
(E)-9-(2-nitrovinyl)anthracene
PPP1R1B
THY1
ethidium
MTA1
CTBP2
KCNIP3
SCP2
metyrapone
2-bromoethylamine
STAP2
TEX11
DACT3
proanthocyanidin derivative
RAB11FIP3
CDC25B
PHLDA1
SNRK
SLC9A3R2
carboxyamido-triazole
C25-140
yohimbine
FGF21
Ccl2
enalapril
WDR5
IL2RA
PAX3-FOXO1
RNF187
Gpcr
FZD7
MCF2
rigosertib
RBM14
PSAP
NME3
SH2B2
CLDN2
dimaprit
IGFBP5
RACK1
TGFB1I1
LCAT
chelerythrine
KLF5
XBP1
miR-337-5p (miRNAs w/seed AACGGCU)
MTA2
CD9
ciprofloxacin
NTF3
gentamicin C
Tcf 1/3/4
ZMIZ1
Crnde
BPIFB1
PLSCR1
SLC7A11-AS1
EGFL7
TRIM33
STX2
RASA1
CIMO
saquinavir
PRKAR2B
NR5A1
ROCK1
SUB1
TCF7L1
miR-6883-3p (miRNAs w/seed UCCCUAU)
C1orf61
ATE1
RAP1GAP
CBR3-AS1
PDE3A
temsirolimus
volinanserin
CDK4/6
H2AZ1
ALDH1A2
PRKG1
PRLR
LCK
taurine
PTTG1
ARHGEF1
EFEMP1
ARAF
WNT7B
PROKR1
PBX2
MM-401
buserelin
artemisinin
cordycepin
N,N-dimethylarginine
Calcineurin A
KIN001-043
indole-3-carbinol
4-nonylphenol
Bcl9-Cbp/p300-Ctnnb1-Lef/Tcf
CTDSP1
bicyclol
MPL
BRMS1
USP17L2 (includes others)
CITCO
acrylamide
cyclopentenone
CCAR2
ALOX5
LHCGR
phenobarbital
ethionine
L-threonine
NAP1L1
CAMK2N2
Shc
BCO2
Sox2ot

**2963 elements included exclusively in "Calu-3_SARS-CoV-2":**
IRF7
IFNL1
NKX2-3
Ifnar
RC3H1
IRF2
TRIM24
DDX58
IRF5
IFNAR2
stallimycin
RNASEH2A
MAVS
STAT2
RNY3
PNPT1
E. coli serotype 0127B8 lipopolysaccharide
IRF9
SASH1
CNOT7
DOCK8
PARP9
SREBF2
NFAT5
IFN type 1
ziritaxestat
JAK
ACKR2
IL10RA
SCAP
IFNE
IFN alpha/beta
mir-21
l-asparaginase
lysophosphatidylinositol
miR-3083-5p (and other miRNAs w/seed GGCUGGG)
chloroquine
IFNL4
CGAS
let-7a-5p (and other miRNAs w/seed GAGGUAG)
mir-96
ribavirin
emactuzumab
PROM1
(-)-norephedrine
interferon beta-1a
CpG ODN 1668
USP18
SATB1
laminaran
CERK
SUMO3
ATP-gamma-S
SMPD1
Irgm1
edaravone
miR-24-3p (and other miRNAs w/seed GGCUCAG)
MTOR
miR-124-3p (and other miRNAs w/seed AAGGCAC)
KDM5B
IFNA4
POR
ezetimibe
mezerein
CAMP
miR-5624-3p (and other miRNAs w/seed UAAGGCA)
miR-762 (and other miRNAs w/seed GGGCUGG)
Igm
isoquercitrin
IFNK
pitavastatin
CD38
CLDN7
IL21
SAMHD1
EGLN1
IL7
miR-1283 (miRNAs w/seed CUACAAA)
NLRC5
2-aminopurine
poly dA-dT
ALB
miR-6756-5p (and other miRNAs w/seed GGGUGGG)
miR-149-3p (and other miRNAs w/seed GGGAGGG)
DNASE2
EGLN
Nr1h
BAX
miR-6079 (miRNAs w/seed UGGAAGC)
CCL3
MFSD2A
SUMO2
E. coli lipopolysaccharide
JAG2
zymosan
isotretinoin
NS-398
IKZF3
IFNL3
C3AR1
PTGER2
miR-1284 (and other miRNAs w/seed CUAUACA)
miR-6828-5p (miRNAs w/seed GGAAGCA)
mitoxantrone
miR-515-3p (and other miRNAs w/seed AGUGCCU)
miR-6795-5p (and other miRNAs w/seed GGGGGGA)
miR-4731-5p (miRNAs w/seed GCUGGGG)
paraquat
TAP1
KIT
12-(3-adamantan-1-yl-ureido) dodecanoic acid
HSPB1
miR-337-3p (miRNAs w/seed UCCUAUA)
TYROBP
EREG
miR-661 (miRNAs w/seed GCCUGGG)
Fibrinogen
TREX1
miR-548v (miRNAs w/seed GCUACAG)
mir-155
let-7a-3p (and other miRNAs w/seed UAUACAA)
miR-29b-3p (and other miRNAs w/seed AGCACCA)
CYP51A1
PEX5L
SHARPIN
ZBTB16
CREBZF
BMP6
miR-182-5p (and other miRNAs w/seed UUGGCAA)
miR-1289 (miRNAs w/seed GGAGUCC)
miR-6509-3p (miRNAs w/seed UCCACUG)
miR-4727-3p (miRNAs w/seed UAGUGGG)
SS18
miR-208a-3p (and other miRNAs w/seed UAAGACG)
UM101
miR-125b-5p (and other miRNAs w/seed CCCUGAG)
PIK3CG
cepharanthine
ZBED2
SIRT2
miR-4656 (and other miRNAs w/seed GGGCUGA)
IFNA10
IFNA21
IFNA5
IFNA7
IFNA14
IFNA6
2'3'-cyclic guanosine monophosphate-adenosine monophosphate
miR-137-3p (miRNAs w/seed UAUUGCU)
PLX5622
3M-001
salicylic acid
KLF3
advanced glycation end-products
3-deoxy-2-octulosonic acid(2)-lipid A
SIRT6
rifampin
lipoteichoic acid
miR-199a-5p (and other miRNAs w/seed CCAGUGU)
miR-4747-5p (and other miRNAs w/seed GGGAAGG)
miR-128-3p (and other miRNAs w/seed CACAGUG)
miR-365-3p (and other miRNAs w/seed AAUGCCC)
miR-4514 (and other miRNAs w/seed CAGGCAG)
PPARD
miR-6829-5p (miRNAs w/seed GGGCUGC)
CARD9
miR-302c-3p (and other miRNAs w/seed AGUGCUU)
miR-221-5p (and other miRNAs w/seed CCUGGCA)
ABT-737
SOCS6
PPP1R15A
let-7c-1-3p (and other miRNAs w/seed UGUACAA)
IFNA8

**2320 common elements in "Caco-2_SARS-CoV-2" and "Calu-3_SARS-CoV-2":**
PDGF BB
TNF
PD98059
U0126
beta-estradiol
ERK
IRAK4
HGF
dexamethasone
LY294002
EGF
NR3C1
NFkB (complex)
IL1B
RELA
GPER1
triamcinolone acetonide
Mek
F2
forskolin
4-hydroxytamoxifen
IGF1
E. coli B5 lipopolysaccharide
paclitaxel
trovafloxacin
HRAS
TP53
GnRH-A
IL1A
cycloheximide
RAF1
lysophosphatidic acid
JUN
SP600125
NRG1
CREB1
IL2
actinomycin D
tetradecanoylphorbol acetate
AGT
TP63
Raf
lipopolysaccharide
TGFB1
OSM
poly rI:rC-RNA
hydrogen peroxide
FOS
thapsigargin
mifepristone
IL6
IGF2
ERBB2
FADD
IFNG
ERK1/2
methylprednisolone
MYC
curcumin
Ca2+
CREM
PRKCD
Growth hormone
FGF2
MAP3K1
N-acetyl-L-cysteine
anisomycin
tretinoin
IKBKB
deferoxamine
NFKB1
FOXO3
bicuculline
MAPK8
TGFA
SMARCA4
Tgf beta
estrogen receptor
progesterone
FSH
dalfampridine
Jnk
Vegf
Pkc(s)
tunicamycin
L-triiodothyronine
prostaglandin E2
epigallocatechin-gallate
P38 MAPK
salmonella minnesota R595 lipopolysaccharides
INSR
CEBPB
ESR1
NFKBIA
kainic acid
EGFR
INS
SMAD4
Pdgf (complex)
Sb202190
PI3K (complex)
gentamicin
cisplatin
resveratrol
bucladesine
ATF3
D-glucose
ADRB
S100A8
CG
2-amino-5-phosphonovaleric acid
MYD88
romidepsin
NDRG1
NGF
PDX1
arsenite
RAC1
doxorubicin
EGR1
F7
SP1
CREBBP
Alpha catenin
estrogen
ZFP36
FOXL2
nitric oxide
PRKCA
SRC
ethanol
CXCL12
Mt1
MAPK9
calphostin C
calcitriol
trichostatin A
CDK19
EIF2AK2
CHUK
simvastatin
Ins1
SP3
TLR7
Ap1
STAT3
IgG
LDL
FAS
TNFSF11
arachidonic acid
EP300
glutamine
carrageenan
SMAD3
PRKCE
MAPK1
APOE
EIF2AK3
TERT
MAP2K1/2
15-deoxy-delta-12,14 -PGJ 2
MMP1
DETA-NONOate
EPAS1
leukotriene D4
CDK9
HMGA1
SU6656
Z-LLL-CHO
REL
CD40LG
dihydrotestosterone

RANBP9
EXT1
INCB054329
SS18-SSX1
BQ-788
T3-TR-RXR
halofuginone
GPR132
CCL1
PTCSC3
PIKFYVE
UBR5
7-(4-fluorobenzylamino)-1,3,4,8-tetrahydropyrrolo[4,3,2-de]quinolin-8(1H)-one
pterostilbene
triptorelin
Cu2+
lipid
DIO2
embelin
SOX2-OT
CCAT1
CCNT1
PAX4
pentylenetetrazol
baclofen
trientine
NAD+
miR-3121-3p (miRNAs w/seed AAAUAGA)
flutamide
RUNX1-RUNX1T1
miR-3681-5p (and other miRNAs w/seed AGUGGAU)
roscovitine
NR1I2
digoxin
BCO1
CSNK2B
FGF23
EN1
BCAR1
SPINT2
aristolochic acid I
SKF-38393
astressin 2B
GATA2
miR-4696 (miRNAs w/seed GCAAGAC)
PRMT5
APLN
bicalutamide
PTBP1
ILF3
HNRNPD
ROCK2
methimazole
THRAP3
APBB1
mir-199
GHRHR
PER2
TGFBR3
cyproterone acetate
miR-4786-5p (miRNAs w/seed GAGACCA)
prostaglandin A2
glycerol
BMP10
BLNK
BHLHA15
miR-6772-3p (miRNAs w/seed UGCUCCU)
3-methylcholanthrene
GIP
DNAJB6
SMURF2
7-nitroindazole
decursin
glutamyl-Se-methylselenocysteine
GNAS
ZEB2
NMDA Receptor
ARHGDIG
MDM4
CD22
XPO1
YY2
quinpirole
methylphenidate
acyclic retinoid
HES1
carfilzomib
PTPN2
DBP
MUC4
EMD
vanadate
miR-4800-5p (miRNAs w/seed GUGGACC)
FLI1
miR-6731-3p (miRNAs w/seed CUAUUCC)
miR-193a-5p (miRNAs w/seed GGGUCUU)
cyclooxygenase
KLF10
AURKB
lapatinib/pazopanib
RND3
HNRNPA1
9-hydroxy-(S)-10,12-octadecadienoic acid
pelargonidin
2'-adenylic acid
peptide YY 3-36
G protein alpha
growth factor receptor
Ahr-aryl hydrocarbon
2,2',4,4',5,5'-hexabromodiphenyl ether
5,7-dimethoxyflavone
DE 71
Ahr-aryl hydrocarbon-RelA
NR2F1-AS1
G-Actin
tandospirone
PKR dimer
GSK 189254
CLTA
(-)-OSU 6162
GJC2
PMM2
NCK
pridopidine
FBXO31
NLRP2
FBXO22
SRF-ELK1
WFDC1
KCNJ12
CALCOCO1
URGCP
RND2
N-arachidonylglycine
TMEM184A
DEPDC1
NACC2
MYADM
MACROD1
CCDC22
CCDC62
KCTD10
harman
FGF14
TRPM3
PLAC1
RNF181
FANK1
TCF4-CTNNβ
hispidulin
NNZ 2566
vasopressins
black cohosh extract
Integrin alpha 5 beta 1
mediator
Mapk kinase
Rho gdi
GLIPR1
NSMCE3
ARHGEF17
PRCC
MEDI-547
Tenascin
DYNLRB1
RBMS1
AHRR
KIFAP3
MED7
PPY
CDC45
GPHA2
dodecylbenzenesulfonic acid
SLC52A1
ADCY2
CLTB
mir-363
mir-1301
TIMELESS
BTC
IFNA16
OPA1
PAEP
TRIB3
sterol
mir-15
miR-1233-5p (and other miRNAs w/seed GUGGGAG)
miR-669c-3p (and other miRNAs w/seed ACACACA)
miR-635 (and other miRNAs w/seed CUUGGGC)
PPARGC1A
gossypol
C-miR146a
aldesleukin
mir-25
miR-486-3p (and other miRNAs w/seed GGGGCAG)
miR-5694 (miRNAs w/seed AGAUCAU)
RABL6
miR-503-5p (miRNAs w/seed AGCAGCG)
miR-3104-5p (and other miRNAs w/seed AGGGGGC)
ORMDL3
cis-urocanic acid
MAPK13
miR-135b-3p (and other miRNAs w/seed UGUAGGG)
miR-4722-3p (and other miRNAs w/seed CCUGCCA)
3M-011
imipramine blue
RPL22
bempedoic acid
C4BP
DPP-23
miR-3907 (miRNAs w/seed GGUGCUC)
HDL-cholesterol
LTA
CLEC12A
Gm35986
IL6R
miR-328-5p (and other miRNAs w/seed GGGGGGC)
vismodegib
BAK1
miR-3612 (and other miRNAs w/seed GGAGGCA)
miR-6967-5p (and other miRNAs w/seed AGGGAGG)
IL12B
miR-12192-5p (and other miRNAs w/seed GUGGGGU)
miR-491-5p (and other miRNAs w/seed GUGGGGA)
tetrandrine
cannabidiol
miR-374c-5p (and other miRNAs w/seed UAAUACA)
miR-5006-5p (miRNAs w/seed UGCCAGG)
miR-1270 (and other miRNAs w/seed UGGAGAU)
miR-4649-3p (miRNAs w/seed CUGAGGC)
ATF6
miR-6929-5p (and other miRNAs w/seed GGGAGGA)
MSR1
miR-8065 (miRNAs w/seed GUAGGAA)
miR-548an (miRNAs w/seed AAAGGCA)
ISGF3
manumycin A
enterotoxin B
KRT17
miR-607 (miRNAs w/seed UUCAAAU)
fontolizumab
SAA
pristane
miR-485-5p (and other miRNAs w/seed GAGGCUG)
miR-5002-5p (miRNAs w/seed AUUUGGU)
IFNA17
inosine
BTNL2
IL36B
IL37
PAX3
miR-6511a-5p (and other miRNAs w/seed AGGCAGA)
n-3 fatty acids
CLEC4G
let-7f-2-3p (and other miRNAs w/seed UAUACAG)
miR-4651 (and other miRNAs w/seed GGGGUGG)
etanercept
RNASEL
miR-203b-5p (and other miRNAs w/seed AGUGGUC)
Lymphotoxin
miR-27b-5p (miRNAs w/seed GAGCUUA)
miR-4693-5p (miRNAs w/seed UACUGUG)
TRG
UBA7
CTH
SR1078
miR-4436b-3p (and other miRNAs w/seed AGGGCAG)
miR-185-3p (and other miRNAs w/seed GGGGCUG)
miR-3127-3p (and other miRNAs w/seed CCCCUUC)
lipid A
HCAR2
miR-363-5p (and other miRNAs w/seed GGGUGGA)
miR-939-3p (miRNAs w/seed CCUGGGC)
miR-23a-3p (and other miRNAs w/seed UCACAUU)
miR-12183-5p (and other miRNAs w/seed CUUCUUC)
miR-6721-5p (miRNAs w/seed GGGCAGG)
NPC2
NOS2
KDM3B
icilin
DDB2
miR-570-3p (miRNAs w/seed GAAAACA)
SOD2
miR-4668-5p (miRNAs w/seed GGGAAAA)
miR-4804-5p (miRNAs w/seed UGGACGG)
zinc
miR-4433b-5p (miRNAs w/seed UGUCCCA)
miR-4802-3p (and other miRNAs w/seed ACAUGGA)
miR-1273h-5p (and other miRNAs w/seed UGGGAGG)
VitaminD3-VDR-RXR
PRC2
IFNGR1
IL12A
KIF3A
cuprizone
miR-138-5p (miRNAs w/seed GCUGGUG)
ANKRD42
poly(dG-dC)
TRIM8
Immunoglobulin Lambda Light Chain
dacarbazine
miR-144-3p (miRNAs w/seed ACAGUAU)
PLK2
TRPS1
miR-3082-3p (and other miRNAs w/seed ACAUGGC)
miR-3116 (miRNAs w/seed GCCUGGA)
miR-1226-5p (and other miRNAs w/seed UGAGGGC)
SELP
miR-3617-5p (and other miRNAs w/seed AAGACAU)
miR-664-1-5p (and other miRNAs w/seed UGGCUGG)
miR-92a-1-5p (miRNAs w/seed GGUUGGG)
CBX7
grepafloxacin
miR-146a-5p (and other miRNAs w/seed GAGAACU)
miR-6852-5p (and other miRNAs w/seed CCUGGGG)
miR-22-3p (miRNAs w/seed AGCUGCC)
miR-646 (miRNAs w/seed AGCAGCU)
miR-5584-5p (miRNAs w/seed AGGGAAA)
IRAK1
miR-4460 (miRNAs w/seed UAGUGGU)
JAK1/2
LY96
FCGR1A
tranylcypromine
caffeic acid
miR-4740-5p (miRNAs w/seed GGACUGA)
RNASEH2B
PARP14
B4GALT6
LGALS8
LGALS7/LGALS7B
NFKBIE
CYBA
TPCA-1
miR-4650-3p (miRNAs w/seed GGUAGAA)
NGLY1
CTSS
BCAP31
PLK4
SL 327
CFB
TRIM38
SIRPA
miR-1909-3p (and other miRNAs w/seed GCAGGGG)
SCD
miR-3473b (and other miRNAs w/seed GGCUGGA)
miR-628-3p (miRNAs w/seed CUAGUAA)
LGALS1
miR-1285-3p (and other miRNAs w/seed CUGGGCA)
miR-6892-3p (miRNAs w/seed CCCUCUC)
miR-5586-5p (miRNAs w/seed AUCCAGC)
miR-4666a-5p (miRNAs w/seed UACAUGU)
miR-4283 (miRNAs w/seed GGGGCUC)
miR-9-5p (and other miRNAs w/seed CUUUGGU)
kawain
IFNW1
ERVW-1
PP2/AG1879 tyrosine kinase inhibitor
CSF2
MAP2K4
SB203580
AKT1
SOX2
EDN1
NEDD9
ESR2
wortmannin
COL18A1
thioacetamide
Insulin
RASSF1
GNAQ
EPHB1
BCL3
C5
KRAS
sphingosine-1-phosphate
Hdac
PLAUR
AGTR1
S100A9
raloxifene
Mt2
cyclic AMP
HDAC3
HIF1A
Ngf
carbon tetrachloride
F3
troglitazone
RNA polymerase II
trans-hydroxytamoxifen
THPO
F2R
ITGB1
vorinostat
palmitic acid
HDAC1
EPCAM
HDAC6
thyroid hormone
TICAM1
triptolide
5-fluorouracil
herbimycin
camptothecin
5-N-ethylcarboxamido adenosine
STAT5A
TP73
PAF1
MAPK14
NCOA1
LEP
methylnitronitrosoguanidine
FOSL1
cocaine
Tnf (family)
MAP2K1
SERCA
6,7-dinitroquinoxaline-2,3-dione
6-cyano-7-nitroquinoxaline-2,3-dione
PTEN
KLF6
rottlerin
fenretinide
Gsk3
APP
PGR
cytokine
NRAS
crocidolite asbestos
DUSP1
PPARG
platelet activating factor
H1-2
collagenase
etoposide
DDIT3
mycophenolic acid
Fgf
Cdk
atorvastatin
amphetamine
TLR3
MAP2K7
luteolin
NFYA
MAPK3
tamoxifen
AHR
cyclosporin A
Akt
FGF1
IL17A
PRKCB
Salmonella enterica serotype abortus equi lipopolysaccharide
IL4
indirubin
LIF
R5020
Creb
TCF
ELK1
CD40
xanthohumol
ARRB2
MST1R
SIN3A
FOXO1
ATF2
erlotinib
okadaic acid
RBCK1
aplidine
CSF3
CP-55940
GNRH
RAS
genistein
CTNNB1
AR
MET
tributyrin
25-hydroxycholesterol
resiquimod
DACH1
carbamylcholine
WT1
Ras homolog
EDN3
IL1
quercetin
propranolol
H-7
TNFSF10
HNF1A
sphingosylphosphocholine
MEMO1
RTKN
TAZ
PLAU
DICER1
salinosporamide A
RPS6KA5
ionomycin
decitabine
TLR9
Laminin (complex)
D-galactosamine
hydrocortisone
VEGFA
RET
BRD7
evodiamine
lysophosphatidylcholine
MEF2D
silibinin
caffeic acid phenethyl ester
KLHL21
STRAP
2-amino-3-phosphonopropionic acid
acetic acid
phenylephrine
NR5A2
zerumbone
butyric acid
pirinixic acid
MAP3K8
JAK2
Y 27632
CSF1
dibutyl phthalate
tosylphenylalanyl chloromethyl ketone
hyaluronic acid
ADRA1B

| | | |
|---|---|---|
| NPVF | TLR1 | ATP |
| RAB22A | S-(2,3-bispalmitoyloxypropyl)-cysteine-GDPKHPKSF | calcimycin |
| BORCS8-MEF2B | ATP7B | CCL5 |
| SLC30A9 | miR-21-5p (and other miRNAs w/seed AGCUUAU) | NFATC2 |
| CNOT9 | miR-6980-5p (and other miRNAs w/seed UGGGGGG) | aldosterone |
| CAMK2 | ATP5IF1 | ST3-Hel2A-2 |
| EPSTI1 | miR-1468-3p (miRNAs w/seed GCAAAAU) | (+)-MK-801 |
| TOB2 | miR-204-3p (and other miRNAs w/seed CUGGGAA) | TAF4B |
| SLC1A2 | NFYB | BAPTA-AM |
| GTPBP4 | miR-3529-3p (miRNAs w/seed ACAACAA) | GH1 |
| GPHB5 | CpG ODN 1826 | FN1 |
| SCN7A | miR-3202 (miRNAs w/seed GGAAGGG) | NOTCH1 |
| RNF7 | KDM8 | BDNF |
| NAPB | miR-6762-5p (and other miRNAs w/seed GGGGCCA) | USF1 |
| ANKS1A | miR-4446-3p (miRNAs w/seed AGGGCUG) | oleic acid |
| NKX2-8 | miR-483-3p (miRNAs w/seed CACUCCU) | ANGPT2 |
| PTPRU | resolvin D2 | Ige |
| MED21 | CYP7A1 | Immunoglobulin |
| PMS2 | N-(3-(aminomethyl)benzyl)acetamidine | ZBTB17 |
| Sin3b | rosuvastatin | PLK1 |
| EPGN | CpG ODN 2216 | ADRA1A |
| RSL1D1 | JAK inhibitor I | 9,10-dimethyl-1,2-benzanthracene |
| TAX1BP3 | hydroxypropyl-beta-cyclodextrin | staurosporine |
| Tnxa-ps1 | miR-3063-5p (and other miRNAs w/seed GGAGAUC) | TCF4 |
| PRDM2 | miR-4646-3p (miRNAs w/seed UUGUCCC) | ITGA3 |
| RAB5B | miR-541-3p (and other miRNAs w/seed GGUGGGC) | fulvestrant |
| CDK20 | Collagen(s) | 5-O-mycolyl-beta-araf-(1->2)-5-O-mycolyl-alpha-araf-(1->1')-glycerol |
| GDF10 | FLT3 | ETS1 |
| cytarabine/filgrastim/fludarabine phosphate | miR-2682-5p (and other miRNAs w/seed AGGCAGU) | selumetinib |
| ETS1-AS1 | miR-4715-5p (miRNAs w/seed AGUUGGC) | PIK3R1 |
| PTOV1 | miR-1294 (and other miRNAs w/seed GUGAGGU) | reactive oxygen species |
| PRDX6 | n-nitrosomethylbenzylamine | NFKBIB |
| INHBE | miR-222-5p (miRNAs w/seed UCAGUAG) | tanespimycin |
| EXOSC10 | miR-550a-5p (and other miRNAs w/seed GUGCCUG) | carbonyl cyanide m-chlorophenyl hydrazone |
| PPP2R3A | miR-505-3p (miRNAs w/seed GUCAACA) | FGF19 |
| ZBTB14 | FZD9 | CSF2RA |
| RPS20 | LBP | TLE1 |
| CHRM2 | miR-874-5p (miRNAs w/seed GGCCCCA) | RPS6KA4 |
| Flt1 | miR-4763-5p (miRNAs w/seed GCCUGCC) | PPP5C |
| molibresib | miR-2681-3p (miRNAs w/seed AUCAUGG) | LLGL2 |
| oligomycin A | miR-6132 (and other miRNAs w/seed GCAGGGC) | ADIPOQ |
| CML advanced glycation end products | miR-3194-5p (miRNAs w/seed GCCAGCC) | MIB2 |
| dihydrotanshinone I | Pro-inflammatory Cytokine | EIF3I |
| NMS-873 | miR-6747-3p (and other miRNAs w/seed CCUGCCU) | GCLC |
| RPL21 | miR-7851-3p (miRNAs w/seed ACCUGGG) | atipamezole |
| SLC25A5 | miR-6771-5p (miRNAs w/seed UCGGGAG) | di(2-ethylhexyl) phthalate |
| RPLP0 | miR-3616-3p (miRNAs w/seed GAGGGCA) | gefitinib |
| renin-angiotensin-aldosterone system blocking agent | miR-135a-5p (and other miRNAs w/seed AUGGCUU) | IL33 |
| NBI 27914 | miR-767 (and other miRNAs w/seed GCACCAU) | HNF4A |
| alpha-cyano-4-hydroxycinnamate | Esrra | RHOA |
| SB202474 | miR-4742-5p (miRNAs w/seed CAGGCAA) | MYB |
| impromidine | miR-4536-5p (miRNAs w/seed GUGGUAG) | MEF2C |
| guanfacine | miR-6832-3p (miRNAs w/seed CCCUUUU) | PRKCZ |
| amlexanox | miR-1909-5p (miRNAs w/seed GAGUGCC) | AG490 |
| sterigmatocystin | miR-5094 (miRNAs w/seed AUCAGUG) | acetaminophen |
| ondansetron | miR-3174 (miRNAs w/seed AGUGAGU) | VDR |
| cyclothiazide | CASP8 | SPP1 |
| SU6657 | miR-4670-3p (miRNAs w/seed GAAGUUA) | NUPR1 |
| flurothyl | miR-6856-3p (miRNAs w/seed ACAGCCC) | glucocorticoid |
| L 733060 | PADI4 | HLX |
| SC-19220 | ARID3B | TAB1 |
| sulfaphenazole | cyanidin 3-O-glucoside | cadmium chloride |
| remikiren | COPA | PTGS2 |
| selfotel | BLM | CD36 |
| (R)-5-(2-azetidnylmethoxy)-2-chloropyridine | huperzine A | IKBKG |
| Ro 20-1724 | miR-499-5p (and other miRNAs w/seed UAAGACU) | mitomycin C |
| BTG3-AS1 | BNIP3L | KLF4 |
| GS-626510 | belnacasan | topotecan |
| pubchem compound 353999 | Ptprd | 1'-acetoxychavicol acetate |
| triazolam | FH | TCF/LEF |
| thiabendazole | PLCE1 | prazosin |
| arachidonyl-2-chloroethylamide | LIN9 | gamma-tocotrienol |
| naphthalene-1-2-dione | HMGB1 | PC-SPES |
| trans-7,8-dihydroxy-7,8-dihydrobenzo(a)pyrene | miR-4667-3p (miRNAs w/seed CCCUCCU) | PLAT |
| anthracene | miR-501-3p (and other miRNAs w/seed AUGCACC) | fisetin |
| vitamin K1 | ARHGAP31 | CXCR4 |
| sodium 2-((6Z,15Z)-20-hydroxyicosa-6,15-dienamido)acetate | CTLA4 | allopurinol |
| ACS-1 | miR-6854-3p (miRNAs w/seed GCGUUUC) | bee venom |
| 2-amino-3-methylimidazo(4,5-f)quinoline | ibrutinib | LGALS3 |
| ONO-8711 | amitriptyline | mir-8 |
| anti-miR-302d | ceruletide | bortezomib |
| AP-1 decoy | teriflunomide | N-nitro-L-arginine methyl ester |
| anti-miR-302c inhibitor | APOL1 | formaldehyde |
| beta-penta-O-galloyl-glucose | YOD1 | Pka |
| BRD4-NUTM1 | ABCA4 | dopamine |
| ZBTB16-RARA | Fcor | bexarotene |
| SU11333 | PDE3B | vancomycin |
| CTOP | IL1R2 | nicotine |
| PF-4800567 | J11-Cl | CAV1 |
| chymostatin | acriflavine | BRD4 |
| NCB-0846 | orlistat | rotenone |
| Ac-DEVD-CHO | rosmarinic acid | methylselenic acid |
| ibutamoren | miR-125b-2-3p (and other miRNAs w/seed CACAAGU) | CSF2RB |
| SMAD3-EP300 | miR-627-3p (miRNAs w/seed CUUUUCU) | PLCG1 |
| 3-methoxyapigenin | miR-1343-3p (and other miRNAs w/seed UCCUGGG) | IL2RB |
| TEI-F00806 | miR-766-3p (miRNAs w/seed CUCCAGC) | S1PR3 |
| berberrubine | SNCA | exenatide |
| columbamine | miR-1231-3p (miRNAs w/seed GCCCUGU) | phorbol 12,13-dibutyrate |
| 2-methoxy-5-(2',3',4'-trimethoxyphenyl)-2,4,6-cycloheptatrien-1-one | BMI1 | ADRA1 |
| harmaline | black raspberry extract | RAB7A |
| LY 354740 | mir-1 | GCLM |
| droxidopa | 3-methyladenine | MUC1 |
| S(-)-carbidopa | IL17R | lomustine |
| 3'-adenylic acid | VGLL3 | CEBPA |
| 2'-fucosyllactose | neuroprotectin D1 | TREM1 |
| sarcosine | melphalan | cigarette smoke |
| allose | miR-218-1-3p (miRNAs w/seed UGGUUCC) | Interferon alpha |
| estramustine | miR-6846-5p (and other miRNAs w/seed GGGGGCU) | POLR2A |
| CUX1 | 4-(2-aminoethyl)benzenesulfonylfluoride | TBP |
| miR-223-3p (miRNAs w/seed GUCAGUU) | miR-4788 (miRNAs w/seed UACGGAC) | glutathione |
| CD 437 | miR-7977 (miRNAs w/seed UCCCAGC) | pCPT-cAMP |
| NANOG | PLG | Integrin |
| MBD2 | miR-4655-5p (miRNAs w/seed ACCGGGG) | DDX17 |
| LGR5 | prostaglandin D2 | silicon dioxide |
| PER1 | fucoidin | Sos |
| CDK8 | PPARGC1B | PSEN1 |
| miR-214-3p (and other miRNAs w/seed CAGCAGG) | lactosylceramide | TFAP4 |
| NFIX | BCOR | CARM1 |
| MAP3K5 | P2RX7 | H89 |
| CCN3 | aphidicolin | Mapk |
| PDGFC | miR-3144-5p (miRNAs w/seed GGGGACC) | EZH2 |
| HSP90AB1 | miR-609 (miRNAs w/seed GGGUGUU) | JUNB |
| selenomethylselenocysteine | TICAM2 | SNAI1 |
| miR-561-5p (miRNAs w/seed UCAAGGA) | immethridine | S100B |
| DKK1 | geranylgeranyl pyrophosphate | berberine |
| MGP | miR-3151-5p (and other miRNAs w/seed GUGGGGC) | GLI3 |
| EYA1 | miR-876-3p (miRNAs w/seed GGUGGUU) | ID1 |
| CNR2 | miR-1293 (and other miRNAs w/seed GGGUGGU) | sulindac sulfide |
| DYRK1A | miR-299a-3p (and other miRNAs w/seed AUGUGGG) | Pam3-Cys-Ser-Lys4 |
| 2,4-dinitrofluorobenzene | miR-1303 (miRNAs w/seed UUAGAGA) | Histone h3 |
| GDNF | ITGB2 | peptidoglycan |
| ADORA2A | ABCA1 | NCOA2 |
| HIPK2 | CERS5 | SPARC |
| KISS1 | RNU1-1 | ATM |
| COL4A3 | FLT4 | PTHLH |
| VIM | ZNF395 | 2-deoxyglucose |
| HEY1 | NLRP11 | medroxyprogesterone acetate |
| bilirubin | GMFG | NORAD |
| SOX1 | coxsackievirus A21 | TLR7/8 |
| E2F2 | EGTA acetoxymethyl ester | benzyl isothiocyanate |
| miR-4523 (miRNAs w/seed ACCGAGA) | miR-892b (miRNAs w/seed ACUGGCU) | bisindolylmaleimide I |
| CHD7 | TAC1 | quisqualic acid |
| GHRH | doxycycline | Calcineurin protein(s) |
| SDCBP | FCGR2B | AGER |
| SSTR2 | miR-4755-3p (miRNAs w/seed GCCAGGC) | PI3K (family) |
| miR-3186-3p (and other miRNAs w/seed CACGCGG) | miR-6777-3p (miRNAs w/seed CCACUCU) | POU5F1 |
| STK40 | miR-4659a-3p (and other miRNAs w/seed UUCUUCU) | pentoxifylline |
| thyroid hormone receptor | miR-4531 (miRNAs w/seed UGGAGAA) | isobutylmethylxanthine |
| HDAC9 | nystatin | BMP2 |
| PSMD10 | SELENOS | ERBB3 |
| diazoxide | ZBTB33 | NTRK2 |
| cyanocobalamin | mir-140 | BMP4 |
| miR-524-3p (and other miRNAs w/seed AAGGCGC) | INSIG2 | KL |
| CDX2 | NRP1 | pituitary adenylate cyclase-activating polypeptide |
| CPE | IL18R1 | TP53COR1 |
| isoflurane | dexmedetomidine | CSNK2A1 |
| miR-4677-5p (miRNAs w/seed UGUUCUU) | CALC | sulfasalazine |
| miR-452-3p (miRNAs w/seed UCAUCUG) | miR-4789-3p (miRNAs w/seed ACACAUA) | lapatinib |
| miR-4687-5p (miRNAs w/seed AGCCCUC) | miR-4721 (and other miRNAs w/seed GAGGGCU) | SRF |
| estradiol benzoate | miR-921 (miRNAs w/seed UAGUGAG) | nitrofurantoin |
| HMGN1 | miR-3670 (miRNAs w/seed GAGCUCA) | methyl methanesulfonate |
| USP7 | CX3CL1 | baicalein |
| apomorphine | miR-96-5p (and other miRNAs w/seed UUGGCAC) | MAPK7 |
| progestin | miR-4259 (miRNAs w/seed AGUUGGG) | PRL |
| miR-1237-3p (and other miRNAs w/seed CCUUCUG) | miR-203b-3p (miRNAs w/seed UGAACUG) | STAT5B |
| Rb | miR-4470 (miRNAs w/seed GGCAAAC) | SIRT1 |
| MBD3 | PLAAT4 | Ro31-8220 |
| miR-3940-3p (miRNAs w/seed AGCCCGG) | miR-4718 (miRNAs w/seed GCUGUAC) | ADRB3 |
| cysteinyl-leukotriene | Collagen type II | SIN3B |
| L-leucine | miR-4324 (miRNAs w/seed CCUGAGA) | epirubicin |
| isopropyl thiogalactoside | miR-421-3p (and other miRNAs w/seed UCAACAG) | GSK3B |
| ginsenoside Rh2 | LRP1 | MMP9 |
| Cop9 Signalosome | miR-26a-5p (and other miRNAs w/seed UCAAGUA) | IFI16 |
| CERT1 | IDR-1002 | PTPN11 |
| Pak | miR-376a-3p (and other miRNAs w/seed UCAUAGA) | SFTPA1 |
| Cofilin | miR-4645-5p (and other miRNAs w/seed CCAGGCA) | ciglitazone |
| 2,2',4,4'-tetrabromodiphenyl ether | HCAR1 | androgen |
| hexadecanedioic acid | LECT2 | 5-azacytidine |

nitrosomethylurethane
canthaxanthin
Rab11
USP42
RBMS3
ADRM1
REM2
OSI-027
GPR4
BCAS3
USP36
BATF2
VENTX
FKRP
MRGPRX1
ETS-ELK1
RYR3
INTERLEUKIN
EIF4H
RNMT
NOSIP
MCF2L
CSDE1
ATP1A1
MMP10
PTPRF
HLTF
DAPK3
ZNF224
RAC3
PTPA
AMD1
LAMA1
STAM
mir-605
RPL15
CCNDBP1
NASP
ARHGEF28
FGB
GAD1
VANGL1
NEK4
FBXO4
BCLAF1
TIAM2
POU4F3
GABRG2
ILKAP
WASHC1
TH
RPA2
silvestrol
RPS25
DPH1
STAMBP
GRAP
alirocumab
FRAT1
TRIP11
UBE2V1
PPP2R5A
RAMAC
TMPRSS4
RNF11
STYX
sorafenib derivative SC-1
LY3039478
1-(3-chlorophenyl)piperazine
3-iodobenzylguanidine
NPC 15437
amsacrine
phenoxyacetic acid
palytoxin
alpha-endosulfan
phosphorylethanolamine
sibutramine
imidapril
neratinib
butyrine
chlordiazepoxide
GYKI 52466
butylbenzyl phthalate
sodium fluoride
EWSR1-WT1
procaterol
aprotinin
enalaprilat
ropinirole
tegaserod
veratridine
teleocidins
5-oxo-6-8-11-14-(e,z,z,z)-eicosatetraenoic acid
cyclosporin H
5-thio-D-glucose
3-O-methylglucose
flunisolide
letrozole
succinic acid
miR-6794-3p (miRNAs w/seed UCACUCU)
miR-510-3p (miRNAs w/seed UUGAAAC)
ALKBH5
CRTC2
GNAI2
LTB4R
oltipraz
R-WIN 55,212
maslinic acid
miR-3908 (miRNAs w/seed AGCAAUG)
NR2E1
FECH
SRSF1
PRKAR1A
DRD2
Hmgn3
clodronic acid
4-tert-octylphenol
butylated hydroxyanisol
miR-552-5p (miRNAs w/seed UUUAACC)
PTH1R
NF2
SOX10
TNFSF15
betulinic acid
miR-4634 (miRNAs w/seed GGCGCGA)
ZFHX3
miR-6853-5p (miRNAs w/seed GCGUGGG)
WNT4
AHCY
GDF11
oleanolic acid
RAB1B
SKP2
mir-126
miR-4768-5p (and other miRNAs w/seed UUCUCUC)
miR-6740-3p (miRNAs w/seed GUCUUCU)
miR-3194-3p (miRNAs w/seed GCUCUGC)
DSCAM
GSTP1
miR-6757-3p (miRNAs w/seed ACACUGG)
pregnenolone sulfate
AMOT
ANTXR2
14,15-DHET
DUB
myosin-light-chain kinase
FKHR
lipoxygenase
PLC gamma
Stat1-Stat3
diosmin
sethoxydim
cetylpyridinium
Corticosteroid-GCR
NSD3
Jun-ATF2
LZTS2
RFFL
PUM1
NOP58
SPRED2
H1-3
CNOT1
GPC6
NDUFAF3
SENP8
MYLIP
Ribosomal Subunit 80S
PVT1
HDGF
AMOTL1
OLA1
Trk Receptor
UBE2D3
Gap
SERTAD1
RRAD
MAT2B
SRT1720
PELI1
NELFB
LILRB4
ARF6
RNF216
DBI
BEX2
HNF1A-AS1
BIRC2
taxifolin
miR-6881-5p (miRNAs w/seed GGGGUAA)
miR-1200 (miRNAs w/seed UCCUGAG)
miR-2110 (and other miRNAs w/seed UGGGGAA)
miR-4423-5p (miRNAs w/seed GUUGCCU)
CIITA
Cdc42
CD300LF
MALT1
VCP
6-amino-4-(4-phenoxyphenylethylamino)quinazoline
bendamustine
NEK7
HA900
PD 168393
miR-6849-3p (miRNAs w/seed CCAGCCU)
CRH
IL17a dimer
FGF4
BMP15
CXCR2
EIF4EBP2
desipramine
ilomastat
miR-1247-3p (and other miRNAs w/seed GGGAACG)
miR-3170 (and other miRNAs w/seed UGGGGUU)
miR-95-5p (miRNAs w/seed CAAUAAA)
ATF6B
HECTD3
Carlr
ASCC1
TRIL
UFD1
MUC2
NPLOC4
BICD2
CDC20
VR23
IgG-opsonized ovalbumin
miR-6825-5p (and other miRNAs w/seed GGGGAGG)
miR-432-3p (and other miRNAs w/seed UGGAUGG)
miR-3918 (miRNAs w/seed CAGGGCC)
miR-1587 (and other miRNAs w/seed UGGGCUG)
miR-4707-3p (miRNAs w/seed GCCCGCC)
miR-3173-5p (and other miRNAs w/seed GCCCUGC)
miR-1288-3p (miRNAs w/seed GGACUGC)
miR-4667-5p (and other miRNAs w/seed CUGGGGA)
miR-6501-5p (miRNAs w/seed GUUGCCA)
miR-142-3p (and other miRNAs w/seed GUAGUGU)
miR-3162-5p (miRNAs w/seed UAGGGAG)
miR-7150 (miRNAs w/seed UGGCAGG)
miR-3183 (and other miRNAs w/seed CCUCUCU)
miR-1178-5p (miRNAs w/seed AGGGUCA)
miR-3199 (and other miRNAs w/seed GGGACUG)
miR-3609 (and other miRNAs w/seed AAAGUGA)
miR-4260 (and other miRNAs w/seed UUGGGGC)
miR-129-1-3p (and other miRNAs w/seed AGCCCUU)
miR-4779 (miRNAs w/seed AGGAGGG)
miR-4671-3p (miRNAs w/seed UAGUGCA)
BCL2L1
birabresib
miR-6748-5p (and other miRNAs w/seed GUGGGUG)
miR-1287-3p (and other miRNAs w/seed UCUAGCC)
miR-515-5p (and other miRNAs w/seed UCUCCAA)
miR-6874-5p (and other miRNAs w/seed UGGAGCU)
CORT
misoprostol
IL1RL2
HIF1AN
MAPKAP1
mevastatin
miR-3065-5p (and other miRNAs w/seed CAACAAA)
miR-875-5p (and other miRNAs w/seed AUACCUC)
AIRE
miR-6762-3p (miRNAs w/seed GGCUGCU)
HOXA10
miR-105-5p (and other miRNAs w/seed CAAAUGC)
miR-1913 (miRNAs w/seed CUGCCCC)
RUNX1
miR-4709-5p (miRNAs w/seed CAACAGU)
miR-6499-3p (miRNAs w/seed GCAGUGU)
2-(4-acetoxyphenyl)-2-chloro-N-methylethylamine
1,25-dihydroxyvitamin D
TLR8
SERPINA1
Hbb-b2
ZNF217
miR-6509-5p (miRNAs w/seed UUAGGUA)
miR-6879-3p (miRNAs w/seed GUCACCC)
ssRNA40
PHLPP1
miR-5187-5p (miRNAs w/seed GGGAUGA)
STK4
HSPA5
miR-211-3p (miRNAs w/seed CAGGGAC)
infliximab
miR-3473h-5p (and other miRNAs w/seed AGGGGCU)
RGS10
miR-4783-3p (miRNAs w/seed CCCGGUG)
miR-1305 (miRNAs w/seed UUUCAAC)
miR-6832-5p (miRNAs w/seed GUAGAGA)
miR-132-3p (and other miRNAs w/seed AACAGUC)
miR-4695-3p (miRNAs w/seed GAUCUCA)
miR-1207-5p (and other miRNAs w/seed GGCAGGG)
miR-4758-3p (miRNAs w/seed GCCCCAC)
miR-4654 (and other miRNAs w/seed GUGGGAU)
miR-4484 (miRNAs w/seed AAAGGCG)
miR-1976 (and other miRNAs w/seed CUCCUGC)
miR-4638-3p (and other miRNAs w/seed CUGGACA)
miR-3145-5p (miRNAs w/seed ACUCCAA)
MITF
miR-6878-3p (and other miRNAs w/seed UGGCCUC)
PCGEM1
miR-4733-3p (and other miRNAs w/seed CACCAGG)
miR-1262 (and other miRNAs w/seed UGGGUGA)
tetrodotoxin
FSTL1
B4GALNT1
PIM3
6-aminopyrazolopyrimidine derivative compound II
cytochalasin B
Complement
miR-2682-3p (and other miRNAs w/seed GCCUCUU)
NPPB
MMP14
IL17RA
miR-3169 (miRNAs w/seed AGGACUG)
miR-7702 (miRNAs w/seed UUAGACU)
miR-6734-5p (and other miRNAs w/seed UGAGGGG)
GSK2816126
MYOC
miR-192-3p (miRNAs w/seed UGCCAAU)
miR-1237-5p (and other miRNAs w/seed GGGGGCG)
miR-6801-3p (and other miRNAs w/seed CCCCUGC)
TEAD3
IRF6
brefeldin A
miR-151-5p (and other miRNAs w/seed CGAGGAG)
MSC
miR-290b-3p (and other miRNAs w/seed AGUGCCC)
miR-5682 (miRNAs w/seed UAGCACC)
miR-617 (miRNAs w/seed GACUUCC)
miR-4494 (miRNAs w/seed CAGACUG)
miR-6512-5p (miRNAs w/seed ACCAUUA)
phytohemagglutinin
miR-4736 (and other miRNAs w/seed GGCAGGU)
miR-4712-5p (and other miRNAs w/seed CCAGUAC)
CLEC7A
PD 123319
epoxomicin
trinitrobenzenesulfonic acid
miR-4720-5p (and other miRNAs w/seed CUGGCAU)
miR-296-5p (miRNAs w/seed GGGCCCC)
miR-3714 (miRNAs w/seed AAGGCAG)
O6-benzylguanine
anacardic acid
ANKRD17
rhesus theta-defensin 1
IL10RB
ANG
ITGA4
LUM
PRKRA
CXCL16
TRAF1
COP1
pubchem compound 11368987
mastoparan
PTH
N-acetylsphingosine
nicotinic acetylcholine receptor
AKIRIN2
thenoyltrifluoroacetone
Muscarinic cholinergic receptor
PARP1
mir-17
ACVR1C
LRP6
SPHK1
SB 216763
C11orf95-RELA
LIPE
MACROH2A1
MTPN
STAT1
PROC
ADRA1D
HNRNPK
quinolinic acid
LDLR
ADCYAP1
YY1
TFPI2
fluoxetine
ITGA5
2-(4-amino-1-isopropyl-1H-pyrazolo[3,4-d]pyrimidin-3-yl)-1H-indol-5-ol
NLRX1
CCN1
dexamethasone phosphate
Ciap
IPMK
HOXA5
bisindolylmaleimide iv
Ni2+
IRS1
IL1RN
saikosaponin A
deoxycholate
methotrexate
WNT1
FGFR2
AREG
ERBB4
nitroprusside
indomethacin
calcipotriene
NfkB-RelA
P2RY2
piceatannol
kaempferol
tert-butyl-hydroquinone
8-bromo-cAMP
tacrolimus
phorbol esters
BAG1
KN-62
amino acids
linolenic acid
methyl-beta-cyclodextrin
Rock
TRAF3
methapyrilene
SOCS3
Ck2
ICAM1
JAG1
IRF8
MAC
CTSB
ECSIT
ozone
ceramide
rosiglitazone
TAF4
SYVN1
lactacystin
FGFR1
NCOR2
sulforafan
TRADD
hydrochloric acid
olanzapine
GRP
IRAK3
fludarabine
DAB2IP
Igkv1-117
MAPKAPK2
fenofibrate
ELAVL1
PTGES
CDKN1A
POMC
CEBPD
PRKDC
dithiothreitol
bardoxolone methyl
TFAP2A
RBPJ
levodopa
APEX1
bufalin
E2F1
THRB
SNHG20
PIAS2
DRD1
VLDLR
AFP
clioquinol
clonidine
OSMR
sodium arsenite
STAT6
potassium chloride
IFNAR1
GPS2
napabucasin
Sn50 peptide
Stat3-Stat3
grape seed extract
ROR1
vitamin K3
MSK1/2
FBXO25
OMG
NMB
RAMP3
nitrosonifedipine
GLI2
CCN2
PDGFB
Go 6976
MED1
VIP
CD24
BCL2
ITGAV
ALOX15
honokiol
TSH
CD3E
methylmercury
triamterene
miR-155-5p (miRNAs w/seed UAAUGCU)
TNFRSF1A
L-histidine
RB1CC1
SR 144528
pyrrolidine dithiocarbamate
puromycin aminonucleoside
IL13
SHC1
VCAN
ANXA2
ARRB1
CDC42
green tea polyphenol
miR-200b-3p (and other miRNAs w/seed AAUACUG)
CpG oligonucleotide
KCNE3
Map3k7
3,3'-diindolylmethane
diethylnitrosamine
metribolone
PML
TAL1
PRKAG3
IKK (complex)
SETD2
SPDEF
Collagen type I (complex)
fenamic acid
testosterone
EPO
TRAF6

SPTBN1
AGAP2
BPTF
mir-497
mir-383
MAPK15
STRN
SLC1A1
RPS14
PRDX4
apitolisib
PUM2
aurora kinase inhibitor III
FOXO6
RALGDS
NCOA5
SCN1B
MGAT4A
SLC3A2
MED26
sulfurbutylether-beta-cyclodextrin
PTTG1IP
SRPK2
ADCY6
GCNT1
SIGLEC10
LSS
SLC12A6
CTAG1A/CTAG1B
RASSF7
STAM2
PES1
RBBP8
CNPY2
ARHGEF11
EEF1A1
Hspa1b
RPL23A
H1-1
RDH16
NOLC1
(+)-R-3-(4-chlorophenyl)-3-(1-hydroxymethylcyclopropylmethoxy)-2-(4-nitrobenzyl)-2,3-dihydroisoindol-1-one
BCKDHA
capivasertib
umbralisib
mir-297
singlet oxygen
compound 48/80
2,2-dimethylbutyric acid
p-chloroamphetamine
oxotremorine
ethyl ether
rocuronium
devazepide
tamsulosin
3-nitrobenzanthrone
betamethasone valerate
salsalate
ethyl gallate
taprostene
phloretin
lanthanum chloride
sodium iodide
C66
3,4-methylenedioxyamphetamine
glutathione diethyl ester
GNE-317
R191
R-etodolac
muscarine
AVI-4126
mizoribine
D,L-propargylglycine
CH6953755
raffinose
prostratin
ponasterone A
Mn2+
miR-3118 (and other miRNAs w/seed GUGACUG)
miR-6809-3p (miRNAs w/seed UUCUCUU)
Ap2
TFDP1
GTF2B
CX3CR1
miR-1257 (miRNAs w/seed GUGAAUG)
miR-4668-3p (miRNAs w/seed AAAAUCC)
IGHM
IL9
S-adenosylmethionine
NR0B2
miR-1471 (miRNAs w/seed CCCGCGU)
miR-3152-3p (and other miRNAs w/seed GUGUUAG)
SHH
miR-1180-3p (miRNAs w/seed UUCCGGC)
miR-769-5p (miRNAs w/seed GAGACCU)
NR2F1
CGA
miR-4681 (miRNAs w/seed ACGGGAA)
LG100268
let-7d-3p (miRNAs w/seed UAUACGA)
miR-4477a (miRNAs w/seed UAUUAAG)
miR-526a-5p (and other miRNAs w/seed UCUAGAG)
memantine
13(S)-hydroxyoctadecadienoic acid
CGP 74514A
6-dimethylaminopurine
adenine
PD 180970
L-alanine
ribose
inecalcitol
Osteocalcin
chondroitin sulfate B
darbepoetin alfa
SHP
tyrosine kinase
Stat dimer
Esr1-Esr1-estrogen-estrogen
1-methyl-2-pyrrolidinone
aluminum hydroxide
benoxaprofen
CINP
GPR65
DNAJB4
AMOTL2
GLIPR2
ENTPD2
GJB6
SLCO1C1
endothelin receptor
Slfn1
ORAI3
MUCL1
SLC39A4
XRN2
Sapk
VLDL
PDLIM1
BIIB021
Casein
Nc2
ZNF467
bitertanol
N-docosahexaenoylethanolamine
KCNJ2
TLCD3A
ADRA2B
ARTN
PSME3
G0S2
ARID1B
mir-338
mir-138
mir-32
TOPORS
CALCR
LIMK1
TIAM1
SEPTIN9
CALM1 (includes others)
HUNK
GALR1
TCF20
lanatoside C
GIPC1
NME2
UBTF
SOS1
ABI1
Cyp2c70
CTNNAL1
CCKAR
ADRA2A
ARHGEF2
FOXH1
LETMD1
SERPINB7
AKAP13
poly(U)RNA
coconut oil
bis(3',5')-cyclic diguanylic acid
FIRRE
CACNA1C
GNE
SDC4
MARCO
PROK1
ISG15
C4A/C4B
IL18RAP
Ptgs2os2
ubiquinone 9
RWJ 67657
borrelia burgdorferi strain B31 peptidoglycan
miR-1278 (miRNAs w/seed AGUACUG)
bevacizumab
Foxo
Angiotensin II receptor type 1
FKBP4
LAMA5
IL1RAP
TNK1
Tnfsf9
TARBP2
phenyl-N-tert-butylnitrone
cerulenin
D609
oxysterol
garcinol
IL36G
GABA
miR-6761-5p (and other miRNAs w/seed CUGAGAG)
EBF1
miR-7855-5p (miRNAs w/seed UGGUGAG)
miR-6778-3p (miRNAs w/seed GCCUCCC)
miR-4305 (miRNAs w/seed CUAGACA)
miR-345-5p (and other miRNAs w/seed CUGACCC)
TNFRSF18
YWHAZ
GNAI3
TRIM21
miR-6880-5p (miRNAs w/seed GGUGGAG)
miR-4719 (miRNAs w/seed CACAAAU)
miR-1306-5p (miRNAs w/seed CACCUCC)
miR-1188-3p (and other miRNAs w/seed GAGGCUC)
N-formyl-Met-Leu-Phe
maneb
miR-3913-3p (miRNAs w/seed GACAUCA)
miR-147-5p (and other miRNAs w/seed GGAAACA)
ZBED6
miR-3923 (miRNAs w/seed ACUAGUA)
miR-3103-5p (and other miRNAs w/seed GAGGGAG)
miR-1249-5p (and other miRNAs w/seed GGAGGGA)
miR-4524a-5p (and other miRNAs w/seed UAGCAGC)
miR-6758-5p (and other miRNAs w/seed AGAGAGG)
miR-3090-3p (and other miRNAs w/seed CCCAGGU)
miR-11181-5p (and other miRNAs w/seed UCUGACC)
miR-548e-5p (miRNAs w/seed AAAAGCA)
miR-6786-3p (miRNAs w/seed GACGCCC)
miR-7976 (miRNAs w/seed GCCCUGA)
miR-5707 (miRNAs w/seed CGUUUGA)
miR-718 (miRNAs w/seed UUCCGCC)
miR-210-5p (and other miRNAs w/seed GCCACUG)
miR-545-3p (miRNAs w/seed CAGCAAA)
miR-4271 (and other miRNAs w/seed GGGGAAG)
miR-593-5p (and other miRNAs w/seed GGCACCA)
miR-6850-5p (miRNAs w/seed UGCGGAA)
LPIN1
KDM6B
VAV3
suramin
N(G)-monomethyl-D-arginine
miR-450b-3p (and other miRNAs w/seed UGGGAUC)
miR-6871-5p (miRNAs w/seed AUGGGAG)
ZNF106
miR-122b-3p (and other miRNAs w/seed AACACCA)
miR-155-3p (miRNAs w/seed UCCUACA)
miR-4521 (miRNAs w/seed CUAAGGA)
ZAP70
delta-9-tetrahydrocannabinol
ARID1A
miR-670-3p (miRNAs w/seed UUCCUCA)
miR-519d-5p (miRNAs w/seed CUCCAAA)
CD3 group
miR-4489 (miRNAs w/seed GGGGCUA)
POU2F2
miR-1953 (and other miRNAs w/seed GGGAAAG)
miR-1839-5p (and other miRNAs w/seed AGGUAGA)
miR-3189-5p (miRNAs w/seed GCCCCAU)
miR-638 (miRNAs w/seed GGGAUCG)
CDKN2B-AS1
TRAF3IP3
NAE1
RGCC
LAMC1
N-(3-oxododecanoyl)-homoserine lactone
picryl chloride
spermine
loxoribine
manganese
miR-4672 (miRNAs w/seed UACACAG)
miR-4468 (miRNAs w/seed GAGCAGA)
Hif1
miR-6737-5p (and other miRNAs w/seed UGGGGUG)
miR-4675 (and other miRNAs w/seed GGGCUGU)
miR-1302 (and other miRNAs w/seed UGGGACA)
miR-3154 (miRNAs w/seed AGAAGGG)
miR-5129-5p (and other miRNAs w/seed UGUGGGG)
ITGAM
miR-196a-3p (miRNAs w/seed GGCAACA)
miR-4640-5p (and other miRNAs w/seed GGGCCAG)
ASPSCR1-TFE3
miR-292-3p (and other miRNAs w/seed AGUGCCG)
miR-3158-3p (miRNAs w/seed AGGGCUU)
miR-6862-5p (and other miRNAs w/seed GGGCAUG)
miR-125b-1-3p (miRNAs w/seed CGGGUUA)
miR-1914-3p (and other miRNAs w/seed GAGGGGU)
miR-1269a (and other miRNAs w/seed UGGACUG)
miR-1224-3p (miRNAs w/seed CCCACCU)
LILRB1
miR-217-5p (and other miRNAs w/seed ACUGCAU)
miR-6746-5p (miRNAs w/seed CGGGAGA)
Ctbp
LRBA
hemoglobin
PLCG2
FDFT1
superoxide
sertraline
miR-3651 (miRNAs w/seed AUAGCCC)
ursodeoxycholic acid
FCER1G
miR-4456 (and other miRNAs w/seed CUGGUGG)
miR-219a-5p (and other miRNAs w/seed GAUUGUC)
miR-3126-3p (miRNAs w/seed AUCUGGC)
miR-4640-3p (miRNAs w/seed ACCCCCU)
miR-146a-3p (and other miRNAs w/seed CUCUGAA)
miR-4652-5p (miRNAs w/seed GGGGACU)
estriol
GPX1
N-[N-(3,5-difluorophenacetyl-L-Ala)]-S-phenylglycine t-butyl ester
miR-4660 (miRNAs w/seed GCAGCUC)
alefacept
pyridaben
FOSL2
miR-181a-2-3p (and other miRNAs w/seed CCACUGA)
ATG7
TBX2
miR-1275 (and other miRNAs w/seed UGGGGGA)
miR-3147 (miRNAs w/seed GUUGGGC)
miR-6733-3p (and other miRNAs w/seed CAGUGUC)
miR-6720-3p (miRNAs w/seed GCGCCUG)
miR-3622a-5p (miRNAs w/seed AGGCACG)
miR-183-5p (miRNAs w/seed AUGGCAC)
TCOF1
miR-205-5p (and other miRNAs w/seed CCUUCAU)
miR-494-3p (miRNAs w/seed GAAACAU)
miR-3606-5p (miRNAs w/seed UAGUGAA)
miR-8083 (miRNAs w/seed AGGACUU)
Hbb-b1
miR-4705 (and other miRNAs w/seed CAAUCAC)
PPP3CA
PPP1R15B
Traj18
WWP2
IL18BP
S100A7
pepstatin
Z-DEVD-FMK
mimosine
nandrolone
miR-367-5p (miRNAs w/seed CUGUUGC)
miR-2116-3p (miRNAs w/seed CUCCCAU)
ETV4
rolipram
miR-4527 (and other miRNAs w/seed GGUCUGC)
bromobenzene
CDKN2A
L-glutamic acid
CHRM1
ELK4
gambogic acid
ACVRL1
amiodarone
PML-RARA
gemfibrozil
HTT
pioglitazone
linoleic acid
GNA14
mir-34
FBXW7
lipoxin A4
TRH
XDH
CAMK4
GNB2
norepinephrine
miR-10394-3p (and other miRNAs w/seed GGGCGCG)
TRAF3IP2
tyrphostin AG 1478
tetrachlorodibenzodioxin
MEG3
PIAS4
HOXA4
MVP
pentobarbital
noscapine
growth factor
Cd2+
TWIST2
ursolic acid
BCL6
JQ1
GW3965
aspirin
NOD2
cadmium
GNB1
NR1D1
MIF
Il3
PF4
TLR4
miR-101-3p (and other miRNAs w/seed ACAGUAC)
BRCA1
PRNP
cyclophosphamide
cerivastatin
ENG
ABL1
histone deacetylase
propofol
lonafarnib
TIFA
48s
Betacatenin/TCF
SP2
GTF2I
FOXP3
heparin
GATA3
celecoxib
PP1
MAP3K7
HMOX1
Lh
PD173074
KDM3A
IRS2
epoprostenol
cyclopiazonic acid
myricetin
CXCL8
MAP3K14
GAST
exendin(9-39) amide
potassium channel
CSNK1E
ELL
SGI 1776
TOR2A
TRAF7
RING1
di-2-pyridylketone 4-cyclohexyl-4-methyl-3-thiosemicarbazone
tarenflurbil
metformin
verapamil
CNTF
TRAF2
TSC2
ATF4
NAMPT
leukotriene C4
triclosan
CHD1
CCAT2
NEDD4
THRA
HDAC4
cobalt chloride
ZMPSTE24
bleomycin
SOD1
LYN
histamine
miR-4281 (miRNAs w/seed GGUCCCG)
HOTAIR
IFNLR1
BRAF
NR3C2
miR-4753-5p (miRNAs w/seed AAGGCCA)
GLIS2
CRBN
Endothelin
mir-154
S1PR2
RPS6KA3
lisinopril
acadesine
chenodeoxycholic acid
SBDS
IKBKE
TGFBR1
IL3
K+
SOCS1
SP110
NCOA3
PKD1
TGFBR2
AVP
RARG
GATA4
prostaglandin E1
NOD1
MNT
PDCD4
TREM2
ACTN4
PS-1145
peroxynitrite
IFIH1
IL11RA
TXN
CCL2
lovastatin
SQSTM1
BSG
RETN
elaidic acid
ERG
SAV1
mir-486
S1PR1
farnesol
7-ketocholesterol
CFL1
ibotenic acid
KMT2A
Notch
miR-100-3p (miRNAs w/seed AAGCUUG)
DGCR8
TNFAIP3
MAP2K5
TIMP3
candesartan
valsartan
YAP1
Histone h4
methamphetamine
PRKAA1
GFI1
daidzein

chelidonine
S3I-1757
COL1A2
CRLF1
PLAC8
ARF1
TOP2A
PODXL
RPL5
TBPL1
3',4'-dimethoxyflavone
LY5
benfur
SRT2183
5,6,7,8-tetrahydrobiopterin
apicularen A
acetoacetic acid
ketanserin
NSC719239
tipifarnib
KP-SD-1
clomiphene
bilobalide
liarozole
alpha-methylhydrocinnamic acid
phenylhydrazine
glabridin
pentamidine
lomefloxacin
cromakalim
nitrendipine
salicylamide
4-methylcatechol
methylpiperidinyl-3b-hydroxycholenamide
MS645
CCI-007
WZB117
Phe-Pro-Arg-chloromethyl ketone
bisindolylmaleimide II
resiniferatoxin
galangin
nor-binaltorphimine
colcemid
palmatine
tyrphostin AG 18
iodixanol
L-asparagine
N-butyldeoxynojirimycin
Collagen type VI
trehalose
cholesterol ester
pyocyanin
Co2+
Pb2+
miR-6728-3p (miRNAs w/seed CUCUGCU)
miR-4317 (miRNAs w/seed CAUUGCC)
MSX2
PXR ligand-PXR-Retinoic acid-RXRα
SLC27A2
miR-454-5p (miRNAs w/seed CCCUAUC)
miR-1970 (and other miRNAs w/seed GUGUCAC)
miR-6076 (and other miRNAs w/seed GCAUGAC)
SKIL
SST
NME1
miR-411-5p (and other miRNAs w/seed AGUAGAC)
miR-1291 (and other miRNAs w/seed UGGCUCU)
miR-4664-3p (miRNAs w/seed UUCCGGU)
miR-6124 (miRNAs w/seed GGAAAAG)
1,2-dioctanoyl-sn-glycerol
L-aspartic acid
p38 Sapk
ILX-23-7553
C9orf116
Hbb
ATPase
Erm
Ho
1,2,3,4,6,7,8-heptachlorodibenzofuran
olmesartan
SMPD3
DRD3
MELK
KLF8
GPR17
SSBP2
MTF2
FOXK2
Ftx
UTP18
ZDHHC2
SNHG3
CDH16
PCA3
RPE65
GPR68
harmol
Cathepsin
7S NGF
MAP4K1
Atf
GRAP2
ZIC1
FAF1
PUF60
RNF34
mir-190
MIR4269
SFRP2
MRAS
ADAR
LSP1
MAPKAPK5
UTS2
PENK
FLOT1
ARHGEF25
RAP1GDS1
PORCN
MYCT1
BCAR3
CEL
Tmsb4x (includes others)
CMA1
VCAM1
GNL2
CDH13
Nrg1
KRT10
Foxe3
Mcpt1
H1f4
ILF2
LGH447
gabapentin
2-cyclohexen-1-one
LY379196
silicon phthalocyanine
lindane
triiodothyronine, reverse
ranitidine
2,3,7,8-tetrachlorodibenzofuran
etidronic acid
orantinib
opioid
H-8
4-aminophenol
butylphen
sodium azide
dexfenfluramine
cannabinoid drugs
phloroglucinol
5-hydroxydecanoic acid
5-hydroxytryptophan
Hg2+
miR-6817-3p (miRNAs w/seed CUCUCUG)
miR-7844-5p (miRNAs w/seed AAACUAG)
miR-6902-3p (miRNAs w/seed CAUGUGA)
miR-4751 (miRNAs w/seed GAGGACC)
miR-6753-5p (miRNAs w/seed ACCAGGG)
miR-891a-3p (miRNAs w/seed GUGGCAC)
20-hydroxyeicosatetraenoic acid
adenosine dialdehyde
nitrite
L-glutamine
Iberiotoxin
Neurotrophin
F Actin
DPPA3
Beta Arrestin
EIF4A3
CCAR1
KCNK2
ANKHD1/ANKHD1-EIF4EBP3
PDZK1IP1
AQP11
lumefantrine
taspoglutide

miR-3198 (and other miRNAs w/seed UGGAGUC)
miR-3689d (and other miRNAs w/seed GGAGGUG)
miR-384-3p (and other miRNAs w/seed UUCCUAG)
miR-4684-5p (miRNAs w/seed UCUCUAC)
miR-4633-3p (and other miRNAs w/seed GGAGCUA)
miR-1915-5p (miRNAs w/seed CCUUGCC)
miR-4778-5p (miRNAs w/seed AUUCUGU)
diallyl disulfide
acetylcholine
ETV5
miR-135a-1-3p (and other miRNAs w/seed AUAGGGA)
STUB1
miR-4495 (miRNAs w/seed AUGUAAA)
eritoran
beta-glucan
1-palmitoyl-2-oleoylglycero-3-phosphoglycerol
SPINDOC
TRIM58
Tlr12
FGA
SPIN1
ARL5B
TNFAIP8
PLAA
polyinosine-polycytidylic acid/polyethylenimine formulation
pubchem compound 11483544
sulfamethoxazole/trimethoprim
sappanone A
miR-558 (and other miRNAs w/seed GAGCUGC)
eflornithine
galactosylceramide-alpha
SIRT3
miR-4529-3p (miRNAs w/seed UUGGACU)
ATN1
MASTL
miR-3124-3p (and other miRNAs w/seed CUUUCCU)
miR-499-3p (and other miRNAs w/seed ACAUCAC)
miR-4708-3p (miRNAs w/seed GCAAGGC)
TEAD1
miR-1199-5p (and other miRNAs w/seed CUGAGCC)
SGPL1
miR-5583-5p (and other miRNAs w/seed AACUAAU)
miR-6513-5p (miRNAs w/seed UUGGGAU)
miR-4425 (miRNAs w/seed GUUGGGA)
miR-6724-5p (and other miRNAs w/seed UGGGCCC)
miR-190a-5p (and other miRNAs w/seed GAUAUGU)
CST5
miR-516b-5p (miRNAs w/seed UCUGGAG)
Cyp4a14
Tlr13
HS6ST1
FALEC
TAPBP
Duxbl1 (includes others)
ULBP1
COTI-2
vadimezan
latrunculin A
levofloxacin
monophosphoryl lipid A
amphotericin B
cetuximab
IFNL2
FOXG1
AZD8055
SMC3
CAPN3
BDKRB2
RGS1
RFX5
lauric acid
cobalt
miR-1896 (and other miRNAs w/seed GGUGGGU)
ITCH
hymecromone
miR-548av-3p (miRNAs w/seed AAACUGC)
miR-587 (miRNAs w/seed UUCCAUA)
ZBP1
TMBIM6
RARRES2
thiazolidinedione
idarubicin
vitamin K2
miR-6901-5p (miRNAs w/seed GCAGGAA)
miR-2117 (miRNAs w/seed GUUCUCU)
miR-140-3p (and other miRNAs w/seed ACCACAG)
miR-548au-3p (miRNAs w/seed GGCAGUU)
miR-3186-5p (miRNAs w/seed AGGCGUC)
miR-513b-3p (miRNAs w/seed AAUGUCA)
MAP4K4
miR-361-3p (miRNAs w/seed CCCCCAG)
miR-3171 (miRNAs w/seed GAUGUAU)
miR-3684 (miRNAs w/seed UAGACCU)
miR-6515-3p (miRNAs w/seed CUCUUCA)
miR-18a-3p (and other miRNAs w/seed CUGCCCU)
miR-410-5p (and other miRNAs w/seed GGUUGUC)
Mir122a,b
miR-325-5p (and other miRNAs w/seed CUAGUAG)
miR-450b-3p (and other miRNAs w/seed UUGGGGA)
miR-4297 (and other miRNAs w/seed GCCUUCC)
miR-3585-5p (and other miRNAs w/seed UCACAAG)
miR-4267 (miRNAs w/seed CCAGCUC)
miR-4421 (and other miRNAs w/seed CCUGUCU)
miR-4679 (miRNAs w/seed CUGUGAU)
miR-4501 (and other miRNAs w/seed AUGUGAC)
miR-122-3p (miRNAs w/seed ACGCCAU)
miR-4472 (and other miRNAs w/seed GUGGGGG)
miR-6810-5p (and other miRNAs w/seed UGGGGAC)
miR-3179 (miRNAs w/seed GAAGGGG)
miR-466 (miRNAs w/seed UACACAU)
PPP3R1
miR-6510-3p (miRNAs w/seed ACCGACU)
miR-4303 (miRNAs w/seed UCUGAGC)
miR-548ag (and other miRNAs w/seed AAGGUAA)
miR-4666a-3p (miRNAs w/seed AUACAAU)
CISH
UBE2I
miR-10394-5p (and other miRNAs w/seed CUGCAGG)
risperidone
vasoactive intestinal peptide
HOXA13
ETV1
oxazolone
SB 290157
miR-669d-5p (and other miRNAs w/seed CUUGUGU)
miR-133a-5p (miRNAs w/seed GCUGGUA)
miR-623 (miRNAs w/seed UCCCUUG)
miR-6808-5p (and other miRNAs w/seed AGGCAGG)
miR-497-3p (and other miRNAs w/seed CAAACCA)
miR-4314 (and other miRNAs w/seed UCUGGGA)
miR-4714-5p (and other miRNAs w/seed ACUCUGA)
MEX3A
KRT14
miR-431-5p (and other miRNAs w/seed GUCUUGC)
miR-561-3p (miRNAs w/seed AAAGUUU)
miR-1245a (and other miRNAs w/seed AGUGAUC)
miR-571 (miRNAs w/seed GAGUUGG)
EWSR1-FLI1
miR-3688-5p (miRNAs w/seed GUGGCAA)
FLZ
ALOX12
CTNNBIP1
PIK3R2
CLCA2
OGG1
KRIT1
miR-5112 (and other miRNAs w/seed AGCUCAG)
bezafibrate
miR-4649-5p (and other miRNAs w/seed GGGCGAG)
miR-625-5p (and other miRNAs w/seed GGGGGAA)
dacinostat
CDK1
mir-192
STAU1
Usp17la (includes others)
ammonium chloride
elovanoid N32
lopinavir
PDK
MLN8054
C1QTNF1
SPON2
EDEM1
SPATA2
satratoxin G
DHX15
GNLY
CRT-0066101
RAB10
IL36RN
SNAPIN
HES6
DHCR7
EXOC7
PTPN12
HERC5
iso[4]levuglandin E2
14-oxoDHA

Aldose Reductase
Collagen type IV
SP100
GDF15
SMARCE1
A-Fos
PCDH11Y
PD 169316
2-mercaptoacetate
PD 153035
acetaldehyde
IL15
RARA
NCOR1
26s Proteasome
SMAD7
MYCN
IGFBP2
ADM
STK11
IL5
miR-4302 (miRNAs w/seed CAGUGUG)
L-methionine
NKX3-1
salirasib
TEAD4
clofibrate
NFE2L2
MAPK12
PTAFR
imiquimod
IL10
CD3
oblimersen
CAT
SFRP1
IL2RG
colchicine
benzo(a)pyrene
alitretinoin
phosphate
TCR
MKNK1
miR-34a-3p (miRNAs w/seed AAUCAGC)
PTPRJ
PTPN6
LEF1
trans-cinnamaldehyde
Pkg
Fibrin
NADPH oxidase
CTR9
HTATIP2
CX-5461
PD184352
pifithrin alpha
lipoarabinomannan
diallyl trisulfide
STAT4
miR-7110-3p (miRNAs w/seed CUCUCUC)
Nfat (family)
hexachlorobenzene
SERPINF1
GNRH1
TNFRSF1B
RXRA
EGR2
sGC
KIDINS220
Muc1
scoparone
butylated hydroxytoluene
TCF12
trichloroethylene
benzene
isoproterenol
Ikb
NFAT (complex)
PDGF-AA
RNF20
MAP3K3
THZ2
diosgenin
sirolimus
UCP1
miR-188-3p (miRNAs w/seed UCCCACA)
PTK2
propylthiouracil
fatty acid
adenosine
IL1R1
diphenyleneiodonium
phenylbutazone
thioctic acid
BIRC3
plumbagin
SLPI
BCL2L11
TWIST1
NR1I3
mir-181
N-acetylmuramyl-L-alanyl-D-isoglutamine
NfkB1-RelA
CHI3L1
CDC73
XIAP
KAT2A
ACE2
HMGB2
PRKAA2
miR-5003-5p (miRNAs w/seed CACAACA)
ETV6-RUNX1
PROCR
DTX1
WR 1065
miR-4269 (and other miRNAs w/seed CAGGCAC)
3-deazaneplanocin
F2RL1
DDX5
vanillyl-N-nonylamide
LINC00842
ghrelin
PTPRR
CARD14
F2RL3
YWHAB
HIVEP2
famotidine
dibenzoylmethane
catechol
apamin
TGFB3
BMP7
BSCL2
NRG2
SNAI2
E2F3
choline fenofibrate
REN
CCR1
GRN
imipramine
arsenic
MYOD1
diphtheria toxin
Tlr
TGFB2
5-hydroxytryptamine
azoxymethane
HOXA9
HOXB13
S100A6
EPOR
NPPA
stearic acid
MRTFA
KLF2
GnRH analog
caffeine
HNF1B
miR-6826-3p (miRNAs w/seed UCCCCUC)
CCND1
MSTN
OGA
miR-1911-5p (miRNAs w/seed GAGUACC)
KITLG
GC-GCR dimer
leucine
C1QA
SFRP4
urethane
patulin
sphingomyelinase
AGR2
DAB2
MAD2L2
MED13L
AP20187

LDL-cholesterol
GSK690693
PXN
PRKACB
TFPI
PRKD2
CSRP3
BAD
OPRK1
RBBP4
CLDN1
Pzp
GNB5
mir-637
SNIP1
Nisch
TRPC3
SIX3
OXSR1
TDG
GPS1
ECT2
TFF2
BARD1
MTUS1
SRGN
OMP-52M51
equol
enzastaurin
2-morpholin-4-yl-6-thianthren-1-yl-pyran-4-one
halothane
eprosartan
juglone
benz[a]anthracene
ruboxistaurin
curcuminoids
EPZ005687
GSI34
PDZ1i
Z-VEID-FMK
trigonelline
edelfosine
remifentanil
3',4',7-trihydroxyisoflavone
8-chlorophenylthio-adenosine 3',5'-cyclic monophosphate
miR-3187-3p (miRNAs w/seed UGGCCAU)
miR-489-5p (miRNAs w/seed GUCGUAU)
RTN4
CD274
GRB2
EPHB4
3-nitropropionic acid
miR-5088-3p (miRNAs w/seed CCCUUCU)
CD5
miR-1292-3p (miRNAs w/seed CGCGCCC)
L-proline
GPRC6A
olaparib
JDP2
penfluridol
HNRNPR
PRKG2
DVL1
CCN4
BANP
WNK1
PHIP
SNCB
NUP107
EGR4
DLC1
BANCR
GNAO1
P2RY4
TBXA2R
KHDRBS1
TP53INP1
FKBP1A
OXTR
FKBP5
CUL1
PDK1
DMTF1
SHCBP1
PGK1
CAPNS1
GLP1R
NPAS2
FUBP1
H4C3
TNIK
VGF
N-methyl mesoporphyrin IX
Gm26313
anethole
CHS-828
bergamottin
ferric chloride
phytanic acid
non-esterified fatty acid
acolbifene
miR-614 (miRNAs w/seed AACGCCU)
Pln
ELL2
telmisartan
miR-4474-5p (miRNAs w/seed UAGUCUC)
15-epi-lipoxin A4
L-phenylalanine
ganglioside GM3
N-Cadherin
meldonium
rhodamine 6G
2,5-bis(5-hydroxymethyl-2-thienyl)furan
citrinin
Cyclin A
tolfenamic acid
TFIIA
DBH-AS1
UCA1
LINC00461
Retinoic acid-RAR-RXR
MRE11
1,3-bis(4-hydroxyphenyl)-4-methyl-5-[4-(2-piperidinylethoxy)phenol]-1H-pyrazole
VTCN1
CYTOR
DUOXA1
ZNF382
LTBP4
CYSLTR2
IL-2R
Lefty
PDS5B
STAG1
parathyroid hormone-related protein
hydrogel
HDAC10
evofosfamide
BAG6
HNRNPC
SLC25A4
MAP3K12
PLCD1
CXCR1
mir-326
SMARCA1
RGS19
GAL3ST1
CYSLTR1
CYP4A11
DLG4
KSR1
USP2
MAOA
MFGE8
DES
APCS
DGKZ
RBM38
CSNK2A2
PLP2
LASP1
RASSF10
GRB10
TBL1X
NEU3
AQP7
PIWIL2
RGS16
TSPO
SULF1
CCDC80
ACTA2
CCL7
H3C6
VIPR2
ethyl 2-[[(6,7-dimethyl-3-oxo-1,2,3,4-tetrahydro-2-quinoxalinyl)acetyl]amino}-4,5-dimethyl-3-thiophenecarboxylate
GADD45GIP1
TBL1XR1
SKF-83959
PX-12
trimethyloxamine
gabexate
LMK-235
magnesium chloride
methoxyluteolin
5-phenylvaleric acid
titanium
pegylated leptin antagonist
miR-377-5p (and other miRNAs w/seed GAGGUUG)
miR-6795-3p (miRNAs w/seed CCCCUCG)
miR-6882-3p (miRNAs w/seed GCUGCCU)
epothilone B
fluoride
DPH5
PFKFB3
ZNF148
EPHX2
CDK6
TNFRSF12A
VIPR1
IRAK2
101.10 peptide
cystamine
erythromycin
miR-2277-3p (miRNAs w/seed GACAGCG)
ATG5
B2M
SND1
dicarbethoxydihydrocollidine
deoxynivalenol
carbenoxolone
miR-6857-5p (miRNAs w/seed UGGGGAU)
miR-6751-5p (and other miRNAs w/seed UGGGGGU)
miR-5582-5p (miRNAs w/seed AGGCACA)
miR-629-3p (miRNAs w/seed UUCUCCC)
miR-551b-5p (miRNAs w/seed AAAUCAA)
miR-6835-5p (and other miRNAs w/seed GGGGGUA)
miR-122b-5p (miRNAs w/seed UUAGUGU)
miR-4471 (and other miRNAs w/seed GGGAACU)
miR-4642 (miRNAs w/seed UGGCAUC)
miR-6134 (miRNAs w/seed GAGGUGG)
miR-5092 (miRNAs w/seed AUCCACG)
LMNB1
miR-452-5p (and other miRNAs w/seed ACUGUUU)
DNMT3B
TYK2
KMT2D
TCF7L2
SERPINE2
discodermolide
sucrose
miR-4776-5p (miRNAs w/seed UGGACCA)
miR-6783-5p (miRNAs w/seed AGGGGAA)
miR-6888-3p (and other miRNAs w/seed UCUGUCU)
miR-1915-3p (and other miRNAs w/seed CCCAGGG)
mir-214
miR-1291 (and other miRNAs w/seed GGCCCUG)
miR-540-3p (and other miRNAs w/seed GGUCAGA)
miR-3936 (miRNAs w/seed AAGGGGU)
miR-3175 (miRNAs w/seed GGGGAGA)
miR-744-3p (miRNAs w/seed UGUUGCC)
miR-6742-3p (miRNAs w/seed CCUGGGU)
miR-518c-5p (miRNAs w/seed CUCUGGA)
miR-602 (miRNAs w/seed ACACGGG)
miR-1184 (miRNAs w/seed CUGCAGC)
miR-520a-5p (and other miRNAs w/seed UCCAGAG)
FFAR3
miR-3611 (miRNAs w/seed UGUGAAG)
miR-600 (miRNAs w/seed CUUACAG)
CpG ODN 1585
cytidylyl-3'-5'-guanosine
SLC16A3
salmonella typhimurium lipopolysaccharide
pictilisib
PRTN3
NEK6
1,1-bis(3'-indolyl)-1-(4-hydroxyphenyl)methane
cyclic guanosine monophosphate-adenosine monophosphate
raltitrexed
calcium chloride
arachidic acid
miR-370-3p (and other miRNAs w/seed CCUGCUG)
miR-7155-5p (miRNAs w/seed CUGGGGU)
miR-6813-3p (miRNAs w/seed ACCUUGG)
FLT3LG
miR-3664-3p (miRNAs w/seed CUCAGGA)
miR-1260a (and other miRNAs w/seed UCCCACC)
miR-10400-3p (and other miRNAs w/seed UGGGCUC)
VEGFB
miR-1301-3p (and other miRNAs w/seed UGCAGCU)
miR-4540 (miRNAs w/seed UAGUCCU)
miR-5004-3p (miRNAs w/seed UUGGAUU)
miR-147 (and other miRNAs w/seed UGUGCGG)
miR-10a-5p (and other miRNAs w/seed ACCCUGU)
miR-3065-3p (miRNAs w/seed CAGCACC)
miR-4666b (miRNAs w/seed UGCAUGU)
mir-135
GSKJ4
miR-873-5p (and other miRNAs w/seed CAGGAAC)
SIGIRR
PARG
IDO1
RNASE1
CCL4
ADORA3
tranilast
elovanoid N34
butaprost
miR-3945 (miRNAs w/seed GGGCAUA)
miR-3622b-5p (miRNAs w/seed GGCAUGG)
fluocinolone acetonide
fish oils
KAT2B
miR-4759 (miRNAs w/seed AGGACUA)
miR-2052 (miRNAs w/seed GUUUUGA)
FOXA1
miR-101b-3p (miRNAs w/seed UACAGUA)
miR-4480 (miRNAs w/seed GCCAAGU)
pexidartinib
miR-6895-3p (miRNAs w/seed GUCUCUC)
miR-3177-3p (miRNAs w/seed GCACGGC)
let-7a-2-3p (and other miRNAs w/seed UGUACAG)
nicotinic acid
RAC2
miR-7151-3p (miRNAs w/seed UACAGGC)
miR-6078 (miRNAs w/seed CGCCUGA)
miR-943 (miRNAs w/seed UGACUGU)
miR-3925-3p (miRNAs w/seed CUCCAGU)
psychosine
3'-O-(4-benzoyl)benzoyladenosine 5'-triphosphate
(R)-limonene
LRRC26
RAB7B
CTSC
TLR10
TPR
CASP4
resolvin D5
PPM1L
PIP5K1B
GJA8
glaucocalyxin A
mito-TEMPO
KPNA2
bepafant
GABA receptor
ZFA-fmk
p85 (pik3r)
IL-17f dimer
mir-7
mir-205
IFIT2
HDC
IL19
nimesulide
rofecoxib
clenbuterol
miR-1273h-3p (miRNAs w/seed UGCAGAC)
azithromycin
AKT inhibitor VIII
BPIFA1
LILRA2
(±)-2-hydroxyoleic acid
PLA2G2D
IgG2a
MBTPS1
TRPV1
mir-127
TIA1
PEMT
PLA2G2E
BHLHE41
CCL3L3
HPSE
iodoacetic acid
cantharidin
nicorandil
gliotoxin
butylidenephthalide
diazepam
manidipine
miR-508-3p inhibitor
cyclosporin
RIPK2
JAK1
SB-431542
CLOCK
PELP1
ITGB3
entinostat
VAV1
PTX3
ACKR3
ramipril
proteasome inhibitor PSI
vinblastine
lithium chloride
STAT5a/b
Fc gamma receptor
EIF2AK4
UXT
irbesartan
KN 93
RNF31
KLF11
GJA1
ZBTB7A
SSRP1
PDPK1
VTN
SMARCA5
Vhl
L-type Calcium Channel
LPA
SERPINH1
R59949
tosyllysine chloromethyl ketone
PITX2
filgrastim
daporinad
CDK5
bryostatin 1
CD28
dinoprost
MAP2K6
RELB
captopril
GNA12
GCG
1-methyl-4-phenyl-1,2,3,6-tetrahydropyridine
ING1
diclofenac
sodium orthovanadate
SN-38
BCR (complex)
NR4A1
MDM2
VHL
N-Ac-Leu-Leu-norleucinal
CRNDE
arginine
EIF4EBP1
NLRP12
GAPDH
IGF1R
ONECUT1
L-cysteine
Agtr1b
C8
BLVRA
Ferritin
CD244
GLRX
PSMB9
ATG16L1
ING4
CD160
SCUBE3
MCAM
C7
DEK
asbestos
capsazepine
RP 73401
JMF3086
I-BOP
miR-133a-3p (and other miRNAs w/seed UUGGUCC)
ZEB1
fingolimod
miR-126a-3p (and other miRNAs w/seed CGUACCG)
FOXM1
mibolerone
KDM1A
miR-181a-5p (and other miRNAs w/seed ACAUUCA)
PLP1
CTF1
TANK
FOSB
ELF4
histone deacetylase inhibitor
IL12 (complex)
NFKB2
ascorbic acid
CD44
PPARA
TCF3
GMNN
miR-4688 (and other miRNAs w/seed AGGGGCA)
STAT
SERPINE1
FGFR3
TLR5
PAK2
mevalonic acid
tempol
ethylene glycol tetraacetic acid
thalidomide
SPIB
CFTR
ERN1
ID2
CDH1
miR-141-3p (and other miRNAs w/seed AACACUG)
NSUN6
FCGR2A
MEOX2
PRKN
sorafenib
CGS 21680
parthenolide
ACOX1
LINC01139
FAT1
cercosporin
AMBP
NCK1
HCRT
IL9R
SETD7
NPAT
N4BP1
KLB
diltiazem
calmidazolium
tiron
SU1498
miR-495-3p (and other miRNAs w/seed AACAAAC)
lithium
homocysteine
IL27
ciprofibrate
miR-5579-3p (miRNAs w/seed UAGCUUA)
miR-27a-3p (and other miRNAs w/seed UCACAGU)
NOX4
pimagedine
haloperidol
MAPT
TFRC
tazemetostat
PGF
HSPA9
THBS1
4-methylnitrosoamino-1-(3-pyridinyl)-1-butanone
valproic acid
MAX
cinnamaldehyde
let-7
miR-3613-3p (miRNAs w/seed CAAAAAA)
miR-4446-5p (miRNAs w/seed UUUCCCU)
PBRM1
G2535
RLIM
ENTPD1
CNB-001

proteasome inhibitor
intralipid
naproxen
BAY 61-3606
itraconazole
NCX-4040
coumestrol
pranlukast
myxothiazol
vandetanib
citalopram
venlafaxine
carnosol
5-nitro-2-(3-phenylpropylamino)benzoic acid
N-methyl-3,4-methylenedioxyamphetamine
tyrphostin
dehydrocostus lactone
dihematoporphyrin ether
pyrogallol
miR-4757-3p (miRNAs w/seed AUGACGU)
miR-3124-5p (miRNAs w/seed UCGCGGG)
miR-7158-5p (miRNAs w/seed GCUCAAU)
miR-4781-3p (miRNAs w/seed AUGUUGG)
miR-6824-5p (miRNAs w/seed UAGGGGA)
miR-3176 (and other miRNAs w/seed CUGGCCU)
miR-187-3p (miRNAs w/seed CGUGUCU)
miR-1893 (and other miRNAs w/seed GCGCGGG)
Smad2/3
E2F6
miR-12202-3p (and other miRNAs w/seed CUUCUCU)
geneticin
cholesterol sulfate
ganglioside GD3
BMS-387032
CRTC3
AFF4
TACSTD2
CPEB4
KDM4B
LRRC32
nicotiflorin
PRRX1
AGTRAP
AKAP12
KHSRP
PDGF (family)
DLX1
FGF3
ZIC2
AGRP
MED15
HGS
DKK2
Ccl6
GUSB
PRKCG
salinomycin
genipin
WNT5B
PTGES3
UBQLN1
CDKN2B
CCND2
MSH2
RPL11
DIAPH1
EIF3A
USP37
PINK1
HP
RECK
muscimol
7,8-dihydro-7,8-dihydroxybenzo(a)pyrene 9,10-oxide
miR-182 inhibitor
iodine
oleoylethanolamide
5-iodotubercidin
lead acetate

adefovir dipivoxil
miR-6732-5p (and other miRNAs w/seed AGGGGGU)
miR-6075 (miRNAs w/seed CGGCCCA)
miR-634 (miRNAs w/seed ACCAGCA)
miR-1287-5p (miRNAs w/seed GCUGGAU)
miR-6765-3p (miRNAs w/seed CACCUGG)
miR-148a-5p (and other miRNAs w/seed AAGUUCU)
miR-1207-3p (and other miRNAs w/seed CAGCUGG)
ACSL4
miR-4535 (miRNAs w/seed UGGACCU)
miR-5700 (miRNAs w/seed AAUGCAU)
miR-6507-3p (miRNAs w/seed AAAGUCC)
PYCARD
SGPP2
miR-3615 (miRNAs w/seed CUCUCGG)
miR-4433a-5p (miRNAs w/seed GUCCCAC)
miR-4292 (and other miRNAs w/seed CCCUGGG)
phenethyl isothiocyanate
miR-4698 (miRNAs w/seed CAAAAUG)
miR-5585-5p (miRNAs w/seed GAAGUAC)
miR-4254 (and other miRNAs w/seed CCUGGAG)
miR-6730-3p (miRNAs w/seed CUGACAC)
miR-203a-3p (and other miRNAs w/seed UGAAAUG)
miR-30c-1-3p (and other miRNAs w/seed UGGGAGA)
miR-6072 (and other miRNAs w/seed CCUCAUC)
miR-30a-3p (and other miRNAs w/seed UUUCAGU)
MYOCD
ESRRG
anakinra
STS
miR-7032-3p (and other miRNAs w/seed UCCUCUC)
miR-5589-3p (miRNAs w/seed GCACAUG)
miR-1911-3p (miRNAs w/seed ACCAGGC)
RORA
mir-10
miR-4485-3p (miRNAs w/seed AACGGCC)
miR-6823-5p (miRNAs w/seed CAGGGUU)
miR-4520-2-3p (miRNAs w/seed UUGGACA)
miR-3660 (and other miRNAs w/seed CUGACAG)
XRCC6
ITGA9
TLR6
apicidin
miR-18b-3p (miRNAs w/seed GCCCUAA)
miR-340-3p (and other miRNAs w/seed CCGUCUC)
miR-8075 (miRNAs w/seed GCUGAUG)
miR-6878-5p (miRNAs w/seed GGGAGAA)
ITK
miR-4435 (miRNAs w/seed UGGCCAG)
miR-6838-3p (miRNAs w/seed AGUCCUG)
ADRB2
miR-7108-3p (miRNAs w/seed CCCGCCC)
miR-150-5p (and other miRNAs w/seed CUCCCAA)
miR-4785 (miRNAs w/seed GAGUCGG)
ADAM17
miR-324-3p (and other miRNAs w/seed CACUGCC)
miR-1281 (miRNAs w/seed CGCCUCC)
miR-3130-5p (and other miRNAs w/seed ACCCAGU)
miR-4750-5p (miRNAs w/seed UCGGGCG)
miR-3939 (miRNAs w/seed ACGCGCA)
miR-5104b-5p (and other miRNAs w/seed GGGCCAU)
miR-190a-3p (miRNAs w/seed UAUAUAU)
EHMT1
miR-1537-3p (miRNAs w/seed AAACCGU)
miR-889-5p (miRNAs w/seed AUGGCUG)
miR-343 (and other miRNAs w/seed CUCCCUU)
miR-513a-5p (miRNAs w/seed UCACAGG)
miR-7705 (miRNAs w/seed AUAGCUC)
miR-548av-5p (and other miRNAs w/seed AAAGUAC)
CALR
JINK1/2
GAS6
miR-6866-5p (miRNAs w/seed UAGAGGC)
miR-451a (and other miRNAs w/seed AACCGUU)
miR-4433a-3p (miRNAs w/seed CAGGAGU)
KLK4
telapristone acetate
SOD3
DLK1
TACR1
GAB1
NCR2
antimycin A
batimastat
miR-4768-3p (and other miRNAs w/seed CAGGAGA)
miR-4690-3p (and other miRNAs w/seed CAGCCCA)
miR-624-3p (miRNAs w/seed ACAAGGU)
miR-3123 (miRNAs w/seed AGAGAAU)
POSTN
6-mercaptopurine
1L-6-hydroxymethyl-chiro-inositol 2-(R)-2-O-methyl-3-O-octadecylcarbonate
belinostat
Stat1-Stat2
F630028O10Rik
HOXA11-AS
PF-3084014
DCLK1
NCF4
SELE
PDE4B
HOXB5
HOXB7
GPC1
IGKC
CTTN
ATF5
butylhydroxybutylnitrosamine
farglitazar
largazole
tridecanoic acid
POU2AF1
BECN1
IKZF2
IL27RA
miR-4291 (and other miRNAs w/seed UCAGCAG)
miR-338-5p (miRNAs w/seed ACAAUAU)
miR-1964-5p (and other miRNAs w/seed GCUGGAG)
miR-4274 (miRNAs w/seed AGCAGUC)
miR-4764-3p (miRNAs w/seed UAACUCC)
miR-6511b-5p (and other miRNAs w/seed UGCAGGC)
ginsenoside Rb1
Ahr-aryl hydrocarbon-Arnt
MFN2
N(2)-(gamma-D-glutamyl)-meso-2,2'-diaminopimelic acid
TAC4
BMX
RALA
ADAM15
DMH1
clasto-lactacystin beta-lactone
hyperforin
D-tubocurarine
E64d
beta-1,3-glucan
auranofin
miR-3150b-3p (and other miRNAs w/seed GAGGAGA)
miR-4528 (miRNAs w/seed CAUUAUA)
miR-6802-5p (miRNAs w/seed UAGGUGG)
miR-3119 (miRNAs w/seed GGCUUUU)
miR-337-3p (and other miRNAs w/seed CAGCUCC)
miR-6865-3p (and other miRNAs w/seed CACCCUC)
miR-1279 (miRNAs w/seed CAUAUUG)
MMP12
miR-652-5p (miRNAs w/seed AACCCUA)
DNMT3A
miR-2276-3p (miRNAs w/seed CUGCAAG)
miR-1276 (miRNAs w/seed AAAGAGC)
miR-328-3p (and other miRNAs w/seed UGGCCCU)
miR-671-5p (miRNAs w/seed GGAAGCC)
miR-3682-5p (miRNAs w/seed UACUUCU)
NR4A2
miR-549a-3p (miRNAs w/seed GACAACU)
OLR1
miR-6840-3p (miRNAs w/seed CCCAGGA)
ALDH2
SULT1E1
TUG1
DL-fructose
miR-544-5p (miRNAs w/seed CUUGUUA)
miR-6717-5p (miRNAs w/seed GGCGAUG)
miR-7064-3p (and other miRNAs w/seed AGGGCCC)
miR-4512 (miRNAs w/seed AGGGCCU)
miR-4293 (miRNAs w/seed AGCCUGA)
miR-593-3p (miRNAs w/seed GUCUCUG)
miR-30b-3p (and other miRNAs w/seed UGGGAUG)
miR-3678-5p (miRNAs w/seed CCGUACA)
miR-6800-5p (miRNAs w/seed UAGGUGA)
miR-6791-3p (and other miRNAs w/seed GCCUCCU)
miR-4659a-5p (and other miRNAs w/seed UGCCAUG)
miR-4708-5p (miRNAs w/seed GAGAUGC)
miR-374c-3p (miRNAs w/seed ACUUAGC)
miR-7848-3p (miRNAs w/seed UACCCUC)
miR-3072 (and other miRNAs w/seed GCCCCCU)
collagen type i (family)
miR-888-3p (miRNAs w/seed ACUGACA)
miR-3925-5p (miRNAs w/seed AGAGAAC)
KDM2B
IFT88

Ncoa6
tyrphostin AG 1296
ATF1
chloropromazine
S-nitrosoglutathione
GDF2
BCR-ABL1
miR-709 (and other miRNAs w/seed GAGGCAG)
AGN194204
S100A4
HBB
SRSF2
retinoid
4-hydroxynonenal
mir-30 (includes others)
ouabain
17-alpha-ethinylestradiol
TGM2
diethylstilbestrol
SP2509
miR-3614-3p (miRNAs w/seed AGCCUUC)
ANGPT1
CDK2
miR-19b-3p (and other miRNAs w/seed GUGCAAA)
DEPTOR
CST3
UBA1
SH3KBP1
mir-379
C9
bisphenol A diglycidyl ether
D-sphingosine
putrescine
miR-3679-3p (miRNAs w/seed UUCCCCC)
miR-3691-5p (miRNAs w/seed GUGGAUG)
HBEGF
TXNIP
TBK1
Ifn gamma
D-fructose
streptozocin
miR-1268a (and other miRNAs w/seed GGGCGUG)
miR-3156-3p (miRNAs w/seed UCCCACU)
miR-204-5p (and other miRNAs w/seed UCCCUUU)
TO-901317
eicosapentenoic acid
SMAD1
vincristine
mono-(2-ethylhexyl)phthalate
desoxycorticosterone
Pdgfr
trypsin
harmine
CRY2
SDC1
PTGER1
CRY1
HSPA1A/HSPA1B
1,2-dimethylhydrazine
hydroquinone
N,N-dimethylsphingosine
verteporfin
miR-202-5p (miRNAs w/seed UCCUAUG)
miR-4476 (and other miRNAs w/seed AGGAAGG)
PPRC1
zVAD-FMK
RPTOR
N-cor
SCARB1
EGR3
MYBL2
AIP
MRTFB
lenalidomide
NR1H2
xanthine
glycochenodeoxycholate
Rac
IDH2
PSMB10
F10
SRSF3
TRAF4
ST14
ebselen
dimethyl fumarate
pyruvic acid
salmeterol
nickel chloride
anti-benzo(a)pyrene-diol-epoxide
buthionine sulfoximine
CD86
PRKD1
miR-30c-5p (and other miRNAs w/seed GUAAACA)
fluticasone propionate
IFN Beta
SMARCB1
TSC22D3
IKZF1
NFATC1
RARB
SRC (family)
IDH1
CCN5
TNFSF12
cyclic GMP
ADP
Nos
IGFBP7
PCSK9
AIMP1
PNN
HOXB4
GNA13
EDNRA
TOPBP1
Mn(III)-tetrakis-(4-benzoic acid) porphyrin
24-hydroxycholesterol
uric acid
miR-512-3p (miRNAs w/seed AGUGCUG)
GHR
FOXO4
KAT5
plicamycin
PRDM1
EIF2S1
PTPN1
iron
MAPK10
CXCL2
PRDX2
MAML1
BCL10
RHOB
4-nitroquinoline-1-oxide
concanamycin A
farnesyl pyrophosphate
reserpine
mannose
HDL
DCN
carboplatin
SYK
GNA15
miR-4716-3p (and other miRNAs w/seed AGGGGGA)
WWTR1
EBI3
miR-1-3p (and other miRNAs w/seed GGAAUGU)
miR-3157-5p (miRNAs w/seed UCAGCCA)
NFkB (family)
Smad2/3-Smad4
PSMB8
PPP2R5C
GPI
MAZ
DAXX
KLK5
premarin
3-aminobenzamide
nimodipine
zearalenone
losartan potassium
PAX5
BTK
PIM1
CD14
CCL11
IFNA1/IFNA13
miR-585-5p (miRNAs w/seed UAGCACA)
miR-3934-3p (miRNAs w/seed GCUCAGG)
miR-4765 (miRNAs w/seed GAGUGAU)
CBX5
emodin
heme
15(S)-HETE
glycine
mannitol
Pdgf Ab

miR-509-3p (miRNAs w/seed GAUUGGU)
miR-4787-5p (and other miRNAs w/seed CGGGGGU)
miR-4744 (miRNAs w/seed CUAAAGA)
LRP5
PDCD1
miR-2114-3p (and other miRNAs w/seed GAGCCUC)
miR-3922-5p (miRNAs w/seed CAAGGCC)
GPD1
miR-4300 (and other miRNAs w/seed GGGAGCU)
miR-185-5p (and other miRNAs w/seed GGAGAGA)
miR-760-3p (and other miRNAs w/seed GGCUCUG)
miR-4794 (and other miRNAs w/seed CUGGCUA)
miR-4749-3p (miRNAs w/seed GCCCCUC)
miR-4318 (miRNAs w/seed ACUGUGG)
artesunic acid
SLC29A1
ITGB4
HNRNPU
CLEC4E
IL1RL1
NRTN
CEP-1347
triflusal
pirfenidone
CpdX
triolein
sesamol
Tlr11
PDCD10
USP25
TRIM29
TP73-AS1
FNIP1
ZC3HAV1
APC (complex)
tofacitinib
SERPINC1
HMGCR
mir-320
PIK3C3
LPAR2
PPM1A
PLA2G2A
USP21
HLA-B
BP-1-102
benzoquinone
lemairamin
ISRIB
eugenol
dBET6
miR-545-5p (miRNAs w/seed CAGUAAA)
miR-1296-3p (and other miRNAs w/seed AGUGGGG)
miR-218-2-3p (and other miRNAs w/seed AUGGUUC)
RICTOR
miR-3120 (and other miRNAs w/seed ACAGCAA)
miR-3690 (miRNAs w/seed CCUGGAC)
miR-6881-3p (miRNAs w/seed UCCUCUU)
miR-1-5p (and other miRNAs w/seed CAUACUU)
miR-5189-3p (and other miRNAs w/seed GCCAACC)
miR-136-5p (miRNAs w/seed CUCCAUU)
miR-4477b (miRNAs w/seed UUAAGGA)
PTP4A1
miR-383-5p (miRNAs w/seed AGAUCAG)
TXNRD1
mir-210
miR-3618 (miRNAs w/seed GUCUACA)
miR-3912-5p (miRNAs w/seed UGUCCAU)
miR-5580-5p (and other miRNAs w/seed GCUGGCU)
miR-485-3p (and other miRNAs w/seed UCAUACA)
miR-3141 (miRNAs w/seed AGGGCGG)
miR-548ao-5p (and other miRNAs w/seed GAAGUAA)
miR-3605-5p (and other miRNAs w/seed GAGGAUG)
miR-1202 (and other miRNAs w/seed UGCCAGC)
miR-4515 (miRNAs w/seed GGACUGG)
LINC00963
USP19
1-methyl-4-phenylpyridinium
3-D-glucosyl-1,2-diacylglycerol
behenic acid
treprostinil
ITPR
chitinase
nebivolol
CACTIN
PFKFB4
ZXDC
ROBO3
TNIP3
USP28
USE1
ATP6V0D2
ABRAXAS2
B3GNT2
ZFP64
RNF122
EHD2
CUEDC2
LRRC19
Eif2
P-TEFb
nintedanib
danusertib
cerotic acid
crocin
ORM1
MEP1A
BAG2
IL17B
A4GALT
CD163
RNF139
APOD
NLRC3
PLAGL2
Rps6ka5
SKLB023
VSNL1
CSTB
FLLL-32
NEK2
PPP1CC
HS6ST2
CAY10397
N-hydroxy-2,2-diphenylacetamide
(+)-epicatechin
1-(2-hydroxy-5-methylphenyl)-3-phenyl-1,3-propanedione
ITF3056
methylamine
PTIO
pegintron
dihydrosphingosine 1-phosphate
MCC950
ethyl linoleate
MR-409
AC083837.1
miR-499a-5p inhibitor
miR-208b inhibitor
galanthamine
CX-6258
oxamic acid
Vi capsular polysaccharide
thymeleatoxin
CRKL
SPHK2
TNFAIP2
GABBR1
CRTC1
Z-551
N-ethyl-N-nitrosourea
amlodipine
asiatic acid
TNFRSF9
miR-3093-3p (and other miRNAs w/seed GUGGACA)
miR-1266-5p (and other miRNAs w/seed CUCAGGG)
miR-4693-3p (miRNAs w/seed GAGAGUG)
miR-6821-3p (and other miRNAs w/seed GACCUCU)
miR-374b-3p (miRNAs w/seed UUAGCAG)
CHADL
NFIL3
miR-5681b (miRNAs w/seed GGUAUUG)
tannic acid
ZFAS1
HOXA-AS2
C9orf72
SARM1
SLC7A5
MED14
Ifnz (includes others)
sevoflurane
2-aminoethoxydiphenylborane
phospholipid
theophylline
canrenoate potassium
miR-1277-3p (and other miRNAs w/seed ACGUAGA)
GRIN3A
CCR2
miR-4530 (miRNAs w/seed CCAGCAG)

PLC
PKNOX2
PI-103
PADI2
LYL1
DEF6
HOTAIRM1
PDGFRA
PLA2G4A
CXCL3
MAPKAPK3
miR-4645-3p (miRNAs w/seed GACAGUA)
dextran sulfate
1,4-bis[2-(3,5-dichloropyridyloxy)]benzene
TLR2
miR-6729-3p (miRNAs w/seed CAUCCCC)
AMPK
POU2F1
miR-6861-3p (miRNAs w/seed GGACCUC)
sunitinib
IL11
clozapine
ARNT
miR-6752-5p (and other miRNAs w/seed GGGGGUG)
Srebp
trastuzumab
TMSB4
BAP1
diethylmaleate
helenalin
dichlororibofuranosylbenzimidazole
SPI1
carbon monoxide
PSMB11
miR-517a-3p (and other miRNAs w/seed UCGUGCA)
miR-148b-5p (and other miRNAs w/seed AGUUCUG)
miR-3168 (miRNAs w/seed AGUUCUA)
3M-002
ID3
Fcer1
HOXD10
USF2
N-methyl-D-aspartate
glucosamine
MIR17HG
E. coli B4 lipopolysaccharide
puromycin
MPZ
CDK5R1
IAPP
PIM2
LY6E
DUSP5
PTK2B
NAB2
pevonedistat
AS1842856
NCD-38
(1S,2R)-NCL-1
salvin
miR-148a-3p (and other miRNAs w/seed CAGUGCA)
miR-6867-3p (and other miRNAs w/seed UCUCCCU)
miR-5107-3p (and other miRNAs w/seed AACCUGU)
geldanamycin
PRMT1
sulindac
miR-5131 (and other miRNAs w/seed GGCGCCC)
RUNX3
MECP2
miR-520d-5p (and other miRNAs w/seed UACAAAG)
hydroxyurea
Stat5 dimer
trametinib
MED12
SPZ1
ACKR1
CYP2J2
PARK7
W7
pilocarpine
nickel
IRF3
dimethyl sulfoxide
miR-519a-3p (and other miRNAs w/seed AAGUGCA)
GATA1
WNT3A
vitamin E
CIP2A
cytarabine
WNT5A
IFNB1
miR-4684-3p (miRNAs w/seed GUUGCAA)
miR-8064 (miRNAs w/seed GCACACU)
miR-4493 (miRNAs w/seed GAAGGCC)
TNFSF13B
CaMKII
EIF4G2
CCK
GADD45A
PTPN3
ketamine
GLI1
MYRF
EFNA1
gemcitabine
miR-12185-3p (and other miRNAs w/seed CAGCCAC)
APC
miR-145-5p (and other miRNAs w/seed UCCAGUU)
clopidogrel
SUZ12
miR-4671-5p (miRNAs w/seed CCGAAGA)
zidovudine
pregna-4,17-diene-3,16-dione
FAM3B
Pde4
TF
TNFAIP6
Sch-23390
epicatechin gallate
9Z,11E-octadecadienoic acid
NFATC3
PKM
MAP2K3
IL22
CASR
miR-2355-5p (miRNAs w/seed UCCCCAG)
miR-605-3p (miRNAs w/seed GAAGGCA)
miR-3190-5p (miRNAs w/seed CUGGCCA)
miR-7854-3p (miRNAs w/seed GAGGUGA)
miR-6894-3p (miRNAs w/seed UGCCUGC)
ADCY
JUN/JUNB/JUND
UCN
WNT7A
SAFB2
NTRK1
ACE
THZ1
SOX11
molybdenum disulfide
PIK3CA
HSF1
miR-4752 (miRNAs w/seed UGUGGAU)
Tcf7
ritonavir
miR-130a-3p (and other miRNAs w/seed AGUGCAA)
leukotriene B4
calpain
Smad
E2F5
SOX6
LRPAP1
UBE3A
HEXIM1
COMMD1
TRPV4
PCYT1A
UCN-01
miR-450b-5p (miRNAs w/seed UUUGCAA)
minocycline
nifedipine
miR-200a-5p (and other miRNAs w/seed AUCUUAC)
corticosterone
CNR1
DCAF1
OXT
MST1
CXCL10
RCAN1
MTA3
SUPT16H
NOS1
HSPD1
K-252
calyculin A
arotinoid acid
RBL1
UCHL1

| | |
|---|---|
| cytochalasin D | FGF7 |
| miR-630 (miRNAs w/seed GUAUUCU) | ROR2 |
| miR-488-5p (miRNAs w/seed CCAGAUA) | Bay 11-7082 |
| SLC25A13 | miR-4504 (miRNAs w/seed GUGACAA) |
| miR-6734-3p (miRNAs w/seed CCUUCCC) | miR-532-5p (and other miRNAs w/seed AUGCCUU) |
| miR-941 (miRNAs w/seed ACCCGGC) | HDAC5 |
| miR-3160-5p (miRNAs w/seed GCUUUCU) | TBX5 |
| miR-22-5p (and other miRNAs w/seed GUUCUUC) | DYSF |
| miR-590-3p (miRNAs w/seed AAUUUUA) | picrotoxin |
| GHRL | UTP |
| miR-6976-5p (and other miRNAs w/seed AGGGAAG) | p70 S6k |
| miR-4701-5p (and other miRNAs w/seed UGGCCAC) | AMH |
| miR-6799-5p (miRNAs w/seed GGGAGGU) | vemurafenib |
| miR-6802-3p (miRNAs w/seed UCACCCC) | NCL |
| miR-3594-5p (and other miRNAs w/seed CCAGGGC) | mir-148 |
| miR-3191-3p (miRNAs w/seed GGGGACG) | BGN |
| CLEC11A | MAP2K2 |
| miR-3924 (miRNAs w/seed UAUGUAU) | miR-1255b-5p (and other miRNAs w/seed GGAUGAG) |
| miR-4714-3p (miRNAs w/seed CAACCUA) | miR-4433b-3p (miRNAs w/seed AGGAGUG) |
| miR-153-5p (miRNAs w/seed CAUUUUU) | NR1H3 |
| miR-7113-5p (and other miRNAs w/seed CCAGGGA) | RETNLB |
| miR-4786-3p (miRNAs w/seed GAAGCCA) | PIN1 |
| miR-6809-5p (miRNAs w/seed GGCAAGG) | SOX3 |
| miR-4270 (and other miRNAs w/seed CAGGGAG) | nelfinavir |
| CYB5R4 | miR-4503 (miRNAs w/seed UUAAGCA) |
| dactolisib | miR-3926 (miRNAs w/seed GGCCAAA) |
| TRAIP | miR-129-5p (and other miRNAs w/seed UUUUUGC) |
| mir-218 | miR-4301 (miRNAs w/seed CCCACUA) |
| MLX | JUND |
| DHX9 | cholesterol |
| LCP2 | NCF1 |
| RPA1 | ADA |
| biochanin A | TAF6 |
| leptomycin B | MALP-2s |
| betaine | phorbol 12,13-didecanoate |
| chitin | miR-5008-5p (miRNAs w/seed GAGGCCC) |
| miR-3584-5p (and other miRNAs w/seed GGAGGAG) | miR-8088 (miRNAs w/seed CUCGGUA) |
| miR-5010-3p (and other miRNAs w/seed UUUGUGU) | miR-3692-3p (miRNAs w/seed UUCCACA) |
| miR-196a-5p (and other miRNAs w/seed AGGUAGU) | miR-6888-5p (miRNAs w/seed AGGAGAU) |
| miR-4733-5p (miRNAs w/seed AUCCCAA) | miR-567 (miRNAs w/seed GUAUGUU) |
| USP22 | C3 |
| miR-591 (miRNAs w/seed GACCAUG) | FLT1 |
| miR-5699-5p (miRNAs w/seed GCCCCAA) | miR-34a-5p (and other miRNAs w/seed GGCAGUG) |
| miR-6875-3p (miRNAs w/seed UUCUUCC) | miR-6800-3p (miRNAs w/seed ACCUCUC) |
| miR-6505-3p (and other miRNAs w/seed GACUUCU) | PTGER4 |
| miR-4774-3p (miRNAs w/seed UUGCCUA) | chartreusin |
| miR-1282 (miRNAs w/seed CGUUUGC) | PLA2 |
| miR-877-3p (miRNAs w/seed CCUCUUC) | diosmetin |
| miR-3189-3p (miRNAs w/seed CCUUGGG) | DF 203 |
| miR-323-5p (and other miRNAs w/seed GGUGGUC) | NPS |
| miR-6788-3p (miRNAs w/seed UCGCCAC) | Rps15a-ps4 |
| PRKCQ | FAM168A |
| miR-4524a-3p (miRNAs w/seed GAGACAG) | TRIM71 |
| TNFRSF11B | MLXIP |
| BTRC | Gm-Csf Receptor |
| NMU | MAP3K21 |
| EGLN3 | dibromoacetic acid |
| miR-6882-5p (miRNAs w/seed ACAAGUC) | MAPK8IP2 |
| miR-154-3p (and other miRNAs w/seed AUCAUAC) | mir-331 |
| miR-4438 (miRNAs w/seed ACAGGCU) | mir-105 |
| plerixafor | CENPX |
| miR-4782-5p (and other miRNAs w/seed UCUGGAU) | RPS6KA2 |
| miR-504-3p (and other miRNAs w/seed GGGAGAG) | Hnrpfl |
| miR-6755-3p (miRNAs w/seed GUUGUCA) | ING2 |
| Brd4 | CRTAP |
| miR-6510-5p (miRNAs w/seed AGCAGGG) | Bhlhe41 |
| miR-4279 (miRNAs w/seed UCUCCUC) | RPS4Y1 |
| miR-3157-3p (miRNAs w/seed UGCCCUA) | ZFYVE16 |
| miR-7704 (miRNAs w/seed GGGGUCG) | verticillin A |
| miR-1250-3p (miRNAs w/seed CAUUUUC) | 2,2-(2-chlorophenyl-4'-chlorophenyl)-1,1-dichloroethene |
| budesonide | primaquine |
| miR-6873-5p (miRNAs w/seed AGAGGGA) | sarpogrelate |
| miR-3115 (miRNAs w/seed UAUGGGU) | methiothepin |
| miR-8070 (miRNAs w/seed UGUGAUU) | dibenzo(a,l)pyrene |
| miR-6803-3p (miRNAs w/seed CCCUCGC) | chalcone-24 |
| H1-6 | (S)-2-((6Z,15Z)-20- hydroxyicosa-6,15-dienamido)succinate |
| H1f1 | benzo(a)pyrene-7,8-dione |
| miR-574-5p (miRNAs w/seed GAGUGUG) | nisoldipine |
| miR-3097-5p (and other miRNAs w/seed ACAGGUG) | AGI-1095 |
| miR-218-5p (and other miRNAs w/seed UGUGCUU) | lavendustin A |
| miR-637 (and other miRNAs w/seed CUGGGGG) | indolo(3,2-b)carbazole |
| miR-3685 (miRNAs w/seed UUCCUAC) | kenpaullone |
| miR-577 (miRNAs w/seed AGAUAAA) | tyrphostin B56 |
| miR-6739-3p (miRNAs w/seed UUGUUCU) | tibolone |
| miR-4695-5p (and other miRNAs w/seed AGGAGGC) | digitoxin |
| miR-4427 (miRNAs w/seed CUGAAUA) | 1-phenazinecarboxylic acid |
| miR-682 (miRNAs w/seed UGCAGUC) | miR-493-5p (miRNAs w/seed UGUACAU) |
| resatorvid | miR-6887-3p (miRNAs w/seed CCCCUCC) |
| UNC93B1 | miR-6815-5p (and other miRNAs w/seed AGGUGGC) |
| GAS2L3 | taurocholic acid |
| MESP1 | crizotinib |
| PURA | SH3TC2 |
| GPNMB | ACTL6A |
| TGIF1 | MEL S3 |
| IGHE | CFLAR |
| STK3 | ketoconazole |
| IFT57 | hemozoin |
| 10-nitrooleate | miR-6516-3p (miRNAs w/seed UCAUGUA) |
| OSBPL8 | miR-512-5p (miRNAs w/seed ACUCAGC) |
| hydrogen sulfide | miR-6732-3p (miRNAs w/seed AACCCUG) |
| miR-3934-5p (miRNAs w/seed CAGGUGU) | 3,4,5,3',4'-pentachlorobiphenyl |
| miR-7159-5p (miRNAs w/seed UCAACAA) | AICAR |
| miR-938 (miRNAs w/seed GCCCUUA) | indican |
| miR-6767-5p (miRNAs w/seed CGCAGAC) | tosedostat |
| miR-653-3p (and other miRNAs w/seed UCACUGG) | RPSA |
| miR-663b (miRNAs w/seed GUGGCCC) | SIAH2 |
| miR-644a (miRNAs w/seed GUGUGGC) | PRKCI |
| miR-6812-3p (miRNAs w/seed CGCUCUU) | trabectedin |
| cardiotoxin | miR-3192-5p (miRNAs w/seed CUGGGAG) |
| miR-3191-5p (miRNAs w/seed UCUCUGG) | miR-6789-5p (miRNAs w/seed UAGGGGC) |
| miR-3975 (miRNAs w/seed GAGGCUA) | miR-3691-3p (miRNAs w/seed CCAAGUC) |
| TNIP1 | 10E,12Z-octadecadienoic acid |
| miR-6759-5p (miRNAs w/seed UGUGGGU) | miR-6748-3p (miRNAs w/seed CCUGUCC) |
| FANCC | prednisolone |
| miR-670-5p (miRNAs w/seed UCCCUGA) | miR-5571-5p (miRNAs w/seed AAUUCUC) |
| miR-8081 (miRNAs w/seed UUGAGUC) | FEV |
| miR-302c-5p (miRNAs w/seed UUAACAU) | UPF2 |
| MM-589 | MECOM |
| PTC-209 | KNG1 |
| miR-4711-3p (miRNAs w/seed GUGUCUU) | VAV2 |
| miR-3074-5p (miRNAs w/seed UUCCUGC) | miR-3146 (miRNAs w/seed AUGCUAG) |
| miR-501-5p (miRNAs w/seed AUCCUUU) | miR-6840-5p (miRNAs w/seed CCCCCGG) |
| miR-935 (miRNAs w/seed CAGUUAC) | NPC1 |
| miR-576-3p (miRNAs w/seed AGAUGUG) | miR-544-3p (and other miRNAs w/seed UUCUGCA) |
| miR-5689 (miRNAs w/seed GCAUACA) | miR-582-5p (miRNAs w/seed UACAGUU) |
| ARNT2 | miR-4648 (miRNAs w/seed GUGGGAC) |
| RGS2 | LCN2 |
| miR-4771 (miRNAs w/seed GCAGACU) | niacinamide |
| miR-526b-5p (miRNAs w/seed UCUUGAG) | deferasirox |
| miR-3117-5p (miRNAs w/seed GACACUA) | TIRAP |
| MXD1 | TNFSF14 |
| mir-122 | SMAD5 |
| miR-615-5p (and other miRNAs w/seed GGGGUCC) | linsidomine |
| miR-7845-5p (miRNAs w/seed AGGGACA) | miR-17-5p (and other miRNAs w/seed AAAGUGC) |
| miR-6864-5p (miRNAs w/seed UGAAGGG) | miR-6798-5p (miRNAs w/seed CAGGGGG) |
| miR-425-3p (miRNAs w/seed UCGGGAA) | 4-phenylbutyric acid |
| miR-24-1-5p (and other miRNAs w/seed GCCUACU) | hemin |
| SYK/ZAP | SREBF1 |
| Klrk1 | miR-4700-3p (miRNAs w/seed ACAGGAC) |
| miR-122-5p (miRNAs w/seed GGAGUGU) | SAMSN1 |
| 12(S)-hydroxyeicosatetraenoic acid | nocodazole |
| pyrophosphate | MARK2 |
| SLC39A14 | vitamin A |
| CERS6 | eplerenone |
| GSTK1 | 6-hydroxydopamine |
| HDAC11 | PD 0325901 |
| ADGRF5 | OSCAR |
| S100A10 | ABCB4 |
| STC1 | GAB2 |
| CD5L | EZR |
| SDC2 | PAK1 |
| CLTC | EWSR1 |
| SOAT1 | MEIS1 |
| filgotinib | daunorubicin |
| TNRC6A | bardoxolone |
| TNFRSF10B | miR-6749-3p (miRNAs w/seed UCCUCCC) |
| cholestyramine | miR-1252-5p (miRNAs w/seed GAAGGAA) |
| benzoic acid | miR-936 (miRNAs w/seed CAGUAGA) |
| ETV6-NTRK3 | LMNA |
| PCM1-JAK2 | PDLIM2 |
| epoxyeicosatrienoic acid analog B | miR-224-5p (miRNAs w/seed AAGUCAC) |
| epoxyeicosatrienoic acid analog A | miR-31-3p (and other miRNAs w/seed GCUAUGC) |
| miR-1304-5p (miRNAs w/seed UUGAGGC) | miR-490-5p (miRNAs w/seed CAUGGAU) |
| miR-4638-5p (miRNAs w/seed CUCGGCU) | NLRP3 |
| miR-7112-5p (miRNAs w/seed CGGGCAG) | GW9662 |
| beraprost | resolvin D1 |
| PP2A | TERC |
| chlorcyclizine | CYBB |
| naringin | IL36A |
| STAT1/3/5 dimer | IL17F |
| LAMTOR1 | bafilomycin A1 |
| GADD45G | methylnitrosourea |
| HSPB8 | FUS-DDIT3 |
| BAG3 | andrographolide |
| ELN | naringenin |
| CACNA1A | miR-509-5p (and other miRNAs w/seed ACUCCAG) |
| CXCL1 | miR-583 (miRNAs w/seed AAAGAGG) |
| HNRNPAB | docosahexaenoic acid |

quinacrine
1-((2-chlorophenyl)diphenylmethyl)-1H-pyrazole
sanglifehrin A
4-octyl itaconate
roflumilast N-oxide
midostaurin
8-oxo-7-hydrodeoxyguanosine
diaminopimelic acid
Mt
beta-cyclodextrin
miR-1247-3p (miRNAs w/seed CCCGGGA)
Iga
SRA1
HDAC7
ODC1
LIPG
ST8SIA1
miR-2355-3p (miRNAs w/seed UUGUCCU)
miR-8080 (miRNAs w/seed AAGGACA)
IL23
miR-8076 (miRNAs w/seed AUAUGGA)
Ubiquitin
miR-659-5p (miRNAs w/seed GGACCUU)
Trp53cor1
miR-3166 (miRNAs w/seed GCAGACA)
miR-1258-5p (and other miRNAs w/seed GCUGAGC)
miR-4443 (miRNAs w/seed UGGAGGC)
miR-566 (and other miRNAs w/seed GGCGCCU)
miR-881-3p (and other miRNAs w/seed ACUGUGU)
miR-5586-3p (miRNAs w/seed AGAGUGA)
miR-4652-3p (miRNAs w/seed UUCUGUU)
miR-3680-3p (miRNAs w/seed UUUGCAU)
ADAM10
miR-128-1-5p (and other miRNAs w/seed GGGGCCG)
miR-1250-5p (miRNAs w/seed CGGUGCU)
miR-6082 (miRNAs w/seed AAUACGU)
miR-6763-3p (miRNAs w/seed UCCCCGG)
miR-1468-5p (miRNAs w/seed UCCGUUU)
miR-5090 (and other miRNAs w/seed CGGGGCA)
miR-29c-5p (miRNAs w/seed GACCGAU)
miR-6785-3p (miRNAs w/seed CAUCGCC)
miR-4529-5p (miRNAs w/seed GGCCAUC)
miR-659-3p (miRNAs w/seed UUGGUUC)
miR-6719-3p (miRNAs w/seed CUGACAU)
miR-3910 (miRNAs w/seed AAGGCAU)
miR-379-5p (and other miRNAs w/seed GGUAGAC)
miR-6857-3p (miRNAs w/seed GACUGAG)
miR-4491 (and other miRNAs w/seed AUGUGGA)
miR-4276 (and other miRNAs w/seed UCAGUGA)
miR-2278 (miRNAs w/seed AGAGCAG)
miR-378g (miRNAs w/seed CUGGGCU)
miR-606 (miRNAs w/seed AACUACU)
miR-4731-3p (and other miRNAs w/seed ACACAAG)
CLPP
ARNTL
RNASE2
CD4
miR-6798-3p (miRNAs w/seed UACCCCC)
miR-6515-5p (miRNAs w/seed UGGAGGG)
FGF8
miR-6806-5p (miRNAs w/seed GUAGGCA)
miR-8062 (miRNAs w/seed AGUGAUU)
miR-6774-3p (miRNAs w/seed CGUGUCC)
CBFB
miR-203a-5p (and other miRNAs w/seed GUGGUUC)
miR-324-3p (miRNAs w/seed CCACUGC)
miR-4773 (miRNAs w/seed AGAACAG)
ZC3H14
Hmgb1
MESP2
UCP2
ABCC8
NBEAL2
C5AR2
miR-370-5p (and other miRNAs w/seed AGGUCAC)
miR-339-5p (and other miRNAs w/seed CCCUGUC)
miR-5684 (miRNAs w/seed ACUCUAG)
miR-4774-5p (miRNAs w/seed CUGGUAU)
miR-299a-5p (and other miRNAs w/seed GGUUUAC)
miR-3671 (miRNAs w/seed UCAAAUA)
miR-6409 (and other miRNAs w/seed GCGGGUG)
miR-2054 (miRNAs w/seed UGUAAUA)
miR-3943 (miRNAs w/seed AGCCCCC)
miR-181b-1-3p (and other miRNAs w/seed UCACUGA)
Rxr
miR-130b-5p (and other miRNAs w/seed CUCUUUC)
miR-4264 (miRNAs w/seed CUCAGUC)
miR-34a-3p (and other miRNAs w/seed AUCAGCA)
NOG
miR-3944-5p (miRNAs w/seed GUGCAGC)
miR-6868-3p (miRNAs w/seed UCCUUCU)
miR-544b (miRNAs w/seed CCUGAGG)
miR-3150a-3p (and other miRNAs w/seed UGGGGAG)
miR-3128 (and other miRNAs w/seed CUGGCAA)
miR-6815-3p (miRNAs w/seed GGCUUCU)
miR-6834-5p (miRNAs w/seed UGAGGGA)
miR-3605-3p (miRNAs w/seed CUCCGUG)
miR-6859-3p (miRNAs w/seed GACCCCC)
miR-4520-5p (miRNAs w/seed CUGCGUG)
miR-626 (and other miRNAs w/seed GCUGUCU)
miR-3184-3p (miRNAs w/seed AAGUCUC)
miR-3613-5p (miRNAs w/seed GUUGUAC)
miR-486-5p (and other miRNAs w/seed CCUGUAC)
miR-3976 (miRNAs w/seed AUAGAGA)
miR-6830-3p (miRNAs w/seed GUCUUUC)
L-tryptophan
Tnf receptor
IgG1
SPOP
CXCR3
NOX1
PBX3
CYP27A1
TERF2IP
alendronic acid
miR-4437 (miRNAs w/seed GGGCUCA)
IL-1R
mir-142
NKX2-2-AS1
nilvadipine
SLC9A3
NFKBIZ
ESRRA
RORC
miR-592 (and other miRNAs w/seed UGUGUCA)
miR-4436b-5p (miRNAs w/seed UCCACUU)
miR-1900 (and other miRNAs w/seed GCCGCCC)
purvalanol A
4-aminopyrazolo(3,4-d)pyrimidine
diphosphoryl lipid A
1,4-glucan
arbutin
syringin
7beta-hydroxycholesterol
prostaglandin E3
abciximab
Plc beta
Rab5
glemanserin
Mitochondrial complex 1
SFTPA2
T-type Calcium Channel
Rbx1
EIF4B
TRIM50
UPK3A
NMRAL1
POPDC2
ARL16
LYST
Hsf3
MARCHF2
SGF29
SMYD5
PHOSPHO1
ZNF385B
NCEH1
ARL11
SENP5
PACS2
Plk
AFAP1L2
ECD
soy isoflavones
ganglioside GM3 (d18:1/18:0)
ganglioside GM3 (d18:1/20:0)
ganglioside GM3 (d18:1/22:0)
Par
IKKA/B
diaziquone
RPL19
pimasertib
AZD4547
Raet1b
IVNS1ABP
CELA1
TRIM5
cyproconazole

Rp-8-Br-cGMPS
sangivamycin
thioguanine
glycodeoxycholic acid
IGF receptor
1-palmitoyl-2-arachidonoyl-phosphatidylcholine
benzo(a)pyrene 7,8-dihydrodiol
1,2,5,6-dibenzanthracene
Esr1-Estrogen-Sp1
prochloraz
INTS11
CHD5
ZNF451
PRR7
C1QL1
PSMD14
ZNF300
NAA30
ASH1L
PTPN23
EIF4F
ATP1A2
defibrotide
EMP2
PURB
YARS1
U2AF2
riociguat
PBK
HSPB3
KLF16
DOK2
EIF2B1
STK16
DLST
GFRA2
RGS3
KPNB1
KRT5
ELOA
CDK5R2
BTG1
INHBC
ERC1
IL20RA
Miat
8-butyl-10-[(3-methoxy-5-pyrrol-2-ylidenepyrrol-2-ylidene)methyl]-11-azabicyclo[7.2.1]dodeca-1
Irs4
TIGAR
nordihydrocapsiate
nitrous oxide
mecamylamine
amantadine
prinaberel
filipin
beta-lapachone
albendazole
cycloserine
minodronate
oenothein B
modafinil
MLN-4760
TTI-101
mycophenolate mofetil
nickel subsulfide
5,10,15,20-tetrakis(4-sulfonatophenyl)porphyrinato iron (III)
senexin B
actinomycin
ODN-BW006
(indol-3-yl)pyruvic acid
3,7-dimethyl-1-propargylxanthine
oleandrin
EHF
miR-6069 (miRNAs w/seed GGCUAGG)
miR-5008-3p (and other miRNAs w/seed CUGUGCU)
miR-542-3p (miRNAs w/seed GUGACAG)
8-bromoguanosine 3',5'-cyclic monophosphate
PRDM16
CASP1
IGFBP3
mercuric chloride
SMAD2
miR-193a-3p (and other miRNAs w/seed ACUGGCC)
miR-4755-5p (and other miRNAs w/seed UUCCCUU)
CpG ODN 2006
PIAS1
RAD21
alvocidib
miR-891a-5p (miRNAs w/seed GCAACGA)
miR-5581-3p (miRNAs w/seed UCCAUGC)
miR-3148 (miRNAs w/seed GGAAAAA)
LEPR
RB1
nitrofen
NSD2
TSLP
GDF9
NQO1
SPRY2
MMP2
ADORA2B
cilostazol
steroid
RBL2
miR-6822-3p (miRNAs w/seed GGCUCUA)
miR-589-5p (and other miRNAs w/seed GAGAACC)
miR-4432 (miRNAs w/seed AAGACUC)
IL18
CYLD
paricalcitol
fluvastatin
INSIG1
NR1H4
miR-455-5p (and other miRNAs w/seed AUGUGCC)
INHA
miR-1226-3p (miRNAs w/seed CACCAGC)
NKX2-1
EIF3E
CEACAM1
miR-3687 (and other miRNAs w/seed CCGGACA)
IRF4
IRF1
MMP3
conjugated linoleic acid
LGR4
miR-6818-3p (miRNAs w/seed UGUCUCU)
colfosceril palmitate
Rsk
CC8490
tylophorine
NPSR1
PDE8A
LINC00887
CIRBP
RAB7
PF-4691502
HSPB6
CHRM4
WARS1
ERO1A
HSPH1
MAD1L1
OSU-CG12
abrocitinib
hoechst 33342
tramadol
bupropion
1,1-diethyl-2-hydroxy-2-nitrosohydrazine
microcystin-LR
miR-3571 (and other miRNAs w/seed ACACACU)
miR-221-3p (and other miRNAs w/seed GCUACAU)
CD2
PEBP1
PTGS1
EFNA4
CCR5
FASLG
ibuprofen
everolimus
SAHM1
LDB1
miR-1973 (miRNAs w/seed CCGUGCA)
miR-12201-5p (and other miRNAs w/seed GGAAGGA)
miR-875-3p (miRNAs w/seed CUGGAAA)
INHBA
colistin
LMO2
RRP1B
SOX4
EED
RPS6KB1
SELPLG
DPP4
ARHGAP21
EFNA3
fasudil
beta-carotene
miR-4722-5p (miRNAs w/seed GCAGGAG)

| | |
|---|---|
| LAMA3 | kanamycin A |
| TPPP3 | miR-5135 (and other miRNAs w/seed GGUCUAG) |
| H60a | TNC |
| GABARAP | ELANE |
| POLDIP3 | ANXA1 |
| IL1RAPL1 | CITED2 |
| GATAD2B | ILK |
| DCTN4 | U73122 |
| ZNF410 | miR-192-5p (and other miRNAs w/seed UGACCUA) |
| CCT5 | GATA6 |
| AZU1 | miR-503-3p (and other miRNAs w/seed GAGUAUU) |
| MUC16 | EOMES |
| DST | miR-199a-3p (and other miRNAs w/seed CAGUAGU) |
| SLC7A11 | miR-500a-5p (miRNAs w/seed AAUCCUU) |
| DDX24 | miR-6752-3p (miRNAs w/seed CCCUGCC) |
| ZFP36L1 | miR-4290 (miRNAs w/seed GCCCUCC) |
| CRADD | miR-3686 (and other miRNAs w/seed UCUGUAA) |
| ATP9A | miR-3117-3p (miRNAs w/seed UAGGACU) |
| IMPACT | RBM5 |
| GPR32 | A2M |
| TMEM9B | EFNA5 |
| CD8B | PCGF2 |
| KHDRBS2 | dimethylnitrosamine |
| GAB3 | acetovanillone |
| GALR2 | miR-4713-5p (miRNAs w/seed UCUCCCA) |
| 3-hydroxydodecanoic acid | sodium bisulfide |
| 6-n-octylaminouracil | PRKAA |
| CYP24A1 | mir-146 |
| MED6 | MED13 |
| CD300E | MEF2A |
| ralimetinib | RIPK1 |
| DDT | CRP |
| CGGBP1 | PRKACA |
| IL17D | Pam3-Cys |
| HUS1 | temozolomide |
| EYA4 | miR-153-3p (miRNAs w/seed UGCAUAG) |
| LPCAT1 | metronidazole |
| ATB-346 | L-alpha-lysophosphatidylcholine, palmitoyl |
| IPP-204106N | styrene |
| polysaccharide TA-1 | MNK1/2 |
| BAI | Cpla2 |
| Mia2 | DGKE |
| bimekizumab | MARCHF3 |
| hydroxyl radical | BCAS2 |
| minalrestat | BPI |
| buformin | PYHIN1 |
| semapimod | ERN2 |
| neocuproine | TYMS |
| pyridoxal | NCK2 |
| indapamide | LOC102724788/PRODH |
| E-4031 | APAF1 |
| dofetilide | BRCA2 |
| desethylamiodarone | RPS3 |
| dilazep | CCL22 |
| BAY-u9773 | PRMT2 |
| CGP77675 | SENP6 |
| bumetanide | ACOD1 |
| butenafine | PELI2 |
| terpenoid | CDC27 |
| wax ester | CC-122 |
| LZ1 peptide | MI-773 |
| moringa oleifera aqueous seed extract | U 50488H |
| moringa oleifera diluted seed extract | mevalonolactone |
| dapansutrile | tranexamic acid |
| Z-YVAD-FMK | Collagen type V |
| D-JNK inhibitor I | roflumilast |
| Z-WEHD-FMK | paxilline |
| SR1555 | FT671 |
| SR2211 | aclarubicin |
| alsterpaullone | phytol |
| alpha-TEA | methyllycaconitine |
| tetrahydropalmatine | acetyl-11-keto-beta-boswellic acid |
| D-lysergic acid diethylamide | miR-6726-3p (miRNAs w/seed UCGCCCU) |
| ceftazidime | melatonin |
| trehalose dimycolate | obeticholic acid |
| 12(S)-HPETE | miR-675-3p (miRNAs w/seed UGUAUGC) |
| L-canavanine | miR-3977 (miRNAs w/seed UGCUUCA) |
| miR-376b-5p (and other miRNAs w/seed GUGGAUA) | miR-202-3p (and other miRNAs w/seed GAGGUAU) |
| miR-548m (miRNAs w/seed AAAGGUA) | cholecalciferol |
| SGK1 | Hsp27 |
| miR-6863 (miRNAs w/seed AGACGUG) | MEN1 |
| miR-7843-3p (miRNAs w/seed UGAAGCC) | FYN |
| miR-6760-5p (miRNAs w/seed AGGGAGA) | NOS3 |
| miR-1227-3p (miRNAs w/seed GUGCCAC) | NUP98-DDX10 |
| miR-3913-5p (and other miRNAs w/seed UUGGGAC) | EIF4E |
| miR-619-3p (miRNAs w/seed ACCUGGA) | miR-140-5p (and other miRNAs w/seed AGUGGUU) |
| miR-453 (and other miRNAs w/seed GGUUGCC) | miR-92a-3p (and other miRNAs w/seed AUUGCAC) |
| miR-5191 (miRNAs w/seed GGAUAGG) | miR-291a-3p (and other miRNAs w/seed AAGUGCU) |
| miR-34b-3p (miRNAs w/seed AAUCACU) | miR-4251 (miRNAs w/seed CUGAGAA) |
| miR-556-5p (miRNAs w/seed AUGAGCU) | nilotinib |
| miR-4732-5p (miRNAs w/seed GUAGAGC) | IL24 |
| FBXO32 | LTBR |
| spironolactone | Ifn |
| inositol | Hsp70 |
| teniposide | Hedgehog |
| [Lys15,Arg16,Leu27]VIP(1-7)GRF(8-27) | AURK |
| AOPEP | ANLN |
| dienogest | miR-6529-3p (and other miRNAs w/seed CUGUGCC) |
| rintatolimod | 8-hydroxyguanine |
| RAR-RXR | histidinol |
| IFN alpha receptor | norethindrone acetate |
| CpG ODN M362 | Dynamin |
| RNF5 | tacedinaline |
| GATM | obatoclax |
| HYAL1 | KLF17 |
| benzenesulfonic acid | RGMB |
| TAF9 | CCN6 |
| dinaciclib | peptide T |
| IgG2b | RABGEF1 |
| Mucin | HSPB2 |
| TRAFD1 | PROK2 |
| DCB 3503 | entolimod |
| STX11 | NUCB2 |
| AHSG | Irs3 |
| IL31RA | MAGED1 |
| HAMP | ETV3 |
| ZBTB46 | AATF |
| PPP1R13B | RNF111 |
| GAS7 | OTUD7B |
| WSB1 | GRM5 |
| PROS1 | DYNLL1 |
| FGG | CCL15 |
| PILRB | CFH |
| Orm1 (includes others) | STK38 |
| EIF2AK1 | CTSK |
| WFS1 | PPID |
| ADAM8 | XPA |
| SLC37A4 | SERPIND1 |
| SRSF6 | SF1 |
| SGCD | NET1 |
| N-lauroyl-L-phenylalanine | CXXC4 |
| ODN-A151 | ZYX |
| RFXANK | fenebrutinib |
| HG-9-91-01 | COG112 peptide |
| NCGC00186528 | gelam honey |
| 3-hydroxybutyric acid | ambroxol |
| atrasentan | emricasan |
| DIM-C-pPhCO2Me | pyridoxamine |
| L-homocysteine | chlorambucil |
| allosamidin | PK11007 |
| miR-3665 (miRNAs w/seed GCAGGUG) | JWH-015 |
| miR-92a-2-5p (miRNAs w/seed GGUGGGG) | alpha-hydroxyglutarate |
| miR-1197 (and other miRNAs w/seed AGGACAC) | CV 3988 |
| miR-4710 (miRNAs w/seed GGUGAGG) | H2AB3 (includes others) |
| miR-3144-3p (miRNAs w/seed UAUACCU) | TEAD2 |
| monocrotaline | beta-naphthoflavone |
| miR-193b-5p (miRNAs w/seed GGGGUUU) | E2f |
| miR-6845-3p (and other miRNAs w/seed CUCUCCU) | miR-423-5p (and other miRNAs w/seed GAGGGGC) |
| miR-3136-3p (and other miRNAs w/seed GGCCCAA) | miR-4327 (miRNAs w/seed GCUUGCA) |
| miR-2115-5p (miRNAs w/seed GCUUCCA) | miR-4453 (and other miRNAs w/seed AGCUUGG) |
| miR-520g-3p (and other miRNAs w/seed CAAAGUG) | miR-656-3p (miRNAs w/seed AUAUUAU) |
| miR-3074 (and other miRNAs w/seed AUAUCAG) | RUNX2 |
| miR-7112-3p (miRNAs w/seed GCAUCAC) | ZBTB20 |
| miR-143-3p (and other miRNAs w/seed GAGAUGA) | panobinostat |
| CIDEC | mir-183 |
| miR-8055 (miRNAs w/seed UUUGAGC) | miR-6505-5p (miRNAs w/seed UGGAAUA) |
| miR-6126 (miRNAs w/seed UGAAGGC) | miR-6771-3p (miRNAs w/seed AAACCCC) |
| miR-212-5p (miRNAs w/seed CCUUGGC) | miR-660-3p (miRNAs w/seed CCUCCUG) |
| Mmp | bromodeoxyuridine |
| perhexiline | MTORC1 |
| WP1066 | miR-4325 (miRNAs w/seed UGCACUU) |
| CLEC10A | TMPRSS2-ERG |
| particulate matter | miR-432 (and other miRNAs w/seed CUUGGAG) |
| iron dextran | miR-2392 (miRNAs w/seed AGGAUGG) |
| RYR2 | miR-4520-3p (miRNAs w/seed UGGACAG) |
| YWHAQ | CEBPE |
| mir-373 | LPL |
| PTGDR | SAFB |
| MMP8 | Am 580 |
| ATXN3 | miR-582-3p (miRNAs w/seed AACUGGU) |
| VEGFD | miR-642a-3p (and other miRNAs w/seed GACACAU) |
| DUSP14 | miR-4734 (miRNAs w/seed CUGCGGG) |
| CAMKK2 | miR-8074 (miRNAs w/seed UAUGGCG) |
| MIA-602 | MOG |
| 1-chloro-2-(2,2,2-trichloro-1-(4-chlorophenyl)ethyl)benzene | TSHZ3 |
| ethylenediaminetetraacetic acid | FGF10 |
| poly-L-lysine | EHMT2 |
| [D-Ala2,N-Me-Phe4,Gly5-ol]-Enkephalin | miR-1825 (miRNAs w/seed CCAGUGC) |

| | |
|---|---|
| cyclomaltodextrin | alloxan |
| S-equol | pervanadate |
| miR-1914-5p (miRNAs w/seed CCUGUGC) | dopamine D1 receptor |
| miR-564 (and other miRNAs w/seed GGCACGG) | flavokawain B |
| miR-3678-3p (miRNAs w/seed UGCAGAG) | triamcinolone hexacetonide |
| miR-27a-5p (miRNAs w/seed GGGCUUA) | rh-endostatin |
| miR-6792-5p (miRNAs w/seed UAAGCAG) | ISX |
| miR-331-3p (miRNAs w/seed CCCCUGG) | MRPS18B |
| miR-219a-1-3p (miRNAs w/seed GAGUUGA) | ST6GALNAC1 |
| miR-465d-3p (and other miRNAs w/seed GAUCAGG) | ZNF652 |
| miR-6793-3p (miRNAs w/seed CCCCAAC) | SH2D5 |
| miR-6768-5p (miRNAs w/seed ACACAGG) | BCAR4 |
| miR-298-5p (and other miRNAs w/seed GCAGAGG) | SLC5A8 |
| miR-20b-3p (miRNAs w/seed CUGUAGU) | KMT5B |
| miR-3675-3p (miRNAs w/seed AUCUCUA) | TCIM |
| miR-4723-5p (and other miRNAs w/seed GGGGGAG) | iguratimod |
| miR-665 (and other miRNAs w/seed CCAGGAG) | sotrastaurin |
| miR-4730 (miRNAs w/seed UGGCGGA) | Sfk |
| miR-6790-3p (miRNAs w/seed GACCUCG) | Collagen type III |
| miR-4455 (miRNAs w/seed GGGUGUG) | HLA-DQ |
| prostaglandin J2 | ASCL2 |
| TET1 | idelalisib |
| epinephrine | IER3 |
| RXRB | PDE6B |
| miR-513c-5p (and other miRNAs w/seed UCUCAAG) | REV1 |
| miR-4724-5p (miRNAs w/seed ACUGAAC) | MEFV |
| miR-4422 (miRNAs w/seed AAAGCAU) | IFNGR |
| miR-4310 (and other miRNAs w/seed CAGCAUU) | SERPINA4 |
| 2',3'-dialdehyde ATP | ENO1 |
| R 59022 | TSC22D1 |
| carnobyl iron | RBX1 |
| tocilizumab | P2RY1 |
| IRF3 dimer | PTBP3 |
| bacterial lipopolysaccharides | PIP5K1C |
| bafilomycin A | CUL7 |
| RASSF6 | CLIC4 |
| LRRK2 | MLLT3 |
| NAALADL2 | TNKS |
| CD80/CD86 | ERBIN |
| SMAD1/5 | casticin |
| SPTAN1 | ADCYAP1R1 |
| BCKDK | CCR4 |
| ABCD1 | MS4A1 |
| SERPINB2 | KLKB1 |
| THBD | GNMT |
| mir-217 | PILRA |
| RFXAP | Pde4d |
| PLCB1 | RHOC |
| IL4I1 | 15-E2-isoketal modified phosphatidylethanolamine |
| PANDAR | perifosine |
| MC1R | dimercaptopropanol |
| NPC1L1 | aminoglutethimide |
| UACA | trimethyltin |
| IL22RA1 | 4-coumaric acid |
| PDYN | fluphenazine |
| niflumic acid | 2-mercaptoethanol |
| ziprasidone | polyphosphate |
| tyrphostin AG 127 | retroinverso ERG inhibitory peptide 2 |
| L-N6-(1-iminoethyl)-lysine | retroinverso ERG inhibitory peptide 1 |
| miR-548aw (miRNAs w/seed UGCAAAA) | atropine |
| miR-3921 (and other miRNAs w/seed CUCUGAG) | phosphatidic acid |
| miR-4451 (miRNAs w/seed GGUAGAG) | 12-hydroxyeicosatetraenoic acid |
| miR-3177-5p (miRNAs w/seed GUGUACA) | vinpocetine |
| BMPR2 | arsenic trioxide |
| ELOVL5 | docetaxel |
| SFN | miR-6764-3p (and other miRNAs w/seed CUCUGGU) |
| FZD8 | imatinib |
| GFI1B | dorsomorphin |
| PDK4 | BIRC5 |
| propionic acid | mir-24 |
| magnolol | CBL |
| TGAL copolymer | E2F4 |
| glycyrrhetinic acid | miR-5190 (miRNAs w/seed CAGUGAC) |
| irinotecan | IL32 |
| iloprost | EFNA2 |
| tauroursodeoxycholic acid | miR-4757-5p (and other miRNAs w/seed GGCCUCU) |
| miR-4735-5p (miRNAs w/seed CUAAUUU) | miR-5088-5p (miRNAs w/seed AGGGCUC) |
| miR-3938 (miRNAs w/seed AUUCCCU) | WBP2 |
| miR-448-3p (and other miRNAs w/seed UGCAUAU) | miR-4691-5p (and other miRNAs w/seed UCCUCCA) |
| miR-106a-3p (and other miRNAs w/seed UGCAAUG) | miR-548at-5p (miRNAs w/seed AAAGUUA) |
| miR-7158-3p (miRNAs w/seed UGAACUA) | miR-4717-3p (miRNAs w/seed CACAUGG) |
| miR-184 (and other miRNAs w/seed GGACGGA) | mir-204 |
| miR-4797-3p (miRNAs w/seed CUCAGUA) | APOA1 |
| miR-4746-3p (miRNAs w/seed GCGGUGC) | Gcg |
| miR-4999-3p (miRNAs w/seed CACUACC) | miR-369-5p (miRNAs w/seed GAUCGAC) |
| miR-879-5p (and other miRNAs w/seed GAGGCUU) | miR-18a-5p (and other miRNAs w/seed AAGGUGC) |
| YBX1 | miR-296-3p (miRNAs w/seed AGGGUUG) |
| miR-3126-5p (and other miRNAs w/seed GAGGGAC) | miR-292b-5p (and other miRNAs w/seed CUCAAAA) |
| alvespimycin | 1810019D21Rik |
| MGAT5 | pravastatin |
| CETP | IL12 (family) |
| Ro41-5253 | miR-4717-5p (and other miRNAs w/seed AGGCCAC) |
| beta-glycerophosphoric acid | miR-31-5p (and other miRNAs w/seed GGCAAGA) |
| miR-4450 (and other miRNAs w/seed GGGGAUU) | UDP |
| miR-197-5p (and other miRNAs w/seed GGGUAGA) | heparan sulfate |
| miR-3109-3p (and other miRNAs w/seed AGGGCCA) | fingolimod phosphate |
| miR-1251-3p (miRNAs w/seed GCUUUGC) | 2-amino-5-azotoluene |
| miR-548aa (and other miRNAs w/seed AAAACCA) | tectorigenin |
| miR-6886-3p (miRNAs w/seed GCCCUUC) | SENP7 |
| miR-7044-3p (and other miRNAs w/seed UGCAGCC) | PON2 |
| miR-1306-3p (miRNAs w/seed CGUUGGC) | NELFCD |
| miR-4777-3p (miRNAs w/seed UACCUCA) | SULT2B1 |
| miR-4323 (miRNAs w/seed AGCCCCA) | IL26 |
| TSC1 | FANCA |
| miR-1185-5p (and other miRNAs w/seed GAGGAUA) | S100a7a |
| miR-6846-3p (miRNAs w/seed GACCCCU) | FEM1A |
| miR-320b (and other miRNAs w/seed AAAGCUG) | ponesimod |
| miR-3909 (and other miRNAs w/seed GUCCUCU) | ATF7 |
| miR-1668 (and other miRNAs w/seed AAAGGCC) | GALP |
| miR-888-5p (miRNAs w/seed ACUCAAA) | farnesyl transferase |
| miR-3915 (miRNAs w/seed UGAGGAA) | teasaponin |
| miR-297a-5p (and other miRNAs w/seed UGUAUGU) | DIABLO |
| miR-4713-3p (miRNAs w/seed GGGAUCC) | PCBP1 |
| miR-1343-5p (and other miRNAs w/seed GGGGAGC) | raclopride |
| FOXA2 | GW 5074 |
| CLN3 | mechlorethamine |
| CHRNA7 | domoic acid |
| IGF2BP1 | rabeprazole |
| bile acid | AGI-1067 |
| miR-610 (miRNAs w/seed GAGCUAA) | magnesium sulfate |
| miR-191-5p (and other miRNAs w/seed AACGGAA) | palmitoyl-Cys((RS)-2,3-di(palmitoyloxy)-propyl)-Ala-Gly-OH |
| miR-1249-3p (and other miRNAs w/seed CGCCCUU) | theaflavin |
| miR-6834-3p (miRNAs w/seed AUGUCCC) | alpha-tocopherol succinate |
| miR-1298-3p (miRNAs w/seed AUCUGGG) | miR-3654 (miRNAs w/seed ACUGGAC) |
| CDK4 | S-nitroso-N-acetyl-DL-penicillamine |
| miR-597-3p (and other miRNAs w/seed GGUUCUC) | PPP2CA |
| miR-2861 (and other miRNAs w/seed GGGCCUG) | CHD4 |
| miR-655-5p (miRNAs w/seed GAGGUUA) | capsaicin |
| miR-349 (and other miRNAs w/seed AGCCCUG) | miR-3547 (and other miRNAs w/seed GAGCACC) |
| miR-8086 (miRNAs w/seed GCUAGUC) | TET2 |
| BRD2 | TNFRSF8 |
| PRDM5 | HIC1 |
| miR-890 (miRNAs w/seed ACUUGGA) | vitamin D |
| miR-151-3p (and other miRNAs w/seed UAGACUG) | miR-504-5p (and other miRNAs w/seed GACCCUG) |
| miR-194-3p (and other miRNAs w/seed CAGUGGG) | mir-223 |
| miR-4662a-5p (miRNAs w/seed UAGCCAA) | miR-16-5p (and other miRNAs w/seed AGCAGCA) |
| miR-520g-5p (miRNAs w/seed CUAGAGG) | miR-4761-5p (miRNAs w/seed CAAGGUG) |
| miR-4781-5p (miRNAs w/seed AGCGGGG) | miR-6512-3p (and other miRNAs w/seed UCCAGCC) |
| miR-1248 (miRNAs w/seed CCUUCUU) | C5AR1 |
| miR-568 (miRNAs w/seed UGUAUAA) | RHO |
| miR-3127-5p (miRNAs w/seed UCAGGGC) | seocalcitol |
| miR-758-5p (miRNAs w/seed AUGGUUG) | miR-6084 (miRNAs w/seed UCCGCCA) |
| miR-4525 (and other miRNAs w/seed GGGGGAU) | miR-548aj-5p (and other miRNAs w/seed GCAAAAG) |
| IL25 | miR-187-5p (miRNAs w/seed GCUACAA) |
| mir-133 | miR-377-3p (miRNAs w/seed UCACACA) |
| FASN | STING1 |
| COLQ | NPY |
| miR-4513 (and other miRNAs w/seed GACUGAC) | NRIP1 |
| miR-4742-3p (miRNAs w/seed CUGUAUU) | BACH2 |
| ASXL2 | miR-4762-5p (miRNAs w/seed CAAAUCU) |
| SP4 | miR-3551-5p (and other miRNAs w/seed CUUCACC) |
| HOXA7 | miR-5193 (miRNAs w/seed CCUCCUC) |
| silymarin | IFNA2 |
| miR-139-3p (miRNAs w/seed GGAGACG) | ganglioside |
| miR-3689a-5p (and other miRNAs w/seed GUGAUAU) | 16,16-dimethylprostaglandin E2 |
| miR-4796-3p (miRNAs w/seed AAAGUGG) | triciribine |
| miR-639 (miRNAs w/seed UCGCUGC) | L-lysine |
| mocetinostat | bazedoxifene |
| DANCR | Pde |
| Collagen Alpha1 | HTR7 |
| SCAVENGER receptor CLASS A | SNHG7 |
| TOLLIP | FBXO42 |
| CBX2 | HUWE1 |
| RLN2 | TPSAB1/TPSB2 |
| XRCC5 | tryptase |
| MGAT3 | UNC5B |
| PTPRE | CTNND1 |
| RIPK3 | TPM3 |
| RBP3 | PI3 |
| NPFF | mir-873 |
| FPR2 | NELFA |
| ID4 | TSG101 |
| coomassie brilliant blue | GPR183 |
| meclizine | ST6GAL1 |
| CpdX-D3 | COCH |
| N-acetylglucosamine | PPP1CA |
| miR-662 (miRNAs w/seed CCCACGU) | TKT |
| miR-5588-3p (miRNAs w/seed AGUCCCA) | ANPEP |

miR-514a-3p (and other miRNAs w/seed UUGACAC)
miR-4268 (miRNAs w/seed GCUCCUC)
miR-4780 (miRNAs w/seed CCCUUGA)
LATS2
anandamide
CYP1A1
miR-550b-2-5p (and other miRNAs w/seed UGUGCCU)
miR-6804-3p (miRNAs w/seed GCACCUG)
miR-6501-3p (miRNAs w/seed CAGAGCA)
miR-6918-5p (and other miRNAs w/seed GCUGAGG)
miR-5093 (miRNAs w/seed GGAAAUG)
miR-3942-3p (and other miRNAs w/seed UUCAGAU)
FOLR1
miR-5113 (and other miRNAs w/seed CAGAGGA)
miR-4457 (miRNAs w/seed CACAAGG)
shikonin
paroxetine
miR-6125 (miRNAs w/seed CGGAAGG)
miR-3152-5p (miRNAs w/seed UUGCCUC)
miR-6872-3p (miRNAs w/seed CCAUGCC)
miR-3473g (and other miRNAs w/seed AAAAGUG)
miR-3661 (and other miRNAs w/seed GACCUGG)
RHOJ
IL31
CHEK1
SMURF1
NT5E
ACTB
GSK3A
thioridazine
lycopene
miR-142-5p (and other miRNAs w/seed AUAAAGU)
miR-6786-5p (miRNAs w/seed CGGUGGG)
GABPA
miR-6165 (miRNAs w/seed AGCAGGA)
miR-5579-5p (miRNAs w/seed AUGGUAC)
miR-4522 (and other miRNAs w/seed GACUCUG)
miR-449b-3p (and other miRNAs w/seed AGCCACA)
miR-7113-3p (miRNAs w/seed CUCCCUG)
miR-2113 (miRNAs w/seed UUUGUGC)
miR-5681a (and other miRNAs w/seed GAAAGGG)
miR-4680-3p (miRNAs w/seed CUGAAUU)
miR-4445-5p (miRNAs w/seed GAUUGUU)
miR-1183 (miRNAs w/seed ACUGUAG)
cholic acid
miR-6727-5p (miRNAs w/seed UCGGGGC)
sildenafil
choline
miR-3195 (miRNAs w/seed GCGCCGG)
miR-4263 (miRNAs w/seed UUCUAAG)
miR-651-3p (miRNAs w/seed AAGGAAA)
miR-3672 (and other miRNAs w/seed UGAGACU)
miR-5590-5p (miRNAs w/seed UGCCAUA)
miR-3928-5p (and other miRNAs w/seed GAAGCUC)
miR-4294 (miRNAs w/seed GGAGUCU)
miR-556-3p (miRNAs w/seed UAUUACC)
PPIF
CALCA
miR-6776-5p (miRNAs w/seed CUGGGUG)
miR-3108-3p (and other miRNAs w/seed GUCUAGG)
miR-6827-5p (miRNAs w/seed GGGAGCC)
miR-6757-5p (miRNAs w/seed AGGGAUG)
miR-3200-5p (miRNAs w/seed AUCUGAG)
TCL1A
aflatoxin B1
PARP
ATP6AP2
ganetespib
CAVIN1
ADCY7
TYRO3
HSP90AA1
PDK2
MIR320
mir-185
RAMP1
UGDH
HOXB3
TEF
AFF1
SH2B1
SLC6A1
TP53BP2
PCBP2
HDAC8
2-hydroxy-1-naphthylaldehyde isonicotinoyl hydrazone
levamisole
clomipramine
(5-(4-N-methyl-N(2-pyridyl)amino)ethoxy)benzyl thiazolidine-2,4-dione
tin mesoporphyrin
2,4-dinitrobenzenesulfonic acid
polymyxin B
perilla alcohol
dydrogesterone
miR-802-5p (miRNAs w/seed CAGUAAC)
miR-4798-5p (miRNAs w/seed UCGGUAU)
zoledronic acid
semaxinib
miR-8063 (miRNAs w/seed CAAAAUC)
miR-548d-3p (and other miRNAs w/seed AAAAACC)
CTCF
CASP3
ZNF281
mir-132
mir-31
ferulic acid
glycyrrhizic acid
miR-548as-3p (miRNAs w/seed AAAACCC)
miR-4278 (miRNAs w/seed UAGGGGG)
miR-5100 (miRNAs w/seed UCAGAUC)
miR-4444 (miRNAs w/seed UCGAGUU)
miR-1295b-5p (and other miRNAs w/seed ACCCAGA)
miR-498-5p (miRNAs w/seed UUCAAGC)
miR-8084 (miRNAs w/seed AAUACUA)
miR-134-3p (and other miRNAs w/seed UGUGGGC)
miR-361-5p (miRNAs w/seed UAUCAGA)
miR-5195-5p (miRNAs w/seed ACCCCUA)
1,2-dioleoyloxy-3-(trimethylammonium)propane
ganciclovir
roxithromycin
GPIIB-IIIA
Cobra Venom Factor
teichoic acid
Fcgr2
mGLUR Group I
CYP3A
N-hexanoylhomoserine lactone
TLR2/TLR4
glycitein
MN1
CLEC6A
CLEC4D
RDH8
dynasore
H6PD
SMPD2
FGF13
PPP4C
CNGB3
DLX4
CXCL5
ABCD2
RALB
BCL2L12
HAS1
mir-302
ATP2B2
PRSS8
RYK
F13A1
SRPK1
FSTL3
RNF17
TNFRSF14
19,20-epoxydocosapentaenoic acid
Ifna4
Hrg
Paxip1
VTX-2337
SAR-20347
MIA-459
MIA-313
olmesartan medoxomil
XCT790
ferric nitrilotriacetate
10-(6'-ubiquinonyl)decyltriphenylphosphonium
papaverine
propyl gallate
FC-99
scopoletin
RAM2061
NHB2005
N-retinylidene-N-retinylethanolamine
palmitoylethanolamide
sparfosic acid
ginsenoside Rg1
miR-330-3p (and other miRNAs w/seed CAAAGCA)
miR-6885-3p (miRNAs w/seed UUUGCUU)

NELFE
1,1-bis(3'-indolyl)-1-(4-trifluoromethyl-phenyl)methane
prodigiosin
ketorolac
BQ 123
hexamethoxyflavone
miR-3667-3p (miRNAs w/seed CCUUCCU)
miR-29b-1-5p (miRNAs w/seed CUGGUUU)
HNRNPA2B1
morphine
miR-378b (and other miRNAs w/seed UGGACUU)

oxaliplatin
miR-219a-2-3p (miRNAs w/seed GAAUUGU)
miR-6767-3p (miRNAs w/seed CACGUGC)
miR-6868-5p (miRNAs w/seed CUGGCAG)
miR-2115-3p (miRNAs w/seed AUCAGAA)
miR-3668 (miRNAs w/seed AUGUAGA)
miR-1265 (miRNAs w/seed AGGAUGU)
miR-26b-3p (and other miRNAs w/seed CUGUUCU)
miR-4694-3p (miRNAs w/seed AAAUGGA)
miR-1228-5p (miRNAs w/seed UGGGCGG)
miR-124-5p (miRNAs w/seed GUGUUCA)
miR-17-3p (and other miRNAs w/seed CUGCAGU)
HSPG2
miR-548g-3p (miRNAs w/seed AAACUGU)
miR-532-3p (miRNAs w/seed CUCCCAC)
miR-6514-3p (and other miRNAs w/seed UGCCUGU)
miR-455-3p (miRNAs w/seed CAGUCCA)
miR-651-5p (miRNAs w/seed UUAGGAU)
miR-3135a (miRNAs w/seed GCCUAGG)
clarithromycin
estrone
(+)-catechin
PCK1
CBX8
CPEB1
FCER1A
Smad2/3/4
SERTAD2
costunolide
perindopril
ginkgolide B
U18666A
miR-492 (miRNAs w/seed GGACCUG)
ZNF202
MK2206
G6PD
mir-101
PPP1R13L
RBP4
BID
HULC
zinc sulfate
zymosan A
creatine
miR-490-3p (miRNAs w/seed AACCUGG)
miR-4537 (miRNAs w/seed GAGCCGA)
miR-589-3p (and other miRNAs w/seed CAGAACA)
miR-5188 (miRNAs w/seed AUCGGAC)
miR-208a-5p (and other miRNAs w/seed AGCUUUU)
miR-3914 (miRNAs w/seed AGGAACC)
miR-4307 (miRNAs w/seed AUGUUUU)
miR-6811-3p (and other miRNAs w/seed GCCUGUG)
IHH
mir-19
miR-3941 (and other miRNAs w/seed UACACAC)
miR-708-3p (miRNAs w/seed AACUAGA)
miR-5627-5p (and other miRNAs w/seed GAGGGUG)
miR-887-5p (miRNAs w/seed UUGGGAG)
miR-5002-3p (miRNAs w/seed GACUGCC)
RNF2
AKT2
IL4R
miR-7-1-3p (and other miRNAs w/seed AACAAAU)
MYOG
MAPK11
CLU
WAS
THBS4
NUP98-HOXA9
miR-3682-3p (miRNAs w/seed GAUGAUA)
DMD
miR-8068 (miRNAs w/seed GUUUGUU)
miR-451b (miRNAs w/seed AGCAAGA)
miR-96-3p (miRNAs w/seed AUCAUGU)
miR-3663-5p (miRNAs w/seed CUGGUCU)
miR-343 (and other miRNAs w/seed CUCCCUC)
miR-6782-5p (and other miRNAs w/seed AGGGGUG)
miR-548o-3p (and other miRNAs w/seed CAAAACU)
miR-6869-5p (miRNAs w/seed UGAGUAG)
miR-3163 (miRNAs w/seed AUAAAAU)
miR-4481 (and other miRNAs w/seed GAGUGGG)
miR-555 (miRNAs w/seed GGGUAAG)
miR-3064-3p (miRNAs w/seed UGCCACA)
miR-516a-3p (and other miRNAs w/seed GCUUCCU)
miR-3683 (miRNAs w/seed GCGACAU)
miR-2114-5p (miRNAs w/seed AGUCCCU)
miR-1972 (miRNAs w/seed CAGGCCA)
miR-99a-3p (and other miRNAs w/seed AAGCUCG)
miR-2116-5p (miRNAs w/seed GUUCUUA)
miR-5197-5p (miRNAs w/seed AAUGGCA)
miR-4699-3p (miRNAs w/seed AUUUACU)
NPM1
miR-3140-5p (miRNAs w/seed CCUGAAU)
ZFPM1
miR-934 (miRNAs w/seed GUCUACU)
AHI1
ACLY
alpha-tocopherol
miR-1934-5p (and other miRNAs w/seed CUGGUCC)
miR-619-5p (and other miRNAs w/seed CUGGGAU)
HAND2
miR-509-5p (and other miRNAs w/seed ACUGCAG)
ABCB6
FOXC2
miR-3070-5p (and other miRNAs w/seed GCCCCUG)
miR-4777-5p (miRNAs w/seed UCUAGAU)
baicalin
miR-676-5p (miRNAs w/seed CUUCAAC)
miR-6777-5p (and other miRNAs w/seed CGGGGAG)
miR-4653-3p (miRNAs w/seed GGAGUUA)
miR-3675-5p (miRNAs w/seed AUGGGGC)
MAFB
miR-5011-5p (miRNAs w/seed AUAUAUA)
miR-4308 (and other miRNAs w/seed CCCUGGA)
miR-6861-5p (miRNAs w/seed CUGGGUA)
miR-4703-3p (miRNAs w/seed GUAGUUG)
miR-513a-3p (and other miRNAs w/seed AAAUUUC)
miR-100-5p (and other miRNAs w/seed ACCCGUA)
azathioprine
L-serine
Rp-8-Br-cAMPS
echinomycin
1-hydroxy-2-methyl-2-butenyl 4-diphosphate
mGluR
IRF
CERS2
TRIM25
FFAR1
CDCA7L
ruxolitinib
astragalin
DUSP4
APOH
LIMA1
FANCD2
DDR2
C6
CEBPG
SLC9A1
MAS1
OSTM1
PARP2
HLF
CHRNA3
JMJD1C
NDFIP1
CELF1
H3C14
COL17A1
NDP
MAP3K11
I-BET-151
oligomycin
zileuton
lidocaine
myelin oligodendrocyte glycoprotein (35-55)
ryanodine
scopolamine
sphingomyelin
allopregnanolone
miR-676-3p (miRNAs w/seed UGUCCUA)
miR-1233-3p (miRNAs w/seed GAGCCCU)
miR-3589 (and other miRNAs w/seed AGGAAAC)
miR-6765-5p (and other miRNAs w/seed UGAGGCG)
miR-648 (miRNAs w/seed AGUGUGC)
miR-766-5p (and other miRNAs w/seed GGAGGAA)
miR-605-5p (miRNAs w/seed AAAUCCC)
FUT8
CpG ODN 2395
HOXA11
COL2A1
Mir200
CBX3
TNFRSF13B
retinol
miR-4280 (miRNAs w/seed AGUGUAG)
miR-4661-5p (miRNAs w/seed ACUAGCU)
miR-150-3p (miRNAs w/seed UGGUACA)

miR-6835-3p (miRNAs w/seed AAAGCAC)
miR-584-3p (miRNAs w/seed CAGUUCC)
miR-381-3p (and other miRNAs w/seed AUACAAG)
miR-181d-3p (miRNAs w/seed CACCGGG)
TRPM2
mir-193
NOSTRIN
miR-4473 (miRNAs w/seed UAGUGCU)
miR-6825-3p (miRNAs w/seed CGCUGAC)
miR-6819-3p (and other miRNAs w/seed AGCCUCU)
salubrinal
ORAI1
COL1A1
NREP
PMP22
UBD
MIR101
HPX
ANGPTL2
TNFRSF25
IGFBP1
ACACB
PCYT2
Gm12602
CH-223191
Go6983
phenytoin
miR-1301-5p (and other miRNAs w/seed GCUCUAG)
miR-7152-5p (miRNAs w/seed UUCCUGU)
miR-874-3p (miRNAs w/seed UGCCCUG)
miR-548l (miRNAs w/seed AAAGUAU)
miR-3657 (and other miRNAs w/seed GUGUCCC)
rasagiline
CAB39L
MERTK
CD80
phosphatidylethanolamine
sulprostone
N6-cyclohexyladenosine
deoxyguanosine
moxestrol
hyodeoxycholic acid
MAP1LC3
cortisone
C1QTNF12
ixabepilone
Cyclin B
CK1
chlormethiazole
NIK
tolylfluanid
cardamomin
DEFA1 (includes others)
irisolidone
dietary fat derivative
imperatorin
IGF2-AS
TRIM13
KDM4D
polyethyleneimine
CLTRN
CHIA
FREM1
PRMT7
LDLRAP1
DDX41
ELOVL2
TRIM66
Il3r
SMCR8
Mir124a-1hg
hyperoside
2-(3-chlorophenylamino)phenylacetic acid
DGKH
TRIM67
ESRP2
HSD17B14
TRIM36
EXOC3L1
SDF4
SCARB2
INAVA
NAAA
bis(4-hydroxycinnamoyl)methane
ganglioside GM3 (d18:1/16:0)
ganglioside GM3 (d18:1/24:0)
ganglioside GM3 (d18:1/24:1(15Z))
Eotaxin
TH17 Cytokine
isoleucine
ACAT1
pycnogenols
Hspg
Inflammasome
pinoresinol
Pdi
foretinib
DAPK1
GCA
TPSG1
COL3A1
BAG4
17alpha-hydroxyprogesterone caproate
GSTA4
CLIP1
LYZ
PKC delta V1-1 peptide 8-17
HAO1
CASP7
MADD
9-benzyl-8-hydroxy-2-(2-methoxyethoxy)adenine
SGCA
TRIM9
CARD8
CEACAM6
SERPINB3
CEACAM21
IFI30
MID2
GZMA
EID2
PPP1R1A
MKNK2
MLST8
FCAR
DSG1
IFNGR2
STAG2
GC
PDZD2
Defb2
GJC1
Trim30a/Trim30d
Pkhd1
USP27X
CEACAM7
YWHAE
GPX4
URMC-099
VEZF1
CASP8AP2
RAN
TCP1
CASP10
Acp5
CRIP2
SRSF5
NKRF
BUB1B
UIMC1
CNTFR
proTAME
dinitrophenyl R group
AZD-1208
mercaptosteroid 4
mercaptosteroid 6
P2pal-18S
C20-D3-vitamin A
beta2 adrenergic receptor agonist
N6-carboxymethyl-lysine bovine serum albumin
temocapril
methoxyacetic acid
astemizole
ODN1411
phosphorylcholine
triphenyltin
10-hydroxy docosahexaenoic acid
pegaptanib
oseltamivir
K ATP Channel
fosbretabulin
ketotifen
AX15839
10-decarbamoylmitomycin C
nomifensine
idronoxil
4-amino-6-hydrazino-7-beta-D-ribofuranosyl-7H-pyrrolo[2,3-d]-pyrimidine-5-carboxamide
MitoBloCK-6

BRF110
yoda1
potassium tetraperoxochromate
aripiprazole
N(1)-guanyl-1,7-diaminoheptane
NC9
bacitracin
glutathione ethyl ester
tenidap
ellipticine
physostigmine
strychnine
aluminum
SR 31747
danazol
epiallopregnanolone
L-alpha-hydroxyglutarate
miR-450a-1-3p (and other miRNAs w/seed UUGGGAA)
miR-138-1-3p (miRNAs w/seed CUACUUC)
miR-6859-5p (and other miRNAs w/seed AGAGGAA)
miR-3084a-3p (and other miRNAs w/seed UCUGCCA)
miR-580-3p (miRNAs w/seed UGAGAAU)
miR-1264 (and other miRNAs w/seed AAGUCUU)
2-methoxyestradiol
miR-6793-5p (miRNAs w/seed GUGGGUU)
miR-10400-5p (and other miRNAs w/seed GGCGGCG)
TFE3
miR-487a-5p (and other miRNAs w/seed UGGUUAU)
miR-4764-5p (miRNAs w/seed GGAUGUG)
sodium chloride
miR-28-3p (and other miRNAs w/seed ACUAGAU)
miR-1322 (miRNAs w/seed AUGAUGC)
miR-6070 (miRNAs w/seed CGGUUCC)
miR-4669 (miRNAs w/seed GUGUCCG)
miR-6877-5p (miRNAs w/seed GGGCCGA)
miR-6776-3p (miRNAs w/seed AACCACC)
miR-4423-3p (miRNAs w/seed UAGGCAC)
IL17C
miR-1253 (miRNAs w/seed GAGAAGA)
miR-219b-3p (miRNAs w/seed GAAUUGC)
miR-5196-3p (miRNAs w/seed CAUCCUC)
miR-4511 (miRNAs w/seed AAGAACU)
TFAP2C
miR-1247-5p (miRNAs w/seed CCCGUCC)
miR-4746-5p (miRNAs w/seed CGGUCCC)
miR-6843-3p (and other miRNAs w/seed UGGUCUC)
miR-23a-5p (and other miRNAs w/seed GGGUUCC)
FMR1
phosphatidylinositol
CXCL6
Cd209b
Sphk
doramapimod
blinatumomab
PRCP
PIMREG
EIF2A
PTK
CC2D1A
CXCL17
SFRP5
EHT-1864
saracatinib
valine
LAX1
catumaxomab
nadifloxacin
PDGFD
SELL
EZH1
PTPRB
TDO2
CYP4F2
TPSD1
MGLL
CHGA
NKX3-2
TRPA1
ITGB8
FOXN3
PDE4D
BC-1215
CB-5083
hydroxychloroquine
nigericin
zimelidine
poly rI:rC-RNA HMW
diacerein
BAR501
murabutide
geranylgeraniol
vinorelbine
tyrphostin AG825
noladin ether
S-allyl-L-cysteine
miR-6716-5p (miRNAs w/seed GGGAAUG)
miR-7109-3p (miRNAs w/seed AAGCCUC)
miR-6770-3p (miRNAs w/seed UGGCGGC)
miR-6828-3p (miRNAs w/seed UCUGCUC)
miR-3110-5p (and other miRNAs w/seed UCUGCCU)
miR-6502-3p (miRNAs w/seed AGACCAU)
ANXA7
miR-181a-1-3p (and other miRNAs w/seed CCAUCGA)
miR-6894-5p (and other miRNAs w/seed GGAGGAU)
miR-764 (miRNAs w/seed CAGGUGC)
miR-647 (miRNAs w/seed UGGCUGC)
bisphenol A
miR-548q (miRNAs w/seed CUGGUGC)
miR-578 (and other miRNAs w/seed UUCUUGU)
miR-4486 (miRNAs w/seed CUGGGCG)
miR-1227-5p (miRNAs w/seed UGGGGCC)
BMS-690514
HSD11B1
CR1L
miR-3649 (miRNAs w/seed GGGACCU)
miR-4428 (miRNAs w/seed AAGGAGA)
miR-20a-3p (miRNAs w/seed CUGCAUU)
miR-6506-3p (miRNAs w/seed CGUAUCA)
miR-378a-3p (and other miRNAs w/seed CUGGACU)
miR-33-3p (and other miRNAs w/seed AAUGUUU)

**Supplementary Table S22. Unique and overlapping Upstream Regulators for the compassion of the conditions "Caco-2_SARS-CoV" and "Calu-3_SARS-CoV".** Comparative analysis was done using VENNY 2.1. Data visualization was done by R-based Venn diagrams shown in Figure 5.

**1265 elements included exclusively in "Caco-2_SARS-CoV":**
miR-4640-5p (and other miRNAs w/seed GGGCCAG)
EPCAM
EIF3I
abrocitinib
MYCN
FGF19
ENG
miR-324-5p (miRNAs w/seed GCAUCCC)
miR-3473b (and other miRNAs w/seed GGCUGGA)
scoparone
GFAP
myricetin
miR-4749-3p (miRNAs w/seed GCCCCUC)
miR-485-5p (and other miRNAs w/seed GAGGCUG)
miR-6880-5p (miRNAs w/seed GGUGGAG)
miR-92a-2-5p (miRNAs w/seed GGUGGGG)
SFRP4
EDN3
AXIN1
CDK8
GPC3
SGI 1776
R-etodolac
miR-3189-5p (miRNAs w/seed GCCCCAU)
miR-6762-5p (and other miRNAs w/seed GGGGCCA)
CTCF
miR-7108-3p (miRNAs w/seed CCCGCCC)
LTBP4
IGFBP5
diosgenin
miR-194-3p (and other miRNAs w/seed CAGUGGG)
roscovitine
miR-1972 (miRNAs w/seed CAGGCCA)
miR-330-5p (and other miRNAs w/seed CUCUGGG)
Ap2
epoprostenol
AZGP1
miR-4476 (and other miRNAs w/seed AGGAAGG)
SHH
DSCAM
PPP1R1B
chelidonine
ACP1
NSC719239
alpha-methylhydrocinnamic acid
DGCR8
FGF21
miR-4722-3p (and other miRNAs w/seed CCUGCCA)
ARID4B
DNMT3A
HSPA9
miR-4751 (miRNAs w/seed GAGGACC)
miR-6782-5p (and other miRNAs w/seed AGGGGUG)
mir-143
leuprolide
maslinic acid
miR-4316 (miRNAs w/seed GUGAGGC)
OIP5-AS1
ARID4A
PKM
miR-6732-5p (and other miRNAs w/seed AGGGGGU)
LHX1
CSNK2A1
MNK1/2
rigosertib
CLDN2
phloroglucinol
5-hydroxytryptophar
miR-6793-5p (miRNAs w/seed GUGGGUU)
E2F2
miR-4768-3p (and other miRNAs w/seed CAGGAGA)
miR-4687-5p (miRNAs w/seed AGCCCUC)
PRKG1
Stat5 dimer
Collagen type IV
epirubicin
propylthiouracil
miR-8071 (and other miRNAs w/seed GGUGGAC)
embelin
PAX3-FOXO1
miR-6818-5p (miRNAs w/seed UGUGUGA)
L-buthionine (SR)-sulfoximine
captopril
miR-6751-5p (and other miRNAs w/seed UGGGGGU)
miR-3192-5p (miRNAs w/seed CUGGGAG)
Rar
Smad2/3
1,2-dimethylhydrazine
miR-7112-5p (miRNAs w/seed CGGGCAG)
FAM3B
NGFR
KIN001-043
miR-1289 (miRNAs w/seed GGAGUCC)
LDL-cholesterol
TRIM33
fenebrutinib
CIMO
miR-7704 (miRNAs w/seed GGGGUCG)
miR-550a-5p (and other miRNAs w/seed GUGCCUG)
MYBL2
miR-4308 (and other miRNAs w/seed CCCUGGA)
miR-1233-5p (and other miRNAs w/seed GUGGGAG)
TFDP1
ETV4
GRHL2
ETV1
BSCL2
dihydroartemisinin
HOXA4
zeranol
miR-5010-3p (and other miRNAs w/seed UUUGUGU)
miR-1231-3p (miRNAs w/seed GCCCUGU)
histone deacetylase inhibitor
miR-6777-5p (and other miRNAs w/seed CGGGGAG)
miR-6788-3p (miRNAs w/seed UCGCCAC)
phenobarbital
TCF/LEF
SH2D5
AKIRIN2
sphingomyelinase
PDGFD
idelalisib
DAB2
RBX1
EGR4
PDK1
SF3B2
MED13L
PHC2
thenoyltrifluoroacetone
temsirolimus
cyclosporin
rutin
phosphatidic acid
thyroid hormone receptor
CD9
ethionine
miR-1471 (miRNAs w/seed CCCGCGU)
SMO
miR-5196-3p (miRNAs w/seed CAUCCUC)
lonaprisan
Rab11
RPRD1B
CCDC88C
CTNNβ-LEF1
OMG
NOSIP
dibromoacetic acid
mir-153
mir-331
STAMBP
UBE2V1
sorafenib derivative SC-1
3-iodobenzylguanidine
inostamycin
(S)-flurbiprofen
EWSR1-WT1
EPHB4
miR-2861 (and other miRNAs w/seed GGCCCUG)
CEACAM1
PLCG1

**2512 elements included exclusively in "Calu-3_SARS-CoV":**
IFNA2
IFNL1
IRF7
IRF3
Ifnar
Ifn
DDX58
TRIM24
IFNA1/IFNA13
IFNAR2
CpG ODN 2006
IRF5
MAVS
RNY3
PNPT1
STAT2
CNOT7
IRF9
stallimycin
ACKR2
IFNE
NR1H3
JAK
PARP9
CGAS
mir-96
IFNL4
chloroquine
USP18
ribavirin
emactuzumab
NFAT5
GAPDH
IFN alpha/beta
miR-4524a-5p (and other miRNAs w/seed UAGCAGC)
2-aminopurine
SAFB
IFNA4
IL21
IFNK
PLX5622
NLRC5
SAMHD1
ZBED2
miR-503-5p (miRNAs w/seed AGCAGCG)
JAG2
mezerein
mitoxantrone
Irgm1
miR-9-5p (and other miRNAs w/seed CUUUGGU)
miR-3065-5p (and other miRNAs w/seed CAACAAA)
DNASE2
IL37
IRAK1
RIPK2
TAP1
TREX1
MAP2K3
ALB
CCL3
miR-12183-5p (and other miRNAs w/seed CUUCUUC)
FOXM1
let-7c-1-3p (and other miRNAs w/seed UGUACAA)
miR-1468-3p (miRNAs w/seed GCAAAAU)
miR-515-3p (and other miRNAs w/seed AGUGCCU)
IFNA10
IFNA21
IFNA5
IFNA7
IFNA14
IFNA6
2'3'-cyclic guanosine monophosphate-adenosine monophosphate
miR-548v (miRNAs w/seed GCUACAG)
TCF3
salicylic acid
ANKRD42
PRDM16
TNFSF14
miR-5689 (miRNAs w/seed GCAUACA)
IL12B
miR-1284 (and other miRNAs w/seed CUAUACA)
miR-140-5p (and other miRNAs w/seed AGUGGUU)
IFNA8
IFNA16
UM101
GJA1
Pam3-Cys
GnRH analog
MAPK13
BAK1
miR-4693-5p (miRNAs w/seed UACUGUG)
LGALS8
mastoparan
C-miR146a
CEP-1347
miR-153-5p (miRNAs w/seed CAUUUUU)
SHARPIN
EPHX2
let-7a-3p (and other miRNAs w/seed UAUACAA)
BAX
TRIM38
bempedoic acid
TF
desipramine
IL36A
miR-133a-3p (and other miRNAs w/seed UUGGUCC)
anakinra
HSPB1
interferon beta-1a
kawain
MSR1
HSF1
RNASEL
CLEC12A
EGFL7
miR-432 (and other miRNAs w/seed CUUGGAG)
fontolizumab
IKZF3
miR-146a-5p (and other miRNAs w/seed GAGAACU)
ISGF3
Fcor
IFNA17
Fibrinogen
ACKR1
miR-325-5p (and other miRNAs w/seed CUAGUAG)
IFNL3
ritonavir
miR-3660 (and other miRNAs w/seed CUGACAG)
miR-26a-5p (and other miRNAs w/seed UCAAGUA)
LRBA
DPP4
aldesleukin
mir-132
lactosylceramide
PLK2
ACE2
geranylgeranyl pyrophosphate
AZD8055
miR-217-5p (and other miRNAs w/seed ACUGCAU)
ING4
NR1H2
miR-669c-3p (and other miRNAs w/seed ACACACA)
heme
miR-205-5p (and other miRNAs w/seed CCUUCAU)
icilin
DDB2
ARF6
KRT17
BIRC2
taxifolin
miR-4802-3p (and other miRNAs w/seed ACAUGGA)
CASP8
DCN
CD86
IL17a dimer
FGF4
ilomastat
miR-3120 (and other miRNAs w/seed ACAGCAA)
miR-6516-3p (miRNAs w/seed UCAUGUA)
EBI3
VitaminD3-VDR-RXR

**2471 common elements in "Caco-2_SARS-CoV" and "Calu-3_SARS-CoV":**
PDGF BB
U0126
PD98059
beta-estradiol
TP63
EGF
dexamethasone
IL1B
TNF
TGFB1
NFkB (complex)
Mek
HGF
NRG1
ERK
tetradecanoylphorbol acetate
paclitaxel
NR3C1
TP53
HRAS
Vegf
IL2
ESR1
CG
F2
Raf
Salmonella enterica serotype abortus equi lipopolysaccharide
IGF2
IGF1
4-hydroxytamoxifen
CDK19
cisplatin
gefitinib
FGF2
cycloheximide
hydrogen peroxide
INSR
IFNG
KRAS
NUPR1
P38 MAPK
Tgf beta
PRKCD
LY294002
etoposide
RAF1
EGFR
ERK1/2
tretinoin
IRAK4
camptothecin
estrogen receptor
GPER1
poly rI:rC-RNA
RELA
SMARCA4
Pkc(s)
Ige
ERBB2
lipopolysaccharide
D-glucose
F7
AGT
CREBBP
Hdac
triamcinolone acetonide
MAPK3
methylprednisolone
OSM
TREM1
COL18A1
NFKBIA
SP1
RNA polymerase II
bucladesine
NGF
butyric acid
IL1A
PTEN
cocaine
STAT3
tributyrin
JUN
actinomycin D
PGR
SP600125
bicuculline
EDN1
RAS
mifepristone
Jnk
forskolin
dalfampridine
GNAQ
deferoxamine
RASSF1
CREB1
NOTCH1
EP300
THPO
TNFSF11
arsenite
ADRB
FOXO3
anisomycin
SMAD4
5-O-mycolyl-beta-araf-(1->2)-5-O-mycolyl-alpha-araf-(1->1')-glycerol
mycophenolic acid
SB203580
EZH2
FOXL2
CSF2
PI3K (complex)
FSH
WT1
lysophosphatidic acid
FOS
trichostatin A
trovafloxacin
Pdgf (complex)
MYC
STAT5A
TAZ
L-triiodothyronine
AHR
ELK1
androgen
2-amino-5-phosphonovaleric acid
GnRH-A
PTGS2
Growth hormone
EPAS1
HIF1A
progesterone
doxorubicin
5-N-ethylcarboxamido adenosine
romidepsin
prostaglandin E2
Ro31-8220
MAPK14
tunicamycin
evodiamine
leukotriene D4
bee venom
Z-LLL-CHO
5-fluorouracil
MAP2K1
CD40LG
C11orf95-RELA
trans-hydroxytamoxifen
SOX2
GNA15
E. coli B5 lipopolysaccharide
IL6
CREM
thapsigargin
F2R
IL1
raloxifene
BMP2
miR-6752-5p (and other miRNAs w/seed GGGGGUG)

NREP
CGB3 (includes others)
2-acetylaminofluorene
KT5823
MYOC
PLAG1
carboplatin
gemfibrozil
miR-3914 (miRNAs w/seed AGGAACC)
miR-6747-3p (and other miRNAs w/seed CCUGCCU)
miR-343 (and other miRNAs w/seed CUCCCUC)
FOXA2
T3-TR-RXR
miR-7973 (miRNAs w/seed GUGACCC)
PTPN6
DSCAML1
KDM8
ARHGEF1
CXCR1
SMARCA1
NEU3
MM-401
R59949
GW 5074
N,N-dimethylarginine
SAHM1
THY1
DAXX
ethidium
miR-7064-3p (and other miRNAs w/seed AGGGCCC)
SUB1
letrozole
miR-6791-3p (and other miRNAs w/seed GCCUCCU)
miR-661 (miRNAs w/seed GCCUGGG)
miR-615-5p (and other miRNAs w/seed GGGGUCC)
miR-5691 (and other miRNAs w/seed UGCUCUG)
miR-342-5p (and other miRNAs w/seed GGGGUGC)
N(6)-(3-iodobenzyl)-5'-N-methylcarboxamidoadenosine
antimycin A
ADAMTS18
miR-634 (miRNAs w/seed ACCAGCA)
EOMES
miR-3177-3p (miRNAs w/seed GCACGGC)
L-cysteine
bazedoxifene
KDM4B
TPM3
CDKN2B
ST6GAL1
YBX3
MCAM
MPL
USP17L2 (includes others)
cyclopentenone
Focal adhesion kinase
PRKAR1A
NF1
HES1
miR-10394-3p (and other miRNAs w/seed GGGCGCG)
miR-6747-5p (miRNAs w/seed GGGGUGU)
miR-3594-5p (and other miRNAs w/seed CCAGGGC)
prostaglandin E1
ITGA6
olanzapine
miR-92b-5p (miRNAs w/seed GGGACGG)
FLI1
NF2
selenium
miR-6081 (miRNAs w/seed GGAGCAG)
CDKN1B
miR-363-5p (and other miRNAs w/seed GGGUGGA)
miR-3150b-3p (and other miRNAs w/seed GAGGAGA)
miR-4433b-3p (miRNAs w/seed AGGAGUG)
miR-744-5p (and other miRNAs w/seed GCGGGGC)
HOXD3
finasteride
miR-542-5p (miRNAs w/seed CGGGGAU)
omeprazole
sethoxydim
NIBAN2
Trk Receptor
EIF4F
SRGAP1
EMP2
FOXO6
OLIG1
AHNAK
STRAP
TOR2A
YWHAE
CYP2C9
ATAD2
(E)-1-(2-nitrovinyl)naphthalene
(E)-9-(2-nitrovinyl)anthracene
ditiocarb
filipin
2,2-dimethylbutyric acid
rocuronium
betamethasone valerate
4-amino-6-hydrazino-7-beta-D-ribofuranosyl-7H-pyrrolo[2,3-d]-pyrimidine-5-carboxamide
glutathione ethyl ester
CAMK2N2
LINC01139
Shc
SPRY4-IT1
RYBP
SH2B1
ATF5
FUS
BQ-788
miR-3070-5p (and other miRNAs w/seed GCCCCUG)
phosphate
miR-6858-3p (miRNAs w/seed AGCCAGC)
SNAI2
miR-6798-5p (miRNAs w/seed CAGGGGG)
miR-10396b-5p (and other miRNAs w/seed GGCGGGG)
bicalutamide
SOX17
CDC42
LGR5
SMARCE1
PCDH11Y
miR-7154-3p (miRNAs w/seed GGAGGAC)
miR-4437 (miRNAs w/seed GGGCUCA)
miR-7113-3p (miRNAs w/seed CUCCCUG)
miR-3186-5p (miRNAs w/seed AGGCGUC)
miR-1249-3p (and other miRNAs w/seed CGCCCUU)
miR-4440 (miRNAs w/seed GUCGUGG)
miR-4258 (miRNAs w/seed CCCGCCA)
miR-939-3p (miRNAs w/seed CCUGGGC)
miR-3918 (miRNAs w/seed CAGGGCC)
miR-6836-3p (miRNAs w/seed UGCCUCC)
ETS
IgG1
CCN3
L-serine
C9orf72
MCL1
ELK3
H3C14
clonidine
7-(4-fluorobenzylamino)-1,3,4,8-tetrahydropyrrolo[4,3,2-de]quinolin-8(1H)-one
SS18-SSX2
delphinidin
hesperetin
miR-129-5p (and other miRNAs w/seed UUUUUGC)
miR-5131 (and other miRNAs w/seed GGCGCCC)
COLQ
PNPLA2
CDX2
CCN2
miR-6724-5p (and other miRNAs w/seed UGGGCCC)
alvespimycin
Fibrin
WDR5
HTATIP2
CCAR2
CUL3
IGFBP2
miR-4746-5p (miRNAs w/seed CGGUCCC)
miR-4292 (and other miRNAs w/seed CCCUGGG)
CDK4/6
miR-6753-5p (miRNAs w/seed ACCAGGG)
miR-4537 (miRNAs w/seed GAGCCGA)
dovitinib
UCN2
USP4
FCGR1A
etanercept
CORT
miR-3691-5p (miRNAs w/seed GUGGAUG)
miR-875-5p (and other miRNAs w/seed AUACCUC)
FIRRE
TRIM8
dacarbazine
miR-95-5p (miRNAs w/seed CAAUAAA)
GW9662
IFNW1
NCD-38
(1S,2R)-NCL-1
USP19
SIRPA
brefeldin A
enterotoxin B
lenalidomide
miR-144-3p (miRNAs w/seed ACAGUAU)
miR-4668-5p (miRNAs w/seed GGGAAAA)
miR-5694 (miRNAs w/seed AGAUCAU)
6-mercaptopurine
belinostat
RNASEH2B
PARP14
B4GALT6
TRAF1
TPCA-1
let-7a-2-3p (and other miRNAs w/seed UGUACAG)
ACOD1
grepafloxacin
INSIG1
miR-21-5p (and other miRNAs w/seed AGCUUAU)
miR-4671-3p (miRNAs w/seed UAGUGCA)
ITGB2
IL1RAP
Tnfsf9
miR-4715-5p (miRNAs w/seed AGUUGGC)
BTNL2
GNAI3
edaravone
miR-6733-3p (and other miRNAs w/seed CAGUGUC)
CTLA4
miR-6730-3p (miRNAs w/seed CUGACAC)
FCGR2B
pristane
ARID1A
miR-4727-3p (miRNAs w/seed UAGUGGG)
miR-3686 (and other miRNAs w/seed UCUGUAA)
miR-4672 (miRNAs w/seed UACACAG)
ANXA1
miR-5135 (and other miRNAs w/seed GGUCUAG)
TAC1
vismodegib
suramin
miR-365-3p (and other miRNAs w/seed AAUGCCC)
miR-4714-3p (miRNAs w/seed CAACCUA)
NGLY1
CpG ODN 1826
CD3E
LCN2
Hsp70
miR-628-3p (miRNAs w/seed CUAGUAA)
FCER1G
Collagen(s)
miR-27b-5p (miRNAs w/seed GAGCUUA)
glycine
PAEP
NCR2
melphalan
ORMDL3
MAPK12
GADD45A
fingolimod phosphate
PADI4
ARID3B
cyanidin 3-O-glucoside
FEM1A
BLM
AGI-1067
levofloxacin
huperzine A
HOXA-AS2
nandrolone
miR-374c-3p (miRNAs w/seed ACUUAGC)
miR-374c-5p (and other miRNAs w/seed UAAUACA)
CARD9
IDR-1002
RGS10
miR-8065 (miRNAs w/seed GUAGGAA)
miR-4719 (miRNAs w/seed CACAAAU)
H2AB3 (includes others)
miR-4460 (miRNAs w/seed UAGUGGU)
miR-328-5p (and other miRNAs w/seed GGGGGGC)
miR-155-3p (miRNAs w/seed UCCUACA)
IFNGR1
CRP
PLK4
miR-1200 (miRNAs w/seed UCCUGAG)
miR-548an (miRNAs w/seed AAAGGCA)
IL18R1
BTC
IRF6
miR-4324 (miRNAs w/seed CCUGAGA)
SMAD2
miR-6980-5p (and other miRNAs w/seed UGGGGGG)
BACH2
CREBZF
ZBP1
thiazolidinedione
miR-4480 (miRNAs w/seed GCCAAGU)
miR-582-3p (miRNAs w/seed AACUGGU)
KLF3
miR-630 (miRNAs w/seed GUAUUCU)
miR-544-3p (and other miRNAs w/seed UUCUGCA)
LTA
APOA1
3M-002
fingolimod
CpG ODN 2216
miR-1250-3p (miRNAs w/seed CAUUUUC)
PIK3CG
bromobenzene
LECT2
RNF216
IL17RA
miR-150-3p (miRNAs w/seed UGGUACA)
C3
8-hydroxyguanine
CERS5
RNU1-1
FLT4
NLRP11
TPR
entolimod
FSTL3
SH2B3
STK38
coxsackievirus A21
PK11007
miR-6828-5p (miRNAs w/seed GGAAGCA)
miR-1306-5p (miRNAs w/seed CACCUCC)
resolvin D2
miR-4659a-5p (and other miRNAs w/seed UGCCAUG)
miR-135a-5p (and other miRNAs w/seed AUGGCUU)
RUNX1
CNR1
TLR1
DPH5
MAPKAP1
10-nitrooleate
IRAK2
tranylcypromine
BLVRA
mir-873
NELFA
UBA7
raltitrexed
arachidic acid
miR-545-3p (miRNAs w/seed CAGCAAA)
ATG5
dicarbethoxydihydrocollidine
Collagen type II
green tea polyphenol
SERPINE2
alefacept
miR-4659a-3p (and other miRNAs w/seed UUCUUCU)
F3
MAPK8
AG490
Ca2+
nitric oxide
kainic acid
GLI3
IKBKB
EGR1
Sb202190
PPARG
GNA14
Histone h3
curcumin
miR-4716-3p (and other miRNAs w/seed AGGGGGA)
seocalcitol
ECSIT
MAPK9
SU6656
pyrrolidine dithiocarbamate
MAPK1
epigallocatechin-gallate
MAP3K1
AKT1
CRNDE
cytokine
decitabine
CTNNB1
SP3
PI3K (family)
tamoxifen
LDL
MAPK7
miR-4651 (and other miRNAs w/seed GGGGUGG)
BMP4
CEBPB
ESR2
triptolide
N-acetyl-L-cysteine
Akt
herbimycin
MAP2K4
atorvastatin
troglitazone
FOXO1
bexarotene
glutamine
TGFA
DACH1
salmonella minnesota R595 lipopolysaccharides
Alpha catenin
MET
zerumbone
THZ2
TAF4
2-(4-amino-1-isopropyl-1H-pyrazolo[3,4-d]pyrimidin-3-yl)-1H-indol-5-ol
calcitriol
POU5F1
SRC
VEGFA
cobalt chloride
MAP3K8
ITGB1
BCL3
IgG
ethanol
MYD88
NFKB1
topotecan
FADD
BDNF
SMAD3
platelet activating factor
TLR4
BRD4
MAC
ATF4
capsazepine
noscapine
growth factor
CSF3
ACVRL1
INS
MAP2K1/2
MYB
FAS
CCL2
Ngf
resveratrol
NCOR2
miR-3151-5p (and other miRNAs w/seed GUGGGGC)
OGA
filgrastim
lactacystin
erlotinib
selumetinib
staurosporine
1'-acetoxychavicol acetate
estrogen
MRTFB
miR-149-3p (and other miRNAs w/seed GGGAGGG)
dihydrotestosterone
SRF
salinosporamide A
REL
NDRG1
BAPTA-AM
gentamicin
5-azacytidine
TP73
Creb
PTH
reactive oxygen species
PDX1
SYK
pimagedine
tyrphostin AG 1478
H1-2
crocidolite asbestos
EIF2AK2
CDKN2A
lipoarabinomannan
CCN1
IRF2
Ap1
deoxycholate
miR-4667-5p (and other miRNAs w/seed CUGGGGA)
TICAM1
miR-3072 (and other miRNAs w/seed GCCCCCU)
BMP7
H-7
vorinostat
ERBB3
sodium arsenite
miR-6825-5p (and other miRNAs w/seed GGGGAGG)
DUSP1
HOXA5
let-7a-5p (and other miRNAs w/seed GAGGUAG)
EPHB1
ST3-Hel2A-2
tetrachlorodibenzodioxin
FN1
CHUK
NEDD4
RAC1
simvastatin
Cdk
VTN
miR-12192-5p (and other miRNAs w/seed GUGGGGU)
Insulin
IL17A
miR-1909-3p (and other miRNAs w/seed GCAGGGG)
DDIT3
oleic acid
R5020
tosylphenylalanyl chloromethyl ketone
MMP1
ATF3
IL5
thioacetamide
LIF
miR-29b-3p (and other miRNAs w/seed AGCACCA)
methyl methanesulfonate
baicalein
IL4
SMAD7
Ins1

EFNB1
FOXD3
ITGA3
BAG3
miR-6781-5p (miRNAs w/seed GGGCCGG)
GATA2
WNT7A
COL4A3
TGFB1I1
LCAT
miR-1976 (and other miRNAs w/seed CUCCUGC)
miR-122-5p (miRNAs w/seed GGAGUGU)
2,4,5,2',4',5'-hexachlorobiphenyl
CGP 74514A
6-dimethylaminopurine
PD 180970
Esr1-Esr1-estrogen-estrogen
benoxaprofen
TEX11
XRN2
DACT3
Sapk
BIIB021
firtecan pegol
ZNF467
CDC25B
ARTN
PHLDA1
anatabine
NME2
CHRM4
RGD1560225
SNRK
CDC20
SLC9A3R2
PODXL
WTAP
carboxyamido-triazole
hoechst 33342
lansoprazole
clomiphene
CCI-007
C25-140
yohimbine
galanthamine
tyrphostin AG 18
Co2+
miR-1225-3p (miRNAs w/seed GAGCCCC)
SFRP1
3-methylcholanthrene
SLC13A1
CHD7
E2F5
SSTR2
elaidic acid
miR-891a-5p (miRNAs w/seed GCAACGA)
digoxin
CSNK2B
FGF23
EN1
ALCAM
astressin 2B
PAX5
miR-3975 (miRNAs w/seed GAGGCUA)
miR-187-3p (miRNAs w/seed CGUGUCU)
miR-4270 (and other miRNAs w/seed CAGGGAG)
PHF1
AKT3
HDAC9
zoledronic acid
miR-344a-5p (and other miRNAs w/seed CAGGCUC)
PIM1
RORC
miR-1915-3p (and other miRNAs w/seed CCCAGGG)
miR-6862-3p (and other miRNAs w/seed CUCACCC)
miR-6845-3p (and other miRNAs w/seed CUCUCCU)
5'-methylthioadenosine
PTBP1
MSX2
ASXL1
miR-4314 (and other miRNAs w/seed UCUGGGA)
miR-887-3p (miRNAs w/seed UGAACGG)
lithocholic acid
BMP
miR-1228-3p (miRNAs w/seed CACACCU)
NOTCH3
miR-6846-3p (miRNAs w/seed GACCCCU)
SLC27A2
mir-150
RPS6KB1
miR-184 (and other miRNAs w/seed GGACGGA)
miR-6728-3p (miRNAs w/seed CUCUGCU)
ILX-23-7553
Mst/krs
Cpla2
(-)-gossypol
aurapten
RAR-RXR
MELK
KLF8
MTF2
ZMIZ2
Gata
UTP18
GPR68
Vla-4
TAF9
carvacrol
CSF
Pik3r
MAP4K1
FZD7
MCF2
RNF34
TLN1
RBM14
WNK4
PSAP
CDH4
NME3
SH2B2
STK17B
lindane
atrasentan
dimaprit
Collagen type V
butylphen
cannabinoid drugs
NADPH
mono-(2-ethylhexyl)phthalate
imipramine
NME1
cyclic GMP
ADP
NSD1
IGFBP7
APBB1
mir-322
mir-196
CSK
PER2
VEGFC
HOXB4
SP2
TGFBR3
PTGFR
hydroxyflutamide
zinc protoporphyrin IX
NUP98-KDM5A
NUP98-NSD1
miR-512-5p (miRNAs w/seed ACUCAGC)
miR-4706 (and other miRNAs w/seed GCGGGGA)
dehydroisoandrosterone
miR-216b-3p (and other miRNAs w/seed CACACUU)
miR-4266 (miRNAs w/seed UAGGAGG)
miR-3960 (and other miRNAs w/seed GCGGCGG)
miR-128-1-5p (and other miRNAs w/seed GGGGCCG)
ROCK1
PTCH1
PSEN2
miR-5192 (miRNAs w/seed GGAGAGU)
miR-1188-3p (and other miRNAs w/seed GAGGCUC)
miR-759 (miRNAs w/seed CAGAGUG)
miR-4281 (miRNAs w/seed GGUCCCG)
miR-4428 (miRNAs w/seed AAGGAGA)
miR-1234-3p (and other miRNAs w/seed CGGCCUG)
miR-6872-3p (miRNAs w/seed CCAUGCC)
phytohemagglutinin
miR-6835-3p (miRNAs w/seed AAAGCAC)
IL36G
amitriptyline
miR-23a-3p (and other miRNAs w/seed UCACAUU)
miR-337-3p (miRNAs w/seed UCCUAUA)
miR-6719-3p (miRNAs w/seed CUGACAU)
miR-5188 (miRNAs w/seed AUCGGAC)
miR-376b-5p (and other miRNAs w/seed GUGGAUA)
TRIM28
miR-17-3p (and other miRNAs w/seed CUGCAGU)
PAX3
miR-4291 (and other miRNAs w/seed UCAGCAG)
miR-1-5p (and other miRNAs w/seed CAUACUU)
estriol
miR-3692-3p (miRNAs w/seed UUCCACA)
treprostinil
HECTD3
ASCC1
TRIL
UFD1
NPLOC4
CD163
BICD2
LPAR3
VR23
vapreotide
IgG-opsonized ovalbumin
WWTR1
prostaglandin D2
fucoidin
mir-223
CYP2J2
ADORA3
2-mercaptoacetate
tranilast
FSTL1
B4GALNT1
RPL22
6-aminopyrazolopyrimidine derivative compound II
ITGAM
miR-3166 (miRNAs w/seed GCAGACA)
Foxo
triclosan
FZD9
IL-17f dimer
LBP
LAMA5
TNK1
phenyl-N-tert-butylnitrone
miR-34a-3p (miRNAs w/seed AAUCAGC)
budesonide
miR-7150 (miRNAs w/seed UGGCAGG)
miR-331-5p (and other miRNAs w/seed UAGGUAU)
miR-2116-3p (miRNAs w/seed CUCCCAU)
miR-4444 (miRNAs w/seed UCGAGUU)
TICAM2
arotinoid acid
alpha-tocopherol
SGPP2
tripterine
miR-183-5p (miRNAs w/seed AUGGCAC)
AIRE
clodronic acid
miR-138-1-3p (miRNAs w/seed CUACUUC)
miR-3171 (miRNAs w/seed GAUGUAU)
F630028O10Rik
ANKRD17
rhesus theta-defensin 1
SELE
LUM
CTTN
poly(U)RNA
miR-223-3p (miRNAs w/seed GUCAGUU)
NOX1
cuprizone
ezetimibe
miR-3687 (and other miRNAs w/seed CCGGACA)
ATF6
miR-3907 (miRNAs w/seed GGUGCUC)
miR-3682-5p (miRNAs w/seed UACUUCU)
Sphk
bis(3',5')-cyclic diguanylic acid
poly(dG-dC)
iguratimod
HLA-DQ
REV1
MARCO
ISG15
CLIC4
C4A/C4B
Ptgs2os2
cepharanthine
BC-1215
Immunoglobulin Lambda Light Chain
ubiquinone 9
lemairamin
polyphosphate
borrelia burgdorferi strain B31 peptidoglycan
miR-10a-5p (and other miRNAs w/seed ACCCUGU)
PEBP1
miR-4477b (miRNAs w/seed UUAAGGA)
HCAR2
OPA1
IL17R
superoxide
sertraline
miR-4999-3p (miRNAs w/seed CACUACC)
GAS6
MALT1
miR-2681-3p (miRNAs w/seed AUCAUGG)
Ahr-aryl hydrocarbon-Arnt
Collagen Alpha1
RALA
dimethyl fumarate
coomassie brilliant blue
picryl chloride
spermidine
spermine
miR-587 (miRNAs w/seed UUCCAUA)
miR-141-5p (and other miRNAs w/seed AUCUUCC)
IKZF2
mir-10
miR-3169 (miRNAs w/seed AGGACUG)
oxaliplatin
JAK1/2
n-3 fatty acids
miR-5700 (miRNAs w/seed AAUGCAU)
miR-6509-3p (miRNAs w/seed UCCACUG)
miR-3063-5p (and other miRNAs w/seed GGAGAUC)
PPARD
miR-648 (miRNAs w/seed AGUGUGC)
CXCR2
miR-6499-3p (miRNAs w/seed GCAGUGU)
ASPSCR1-TFE3
KDM2B
dimethyl sulfoxide
miR-146a-3p (and other miRNAs w/seed CUCUGAA)
CCR1
hydroxypropyl-beta-cyclodextrin
MMP14
LRP5
miR-548ao-5p (and other miRNAs w/seed GAAGUAA)
MOG
miR-5003-5p (miRNAs w/seed CACAACA)
miR-600 (miRNAs w/seed CUUACAG)
DPP-23
DYSF
vitamin D
miR-103-3p (and other miRNAs w/seed GCAGCAU)
KDM3B
ELANE
U73122
Traj18
miR-6509-5p (miRNAs w/seed UUAGGUA)
miR-4771 (miRNAs w/seed GCAGACU)
miR-3117-3p (miRNAs w/seed UAGGACU)
miR-4742-5p (miRNAs w/seed CAGGCAA)
TCF12
miR-4666a-3p (miRNAs w/seed AUACAAU)
monophosphoryl lipid A
amphotericin B
nystatin
Nos
IFNL2
ZBTB33
mir-140
VHL
PD173074
Gsk3
miR-637 (and other miRNAs w/seed CUGGGGG)
miR-6756-5p (and other miRNAs w/seed GGGUGGG)
15-deoxy-delta-12,14 -PGJ 2
CIC
VDR
CD24
KCNE3
calphostin C
CSF1
JAK2
miR-4271 (and other miRNAs w/seed GGGGAAG)
PLAU
SS18
ceramide
FGFR1
CD36
miR-6929-5p (and other miRNAs w/seed GGGAGGA)
TLR9
methotrexate
miR-4731-5p (miRNAs w/seed GCUGGGG)
CNTF
TLR3
FGF1
miR-762 (and other miRNAs w/seed GGGCUGG)
SPHK1
ADIPOQ
ELK4
miR-3083-5p (and other miRNAs w/seed GGCUGGG)
HNF1A
aspirin
PTHLH
miR-874-5p (miRNAs w/seed GGCCCCA)
MYOD1
cyclosporin A
cyclic AMP
TERT
palmitic acid
miR-4723-5p (and other miRNAs w/seed GGGGGAG)
AGER
ATP
PAF1
Lh
methylnitronitrosoguanidine
fludarabine
IL33
methylselenic acid
hydrocortisone
S100A8
CARM1
CEBPA
ERBB4
KLF2
SIN3A
pyruvic acid
Nfat (family)
E2F1
bardoxolone methyl
NCOA3
Tnf (family)
CXCL12
PF4
miR-1273h-5p (and other miRNAs w/seed UGGGAGG)
APP
napabucasin
wortmannin
carrageenan
NORAD
TLR7/8
benzyl isothiocyanate
APEX1
BCR-ABL1
POU2F1
JQ1
S100A9
TCF4
PRKCE
miR-6829-5p (miRNAs w/seed GGGCUGC)
NRG2
PTGER4
H89
lonafarnib
GNA13
TCF
YAP1
KDM3A
JUNB
SNAI1
IL3
PTPN11
KLF6
sphingosine-1-phosphate
RARA
IKBKG
vitamin K3
TSH
pregna-4,17-diene-3,16-dione
TFPI2
CX-5461
PD184352
caffeic acid phenethyl ester
DETA-NONOate
KLF4
KMT2A
ATF2
EIF2AK3
EHMT1
STAT5B
TBP
quercetin
miR-6887-3p (miRNAs w/seed CCCCUCC)
okadaic acid
oblimersen
CDK9
carbonyl cyanide m-chlorophenyl hydrazone
caffeine
Ikb
NTRK1
LEP
MACROH2A1
miR-1913 (miRNAs w/seed CUGCCCC)
ID2
bisindolylmaleimide I
SPDEF
HDAC3
PC-SPES
ANXA2
fisetin
NQO2
ZMPSTE24
MAP2K7
luteolin
JAK1
WNT3A
plumbagin
piceatannol
PP2/AG1879 tyrosine kinase inhibitor
CCND1
ID3
TEAD4
cigarette smoke
leukotriene C4
IKK (complex)
formaldehyde
fulvestrant
N-acetylsphingosine
miR-491-5p (and other miRNAs w/seed GUGGGGA)
MRTFA
THRB
BMP6
NCOA1
miR-6967-5p (and other miRNAs w/seed AGGGAGG)
pirinixic acid
miR-5008-5p (miRNAs w/seed GAGGCCC)
PRKAG3
lysophosphatidylcholine
ATM
2-deoxyglucose
amphetamine
histamine
E. coli serotype 0127B8 lipopolysaccharide
CDC73
SMAD1
SB 216763
phorbol 12,13-dibutyrate

Wnt
IGFBP1
IL2RB
BHLHA15
STMN1
4-nitroquinoline-1-oxide
Hif
miR-466d-5p (and other miRNAs w/seed GUGUGUG)
TCF7L1
FGFR4
LTB4R
miR-6741-3p (miRNAs w/seed CGGCUCU)
miR-3116 (miRNAs w/seed GCCUGGA)
miR-4787-5p (and other miRNAs w/seed CGGGGGU)
miR-3151-3p (miRNAs w/seed CUGAUCC)
MTA1
miR-3135a (miRNAs w/seed GCCUAGG)
CDH1
SRSF1
MECOM
histidinol
P110
LINC00842
CCAR1
ZMIZ1
ANKHD1/ANKHD1-EIF4EBP3
Crnde
PIWIL4
BPIFB1
Ap2 alpha
lumefantrine
GSK690693
PRKD2
PLSCR1
BAD
BCL11A
Pzp
ETV3
RYK
LRIG1
OXSR1
STX2
TFF2
YWHAB
EPHA4
HIVEP2
MATK
SMARCD1
Hmgb2 (includes others)
BCL2A1
ZYX
2-morpholin-4-yl-6-thianthren-1-yl-pyran-4-one
PDZ1i
MBD2
MAFB
HNRNPK
MXI1
MAZ
SMAD6
decursin
glutamyl-Se-methylselenocysteine
GATA6
miR-658 (miRNAs w/seed GCGGAGG)
miR-673-3p (and other miRNAs w/seed CCGGGGC)
miR-6809-5p (miRNAs w/seed GGCAAGG)
miR-1199-5p (and other miRNAs w/seed CUGAGCC)
TET3
SOX10
betulinic acid
miR-4648 (miRNAs w/seed GUGGGAC)
miR-3126-5p (and other miRNAs w/seed GAGGGAC)
miR-6853-5p (miRNAs w/seed GCGUGGG)
miR-6854-3p (miRNAs w/seed GCGUUUC)
KMT2D
miR-4522 (and other miRNAs w/seed GACUCUG)
miR-5589-5p (miRNAs w/seed GCUGGGU)
laquinimod
miR-508-3p (miRNAs w/seed GAUUGUA)
PPP4R3B
ARHGDIG
PDGFRA
AKR1B1
CD22
XPO1
YY2
ellagic acid
latrunculin B
acyclic retinoid
piperine
REST
WNT4
miR-3176 (and other miRNAs w/seed CUGGCCU)
miR-711 (miRNAs w/seed GGACCCA)
miR-879-5p (and other miRNAs w/seed GAGGCUU)
miR-4681 (miRNAs w/seed ACGGGAA)
miR-802-3p (and other miRNAs w/seed CGGAGAG)
ROCK2
miR-4297 (and other miRNAs w/seed GCCUUCC)
miR-3917 (miRNAs w/seed CUCGGAC)
phosphatidylinositol
MRPS18B
C1orf61
BCAR4
QRFP
Collagen type III
ASCL2
DVL1
RAP1GAP
CCN4
PTPRB
CBR3-AS1
LHB
WNK1
STRA6
ENO1
PDE3A
TDO2
BANCR
GABBR2
MAD2L2
MLLT3
TNKS
PGK1
aminoglutethimide
volinanserin
zimelidine
retroinverso ERG inhibitory peptide 2
retroinverso ERG inhibitory peptide 1
miR-124 mimic
geranylgeraniol
acolbifene
GAB2
RXRG
PAK1
miR-5113 (and other miRNAs w/seed CAGAGGA)
TMSB4
RHOJ
SULF2
DAG1
CTBP2
thioridazine
miR-2278 (miRNAs w/seed AGAGCAG)
FSHR
CDX1
miR-483-5p (miRNAs w/seed AGACGGG)
miR-4286 (miRNAs w/seed CCCCACU)
miR-3937 (miRNAs w/seed CAGGCGG)
miR-504-5p (and other miRNAs w/seed GACCCUG)
miR-6776-5p (miRNAs w/seed CUGGGUG)
miR-6814-3p (and other miRNAs w/seed CUCGCAU)
ESRRA
miR-4743-5p (miRNAs w/seed GGCCGGA)
miR-1281 (miRNAs w/seed CGCCUCC)
miR-6827-5p (miRNAs w/seed GGGAGCC)
IAPP
EML4-ALK
naloxone
miR-551b-3p (and other miRNAs w/seed CGACCCA)
miR-3649 (miRNAs w/seed GGGACCU)
miR-6772-3p (miRNAs w/seed UGCUCCU)
miR-6877-5p (miRNAs w/seed GGGCCGA)
miR-4710 (miRNAs w/seed GGUGAGG)
CD5
enalapril
rhodamine 6G
HJV
DUOXA1
lauric acid
miR-548e-5p (miRNAs w/seed AAAAGCA)
H1-6
H1f1
ADCY
STK4
SAFB2
oxazolone
galactosylceramide-alpha
miR-8086 (miRNAs w/seed GCUAGUC)
miR-374b-3p (miRNAs w/seed UUAGCAG)
miR-888-5p (miRNAs w/seed ACUCAAA)
SIRT6
CLEC7A
B2M
MMP3
miR-570-3p (miRNAs w/seed GAAAACA)
miR-30c-1-3p (and other miRNAs w/seed UGGGAGA)
miR-452-5p (and other miRNAs w/seed ACUGUUU)
miR-10394-5p (and other miRNAs w/seed CUGCAGG)
miR-7705 (miRNAs w/seed AUAGCUC)
miR-4655-5p (miRNAs w/seed ACCGGGG)
trinitrobenzenesulfonic acid
Cyp4a14
Tlr13
FANCA
COPA
TAPBP
teasaponin
ULBP1
vadimezan
mechlorethamine
theaflavin
miR-6792-5p (miRNAs w/seed UAAGCAG)
puromycin
ssRNA40
miR-549a-3p (miRNAs w/seed GACAACU)
eritoran
1-palmitoyl-2-oleoylglycero-3-phosphoglycerol
MARCHF3
SPINDOC
Tlr12
FGA
SPIN1
ARL5B
PLAA
polyinosine-polycytidylic acid/polyethylenimine formulation
sulfamethoxazole/trimethoprim
3-hydroxybutyric acid
tranexamic acid
roflumilast
paxilline
sappanone A
allosamidin
miR-377-3p (miRNAs w/seed UCACACA)
miR-132-3p (and other miRNAs w/seed AACAGUC)
miR-101b-3p (miRNAs w/seed UACAGUA)
PLAAT4
miR-192-5p (and other miRNAs w/seed UGACCUA)
miR-4752 (miRNAs w/seed UGUGGAU)
SERPINA1
miR-219a-5p (and other miRNAs w/seed GAUUGUC)
miR-105-5p (and other miRNAs w/seed CAAAUGC)
miR-130b-5p (and other miRNAs w/seed CUCUUUC)
leukotriene B4
GABA
LPIN1
miR-34a-3p (and other miRNAs w/seed AUCAGCA)
inosine
misoprostol
IL1RL2
UNC93B1
PFKFB3
IGHE
IFT57
CDK6
VIPR1
101.10 peptide
concanamycin A
erythromycin
miR-6507-3p (miRNAs w/seed AAAGUCC)
miR-6777-3p (miRNAs w/seed CCACUCU)
SRT1720
LILRB4
PIK3R2
OGG1
miR-208a-3p (and other miRNAs w/seed UAAGACG)
miR-4456 (and other miRNAs w/seed CUGGUGG)
miR-203b-5p (and other miRNAs w/seed AGUGGUC)
miR-499-5p (and other miRNAs w/seed UAAGACU)
miR-10400-5p (and other miRNAs w/seed GGCGGCG)
miR-148b-5p (and other miRNAs w/seed AGUUCUG)
miR-292b-5p (and other miRNAs w/seed CUCAAAA)
C3AR1
miR-2052 (miRNAs w/seed GUUUUGA)
miR-383-5p (miRNAs w/seed GAUCAGA)
miR-1953 (and other miRNAs w/seed GGGAAAG)
miR-26b-3p (and other miRNAs w/seed CUGUUCU)
RNASE1
CCL4
Mir200
LIPG
ST8SIA1
chrysin
miR-624-3p (miRNAs w/seed ACAAGGU)
HISTONE
CFB
CCR5
miR-4638-3p (and other miRNAs w/seed CUGGACA)
miR-3682-3p (miRNAs w/seed GAUGAUA)
miR-627-3p (miRNAs w/seed CUUUUCU)
miR-6079 (miRNAs w/seed UGGAAGC)
miR-6763-3p (miRNAs w/seed UCCCCGG)
miR-3183 (and other miRNAs w/seed CCUCUCU)
miR-499-3p (and other miRNAs w/seed ACAUCAC)
ABCA1
miR-490-5p (miRNAs w/seed CAUGGAU)
AICAR
PTPN1
miR-875-3p (miRNAs w/seed CUGGAAA)
miR-548av-5p (and other miRNAs w/seed AAAGUAC)
miR-4755-5p (and other miRNAs w/seed UUCCCUU)
IFIT2
cerulenin
clenbuterol
miR-5094 (miRNAs w/seed AUCAGUG)
miR-8081 (miRNAs w/seed UUGAGUC)
miR-568 (miRNAs w/seed UGUAUAA)
cholecalciferol
miR-4501 (and other miRNAs w/seed AUGUGAC)
NOS2
miR-4740-5p (miRNAs w/seed GGACUGA)
docetaxel
miR-93-3p (miRNAs w/seed CUGCUGA)
N-(3-(aminomethyl)benzyl)acetamidine
16,16-dimethylprostaglandin E2
tocilizumab
CpG ODN 1585
HTR7
salmonella typhimurium lipopolysaccharide
PI3
NEK6
ANPEP
CTH
cyclic guanosine monophosphate-adenosine monophosphate
BQ 123
L-N6-(1-iminoethyl)-lysine
miR-3119 (miRNAs w/seed GGCUUUU)
miR-513a-5p (miRNAs w/seed UCACAGG)
MAPK8IP1
XRCC6
TLR6
P2RX7
aphidicolin
BCAP31
SL 327
PLA2G2D
PTGDR
PON1
CCL3L3
HPSE
hydroquinone
Complement
1-chloro-2-(2,2,2-trichloro-1-(4-chlorophenyl)ethyl)benzene
spermine nitric oxide complex
[D-Ala2,N-Me-Phe4,Gly5-ol]-Enkephalin
palmitoleic acid
PKD1
GH1
NFYA
Calcineurin protein(s)
genistein
S-nitroso-N-acetyl-DL-penicillamine
PI-103
mir-154
PPRC1
NUP98-DDX10
AFP
TFRC
TGFBR1
HDAC1
rosiglitazone
26s Proteasome
CISH
XDH
ARRB2
STAT4
MAX
NFATC2
CASR
SERCA
CXCR4
miR-101-3p (and other miRNAs w/seed ACAGUAC)
GATA3
RARG
thalidomide
NR3C2
bortezomib
ACTN4
miR-17-5p (and other miRNAs w/seed AAAGUGC)
IL13
ZFP36
STK11
mir-8
OSMR
Pam3-Cys-Ser-Lys4
MEOX2
tacrolimus
IL10RA
PLK1
MAP2K5
9,10-dimethyl-1,2-benzanthracene
vancomycin
PRKCA
3-deazaneplanocin
Immunoglobulin
LIPE
bleomycin
ABL1
DUSP5
ANGPT2
PDGFB
lomustine
25-hydroxycholesterol
MDM2
IFI16
rottlerin
10E,12Z-octadecadienoic acid
PDLIM2
STAT5a/b
C5
berberine
IL15
miR-1914-3p (and other miRNAs w/seed GAGGGGU)
BRAF
isobutylmethylxanthine
nelfinavir
EGLN1
miR-125b-5p (and other miRNAs w/seed CCCUGAG)
PRKDC
peptidoglycan
PP1
AGTR1
NRAS
RUNX3
DICER1
CXCL8
mir-17
MAP3K14
POLR2A
glutathione
cytarabine
CHRM1
TLE1
VEGFD
PTGER1
gambogic acid
tazemetostat
ABT-737
PD 153035
TMPRSS2-ERG
EIF2S1
iron
AR
miR-6132 (and other miRNAs w/seed GCAGGGC)
NFKB2
phenacetin
AGR2
PROK1
ADCYAP1R1
NFE2L2
bufalin
miR-4758-3p (miRNAs w/seed GCCCCAC)
Igm
HMGN2
2-amino-3-phosphonopropionic acid
CD40
hyaluronic acid
CAT
JAG1
apigenin
IRS1
WNT5A
CRH
HMGA1
Laminin (complex)
EED
pituitary adenylate cyclase-activating polypeptide
D-galactosamine
calcimycin
clofibrate
Interferon alpha
RPS6KA5
PTPN3
ketamine
PRL
collagenase
KN 93
PTPRJ
FGFR2
miR-423-5p (and other miRNAs w/seed GAGGGGC)
resiquimod
TGFB2
miR-3943 (miRNAs w/seed AGCCCCC)
miR-4290 (miRNAs w/seed GCCCUCC)
indomethacin
POMC
miR-4472 (and other miRNAs w/seed GUGGGGG)
ITGB3
sulindac sulfide
miR-5586-5p (miRNAs w/seed AUCCAGC)
PIK3R1
TCR
TNFSF10
diethylstilbestrol
SP2509
Pkg
HBEGF
miR-709 (and other miRNAs w/seed GAGGCAG)
paraquat
MEF2D
silibinin
ionomycin
TFAP2A
SMARCB1
miR-2682-5p (and other miRNAs w/seed AGGCAGU)
ARRB1
daunorubicin
chenodeoxycholic acid
AREG
NEDD9
miR-5193 (miRNAs w/seed CCUCCUC)
RBCK1
aplidine

SULT2B1
ZNF382
CYSLTR2
parathyroid hormone-related protein
LGI1
RGS19
GAL3ST1
GRIP1
MAOA
HEYL
WNT7B
GRB10
SNW1
PROKR1
CCDC80
PCBP1
proteasome inhibitor
naproxen
NCX-4040
pranlukast
vandetanib
artemisinin
citric acid
cordycepin
Bvht
PTTG1
TFAP2C
miR-760-3p (and other miRNAs w/seed GGCUCUG)
miR-370-5p (and other miRNAs w/seed AGGUCAC)
miR-4650-5p (miRNAs w/seed CAGGCCU)
miR-4724-3p (and other miRNAs w/seed UACCUUC)
DNA-methyltransferase
YWHAZ
FBN1
HDAC7
NTN1
PPP4R3A
SCGB1A1
nickel
miR-505-3p (miRNAs w/seed UCAACAC)
miR-338-3p (miRNAs w/seed CCAGCAU)
cholic acid
CLEC11A
miR-675-5p (and other miRNAs w/seed GGUGCGG)
SERPINE1
miR-7106-3p (miRNAs w/seed GCUCCCU)
miR-6859-3p (miRNAs w/seed GACCCCC)
miR-6769a-3p (miRNAs w/seed AGCCCCU)
NFIX
ITGA2
KIF3A
CXCR3
TERF2IP
PDGFC
HSP90AB1
alendronic acid
miR-874-3p (miRNAs w/seed UGCCCUG)
miR-6868-5p (miRNAs w/seed CUGGCAG)
miR-1266-3p (miRNAs w/seed CCUGUUC)
miR-182-3p (miRNAs w/seed GGUUCUA)
NR2F1
bromocriptine
miR-1470 (miRNAs w/seed CCCUCCG)
trichloroethylene
ZEB2
benzene
miR-349 (and other miRNAs w/seed AGCCCUG)
miR-3940-3p (miRNAs w/seed AGCCCGG)
novobiocin
NRDE2
Agtr1b
atrazine
4-nonylphenol
Bcl9-Cbp/p300-Ctnnb1-Lef/Tcf
BMS-387032
PHF8
AFF4
CTDSP1
EGLN2
LRRC32
CTNND1
DKK2
salinomycin
PTGES3
RPL11
USP37
PINK1
GPR183
SCUBE3
TKT
1,1-bis(3'-indolyl)-1-(4-hydroxyphenyl)methane
benazepril
CITCO
iodine
tyrphostin AG 127
5-iodotubercidin
miR-1236-3p (miRNAs w/seed CUCUUCC)
miR-4432 (miRNAs w/seed AAGACUC)
RPTOR
miR-4790-5p (miRNAs w/seed UCGCUUU)
miR-551b-5p (miRNAs w/seed AAAUCAA)
miR-6786-5p (miRNAs w/seed CGGUGGG)
miR-1204 (miRNAs w/seed CGUGGCC)
MGP
ALDH2
CRHR2
CNR2
FOXF2
2,4-dinitrofluorobenzene
cafestol
miR-3195 (miRNAs w/seed GCGCCGG)
miR-670-5p (miRNAs w/seed UCCCUGA)
miR-10400-3p (and other miRNAs w/seed UGGGCUC)
miR-6735-3p (miRNAs w/seed GGCCUGU)
miR-23a-5p (and other miRNAs w/seed GGGUUCC)
9-hydroxy-(S)-10,12-octadecadienoic acid
N(6)-methyl-2'-deoxyadenosine
acyclovir
pelargonidin
azide
thromboxane A2
2'-adenylic acid
flavokawain C
ABLIM2
cyanuric acid
peptide YY 3-36
hydroxamic acid
LTB4R/LTB4R2
Gαq
NDPK
G protein alpha
beta-glucuronidase
Mlcp
glutathione peroxidase
MKK3/6
growth factor receptor
G-protein gamma
G-protein beta
Ahr-aryl hydrocarbon
2,2',4,4',5,5'-hexabromodiphenyl ether
5,7-dimethoxyflavone
DE 71
3',5'-methoxyflavone
Ahr-aryl hydrocarbon-RelA
NR2F1-AS1
PKR dimer
MAP2K4/7
NF279
sodium thiosulfate
GSK 189254
CLTA
(-)-OSU 6162
GJC2
PMM2
NCK
SEC16B
Pad2
pridopidine
FBXO31
EMC2
NLRP2
WTIP
LOC299282
RGN
FBXO22
SRF-ELK1
WFDC1
miR-92a-1-5p (miRNAs w/seed GGUUGGG)
mir-1
FOSL2
phosphatidylethanolamine
hyodeoxycholic acid
PDK
CK1
MLN8054
prochloraz
C1QTNF1
SPON2
SPATA2
NAA30
satratoxin G
DHX15
GNLY
CRT-0066101
RAB10
IL36RN
KPNB1
SNAPIN
CDK5R2
8-butyl-10-[(3-methoxy-5-pyrrol-2-ylidenepyrrol-2-ylidene)methyl]-11-azabicyclo[7.2.1]dodeca-1(12),9-diene
SRSF5
EXOC7
PTPN12
HERC5
iso[4]levuglandin E2
14-oxoDHA
SKF-83959
PX-12
trimethyloxamine
gabexate
LMK-235
oenothein B
AX15839
idronoxil
ethyl gallate
magnesium chloride
methoxyluteolin
titanium
pegylated leptin antagonist
miR-7702 (miRNAs w/seed UUAGACU)
EBF1
miR-4320 (miRNAs w/seed GGAUUCU)
miR-6767-5p (miRNAs w/seed CGCAGAC)
miR-4484 (miRNAs w/seed AAAGGCG)
miR-766-5p (and other miRNAs w/seed GGAGGAA)
miR-3145-5p (miRNAs w/seed ACUCCAA)
miR-4714-5p (and other miRNAs w/seed ACUCUGA)
miR-4783-3p (miRNAs w/seed CCCGGUG)
miR-3923 (miRNAs w/seed ACUAGUA)
miR-122b-5p (miRNAs w/seed UUAGUGU)
miR-881-3p (and other miRNAs w/seed ACUGUGU)
miR-367-5p (miRNAs w/seed CUGUUGC)
miR-5011-5p (miRNAs w/seed AUAUAUA)
miR-6757-3p (miRNAs w/seed ACACUGG)
miR-4259 (miRNAs w/seed AGUUGGG)
miR-335-3p (miRNAs w/seed UUUUCAU)
miR-4684-5p (miRNAs w/seed UCUCUAC)
miR-4274 (miRNAs w/seed AGCAGUC)
miR-1298-3p (miRNAs w/seed AUCUGGG)
mannitol
DLK1
CXCL3
GAB1
neuroprotectin D1
miR-3688-5p (miRNAs w/seed GUGGCAA)
psychosine
3'-O-(4-benzoyl)benzoyladenosine 5'-triphosphate
roxithromycin
(R)-limonene
N-hexanoylhomoserine lactone
LRRC26
CTSC
TLR10
RGMB
ZNF395
resolvin D5
PPM1L
AATF
GMFG
PIP5K1B
glaucocalyxin A
RAP1A
CBLC
SERPIND1
KPNA2
gelam honey
bepafant
10-(6'-ubiquinonyl)decyltriphenylphosphonium
GABA receptor
dieldrin
JWH-015
apamin
ZFA-fmk
RAM2061
N-retinylidene-N-retinylethanolamine
miR-888-3p (miRNAs w/seed ACUGACA)
methamphetamine
miR-4531 (miRNAs w/seed UGGAGAA)
miR-4470 (miRNAs w/seed GGCAAAC)
miR-6500-3p (miRNAs w/seed CACUUGU)
SGPL1
Pde4
pitavastatin
miR-24-1-5p (and other miRNAs w/seed GCCUACU)
miR-135a-1-3p (and other miRNAs w/seed AUAGGGA)
GPX1
miR-6748-3p (miRNAs w/seed CCUGUCC)
MSC
miR-3909 (and other miRNAs w/seed GUCCUCU)
miR-3198 (and other miRNAs w/seed UGGAGUC)
miR-1302 (and other miRNAs w/seed UGGGACA)
RAB1B
ABCB4
SKP2
miR-4724-5p (miRNAs w/seed ACUGAAC)
MEG3
mocetinostat
belnacasan
MFN2
N(2)-(gamma-D-glutamyl)-meso-2,2'-diaminopimelic acid
PSMB10
CSF3R
BMX
ADAM15
ID4
clasto-lactacystin beta-lactone
anti-benzo(a)pyrene-diol-epoxide
D-tubocurarine
loxoribine
miR-369-5p (miRNAs w/seed GAUCGAC)
miR-191-5p (and other miRNAs w/seed AACGGAA)
miR-1262 (and other miRNAs w/seed UGGGUGA)
miR-943 (miRNAs w/seed UGACUGU)
miR-6716-3p (miRNAs w/seed CCGAACU)
RBL1
PLG
aflatoxin B1
Stat1-Stat2
PARP
ANG
DCLK1
NCF4
PDE4B
COP1
HDAC8
CYBA
pubchem compound 11368987
mesalamine
miR-545-5p (miRNAs w/seed CAGUAAA)
CHADL
epoxomicin
corticosterone
TLR8
PRKACA
D-fructose
GW501516
miR-520d-5p (and other miRNAs w/seed UACAAAG)
miR-500a-5p (miRNAs w/seed AAUCCUU)
miR-449b-3p (and other miRNAs w/seed AGCCACA)
TCOF1
E2F4
miR-7108-5p (miRNAs w/seed UGUGGCC)
HNRNPU
CLEC4E
miR-4433b-5p (miRNAs w/seed UGUCCCA)
HNF1B
GSK3B
GW3965
cetuximab
TIFA
48s
prazosin
miR-218-1-3p (miRNAs w/seed UGGUUCC)
RORA
EPO
HNF4A
Mt2
bisindolylmaleimide iv
niacinamide
cinnamaldehyde
methapyrilene
phenylephrine
ursolic acid
miR-558 (and other miRNAs w/seed GAGCUGC)
ARNT
diphenyleneiodonium
HDAC6
miR-185-3p (and other miRNAs w/seed GGGGCUG)
LDLR
miR-6861-3p (miRNAs w/seed GGACCUC)
potassium chloride
RHOA
MIF
NOD2
ADCYAP1
MED1
SOCS1
KITLG
IL11
mir-181
GDF2
phorbol esters
YY1
PROC
ADRA1B
BIRC3
P2RY2
PCYT1A
miR-291a-3p (and other miRNAs w/seed AAGUGCU)
EIF4EBP1
amiodarone
uric acid
VIP
valproic acid
MIR101
S1PR3
BCL6
BRCA1
Pka
SYVN1
miR-361-3p (miRNAs w/seed CCCCCAG)
PRNP
FOSL1
cyclophosphamide
POR
ITGA5
IL11RA
sulforafan
lipoxin A4
xanthohumol
CTF1
FOSB
ELF4
alvocidib
sphingosylphosphocholine
MEMO1
RTKN
I-BOP
NR5A2
ACOX1
GNA12
CCL5
NfkB1-RelA
TAF4B
HMGB2
KN-62
benzo(a)pyrene
carbon tetrachloride
PPARA
CpG oligonucleotide
acetaminophen
miR-19b-3p (and other miRNAs w/seed GUGCAAA)
miR-199a-5p (and other miRNAs w/seed CCAGUGU)
NLRX1
PRKD1
IL27
lovastatin
TGFBR2
GPS2
rotenone
HDAC5
MAP3K7
fenretinide
KLF11
NFkB (family)
Integrin
CHD1
PPP2R5C
DDX17
premarin
silicon dioxide
TCF7L2
TWIST2
NQO1
PAK2
SPP1
nitrofurantoin
miR-1343-5p (and other miRNAs w/seed GGGGAGC)
miR-6806-5p (miRNAs w/seed GUAGGCA)
NPSR1
HSPB6
atipamezole
TSC22D3
ADRA1A
BCL2L1
miR-4267 (miRNAs w/seed CCAGCUC)
Mt1
WR 1065
miR-625-5p (and other miRNAs w/seed GGGGGAA)
TWIST1
miR-5129-5p (and other miRNAs w/seed UGUGGGG)
miR-4667-3p (miRNAs w/seed CCCUCCU)
flutamide
RUNX1-RUNX1T1
sorafenib
fluvastatin
parthenolide
SRC (family)
nitroprusside
GFI1
miR-193a-3p (and other miRNAs w/seed ACUGGCC)
miR-214-3p (and other miRNAs w/seed CAGCAGG)
miR-1249-5p (and other miRNAs w/seed GGAGGGA)
MTOR
allopurinol
MTPN
STAT6
miR-6749-3p (miRNAs w/seed UCCUCCC)
TRADD
WNT1
PLC
RPS6KA3
indirubin
acadesine
pioglitazone
MEF2C
miR-92a-3p (and other miRNAs w/seed AUUGCAC)
ETV6-RUNX1
RBPJ
imiquimod
BCR (complex)
Rock
miR-5107-3p (and other miRNAs w/seed AACCUGU)
GRP
NPPA
stearic acid
INHBA
conjugated linoleic acid
PGF
PLAUR
propranolol

ABLIM3
KCNJ12
CALCOCO1
URGCP
MARCHF4
RND2
FCN1
procyanidin B2
TMEM184A
DEPDC1
Ctf2
NACC2
MYADM
TRIM17
SLC17A6
NBR2
ZNF580
CCDC22
USP50
NLRP9
CCDC62
RPS6KA
MPC1
CCDC47
ZG16B
KCTD10
harman
FGF14
EMC6
TRPM3
Gm13570/Gm4984
PLAC1
RNF181
MARCHF9
METAP2
DHODH
TCF4-CTNNβ
nickel sulfide
rimantadine
tandutinib
Tpl2 kinase inhibitor
alisertib
hispidulin
MEF2D-NFAT2-p300
mapatumumab
NNZ 2566
vasopressins
black cohosh extract
Integrin alpha 5 beta 1
mediator
Mapk kinase
Rho gdi
GLIPR1
NSMCE3
TOMM22
DLAT
binimetinib
cucurbitacin E
ARHGEF17
voxtalisib
pidilizumab
EEFSEC
IRGM
PRCC
MEDI-547
Tenascin
DYNLRB1
RBMS1
MED25
EDN2
AHRR
KIFAP3
NAPA
MED7
KRT1
PPY
CDC45
CHKA
GPHA2
PRL-3 inhibitor I
SLC52A1
KSR2
ADCY2
CLTB
CFI
mir-363
miR-690 (miRNAs w/seed AAGGCUA)
Mir690
mir-1301
DYNLT3
NPVF
SLC30A9
CNOT9
CAMK2
SIGLEC7
SIGLEC9
TOB2
TNPO2
TYMP
SLC1A2
GTPBP4
GPHB5
SCN7A
NXF3
NAPB
LRRN1
aloperine
ANKS1A
5-methoxytryptophan
venetoclax
TM7SF2
KIF11
LUC7L3
PTPRU
WBP1
TFF1
MED21
PTCRA
Pla2g2a
PMS2
Sin3b
EPGN
WDR83OS
TAX1BP3
RIT1
CORO2A
CFD
ABLIM1
THEM4
BARX1
Tnxa-ps1
PRDM2
16:0 ceramide-1-phosphate
cytarabine/idarubicin
RAB5B
CDK20
ADAM19
GDF10
CD164
cytarabine/filgrastim/fludarabine phosphate
ETS1-AS1
PRC1
PTOV1
tetramethylpyrazine
INHBE
EXOSC10
PPP2R3A
TPD52
ZBTB14
RPS20
CHRM2
Flt1
MPT0E028
PEX14
molibresib
spliceostatin A
oligomycin A
CID-2011756
CML advanced glycation end products
SR11256
dihydrotanshinone I
NMS-873
RPL21
SLC25A5
RPLP0
3,4-(methylenedioxy)cinnamic acid
kurarinone

chlorogenic acid
miR-548ae-3p (and other miRNAs w/seed AAAAACU)
UCN
CYP7A1
miR-1178-5p (miRNAs w/seed AGGGUCA)
miR-3144-3p (miRNAs w/seed UAUACCU)
miR-6813-3p (miRNAs w/seed ACCUUGG)
CUX1
miR-4423-5p (miRNAs w/seed GUUGCCU)
miR-4473 (miRNAs w/seed UAGUGCU)
miR-4479 (miRNAs w/seed GCGCGGC)
miR-4777-5p (miRNAs w/seed UCUAGAU)
ING1
miR-3939 (miRNAs w/seed ACGCGCA)
miR-4660 (miRNAs w/seed GCAGCUC)
PTP4A1
ZAP70
pravastatin
miR-548p (miRNAs w/seed AGCAAAA)
miR-6762-3p (miRNAs w/seed GGCUGCU)
miR-8063 (miRNAs w/seed CAAAAUC)
semaxinib
miR-190a-3p (miRNAs w/seed UAUAUAU)
LGALS1
KDR
GSK2816126
miR-203b-3p (miRNAs w/seed UGAACUG)
miR-3185 (miRNAs w/seed GAAGAAG)
miR-8066 (miRNAs w/seed AAUGUGA)
miR-766-3p (miRNAs w/seed CUCCAGC)
miR-2682-3p (and other miRNAs w/seed GCCUCUU)
miR-4325 (miRNAs w/seed UGCACUU)
miR-5681b (miRNAs w/seed GGUAUUG)
miR-4515 (miRNAs w/seed GGACUGG)
miR-501-3p (and other miRNAs w/seed AUGCACC)
miR-4527 (and other miRNAs w/seed GGUCUGC)
miR-4305 (miRNAs w/seed CUAGACA)
miR-548av-3p (miRNAs w/seed AAACUGC)
FOXG1
SPTLC2
CRKL
SPHK2
TNFAIP2
GABBR1
RGS1
U18666A
cobalt
miR-4716-5p (miRNAs w/seed CCAUGUU)
miR-1277-5p (miRNAs w/seed AAUAUAU)
miR-6786-3p (miRNAs w/seed GACGCCC)
miR-4652-5p (miRNAs w/seed GGGGACU)
miR-652-5p (miRNAs w/seed AACCCUA)
miR-3121-5p (miRNAs w/seed CCUUUGC)
deoxycorticosterone acetate
NODAL
BTG2
miR-606 (miRNAs w/seed AACUACU)
HSPG2
Klrk1
miR-6857-5p (miRNAs w/seed UGGGGAU)
miR-3162-3p (miRNAs w/seed CCCUACC)
CD3 group
miR-4633-5p (miRNAs w/seed UAUGCCU)
miR-924 (miRNAs w/seed GAGUCUU)
ERVW-1
CDK1
MPZ
TRAIP
DHX9
RBP4
NAB2
zinc sulfate
elovanoid N32
miR-22-5p (and other miRNAs w/seed GUUCUUC)
mir-19
OLR1
miR-4695-3p (miRNAs w/seed GAUCUCA)
miR-3124-3p (and other miRNAs w/seed CUUUCCU)
miR-383-5p (miRNAs w/seed AGAUCAG)
GHRL
miR-6738-3p (miRNAs w/seed UUCUGCC)
miR-1185-5p (and other miRNAs w/seed GAGGAUA)
miR-6793-3p (miRNAs w/seed CCCCAAC)
pervanadate
flavokawain B
triamcinolone hexacetonide
coconut oil
Tlr11
USP25
TRIM29
tofacitinib
nadifloxacin
PDE6B
SERPINA4
SDC4
P2RY1
CCR4
USP21
HLA-B
GNMT
PILRA
BP-1-102
dimercaptopropanol
ISRIB
diacerein
miR-7153-3p (miRNAs w/seed ACCAUGG)
miR-381-3p (and other miRNAs w/seed AUACAAG)
ZFAS1
ABCA4
PPP1R15B
SARM1
G2535
APOH
Ifnz (includes others)
miR-617 (miRNAs w/seed GACUUCC)
MAPK11
STS
miR-17-2-3p (and other miRNAs w/seed CUGCACU)
Ccl2
miR-3154 (miRNAs w/seed AGAAGGG)
MITF
NPM1
RNASE2
IL27RA
LTBR
miR-6764-3p (and other miRNAs w/seed CUCUGGU)
ABCB6
miR-1278 (miRNAs w/seed AGUACUG)
miR-4655-3p (miRNAs w/seed CCCUCGU)
miR-621 (miRNAs w/seed GCUAGCA)
SIRT2
Trp53cor1
MTA3
SUPT16H
miR-511-3p (miRNAs w/seed AUGUGUA)
miR-3689a-5p (and other miRNAs w/seed GUGAUAU)
miR-524-3p (and other miRNAs w/seed AAGGCGC)
miR-4491 (and other miRNAs w/seed AUGUGGA)
miR-1276 (miRNAs w/seed AAAGAGC)
miR-548g-3p (miRNAs w/seed AAACUGU)
miR-889-5p (miRNAs w/seed AUGGCUG)
miR-6072 (and other miRNAs w/seed CCUCAUC)
miR-218-5p (and other miRNAs w/seed UGUGCUU)
miR-4650-3p (miRNAs w/seed GGUAGAA)
fluoride
resatorvid
PMP22
TNFRSF25
TNFRSF12A
CH-223191
miR-4529-3p (miRNAs w/seed UUGGACU)
garcinol
SIGIRR
CpG ODN 2395
PARG
HOXA11
trametinib
TRIM21
acetaldehyde
retinol
elovanoid N34
butaprost
SIRT3
miR-3174 (miRNAs w/seed AGUGAGU)
miR-448-3p (and other miRNAs w/seed UGCAUAU)
miR-150-5p (and other miRNAs w/seed CUCCCAA)
miR-379-5p (and other miRNAs w/seed GGUAGAC)

KDM5B
Collagen type I (complex)
gemcitabine
RARB
nicotine
SCAP
miR-4283 (miRNAs w/seed GGGGCUC)
trastuzumab
MST1R
PS-1145
idarubicin
peroxynitrite
miR-6815-5p (and other miRNAs w/seed AGGUGGC)
N-cor
MKNK1
glucocorticoid
clopidogrel
miR-1207-5p (and other miRNAs w/seed GGCAGGG)
miR-4690-3p (and other miRNAs w/seed CAGCCCA)
NR4A1
HOXC6
FASLG
testosterone
IL6R
homocysteine
IFNAR1
tosedostat
LLGL2
RXRA
CBL
di(2-ethylhexyl) phthalate
PIAS2
CHRM3
VLDLR
entinostat
miR-6873-3p (miRNAs w/seed UCUCUCU)
INHA
miR-519a-3p (and other miRNAs w/seed AAGUGCA)
miR-4757-5p (and other miRNAs w/seed GGCCUCU)
mir-486
TP53COR1
lapatinib
salvin
7-ketocholesterol
GNRH1
SBDS
arachidonic acid
vinblastine
miR-6084 (miRNAs w/seed UCCGCCA)
PSMB11
PARP1
miR-34a-5p (and other miRNAs w/seed GGCAGUG)
ADRA1
RSF1
RAB7A
aclarubicin
ANGPT1
dopamine
Sos
miR-6892-3p (miRNAs w/seed CCCUCUC)
miR-202-3p (and other miRNAs w/seed GAGGUAU)
miR-605-3p (miRNAs w/seed GAAGGCA)
E. coli lipopolysaccharide
RNF31
miR-6748-5p (and other miRNAs w/seed GUGGGUG)
Fc gamma receptor
PRKCZ
isoproterenol
CAV1
Ras homolog
SIN3B
W7
miR-130a-3p (and other miRNAs w/seed AGUGCAA)
PRKCB
KLF5
miR-4786-5p (miRNAs w/seed GAGACCA)
SIRT1
DLL4
A2M
PCGF2
advanced glycation end-products
fenofibrate
Mapk
RUNX2
celecoxib
CCNT1
D-sphingosine
miR-664-1-5p (and other miRNAs w/seed UGGCUGG)
NTRK2
miR-6752-3p (miRNAs w/seed CCCUGCC)
AGN194204
adenosine
PTGES
thioctic acid
miR-151-5p (and other miRNAs w/seed CGAGGAG)
ciprofibrate
TRAF3
Fgf
TET1
HLX
ALOX15
TAB1
honokiol
temozolomide
Y 27632
tanespimycin
FOXO4
plicamycin
miR-6134 (miRNAs w/seed GAGGUGG)
miR-3157-5p (miRNAs w/seed UCAGCCA)
cis-urocanic acid
PTAFR
ACVR1C
SATB1
miR-4691-5p (and other miRNAs w/seed UCCUCCA)
NANOG
miR-6852-5p (and other miRNAs w/seed CCUGGGG)
Notch
RET
NCOA2
dinoprost
miR-6512-3p (and other miRNAs w/seed UCCAGCC)
PD 0325901
PLAT
bryostatin 1
bardoxolone
miR-6881-5p (miRNAs w/seed GGGGUAA)
carbon monoxide
miR-6737-5p (and other miRNAs w/seed UGGGGUG)
miR-6515-5p (miRNAs w/seed UGGAGGG)
trypsin
harmine
SDC1
CRY1
PPP5C
PRKAA1
halofuginone
IL1RN
FGF8
NLRP3
miR-1275 (and other miRNAs w/seed UGGGGGA)
GLI1
ADRA1D
quinolinic acid
miR-122b-3p (and other miRNAs w/seed AACACCA)
miR-4763-5p (miRNAs w/seed GCCUGCC)
miR-5627-5p (and other miRNAs w/seed GAGGGUG)
trans-cinnamaldehyde
BRD7
N-methyl-D-aspartate
miR-132-5p (miRNAs w/seed CCGUGGC)
IL17F
FUS-DDIT3
CD3
miR-2117 (miRNAs w/seed GUUCUCU)
vanillyl-N-nonylamide
PTPRR
MIA-459
MIA-313
enzastaurin
6,7-dinitroquinoxaline-2,3-dione
6-cyano-7-nitroquinoxaline-2,3-dione
dibenzoylmethane
quisqualic acid
miR-7976 (miRNAs w/seed GCCCUGA)
IL24
miR-1260a (and other miRNAs w/seed UCCCACC)

diclofenac/hyaluronic acid
RB4
RB3
droxinostat
arsenic trichloride
meayamycin B
amurensin G
BCL201
beractant
NBI 27914
SB202474
S-methylisothiopseudouronium
impromidine
guanfacine
amlexanox
2,5-dihydroxymethylcinnamate
LY311727
BN 50730
sterigmatocystin
ondansetron
nafoxidine
cyclothiazide
SU6657
flurothyl
pustulan
trichloroacetic acid
L 733060
1-(1-glycero)dodeca-1,3,5,7,9-pentaene
methaneselenol
SC-19220
sulfaphenazole
L-threo-safingol
selfotel
melarsoprol
trichlorfon
(R)-5-(2-azetidinylmethoxy)-2-chloropyridine
Ro 20-1724
PD173955
BTG3-AS1
GS-626510
pubchem compound 353999
aspergillus beta-glucan
pneumocystis beta-glucan
flunitrazepam
thiabendazole
arachidonyl-2-chloroethylamide
naphthalene-1-2-dione
trans-7,8-dihydroxy-7,8-dihydrobenzo(a)pyrene
anthracene
tolrestat
ACS-1
2-amino-3-methylimidazo(4,5-f)quinoline
Ro 31-7549
bisindolylmaleimide III
ONO-8711
theaflavin monogallate B
anti-miR-302d
AP-1 decoy
anti-miR-302c inhibitor
beta-penta-O-galloyl-glucose
potassium iodide
bis-T-23
ETV6-PDGFRB
Fgfr2-Pgap2
BRD4-NUTM1
ZBTB16-RARA
CRTC1-MAML2
AuNP@PEG@e14a2
miR-124 inhibitor
anti-miR-32 inhibitor
PT2399
SU11333
CTOP
PF-4800567
N-t-Boc-Phe-D-Leu-Phe-D-Leu-Phe
IP 20
Gly-Gln
Ac-DEVD-CHO
ibutamoren
SMAD3-EP300
3-methoxyapigenin
berberrubine
columbamine
harmaline
LY 354740
droxidopa
S(-)-carbidopa
2-(N-myristoylamino)-1-phenyl-1-propanol
10-hydroxydecanoic acid
n-6 docosapentaenoic acid
long-chain alcohol
3'-adenylic acid
poractant alfa
2'-fucosyllactose
sarcosine
allose
lactulose
gestodene
estramustine
6alpha-fluorotestosterone
V2+
estradiol benzoate
miR-489-5p (miRNAs w/seed GUCGUAU)
tributyltin
M344
memantine
miR-4701-5p (and other miRNAs w/seed UGGCCAC)
miR-583 (miRNAs w/seed AAAGAGG)
miR-4641 (miRNAs w/seed GCCCAUG)
miR-6875-3p (miRNAs w/seed UUCUUCC)
miR-5702 (miRNAs w/seed GAGUCAG)
miR-6802-5p (miRNAs w/seed UAGGUGG)
MBD3
bosentan
Relaxin
bilirubin
sunitinib
FOXC2
miR-3141 (miRNAs w/seed AGGGCGG)
MECP2
miR-1900 (and other miRNAs w/seed GCCGCCC)
PCGEM1
NKX2-5
cyclopamine
L-threonine
niclosamide
anacardic acid
BCO2
Sox2ot
cercosporin
IGF2R
TNFRSF17
HOXB5
SETD7
IGBP1
HES5
KLB
CXCL13
STX3
EXT1
IQGAP1
HOXA1
INCB054329
Trolox C
2-hydroxy-1-naphthylaldehyde isonicotinoyl hydrazone
largazole
SS18-SSX1
miR-4481 (and other miRNAs w/seed GAGUGGG)
miR-214-5p (miRNAs w/seed GCCUGUC)
miR-431-3p (miRNAs w/seed AGGUCGU)
miR-5580-5p (and other miRNAs w/seed GCUGGCU)
miR-3571 (and other miRNAs w/seed ACACACU)
miR-365-1-5p (and other miRNAs w/seed GGGACUU)
mir-9
SOX6
HSD17B4
SDCBP
miR-6819-3p (and other miRNAs w/seed AGCCUCU)
H2AX
miR-636 (miRNAs w/seed GUGCUUG)
miR-4720-5p (and other miRNAs w/seed CUGGCAU)
miR-8060 (miRNAs w/seed CAUGAAG)
miR-3934-3p (miRNAs w/seed GCUCAGG)
NR4A3
BCL11B
COMMD3-BMI1
miR-561-5p (miRNAs w/seed UCAAGGA)
miR-891b (miRNAs w/seed GCAACUU)
mir-193
NOSTRIN
BECN1
3-D-glucosyl-1,2-diacylglycerol
behenic acid
chitinase
tylophorine
CACTIN
PIEZO1
ROBO3
ATF6B
TNIP3
FDX2
ATP6V0D2
ABRAXAS2
B3GNT2
ZFP64
CYP2C8
CIRBP
RNF122
LRRC19
CDCA2
P-TEFb
proanthocyanidin derivative
NCF2
cerotic acid
PF-4691502
GRPR
mir-590
CALCR
ORM1
MEP1A
BAG2
IL17B
A4GALT
APOD
TMSB10/TMSB4X
NLRC3
Rps6ka5
FDXR
WARS1
ARHGEF2
SKLB023
ERO1A
FLLL-32
NEK2
PPP1CC
HS6ST2
CAY10397
N-hydroxy-2,2-diphenylacetamide
(+)-epicatechin
1-(2-hydroxy-5-methylphenyl)-3-phenyl-1,3-propanedione
ITF3056
neopterin
tipifarnib
ibotenic acid
pegintron
dihydrosphingosine 1-phosphate
MCC950
tramadol
4-methylcatechol
chlorophyllin
1,1-diethyl-2-hydroxy-2-nitrosohydrazine
MR-409
AC083837.1
miR-499a-5p inhibitor
miR-208b inhibitor
oxamic acid
Vi capsular polysaccharide
miR-505-3p (miRNAs w/seed GUCAACA)
miR-4778-5p (miRNAs w/seed AUUCUGU)
KAT2B
miR-662 (miRNAs w/seed CCCACGU)
miR-744-3p (miRNAs w/seed UGUUGCC)
miR-5195-5p (miRNAs w/seed ACCCCUA)
miR-5590-5p (miRNAs w/seed UGCCAUA)
SUMO3
CD80
SKIL
NPY
PPP3R1
miR-8056 (miRNAs w/seed GUGGAUU)
miR-1261 (miRNAs w/seed UGGAUAA)
miR-3609 (and other miRNAs w/seed AAAGUGA)
MYOCD
miR-1244 (miRNAs w/seed AGUAGUU)
miR-590-3p (miRNAs w/seed AAUUUUA)
miR-4750-5p (miRNAs w/seed UCGGGCG)
glucagon
IL-1R
NFKBIZ
miR-548m (miRNAs w/seed AAAGGUA)
miR-548ao-3p (miRNAs w/seed AAGACCG)
miR-188-3p (miRNAs w/seed UCCCACA)
miR-20a-3p (miRNAs w/seed CUGCAUU)
miR-4661-5p (miRNAs w/seed ACUAGCU)
UCHL1
mir-182
BRD2
miR-493-5p (miRNAs w/seed UGUACAU)
beraprost
ALOX12
CST3
CLCA2
CACNA1A
1-((2-chlorophenyl)diphenylmethyl)-1H-pyrazole
4-octyl itaconate
putrescine
diaminopimelic acid
Mt
miR-3157-3p (miRNAs w/seed UGCCCUA)
miR-6755-5p (miRNAs w/seed AGGGUAG)
miR-1247-3p (and other miRNAs w/seed GGGAACG)
miR-4789-3p (miRNAs w/seed ACACAUA)
thymoquinone
CTBP1
miR-125b-2-3p (and other miRNAs w/seed CACAAGU)
miR-7974 (miRNAs w/seed GGCUGUG)
BCOR
LILRB1
FLT3LG
miR-4736 (and other miRNAs w/seed GGCAGGU)
miR-7855-5p (miRNAs w/seed UGGUGAG)
miR-629-3p (miRNAs w/seed UUCUCCC)
miR-890 (miRNAs w/seed ACUUGGA)
miR-7843-3p (miRNAs w/seed UGAAGCC)
3-deoxy-2-octulosonic acid(2)-lipid A
bevacizumab
Fcgr3
Sod
ZC3H14
TNFRSF6B
ABCC8
NBEAL2
C5AR2
3-aminobenzamide
7-nitroindazole
miR-3170 (and other miRNAs w/seed UGGGGUU)
miR-6757-5p (miRNAs w/seed AGGGAUG)
miR-4495 (miRNAs w/seed AUGUAAA)
miR-3915 (miRNAs w/seed UGAGGAA)
miR-30a-3p (and other miRNAs w/seed UUUCAGU)
heparan sulfate
N-Cadherin
CERS6
HDAC11
PON2
ADGRF5
NELFCD
immune complex
APBA3
CD33
MTBP
ponesimod
FALEC
filgotinib
GALP
farnesyl transferase
TNRC6A
Duxbl1 (includes others)
benzoic acid
rabeprazole
magnesium sulfate
ETV6-NTRK3
PCM1-JAK2
polaprezinc
PIN1
EWSR1-FLI1
F2RL1
gentamicin C
Sn50 peptide
metribolone
cholesterol
miR-1911-3p (miRNAs w/seed ACCAGGC)
miR-6726-3p (miRNAs w/seed UCGCCCU)
PSEN1
LGALS3
8-bromoguanosine 3',5'-cyclic monophosphate
mir-25
BTK
miR-1291 (and other miRNAs w/seed GGCCCUG)
NFAT (complex)
Ciap
JUN/JUNB/JUND
MAP3K3
Ni2+
miR-3202 (miRNAs w/seed GGAAGGG)
miR-6774-3p (miRNAs w/seed CGUGUCC)
THRA
APC
HDAC4
CLOCK
Go 6976
fish oils
BSG
RETN
xanthine
Rac
IDH2
SRSF3
MGAT3
ST14
ebselen
salmeterol
pentobarbital
nickel chloride
buthionine sulfoximine
Cd2+
miR-4455 (miRNAs w/seed GGGUGUG)
TO-901317
miR-6918-5p (and other miRNAs w/seed GCUGAGG)
TLR7
ZBTB17
triamterene
CBX5
PIAS1
IL22
TNFAIP3
miR-8088 (miRNAs w/seed CUCGGUA)
miR-1285-3p (and other miRNAs w/seed CUGGGCA)
zinc
miR-3064-3p (miRNAs w/seed UGCCACA)
miR-6826-3p (miRNAs w/seed UCCCCUC)
calcipotriene
NfkB-RelA
BCL2L11
LRPAP1
kaempferol
UCN-01
miR-7851-3p (miRNAs w/seed ACCUGGG)
miR-6856-3p (miRNAs w/seed ACAGCCC)
CIP2A
miR-3175 (miRNAs w/seed GGGGAGA)
CCN5
CP-55940
STAT
FGFR3
mevalonic acid
tempol
ethylene glycol tetraacetic acid
medroxyprogesterone acetate
miR-3173-5p (and other miRNAs w/seed GCCCUGC)
miR-6729-3p (miRNAs w/seed CAUCCCC)
Tcf7
TNFSF13B
3,3'-diindolylmethane
N-carbobenzyloxy-leucine-leucine-norvalinal
molybdenum disulfide
miR-145-5p (and other miRNAs w/seed UCCAGUU)
nicotinic acetylcholine receptor
SLC5A8
TCIM
IER3
IFNGR
casticin
MS4A1
perifosine
gliotoxin
MST1
XIAP
BAG1
methyl-beta-cyclodextrin
IRF8
CD38
miR-96-5p (and other miRNAs w/seed UUGGCAC)
MSTN
MYRF
fenamic acid
miR-4433a-5p (miRNAs w/seed GUCCCAC)
NSUN6
TFAP4
NR1D1
FOXP3
LYN
miR-5008-3p (and other miRNAs w/seed CUGUGCU)
miR-541-3p (and other miRNAs w/seed GGUGGGC)
TNFRSF1B
prednisolone
MSK1/2
FBXO25
GATAD2B
RAMP3
BAI
lavendustin A
tetrahydropalmatine
digitoxin
1-phenazinecarboxylic acid
GATA4
miR-3109-3p (and other miRNAs w/seed AGGGCCA)
miR-4646-3p (miRNAs w/seed UUGUCCC)
L-methionine
AKT2
NKX3-1
HTT
miR-3160-3p (miRNAs w/seed GAGCUGA)
miR-518c-5p (miRNAs w/seed CUCUGGA)
miR-11181-5p (and other miRNAs w/seed UCUGACC)
VCAN
IL7R
miR-4717-5p (and other miRNAs w/seed AGGCCAC)
miR-495-3p (and other miRNAs w/seed AACAAAC)
PELP1
BACH1
CSF2RB
MAPK10
PRDX2
MAML1
BCL10
RHOB
cyclopiazonic acid
hydrogen sulfide
mannose
IKBKE
miR-296-5p (miRNAs w/seed GGGCCCC)
ICAM1
IL2RG
miR-4640-3p (miRNAs w/seed ACCCCCU)
miR-4656 (and other miRNAs w/seed GGGCUGA)
miR-4633-3p (and other miRNAs w/seed GGAGCUA)
miR-148a-3p (and other miRNAs w/seed CAGUGCA)
dextran sulfate
GLI2
miR-6882-3p (miRNAs w/seed GCUGCCU)
miR-30c-5p (and other miRNAs w/seed GUAAACA)
mir-155
mir-29
tert-butyl-hydroquinone
miR-4278 (miRNAs w/seed UAGGGGG)
THBS1
isoprenaline
mir-34
ETS2
miR-18a-3p (and other miRNAs w/seed CUGCCCU)

KAT2A
diazoxide
tannic acid
LTB4R2
PBRM1
ACVR2A
CCL1
DUSP4
HCK
PTCSC3
RUVBL2
SART1
PIKFYVE
WNT10B
UBR5
COL17A1
TAS1R3
lidocaine
kukoamine A
pterostilbene
puerarin
Cu2+
PRMT1
miR-3060-5p (and other miRNAs w/seed GGAGUGC)
miR-6749-5p (miRNAs w/seed CGGGCCU)
miR-6716-5p (miRNAs w/seed GGGAAUG)
miR-290b-3p (and other miRNAs w/seed AGUGCCC)
miR-526b-5p (miRNAs w/seed UCUUGAG)
miR-1183 (miRNAs w/seed ACUGUAG)
ciprofloxacin
miR-4532 (miRNAs w/seed CCCGGGG)
miR-185-5p (and other miRNAs w/seed GGAGAGA)
Pka catalytic subunit
NOTCH4
CPE
ESRRB
miR-3944-5p (miRNAs w/seed GUGCAGC)
miR-668-5p (miRNAs w/seed GCGCCUC)
miR-769-5p (miRNAs w/seed GAGACCU)
KLF1
MED13
miR-4666b (miRNAs w/seed UGCAUGU)
miR-6778-3p (miRNAs w/seed GCCUCCC)
miR-518a-5p (and other miRNAs w/seed UGCAAAG)
miR-4728-3p (miRNAs w/seed AUGCUGA)
levothyroxine
miR-7846-3p (miRNAs w/seed AGCGGAG)
miR-7158-5p (miRNAs w/seed GCUCAAU)
miR-6863 (miRNAs w/seed AGACGUG)
miR-3077-5p (and other miRNAs w/seed UGCGGAC)
miR-4636 (miRNAs w/seed ACUCGUG)
SOX2-OT
chlorcyclizine
Il8r
WWC1
S100A12
AFAP1-AS1
PAK4
CTNNBIP1
NTS
JUP
SH3KBP1
ETV6
NUMBL
PAWR
PAX4
HSF4
CDH5
toremifene
bisphenol A diglycidyl ether
trientine
zinostatin
cucurbitacin B
NAD+
androstenediol
miR-3690 (miRNAs w/seed CCUGGAC)
miR-5587-3p (miRNAs w/seed CCCCGGG)
miR-4634 (miRNAs w/seed GGCGCGA)
choline fenofibrate
LATS1
REN
BMPR1B
miR-3190-3p (and other miRNAs w/seed GUGGAAG)
TRAP1
NR1I2
miR-548ad-3p (miRNAs w/seed AAAACGA)
miR-4449 (miRNAs w/seed GUCCCGG)
miR-3118 (and other miRNAs w/seed GUGACUG)
miR-5091 (miRNAs w/seed CGGAGAC)
miR-4774-3p (miRNAs w/seed UUGCCUA)
miR-1587 (and other miRNAs w/seed UGGGCUG)
PRMT5
APLN
AVP
KLF15
miR-7159-3p (miRNAs w/seed UUCUAUG)
miR-6804-3p (miRNAs w/seed GCACCUG)
miR-4285 (miRNAs w/seed CGGCGAG)
DNMT1
DLL1
HOXB13
ketoconazole

miR-4764-3p (miRNAs w/seed UAACUCC)
miR-532-5p (and other miRNAs w/seed AUGCCUU)
miR-4735-5p (miRNAs w/seed CUAAUUU)
miR-338-5p (miRNAs w/seed ACAAUAU)
miR-520g-3p (and other miRNAs w/seed CAAAGUG)
miR-6865-3p (and other miRNAs w/seed CACCCUC)
miR-548ag (and other miRNAs w/seed AAGGUAA)
FABP4
FBXW7
miR-29b-1-5p (miRNAs w/seed CUGGUUU)
miR-4520-2-3p (miRNAs w/seed UUGGACA)
mir-145
miR-4436b-5p (miRNAs w/seed UCCACUU)
miR-8064 (miRNAs w/seed GCACACU)
miR-330-3p (and other miRNAs w/seed CAAAGCA)
CHRNA7
CDK5
TYK2
Cdc42
miR-1273h-3p (miRNAs w/seed UGCAGAC)
miR-4499 (and other miRNAs w/seed AGACUGA)
miR-3659 (miRNAs w/seed GAGUGUU)
miR-202-5p (miRNAs w/seed UCCUAUG)
miR-597-3p (and other miRNAs w/seed GGUUCUC)
miR-1263 (miRNAs w/seed UGGUACC)
RABL6
miR-6505-5p (miRNAs w/seed UGGAAUA)
miR-3115 (miRNAs w/seed UAUGGGU)
miR-3617-5p (and other miRNAs w/seed AAGACAU)
miR-1305 (miRNAs w/seed UUUCAAC)
LMX1B
dipyridamole
tetrandrine
miR-4446-5p (miRNAs w/seed UUUCCCU)
miR-3551-5p (and other miRNAs w/seed CUUCACC)
miR-6882-5p (miRNAs w/seed ACAAGUC)
miR-6895-3p (miRNAs w/seed GUCUCUC)
miR-4796-5p (miRNAs w/seed GUCUAUA)
miR-4302 (miRNAs w/seed CAGUGUG)
FEV
miR-4797-3p (miRNAs w/seed CUCAGUA)
miR-4255 (miRNAs w/seed AGUGUUC)
azithromycin
neomycin
Mmp
CLEC10A
FOXD2-AS1
BPIFA1
LILRA2
iron dextran
IgG2a
mir-127
TIA1
PEMT
PLA2G2E
ATXN3
CAMKK2
iodoacetic acid
cytochalasin B
KT5720
KMT2A-AFF1
ethylenediaminetetraacetic acid
icatibant
poly-L-lysine
adefovir dipivoxil
cyclomaltodextrin
miR-4666a-5p (miRNAs w/seed UACAUGU)
GFI1B
batimastat
pyruvaldehyde
lisinopril
TGAL copolymer
irinotecan
acteoside
carbenoxolone
miR-208a-5p (and other miRNAs w/seed AGCUUUU)
TNIP1
ARNT2
miR-152-5p (miRNAs w/seed GGUUCUG)
TERC
choline
Brd4
miR-3678-5p (miRNAs w/seed CCGUACA)
miR-154-3p (and other miRNAs w/seed AUCAUAC)
miR-187-5p (miRNAs w/seed GCUACAA)
miR-301a-5p (and other miRNAs w/seed CUCUGAC)
miR-6802-3p (miRNAs w/seed UCACCCC)
miR-4452 (miRNAs w/seed UGAAUUC)
miR-592 (and other miRNAs w/seed UGUGUCA)
miR-4451 (miRNAs w/seed GGUAGAG)
thyroid hormone
miR-343 (and other miRNAs w/seed CUCCCUU)
miR-409-5p (and other miRNAs w/seed GGUUACC)
miR-6857-3p (miRNAs w/seed GACUGAG)
miR-671-3p (and other miRNAs w/seed CCGGUUC)
MEN1
miR-450a-5p (and other miRNAs w/seed UUUGCGA)
miR-181a-1-3p (and other miRNAs w/seed CCAUCGA)
miR-3925-3p (miRNAs w/seed CUCCAGU)
miR-466 (miRNAs w/seed UACACAU)
miR-483-3p (miRNAs w/seed CACUCCU)
shikonin
IPMK
UHRF2
paroxetine
miR-938 (miRNAs w/seed GCCCUUA)
miR-1269a (and other miRNAs w/seed UGGACUG)
miR-5089-3p (and other miRNAs w/seed UGCUACU)
miR-4263 (miRNAs w/seed UUCUAAG)
miR-3473g (and other miRNAs w/seed AAAAGUG)
miR-6862-5p (and other miRNAs w/seed GGGCAUG)
miR-3936 (miRNAs w/seed AAGGGGU)
miR-593-3p (miRNAs w/seed GUCUCUG)
SYK/ZAP
NCSTN
mercuric chloride
KDM6B
2',3'-dialdehyde ATP
triciribine
R 59022
L-lysine
carnobyl iron
cytidylyl-3'-5'-guanosine
IRF3 dimer
bacterial lipopolysaccharides
bafilomycin A
RASSF6
LRRK2
Ferritin
UNC5B
SERPINB2
THBD
TRG
ZIC2
mir-217
mir-378
RALBP1
ALOX15B
IL4I1
ERP29
TSG101
IL22RA1
IL12RB2
HA900
7,8-dihydro-7,8-dihydroxybenzo(a)pyrene 9,10-oxide
1,1-bis(3'-indolyl)-1-(4-trifluoromethyl-phenyl)methane
SR1078
pentazocine
miR-3973 (miRNAs w/seed CAAAGUA)
miR-219b-5p (and other miRNAs w/seed GAUGUCC)
miR-6505-3p (and other miRNAs w/seed GACUUCU)
delta-12-prostaglandin J2
beta-glucan
teniposide
[Lys15,Arg16,Leu27]VIP(1-7)GRF(8-27)
AOPEP
rintatolimod
ATPase
edratide
IFN alpha receptor
beta-sitosterol
CpG ODN M362
olmesartan
SMPD3
Gpcr
BCAS2
TRIM58

BCL2
fatty acid
miR-12201-5p (and other miRNAs w/seed GGAAGGA)
miR-4525 (and other miRNAs w/seed GGGGGAU)
morphine
4-phenylbutyric acid
miR-6835-5p (and other miRNAs w/seed GGGGGUA)
miR-4268 (miRNAs w/seed GCUCCUC)
CD44
miR-665 (and other miRNAs w/seed CCAGGAG)
miR-450b-3p (and other miRNAs w/seed UGGGAUC)
NR1I3
IL7
hexachlorobenzene
SERPINF1
IL32
Vhl
S100a7a
LPA
TNFRSF10B
buserelin
palmitoyl-Cys((RS)-2,3-di(palmitoyloxy)-propyl)-Ala-Gly-OH
tosyllysine chloromethyl ketone
IRS2
ATF1
chloropromazine
pCPT-cAMP
Hmgb1
p85 (pik3r)
CCAT2
nimesulide
SAMSN1
nocodazole
miR-4764-5p (miRNAs w/seed GGAUGUG)
miR-3127-3p (and other miRNAs w/seed CCCCUUC)
Histone h4
taurocholic acid
crizotinib
SH3TC2
S100A6
PHB
EPOR
CD14
Gcg
TLR2
PRKAA2
SPARC
KL
SRSF2
Hif1
miR-6866-5p (miRNAs w/seed UAGAGGC)
PRDM1
miR-1825 (miRNAs w/seed CCAGUGC)
miR-638 (miRNAs w/seed GGGAUCG)
SOCS3
miR-6874-5p (and other miRNAs w/seed UGGAGCU)
miR-4489 (miRNAs w/seed GGGGCUA)
Il3
minocycline
PADI2
GLIS2
Endothelin
PLA2G4A
S1PR2
pimozide
magnolol
IFN Beta
ZNF281
RPSA
PRKCI
urethane
dithiothreitol
R-WIN 55,212
2-methoxyestradiol
CITED2
CD28
miR-4649-3p (miRNAs w/seed CUGAGGC)
E. coli B4 lipopolysaccharide
miR-2355-5p (miRNAs w/seed UCCCCAG)
PPP2CA
IL1R1
phenylbutazone
cadmium chloride
MAP2K6
RELB
miR-486-3p (and other miRNAs w/seed GGGGCAG)
IL10
miR-6759-5p (miRNAs w/seed UGUGGGU)
Rxr
miR-138-5p (miRNAs w/seed GCUGGUG)
miR-6846-5p (and other miRNAs w/seed GGGGGCU)
miR-137-3p (miRNAs w/seed UAUUGCU)
RB1
RAC2
dimethylnitrosamine
Ck2
C8
GLRX
PSMB9
C7
DEK
asbestos
PD 168393
JMF3086
hexamethoxyflavone
TBK1
PTX3
NUP98-HOXA9
TGFB3
losartan potassium
heparin
GCG
1-methyl-4-phenyl-1,2,3,6-tetrahydropyridine
HIC1
mitomycin C
miR-4707-3p (miRNAs w/seed GCCCGCC)
miR-155-5p (miRNAs w/seed UAAUGCU)
Srebp
NOD1
SLC29A1
BAP1
MNT
PDCD4
metyrapone
lycopene
Esrra
WBP2
MMP9
TBX5
ciglitazone
miR-7854-3p (miRNAs w/seed GAGGUGA)
PRKAA
PRC2
IFNLR1
miR-345-5p (and other miRNAs w/seed CUGACCC)
miR-5190 (miRNAs w/seed CAGUGAC)
n-nitrosomethylbenzylamine
YBX1
Stat3-Stat3
ROR1
TIRAP
EIF2AK4
PRLR
SMAD5
linsidomine
hymecromone
ETS1
N-Ac-Leu-Leu-norleucinal
miR-133a-5p (miRNAs w/seed GCUGGUA)
IL6ST
maneb
LRP6
vincristine
levodopa
LEF1
N-nitro-L-arginine methyl ester
PPARGC1A
miR-4684-3p (miRNAs w/seed GUUGCAA)
miR-548aj-5p (and other miRNAs w/seed GCAAAAG)
USF1
ZEB1
STAT1
cyclooxygenase
dacinostat
SAV1
dactolisib
STAP2

PYHIN1
G6PC3
GATM
benzenesulfonic acid
IgG2b
TRAFD1
DCB 3503
STX11
AHSG
ERN2
NCK2
PPP1R13B
PROS1
FGG
PILRB
Orm1 (includes others)
EIF2AK1
PFN1
RPS3
CCL22
ADAM8
PRMT2
LILRB3
TNFAIP8
DGKA
ODN-A151
HG-9-91-01
CC-122
MI-773
pubchem compound 11483544
cimetidine
SD-282
PD 150606
ethacrynic acid
phenol derivative
orantinib
FT671
retinaldehyde
methyllycaconitine
acetyl-11-keto-beta-boswellic acid
Hg2+
miR-433-3p (miRNAs w/seed UCAUGAU)
miR-212-5p (miRNAs w/seed CCUUGGC)
miR-296-3p (miRNAs w/seed AGGGUUG)
miR-451b (miRNAs w/seed AGCAAGA)
miR-1306-3p (miRNAs w/seed CGUUGGC)
miR-6780a-3p (miRNAs w/seed UCCUCUG)
miR-4475 (miRNAs w/seed AAGGGAC)
miR-548b-3p (miRNAs w/seed AAGAACC)
miR-450b-3p (and other miRNAs w/seed UUGGGGA)
NRF1
miR-653-5p (miRNAs w/seed UGAAACA)
miR-543-3p (and other miRNAs w/seed AACAUUC)
rifampin
artesunic acid
HNF1A-AS1
TEK
TMBIM6
EPHA2
RARRES2
IL1RL1
triflusal
miR-1226-5p (and other miRNAs w/seed UGAGGGC)
miR-2116-5p (miRNAs w/seed GUUCUUA)
miR-6070 (miRNAs w/seed CGGUUCC)
miR-8062 (miRNAs w/seed AGUGAUU)
miR-4513 (and other miRNAs w/seed GACUGAC)
miR-5007-5p (miRNAs w/seed AGAGUCU)
sorbitol
ginsenoside Rb1
Ptprd
SCAVENGER receptor CLASS A
TOLLIP
TAC4
LPAR1
SAT1
deoxycorticosterone acetate/potassium chloride/sodium chloride
RIPK3
RBP3
FPR2
meclizine
beta-1,3-glucan
miR-6076 (and other miRNAs w/seed GCAUGAC)
S100A4
L-ornithine
diphosphoryl lipid A
1,4-glucan
arbutin
syringin
7beta-hydroxycholesterol
Gelonin
chartreusin
Plc beta
PLA2
Rab5
Max-Myc
glemanserin
diosmetin
DF 203
NPS
METTL21C
Rps15a-ps4
TCHP
EIF4B
ZFAT
TRIM50
MTNR1A
NMRAL1
POPDC2
ARL16
LYST
Hsf3
MARCHF2
linsitinib
GPR4
SMYD5
BATF2
MTNR1B
MLXIP
ARL11
SENP5
Plk
MAP3K21
AFAP1L2
ganglioside GM3 (d18:1/18:0)
ganglioside GM3 (d18:1/20:0)
ganglioside GM3 (d18:1/22:0)
Par
IKKA/B
diaziquone
RPL19
pimasertib
SH3GLB1
AZD4547
Raet1b
MAPK8IP2
IVNS1ABP
CELA1
TRIM5
cyproconazole
NMB
mir-630
TPPP3
lynestrenol
H60a
POLDIP3
IL1RAPL1
DCTN4
CCT5
AZU1
SLC7A11
RPS6KA2
DDX24
GABRG2
ZFP36L1
CRADD
ATP9A
IMPACT
PGAM1
GPR32
TMEM9B
CD8B
KHDRBS2
GAB3
3-hydroxydodecanoic acid
6-n-octylaminouracil
FGF5
KLF10
S1PR1
RND3
Usp17la (includes others)
AS1842856
sulfasalazine
S-(2,3-bispalmitoyloxypropyl)-cysteine-GDPKHPKSF
biochanin A
leptomycin B
1,4-bis[2-(3,5-dichloropyridyloxy)]benzene
IFNB1
miR-302c-3p (and other miRNAs w/seed AGUGCUU)
miR-6511b-5p (and other miRNAs w/seed UGCAGGC)
ITGAV
MARK2
PDPK1
vitamin A
Hsp27
INTS11
PUM1
C1QL1
ZNF300
KLHL21
pycnogenols
ELL
HSPB3
PUM2
TRAF7
GFRA2
INHBC
ERC1
IL20RA
CASP8AP2
di-2-pyridylketone 4-cyclohexyl-4-methyl-3-thiosemicarbazone
tarenflurbil
minodronate
nickel subsulfide
senexin B
ODN-BW006
oleandrin
IL10RB
DAB2IP
mir-503
HCRT
HOXB3
IL9R
N4BP1
Igkv1-117
NFKBIE
levamisole
cortistatin A
acetic acid
retinol acetate
gossypol
tridecanoic acid
ELAVL1
CEBPD
EGLN
EGR2
miR-221-3p (and other miRNAs w/seed GCUACAU)
ursodeoxycholic acid
NEUROG1
sulindac
miR-1294 (and other miRNAs w/seed GUGAGGU)
IL18
miR-139-3p (miRNAs w/seed GGAGACG)
miR-3144-5p (miRNAs w/seed GGGGACC)
IKZF1
TET2
NS-398
miR-589-3p (and other miRNAs w/seed CAGAACA)
IFIH1
2-bromoethylamine
daporinad
Aldose Reductase
MED12
SP100
GDF15
ADRB3
PER1
PPP1R15A
PD 169316
mangiferin
miR-1537-5p (miRNAs w/seed GCUGUAA)
infliximab
azoxymethane
CST5
miR-6795-3p (miRNAs w/seed CCCCUCG)
miR-6822-3p (miRNAs w/seed GGCUCUA)
SOD2
miR-1909-5p (miRNAs w/seed GAGUGCC)
SB-431542
PML
SREBF1
miR-221-5p (and other miRNAs w/seed CCUGGCA)
miR-3680-3p (miRNAs w/seed UUUGCAU)
SQSTM1
Hedgehog
AURK
ANLN
HDAC2
miR-3104-5p (and other miRNAs w/seed AGGGGGC)
miR-141-3p (and other miRNAs w/seed AACACUG)
bafilomycin A1
sodium orthovanadate
andrographolide
naringenin
SN-38
miR-6080 (miRNAs w/seed CUAGUGC)
miR-6801-3p (and other miRNAs w/seed CCCCUGC)
FOXA1
miR-7155-5p (miRNAs w/seed CUGGGGU)
miR-4504 (miRNAs w/seed GUGACAA)
8-bromo-cAMP
prostaglandin A1
MFSD2A
EIF4G2
lithium
echinomycin
YOD1
SNHG20
DRD1
SLC9A1
ENTPD1
CNB-001
Ncoa6
MAP3K11
sevoflurane
ferric ammonium citrate
theophylline
TIMP3
TEAD2
beta-naphthoflavone
ziritaxestat
miR-4688 (and other miRNAs w/seed AGGGGCA)
IGF1R
miR-517a-3p (and other miRNAs w/seed UCGUGCA)
miR-3191-3p (miRNAs w/seed GGGGACG)
miR-6515-3p (miRNAs w/seed CUCUUCA)
sirolimus
APOE
asoprisnil
miR-2110 (and other miRNAs w/seed UGGGGAA)
NR4A2
miR-5681a (and other miRNAs w/seed GAAAGGG)
FANCC
EFNA1
miR-4642 (miRNAs w/seed UGGCAUC)
panobinostat
IDH1
mir-183
SMPD1
miR-18a-5p (and other miRNAs w/seed AAGGUGC)
doxycycline
miR-200b-3p (and other miRNAs w/seed AAUACUG)
miR-663b (miRNAs w/seed GUGGCCC)
miR-516b-5p (miRNAs w/seed UCUGGAG)
HMOX1
zidovudine
Cbp/p300
NADPH oxidase
CTR9
mir-26
Sch-23390
pifithrin alpha
epicatechin gallate
diallyl trisulfide

CD300E
ralimetinib
DDT
CGGBP1
zotiraciclib
parsatuzumab
TMPRSS4
IL17D
HUS1
EYA4
ATB-346
polysaccharide TA-1
verticillin A
bimekizumab
minalrestat
2,2-(2-chlorophenyl-4'-chlorophenyl)-1,1-dichloroethene
buformin
neocuproine
telomestatin
primaquine
indapamide
E-4031
dofetilide
desethylamiodarone
BAY-u9773
bumetanide
oxazepam
dibenzo(a,l)pyrene
chalcone-24
benzo(a)pyrene-7,8-dione
terpenoid
wax ester
nisoldipine
LZ1 peptide
moringa oleifera aqueous seed extract
moringa oleifera diluted seed extract
dapansutrile
Z-YVAD-FMK
D-JNK inhibitor I
Z-WEHD-FMK
SR1555
SR2211
indolo(3,2-b)carbazole
alsterpaullone
kenpaullone
alpha-TEA
teleocidins
D-lysergic acid diethylamide
tyrphostin B56
ceftazidime
trehalose dimycolate
12(S)-HPETE
polyinosinic acid
L-canavanine
KB-R7785
17-epiestriol
miR-6510-3p (miRNAs w/seed ACCGACU)
miR-3163 (miRNAs w/seed AUAAAAU)
miR-3942-3p (and other miRNAs w/seed UUCAGAU)
miR-548aw (miRNAs w/seed UGCAAAA)
IL25
miR-509-5p (and other miRNAs w/seed ACUGCAG)
TNFRSF11B
UBE3A
EGLN3
let-7d-3p (miRNAs w/seed UAUACGA)
HDL-cholesterol
CD4
miR-5707 (miRNAs w/seed CGUUUGA)
miR-8075 (miRNAs w/seed GCUGAUG)
miR-3569 (and other miRNAs w/seed GAGGACA)
miR-3074-5p (miRNAs w/seed UUCCUGC)
CLU
WAS
miR-494-3p (miRNAs w/seed GAAACAU)
miR-1257 (miRNAs w/seed GUGAAUG)
cardiotoxin
miR-669d-5p (and other miRNAs w/seed CUUGUGU)
miR-676-3p (miRNAs w/seed UGUCCUA)
miR-1839-5p (and other miRNAs w/seed AGGUAGA)
miR-2115-5p (miRNAs w/seed GCUUCCA)
miR-4732-5p (miRNAs w/seed GUAGAGC)
miR-4457 (miRNAs w/seed CACAAGG)
CCR2
miR-10b-3p (miRNAs w/seed CAGAUUC)
miR-1255b-2-3p (miRNAs w/seed ACCACUU)
miR-151-3p (and other miRNAs w/seed UAGACUG)
miR-873-5p (and other miRNAs w/seed CAGGAAC)
phenethyl isothiocyanate
miR-3130-5p (and other miRNAs w/seed ACCCAGU)
miR-6075 (miRNAs w/seed CGGCCCA)
CYB5R4
ZNF202
MK2206
mir-192
lapatinib/pazopanib
PTK2B
fumonisin B1
arecoline
creatine
chitin
miR-4711-5p (miRNAs w/seed GCAUCAG)
rasagiline
MERTK
QKI
miR-639 (miRNAs w/seed UCGCUGC)
miR-4693-3p (miRNAs w/seed GAGAGUG)
HSPA5
miR-4269 (and other miRNAs w/seed CAGGCAC)
miR-142-5p (and other miRNAs w/seed AUAAAGU)
miR-6873-5p (miRNAs w/seed AGAGGGA)
miR-3614-5p (miRNAs w/seed CACUUGG)
miR-6771-5p (miRNAs w/seed UCGGGAG)
miR-1322 (miRNAs w/seed AUGAUGC)
miR-1295b-3p (miRNAs w/seed AUAGGCC)
ELF3
TRPM2
mir-221
UXT
TNFSF15
irbesartan
rosuvastatin
CXCL10
LTBP1
cyanocobalamin
miR-323-3p (and other miRNAs w/seed ACAUUAC)
miR-1277-3p (and other miRNAs w/seed ACGUAGA)
miR-7112-3p (miRNAs w/seed GCAUCAC)
miR-297a-5p (and other miRNAs w/seed UGUAUGU)
miR-3941 (and other miRNAs w/seed UACACAC)
miR-3084a-3p (and other miRNAs w/seed UCUGCCA)
PDCD1
O6-benzylguanine
SQ 22536
1L-6-hydroxymethyl-chiro-inositol 2-(R)-2-O-methyl-3-O-octadecylcarbonate
APC/APC2
HOXA11-AS
TYRO3
PDK2
AMBP
RAMP1
UGDH
PRKRA
NCR1
NPAT
ERRFI1
HOXB7
SLC6A1
TP53BP2
CXCL16
farglitazar
IC87114
(5-(4-N-methyl-N(2-pyridyl)amino)ethoxy)benzyl thiazolidine-2,4-dione
midazolam
2,4-dinitrobenzenesulfonic acid
perilla alcohol
THRAP3
PROM1
SELENOS
PCSK9
HIRA
PCK1
SMC3
PNN
CAPN3
CRTC1
Z-551

cannabidiol
miR-4675 (and other miRNAs w/seed GGGCUGU)
TRAF6
miR-767 (and other miRNAs w/seed GCACCAU)
miR-3102-3p (and other miRNAs w/seed AGCACCC)
TANK
CAMK4
miR-548au-3p (miRNAs w/seed GGCAGUU)
MTORC1
ADM
SOD1
miR-3473h-5p (and other miRNAs w/seed AGGGGCU)
LDB1
miR-6803-3p (miRNAs w/seed CCCUCGC)
NFATC3
ID1
lipoteichoic acid
miR-574-5p (miRNAs w/seed GAGUGUG)
SOX4
miR-6734-3p (miRNAs w/seed CCUUCCC)
L-histidine
STAT1/3/5 dimer
NELFB
mir-379
RB1CC1
C9
KRIT1
SR 144528
quinacrine
midostaurin
8-oxo-7-hydrodeoxyguanosine
LMO2
miR-610 (miRNAs w/seed GAGCUAA)
fluticasone propionate
norepinephrine
miR-4713-5p (miRNAs w/seed UCUCCCA)
miR-4251 (miRNAs w/seed CUGAGAA)
NFKBIB
Am 580
miR-4512 (miRNAs w/seed AGGGCCU)
nitrofen
GNRH
GDF9
TLR5
ADORA2B
miR-1237-5p (and other miRNAs w/seed GGGGGCG)
docosahexaenoic acid
dexamethasone phosphate
PDGF-AA
HOXA13
HEY1
THZ1
vanillin
PITX2
linoleic acid
TEAD1
miR-3191-5p (miRNAs w/seed UCUCUGG)
colfosceril palmitate
Rsk
CC8490
MIB2
USP28
Carlr
GCLC
lanatoside C
pentamidine
bupropion
pyocyanin
miR-4645-5p (and other miRNAs w/seed CCAGGCA)
pyridaben
colchicine
NKX2-3
RXRB
SOX1
mir-22
FCGR2A
diethylnitrosamine
miR-5682 (miRNAs w/seed UAGCACC)
let-7
TSC2
miR-6834-3p (miRNAs w/seed AUGUCCC)
miR-4649-5p (and other miRNAs w/seed GGGCGAG)
miR-199a-3p (and other miRNAs w/seed CAGUAGU)
miR-4779 (miRNAs w/seed AGGAGGG)
CDKN1A
17-alpha-ethinylestradiol
miR-3190-5p (miRNAs w/seed CUGGCCA)
dibutyl phthalate
Smad
COMMD1
LCK
ADAM17
miR-4446-3p (miRNAs w/seed AGGGCUG)
miR-4530 (miRNAs w/seed CCAGCAG)
miR-4753-5p (miRNAs w/seed AAGGCCA)
desoxycorticosterone
Pdgfr
Ppp2c
TRPV1
HIF3A
CSF2RA
MED16
HBP1
CRY2
BCAR1
RPS6KA4
MMP8
DUSP14
HSPA1A/HSPA1B
cantharidin
N,N-dimethylsphingosine
verteporfin
miR-4520-3p (miRNAs w/seed UGGACAG)
miR-3689d (and other miRNAs w/seed GGAGGUG)
NOX4
carbamylcholine
histone deacetylase
propofol
miR-376c-3p (and other miRNAs w/seed ACAUAGA)
miR-614 (miRNAs w/seed AACGCCU)
miR-3150a-3p (and other miRNAs w/seed UGGGGAG)
zymosan
isotretinoin
cadmium
miR-124-3p (and other miRNAs w/seed AAGGCAC)
CLEC4G
miR-6808-5p (and other miRNAs w/seed AGGCAGG)
miR-6720-3p (miRNAs w/seed GCGCCUG)
miR-4276 (and other miRNAs w/seed UCAGUGA)
SUZ12
L-glutamic acid
KIT
CHI3L1
DCAF1
OXT
RCAN1
NOS1
K-252
calyculin A
amino acids
linolenic acid
STK40
ZNF217
nifedipine
mir-204
miR-181a-5p (and other miRNAs w/seed ACAUUCA)
miR-328-3p (and other miRNAs w/seed UGGCCCU)
daidzein
miR-24-3p (and other miRNAs w/seed GGCUCAG)
diphtheria toxin
Tlr
miR-16-5p (and other miRNAs w/seed AGCAGCA)
HOXA10
PTK2
MAPKAPK2
emodin
NCOR1
glycochenodeoxycholate
CDKN2B-AS1
PLCE1
F10
PIAS4
PTPRE
LAMC1
MVP
N-(3-oxododecanoyl)-homoserine lactone
auranofin

| | |
|---|---|
| asiatic acid | EFNA4 |
| isoquercitrin | ibuprofen |
| deguelin | everolimus |
| CALC | pentoxifylline |
| miR-6826-5p (miRNAs w/seed CAAUAGG) | miR-4323 (miRNAs w/seed AGCCCCA) |
| l-asparaginase | CD300LF |
| miR-4536-5p (miRNAs w/seed GUGGUAG) | vemurafenib |
| miR-3090-3p (and other miRNAs w/seed CCCAGGU) | PROCR |
| desmopressin | mir-148 |
| miR-1324 (miRNAs w/seed CAGACAG) | DTX1 |
| miR-1225-5p (miRNAs w/seed UGGGUAC) | MAP2K2 |
| uranyl nitrate | CTSB |
| miR-4509 (miRNAs w/seed CUAAAGG) | VCP |
| miR-517-5p (miRNAs w/seed CUCUAGA) | ozone |
| miR-6816-3p (miRNAs w/seed AAGGACC) | isoflurane |
| miR-3082-3p (and other miRNAs w/seed ACAUGGC) | FGF7 |
| miR-3926 (miRNAs w/seed GGCCAAA) | ROR2 |
| miR-1273c (miRNAs w/seed GCGACAA) | Bay 11-7082 |
| miR-500a-3p (miRNAs w/seed UGCACCU) | TNFRSF1A |
| miR-4464 (and other miRNAs w/seed AGGUUUG) | SPI1 |
| miR-1537-3p (miRNAs w/seed AAACCGU) | NR1H4 |
| XBP1 | miR-593-5p (and other miRNAs w/seed GGCACCA) |
| miR-100-5p (and other miRNAs w/seed ACCCGUA) | miR-6888-5p (miRNAs w/seed AGGAGAU) |
| miR-197-5p (and other miRNAs w/seed GGGUAGA) | RNF2 |
| miR-4670-3p (miRNAs w/seed GAAGUUA) | capsaicin |
| miR-4427 (miRNAs w/seed CUGAAUA) | miR-3547 (and other miRNAs w/seed GAGCACC) |
| SGK1 | miR-153-3p (miRNAs w/seed UGCAUAG) |
| miR-2355-3p (miRNAs w/seed UUGUCCU) | miR-5588-3p (miRNAs w/seed AGUCCCA) |
| miR-3611 (miRNAs w/seed UGUGAAG) | miR-3148 (miRNAs w/seed GGAAAAA) |
| miR-5187-5p (miRNAs w/seed GGGAUGA) | miR-540-3p (and other miRNAs w/seed GGUCAGA) |
| miR-425-5p (and other miRNAs w/seed AUGACAC) | SELPLG |
| miR-4782-5p (and other miRNAs w/seed UCUGGAU) | ARHGAP21 |
| miR-488-5p (miRNAs w/seed CCAGAUA) | EFNA3 |
| Iga | HBB |
| COL2A1 | retinoid |
| IDO1 | mir-30 (includes others) |
| TNFRSF13B | ouabain |
| PPIF | IL12 (complex) |
| BMS-690514 | ERG |
| ARHGAP31 | EIF4E |
| imipramine blue | miR-4318 (miRNAs w/seed ACUGUGG) |
| miR-1245b-5p (and other miRNAs w/seed AGGCCUU) | SFTPA1 |
| KAT5 | SREBF2 |
| miR-1301-5p (and other miRNAs w/seed GCUCUAG) | (+)-MK-801 |
| miR-3073a-5p (and other miRNAs w/seed UGGUCAC) | SP110 |
| sodium bisulfide | miR-4721 (and other miRNAs w/seed GAGGGCU) |
| MASTL | NKX2-1 |
| miR-5004-3p (miRNAs w/seed UUGGAUU) | miR-128-3p (and other miRNAs w/seed CACAGUG) |
| mir-142 | imatinib |
| miR-487b-3p (miRNAs w/seed AUCGUAC) | miR-718 (miRNAs w/seed UUCCGCC) |
| 1,2-dioleoyloxy-3-(trimethylammonium)propane | miR-1268a (and other miRNAs w/seed GGGCGUG) |
| ganciclovir | L-alpha-lysophosphatidylcholine, palmitoyl |
| GPIIB-IIIA | dienogest |
| teichoic acid | styrene |
| Fcgr2 | BPI |
| mGLUR Group I | sGC |
| Dynamin | Mucin |
| glycitein | IL31RA |
| BICC1 | LOC102724788/PRODH |
| RAB7B | APAF1 |
| KCNK2 | KIDINS220 |
| SNHG15 | SENP6 |
| KLF17 | PELI2 |
| CLEC6A | Muc1 |
| CLEC4D | GCLM |
| CCN6 | 2-cyclohexen-1-one |
| TRPM7 | mevalonolactone |
| RDH8 | butylated hydroxytoluene |
| dynasore | miR-3103-5p (and other miRNAs w/seed GAGGGAG) |
| RABGEF1 | TNFRSF8 |
| SMPD2 | TYROBP |
| FGF13 | N-acetylmuramyl-L-alanyl-D-isoglutamine |
| PPP4C | BMPR2 |
| DLX4 | TRIB3 |
| CXCL5 | PRKAR2B |
| ABCD2 | GRN |
| RALB | JAK inhibitor I |
| BCL2L12 | hydrochloric acid |
| CASP4 | phorbol 12,13-didecanoate |
| AMBN | SOX3 |
| MAGED1 | delta-9-tetrahydrocannabinol |
| RNF111 | miR-550b-2-5p (and other miRNAs w/seed UGUGCCU) |
| F13A1 | miR-3150a-5p (and other miRNAs w/seed AACCUCG) |
| SRPK1 | AMPK |
| GRM5 | IRF4 |
| DYNLL1 | IRF1 |
| FZD5 | metformin |
| GJA8 | NAMPT |
| CTSK | AIMP1 |
| PPID | BDKRB2 |
| Ifna4 | NRP1 |
| Ppp1cc | GTF2I |
| Paxip1 | EDNRA |
| VTX-2337 | TOPBP1 |
| CXXC4 | dexmedetomidine |
| mito-TEMPO | amlodipine |
| COG112 peptide | Mn(III)-tetrakis-(4-benzoic acid) porphyrin |
| olmesartan medoxomil | gamma-tocotrienol |
| XCT790 | miR-4708-3p (miRNAs w/seed GCAAGGC) |
| emricasan | miR-4777-3p (miRNAs w/seed UACCUCA) |
| betaxolol | CAMP |
| catechol | RHO |
| FC-99 | CSHL1 |
| Z-IETD-FMK | JUND |
| zVAD | miR-6732-3p (miRNAs w/seed AACCCUG) |
| scopoletin | miR-100-3p (miRNAs w/seed AAGCUUG) |
| NHB2005 | ERN1 |
| alpha-hydroxyglutarate | streptozocin |
| CV 3988 | miR-1668 (and other miRNAs w/seed AAAGGCC) |
| palmitoylethanolamide | miR-7110-3p (miRNAs w/seed CUCUCUC) |
| sparfosic acid | miR-1224-3p (miRNAs w/seed CCCACCU) |
| zofenopril | miR-337-3p (and other miRNAs w/seed CAGCUCC) |
| miR-6830-5p (miRNAs w/seed CAAGGAA) | miR-4445-3p (and other miRNAs w/seed ACGGCAA) |
| miR-6823-5p (miRNAs w/seed CAGGGUU) | ACTL6A |
| miR-384-3p (and other miRNAs w/seed UUCCUAG) | ASXL2 |
| miR-133a-5p (and other miRNAs w/seed CUGGUAA) | CFLAR |
| miR-3621 (miRNAs w/seed GCGGGUC) | EFNA5 |
| miR-181a-2-3p (and other miRNAs w/seed CCACUGA) | TXNIP |
| miR-8083 (miRNAs w/seed AGGACUU) | FLT3 |
| miR-181c-3p (miRNAs w/seed ACCAUCG) | acetovanillone |
| miR-4450 (and other miRNAs w/seed GGGGAUU) | Rb |
| miR-7113-5p (and other miRNAs w/seed CCAGGGA) | ascorbic acid |
| miR-5687 (miRNAs w/seed UAGAACG) | miR-22-3p (miRNAs w/seed AGCUGCC) |
| miR-6715b-3p (miRNAs w/seed UCAAACC) | Fcer1 |
| EIF3E | STING1 |
| miR-339-3p (miRNAs w/seed GAGCGCC) | HOXD10 |
| miR-3654 (miRNAs w/seed ACUGGAC) | NRIP1 |
| miR-3585-5p (and other miRNAs w/seed UCACAAG) | glucosamine |
| miR-3137 (miRNAs w/seed CUGUAGC) | miR-1258-5p (and other miRNAs w/seed GCUGAGC) |
| miR-329-3p (and other miRNAs w/seed ACACACC) | miR-708-5p (and other miRNAs w/seed AGGAGCU) |
| miR-933 (miRNAs w/seed GUGCGCA) | miR-3140-5p (miRNAs w/seed CCUGAAU) |
| miR-526a-5p (and other miRNAs w/seed UCUAGAG) | miR-4445-5p (miRNAs w/seed GAUUGUU) |
| salubrinal | GMNN |
| ORAI1 | TRAF2 |
| MESP1 | miR-4326 (miRNAs w/seed GUUCCUC) |
| BMP10 | miR-6810-5p (and other miRNAs w/seed UGGGGAC) |
| PURA | miR-644a (miRNAs w/seed GUGUGGC) |
| ZNF148 | 1,25-dihydroxyvitamin D |
| RAPGEF1 | mir-146 |
| ANGPTL2 | MEF2A |
| STK3 | RIPK1 |
| STIM1 | prostaglandin A2 |
| OSBPL8 | epothilone B |
| Gm12602 | C1q |
| aroclor 1254 | COL1A1 |
| Go6983 | GAS2L3 |
| cystamine | HIF1AN |
| allyl sulfide | UBD |
| miR-1301-3p (and other miRNAs w/seed UGCAGCU) | TGIF1 |
| miR-675-3p (miRNAs w/seed UGUAUGC) | LY96 |
| miR-4425 (miRNAs w/seed GUUGGGA) | CXCL2 |
| FFAR3 | mevastatin |
| FLT1 | indole |
| IL23 | farnesyl pyrophosphate |
| miR-501-5p (miRNAs w/seed AUCCUUU) | caffeic acid |
| miR-548as-3p (miRNAs w/seed AAAACCC) | reserpine |
| miR-4705 (and other miRNAs w/seed CAAUCAC) | lithium chloride |
| IGF2BP1 | miR-6868-3p (miRNAs w/seed UCCUUCU) |
| MEIS1 | indican |
| miR-4999-5p (miRNAs w/seed GCUGUAU) | leucine |
| miR-1197 (and other miRNAs w/seed AGGACAC) | C1QA |
| miR-486-5p (and other miRNAs w/seed CCUGUAC) | SIAH2 |
| miR-6821-3p (and other miRNAs w/seed GACCUCU) | mir-135 |
| IL4R | N(G)-monomethyl-D-arginine |
| TSC1 | miR-892b (miRNAs w/seed ACUGGCU) |
| miR-582-5p (miRNAs w/seed UACAGUU) | miR-2114-5p (miRNAs w/seed AGUCCCU) |
| 1-hydroxy-2-methyl-2-butenyl 4-diphosphate | CFTR |

mGluR
IRF
METTL14
Kallikrein
SLC7A5
MED14
LIMA1
FANCD2
C6
MAS1
CHRNA3
NDFIP1
CELF1
myelin oligodendrocyte glycoprotein (35-55)
phospholipid
mimosine
miR-6820-5p (miRNAs w/seed GCGGCAG)
miR-5002-3p (miRNAs w/seed GACUGCC)
collagen type i (family)
Mir122a,b
ADA
titanium dioxide
L-tryptophan
PLA2G6
MAP3K5
PBX3
coenzyme Q10
agmatine
selenomethylselenocysteine
hemin
miR-4731-3p (and other miRNAs w/seed ACACAAG)
miR-4793-5p (miRNAs w/seed CAUCCUG)
miR-1206 (miRNAs w/seed GUUCAUG)
miR-935 (miRNAs w/seed CAGUUAC)
miR-3925-5p (miRNAs w/seed AGAGAAC)
miR-682 (miRNAs w/seed UGCAGUC)
miR-6841-3p (miRNAs w/seed CCUUGCA)
miR-6508-5p (and other miRNAs w/seed CUAGAAA)
miR-103b (miRNAs w/seed CAUAGCC)
miR-3714 (miRNAs w/seed AAGGCAG)
miR-4694-5p (miRNAs w/seed GGUGUUA)
miR-3192-3p (miRNAs w/seed UCUGAUC)
miR-8068 (miRNAs w/seed GUUUGUU)
TBX2
miR-96-3p (miRNAs w/seed AUCAUGU)
miR-5589-3p (miRNAs w/seed GCACAUG)
miR-5591-3p (miRNAs w/seed UACCCAU)
N-formyl-Met-Leu-Phe
miR-3910 (miRNAs w/seed AAGGCAU)
miR-6850-5p (miRNAs w/seed UGCGGAA)
miR-4742-3p (miRNAs w/seed CUGUAUU)
miR-5584-5p (miRNAs w/seed AGGGAAA)
miR-6739-3p (miRNAs w/seed UUGUUCU)
miR-6760-3p (miRNAs w/seed CACUGUC)
miR-4695-5p (and other miRNAs w/seed AGGAGGC)
ZC3H12A
ABCG1
PRKCQ
UPF1
nicotinic acid
miR-510-3p (miRNAs w/seed UUGAAAC)
miR-1288-3p (miRNAs w/seed GGACUGC)
miR-4682 (miRNAs w/seed CUGAGUU)
miR-514a-3p (and other miRNAs w/seed UUGACAC)
GRIN3A
miR-2392 (miRNAs w/seed AGGAUGG)
miR-1258 (miRNAs w/seed GUUAGGA)
miR-7845-5p (miRNAs w/seed AGGGACA)
miR-3671 (miRNAs w/seed UCAAAUA)
miR-656-3p (miRNAs w/seed AUAUUAU)
miR-3194-3p (miRNAs w/seed GCUCUGC)
miR-4677-3p (miRNAs w/seed CUGUGAG)
miR-483-3p (miRNAs w/seed ACUCCUC)
miR-3117-5p (miRNAs w/seed GACACUA)
miR-3683 (miRNAs w/seed GCGACAU)
miR-497-3p (and other miRNAs w/seed CAAACCA)
miR-4303 (miRNAs w/seed UCUGAGC)
MESP2
mir-7
mir-205
ARG1
NCAM1
FABP5
TARBP2
ECM1
IL19
ethyl pyruvate
rofecoxib
isoliquiritigenin
oxysterol
dasatinib
MEF2
SP4
MEL S3
silymarin
nitroarginine
POSTN
miR-193b-5p (miRNAs w/seed GGGGUUU)
miR-4697-3p (miRNAs w/seed GUCAGUG)
miR-3913-5p (and other miRNAs w/seed UUGGGAC)
miR-6844 (miRNAs w/seed UCUUUGU)
TSPYL5
SKI
TNFAIP6
TUG1
beta-glycerophosphoric acid
DL-fructose
nilotinib
baicalin
LRP1
JAK3
miR-7-1-3p (and other miRNAs w/seed AACAAAU)
miR-651-5p (miRNAs w/seed UUAGGAU)
dorsomorphin
CYP1B1
miR-181d-3p (miRNAs w/seed CACCGGG)
miR-5579-5p (miRNAs w/seed AUGGUAC)
miR-28-3p (and other miRNAs w/seed ACUAGAU)
miR-376a-3p (and other miRNAs w/seed UCAUAGA)
miR-4321 (miRNAs w/seed UAGCGGU)
CYP1A1
pexidartinib
miR-219a-2-3p (miRNAs w/seed GAAUUGU)
miR-3685 (miRNAs w/seed UUCCUAC)
miR-4712-5p (and other miRNAs w/seed CCAGUAC)
miR-5002-5p (miRNAs w/seed AUUUGGU)
miR-626 (and other miRNAs w/seed GCUGUCU)
miR-211-3p (miRNAs w/seed CAGGGAC)
CYP27B1
miR-7978 (miRNAs w/seed CUGGUGU)
miR-12194-3p (and other miRNAs w/seed CUGUGGG)
24(S),25-epoxycholesterol
dopamine D1 receptor
CXCL6
Cd209b
sesamol
Stat1 dimer
doramapimod
blinatumomab
PRCP
PDCD10
PIMREG
EIF2A
ST6GALNAC1
PTK
CC2D1A
TP73-AS1
KMT5B
ADAM21
SFRP5
FNIP1
ZC3HAV1
valine
catumaxomab
SELL
EZH1
DUSP10
CCNA2
mir-296
PTBP3
P2RY4
PIK3C3
MGLL
PIP5K1C
FKBP1A
TRPA1
GLP1R
fluoxetine
spironolactone
miR-1226-3p (miRNAs w/seed CACCAGC)
12-(3-adamantan-1-yl-ureido) dodecanoic acid
ARNTL
MUC1
miR-504-3p (and other miRNAs w/seed GGGAGAG)
miR-6867-3p (and other miRNAs w/seed UCUCCCU)
HMGB1
3,4,5,3',4'-pentachlorobiphenyl
miR-4733-3p (and other miRNAs w/seed CACCAGG)
miR-6840-5p (miRNAs w/seed CCCCCGG)
let-7f-2-3p (and other miRNAs w/seed UAUACAG)
caspase
VAV1
KNG1
ACKR3
VAV2
ramipril
proteasome inhibitor PSI
miR-339-5p (and other miRNAs w/seed CCCUGUC)
Iberiotoxin
tacedinaline
obatoclax
ghrelin
HSPB2
PROK2
SOX18
NUCB2
OTUD7B
DNAJA3
CARD14
CCL15
F2RL3
XPA
ambroxol
EGTA acetoxymethyl ester
ferric nitrilotriacetate
famotidine
pyridoxamine
chlorambucil
propyl gallate
methylmercury
NFYB
miR-6795-5p (and other miRNAs w/seed GGGGGGA)
miR-6783-5p (miRNAs w/seed AGGGGAA)
miR-4514 (and other miRNAs w/seed CAGGCAG)
miR-6878-3p (and other miRNAs w/seed UGGCCUC)
miR-4503 (miRNAs w/seed UUAAGCA)
miR-6776-3p (miRNAs w/seed AACCACC)
miR-4524a-3p (miRNAs w/seed GAGACAG)
lysophosphatidylinositol
FYN
doxifluridine
Smad2/3-Smad4
Angiotensin II receptor type 1
GIP
PHB2
FKBP4
PSMB8
GPI
KLK5
D609
nimodipine
TNFSF12
miR-646 (miRNAs w/seed AGCAGCU)
ATP-gamma-S
SHC1
haloperidol
miR-5699-5p (miRNAs w/seed GCCCCAA)
grape seed extract
ITCH
PTH1R
miR-520g-5p (miRNAs w/seed CUAGAGG)
SCARB1
EGR3
TXN
monocrotaline
miR-3614-3p (miRNAs w/seed AGCCUUC)
miR-3529-3p (miRNAs w/seed ACAACAA)
miR-204-5p (and other miRNAs w/seed UCCCUUU)
miR-635 (and other miRNAs w/seed CUUGGGC)
miR-6800-3p (miRNAs w/seed ACCUCUC)
miR-1-3p (and other miRNAs w/seed GGAAUGU)
Map3k7
NPC1
miR-6758-5p (and other miRNAs w/seed AGAGAGG)
miR-27a-3p (and other miRNAs w/seed UCACAGU)
miR-3136-3p (and other miRNAs w/seed GGCCCAA)
E2F3
miR-4730 (miRNAs w/seed UGGCGGA)
15(S)-HETE
Pdgf Ab
PKNOX2
VGLL3
CRBN
hemoglobin
LYL1
S100B
TNFRSF13C
PLCG2
EREG
MAPKAPK3
cytochalasin D
DOCK8
prostaglandin J2
3M-011
SSRP1
UBE2I
AHCY
GDF11
eplerenone
miR-3584-5p (and other miRNAs w/seed GGAGGAG)
DIO2
miR-624-5p (miRNAs w/seed AGUACCA)
miR-3679-3p (miRNAs w/seed UUCCCCC)
miR-515-5p (and other miRNAs w/seed UCUCCAA)
miR-519d-5p (miRNAs w/seed CUCCAAA)
miR-6871-5p (miRNAs w/seed AUGGGAG)
8-chlorophenylthio-adenosine 3',5'-cyclic monophosphate
IFN type 1
valsartan
miR-136-5p (miRNAs w/seed CUCCAUU)
TGM2
miR-3684 (miRNAs w/seed UAGACCU)
miR-224-5p (miRNAs w/seed AAGUCAC)
alloxan
patulin
saracatinib
MEFV
TSC22D1
TPSD1
TBXA2R
LPAR2
IL18RAP
PLA2G2A
KLKB1
NPAS2
RHOC
15-E2-isoketal modified phosphatidylethanolamine
AP20187
nicorandil
benzoquinone
4-coumaric acid
fluphenazine
2-mercaptoethanol
RWJ 67657
diazepam
manidipine
eugenol
miR-508-3p inhibitor
atropine
12-hydroxyeicosatetraenoic acid
Muscarinic cholinergic receptor
vinpocetine
OSCAR
ZBED6
EZR
SELP
EWSR1
LOC105372576
4-(2-aminoethyl)benzenesulfonylfluoride
miR-455-5p (and other miRNAs w/seed AUGUGCC)
miR-4421 (and other miRNAs w/seed CCUGUCU)
miR-5088-5p (miRNAs w/seed AGGGCUC)
SASH1
Fgfr

KLRB1
CLEC2A
Eda
Pde4d
triacetyl-3-hydroxyphenyladenosine
trimethyltin
hydroxychloroquine
PQQ cofactor
nigericin
poly rI:rC-RNA HMW
Ki16425
BAR501
dBET6
Boc-D-FMK
murabutide
vinorelbine
tyrphostin AG825
20alpha-hydroxycholesterol
miR-4677-5p (miRNAs w/seed UGUUCUU)
miR-4680-3p (miRNAs w/seed CUGAAUU)
miR-692 (and other miRNAs w/seed UCUCUUU)
clobetasol propionate
SLC7A2
STAR
PRAME
AIM2
KU-60019
doxazosin
sanglifehrin A
rhein
tetrathiomolybdate
miR-4424 (miRNAs w/seed GAGUUAA)
miR-645 (miRNAs w/seed CUAGGCU)
miR-143-5p (and other miRNAs w/seed GUGCAGU)
miR-487a-5p (and other miRNAs w/seed UGGUUAU)
miR-336-3p (and other miRNAs w/seed CUGGAUU)
miR-7032-3p (and other miRNAs w/seed UCCUCUC)
miR-555 (miRNAs w/seed GGGUAAG)
RBL2
miR-1245b-3p (miRNAs w/seed CAGAUGA)
FOXP1
miR-4662a-5p (miRNAs w/seed UAGCCAA)
miR-490-3p (miRNAs w/seed AACCUGG)
miR-193a-5p (miRNAs w/seed GGGUCUU)
miR-5112 (and other miRNAs w/seed AGCUCAG)
fluocinolone acetonide
sulprostone
Rp-8-Br-cGMPS
sangivamycin
N6-cyclohexyladenosine
deoxyguanosine
lopinavir
trapidil
ergosterol-5,8-peroxide
stigmasterol
glycodeoxycholic acid
IGF receptor
MAP1LC3
monooleylphosphatidic acid
cortisone
C1QTNF12
omalizumab
(diaminocyclohexane)(diacetato)(dichloro)platinum
AMOT
ixabepilone
Cyclin B
DUB
chlormethiazole
methysticin
yangonin
NIK
1,2,5,6-dibenzanthracene
cardamomin
DEFA1 (includes others)
irisolidone
dietary fat derivative
imperatorin
IGF2-AS
TRIM13
KDM4D
polyethyleneimine
CISD1
CHIA
DDX41
PTPMT1
TRIM66
Il3r
CHD5
SMCR8
ZNF451
PRR7
EDEM1
Mir124a-1hg
KLF14
PSMD14
potassium channel
Ribosomal Subunit 80S
DGKH
TRIM67
ESRP2
AMOTL1
ASH1L
TRIM36
PTPN23
SCARB2
INAVA
NAAA
ganglioside GM3 (d18:1/16:0)
ganglioside GM3 (d18:1/24:0)
ganglioside GM3 (d18:1/24:1(15Z))
TH17 Cytokine
isoleucine
ATP1A2
pinoresinol
vistusertib
foretinib
DAPK1
GCA
MR1
TPSG1
BAG4
PSMC5
YARS1
17alpha-hydroxyprogesterone caproate
CLIP1
U2AF2
LYZ
PKC delta V1-1 peptide 8-17
CLCN3
CUL4A
MADD
9-benzyl-8-hydroxy-2-(2-methoxyethoxy)adenine
SLC1A1
TRIM9
PBK
GW 6471
CARD8
KLF16
CEACAM6
SERPINB3
CEACAM21
IFI30
EIF2B1
MID2
GZMA
EID2
MKNK2
MLST8
DLST
FCAR
IFNGR2
STAG2
LSS
Defb2
GJC1
KRT5
Trim30a/Trim30d
TRERF1
USP27X
CEACAM7
RASSF7
ELOA
BTG1
NPHS2
HES6
DHCR7
ARHGEF11
CBX7
IL31
NUMB
SMURF1
ITGB4
KCNIP3
CCNH
GSK3A
MUC4
TREM2
EMD
NRTN
diethylmaleate
helenalin
dichlororibofuranosylbenzimidazole
miR-134-3p (and other miRNAs w/seed UGUGGGC)
ZBTB20
TEAD3
2,3,4,7,8-pentachlorodibenzofuran
miR-936 (miRNAs w/seed CAGUAGA)
aldosterone
miR-6815-3p (miRNAs w/seed GGCUUCU)
miR-190a-5p (and other miRNAs w/seed GAUAUGU)
miR-8074 (miRNAs w/seed UAUGGCG)
miR-6511a-5p (and other miRNAs w/seed AGGCAGA)
mir-21
RC3H1
resolvin D1
CYBB
sildenafil
diclofenac
indole-3-carbinol
miR-4718 (miRNAs w/seed GCUGUAC)
bromodeoxyuridine
GHR
miR-548at-5p (miRNAs w/seed AAAGUUA)
TRAF3IP2
miR-4704-3p (miRNAs w/seed CAGUCAC)
miR-1247-3p (miRNAs w/seed CCCGGGA)
miR-556-5p (miRNAs w/seed AUGAGCU)
11,12-epoxyeicosatrienoic acid
PHLPP1
CDK5R1
mir-101
mir-218
MLX
AURKB
LY6E
RPA1
pevonedistat
HULC
ammonium chloride
farnesol
miR-139-5p (miRNAs w/seed CUACAGU)
miR-3065-3p (miRNAs w/seed CAGCACC)
IL12 (family)
LMNA
CEBPE
LPL
salirasib
arginine
CASP1
IGFBP3
NLRP12
rolipram
PML-RARA
exenatide
miR-182-5p (and other miRNAs w/seed UUGGCAA)
CALCA
melatonin
5-hydroxytryptamine
geldanamycin
miR-452-3p (miRNAs w/seed UCAUCUG)
miR-203a-3p (and other miRNAs w/seed UGAAAUG)
miR-3613-3p (miRNAs w/seed CAAAAAA)
SOX11
miR-6511a-3p (and other miRNAs w/seed CUCACCA)
UCP1
miR-421-3p (and other miRNAs w/seed UCAACAG)
miR-4535 (miRNAs w/seed UGGACCU)
HDL
FGF10
EHF
miR-4747-5p (and other miRNAs w/seed GGGAAGG)
miR-4686 (miRNAs w/seed AUCUGCU)
15-epi-lipoxin A4
UDP
saikosaponin A
2,5-bis(5-hydroxymethyl-2-thienyl)furan
L-type Calcium Channel
SENP7
GSTK1
IL-2R
IL26
HDAC10
BAG6
SLC25A4
CYSLTR1
HS6ST1
EFEMP1
USP2
ARAF
ATF7
MAP3K10
TBL1X
TSPO
DIABLO
SERPINH1
TBL1XR1
raclopride
domoic acid
carnosol
tyrphostin
alpha-tocopherol succinate
miR-3156-3p (miRNAs w/seed UCCCACU)
3M-001
TRH
PLP1
GNB2
CUL4B
RAD21
miR-1252-5p (miRNAs w/seed GAAGGAA)
miR-1237-3p (and other miRNAs w/seed CCUUCUG)
Lymphotoxin
TNFRSF18
SOCS6
A-Fos
PARK7
pilocarpine
miR-4300 (and other miRNAs w/seed GGGAGCU)
Ifn gamma
miR-196a-3p (miRNAs w/seed GGCAACA)
ATG7
miR-3612 (and other miRNAs w/seed GGAGGCA)
ACSL4
BIRC5
miR-4713-3p (miRNAs w/seed GGGAUCC)
miR-571 (miRNAs w/seed GAGUUGG)
eicosapentenoic acid
3-methyladenine
CERK
NSD2
TSLP
SPRY2
SAA1
MMP2
cilostazol
steroid
GATA1
cerivastatin
miR-1934-5p (and other miRNAs w/seed CUGGUCC)
miR-1287-5p (miRNAs w/seed GCUGGAU)
miR-7152-5p (miRNAs w/seed UUCCUGU)
miR-3098-3p (and other miRNAs w/seed UCUGCUG)
EFNA2
ETV5
miR-3913-3p (miRNAs w/seed GACAUCA)
CaMKII
Tnf receptor
CCK
GLP-1-(7-34)-amide
wogonin
miR-4436b-3p (and other miRNAs w/seed AGGGCAG)
ZBTB16
miR-1343-3p (and other miRNAs w/seed UCCUGGG)
SAA
CYLD

Miat
URMC-099
VEZF1
RAN
TCP1
CASP10
RING1
Acp5
CRIP2
Irs4
NKRF
TIGAR
IRX1
UIMC1
CNTFR
mercaptosteroid 4
mercaptosteroid 6
P2pal-18S
C20-D3-vitamin A
beta2 adrenergic receptor agonist
N6-carboxymethyl-lysine bovine serum albumin
singlet oxygen
temocapril
nordihydrocapsiate
methoxyacetic acid
nitrous oxide
dichlorodiphenyl dichloroethylene
tempo
mecamylamine
amantadine
prinaberel
fomepizole
astemizole
BB-2116
ODN1411
cibacron blue F 3GA
beta-lapachone
phosphorylcholine
triphenyltin
albendazole
10-hydroxy docosahexaenoic acid
cycloserine
pegaptanib
oseltamivir
DL-threo-dihydrosphingosine
ketotifen
modafinil
10-decarbamoylmitomycin C
nomifensine
MLN-4760
phloretin
mycophenolate mofetil
sodium iodide
BRF110
SYUIQ-5
5-phenylvaleric acid
C66
yoda1
potassium tetraperoxochromate
aripiprazole
L-JNK inhibitor I
TAPI
glutathione diethyl ester
Z-LEHD-FMK
tenidap
(indol-3-yl)pyruvic acid
muscarine
ellipticine
physostigmine
3,7-dimethyl-1-propargylxanthine
strychnine
SR 31747
CH6953755
danazol
epiallopregnanolone
miR-4788 (miRNAs w/seed UACGGAC)
GC-GCR dimer
mir-31
VAV3
ferulic acid
plerixafor
glycyrrhizic acid
miR-382-5p (miRNAs w/seed AAGUUGU)
miR-4698 (miRNAs w/seed CAAAAUG)
miR-544-5p (miRNAs w/seed CUUGUUA)
miR-5006-5p (miRNAs w/seed UGCCAGG)
miR-3672 (and other miRNAs w/seed UGAGACU)
miR-5695 (miRNAs w/seed CUCCAAG)
SIM1
miR-12204-5p (and other miRNAs w/seed UGAGUCU)
miR-1468-5p (miRNAs w/seed UCCGUUU)
GNL3
1-methyl-4-phenylpyridinium
8-(4-chlorophenylthio)-guanosine-3', 5'-cyclic monophosphate
Ctbp
NMDA Receptor
KLK4
FZD8
DEF6
SOD3
TACR1
ITGAL
PDK4
pomalidomide
methylphenidate
L-carnitine
propionic acid
glycyrrhetinic acid
iloprost
miR-8055 (miRNAs w/seed UUUGAGC)
miR-498-5p (miRNAs w/seed UUCAAGC)
miR-4652-3p (miRNAs w/seed UUCUGUU)
folic acid
miR-4679 (miRNAs w/seed CUGUGAU)
miR-125b-1-3p (miRNAs w/seed CGGGUUA)
miR-509-5p (and other miRNAs w/seed ACUCCAG)
miR-4540 (miRNAs w/seed UAGUCCU)
miR-324-3p (miRNAs w/seed CCACUGC)
POU2AF1
miR-12185-3p (and other miRNAs w/seed CAGCCAC)
ZNF106
ILK
miR-651-3p (miRNAs w/seed AAGGAAA)
RETNLB
miR-3618 (miRNAs w/seed GUCUACA)
miR-3924 (miRNAs w/seed UAUGUAU)
miR-1270 (and other miRNAs w/seed UGGAGAU)
miR-574-3p (miRNAs w/seed ACGCUCA)
miR-5092 (miRNAs w/seed AUCCACG)
miR-451a (and other miRNAs w/seed AACCGUU)
PIK3CA
miR-3200-3p (miRNAs w/seed ACCUUGC)
miR-589-5p (and other miRNAs w/seed GAGAACC)
miR-4665-3p (miRNAs w/seed UCGGCCG)
miR-873-3p (miRNAs w/seed GAGACUG)
Ubiquitin
Nuclear factor 1
Rhox5
DRD2
miR-3922-5p (miRNAs w/seed CAAGGCC)
miR-1286 (miRNAs w/seed GCAGGAC)
miR-3184-3p (miRNAs w/seed AAGUCUC)
miR-6516-5p (miRNAs w/seed UUGCAGU)
miR-1282 (miRNAs w/seed CGUUUGC)
MXD1
miR-6853-3p (miRNAs w/seed GUUCAUU)
miR-4756-3p (miRNAs w/seed CAGAGAU)
telmisartan
miR-4774-5p (miRNAs w/seed CUGGUAU)
miR-4773 (miRNAs w/seed AGAACAG)
miR-3920 (miRNAs w/seed CUGAUUA)
CGA
miR-224-3p (and other miRNAs w/seed AAAUGGU)
miR-4327 (miRNAs w/seed GCUUGCA)
MEX3A
miR-374b-5p (and other miRNAs w/seed UAUAAUA)
miR-302c-5p (miRNAs w/seed UUAACAU)
MM-589
PTC-209
miR-5586-3p (miRNAs w/seed AGAGUGA)
AKT inhibitor VIII
particulate matter
GPBAR1
RYR2
YWHAQ
CTSG
mir-373
GNB1
PRKN
CGS 21680
miR-567 (miRNAs w/seed GUAUGUU)
miR-143-3p (and other miRNAs w/seed GAGAUGA)
CCL11
CpG ODN 1668
miR-544b (miRNAs w/seed CCUGAGG)
miR-8053 (miRNAs w/seed GGCGAUU)
miR-3667-3p (miRNAs w/seed CCUUCCU)
CLDN7
miR-465d-3p (and other miRNAs w/seed GAUCAGG)
ganglioside
cholesterol sulfate
Pde
FBXO42
NEK7
HUWE1
SMAD1/5
SPTAN1
pictilisib
CD244
PRTN3
ATG16L1
GUSB
CD160
COCH
MC1R
BRMS1
NELFE
RECK
niflumic acid
RP 73401
prodigiosin
ketorolac
HNRNPA2B1
miR-659-3p (miRNAs w/seed UUGGUUC)
miR-2277-3p (miRNAs w/seed GACAGCG)
miR-5582-5p (miRNAs w/seed AGGCACA)
miR-887-5p (miRNAs w/seed UUGGGAG)
miR-450b-5p (miRNAs w/seed UUUGCAA)
1810019D21Rik
miR-3152-5p (miRNAs w/seed UUGCCUC)
miR-4759 (miRNAs w/seed AGGACUA)
miR-3093-3p (and other miRNAs w/seed GUGGACA)
BMP15
EIF4EBP2
9Z,11E-octadecadienoic acid
miR-4264 (miRNAs w/seed CUCAGUC)
miR-6721-5p (miRNAs w/seed GGGCAGG)
MIR17HG
miR-1283 (miRNAs w/seed CUACAAA)
miR-548o-3p (and other miRNAs w/seed CAAAACU)
miR-3168 (miRNAs w/seed AGUUCUA)
miR-4722-5p (miRNAs w/seed GCAGGAG)
miR-1253 (miRNAs w/seed GAGAAGA)
miR-6742-3p (miRNAs w/seed CCUGGGU)
CHD4
PTGER2
MUC2
GAST
sodium chloride
miR-4494 (miRNAs w/seed CAGACUG)
miR-19b-1-5p (and other miRNAs w/seed GUUUUGC)
miR-6894-3p (miRNAs w/seed UGCCUGC)
LGR4
RNASEH2A
4-methylnitrosoamino-1-(3-pyridinyl)-1-butanone
eflornithine
laminaran
miR-512-3p (miRNAs w/seed AGUGCUG)
miR-1255b-5p (and other miRNAs w/seed GGAUGAG)
arsenic trioxide
miR-298 (miRNAs w/seed GCAGAAG)
TAL1
risperidone
RNF20
IL36B
vasoactive intestinal peptide
LINC00963
BMPER
PLD1
FOXC1
RACK1
chelerythrine
poly dA-dT
SB 290157
STUB1
clozapine
miR-3616-3p (miRNAs w/seed GAGGGCA)
miR-4785 (miRNAs w/seed GAGUCGG)
miR-6501-5p (miRNAs w/seed GUUGCCA)
miR-6746-5p (miRNAs w/seed CGGGAGA)
DNMT3B
miR-642a-3p (and other miRNAs w/seed GACACAU)
miR-4733-5p (miRNAs w/seed AUCCCAA)
miR-542-3p (miRNAs w/seed GUGACAG)
miR-6818-3p (miRNAs w/seed UGUCUCU)
miR-4755-3p (miRNAs w/seed GCCAGGC)
CSF1R
PTGS1
S-nitrosoglutathione
FAT1
ganetespib
PF-3084014
ITGA4
NCK1
GPC1
PCBP2
butylhydroxybutylnitrosamine
diltiazem
allyl isothiocyanate
calmidazolium
tiron
SU1498
tin mesoporphyrin
polymyxin B
miR-5624-3p (and other miRNAs w/seed UAAGGCA)
miR-4493 (miRNAs w/seed GAAGGCC)
miR-31-3p (and other miRNAs w/seed GCUAUGC)
mir-27
SCD
C5AR1
miR-5579-3p (miRNAs w/seed UAGCUUA)
miR-331-3p (miRNAs w/seed CCCCUGG)
miR-513b-3p (miRNAs w/seed AAUGUCA)
NFATC1
miR-4767 (miRNAs w/seed GCGGGCG)
ONECUT1
FBXO32
SPIB
miR-1911-5p (miRNAs w/seed GAGUACC)
miR-1184 (miRNAs w/seed CUGCAGC)
SMARCA5
miR-6798-3p (miRNAs w/seed UACCCCC)
SLPI
mir-214
BTRC
ibrutinib
HEXIM1
TRPV4
ceruletide
miR-584-3p (miRNAs w/seed CAGUUCC)
miR-3945 (miRNAs w/seed GGGCAUA)
PTPRC
fasudil
beta-carotene
4-hydroxynonenal
miR-31-5p (and other miRNAs w/seed GGCAAGA)
miR-1973 (miRNAs w/seed CCGUGCA)
miR-3575 (and other miRNAs w/seed CUGCUGG)
miR-1304-5p (miRNAs w/seed UUGAGGC)
miR-1207-3p (and other miRNAs w/seed CAGCUGG)
ITK
miR-6883-3p (miRNAs w/seed UCCCUAU)
miR-323b-3p (miRNAs w/seed CCAAUAC)
HOXA9
LEPR
miR-3200-5p (miRNAs w/seed AUCUGAG)
DDX5
miR-142-3p (and other miRNAs w/seed GUAGUGU)
TNC
CDK2
miR-4653-3p (miRNAs w/seed GGAGUUA)
Nr1h
alitretinoin
PYCARD

CCL21
LMX1A
PIM3
UGCG
PEX5L
OPRM1
MIA-602
RV 538
isatoribine
S-equol
NCOR-LXR-Oxysterol-RXR-9 cis RA
NMU
miR-3152-3p (and other miRNAs w/seed GUGUUAG)
miR-4329 (miRNAs w/seed CUGAGAC)
Hsp90
TCL1A
miR-6499-5p (miRNAs w/seed CGGGCGC)
miR-218-2-3p (and other miRNAs w/seed AUGGUUC)
miR-1247-5p (miRNAs w/seed CCCGUCC)
miR-122-3p (miRNAs w/seed ACGCCAU)
miR-4712-3p (miRNAs w/seed AUGAGAG)
miR-767-3p (miRNAs w/seed CUGCUCA)
miR-507 (and other miRNAs w/seed UUUGCAC)
miR-6770-5p (miRNAs w/seed GAGAAGG)
miR-3189-3p (miRNAs w/seed CCUUGGG)
CHEK1
PTPN2
NT5E
KLRC4-KLRK1/KLRK1
pirfenidone
vitamin K2
CpdX
vanadate
miR-1229-3p (miRNAs w/seed UCUCACC)
miR-1970 (and other miRNAs w/seed GUGUCAC)
miR-6785-3p (miRNAs w/seed CAUCGCC)
miR-6812-3p (miRNAs w/seed CGCUCUU)
miR-3938 (miRNAs w/seed AUUCCCU)
miR-1243 (miRNAs w/seed ACUGGAU)
miR-454-5p (miRNAs w/seed CCCUAUC)
miR-8076 (miRNAs w/seed AUAUGGA)
miR-6760-5p (miRNAs w/seed AGGGAGA)
BNIP3L
miR-7975 (miRNAs w/seed UCCUAGU)
miR-7162-3p (miRNAs w/seed CUGAGGU)
12(S)-hydroxyeicosatetraenoic acid
L-phenylalanine
ganglioside GM3
ganglioside GD1a
GCS-100
2-amino-5-azotoluene
REL/RELA/RELB
ibudilast
tectorigenin
CYTOR
GPR84
PPT1
kremezin
SLC39A14
C-21
PNU282987
S100A10
hydrogel
AZI2
LITAF
CD8A
CD5L
PLCD1
DNM1L
DGKZ
SDC2
RNF41
GHSR
CLTC
PLA2G5
Havcr1
PBX2
SLC19A1
CAPN2
NCR3
L-lactic acid
myxothiazol
latrunculin A
epoxyeicosatrienoic acid analog B
epoxyeicosatrienoic acid analog A
dihematoporphyrin ether
miR-485-3p (and other miRNAs w/seed UCAUACA)
bisphenol A
miR-941 (miRNAs w/seed ACCCGGC)
ADORA2A
miR-7848-3p (miRNAs w/seed UACCCUC)
CD274
miR-30b-3p (and other miRNAs w/seed UGGGAUG)
miR-4435 (miRNAs w/seed UGGCCAG)
TNFRSF9
paricalcitol
miR-3921 (and other miRNAs w/seed CUCUGAG)
RUVBL1
Hbb-b2
miR-5011-3p (and other miRNAs w/seed UGCAUGG)
miR-6830-3p (miRNAs w/seed GUCUUUC)
miR-4699-5p (miRNAs w/seed GAAGAUU)
miR-3605-5p (and other miRNAs w/seed GAGGAUG)
miR-6772-5p (miRNAs w/seed GGGUGUA)
miR-6831-3p (miRNAs w/seed GACUAAC)
miR-380-3p (and other miRNAs w/seed AUGUAGU)
miR-4765 (miRNAs w/seed GAGUGAU)
miR-383-3p (miRNAs w/seed CAGCACU)
miR-320e (miRNAs w/seed AAGCUGG)
miR-1227-3p (miRNAs w/seed GUGCCAC)
miR-7154-5p (miRNAs w/seed UCAUGAA)
miR-1231 (miRNAs w/seed UGUCUGG)
RAG2
SPRY1
curdlan
miR-4703-3p (miRNAs w/seed GUAGUUG)
miR-6881-3p (miRNAs w/seed UCCUCUU)
tetracycline
PLA2G10
miR-140-3p (and other miRNAs w/seed ACCACAG)
miR-6501-3p (miRNAs w/seed CAGAGCA)
miR-3158-3p (miRNAs w/seed AGGGCUU)
miR-425-3p (miRNAs w/seed UCGGGAA)
miR-203a-5p (and other miRNAs w/seed GUGGUUC)
miR-6859-5p (and other miRNAs w/seed AGAGGAA)
miR-4528 (miRNAs w/seed CAUUAUA)
miR-4694-3p (miRNAs w/seed AAAUGGA)
miR-6878-5p (miRNAs w/seed GGGAGAA)
miR-194-5p (miRNAs w/seed GUAACAG)
SETDB1
NPPB
miR-3606-5p (miRNAs w/seed UAGUGAA)
miR-5581-3p (miRNAs w/seed UCCAUGC)
miR-7109-3p (miRNAs w/seed AAGCCUC)
miR-4669 (miRNAs w/seed GUGUCCG)
miR-7056-5p (and other miRNAs w/seed GUGGAGG)
miR-3160-5p (miRNAs w/seed GCUUUCU)
GAS5
PKNOX1
LCP2
STAU1
BID
acrolein
zymosan A
miR-3064-5p (and other miRNAs w/seed CUGGCUG)
ZBTB7B
miR-576-3p (miRNAs w/seed AGAUGUG)
miR-4680-5p (miRNAs w/seed GAACUCU)
exisulind
Ginkgo biloba
FFAR4
SGPP1
TRAF3IP3
FH
TNFAIP8L2
elastase
PECAM1
APOA4
CBLB
CBX2
XRCC5
TRAF4
NHLH2
NPFF
GRIN1
HNRNPD
DMH1
PSMD10
HSPD1
immethridine
colistin
miR-5585-5p (miRNAs w/seed GAAGUAC)
miR-876-3p (miRNAs w/seed GGUGGUU)
KDM1A
miR-292-3p (and other miRNAs w/seed AGUGCCG)
nitroglycerin
teriflunomide
coal tar
APOL1
T 0070907
TRIM25
FFAR1
ruxolitinib
astragalin
GPR132
RLIM
PDE3B
IL1R2
WWP2
CEBPG
IL18BP
JMJD1C
S100A7
J11-Cl
acriflavine
oligomycin
2-aminoethoxydiphenylborane
zileuton
clioquinol
orlistat
manumycin A
rosmarinic acid
Z-DEVD-FMK
scopolamine
tyrphostin AG 1296
allopregnanolone
canrenoate potassium
miR-6734-5p (and other miRNAs w/seed UGAGGGG)
miR-3199 (and other miRNAs w/seed GGGACUG)
birabresib
miR-664b-5p (miRNAs w/seed GGGCUAA)
miR-877-3p (miRNAs w/seed CCUCUUC)
PRDM5
puromycin aminonucleoside
miR-655-5p (miRNAs w/seed GAGGUUA)
miR-431-5p (and other miRNAs w/seed GUCUUGC)
lipid A
RBM5
N-[N-(3,5-difluorophenacetyl-L-Ala)]-S-phenylglycine t-butyl ester
miR-921 (miRNAs w/seed UAGUGAG)
miR-453 (and other miRNAs w/seed GGUUGCC)
kanamycin A
miR-3162-5p (miRNAs w/seed UAGGGAG)
BMI1
hydroxyurea
JINK1/2
p70 S6k
AMH
NCL
CTSS
BGN
SLC9A3
acetylcholine
miR-5684 (miRNAs w/seed ACUCUAG)
miR-200a-5p (and other miRNAs w/seed AUCUUAC)
miR-6886-3p (miRNAs w/seed GCCCUUC)
HOTAIR
IL12A
hexamethylene bisacetamide
miR-6901-5p (miRNAs w/seed GCAGGAA)
miR-6078 (miRNAs w/seed CGCCUGA)
miR-129-1-3p (and other miRNAs w/seed AGCCCUU)
vitamin E
HAND2
miR-4645-3p (miRNAs w/seed GACAGUA)
miR-6888-3p (and other miRNAs w/seed UCUGUCU)
miR-1296-3p (and other miRNAs w/seed AGUGGGG)
black raspberry extract
miR-5189-3p (and other miRNAs w/seed GCCAACC)
miR-609 (miRNAs w/seed GGGUGUU)
PP2A
naringin
highly active antiretroviral therapy
METTL3
HCAR1
DEPTOR
FLZ
PELI1
CCAT1
UBA1
NACC1
HSPB8
BEX2
CXCL1
HNRNPAB
roflumilast N-oxide
miR-6125 (miRNAs w/seed CGGAAGG)
2-(4-acetoxyphenyl)-2-chloro-N-methylethylamine
NCF1
HMGN1
TAF6
MALP-2s
apomorphine
arsenic
sterol
obeticholic acid
TCF7
K+
tetrodotoxin
CX3CL1
miR-585-5p (miRNAs w/seed UAGCACA)
miR-6513-5p (miRNAs w/seed UUGGGAU)
miR-670-3p (miRNAs w/seed UUCCUCA)
POU2F2
miR-4744 (miRNAs w/seed CUAAAGA)
miR-6849-3p (miRNAs w/seed CCAGCCU)
miR-126a-3p (and other miRNAs w/seed CGUACCG)
mibolerone
miR-1287-3p (and other miRNAs w/seed UCUAGCC)
Pro-inflammatory Cytokine
mir-15
miR-340-3p (and other miRNAs w/seed CCGUCUC)
NOS3
zVAD-FMK
6-hydroxydopamine
SND1
NFIL3
NPC2
IRAK3
PD 123319
hemozoin
deoxynivalenol
miR-1914-5p (miRNAs w/seed CCUGUGC)
metronidazole
miR-548s (miRNAs w/seed UGGCCAA)
miR-124-5p (miRNAs w/seed GUGUUCA)

CpdX-D3
diacylglycerol
E64d
N-acetylglucosamine
manganese
USP22
LATS2
anandamide
miR-210-5p (and other miRNAs w/seed GCCACUG)
miR-147-5p (and other miRNAs w/seed GGAAACA)
miR-3164 (and other miRNAs w/seed GUGACUU)
miR-324-3p (and other miRNAs w/seed CACUGCC)
miR-6768-5p (miRNAs w/seed ACACAGG)

**Supplementary Table S23. Unique and overlapping Upstream Regulators for the compassion of the conditions "Caco-2_SARS-CoV-2" and "Caco-2_SARS-CoV".** Comparative analysis was done using VENNY 2.1. Data visualization was done by R-based Venn diagrams shown in Figure 5.

**1235 elements included exclusively in "Caco-2_SARS-CoV-2":**

thyroid hormone
MAPK8IP1
IPMK
OGT
NMB
nitrosonifedipine
CD3E
green tea polyphenol
SETD2
bromobenzene
FBXW7
MEG3
Betacatenin/TCF
exendin(9-39) amide
potassium channel
KLHL2
ADI PEG20
CSNK1E
ASS1
RING1
verapamil
triclosan
lisinopril
SCP2
proanthocyanidin derivative
RAB11FIP3
CFL1
ibotenic acid
candesartan
methamphetamine
2-mercaptoacetate
acetaldehyde
miR-4302 (miRNAs w/seed CAGUGUG)
MAPK12
Ccl2
miR-34a-3p (miRNAs w/seed AAUCAGC)
IL2RA
RNF187
Gpcr
TCF12
miR-188-3p (miRNAs w/seed UCCCACA)
XBP1
miR-337-5p (miRNAs w/seed AACGGCU)
MTA2
ACE2
miR-5003-5p (miRNAs w/seed CACAACA)
NTF3
miR-4269 (and other miRNAs w/seed CAGGCAC)
Tcf 1/3/4
SLC7A11-AS1
EGFL7
RASA1
saquinavir
catechol
apamin
CCR1
NR5A1
GnRH analog
GC-GCR dimer
ATE1
butylidenephthalide
RIPK2
H2AZ1
ALDH1A2
UXT
irbesartan
GJA1
ZBTB7A
taurine
PBX2
Calcineurin A
CDK5
ING1
GAPDH
BLVRA
Ferritin
bicyclol
ING4
acrylamide
miR-133a-3p (and other miRNAs w/seed UUGGUCC)
fingolimod
FOXM1
ALOX5
TCF3
LHCGR
NAP1L1
AMBP
NPAT
RANBP9
MAPT
miR-4446-5p (miRNAs w/seed UUUCCCU)
G2535
triptorelin
S100A4
lipid
CST3
pentylenetetrazol
baclofen
putrescine
miR-3691-5p (miRNAs w/seed GUGGAUG)
miR-3121-3p (miRNAs w/seed AAAUAGA)
D-fructose
miR-3681-5p (and other miRNAs w/seed AGUGGAU)
BCO1
SPINT2
aristolochic acid I
hydroquinone
SKF-38393
miR-202-5p (miRNAs w/seed UCCUAUG)
miR-4696 (miRNAs w/seed GCAAGAC)
AIP
lenalidomide
NR1H2
PSMB10
TRAF4
ILF3
HNRNPD
dimethyl fumarate
anti-benzo(a)pyrene-diol-epoxide
CD86
methimazole
Nos
THRAP3
PCSK9
PNN
mir-199
GHRHR
cyproterone acetate
24-hydroxycholesterol
KAT5
PTPN1
glycerol
BMP10
BLNK
concanamycin A
DCN
WWTR1

**1332 elements included exclusively in "Caco-2_SARS-CoV":**

miR-4651 (and other miRNAs w/seed GGGGUGG)
miR-3151-5p (and other miRNAs w/seed GUGGGGC)
miR-4640-5p (and other miRNAs w/seed GGGCCAG)
miR-149-3p (and other miRNAs w/seed GGGAGGG)
IRF2
miR-4667-5p (and other miRNAs w/seed CUGGGGA)
miR-3072 (and other miRNAs w/seed GCCCCCU)
miR-6825-5p (and other miRNAs w/seed GGGGAGG)
let-7a-5p (and other miRNAs w/seed GAGGUAG)
miR-12192-5p (and other miRNAs w/seed GUGGGGU)
miR-1909-3p (and other miRNAs w/seed GCAGGGG)
miR-29b-3p (and other miRNAs w/seed AGCACCA)
miR-637 (and other miRNAs w/seed CUGGGGG)
miR-6756-5p (and other miRNAs w/seed GGGUGGG)
miR-4271 (and other miRNAs w/seed GGGGAAG)
SS18
miR-6929-5p (and other miRNAs w/seed GGGAGGA)
miR-4731-5p (miRNAs w/seed GCUGGGG)
miR-762 (and other miRNAs w/seed GGGCUGG)
miR-3083-5p (and other miRNAs w/seed GGCUGGG)
miR-874-5p (miRNAs w/seed GGCCCCA)
miR-4723-5p (and other miRNAs w/seed GGGGGAG)
miR-1273h-5p (and other miRNAs w/seed UGGGAGG)
miR-324-5p (miRNAs w/seed GCAUCCC)
miR-6829-5p (and other miRNAs w/seed GGGCUGC)
EHMT1
miR-3473b (and other miRNAs w/seed GGCUGGA)
miR-1913 (miRNAs w/seed CUGCCCC)
NQO2
miR-491-5p (and other miRNAs w/seed GUGGGGA)
BMP6
miR-6967-5p (and other miRNAs w/seed AGGGAGG)
E. coli serotype 0127B8 lipopolysaccharide
miR-4749-3p (miRNAs w/seed GCCCCUC)
CISH
miR-485-5p (and other miRNAs w/seed GAGGCUG)
miR-6880-5p (miRNAs w/seed GGUGGAG)
IL10RA
miR-92a-2-5p (miRNAs w/seed GGUGGGG)
miR-1914-3p (and other miRNAs w/seed GAGGGGU)
EGLN1
miR-125b-5p (and other miRNAs w/seed CCCUGAG)
VEGFD
ABT-737
miR-6132 (and other miRNAs w/seed GCAGGGC)
PROK1
miR-4758-3p (miRNAs w/seed GCCCCAC)
Igm
miR-3189-5p (miRNAs w/seed GCCCCAU)
CRH
miR-6762-5p (and other miRNAs w/seed GGGGCCA)
CTCF
miR-3943 (miRNAs w/seed AGCCCCC)
miR-4472 (and other miRNAs w/seed GUGGGGG)
miR-5586-5p (miRNAs w/seed AUCCAGC)
miR-7108-3p (miRNAs w/seed CCCGCCC)
paraquat
miR-2682-5p (and other miRNAs w/seed AGGCAGU)
miR-4433b-5p (miRNAs w/seed UGUCCCA)
cetuximab
miR-218-1-3p (miRNAs w/seed UGGUUCC)
RORA
miR-558 (and other miRNAs w/seed GAGCUGC)
miR-194-3p (and other miRNAs w/seed CAGUGGG)
miR-185-3p (and other miRNAs w/seed GGGGCUG)
miR-1972 (miRNAs w/seed CAGGCCA)
miR-330-5p (and other miRNAs w/seed CUCUGGG)
MIR101
miR-361-3p (miRNAs w/seed CCCCCAG)
POR
miR-199a-5p (and other miRNAs w/seed CCAGUGU)
TCF7L2
miR-1343-5p (and other miRNAs w/seed GGGGAGC)
miR-6806-5p (miRNAs w/seed GUAGGCA)
BCL2L1
miR-4267 (miRNAs w/seed CCAGCUC)
miR-625-5p (and other miRNAs w/seed GGGGGAA)
miR-5129-5p (and other miRNAs w/seed UGUGGGG)
miR-4667-3p (miRNAs w/seed CCCUCCU)
miR-4722-3p (and other miRNAs w/seed CCUGCCA)
miR-1249-5p (and other miRNAs w/seed GGAGGGA)
MTOR
ARID4B
DNMT3A
KDM5B
miR-6782-5p (and other miRNAs w/seed AGGGGUG)
SCAP
miR-4283 (miRNAs w/seed GGGGCUC)
mir-143
leuprolide
idarubicin
miR-1207-5p (and other miRNAs w/seed GGCAGGG)
miR-4690-3p (and other miRNAs w/seed CAGCCCA)
HOXC6
IL6R
miR-4316 (miRNAs w/seed GUGAGGC)
ARID4A
miR-6732-5p (and other miRNAs w/seed AGGGGGU)
LHX1
RSF1
miR-6793-5p (miRNAs w/seed GUGGGUU)
miR-4768-3p (and other miRNAs w/seed CAGGAGA)
miR-6892-3p (and other miRNAs w/seed CCCUCUC)
E. coli lipopolysaccharide
miR-6748-5p (and other miRNAs w/seed GUGGGUG)
DLL4
advanced glycation end-products
miR-8071 (and other miRNAs w/seed GGUGGAC)
miR-664-1-5p (and other miRNAs w/seed UGGCUGG)
miR-6818-5p (miRNAs w/seed UGUGUGA)
miR-151-5p (and other miRNAs w/seed CGAGGAG)
TET1
miR-6134 (miRNAs w/seed GAGGUGG)
cis-urocanic acid
SATB1
miR-6852-5p (and other miRNAs w/seed CCUGGGG)
miR-6751-5p (and other miRNAs w/seed UGGGGGU)
Rar
miR-6881-5p (miRNAs w/seed GGGGUAA)
miR-6737-5p (and other miRNAs w/seed UGGGGUG)
miR-6515-5p (miRNAs w/seed UGGAGGG)
miR-7112-5p (miRNAs w/seed CGGGCAG)
FGF8
miR-1275 (and other miRNAs w/seed UGGGGGA)
miR-122b-3p (and other miRNAs w/seed AACACCA)
miR-4763-5p (miRNAs w/seed GCCUGCC)
miR-5627-5p (and other miRNAs w/seed GAGGGUG)
miR-132-5p (miRNAs w/seed CCGUGGC)
miR-1289 (miRNAs w/seed GGAGUCC)
miR-2117 (miRNAs w/seed GUUCUCU)
MIA-459
MIA-313
miR-7704 (miRNAs w/seed GGGGUCG)
miR-550a-5p (and other miRNAs w/seed GUGCCUG)
miR-7976 (miRNAs w/seed GCCCUGA)
miR-1260a (and other miRNAs w/seed UCCCACC)
EWSR1-FLI1

**2404 common elements in "Caco-2_SARS-CoV-2" and "Caco-2_SARS-Co**

PDGF BB
TNF
PD98059
U0126
beta-estradiol
ERK
IRAK4
HGF
dexamethasone
LY294002
EGF
NR3C1
NFkB (complex)
IL1B
RELA
GPER1
triamcinolone acetonide
Mek
F2
forskolin
4-hydroxytamoxifen
IGF1
E. coli B5 lipopolysaccharide
paclitaxel
trovafloxacin
HRAS
TP53
GnRH-A
IL1A
cycloheximide
RAF1
lysophosphatidic acid
JUN
SP600125
NRG1
CREB1
IL2
actinomycin D
tetradecanoylphorbol acetate
AGT
TP63
Raf
lipopolysaccharide
TGFB1
OSM
poly rI:rC-RNA
hydrogen peroxide
FOS
thapsigargin
mifepristone
IL6
IGF2
ERBB2
FADD
IFNG
ERK1/2
methylprednisolone
MYC
curcumin
Ca2+
CREM
PRKCD
Growth hormone
FGF2
MAP3K1
N-acetyl-L-cysteine
anisomycin
tretinoin
IKBKB
deferoxamine
NFKB1
FOXO3
bicuculline
MAPK8
TGFA
SMARCA4
Tgf beta
estrogen receptor
progesterone
FSH
dalfampridine
Jnk
Vegf
Pkc(s)
tunicamycin
L-triiodothyronine
prostaglandin E2
epigallocatechin-gallate
P38 MAPK
salmonella minnesota R595 lipopolysaccharides
INSR
CEBPB
ESR1
NFKBIA
kainic acid
EGFR
INS
SMAD4
Pdgf (complex)
Sb202190
PI3K (complex)
gentamicin
cisplatin
resveratrol
bucladesine
ATF3
D-glucose
ADRB
S100A8
CG
2-amino-5-phosphonovaleric acid
MYD88
romidepsin
NDRG1
NGF
PDX1
arsenite
RAC1
doxorubicin
EGR1
F7
SP1
CREBBP
Alpha catenin
estrogen
ZFP36
FOXL2
nitric oxide
PRKCA
SRC
ethanol
CXCL12
Mt1
MAPK9
calphostin C
calcitriol
trichostatin A

| | | |
|---|---|---|
| EBI3 | miR-4308 (and other miRNAs w/seed CCCUGGA) | CDK19 |
| DNAJB6 | miR-1911-3p (miRNAs w/seed ACCAGGC) | EIF2AK2 |
| SMURF2 | miR-1233-5p (and other miRNAs w/seed GUGGGAG) | CHUK |
| 3-aminobenzamide | ETV4 | simvastatin |
| 7-nitroindazole | mir-25 | Ins1 |
| zearalenone | miR-1291 (and other miRNAs w/seed GGCCCUG) | SP3 |
| GNAS | GRHL2 | TLR7 |
| IFNA1/IFNA13 | ETV1 | Ap1 |
| miR-4765 (miRNAs w/seed GAGUGAU) | miR-3202 (miRNAs w/seed GGAAGGG) | STAT3 |
| heme | miR-6774-3p (miRNAs w/seed CGUGUCC) | IgG |
| glycine | fish oils | LDL |
| mannitol | MGAT3 | FAS |
| NMDA Receptor | zeranol | TNFSF11 |
| DEF6 | miR-4455 (miRNAs w/seed GGGUGUG) | arachidonic acid |
| HOTAIRM1 | miR-6918-5p (and other miRNAs w/seed GCUGAGG) | EP300 |
| MDM4 | miR-5010-3p (and other miRNAs w/seed UUUGUGU) | glutamine |
| CXCL3 | miR-1231-3p (miRNAs w/seed GCCCUGU) | carrageenan |
| quinpirole | miR-1285-3p (and other miRNAs w/seed CUGGGCA) | SMAD3 |
| methylphenidate | zinc | PRKCE |
| carfilzomib | miR-3064-3p (miRNAs w/seed UGCCACA) | MAPK1 |
| PTPN2 | miR-7851-3p (miRNAs w/seed ACCUGGG) | APOE |
| DBP | miR-6856-3p (miRNAs w/seed ACAGCCC) | EIF2AK3 |
| vanadate | miR-3175 (miRNAs w/seed GGGGAGA) | TERT |
| miR-4800-5p (miRNAs w/seed GUGGACC) | miR-6777-5p (and other miRNAs w/seed CGGGGAG) | MAP2K1/2 |
| miR-6731-3p (miRNAs w/seed CUAUUCC) | miR-6788-3p (miRNAs w/seed UCGCCAC) | 15-deoxy-delta-12,14 -PGJ 2 |
| miR-193a-5p (miRNAs w/seed GGGUCUU) | miR-3173-5p (and other miRNAs w/seed GCCCUGC) | MMP1 |
| miR-148b-5p (and other miRNAs w/seed AGUUCUG) | N-carbobenzyloxy-leucine-leucine-norvalinal | DETA-NONOate |
| 3M-002 | PDGFD | EPAS1 |
| USF2 | SF3B2 | leukotriene D4 |
| puromycin | PHC2 | CDK9 |
| MPZ | rutin | HMGA1 |
| lapatinib/pazopanib | CD38 | SU6656 |
| PIM2 | miR-96-5p (and other miRNAs w/seed UUGGCAC) | Z-LLL-CHO |
| PTK2B | miR-4433a-5p (miRNAs w/seed GUCCCAC) | REL |
| NAB2 | SMO | CD40LG |
| HNRNPA1 | miR-541-3p (and other miRNAs w/seed GGUGGGC) | dihydrotestosterone |
| NCD-38 | miR-5196-3p (miRNAs w/seed CAUCCUC) | PP2/AG1879 tyrosine kinase inhibitor |
| (1S,2R)-NCL-1 | mir-153 | CSF2 |
| G-Actin | GATAD2B | MAP2K4 |
| tandospirone | BAI | SB203580 |
| N-arachidonylglycine | tetrahydropalmatine | AKT1 |
| MACROD1 | miR-3109-3p (and other miRNAs w/seed AGGGCCA) | SOX2 |
| FANK1 | miR-4646-3p (miRNAs w/seed UUGUCCC) | EDN1 |
| dodecylbenzenesulfonic acid | AKT2 | NEDD9 |
| TIMELESS | miR-3160-3p (miRNAs w/seed GAGCUGA) | ESR2 |
| RAB22A | miR-518c-5p (miRNAs w/seed CUCUGGA) | wortmannin |
| BORCS8-MEF2B | miR-11181-5p (and other miRNAs w/seed UCUGACC) | COL18A1 |
| EPSTI1 | IL7R | thioacetamide |
| RNF7 | miR-2861 (and other miRNAs w/seed GGGCCUG) | Insulin |
| NKX2-8 | BACH1 | RASSF1 |
| RSL1D1 | NREP | GNAQ |
| PRDX6 | CGB3 (includes others) | EPHB1 |
| renin-angiotensin-aldosterone system blocking agent | hydrogen sulfide | BCL3 |
| alpha-cyano-4-hydroxycinnamate | KT5823 | C5 |
| remikiren | MYOC | KRAS |
| triazolam | miR-296-5p (miRNAs w/seed GGGCCCC) | sphingosine-1-phosphate |
| vitamin K1 | PLAG1 | Hdac |
| sodium 2-((6Z,15Z)-20-hydroxyicosa-6,15-dienamido)acetate | miR-4640-3p (miRNAs w/seed ACCCCCU) | PLAUR |
| chymostatin | miR-3914 (miRNAs w/seed AGGAACC) | AGTR1 |
| NCB-0846 | miR-6747-3p (and other miRNAs w/seed CCUGCCU) | S100A9 |
| TEI-F00806 | miR-4656 (and other miRNAs w/seed GGGCUGA) | raloxifene |
| 2-methoxy-5-(2',3',4'-trimethoxyphenyl)-2,4,6-cycloheptatrien-1-one | miR-4633-3p (and other miRNAs w/seed GGAGCUA) | Mt2 |
| CUX1 | miR-343 (and other miRNAs w/seed CUCCCUC) | cyclic AMP |
| miR-223-3p (miRNAs w/seed GUCAGUU) | miR-6882-3p (miRNAs w/seed GCUGCCU) | HDAC3 |
| miR-520d-5p (and other miRNAs w/seed UACAAAG) | mir-155 | HIF1A |
| CD 437 | mir-29 | Ngf |
| trametinib | miR-4278 (miRNAs w/seed UAGGGGG) | carbon tetrachloride |
| SPZ1 | FOXA2 | F3 |
| ACKR1 | miR-7973 (miRNAs w/seed GUGACCC) | troglitazone |
| CYP2J2 | miR-18a-3p (and other miRNAs w/seed CUGCCCU) | RNA polymerase II |
| IRF3 | miR-4525 (and other miRNAs w/seed GGGGGAU) | trans-hydroxytamoxifen |
| dimethyl sulfoxide | DSCAML1 | THPO |
| miR-8064 (miRNAs w/seed GCACACU) | miR-6835-5p (and other miRNAs w/seed GGGGGUA) | F2R |
| MAP3K5 | miR-4268 (miRNAs w/seed GCUCCUC) | ITGB1 |
| GADD45A | miR-665 (and other miRNAs w/seed CCAGGAG) | vorinostat |
| selenomethylselenocysteine | miR-450b-3p (and other miRNAs w/seed UGGGAUC) | palmitic acid |
| miR-12185-3p (and other miRNAs w/seed CAGCCAC) | KDM8 | HDAC1 |
| DKK1 | IL7 | EPCAM |
| miR-4671-5p (miRNAs w/seed CCGAAGA) | TNFRSF10B | HDAC6 |
| Pde4 | Hmgb1 | TICAM1 |
| TF | p85 (pik3r) | triptolide |
| EYA1 | nimesulide | 5-fluorouracil |
| TNFAIP6 | miR-4764-5p (miRNAs w/seed GGAUGUG) | herbimycin |
| DYRK1A | miR-3127-3p (and other miRNAs w/seed CCCCUUC) | camptothecin |
| GDNF | miR-7064-3p (and other miRNAs w/seed AGGGCCC) | 5-N-ethylcarboxamido adenosine |
| MAP2K3 | miR-6791-3p (and other miRNAs w/seed GCCUCCU) | STAT5A |
| ADORA2A | Hif1 | TP73 |
| HIPK2 | miR-6866-5p (miRNAs w/seed UAGAGGC) | PAF1 |
| ADCY | miR-638 (miRNAs w/seed GGGAUCG) | MAPK14 |
| KISS1 | miR-661 (miRNAs w/seed GCCUGGG) | NCOA1 |
| UCN | miR-615-5p (and other miRNAs w/seed GGGGUCC) | LEP |
| SAFB2 | miR-6874-5p (and other miRNAs w/seed UGGAGCU) | methylnitronitrosoguanidine |
| VIM | miR-4489 (miRNAs w/seed GGGGCUA) | FOSL1 |
| ACE | miR-5691 (and other miRNAs w/seed UGCUCUG) | cocaine |
| PIK3CA | miR-342-5p (and other miRNAs w/seed GGGGUGC) | Tnf (family) |
| HSF1 | antimycin A | MAP2K1 |
| miR-4752 (miRNAs w/seed UGUGGAU) | pimozide | SERCA |
| ritonavir | magnolol | 6,7-dinitroquinoxaline-2,3-dione |
| miR-4523 (miRNAs w/seed ACCGAGA) | ADAMTS18 | 6-cyano-7-nitroquinoxaline-2,3-dione |
| leukotriene B4 | ZNF281 | PTEN |
| calpain | miR-634 (miRNAs w/seed ACCAGCA) | KLF6 |
| GHRH | 2-methoxyestradiol | rottlerin |
| UBE3A | miR-4649-3p (miRNAs w/seed CUGAGGC) | fenretinide |
| miR-3186-3p (and other miRNAs w/seed CACGCGG) | miR-486-3p (and other miRNAs w/seed GGGGCAG) | Gsk3 |
| corticosterone | miR-6759-5p (miRNAs w/seed UGUGGGU) | APP |
| CNR1 | Rxr | PGR |
| CXCL10 | miR-138-5p (miRNAs w/seed GCUGGUG) | cytokine |
| MTA3 | miR-3177-3p (miRNAs w/seed GCACGGC) | NRAS |
| SUPT16H | miR-6846-5p (and other miRNAs w/seed GGGGGCU) | crocidolite asbestos |
| arotinoid acid | miR-137-3p (miRNAs w/seed UAUUGCU) | DUSP1 |
| cyanocobalamin | RAC2 | PPARG |
| RBL1 | PD 168393 | platelet activating factor |
| UCHL1 | Focal adhesion kinase | H1-2 |
| miR-524-3p (and other miRNAs w/seed AAGGCGC) | NUP98-HOXA9 | collagenase |
| miR-532-5p (and other miRNAs w/seed AUGCCUU) | miR-4707-3p (miRNAs w/seed GCCCGCC) | etoposide |
| DYSF | miR-6747-5p (miRNAs w/seed GGGGUGU) | DDIT3 |
| picrotoxin | miR-3594-5p (and other miRNAs w/seed CCAGGGC) | mycophenolic acid |
| UTP | SLC29A1 | Fgf |
| NR1H3 | ITGA6 | Cdk |
| RETNLB | lycopene | atorvastatin |
| miR-4677-5p (miRNAs w/seed UGUUCUU) | Esrra | amphetamine |
| miR-3926 (miRNAs w/seed GGCCAAA) | miR-92b-5p (miRNAs w/seed GGGACGG) | TLR3 |
| miR-4301 (miRNAs w/seed CCCACUA) | PRC2 | MAP2K7 |
| USP7 | miR-345-5p (and other miRNAs w/seed CUGACCC) | luteolin |
| ADA | n-nitrosomethylbenzylamine | NFYA |
| progestin | YBX1 | MAPK3 |
| miR-3692-3p (miRNAs w/seed UUCCACA) | hymecromone | tamoxifen |
| C3 | selenium | AHR |
| FLT1 | miR-133a-5p (miRNAs w/seed GCUGGUA) | cyclosporin A |
| cysteinyl-leukotriene | miR-6081 (miRNAs w/seed GGAGCAG) | Akt |
| L-leucine | maneb | FGF1 |
| isopropyl thiogalactoside | miR-363-5p (and other miRNAs w/seed GGGUGGA) | IL17A |
| ginsenoside Rh2 | miR-3150b-3p (and other miRNAs w/seed GAGGAGA) | NGFR |
| Cop9 Signalosome | PPARGC1A | PRKCB |
| CERT1 | miR-744-5p (and other miRNAs w/seed GCGGGGC) | Salmonella enterica serotype abortus equi lipopolysaccharide |
| chartreusin | dacinostat | IL4 |
| Pak | dactolisib | indirubin |
| Cofilin | HOXD3 | LIF |
| PLA2 | Usp17la (includes others) | R5020 |
| 2,2',4,4'-tetrabromodiphenyl ether | S-(2,3-bispalmitoyloxypropyl)-cysteine-GDPKHPKSF | Creb |
| hexadecanedioic acid | biochanin A | TCF |
| diosmetin | leptomycin B | ELK1 |

DF 203
NPS
nitrosomethylurethane
canthaxanthin
Rps15a-ps4
USP42
RBMS3
FAM168A
ADRM1
REM2
OSI-027
TRIM71
GPR4
BCAS3
USP36
BATF2
VENTX
MLXIP
FKRP
MRGPRX1
ETS-ELK1
Gm-Csf Receptor
MAP3K21
RYR3
INTERLEUKIN
EIF4H
RNMT
MCF2L
MAPK8IP2
CSDE1
ATP1A1
MMP10
PTPRF
HLTF
DAPK3
ZNF224
RAC3
PTPA
AMD1
LAMA1
STAM
mir-605
mir-105
CENPX
RPL15
CCNDBP1
NASP
ARHGEF28
FGB
GAD1
VANGL1
NEK4
FBXO4
BCLAF1
TIAM2
RPS6KA2
Hnrpfl
POU4F3
GABRG2
ILKAP
ING2
WASHC1
TH
RPA2
silvestrol
RPS25
DPH1
GRAP
alirocumab
FRAT1
TRIP11
PPP2R5A
RAMAC
TMPRSS4
CRTAP
Bhlhe41
RNF11
STYX
RPS4Y1
ZFYVE16
verticillin A
LY3039478
2,2-(2-chlorophenyl-4'-chlorophenyl)-1,1-dichloroethene
1-(3-chlorophenyl)piperazine
NPC 15437
amsacrine
phenoxyacetic acid
palytoxin
alpha-endosulfan
phosphorylethanolamine
primaquine
sarpogrelate
methiothepin
sibutramine
imidapril
neratinib
butyrine
chlordiazepoxide
GYKI 52466
dibenzo(a,l)pyrene
chalcone-24
(S)-2-((6Z,15Z)-20- hydroxyicosa-6,15-dienamido)succinate
benzo(a)pyrene-7,8-dione
butylbenzyl phthalate
nisoldipine
AGI-1095
sodium fluoride
procaterol
aprotinin
enalaprilat
ropinirole
indolo(3,2-b)carbazole
tegaserod
kenpaullone
veratridine
teleocidins
tyrphostin B56
5-oxo-6-8-11-14-(e,z,z,z)-eicosatetraenoic acid
cyclosporin H
5-thio-D-glucose
3-O-methylglucose
tibolone
flunisolide
miR-493-5p (miRNAs w/seed UGUACAU)
MEL S3
succinic acid
miR-6794-3p (miRNAs w/seed UCACUCU)
miR-6516-3p (miRNAs w/seed UCAUGUA)
miR-510-3p (miRNAs w/seed UUGAAAC)
ALKBH5
AICAR
CRTC2
GNAI2
oltipraz
trabectedin
miR-6789-5p (miRNAs w/seed UAGGGGC)
miR-3908 (miRNAs w/seed AGCAAUG)
miR-3691-3p (miRNAs w/seed CCAAGUC)
miR-6748-3p (miRNAs w/seed CCUGUCC)
miR-5571-5p (miRNAs w/seed AAUUCUC)
FEV
NR2E1
finasteride
miR-542-5p (miRNAs w/seed CGGGGAU)
miR-302c-3p (and other miRNAs w/seed AGUGCUU)
miR-6511b-5p (and other miRNAs w/seed UGCAGGC)
pycnogenols
OLIG1
YWHAE
CASP8AP2
ditiocarb
4-amino-6-hydrazino-7-beta-D-ribofuranosyl-7H-pyrrolo[2,3-d]-pyrimidine-5-carboxamide
glutathione ethyl ester
IL10RB
mir-503
SPRY4-IT1
HOXB3
RYBP
SH2B1
NFKBIE
ATF5
levamisole
cortistatin A
retinol acetate
gossypol
tridecanoic acid
miR-3070-5p (and other miRNAs w/seed GCCCCUG)
EGLN
miR-6858-3p (miRNAs w/seed AGCCAGC)
ursodeoxycholic acid
NEUROG1
miR-1294 (and other miRNAs w/seed GUGAGGU)
miR-139-3p (miRNAs w/seed GGAGACG)
miR-3144-5p (miRNAs w/seed GGGGACC)
miR-10396b-5p (and other miRNAs w/seed GGCGGGG)
NS-398
miR-589-3p (and other miRNAs w/seed CAGAACA)
PPP1R15A
mangiferin
miR-7154-3p (miRNAs w/seed GGAGGAC)
miR-1537-5p (miRNAs w/seed GCUGUAA)
infliximab
miR-4437 (miRNAs w/seed GGGCUCA)
CST5
miR-7113-3p (miRNAs w/seed CUCCCUG)
miR-6795-3p (miRNAs w/seed CCCCUCG)
SOD2
miR-1909-5p (miRNAs w/seed GAGUGCC)
miR-3186-5p (miRNAs w/seed AGGCGUC)
miR-1249-3p (and other miRNAs w/seed CGCCCUU)
miR-221-5p (and other miRNAs w/seed CCUGGCA)
miR-3680-3p (miRNAs w/seed UUUGCAU)
miR-4440 (miRNAs w/seed GUCGUGG)
miR-4258 (miRNAs w/seed CCCGCCA)
miR-3104-5p (and other miRNAs w/seed AGGGGGC)
miR-6080 (miRNAs w/seed CUAGUGC)
miR-939-3p (miRNAs w/seed CCUGGGC)
miR-6801-3p (and other miRNAs w/seed CCCCUGC)
FOXA1
miR-7155-5p (miRNAs w/seed CUGGGGU)
miR-3918 (miRNAs w/seed CAGGGCC)
miR-6836-3p (miRNAs w/seed UGCCUCC)
prostaglandin A1
MFSD2A
IgG1
L-serine
echinomycin
C9orf72
YOD1
MCL1
SLC9A1
H3C14
MAP3K11
sevoflurane
ferric ammonium citrate
SS18-SSX2
delphinidin
hesperetin
theophylline
ziritaxestat
miR-3191-3p (miRNAs w/seed GGGGACG)
miR-6515-3p (miRNAs w/seed CUCUUCA)
asoprisnil
miR-2110 (and other miRNAs w/seed UGGGGAA)
NR4A2
miR-5681a (and other miRNAs w/seed GAAAGGG)
FANCC
COLQ
miR-4642 (miRNAs w/seed UGGCAUC)
PNPLA2
SMPD1
miR-6724-5p (and other miRNAs w/seed UGGGCCC)
doxycycline
miR-663b (miRNAs w/seed GUGGCCC)
miR-516b-5p (miRNAs w/seed UCUGGAG)
alvespimycin
Cbp/p300
mir-26
cannabidiol
miR-4675 (and other miRNAs w/seed GGGCUGU)
miR-767 (and other miRNAs w/seed GCACCAU)
miR-3102-3p (and other miRNAs w/seed AGCACCC)
miR-548au-3p (miRNAs w/seed GGCAGUU)
CUL3
miR-4746-5p (miRNAs w/seed CGGUCCC)
miR-4292 (and other miRNAs w/seed CCCUGGG)
miR-3473h-5p (and other miRNAs w/seed AGGGGCU)
miR-6803-3p (miRNAs w/seed CCCUCGC)
lipoteichoic acid
miR-574-5p (miRNAs w/seed GAGUGUG)
miR-4537 (miRNAs w/seed GAGCCGA)
miR-6734-3p (miRNAs w/seed CCUUCCC)
dovitinib
UCN2
STAT1/3/5 dimer
NELFB
USP4
EFNB1
FOXD3
KRIT1
BAG3
quinacrine
midostaurin
8-oxo-7-hydrodeoxyguanosine
miR-610 (miRNAs w/seed GAGCUAA)
miR-6781-5p (miRNAs w/seed GGGCCGG)
miR-4512 (miRNAs w/seed AGGGCCU)
miR-1237-5p (and other miRNAs w/seed GGGGGCG)
HOXA13
vanillin
TEAD1
miR-1976 (and other miRNAs w/seed CUCCUGC)
miR-3191-5p (miRNAs w/seed UCUCUGG)
miR-122-5p (miRNAs w/seed GGAGUGU)
2,4,5,2',4',5'-hexachlorobiphenyl
USP28
Carlr
firtecan pegol
anatabine
RGD1560225
CDC20
WTAP
lansoprazole
galanthamine
CD40
xanthohumol
ARRB2
MST1R
SIN3A
FOXO1
ATF2
erlotinib
okadaic acid
RBCK1
aplidine
CSF3
CP-55940
GNRH
RAS
genistein
CTNNB1
AR
MET
tributyrin
25-hydroxycholesterol
resiquimod
DACH1
carbamylcholine
WT1
Ras homolog
EDN3
IL1
quercetin
propranolol
H-7
TNFSF10
HNF1A
sphingosylphosphocholine
MEMO1
RTKN
TAZ
PLAU
DICER1
salinosporamide A
RPS6KA5
ionomycin
decitabine
TLR9
Laminin (complex)
D-galactosamine
hydrocortisone
VEGFA
RET
BRD7
evodiamine
lysophosphatidylcholine
ETS2
MEF2D
silibinin
caffeic acid phenethyl ester
KLHL21
GPC3
STRAP
2-amino-3-phosphonopropionic acid
acetic acid
phenylephrine
NR5A2
zerumbone
butyric acid
pirinixic acid
MAP3K8
JAK2
Y 27632
CSF1
dibutyl phthalate
tosylphenylalanyl chloromethyl ketone
hyaluronic acid
ADRA1B
ATP
calcimycin
CCL5
NFATC2
aldosterone
ST3-Hel2A-2
(+)-MK-801
TAF4B
BAPTA-AM
GH1
FN1
NOTCH1
BDNF
USF1
oleic acid
ANGPT2
Ige
Immunoglobulin
ZBTB17
PLK1
ADRA1A
9,10-dimethyl-1,2-benzanthracene
staurosporine
TCF4
CIC
ITGA3
fulvestrant
5-O-mycolyl-beta-araf-(1->2)-5-O-mycolyl-alpha-araf-(1->1')-glycerol
ETS1
selumetinib
PIK3R1
reactive oxygen species
NFKBIB
tanespimycin
carbonyl cyanide m-chlorophenyl hydrazone
FGF19
CSF2RA
TLE1
RPS6KA4
PPP5C
LLGL2
ADIPOQ
MIB2
EIF3I
GCLC
ACP1
atipamezole
di(2-ethylhexyl) phthalate
gefitinib
IL33
HNF4A
RHOA
MYB
MEF2C
PRKCZ
AG490
acetaminophen
VDR
SPP1
NUPR1
glucocorticoid
HLX
TAB1
cadmium chloride
PTGS2
CD36
IKBKG
mitomycin C

FECH
UPF2
DRD2
Hmgn3
clodronic acid
4-tert-octylphenol
butylated hydroxyanisol
miR-3146 (miRNAs w/seed AUGCUAG)
miR-544-3p (and other miRNAs w/seed UUCUGCA)
miR-582-5p (miRNAs w/seed UACAGUU)
LCN2
miR-552-5p (miRNAs w/seed UUUAACC)
deferasirox
TNFSF14
TNFSF15
ZFHX3
hemin
miR-4700-3p (miRNAs w/seed ACAGGAC)
oleanolic acid
RAB1B
ABCB4
SKP2
mir-126
MEIS1
miR-4768-5p (and other miRNAs w/seed UUCUCUC)
miR-6740-3p (miRNAs w/seed GUCUUCU)
miR-3194-3p (miRNAs w/seed GCUCUGC)
miR-490-5p (miRNAs w/seed CAUGGAU)
GW9662
GSTP1
TERC
IL36A
methylnitrosourea
miR-509-5p (and other miRNAs w/seed ACUCCAG)
miR-6757-3p (miRNAs w/seed ACACUGG)
Rp-8-Br-cGMPS
sangivamycin
thioguanine
glycodeoxycholic acid
pregnenolone sulfate
IGF receptor
1-palmitoyl-2-arachidonoyl-phosphatidylcholine
AMOT
ANTXR2
benzo(a)pyrene 7,8-dihydrodiol
14,15-DHET
DUB
myosin-light-chain kinase
FKHR
lipoxygenase
PLC gamma
Stat1-Stat3
1,2,5,6-dibenzanthracene
diosmin
Esr1-Estrogen-Sp1
cetylpyridinium
Corticosteroid-GCR
prochloraz
NSD3
Jun-ATF2
LZTS2
RFFL
NOP58
CHD5
SPRED2
H1-3
ZNF451
PRR7
CNOT1
GPC6
NDUFAF3
SENP8
MYLIP
PSMD14
Ribosomal Subunit 80S
PVT1
NAA30
HDGF
AMOTL1
ASH1L
PTPN23
OLA1
UBE2D3
ATP1A2
defibrotide
Gap
SERTAD1
RRAD
MAT2B
PURB
YARS1
SPTBN1
AGAP2
U2AF2
BPTF
riociguat
mir-497
mir-383
MAPK15
STRN
SLC1A1
RPS14
PBK
PRDX4
apitolisib
KLF16
aurora kinase inhibitor III
RALGDS
NCOA5
SCN1B
DOK2
MGAT4A
SLC3A2
EIF2B1
STK16
MED26
sulfurbutylether-beta-cyclodextrin
PTTG1IP
DLST
SRPK2
ADCY6
GCNT1
RGS3
SIGLEC10
LSS
KPNB1
SLC12A6
KRT5
CTAG1A/CTAG1B
RASSF7
ELOA
CDK5R2
BTG1
STAM2
PES1
RBBP8
CNPY2
ARHGEF11
Miat
8-butyl-10-[(3-methoxy-5-pyrrol-2-ylidenepyrrol-2-ylidene)methyl]-11-azabicyclo[7.2.1]dodeca-1(12),9-diene
EEF1A1
Hspa1b
miR-4645-5p (and other miRNAs w/seed CCAGGCA)
miR-1225-3p (miRNAs w/seed GAGCCCC)
pyridaben
NKX2-3
RXRB
mir-22
SLC13A1
miR-5682 (miRNAs w/seed UAGCACC)
miR-6834-3p (miRNAs w/seed AUGUCCC)
miR-4649-5p (and other miRNAs w/seed GGGCGAG)
miR-4779 (miRNAs w/seed AGGAGGG)
ADAM17
miR-4446-3p (miRNAs w/seed AGGGCUG)
miR-4530 (miRNAs w/seed CCAGCAG)
Ppp2c
TRPV1
HIF3A
MED16
HBP1
MMP8
ALCAM
DUSP14
cantharidin
miR-3689d (and other miRNAs w/seed GGAGGUG)
miR-376c-3p (and other miRNAs w/seed ACAUAGA)
miR-3150a-3p (and other miRNAs w/seed UGGGGAG)
zymosan
isotretinoin
miR-124-3p (and other miRNAs w/seed AAGGCAC)
CLEC4G
miR-6808-5p (and other miRNAs w/seed AGGCAGG)
miR-6720-3p (miRNAs w/seed GCGCCUG)
miR-4276 (and other miRNAs w/seed UCAGUGA)
miR-3975 (miRNAs w/seed GAGGCUA)
KIT
miR-4270 (and other miRNAs w/seed CAGGGAG)
PHF1
AKT3
zoledronic acid
ZNF217
miR-344a-5p (and other miRNAs w/seed CAGGCUC)
RORC
miR-328-3p (and other miRNAs w/seed UGGCCCU)
miR-1915-3p (and other miRNAs w/seed CCCAGGG)
miR-6862-3p (and other miRNAs w/seed CUCACCC)
miR-24-3p (and other miRNAs w/seed GGCUCAG)
HOXA10
miR-6845-3p (and other miRNAs w/seed CUCUCCU)
5'-methylthioadenosine
CDKN2B-AS1
PLCE1
PTPRE
LAMC1
N-(3-oxododecanoyl)-homoserine lactone
auranofin
ASXL1
miR-4314 (and other miRNAs w/seed UCUGGGA)
miR-4323 (miRNAs w/seed AGCCCCA)
miR-887-3p (miRNAs w/seed UGAACGG)
lithocholic acid
CD300LF
BMP
VCP
miR-1228-3p (miRNAs w/seed CACACCU)
miR-593-5p (and other miRNAs w/seed GGCACCA)
NOTCH3
RNF2
miR-5588-3p (miRNAs w/seed AGUCCCA)
miR-6846-3p (miRNAs w/seed GACCCCU)
miR-540-3p (and other miRNAs w/seed GGUCAGA)
mir-150
miR-184 (and other miRNAs w/seed GGACGGA)
miR-4318 (miRNAs w/seed ACUGUGG)
SREBF2
miR-4721 (and other miRNAs w/seed GAGGGCU)
miR-128-3p (and other miRNAs w/seed CACAGUG)
miR-718 (miRNAs w/seed UUCCGCC)
Mst/krs
dienogest
(-)-gossypol
aurapten
RAR-RXR
Gata
Vla-4
TAF9
carvacrol
CSF
Mucin
Pik3r
IL31RA
TLN1
WNK4
CDH4
STK17B
atrasentan
NADPH
miR-3103-5p (and other miRNAs w/seed GAGGGAG)
TYROBP
BMPR2
TRIB3
JAK inhibitor I
delta-9-tetrahydrocannabinol
miR-550b-2-5p (and other miRNAs w/seed UGUGCCU)
miR-3150a-5p (and other miRNAs w/seed AACCUCG)
NSD1
mir-322
mir-196
CSK
VEGFC
BDKRB2
NRP1
PTGFR
dexmedetomidine
hydroxyflutamide
amlodipine
zinc protoporphyrin IX
NUP98-KDM5A
NUP98-NSD1
miR-4708-3p (miRNAs w/seed GCAAGGC)
miR-4777-3p (miRNAs w/seed UACCUCA)
CAMP
CSHL1
miR-4706 (and other miRNAs w/seed GCGGGGA)
miR-1668 (and other miRNAs w/seed AAAGGCC)
miR-216b-3p (and other miRNAs w/seed CACACUU)
miR-1224-3p (miRNAs w/seed CCCACCU)
miR-4266 (miRNAs w/seed UAGGAGG)
miR-337-3p (and other miRNAs w/seed CAGCUCC)
miR-4445-3p (and other miRNAs w/seed ACGGCAA)
miR-3960 (and other miRNAs w/seed GCGGCGG)
miR-128-1-5p (and other miRNAs w/seed GGGGCCG)
ASXL2
PTCH1
FLT3
PSEN2
miR-5192 (miRNAs w/seed GGAGAGU)
miR-1188-3p (and other miRNAs w/seed GAGGCUC)
miR-22-3p (miRNAs w/seed AGCUGCC)
miR-759 (miRNAs w/seed CAGAGUG)
miR-4428 (miRNAs w/seed AAGGAGA)
miR-1258-5p (and other miRNAs w/seed GCUGAGC)
miR-1234-3p (and other miRNAs w/seed CGGCCUG)
KLF4
topotecan
1'-acetoxychavicol acetate
TCF/LEF
prazosin
gamma-tocotrienol
PC-SPES
PLAT
fisetin
CXCR4
allopurinol
bee venom
LGALS3
mir-8
bortezomib
N-nitro-L-arginine methyl ester
formaldehyde
Pka
dopamine
bexarotene
vancomycin
nicotine
CAV1
BRD4
rotenone
methylselenic acid
CSF2RB
PLCG1
IL2RB
S1PR3
GFAP
exenatide
phorbol 12,13-dibutyrate
ADRA1
ZMIZ2
RAB7A
GCLM
MUC1
lomustine
CEBPA
TREM1
cigarette smoke
Interferon alpha
POLR2A
TBP
glutathione
pCPT-cAMP
Integrin
DDX17
silicon dioxide
Sos
PSEN1
TFAP4
CARM1
H89
Mapk
EZH2
JUNB
SNAI1
N(6)-(3-iodobenzyl)-5'-N-methylcarboxamidoadenosine
S100B
berberine
GLI3
ID1
sulindac sulfide
Pam3-Cys-Ser-Lys4
Histone h3
peptidoglycan
NCOA2
SPARC
ATM
PTHLH
2-deoxyglucose
medroxyprogesterone acetate
NORAD
TLR7/8
benzyl isothiocyanate
bisindolylmaleimide I
quisqualic acid
Calcineurin protein(s)
AGER
PI3K (family)
POU5F1
CSF1R
phenacetin
pentoxifylline
isobutylmethylxanthine
BMP2
ERBB3
NTRK2
BMP4
KL
pituitary adenylate cyclase-activating polypeptide
11,12-epoxyeicosatrienoic acid
TP53COR1
CSNK2A1
sulfasalazine
lapatinib
SRF
nitrofurantoin
methyl methanesulfonate
baicalein
MAPK7
PRL
STAT5B
SIRT1
Ro31-8220
ADRB3
AXIN1
SIN3B
epirubicin
GSK3B
MMP9
IFI16
PTPN11
SFTPA1
ciglitazone
androgen
5-azacytidine
PTH
N-acetylsphingosine
nicotinic acetylcholine receptor
AKIRIN2
thenoyltrifluoroacetone
Muscarinic cholinergic receptor
PARP1
mir-17
ETS
ACVR1C
L-buthionine (SR)-sulfoximine
LRP6
SPHK1
SB 216763
C11orf95-RELA
LIPE
MACROH2A1
MTPN
STAT1
PROC
ADRA1D
HNRNPK
quinolinic acid

Irs4
RPL23A
H1-1
TIGAR
RDH16
NOLC1
(+)-R-3-(4-chlorophenyl)-3-(1-hydroxymethylcyclopropylmethoxy)-2-(4-nitrobenzyl)-2,3-dihydroisoindol-1-one
BCKDHA
capivasertib
umbralisib
mir-297
singlet oxygen
compound 48/80
nordihydrocapsiate
nitrous oxide
mecamylamine
amantadine
prinaberel
p-chloroamphetamine
beta-lapachone
oxotremorine
albendazole
ethyl ether
cycloserine
oenothein B
devazepide
tamsulosin
modafinil
3-nitrobenzanthrone
salsalate
ethyl gallate
taprostene
MLN-4760
phloretin
TTI-101
mycophenolate mofetil
lanthanum chloride
sodium iodide
5,10,15,20-tetrakis(4-sulfonatophenyl)porphyrinato iron (III)
C66
3,4-methylenedioxyamphetamine
actinomycin
glutathione diethyl ester
GNE-317
R191
(indol-3-yl)pyruvic acid
muscarine
3,7-dimethyl-1-propargylxanthine
AVI-4126
mizoribine
D,L-propargylglycine
CH6953755
raffinose
prostratin
ponasterone A
Mn2+
miR-6809-3p (miRNAs w/seed UUCUCUU)
miR-6069 (miRNAs w/seed GGCUAGG)
PRDM16
GTF2B
mercuric chloride
CX3CR1
miR-1257 (miRNAs w/seed GUGAAUG)
SMAD2
miR-4755-5p (and other miRNAs w/seed UUCCCUU)
miR-4668-3p (miRNAs w/seed AAAAUCC)
CpG ODN 2006
miR-5581-3p (miRNAs w/seed UCCAUGC)
IGHM
IL9
S-adenosylmethionine
RBL2
NR0B2
miR-3152-3p (and other miRNAs w/seed GUGUUAG)
miR-1180-3p (miRNAs w/seed UUCCGGC)
miR-589-5p (and other miRNAs w/seed GAGAACC)
paricalcitol
INSIG1
CGA
EIF3E
LG100268
miR-3687 (and other miRNAs w/seed CCGGACA)
MMP3
let-7d-3p (miRNAs w/seed UAUACGA)
miR-4477a (miRNAs w/seed UAUUAAG)
miR-526a-5p (and other miRNAs w/seed UCUAGAG)
13(S)-hydroxyoctadecadienoic acid
adenine
L-alanine
ribose
inecalcitol
Osteocalcin
chondroitin sulfate B
darbepoetin alfa
SHP
tyrosine kinase
Stat dimer
1-methyl-2-pyrrolidinone
aluminum hydroxide
tylophorine
CINP
PDE8A
GPR65
DNAJB4
AMOTL2
GLIPR2
LINC00887
ENTPD2
GJB6
SLCO1C1
CIRBP
endothelin receptor
Slfn1
ORAI3
MUCL1
SLC39A4
VLDL
PDLIM1
Casein
Nc2
RAB7
bitertanol
N-docosahexaenoylethanolamine
PF-4691502
KCNJ2
TLCD3A
ADRA2B
PSME3
G0S2
ARID1B
mir-338
mir-138
mir-32
TOPORS
CALCR
LIMK1
TIAM1
SEPTIN9
CALM1 (includes others)
HUNK
GALR1
TCF20
miR-708-5p (and other miRNAs w/seed AGGAGCU)
miR-3140-5p (miRNAs w/seed CCUGAAU)
miR-6872-3p (miRNAs w/seed CCAUGCC)
miR-4445-5p (miRNAs w/seed GAUUGUU)
miR-4326 (miRNAs w/seed GUUCCUC)
miR-6810-5p (and other miRNAs w/seed UGGGGAC)
miR-644a (miRNAs w/seed GUGUGGC)
1,25-dihydroxyvitamin D
Wnt
epothilone B
C1q
COL1A1
GAS2L3
HIF1AN
UBD
TGIF1
LY96
IGFBP1
STMN1
Hif
mevastatin
indole
caffeic acid
miR-466d-5p (and other miRNAs w/seed GUGUGUG)
miR-6868-3p (miRNAs w/seed UCCUUCU)
mir-135
N(G)-monomethyl-D-arginine
miR-892b (miRNAs w/seed ACUGGCU)
miR-2114-5p (miRNAs w/seed AGUCCCU)
spironolactone
miR-6741-3p (miRNAs w/seed CGGCUCU)
miR-3116 (miRNAs w/seed GCCUGGA)
12-(3-adamantan-1-yl-ureido) dodecanoic acid
ARNTL
miR-504-3p (and other miRNAs w/seed GGGAGAG)
miR-4787-5p (and other miRNAs w/seed CGGGGGU)
miR-3151-3p (miRNAs w/seed CUGAUCC)
miR-3135a (miRNAs w/seed GCCUAGG)
HMGB1
miR-4733-3p (and other miRNAs w/seed CACCAGG)
let-7f-2-3p (and other miRNAs w/seed UAUACAG)
caspase
miR-339-5p (and other miRNAs w/seed CCCUGUC)
P110
PIWIL4
Ap2 alpha
BCL11A
SOX18
RYK
LRIG1
DNAJA3
EPHA4
MATK
SMARCD1
Hmgb2 (includes others)
BCL2A1
EGTA acetoxymethyl ester
ferric nitrilotriacetate
propyl gallate
NFYB
miR-6795-5p (and other miRNAs w/seed GGGGGGA)
miR-6783-5p (miRNAs w/seed AGGGGAA)
miR-4514 (and other miRNAs w/seed CAGGCAG)
miR-6878-3p (and other miRNAs w/seed UGGCCUC)
miR-6776-3p (miRNAs w/seed AACCACC)
miR-4524a-3p (miRNAs w/seed GAGACAG)
MAFB
lysophosphatidylinositol
doxifluridine
Angiotensin II receptor type 1
PHB2
FKBP4
MXI1
SMAD6
D609
miR-658 (miRNAs w/seed GCGGAGG)
miR-673-3p (and other miRNAs w/seed CCGGGGC)
miR-646 (miRNAs w/seed AGCAGCU)
ATP-gamma-S
miR-6809-5p (miRNAs w/seed GGCAAGG)
miR-1199-5p (and other miRNAs w/seed CUGAGCC)
miR-5699-5p (miRNAs w/seed GCCCCAA)
TET3
ITCH
miR-520g-5p (miRNAs w/seed CUAGAGG)
monocrotaline
miR-3529-3p (miRNAs w/seed ACAACAA)
miR-635 (and other miRNAs w/seed CUUGGGC)
miR-3126-5p (and other miRNAs w/seed GAGGGAC)
miR-6854-3p (miRNAs w/seed GCGUUUC)
KMT2D
miR-6758-5p (and other miRNAs w/seed AGAGAGG)
miR-4522 (and other miRNAs w/seed GACUCUG)
miR-5589-5p (miRNAs w/seed GCUGGGU)
miR-3136-3p (and other miRNAs w/seed GGCCCAA)
laquinimod
miR-508-3p (miRNAs w/seed GAUUGUA)
miR-4730 (miRNAs w/seed UGGCGGA)
VGLL3
PPP4R3B
hemoglobin
TNFRSF13C
PLCG2
AKR1B1
EREG
ellagic acid
latrunculin B
piperine
cytochalasin D
REST
DOCK8
prostaglandin J2
3M-011
UBE2I
miR-3584-5p (and other miRNAs w/seed GGAGGAG)
miR-711 (miRNAs w/seed GGACCCA)
miR-624-5p (miRNAs w/seed AGUACCA)
miR-879-5p (and other miRNAs w/seed GAGGCUU)
miR-515-5p (and other miRNAs w/seed UCUCCAA)
miR-519d-5p (miRNAs w/seed CUCCAAA)
miR-802-3p (and other miRNAs w/seed CGGAGAG)
miR-6871-5p (miRNAs w/seed AUGGGAG)
IFN type 1
miR-136-5p (miRNAs w/seed CUCCAUU)
miR-3684 (miRNAs w/seed UAGACCU)
miR-4297 (and other miRNAs w/seed GCCUUCC)
miR-3917 (miRNAs w/seed CUCGGAC)
phosphatidylinositol
QRFP
saracatinib
PTPRB
LHB
STRA6
TDO2
TPSD1
GABBR2
LPAR2
IL18RAP
PLA2G2A
benzoquinone
RWJ 67657
zimelidine
omeprazole
LDLR
apigenin
ADCYAP1
YY1
TFPI2
fluoxetine
ITGA5
2-(4-amino-1-isopropyl-1H-pyrazolo[3,4-d]pyrimidin-3-yl)-1H-indol-5-ol
NLRX1
CCN1
dexamethasone phosphate
Ciap
HOXA5
bisindolylmaleimide iv
Ni2+
IRS1
IL1RN
saikosaponin A
SNW1
deoxycholate
methotrexate
WNT1
FGFR2
AREG
ERBB4
nitroprusside
indomethacin
calcipotriene
NfkB-RelA
P2RY2
piceatannol
kaempferol
tert-butyl-hydroquinone
8-bromo-cAMP
tacrolimus
phorbol esters
BAG1
AZGP1
KN-62
amino acids
linolenic acid
methyl-beta-cyclodextrin
Rock
TRAF3
methapyrilene
SOCS3
Ck2
YBX3
ICAM1
JAG1
IRF8
MAC
CTSB
ECSIT
ozone
ceramide
rosiglitazone
miR-6873-3p (miRNAs w/seed UCUCUCU)
TAF4
SYVN1
lactacystin
FGFR1
NCOR2
HDAC2
sulforafan
TRADD
hydrochloric acid
olanzapine
GRP
PHB
IRAK3
fludarabine
DAB2IP
Igkv1-117
FUS
MAPKAPK2
fenofibrate
ELAVL1
PTGES
CDKN1A
POMC
CEBPD
PRKDC
FGFR4
dithiothreitol
bardoxolone methyl
TFAP2A
RBPJ
levodopa
APEX1
NF1
bufalin
E2F1
THRB
SNHG20
OIP5-AS1
PIAS2
CHRM3
DRD1
VLDLR
AFP
ELK3
clioquinol
clonidine
OSMR
IL6ST
sodium arsenite
STAT6
potassium chloride
IFNAR1
GPS2
napabucasin
Sn50 peptide
Stat3-Stat3
grape seed extract
ROR1
vitamin K3
lonaprisan
MSK1/2
FBXO25
RPRD1B
CCDC88C
CTNNβ-LEF1
OMG
RAMP3
inostamycin
(S)-flurbiprofen
GLI2
CCN2
PDGFB
Go 6976
MED1
VIP
CD24
BCL2
ITGAV
ALOX15
honokiol
TSH
methylmercury
triamterene

| | | |
|---|---|---|
| GIPC1 | eugenol | miR-155-5p (miRNAs w/seed UAAUGCU) |
| UBTF | miR-124 mimic | TNFRSF1A |
| SOS1 | geranylgeraniol | L-histidine |
| ABI1 | ZBED6 | RB1CC1 |
| Cyp2c70 | SELP | SR 144528 |
| CTNNAL1 | RXRG | pyrrolidine dithiocarbamate |
| CCKAR | LOC105372576 | puromycin aminonucleoside |
| ADRA2A | 4-(2-aminoethyl)benzenesulfonylfluoride | IL13 |
| WARS1 | miR-4421 (and other miRNAs w/seed CCUGUCU) | SHC1 |
| ARHGEF2 | miR-5113 (and other miRNAs w/seed CAGAGGA) | VCAN |
| FOXH1 | SASH1 | SOX17 |
| LETMD1 | Fgfr | ANXA2 |
| SERPINB7 | RHOJ | ARRB1 |
| AKAP13 | CBX7 | CDC42 |
| S3I-1757 | SULF2 | miR-200b-3p (and other miRNAs w/seed AAUACUG) |
| COL1A2 | IL31 | CpG oligonucleotide |
| CRLF1 | DAG1 | KCNE3 |
| PLAC8 | NUMB | Map3k7 |
| ERO1A | SMURF1 | 3,3'-diindolylmethane |
| HSPH1 | ITGB4 | diethylnitrosamine |
| ARF1 | CCNH | metribolone |
| TOP2A | GSK3A | PML |
| RPL5 | NRTN | TAL1 |
| TBPL1 | thioridazine | PRKAG3 |
| MAD1L1 | miR-134-3p (and other miRNAs w/seed UGUGGGC) | IKK (complex) |
| 3',4'-dimethoxyflavone | miR-2278 (miRNAs w/seed AGAGCAG) | SPDEF |
| LY5 | FSHR | Collagen type I (complex) |
| OSU-CG12 | TEAD3 | fenamic acid |
| benfur | CDX1 | testosterone |
| SRT2183 | 2,3,4,7,8-pentachlorodibenzofuran | EPO |
| 5,6,7,8-tetrahydrobiopterin | miR-483-5p (miRNAs w/seed AGACGGG) | TRAF6 |
| apicularen A | miR-4286 (miRNAs w/seed CCCCACU) | CDKN2A |
| acetoacetic acid | miR-6815-3p (miRNAs w/seed GGCUUCU) | L-glutamic acid |
| ketanserin | miR-190a-5p (and other miRNAs w/seed GAUAUGU) | CHRM1 |
| tipifarnib | miR-6511a-5p (and other miRNAs w/seed AGGCAGA) | ELK4 |
| KP-SD-1 | mir-21 | gambogic acid |
| bilobalide | miR-3937 (miRNAs w/seed CAGGCGG) | ACVRL1 |
| liarozole | RC3H1 | amiodarone |
| phenylhydrazine | sildenafil | PML-RARA |
| glabridin | miR-4718 (miRNAs w/seed GCUGUAC) | gemfibrozil |
| tramadol | miR-6776-5p (miRNAs w/seed CUGGGUG) | HTT |
| lomefloxacin | miR-6814-3p (and other miRNAs w/seed CUCGCAU) | pioglitazone |
| cromakalim | ESRRA | linoleic acid |
| nitrendipine | miR-4743-5p (miRNAs w/seed GGCCGGA) | GNA14 |
| salicylamide | miR-1281 (miRNAs w/seed CGCCUCC) | mir-34 |
| 4-methylcatechol | miR-6827-5p (miRNAs w/seed GGGAGCC) | lipoxin A4 |
| methylpiperidinyl-3b-hydroxycholenamide | miR-4704-3p (miRNAs w/seed CAGUCAC) | TRH |
| 1,1-diethyl-2-hydroxy-2-nitrosohydrazine | miR-1247-3p (miRNAs w/seed CCCGGGA) | XDH |
| MS645 | miR-556-5p (miRNAs w/seed AUGAGCU) | CAMK4 |
| WZB117 | PHLPP1 | GNB2 |
| Phe-Pro-Arg-chloromethyl ketone | mir-101 | CDKN1B |
| microcystin-LR | mir-218 | norepinephrine |
| bisindolylmaleimide II | MLX | miR-10394-3p (and other miRNAs w/seed GGGCGCG) |
| resiniferatoxin | RPA1 | TRAF3IP2 |
| galangin | HULC | tyrphostin AG 1478 |
| nor-binaltorphimine | ammonium chloride | tetrachlorodibenzodioxin |
| colcemid | EML4-ALK | dihydroartemisinin |
| palmatine | naloxone | PIAS4 |
| iodixanol | miR-551b-3p (and other miRNAs w/seed CGACCCA) | HOXA4 |
| L-asparagine | miR-139-5p (miRNAs w/seed CUACAGU) | MVP |
| N-butyldeoxynojirimycin | miR-3065-3p (miRNAs w/seed CAGCACC) | pentobarbital |
| Collagen type VI | miR-3649 (miRNAs w/seed GGGACCU) | noscapine |
| trehalose | rolipram | growth factor |
| cholesterol ester | miR-6877-5p (miRNAs w/seed GGGCCGA) | $Cd^{2+}$ |
| $Pb^{2+}$ | miR-182-5p (and other miRNAs w/seed UUGGCAA) | TWIST2 |
| miR-4317 (miRNAs w/seed CAUUGCC) | CALCA | ursolic acid |
| CD2 | miR-4710 (miRNAs w/seed GGUGAGG) | BCL6 |
| PEBP1 | miR-203a-3p (and other miRNAs w/seed UGAAAUG) | JQ1 |
| CCR5 | miR-6511a-3p (and other miRNAs w/seed CUCACCA) | GW3965 |
| miR-875-3p (miRNAs w/seed CUGGAAA) | miR-421-3p (and other miRNAs w/seed UCAACAG) | aspirin |
| RRP1B | miR-4535 (miRNAs w/seed UGGACCU) | NOD2 |
| PXR ligand-PXR-Retinoic acid-RXRα | miR-4747-5p (and other miRNAs w/seed GGGAAGG) | cadmium |
| DPP4 | miR-4686 (miRNAs w/seed AUCUGCU) | GNB1 |
| miR-454-5p (miRNAs w/seed CCCUAUC) | GSTK1 | NR1D1 |
| miR-1970 (and other miRNAs w/seed GUGUCAC) | HJV | MIF |
| miR-6076 (and other miRNAs w/seed GCAUGAC) | LGI1 | Il3 |
| miR-5135 (and other miRNAs w/seed GGUCUAG) | HS6ST1 | PF4 |
| SKIL | GRIP1 | TLR4 |
| ELANE | HEYL | miR-101-3p (and other miRNAs w/seed ACAGUAC) |
| ANXA1 | MAP3K10 | BRCA1 |
| ILK | citric acid | PRNP |
| SST | Bvht | cyclophosphamide |
| U73122 | TFAP2C | cerivastatin |
| miR-192-5p (and other miRNAs w/seed UGACCUA) | miR-760-3p (and other miRNAs w/seed GGCUCUG) | ENG |
| miR-411-5p (and other miRNAs w/seed AGUAGAC) | miR-370-5p (and other miRNAs w/seed AGGUCAC) | ABL1 |
| miR-503-3p (and other miRNAs w/seed GAGUAUU) | 3M-001 | histone deacetylase |
| miR-1291 (and other miRNAs w/seed UGGCUCU) | CUL4B | propofol |
| miR-4664-3p (miRNAs w/seed UUCCGGU) | miR-4650-5p (miRNAs w/seed CAGGCCU) | lonafarnib |
| miR-500a-5p (miRNAs w/seed AAUCCUU) | miR-4724-3p (and other miRNAs w/seed UACCUUC) | TIFA |
| miR-6124 (miRNAs w/seed GGAAAAG) | DNA-methyltransferase | 48s |
| miR-3686 (and other miRNAs w/seed UCUGUAA) | Lymphotoxin | SP2 |
| miR-3117-3p (miRNAs w/seed UAGGACU) | TNFRSF18 | GTF2I |
| sodium bisulfide | YWHAZ | FOXP3 |
| CRP | FBN1 | heparin |
| PRKACA | HDAC7 | GATA3 |
| Pam3-Cys | NTN1 | celecoxib |
| 1,2-dioctanoyl-sn-glycerol | SOCS6 | PP1 |
| L-aspartic acid | PPP4R3A | isoprenaline |
| p38 Sapk | SCGB1A1 | MAP3K7 |
| C9orf116 | miR-505-3p (miRNAs w/seed UCAACAC) | HMOX1 |
| Hbb | miR-4300 (and other miRNAs w/seed GGGAGCU) | Lh |
| ATPase | miR-196a-3p (miRNAs w/seed GGCAACA) | PD173074 |
| Erm | miR-338-3p (miRNAs w/seed CCAGCAU) | KDM3A |
| Ho | cholic acid | IRS2 |
| 1,2,3,4,6,7,8-heptachlorodibenzofuran | ATG7 | epoprostenol |
| olmesartan | miR-3612 (and other miRNAs w/seed GGAGGCA) | cyclopiazonic acid |
| DGKE | ACSL4 | 2-acetylaminofluorene |
| SMPD3 | CLEC11A | myricetin |
| DRD3 | miR-4713-3p (miRNAs w/seed GGGAUCC) | CXCL8 |
| MARCHF3 | miR-571 (miRNAs w/seed GAGUUGG) | MAP3K14 |
| BCAS2 | miR-675-5p (and other miRNAs w/seed GGUGCGG) | GAST |
| GPR17 | 3-methyladenine | dehydroisoandrosterone |
| SSBP2 | CERK | NIBAN2 |
| FOXK2 | SAA1 | SRGAP1 |
| Ftx | miR-7106-3p (miRNAs w/seed GCUCCCU) | ELL |
| PYHIN1 | miR-1934-5p (and other miRNAs w/seed CUGGUCC) | SGI 1776 |
| ZDHHC2 | miR-1287-5p (miRNAs w/seed GCUGGAU) | AHNAK |
| SNHG3 | miR-7152-5p (miRNAs w/seed UUCCUGU) | TOR2A |
| CDH16 | miR-3098-3p (and other miRNAs w/seed UCUGCUG) | TRAF7 |
| PCA3 | miR-6859-3p (miRNAs w/seed GACCCCC) | HMGN2 |
| RPE65 | ETV5 | CYP2C9 |
| harmol | miR-3913-3p (miRNAs w/seed GACAUCA) | ATAD2 |
| Cathepsin | miR-6769a-3p (miRNAs w/seed AGCCCCU) | (E)-1-(2-nitrovinyl)naphthalene |
| 7S NGF | Tnf receptor | (E)-9-(2-nitrovinyl)anthracene |
| Atf | ITGA2 | di-2-pyridylketone 4-cyclohexyl-4-methyl-3-thiosemicarbazone |
| GRAP2 | KIF3A | tarenflurbil |
| ZIC1 | CXCR3 | metformin |
| FAF1 | TERF2IP | CNTF |
| ERN2 | alendronic acid | TRAF2 |
| PUF60 | GLP-1-(7-34)-amide | TSC2 |
| TYMS | wogonin | ATF4 |
| NCK2 | miR-4436b-3p (and other miRNAs w/seed AGGGCAG) | NAMPT |
| mir-190 | ZBTB16 | leukotriene C4 |
| MIR4269 | miR-874-3p (miRNAs w/seed UGCCCUG) | PPP1R1B |
| SFRP2 | miR-6868-5p (miRNAs w/seed CUGGCAG) | CHD1 |
| MRAS | miR-1266-3p (miRNAs w/seed CCUGUUC) | THY1 |
| ADAR | miR-182-3p (miRNAs w/seed GGUUCUA) | CCAT2 |
| LSP1 | miR-1343-3p (and other miRNAs w/seed UCCUGGG) | NEDD4 |
| MAPKAPK5 | SAA | ethidium |
| UTS2 | bromocriptine | THRA |
| PENK | miR-1470 (miRNAs w/seed CCCUCCG) | HDAC4 |
| FLOT1 | miR-143-3p (and other miRNAs w/seed GAGAUGA) | cobalt chloride |

ARHGEF25
RAP1GDS1
PORCN
BRCA2
RPS3
MYCT1
CCL22
BCAR3
CEL
PRMT2
Tmsb4x (includes others)
ACOD1
CMA1
VCAM1
CDC27
GNL2
CDH13
Nrg1
KRT10
Foxe3
Mcpt1
H1f4
ILF2
LGH447
CC-122
MI-773
U 50488H
gabapentin
LY379196
silicon phthalocyanine
triiodothyronine, reverse
ranitidine
tranexamic acid
2,3,7,8-tetrachlorodibenzofuran
etidronic acid
roflumilast
paxilline
orantinib
opioid
H-8
4-aminophenol
sodium azide
FT671
dexfenfluramine
phytol
methyllycaconitine
5-hydroxydecanoic acid
acetyl-11-keto-beta-boswellic acid
Hg2+
miR-675-3p (miRNAs w/seed UGUAUGC)
miR-3977 (miRNAs w/seed UGCUUCA)
cholecalciferol
MEN1
miR-6817-3p (miRNAs w/seed CUCUCUG)
miR-140-5p (and other miRNAs w/seed AGUGGUU)
miR-7844-5p (miRNAs w/seed AAACUAG)
nilotinib
LTBR
miR-6902-3p (miRNAs w/seed CAUGUGA)
Ifn
Hsp70
miR-6529-3p (and other miRNAs w/seed CUGUGCC)
miR-891a-3p (miRNAs w/seed GUGGCAC)
20-hydroxyeicosatetraenoic acid
adenosine dialdehyde
8-hydroxyguanine
nitrite
L-glutamine
norethindrone acetate
Neurotrophin
Dynamin
F Actin
DPPA3
Beta Arrestin
EIF4A3
KCNK2
KLF17
PDZK1IP1
RGMB
CCN6
AQP11
taspoglutide
peptide T
RABGEF1
PXN
PRKACB
TFPI
CSRP3
OPRK1
RBBP4
CLDN1
entolimod
GNB5
mir-637
SNIP1
Irs3
Nisch
MAGED1
AATF
RNF111
TRPC3
GRM5
DYNLL1
SIX3
CFH
STK38
TDG
GPS1
ECT2
CTSK
PPID
BARD1
SERPIND1
MTUS1
SF1
SRGN
OMP-52M51
NET1
equol
CXXC4
COG112 peptide
gelam honey
emricasan
halothane
eprosartan
PK11007
juglone
benz[a]anthracene
ruboxistaurin
JWH-015
curcuminoids
EPZ005687
GSI34
Z-VEID-FMK
trigonelline
alpha-hydroxyglutarate
edelfosine
CV 3988
remifentanil
3',4',7-trihydroxyisoflavone
H2AB3 (includes others)
E2f

CpG ODN 1668
miR-544b (miRNAs w/seed CCUGAGG)
miR-8053 (miRNAs w/seed GGCGAUU)
CLDN7
miR-349 (and other miRNAs w/seed AGCCCUG)
miR-465d-3p (and other miRNAs w/seed GAUCAGG)
novobiocin
NRDE2
atrazine
PHF8
NEK7
EGLN2
SMAD1/5
SPTAN1
pictilisib
PRTN3
MC1R
1,1-bis(3'-indolyl)-1-(4-hydroxyphenyl)methane
niflumic acid
benazepril
tyrphostin AG 127
miR-1236-3p (miRNAs w/seed CUCUUCC)
miR-659-3p (miRNAs w/seed UUGGUUC)
miR-2277-3p (miRNAs w/seed GACAGCG)
miR-5582-5p (miRNAs w/seed AGGCACA)
miR-887-5p (miRNAs w/seed UUGGGAG)
miR-4790-5p (miRNAs w/seed UCGCUUU)
miR-551b-5p (miRNAs w/seed AAAUCAA)
miR-3152-5p (miRNAs w/seed UUGCCUC)
miR-6786-5p (miRNAs w/seed CGGUGGG)
miR-4759 (miRNAs w/seed AGGACUA)
miR-3093-3p (and other miRNAs w/seed GUGGACA)
miR-1204 (miRNAs w/seed CGUGGCC)
ALDH2
BMP15
CRHR2
FOXF2
EIF4EBP2
cafestol
miR-3195 (miRNAs w/seed GCGCCGG)
miR-4264 (miRNAs w/seed CUCAGUC)
miR-6721-5p (miRNAs w/seed GGGCAGG)
miR-670-5p (miRNAs w/seed UCCCUGA)
miR-1283 (miRNAs w/seed CUACAAA)
miR-548o-3p (and other miRNAs w/seed CAAAACU)
miR-10400-3p (and other miRNAs w/seed UGGGCUC)
miR-6735-3p (miRNAs w/seed GGCCUGU)
miR-1253 (miRNAs w/seed GAGAAGA)
miR-6742-3p (miRNAs w/seed CCUGGGU)
miR-23a-5p (and other miRNAs w/seed GGGUUCC)
PTGER2
N(6)-methyl-2'-deoxyadenosine
acyclovir
azide
thromboxane A2
flavokawain C
ABLIM2
cyanuric acid
hydroxamic acid
LTB4R/LTB4R2
Gαq
NDPK
beta-glucuronidase
Mlcp
glutathione peroxidase
MKK3/6
G-protein gamma
G-protein beta
3',5'-methoxyflavone
MAP2K4/7
NF279
sodium thiosulfate
SEC16B
Pad2
EMC2
WTIP
LOC299282
RGN
ABLIM3
MARCHF4
FCN1
procyanidin B2
Ctf2
TRIM17
SLC17A6
NBR2
ZNF580
USP50
NLRP9
RPS6KA
MPC1
CCDC47
ZG16B
EMC6
Gm13570/Gm4984
MARCHF9
METAP2
DHODH
nickel sulfide
rimantadine
tandutinib
Tpl2 kinase inhibitor
alisertib
MEF2D-NFAT2-p300
mapatumumab
TOMM22
DLAT
binimetinib
cucurbitacin E
voxtalisib
pidilizumab
EEFSEC
IRGM
MED25
EDN2
NAPA
KRT1
CHKA
PRL-3 inhibitor I
KSR2
CFI
miR-690 (miRNAs w/seed AAGGCUA)
Mir690
DYNLT3
SIGLEC7
SIGLEC9
TNPO2
TYMP
NXF3
LRRN1
aloperine
5-methoxytryptophan
venetoclax
TM7SF2
KIF11
LUC7L3
WBP1
TFF1
PTCRA
Pla2g2a
WDR83OS
RIT1

ZMPSTE24
bleomycin
SOD1
LYN
histamine
miR-4281 (miRNAs w/seed GGUCCCG)
HOTAIR
IFNLR1
BRAF
NR3C2
miR-4753-5p (miRNAs w/seed AAGGCCA)
GLIS2
CRBN
Endothelin
mir-154
S1PR2
RPS6KA3
acadesine
chenodeoxycholic acid
SBDS
IKBKE
TGFBR1
IL3
MTA1
K+
SOCS1
SP110
NCOA3
PKD1
TGFBR2
AVP
RARG
GATA4
prostaglandin E1
NOD1
CTBP2
KCNIP3
MNT
PDCD4
TREM2
ACTN4
metyrapone
PS-1145
peroxynitrite
IFIH1
2-bromoethylamine
IL11RA
TXN
CCL2
lovastatin
SQSTM1
BSG
RETN
elaidic acid
ERG
SAV1
STAP2
mir-486
S1PR1
farnesol
7-ketocholesterol
TEX11
DACT3
CDC25B
PHLDA1
SNRK
SLC9A3R2
carboxyamido-triazole
C25-140
yohimbine
KMT2A
Notch
miR-100-3p (miRNAs w/seed AAGCUUG)
DGCR8
FGF21
TNFAIP3
MAP2K5
TIMP3
valsartan
YAP1
Histone h4
PRKAA1
GFI1
daidzein
Aldose Reductase
Collagen type IV
SP100
GDF15
SMARCE1
A-Fos
PCDH11Y
PD 169316
PD 153035
IL15
RARA
NCOR1
26s Proteasome
SMAD7
MYCN
IGFBP2
ADM
STK11
IL5
L-methionine
NKX3-1
salirasib
TEAD4
clofibrate
NFE2L2
PTAFR
imiquimod
IL10
CD3
oblimersen
CAT
SFRP1
IL2RG
enalapril
colchicine
benzo(a)pyrene
alitretinoin
phosphate
TCR
MKNK1
PTPRJ
PTPN6
LEF1
trans-cinnamaldehyde
Pkg
Fibrin
NADPH oxidase
CTR9
WDR5
HTATIP2
CX-5461
PD184352
pifithrin alpha
lipoarabinomannan
diallyl trisulfide
STAT4
PAX3-FOXO1
miR-7110-3p (miRNAs w/seed CUCUCUC)

miR-4327 (miRNAs w/seed GCUUGCA)
miR-4453 (and other miRNAs w/seed AGCUUGG)
miR-3187-3p (miRNAs w/seed UGGCCAU)
miR-656-3p (miRNAs w/seed AUAUUAU)
RTN4
miR-6505-5p (miRNAs w/seed UGGAAUA)
miR-6771-3p (miRNAs w/seed AAACCCC)
miR-660-3p (miRNAs w/seed CCUCCUG)
CD274
miR-4325 (miRNAs w/seed UGCACUU)
miR-432 (and other miRNAs w/seed CUUGGAG)
miR-2392 (miRNAs w/seed AGGAUGG)
GRB2
SAFB
3-nitropropionic acid
miR-582-3p (miRNAs w/seed AACUGGU)
miR-4734 (miRNAs w/seed CUGCGGG)
miR-5088-3p (miRNAs w/seed CCCUUCU)
MOG
TSHZ3
EHMT2
miR-1292-3p (miRNAs w/seed CGCGCCC)
L-proline
pervanadate
dopamine D1 receptor
flavokawain B
triamcinolone hexacetonide
rh-endostatin
GPRC6A
ISX
ST6GALNAC1
ZNF652
KMT5B
iguratimod
sotrastaurin
olaparib
Sfk
HLA-DQ
JDP2
penfluridol
HNRNPR
PRKG2
PDE6B
REV1
BANP
SERPINA4
PHIP
SNCB
NUP107
DLC1
GNAO1
P2RY1
PTBP3
P2RY4
KHDRBS1
PIP5K1C
TP53INP1
CUL7
FKBP1A
OXTR
FKBP5
CUL1
CLIC4
DMTF1
SHCBP1
ERBIN
CCR4
CAPNS1
GLP1R
FUBP1
H4C3
GNMT
TNIK
PILRA
Pde4d
VGF
N-methyl mesoporphyrin IX
Gm26313
dimercaptopropanol
trimethyltin
anethole
CHS-828
bergamottin
polyphosphate
ferric chloride
phytanic acid
non-esterified fatty acid
docetaxel
miR-6764-3p (and other miRNAs w/seed CUCUGGU)
dorsomorphin
mir-24
E2F4
Pln
miR-4717-3p (miRNAs w/seed CACAUGG)
ELL2
APOA1
miR-369-5p (miRNAs w/seed GAUCGAC)
miR-296-3p (miRNAs w/seed AGGGUUG)
miR-292b-5p (and other miRNAs w/seed CUCAAAA)
telmisartan
pravastatin
miR-4474-5p (miRNAs w/seed UAGUCUC)
L-phenylalanine
ganglioside GM3
heparan sulfate
N-Cadherin
meldonium
fingolimod phosphate
2-amino-5-azotoluene
citrinin
Cyclin A
tolfenamic acid
TFIIA
DBH-AS1
UCA1
LINC00461
Retinoic acid-RAR-RXR
MRE11
1,3-bis(4-hydroxyphenyl)-4-methyl-5-[4-(2-piperidinylethoxy)phenol]-1H-pyrazole
tectorigenin
VTCN1
CYTOR
PON2
NELFCD
Lefty
PDS5B
STAG1
hydrogel
evofosfamide
FANCA
HNRNPC
MAP3K12
PLCD1
mir-326
FEM1A
ponesimod
CYP4A11
DLG4
KSR1
MFGE8
DES
APCS
CORO2A
CFD
ABLIM1
THEM4
BARX1
16:0 ceramide-1-phosphate
cytarabine/idarubicin
ADAM19
CD164
PRC1
tetramethylpyrazine
TPD52
MPT0E028
PEX14
spliceostatin A
CID-2011756
SR11256
3,4-(methylenedioxy)cinnamic acid
kurarinone
diclofenac/hyaluronic acid
RB4
RB3
droxinostat
arsenic trichloride
meayamycin B
amurensin G
BCL201
beractant
S-methylisothiopseudouronium
2,5-dihydroxymethylcinnamate
LY311727
BN 50730
nafoxidine
pustulan
trichloroacetic acid
1-(1-glycero)dodeca-1,3,5,7,9-pentaene
methaneselenol
L-threo-safingol
melarsoprol
trichlorfon
PD173955
aspergillus beta-glucan
pneumocystis beta-glucan
flunitrazepam
tolrestat
MUC2
Ro 31-7549
bisindolylmaleimide III
theaflavin monogallate B
potassium iodide
bis-T-23
ETV6-PDGFRB
Fgfr2-Pgap2
CRTC1-MAML2
AuNP@PEG@e14a2
miR-124 inhibitor
anti-miR-32 inhibitor
PT2399
N-t-Boc-Phe-D-Leu-Phe-D-Leu-Phe
IP 20
Gly-Gln
2-(N-myristoylamino)-1-phenyl-1-propanol
10-hydroxydecanoic acid
n-6 docosapentaenoic acid
long-chain alcohol
poractant alfa
lactulose
gestodene
6alpha-fluorotestosterone
V2+
sodium chloride
tributyltin
miR-4494 (miRNAs w/seed CAGACUG)
miR-19b-1-5p (and other miRNAs w/seed GUUUUGC)
RNASEH2A
M344
eflornithine
laminaran
miR-4701-5p (and other miRNAs w/seed UGGCCAC)
miR-4641 (miRNAs w/seed GCCCAUG)
miR-6875-3p (miRNAs w/seed UUCUUCC)
miR-5702 (miRNAs w/seed GAGUCAG)
miR-298 (miRNAs w/seed GCAGAAG)
miR-6802-5p (miRNAs w/seed UAGGUGG)
risperidone
bosentan
IL36B
Relaxin
vasoactive intestinal peptide
LINC00963
BMPER
PLD1
FOXC1
poly dA-dT
SB 290157
FOXC2
STUB1
miR-3616-3p (miRNAs w/seed GAGGGCA)
miR-4785 (miRNAs w/seed GAGUCGG)
miR-6501-5p (miRNAs w/seed GUUGCCA)
miR-3141 (miRNAs w/seed AGGGCGG)
miR-6746-5p (miRNAs w/seed CGGGAGA)
DNMT3B
miR-4733-5p (miRNAs w/seed AUCCCAA)
miR-4755-3p (miRNAs w/seed GCCAGGC)
miR-1900 (and other miRNAs w/seed GCCGCCC)
PCGEM1
NKX2-5
cyclopamine
niclosamide
anacardic acid
ganetespib
PF-3084014
IGF2R
TNFRSF17
ITGA4
HOXB5
IGBP1
HES5
GPC1
CXCL13
PCBP2
STX3
IQGAP1
HOXA1
butylhydroxybutylnitrosamine
allyl isothiocyanate
Trolox C
2-hydroxy-1-naphthylaldehyde isonicotinoyl hydrazone
largazole
tin mesoporphyrin
polymyxin B
miR-4481 (and other miRNAs w/seed GAGUGGG)
miR-5624-3p (and other miRNAs w/seed UAAGGCA)
miR-214-5p (miRNAs w/seed GCCUGUC)
miR-431-3p (miRNAs w/seed AGGUCGU)
mir-27
SCD
miR-5580-5p (and other miRNAs w/seed GCUGGCU)
miR-331-3p (miRNAs w/seed CCCCUGG)
miR-513b-3p (miRNAs w/seed AAUGUCA)
miR-4767 (miRNAs w/seed GCGGGCG)
Nfat (family)
hexachlorobenzene
SERPINF1
GNRH1
TNFRSF1B
RXRA
EGR2
sGC
FZD7
MCF2
rigosertib
RBM14
PSAP
KIDINS220
NME3
SH2B2
Muc1
CLDN2
scoparone
dimaprit
butylated hydroxytoluene
trichloroethylene
benzene
isoproterenol
Ikb
NFAT (complex)
PDGF-AA
RNF20
MAP3K3
IGFBP5
RACK1
TGFB1I1
LCAT
THZ2
chelerythrine
diosgenin
sirolimus
UCP1
PTK2
KLF5
propylthiouracil
fatty acid
adenosine
IL1R1
diphenyleneiodonium
phenylbutazone
thioctic acid
BIRC3
plumbagin
SLPI
BCL2L11
TWIST1
NR1I3
mir-181
N-acetylmuramyl-L-alanyl-D-isoglutamine
NfkB1-RelA
CHI3L1
CDC73
XIAP
CD9
KAT2A
HMGB2
PRKAA2
ciprofloxacin
ETV6-RUNX1
PROCR
DTX1
WR 1065
3-deazaneplanocin
F2RL1
DDX5
gentamicin C
vanillyl-N-nonylamide
LINC00842
ZMIZ1
Crnde
BPIFB1
ghrelin
PLSCR1
PTPRR
CARD14
TRIM33
F2RL3
STX2
YWHAB
HIVEP2
CIMO
famotidine
dibenzoylmethane
TGFB3
BMP7
BSCL2
NRG2
SNAI2
E2F3
choline fenofibrate
REN
PRKAR2B
GRN
imipramine
arsenic
MYOD1
diphtheria toxin
Tlr
TGFB2
5-hydroxytryptamine
azoxymethane
HOXA9
ROCK1
SUB1
HOXB13
S100A6
EPOR
NPPA
stearic acid
MRTFA
KLF2
caffeine
HNF1B
miR-6826-3p (miRNAs w/seed UCCCCUC)
CCND1
MSTN
OGA
miR-1911-5p (miRNAs w/seed GAGUACC)
KITLG
TCF7L1
leucine
C1QA
SFRP4
urethane
miR-6883-3p (miRNAs w/seed UCCCUAU)
patulin
C1orf61
sphingomyelinase
RAP1GAP
AGR2
CBR3-AS1
DAB2
PDE3A
MAD2L2
MED13L
AP20187

DGKZ
RBM38
CSNK2A2
PLP2
LASP1
RASSF10
AQP7
PIWIL2
RGS16
SULF1
GALP
farnesyl transferase
teasaponin
ACTA2
CCL7
H3C6
VIPR2
ethyl 2-[[(6,7-dimethyl-3-oxo-1,2,3,4-tetrahydro-2-quinoxalinyl)acetyl]amino}-4,5-dimethyl-3-thiophenecarboxylate
GADD45GIP1
intralipid
BAY 61-3606
itraconazole
coumestrol
mechlorethamine
myxothiazol
citalopram
rabeprazole
venlafaxine
AGI-1067
5-nitro-2-(3-phenylpropylamino)benzoic acid
magnesium sulfate
N-methyl-3,4-methylenedioxyamphetamine
dehydrocostus lactone
dihematoporphyrin ether
theaflavin
pyrogallol
miR-4757-3p (miRNAs w/seed AUGACGU)
miR-3124-5p (miRNAs w/seed UCGCGGG)
miR-3654 (miRNAs w/seed ACUGGAC)
vitamin D
miR-4781-3p (miRNAs w/seed AUGUUGG)
miR-6824-5p (miRNAs w/seed UAGGGGA)
mir-223
miR-4761-5p (miRNAs w/seed CAAGGUG)
miR-1893 (and other miRNAs w/seed GCGCGGG)
E2F6
miR-187-5p (miRNAs w/seed GCUACAA)
miR-377-3p (miRNAs w/seed UCACACA)
NPY
BACH2
miR-4762-5p (miRNAs w/seed CAAAUCU)
miR-12202-3p (and other miRNAs w/seed CUUCUCU)
miR-3551-5p (and other miRNAs w/seed CUUCACC)
IFNA2
16,16-dimethylprostaglandin E2
triciribine
L-lysine
geneticin
ganglioside GD3
CRTC3
HTR7
SNHG7
TACSTD2
CPEB4
TPSAB1/TPSB2
tryptase
nicotiflorin
PRRX1
UNC5B
AGTRAP
AKAP12
KHSRP
PDGF (family)
DLX1
FGF3
ZIC2
PI3
AGRP
mir-873
MED15
HGS
Ccl6
PRKCG
genipin
WNT5B
UBQLN1
CCND2
MSH2
NELFA
DIAPH1
EIF3A
TSG101
PPP1CA
HP
ANPEP
muscimol
7,8-dihydro-7,8-dihydroxybenzo(a)pyrene 9,10-oxide
1,1-bis(3'-indolyl)-1-(4-trifluoromethyl-phenyl)methane
miR-182 inhibitor
BQ 123
oleoylethanolamide
lead acetate
miR-29b-1-5p (miRNAs w/seed CUGGUUU)
miR-378b (and other miRNAs w/seed UGGACUU)
miR-365-1-5p (and other miRNAs w/seed GGGACUU)
FBXO32
miR-1184 (miRNAs w/seed CUGCAGC)
miR-6798-3p (miRNAs w/seed UACCCCC)
mir-9
mir-214
BTRC
ibrutinib
HSD17B4
ceruletide
miR-584-3p (miRNAs w/seed CAGUUCC)
miR-6819-3p (and other miRNAs w/seed AGCCUCU)
miR-3945 (miRNAs w/seed GGGCAUA)
H2AX
PTPRC
miR-3575 (and other miRNAs w/seed CUGCUGG)
miR-1304-5p (miRNAs w/seed UUGAGGC)
miR-1207-3p (and other miRNAs w/seed CAGCUGG)
ITK
miR-636 (miRNAs w/seed GUGCUUG)
miR-4720-5p (and other miRNAs w/seed CUGGCAU)
miR-8060 (miRNAs w/seed CAUGAAG)
miR-323b-3p (miRNAs w/seed CCAAUAC)
miR-3200-5p (miRNAs w/seed AUCUGAG)
NR4A3
BCL11B
miR-142-3p (and other miRNAs w/seed GUAGUGU)
COMMD3-BMI1
miR-4653-3p (miRNAs w/seed GGAGUUA)
Nr1h
PYCARD
immethridine
miR-5585-5p (miRNAs w/seed GAAGUAC)
miR-876-3p (miRNAs w/seed GGUGGUU)
miR-292-3p (and other miRNAs w/seed AGUGCCG)
nitroglycerin
tannic acid
teriflunomide
coal tar
APOL1
T 0070907
TRIM25
LTB4R2
FFAR1
ruxolitinib
astragalin
ACVR2A
DUSP4
HCK
PDE3B
IL1R2
WWP2
RUVBL2
SART1
CEBPG
IL18BP
JMJD1C
WNT10B
COL17A1
S100A7
TAS1R3
J11-Cl
acriflavine
oligomycin
2-aminoethoxydiphenylborane
zileuton
orlistat
manumycin A
lidocaine
kukoamine A
rosmarinic acid
Z-DEVD-FMK
puerarin
scopolamine
allopregnanolone
canrenoate potassium
miR-6734-5p (and other miRNAs w/seed UGAGGGG)
miR-3199 (and other miRNAs w/seed GGGACUG)
birabresib
miR-664b-5p (miRNAs w/seed GGGCUAA)
miR-877-3p (miRNAs w/seed CCUCUUC)
PRDM5
miR-655-5p (miRNAs w/seed GAGGUUA)
miR-3060-5p (and other miRNAs w/seed GGAGUGC)
miR-431-5p (and other miRNAs w/seed GUCUUGC)
miR-6749-5p (miRNAs w/seed CGGGCCU)
lipid A
N-[N-(3,5-difluorophenacetyl-L-Ala)]-S-phenylglycine t-butyl ester
miR-6716-5p (miRNAs w/seed GGGAAUG)
miR-290b-3p (and other miRNAs w/seed AGUGCCC)
miR-526b-5p (miRNAs w/seed UCUUGAG)
miR-921 (miRNAs w/seed UAGUGAG)
miR-1183 (miRNAs w/seed ACUGUAG)
miR-453 (and other miRNAs w/seed GGUUGCC)
miR-3162-5p (miRNAs w/seed UAGGGAG)
BMI1
miR-4532 (miRNAs w/seed CCCGGGG)
miR-185-5p (and other miRNAs w/seed GGAGAGA)
JINK1/2
Pka catalytic subunit
NOTCH4
CTSS
SLC9A3
ESRRB
acetylcholine
miR-3944-5p (miRNAs w/seed GUGCAGC)
miR-668-5p (miRNAs w/seed GCGCCUC)
miR-5684 (miRNAs w/seed ACUCUAG)
miR-6886-3p (miRNAs w/seed GCCCUUC)
KLF1
IL12A
hexamethylene bisacetamide
miR-6901-5p (miRNAs w/seed GCAGGAA)
miR-6078 (miRNAs w/seed CGCCUGA)
miR-4666b (miRNAs w/seed UGCAUGU)
miR-6778-3p (miRNAs w/seed GCCUCCC)
miR-129-1-3p (and other miRNAs w/seed AGCCCUU)
miR-518a-5p (and other miRNAs w/seed UGCAAAG)
miR-4728-3p (miRNAs w/seed AUGCUGA)
HAND2
levothyroxine
miR-7846-3p (miRNAs w/seed AGCGGAG)
miR-6888-3p (and other miRNAs w/seed UCUGUCU)
miR-6863 (miRNAs w/seed AGACGUG)
miR-1296-3p (and other miRNAs w/seed AGUGGGG)
miR-3077-5p (and other miRNAs w/seed UGCGGAC)
black raspberry extract
miR-5189-3p (and other miRNAs w/seed GCCAACC)
miR-4636 (miRNAs w/seed ACUCGUG)
miR-609 (miRNAs w/seed GGGUGUU)
PP2A
chlorcyclizine
naringin
highly active antiretroviral therapy
Il8r
METTL3
HCAR1
WWC1
S100A12
AFAP1-AS1
FLZ
PELI1
nicorandil
temsirolimus
volinanserin
gliotoxin
diazepam
manidipine
miR-508-3p inhibitor
cyclosporin
JAK1
SB-431542
CLOCK
PELP1
CDK4/6
ITGB3
entinostat
VAV1
PTX3
ACKR3
ramipril
proteasome inhibitor PSI
vinblastine
lithium chloride
STAT5a/b
Fc gamma receptor
PRKG1
EIF2AK4
PRLR
KN 93
RNF31
LCK
KLF11
SSRP1
PDPK1
VTN
SMARCA5
PTTG1
Vhl
L-type Calcium Channel
ARHGEF1
EFEMP1
LPA
ARAF
WNT7B
PROKR1
SERPINH1
MM-401
R59949
buserelin
artemisinin
cordycepin
N,N-dimethylarginine
tosyllysine chloromethyl ketone
PITX2
filgrastim
daporinad
bryostatin 1
CD28
dinoprost
MAP2K6
RELB
captopril
GNA12
GCG
1-methyl-4-phenyl-1,2,3,6-tetrahydropyridine
diclofenac
sodium orthovanadate
KIN001-043
indole-3-carbinol
SN-38
BCR (complex)
NR4A1
MDM2
VHL
N-Ac-Leu-Leu-norleucinal
CRNDE
arginine
EIF4EBP1
NLRP12
IGF1R
ONECUT1
L-cysteine
Agtr1b
4-nonylphenol
C8
Bcl9-Cbp/p300-Ctnnb1-Lef/Tcf
CTDSP1
CD244
GLRX
PSMB9
ATG16L1
CD160
SCUBE3
MCAM
MPL
C7
BRMS1
USP17L2 (includes others)
DEK
asbestos
capsazepine
CITCO
cyclopentenone
RP 73401
JMF3086
I-BOP
ZEB1
miR-126a-3p (and other miRNAs w/seed CGUACCG)
mibolerone
KDM1A
miR-181a-5p (and other miRNAs w/seed ACAUUCA)
CCAR2
PLP1
CTF1
TANK
FOSB
ELF4
histone deacetylase inhibitor
IL12 (complex)
NFKB2
ascorbic acid
CD44
PPARA
GMNN
miR-4688 (and other miRNAs w/seed AGGGGCA)
STAT
SERPINE1
FGFR3
TLR5
PAK2
mevalonic acid
tempol
ethylene glycol tetraacetic acid
thalidomide
SPIB
CFTR
phenobarbital
ERN1
ID2
CDH1
miR-141-3p (and other miRNAs w/seed AACACUG)
NSUN6
FCGR2A

PAK4
CTNNBIP1
NTS
JUP
NACC1
ETV6
NUMBL
HSPB8
PAWR
HSF4
CDH5
BEX2
CXCL1
HNRNPAB
toremifene
roflumilast N-oxide
zinostatin
cucurbitacin B
androstenediol
miR-3690 (miRNAs w/seed CCUGGAC)
miR-5587-3p (miRNAs w/seed CCCCGGG)
miR-6125 (miRNAs w/seed CGGAAGG)
2-(4-acetoxyphenyl)-2-chloro-N-methylethylamine
LATS1
BMPR1B
sterol
TCF7
tetrodotoxin
CX3CL1
miR-6513-5p (miRNAs w/seed UUGGGAU)
miR-3190-3p (and other miRNAs w/seed GUGGAAG)
miR-670-3p (miRNAs w/seed UUCCUCA)
TRAP1
POU2F2
miR-4744 (miRNAs w/seed CUAAAGA)
miR-6849-3p (miRNAs w/seed CCAGCCU)
miR-548ad-3p (miRNAs w/seed AAAACGA)
miR-1287-3p (and other miRNAs w/seed UCUAGCC)
miR-4449 (miRNAs w/seed GUCCCGG)
Pro-inflammatory Cytokine
mir-15
miR-5091 (miRNAs w/seed CGGAGAC)
miR-4774-3p (miRNAs w/seed UUGCCUA)
miR-340-3p (and other miRNAs w/seed CCGUCUC)
miR-1587 (and other miRNAs w/seed UGGGCUG)
KLF15
miR-7159-3p (miRNAs w/seed UUCUAUG)
miR-6804-3p (miRNAs w/seed GCACCUG)
miR-4285 (miRNAs w/seed CGGCGAG)
DNMT1
DLL1
SND1
NFIL3
NPC2
PD 123319
deoxynivalenol
miR-1914-5p (miRNAs w/seed CCUGUGC)
miR-548s (miRNAs w/seed UGGCCAA)
miR-124-5p (miRNAs w/seed GUGUUCA)

MEOX2
PRKN
sorafenib
CGS 21680
parthenolide
ethionine
ACOX1
L-threonine
CAMK2N2
LINC01139
FAT1
Shc
BCO2
Sox2ot
cercosporin
NCK1
HCRT
IL9R
SETD7
N4BP1
KLB
EXT1
INCB054329
diltiazem
calmidazolium
tiron
SU1498
SS18-SSX1
BQ-788
miR-495-3p (and other miRNAs w/seed AACAAAC)
lithium
homocysteine
IL27
ciprofibrate
miR-5579-3p (miRNAs w/seed UAGCUUA)
miR-27a-3p (and other miRNAs w/seed UCACAGU)
NOX4
pimagedine
haloperidol
TFRC
tazemetostat
T3-TR-RXR
PGF
HSPA9
THBS1
4-methylnitrosoamino-1-(3-pyridinyl)-1-butanone
valproic acid
halofuginone
MAX
cinnamaldehyde
let-7
miR-3613-3p (miRNAs w/seed CAAAAAA)
PBRM1
GPR132
CCL1
RLIM
PTCSC3
PIKFYVE
ENTPD1
CNB-001
Ncoa6
UBR5
7-(4-fluorobenzylamino)-1,3,4,8-tetrahydropyrrolo[4,3,2-de]quinolin-8(1H
pterostilbene
tyrphostin AG 1296
Cu2+
ATF1
chloropromazine
S-nitrosoglutathione
GDF2
BCR-ABL1
miR-709 (and other miRNAs w/seed GAGGCAG)
AGN194204
HBB
SRSF2
retinoid
4-hydroxynonenal
mir-30 (includes others)
ouabain
17-alpha-ethinylestradiol
TGM2
DIO2
diethylstilbestrol
SP2509
miR-3614-3p (miRNAs w/seed AGCCUUC)
ANGPT1
CDK2
miR-19b-3p (and other miRNAs w/seed GUGCAAA)
embelin
SOX2-OT
DEPTOR
CCAT1
UBA1
SH3KBP1
mir-379
CCNT1
PAX4
C9
bisphenol A diglycidyl ether
D-sphingosine
trientine
NAD+
miR-3679-3p (miRNAs w/seed UUCCCCC)
HBEGF
TXNIP
TBK1
Ifn gamma
flutamide
RUNX1-RUNX1T1
streptozocin
miR-1268a (and other miRNAs w/seed GGGCGUG)
miR-3156-3p (miRNAs w/seed UCCCACU)
miR-204-5p (and other miRNAs w/seed UCCCUUU)
TO-901317
eicosapentenoic acid
roscovitine
SMAD1
vincristine
NR1I2
mono-(2-ethylhexyl)phthalate
digoxin
desoxycorticosterone
Pdgfr
trypsin
harmine
CSNK2B
FGF23
EN1
CRY2
BCAR1
SDC1
PTGER1
CRY1
HSPA1A/HSPA1B
1,2-dimethylhydrazine
N,N-dimethylsphingosine
astressin 2B
verteporfin
GATA2
miR-4476 (and other miRNAs w/seed AGGAAGG)
PRMT5
APLN

PPRC1
zVAD-FMK
RPTOR
N-cor
SCARB1
EGR3
MYBL2
bicalutamide
MRTFB
xanthine
glycochenodeoxycholate
Rac
IDH2
PTBP1
F10
SRSF3
ST14
ebselen
pyruvic acid
salmeterol
nickel chloride
buthionine sulfoximine
PRKD1
miR-30c-5p (and other miRNAs w/seed GUAAACA)
fluticasone propionate
IFN Beta
SMARCB1
ROCK2
TSC22D3
IKZF1
NFATC1
RARB
SRC (family)
IDH1
CCN5
TNFSF12
cyclic GMP
ADP
IGFBP7
AIMP1
APBB1
PER2
HOXB4
GNA13
TGFBR3
EDNRA
TOPBP1
Mn(III)-tetrakis-(4-benzoic acid) porphyrin
uric acid
miR-512-3p (miRNAs w/seed AGUGCUG)
GHR
FOXO4
plicamycin
PRDM1
EIF2S1
iron
miR-4786-5p (miRNAs w/seed GAGACCA)
prostaglandin A2
MAPK10
CXCL2
PRDX2
MAML1
BCL10
BHLHA15
RHOB
4-nitroquinoline-1-oxide
farnesyl pyrophosphate
reserpine
mannose
miR-6772-3p (miRNAs w/seed UGCUCCU)
HDL
carboplatin
3-methylcholanthrene
SYK
GNA15
miR-4716-3p (and other miRNAs w/seed AGGGGGA)
miR-1-3p (and other miRNAs w/seed GGAAUGU)
miR-3157-5p (miRNAs w/seed UCAGCCA)
NFkB (family)
Smad2/3-Smad4
GIP
PSMB8
PPP2R5C
GPI
MAZ
DAXX
KLK5
premarin
decursin
nimodipine
glutamyl-Se-methylselenocysteine
losartan potassium
PAX5
BTK
PIM1
CD14
ZEB2
CCL11
miR-585-5p (miRNAs w/seed UAGCACA)
miR-3934-3p (miRNAs w/seed GCUCAGG)
CBX5
emodin
15(S)-HETE
Pdgf Ab
PLC
PKNOX2
PI-103
PADI2
LYL1
ARHGDIG
PDGFRA
PLA2G4A
CD22
XPO1
MAPKAPK3
YY2
acyclic retinoid
miR-4645-3p (miRNAs w/seed GACAGUA)
dextran sulfate
1,4-bis[2-(3,5-dichloropyridyloxy)]benzene
TLR2
miR-6729-3p (miRNAs w/seed CAUCCCC)
AMPK
POU2F1
miR-6861-3p (miRNAs w/seed GGACCUC)
sunitinib
IL11
HES1
clozapine
ARNT
miR-6752-5p (and other miRNAs w/seed GGGGGUG)
Srebp
trastuzumab
TMSB4
BAP1
MUC4
EMD
diethylmaleate
helenalin
dichlororibofuranosylbenzimidazole
SPI1
FLI1

carbon monoxide
PSMB11
miR-517a-3p (and other miRNAs w/seed UCGUGCA)
miR-3168 (miRNAs w/seed AGUUCUA)
ID3
Fcer1
HOXD10
N-methyl-D-aspartate
glucosamine
MIR17HG
E. coli B4 lipopolysaccharide
cyclooxygenase
CDK5R1
KLF10
AURKB
IAPP
LY6E
RND3
DUSP5
pevonedistat
AS1842856
salvin
miR-148a-3p (and other miRNAs w/seed CAGUGCA)
9-hydroxy-(S)-10,12-octadecadienoic acid
pelargonidin
2'-adenylic acid
peptide YY 3-36
G protein alpha
growth factor receptor
Ahr-aryl hydrocarbon
2,2',4,4',5,5'-hexabromodiphenyl ether
5,7-dimethoxyflavone
DE 71
Ahr-aryl hydrocarbon-RelA
NR2F1-AS1
PKR dimer
GSK 189254
CLTA
(-)-OSU 6162
GJC2
PMM2
NCK
pridopidine
FBXO31
NLRP2
FBXO22
SRF-ELK1
WFDC1
KCNJ12
CALCOCO1
URGCP
RND2
TMEM184A
DEPDC1
NACC2
MYADM
CCDC22
CCDC62
KCTD10
harman
FGF14
TRPM3
PLAC1
RNF181
TCF4-CTNNβ
hispidulin
NNZ 2566
vasopressins
black cohosh extract
Integrin alpha 5 beta 1
mediator
Mapk kinase
Rho gdi
GLIPR1
NSMCE3
ARHGEF17
PRCC
MEDI-547
Tenascin
DYNLRB1
RBMS1
AHRR
KIFAP3
MED7
PPY
CDC45
GPHA2
SLC52A1
ADCY2
CLTB
mir-363
mir-1301
NPVF
SLC30A9
CNOT9
CAMK2
TOB2
SLC1A2
GTPBP4
GPHB5
SCN7A
NAPB
ANKS1A
PTPRU
MED21
PMS2
Sin3b
EPGN
TAX1BP3
Tnxa-ps1
PRDM2
RAB5B
CDK20
GDF10
cytarabine/filgrastim/fludarabine phosphate
ETS1-AS1
PTOV1
INHBE
EXOSC10
PPP2R3A
ZBTB14
RPS20
CHRM2
Flt1
molibresib
oligomycin A
CML advanced glycation end products
dihydrotanshinone I
NMS-873
RPL21
SLC25A5
RPLP0
NBI 27914
SB202474
impromidine
guanfacine
amlexanox
sterigmatocystin
ondansetron
cyclothiazide
SU6657
flurothyl

L 733060
SC-19220
sulfaphenazole
selfotel
(R)-5-(2-azetidnylmethoxy)-2-chloropyridine
Ro 20-1724
BTG3-AS1
GS-626510
pubchem compound 353999
thiabendazole
arachidonyl-2-chloroethylamide
naphthalene-1-2-dione
trans-7,8-dihydroxy-7,8-dihydrobenzo(a)pyrene
anthracene
ACS-1
2-amino-3-methylimidazo(4,5-f)quinoline
ONO-8711
anti-miR-302d
AP-1 decoy
anti-miR-302c inhibitor
beta-penta-O-galloyl-glucose
BRD4-NUTM1
ZBTB16-RARA
SU11333
CTOP
PF-4800567
Ac-DEVD-CHO
ibutamoren
SMAD3-EP300
3-methoxyapigenin
berberrubine
columbamine
harmaline
LY 354740
droxidopa
S(-)-carbidopa
3'-adenylic acid
2'-fucosyllactose
sarcosine
allose
estramustine
miR-6867-3p (and other miRNAs w/seed UCUCCCU)
miR-5107-3p (and other miRNAs w/seed AACCUGU)
geldanamycin
PRMT1
sulindac
miR-5131 (and other miRNAs w/seed GGCGCCC)
RUNX3
MECP2
NANOG
MBD2
hydroxyurea
Stat5 dimer
LGR5
MED12
PER1
CDK8
PARK7
W7
pilocarpine
nickel
miR-519a-3p (and other miRNAs w/seed AAGUGCA)
miR-214-3p (and other miRNAs w/seed CAGCAGG)
GATA1
WNT3A
vitamin E
CIP2A
cytarabine
WNT5A
IFNB1
miR-4684-3p (miRNAs w/seed GUUGCAA)
miR-4493 (miRNAs w/seed GAAGGCC)
TNFSF13B
CaMKII
EIF4G2
CCK
NFIX
CCN3
PTPN3
PDGFC
HSP90AB1
ketamine
GLI1
MYRF
EFNA1
gemcitabine
miR-561-5p (miRNAs w/seed UCAAGGA)
APC
miR-145-5p (and other miRNAs w/seed UCCAGUU)
clopidogrel
SUZ12
zidovudine
pregna-4,17-diene-3,16-dione
FAM3B
MGP
CNR2
Sch-23390
epicatechin gallate
2,4-dinitrofluorobenzene
9Z,11E-octadecadienoic acid
NFATC3
PKM
IL22
CASR
miR-2355-5p (miRNAs w/seed UCCCCAG)
miR-605-3p (miRNAs w/seed GAAGGCA)
miR-3190-5p (miRNAs w/seed CUGGCCA)
miR-7854-3p (miRNAs w/seed GAGGUGA)
miR-6894-3p (miRNAs w/seed UGCCUGC)
JUN/JUNB/JUND
WNT7A
COL4A3
NTRK1
HEY1
THZ1
bilirubin
SOX11
molybdenum disulfide
SOX1
E2F2
Tcf7
miR-130a-3p (and other miRNAs w/seed AGUGCAA)
CHD7
Smad
E2F5
SOX6
LRPAP1
HEXIM1
COMMD1
TRPV4
SDCBP
SSTR2
PCYT1A
UCN-01
miR-450b-5p (miRNAs w/seed UUUGCAA)
STK40
minocycline
nifedipine
miR-200a-5p (and other miRNAs w/seed AUCUUAC)
thyroid hormone receptor
DCAF1
OXT

MST1
RCAN1
HDAC9
NOS1
PSMD10
HSPD1
diazoxide
K-252
calyculin A
FGF7
ROR2
Bay 11-7082
CDX2
miR-4504 (miRNAs w/seed GUGACAA)
HDAC5
TBX5
p70 S6k
AMH
vemurafenib
NCL
mir-148
BGN
MAP2K2
CPE
isoflurane
miR-1255b-5p (and other miRNAs w/seed GGAUGAG)
miR-4433b-3p (miRNAs w/seed AGGAGUG)
PIN1
SOX3
nelfinavir
miR-452-3p (miRNAs w/seed UCAUCUG)
miR-4503 (miRNAs w/seed UUAAGCA)
miR-129-5p (and other miRNAs w/seed UUUUUGC)
miR-4687-5p (miRNAs w/seed AGCCCUC)
estradiol benzoate
JUND
cholesterol
NCF1
HMGN1
TAF6
MALP-2s
apomorphine
phorbol 12,13-didecanoate
miR-1237-3p (and other miRNAs w/seed CCUUCUG)
miR-5008-5p (miRNAs w/seed GAGGCCC)
miR-8088 (miRNAs w/seed CUCGGUA)
miR-6888-5p (miRNAs w/seed AGGAGAU)
miR-567 (miRNAs w/seed GUAUGUU)
Rb
MBD3
miR-34a-5p (and other miRNAs w/seed GGCAGUG)
miR-6800-3p (miRNAs w/seed ACCUCUC)
PTGER4
miR-3940-3p (miRNAs w/seed AGCCCGG)
Rab11
NOSIP
dibromoacetic acid
mir-331
STAMBP
UBE2V1
sorafenib derivative SC-1
3-iodobenzylguanidine
lavendustin A
EWSR1-WT1
digitoxin
1-phenazinecarboxylic acid
miR-6887-3p (miRNAs w/seed CCCCUCC)
miR-6815-5p (and other miRNAs w/seed AGGUGGC)
taurocholic acid
crizotinib
SH3TC2
ACTL6A
CFLAR
ketoconazole
letrozole
hemozoin
miR-512-5p (miRNAs w/seed ACUCAGC)
miR-6732-3p (miRNAs w/seed AACCCUG)
3,4,5,3',4'-pentachlorobiphenyl
indican
tosedostat
RPSA
SIAH2
PRKCI
LTB4R
R-WIN 55,212
miR-3192-5p (miRNAs w/seed CUGGGAG)
maslinic acid
10E,12Z-octadecadienoic acid
prednisolone
SRSF1
PRKAR1A
MECOM
KNG1
VAV2
miR-6840-5p (miRNAs w/seed CCCCCGG)
NPC1
miR-4648 (miRNAs w/seed GUGGGAC)
niacinamide
TIRAP
PTH1R
NF2
SOX10
SMAD5
linsidomine
betulinic acid
miR-4634 (miRNAs w/seed GGCGCGA)
miR-17-5p (and other miRNAs w/seed AAAGUGC)
miR-6798-5p (miRNAs w/seed CAGGGGG)
4-phenylbutyric acid
miR-6853-5p (miRNAs w/seed GCGUGGG)
SREBF1
SAMSN1
nocodazole
WNT4
MARK2
AHCY
GDF11
vitamin A
eplerenone
6-hydroxydopamine
PD 0325901
OSCAR
GAB2
EZR
PAK1
EWSR1
daunorubicin
bardoxolone
miR-6749-3p (miRNAs w/seed UCCUCCC)
miR-1252-5p (miRNAs w/seed GAAGGAA)
miR-936 (miRNAs w/seed CAGUAGA)
LMNA
PDLIM2
miR-224-5p (miRNAs w/seed AAGUCAC)
miR-31-3p (and other miRNAs w/seed GCUAUGC)
DSCAM
NLRP3
resolvin D1
CYBB
IL17F
bafilomycin A1

FUS-DDIT3
andrographolide
naringenin
miR-583 (miRNAs w/seed AAAGAGG)
docosahexaenoic acid
sethoxydim
INTS11
PUM1
C1QL1
ZNF300
Trk Receptor
EIF4F
EMP2
HSPB3
PUM2
FOXO6
GFRA2
INHBC
ERC1
IL20RA
filipin
2,2-dimethylbutyric acid
rocuronium
minodronate
betamethasone valerate
nickel subsulfide
senexin B
ODN-BW006
R-etodolac
oleandrin
EHF
miR-3118 (and other miRNAs w/seed GUGACUG)
miR-5008-3p (and other miRNAs w/seed CUGUGCU)
miR-542-3p (miRNAs w/seed GUGACAG)
Ap2
8-bromoguanosine 3',5'-cyclic monophosphate
CASP1
TFDP1
IGFBP3
miR-193a-3p (and other miRNAs w/seed ACUGGCC)
PIAS1
RAD21
alvocidib
miR-891a-5p (miRNAs w/seed GCAACGA)
miR-3148 (miRNAs w/seed GGAAAAA)
LEPR
RB1
nitrofen
NSD2
TSLP
GDF9
NQO1
SPRY2
MMP2
ADORA2B
cilostazol
steroid
miR-1471 (miRNAs w/seed CCCGCGU)
miR-6822-3p (miRNAs w/seed GGCUCUA)
SHH
miR-769-5p (miRNAs w/seed GAGACCU)
miR-4432 (miRNAs w/seed AAGACUC)
IL18
CYLD
NR2F1
fluvastatin
NR1H4
miR-455-5p (and other miRNAs w/seed AUGUGCC)
INHA
miR-4681 (miRNAs w/seed ACGGGAA)
miR-1226-3p (miRNAs w/seed CACCAGC)
NKX2-1
CEACAM1
IRF4
IRF1
conjugated linoleic acid
LGR4
memantine
miR-6818-3p (miRNAs w/seed UGUCUCU)
CGP 74514A
6-dimethylaminopurine
PD 180970
colfosceril palmitate
Rsk
Esr1-Esr1-estrogen-estrogen
CC8490
benoxaprofen
NPSR1
XRN2
Sapk
BIIB021
ZNF467
ARTN
lanatoside C
HSPB6
NME2
CHRM4
chelidonine
PODXL
abrocitinib
NSC719239
hoechst 33342
clomiphene
alpha-methylhydrocinnamic acid
pentamidine
bupropion
CCI-007
tyrphostin AG 18
pyocyanin
Co2+
miR-6728-3p (miRNAs w/seed CUCUGCU)
miR-3571 (and other miRNAs w/seed ACACACU)
miR-221-3p (and other miRNAs w/seed GCUACAU)
MSX2
PTGS1
EFNA4
FASLG
ibuprofen
everolimus
SAHM1
LDB1
miR-1973 (miRNAs w/seed CCGUGCA)
miR-12201-5p (and other miRNAs w/seed GGAAGGA)
INHBA
colistin
LMO2
SOX4
SLC27A2
EED
RPS6KB1
SELPLG
ARHGAP21
EFNA3
fasudil
beta-carotene
miR-4722-5p (miRNAs w/seed GCAGGAG)
kanamycin A
TNC
CITED2
NME1
GATA6
EOMES

miR-199a-3p (and other miRNAs w/seed CAGUAGU)
miR-6752-3p (miRNAs w/seed CCCUGCC)
miR-4290 (miRNAs w/seed GCCCUCC)
RBM5
A2M
EFNA5
PCGF2
dimethylnitrosamine
acetovanillone
miR-4713-5p (miRNAs w/seed UCUCCCA)
PRKAA
mir-146
MED13
MEF2A
RIPK1
temozolomide
miR-153-3p (miRNAs w/seed UGCAUAG)
metronidazole
ILX-23-7553
L-alpha-lysophosphatidylcholine, palmitoyl
styrene
MNK1/2
Cpla2
MELK
KLF8
BPI
MTF2
UTP18
GPR68
MAP4K1
RNF34
LOC102724788/PRODH
APAF1
SENP6
PELI2
2-cyclohexen-1-one
mevalonolactone
lindane
Collagen type V
butylphen
aclarubicin
cannabinoid drugs
phloroglucinol
5-hydroxytryptophan
miR-6726-3p (miRNAs w/seed UCGCCCU)
melatonin
obeticholic acid
miR-202-3p (and other miRNAs w/seed GAGGUAU)
Hsp27
FYN
NOS3
NUP98-DDX10
EIF4E
miR-92a-3p (and other miRNAs w/seed AUUGCAC)
miR-291a-3p (and other miRNAs w/seed AAGUGCU)
miR-4251 (miRNAs w/seed CUGAGAA)
IL24
miR-4751 (miRNAs w/seed GAGGACC)
Hedgehog
AURK
ANLN
miR-6753-5p (miRNAs w/seed ACCAGGG)
histidinol
Iberiotoxin
tacedinaline
obatoclax
CCAR1
ANKHD1/ANKHD1-EIF4EBP3
lumefantrine
LDL-cholesterol
GSK690693
PRKD2
HSPB2
BAD
PROK2
Pzp
NUCB2
ETV3
OTUD7B
CCL15
OXSR1
TFF2
XPA
ZYX
fenebrutinib
enzastaurin
ambroxol
2-morpholin-4-yl-6-thianthren-1-yl-pyran-4-one
pyridoxamine
chlorambucil
PDZ1i
8-chlorophenylthio-adenosine 3',5'-cyclic monophosphate
TEAD2
beta-naphthoflavone
miR-423-5p (and other miRNAs w/seed GAGGGGC)
miR-489-5p (miRNAs w/seed GUCGUAU)
RUNX2
ZBTB20
panobinostat
mir-183
bromodeoxyuridine
MTORC1
TMPRSS2-ERG
miR-4520-3p (miRNAs w/seed UGGACAG)
CEBPE
LPL
EPHB4
Am 580
miR-642a-3p (and other miRNAs w/seed GACACAU)
miR-8074 (miRNAs w/seed UAUGGCG)
FGF10
CD5
miR-1825 (miRNAs w/seed CCAGUGC)
alloxan
MRPS18B
SH2D5
BCAR4
SLC5A8
TCIM
Collagen type III
ASCL2
idelalisib
IER3
DVL1
CCN4
MEFV
IFNGR
WNK1
ENO1
TSC22D1
RBX1
EGR4
BANCR
TBXA2R
PDK1
MLLT3
TNKS
casticin
ADCYAP1R1
PGK1
MS4A1
KLKB1

NPAS2
RHOC
15-E2-isoketal modified phosphatidylethanolamine
perifosine
aminoglutethimide
4-coumaric acid
fluphenazine
2-mercaptoethanol
retroinverso ERG inhibitory peptide 2
retroinverso ERG inhibitory peptide 1
atropine
phosphatidic acid
12-hydroxyeicosatetraenoic acid
acolbifene
vinpocetine
arsenic trioxide
imatinib
BIRC5
CBL
miR-5190 (miRNAs w/seed CAGUGAC)
miR-614 (miRNAs w/seed AACGCCU)
IL32
EFNA2
miR-4757-5p (and other miRNAs w/seed GGCCUCU)
miR-5088-5p (miRNAs w/seed AGGGCUC)
WBP2
miR-4691-5p (and other miRNAs w/seed UCCUCCA)
miR-548at-5p (miRNAs w/seed AAAGUUA)
mir-204
Gcg
miR-18a-5p (and other miRNAs w/seed AAGGUGC)
1810019D21Rik
IL12 (family)
miR-4717-5p (and other miRNAs w/seed AGGCCAC)
miR-31-5p (and other miRNAs w/seed GGCAAGA)
15-epi-lipoxin A4
UDP
rhodamine 6G
2,5-bis(5-hydroxymethyl-2-thienyl)furan
SENP7
DUOXA1
SULT2B1
ZNF382
LTBP4
CYSLTR2
IL-2R
IL26
parathyroid hormone-related protein
HDAC10
BAG6
SLC25A4
S100a7a
CXCR1
SMARCA1
RGS19
GAL3ST1
CYSLTR1
USP2
MAOA
ATF7
GRB10
TBL1X
NEU3
TSPO
CCDC80
DIABLO
PCBP1
TBL1XR1
proteasome inhibitor
naproxen
raclopride
NCX-4040
GW 5074
domoic acid
pranlukast
vandetanib
carnosol
tyrphostin
palmitoyl-Cys((RS)-2,3-di(palmitoyloxy)-propyl)-Ala-Gly-OH
alpha-tocopherol succinate
S-nitroso-N-acetyl-DL-penicillamine
PPP2CA
CHD4
capsaicin
miR-3547 (and other miRNAs w/seed GAGCACC)
miR-7158-5p (miRNAs w/seed GCUCAAU)
TET2
TNFRSF8
HIC1
miR-504-5p (and other miRNAs w/seed GACCCUG)
miR-3176 (and other miRNAs w/seed CUGGCCU)
miR-16-5p (and other miRNAs w/seed AGCAGCA)
miR-187-3p (miRNAs w/seed CGUGUCU)
miR-6512-3p (and other miRNAs w/seed UCCAGCC)
Smad2/3
C5AR1
RHO
seocalcitol
miR-6084 (miRNAs w/seed UCCGCCA)
miR-548aj-5p (and other miRNAs w/seed GCAAAAG)
STING1
NRIP1
miR-5193 (miRNAs w/seed CCUCCUC)
ganglioside
cholesterol sulfate
bazedoxifene
Pde
BMS-387032
FBXO42
AFF4
KDM4B
LRRC32
HUWE1
CTNND1
TPM3
DKK2
GUSB
salinomycin
PTGES3
CDKN2B
RPL11
USP37
PINK1
GPR183
ST6GAL1
COCH
TKT
NELFE
RECK
prodigiosin
ketorolac
hexamethoxyflavone
iodine
5-iodotubercidin
miR-3667-3p (miRNAs w/seed CCUUCCU)
HNRNPA2B1
morphine

**Supplementary Table S24. Unique and overlapping Upstream Regulators for the compassion of the conditions "Calu-3_SARS-CoV-2" and "Calu-3_SARS-CoV".** Comparative analysis was done using VENNY 2.1. Data visualization was done by R-based Venn diagrams shown in Figure 5.

**989 elements included exclusively in "Calu-3_SARS-CoV-2":**
(-)-norephedrine
RPTOR
elaidic acid
SUMO2
miR-661 (miRNAs w/seed GCCUGGG)
CYP51A1
propylthiouracil
miR-1289 (miRNAs w/seed GGAGUCC)
miR-1233-5p (and other miRNAs w/seed GUGGGAG)
miR-135b-3p (and other miRNAs w/seed UGUAGGG)
miR-4722-3p (and other miRNAs w/seed CCUGCCA)
C4BP
Gm35986
mono-(2-ethylhexyl)phthalate
miR-607 (miRNAs w/seed UUCAAAU)
miR-485-5p (and other miRNAs w/seed GAGGCUG)
miR-363-5p (and other miRNAs w/seed GGGUGGA)
miR-939-3p (miRNAs w/seed CCUGGGC)
miR-4804-5p (miRNAs w/seed UGGACGG)
FGF19
KIF3A
TRPS1
miR-3116 (miRNAs w/seed GCCUGGA)
FAM3B
LGALS7/LGALS7B
UPF2
miR-3473b (and other miRNAs w/seed GGCUGGA)
IAPP
ATP7B
ATP5IF1
miR-204-3p (and other miRNAs w/seed CUGGGAA)
KDM8
miR-6762-5p (and other miRNAs w/seed GGGGCCA)
miR-222-5p (miRNAs w/seed UCAGUAG)
miR-550a-5p (and other miRNAs w/seed GUGCCUG)
miR-3194-5p (miRNAs w/seed GCCAGCC)
miR-6747-3p (and other miRNAs w/seed CCUGCCU)
miR-6832-3p (miRNAs w/seed CCCUUUU)
MAPT
LIN9
miR-6854-3p (miRNAs w/seed GCGUUUC)
SNCA
miR-1231-3p (miRNAs w/seed GCCCUGU)
PKM
miR-7977 (miRNAs w/seed UCCCAGC)
PPARGC1B
miR-1293 (and other miRNAs w/seed GGGUGGU)
miR-299a-3p (and other miRNAs w/seed AUGUGGG)
miR-1303 (miRNAs w/seed UUAGAGA)
LINC00842
TCF/LEF
INSIG2
miR-4761-5p (miRNAs w/seed CAAGGUG)
miR-3670 (miRNAs w/seed GAGCUCA)
DBI
CIITA
6-amino-4-(4-phenoxyphenylethylamino)quinazoline
bendamustine
CHRM4
CDC20
miR-432-3p (and other miRNAs w/seed UGGAUGG)
miR-3918 (miRNAs w/seed CAGGGCC)
miR-1587 (and other miRNAs w/seed UGGGCUG)
miR-4260 (and other miRNAs w/seed UUGGGGC)
miR-4709-5p (miRNAs w/seed CAACAGU)
MED13
miR-6879-3p (miRNAs w/seed GUCACCC)
mir-24
sunitinib
miR-6832-5p (miRNAs w/seed GUAGAGA)
miR-4654 (and other miRNAs w/seed GUGGGAU)
miR-1976 (and other miRNAs w/seed CUCCUGC)
PCGEM1
MYOC
RRP1B
miR-192-3p (miRNAs w/seed UGCCAAU)
miR-512-5p (miRNAs w/seed ACUCAGC)
miR-290b-3p (and other miRNAs w/seed AGUGCCC)
miR-6512-5p (miRNAs w/seed ACCAUUA)
miR-129-5p (and other miRNAs w/seed UUUUUGC)
miR-4720-5p (and other miRNAs w/seed CUGGCAU)
anacardic acid
CACNA1C
GNE
thenoyltrifluoroacetone
miR-6761-5p (and other miRNAs w/seed CUGAGAG)
miR-6778-3p (miRNAs w/seed GCCUCCC)
YWHAZ
miR-6880-5p (miRNAs w/seed GGUGGAG)
miR-1188-3p (and other miRNAs w/seed GAGGCUC)
miR-4521 (miRNAs w/seed CUAAGGA)
MYBL2
miR-3189-5p (miRNAs w/seed GCCCCAU)
NAE1
RGCC
HOXA4
miR-4468 (miRNAs w/seed GAGCAGA)
miR-4640-5p (and other miRNAs w/seed GGGCCAG)
FDFT1
miR-3651 (miRNAs w/seed AUAGCCC)
miR-3126-3p (miRNAs w/seed AUCUGGC)
SFRP1
miR-4301 (miRNAs w/seed CCCACUA)
miR-3147 (miRNAs w/seed GUUGGGC)
miR-3622a-5p (miRNAs w/seed AGGCACG)
Hbb-b1
PPP3CA
pepstatin
ETV4
miR-1915-5p (miRNAs w/seed CCUUGCC)
diallyl disulfide
miR-4762-5p (miRNAs w/seed CAAAUCU)
ATN1
miR-1199-5p (and other miRNAs w/seed CUGAGCC)
miR-5583-5p (and other miRNAs w/seed AACUAAU)
miR-6724-5p (and other miRNAs w/seed UGGGCCC)
COTI-2
RFX5
miR-1896 (and other miRNAs w/seed GGUGGGU)
miR-3186-5p (miRNAs w/seed AGGCGUC)
PAX5
MAP4K4
SAHM1
miR-410-5p (and other miRNAs w/seed GGUUGUC)
miR-4297 (and other miRNAs w/seed GCCUUCC)
miR-660-3p (miRNAs w/seed CCUCCUG)
miR-5131 (and other miRNAs w/seed GGCGCCC)
miR-3179 (miRNAs w/seed GAAGGGG)
ETV1
miR-623 (miRNAs w/seed UCCCUUG)
miR-4314 (and other miRNAs w/seed UCUGGGA)
KRT14
miR-561-3p (miRNAs w/seed AAAGUUU)
miR-1245a (and other miRNAs w/seed AGUGAUC)
CTNNBIP1
SH3KBP1
bisphenol A diglycidyl ether
bezafibrate
defibrotide
STRAP
miR-377-5p (and other miRNAs w/seed GAGGUUG)
miR-6751-5p (and other miRNAs w/seed UGGGGGU)
miR-551b-5p (miRNAs w/seed AAAUCAA)
miR-4471 (and other miRNAs w/seed GGGAACU)
LMNB1
KMT2D
miR-504-5p (and other miRNAs w/seed GACCCUG)
discodermolide
sucrose
miR-4776-5p (miRNAs w/seed UGGACCA)
miR-1915-3p (and other miRNAs w/seed CCCAGGG)
miR-3192-5p (miRNAs w/seed CUGGGAG)
miR-602 (miRNAs w/seed ACACGGG)
miR-520a-5p (and other miRNAs w/seed UCCAGAG)
miR-10394-3p (and other miRNAs w/seed GGGCGCG)
SLC16A3
ST6GAL1
1,1-bis(3'-indolyl)-1-(4-hydroxyphenyl)methane
calcium chloride
miR-370-3p (and other miRNAs w/seed CCUGCUG)
miR-3664-3p (miRNAs w/seed CUCAGGA)

**689 elements included exclusively in "Calu-3_SARS-CoV":**
EGFL7
miR-452-3p (miRNAs w/seed UCAUCUG)
SH2B3
chelerythrine
TRIM28
LPAR3
vapreotide
miR-1537-5p (miRNAs w/seed GCUGUAA)
miR-331-5p (and other miRNAs w/seed UAGGUAU)
tripterine
clodronic acid
miR-223-3p (miRNAs w/seed GUCAGUU)
prostaglandin A1
spermidine
miR-141-5p (and other miRNAs w/seed AUCUUCC)
NANOG
miR-3102-3p (and other miRNAs w/seed AGCACCC)
metyrapone
miR-103-3p (and other miRNAs w/seed GCAGCAU)
R-WIN 55,212
C1q
apigenin
miR-383-5p (miRNAs w/seed GAUCAGA)
chrysin
HISTONE
miR-624-5p (miRNAs w/seed AGUACCA)
miR-93-3p (miRNAs w/seed CUGCUGA)
PTPRC
cholesterol sulfate
BRMS1
MAPK8IP1
isoflurane
PON1
spermine nitric oxide complex
palmitoleic acid
ethyl gallate
miR-4320 (miRNAs w/seed GGAUUCU)
AHCY
miR-6757-3p (miRNAs w/seed ACACUGG)
miR-335-3p (miRNAs w/seed UUUUCAU)
Iberiotoxin
RAP1A
CBLC
dieldrin
miR-6500-3p (miRNAs w/seed CACUUGU)
IL6ST
8-chlorophenylthio-adenosine 3',5'-cyclic monophosphate
miR-3150a-5p (and other miRNAs w/seed AACCUCG)
RAB1B
SKP2
CSF3R
miR-6716-3p (miRNAs w/seed CCGAACU)
cortistatin A
mesalamine
flutamide
GW501516
miR-7108-5p (miRNAs w/seed UGUGGCC)
chlorogenic acid
miR-548ae-3p (and other miRNAs w/seed AAAAACU)
CUX1
miR-4479 (miRNAs w/seed GCGCGGC)
indole-3-carbinol
miR-548p (miRNAs w/seed AGCAAAA)
KDR
miR-3185 (miRNAs w/seed GAAGAAG)
miR-8066 (miRNAs w/seed AAUGUGA)
DIO2
SPTLC2
miR-4716-5p (miRNAs w/seed CCAUGUU)
miR-1277-5p (miRNAs w/seed AAUAUAU)
miR-3121-5p (miRNAs w/seed CCUUUGC)
deoxycorticosterone acetate
NODAL
BTG2
asoprisnil
miR-3162-3p (miRNAs w/seed CCCUACC)
miR-4633-5p (miRNAs w/seed UAUGCCU)
miR-924 (miRNAs w/seed GAGUCUU)
11,12-epoxyeicosatrienoic acid
RND3
CSF1R
phenacetin
miR-6738-3p (miRNAs w/seed UUCUGCC)
miR-7153-3p (miRNAs w/seed ACCAUGG)
nitroglycerin
T 0070907
GPR132
caspase
miR-17-2-3p (and other miRNAs w/seed CUGCACU)
Ccl2
miR-4655-3p (miRNAs w/seed CCCUCGU)
miR-621 (miRNAs w/seed GCUAGCA)
miR-511-3p (miRNAs w/seed AUGUGUA)
miR-524-3p (and other miRNAs w/seed AAGGCGC)
CSHL1
miR-891b (miRNAs w/seed GCAACUU)
PIEZO1
FDX2
CYP2C8
CDCA2
proanthocyanidin derivative
NCF2
GRPR
mir-590
CALCR
lanatoside C
TMSB10/TMSB4X
FDXR
ARHGEF2
neopterin
tipifarnib
pentamidine
4-methylcatechol
chlorophyllin
pyocyanin
SKIL
miR-8056 (miRNAs w/seed GUGGAUU)
miR-1261 (miRNAs w/seed UGGAUAA)
miR-1244 (miRNAs w/seed AGUAGUU)
glucagon
miR-548ao-3p (miRNAs w/seed AAGACCG)
mir-182
METTL3
CIC
miR-6755-5p (miRNAs w/seed AGGGUAG)
thymoquinone
CTBP1
miR-4686 (miRNAs w/seed AUCUGCU)
miR-7974 (miRNAs w/seed GGCUGUG)
Fcgr3
Sod
PHB2
TNFRSF6B
7-nitroindazole
miR-6873-3p (miRNAs w/seed UCUCUCU)
N-Cadherin
immune complex
APBA3
CD33
MTBP
TSPO
carnosol
polaprezinc
FABP4
PRKAR2B
mir-145
miR-4499 (and other miRNAs w/seed AGACUGA)
miR-3659 (miRNAs w/seed GAGUGUU)
miR-3575 (and other miRNAs w/seed CUGCUGG)
miR-1263 (miRNAs w/seed UGGUACC)
LMX1B
dipyridamole
miR-4326 (miRNAs w/seed GUUCCUC)
SOX1
miR-4796-5p (miRNAs w/seed GUCUAUA)
miR-4255 (miRNAs w/seed AGUGUUC)
miR-3098-3p (and other miRNAs w/seed UCUGCUG)
neomycin
FOXD2-AS1
KT5720
KMT2A-AFF1

**4294 common elements in "Calu-3_SARS-CoV-2" and "Calu-3_SARS-CoV":**
lipopolysaccharide
IFNG
poly rI:rC-RNA
Interferon alpha
TNF
IFNA2
MAPK1
dexamethasone
STAT3
beta-estradiol
IRF7
IFNL1
resiquimod
STAT1
IL1B
NKX2-3
TGFB1
NFkB (complex)
PRL
IFNB1
IRF1
SB203580
OSM
TLR3
IL1RN
CD40LG
NR3C1
TP53
Ifnar
IRF3
ESR1
IFNAR1
RELA
Immunoglobulin
camptothecin
IFN Beta
CSF2
FOXO3
filgrastim
EIF2AK2
Salmonella enterica serotype abortus equi lipopolysaccharide
salmonella minnesota R595 lipopolysaccharides
CSF1
LY294002
calcitriol
IL6
IL4
tretinoin
SMARCA4
IL27
Ifn
F2
NFKBIA
KRAS
U0126
RC3H1
HGF
prostaglandin E2
TICAM1
PAF1
cycloheximide
IRF2
TCR
IL10
Ige
TLR4
Vegf
MAPK7
TRIM24
NFATC2
ERBB2
IRAK4
NFKB1
tetradecanoylphorbol acetate
lysophosphatidylcholine
ESR2
5-O-mycolyl-beta-araf-(1->2)-5-O-mycolyl-alpha-araf-(1->1')-glycerol
STAT6
PTEN
ETV6-RUNX1
SP1
LDL
DDX58
P38 MAPK
PDGF BB
hydrogen peroxide
PD98059
TREM1
IKBKB
APP
cholesterol
Pam3-Cys-Ser-Lys4
PML
TCF4
DUSP1
thapsigargin
TLR9
progesterone
FAS
IL1A
deferoxamine
paclitaxel
TP73
bromodeoxyuridine
PRKCD
CREB1
IL5
SAMSN1
bee venom
AGT
VCAN
IRF5
MYC
NUPR1
IFNAR2
IFNLR1
stallimycin
TNFSF11
fluticasone propionate
PGR
RNASEH2A
HRAS
LDLR
CD40
CDK19
IFNA1/IFNA13
IL15
MAVS
NR1H3
JUN
SP600125
TGM2
IL1
actinomycin D
STING1
STAT2
forskolin
TAB1
IL2
PI3K (complex)
erlotinib
FOS
oblimersen
GPER1
TLR7
2-(4-amino-1-isopropyl-1H-pyrazolo[3,4-d]pyrimidin-3-yl)-1H-indol-5-ol
RNY3
IL17A
CpG ODN 2006
ID2
ERK1/2
butyric acid
PNPT1
ADRB
trichostatin A
FOXO1
cisplatin
ID3
troglitazone
PI3K (family)
SOCS1

miR-10400-3p (and other miRNAs w/seed UGGGCUC)
VEGFB
EHMT2
miR-147 (and other miRNAs w/seed UGUGCGG)
miR-4666b (miRNAs w/seed UGCAUGU)
GSKJ4
MYCN
SPZ1
SMARCE1
miR-3622b-5p (miRNAs w/seed GGCAUGG)
miR-3177-3p (miRNAs w/seed GCACGGC)
miR-7151-3p (miRNAs w/seed UACAGGC)
histidinol
NET1
HDC
(±)-2-hydroxyoleic acid
MBTPS1
BHLHE41
miR-6732-5p (and other miRNAs w/seed AGGGGGU)
miR-634 (miRNAs w/seed ACCAGCA)
miR-6765-3p (miRNAs w/seed CACCUGG)
miR-148a-5p (and other miRNAs w/seed AAGUUCU)
miR-3615 (miRNAs w/seed CUCUCGG)
miR-4292 (and other miRNAs w/seed CCCUGGG)
miR-4254 (and other miRNAs w/seed CCUGGAG)
ESRRG
HSPA9
miR-4485-3p (miRNAs w/seed AACGGCC)
miR-4700-3p (miRNAs w/seed ACAGGAC)
ITGA9
apicidin
miR-18b-3p (miRNAs w/seed GCCCUAA)
CDH1
miR-6838-3p (miRNAs w/seed AGUCCUG)
ADRB2
miR-7108-3p (miRNAs w/seed CCCGCCC)
miR-1281 (miRNAs w/seed CGCCUCC)
miR-5104b-5p (and other miRNAs w/seed GGGCCAU)
CALR
miR-4433a-3p (miRNAs w/seed CAGGAGU)
telapristone acetate
PDGFRA
antimycin A
miR-4768-3p (and other miRNAs w/seed CAGGAGA)
miR-6798-5p (miRNAs w/seed CAGGGGG)
miR-3123 (miRNAs w/seed AGAGAAU)
HOXB5
KLB
IGKC
ATF5
largazole
PIM1
miR-1964-5p (and other miRNAs w/seed GCUGGAG)
hyperforin
miR-3150b-3p (and other miRNAs w/seed GAGGAGA)
miR-6802-5p (miRNAs w/seed UAGGUGG)
IGFBP2
miR-1279 (miRNAs w/seed CAUAUUG)
MMP12
DNMT3A
miR-2276-3p (miRNAs w/seed CUGCAAG)
miR-671-5p (miRNAs w/seed GGAAGCC)
AVP
miR-6840-3p (miRNAs w/seed CCCAGGA)
Fibrin
ALDH2
SULT1E1
miR-6717-5p (miRNAs w/seed GGCGAUG)
miR-7064-3p (and other miRNAs w/seed AGGGCCC)
miR-4293 (miRNAs w/seed AGCCUGA)
miR-6800-5p (miRNAs w/seed UAGGUGA)
miR-6791-3p (and other miRNAs w/seed GCCUCCU)
miR-4708-5p (miRNAs w/seed GAGAUGC)
choline fenofibrate
imipramine
IFT88
miR-509-3p (miRNAs w/seed GAUUGGU)
miR-4787-5p (and other miRNAs w/seed CGGGGGU)
SNAI2
miR-2114-3p (and other miRNAs w/seed GAGCCUC)
GPD1
miR-185-5p (and other miRNAs w/seed GGAGAGA)
miR-3146 (miRNAs w/seed AUGCUAG)
miR-760-3p (and other miRNAs w/seed GGCUCUG)
miR-4794 (and other miRNAs w/seed CUGGCUA)
gemfibrozil
miR-4749-3p (miRNAs w/seed GCCCCUC)
prostaglandin E1
triolein
BCAR4
APC (complex)
sphingomyelinase
ASCL2
SERPINC1
HMGCR
mir-320
CUL7
PPM1A
MLLT3
MED13L
retroinverso ERG inhibitory peptide 2
retroinverso ERG inhibitory peptide 1
RICTOR
miR-3690 (miRNAs w/seed CCUGGAC)
miR-3934-3p (miRNAs w/seed GCUCAGG)
TXNRD1
mir-210
CCN2
miR-3912-5p (miRNAs w/seed UGUCCAU)
miR-5580-5p (and other miRNAs w/seed GCUGGCU)
miR-3141 (miRNAs w/seed AGGGCGG)
carboplatin
miR-1202 (and other miRNAs w/seed UGCCAGC)
WNT7A
ACE
diosgenin
ITPR
nebivolol
PFKFB4
PDE8A
ZXDC
USE1
LINC00887
EHD2
CUEDC2
Eif2
nintedanib
danusertib
RAB7
EIF3I
crocin
RNF139
PLAGL2
VSNL1
CFL1
CSTB
HSPH1
MAD1L1
OSU-CG12
abrocitinib
methylamine
hoechst 33342
PTIO
ethyl linoleate
microcystin-LR
galanthamine
CX-6258
thymeleatoxin
SP2
N-ethyl-N-nitrosourea
24-hydroxycholesterol
miR-1266-5p (and other miRNAs w/seed CUCAGGG)
CDC42
HOXB13
ketoconazole
tannic acid
C9orf72
PBRM1
SLC25A13
miR-6976-5p (and other miRNAs w/seed AGGGAAG)
miR-4701-5p (and other miRNAs w/seed UGGCCAC)
miR-6799-5p (miRNAs w/seed GGGAGGU)
miR-6771-3p (miRNAs w/seed AAACCCC)
miR-3594-5p (and other miRNAs w/seed CCAGGGC)
PTPN6
CLEC11A
miR-4648 (miRNAs w/seed GUGGGAC)

icatibant
pimozide
pyruvaldehyde
PHB
acteoside
isoprenaline
miR-376c-3p (and other miRNAs w/seed ACAUAGA)
miR-152-5p (miRNAs w/seed GGUUCUG)
miR-301a-5p (and other miRNAs w/seed CUCUGAC)
miR-4452 (miRNAs w/seed UGAAUUC)
miR-4445-3p (and other miRNAs w/seed ACGGCAA)
miR-409-5p (and other miRNAs w/seed GGUUACC)
miR-671-3p (and other miRNAs w/seed CCGGUUC)
miR-450a-5p (and other miRNAs w/seed UUUGCGA)
UHRF2
miR-5089-3p (and other miRNAs w/seed UGCUACU)
HOXC6
NCSTN
ZIC2
mir-378
GUSB
RALBP1
ALOX15B
ERP29
RECK
IL12RB2
7,8-dihydro-7,8-dihydroxybenzo(a)pyrene 9,10-oxide
pentazocine
miR-3973 (miRNAs w/seed CAAAGUA)
miR-219b-5p (and other miRNAs w/seed GAUGUCC)
delta-12-prostaglandin J2
ATPase
edratide
beta-sitosterol
olmesartan
SMPD3
Gpcr
G6PC3
RSF1
PFN1
LILRB3
DGKA
2-cyclohexen-1-one
cimetidine
SD-282
PD 150606
ethacrynic acid
phenol derivative
orantinib
retinaldehyde
Hg2+
miR-433-3p (miRNAs w/seed UCAUGAU)
miR-6780a-3p (miRNAs w/seed UCCUCUG)
miR-4475 (miRNAs w/seed AAGGGAC)
miR-548b-3p (miRNAs w/seed AAGAACC)
mir-27
NRF1
miR-653-5p (miRNAs w/seed UGAAACA)
2-bromoethylamine
miR-543-3p (and other miRNAs w/seed AACAUUC)
Fgfr
NUMB
TEK
CCNH
EPHA2
miR-708-5p (and other miRNAs w/seed AGGAGCU)
miR-214-3p (and other miRNAs w/seed CAGCAGG)
miR-5007-5p (miRNAs w/seed AGAGUCU)
sorbitol
LPAR1
SAT1
deoxycorticosterone acetate/potassium chloride/sodium chloride
miR-6076 (and other miRNAs w/seed GCAUGAC)
L-ornithine
Gelonin
Max-Myc
METTL21C
TCHP
ZFAT
MTNR1A
linsitinib
GPR4
BATF2
MTNR1B
SH3GLB1
mir-630
lynestrenol
GABRG2
PGAM1
FGF5
zotiraciclib
parsatuzumab
TMPRSS4
telomestatin
oxazepam
teleocidins
polyinosinic acid
KB-R7785
17-epiestriol
miR-4767 (miRNAs w/seed GCGGGCG)
let-7d-3p (miRNAs w/seed UAUACGA)
miR-3569 (and other miRNAs w/seed GAGGACA)
miR-323b-3p (miRNAs w/seed CCAAUAC)
miR-1257 (miRNAs w/seed GUGAAUG)
miR-10b-3p (miRNAs w/seed CAGAUUC)
miR-1255b-2-3p (miRNAs w/seed ACCACUU)
gentamicin C
lapatinib/pazopanib
fumonisin B1
arecoline
miR-4711-5p (miRNAs w/seed GCAUCAG)
QKI
miR-139-5p (miRNAs w/seed CUACAGU)
miR-3614-5p (miRNAs w/seed CACUUGG)
miR-1295b-3p (miRNAs w/seed AUAGGCC)
ELF3
mir-221
TNFSF15
LTBP1
PSMD10
cyanocobalamin
miR-323-3p (and other miRNAs w/seed ACAUUAC)
SQ 22536
APC/APC2
NQO2
mir-503
NCR1
ERRFI1
allyl isothiocyanate
IC87114
midazolam
retinol acetate
THRAP3
HIRA
N-carbobenzyloxy-leucine-leucine-norvalinal
deguelin
miR-6826-5p (miRNAs w/seed CAAUAGG)
desmopressin
miR-1324 (miRNAs w/seed CAGACAG)
miR-1225-5p (miRNAs w/seed UGGGUAC)
2,3,4,7,8-pentachlorodibenzofuran
uranyl nitrate
miR-4509 (miRNAs w/seed CUAAAGG)
miR-517-5p (miRNAs w/seed CUCUAGA)
miR-6816-3p (miRNAs w/seed AAGGACC)
miR-1273c (miRNAs w/seed GCGACAA)
miR-500a-3p (miRNAs w/seed UGCACCU)
miR-4464 (and other miRNAs w/seed AGGUUUG)
mir-22
XBP1
KLF5
miR-425-5p (and other miRNAs w/seed AUGACAC)
PER1
mangiferin
miR-1245b-5p (and other miRNAs w/seed AGGCCUU)
miR-3073a-5p (and other miRNAs w/seed UGGUCAC)
BACH1
miR-487b-3p (miRNAs w/seed AUCGUAC)
BICC1
KCNK2
SNHG15
TRPM7
SOX18
AMBN
DNAJA3
FZD5
Ppp1cc

TNFSF10
SIRT1
E. coli serotype 0127B8 lipopolysaccharide
IRF9
CG
Tgf beta
SASH1
CHUK
CNOT7
AG490
C11orf95-RELA
etoposide
ECSIT
N-acetyl-L-cysteine
FADD
INSR
IKBKG
doxorubicin
YY1
ERK
RAF1
leukotriene D4
IgG
tributyrin
25-hydroxycholesterol
Ifn gamma
MYD88
epigallocatechin-gallate
HIF1A
Akt
GFI1
DOCK8
E. coli B5 lipopolysaccharide
AHR
REL
TCF3
chloropromazine
mycophenolic acid
F2RL1
PARP9
HNF4A
PTGER4
E. coli B4 lipopolysaccharide
decitabine
FSH
GNAQ
MAP3K7
SREBF2
PF4
4-hydroxytamoxifen
ZFP36
cigarette smoke
NFAT5
IRF8
methylselenic acid
EIF2AK3
ZBTB17
IFN type 1
ziritaxestat
C5
methylprednisolone
IL33
STAT5A
SMAD3
palmitic acid
GNA14
Creb
EGF
RARA
Mek
mir-183
JAK
ACKR2
SH3TC2
EGR1
AKT1
KLF2
TBK1
JUNB
MAPK9
STAT4
JAK1
D-glucose
genistein
SREBF1
cyclosporin A
RAS
ATF3
NRG1
IGF1
IL10RA
F7
IKZF1
EGFR
lactacystin
NOD2
DDIT3
MAPK14
glucocorticoid
glutamine
GAPDH
ATF4
MEOX2
ritonavir
FGF2
NPC1
IRF4
TRADD
PDX1
SCAP
atorvastatin
IFNE
PPARA
EP300
IFN alpha/beta
miR-130a-3p (and other miRNAs w/seed AGUGCAA)
cytokine
NLRX1
PTGS2
Pkc(s)
5-N-ethylcarboxamido adenosine
THPO
INSIG1
MAPK3
mir-21
l-asparaginase
TMPRSS2-ERG
GnRH-A
LMO2
Z-LLL-CHO
MAP2K5
IKBKE
reactive oxygen species
lysophosphatidylinositol
Ca2+
15-deoxy-delta-12,14 -PGJ 2
TP63
miR-3083-5p (and other miRNAs w/seed GGCUGGG)
MAP3K14
Lh
FOXO4
clopidogrel
medroxyprogesterone acetate
chloroquine
PIK3R1
peptidoglycan
miR-30c-5p (and other miRNAs w/seed GUAAACA)
IFNL4
SPI1
lysophosphatidic acid
KLF6
PRKAG3
Pka
EGR2
FOXM1
vorinostat
imiquimod
Jnk
CD3
ELK1
valproic acid
LDB1
platelet activating factor
SAFB
FOXL2
tunicamycin
gefitinib

miR-6789-5p (miRNAs w/seed UAGGGGC)
miR-4786-3p (miRNAs w/seed GAAGCCA)
miR-6809-5p (miRNAs w/seed GGCAAGG)
miR-4270 (and other miRNAs w/seed CAGGGAG)
betaine
miR-5010-3p (and other miRNAs w/seed UUUGUGU)
miR-196a-5p (and other miRNAs w/seed AGGUAGU)
miR-591 (miRNAs w/seed GACCAUG)
miR-6875-3p (miRNAs w/seed UUCUUCC)
miR-4774-3p (miRNAs w/seed UUGCCUA)
GATA6
DGCR8
miR-323-5p (and other miRNAs w/seed GGUGGUC)
miR-6788-3p (miRNAs w/seed UCGCCAC)
calpain
miR-4438 (miRNAs w/seed ACAGGCU)
trabectedin
miR-6755-3p (miRNAs w/seed GUUGUCA)
miR-6510-5p (miRNAs w/seed AGCAGGG)
miR-4279 (miRNAs w/seed UCUCCUC)
miR-7704 (miRNAs w/seed GGGGUCG)
miR-8070 (miRNAs w/seed UGUGAUU)
miR-4453 (and other miRNAs w/seed AGCUUGG)
miR-3097-5p (and other miRNAs w/seed ACAGGUG)
miR-577 (miRNAs w/seed AGAUAAA)
epoprostenol
GPNMB
4-nitroquinoline-1-oxide
miR-3934-5p (miRNAs w/seed CAGGUGU)
miR-7159-5p (miRNAs w/seed UCAACAA)
miR-653-3p (and other miRNAs w/seed UCACUGG)
miR-3975 (miRNAs w/seed GAGGCUA)
miR-670-5p (miRNAs w/seed UCCCUGA)
miR-4711-3p (miRNAs w/seed GUGUCUU)
RGS2
miR-526b-5p (miRNAs w/seed UCUUGAG)
mir-122
miR-615-5p (and other miRNAs w/seed GGGGUCC)
miR-6864-5p (miRNAs w/seed UGAAGGG)
miR-122-5p (miRNAs w/seed GGAGUGU)
pyrophosphate
SULT2B1
STC1
SOAT1
PCBP1
cholestyramine
R59949
GW 5074
miR-4638-5p (miRNAs w/seed CUCGGCU)
benzene
miR-7112-5p (miRNAs w/seed CGGGCAG)
chlorcyclizine
LAMTOR1
GADD45G
ITGA3
BAG3
ELN
beta-cyclodextrin
Stat5 dimer
Collagen type IV
SRA1
HDAC7
ODC1
nickel
miR-8080 (miRNAs w/seed AAGGACA)
MECOM
miR-659-5p (miRNAs w/seed GGACCUU)
KAT2A
miR-4443 (miRNAs w/seed UGGAGGC)
miR-566 (and other miRNAs w/seed GGCGCCU)
ADAM10
miR-128-1-5p (and other miRNAs w/seed GGGGCCG)
miR-1250-5p (miRNAs w/seed CGGUGCU)
miR-6082 (miRNAs w/seed AAUACGU)
miR-5090 (and other miRNAs w/seed CGGGGCA)
miR-29c-5p (miRNAs w/seed GACCGAU)
miR-4529-5p (miRNAs w/seed GGCCAUC)
miR-2278 (miRNAs w/seed AGAGCAG)
miR-378g (miRNAs w/seed CUGGGCU)
BSCL2
CLPP
PRMT1
histone deacetylase inhibitor
CBFB
captopril
UCP2
MAZ
DAXX
zearalenone
miR-370-5p (and other miRNAs w/seed AGGUCAC)
miR-299a-5p (and other miRNAs w/seed GGUUUAC)
miR-6069 (miRNAs w/seed GGCUAGG)
miR-6409 (and other miRNAs w/seed GCGGGUG)
miR-2054 (miRNAs w/seed UGUAAUA)
miR-891a-5p (miRNAs w/seed GCAACGA)
miR-181b-1-3p (and other miRNAs w/seed UCACUGA)
miR-4717-3p (miRNAs w/seed CACAUGG)
deferasirox
NOG
miR-3944-5p (miRNAs w/seed GUGCAGC)
miR-3128 (and other miRNAs w/seed CUGGCAA)
miR-6834-5p (miRNAs w/seed UGAGGGA)
miR-3605-3p (miRNAs w/seed CUCCGUG)
miR-6859-3p (miRNAs w/seed GACCCCC)
miR-4520-5p (miRNAs w/seed CUGCGUG)
miR-3613-5p (miRNAs w/seed GUUGUAC)
miR-3976 (miRNAs w/seed AUAGAGA)
IgG1
SPOP
CXCR3
CYP27A1
TERF2IP
alendronic acid
miR-4437 (miRNAs w/seed GGGCUCA)
UTP
NKX2-2-AS1
nilvadipine
EPCAM
ESRRA
RORC
RPS6KB1
miR-1900 (and other miRNAs w/seed GCCGCCC)
purvalanol A
4-aminopyrazolo(3,4-d)pyrimidine
prostaglandin E3
abciximab
Mitochondrial complex 1
SFTPA2
T-type Calcium Channel
Rbx1
FAM168A
UPK3A
SGF29
TRIM71
PHOSPHO1
ZNF385B
NCEH1
PACS2
Gm-Csf Receptor
ECD
soy isoflavones
OMG
dibromoacetic acid
LAMA3
mir-331
mir-105
GABARAP
CENPX
ZNF410
MUC16
DST
Hnrpfl
ING2
GALR2
CYP24A1
MED6
nitrosonifedipine
CRTAP
Bhlhe41
RPS4Y1
LPCAT1
ZFYVE16
IPP-204106N
Mia2
hydroxyl radical
semapimod
pyridoxal

enzastaurin
betaxolol
Z-IETD-FMK
zVAD
zofenopril
miR-6830-5p (miRNAs w/seed CAAGGAA)
miR-133a-5p (and other miRNAs w/seed CUGGUAA)
miR-3621 (miRNAs w/seed GCGGGUC)
miR-181c-3p (miRNAs w/seed ACCAUCG)
LCK
miR-5687 (miRNAs w/seed UAGAACG)
miR-6715b-3p (miRNAs w/seed UCAAACC)
miR-339-3p (miRNAs w/seed GAGCGCC)
miR-3137 (miRNAs w/seed CUGUAGC)
miR-329-3p (and other miRNAs w/seed ACACACC)
miR-933 (miRNAs w/seed GUGCGCA)
miR-526a-5p (and other miRNAs w/seed UCUAGAG)
prostaglandin A2
BMP10
RAPGEF1
STIM1
aroclor 1254
allyl sulfide
indole
miR-664b-5p (miRNAs w/seed GGGCUAA)
halofuginone
LOC105372576
miR-4999-5p (miRNAs w/seed GCUGUAU)
RUNX1-RUNX1T1
coal tar
METTL14
Kallikrein
CHRM3
ferric ammonium citrate
miR-6820-5p (miRNAs w/seed GCGGCAG)
HMGN1
titanium dioxide
apomorphine
PLA2G6
MAP3K5
coenzyme Q10
GLP-1-(7-34)-amide
wogonin
agmatine
selenomethylselenocysteine
miR-4704-3p (miRNAs w/seed CAGUCAC)
miR-4793-5p (miRNAs w/seed CAUCCUG)
miR-1206 (miRNAs w/seed GUUCAUG)
miR-6841-3p (miRNAs w/seed CCUUGCA)
miR-6508-5p (and other miRNAs w/seed CUAGAAA)
miR-103b (miRNAs w/seed CAUAGCC)
miR-4694-5p (miRNAs w/seed GGUGUUA)
miR-3192-3p (miRNAs w/seed UCUGAUC)
STK40
miR-5591-3p (miRNAs w/seed UACCCAU)
miR-6760-3p (miRNAs w/seed CACUGUC)
ZC3H12A
ABCG1
UPF1
miR-510-3p (miRNAs w/seed UUGAAAC)
miR-4682 (miRNAs w/seed CUGAGUU)
miR-1258 (miRNAs w/seed GUUAGGA)
miR-3194-3p (miRNAs w/seed GCUCUGC)
miR-4677-3p (miRNAs w/seed CUGUGAG)
miR-483-3p (miRNAs w/seed ACUCCUC)
doxifluridine
GIP
ARG1
NCAM1
FABP5
ECM1
ethyl pyruvate
isoliquiritigenin
dasatinib
MEF2
nitroarginine
miR-4697-3p (miRNAs w/seed GUCAGUG)
miR-6844 (miRNAs w/seed UCUUUGU)
Cbp/p300
TSPYL5
mir-26
SKI
JAK3
CYP1B1
miR-4321 (miRNAs w/seed UAGCGGU)
miR-6080 (miRNAs w/seed CUAGUGC)
miR-548s (miRNAs w/seed UGGCCAA)
CYP27B1
miR-7978 (miRNAs w/seed CUGGUGU)
miR-12194-3p (and other miRNAs w/seed CUGUGGG)
24(S),25-epoxycholesterol
Stat1 dimer
ADAM21
DUSP10
CCNA2
mir-296
P2RY4
TBXA2R
FKBP1A
GLP1R
NPAS2
KLRB1
CLEC2A
Eda
triacetyl-3-hydroxyphenyladenosine
PQQ cofactor
Ki16425
Boc-D-FMK
20alpha-hydroxycholesterol
miR-4677-5p (miRNAs w/seed UGUUCUU)
miR-692 (and other miRNAs w/seed UCUCUUU)
clobetasol propionate
highly active antiretroviral therapy
SLC7A2
STAR
CCAT1
PRAME
NACC1
CCNT1
AIM2
KU-60019
doxazosin
rhein
tetrathiomolybdate
miR-4424 (miRNAs w/seed GAGUUAA)
miR-645 (miRNAs w/seed CUAGGCU)
miR-143-5p (and other miRNAs w/seed GUGCAGU)
miR-336-3p (and other miRNAs w/seed CUGGAUU)
miR-1245b-3p (miRNAs w/seed CAGAUGA)
FOXP1
miR-193a-5p (miRNAs w/seed GGGUCUU)
miR-6883-3p (miRNAs w/seed UCCCUAU)
trapidil
ergosterol-5,8-peroxide
stigmasterol
monooleylphosphatidic acid
omalizumab
(diaminocyclohexane)(diacetato)(dichloro)platinum
AMOT
DUB
methysticin
yangonin
CISD1
PTPMT1
PUM1
KLF14
Ribosomal Subunit 80S
AMOTL1
vistusertib
MR1
PSMC5
CLCN3
CUL4A
SLC1A1
GW 6471
PUM2
LSS
TRERF1
RASSF7
NPHS2
ARHGEF11
HMGN2
IRX1
singlet oxygen
dichlorodiphenyl dichloroethylene
tempo
fomepizole

metribolone
CGAS
plicamycin
docosahexaenoic acid
PRDM1
CEBPA
MYOD1
NRAS
PPARG
wortmannin
triamcinolone acetonide
IL12 (complex)
simvastatin
anisomycin
fulvestrant
resveratrol
let-7a-5p (and other miRNAs w/seed GAGGUAG)
trans-hydroxytamoxifen
mir-96
ribavirin
SP3
bisindolylmaleimide I
ethanol
CEBPB
IL13
mir-8
NLRP12
CREBBP
bortezomib
tosedostat
MAP3K8
CHD1
RNA polymerase II
rosiglitazone
CpG oligonucleotide
emactuzumab
Map3k7
methotrexate
Tnf (family)
PRKAA1
AREG
VDR
CREM
PROM1
IGF2
SMAD4
OGA
ATP
interferon beta-1a
cerivastatin
26s Proteasome
CpG ODN 1668
USP18
TNFRSF1A
SATB1
BTK
homocysteine
TLR2
CCL5
EPAS1
CDKN1A
PGF
TLR7/8
laminaran
FN1
CSF3
CD24
CERK
SUMO3
TRAF2
ATP-gamma-S
cytarabine
SMPD1
Irgm1
Ro31-8220
IL3
tamoxifen
CD36
lovastatin
EZH2
miR-17-5p (and other miRNAs w/seed AAAGUGC)
miR-148a-3p (and other miRNAs w/seed CAGUGCA)
TAL1
AGN194204
IFIH1
BAPTA-AM
CFTR
raloxifene
nelfinavir
Sb202190
PRKCA
PELP1
edaravone
miR-24-3p (and other miRNAs w/seed GGCUCAG)
TWIST1
MTOR
bicuculline
miR-101-3p (and other miRNAs w/seed ACAGUAC)
PRDM16
INS
arsenite
PLAU
Ni2+
mifepristone
VEGFA
miR-519a-3p (and other miRNAs w/seed AAGUGCA)
RPSA
GW3965
WT1
miR-124-3p (and other miRNAs w/seed AAGGCAC)
5-fluorouracil
TGFA
miR-291a-3p (and other miRNAs w/seed AAGUGCU)
10E,12Z-octadecadienoic acid
KDM5B
SP110
IFNA4
EDN1
POR
RPS6KA5
ezetimibe
mezerein
Growth hormone
BCL3
PTX3
CAMP
pirinixic acid
Hdac
MAPK8
TFRC
miR-5624-3p (and other miRNAs w/seed UAAGGCA)
romidepsin
carrageenan
Insulin
gentamicin
EBI3
miR-762 (and other miRNAs w/seed GGGCUGG)
diphenyleneiodonium
Igm
TNFSF13B
IL18
estrogen
MYB
dextran sulfate
Alpha catenin
AS1842856
MAP2K1
BRCA1
nitric oxide
kainic acid
isoquercitrin
CDKN2A
WNT5A
collagenase
APOE
miR-19b-3p (and other miRNAs w/seed GUGCAAA)
IFNK
pitavastatin
ACE2
FCGR2A
sorafenib
CD38
okadaic acid
SQSTM1
CLDN7
DETA-NONOate
IL21

sarpogrelate
methiothepin
dilazep
CGP77675
butenafine
(S)-2-((6Z,15Z)-20- hydroxyicosa-6,15-dienamido)succinate
AGI-1095
tibolone
miR-6863 (miRNAs w/seed AGACGUG)
miR-619-3p (miRNAs w/seed ACCUGGA)
miR-5191 (miRNAs w/seed GGAUAGG)
miR-34b-3p (miRNAs w/seed AAUCACU)
inositol
MNK1/2
Cpla2
RAR-RXR
DGKE
RNF5
HYAL1
TAF9
dinaciclib
HAMP
ZBTB46
TYMS
GAS7
WSB1
WFS1
BRCA2
SLC37A4
SRSF6
SGCD
CDC27
N-lauroyl-L-phenylalanine
RFXANK
scoparone
NCGC00186528
U 50488H
atrasentan
Collagen type V
DIM-C-pPhCO2Me
phytol
L-homocysteine
miR-3665 (miRNAs w/seed GCAGGUG)
miR-92a-2-5p (miRNAs w/seed GGUGGGG)
miR-4710 (miRNAs w/seed GGUGAGG)
AIP
miR-6845-3p (and other miRNAs w/seed CUCUCCU)
miR-3074 (and other miRNAs w/seed AUAUCAG)
CIDEC
miR-6126 (miRNAs w/seed UGAAGGC)
miR-4734 (miRNAs w/seed CUGCGGG)
MECP2
perhexiline
WP1066
1,2-dimethylhydrazine
miR-564 (and other miRNAs w/seed GGCACGG)
miR-3678-3p (miRNAs w/seed UGCAGAG)
miR-27a-5p (miRNAs w/seed GGGCUUA)
miR-219a-1-3p (miRNAs w/seed GAGUUGA)
TSHZ3
miR-298-5p (and other miRNAs w/seed GCAGAGG)
miR-583 (miRNAs w/seed AAAGAGG)
miR-20b-3p (miRNAs w/seed CUGUAGU)
miR-3675-3p (miRNAs w/seed AUCUCUA)
miR-3977 (miRNAs w/seed UGCUUCA)
miR-6790-3p (miRNAs w/seed GACCUCG)
ZBTB7A
epinephrine
miR-513c-5p (and other miRNAs w/seed UCUCAAG)
miR-4422 (miRNAs w/seed AAAGCAU)
miR-4310 (and other miRNAs w/seed CAGCAUU)
L-cysteine
bazedoxifene
Agtr1b
SNHG7
NAALADL2
TPSAB1/TPSB2
CD80/CD86
tryptase
BCKDK
ABCD1
CTNND1
TPM3
RFXAP
PLCB1
PANDAR
GPR183
SCUBE3
NPC1L1
UACA
PPP1CA
MCAM
TKT
PDYN
ziprasidone
tyrphostin AG 127
miR-3177-5p (miRNAs w/seed GUGUACA)
ELOVL5
REN
SFN
miR-4433b-3p (miRNAs w/seed AGGAGUG)
HOTAIRM1
tauroursodeoxycholic acid
miR-106a-3p (and other miRNAs w/seed UGCAAUG)
miR-7158-3p (miRNAs w/seed UGAACUA)
miR-184 (and other miRNAs w/seed GGACGGA)
miR-4746-3p (miRNAs w/seed GCGGUGC)
miR-879-5p (and other miRNAs w/seed GAGGCUU)
miR-3126-5p (and other miRNAs w/seed GAGGGAC)
alvespimycin
HTATIP2
MGAT5
CETP
Ro41-5253
miR-1251-3p (miRNAs w/seed GCUUUGC)
miR-548aa (and other miRNAs w/seed AAAACCA)
miR-7044-3p (and other miRNAs w/seed UGCAGCC)
miR-6846-3p (miRNAs w/seed GACCCCU)
miR-320b (and other miRNAs w/seed AAAGCUG)
FOXA2
GAB2
CLN3
PAK1
bile acid
miR-1249-3p (and other miRNAs w/seed CGCCCUU)
CDK4
miR-2861 (and other miRNAs w/seed GGGCCUG)
miR-349 (and other miRNAs w/seed AGCCCUG)
candesartan
miR-194-3p (and other miRNAs w/seed CAGUGGG)
E2f
miR-4781-5p (miRNAs w/seed AGCGGGG)
miR-1248 (miRNAs w/seed CCUUCUU)
miR-3127-5p (miRNAs w/seed UCAGGGC)
miR-758-5p (miRNAs w/seed AUGGUUG)
USF2
mir-133
FASN
COLQ
HOXA7
miR-4796-3p (miRNAs w/seed AAAGUGG)
DANCR
RLN2
miR-4780 (miRNAs w/seed CCCUUGA)
CEACAM1
ENG
miR-6804-3p (miRNAs w/seed GCACCUG)
miR-5093 (miRNAs w/seed GGAAAUG)
FOLR1
miR-5113 (and other miRNAs w/seed CAGAGGA)
miR-6872-3p (miRNAs w/seed CCAUGCC)
miR-3661 (and other miRNAs w/seed GACCUGG)
TMSB4
RHOJ
ACTB
thioridazine
miR-6786-5p (miRNAs w/seed CGGUGGG)
GABPA
miR-6165 (miRNAs w/seed AGCAGGA)
miR-4522 (and other miRNAs w/seed GACUCUG)
miR-7113-3p (miRNAs w/seed CUCCCUG)
miR-2113 (miRNAs w/seed UUUGUGC)
miR-1183 (miRNAs w/seed ACUGUAG)
cholic acid
miR-6727-5p (miRNAs w/seed UCGGGGC)
SETD2
methylnitrosourea

BB-2116
cibacron blue F 3GA
DL-threo-dihydrosphingosine
phloretin
sodium iodide
SYUIQ-5
C66
L-JNK inhibitor I
TAPI
glutathione diethyl ester
Z-LEHD-FMK
muscarine
CH6953755
miR-382-5p (miRNAs w/seed AAGUUGU)
miR-5695 (miRNAs w/seed CUCCAAG)
SIM1
ETS2
miR-12204-5p (and other miRNAs w/seed UGAGUCU)
BMPER
PLD1
FOXC1
RACK1
GNL3
HEY1
vanillin
8-(4-chlorophenylthio)-guanosine-3', 5'-cyclic monophosphate
NMDA Receptor
TNFRSF13C
ITGAL
pomalidomide
methylphenidate
L-carnitine
IL7R
CUL4B
NEUROG1
folic acid
miR-574-3p (miRNAs w/seed ACGCUCA)
miR-19b-1-5p (and other miRNAs w/seed GUUUUGC)
miR-3200-3p (miRNAs w/seed ACCUUGC)
miR-4665-3p (miRNAs w/seed UCGGCCG)
miR-873-3p (miRNAs w/seed GAGACUG)
miR-6511a-3p (and other miRNAs w/seed CUCACCA)
Nuclear factor 1
Rhox5
DRD2
miR-1286 (miRNAs w/seed GCAGGAC)
miR-6516-5p (miRNAs w/seed UUGCAGU)
miR-614 (miRNAs w/seed AACGCCU)
miR-6853-3p (miRNAs w/seed GUUCAUU)
miR-4756-3p (miRNAs w/seed CAGAGAU)
telmisartan
miR-3920 (miRNAs w/seed CUGAUUA)
CGA
miR-224-3p (and other miRNAs w/seed AAAUGGU)
SAA1
miR-374b-5p (and other miRNAs w/seed UAUAAUA)
miR-298 (miRNAs w/seed GCAGAAG)
GPBAR1
Ppp2c
CTSG
CCL21
HIF3A
MED16
HBP1
BCAR1
LMX1A
UGCG
OPRM1
RV 538
isatoribine
DLL4
NCOR-LXR-Oxysterol-RXR-9 cis RA
miR-3152-3p (and other miRNAs w/seed GUGUUAG)
miR-4329 (miRNAs w/seed CUGAGAC)
Hsp90
mir-29
miR-6499-5p (miRNAs w/seed CGGGCGC)
miR-4712-3p (miRNAs w/seed AUGAGAG)
miR-1237-3p (and other miRNAs w/seed CCUUCUG)
miR-767-3p (miRNAs w/seed CUGCUCA)
HDAC2
miR-3160-3p (miRNAs w/seed GAGCUGA)
miR-507 (and other miRNAs w/seed UUUGCAC)
miR-6770-5p (miRNAs w/seed GAGAAGG)
PTPN2
KCNIP3
KLRC4-KLRK1/KLRK1
MUC4
EMD
vanadate
miR-1229-3p (miRNAs w/seed UCUCACC)
miR-1970 (and other miRNAs w/seed GUGUCAC)
Rb
miR-1243 (miRNAs w/seed ACUGGAU)
miR-454-5p (miRNAs w/seed CCCUAUC)
miR-7975 (miRNAs w/seed UCCUAGU)
miR-7162-3p (miRNAs w/seed CUGAGGU)
PTH1R
PRLR
15-epi-lipoxin A4
L-phenylalanine
ganglioside GM3
ganglioside GD1a
GCS-100
2,5-bis(5-hydroxymethyl-2-thienyl)furan
REL/RELA/RELB
ibudilast
CYTOR
GPR84
PPT1
kremezin
IL-2R
C-21
PNU282987
hydrogel
AZI2
LITAF
HDAC10
BAG6
CD8A
SLC25A4
PLCD1
CYSLTR1
DNM1L
EFEMP1
USP2
ARAF
DGKZ
RNF41
GHSR
MAP3K10
TBL1X
PLA2G5
Havcr1
PBX2
SLC19A1
CAPN2
NCR3
TBL1XR1
L-lactic acid
myxothiazol
tyrphostin
buserelin
dihematoporphyrin ether
ADORA2A
CD274
RUVBL1
hexamethylene bisacetamide
miR-5011-3p (and other miRNAs w/seed UGCAUGG)
miR-4699-5p (miRNAs w/seed GAAGAUU)
miR-6772-5p (miRNAs w/seed GGGUGUA)
miR-6831-3p (miRNAs w/seed GACUAAC)
miR-380-3p (and other miRNAs w/seed AUGUAGU)
miR-383-3p (miRNAs w/seed CAGCACU)
miR-320e (miRNAs w/seed AAGCUGG)
miR-7154-5p (miRNAs w/seed UCAUGAA)
miR-8053 (miRNAs w/seed GGCGAUU)
miR-1231 (miRNAs w/seed UGUCUGG)
RAG2
SPRY1
curdlan
tetracycline
PLA2G10
miR-194-5p (miRNAs w/seed GUAACAG)
SETDB1
GDF11
miR-7056-5p (and other miRNAs w/seed GUGGAGG)

aspirin
SAMHD1
TRAF6
NFYA
EGLN1
NR3C2
miR-3613-3p (miRNAs w/seed CAAAAAA)
lenalidomide
IL7
pyrrolidine dithiocarbamate
SPHK1
CCL2
miR-1283 (miRNAs w/seed CUACAAA)
curcumin
bexarotene
topotecan
Histone h4
HDL
MAPKAPK2
OSMR
NLRC5
PP2/AG1879 tyrosine kinase inhibitor
miR-27a-3p (and other miRNAs w/seed UCACAGU)
2-aminopurine
poly dA-dT
ALB
MET
Gsk3
miR-6756-5p (and other miRNAs w/seed GGGUGGG)
CTNNB1
FGF1
E2F4
trovafloxacin
3,3'-diindolylmethane
CXCL12
Sn50 peptide
miR-149-3p (and other miRNAs w/seed GGGAGGG)
DNASE2
JUND
estrogen receptor
POU5F1
miR-34a-5p (and other miRNAs w/seed GGCAGUG)
CASR
cyclic AMP
EGLN
vinblastine
Nr1h
Raf
IL24
NOD1
PS-1145
Go 6976
BAX
miR-6079 (miRNAs w/seed UGGAAGC)
miR-4716-3p (and other miRNAs w/seed AGGGGGA)
RASSF1
hyaluronic acid
SYVN1
nicotine
BIRC3
CCL3
MFSD2A
oleic acid
E. coli lipopolysaccharide
triptolide
miR-1252-5p (miRNAs w/seed GAAGGAA)
Pam3-Cys
SPP1
LIPE
SFTPA1
BCL6
EIF2AK4
JAG2
NDRG1
zymosan
isotretinoin
NS-398
miR-200b-3p (and other miRNAs w/seed AAUACUG)
sphingosine-1-phosphate
IKZF3
entinostat
ARHGAP21
D-galactosamine
DEPTOR
nitrofurantoin
IFNL3
S100A6
stearic acid
quercetin
SYK
PIN1
FOXP3
C3AR1
COL18A1
arsenic trioxide
BRAF
Tlr
TNFAIP3
H2AB3 (includes others)
ADRA1A
IL2RG
NR1H2
PTGER2
staurosporine
prednisolone
indomethacin
miR-16-5p (and other miRNAs w/seed AGCAGCA)
PDLIM2
SMAD7
miR-1284 (and other miRNAs w/seed CUAUACA)
GnRH analog
hydrocortisone
uric acid
miR-6828-5p (miRNAs w/seed GGAAGCA)
ATM
mitoxantrone
cocaine
miR-515-3p (and other miRNAs w/seed AGUGCCU)
miR-6795-5p (and other miRNAs w/seed GGGGGGA)
bucladesine
miR-4731-5p (miRNAs w/seed GCUGGGG)
nitroprusside
UCP1
paraquat
tyrphostin AG 1478
ERN1
TIFA
TAP1
C3
HTT
Histone h3
tacrolimus
KIT
12-(3-adamantan-1-yl-ureido) dodecanoic acid
PRKCE
HSPB1
miR-337-3p (miRNAs w/seed UCCUAUA)
TYROBP
KDM1A
5-azacytidine
SCARB1
TRAF3
EREG
zerumbone
Ikb
PRKAA2
Fibrinogen
TREX1
miR-548v (miRNAs w/seed GCUACAG)
mir-155
IFI16
thalidomide
metformin
let-7a-3p (and other miRNAs w/seed UAUACAA)
SOCS3
miR-29b-3p (and other miRNAs w/seed AGCACCA)
PTGS1
FASLG
RAC1
PEX5L
sulindac sulfide
Nfat (family)
niacinamide
LEP
VIP
MALP-2s
SU6656

phosphate
trichloroethylene
miR-3195 (miRNAs w/seed GCGCCGG)
miR-3928-5p (and other miRNAs w/seed GAAGCUC)
miR-5571-5p (miRNAs w/seed AAUUCUC)
miR-4294 (miRNAs w/seed GGAGUCU)
miR-556-3p (miRNAs w/seed UAUUACC)
miR-4432 (miRNAs w/seed AAGACUC)
miR-6776-5p (miRNAs w/seed CUGGGUG)
miR-3108-3p (and other miRNAs w/seed GUCUAGG)
miR-6827-5p (miRNAs w/seed GGGAGCC)
ATP6AP2
LINC01139
CAVIN1
cercosporin
ADCY7
HSP90AA1
MIR320
mir-185
SETD7
TEF
AFF1
SH2B1
2-hydroxy-1-naphthylaldehyde isonicotinoyl hydrazone
clomipramine
dydrogesterone
miR-802-5p (miRNAs w/seed CAGUAAC)
miR-4798-5p (miRNAs w/seed UCGGUAU)
zoledronic acid
miR-548d-3p (and other miRNAs w/seed AAAAACC)
CTCF
CASP3
SFRP4
miR-5100 (miRNAs w/seed UCAGAUC)
miR-1295b-5p (and other miRNAs w/seed ACCCAGA)
miR-378b (and other miRNAs w/seed UGGACUU)
miR-8084 (miRNAs w/seed AAUACUA)
miR-361-5p (miRNAs w/seed UAUCAGA)
Cobra Venom Factor
norethindrone acetate
CYP3A
TLR2/TLR4
MN1
peptide T
H6PD
CNGB3
HAS1
mir-302
Irs3
ATP2B2
ETV3
PRSS8
RYK
CFH
YWHAB
RNF17
TNFRSF14
HIVEP2
19,20-epoxydocosapentaenoic acid
SF1
Hrg
ZYX
SAR-20347
fenebrutinib
papaverine
ginsenoside Rg1
miR-6885-3p (miRNAs w/seed UUUGCUU)
miR-3691-3p (miRNAs w/seed CCAAGUC)
miR-6767-3p (miRNAs w/seed CACGUGC)
miR-4281 (miRNAs w/seed GGUCCCG)
miR-6868-5p (miRNAs w/seed CUGGCAG)
miR-2115-3p (miRNAs w/seed AUCAGAA)
miR-3668 (miRNAs w/seed AUGUAGA)
miR-1265 (miRNAs w/seed AGGAUGU)
E2F5
SOX6
miR-1228-5p (miRNAs w/seed UGGGCGG)
miR-532-3p (miRNAs w/seed CUCCCAC)
miR-6514-3p (and other miRNAs w/seed UGCCUGU)
miR-455-3p (miRNAs w/seed CAGUCCA)
miR-3135a (miRNAs w/seed GCCUAGG)
cyclic GMP
clarithromycin
estrone
ADP
(+)-catechin
IGFBP7
Betacatenin/TCF
CBX8
CPEB1
FCER1A
HOXB4
Smad2/3/4
SERTAD2
costunolide
perindopril
ginkgolide B
miR-492 (miRNAs w/seed GGACCUG)
G6PD
PPP1R13L
PIM2
CSNK2A1
miR-4537 (miRNAs w/seed GAGCCGA)
miR-3914 (miRNAs w/seed AGGAACC)
miR-4307 (miRNAs w/seed AUGUUUU)
miR-6811-3p (and other miRNAs w/seed GCCUGUG)
IHH
CD2
olanzapine
miR-708-3p (miRNAs w/seed AACUAGA)
HNRNPK
MYOG
THBS4
DMD
miR-3663-5p (miRNAs w/seed CUGGUCU)
miR-343 (and other miRNAs w/seed CUCCCUC)
miR-6782-5p (and other miRNAs w/seed AGGGGUG)
miR-6869-5p (miRNAs w/seed UGAGUAG)
miR-4481 (and other miRNAs w/seed GAGUGGG)
miR-516a-3p (and other miRNAs w/seed GCUUCCU)
miR-1972 (miRNAs w/seed CAGGCCA)
miR-99a-3p (and other miRNAs w/seed AAGCUCG)
miR-5197-5p (miRNAs w/seed AAUGGCA)
miR-4699-3p (miRNAs w/seed AUUUACU)
ZFPM1
miR-934 (miRNAs w/seed GUCUACU)
AHI1
ACLY
miR-619-5p (and other miRNAs w/seed CUGGGAU)
miR-3571 (and other miRNAs w/seed ACACACU)
verapamil
FOXC2
miR-3070-5p (and other miRNAs w/seed GCCCCUG)
miR-676-5p (miRNAs w/seed CUUCAAC)
miR-6777-5p (and other miRNAs w/seed CGGGGAG)
miR-3675-5p (miRNAs w/seed AUGGGGC)
MAFB
miR-4308 (and other miRNAs w/seed CCCUGGA)
miR-503-3p (and other miRNAs w/seed GAGUAUU)
miR-6861-5p (miRNAs w/seed CUGGGUA)
miR-4476 (and other miRNAs w/seed AGGAAGG)
miR-513a-3p (and other miRNAs w/seed AAAUUUC)
azathioprine
L-serine
Rp-8-Br-cAMPS
CERS2
CDCA7L
DUSP4
DDR2
OSTM1
PARP2
HLF
H3C14
COL17A1
NDP
I-BET-151
clonidine
lidocaine
ryanodine
sphingomyelin
miR-1233-3p (miRNAs w/seed GAGCCCU)
miR-3589 (and other miRNAs w/seed AGGAAAC)
miR-6765-5p (and other miRNAs w/seed UGAGGCG)
miR-605-5p (miRNAs w/seed AAAUCCC)
FUT8
EDN3
CBX3
cyclooxygenase
STAP2
KLF10
GAS5
AURKB
PKNOX1
acrolein
miR-3064-5p (and other miRNAs w/seed CUGGCUG)
TCF7
ZBTB7B
miR-4680-5p (miRNAs w/seed GAACUCU)
exisulind
Ginkgo biloba
FFAR4
SGPP1
TNFAIP8L2
elastase
PECAM1
APOA4
CBLB
NHLH2
GRIN1
HNRNPD
diacylglycerol
miR-3164 (and other miRNAs w/seed GUGACUU)
miR-132-5p (miRNAs w/seed CCGUGGC)
miR-4786-5p (miRNAs w/seed GAGACCA)
GNB1
miR-4688 (and other miRNAs w/seed AGGGGCA)
LIF
ionomycin
SHARPIN
CAT
mibolerone
SRC
PLK1
SMAD1
L-triiodothyronine
CD28
PTAFR
HOXA9
miR-4251 (miRNAs w/seed CUGAGAA)
ZBTB16
CREBZF
BMP6
miR-182-5p (and other miRNAs w/seed UUGGCAA)
SOX2
CCN1
ST3-Hel2A-2
JAK2
SNAI1
dinoprost
TERT
phorbol esters
R5020
miR-6509-3p (miRNAs w/seed UCCACUG)
molybdenum disulfide
calcimycin
miR-4727-3p (miRNAs w/seed UAGUGGG)
SS18
dihydrotestosterone
miR-208a-3p (and other miRNAs w/seed UAAGACG)
dalfampridine
methylnitronitrosoguanidine
UM101
PTPRJ
pimagedine
miR-125b-5p (and other miRNAs w/seed CCCUGAG)
PIK3CG
sirolimus
NCOA2
CITED2
methyl methanesulfonate
PROC
ADRA1B
ADRA1D
EPO
cepharanthine
ZBED2
PSMB9
ING4
MUC1
Ins1
PC-SPES
SIRT2
Cdk
VTN
miR-153-3p (miRNAs w/seed UGCAUAG)
mitomycin C
miR-221-3p (and other miRNAs w/seed GCUACAU)
AR
miR-4656 (and other miRNAs w/seed GGGCUGA)
IFNA10
IFNA21
IFNA5
IFNA7
IFNA14
IFNA6
MVP
2'3'-cyclic guanosine monophosphate-adenosine monophosphate
miR-137-3p (miRNAs w/seed UAUUGCU)
PLX5622
3M-001
GNB2
Pkg
salicylic acid
bleomycin
H-7
KLF3
TFAP2A
TAZ
ZMPSTE24
A2M
advanced glycation end-products
miR-432 (and other miRNAs w/seed CUUGGAG)
MPZ
3-deoxy-2-octulosonic acid(2)-lipid A
GNA15
leucine
PARP1
SIRT6
CXCR4
carbon monoxide
rifampin
S100A8
lipoteichoic acid
BMP7
MAP2K4
MEF2C
miR-199a-5p (and other miRNAs w/seed CCAGUGU)
OSCAR
miR-4747-5p (and other miRNAs w/seed GGGAAGG)
miR-128-3p (and other miRNAs w/seed CACAGUG)
L-methionine
NFKBIB
Am 580
miR-92a-3p (and other miRNAs w/seed AUUGCAC)
MAC
ANGPT2
TLR5
mevalonic acid
PRKD1
miR-365-3p (and other miRNAs w/seed AAUGCCC)
ITGB1
temozolomide
miR-605-3p (miRNAs w/seed GAAGGCA)
arachidonic acid
miR-4514 (and other miRNAs w/seed CAGGCAG)
silibinin
2-deoxyglucose
losartan potassium
LYN
SR 144528
Tcf7
PPARD
miR-6829-5p (miRNAs w/seed GGGCUGC)
CARD9
miR-302c-3p (and other miRNAs w/seed AGUGCUU)
RELB
caffeine
isobutylmethylxanthine
Pdgf (complex)
CEBPD
celecoxib
potassium chloride
ETS1
NFKB2
miR-221-5p (and other miRNAs w/seed CCUGGCA)
SN-38
HNRNPA2B1
TSC22D3
NOTCH1
ABT-737
ADRB3
SOCS6
PPP1R15A
rotenone
SB 216763
parthenolide
let-7c-1-3p (and other miRNAs w/seed UGUACAA)
IFNA8
BTC
IFNA16
VHL
IL22
TCF
miR-936 (miRNAs w/seed CAGUAGA)
OPA1
PAEP
HDAC3
Bay 11-7082
miR-6512-3p (and other miRNAs w/seed UCCAGCC)
TRIB3
sterol

PCDH11Y
epirubicin
miR-4280 (miRNAs w/seed AGUGUAG)
miR-4671-5p (miRNAs w/seed CCGAAGA)
miR-6825-3p (miRNAs w/seed CGCUGAC)
miR-6819-3p (and other miRNAs w/seed AGCCUCU)
PLCG1
NREP
HPX
IGFBP1
ACACB
IL2RB
PCYT2
phenytoin
myricetin
miR-874-3p (miRNAs w/seed UGCCCUG)
miR-548l (miRNAs w/seed AAAGUAU)
miR-3657 (and other miRNAs w/seed GUGUCCC)
CAB39L
SERPINE1
thioguanine
moxestrol
1-palmitoyl-2-arachidonoyl-phosphatidylcholine
benzo(a)pyrene 7,8-dihydrodiol
tolylfluanid
Esr1-Estrogen-Sp1
CLTRN
FREM1
PRMT7
LDLRAP1
ELOVL2
hyperoside
exendin(9-39) amide
2-(3-chlorophenylamino)phenylacetic acid
HSD17B14
EXOC3L1
SDF4
bis(4-hydroxycinnamoyl)methane
EIF4F
Eotaxin
ACAT1
Hspg
Inflammasome
CSNK1E
Pdi
COL3A1
EMP2
PURB
GSTA4
HAO1
CASP7
riociguat
SGCA
SGI 1776
DOK2
STK16
PPP1R1A
TOR2A
DSG1
RGS3
GC
PDZD2
Pkhd1
YWHAE
GPX4
BUB1B
proTAME
dinitrophenyl R group
AZD-1208
filipin
K ATP Channel
fosbretabulin
4-amino-6-hydrazino-7-beta-D-ribofuranosyl-7H-pyrrolo[2,3-d]-pyrimidine-5-carboxamide
TTI-101
MitoBloCK-6
5,10,15,20-tetrakis(4-sulfonatophenyl)porphyrinato iron (III)
N(1)-guanyl-1,7-diaminoheptane
NC9
actinomycin
bacitracin
glutathione ethyl ester
aluminum
L-alpha-hydroxyglutarate
miR-450a-1-3p (and other miRNAs w/seed UUGGGAA)
EOMES
miR-580-3p (miRNAs w/seed UGAGAAU)
miR-1264 (and other miRNAs w/seed AAGUCUU)
miR-6793-5p (miRNAs w/seed GUGGGUU)
TFE3
miR-6877-5p (miRNAs w/seed GGGCCGA)
miR-4423-3p (miRNAs w/seed UAGGCAC)
picrotoxin
IL17C
miR-219b-3p (miRNAs w/seed GAAUUGC)
miR-5196-3p (miRNAs w/seed CAUCCUC)
miR-4511 (miRNAs w/seed AAGAACU)
TFAP2C
miR-4746-5p (miRNAs w/seed CGGUCCC)
miR-6529-3p (and other miRNAs w/seed CUGUGCC)
miR-6843-3p (and other miRNAs w/seed UGGUCUC)
miR-23a-5p (and other miRNAs w/seed GGGUUCC)
FMR1
phosphatidylinositol
rh-endostatin
ISX
MRPS18B
ZNF652
SH2D5
AKIRIN2
CXCL17
EHT-1864
LAX1
sotrastaurin
Sfk
Collagen type III
PDGFD
idelalisib
PTPRB
DAB2
ENO1
RBX1
TDO2
CYP4F2
MAD2L2
CHGA
TNKS
ERBIN
NKX3-2
ITGB8
FOXN3
PDE4D
CB-5083
aminoglutethimide
zimelidine
butylidenephthalide
cyclosporin
geranylgeraniol
phosphatidic acid
noladin ether
S-allyl-L-cysteine
miR-6716-5p (miRNAs w/seed GGGAAUG)
miR-6770-3p (miRNAs w/seed UGGCGGC)
miR-6828-3p (miRNAs w/seed UCUGCUC)
miR-3110-5p (and other miRNAs w/seed UCUGCCU)
miR-6502-3p (miRNAs w/seed AGACCAU)
ANXA7
miR-6894-5p (and other miRNAs w/seed GGAGGAU)
miR-764 (miRNAs w/seed CAGGUGC)
miR-647 (miRNAs w/seed UGGCUGC)
miR-548q (miRNAs w/seed CUGGUGC)
miR-578 (and other miRNAs w/seed UUCUUGU)
miR-4486 (miRNAs w/seed CUGGGCG)
miR-1227-5p (miRNAs w/seed UGGGGCC)
HSD11B1
CR1L
miR-3649 (miRNAs w/seed GGGACCU)
miR-4428 (miRNAs w/seed AAGGAGA)
miR-6506-3p (miRNAs w/seed CGUAUCA)
miR-378a-3p (and other miRNAs w/seed CUGGACU)
miR-33-3p (and other miRNAs w/seed AAUGUUU)

mir-15
gemcitabine
miR-669c-3p (and other miRNAs w/seed ACACACA)
aldosterone
miR-635 (and other miRNAs w/seed CUUGGGC)
PPARGC1A
H89
NRG2
STK11
SRC (family)
eicosapentenoic acid
EIF4E
tiron
gossypol
C-miR146a
CBX5
MAP2K1/2
ciglitazone
fenretinide
E2F1
sulforafan
miR-493-5p (miRNAs w/seed UGUACAU)
aldesleukin
NfkB-RelA
COMMD1
piceatannol
tosylphenylalanyl chloromethyl ketone
CASP1
mir-25
IL12 (family)
pioglitazone
androgen
NAMPT
RIPK2
DACH1
LCN2
miR-486-3p (and other miRNAs w/seed GGGGCAG)
IL32
miR-5694 (miRNAs w/seed AGAUCAU)
RABL6
miR-503-5p (miRNAs w/seed AGCAGCG)
miR-3104-5p (and other miRNAs w/seed AGGGGGC)
ORMDL3
cis-urocanic acid
MAPK13
PTPN3
selumetinib
IRAK3
STAT5B
HDAC4
dimethyl sulfoxide
4-phenylbutyric acid
TXN
miR-6815-5p (and other miRNAs w/seed AGGUGGC)
3M-011
PDPK1
imipramine blue
Pdgfr
RPL22
PTGER1
bempedoic acid
DICER1
L-type Calcium Channel
BAP1
DPP-23
miR-3907 (miRNAs w/seed GGUGCUC)
Il3
nifedipine
HDL-cholesterol
XDH
ARRB2
NORAD
PLAUR
miR-520d-5p (and other miRNAs w/seed UACAAAG)
CD14
LTA
APOA1
IRS1
CXCL2
fingolimod
SERCA
CLEC12A
IL6R
Sos
DCAF1
XIAP
HMGB2
miR-328-5p (and other miRNAs w/seed GGGGGGC)
NGF
miR-5008-5p (miRNAs w/seed GAGGCCC)
MED1
vismodegib
BAK1
miR-3612 (and other miRNAs w/seed GGAGGCA)
ARNT
miR-6967-5p (and other miRNAs w/seed AGGGAGG)
diphtheria toxin
mir-146
IL12B
miR-12192-5p (and other miRNAs w/seed GUGGGGU)
miR-491-5p (and other miRNAs w/seed GUGGGGA)
EZR
ARRB1
PLAT
PD184352
tetrandrine
cannabidiol
miR-374c-5p (and other miRNAs w/seed UAAUACA)
miR-5006-5p (miRNAs w/seed UGCCAGG)
STAT
Calcineurin protein(s)
miR-1270 (and other miRNAs w/seed UGGAGAU)
HMOX1
tanespimycin
CDK9
miR-4649-3p (miRNAs w/seed CUGAGGC)
ATF6
1-methyl-4-phenyl-1,2,3,6-tetrahydropyridine
MIF
miR-6929-5p (and other miRNAs w/seed GGGAGGA)
ELAVL1
vancomycin
caffeic acid phenethyl ester
TIMP3
9,10-dimethyl-1,2-benzanthracene
F2R
PTH
SAV1
MSR1
LY6E
TP53COR1
lapatinib
miR-8065 (miRNAs w/seed GUAGGAA)
SP2509
miR-548an (miRNAs w/seed AAAGGCA)
PROCR
ozone
ISGF3
manumycin A
miR-145-5p (and other miRNAs w/seed UCCAGUU)
LRP6
YAP1
enterotoxin B
HDAC5
DYSF
KRT17
SMARCB1
fontolizumab
SAA
CARM1
pristane
IL36A
bafilomycin A1
formaldehyde
miR-5002-5p (miRNAs w/seed AUUUGGU)
IFNA17
ST14
nickel chloride
HDAC1
inosine
mir-181
clozapine
BTNL2
L-glutamic acid
ERBB3
dexamethasone phosphate
IL36B

IL37
miR-4520-3p (miRNAs w/seed UGGACAG)
N-Ac-Leu-Leu-norleucinal
PAX3
MRTFB
miR-6511a-5p (and other miRNAs w/seed AGGCAGA)
zVAD-FMK
n-3 fatty acids
GW9662
TGFBR2
Ap1
CLEC4G
GSK3B
let-7f-2-3p (and other miRNAs w/seed UAUACAG)
baicalein
MAP2K3
miR-4651 (and other miRNAs w/seed GGGGUGG)
Notch
etanercept
RNASEL
TIRAP
TNFSF14
vitamin K3
CXCL8
miR-203b-5p (and other miRNAs w/seed AGUGGUC)
EIF4EBP1
ADIPOQ
CD44
miR-1-3p (and other miRNAs w/seed GGAAUGU)
amphetamine
Lymphotoxin
miR-27b-5p (miRNAs w/seed GAGCUUA)
miR-4693-5p (miRNAs w/seed UACUGUG)
diethylstilbestrol
miR-5135 (and other miRNAs w/seed GGUCUAG)
HMGA1
FBXO42
TRG
UBA7
CTH
SR1078
TGFB2
5-hydroxytryptamine
miR-4436b-3p (and other miRNAs w/seed AGGGCAG)
Gcg
miR-185-3p (and other miRNAs w/seed GGGGCUG)
miR-3127-3p (and other miRNAs w/seed CCCCUUC)
GLI2
MAP2K6
FOSL1
lipid A
15(S)-HETE
PLC
HCAR2
PADI2
PLA2G4A
testosterone
LRPAP1
P2RY2
Fcer1
miR-6752-3p (miRNAs w/seed CCCUGCC)
SOX4
EIF2S1
MAP3K1
MMP1
miR-23a-3p (and other miRNAs w/seed UCACAUU)
miR-12183-5p (and other miRNAs w/seed CUUCUUC)
Ngf
miR-6721-5p (miRNAs w/seed GGGCAGG)
NPC2
TANK
NOS2
KDM3B
TRAF3IP2
prazosin
icilin
DDB2
miR-570-3p (miRNAs w/seed GAAAACA)
tetrachlorodibenzodioxin
HLX
GJA1
SOD2
miR-4668-5p (miRNAs w/seed GGGAAAA)
emodin
RETN
miR-141-3p (and other miRNAs w/seed AACACUG)
CNTF
zinc
BCR (complex)
miR-4433b-5p (miRNAs w/seed UGUCCCA)
miR-4802-3p (and other miRNAs w/seed ACAUGGA)
DDX5
SMARCA5
isoproterenol
miR-1273h-5p (and other miRNAs w/seed UGGGAGG)
HOTAIR
VitaminD3-VDR-RXR
PRC2
IFNGR1
IL12A
CRP
salinosporamide A
cuprizone
F3
ICAM1
DCN
TO-901317
miR-138-5p (miRNAs w/seed GCUGGUG)
WBP2
ANKRD42
patulin
poly(dG-dC)
TCIM
TRIM8
ADCYAP1R1
Immunoglobulin Lambda Light Chain
dacarbazine
manidipine
N-nitro-L-arginine methyl ester
NFE2L2
CCND1
PTPN11
(+)-MK-801
tert-butyl-hydroquinone
miR-144-3p (miRNAs w/seed ACAGUAU)
PPP2CA
TGFB3
TET2
KITLG
NfkB1-RelA
MST1
MTA3
PLK2
linolenic acid
miR-3082-3p (and other miRNAs w/seed ACAUGGC)
tempol
NR5A2
berberine
miR-1226-5p (and other miRNAs w/seed UGAGGGC)
ANXA2
SELP
daunorubicin
miR-3617-5p (and other miRNAs w/seed AAGACAU)
miR-664-1-5p (and other miRNAs w/seed UGGCUGG)
AMPK
JQ1
GATA1
WNT3A
miR-92a-1-5p (miRNAs w/seed GGUUGGG)
CBX7
PDCD4
TREM2
helenalin
peroxynitrite
L-alpha-lysophosphatidylcholine, palmitoyl
BPI
SENP6
ACOD1
PELI2
grepafloxacin
NTRK2
GLI1
CBL
miR-146a-5p (and other miRNAs w/seed GAGAACU)
miR-585-5p (miRNAs w/seed UAGCACA)
miR-4757-5p (and other miRNAs w/seed GGCCUCU)
miR-6852-5p (and other miRNAs w/seed CCUGGGG)

miR-22-3p (miRNAs w/seed AGCUGCC)
miR-133a-3p (and other miRNAs w/seed UUGGUCC)
THRB
N-acetylmuramyl-L-alanyl-D-isoglutamine
miR-646 (miRNAs w/seed AGCAGCU)
miR-5584-5p (miRNAs w/seed AGGGAAA)
Laminin (complex)
SELPLG
IRAK1
HBB
mir-30 (includes others)
miR-155-5p (miRNAs w/seed UAAUGCU)
17-alpha-ethinylestradiol
8-bromo-cAMP
RXRA
BRD4
melatonin
miR-4460 (miRNAs w/seed UAGUGGU)
JAK1/2
LY96
FCGR1A
tranylcypromine
caffeic acid
mannose
TF
TNFAIP6
BRD7
CX-5461
miR-4740-5p (miRNAs w/seed GGACUGA)
fluvastatin
NCOR2
MAP2K7
RNASEH2B
PARP14
B4GALT6
LGALS8
N4BP1
NFKBIE
CYBA
TPCA-1
miR-4650-3p (miRNAs w/seed GGUAGAA)
NGLY1
IL17F
diclofenac
FUS-DDIT3
naringenin
CTSS
BCAP31
PLK4
SL 327
N-cor
CFB
APEX1
proteasome inhibitor PSI
TRIM38
ELK4
RPS6KA4
SIRPA
miR-1909-3p (and other miRNAs w/seed GCAGGGG)
SCD
C5AR1
seocalcitol
KLF11
CIP2A
SRF
miR-628-3p (miRNAs w/seed CUAGUAA)
LGALS1
MEF2D
miR-4713-5p (miRNAs w/seed UCUCCCA)
RARB
ADM
MTPN
PRNP
miR-1285-3p (and other miRNAs w/seed CUGGGCA)
miR-6892-3p (miRNAs w/seed CCCUCUC)
miR-5586-5p (miRNAs w/seed AUCCAGC)
miR-4666a-5p (miRNAs w/seed UACAUGU)
Mapk
miR-5088-5p (miRNAs w/seed AGGGCUC)
BDNF
miR-4283 (miRNAs w/seed GGGGCUC)
miR-9-5p (and other miRNAs w/seed CUUUGGU)
kawain
IFNW1
ERVW-1
CDK5R1
TLR1
DUSP5
NCD-38
(1S,2R)-NCL-1
S-(2,3-bispalmitoyloxypropyl)-cysteine-GDPKHPKSF
LGALS3
miR-6748-3p (miRNAs w/seed CCUGUCC)
miR-21-5p (and other miRNAs w/seed AGCUUAU)
miR-6980-5p (and other miRNAs w/seed UGGGGGG)
NRIP1
miR-1468-3p (miRNAs w/seed GCAAAAU)
LEPR
let-7
NFYB
lithium chloride
levodopa
KCNE3
miR-3529-3p (miRNAs w/seed ACAACAA)
CpG ODN 1826
miR-3202 (miRNAs w/seed GGAAGGG)
PIK3CA
CRNDE
miR-4446-3p (miRNAs w/seed AGGGCUG)
S100A9
miR-483-3p (miRNAs w/seed CACUCCU)
ADCY
NFAT (complex)
PDGF-AA
resolvin D2
CYP7A1
THZ1
iron
N-(3-(aminomethyl)benzyl)acetamidine
rosuvastatin
CpG ODN 2216
NCF1
CCR1
JAK inhibitor I
hydroxypropyl-beta-cyclodextrir
miR-3063-5p (and other miRNAs w/seed GGAGAUC)
miR-4646-3p (miRNAs w/seed UUGUCCC)
miR-541-3p (and other miRNAs w/seed GGUGGGC)
Collagen(s)
FLT3
dimethylnitrosamine
miR-455-5p (and other miRNAs w/seed AUGUGCC)
miR-2682-5p (and other miRNAs w/seed AGGCAGU)
miR-4715-5p (miRNAs w/seed AGUUGGC)
miR-1294 (and other miRNAs w/seed GUGAGGU)
n-nitrosomethylbenzylamine
heme
ATF2
miR-505-3p (miRNAs w/seed GUCAACA)
leukotriene C4
FZD9
LBP
PPP2R5C
silicon dioxide
miR-874-5p (miRNAs w/seed GGCCCCA)
miR-34a-3p (miRNAs w/seed AAUCAGC)
miR-4763-5p (miRNAs w/seed GCCUGCC)
miR-202-3p (and other miRNAs w/seed GAGGUAU)
3-deazaneplanocin
dopamine
miR-2681-3p (miRNAs w/seed AUCAUGG)
miR-6132 (and other miRNAs w/seed GCAGGGC)
Pro-inflammatory Cytokine
EFNA1
MMP9
RNF31
TCF12
propranolol
miR-204-5p (and other miRNAs w/seed UCCCUUU)
miR-7851-3p (miRNAs w/seed ACCUGGG)
miR-6771-5p (miRNAs w/seed UCGGGAG)
miR-3616-3p (miRNAs w/seed GAGGGCA)
miR-135a-5p (and other miRNAs w/seed AUGGCUU)
miR-767 (and other miRNAs w/seed GCACCAU)
NR4A1
Esrra
miR-4742-5p (miRNAs w/seed CAGGCAA)

miR-4536-5p (miRNAs w/seed GUGGUAG)
miR-1909-5p (miRNAs w/seed GAGUGCC)
miR-5094 (miRNAs w/seed AUCAGUG)
miR-3174 (miRNAs w/seed AGUGAGU)
CASP8
carbonyl cyanide m-chlorophenyl hydrazone
miR-4670-3p (miRNAs w/seed GAAGUUA)
miR-6856-3p (miRNAs w/seed ACAGCCC)
2-amino-5-phosphonovaleric acid
PADI4
ARID3B
cyanidin 3-O-glucoside
IL26
FEM1A
COPA
BLM
DIABLO
AGI-1067
aplidine
palmitoyl-Cys((RS)-2,3-di(palmitoyloxy)-propyl)-Ala-Gly-OH
huperzine A
CTF1
CAMK4
alvocidib
miR-499-5p (and other miRNAs w/seed UAAGACU)
TSC2
docetaxel
adenosine
S-nitroso-N-acetyl-DL-penicillamine
IL1R1
BNIP3L
belnacasan
Ptprd
FH
PLCE1
pyruvic acid
noscapine
PRKAA
D-fructose
trametinib
PD 169316
PD 153035
miR-8088 (miRNAs w/seed CUCGGUA)
HMGB1
cobalt chloride
miR-4667-3p (miRNAs w/seed CCCUCCU)
miR-501-3p (and other miRNAs w/seed AUGCACC)
GH1
ARHGAP31
eplerenone
CTLA4
bromobenzene
miR-6888-5p (miRNAs w/seed AGGAGAU)
ACOX1
leukotriene B4
calcipotriene
BCL2L11
ibrutinib
amitriptyline
ceruletide
kaempferol
hemozoin
miR-3692-3p (miRNAs w/seed UUCCACA)
EPHB1
teriflunomide
APOL1
YOD1
ABCA4
Fcor
RLIM
PDE3B
IL1R2
CNB-001
J11-Cl
acriflavine
orlistat
rosmarinic acid
miR-125b-2-3p (and other miRNAs w/seed CACAAGU)
SB-431542
miR-627-3p (miRNAs w/seed CUUUUCU)
miR-1343-3p (and other miRNAs w/seed UCCUGGG)
CCR5
S-nitrosoglutathione
miR-766-3p (miRNAs w/seed CUCCAGC)
miR-6764-3p (and other miRNAs w/seed CUCUGGU)
TNFRSF8
miR-5107-3p (and other miRNAs w/seed AACCUGU)
PSEN1
miR-4755-5p (and other miRNAs w/seed UUCCCUU)
BMI1
black raspberry extract
mir-1
3-methyladenine
CP-55940
TWIST2
HDAC6
miR-423-5p (and other miRNAs w/seed GAGGGGC)
Pdgf Ab
IL17R
PI-103
VGLL3
CRBN
LYL1
MAPKAPK3
neuroprotectin D1
melphalan
6-hydroxydopamine
cholecalciferol
IDH1
miR-218-1-3p (miRNAs w/seed UGGUUCC)
miR-6846-5p (and other miRNAs w/seed GGGGGCU)
daporinad
4-(2-aminoethyl)benzenesulfonylfluoride
haloperidol
RBPJ
MACROH2A1
luteolin
miR-4788 (miRNAs w/seed UACGGAC)
KL
DPP4
PLG
miR-4655-5p (miRNAs w/seed ACCGGGG)
RHO
SIN3A
prostaglandin D2
indican
LLGL2
PRKDC
C1QA
SIAH2
fucoidin
allopurinol
lactosylceramide
BCOR
GADD45A
P2RX7
aphidicolin
miR-3691-5p (miRNAs w/seed GUGGAUG)
glutathione
miR-3144-5p (miRNAs w/seed GGGGACC)
miR-4691-5p (and other miRNAs w/seed UCCUCCA)
miR-609 (miRNAs w/seed GGGUGUU)
TICAM2
NOS1
immethridine
geranylgeranyl pyrophosphate
methyl-beta-cyclodextrin
miR-3151-5p (and other miRNAs w/seed GUGGGGC)
miR-876-3p (miRNAs w/seed GGUGGUU)
acetaminophen
ITGB2
NOS3
ABCA1
8-hydroxyguanine
CERS5
RNU1-1
FLT4
ZNF395
NLRP11
GMFG
STK38
XPA
coxsackievirus A21
EGTA acetoxymethyl ester
6,7-dinitroquinoxaline-2,3-dione
pyridoxamine
6-cyano-7-nitroquinoxaline-2,3-dione

PK11007
CV 3988
quisqualic acid
miR-892b (miRNAs w/seed ACUGGCU)
SBDS
TAC1
doxycycline
FCGR2B
miR-3551-5p (and other miRNAs w/seed CUUCACC)
miR-4755-3p (miRNAs w/seed GCCAGGC)
miR-6777-3p (miRNAs w/seed CCACUCU)
miR-4659a-3p (and other miRNAs w/seed UUCUUCU)
miR-4531 (miRNAs w/seed UGGAGAA)
miR-548at-5p (miRNAs w/seed AAAGUUA)
nystatin
lonafarnib
SELENOS
AIMP1
ZBTB33
mir-140
NRP1
IL18R1
dexmedetomidine
gamma-tocotrienol
CALC
miR-4789-3p (miRNAs w/seed ACACAUA)
MTORC1
E2F3
miR-4645-3p (miRNAs w/seed GACAGUA)
heparin
POU2F1
miR-4721 (and other miRNAs w/seed GAGGGCU)
AGER
miR-921 (miRNAs w/seed UAGUGAG)
HSF1
sodium arsenite
glucosamine
CX3CL1
miR-96-5p (and other miRNAs w/seed UUGGCAC)
miR-4259 (miRNAs w/seed AGUUGGG)
miR-203b-3p (miRNAs w/seed UGAACUG)
miR-4470 (miRNAs w/seed GGCAAAC)
PLAAT4
miR-4718 (miRNAs w/seed GCUGUAC)
Collagen type II
TNC
ANXA1
miR-4324 (miRNAs w/seed CCUGAGA)
imatinib
CAV1
miR-421-3p (and other miRNAs w/seed UCAACAG)
NCOA3
WNT1
SOD1
LRP1
miR-26a-5p (and other miRNAs w/seed UCAAGUA)
IDR-1002
miR-376a-3p (and other miRNAs w/seed UCAUAGA)
methapyrilene
miR-4645-5p (and other miRNAs w/seed CCAGGCA)
L-histidine
HCAR1
LECT2
SRT1720
PELI1
NELFB
LILRB4
ARF6
RNF216
BEX2
HNF1A-AS1
BIRC2
taxifolin
TBX5
miR-6881-5p (miRNAs w/seed GGGGUAA)
miR-1200 (miRNAs w/seed UCCUGAG)
miR-2110 (and other miRNAs w/seed UGGGGAA)
miR-4423-5p (miRNAs w/seed GUUGCCU)
Rock
methamphetamine
ursolic acid
ABL1
NOX4
Cdc42
CD300LF
p70 S6k
vemurafenib
MALT1
NCL
VCP
Mt1
WR 1065
benzo(a)pyrene
carbon tetrachloride
MEMO1
NEK7
GLRX
PI3
mir-873
ATG16L1
NELFA
RTKN
HA900
asbestos
capsazepine
PD 168393
RP 73401
1,1-bis(3'-indolyl)-1-(4-trifluoromethyl-phenyl)methane
JMF3086
miR-6849-3p (miRNAs w/seed CCAGCCU)
PRKCB
CRH
MYRF
GATA4
trans-cinnamaldehyde
IL17a dimer
Pde4
FGF4
BMP15
CXCR2
EIF4EBP2
desipramine
epicatechin gallate
evodiamine
ilomastat
miR-1247-3p (and other miRNAs w/seed GGGAACG)
RB1
GPS2
miR-148b-5p (and other miRNAs w/seed AGUUCUG)
miR-3170 (and other miRNAs w/seed UGGGGUU)
miR-95-5p (miRNAs w/seed CAAUAAA)
RHOA
colfosceril palmitate
Rsk
CC8490
MIB2
NPSR1
ATF6B
HECTD3
Carlr
ASCC1
TRIL
UFD1
MUC2
GCLC
NPLOC4
BICD2
VR23
atipamezole
IgG-opsonized ovalbumin
miR-6726-3p (miRNAs w/seed UCGCCCU)
PSMB11
miR-6825-5p (and other miRNAs w/seed GGGGAGG)
miR-500a-5p (miRNAs w/seed AAUCCUU)
diethylnitrosamine
RBM5
TXNIP
HNF1A
BMP2
INHBA
miR-4707-3p (miRNAs w/seed GCCCGCC)
arginine
ACVRL1
amiodarone
phorbol 12,13-dibutyrate
miR-3173-5p (and other miRNAs w/seed GCCCUGC)
EHF

daidzein
miR-1288-3p (miRNAs w/seed GGACUGC)
miR-4667-5p (and other miRNAs w/seed CUGGGGA)
miR-6501-5p (miRNAs w/seed GUUGCCA)
miR-142-3p (and other miRNAs w/seed GUAGUGU)
APC
miR-3162-5p (miRNAs w/seed UAGGGAG)
miR-7150 (miRNAs w/seed UGGCAGG)
CD86
miR-3183 (and other miRNAs w/seed CCUCUCU)
miR-1178-5p (miRNAs w/seed AGGGUCA)
miR-3199 (and other miRNAs w/seed GGGACUG)
miR-3609 (and other miRNAs w/seed AAAGUGA)
fenofibrate
miR-129-1-3p (and other miRNAs w/seed AGCCCUU)
miR-4779 (miRNAs w/seed AGGAGGG)
3M-002
miR-4671-3p (miRNAs w/seed UAGUGCA)
BCL2L1
birabresib
PTGES
miR-6748-5p (and other miRNAs w/seed GUGGGUG)
miR-1287-3p (and other miRNAs w/seed UCUAGCC)
miR-515-5p (and other miRNAs w/seed UCUCCAA)
miR-4269 (and other miRNAs w/seed CAGGCAC)
miR-6874-5p (and other miRNAs w/seed UGGAGCU)
Fc gamma receptor
ROR1
SMAD5
CORT
miR-4302 (miRNAs w/seed CAGUGUG)
lithium
LGR4
AGTR1
THBS1
benzyl isothiocyanate
4-methylnitrosoamino-1-(3-pyridinyl)-1-butanone
misoprostol
IL1RL2
HIF1AN
MAPKAP1
MAPK10
mevastatin
miR-4327 (miRNAs w/seed GCUUGCA)
miR-6516-3p (miRNAs w/seed UCAUGUA)
miR-3065-5p (and other miRNAs w/seed CAACAAA)
miR-875-5p (and other miRNAs w/seed AUACCUC)
fatty acid
AIRE
miR-6762-3p (miRNAs w/seed GGCUGCU)
HOXA10
miR-105-5p (and other miRNAs w/seed CAAAUGC)
miR-1913 (miRNAs w/seed CUGCCCC)
tazemetostat
RUNX1
miR-292b-5p (and other miRNAs w/seed CUCAAAA)
miR-6499-3p (miRNAs w/seed GCAGUGU)
2-(4-acetoxyphenyl)-2-chloro-N-methylethylamine
hydrochloric acid
1,25-dihydroxyvitamin D
TLR8
SERPINA1
Hbb-b2
RIPK1
NCOA1
RET
ZNF217
miR-6509-5p (miRNAs w/seed UUAGGUA)
ssRNA40
PHLPP1
S1PR1
sulfasalazine
miR-5187-5p (miRNAs w/seed GGGAUGA)
MAP3K3
STK4
NTRK1
Mt2
THZ2
HSPA5
miR-211-3p (miRNAs w/seed CAGGGAC)
infliximab
TSH
CD3E
miR-3473h-5p (and other miRNAs w/seed AGGGGCU)
lipoxin A4
FOSB
RAD21
WWTR1
RGS10
miR-4783-3p (miRNAs w/seed CCCGGUG)
IL11
HIC1
miR-6840-5p (miRNAs w/seed CCCCCGG)
miR-1305 (miRNAs w/seed UUUCAAC)
miR-132-3p (and other miRNAs w/seed AACAGUC)
mir-223
miR-4695-3p (miRNAs w/seed GAUCUCA)
miR-1207-5p (and other miRNAs w/seed GGCAGGG)
miR-4758-3p (miRNAs w/seed GCCCCAC)
miR-4484 (miRNAs w/seed AAAGGCG)
miR-4638-3p (and other miRNAs w/seed CUGGACA)
miR-3145-5p (miRNAs w/seed ACUCCAA)
MITF
miR-6878-3p (and other miRNAs w/seed UGGCCUC)
miR-199a-3p (and other miRNAs w/seed CAGUAGU)
KDM3A
ATF1
EFNA4
everolimus
miR-4733-3p (and other miRNAs w/seed CACCAGG)
miR-3686 (and other miRNAs w/seed UCUGUAA)
NR1H4
thyroid hormone
miR-1262 (and other miRNAs w/seed UGGGUGA)
tetrodotoxin
trypsin
CHRM1
FSTL1
CRY2
B4GALNT1
PIM3
CRY1
HSPA1A/HSPA1B
6-aminopyrazolopyrimidine derivative compound II
cytochalasin B
Complement
N,N-dimethylsphingosine
miR-2682-3p (and other miRNAs w/seed GCCUCUU)
miR-7854-3p (miRNAs w/seed GAGGUGA)
NPPB
MMP14
IL17RA
miR-3169 (miRNAs w/seed AGGACUG)
miR-7702 (miRNAs w/seed UUAGACU)
miR-6734-5p (and other miRNAs w/seed UGAGGGG)
GSK2816126
thioacetamide
vitamin E
miR-1237-5p (and other miRNAs w/seed GGGGGCG)
miR-6801-3p (and other miRNAs w/seed CCCCUGC)
panobinostat
TEAD3
IRF6
brefeldin A
miR-151-5p (and other miRNAs w/seed CGAGGAG)
MSC
EFNA2
miR-5682 (miRNAs w/seed UAGCACC)
miR-617 (miRNAs w/seed GACUUCC)
miR-4494 (miRNAs w/seed CAGACUG)
phytohemagglutinin
miR-4736 (and other miRNAs w/seed GGCAGGU)
miR-4712-5p (and other miRNAs w/seed CCAGUAC)
taurocholic acid
CLEC7A
ACTL6A
CFLAR
PD 123319
epoxomicin
trinitrobenzenesulfonic acid
cilostazol
miR-1226-3p (miRNAs w/seed CACCAGC)
alitretinoin
miR-296-5p (miRNAs w/seed GGGCCCC)
miR-3714 (miRNAs w/seed AAGGCAG)
O6-benzylguanine
ANKRD17

FAT1
rhesus theta-defensin 1
IL10RB
ANG
ITGA4
NCK1
IL9R
LUM
PRKRA
Igkv1-117
CXCL16
TRAF1
COP1
pubchem compound 11368987
mastoparan
poly(U)RNA
triamcinolone hexacetonide
coconut oil
bis(3',5')-cyclic diguanylic acid
FIRRE
iguratimod
REV1
TSC22D1
SDC4
PTBP3
MARCO
PROK1
ISG15
CLIC4
C4A/C4B
IL18RAP
Ptgs2os2
15-E2-isoketal modified phosphatidylethanolamine
AP20187
perifosine
ubiquinone 9
4-coumaric acid
2-mercaptoethanol
gliotoxin
RWJ 67657
borrelia burgdorferi strain B31 peptidoglycan
vinpocetine
miR-6729-3p (miRNAs w/seed CAUCCCC)
miR-1278 (miRNAs w/seed AGUACUG)
bevacizumab
Foxo
Integrin
Angiotensin II receptor type 1
FKBP4
PSMB8
LAMA5
GPI
IL1RAP
TNK1
Tnfsf9
TARBP2
NEDD4
KLK5
premarin
phenyl-N-tert-butylnitrone
cerulenin
D609
oxysterol
ADCYAP1
MAX
garcinol
miR-200a-5p (and other miRNAs w/seed AUCUUAC)
dibutyl phthalate
plumbagin
IL36G
TRPV4
GABA
EBF1
miR-3679-3p (miRNAs w/seed UUCCCCC)
miR-7855-5p (miRNAs w/seed UGGUGAG)
EFNA3
retinoid
miR-4305 (miRNAs w/seed CUAGACA)
miR-345-5p (and other miRNAs w/seed CUGACCC)
SOX11
TNFRSF18
GNAI3
ACKR1
SIN3B
TRIM21
calphostin C
miR-4719 (miRNAs w/seed CACAAAU)
miR-140-5p (and other miRNAs w/seed AGUGGUU)
miR-1306-5p (miRNAs w/seed CACCUCC)
N-formyl-Met-Leu-Phe
maneb
miR-3913-3p (miRNAs w/seed GACAUCA)
mir-204
CCL11
miR-147-5p (and other miRNAs w/seed GGAAACA)
PD 0325901
ZBED6
green tea polyphenol
bryostatin 1
bardoxolone
MRTFA
miR-709 (and other miRNAs w/seed GAGGCAG)
miR-3923 (miRNAs w/seed ACUAGUA)
miR-3103-5p (and other miRNAs w/seed GAGGGAG)
miR-1249-5p (and other miRNAs w/seed GGAGGGA)
rottlerin
miR-4524a-5p (and other miRNAs w/seed UAGCAGC)
miR-6758-5p (and other miRNAs w/seed AGAGAGG)
miR-6752-5p (and other miRNAs w/seed GGGGGUG)
miR-3090-3p (and other miRNAs w/seed CCCAGGU)
miR-11181-5p (and other miRNAs w/seed UCUGACC)
miR-548e-5p (miRNAs w/seed AAAAGCA)
miR-6786-3p (miRNAs w/seed GACGCCC)
miR-7976 (miRNAs w/seed GCCCUGA)
miR-5707 (miRNAs w/seed CGUUUGA)
IGF1R
miR-718 (miRNAs w/seed UUCCGCC)
miR-210-5p (and other miRNAs w/seed GCCACUG)
miR-545-3p (miRNAs w/seed CAGCAAA)
miR-4271 (and other miRNAs w/seed GGGGAAG)
miR-593-5p (and other miRNAs w/seed GGCACCA)
miR-6850-5p (miRNAs w/seed UGCGGAA)
LEF1
NSUN6
CGS 21680
miR-224-5p (miRNAs w/seed AAGUCAC)
Collagen type I (complex)
MSTN
LPIN1
KDM6B
VAV3
suramin
bardoxolone methyl
N(G)-monomethyl-D-arginine
FGFR1
miR-450b-3p (and other miRNAs w/seed UGGGAUC)
miR-6871-5p (miRNAs w/seed AUGGGAG)
miR-544-3p (and other miRNAs w/seed UUCUGCA)
ZNF106
miR-122b-3p (and other miRNAs w/seed AACACCA)
miR-155-3p (miRNAs w/seed UCCUACA)
ZAP70
delta-9-tetrahydrocannabinol
EGR3
ARID1A
IL11RA
miR-4722-5p (miRNAs w/seed GCAGGAG)
miR-670-3p (miRNAs w/seed UUCCUCA)
miR-519d-5p (miRNAs w/seed CUCCAAA)
1,4-bis[2-(3,5-dichloropyridyloxy)]benzene
CD3 group
salirasib
miR-4489 (miRNAs w/seed GGGGCUA)
POU2F2
miR-1953 (and other miRNAs w/seed GGGAAAG)
miR-1839-5p (and other miRNAs w/seed AGGUAGA)
miR-638 (miRNAs w/seed GGGAUCG)
miR-31-5p (and other miRNAs w/seed GGCAAGA)
MEG3
CDKN2B-AS1
TRAF3IP3
PSMB10
F10
LAMC1
ebselen
N-(3-oxododecanoyl)-homoserine lactone
picryl chloride

spermine
loxoribine
growth factor
manganese
MIR17HG
miR-4672 (miRNAs w/seed UACACAG)
RCAN1
SUPT16H
TAF4B
HSPD1
K-252
KN-62
BCL2
Hif1
miR-6737-5p (and other miRNAs w/seed UGGGGUG)
miR-4675 (and other miRNAs w/seed GGGCUGU)
miR-1302 (and other miRNAs w/seed UGGGACA)
miR-3154 (miRNAs w/seed AGAAGGG)
miR-5129-5p (and other miRNAs w/seed UGUGGGG)
resolvin D1
ITGAM
CYBB
PKD1
miR-196a-3p (miRNAs w/seed GGCAACA)
ASPSCR1-TFE3
miR-292-3p (and other miRNAs w/seed AGUGCCG)
miR-3158-3p (miRNAs w/seed AGGGCUU)
miR-181a-5p (and other miRNAs w/seed ACAUUCA)
histamine
miR-6862-5p (and other miRNAs w/seed GGGCAUG)
miR-125b-1-3p (miRNAs w/seed CGGGUUA)
miR-1914-3p (and other miRNAs w/seed GAGGGGU)
miR-1269a (and other miRNAs w/seed UGGACUG)
miR-1224-3p (miRNAs w/seed CCCACCU)
norepinephrine
MAPK12
ACVR1C
LILRB1
ketamine
miR-217-5p (and other miRNAs w/seed ACUGCAU)
miR-6746-5p (miRNAs w/seed CGGGAGA)
glycine
Ctbp
PKNOX2
LRBA
hemoglobin
DEF6
PLCG2
S1PR2
RPS6KA3
CXCL3
superoxide
sertraline
acadesine
ursodeoxycholic acid
PDGFB
lomustine
sulindac
mir-17
FCER1G
STAT5a/b
miR-542-3p (miRNAs w/seed GUGACAG)
miR-4456 (and other miRNAs w/seed CUGGUGG)
miR-219a-5p (and other miRNAs w/seed GAUUGUC)
miR-4640-3p (miRNAs w/seed ACCCCCU)
KLF4
miR-146a-3p (and other miRNAs w/seed CUCUGAA)
miR-4652-5p (miRNAs w/seed GGGGACU)
estriol
EFNA5
GPX1
HBEGF
N-[N-(3,5-difluorophenacetyl-L-Ala)]-S-phenylglycine t-butyl ester
miR-4660 (miRNAs w/seed GCAGCUC)
miR-2355-5p (miRNAs w/seed UCCCCAG)
alefacept
pyridaben
FOSL2
JAG1
colchicine
BSG
miR-181a-2-3p (and other miRNAs w/seed CCACUGA)
ATG7
TBX2
Y 27632
miR-1275 (and other miRNAs w/seed UGGGGGA)
miR-12201-5p (and other miRNAs w/seed GGAAGGA)
miR-6733-3p (and other miRNAs w/seed CAGUGUC)
FEV
miR-6720-3p (miRNAs w/seed GCGCCUG)
miR-183-5p (miRNAs w/seed AUGGCAC)
miR-4493 (miRNAs w/seed GAAGGCC)
TNFRSF1B
GLI3
ID1
TCOF1
ITGB3
miR-205-5p (and other miRNAs w/seed CCUUCAU)
miR-5003-5p (miRNAs w/seed CACAACA)
miR-494-3p (miRNAs w/seed GAAACAU)
miR-3606-5p (miRNAs w/seed UAGUGAA)
miR-8083 (miRNAs w/seed AGGACUU)
miR-4705 (and other miRNAs w/seed CAAUCAC)
ascorbic acid
PPP1R15B
SNHG20
Traj18
WWP2
IL18BP
Ncoa6
S100A7
Z-DEVD-FMK
mimosine
nandrolone
puromycin aminonucleoside
miR-495-3p (and other miRNAs w/seed AACAAAC)
miR-367-5p (miRNAs w/seed CUGUUGC)
miR-2116-3p (miRNAs w/seed CUCCCAU)
8-bromoguanosine 3',5'-cyclic monophosphate
rolipram
PML-RARA
exenatide
miR-1973 (miRNAs w/seed CCGUGCA)
miR-4527 (and other miRNAs w/seed GGUCUGC)
miR-3198 (and other miRNAs w/seed UGGAGUC)
miR-3689d (and other miRNAs w/seed GGAGGUG)
miR-384-3p (and other miRNAs w/seed UUCCUAG)
miR-4684-5p (miRNAs w/seed UCUCUAC)
miR-4633-3p (and other miRNAs w/seed GGAGCUA)
miR-4778-5p (miRNAs w/seed AUUCUGU)
mir-148
DTX1
acetylcholine
ceramide
TEAD4
ETV5
miR-135a-1-3p (and other miRNAs w/seed AUAGGGA)
STUB1
vitamin D
miR-4495 (miRNAs w/seed AUGUAAA)
miR-3926 (miRNAs w/seed GGCCAAA)
RBL1
eritoran
beta-glucan
1-palmitoyl-2-oleoylglycero-3-phosphoglycerol
styrene
MARCHF3
SPINDOC
TRIM58
sGC
Tlr12
ERN2
FGA
APAF1
SPIN1
ARL5B
TNFAIP8
PLAA
CC-122
polyinosine-polycytidylic acid/polyethylenimine formulation
pubchem compound 11483544
sulfamethoxazole/trimethoprim
tranexamic acid
roflumilast
paxilline
sappanone A
acetyl-11-keto-beta-boswellic acid

miR-558 (and other miRNAs w/seed GAGCUGC)
chenodeoxycholic acid
TGFBR1
PP1
eflornithine
galactosylceramide-alpha
SIRT3
miR-4529-3p (miRNAs w/seed UUGGACU)
MASTL
deoxycholate
valsartan
miR-3124-3p (and other miRNAs w/seed CUUUCCU)
miR-499-3p (and other miRNAs w/seed ACAUCAC)
miR-4708-3p (miRNAs w/seed GCAAGGC)
CNR1
miR-377-3p (miRNAs w/seed UCACACA)
TEAD1
kanamycin A
miR-582-3p (miRNAs w/seed AACUGGU)
zidovudine
NADPH oxidase
SGPL1
TFPI2
Sch-23390
lipoarabinomannan
diallyl trisulfide
miR-6513-5p (miRNAs w/seed UUGGGAU)
miR-4425 (miRNAs w/seed GUUGGGA)
miR-5193 (miRNAs w/seed CCUCCUC)
miR-190a-5p (and other miRNAs w/seed GAUAUGU)
CST5
miR-516b-5p (miRNAs w/seed UCUGGAG)
fingolimod phosphate
Cyp4a14
SENP7
Tlr13
S100a7a
HS6ST1
FALEC
LPA
ATF7
TAPBP
RBCK1
teasaponin
Duxbl1 (includes others)
ULBP1
raclopride
vadimezan
mechlorethamine
domoic acid
latrunculin A
magnesium sulfate
levofloxacin
alpha-tocopherol succinate
TBP
monophosphoryl lipid A
amphotericin B
cetuximab
Nos
1'-acetoxychavicol acetate
IFNL2
FOXG1
48s
AZD8055
SMC3
CAPN3
BDKRB2
RGS1
lauric acid
cobalt
ITCH
linsidomine
irbesartan
hymecromone
miR-548av-3p (miRNAs w/seed AAACUGC)
miR-587 (miRNAs w/seed UUCCAUA)
Srebp
trastuzumab
ZBP1
TMBIM6
RARRES2
MST1R
ACTN4
thiazolidinedione
idarubicin
vitamin K2
FGF7
miR-6901-5p (miRNAs w/seed GCAGGAA)
morphine
miR-2117 (miRNAs w/seed GUUCUCU)
miR-140-3p (and other miRNAs w/seed ACCACAG)
miR-548au-3p (miRNAs w/seed GGCAGUU)
miR-1268a (and other miRNAs w/seed GGGCGUG)
miR-513b-3p (miRNAs w/seed AAUGUCA)
xanthohumol
PIAS1
SERPINF1
miR-361-3p (miRNAs w/seed CCCCCAG)
CCN5
PEBP1
pentoxifylline
miR-3171 (miRNAs w/seed GAUGUAU)
miR-3684 (miRNAs w/seed UAGACCU)
RBL2
miR-4753-5p (miRNAs w/seed AAGGCCA)
GATA3
miR-6515-3p (miRNAs w/seed CUCUUCA)
miR-18a-3p (and other miRNAs w/seed CUGCCCU)
Mir122a,b
ADA
TAF6
phorbol 12,13-didecanoate
miR-325-5p (and other miRNAs w/seed CUAGUAG)
miR-450b-3p (and other miRNAs w/seed UUGGGGA)
TNFSF12
miR-3585-5p (and other miRNAs w/seed UCACAAG)
miR-4267 (miRNAs w/seed CCAGCUC)
miR-4421 (and other miRNAs w/seed CCUGUCU)
miR-4679 (miRNAs w/seed CUGUGAU)
miR-4501 (and other miRNAs w/seed AUGUGAC)
miR-122-3p (miRNAs w/seed ACGCCAU)
miR-4472 (and other miRNAs w/seed GUGGGGG)
miR-6810-5p (and other miRNAs w/seed UGGGGAC)
miR-466 (miRNAs w/seed UACACAU)
BACH2
PPP3R1
N-methyl-D-aspartate
miR-6510-3p (miRNAs w/seed ACCGACU)
miR-187-5p (miRNAs w/seed GCUACAA)
LMNA
miR-4303 (miRNAs w/seed UCUGAGC)
miR-548ag (and other miRNAs w/seed AAGGUAA)
miR-4666a-3p (miRNAs w/seed AUACAAU)
AICAR
CISH
Fgf
ALOX15
MARK2
UBE2I
MKNK1
miR-5579-3p (miRNAs w/seed UAGCUUA)
miR-10394-5p (and other miRNAs w/seed CUGCAGG)
risperidone
IPMK
vasoactive intestinal peptide
HOXA13
UCN
HOXA5
oxazolone
bisindolylmaleimide iv
SB 290157
miR-669d-5p (and other miRNAs w/seed CUUGUGU)
NKX2-1
miR-133a-5p (miRNAs w/seed GCUGGUA)
miR-6808-5p (and other miRNAs w/seed AGGCAGG)
miR-497-3p (and other miRNAs w/seed CAAACCA)
GDF2
nocodazole
miR-4714-5p (and other miRNAs w/seed ACUCUGA)
MEX3A
MMP2
ADORA2B
steroid
miR-431-5p (and other miRNAs w/seed GUCUUGC)
SPDEF
fenamic acid
herbimycin

quinolinic acid
miR-571 (miRNAs w/seed GAGUUGG)
fasudil
beta-carotene
4-hydroxynonenal
PD173074
EWSR1-FLI1
miR-3688-5p (miRNAs w/seed GUGGCAA)
FLZ
ALOX12
RB1CC1
PIK3R2
CLCA2
OGG1
C9
KRIT1
miR-5112 (and other miRNAs w/seed AGCUCAG)
ONECUT1
miR-6861-3p (miRNAs w/seed GGACCUC)
miR-4649-5p (and other miRNAs w/seed GGGCGAG)
miR-202-5p (miRNAs w/seed UCCUAUG)
miR-625-5p (and other miRNAs w/seed GGGGGAA)
puromycin
dacinostat
CDK1
mir-192
STAU1
NAB2
pevonedistat
Usp17la (includes others)
ammonium chloride
elovanoid N32
salvin
napabucasin
sangivamycin
lopinavir
PDK
MLN8054
INTS11
C1QTNF1
SPON2
ZNF451
EDEM1
SPATA2
ZNF300
potassium channel
NAA30
KLHL21
PTPN23
satratoxin G
DHX15
ELL
GNLY
YARS1
HSPB3
CRT-0066101
RAB10
DLST
TRAF7
GFRA2
IL36RN
KPNB1
SNAPIN
CDK5R2
INHBC
HES6
ERC1
DHCR7
8-butyl-10-[(3-methoxy-5-pyrrol-2-ylidenepyrrol-2-ylidene)methyl]-11-azabicyclo[7.2.1]dodeca-1(12),9-diene
EXOC7
PTPN12
HERC5
iso[4]levuglandin E2
14-oxoDHA
SKF-83959
PX-12
trimethyloxamine
gabexate
LMK-235
oenothein B
mycophenolate mofetil
magnesium chloride
nickel subsulfide
senexin B
methoxyluteolin
5-phenylvaleric acid
ODN-BW006
3,7-dimethyl-1-propargylxanthine
titanium
pegylated leptin antagonist
2-amino-3-phosphonopropionic acid
oleandrin
miR-6795-3p (miRNAs w/seed CCCCUCG)
miR-6882-3p (miRNAs w/seed GCUGCCU)
colistin
epothilone B
fluoride
DPH5
PFKFB3
ZNF148
H1-2
EPHX2
CDK6
TNFRSF12A
VIPR1
IRAK2
MAML1
BCL10
101.10 peptide
cyclopiazonic acid
cystamine
erythromycin
miR-2277-3p (miRNAs w/seed GACAGCG)
miR-6894-3p (miRNAs w/seed UGCCUGC)
GRP
ATG5
B2M
SND1
dicarbethoxydihydrocollidine
deoxynivalenol
carbenoxolone
fludarabine
miR-6857-5p (miRNAs w/seed UGGGGAU)
miR-5582-5p (miRNAs w/seed AGGCACA)
miR-629-3p (miRNAs w/seed UUCUCCC)
miR-6835-5p (and other miRNAs w/seed GGGGGUA)
miR-122b-5p (miRNAs w/seed UUAGUGU)
miR-1911-5p (miRNAs w/seed GAGUACC)
ZEB1
miR-4642 (miRNAs w/seed UGGCAUC)
miR-6134 (miRNAs w/seed GAGGUGG)
miR-5092 (miRNAs w/seed AUCCACG)
PTPN1
miR-452-5p (and other miRNAs w/seed ACUGUUU)
DNMT3B
TYK2
EWSR1
INHA
TCF7L2
ITGA5
miR-18a-5p (and other miRNAs w/seed AAGGUGC)
miR-188-3p (miRNAs w/seed UCCCACA)
miR-6800-3p (miRNAs w/seed ACCUCUC)
CYLD
TFAP4
SERPINE2
ELANE
U73122
miR-6783-5p (miRNAs w/seed AGGGGAA)
minocycline
corticosterone
CLOCK
miR-3654 (miRNAs w/seed ACUGGAC)
miR-6888-3p (and other miRNAs w/seed UCUGUCU)
SLPI
mir-214
UCN-01
miR-1291 (and other miRNAs w/seed GGCCCUG)
miR-540-3p (and other miRNAs w/seed GGUCAGA)
miR-3936 (miRNAs w/seed AAGGGGU)
miR-3175 (miRNAs w/seed GGGGAGA)
miR-744-3p (miRNAs w/seed UGUUGCC)
miR-6742-3p (miRNAs w/seed CCUGGGU)
miR-518c-5p (miRNAs w/seed CUCUGGA)
miR-1184 (miRNAs w/seed CUGCAGC)
CHD4
phenylbutazone

cadmium chloride
capsaicin
thioctic acid
FFAR3
miR-3611 (miRNAs w/seed UGUGAAG)
miR-600 (miRNAs w/seed CUUACAG)
sphingosylphosphocholine
16,16-dimethylprostaglandin E2
Pde
Ck2
C8
CpG ODN 1585
cytidylyl-3'-5'-guanosine
BLVRA
salmonella typhimurium lipopolysaccharide
HUWE1
pictilisib
PRTN3
CD160
NEK6
ANPEP
C7
NELFE
cyclic guanosine monophosphate-adenosine monophosphate
raltitrexed
prodigiosin
ketorolac
BQ 123
hexamethoxyflavone
arachidic acid
miR-7155-5p (miRNAs w/seed CUGGGGU)
miR-6813-3p (miRNAs w/seed ACCUUGG)
NFATC3
FLT3LG
miR-1260a (and other miRNAs w/seed UCCCACC)
ERBB4
MOG
FGF10
miR-8064 (miRNAs w/seed GCACACU)
miR-1301-3p (and other miRNAs w/seed UGCAGCU)
miR-4540 (miRNAs w/seed UAGUCCU)
miR-5004-3p (miRNAs w/seed UUGGAUU)
miR-10a-5p (and other miRNAs w/seed ACCCUGU)
miR-3065-3p (miRNAs w/seed CAGCACC)
miR-6822-3p (miRNAs w/seed GGCUCUA)
miR-490-5p (miRNAs w/seed CAUGGAU)
mir-135
cyclophosphamide
miR-873-5p (and other miRNAs w/seed CAGGAAC)
Ras homolog
Aldose Reductase
SIGIRR
PARG
MED12
SP100
IDO1
RNASE1
CCL4
ADORA3
2-mercaptoacetate
tranilast
W7
elovanoid N34
butaprost
miR-3945 (miRNAs w/seed GGGCAUA)
fluocinolone acetonide
fish oils
KAT2B
hydroxyurea
triamterene
miR-4759 (miRNAs w/seed AGGACUA)
miR-2052 (miRNAs w/seed GUUUUGA)
FOXA1
miR-101b-3p (miRNAs w/seed UACAGUA)
miR-4480 (miRNAs w/seed GCCAAGU)
pexidartinib
miR-6895-3p (miRNAs w/seed GUCUCUC)
let-7a-2-3p (and other miRNAs w/seed UGUACAG)
IKK (complex)
TERC
ING1
nicotinic acid
RAC2
PCGF2
histone deacetylase
miR-6078 (miRNAs w/seed CGCCUGA)
geldanamycin
miR-943 (miRNAs w/seed UGACUGU)
miR-3925-3p (miRNAs w/seed CUCCAGU)
psychosine
3'-O-(4-benzoyl)benzoyladenosine 5'-triphosphate
(R)-limonene
Dynamin
vanillyl-N-nonylamide
obatoclax
LRRC26
RAB7B
CTSC
TLR10
RGMB
ghrelin
TPR
entolimod
CASP4
resolvin D5
PPM1L
AATF
OTUD7B
PIP5K1B
CARD14
F2RL3
GJA8
glaucocalyxin A
mito-TEMPO
KPNA2
gelam honey
bepafant
chlorambucil
dibenzoylmethane
GABA receptor
JWH-015
ZFA-fmk
alpha-hydroxyglutarate
miR-3168 (miRNAs w/seed AGUUCUA)
linoleic acid
pCPT-cAMP
NFkB (family)
triclosan
Smad2/3-Smad4
p85 (pik3r)
IL-17f dimer
mir-7
mir-205
IFIT2
IL19
nimesulide
rofecoxib
clenbuterol
miR-1273h-3p (miRNAs w/seed UGCAGAC)
azithromycin
desoxycorticosterone
AKT inhibitor VIII
BPIFA1
LILRA2
PLA2G2D
harmine
IgG2a
TRPV1
mir-127
TIA1
PEMT
PLA2G2E
SDC1
CCL3L3
HPSE
iodoacetic acid
cantharidin
verteporfin
adefovir dipivoxil
miR-6075 (miRNAs w/seed CGGCCCA)
miR-1287-5p (miRNAs w/seed GCUGGAU)
NLRP3
miR-1207-3p (and other miRNAs w/seed CAGCUGG)
ACSL4
BIRC5
miR-4535 (miRNAs w/seed UGGACCU)
miR-5700 (miRNAs w/seed AAUGCAU)

miR-6507-3p (miRNAs w/seed AAAGUCC)
CHI3L1
CDC73
OXT
PYCARD
calyculin A
amino acids
SGPP2
SHC1
miR-4433a-5p (miRNAs w/seed GUCCCAC)
phenethyl isothiocyanate
miR-4698 (miRNAs w/seed CAAAAUG)
miR-5585-5p (miRNAs w/seed GAAGUAC)
miR-6730-3p (miRNAs w/seed CUGACAC)
miR-203a-3p (and other miRNAs w/seed UGAAAUG)
miR-30c-1-3p (and other miRNAs w/seed UGGGAGA)
miR-6072 (and other miRNAs w/seed CCUCAUC)
miR-30a-3p (and other miRNAs w/seed UUUCAGU)
MYOCD
sodium bisulfide
anakinra
STS
ACKR3
crocidolite asbestos
miR-7032-3p (and other miRNAs w/seed UCCUCUC)
miR-4290 (miRNAs w/seed GCCCUCC)
BMP4
miR-5589-3p (miRNAs w/seed GCACAUG)
miR-4504 (miRNAs w/seed GUGACAA)
conjugated linoleic acid
IGFBP3
miR-1911-3p (miRNAs w/seed ACCAGGC)
RORA
mir-10
miR-6823-5p (miRNAs w/seed CAGGGUU)
RUNX2
miR-4520-2-3p (miRNAs w/seed UUGGACA)
miR-567 (miRNAs w/seed GUAUGUU)
miR-3660 (and other miRNAs w/seed CUGACAG)
GNRH1
CaMKII
CCK
XRCC6
TLR6
miR-193a-3p (and other miRNAs w/seed ACUGGCC)
miR-340-3p (and other miRNAs w/seed CCGUCUC)
miR-8075 (miRNAs w/seed GCUGAUG)
miR-6878-5p (miRNAs w/seed GGGAGAA)
ITK
miR-4435 (miRNAs w/seed UGGCCAG)
BCR-ABL1
miR-100-3p (miRNAs w/seed AAGCUUG)
ZBTB20
miR-150-5p (and other miRNAs w/seed CUCCCAA)
miR-4785 (miRNAs w/seed GAGUCGG)
ADAM17
miR-324-3p (and other miRNAs w/seed CACUGCC)
miR-3130-5p (and other miRNAs w/seed ACCCAGU)
miR-4750-5p (miRNAs w/seed UCGGGCG)
miR-3939 (miRNAs w/seed ACGCGCA)
miR-509-5p (and other miRNAs w/seed ACUCCAG)
miR-190a-3p (miRNAs w/seed UAUAUAU)
HOXD10
NR1I3
EHMT1
miR-1537-3p (miRNAs w/seed AAACCGU)
miR-889-5p (miRNAs w/seed AUGGCUG)
miR-343 (and other miRNAs w/seed CUCCCUU)
miR-513a-5p (miRNAs w/seed UCACAGG)
miR-7705 (miRNAs w/seed AUAGCUC)
miR-548av-5p (and other miRNAs w/seed AAAGUAC)
vincristine
JINK1/2
GAS6
MAP2K2
CTSB
miR-6866-5p (miRNAs w/seed UAGAGGC)
miR-4752 (miRNAs w/seed UGUGGAU)
miR-451a (and other miRNAs w/seed AACCGUU)
mannitol
KLK4
Endothelin
S100B
SOD3
DLK1
TACR1
GAB1
NCR2
batimastat
miR-4690-3p (and other miRNAs w/seed CAGCCCA)
miR-624-3p (miRNAs w/seed ACAAGGU)
POSTN
ibuprofen
6-mercaptopurine
1L-6-hydroxymethyl-chiro-inositol 2-(R)-2-O-methyl-3-O-octadecylcarbonate
belinostat
Stat1-Stat2
F630028O10Rik
HOXA11-AS
PF-3084014
DCLK1
DAB2IP
AMBP
HCRT
NCF4
SELE
PDE4B
HOXB7
GPC1
CTTN
butylhydroxybutylnitrosamine
calmidazolium
farglitazar
acetic acid
SU1498
tridecanoic acid
ERG
ciprofibrate
POU2AF1
Stat3-Stat3
grape seed extract
UXT
BECN1
PLP1
IKZF2
ELF4
IL27RA
miR-4291 (and other miRNAs w/seed UCAGCAG)
miR-338-5p (miRNAs w/seed ACAAUAU)
miR-4274 (miRNAs w/seed AGCAGUC)
SMAD2
miR-4764-3p (miRNAs w/seed UAACUCC)
miR-6511b-5p (and other miRNAs w/seed UGCAGGC)
ginsenoside Rb1
glycochenodeoxycholate
Rac
Ahr-aryl hydrocarbon-Arnt
MFN2
N(2)-(gamma-D-glutamyl)-meso-2,2'-diaminopimelic acid
IDH2
TAC4
SRSF3
PIAS4
BMX
RALA
ADAM15
DMH1
clasto-lactacystin beta-lactone
dimethyl fumarate
salmeterol
pentobarbital
anti-benzo(a)pyrene-diol-epoxide
D-tubocurarine
E64d
buthionine sulfoximine
beta-1,3-glucan
auranofin
Cd2+
metronidazole
miR-4528 (miRNAs w/seed CAUUAUA)
miR-3119 (miRNAs w/seed GGCUUUU)
miR-337-3p (and other miRNAs w/seed CAGCUCC)
miR-6865-3p (and other miRNAs w/seed CACCCUC)
miR-652-5p (miRNAs w/seed AACCCUA)
miR-517a-3p (and other miRNAs w/seed UCGUGCA)
miR-1276 (miRNAs w/seed AAAGAGC)
miR-328-3p (and other miRNAs w/seed UGGCCCU)

miR-3682-5p (miRNAs w/seed UACUUCU)
NR4A2
miR-549a-3p (miRNAs w/seed GACAACU)
miR-6505-5p (miRNAs w/seed UGGAAUA)
PPRC1
OLR1
NUP98-DDX10
miR-512-3p (miRNAs w/seed AGUGCUG)
pregna-4,17-diene-3,16-dione
CTR9
TUG1
DL-fructose
miR-544-5p (miRNAs w/seed CUUGUUA)
clofibrate
miR-4512 (miRNAs w/seed AGGGCCU)
miR-593-3p (miRNAs w/seed GUCUCUG)
miR-30b-3p (and other miRNAs w/seed UGGGAUG)
PTK2
pravastatin
miR-532-5p (and other miRNAs w/seed AUGCCUU)
miR-3678-5p (miRNAs w/seed CCGUACA)
miR-4659a-5p (and other miRNAs w/seed UGCCAUG)
miR-374c-3p (miRNAs w/seed ACUUAGC)
EED
pituitary adenylate cyclase-activating polypeptide
miR-589-5p (and other miRNAs w/seed GAGAACC)
miR-7848-3p (miRNAs w/seed UACCCUC)
miR-3072 (and other miRNAs w/seed GCCCCCU)
collagen type i (family)
GRN
miR-888-3p (miRNAs w/seed ACUGACA)
miR-3925-5p (miRNAs w/seed AGAGAAC)
KDM2B
GNRH
TSLP
ethylene glycol tetraacetic acid
miR-6818-3p (miRNAs w/seed UGUCUCU)
miR-4744 (miRNAs w/seed CUAAAGA)
miR-6084 (miRNAs w/seed UCCGCCA)
LRP5
honokiol
PDCD1
NKX3-1
miR-3922-5p (miRNAs w/seed CAAGGCC)
miR-4300 (and other miRNAs w/seed GGGAGCU)
miR-5008-3p (and other miRNAs w/seed CUGUGCU)
miR-4318 (miRNAs w/seed ACUGUGG)
artesunic acid
SLC29A1
ITGB4
MNT
HNRNPU
CLEC4E
IL1RL1
NRTN
CEP-1347
triflusal
pirfenidone
diethylmaleate
CpdX
alloxan
pervanadate
flavokawain B
sesamol
nicotinic acetylcholine receptor
Tlr11
PDCD10
USP25
ST6GALNAC1
TRIM29
SLC5A8
TP73-AS1
FNIP1
ZC3HAV1
tofacitinib
HLA-DQ
MEFV
IFNGR
SERPINA4
PIK3C3
LPAR2
PLA2G2A
casticin
KLKB1
USP21
HLA-B
PILRA
BP-1-102
benzoquinone
fluphenazine
lemairamin
ISRIB
diazepam
eugenol
dBET6
miR-508-3p inhibitor
miR-545-5p (miRNAs w/seed CAGUAAA)
LTBR
miR-1296-3p (and other miRNAs w/seed AGUGGGG)
miR-296-3p (miRNAs w/seed AGGGUUG)
miR-218-2-3p (and other miRNAs w/seed AUGGUUC)
miR-3120 (and other miRNAs w/seed ACAGCAA)
miR-6881-3p (miRNAs w/seed UCCUCUU)
miR-1-5p (and other miRNAs w/seed CAUACUU)
miR-5189-3p (and other miRNAs w/seed GCCAACC)
miR-136-5p (miRNAs w/seed CUCCAUU)
miR-4477b (miRNAs w/seed UUAAGGA)
PTP4A1
miR-383-5p (miRNAs w/seed AGAUCAG)
ANGPT1
CDK2
TAF4
methylmercury
miR-3618 (miRNAs w/seed GUCUACA)
miR-485-3p (and other miRNAs w/seed UCAUACA)
miR-548ao-5p (and other miRNAs w/seed GAAGUAA)
miR-4684-3p (miRNAs w/seed GUUGCAA)
miR-3605-5p (and other miRNAs w/seed GAGGAUG)
miR-4515 (miRNAs w/seed GGACUGG)
Ciap
JUN/JUNB/JUND
RNF20
LINC00963
SAFB2
USP19
1-methyl-4-phenylpyridinium
3-D-glucosyl-1,2-diacylglycerol
behenic acid
treprostinil
chitinase
tylophorine
CACTIN
ROBO3
TNIP3
USP28
ATP6V0D2
ABRAXAS2
B3GNT2
ZFP64
CIRBP
RNF122
LRRC19
P-TEFb
cerotic acid
PF-4691502
ORM1
MEP1A
HSPB6
BAG2
IL17B
A4GALT
CD163
APOD
NLRC3
Rps6ka5
WARS1
SKLB023
ERO1A
FLLL-32
NEK2
PPP1CC
HS6ST2
CAY10397
N-hydroxy-2,2-diphenylacetamide
(+)-epicatechin
1-(2-hydroxy-5-methylphenyl)-3-phenyl-1,3-propanedione
ITF3056

ibotenic acid
pegintron
dihydrosphingosine 1-phosphate
MCC950
tramadol
bupropion
1,1-diethyl-2-hydroxy-2-nitrosohydrazine
MR-409
AC083837.1
miR-499a-5p inhibitor
miR-208b inhibitor
oxamic acid
Vi capsular polysaccharide
miR-369-5p (miRNAs w/seed GAUCGAC)
CRKL
SPHK2
TNFAIP2
GNA13
GABBR1
GTF2I
CRTC1
EDNRA
Z-551
amlodipine
asiatic acid
NR1D1
PRKN
TNFRSF9
paricalcitol
miR-3190-5p (miRNAs w/seed CUGGCCA)
mir-34
FBXW7
PTHLH
miR-3093-3p (and other miRNAs w/seed GUGGACA)
miR-1255b-5p (and other miRNAs w/seed GGAUGAG)
miR-4693-3p (miRNAs w/seed GAGAGUG)
miR-6821-3p (and other miRNAs w/seed GACCUCU)
miR-374b-3p (miRNAs w/seed UUAGCAG)
ABCB4
CDK5
MEIS1
fisetin
CHADL
NFIL3
NPPA
miR-3157-5p (miRNAs w/seed UCAGCCA)
miR-5681b (miRNAs w/seed GGUAUUG)
ROR2
GCG
ZFAS1
HOXA-AS2
SARM1
G2535
SLC7A5
MED14
DRD1
VLDLR
AFP
ENTPD1
Ifnz (includes others)
sevoflurane
2-aminoethoxydiphenylborane
phospholipid
theophylline
tyrphostin AG 1296
canrenoate potassium
miR-1277-3p (and other miRNAs w/seed ACGUAGA)
SPIB
GRIN3A
CCR2
miR-4530 (miRNAs w/seed CCAGCAG)
Hsp70
cytochalasin D
miR-630 (miRNAs w/seed GUAUUCU)
hemin
miR-488-5p (miRNAs w/seed CCAGAUA)
miR-6734-3p (miRNAs w/seed CCUUCCC)
miR-941 (miRNAs w/seed ACCCGGC)
miR-3160-5p (miRNAs w/seed GCUUUCU)
miR-22-5p (and other miRNAs w/seed GUUCUUC)
miR-590-3p (miRNAs w/seed AAUUUUA)
GHRL
FGFR2
miR-6802-3p (miRNAs w/seed UCACCCC)
miR-3191-3p (miRNAs w/seed GGGGACG)
acetovanillone
dorsomorphin
di(2-ethylhexyl) phthalate
miR-3924 (miRNAs w/seed UAUGUAU)
miR-4714-3p (miRNAs w/seed CAACCUA)
miR-548aj-5p (and other miRNAs w/seed GCAAAAG)
miR-153-5p (miRNAs w/seed CAUUUUU)
miR-3117-3p (miRNAs w/seed UAGGACU)
miR-675-3p (miRNAs w/seed UGUAUGC)
miR-7113-5p (and other miRNAs w/seed CCAGGGA)
miR-6826-3p (miRNAs w/seed UCCCCUC)
CYB5R4
dactolisib
TRAIP
mir-218
mir-486
MLX
DHX9
LCP2
RPA1
farnesol
biochanin A
leptomycin B
chitin
7-ketocholesterol
miR-3584-5p (and other miRNAs w/seed GGAGGAG)
miR-192-5p (and other miRNAs w/seed UGACCUA)
miR-4733-5p (miRNAs w/seed AUCCCAA)
USP22
carbamylcholine
propofol
miR-5699-5p (miRNAs w/seed GCCCCAA)
streptozocin
miR-6505-3p (and other miRNAs w/seed GACUUCU)
miR-582-5p (miRNAs w/seed UACAGUU)
miR-1282 (miRNAs w/seed CGUUUGC)
miR-877-3p (miRNAs w/seed CCUCUUC)
miR-3189-3p (miRNAs w/seed CCUUGGG)
TEAD2
beta-naphthoflavone
RUNX3
PRKCQ
miR-4524a-3p (miRNAs w/seed GAGACAG)
Smad
TNFRSF11B
BTRC
UBE3A
NMU
EGLN3
PCYT1A
miR-6882-5p (miRNAs w/seed ACAAGUC)
miR-154-3p (and other miRNAs w/seed AUCAUAC)
GC-GCR dimer
urethane
dithiothreitol
plerixafor
miR-4782-5p (and other miRNAs w/seed UCUGGAU)
miR-504-3p (and other miRNAs w/seed GGGAGAG)
Brd4
miR-3157-3p (miRNAs w/seed UGCCCUA)
miR-1250-3p (miRNAs w/seed CAUUUUC)
budesonide
miR-6873-5p (miRNAs w/seed AGAGGGA)
miR-3115 (miRNAs w/seed UAUGGGU)
miR-6803-3p (miRNAs w/seed CCCUCGC)
H1-6
H1f1
miR-6749-3p (miRNAs w/seed UCCUCCC)
miR-574-5p (miRNAs w/seed GAGUGUG)
PRKACA
N-acetylsphingosine
miR-218-5p (and other miRNAs w/seed UGUGCUU)
miR-637 (and other miRNAs w/seed CUGGGGG)
miR-3685 (miRNAs w/seed UUCCUAC)
miR-6739-3p (miRNAs w/seed UUGUUCU)
miR-4695-5p (and other miRNAs w/seed AGGAGGC)
miR-4427 (miRNAs w/seed CUGAAUA)
miR-682 (miRNAs w/seed UGCAGUC)
CSF2RB
resatorvid
UNC93B1
GAS2L3

MESP1
PURA
TGIF1
IGHE
STK3
IFT57
10-nitrooleate
OSBPL8
S1PR3
RHOB
concanamycin A
hydrogen sulfide
farnesyl pyrophosphate
miR-4503 (miRNAs w/seed UUAAGCA)
miR-938 (miRNAs w/seed GCCCUUA)
miR-6767-5p (miRNAs w/seed CGCAGAC)
miR-663b (miRNAs w/seed GUGGCCC)
miR-644a (miRNAs w/seed GUGUGGC)
miR-6812-3p (miRNAs w/seed CGCUCUU)
cardiotoxin
miR-3191-5p (miRNAs w/seed UCUCUGG)
TNIP1
miR-6759-5p (miRNAs w/seed UGUGGGU)
FANCC
miR-8081 (miRNAs w/seed UUGAGUC)
SPARC
miR-302c-5p (miRNAs w/seed UUAACAU)
MM-589
PTC-209
miR-3074-5p (miRNAs w/seed UUCCUGC)
miR-501-5p (miRNAs w/seed AUCCUUU)
miR-3156-3p (miRNAs w/seed UCCCACU)
miR-935 (miRNAs w/seed CAGUUAC)
miR-576-3p (miRNAs w/seed AGAUGUG)
miR-5689 (miRNAs w/seed GCAUACA)
ARNT2
miR-4771 (miRNAs w/seed GCAGACU)
miR-3117-5p (miRNAs w/seed GACACUA)
MXD1
USF1
GAST
miR-7845-5p (miRNAs w/seed AGGGACA)
miR-29b-1-5p (miRNAs w/seed CUGGUUU)
miR-425-3p (miRNAs w/seed UCGGGAA)
miR-24-1-5p (and other miRNAs w/seed GCCUACU)
SYK/ZAP
Klrk1
mercuric chloride
12(S)-hydroxyeicosatetraenoic acid
UDP
Vhl
saikosaponin A
heparan sulfate
2-amino-5-azotoluene
tectorigenin
SLC39A14
CERS6
GSTK1
HDAC11
PON2
ADGRF5
NELFCD
S100A10
FANCA
CD5L
ponesimod
SDC2
CLTC
filgotinib
GALP
farnesyl transferase
TNRC6A
SERPINH1
TNFRSF10B
benzoic acid
rabeprazole
ETV6-NTRK3
PCM1-JAK2
epoxyeicosatrienoic acid analog B
epoxyeicosatrienoic acid analog A
theaflavin
tosyllysine chloromethyl ketone
miR-1304-5p (miRNAs w/seed UUGAGGC)
beraprost
PP2A
naringin
STAT1/3/5 dimer
CST3
UBA1
mir-379
HSPB8
CACNA1A
CXCL1
HNRNPAB
quinacrine
1-((2-chlorophenyl)diphenylmethyl)-1H-pyrazole
D-sphingosine
sanglifehrin A
4-octyl itaconate
roflumilast N-oxide
putrescine
midostaurin
8-oxo-7-hydrodeoxyguanosine
diaminopimelic acid
Mt
miR-1247-3p (miRNAs w/seed CCCGGGA)
Iga
GDF15
LIPG
A-Fos
PARK7
ST8SIA1
acetaldehyde
pilocarpine
miR-2355-3p (miRNAs w/seed UUGUCCU)
obeticholic acid
IL23
K+
miR-8076 (miRNAs w/seed AUAUGGA)
Ubiquitin
KNG1
miR-4717-5p (and other miRNAs w/seed AGGCCAC)
Trp53cor1
BAG1
miR-3166 (miRNAs w/seed GCAGACA)
miR-1258-5p (and other miRNAs w/seed GCUGAGC)
miR-881-3p (and other miRNAs w/seed ACUGUGU)
PITX2
miR-5586-3p (miRNAs w/seed AGAGUGA)
miR-4652-3p (miRNAs w/seed UUCUGUU)
miR-4325 (miRNAs w/seed UGCACUU)
miR-3680-3p (miRNAs w/seed UUUGCAU)
KMT2A
FLT1
miR-6763-3p (miRNAs w/seed UCCCCGG)
miR-6867-3p (and other miRNAs w/seed UCUCCCU)
miR-1468-5p (miRNAs w/seed UCCGUUU)
IRS2
miR-6785-3p (miRNAs w/seed CAUCGCC)
miR-659-3p (miRNAs w/seed UUGGUUC)
miR-6719-3p (miRNAs w/seed CUGACAU)
miR-8074 (miRNAs w/seed UAUGGCG)
miR-3910 (miRNAs w/seed AAGGCAU)
miR-379-5p (and other miRNAs w/seed GGUAGAC)
miR-6857-3p (miRNAs w/seed GACUGAG)
1810019D21Rik
miR-4491 (and other miRNAs w/seed AUGUGGA)
miR-4276 (and other miRNAs w/seed UCAGUGA)
miR-6732-3p (miRNAs w/seed AACCCUG)
miR-450b-5p (miRNAs w/seed UUUGCAA)
miR-606 (miRNAs w/seed AACUACU)
miR-4731-3p (and other miRNAs w/seed ACACAAG)
ARNTL
RNASE2
CD4
miR-6798-3p (miRNAs w/seed UACCCCC)
miR-6515-5p (miRNAs w/seed UGGAGGG)
FGF8
miR-6806-5p (miRNAs w/seed GUAGGCA)
miR-8062 (miRNAs w/seed AGUGAUU)
miR-6774-3p (miRNAs w/seed CGUGUCC)
miR-203a-5p (and other miRNAs w/seed GUGGUUC)
miR-656-3p (miRNAs w/seed AUAUUAU)
miR-324-3p (miRNAs w/seed CCACUGC)
miR-4773 (miRNAs w/seed AGAACAG)
ZC3H14
Hmgb1

MESP2
ABCC8
DDX17
NBEAL2
C5AR2
CCAT2
3-aminobenzamide
nimodipine
GMNN
miR-3547 (and other miRNAs w/seed GAGCACC)
miR-339-5p (and other miRNAs w/seed CCCUGUC)
miR-5684 (miRNAs w/seed ACUCUAG)
miR-126a-3p (and other miRNAs w/seed CGUACCG)
miR-4774-5p (miRNAs w/seed CUGGUAU)
miR-3671 (miRNAs w/seed UCAAAUA)
miR-3943 (miRNAs w/seed AGCCCCC)
KN 93
Rxr
miR-130b-5p (and other miRNAs w/seed CUCUUUC)
miR-4264 (miRNAs w/seed CUCAGUC)
miR-34a-3p (and other miRNAs w/seed AUCAGCA)
CEBPE
LPL
miR-6868-3p (miRNAs w/seed UCCUUCU)
miR-544b (miRNAs w/seed CCUGAGG)
miR-3150a-3p (and other miRNAs w/seed UGGGGAG)
miR-6815-3p (miRNAs w/seed GGCUUCU)
miR-626 (and other miRNAs w/seed GCUGUCU)
miR-3184-3p (miRNAs w/seed AAGUCUC)
miR-486-5p (and other miRNAs w/seed CCUGUAC)
miR-6830-3p (miRNAs w/seed GUCUUUC)
L-tryptophan
Tnf receptor
EIF4G2
NOX1
PBX3
IL-1R
AMH
mir-142
BGN
SLC9A3
NFKBIZ
miR-592 (and other miRNAs w/seed UGUGUCA)
miR-4436b-5p (miRNAs w/seed UCCACUU)
miR-6887-3p (miRNAs w/seed CCCCUCC)
miR-31-3p (and other miRNAs w/seed GCUAUGC)
diphosphoryl lipid A
1,4-glucan
arbutin
syringin
7beta-hydroxycholesterol
chartreusin
Plc beta
MSK1/2
PLA2
Rab5
glemanserin
diosmetin
DF 203
NPS
Rps15a-ps4
EIF4B
TRIM50
FBXO25
NMRAL1
POPDC2
ARL16
LYST
Hsf3
MARCHF2
SMYD5
MLXIP
ARL11
SENP5
Plk
MAP3K21
AFAP1L2
ganglioside GM3 (d18:1/18:0)
ganglioside GM3 (d18:1/20:0)
ganglioside GM3 (d18:1/22:0)
Par
IKKA/B
diaziquone
RPL19
pimasertib
AZD4547
Raet1b
MAPK8IP2
IVNS1ABP
CELA1
TRIM5
cyproconazole
NMB
TPPP3
H60a
POLDIP3
IL1RAPL1
GATAD2B
DCTN4
CCT5
AZU1
SLC7A11
RPS6KA2
DDX24
ZFP36L1
CRADD
ATP9A
IMPACT
GPR32
TMEM9B
CD8B
RAMP3
KHDRBS2
GAB3
3-hydroxydodecanoic acid
6-n-octylaminouracil
CD300E
ralimetinib
DDT
CGGBP1
IL17D
HUS1
EYA4
ATB-346
polysaccharide TA-1
BAI
verticillin A
bimekizumab
minalrestat
2,2-(2-chlorophenyl-4'-chlorophenyl)-1,1-dichloroethene
buformin
neocuproine
primaquine
indapamide
E-4031
dofetilide
desethylamiodarone
BAY-u9773
bumetanide
dibenzo(a,l)pyrene
chalcone-24
benzo(a)pyrene-7,8-dione
terpenoid
wax ester
nisoldipine
lavendustin A
LZ1 peptide
moringa oleifera aqueous seed extract
moringa oleifera diluted seed extract
dapansutrile
Z-YVAD-FMK
D-JNK inhibitor I
Z-WEHD-FMK
SR1555
SR2211
indolo(3,2-b)carbazole
alsterpaullone
kenpaullone
alpha-TEA
tetrahydropalmatine
D-lysergic acid diethylamide
tyrphostin B56
ceftazidime
trehalose dimycolate
12(S)-HPETE
L-canavanine
digitoxin

1-phenazinecarboxylic acid
miR-376b-5p (and other miRNAs w/seed GUGGAUA)
miR-548m (miRNAs w/seed AAAGGUA)
HNF1B
SGK1
miR-7843-3p (miRNAs w/seed UGAAGCC)
miR-6760-5p (miRNAs w/seed AGGGAGA)
miR-1227-3p (miRNAs w/seed GUGCCAC)
miR-3913-5p (and other miRNAs w/seed UUGGGAC)
miR-453 (and other miRNAs w/seed GGUUGCC)
miR-556-5p (miRNAs w/seed AUGAGCU)
miR-4732-5p (miRNAs w/seed GUAGAGC)
FBXO32
fluoxetine
spironolactone
GNA12
teniposide
[Lys15,Arg16,Leu27]VIP(1-7)GRF(8-27)
AOPEP
ADRA1
dienogest
rintatolimod
IFN alpha receptor
CpG ODN M362
BCAS2
PYHIN1
GATM
benzenesulfonic acid
IgG2b
Mucin
TRAFD1
DCB 3503
STX11
AHSG
IL31RA
NCK2
LOC102724788/PRODH
PPP1R13B
PROS1
FGG
PILRB
Orm1 (includes others)
EIF2AK1
KIDINS220
RPS3
CCL22
ADAM8
PRMT2
RAB7A
ODN-A151
Muc1
GCLM
HG-9-91-01
MI-773
3-hydroxybutyric acid
mevalonolactone
butylated hydroxytoluene
FT671
aclarubicin
methyllycaconitine
allosamidin
miR-1197 (and other miRNAs w/seed AGGACAC)
miR-3144-3p (miRNAs w/seed UAUACCU)
nilotinib
monocrotaline
miR-193b-5p (miRNAs w/seed GGGGUUU)
miR-3136-3p (and other miRNAs w/seed GGCCCAA)
miR-2115-5p (miRNAs w/seed GCUUCCA)
miR-520g-3p (and other miRNAs w/seed CAAAGUG)
miR-7112-3p (miRNAs w/seed GCAUCAC)
miR-143-3p (and other miRNAs w/seed GAGAUGA)
GDF9
miR-8055 (miRNAs w/seed UUUGAGC)
miR-212-5p (miRNAs w/seed CCUUGGC)
Mmp
CLEC10A
particulate matter
iron dextran
RYR2
YWHAQ
mir-373
PTGDR
CSF2RA
TLE1
MMP8
ATXN3
VEGFD
PPP5C
DUSP14
CAMKK2
MIA-602
hydroquinone
1-chloro-2-(2,2,2-trichloro-1-(4-chlorophenyl)ethyl)benzene
ethylenediaminetetraacetic acid
poly-L-lysine
[D-Ala2,N-Me-Phe4,Gly5-ol]-Enkephalin
gambogic acid
cyclomaltodextrin
S-equol
miR-1914-5p (miRNAs w/seed CCUGUGC)
miR-6792-5p (miRNAs w/seed UAAGCAG)
miR-331-3p (miRNAs w/seed CCCCUGG)
miR-465d-3p (and other miRNAs w/seed GAUCAGG)
miR-6793-3p (miRNAs w/seed CCCCAAC)
miR-6768-5p (miRNAs w/seed ACACAGG)
NCOR1
miR-4723-5p (and other miRNAs w/seed GGGGGAG)
miR-665 (and other miRNAs w/seed CCAGGAG)
miR-4730 (miRNAs w/seed UGGCGGA)
miR-4455 (miRNAs w/seed GGGUGUG)
3,4,5,3',4'-pentachlorobiphenyl
prostaglandin J2
TET1
ITGAV
SSRP1
vitamin A
RXRB
ILK
KAT5
miR-4724-5p (miRNAs w/seed ACUGAAC)
ganglioside
2',3'-dialdehyde ATP
triciribine
R 59022
L-lysine
carnobyl iron
tocilizumab
IRF3 dimer
bacterial lipopolysaccharides
bafilomycin A
HTR7
RASSF6
LRRK2
SMAD1/5
Ferritin
SPTAN1
UNC5B
SERPINB2
CD244
THBD
mir-217
IL4I1
TSG101
COCH
MC1R
IL22RA1
DEK
niflumic acid
I-BOP
L-N6-(1-iminoethyl)-lysine
miR-548aw (miRNAs w/seed UGCAAAA)
miR-3921 (and other miRNAs w/seed CUCUGAG)
miR-4451 (miRNAs w/seed GGUAGAG)
BMPR2
arsenic
GLIS2
FZD8
mir-154
GFI1B
PDK4
indirubin
propionic acid
magnolol
lisinopril
TGAL copolymer
glycyrrhetinic acid
irinotecan

iloprost
miR-4735-5p (miRNAs w/seed CUAAUUU)
miR-3938 (miRNAs w/seed AUUCCCU)
miR-448-3p (and other miRNAs w/seed UGCAUAU)
AURK
ANLN
miR-4797-3p (miRNAs w/seed CUCAGUA)
miR-4999-3p (miRNAs w/seed CACUACC)
YBX1
miR-2392 (miRNAs w/seed AGGAUGG)
SUZ12
pifithrin alpha
beta-glycerophosphoric acid
9Z,11E-octadecadienoic acid
miR-4450 (and other miRNAs w/seed GGGGAUU)
miR-197-5p (and other miRNAs w/seed GGGUAGA)
miR-3109-3p (and other miRNAs w/seed AGGGCCA)
miR-6886-3p (miRNAs w/seed GCCCUUC)
miR-1306-3p (miRNAs w/seed CGUUGGC)
miR-4777-3p (miRNAs w/seed UACCUCA)
miR-4323 (miRNAs w/seed AGCCCCA)
RETNLB
phenylephrine
TSC1
miR-1185-5p (and other miRNAs w/seed GAGGAUA)
miR-3909 (and other miRNAs w/seed GUCCUCU)
miR-1668 (and other miRNAs w/seed AAAGGCC)
miR-888-5p (miRNAs w/seed ACUCAAA)
miR-3915 (miRNAs w/seed UGAGGAA)
miR-297a-5p (and other miRNAs w/seed UGUAUGU)
miR-4713-3p (miRNAs w/seed GGGAUCC)
miR-1343-5p (and other miRNAs w/seed GGGGAGC)
CHRNA7
IGF2BP1
miR-610 (miRNAs w/seed GAGCUAA)
miR-191-5p (and other miRNAs w/seed AACGGAA)
miR-4765 (miRNAs w/seed GAGUGAU)
miR-6834-3p (miRNAs w/seed AUGUCCC)
miR-1298-3p (miRNAs w/seed AUCUGGG)
miR-597-3p (and other miRNAs w/seed GGUUCUC)
miR-655-5p (miRNAs w/seed GAGGUUA)
miR-8086 (miRNAs w/seed GCUAGUC)
BRD2
PRDM5
miR-890 (miRNAs w/seed ACUUGGA)
miR-151-3p (and other miRNAs w/seed UAGACUG)
miR-4662a-5p (miRNAs w/seed UAGCCAA)
miR-520g-5p (miRNAs w/seed CUAGAGG)
hexachlorobenzene
MMP3
NFATC1
miR-568 (miRNAs w/seed UGUAUAA)
miR-4525 (and other miRNAs w/seed GGGGGAU)
IL25
miR-4513 (and other miRNAs w/seed GACUGAC)
miR-4742-3p (miRNAs w/seed CUGUAUU)
crizotinib
ASXL2
SP4
MEL S3
EPOR
silymarin
miR-139-3p (miRNAs w/seed GGAGACG)
miR-3689a-5p (and other miRNAs w/seed GUGAUAU)
miR-639 (miRNAs w/seed UCGCUGC)
xanthine
mocetinostat
Collagen Alpha1
SCAVENGER receptor CLASS A
TOLLIP
CBX2
XRCC5
MGAT3
PTPRE
TRAF4
RIPK3
RBP3
NPFF
FPR2
ID4
coomassie brilliant blue
meclizine
CpdX-D3
N-acetylglucosamine
miR-662 (miRNAs w/seed CCCACGU)
miR-5588-3p (miRNAs w/seed AGUCCCA)
miR-514a-3p (and other miRNAs w/seed UUGACAC)
miR-4268 (miRNAs w/seed GCUCCUC)
LATS2
EIF3E
anandamide
CYP1A1
miR-550b-2-5p (and other miRNAs w/seed UGUGCCU)
miR-6501-3p (miRNAs w/seed CAGAGCA)
miR-6918-5p (and other miRNAs w/seed GCUGAGG)
miR-3942-3p (and other miRNAs w/seed UUCAGAU)
miR-4457 (miRNAs w/seed CACAAGG)
shikonin
paroxetine
miR-6125 (miRNAs w/seed CGGAAGG)
miR-3152-5p (miRNAs w/seed UUGCCUC)
miR-3473g (and other miRNAs w/seed AAAAGUG)
IL31
CHEK1
SMURF1
NT5E
GSK3A
lycopene
dichlororibofuranosylbenzimidazole
miR-142-5p (and other miRNAs w/seed AUAAAGU)
NEDD9
MEF2A
miR-5579-5p (miRNAs w/seed AUGGUAC)
miR-449b-3p (and other miRNAs w/seed AGCCACA)
miR-5681a (and other miRNAs w/seed GAAAGGG)
miR-642a-3p (and other miRNAs w/seed GACACAU)
miR-4680-3p (miRNAs w/seed CUGAAUU)
miR-4445-5p (miRNAs w/seed GAUUGUU)
sildenafil
sodium orthovanadate
andrographolide
choline
miR-4263 (miRNAs w/seed UUCUAAG)
miR-651-3p (miRNAs w/seed AAGGAAA)
miR-3672 (and other miRNAs w/seed UGAGACU)
miR-5590-5p (miRNAs w/seed UGCCAUA)
PPIF
CALCA
miR-4446-5p (miRNAs w/seed UUUCCCU)
miR-6757-5p (miRNAs w/seed AGGGAUG)
miR-3200-5p (miRNAs w/seed AUCUGAG)
TCL1A
cinnamaldehyde
aflatoxin B1
PARP
ganetespib
TYRO3
PDK2
RAMP1
UGDH
HOXB3
NPAT
SLC6A1
TP53BP2
PCBP2
HDAC8
diltiazem
levamisole
(5-(4-N-methyl-N(2-pyridyl)amino)ethoxy)benzyl thiazolidine-2,4-dione
tin mesoporphyrin
2,4-dinitrobenzenesulfonic acid
polymyxin B
perilla alcohol
semaxinib
miR-8063 (miRNAs w/seed CAAAAUC)
ZNF281
PRKCI
mir-132
mir-31
ferulic acid
glycyrrhizic acid
miR-548as-3p (miRNAs w/seed AAAACCC)
miR-4278 (miRNAs w/seed UAGGGGG)
miR-4444 (miRNAs w/seed UCGAGUU)
miR-498-5p (miRNAs w/seed UUCAAGC)
miR-134-3p (and other miRNAs w/seed UGUGGGC)
UCHL1

miR-5195-5p (miRNAs w/seed ACCCCUA)
1,2-dioleoyloxy-3-(trimethylammonium)propane
ganciclovir
roxithromycin
GPIIB-IIIA
teichoic acid
Fcgr2
mGLUR Group I
tacedinaline
N-hexanoylhomoserine lactone
glycitein
KLF17
CLEC6A
CLEC4D
CCN6
RDH8
dynasore
RABGEF1
SMPD2
HSPB2
FGF13
PPP4C
DLX4
CXCL5
ABCD2
PROK2
RALB
BCL2L12
NUCB2
MAGED1
RNF111
F13A1
PTPRR
SRPK1
GRM5
DYNLL1
FSTL3
CCL15
CTSK
PPID
SERPIND1
Ifna4
Paxip1
VTX-2337
CXXC4
MIA-459
MIA-313
COG112 peptide
ambroxol
olmesartan medoxomil
XCT790
ferric nitrilotriacetate
famotidine
emricasan
10-(6'-ubiquinonyl)decyltriphenylphosphonium
catechol
propyl gallate
FC-99
apamin
scopoletin
RAM2061
NHB2005
N-retinylidene-N-retinylethanolamine
palmitoylethanolamide
sparfosic acid
miR-330-3p (and other miRNAs w/seed CAAAGCA)
miR-5190 (miRNAs w/seed CAGUGAC)
GHR
oxaliplatin
miR-3667-3p (miRNAs w/seed CCUUCCU)
miR-219a-2-3p (miRNAs w/seed GAAUUGU)
miR-26b-3p (and other miRNAs w/seed CUGUUCU)
HEXIM1
miR-4694-3p (miRNAs w/seed AAAUGGA)
RARG
miR-124-5p (miRNAs w/seed GUGUUCA)
THRA
miR-17-3p (and other miRNAs w/seed CUGCAGU)
HSPG2
miR-548g-3p (miRNAs w/seed AAACUGU)
miR-12185-3p (and other miRNAs w/seed CAGCCAC)
miR-651-5p (miRNAs w/seed UUAGGAU)
PCSK9
PCK1
PNN
TOPBP1
Mn(III)-tetrakis-(4-benzoic acid) porphyrin
U18666A
ZNF202
MK2206
mir-101
RBP4
BID
PTK2B
HULC
zinc sulfate
zymosan A
creatine
miR-490-3p (miRNAs w/seed AACCUGG)
miR-589-3p (and other miRNAs w/seed CAGAACA)
miR-5188 (miRNAs w/seed AUCGGAC)
miR-208a-5p (and other miRNAs w/seed AGCUUUU)
mir-19
miR-3941 (and other miRNAs w/seed UACACAC)
miR-5627-5p (and other miRNAs w/seed GAGGGUG)
miR-887-5p (miRNAs w/seed UUGGGAG)
Hsp27
MEN1
FYN
azoxymethane
miR-5002-3p (miRNAs w/seed GACUGCC)
RNF2
AKT2
IL4R
miR-7-1-3p (and other miRNAs w/seed AACAAAU)
MAPK11
VAV1
CLU
WAS
VAV2
ramipril
NUP98-HOXA9
bufalin
miR-3682-3p (miRNAs w/seed GAUGAUA)
miR-8068 (miRNAs w/seed GUUUGUU)
miR-451b (miRNAs w/seed AGCAAGA)
miR-96-3p (miRNAs w/seed AUCAUGU)
miR-548o-3p (and other miRNAs w/seed CAAAACU)
miR-3163 (miRNAs w/seed AUAAAAU)
miR-555 (miRNAs w/seed GGGUAAG)
miR-3064-3p (miRNAs w/seed UGCCACA)
MDM2
miR-3683 (miRNAs w/seed GCGACAU)
miR-2114-5p (miRNAs w/seed AGUCCCU)
miR-5581-3p (miRNAs w/seed UCCAUGC)
miR-2116-5p (miRNAs w/seed GUUCUUA)
NPM1
TRH
miR-875-3p (miRNAs w/seed CUGGAAA)
miR-3140-5p (miRNAs w/seed CCUGAAU)
CXCL10
arotinoid acid
alpha-tocopherol
miR-1934-5p (and other miRNAs w/seed CUGGUCC)
HAND2
POLR2A
miR-509-5p (and other miRNAs w/seed ACUGCAG)
ABCB6
S100A4
SRSF2
ouabain
miR-4777-5p (miRNAs w/seed UCUAGAU)
baicalin
miR-4653-3p (miRNAs w/seed GGAGUUA)
miR-5011-5p (miRNAs w/seed AUAUAUA)
miR-3148 (miRNAs w/seed GGAAAAA)
miR-4703-3p (miRNAs w/seed GUAGUUG)
miR-100-5p (and other miRNAs w/seed ACCCGUA)
echinomycin
1-hydroxy-2-methyl-2-butenyl 4-diphosphate
mGluR
IRF
TRIM25
FFAR1
ruxolitinib
astragalin
PIAS2

APOH
LIMA1
FANCD2
C6
CEBPG
SLC9A1
MAS1
CHRNA3
JMJD1C
NDFIP1
CELF1
MAP3K11
oligomycin
zileuton
clioquinol
myelin oligodendrocyte glycoprotein (35-55)
scopolamine
allopregnanolone
miR-676-3p (miRNAs w/seed UGUCCUA)
miR-1825 (miRNAs w/seed CCAGUGC)
miR-648 (miRNAs w/seed AGUGUGC)
SOX3
miR-766-5p (and other miRNAs w/seed GGAGGAA)
CpG ODN 2395
HOXA11
COL2A1
Mir200
CYP2J2
TNFRSF13B
retinol
miR-4661-5p (miRNAs w/seed ACUAGCU)
miR-150-3p (miRNAs w/seed UGGUACA)
miR-6835-3p (miRNAs w/seed AAAGCAC)
miR-584-3p (miRNAs w/seed CAGUUCC)
miR-3687 (and other miRNAs w/seed CCGGACA)
miR-381-3p (and other miRNAs w/seed AUACAAG)
miR-181d-3p (miRNAs w/seed CACCGGG)
TRPM2
mir-193
PRKCZ
NOSTRIN
miR-4473 (miRNAs w/seed UAGUGCU)
salubrinal
ORAI1
COL1A1
PMP22
UBD
MIR101
ANGPTL2
TNFRSF25
PRDX2
Gm12602
CH-223191
Go6983
reserpine
miR-1301-5p (and other miRNAs w/seed GCUCUAG)
miR-7152-5p (miRNAs w/seed UUCCUGU)
nitrofen
rasagiline
NSD2
NQO1
MERTK
SPRY2
FGFR3
CD80
PAK2
phosphatidylethanolamine
sulprostone
Rp-8-Br-cGMPS
N6-cyclohexyladenosine
deoxyguanosine
hyodeoxycholic acid
glycodeoxycholic acid
IGF receptor
MAP1LC3
cortisone
C1QTNF12
ixabepilone
Cyclin B
CK1
chlormethiazole
NIK
1,2,5,6-dibenzanthracene
cardamomin
DEFA1 (includes others)
prochloraz
irisolidone
dietary fat derivative
imperatorin
IGF2-AS
TRIM13
KDM4D
polyethyleneimine
CHIA
DDX41
TRIM66
Il3r
CHD5
SMCR8
PRR7
Mir124a-1hg
C1QL1
PSMD14
DGKH
TRIM67
ESRP2
ASH1L
TRIM36
SCARB2
INAVA
NAAA
ganglioside GM3 (d18:1/16:0)
ganglioside GM3 (d18:1/24:0)
ganglioside GM3 (d18:1/24:1(15Z))
TH17 Cytokine
isoleucine
ATP1A2
pycnogenols
pinoresinol
foretinib
DAPK1
GCA
TPSG1
BAG4
17alpha-hydroxyprogesterone caproate
CLIP1
U2AF2
LYZ
PKC delta V1-1 peptide 8-17
MADD
9-benzyl-8-hydroxy-2-(2-methoxyethoxy)adenine
TRIM9
PBK
CARD8
KLF16
CEACAM6
SERPINB3
CEACAM21
IFI30
EIF2B1
MID2
GZMA
EID2
MKNK2
MLST8
FCAR
IFNGR2
STAG2
Defb2
GJC1
KRT5
Trim30a/Trim30d
USP27X
CEACAM7
ELOA
BTG1
IL20RA
Miat
URMC-099
VEZF1
CASP8AP2
RAN
TCP1
CASP10
RING1
Acp5
CRIP2

Irs4
SRSF5
NKRF
TIGAR
UIMC1
CNTFR
mercaptosteroid 4
mercaptosteroid 6
P2pal-18S
C20-D3-vitamin A
beta2 adrenergic receptor agonist
N6-carboxymethyl-lysine bovine serum albumin
temocapril
nordihydrocapsiate
methoxyacetic acid
nitrous oxide
mecamylamine
amantadine
prinaberel
astemizole
ODN1411
beta-lapachone
phosphorylcholine
triphenyltin
albendazole
10-hydroxy docosahexaenoic acid
cycloserine
pegaptanib
oseltamivir
di-2-pyridylketone 4-cyclohexyl-4-methyl-3-thiosemicarbazone
tarenflurbil
minodronate
ketotifen
modafinil
AX15839
10-decarbamoylmitomycin C
nomifensine
idronoxil
MLN-4760
BRF110
yoda1
potassium tetraperoxochromate
aripiprazole
tenidap
(indol-3-yl)pyruvic acid
ellipticine
physostigmine
strychnine
SR 31747
danazol
epiallopregnanolone
miR-138-1-3p (miRNAs w/seed CUACUUC)
miR-6859-5p (and other miRNAs w/seed AGAGGAA)
miR-3084a-3p (and other miRNAs w/seed UCUGCCA)
2-methoxyestradiol
miR-10400-5p (and other miRNAs w/seed GGCGGCG)
Hedgehog
miR-487a-5p (and other miRNAs w/seed UGGUUAU)
miR-4764-5p (miRNAs w/seed GGAUGUG)
sodium chloride
miR-28-3p (and other miRNAs w/seed ACUAGAU)
miR-1322 (miRNAs w/seed AUGAUGC)
miR-6070 (miRNAs w/seed CGGUUCC)
miR-4669 (miRNAs w/seed GUGUCCG)
miR-6776-3p (miRNAs w/seed AACCACC)
miR-1253 (miRNAs w/seed GAGAAGA)
cadmium
miR-1247-5p (miRNAs w/seed CCCGUCC)
dopamine D1 receptor
CXCL6
Cd209b
Sphk
doramapimod
blinatumomab
PRCP
PIMREG
EIF2A
PTK
CC2D1A
KMT5B
SFRP5
saracatinib
valine
catumaxomab
nadifloxacin
IER3
SELL
PDE6B
EZH1
AGR2
P2RY1
TPSD1
MGLL
PIP5K1C
CCR4
TRPA1
MS4A1
GNMT
Pde4d
RHOC
BC-1215
nicorandil
dimercaptopropanol
trimethyltin
hydroxychloroquine
nigericin
poly rI:rC-RNA HMW
diacerein
polyphosphate
BAR501
murabutide
atropine
vinorelbine
tyrphostin AG825
12-hydroxyeicosatetraenoic acid
Muscarinic cholinergic receptor
miR-7109-3p (miRNAs w/seed AAGCCUC)
miR-181a-1-3p (and other miRNAs w/seed CCAUCGA)
POMC
miR-7110-3p (miRNAs w/seed CUCUCUC)
bisphenol A
BMS-690514
NPY
miR-3614-3p (miRNAs w/seed AGCCUUC)
miR-20a-3p (miRNAs w/seed CUGCAUU)

**Supplementary Table S25. Unique and overlapping genes composing the specific 25-gene signatures for the compassion of the conditions "Calu-3_SARS-CoV-2_down-regulated" and "Calu-3_SARS-CoV_down-regulated".** Comparative analysis was done using VENNY 2.1. Data visualization was done by R-based Venn diagrams shown in Figure 6B.

**16 elements included exclusively in "Calu-3_SARS-CoV-2_down-regulated":**
GDF1
AC013717.1
CKS1BP3
FBXL21P
AP001282.2
SPTLC1P2
AL355916.3
AC097634.4
PCDHA13
NPC1L1
RF00283
AC080023.2
VTCN1
AL049839.2
SERPINA4
CRPPA

**16 elements included exclusively in "Calu-3_SARS-CoV_down-regulated":**
SFTPA2
URGCP-MRPS24
CGB8
AC005258.1
CTAGE8
AC026464.3
AC126283.2
EPS8L3
RPL24P2
AC068533.4
AP000553.5
AC140479.2
PCDHB8
ALG14
RAB43P1
TMEM189-UBE2V1

**9 common elements in "Calu-3_SARS-CoV-2_down-regulated" and "Calu-3_SARS-CoV_down-regulated":**
CFTRP1
Z98752.3
AC024995.1
MOGAT1
DMBX1
CSN3
ROS1
SCGN
AC105129.1

**Supplementary Table S26. Unique and overlapping genes composing the specific 25-gene signatures for the compassion of the conditions "Calu-3_SARS-CoV-2_up-regulated" and "Calu-3_SARS-CoV_up-regulated".** Comparative analysis was done using VENNY 2.1. Data visualization was done by R-based Venn diagrams shown in Figure 6B.

| **12 elements included exclusively in "Calu-3_SARS-CoV-2_up-regulated"** | **12 elements included exclusively in "Calu-3_SARS-CoV_up-regulated"** | **13 common elements in "Calu-3_SARS-CoV-2_up-regulated" and "Calu-3_SARS-CoV_up-regulated"** |
|---|---|---|
| OASL2P | AL451165.1 | IFNL4 |
| OR4C6 | AL122018.1 | IFNL2 |
| OR10AB1P | OR7E115P | TAAR3P |
| IFNA7 | OR7E108P | IFNL3 |
| AF279873.2 | KRTAP1-1 | IFNB1 |
| IFNA16 | LPA | MMD2 |
| AL031777.2 | CNOT6LP1 | RTL9 |
| AL358075.4 | NPFFR2 | IFNL1 |
| IFNA10 | HAND1 | OR1F1 |
| RFPL1 | HMGB2P1 | ARL14EPL |
| TMEM239 | CEACAM20 | IFNL3P1 |
| SCN3A | OR7E91P | WFDC5 |
| | | ATP4A |

**Supplementary Table S27. Unique and overlapping genes composing the specific 25-gene signatures for the compassion of the conditions "Calu-3_SARS-CoV-2_down-regulated" and "Caco-2_SARS-CoV-2_down-regulated".** Comparative analysis was done using VENNY 2.1. Data visualization was done by R-based Venn diagrams shown in Figure 6B.

| 25 elements included exclusively in "Calu-3_SARS-CoV-2_down-regulated": | 25 elements included exclusively in "Caco-2_SARS-CoV-2_down-regulated": | 0 common elements in "Calu-3_SARS-CoV-2_down-regulated" and "Caco-2_SARS-CoV-2_down-regulated": |
|---|---|---|
| CFTRP1 | AL450063.1 | |
| Z98752.3 | SMLR1 | |
| GDF1 | Z96811.1 | |
| AC024995.1 | AC006548.1 | |
| MOGAT1 | HSPE1P3 | |
| DMBX1 | AHSG | |
| AC013717.1 | TMSB4XP6 | |
| CKS1BP3 | KNG1 | |
| CSN3 | RPS20P14 | |
| FBXL21P | SUGT1P4 | |
| AP001282.2 | SOAT2 | |
| SPTLC1P2 | PLA2G12B | |
| AL355916.3 | AC114737.2 | |
| AC097634.4 | KRT20 | |
| PCDHA13 | PSMC1P1 | |
| NPC1L1 | APOB | |
| ROS1 | SNRPEP4 | |
| RF00283 | SULT2A1 | |
| AC080023.2 | SERPINA6 | |
| VTCN1 | ALPI | |
| AL049839.2 | BX248409.1 | |
| SERPINA4 | GUCY2C | |
| SCGN | AFP | |
| AC105129.1 | AC007347.2 | |
| CRPPA | INO80B-WBP1 | |

**Supplementary Table S28. Unique and overlapping genes composing the specific 25-gene signatures for the compassion of the conditions "Calu-3_SARS-CoV-2_up-regulated" and "Caco-2_SARS-CoV-2_up-regulated".** Comparative analysis was done using VENNY 2.1. Data visualization was done by R-based Venn diagrams shown in Figure 6B.

| 25 elements included exclusively in "Calu-3_SARS-CoV-2_up-regulated" | 25 elements included exclusively in "Caco-2_SARS-CoV-2_up-regulated" | 0 common elements in "Calu-3_SARS-CoV-2_up-regulated" and "Caco-2_SARS-CoV-2_up-regulated" |
|---|---|---|
| OASL2P | AC008770.4 | |
| OR4C6 | MMP20 | |
| IFNL4 | AL589182.3 | |
| OR10AB1P | AC004223.3 | |
| IFNL2 | ACTBP7 | |
| TAAR3P | SCHIP1 | |
| IFNA7 | AC004832.3 | |
| AF279873.2 | IRGM | |
| IFNL3 | AC009163.4 | |
| IFNB1 | TKTL1 | |
| IFNA16 | ZFP91-CNTF | |
| MMD2 | AC109583.2 | |
| RTL9 | AP002981.1 | |
| IFNL1 | IQUB | |
| AL031777.2 | ZNF157 | |
| AL358075.4 | AC098934.3 | |
| OR1F1 | AC008763.2 | |
| ARL14EPL | ZNF554 | |
| IFNA10 | TPH2 | |
| RFPL1 | AL157712.1 | |
| TMEM239 | ZNF442 | |
| IFNL3P1 | ARRDC3 | |
| WFDC5 | AC012435.1 | |
| SCN3A | RSC1A1 | |
| ATP4A | AC124312.1 | |

**Supplementary Table S29. Genes composing the 25-gene signature specific for the condition "Calu-3_SARS-CoV-2_down-regulated".** Gene symbol, gene ID, synonyms, full name(s), information on the gene type and a short description from NCBI are provided.

| Symbol | Gene ID | Synonym | Name | Gene type | Description |
|---|---|---|---|---|---|
| CFTRP1 | 140871 | C20orf189; dJ760C5.1 | CFTR pseudogene 1 | pseudo | |
| Z98752.3 | not found | - | - | - | - |
| GDF1 | 2657 | RAI; DORV; LAG1; UOG1; CERS1; CHTD6; DTGA3; LASS1 | growth differentiation factor 1 | protein coding | This gene encodes a secreted ligand of the TGF-beta (transforming growth factor-beta) superfamily of proteins. Ligands of this family bind various TGF-beta receptors leading to recruitment and activation of SMAD family transcription factors that regulate gene expression. The encoded preproprotein is proteolytically processed to generate each subunit of the disulfide-linked homodimer. Studies in rodents suggest that this protein is involved in the establishment of left-right asymmetry in early embryogenesis and in neural development in later embryogenesis. The encoded protein is translated from a bicistronic mRNA that also encodes ceramide synthase 1. Mutations in this gene are associated with several congenital cardiovascular malformations. [provided by RefSeq, Jul 2016] |
| AC024995.1 | not found | - | - | - | - |
| MOGAT1 | 116255 | MGAT1; DGAT2L; DGAT2L1 | monoacylglycerol O-acyltransferase 1 | protein coding | Acyl-CoA:monoacylglycerol acyltransferase (MOGAT; EC 2.3.1.22) catalyzes the synthesis of diacylglycerols, the precursor of physiologically important lipids such as triacylglycerol and phospholipids (Yen et al., 2002 [PubMed |

| | | | | | |
|---|---|---|---|---|---|
| | | | | | 12077311]).[supplied by OMIM, Mar 2008] |
| DMBX1 | 127343 | MBX; OTX3; PAXB | diencephalon/ mesencephalon homeobox 1 | protein coding | This gene encodes a member of the bicoid sub-family of homeodomain-containing transcription factors. The encoded protein acts as a transcription factor and may play a role in brain and sensory organ development. Two transcript variants encoding distinct isoforms have been identified for this gene. [provided by RefSeq, Jul 2008] |
| AC013717.1 | not found | - | - | - | - |
| CKS1BP3 | 246715 | | CDC28 protein kinase regulatory subunit 1B pseudogene 3 | pseudo | |
| CSN3 | 1448 | KCA; CSNK; CNS10; CSN10 | casein kappa | protein coding | |
| FBXL21P | 26223 | FBL3B; Fbl21; FBXL21; FBXL3B; FBXL3P | F-box and leucine rich repeat protein 21, pseudogene | pseudo | This locus represents a transcribed pseudogene that is related to genes encoding members of the F-box family of proteins. [provided by RefSeq, Nov 2017] |
| AP001282.2 | not found | - | - | - | - |
| SPTLC1P2 | 100874498 | | serine palmitoyltransferase long chain base subunit 1 pseudogene 2 | pseudo | |
| AL355916.3 | not found | - | - | - | - |
| AC097634.4 | not found | - | - | - | - |
| PCDHA13 | 56136 | CNR5; CNRN5; CNRS5; CRNR5; PCDH-ALPHA13 | protocadherin alpha 13 | protein coding | This gene is a member of the protocadherin alpha gene cluster, one of three related gene clusters tandemly linked on chromosome five that demonstrate an unusual genomic organization similar to that of B-cell and T-cell receptor gene clusters. The alpha gene cluster is composed of 15 cadherin superfamily genes related to the |

| | | | | | |
|---|---|---|---|---|---|
| | | | | | mouse CNR genes and consists of 13 highly similar and 2 more distantly related coding sequences. The tandem array of 15 N-terminal exons, or variable exons, are followed by downstream C-terminal exons, or constant exons, which are shared by all genes in the cluster. The large, uninterrupted N-terminal exons each encode six cadherin ectodomains while the C-terminal exons encode the cytoplasmic domain. These neural cadherin-like cell adhesion proteins are integral plasma membrane proteins that most likely play a critical role in the establishment and function of specific cell-cell connections in the brain. Alternative splicing has been observed and additional variants have been suggested but their full-length nature has yet to be determined. [provided by RefSeq, Jul 2008] |
| NPC1L1 | 29881 | LDLCQ7; NPC11L1; SLC65A2 | NPC1 like intracellular cholesterol transporter 1 | protein coding | The protein encoded by this gene is a multi-pass membrane protein. It contains a conserved N-terminal Niemann-Pick C1 (NPC1) domain and a putative sterol-sensing domain (SSD) which includes a YQRL motif functioning as a plasma membrane to trans-Golgi network transport signal in other proteins. This protein takes up free cholesterol into cells through vesicular endocytosis and plays a critical role in the absorption of intestinal cholesterol. It also has the ability to transport alpha-tocopherol (vitamin E). The drug ezetimibe targets this protein and inhibits the absorption of intestinal cholesterol and |

| | | | | | |
|---|---|---|---|---|---|
| | | | | | alpha-tocopherol. In addition, this protein may play a critical role in regulating lipid metabolism. Polymorphic variations in this gene are associated with plasma total cholesterol and low-density lipoprotein cholesterol (LDL-C) levels and coronary heart disease (CHD) risk. Alternatively spliced transcript variants encoding different isoforms have been found for this gene. [provided by RefSeq, Oct 2009] |
| ROS1 | 6098 | ROS; MCF3; c-ros-1 | ROS proto-oncogene 1, receptor tyrosine kinase | protein coding | This proto-oncogene, highly-expressed in a variety of tumor cell lines, belongs to the sevenless subfamily of tyrosine kinase insulin receptor genes. The protein encoded by this gene is a type I integral membrane protein with tyrosine kinase activity. The protein may function as a growth or differentiation factor receptor. [provided by RefSeq, Jul 2008] |
| RF00283 | not found | - | - | - | - |
| AC080023.2 | not found | - | - | - | - |
| VTCN1 | 79679 | B7X; B7H4; B7S1; B7-H4; B7h.5; VCTN1; PRO1291 | V-set domain containing T cell activation inhibitor 1 | protein coding | This gene encodes a protein belonging to the B7 costimulatory protein family. Proteins in this family are present on the surface of antigen-presenting cells and interact with ligand bound to receptors on the surface of T cells. Studies have shown that high levels of the encoded protein has been correlated with tumor progression. A pseudogene of this gene is located on chromosome 20. Multiple transcript variants encoding different isoforms have been found for this gene. [provided by RefSeq, Dec 2011] |

| | | | | | |
|---|---|---|---|---|---|
| AL049839.2 | not found | - | - | - | - |
| SERPINA4 | 5267 | KAL; KST; PI4; KLST; PI-4; kallistatin | serpin family A member 4 | protein coding | |
| SCGN | 10590 | SEGN; CALBL; SECRET; setagin; DJ501N12.8 | secretagogin, EF-hand calcium binding protein | protein coding | The encoded protein is a secreted calcium-binding protein which is found in the cytoplasm. It is related to calbindin D-28K and calretinin. This protein is thought to be involved in KCL-stimulated calcium flux and cell proliferation. [provided by RefSeq, Jul 2008] |
| AC105129.1 | not found | - | - | - | - |
| CRPPA | 729920 | Nip; ISPD; hISPD; MDDGA7; MDDGC7; LGMDR20 | CDP-L-ribitol pyrophosphorylase A | protein coding | This gene encodes a 2-C-methyl-D-erythritol 4-phosphate cytidylyltransferase-like protein. Mutations in this gene are the cause of Walker-Warburg syndrome. Alternate splicing results in multiple transcript variants. [provided by RefSeq, May 2012] |

**Supplementary Table S30. Genes composing the 25-gene signature specific for the condition "Calu-3_SARS-CoV-2_up-regulated".** Gene symbol, gene ID, synonyms, full name(s), information on the gene type and a short description from NCBI are provided.

| Symbol | Gene ID | Synonym | Name | Gene type | Description |
|---|---|---|---|---|---|
| OASL2P | 111216278 | | 2'-5' oligoadenylate synthetase like 2, pseudogene | pseudo | |
| OR4C6 | 219432 | OR11-138 | olfactory receptor family 4 subfamily C member 6 | protein coding | Olfactory receptors interact with odorant molecules in the nose, to initiate a neuronal response that triggers the perception of a smell. The olfactory receptor proteins are members of a large family of G-protein-coupled receptors (GPCR) arising from single coding-exon genes. Olfactory receptors share a 7-transmembrane domain structure with many neurotransmitter and hormone receptors and are responsible for the recognition and G protein-mediated transduction of odorant signals. The olfactory receptor gene family is the largest in the genome. The nomenclature assigned to the olfactory receptor genes and proteins for this organism is independent of other organisms. [provided by RefSeq, Jul 2008] |
| IFNL4 | 101180976 | IFNAN | interferon lambda 4 (gene/pseudogene) | protein coding | This gene is a polymorphic pseudogene which, in some humans, encodes the interferon (IFN) lambda 4 protein. Humans are polymorphic for the dinucleotide TT/deltaG allele. Compared to the ancestral state in non-human primates, the TT allele produces a frameshift in the coding region of this gene which is predicted to |

| | | | | | |
|---|---|---|---|---|---|
| | | | | | induce nonsense-mediated mRNA decay. This allele, and an allele in the first intron of this gene, have experienced a rapid increase in frequency and show indications of positive selection. The ancestral states of these alleles are associated with an impaired ability to clear hepatitis C virus. This gene, like other type III interferons (IFNs), interacts with the IFN lambda receptor complex (IFNLR) whose signaling is generally restricted to epithelial cells. This gene resides in a cluster of four type III IFN genes and at least two pseudogenes on chromosome 19q13.2. In general, interferons are produced in response to viral infection and block viral replication and propagation to uninfected cells by activating the JAK-STAT pathway and up-regulating antiviral genes. Multiple alternatively spliced transcripts have been described for this gene but their biological validity and protein coding status is still being ascertained. [provided by RefSeq, May 2017] |
| OR10AB1P | 390091 | | olfactory receptor family 10 subfamily AB member 1 pseudogene | pseudo | Olfactory receptors interact with odorant molecules in the nose, to initiate a neuronal response that triggers the perception of a smell. The olfactory receptor proteins are members of a large family of G-protein-coupled receptors (GPCR) arising from single coding-exon genes. Olfactory receptors share a 7-transmembrane domain |

| | | | | | |
|---|---|---|---|---|---|
| | | | | | structure with many neurotransmitter and hormone receptors and are responsible for the recognition and G protein-mediated transduction of odorant signals. The olfactory receptor gene family is the largest in the genome. The nomenclature assigned to the olfactory receptor genes and proteins for this organism is independent of other organisms. [provided by RefSeq, Jul 2008] |
| IFNL2 | 282616 | IL28A; IFNL2a; IFNL3a; IL-28A | interferon lambda 2 | protein coding | This gene encodes a cytokine distantly related to type I interferons and the IL-10 family. This gene, interleukin 28B (IL28B), and interleukin 29 (IL29) are three closely related cytokine genes that form a cytokine gene cluster on a chromosomal region mapped to 19q13. Expression of the cytokines encoded by the three genes can be induced by viral infection. All three cytokines have been shown to interact with a heterodimeric class II cytokine receptor that consists of interleukin 10 receptor, beta (IL10RB) and interleukin 28 receptor, alpha (IL28RA). [provided by RefSeq, Jul 2008] |
| TAAR3P | 9288 | GPR57; GPR58; TAAR2; TAAR3 | trace amine associated receptor 3, pseudogene | pseudo | |
| IFNA7 | 3444 | IFNA-J; IFN-alphaJ | interferon alpha 7 | protein coding | |
| AF279873.2 | not found | - | - | - | - |
| IFNL3 | 282617 | IL28B; IL28C; IL-28B; IL-28C; IFN-lambda-3; IFN-lambda-4 | interferon lambda 3 | protein coding | This gene encodes a cytokine distantly related to type I interferons and the IL-10 family. This gene, interleukin 28A (IL28A), and interleukin 29 (IL29) are three closely related cytokine genes that form |

| | | | | | |
|---|---|---|---|---|---|
| | | | | | a cytokine gene cluster on a chromosomal region mapped to 19q13. Expression of the cytokines encoded by the three genes can be induced by viral infection. All three cytokines have been shown to interact with a heterodimeric class II cytokine receptor that consists of interleukin 10 receptor, beta (IL10RB) and interleukin 28 receptor, alpha (IL28RA). [provided by RefSeq, Jul 2008] |
| IFNB1 | 3456 | IFB; IFF; IFNB; IFN-beta | interferon beta 1 | protein coding | This gene encodes a cytokine that belongs to the interferon family of signaling proteins, which are released as part of the innate immune response to pathogens. The protein encoded by this gene belongs to the type I class of interferons, which are important for defense against viral infections. In addition, type I interferons are involved in cell differentiation and anti-tumor defenses. Following secretion in response to a pathogen, type I interferons bind a homologous receptor complex and induce transcription of genes such as those encoding inflammatory cytokines and chemokines. Overactivation of type I interferon secretion is linked to autoimmune diseases. Mice deficient for this gene display several phenotypes including defects in B cell maturation and increased susceptibility to viral infection. [provided by RefSeq, Sep 2015] |
| IFNA16 | 3449 | IFN-alphaO; IFN-alpha-16 | interferon alpha 16 | protein coding | |

| | | | | | |
|---|---|---|---|---|---|
| MMD2 | 221938 | PAQR10 | monocyte to macrophage differentiation associated 2 | protein coding | This gene encodes a member of the PAQR (progestin and adipoQ receptor) family. Members of this family are evolutionarily conserved with significant sequence identity to bacterial hemolysin-like proteins and are defined by a set of seven transmembrane domains. The protein encoded by this gene localizes to the Golgi apparatus to modulate Ras signaling. Alternative splicing results in multiple transcript variants and protein isoforms. [provided by RefSeq, Jun 2012] |
| RTL9 | 57529 | MAR9; MART9; RGAG1; SIRH10 | retrotransposon Gag like 9 | protein coding | |
| IFNL1 | 282618 | IL29; IL-29 | interferon lambda 1 | protein coding | This gene encodes a cytokine distantly related to type I interferons and the IL-10 family. This gene, interleukin 28A (IL28A), and interleukin 28B (IL28B) are three closely related cytokine genes that form a cytokine gene cluster on a chromosomal region mapped to 19q13. Expression of the cytokines encoded by the three genes can be induced by viral infection. All three cytokines have been shown to interact with a heterodimeric class II cytokine receptor that consists of interleukin 10 receptor, beta (IL10RB) and interleukin 28 receptor, alpha (IL28RA). [provided by RefSeq, Jul 2008] |
| AL031777.2 | not found | - | - | - | - |
| AL358075.4 | not found | - | - | - | - |

| | | | | | |
|---|---|---|---|---|---|
| OR1F1 | 4992 | OLFMF; OR1F4; OR1F5; OR1F6; OR1F7; OR1F8; OR1F9; OR1F10; OR16-36; OR16-37; OR16-88; OR16-89; OR16-90; OR1F13P; OR3-145; ORL1023 | olfactory receptor family 1 subfamily F member 1 | protein coding | Olfactory receptors interact with odorant molecules in the nose, to initiate a neuronal response that triggers the perception of a smell. The olfactory receptor proteins are members of a large family of G-protein-coupled receptors (GPCR) arising from single coding-exon genes. Olfactory receptors share a 7-transmembrane domain structure with many neurotransmitter and hormone receptors and are responsible for the recognition and G protein-mediated transduction of odorant signals. The olfactory receptor gene family is the largest in the genome. The nomenclature assigned to the olfactory receptor genes and proteins for this organism is independent of other organisms. [provided by RefSeq, Jul 2008] |
| ARL14EPL | 644100 | | ADP ribosylation factor like GTPase 14 effector protein like | protein coding | |
| IFNA10 | 3446 | IFN-alphaC | interferon alpha 10 | protein coding | This gene encodes a protein that belongs to the type I interferon family of proteins, and is located in a cluster of alpha interferon genes on chromosome 9. Interferons are small regulatory molecules that function in cell signaling in response to viruses and other pathogens or tumor cells. This gene is intronless and the encoded protein is secreted. [provided by RefSeq, Aug 2013] |
| RFPL1 | 5988 | RNF78 | ret finger protein like 1 | protein coding | |
| TMEM239 | 100288797 | | transmembrane protein 239 | protein coding | |

| | | | | | |
|---|---|---|---|---|---|
| IFNL3P1 | 100421129 | IFNL1P1; IL28BP1 | interferon lambda 3 pseudogene 1 | pseudo | |
| WFDC5 | 149708 | PRG5; WAP1; dJ211D12.5 | WAP four-disulfide core domain 5 | protein coding | This gene encodes a member of the WAP-type four-disulfide core (WFDC) domain family. Most WFDC proteins contain only one WFDC domain, and this encoded protein contains two WFDC domains. The WFDC domain, or WAP signature motif, contains eight cysteines forming four disulfide bonds at the core of the protein, and functions as a protease inhibitor. Most WFDC gene members are localized to chromosome 20q12-q13 in two clusters: centromeric and telomeric. This gene belongs to the centromeric cluster. [provided by RefSeq, Jul 2008] |
| SCN3A | 6328 | NAC3; DEE62; EIEE62; FFEVF4; Nav1.3 | sodium voltage-gated channel alpha subunit 3 | protein coding | Voltage-gated sodium channels are transmembrane glycoprotein complexes composed of a large alpha subunit with 24 transmembrane domains and one or more regulatory beta subunits. They are responsible for the generation and propagation of action potentials in neurons and muscle. This gene encodes one member of the sodium channel alpha subunit gene family, and is found in a cluster of five alpha subunit genes on chromosome 2. Multiple transcript variants encoding different isoforms have been found for this gene. [provided by RefSeq, Jul 2008] |
| ATP4A | 495 | ATP6A | ATPase H+/K+ transporting subunit alpha | protein coding | The protein encoded by this gene belongs to a family of P-type cation- |

| | | | | | |
|---|---|---|---|---|---|
| | | | | | transporting ATPases. The gastric H+, K+-ATPase is a heterodimer consisting of a high molecular weight catalytic alpha subunit and a smaller but heavily glycosylated beta subunit. This enzyme is a proton pump that catalyzes the hydrolysis of ATP coupled with the exchange of H(+) and K(+) ions across the plasma membrane. It is also responsible for gastric acid secretion. This gene encodes a catalytic alpha subunit of the gastric H+, K+-ATPase. [provided by RefSeq, Jul 2008] |

**Supplementary Table S31. Genes composing the 25-gene signature specific for the condition "Calu-3_SARS-CoV_down-regulated".** Gene symbol, gene ID, synonyms, full name(s), information on the gene type and a short description from NCBI are provided.

| Symbol | Gene ID | Synonym | Name | Gene type | Description |
|---|---|---|---|---|---|
| CFTRP1 | 140871 | C20orf189; dJ760C5.1 | CFTR pseudogene 1 | pseudo | |
| Z98752.3 | not found | - | - | - | - |
| AC024995.1 | not found | - | - | - | - |
| MOGAT1 | 116255 | MGAT1; DGAT2L; DGAT2L1 | monoacylglycerol O-acyltransferase 1 | protein coding | Acyl-CoA:monoacylglycerol acyltransferase (MOGAT; EC 2.3.1.22) catalyzes the synthesis of diacylglycerols, the precursor of physiologically important lipids such as triacylglycerol and phospholipids (Yen et al., 2002 [PubMed 12077311]).[supplied by OMIM, Mar 2008] |
| DMBX1 | 127343 | MBX; OTX3; PAXB | diencephalon/mesencephalon homeobox 1 | protein coding | This gene encodes a member of the bicoid sub-family of homeodomain-containing transcription factors. The encoded protein acts as a transcription factor and may play a role in brain and sensory organ development. Two transcript variants encoding distinct isoforms have been identified for this gene. [provided by RefSeq, Jul 2008] |
| SFTPA2 | 729238 | PSAP; PSPA; SP-A; SPA2; PSP-A; SFTP1; SP-2A; SPAII; COLEC5; SFTPA2B | surfactant protein A2 | protein coding | This gene is one of several genes encoding pulmonary-surfactant associated proteins (SFTPA) located on chromosome 10. Mutations in this gene and a highly similar gene located nearby, which affect the highly conserved carbohydrate recognition domain, are associated with idiopathic pulmonary fibrosis. The current version of the assembly displays only a single centromeric SFTPA gene pair rather than the |

| | | | | | |
|---|---|---|---|---|---|
| | | | | | two gene pairs shown in the previous assembly which were thought to have resulted from a duplication. [provided by RefSeq, Sep 2009] |
| URGCP-MRPS24 | 100534592 | | URGCP-MRPS24 readthrough | protein coding | This locus represents naturally occurring read-through transcription between the neighboring URGCP (upregulator of cell proliferation) and MRPS24 (mitochondrial ribosomal protein S24) genes on chromosome 7. The read-through transcript is predicted to encode a protein that shares sequence identity with the upstream gene product but its C-terminal region is distinct due to a frameshift relative to the downstream gene. [provided by RefSeq, Mar 2011] |
| CSN3 | 1448 | KCA; CSNK; CNS10; CSN10 | casein kappa | protein coding | |
| CGB8 | 94115 | | chorionic gonadotropin subunit beta 8 | protein coding | This gene is a member of the glycoprotein hormone beta chain family and encodes the beta 8 subunit of chorionic gonadotropin (CG). Glycoprotein hormones are heterodimers consisting of a common alpha subunit and an unique beta subunit which confers biological specificity. CG is produced by the trophoblastic cells of the placenta and stimulates the ovaries to synthesize the steroids that are essential for the maintenance of pregnancy. The beta subunit of CG is encoded by 6 genes which are arranged in tandem and inverted pairs on chromosome 19q13.3 and contiguous with the luteinizing hormone beta subunit |

| | | | | | |
|---|---|---|---|---|---|
| | | | | | gene. [provided by RefSeq, Jul 2008] |
| AC005258.1 | not found | - | - | - | - |
| CTAGE8 | 100142659 | CTAGE4 | CTAGE family member 8 | protein coding | |
| AC026464.3) | not found | - | - | - | - |
| AC126283.2 | not found | - | - | - | - |
| EPS8L3 | 79574 | HYPT5; EPS8R3 | EPS8 like 3 | protein coding | This gene encodes a protein that is related to epidermal growth factor receptor pathway substrate 8 (EPS8), a substrate for the epidermal growth factor receptor. The function of this protein is unknown. Alternatively spliced transcript variants encoding different isoforms exist. [provided by RefSeq, Jul 2008] |
| AC105129.1 | not found | - | - | - | - |
| RPL24P2 | 140760 | dJ872K7.2; RPL24_5_1691 | RPL24 pseudogene 2 | pseudo | |
| AC068533.4 | not found | - | - | - | - |
| AP000553.5 | not found | - | - | - | - |
| AC140479.2 | not found | - | - | - | - |
| ROS1 | 6098 | ROS; MCF3; c-ros-1 | ROS proto-oncogene 1, receptor tyrosine kinase | protein coding | This proto-oncogene, highly-expressed in a variety of tumor cell lines, belongs to the sevenless subfamily of tyrosine kinase insulin receptor genes. The protein encoded by this gene is a type I integral membrane protein with tyrosine kinase activity. The protein may function as a growth or differentiation factor receptor. [provided by RefSeq, Jul 2008] |
| PCDHB8 | 56128 | PCDH3I; PCDH-BETA8 | protocadherin beta 8 | protein coding | This gene is a member of the protocadherin beta gene cluster, one of three related gene clusters tandemly linked on chromosome five. The gene clusters demonstrate an unusual genomic organization similar to that of B-cell and T-cell receptor gene clusters. The beta cluster contains 16 genes and 3 pseudogenes, each encoding 6 extracellular |

| | | | | | |
|---|---|---|---|---|---|
| | | | | | cadherin domains and a cytoplasmic tail that deviates from others in the cadherin superfamily. The extracellular domains interact in a homophilic manner to specify differential cell-cell connections. Unlike the alpha and gamma clusters, the transcripts from these genes are made up of only one large exon, not sharing common 3' exons as expected. These neural cadherin-like cell adhesion proteins are integral plasma membrane proteins. Their specific functions are unknown but they most likely play a critical role in the establishment and function of specific cell-cell neural connections. [provided by RefSeq, Jul 2008] |
| ALG14 | 199857 | CMS15; MEPCA; IDDEBF | ALG14 UDP-N-acetylglucosaminyltransferase subunit | protein coding | This gene is a member of the glycosyltransferase 1 family. The encoded protein and ALG13 are thought to be subunits of UDP-GlcNAc transferase, which catalyzes the first two committed steps in endoplasmic reticulum N-linked glycosylation. Mutations in this gene have been linked to congenital myasthenic syndrome (CMSWTA). Alternatively spliced transcript variants have been identified. [provided by RefSeq, Mar 2015] |
| RAB43P1 | 440375 | RAB41P; RAB43P | RAB43 pseudogene 1 | pseudo | |
| SCGN | 10590 | SEGN; CALBL; SECRET; setagin; DJ501N12.8 | secretagogin, EF-hand calcium binding protein | protein coding | The encoded protein is a secreted calcium-binding protein which is found in the cytoplasm. It is related to calbindin D-28K and calretinin. This protein is thought to be involved in KCL-stimulated calcium flux |

| | | | | | |
|---|---|---|---|---|---|
| | | | | | and cell proliferation. [provided by RefSeq, Jul 2008] |
| TMEM189-UBE2V1 | 387522 | CROC1B; CROC-1B; KUA-UEV; TMEM189-UBE2V1 | PEDS1-UBE2V1 readthrough | protein coding | The TMEM189-UEV mRNA is an infrequent but naturally occurring read-through transcript of the neighboring TMEM189 and UBE2V1 genes. Ubiquitin-conjugating E2 enzyme variant proteins constitute a distinct subfamily within the E2 protein family. They have sequence similarity to other ubiquitin-conjugating enzymes but lack the conserved cysteine residue that is critical for the catalytic activity of E2s. The protein produced by this transcript has UEV1 B domains but the protein is localized to the cytoplasm rather than to the nucleus. The significance of this read-through mRNA and the function of its protein product has not yet been determined. [provided by RefSeq, Oct 2010] |

**Supplementary Table S32. Genes composing the 25-gene signature specific for the condition "Calu-3_SARS-CoV_up-regulated".** Gene symbol, gene ID, synonyms, full name(s), information on the gene type and a short description from NCBI are provided.

| Symbol | Gene ID | Synonym | Name | Gene type | Description |
|---|---|---|---|---|---|
| IFNL4 | 101180976 | IFNAN | interferon lambda 4 (gene/pseudogene) | protein coding | This gene is a polymorphic pseudogene which, in some humans, encodes the interferon (IFN) lambda 4 protein. Humans are polymorphic for the dinucleotide TT/deltaG allele. Compared to the ancestral state in non-human primates, the TT allele produces a frameshift in the coding region of this gene which is predicted to induce nonsense-mediated mRNA decay. This allele, and an allele in the first intron of this gene, have experienced a rapid increase in frequency and show indications of positive selection. The ancestral states of these alleles are associated with an impaired ability to clear hepatitis C virus. This gene, like other type III interferons (IFNs), interacts with the IFN lambda receptor complex (IFNLR) whose signaling is generally restricted to epithelial cells. This gene resides in a cluster of four type III IFN genes and at least two pseudogenes on chromosome 19q13.2. In general, interferons are produced in response to viral |

| | | | | | |
|---|---|---|---|---|---|
| | | | | | infection and block viral replication and propagation to uninfected cells by activating the JAK-STAT pathway and up-regulating antiviral genes. Multiple alternatively spliced transcripts have been described for this gene but their biological validity and protein coding status is still being ascertained. [provided by RefSeq, May 2017] |
| AL451165.1 | not found | - | - | - | - |
| AL122018.1 | not found | - | - | - | - |
| IFNL2 | 282616 | IL28A; IFNL2a; IFNL3a; IL-28A | interferon lambda 2 | protein coding | This gene encodes a cytokine distantly related to type I interferons and the IL-10 family. This gene, interleukin 28B (IL28B), and interleukin 29 (IL29) are three closely related cytokine genes that form a cytokine gene cluster on a chromosomal region mapped to 19q13. Expression of the cytokines encoded by the three genes can be induced by viral infection. All three cytokines have been shown to interact with a heterodimeric class II cytokine receptor that consists of interleukin 10 receptor, beta (IL10RB) and interleukin 28 receptor, alpha (IL28RA). [provided by RefSeq, Jul 2008] |
| IFNL3 | 282617 | IL28B; IL28C; IL-28B; IL-28C; IFN-lambda-3; IFN-lambda-4 | interferon lambda 3 | protein coding | This gene encodes a cytokine distantly related to type I interferons and the IL-10 family. This gene, interleukin 28A (IL28A), and |

| | | | | | |
|---|---|---|---|---|---|
| | | | | | interleukin 29 (IL29) are three closely related cytokine genes that form a cytokine gene cluster on a chromosomal region mapped to 19q13. Expression of the cytokines encoded by the three genes can be induced by viral infection. All three cytokines have been shown to interact with a heterodimeric class II cytokine receptor that consists of interleukin 10 receptor, beta (IL10RB) and interleukin 28 receptor, alpha (IL28RA). [provided by RefSeq, Jul 2008] |
| OR7E115P | 81353 | OR10-2; OST704 | olfactory receptor family 7 subfamily E member 115 pseudogene | pseudo | Olfactory receptors interact with odorant molecules in the nose, to initiate a neuronal response that triggers the perception of a smell. The olfactory receptor proteins are members of a large family of G-protein-coupled receptors (GPCR) arising from single coding-exon genes. Olfactory receptors share a 7-transmembrane domain structure with many neurotransmitter and hormone receptors and are responsible for the recognition and G protein-mediated transduction of odorant signals. The olfactory receptor gene family is the largest in the genome. The nomenclature assigned to the olfactory receptor genes and proteins |

| | | | | | |
|---|---|---|---|---|---|
| | | | | | for this organism is independent of other organisms. [provided by RefSeq, Jul 2008] |
| ARL14EPL | 644100 | | ADP ribosylation factor like GTPase 14 effector protein like | protein coding | |
| IFNB1 | 3456 | IFB; IFF; IFNB; IFN-beta | interferon beta 1 | protein coding | This gene encodes a cytokine that belongs to the interferon family of signaling proteins, which are released as part of the innate immune response to pathogens. The protein encoded by this gene belongs to the type I class of interferons, which are important for defense against viral infections. In addition, type I interferons are involved in cell differentiation and anti-tumor defenses. Following secretion in response to a pathogen, type I interferons bind a homologous receptor complex and induce transcription of genes such as those encoding inflammatory cytokines and chemokines. Overactivation of type I interferon secretion is linked to autoimmune diseases. Mice deficient for this gene display several phenotypes including defects in B cell maturation and increased susceptibility to viral infection. [provided by RefSeq, Sep 2015] |
| MMD2 | 221938 | PAQR10 | monocyte to macrophage | protein coding | This gene encodes a member of the |

| | | | | | |
|---|---|---|---|---|---|
| | | | differentiation associated 2 | | PAQR (progestin and adipoQ receptor) family. Members of this family are evolutionarily conserved with significant sequence identity to bacterial hemolysin-like proteins and are defined by a set of seven transmembrane domains. The protein encoded by this gene localizes to the Golgi apparatus to modulate Ras signaling. Alternative splicing results in multiple transcript variants and protein isoforms. [provided by RefSeq, Jun 2012] |
| OR7E108P | 81363 | OST726 | olfactory receptor family 7 subfamily E member 108 pseudogene | pseudo | Olfactory receptors interact with odorant molecules in the nose, to initiate a neuronal response that triggers the perception of a smell. The olfactory receptor proteins are members of a large family of G-protein-coupled receptors (GPCR) arising from single coding-exon genes. Olfactory receptors share a 7-transmembrane domain structure with many neurotransmitter and hormone receptors and are responsible for the recognition and G protein-mediated transduction of odorant signals. The olfactory receptor gene family is the largest in the genome. The nomenclature assigned to the olfactory receptor genes and proteins |

| | | | | | |
|---|---|---|---|---|---|
| | | | | | for this organism is independent of other organisms. [provided by RefSeq, Jul 2008] |
| KRTAP1-1 | 81851 | HB2A; KAP1.1; KAP1.6; KAP1.7; KAP1.1A; KAP1.1B; KRTAP1A; hKAP1.7; KRTAP1.1 | keratin associated protein 1-1 | protein coding | This protein is a member of the keratin-associated protein (KAP) family. The KAP proteins form a matrix of keratin intermediate filaments which contribute to the structure of hair fibers. KAP family members appear to have unique, family-specific amino- and carboxyl-terminal regions and are subdivided into three multi-gene families according to amino acid composition: the high sulfur, the ultrahigh sulfur, and the high tyrosine/glycine KAPs. This protein is a member of the high sulfur KAP family and the gene is localized to a cluster of KAPs at 17q12-q21. [provided by RefSeq, Jul 2008] |
| TAAR3P | 9288 | GPR57; GPR58; TAAR2; TAAR3 | trace amine associated receptor 3, pseudogen | pseudo | |
| IFNL1 | 282618 | IL29; IL-29 | interferon lambda 1 | protein coding | This gene encodes a cytokine distantly related to type I interferons and the IL-10 family. This gene, interleukin 28A (IL28A), and interleukin 28B (IL28B) are three closely related cytokine genes that form a cytokine gene cluster on a chromosomal region mapped to 19q13. Expression of the cytokines encoded by the three genes can be induced by viral infection. All |

| | | | | | |
|---|---|---|---|---|---|
| | | | | | three cytokines have been shown to interact with a heterodimeric class II cytokine receptor that consists of interleukin 10 receptor, beta (IL10RB) and interleukin 28 receptor, alpha (IL28RA). [provided by RefSeq, Jul 2008] |
| LPA | 4018 | LP; AK38; APOA | lipoprotein(a) | protein coding | The protein encoded by this gene is a serine proteinase that inhibits the activity of tissue-type plasminogen activator I. The encoded protein constitutes a substantial portion of lipoprotein(a) and is proteolytically cleaved, resulting in fragments that attach to atherosclerotic lesions and promote thrombogenesis. Elevated plasma levels of this protein are linked to atherosclerosis. Depending on the individual, the encoded protein contains 2-43 copies of kringle-type domains. The allele represented here contains 15 copies of the kringle-type repeats and corresponds to that found in the reference genome sequence. [provided by RefSeq, Dec 2009] |
| WFDC5 | 149708 | PRG5; WAP1; dJ211D12.5 | WAP four-disulfide core domain 5 | protein coding | This gene encodes a member of the WAP-type four-disulfide core (WFDC) domain family. Most WFDC proteins contain only one WFDC domain, and this encoded protein contains two WFDC domains. The WFDC |

| | | | | | |
|---|---|---|---|---|---|
| | | | | | domain, or WAP signature motif, contains eight cysteines forming four disulfide bonds at the core of the protein, and functions as a protease inhibitor. Most WFDC gene members are localized to chromosome 20q12-q13 in two clusters: centromeric and telomeric. This gene belongs to the centromeric cluster. [provided by RefSeq, Jul 2008] |
| CNOT6LP1 | 729530 | | CNOT6L pseudogene 1 | pseudo | |
| RTL9 | 57529 | MAR9; MART9; RGAG1; SIRH10 | retrotransposon Gag like 9 | protein coding | |
| ATP4A | 495 | ATP6A | ATPase H+/K+ transporting subunit alpha | protein coding | The protein encoded by this gene belongs to a family of P-type cation-transporting ATPases. The gastric H+, K+-ATPase is a heterodimer consisting of a high molecular weight catalytic alpha subunit and a smaller but heavily glycosylated beta subunit. This enzyme is a proton pump that catalyzes the hydrolysis of ATP coupled with the exchange of H(+) and K(+) ions across the plasma membrane. It is also responsible for gastric acid secretion. This gene encodes a catalytic alpha subunit of the gastric H+, K+-ATPase. [provided by RefSeq, Jul 2008] |
| NPFFR2 | 10886 | GPR74; NPFF2; NPGPR; HLWAR77 | neuropeptide FF receptor 2 | protein coding | This gene encodes a member of a subfamily of G-protein-coupled |

| | | | | | |
|---|---|---|---|---|---|
| | | | | | neuropeptide receptors. This protein is activated by the neuropeptides A-18-amide (NPAF) and F-8-amide (NPFF) and may function in pain modulation and regulation of the opioid system. Alternative splicing results in multiple transcript variants. [provided by RefSeq, Jan 2009] |
| HAND1 | 9421 | Hxt; eHand; Thing1; bHLHa27 | heart and neural crest derivatives expressed 1 | protein coding | The protein encoded by this gene belongs to the basic helix-loop-helix family of transcription factors. This gene product is one of two closely related family members, the HAND proteins, which are asymmetrically expressed in the developing ventricular chambers and play an essential role in cardiac morphogenesis. Working in a complementary fashion, they function in the formation of the right ventricle and aortic arch arteries, implicating them as mediators of congenital heart disease. In addition, it has been suggested that this transcription factor may be required for early trophoblast differentiation. [provided by RefSeq, Jul 2008] |
| HMGB2P1 | 729119 | | high mobility group box 2 pseudogene 1 | pseudo | |
| OR1F1 | 4992 | OLFMF; OR1F4; OR1F5; OR1F6; OR1F7; OR1F8; | olfactory receptor family 1 subfamily F member 1 | protein coding | Olfactory receptors interact with odorant molecules in the nose, to initiate a neuronal response that triggers the |

| | | | | | |
|---|---|---|---|---|---|
| | | OR1F9; OR1F10; OR16-36; OR16-37; OR16-88; OR16-89; OR16-90; OR1F13P; OR3-145; ORL1023 | | | perception of a smell. The olfactory receptor proteins are members of a large family of G-protein-coupled receptors (GPCR) arising from single coding-exon genes. Olfactory receptors share a 7-transmembrane domain structure with many neurotransmitter and hormone receptors and are responsible for the recognition and G protein-mediated transduction of odorant signals. The olfactory receptor gene family is the largest in the genome. The nomenclature assigned to the olfactory receptor genes and proteins for this organism is independent of other organisms. [provided by RefSeq, Jul 2008] |
| IFNL3P1 | 100421129 | IFNL1P1; IL28BP1 | interferon lambda 3 pseudogene 1 | pseudo | |
| CEACAM20 | 125931 | UNQ9366 | CEA cell adhesion molecule 20 | protein coding | |
| OR7E91P | 79315 | | olfactory receptor family 7 subfamily E member 91 pseudogene | pseudo | Olfactory receptors interact with odorant molecules in the nose, to initiate a neuronal response that triggers the perception of a smell. The olfactory receptor proteins are members of a large family of G-protein-coupled receptors (GPCR) arising from single coding-exon genes. Olfactory receptors share a 7-transmembrane domain structure with many neurotransmitter and hormone receptors and are responsible for the recognition and G protein- |

| | | | | | |
|---|---|---|---|---|---|
| | | | | | mediated transduction of odorant signals. The olfactory receptor gene family is the largest in the genome. The nomenclature assigned to the olfactory receptor genes and proteins for this organism is independent of other organisms. [provided by RefSeq, Jul 2008] |

**Supplementary Table S33. Genes composing the 25-gene signature specific for the condition "Caco-2_SARS-CoV-2_down-regulated".** Gene symbol, gene ID, synonyms, full name(s), information on the gene type and a short description from NCBI are provided.

| Symbol | Gene ID | Synonym | Name | Gene type | Description |
|---|---|---|---|---|---|
| AL450063.1 | not found | - | - | - | - |
| SMLR1 | 100507203 | | small leucine rich protein 1 | protein coding | |
| Z96811.1 | not found | - | - | - | - |
| AC006548.1 | not found | - | - | - | - |
| HSPE1P3 | 100507046 | | heat shock protein family E (Hsp10) member 1 pseudogene 3 | pseudo | |
| AHSG | 197 | AHS; A2HS; HSGA; APMR1; FETUA | alpha 2-HS glycoprotein | protein coding | The protein encoded by this gene is a negatively-charged serum glycoprotein that is synthesized by hepatocytes. The encoded protein consists of two polypeptide chains, which are both cleaved from a proprotein encoded from a single mRNA. It is involved in several processes, including endocytosis, brain development, and the formation of bone tissue. Defects in this gene are a cause of susceptibility to leanness. [provided by RefSeq, Aug 2017] |
| TMSB4XP6 | 7120 | TMSL6; TMSB4L | TMSB4X pseudogene 6 | pseudo | |
| KNG1 | 3827 | BK; BDK; KNG; HMWK | kininogen 1 | protein coding | This gene uses alternative splicing to generate two different proteins- high molecular weight kininogen (HMWK) and low molecular weight kininogen (LMWK). HMWK is essential for blood coagulation and assembly of the kallikrein-kinin system. Also, bradykinin, a peptide causing numerous physiological effects, is released from HMWK. Bradykinin also functions as an antimicrobial peptide with antibacterial and |

| | | | | | |
|---|---|---|---|---|---|
| | | | | | antifungal activity. In contrast to HMWK, LMWK is not involved in blood coagulation. Infection with severe acute respiratory syndrome coronavirus 2 (SARS-CoV-2) reduces or depletes angiotensin converting enzyme 2 (ACE2), which results in an increase in levels of des-Arg(9)-bradykinin, a bioactive metabolite of bradykinin that is associated with lung injury and inflammation. Three transcript variants encoding different isoforms have been found for this gene. [provided by RefSeq, Sep 2020] |
| RPS20P14 | 440992 | RPS20_6_446 | ribosomal protein S20 pseudogene 14 | pseudo | |
| SUGT1P4 | 112272564 | | SUGT1 pseudogene 4 | pseudo | |
| SOAT2 | 8435 | ACAT2; ARGP2; ACACT2 | sterol O-acyltransferase 2 | protein coding | This gene is a member of a small family of acyl coenzyme A:cholesterol acyltransferases. The gene encodes a membrane-bound enzyme localized in the endoplasmic reticulum that produces intracellular cholesterol esters from long-chain fatty acyl CoA and cholesterol. The cholesterol esters are then stored as cytoplasmic lipid droplets inside the cell. The enzyme is implicated in cholesterol absorption in the intestine and in the assembly and secretion of apolipoprotein B-containing lipoproteins such as very low density lipoprotein (VLDL). Several alternatively spliced transcript variants of this gene have been described, but their full-length nature is not |

| | | | | | |
|---|---|---|---|---|---|
| | | | | | known. [provided by RefSeq, Jul 2008] |
| PLA2G12B | 84647 | GXIIB; FKSG71; PLA2G13; GXIIIsPLA2; sPLA2-GXIIB | phospholipase A2 group XIIB | protein coding | The protein encoded by this gene belongs to the phospholipase A2 (PLA2) group of enzymes, which function in glycolipid hydrolysis with the release of free fatty acids and lysophospholipids. This family member has altered phospholipid-binding properties and is catalytically inactive. The protein is secreted, and together with microsomal triglyceride transfer protein, it functions to regulate HNF4alpha-induced hepatitis C virus infectivity. The expression of this gene is down-regulated in various tumors, suggesting that it may function as a negative regulator of tumor progression. Alternative splicing of this gene results in multiple transcript variants. [provided by RefSeq, Dec 2015] |
| AC114737.2 | not found | - | - | - | - |
| KRT20 | 54474 | K20; CD20; CK20; CK-20; KRT21 | keratin 20 | protein coding | The protein encoded by this gene is a member of the keratin family. The keratins are intermediate filament proteins responsible for the structural integrity of epithelial cells and are subdivided into cytokeratins and hair keratins. The type I cytokeratins consist of acidic proteins which are arranged in pairs of heterotypic keratin chains. This cytokeratin is a major cellular protein of mature enterocytes and goblet cells and is specifically expressed in the gastric and intestinal mucosa. The type I cytokeratin genes are clustered in a |

| | | | | | |
|---|---|---|---|---|---|
| | | | | | region of chromosome 17q12-q21. [provided by RefSeq, Jul 2008] |
| PSMC1P1 | 151645 | PSMC1P; bcm3415 | proteasome 26S subunit, ATPase 1 pseudogene 1 | pseudo | |
| APOB | 338 | FLDB; FCHL2; LDLCQ4; apoB-48; apoB-100 | apolipoprotein B | protein coding | This gene product is the main apolipoprotein of chylomicrons and low density lipoproteins (LDL), and is the ligand for the LDL receptor. It occurs in plasma as two main isoforms, apoB-48 and apoB-100: the former is synthesized exclusively in the gut and the latter in the liver. The intestinal and the hepatic forms of apoB are encoded by a single gene from a single, very long mRNA. The two isoforms share a common N-terminal sequence. The shorter apoB-48 protein is produced after RNA editing of the apoB-100 transcript at residue 2180 (CAA->UAA), resulting in the creation of a stop codon, and early translation termination. Mutations in this gene or its regulatory region cause hypobetalipoproteinemia, normotriglyceridemic hypobetalipoproteinemia, and hypercholesterolemia due to ligand-defective apoB, diseases affecting plasma cholesterol and apoB levels. [provided by RefSeq, Dec 2019] |
| SNRPEP4 | 100130109 | | SNRPE pseudogene 4 | pseudo | |
| SULT2A1 | 6822 | HST; ST2; STD; hSTa; DHEAS; ST2A1; ST2A3; DHEA-ST; SULT2A3; DHEA-ST8 | sulfotransferase family 2A member 1 | protein coding | This gene encodes a member of the sulfotransferase family. Sulfotransferases aid in the metabolism of drugs and endogenous compounds by converting these substances into more hydrophilic water- |

| | | | | | |
|---|---|---|---|---|---|
| | | | | | soluble sulfate conjugates that can be easily excreted. This protein catalyzes the sulfation of steroids and bile acids in the liver and adrenal glands, and may have a role in the inherited adrenal androgen excess in women with polycystic ovary syndrome. [provided by RefSeq, Mar 2010] |
| SERPINA6 | 866 | CBG | serpin family A member 6 | protein coding | This gene encodes an alpha-globulin protein with corticosteroid-binding properties. This is the major transport protein for glucorticoids and progestins in the blood of most vertebrates. The gene localizes to a chromosomal region containing several closely related serine protease inhibitors which may have evolved by duplication events. [provided by RefSeq, Jul 2008] |
| ALPI | 248 | IAP | alkaline phosphatase, intestinal | protein coding | There are at least four distinct but related alkaline phosphatases: intestinal, placental, placental-like, and liver/bone/kidney (tissue non-specific). The intestinal alkaline phosphatase gene encodes a digestive brush-border enzyme. This enzyme is a component of the gut mucosal defense system and is thought to function in the detoxification of lipopolysaccharide, and in the prevention of bacterial translocation in the gut. [provided by RefSeq, Dec 2014] |
| BX248409.1 | not found | - | - | - | - |
| GUCY2C | 2984 | GC-C; STAR; DIAR6; GUC2C; MECIL; MUCIL | guanylate cyclase 2C | protein coding | This gene encodes a transmembrane protein that functions as a receptor for endogenous peptides guanylin and |

| | | | | | |
|---|---|---|---|---|---|
| | | | | | uroguanylin, and the heat-stable E. coli enterotoxin. The encoded protein activates the cystic fibrosis transmembrane conductance regulator. Mutations in this gene are associated with familial diarrhea (autosomal dominant) and meconium ileus (autosomal recessive). [provided by RefSeq, Nov 2016] |
| AFP | 174 | AFPD; FETA; HPAFP | alpha fetoprotein | protein coding | This gene encodes alpha-fetoprotein, a major plasma protein produced by the yolk sac and the liver during fetal life. Alpha-fetoprotein expression in adults is often associated with hepatocarcinoma and with teratoma, and has prognostic value for managing advanced gastric cancer. However, hereditary persistance of alpha-fetoprotein may also be found in individuals with no obvious pathology. The protein is thought to be the fetal counterpart of serum albumin, and the alpha-fetoprotein and albumin genes are present in tandem in the same transcriptional orientation on chromosome 4. Alpha-fetoprotein is found in monomeric as well as dimeric and trimeric forms, and binds copper, nickel, fatty acids and bilirubin. The level of alpha-fetoprotein in amniotic fluid is used to measure renal loss of protein to screen for spina bifida and anencephaly. [provided by RefSeq, Oct 2019] |
| AC007347.2 | not found | - | - | - | - |
| INO80B-WBP1 | 100532735 | | INO80B-WBP1 readthrough | ncRNA | This locus represents naturally occurring read-through transcription |

| | | | | | |
|---|---|---|---|---|---|
| | | | (NMD candidate) | | between the neighboring INO80B (INO80 complex subunit B) and WBP1 (WW domain-binding protein 1) genes on chromosome 2. The read-through transcript is a candidate for nonsense-mediated mRNA decay (NMD), and is unlikely to produce a protein product. [provided by RefSeq, Feb 2011] |

**Supplementary Table S34. Genes composing the 25-gene signature specific for the condition "Caco-2_SARS-CoV-2_up-regulated".** Gene symbol, gene ID, synonyms, full name(s), information on the gene type and a short description from NCBI are provided.

| Symbol | Gene ID | Synonym | Name | Gene type | Description |
|---|---|---|---|---|---|
| AC008770.4 | not found | - | - | - | - |
| MMP20 | 9313 | AI2A2; MMP-20 | matrix metallopeptidase 20 | protein coding | Proteins of the matrix metalloproteinase (MMP) family are involved in the breakdown of extracellular matrix in normal physiological processes, such as embryonic development, reproduction, and tissue remodeling, as well as in disease processes, such as arthritis and metastasis. Most MMP's are secreted as inactive proproteins which are activated when cleaved by extracellular proteinases. The protein encoded by this gene degrades amelogenin, the major protein component of dental enamel matrix, and thus thought to play a role in tooth enamel formation. A mutation in this gene, which alters the normal splice pattern and results in premature termination of the encoded protein, has been associated with amelogenesis imperfecta. This gene is part of a cluster of MMP genes located on chromosome 11q22.3. [provided by RefSeq, Aug 2011] |
| AL589182.3 | not found | - | - | - | - |
| AC004223.3 | not found | - | - | - | - |
| ACTBP7 | 67 | | ACTB pseudogene 7 | pseudo | |

| | | | | | |
|---|---|---|---|---|---|
| SCHIP1 | 29970 | SCHIP-1 | schwannomin interacting protein 1 | protein coding | |
| AC004832.3 | not found | - | - | - | - |
| IRGM | 345611 | IFI1; IRGM1; LRG47; LRG-47 | immunity related GTPase M | protein coding | This gene encodes a member of the p47 immunity-related GTPase family. The encoded protein may play a role in the innate immune response by regulating autophagy formation in response to intracellular pathogens. Polymorphisms that affect the normal expression of this gene are associated with a susceptibility to Crohn's disease and tuberculosis. Alternative splicing results in multiple transcript variants encoding different isoforms. [provided by RefSeq, Oct 2016] |
| AC009163.4 | not found | - | - | - | - |
| TKTL1 | 8277 | TKR; TKT2 | transketolase like 1 | protein coding | The protein encoded by this gene is a transketolase that acts as a homodimer and catalyzes the conversion of sedoheptulose 7-phosphate and D-glyceraldehyde 3-phosphate to D-ribose 5-phosphate and D-xylulose 5-phosphate. This reaction links the pentose phosphate pathway with the glycolytic pathway. Variations in this gene may be the cause of Wernicke-Korsakoff syndrome. Three transcript variants encoding different isoforms have been found for this gene. [provided by RefSeq, Mar 2011] |
| ZFP91-CNTF | 386607 | ZFP91; ZNF757; Zfp-91 | ZFP91-CNTF readthrough | ncRNA | This gene represents a read-through transcript composed of ZFP91 and |

| | | | | | |
|---|---|---|---|---|---|
| | | | (NMD candidate) | | CNTF sequence. This transcript is thought to be non-coding because it would be subject to nonsense-mediated mRNA decay (NMD). Read-through transcription of ZFP91 and CNTF has also been observed in mouse. [provided by RefSeq, Aug 2008] |
| AC109583.2 | not found | - | - | - | - |
| AP002981.1 | not found | - | - | - | - |
| IQUB | 154865 | | IQ motif and ubiquitin domain containing | protein coding | |
| ZNF157 | 7712 | HZF22 | zinc finger protein 157 | protein coding | This gene product is a likely zinc finger family transcription factor. It contains KRAB-A and KRAB-B domains that act as transcriptional repressors in related proteins, and multiple zinc finger DNA binding motifs and finger linking regions characteristic of the Kruppel family. This gene is part of a gene cluster on chromosome Xp11.23. [provided by RefSeq, Jul 2008] |
| AC098934.3 | not found | - | - | - | - |
| AC008763.2 | not found | - | - | - | - |
| ZNF554 | 115196 | | zinc finger protein 554 | protein coding | |
| TPH2 | 121278 | NTPH; ADHD7 | tryptophan hydroxylase 2 | protein coding | This gene encodes a member of the pterin-dependent aromatic acid hydroxylase family. The encoded protein catalyzes the first and rate limiting step in the biosynthesis of serotonin, an important hormone and neurotransmitter. Mutations in this gene may be associated with psychiatric diseases such as bipolar affective disorder and major |

| | | | | | |
|---|---|---|---|---|---|
| | | | | | depression. [provided by RefSeq, Feb 2016] |
| AL157712.1 | not found | - | - | - | - |
| ZNF442 | 79973 | | zinc finger protein 442 | protein coding | |
| ARRDC3 | 57561 | TLIMP | arrestin domain containing 3 | protein coding | This gene encodes a member of the arrestin family of proteins, which regulate G protein-mediated signaling. The encoded protein is thought to act as a regulator of breast cancer growth and progression by binding to a phosphorylated form of integrin beta4, a tumor-related antigen, targeting the integrin for internalization and degradation. [provided by RefSeq, Jul 2016] |
| AC012435.1 | not found | - | - | - | - |
| RSC1A1 | 6248 | RS1 | regulator of solute carriers 1 | protein coding | The protein encoded by this intronless gene inhibits the expression of the solute carrier family 5 (sodium/glucose cotransporter), member 1 gene (SLC5A1) and downregulates exocytosis of the SLC5A1 protein. The encoded protein is sometimes found coating the trans-Golgi network and other times is localized to the nucleus, depending on the cell cycle stage. This protein also inhibits the expression of solute carrier family 22 (organic cation transporter), member 2 (SLC22A2). [provided by RefSeq, Dec 2015] |
| AC124312.1 | not found | - | - | - | - |

**Supplementary Table S35. Gene Set Table for the 50-gene signature specific for the condition "Calu-3_SARS-CoV-2_down-regulated_and_up-regulated".** The genes of the input list representing the 50-gene signature was aligned to known biological processes by the use of the *Gene Set Enrichment* tool in GENEVESTIGATOR. For this type of analysis the 25-gene signature covering the down-regulated genes for a given condition was combined with the 25-gene signatures covering the up-regulated genes for the same condition. The p-values and the false discovery rates (FDR) are indicated. Only significant outcomes (p < 0.05) are shown.

| Intersection | p-value | min FDR | Gene Set |
|---|---|---|---|
| 8 | 1.20281E-11 | 2.30797E-06 | cytokine activity GO:0005125 |
| 7 | 2.84255E-10 | 2.72715E-05 | defense response to virus GO:0051607 |
| 4 | 9.6676E-10 | 6.18342E-05 | type I interferon receptor binding GO:0005132 |
| 4 | 1.57253E-09 | 7.54344E-05 | natural killer cell activation involved in immune response GO:0002323 |
| 4 | 2.42522E-09 | 9.30706E-05 | positive regulation of peptidyl-serine phosphorylation of STAT protein GO:003314[illegible] |
| 4 | 3.58384E-09 | 0.000114611 | T cell activation involved in immune response GO:0002286 |
| 7 | 5.02777E-09 | 0.000137819 | cytokine-mediated signaling pathway GO:0019221 |
| 4 | 8.26162E-09 | 0.000198156 | cytokine receptor binding GO:0005126 |
| 4 | 1.86446E-08 | 0.000357753 | B cell proliferation GO:0042100 |
| 4 | 1.86446E-08 | 0.000357753 | response to exogenous dsRNA GO:0043330 |
| 3 | 6.31042E-08 | 0.001100771 | positive regulation of immune response GO:0050778 |
| 4 | 1.45703E-07 | 0.002329798 | humoral immune response GO:0006959 |
| 11 | 2.21838E-07 | 0.003274344 | extracellular space GO:0005615 |
| 4 | 2.84178E-07 | 0.003894872 | type I interferon signaling pathway GO:0060337 |
| 12 | 5.055E-07 | 0.006466373 | extracellular region GO:0005576 |
| 4 | 5.58354E-07 | 0.006696088 | B cell differentiation GO:0030183 |
| 3 | 2.11034E-06 | 0.023819623 | receptor signaling pathway via JAK-STAT GO:0007259 |
| 4 | 8.86284E-06 | 0.094478176 | defense response GO:0006952 |
| 2 | 1.39655E-05 | 0.141037533 | negative regulation of T cell differentiation GO:0045581 |
| 4 | 2.21652E-05 | 0.212653524 | blood coagulation GO:0007596 |
| 5 | 2.67429E-05 | 0.24435412 | adaptive immune response GO:0002250 |
| 4 | 0.000265144 | 1 | signaling receptor binding GO:0005102 |
| 2 | 0.000509768 | 1 | negative regulation of viral genome replication GO:0045071 |
| 1 | 0.000824851 | 1 | regulation of lung goblet cell differentiation GO:1901249 |
| 1 | 0.000824851 | 1 | cytidylyltransferase activity GO:0070567 |
| 1 | 0.000824851 | 1 | negative regulation of lung goblet cell differentiation GO:1901250 |
| 1 | 0.000824851 | 1 | lung secretory cell differentiation GO:0061140 |
| 1 | 0.000824851 | 1 | chloramphenicol O-acetyltransferase activity GO:0008811 |
| 1 | 0.000824851 | 1 | interleukin-28 receptor binding GO:0032003 |
| 1 | 0.000824851 | 1 | B cell activation involved in immune response GO:0002312 |
| 1 | 0.000824851 | 1 | D-ribitol-5-phosphate cytidylyltransferase activity GO:0047349 |
| 1 | 0.000824851 | 1 | response to protozoan GO:0001562 |
| 1 | 0.000824851 | 1 | columnar/cuboidal epithelial cell development GO:0002066 |
| 1 | 0.000824851 | 1 | cellular response to sterol depletion GO:0071501 |
| 1 | 0.000824851 | 1 | regulation of MHC class I biosynthetic process GO:0045343 |
| 1 | 0.001649035 | 1 | interleukin-21 secretion GO:0072619 |
| 1 | 0.001649035 | 1 | innate vocalization behavior GO:0098582 |
| 1 | 0.001649035 | 1 | positive regulation of cysteine-type endopeptidase activity involved in execution phase of apoptosis GO:200127[illegible] |
| 1 | 0.001649035 | 1 | negative regulation of memory T cell differentiation GO:0043381 |
| 1 | 0.002472554 | 1 | regulation of phosphate transport GO:0010966 |
| 1 | 0.002472554 | 1 | intestinal lipid absorption GO:0098856 |
| 1 | 0.002472554 | 1 | interleukin-28 receptor complex GO:0032002 |
| 1 | 0.002472554 | 1 | negative regulation of interleukin-13 production GO:0032696 |
| 1 | 0.002472554 | 1 | positive regulation of hydrogen peroxide-induced cell death GO:1905206 |
| 1 | 0.002472554 | 1 | potassium:proton exchanging ATPase activity GO:0008900 |
| 1 | 0.002472554 | 1 | pre-B cell differentiation GO:0002329 |
| 2 | 0.002776632 | 1 | serine-type endopeptidase inhibitor activity GO:0004867 |
| 1 | 0.003295406 | 1 | negative regulation of T-helper 2 cell cytokine production GO:2000552 |
| 1 | 0.003295406 | 1 | positive regulation of interleukin-2 secretion GO:1900042 |
| 1 | 0.003295406 | 1 | immunoglobulin V(D)J recombination GO:0033152 |
| 1 | 0.003295406 | 1 | diacylglycerol biosynthetic process GO:0006651 |
| 1 | 0.003295406 | 1 | diacylglycerol O-acyltransferase activity GO:0004144 |
| 1 | 0.003295406 | 1 | regulation of action potential GO:0098900 |
| 2 | 0.003709677 | 1 | response to virus GO:0009615 |
| 1 | 0.004117593 | 1 | T follicular helper cell differentiation GO:0061470 |
| 1 | 0.004117593 | 1 | negative regulation of interleukin-5 production GO:0032714 |
| 1 | 0.004117593 | 1 | positive regulation of MHC class I biosynthetic process GO:0045345 |
| 1 | 0.004117593 | 1 | cellular response to ionomycin GO:1904637 |
| 2 | 0.004620277 | 1 | negative regulation of peptidase activity GO:0010466 |
| 2 | 0.004620277 | 1 | peptidase inhibitor activity GO:0030414 |
| 11 | 0.004833574 | 1 | integral component of membrane GO:0016021 |
| 1 | 0.004939115 | 1 | 2-acylglycerol O-acyltransferase activity GO:0003846 |
| 1 | 0.004939115 | 1 | regulation of cardiac muscle cell proliferation GO:0060043 |
| 1 | 0.004939115 | 1 | spanning component of plasma membrane GO:0044214 |
| 1 | 0.004939115 | 1 | mucosal immune response GO:0002385 |
| 1 | 0.004939115 | 1 | negative regulation of type 2 immune response GO:0002829 |
| 1 | 0.004939115 | 1 | interleukin-4 secretion GO:0072602 |
| 1 | 0.005759974 | 1 | potassium-transporting ATPase activity GO:0008556 |
| 2 | 0.006201202 | 1 | negative regulation of endopeptidase activity GO:0010951 |
| 1 | 0.006580169 | 1 | positive regulation of immunoglobulin production GO:0002639 |
| 1 | 0.006580169 | 1 | interferon-gamma secretion GO:0072643 |
| 1 | 0.006580169 | 1 | ventral spinal cord development GO:0021517 |
| 1 | 0.0073997 | 1 | negative regulation of cell division GO:0051782 |

| | | | |
|---|---|---|---|
| 1 | 0.0073997 | 1 | intestinal cholesterol absorption GO:0030299 |
| 1 | 0.008218569 | 1 | smooth muscle tissue development GO:0048745 |
| 1 | 0.008218569 | 1 | adult feeding behavior GO:0008343 |
| 1 | 0.009036776 | 1 | sodium:potassium-exchanging ATPase activity GO:0005391 |
| 3 | 0.009078681 | 1 | innate immune response GO:0045087 |
| 1 | 0.009854322 | 1 | regulation of long-term synaptic potentiation GO:1900271 |
| 1 | 0.010671206 | 1 | sodium ion export across plasma membrane GO:0036376 |
| 1 | 0.010671206 | 1 | cellular potassium ion homeostasis GO:0030007 |
| 3 | 0.010826714 | 1 | protein kinase activity GO:0004672 |
| 1 | 0.01148743 | 1 | regulation of presynaptic cytosolic calcium ion concentration GO:0099509 |
| 1 | 0.01148743 | 1 | glycerol metabolic process GO:0006071 |
| 1 | 0.01148743 | 1 | platelet dense granule lumen GO:0031089 |
| 1 | 0.01148743 | 1 | voltage-gated sodium channel complex GO:0001518 |
| 1 | 0.01148743 | 1 | striatum development GO:0021756 |
| 1 | 0.01148743 | 1 | establishment or maintenance of transmembrane electrochemical gradient GO:0010248 |
| 1 | 0.01148743 | 1 | negative regulation of cell growth involved in cardiac muscle cell development GO:0061052 |
| 1 | 0.012302994 | 1 | isoprenoid biosynthetic process GO:0008299 |
| 1 | 0.012302994 | 1 | 1-phosphatidylinositol-3-kinase regulator activity GO:0046935 |
| 3 | 0.012413048 | 1 | negative regulation of transcription, DNA-templated GO:0045892 |
| 1 | 0.013117899 | 1 | membrane depolarization during action potential GO:0086010 |
| 2 | 0.013837367 | 1 | magnesium ion binding GO:0000287 |
| 1 | 0.013932145 | 1 | cardiovascular system development GO:0072358 |
| 1 | 0.013932145 | 1 | myosin V binding GO:0031489 |
| 1 | 0.013932145 | 1 | negative regulation of T cell activation GO:0050868 |
| 1 | 0.013932145 | 1 | cellular sodium ion homeostasis GO:0006883 |
| 1 | 0.014745732 | 1 | cellular response to exogenous dsRNA GO:0071360 |
| 1 | 0.014745732 | 1 | regulation of phosphatidylinositol 3-kinase activity GO:0043551 |
| 1 | 0.014745732 | 1 | triglyceride biosynthetic process GO:0019432 |
| 1 | 0.015558661 | 1 | protein O-linked mannosylation GO:0035269 |
| 1 | 0.015558661 | 1 | cation binding GO:0043169 |
| 1 | 0.015558661 | 1 | voltage-gated sodium channel activity GO:0005248 |
| 3 | 0.01583665 | 1 | protein phosphorylation GO:0006468 |
| 1 | 0.016370934 | 1 | transferase activity, transferring acyl groups other than amino-acyl groups GO:0016747 |
| 1 | 0.016370934 | 1 | cytolysis GO:0019835 |
| 1 | 0.017182549 | 1 | regulation of TOR signaling GO:0032006 |
| 1 | 0.017182549 | 1 | cellular response to interferon-beta GO:0035458 |
| 1 | 0.017182549 | 1 | phosphatidylinositol 3-kinase complex GO:0005942 |
| 1 | 0.019613459 | 1 | lipoprotein metabolic process GO:0042157 |
| 1 | 0.019613459 | 1 | natural killer cell activation GO:0030101 |
| 1 | 0.020422452 | 1 | positive regulation of innate immune response GO:0045089 |
| 1 | 0.021230791 | 1 | positive regulation of mesenchymal cell proliferation GO:0002053 |
| 1 | 0.021230791 | 1 | cardiac muscle cell differentiation GO:0055007 |
| 1 | 0.022038477 | 1 | motor neuron axon guidance GO:0008045 |
| 1 | 0.022038477 | 1 | regulation of ERK1 and ERK2 cascade GO:0070372 |
| 1 | 0.022845509 | 1 | cholesterol transport GO:0030301 |
| 1 | 0.023651888 | 1 | neuronal action potential GO:0019228 |
| 1 | 0.023651888 | 1 | developmental growth GO:0048589 |
| 2 | 0.024335976 | 1 | ion transmembrane transport GO:0034220 |
| 1 | 0.024457615 | 1 | positive regulation of Ras protein signal transduction GO:0046579 |
| 1 | 0.024457615 | 1 | ATP hydrolysis coupled proton transport GO:0015991 |
| 1 | 0.024457615 | 1 | positive regulation of apoptotic signaling pathway GO:2001235 |
| 1 | 0.024457615 | 1 | positive regulation of receptor signaling pathway via JAK-STAT GO:0046427 |
| 1 | 0.026067116 | 1 | peptidyl-tyrosine autophosphorylation GO:0038083 |
| 1 | 0.02687089 | 1 | sodium channel activity GO:0005272 |
| 1 | 0.02687089 | 1 | response to testosterone GO:0033574 |
| 1 | 0.028476488 | 1 | lipid transporter activity GO:0005319 |
| 1 | 0.029278313 | 1 | negative regulation of mitotic cell cycle GO:0045930 |
| 1 | 0.032479134 | 1 | negative regulation of T cell proliferation GO:0042130 |
| 1 | 0.033277721 | 1 | cholesterol biosynthetic process GO:0006695 |
| 1 | 0.034075663 | 1 | transforming growth factor beta receptor binding GO:0005160 |
| 3 | 0.034182187 | 1 | protein homodimerization activity GO:0042803 |
| 1 | 0.034872959 | 1 | potassium ion import across plasma membrane GO:1990573 |
| 1 | 0.03566961 | 1 | positive regulation of interferon-gamma production GO:0032729 |
| 1 | 0.036465617 | 1 | lactation GO:0007595 |
| 1 | 0.03726098 | 1 | transport vesicle membrane GO:0030658 |
| 1 | 0.038055699 | 1 | sarcomere organization GO:0045214 |
| 1 | 0.038849776 | 1 | positive regulation of pathway-restricted SMAD protein phosphorylation GO:0010862 |
| 1 | 0.03964321 | 1 | signal transduction by protein phosphorylation GO:0023014 |
| 1 | 0.03964321 | 1 | phosphatidylinositol phosphorylation GO:0046854 |
| 12 | 0.039705808 | 1 | membrane GO:0016020 |
| 2 | 0.042064077 | 1 | detection of chemical stimulus involved in sensory perception of smell GO:0050911 |
| 2 | 0.042641783 | 1 | olfactory receptor activity GO:0004984 |
| 1 | 0.042810533 | 1 | adult locomotory behavior GO:0008344 |
| 1 | 0.042810533 | 1 | transmembrane receptor protein tyrosine kinase activity GO:0004714 |
| 1 | 0.043600762 | 1 | brush border membrane GO:0031526 |
| 1 | 0.045179304 | 1 | cell development GO:0048468 |
| 1 | 0.045967617 | 1 | skeletal muscle tissue development GO:0007519 |
| 1 | 0.047542331 | 1 | SMAD protein signal transduction GO:0060395 |
| 1 | 0.047542331 | 1 | positive regulation of protein kinase activity GO:0045860 |
| 1 | 0.047542331 | 1 | regulation of protein localization GO:0032880 |
| 2 | 0.047772213 | 1 | sensory perception of smell GO:0007608 |

| | | | |
|---|---|---|---|
| 1 | 0.048328732 | 1 | negative regulation of G2/M transition of mitotic cell cycle GO:0010972 |
| 1 | 0.049899626 | 1 | manganese ion binding GO:0030145 |

**Supplementary Table S36. Gene Set Table for the 50-gene signature specific for the condition "Calu-3_SARS-CoV_down-regulated_and_up-regulated".** The genes of the input list representing the 50-gene signature was aligned to known biological processes by the use of the *Gene Set Enrichment* tool in GENEVESTIGATOR. For this type of analysis the 25-gene signature covering the down-regulated genes for a given condition was combined with the 25-gene signatures covering the up-regulated genes for the same condition. The p-values and the false discovery rates (FDR) are indicated. Only significant outcomes (p < 0.05) are shown.

| Intersection | p-value | min FDR | Gene Set |
|---|---|---|---|
| 3 | 6.31042E-08 | 0.012108479 | positive regulation of immune response GO:0050778 |
| 3 | 2.11034E-06 | 0.202466792 | receptor signaling pathway via JAK-STAT GO:0007259 |
| 11 | 3.78611E-06 | 0.242160709 | extracellular region GO:0005576 |
| 2 | 1.39655E-05 | 0.66992828 | negative regulation of T cell differentiation GO:0045581 |
| 4 | 2.12978E-05 | 0.817325613 | defense response to virus GO:0051607 |
| 4 | 3.35072E-05 | 1 | cytokine activity GO:0005125 |
| 4 | 0.000106111 | 1 | cytokine-mediated signaling pathway GO:0019221 |
| 8 | 0.000114051 | 1 | extracellular space GO:0005615 |
| 2 | 0.000509768 | 1 | negative regulation of viral genome replication GO:0045071 |
| 1 | 0.000824851 | 1 | interleukin-28 receptor binding GO:0032003 |
| 1 | 0.000824851 | 1 | regulation of adenylate cyclase activity GO:0045761 |
| 1 | 0.000824851 | 1 | columnar/cuboidal epithelial cell development GO:0002066 |
| 1 | 0.000824851 | 1 | regulation of MHC class I biosynthetic process GO:0045343 |
| 1 | 0.000824851 | 1 | plasma lipoprotein particle GO:0034358 |
| 1 | 0.000824851 | 1 | chloramphenicol O-acetyltransferase activity GO:0008811 |
| 1 | 0.000824851 | 1 | B cell activation involved in immune response GO:0002312 |
| 1 | 0.000824851 | 1 | embryonic heart tube formation GO:0003144 |
| 1 | 0.001649035 | 1 | N-acetylglucosaminyldiphosphodolichol N-acetylglucosaminyltransferase activity GO:0004577 |
| 1 | 0.001649035 | 1 | argininosuccinate lyase activity GO:0004056 |
| 1 | 0.001649035 | 1 | cardiac left ventricle formation GO:0003218 |
| 1 | 0.001649035 | 1 | negative regulation of memory T cell differentiation GO:0043381 |
| 1 | 0.002472554 | 1 | regulation of phosphate transport GO:0010966 |
| 1 | 0.002472554 | 1 | UDP-N-acetylglucosamine transferase complex GO:0043541 |
| 1 | 0.002472554 | 1 | potassium:proton exchanging ATPase activity GO:0008900 |
| 1 | 0.002472554 | 1 | negative regulation of interleukin-13 production GO:0032696 |
| 1 | 0.002472554 | 1 | arginine biosynthetic process via ornithine GO:0042450 |
| 1 | 0.002472554 | 1 | RNA polymerase complex GO:0030880 |
| 1 | 0.002472554 | 1 | interleukin-28 receptor complex GO:0032002 |
| 1 | 0.002472554 | 1 | regulation of cAMP-dependent protein kinase activity GO:2000479 |
| 1 | 0.003295406 | 1 | ornithine decarboxylase inhibitor activity GO:0008073 |
| 1 | 0.003295406 | 1 | diacylglycerol O-acyltransferase activity GO:0004144 |
| 1 | 0.003295406 | 1 | detection of abiotic stimulus GO:0009582 |
| 1 | 0.003295406 | 1 | diacylglycerol biosynthetic process GO:0006651 |
| 1 | 0.003295406 | 1 | cardiac right ventricle formation GO:0003219 |
| 1 | 0.003295406 | 1 | negative regulation of T-helper 2 cell cytokine production GO:2000552 |
| 1 | 0.003295406 | 1 | opioid receptor binding GO:0031628 |
| 3 | 0.003711821 | 1 | signaling receptor binding GO:0005102 |
| 1 | 0.004117593 | 1 | mesenchyme development GO:0060485 |
| 1 | 0.004117593 | 1 | positive regulation of MHC class I biosynthetic process GO:0045345 |
| 1 | 0.004117593 | 1 | negative regulation of interleukin-5 production GO:0032714 |
| 11 | 0.004833574 | 1 | integral component of membrane GO:0016021 |
| 1 | 0.004939115 | 1 | negative regulation of type 2 immune response GO:0002829 |
| 1 | 0.004939115 | 1 | regulation of hair cycle GO:0042634 |
| 1 | 0.004939115 | 1 | 2-acylglycerol O-acyltransferase activity GO:0003846 |
| 1 | 0.004939115 | 1 | mucosal immune response GO:0002385 |
| 1 | 0.005759974 | 1 | cartilage morphogenesis GO:0060536 |
| 1 | 0.005759974 | 1 | negative regulation of RNA polymerase II regulatory region sequence-specific DNA binding GO:1903026 |
| 1 | 0.005759974 | 1 | potassium-transporting ATPase activity GO:0008556 |
| 2 | 0.006201202 | 1 | negative regulation of endopeptidase activity GO:0010951 |
| 1 | 0.006580169 | 1 | lamellar body GO:0042599 |
| 1 | 0.006580169 | 1 | polyamine metabolic process GO:0006595 |
| 1 | 0.0073997 | 1 | determination of heart left/right asymmetry GO:0061371 |
| 2 | 0.00818457 | 1 | serine-type endopeptidase activity GO:0004252 |
| 1 | 0.008218569 | 1 | adult feeding behavior GO:0008343 |
| 4 | 0.008738876 | 1 | endoplasmic reticulum membrane GO:0005789 |
| 1 | 0.009036776 | 1 | surfactant homeostasis GO:0043129 |
| 1 | 0.009036776 | 1 | sodium:potassium-exchanging ATPase activity GO:0005391 |
| 3 | 0.009078681 | 1 | innate immune response GO:0045087 |
| 1 | 0.009854322 | 1 | postreplication repair GO:0006301 |
| 1 | 0.009854322 | 1 | regulation of long-term synaptic potentiation GO:1900271 |
| 1 | 0.009854322 | 1 | vesicle cargo loading GO:0035459 |
| 1 | 0.009854322 | 1 | trophoblast giant cell differentiation GO:0060707 |
| 1 | 0.010671206 | 1 | cellular metabolic process GO:0044237 |
| 1 | 0.010671206 | 1 | cellular potassium ion homeostasis GO:0030007 |
| 1 | 0.010671206 | 1 | low-density lipoprotein particle remodeling GO:0034374 |
| 1 | 0.010671206 | 1 | sodium ion export across plasma membrane GO:0036376 |
| 1 | 0.01148743 | 1 | establishment or maintenance of transmembrane electrochemical gradient GO:0010248 |
| 1 | 0.01148743 | 1 | Golgi transport complex GO:0017119 |
| 1 | 0.01148743 | 1 | cardiac septum morphogenesis GO:0060411 |
| 1 | 0.01148743 | 1 | dolichol-linked oligosaccharide biosynthetic process GO:0006488 |
| 1 | 0.01148743 | 1 | regulation of presynaptic cytosolic calcium ion concentration GO:0099509 |
| 1 | 0.01148743 | 1 | glycerol metabolic process GO:0006071 |

| | | | |
|---|---|---|---|
| 1 | 0.012302994 | 1 | positive regulation of ruffle assembly GO:1900029 |
| 1 | 0.012302994 | 1 | monosaccharide binding GO:0048029 |
| 1 | 0.012302994 | 1 | trophectodermal cell differentiation GO:0001829 |
| 3 | 0.012413048 | 1 | negative regulation of transcription, DNA-templated GO:0045892 |
| 1 | 0.013117899 | 1 | neuropeptide receptor activity GO:0008188 |
| 1 | 0.013932145 | 1 | Rac guanyl-nucleotide exchange factor activity GO:0030676 |
| 1 | 0.013932145 | 1 | type I interferon receptor binding GO:0005132 |
| 1 | 0.013932145 | 1 | cellular sodium ion homeostasis GO:0006883 |
| 1 | 0.013932145 | 1 | apolipoprotein binding GO:0034185 |
| 1 | 0.014745732 | 1 | cellular response to exogenous dsRNA GO:0071360 |
| 1 | 0.014745732 | 1 | triglyceride biosynthetic process GO:0019432 |
| 1 | 0.015558661 | 1 | natural killer cell activation involved in immune response GO:0002323 |
| 1 | 0.016370934 | 1 | embryonic heart tube development GO:0035050 |
| 1 | 0.016370934 | 1 | peptide hormone processing GO:0016486 |
| 1 | 0.016370934 | 1 | cytolysis GO:0019835 |
| 1 | 0.016370934 | 1 | transferase activity, transferring acyl groups other than amino-acyl groups GO:0016747 |
| 1 | 0.016370934 | 1 | female gamete generation GO:0007292 |
| 2 | 0.016615114 | 1 | DNA-binding transcription repressor activity, RNA polymerase II-specific GO:0001227 |
| 1 | 0.017182549 | 1 | positive regulation of peptidyl-serine phosphorylation of STAT protein GO:0033141 |
| 1 | 0.017182549 | 1 | regulation of TOR signaling GO:0032006 |
| 1 | 0.017182549 | 1 | cellular response to interferon-beta GO:0035458 |
| 13 | 0.0176285 | 1 | membrane GO:0016020 |
| 1 | 0.018803811 | 1 | T cell activation involved in immune response GO:0002286 |
| 1 | 0.018803811 | 1 | developmental process GO:0032502 |
| 1 | 0.018803811 | 1 | blastocyst development GO:0001824 |
| 1 | 0.019613459 | 1 | natural killer cell activation GO:0030101 |
| 1 | 0.019613459 | 1 | microvillus membrane GO:0031528 |
| 1 | 0.020422452 | 1 | positive regulation of innate immune response GO:0045089 |
| 1 | 0.020422452 | 1 | endoplasmic reticulum exit site GO:0070971 |
| 1 | 0.020422452 | 1 | bHLH transcription factor binding GO:0043425 |
| 1 | 0.020422452 | 1 | ventricular cardiac muscle tissue morphogenesis GO:0055010 |
| 1 | 0.021230791 | 1 | clathrin-coated endocytic vesicle GO:0045334 |
| 3 | 0.021409584 | 1 | calcium ion binding GO:0005509 |
| 1 | 0.022038477 | 1 | regulation of ERK1 and ERK2 cascade GO:0070372 |
| 1 | 0.022038477 | 1 | fibronectin binding GO:0001968 |
| 1 | 0.022845509 | 1 | cytokine receptor binding GO:0005126 |
| 1 | 0.023651888 | 1 | developmental growth GO:0048589 |
| 1 | 0.024457615 | 1 | positive regulation of apoptotic signaling pathway GO:2001235 |
| 1 | 0.024457615 | 1 | positive regulation of Ras protein signal transduction GO:0046579 |
| 1 | 0.024457615 | 1 | positive regulation of receptor signaling pathway via JAK-STAT GO:0046427 |
| 1 | 0.024457615 | 1 | ATP hydrolysis coupled proton transport GO:0015991 |
| 1 | 0.025262691 | 1 | positive regulation of cytokine production GO:0001819 |
| 1 | 0.026067116 | 1 | peptidyl-tyrosine autophosphorylation GO:0038083 |
| 1 | 0.026067116 | 1 | phospholipase A2 activity GO:0004623 |
| 4 | 0.026984582 | 1 | integral component of plasma membrane GO:0005887 |
| 1 | 0.027674013 | 1 | B cell proliferation GO:0042100 |
| 1 | 0.027674013 | 1 | response to exogenous dsRNA GO:0043330 |
| 1 | 0.027674013 | 1 | multivesicular body GO:0005771 |
| 1 | 0.027674013 | 1 | respiratory gaseous exchange GO:0007585 |
| 1 | 0.028476488 | 1 | mesoderm formation GO:0001707 |
| 1 | 0.028476488 | 1 | lipopolysaccharide binding GO:0001530 |
| 1 | 0.028476488 | 1 | intra-Golgi vesicle-mediated transport GO:0006891 |
| 1 | 0.03007949 | 1 | blood circulation GO:0008015 |
| 1 | 0.030880018 | 1 | endopeptidase inhibitor activity GO:0004866 |
| 1 | 0.031679899 | 1 | protein K63-linked ubiquitination GO:0070534 |
| 1 | 0.031679899 | 1 | ubiquitin conjugating enzyme activity GO:0061631 |
| 1 | 0.034872959 | 1 | DNA-templated transcription, initiation GO:0006352 |
| 1 | 0.034872959 | 1 | potassium ion import across plasma membrane GO:1990573 |
| 1 | 0.03566961 | 1 | cellular response to hormone stimulus GO:0032870 |
| 1 | 0.03566961 | 1 | toll-like receptor signaling pathway GO:0002224 |
| 1 | 0.03566961 | 1 | positive regulation of interferon-gamma production GO:0032729 |
| 1 | 0.036465617 | 1 | lactation GO:0007595 |
| 1 | 0.03726098 | 1 | transport vesicle membrane GO:0030658 |
| 1 | 0.03964321 | 1 | positive regulation of phagocytosis GO:0050766 |
| 1 | 0.03964321 | 1 | signal transduction by protein phosphorylation GO:0023014 |
| 1 | 0.041228153 | 1 | hormone-mediated signaling pathway GO:0009755 |
| 1 | 0.042810533 | 1 | scavenger receptor activity GO:0005044 |
| 1 | 0.042810533 | 1 | adult locomotory behavior GO:0008344 |
| 1 | 0.042810533 | 1 | protein secretion GO:0009306 |
| 1 | 0.042810533 | 1 | transmembrane receptor protein tyrosine kinase activity GO:0004714 |
| 1 | 0.045179304 | 1 | humoral immune response GO:0006959 |
| 1 | 0.047542331 | 1 | regulation of protein localization GO:0032880 |
| 1 | 0.047542331 | 1 | odontogenesis of dentin-containing tooth GO:0042475 |
| 1 | 0.047542331 | 1 | positive regulation of protein kinase activity GO:0045860 |
| 1 | 0.048328732 | 1 | heart looping GO:0001947 |
| 1 | 0.049899626 | 1 | negative regulation of catalytic activity GO:0043086 |

**Supplementary Table S37. Gene Set Table for the 50-gene signature specific for the condition "Caco-2_SARS-CoV-2_down-regulated_and_up-regulated".** The genes of the input list representing the 50-gene signature was aligned to known biological processes by the use of the *Gene Set Enrichment* tool in GENEVESTIGATOR. For this type of analysis the 25-gene signature covering the down-regulated genes for a given condition was combined with the 25-gene signatures covering the up-regulated genes for the same condition. The p-values and the false discovery rates (FDR) are indicated. Only significant outcomes (p < 0.05) are shown.

| Intersection | p-value | min FDR | Gene Set |
|---|---|---|---|
| 2 | 3.64992E-05 | 1 | very-low-density lipoprotein particle assembly GO:0034379 |
| 4 | 3.78727E-05 | 1 | cellular protein metabolic process GO:0044267 |
| 3 | 7.37126E-05 | 1 | cholesterol homeostasis GO:0042632 |
| 4 | 0.000113186 | 1 | endoplasmic reticulum lumen GO:0005788 |
| 3 | 0.0001563 | 1 | lipid catabolic process GO:0016042 |
| 2 | 0.000166837 | 1 | cholesterol efflux GO:0033344 |
| 3 | 0.000167629 | 1 | steroid metabolic process GO:0008202 |
| 2 | 0.000181908 | 1 | low-density lipoprotein particle clearance GO:0034383 |
| 4 | 0.000188922 | 1 | post-translational protein modification GO:0043687 |
| 3 | 0.00022755 | 1 | negative regulation of endopeptidase activity GO:0010951 |
| 2 | 0.000305346 | 1 | digestion GO:0007586 |
| 2 | 0.000412605 | 1 | cysteine-type endopeptidase inhibitor activity GO:0004869 |
| 8 | 0.00079998 | 1 | extracellular region GO:0005576 |
| 1 | 0.000824851 | 1 | chylomicron remnant GO:0034360 |
| 1 | 0.000824851 | 1 | negative regulation of heat generation GO:0031651 |
| 1 | 0.000824851 | 1 | beta-3 adrenergic receptor binding GO:0031699 |
| 1 | 0.000824851 | 1 | bile-salt sulfotransferase activity GO:0047704 |
| 1 | 0.000824851 | 1 | negative regulation of adenylate cyclase-activating adrenergic receptor signaling pathway GO:007187 |
| 1 | 0.000824851 | 1 | mature chylomicron GO:0034359 |
| 1 | 0.000824851 | 1 | vesicle lumen GO:0031983 |
| 2 | 0.001424597 | 1 | platelet alpha granule lumen GO:0031093 |
| 1 | 0.001649035 | 1 | cholesterol O-acyltransferase activity GO:0034736 |
| 1 | 0.001649035 | 1 | regulation of enamel mineralization GO:0070173 |
| 1 | 0.001649035 | 1 | sterol O-acyltransferase activity GO:0004772 |
| 1 | 0.001649035 | 1 | negative regulation of locomotion involved in locomotory behavior GO:009032 |
| 1 | 0.001649035 | 1 | tryptophan 5-monooxygenase activity GO:0004510 |
| 1 | 0.002472554 | 1 | cellular response to prostaglandin stimulus GO:0071379 |
| 1 | 0.002472554 | 1 | transketolase activity GO:0004802 |
| 1 | 0.002472554 | 1 | serotonin biosynthetic process GO:0042427 |
| 1 | 0.002472554 | 1 | negative regulation of transport GO:0051051 |
| 1 | 0.002472554 | 1 | bile acid catabolic process GO:0030573 |
| 2 | 0.002834854 | 1 | cholesterol metabolic process GO:0008203 |
| 9 | 0.003253386 | 1 | metal ion binding GO:0046872 |
| 1 | 0.003295406 | 1 | cholesterol esterification GO:0034435 |
| 1 | 0.003295406 | 1 | protein lipidation involved in autophagosome assembly GO:0061739 |
| 1 | 0.003295406 | 1 | triglyceride mobilization GO:0006642 |
| 1 | 0.003295406 | 1 | thiamine metabolic process GO:0006772 |
| 1 | 0.003295406 | 1 | CAMKK-AMPK signaling cascade GO:0061762 |
| 1 | 0.003295406 | 1 | pinocytosis GO:0006907 |
[truncated: 9,298 more chars]
